# *Trichoderma tlahuicanensis* sp. nov. (Hypocreaceae), a novel mycoparasite of *Fusarium oxysporum* and *Phytophthora capsici* isolated from a traditional Mexican milpa

**DOI:** 10.3389/fmicb.2025.1714079

**Published:** 2025-11-27

**Authors:** Mario Iza-Arteaga, Verónica Lira-Ruan, Edgar Balcázar-López, Jorge Luis Folch-Mallol, Ayixon Sánchez-Reyes

**Affiliations:** 1 Centro de Investigación en Biotecnología, Universidad Autónoma del Estado de Morelos, Cuernavaca, Morelos, Mexico; 2 Centro de Investigación en Dinámica Celular, Instituto de Investigación en Ciencias Básicas y Aplicadas, Universidad Autónoma del Estado de Morelos, Cuernavaca, Morelos, Mexico; 3 Departamento de Farmacobiología, Centro Universitario de Ciencias Exactas e Ingeniería, Universidad de Guadalajara, Guadalajara, Jalisco, Mexico; 4 Investigador por México, SECIHTI-Instituto de Biotecnología, Universidad Nacional Autónoma de México, Cuernavaca, Morelos, Mexico

**Keywords:** *Trichoderma*, mycoparasitism, plant growth promotion, phylophenetic species concept, Bayesian speciation test, abiotic stress tolerance, milpa agroecosystem

## Abstract

**Introduction:**

The genus *Trichoderma encompasses* cosmopolitan fungi that play key ecological and biotechnological roles, including mycoparasitism, plant growth promotion, and tolerance to abiotic stress. As the catalog of undescribed species continues to expand, the need for precise species delimitation is increasingly evident.

**Methods:**

In this work, we isolated strain BMH-0061 as a root endophyte of chili (*Capsicum annuum*) and assessed its phenotypic and antagonistic potential. This strain originated from Mexico’s milpa agroecosystem, an underexplored reservoir of microbial diversity. To robustly evaluate its taxonomic status, we generated a near-complete, telomere-to-telomere genome assembly and assessed its completeness with BUSCO. Subsequently, we evaluated a comprehensive set of genomic coherence metrics, including Average Nucleotide Identity (ANI), Average Amino Acid Identity (AAI), Percentage of Conserved Proteins (POCP), and k-mer composition.

**Results:**

Integrative phylogenomic analyses, supported by Bayesian speciation models and clustering-based comparisons, consistently recovered BMH-0061 as an independent lineage. The strain exhibited broad-spectrum mycoparasitism against phytopathogens and tolerance to moderate abiotic stress. Morphological characterization, growth profiles, and multi-gene phylogenies suggested its placement within the *Trichoderma* genus, but it is distinct from known species. A near-complete telomere-to-telomere genome assembly was obtained, showing a BUSCO completeness of 99.08%. Genomic coherence analyses revealed that strain BMH-0061 shared ANI values ranging from 83.93% to 94.43% with its closest *Trichoderma* relatives, supporting its distinction as a separate lineage.

**Discussion:**

We formally describe this new taxon as Trichoderma tlahuicanensis sp. nov., in honor of the indigenous pre-Hispanic Tlahuica people of Morelos, Mexico. Our work demonstrates how a phylophenetic framework, combining genomic, phylogenetic, genetic diversity, and evolutionary analyses, provides a powerful approach to species delimitation. This integrative strategy confirms the recognition of T. tlahuicanensis as a distinct species while offering a methodological pathway for peers engaged in fungal systematics

## 1 Introduction

*Trichoderma* Pers. 1794 (Syn. *Hypocrea*, Hypocreales) is a widely distributed fungal genus comprising species with mycotrophic and phytosaprophytic lifestyles (Persoon, 1794). These species are often isolated from soils across diverse ecosystems, including agricultural fields, forests, deserts, and aquatic environments such as freshwater and marine systems (Woo et al., 2023). Notably, certain *Trichoderma* species are recognized for their roles as mycoparasites against plant fungal pathogens. Beyond their biocontrol capabilities, these fungi enhance plant growth by secreting plant hormones that reorganize root architecture and facilitate the chemical transformation of minerals into bioavailable forms (Yedidia et al., 2001).

The *Trichoderma*-plant interaction also activates plant defense mechanisms, including Induced Systemic Response (ISR) and Acquired Systemic Response (ASR), enhancing resistance to a wide range of phytopathogens. Furthermore, *Trichoderma* species induce stress-related pathways, enabling plants to tolerate abiotic stresses like drought and salinity (Mastouri et al., 2012; Guler et al., 2016). Given the escalating challenges posed by climate change and the growing need to reduce the use of fertilizers and pesticides in agriculture, the interest in harnessing *Trichoderma* species as eco-friendly bioagents has surged. These fungi offer a promising avenue for sustainable agriculture, capable of both protecting plants and reducing environmental contamination.

To leverage *Trichoderma* biodiversity effectively, accurate classification of wild isolates is essential. Reliable identification involves integrative approaches that include morphological, phenotypic, and molecular techniques. Traditionally, the 28S internal transcribed spacer (ITS) has been the primary molecular marker for classifying new isolates through phylogenetic analyses. However, ITS-based classification has proven to be insufficient to verify the species hypothesis. Recent studies support the use of at least four molecular markers—typically RNA Polymerase II (RPB2), calmodulin, β-tubulin, and Translation Elongation Factor 1 (TEF1)—to achieve better resolution and accuracy (Brandon Matheny et al., 2007; Stielow et al., 2015; Lücking et al., 2020). Over the past five decades, advancements in molecular techniques have led to the identification of more than 400 species (Cai and Druzhinina, 2021).

In recent years, whole-genome sequencing (WGS) has emerged as a powerful tool to address the limitations of traditional marker-based approaches. WGS provides comprehensive genomic data that enable robust species identification, genomic mining, and the investigation of phylogenetic and population-level relationships (Ullmann et al., 2022). The advent of third-generation sequencing technologies, such as Oxford Nanopore sequencing, has revolutionized this field by enabling rapid and cost-effective generation of complete genomic sequences with long reads. Together, the technical, conceptual, and delimitation advances offer unprecedented resolution for distinguishing closely related microbial species.

In this context, we examined *Trichoderma* strain BMH-0061, isolated as a root endophyte from chili (*Capsicum annuum*) plants grown in a “milpa” system in Cuernavaca, Morelos, México (18°58′35′′ N, 99°13′36′′ W, 1824) (Zúñiga-Silgado et al., 2020). The milpa, a sustainable pre-Columbian agricultural system, integrates maize (*Zea mays*) with other crops, including beans (*Phaseolus vulgaris*), chili (*C. annuum*), and tomato (*Solanum lycopersicum*), alongside endemic herbaceous species (Fonteyne et al., 2023). Strain BMH-0061 consistently exhibited high phosphorus solubilization across different soil types, reaching concentrations up to 34.65 mg L^−1^, over 11 times higher than the control, and demonstrating versatility under acidic conditions (pH 2.8–3.3). Initial ITS-based phylogenetic analysis classified the strain within the *Trichoderma* genus but placed it on a distinct branch related to the *koreanum* subclade, suggesting it might represent a new species. Subsequent analyses using four additional markers (β-tubulin, calmodulin, TEF1, and RNA Polymerase II) consistently positioned the strain in unique branches, distinct from known *Trichoderma* species (Zúñiga-Silgado et al., 2020). However, few studies have explored *Trichoderma* diversity in agroecological systems such as the Mexican milpa, where genomic adaptation and ecological specialization remain underexplored.

In this study, we utilized Oxford Nanopore MinION technology to perform *de novo* genome assembly of *Trichoderma* strain BMH-0061, isolated from a milpa in Morelos, México. We employed an integrative conceptual framework, specifically the phylophenetic species concept (Rosselló-Mora and Amann, 2001; Ide-Pérez et al., 2024), to evaluate the species hypothesis. This approach combined genomic, phylogenetic, and phenotypic data, alongside molecular speciation analyses conducted under rigorous Bayesian models, to confidently identify the strain as a novel species. We have named this species *Trichoderma tlahuicanensis* sp. nov., in honor of the ancient Tlahuica people, who inhabited the region now known as the State of Morelos between 1100 A.D. and 1521. This newly described species demonstrates significant potential as a biocontrol agent, supported by compelling evidence of its mycoparasitic behavior, observed both macroscopically and microscopically.

## 2 Materials and methods

### 2.1 Culture conditions, genome sequencing, and assembly

Strain BMH-0061 was isolated as an endophyte from chili (*C. annuum*) roots growing in “milpa” located in Morelos, México in 2020 (18°58′35′′ N, 99°13′36′′ W, 1824) (Zúñiga-Silgado et al., 2020). For DNA extraction, strain BMH-0061 was cultured in Potato-Dextrose Agar media (PDA Sigma-Aldrich Química, SL Toluca, México) at 25 °C for 5 days in the dark.

High molecular weight DNA was purified from a liquid culture by centrifuging two 50 mL aliquots of the medium in conical tubes at 7000 rpm for 15 min, followed by phenol-chloroform extraction (Sambrook and Russell, 2001). The extracted DNA was quantified fluorometrically using the dsDNA High Sensitivity Kit and a DS-11 FX fluorometer (DeNovix). Sample integrity was assessed by running 100 ng of DNA on a 0.5% agarose gel. Library preparation was performed according to the Native Barcoding Kit 96 V14 (SQK-NBD114.96) protocol from Oxford Nanopore Technologies. Prior to sequencing, the library was quantified fluorometrically using the same dsDNA High Sensitivity Kit and DS-11 FX fluorometer. The quantified library was then used to calculate the loading concentration to achieve the desired sequencing yield (~40X). Sequencing was conducted on an Oxford Nanopore MinION device using a FLO-MIN114 (R10.4.1) flow cell (ID: FAZ08078).

The raw sequencing data were processed using Guppy v3.2.2 (ONT) on a high-performance computing (HPC) cluster for base-calling. Read length, quality, and yield were evaluated as part of the quality control process using NanoPlot v1.33.0 (De Coster and Rademakers, 2023) and Porechop v0.2.4.^1^ Filtlong^2^ was used to filter long reads by quality. Several genome assemblies were generated with Canu v2.1.1 (Koren et al., 2017), Shasta v0.8.0 (Shafin et al., 2020), Flye v2.9 (Kolmogorov et al., 2019), Raven v1.8.1 (Vaser and Šikić, 2021), Unicycler v0.4.4 (Wick et al., 2017), and Dragonflye v1.0.13 (Petit, 2024). The best and more contiguous assembly was obtained with Quickmerge (Chakraborty et al., 2016), which was refined with Proovframe 0.9.7 (Hackl et al., 2021) to correct errors and restore reading frame fidelity. All assemblers and polishing tools were executed using default parameters. The final assembly was selected based on contiguity (N50) and consistency with expected genome size for the genus.

Quast v5.0.2 (Gurevich et al., 2013) was used as genome quality evaluation tool. Sequence integrity was assessed using BUSCO v5.8.2 with predefined ortholog datasets specific to the Fungi group (Manni et al., 2021). Gene prediction for eukaryotic sequences was performed using FunGAP v1.1.1 (Min et al., 2017), with gene models based on *Fusarium graminearum*. Functional annotation of predicted proteins was conducted using multiple tools, including KofamKOALA, BlastKOALA, which assigned orthologs based on the KEGG (Kyoto Encyclopedia of Genes and Genomes) database (Moriya et al., 2007; Kanehisa et al., 2016; Aramaki et al., 2020).

### 2.2 Determination of genomic coherence metrics

The taxonomic context of strain BMH-0061 was assessed using genomic coherence metrics. Mash v2.3 was used to calculate Mash distance (D) by generating genomic sketches to estimate global mutation distances (Ondov et al., 2016). A Mash distance of 𝐷≤0.05 indicates high genomic coherence and likely taxonomic similarity. We compared the genome of strain BMH-0061 against 9,834 fungal genomes retrieved from the NCBI Assembly Type Material database (downloaded February 5, 2022) using the query *(“Fungi”[Organism] OR Fungi[All Fields]) AND ((latest[filter] OR “latest genbank”[filter]) AND (all[filter] NOT anomalous[filter] AND all[filter] NOT partial[filter]))*. The 20 closest genomes based on Mash distance were selected for further analysis. Average Nucleotide Identity (ANI) was calculated using OrthoANI v0.5.0 and FastANI (Lee et al., 2016; Jain et al., 2018). Average Amino Acid Identity (AAI) was analyzed with CompareM v0.1.2,^3^ while Percentage of Conserved Proteins (POCP) was calculated following (Qin et al., 2014). We used Jellyfish 2.3.1 to extract K-mer (6-mer) profiles and compare genomic sequences based on k-mer composition (Marçais and Kingsford, 2011).

Finally, the complete analysis was integrated using the Fast-Fungal-Genome-Classifier pipeline,^4^ which we employed as an integrative tool to combine multiple genomic coherence metrics, phylogenomic analysis and robust species delimitation.

### 2.3 Comparative phylogenetic analysis

The genome of strain BMH-0061 was compared to 127 genomes from the *Trichoderma* genus (35 RefSeq and 92 GenBank, downloaded from NCBI on August 19, 2022). The analysis included JolyTree v1.1b.191021ac for inferring phylogenies based on alignment-free distance methods between genomes (Criscuolo and Morgenstern, 2020). In addition, a multi-locus phylogeny was estimated with nucleotide sequences of ITS, RPB2, and TEF1-α markers extracted from the genome of strain BMH-0061 using BLASTn v2.13.0. These sequences were compared with type-confirmed sequences from NCBI’s GenBank database and recent phylogenies, focusing on the *Harzianum* section (Zhao and Yi, 2010; Kubicek et al., 2019; Gu et al., 2020; Inglis et al., 2020; Bustamante et al., 2021; Zheng et al., 2021) (Table 1). Sequences were independently aligned with MAFFT v7.508 and curated using TRIMAL v1.4.rev15 -gappyout option (Capella-Gutiérrez et al., 2009; Katoh and Standley, 2013). The concatenation of ITS, TEF1-α, and RPB2 sequences was performed using Seaview, and phylogenetic inference was conducted with IQ-TREE v2.2.0.3 using standard model selection and SH-aLRT with 1,000 replicates for branch support (Gouy et al., 2010; Nguyen et al., 2015). Phylogenetic trees were visualized and edited with FigTree (v1.4.2). Nucleotide diversity (*π*), the number of segregating sites, parsimony-informative sites, and Tajima’s D statistic were estimated from the concatenated alignment of the ITS, RPB2, and TEF genes to assess genetic variation and test for neutrality using PopART version 1.7 (Leigh and Bryant, 2015).

**Table 1:** List of species and GenBank accession numbers of sequences used in the multi-locus phylogenetic analysis conducted in this study.

		ITS	RPB2	TEF1-α
*Trichoderma afarasin*	DIS 314F ^T^	FJ442259	FJ442778	FJ463400
*Trichoderma afroharzianum*	GJS 04–186 ^T^	FJ442265	FJ442691	FJ463301
*Trichoderma afroharzianum*	CBS 466.94	KP009262	KP009150	KP008851
*Trichoderma alni*	CBS120633	EU518651	EU498349	EU498312
*Trichoderma alpinum*	HMAS 248821 ^T^	KY687906	KY687958	KY688012
*Trichoderma anaharzianum*	YMF 1.00241 ^T^	MH262584	MH262577	MH236493
*Trichoderma anaharzianum*	YMF 1.00383	MH113931	MH158995	MH183182
*Trichoderma asiaticum*	YMF 1.00352	MH113930	MH158994	MH183183
*Trichoderma asiaticum*	YMF1.00168	MH262582	MH262575	MH236492
*Trichoderma atrobrunneum*	GJS 90–254 ^T^	AF443926	FJ442735	AF443943
*Trichoderma atrobrunneum*	GJS 04–67	FJ442273	FJ442724	FJ463360
*Trichoderma atrobrunneum*	T42	KX632515	KX632572	KX632629
*Trichoderma azevedoi*	CEN1422	MK714902	MK696821	MK696660
*Trichoderma bannaense*	HMAS:248840 ^T^	NR_154570	KY687979	KY688037
*Trichoderma breve*	HMAS 248844 ^T^	KY687927	KY687983	KY688045
*Trichoderma concentricum*	HMAS 248833 ^T^	KY687915	KY687971	KY688027
*Trichoderma endophyticum*	549F18C-AC	MK713513	MT311144	MT337595
*Trichoderma endophyticum*	23F18C-AC	MK713502	MT311146	MT337596
*Trichoderma guizhouense*	HGUP0038	JN191311	JQ901400	JN215484
*Trichoderma guizhouense*	HGUP0039	JX089584	JQ901401	JX089585
*Trichoderma harzianum*	CBS 226.95 ^T^	AY605713	AF545549	AF348101
*Trichoderma harzianum*	GJS 05107	FJ442679	FJ442708	FJ463329
*Trichoderma inhamatum*	CBS 273.78 ^T^	NR_134378	FJ442725	AF348099
*Trichoderma lentiforme*	DIS 253B	FJ442619	FJ442756	FJ851875
*Trichoderma lentiforme*	DIS 94D	FJ442615	FJ442749	FJ463379
*Trichoderma lentiforme*	DIS 218E	FJ442220	FJ442793	FJ463310
*Trichoderma lentiforme*	DIS 354A	FJ442229	FJ442734	FJ463339
*Trichoderma lentiforme*	CEN1428	MK714909	MK696827	MK696667
*Trichoderma lentiforme*	CEN1429	MK714910	MK696828	MK696668
*Trichoderma lentinulae*	CGMCC 3.19847 ^T^	MN594469	MN605867	MN605878
*Trichoderma lixii*	CBS 110080 ^T^	NR_131264	KJ665290	AF443938
*Trichoderma pollinicola*	LC11686 ^T^	MF939593	MF939605	MF939620
*Trichoderma pseudoasiaticum*	YMF 1.06200 ^T^	MN977792	MT052183	MT070155
*Trichoderma pseudodensum*	HMAS 248828 ^T^	KY687910	KY687967	KY688023
*Trichoderma pyramidale*	CBS 135574 ^T^	KX632513	KJ665334	KJ665699
*Trichoderma rifaii*	DIS 337F ^T^	FJ442621	FJ442720	FJ463321
*Trichoderma rugulosum*	SFC20180301-001	MH050353	MH025986	MH025984
*Trichoderma simile*	YMF 1.06201 ^T^	MN977793	MT052184	MT070154
*Trichoderma simile*	YMF_1.6180	MN977794	MT052185	MT070153
*Trichoderma simmonsii*	G.J.S. 91–138 ^T^	AF443917	FJ442757	AF443935
*Trichoderma simmonsii*	G.J.S. 90–127	AF443918	FJ442798	AF443936
*Trichoderma vermifimicola*	CGMCC 3.19694 ^T^	MN594473	MN605871	MN605882
*Trichoderma xixiacum*	CGMCC 3.19697 ^T^	MN594476	MN605874	MN605885
*Trichoderma zayuense*	HMAS 248835 ^T^	KY687918	KY687974	KY688031
*Trichoderma zelobreve*	CGMCC 3.19695 ^T^	MN594474	MN605872	MN605883
*Trichoderma zeloharzianum*	YMF1.00268 ^T^	MH113932	MH158996	MH183181
***Trichoderma tlahuicanensis***	**BMH-0061**	OR710780	OR711907	OR711908
*Protocrea pallida*	CBS 299.78	NR_111329	EU703948	EU703900

ITS, internal transcribed spacer regions; RPB2, gene encoding of the second largest nuclear RNA polymerase subunit; TEF1-α, translation elongation factor-1α; *T. tlahuicanensis* data are indicated in bold. ^T^ from TYPE material.

### 2.4 Species delimitation using Bayesian Poisson tree processes (bPTP) and generalized mixed yule coalescent (GMYC) approaches

We performed species delimitation using the Bayesian Poisson Tree Processes (bPTP v0.51) (Zhang et al., 2013) and Generalized Mixed Yule Coalescent (GMYC) models (Fujisawa et al., 2016). Both methods were implemented in Python and configured to accept non-ultrametric trees as input.^5^ Newick-format trees from prior phylogenetic analyses (JolyTree, Multilocus) were used as input for independent runs. Convergence was assessed through 10^6^ Markov Chain Monte Carlo (MCMC) iterations with four seed replicates.

### 2.5 Clustering-based species analyses

We performed a clustering-based species analysis using hexamer frequency profiles to compare *Trichoderma* sp. BMH-0061 with its 20 closest genomic neighbors (as determined by Mash distances). To assess group structure, we calculated weighted Jaccard and Containment indices, Euclidean distance, and Pearson correlation to capture compositional differences. A dendrogram using Ward’s method was generated to visualize genomic relationships. The ggplot2 package in R was used to generate graphical outputs (Wickham, 2011).

### 2.6 Genomic differentiation across geographic location

We conducted a Mantel test to evaluate the relationship between mutational genomic distance and geographic separation, using two distance matrices: (1) a pairwise genomic matrix derived from mutational distance and (2) a categorical distance matrix reflecting geographic origin. The analysis was performed using both Spearman and Kendall correlation methods to account for potential non-linear relationships. We focused on a subset of 20 genomes with mutational distances ≤0.1, representing a cluster of higher genomic coherence. This selection criterion was applied to minimize noise and strengthen the interpretability of the observed patterns.

A comparative genomic analysis between *Trichoderma* sp. BMH-0061 and its closest reference genome (*T. harzianum*, GCA_019097725.1) was performed using whole-genome alignment and variant detection with GSAlign. Insertions larger than 31 bp—corresponding to the standard k-mer size (k = 31) used in genome assembly and variant discovery—were filtered and prioritized to identify structurally and functionally relevant events (Lin and Hsu, 2020).

### 2.7 Growth and phenotypic analysis

To determine the optimum growth temperature of strain BMH-0061, mycelial disk of 5 mm diameter from a PDA previous culture were placed in the center of a Petri dish containing either PDA media, Malt Extract Agar (MEA) media (malt 15 g/L, agar 15 g/L) or Minimum Media (MM) (KH_2_PO_4_ 1 g/L, NH_4_NO_3_ 1 g/L, MgSO_4_ 0.5 g, KCl 0.05 g/L, glucose 4%, agar 2.5%). Petri dishes with the cultures in the three media were incubated at 18, 25, 28 and 37 °C during 5 days at 12 h light/ 12 h/ dark. The colony diameter was measured every 24 h using a mechanical Vernier scale. Three repetitions (biological replicates) form each culture media were performed.

Morphological aspects of strain BMH-0061 as the colony appearance and color were analyzed under a stereomicroscope Olympus SZX12 and photographed with the camera from a telephone OPPO RENO 10. Detailed analyses of conidiophores, philiades and conidia were performed. A drop of lactophenol cotton blue stain (20% lactophenol and 10% cotton blue) was added to stain the samples that were analyzed in an inverted optical microscope (Zeiss axio observer 440,782–9,902-000 and a camera ORCA flash 0.4 and software Toup View v.3.7).

### 2.8 Antagonistic assays against phytopathogenic fungi

To evaluate the mycoparasitism of strain BMH-0061 against phytopathogenic fungi, dual culture antagonism assays were performed. Six Trichoderma species (*T. asperellum*, *T. atroviride*, *T. citrinoviride*, *T. reesei*, *T. virens*) and strain BMH-0061 were evaluated *in vitro* against two major phytopathogenic microbes: the fungus *Fusarium oxysporum* and the Oomycete *Phytophthora capsici.* All the strains used belong to our laboratory collection.

First it was stablished that under culture conditions (PDA media and 25 ± 2 °C with 12 h light/12 h dark) all *Trichoderma* strains grow to occupy 50% of Petri dish surface around 24 h, and *F. oxysporum* and *P. capsici* reach the same area at 48 h. Thus, for dual culture assays the phytopathogenic microbes were inoculated 24 h before the *Trichoderma* species. Dual cultures were performed by placing the *Trichoderma* strain and phytopathogenic fungi in the same PDA 9 mm dish; a 5 mm diameter mycelial disk of each fungus were placed in opposite extremes of the Petri dish and 1 cm away from dish border. Each phytopathogen was also cultured alone to use it as reference of growth. Plates were incubated at 25 ± 2 °C with 12 h light/12 h dark for 9 days.

To calculate the percentage of inhibition of radial growth (PIRG) the following formula was used:


PIRG=(R1−R2)/R1×100

where R1 is the radius of pathogenic fungus when growing alone and R2 is the radius of pathogenic fungus growing together with *Trichoderma* (Rajani et al., 2021).

The Petri dishes were photographed with the camera from a telephone OPPO RENO 10 at the end of the assay and the measurements were performed using ImageJ software (Abramoff, 2007). The assay was performed three times. Data were analyzed with ANOVA followed by Tukey’s test (*p* ≤ 0.05) using SPSS for windows software (SPSS, Inc., Chicago, IL, United States).

### 2.9 Mycoparasitism evaluation

Mycoparasitism was analyzed using the humid chamber method described by Harris (1986). Spores of *Trichoderma* sp. BMH-0061 and *F. oxysporum* or *P. capsici* were inoculated at the opposed extremes of a PDA media rectangle (1 × 2 cm) over a slide, the sample was covered with a coverslide and the preparation was placed inside a sterile Petri dish containing a sheet of filter paper humidified with sterile water, the humid camera was incubated in darkness at 28 °C for 48 h. The interaction was analyzed in an inverted microscope and photographed (Zeiss axio observer 440782-9902-000 and a camera ORCA flash 0.4 and software ToupView v.3.7).

## 3 Results and discussion

### 3.1 Telomere-to-telomere genome assembly and testing of genomic coherence hypotheses

The *Trichoderma* sp. BMH-0061 genome sequencing yielded 291,933 long-reads, with an average length of 6,231.9 bp and read N50 of 9,514.00 bp. We used Quickmerge to combine the outputs of multiple *de novo* assemblers to improve contiguity and overall assembly quality. The assembly produced a highly contiguous genome (overall size of 39.9 Mb, N50 of 6.8 Mb) with seven telomere-to-telomere scaffolds and all scaffolds assembled at the chromosome scale (Table 2 and Supplementary Table S1). In addition, we successfully identified the mitochondrial genome with a size of 31,755 bp (Table 2). We identified telomeric repeats (*TTAGGG* and its reverse complement, *CCCTAA*) consistent with those found in other *Trichoderma* genomes (Li, W. et al., 2021). The assembly size agrees with the size observed in other strains of this genus (~32–42 Mb)^6^ (Li, W. et al., 2021, Li, L. et al., 2021; Schalamun and Schmoll, 2022). Furthermore, the assembly’s completeness of 99.08% and the absence of duplicated BUSCO genes supports that this is a haploid genome version without evidence of contamination. With only 0.92% of missing genes and no fragmented genes, the assembly represents a reliable resource for gene prediction and functional annotation, maintaining high integrity, consequently, the pseudogene rate is expected to be low. We preliminarily concluded that the karyotype of strain BMH-0061 consists of seven nuclear chromosomes and one mitochondrial chromosome, with an approximate genome size of ~39.9 Mb. It is noteworthy that this proposed karyotype is similar to that of *T. simmonsii*, a close relative (Chung et al., 2021).

**Table 2:** Genome assembly statistics of the strain BMH-0061.

Assembly metrics	
Genome size	39,798,537 bp
Number of organelles	1
Number of scaffolds	8
Contig N50	6.8 Mb
Contig L50	3
GC percent	48.54
Genome coverage	45.0x
Assembly level	Scaffold
GenBank assembly accession	GCA_030015385.1
Taxon	*Trichoderma* sp. BMH-0061
WGS project	JARWZN01
Assembly type	haploid
Non-nuclear (Mitochondrion MT) size	31,755 bp
BUSCO predictions	
Total core genes queried	758
BUSCO completeness	99.08%
Complete BUSCOs	751
Complete and single-copy BUSCOs	751
Complete and duplicated BUSCOs	0
Fragmented BUSCOs	0
Missing BUSCOs	7
Total BUSCO groups searched	758
Annotation predictions	
Protein-coding genes	12,629
tRNAs	161
rRNAs	8
KEGG annotations	5,824

Gene prediction for the BMH-0061 strain was performed using FunGap (Min et al., 2017), that identified 12,629 genes (Table 2). This number concurs with predictions for other *Trichoderma* species, such as 13,120 genes for *T. simmonsii* (Chung et al., 2021), 11,865 genes for *T. atroviride*, 12,518 genes for *T. virens*, and 9,143 genes for *T. reesei* (Kubicek et al., 2011). Functional annotation of protein-coding genes resulted in 4,419 KEGG Orthology (KO) assignments, representing 35% of unified functional annotations (Supplementary Table S2). This proportion aligns with the typical range observed for annotated genes in newly assembled genomes lacking prior annotation frameworks (30–50%) (Griesemer et al., 2018).

To test the hypothesis of genomic coherence of strain BMH-0061, we calculated multiple genomic metrics, including Mash distance, ANI, AAI, POCP, this also allowed us to investigate the taxonomic boundaries of strain BMH-0061. The closest hits based on the observed indices (e.g., D < 0.05), include *T. harzianum* (GCA_019097725.1) with D = 0.038 and *T. simmonsii* (GCA_019565615.1) D = 0.039 (Table 3). However, within this range, several species from different taxonomic contexts were also observed, indicating that genomic coherence is high within the group of close phylogenetic neighbors and D < 0.05 threshold does not provide a robust criterion for reliable classification (Ondov et al., 2016).

**Table 3:** Overall genome relatedness indices of the strain BMH-0061 against 20 nearest representatives in genomic distance of the genus *Trichoderma*.

Strain	Assembly accession	D	ANI	AAI	POCP	Hexamer frequency	Country of origin
*Trichoderma harzianum*	GCA_019097725.1	0.038	94.43	94.62	95.10	81.97	Italy
*Trichoderma* sp. *IMV 00454*	GCA_001931985.1	0.038	94.35	94.65	94.99	80.14	Hungary
*Trichoderma simmonsii*	GCA_019565615.1	0.039	94.38	94.65	95.18	75.93	United States
*Trichoderma guizhouense*	GCA_002022785.1	0.045	93.34	94.12	94.62	82.60	Austria
*Trichoderma semiorbis*	GCA_020045945.2	0.048	92.70	93.56	93.63	65.00	China
*Trichoderma lentiforme*	GCA_011066345.1	0.048	92.93	93.80	93.86	94.15	Brazil
*Trichoderma atrobrunneum*	GCA_003439915.1	0.049	92.43	93.36	93.30	74.53	Australia
*Trichoderma harzianum*	GCA_001990665.1	0.050	92.87	93.72	93.49	64.50	United States
*Trichoderma harzianum CBS 226.95*	GCA_003025095.1	0.051	92.86	93.59	93.43	67.66	Italy
*Trichoderma harzianum*	GCA_002838845.1	0.051	92.91	93.64	93.39	65.21	Australia
*Trichoderma lixii*	GCA_014468695.1	0.051	92.91	93.68	92.03	90.34	Brazil
*Trichoderma harzianum*	GCA_019393575.1	0.053	92.88	93.61	92.35	83.64	Italy
*Trichoderma brevicrassum*	GCA_017311225.1	0.053	92.60	93.59	94.07	95.61	Germany
*Trichoderma afroharzianum*	GCA_020736905.1	0.059	91.40	92.95	92.59	62.55	China
*Trichoderma harzianum*	GCA_000988865.1	0.060	91.49	92.93	92.32	67.72	China
*Trichoderma afroharzianum*	GCA_016490745.1	0.060	91.45	92.93	92.46	75.37	Brazil
*Trichoderma harzianum*	GCA_010015525.1	0.061	91.27	92.82	92.34	58.96	Korea
*Trichoderma harzianum*	GCA_002894145.1	0.084	88.09	90.25	91.20	77.24	China
*Trichoderma pleuroti*	GCA_001721665.1	0.088	87.56	89.05	88.25	75.93	Taiwan
*Trichoderma virens FT-333*	GCA_020647705.1	0.128	83.93	85.06	86.13	54.83	India
*Trichoderma* sp. BMH-0061	GCA_030015385.1	0.0	100	99.99	99.99	-	Mexico
*Beauveria brongniartii RCEF 3172*	GCA_001636735.1	0.230	76.84	63.08	61.40	-	China
*Escovopsis weberi*	GCA_003055145.1	0.263	78.61	65.97	57.39	-	United Kingdom

ANI, AAI, POCP and hexamer frequency are expressed as percentage.

BMH-0061 exhibited ANI values with other *Trichoderma* strains ranged from 83.93 to 94.43%, with the closest relatives being *Trichoderma harzianum* (GCA_019097725.1) at 94.43% ANI, 94.62% AAI, and 95.10% POCP; and *Trichoderma simmonsii* (GCA_019565615.1) at 94.38% ANI, 94.65% AAI, and 95.18% POCP. *T. guizhouense*, *T. semiorbis*, *T. lentiforme*, *T. atrobrunneum* also share between 92 and 94% identity at the nucleotide and/or protein level. Although closely related, the values fall below the 95% species delineation threshold (ANI or AAI), which in fungi generally marks conspecificity, indicating that BMH-0061 is genetically distinct.

However, as broader genomic comparisons are conducted, ANI values below ~98% have been linked to distinct species, suggesting that speciation can occur even in the presence of high overall genomic similarity (Gostinčar, 2020; Lalanne and Silar, 2025). We observed a significant space of coherence in the ‘population’ with ANI values consistently above 90% for most *Trichoderma* strains, suggesting that BMH-0061 shares a considerable degree of genomic similarity with these strains, even if it does not meet the strict criteria for species-level relatedness. This “space of coherence” implies that BMH-0061 shares a common evolutionary framework with these strains, despite its genetic distinctness. The most distant relative, *Trichoderma virens* FT-333 (GCA_020647705.1), showed significantly lower values (ANI: 83.93%, AAI: 85.06%, POCP: 86.13%), further emphasizing the genetic divergence of BMH-0061 from some members of the genus.

Notably, while some genomic coherence values are near the 95% threshold, there is a significant variation in hexamer frequency among BMH-0061 and its close neighbors. When analyzing the proportion of shared hexamers between genomes using the Jaccard index (considering all unique hexamers), *T. harzianum*, *T. simmonsii*, and *T. guizhouense* shared less than 82% of their kmers. In contrast, the more phylogenetically distant species, *T. brevicrassum* and *T. lentiforme*, exhibited a higher degree of kmer similarity, sharing approximately 95% of their hexamers (Table 3 and Supplementary Figure S1). This variation suggests differences in genome organization/composition regardless of their genomic distance, which could reflect unique evolutionary adaptations in gene composition. The hexamer frequency estimator is widely recognized for its implications in gene content analysis, as it can effectively differentiate coding and non-coding regions within a genome. This property enables hexamer frequency to function as a content-based sensor, aiding in the accurate prediction of gene-rich regions and overall genome composition (Hutchinson, 1996; Wang et al., 2013; Klapproth et al., 2023). High kmer similarity between *T. brevicrassum* and *T. lentiforme* could result from conserved genomic regions, horizontal gene transfer events, or similar selective pressures shaping their genome composition. Additionally, variations in repetitive elements, genome size, and compositional biases may contribute to the observed discrepancies, reflecting the influence of genomic architecture and sequence composition on genome evolution. This genetic distinctness, combined with the significant “space of coherence” (ANI > 90%), predict that BMH-0061 is part of the *Trichoderma* genus but occupies a unique phylogenetic position, potentially reflecting novel functional or ecological adaptations, warranting further investigation into its taxonomic classification and biological significance.

### 3.2 Clustering-based species analysis and geographic genomic differentiation

**Figure 1:**
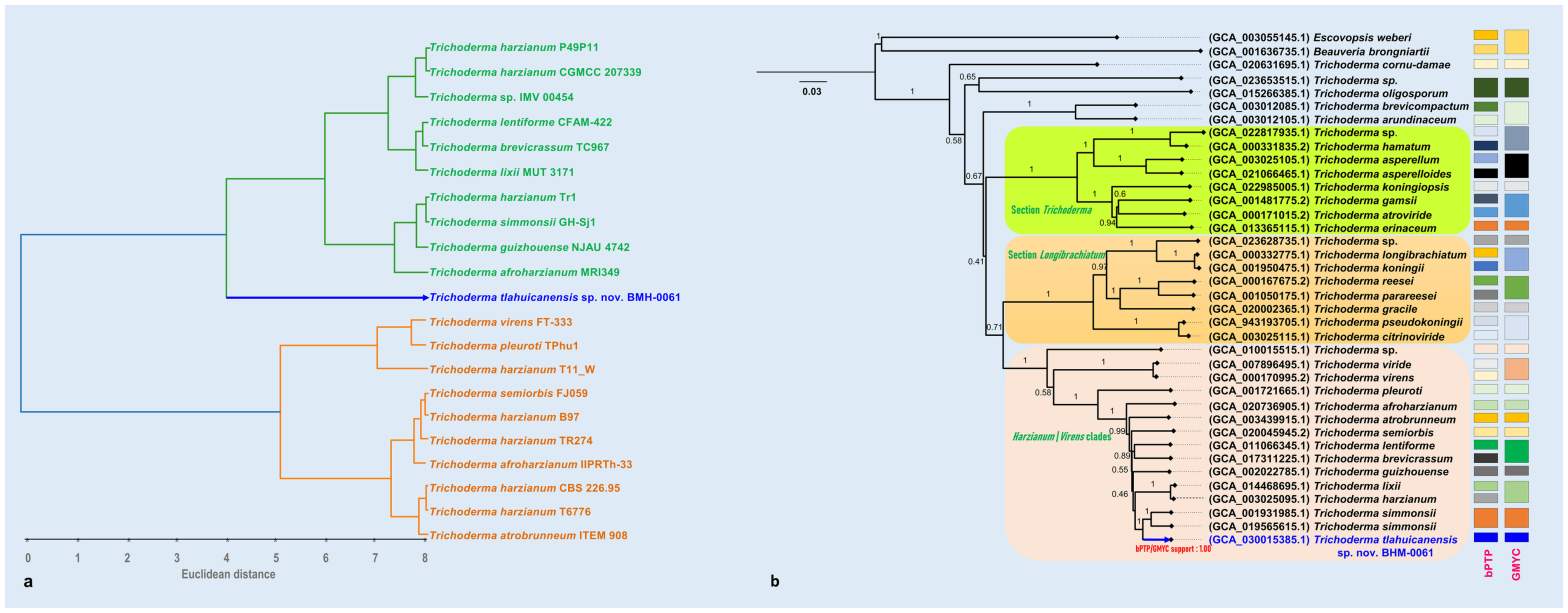
Clustering-based species delimitation of *T. tlahuicanensis* BMH-0061 based on hexamer frequency profiles **(a)**. Phylogenomic tree reconstructed with JolyTree illustrating the evolutionary relationships among the same set of genomes **(b)**. Both analyses consistently position *Trichoderma* sp. BMH-0061 as a distinct lineage, supporting its separation from closely related *Trichoderma* species. Evolutionary species delimitation results (bPTP and GMYC) are shown as vertical bars on the right.

To explore the group structure within the dataset composed of the genomes closest to BMH-0061, we conducted a clustering-based species analysis using genomic similarity data and hexamer content expressed as frequency (Figure 1a). We observe two major clusters or subgroups; however, BMH-0061 is located on a completely separate branch, supporting its distinctiveness. Furthermore, a Mantel test based on mutational distance and genomes geographic origin using Spearman (*r* = 0.11, *p* = 0.034) and Kendall (*r* = 0.087, *p* = 0.046) supports the hypothesis of allopatric isolation, suggesting a low but statistically significant correlation between genomic distances and geographic separation (Supplementary Figure S2). These findings suggest that spatial separation may contribute to genetic divergence, aligning with the idea that restricted gene flow due to geographic barriers can drive genomic differentiation; a pattern also observed in other ascomycete fungi (Giraud and Gourbière, 2012; Sur et al., 2021). However, while these results are statistically significant, they are not fully predictive, and the low correlation values suggest that other factors may also play a crucial role. Additionally, a larger sample size would provide a more robust estimation of the relationship, reducing variability and increasing confidence in the observed trend. Nevertheless, this outcome supports the premise that geographic distance influences genomic divergence, warranting further investigation into regional patterns of genetic structure in the group.

### 3.3 Phylogenetic hypothesis and species delimitation

**Figure 2:**
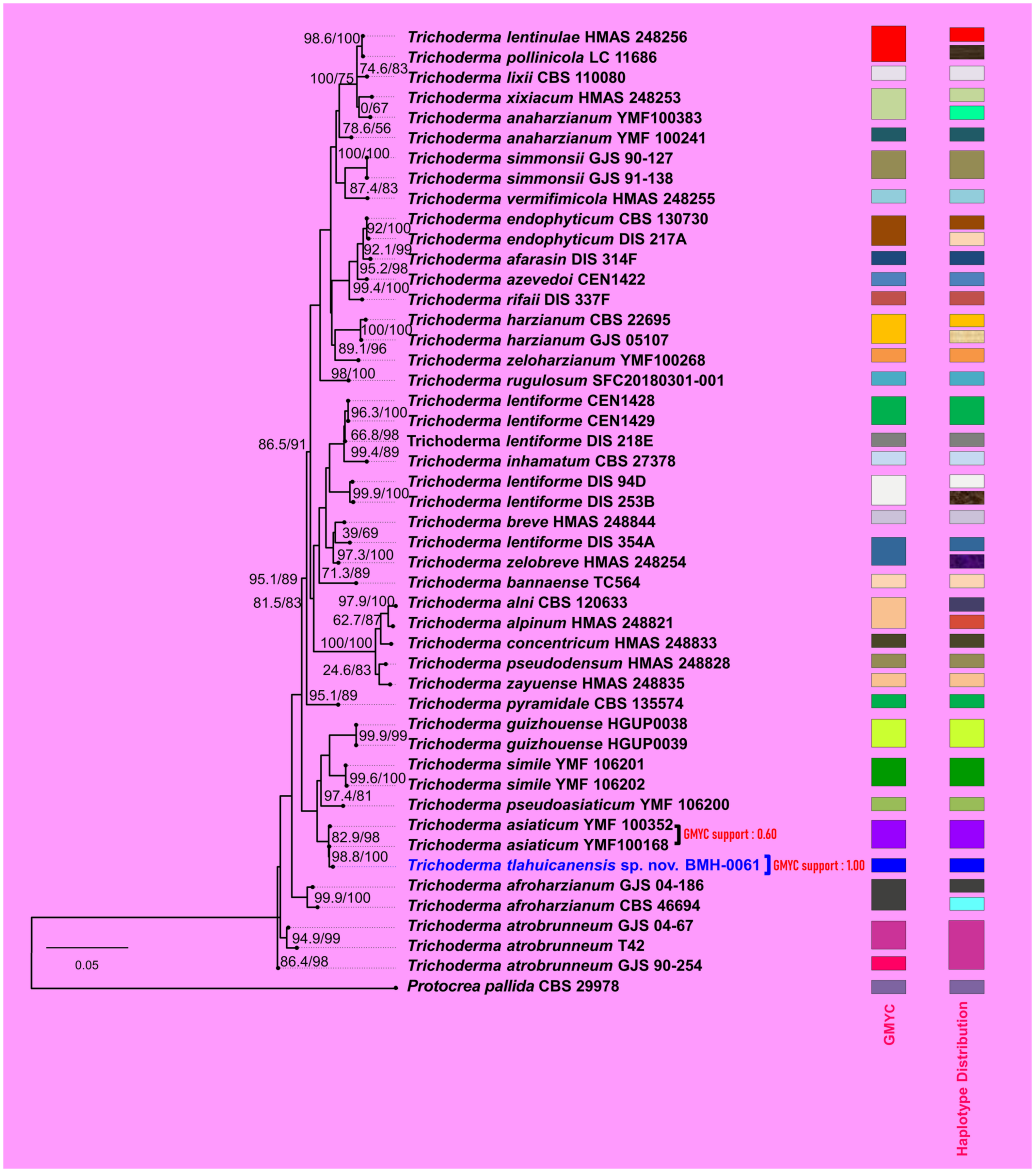
Multilocus phylogeny of *T. tlahuicanensis* BMH-0061. The tree was inferred from concatenated nucleotide sequences of the ITS, RPB2, and TEF1-α markers. GMYC species delimitation test and haplotype distributions are shown as vertical bars on the right-hand side.

We assessed the phylogenetic hypothesis using multiple reconstruction methods. This included an alignment-free, distance-based method applied directly to genome contig sequences using Jolytree, as well as three alignment-based multi-gene approaches, as detailed in the MM section. In both analyses, the results supported the prediction that BMH-0061 occupies a distinct phylogenetic position. In genomic phylogeny, it was placed on a sister branch of *T. simmonsii* (Figure 1b), whereas in MLST-based phylogeny, it appeared on a sister branch to *T. asiaticum* (Figure 2). The observed discrepancies between genome-based and MLST phylogeny are mainly attributable to the inclusion of species in the MLST analyses for which genome sequences were not available at the time of writing this paper.

BHM-0061 is most closely related to *T. simmonsii*, with a branch length of 0.0123, while its distance to *Trichoderma* sp. IMV_00454 (GCA_001931985.1 – also belonging to the species *T. simmonsii*) is slightly greater at 0.0125. This clade is nested within a larger group comprising several *Trichoderma* species, indicating a shared evolutionary history. The branch support values confirm the reliability of this grouping. Overall, the phylogenetic placement of BHM-0061 suggests that it is a distinct strain with close affinities within *Harzianum*/*Virens* clades, supporting its characterization as a novel species. Interestingly, compared to species such as *T. azevedoi*, reported from Brazilian crop soils and recognized for its effectiveness in the biocontrol of white mold in beans, or *T. endophyticum* and *T. rifaii*, commonly isolated as endophytes from tropical trees, *T. tlahuicanensis* occupies a phylogenetically distant position in the MLST tree. Nonetheless, its placement suggests that biological control traits are evolutionarily conserved within this lineage, indicating that further diversification in this clade may reveal additional taxa with valuable biotechnological potential (Chaverri et al., 2015; Inglis et al., 2020).

To explore the potential species boundaries of the strain BMH-0061, we carried out two delimitation tests, the Bayesian Poisson Tree Processes (bPTP) and the Generalized Mixed Yule Coalescent (GMYC) approaches. These tests were conducted under former observations that BMH-0061 has closely related neighbors with high genomic coherence values, while still remains genetically distinct as demonstrated by kmers frequency data and phylogenetics. High Bayesian support values for species delimitation of BMH-0061 were obtained across multiple tools (e.g., bPTP and GMYC models both achieving 0.94 and 1.00, respectively). Similarly, the Bayesian speciation test on the MLST phylogeny is strongly supported for the BMH-0061 branch (Bayesian support value for GMYC = 1.00). This provides strong evidence for the unique phylogenetic position of BMH-0061, underscoring its potential status as an independently evolving lineage as supported by diverse phylogenetic and evolutionary approaches.

Further characterization of the genetic diversity underlying the multilocus phylogenetic patterns, revealed that nucleotide diversity across the concatenated alignment was moderate (*π* = 0.0262), with 174 segregating sites, of which 122 were parsimony-informative. The analysis identified 41 haplotypes with a high haplotype diversity (Hd = 0.9929), indicating a broad distribution of unique sequence variants within the dataset (Supplementary Table S3). Notably, strain BMH-0061 was identified as an independent haplotype (Figure 2), a finding that aligns with its strong phylogenetic support and suggests it may represent a divergent lineage as a separate species. These values reflect a substantial level of genetic variation and support the phylogenetic resolution provided by this multilocus dataset. Tajima’s D was negative (D = −0.618), suggesting a slight excess of low-frequency polymorphisms; however, the result was not statistically significant (*p* = 0.713), implying no strong deviation from neutral evolution. Overall, the ITS–RPB2–TEF combination reveals a genetically diverse but evolutionarily neutral structure for this group of sequences.

This study provides integrated genomic, phylogenetic, and taxonomic evidence supporting the classification of *Trichoderma* sp. BMH-0061 as a distinct lineage within the genus. Genome assembly, genomic coherence hypothesis testing, phylogenetic analyses, Bayesian speciation tests, and measures of genetic diversity collectively confirm its unique evolutionary position. This evidence indicates that it likely constitutes a new species, for which we propose the name *Trichoderma tlahuicanensis* sp. nov., in honor of the ancient Tlahuica people, who inhabited the region now known as the State of Morelos, between 1100 A.D. and 1521. These findings provide a foundation for further functional and ecological studies, including future transcriptomic and metabolomic analyses to explore potential adaptations and biological significance (Contreras-Cornejo et al., 2024).

### 3.4 Comparative genomic analysis of major insertions in BMH-0061

Table 4 summarizes major insertion events (SNVs) detected in the BMH-0061 genome compared to the *Trichoderma harzianum* closest reference genome (GCA_019097725.1). Overall, GSAlign identifies 690,135 SNVs, 31,529 insertions and 39,932 deletions (Supplementary Table S4). An examination of the data confirmed at least 14 insertions larger than 31 bp, a typical kmer size threshold used in *de novo* genome assembly and variant detection. These insertions may be particularly relevant because they are more likely to introduce structural complexity in genome structure and gene function.

**Table 4:** Characterization of major sequence insertions and associated genes in BMH-0061 compared to *T. harzianum* reference genome.

#CHROM	POS	REF	ALT-INSERTION	INFO	Accession	Function
CM032588.1	693243	C	CAGTCATGGCCTCAACCACAACGCATCTGCATCTAGATGGTCT	TYPE = INSERT	QYS95571.1	Translation factor GUF1, mitochondrial/fidelity factor of mitochondrial protein synthesis (translation, ribosomal structure and biogenesis)
CM032588.1	1014012	t	ttcgtcttcgtcctttgccttgtcttcgtct	TYPE = INSERT	QYS95678.1	Hypothetical protein
CM032588.1	3932529	C	CCGACAACAAGTCAGAGAGACTGTTAATCAACAATACCAACAGAT	TYPE = INSERT	QYS96675.1	Hypothetical protein
CM032588.1	4294894	G	GGCTCGGACGCACTACGTGAGCCGGCTACGAGTGTTTTGAGCACCTCGGAAGGACAACAACATCAGCAA	TYPE = INSERT	QYS96808.1	Zn(2)-C6 fungal-type domain-containing protein fungal transcriptional regulatory proteins/regulation of the rate of DNA-templated transcription
CM032588.1	6884912	C	CTACACGACGAGCTGTGGCGCAGCGAGATCAGAATCGCGAGCGAGACGATGCT	TYPE = INSERT	QYS97733.1	Phosphatidylglycerophosphate synthase
						Phospholipid biosynthesis
CM032591.1	1709627	C	CTTGGACCTATCCCAGACTTGGACCTATCCCAGAC	TYPE = INSERT	QYT02427.1	Hypothetical protein
CM032591.1	2509827	A	ACCATTGAGAAAGTAAAGGGAAACGATGATGGACA	TYPE = INSERT	QYT02698.1	Hypothetical protein
CM032593.1	742413	A	ACAACCCCAGGCTCCTCCTGTCACTCAAATCAGCGATGGT	TYPE = INSERT	QYT05221.1	Hypothetical protein
CM032593.1	742452	T	TGGccagcctcaagctcctccCGTTACTCAAATCAGCGAC	TYPE = INSERT	QYT05221.1	Hypothetical protein
CM032593.1	1045489	A	AGAAGTCTACTGAGCTCCTGATCCGCAAGCTACCCTTCCAGCGTCTNGGTAAGAAcacgaagccatcaccacccaCTGATCGTCGCNNNTATGTTCCACAGCCAACAAGCCTCACCGTTATAAACCTGATACCGTCGCCCTCCGTGAGATTCGTCGATACCA	TYPE = INSERT	QYT05335.1	Histone H3 chromatin remodeling and transcriptional regulation during development
CM032593.1	1052881	A	AGGAGATCGCCGCCAGTTGTTGTGTTTGTCAGATTCCCAATTACTCCTCCAAGGTCGGTGTTATTCAATAGTCCCCCTGTTCCAACACCAAGAGTACTGTTCAATAGACCTTCTGTTCCTGCTCCTAATCCGCCAGTCGCTGTGGAGTTGTCAGGGGTCCCAGGTCCTAATAATCCTCCCA	TYPE = INSERT	QYT05335.1	Histone H3 nucleosome assembly/microdiversity of chromatin/convert “closed” chromatin, where transcription is disfavored, into “open” chromatin that is favorable for transcription
CM032593.1	1855174	g	gatttttgcttttgcttcttcaagtcTTTTTCGAGCTTATCCGCgt	TYPE = INSERT	QYT05340.1	Hypothetical protein
CM032593.1	2369775	G	GGCCCTCATCCATATGGCCCTCATCCATATA	TYPE = INSERT	QYT05648.1	GPI inositol-deacylase, lipid transport, and metabolism
CM032593.1	3703706	G	gttgttgttgatgctgatgacgaagttgatgatg	TYPE = INSERT	QYT05811.1	Hypothetical protein

These insertions span multiple chromosomes and affect a variety of genomic regions, including both functionally characterized genes and hypothetical proteins. Notably, insertions were identified in genes related to essential cellular processes such as the mitochondrial translation fidelity factor GUF1, critical for stress tolerance like temperature-sensitive growth under nutrient-limiting conditions (Bauerschmitt et al., 2008), phosphatidylglycerophosphate synthase (involved in phospholipid biosynthesis), and a Zn(2)-C6 fungal-type transcription factor, potentially impacting gene regulatory networks. The presence of insertions in histone H3 genes suggests possible epigenetic remodeling capacity, which may enhance chromatin plasticity and transcriptional responsiveness to environmental stimuli. Other target genes are involved in lipid metabolism and membrane structure, such as GPI inositol-deacylase. The size and complexity of some insertions (e.g., >50 bp) suggest possible recombination events or the influence of mobile elements. The distribution and nature of these insertions imply that BMH-0061 harbors both conserved and divergent genomic regions, potentially reflecting adaptive evolution and functional specialization related to stress tolerance and environmental resilience.

### 3.5 Phenotypic analysis

To better know BMH-0061 characteristics, the relative growth rate was analyzed at three different temperatures during 5 days in PDA media.

**Figure 3:**
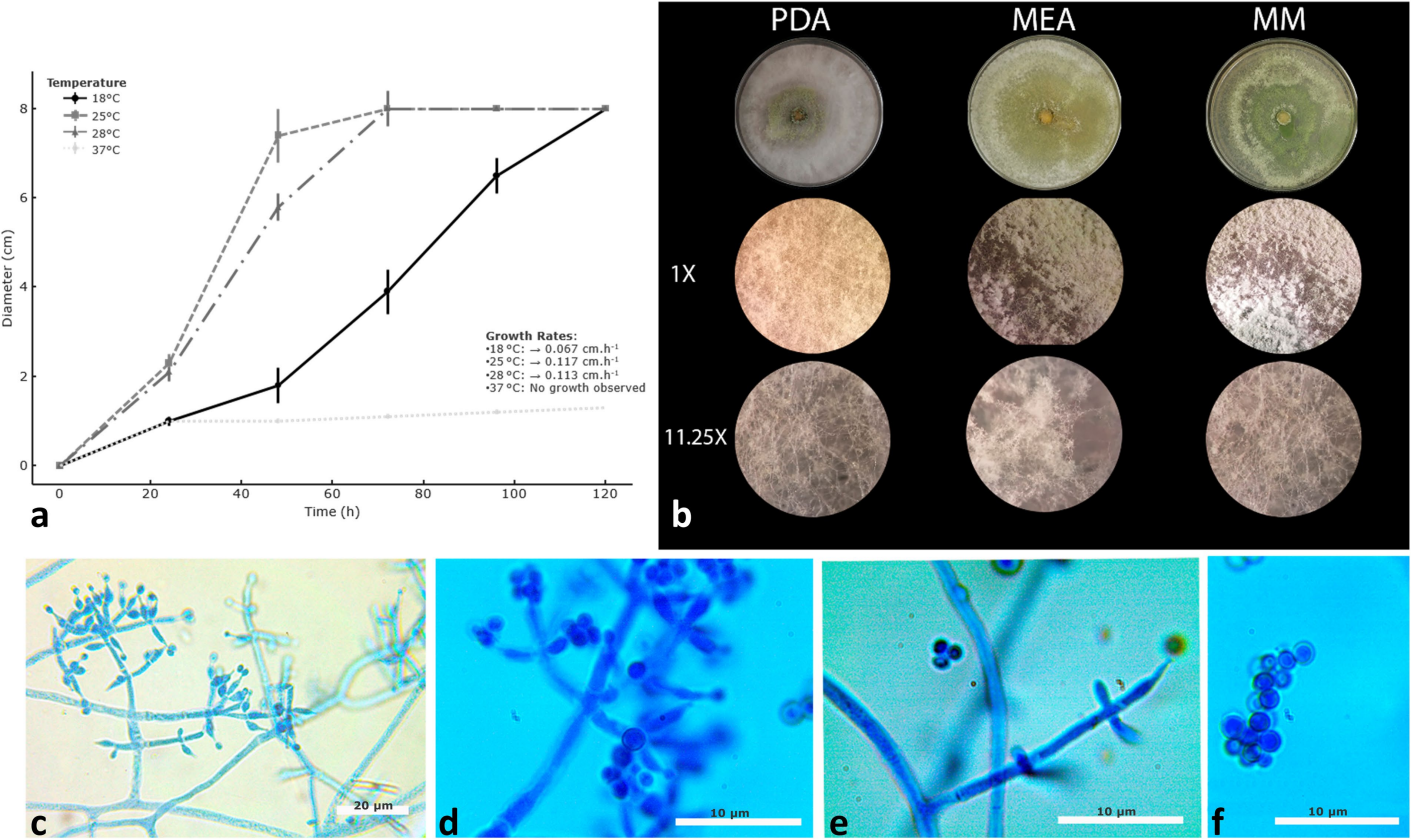
Growth performance, colony morphology, and microscopic features of *T. tlahuicanensis* BMH-0061. Relative growth rates were assessed at 18 °C, 25 °C, 28 °C **(a)**. Colony morphology after 48 h at 25 °C **(b)**. Microscopic features showing conidiophores with multiple ampulliform phialides **(c)**, globose to subglobose conidia **(d)**, phialides **(e)**, and conidia **(f)**.

As shown in Figure 3a, the highest growth rates were recorded at 25 and 28 °C, with colony development rates of 0.117 cm/h and 0.113 cm/h, respectively. At 18 °C, growth was markedly slower, with a rate of 0.067 cm/h. No growth was observed at 37 °C. At 48 h, colony diameters reached 7.3 cm on PDA and 4.9 cm on MM at 25 °C, confirming that BMH-0061 grows best at this temperature. These results align with the growth optima reported for phylogenetically related species such as *T. simmonsii* and *T. asiaticum*, which exhibit optimal growth at 30 °C and 25 °C, respectively (Chung et al., 2021; Zheng et al., 2021). The analysis of morphological characteristics of *Trichoderma* BMH-0061 showed that on PDA the colonies are granulated, white in the surroundings and olive green in the center with abundant ramified aerial mycelia. On MEA, colonies exhibit a more pigmented yellow-green surface with concentric zones. On MM, colonies are greener, with a denser aerial mycelium and slightly irregular margins (Figure 3b). No pigment diffused nor particular odor was perceived.

Microscopic examination revealed typical asexual reproductive structures consistent with the genus. Conidiophores were branched and verticillate, bearing multiple phialides (6 ± 1.5 μm long × 2 ± 0.2 μm wide) (Figure 3c). These phialides were ampulliform and produced globose to subglobose conidia arranged in compact heads (Figures 3c,d). Some conidiophores displayed solitary phialides and acropetal conidiogenesis (Figures 3d,e). The conidia were smooth-walled, hyaline, and globular or semiglobular in shape, typically forming short chains (Figure 3f).

### 3.6 Taxonomy

*Trichoderma tlahuicanensis* Iza-Arteaga et al., sp. nov. *Typification*: Fungal Names: Holotype deposited under accession FN571825 (Figures 3b–f), collected in Cuernavaca, Morelos, Mexico, as endophyte of *Capsicum annuum* (chili), 2020, type: BMH-0061.

The Holotype is deposited in the Laboratory of Molecular Biology of Fungi, Biotechnology Research Center, Autonomous University of the State of Morelos (CEIB, UAEM), Mexico, under the accession code: BMH-0061. Associated GenBank accession numbers: ITS = OR710780; *tef1* = OR711908; *rpb2* = OR711907.

#### Etymology

“tlahuicanensis”; is originally from Tlahuica or related to Tlahuica. The term “tlahuicanensis”; refers to an indigenous ethnic group that inhabited the region that is now the state of Morelos, Mexico, during the pre-Hispanic era and is the place of the first isolation of the type strain. Holotype BMH-0061, isolated as an endophyte from chili plants (*Capsicum annuum*), in “Milpa” crops in Cuernavaca, Morelos, Mexico, September 2020, preserved in 15% glycerol in a metabolically inactive state, at the Biotechnology Research Center, Autonomous University of the State of Morelos.

#### Culture characteristics

*Colonies on PDA*: Optimum growth temperature at 25 °C, 74 mm after 48 h, 59 mm at 28 °C, no growth at 37 °C. At 25 °C mycelium mostly on surface, white aerial, olive-green sporulation, beginning in the colony center with the formation of concentric rings; no pigmentation in the medium; no distinctive smell. The formation of concentric rings suggests that this strain is responsive to circadian cycles (Henríquez-Urrutia et al., 2022).

*Colonies on MEA*: Optimum growth temperature at 25 °C, 64 mm after 48 h, 60 mm at 28 °C, no growth at 37 °C; mycelium mainly hyaline, low with olive-green sporulation, no formation of concentric rings.

*Colonies on MM*: Optimum growth temperature at 25 °C, 49 mm after 48 h, 59 mm at 28 °C, no growth at 37 °C. Mycelium hyaline and smooth, low with olive-green sporulation; absence of concentric rings and no pigmentation; conidia are mostly globose to subglobose, with dimensions ranging from 2 to 2.5 μm in width and 2 to 2.8 μm in length. The conidiophores develop as branches with a terminal whorl of multiple phialides. The average dimensions of the phialides are 6 ± 1.5 μm in length and 2 ± 0.2 μm in width. Chlamydospores were not observed in these assays on PDA.

*Notes*: *Trichoderma tlahuicanensis* is phylogenetically close to *T. simmonsii* and *T. asiaticum* in the Harzianum/Virens clade. Chlamydospores are not a prominent feature in members of the Harzianum/Virens clade. Although *T. tlahuicanensis* shares the same optimal growth temperature as *T. asiaticum*, it is completely different from *T. simmonsii*. Morphologically, *T. tlahuicanensis* exhibits abundant aerial mycelium, whitish and greenish granular colonies, loose, arachnoid aerial hyphae with evident branching. No diffusible pigment is observed, and no distinctive odor is detected on PDA medium.

*Trichoderma tlahuicanensis* BMH-0061 can be distinguished from its closest relatives, *T. simmonsii* and *T. asiaticum*, based on a combination of genetic, physiological, and morphological characteristics. Genomic comparisons revealed that BMH-0061 shares borderline levels of genetic coherence with its closest relatives. With *T. simmonsii*, it exhibits ANI (94.38%) and AAI (94.65%) values that suggest recent divergence and place it at the interface between genomic cohesion and a continuum of diversity. In contrast, *T. asiaticum* shows the closest multilocus genetic distance (ITS–RPB2–TEF1: 0.0034), consistent with an early stage of speciation. However, BMH-0061 can still be distinguished from both taxa: it differs from *T. simmonsii* in genome organization and genetic content, sharing less than 82% of hexamer composition; and from *T. asiaticum* by its placement in multilocus phylogenies and independent evolutionary trajectory. BMH-0061 is a sister lineage to *T. simmonsii* in whole-genome-based tree and to *T. asiaticum* in multi-locus phylogeny, species delimitation models (bPTP and GMYC and haplotype diversity) strongly supported BMH-0061 as a separate evolutionary entity.

Physiologically, *T. tlahuicanensis* exhibits optimal growth at 25 °C, similar to *T. asiaticum* but clearly distinct from *T. simmonsii* (30 °C), and is unable to grow at 37 °C. Its conidiophores are branched and verticillate, bearing ampulliform phialides (6 ± 1.5 μm × 2 ± 0.2 μm) and globose to subglobose, smooth-walled, hyaline conidia (2–2.8 μm). Chlamydospores were not observed, a feature consistent with members of the Harzianum/Virens clade. Together, these characteristics confirm the distinctiveness of *T. tlahuicanensis* as a novel species.

#### Ecology

*Trichoderma tlahuicanensi*s is a root endophyte of chili (*Capsicum annuum*) grown in the traditional milpa polyculture system of central Mexico. Within this biodiversity-rich agroecosystem, the species likely benefits from and contributes to plant–microbe interactions. It functions as a versatile phosphorus solubilizer across contrasting soil types (Alfisol, Vertisol, and Andisol), efficiently mobilizing inorganic phosphorus (rock phosphate) through the production of organic acids (Li et al., 2015; Zúñiga-Silgado et al., 2020). This trait, together with its antagonism against phytopathogens, suggests an ecological role in enhancing host nutrition and resilience, consistent with endophytic strategies observed across the genus.

### 3.7 Antagonistic analysis

**Figure 4:**
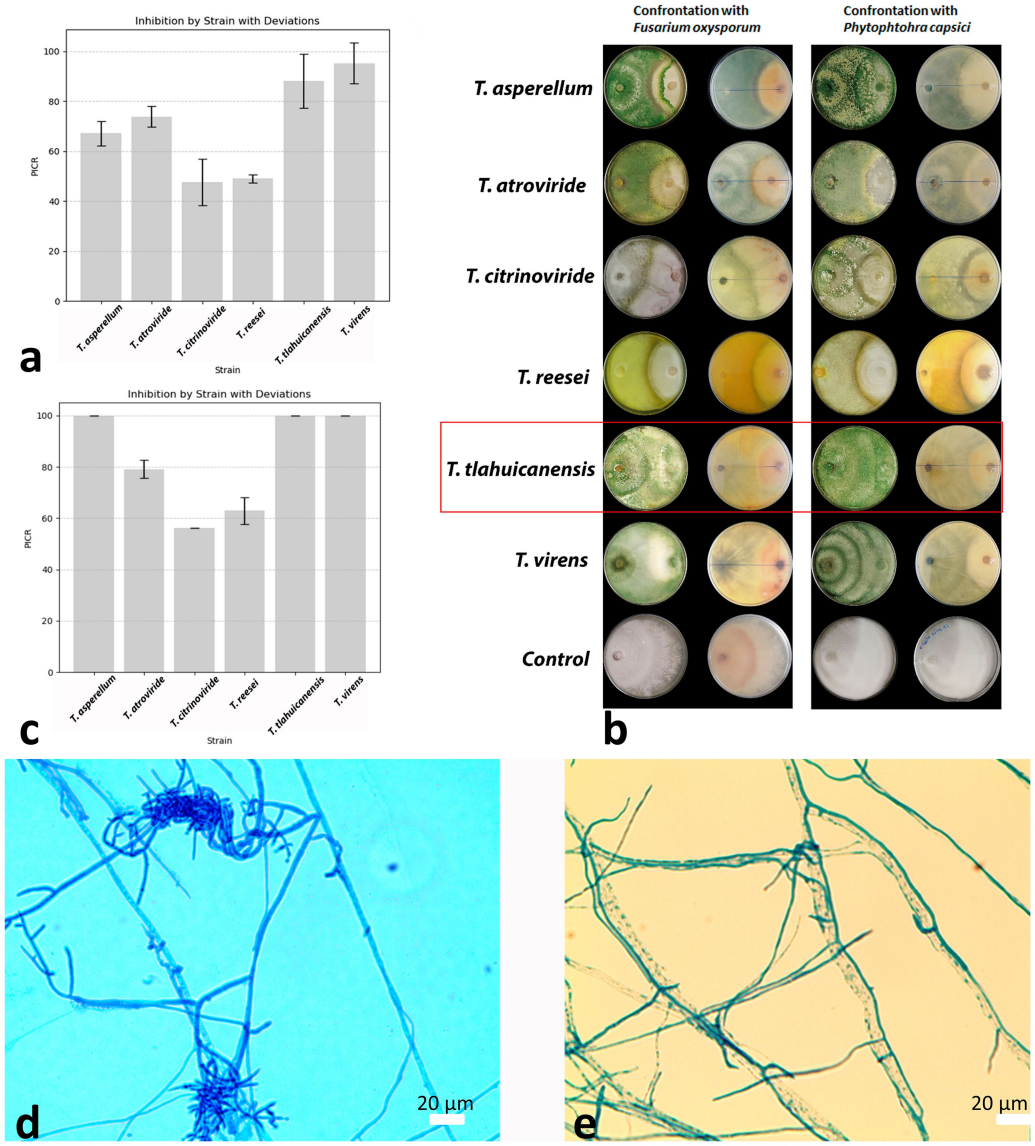
Antagonistic activity and mycoparasitism of *T. tlahuicanensis* BMH-0061 **(4a–c)**. Inhibition of *F. oxysporum* **(a)** and *P. capsici* growth **(c)**. Microscopic observations of the BMH-0061 hyphae coiling around pathogen hyphae and forming haustoria **(d,e)**.

The results of antagonistic assays are shown in Figures 4a–c. As can be seen, not all *Trichoderma* species have the same effect on *F. oxysporum* and *P. capsici* growth, but strain BMH-0061 had an excellent behavior antagonizing both phytopathogenic fungi. The growth of *P. capsici* was 100% inhibited by *Trichoderma* sp. BMH-0061 as well as *T. virens* and *T. asperellum* did. Similar results were reported by Andrade-hoyos (2019), they found that several *Trichoderma* strains inhibited the growth of *P. capscisi.* The antagonistic effects of *T. tlahuicanensis* BMH-0061 against *F. oxysporum* were also remarkable. In dual culture assays, growth inhibition averaged 88 ± 10.9% (*n* = 3 biological replicates), which was statistically similar to the inhibition levels observed for *T. virens* and *T. asperellum* (95% ± 8.2% and 67 ± 4.9%, respectively [ANOVA one way, followed by Tukey’s multiple comparison test (*p* ≤ 0.05)]. For comparison, previous reports indicated that *T. virens* and *T. harzianum* inhibit *F. oxysporum* and *F. subglutinans* by approximately 60%, supporting their potential as biocontrol agents (Michel-Aceves et al., 2019).

We also explored microscopically the mycoparasitism ability of *Trichoderma tlahuicanensis* BMH-0061 on some pathogenic fungi: a sample of the region of contact between the two fungi from the antagonistic assay was analyzed under the microscope. As can be seen in Figures 4d,e, *Trichoderma* BMH-0061 hyphae can roll over *F. oxysporum* and *P. capsici* hyphae and form haustoria. These results demonstrate that *Trichoderma* sp. BMH-0061 like other *Trichoderma* species, has the ability to inhibit the growth of phytopathogenic fungi.

We conclude then that *T. tlahuicanensis* is a new, undescribed species with great biotechnological potential. It proved to be a good mycoparasite due to high levels of fungal phytopathogens growth inhibition. Also, in a previous paper, we described that it showed a very efficient Phosphorous solubilization and mineralization abilities that can be important to plant growth promotion (Zúñiga-Silgado et al., 2020).

## Data Availability

The datasets presented in this study can be found in online repositories. The names of the repository/repositories and accession number(s) can be found in the article/Supplementary material.

## Footnotes

1
https://github.com/rrwick/Porechop

2
https://github.com/rrwick/Filtlong

3
https://github.com/dparks1134/CompareM

4
https://github.com/ayixon/Fast-Fungal-Genome-Classifier

5 https://github.com/zhangjiajie/PTP; https://github.com/iTaxoTools/GMYC-pyqt5

6 www.ncbi.nlm.nih.gov/datasets/genome/?taxon=5543&reference_only=true&typical_only=true&exclude_mags=true.
