# Supplementary material for: Trichoderma tlahuicanensis sp. nov. (Hypocreaceae), a novel mycoparasite of Fusarium oxysporum and Phytophthora capsici isolated from a traditional Mexican milpa
Source: Front Microbiol. 2025 Nov 27;16:1714079. doi: 10.3389/fmicb.2025.1714079 (PMC12696181; doi:10.3389/fmicb.2025.1714079)
Supplement: Supplementary file 1 [file Supplementary Material_all.pdf › Supplementary Material_all.pdf]

Supplementary Material for the paper:

***Trichoderma tlahuicanensis* sp. nov. (*Hypocreaceae*), an undescribed mycoparasite and plant growth promoting species of the genus *Trichoderma* isolated from a "milpa" in Mexico.**

Mario Iza-Arteaga[1], Verónica Lira-Ruan[2], Edgar Balcázar-López[3], Jorge Luis Folch-Mallol[1*] and Ayixon Sánchez-Reyes[4*].

[1]Centro de Investigación en Biotecnología. Universidad Autónoma del Estado de Morelos. Av. Universidad No. 1001, Col Chamilpa, Cuernavaca, Morelos, México. C.P. 62209. jordi@uaem.mx (JLFM)

[2]Centro de Investigación en Dinámica Celular. Instituto de Investigación en Ciencias Básicas y Aplicadas. Universidad Autónoma del Estado de Morelos. Av. Universidad No. 1001, Col Chamilpa, Cuernavaca, Morelos, México. C.P. 62209, katlira@uaem.mx

[3]Departamento de Farmacobiología, Centro Universitario de Ciencias Exactas e Ingeniería, Universidad de Guadalajara, Blvd. M. García Barragán # 1451, Guadalajara 44430, Jalisco C.P., México. edgar.balcazar@academicos.udg.mx

[4]Investigador por México, SECIHTI-Instituto de Biotecnología. Universidad Nacional Autónoma de México. Ave. Universidad 2001, Col. Chamilpa 62210. Cuernavaca Morelos, México ayixon.sanchez@ibt.unam.mx

*Correspondence: *ayixon.sanchez@ibt.unam.mx*, *jordi@uaem.mx*

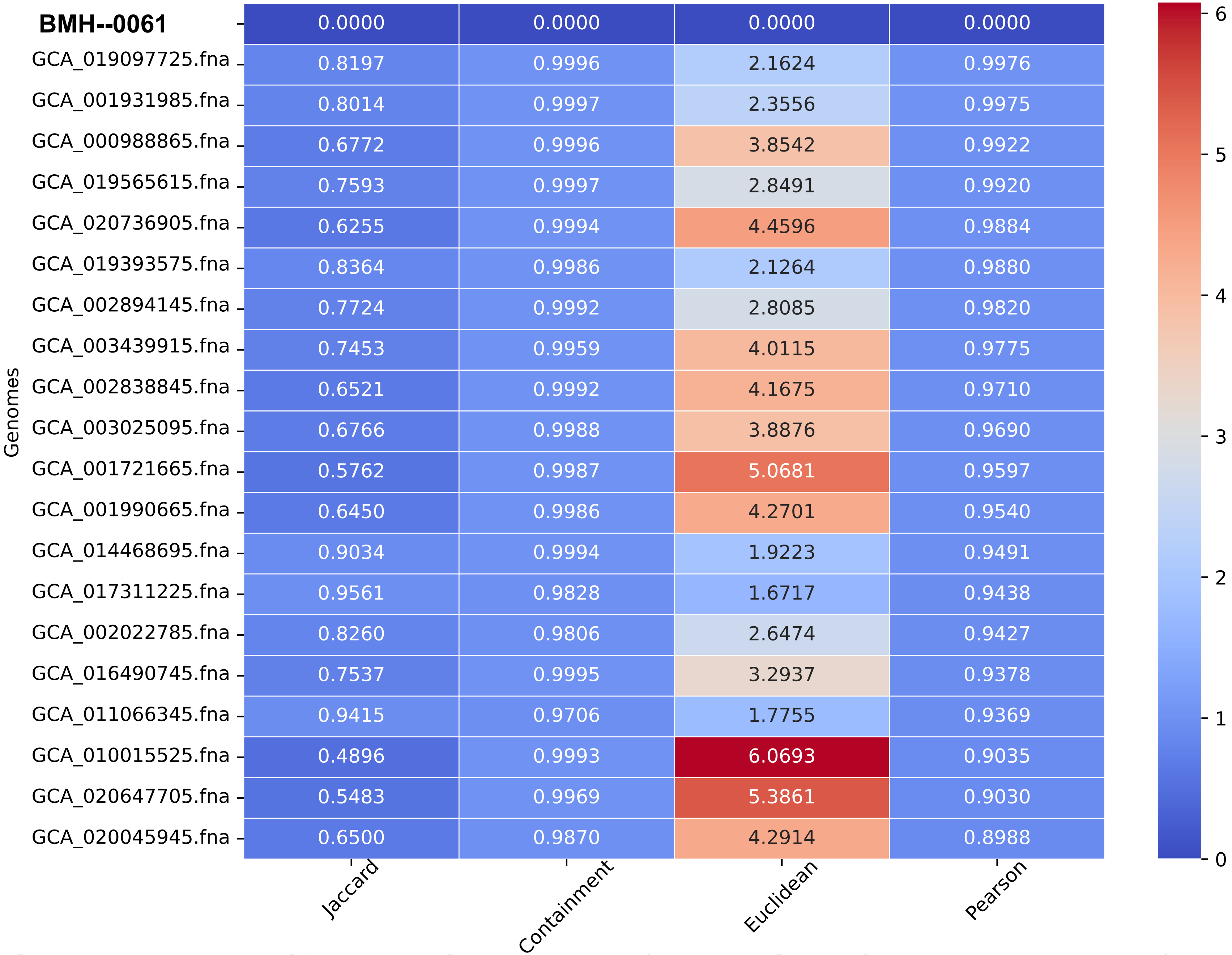

Supplementary Figure S1. Hexamer Similarity Matrix (Including Query, Ordered by Jaccard Index)

```
=======================================================
Mantel test - Method: Spearman
=======================================================
Mantel statistic based on Spearman's rank correlation rho
Mantel statistic r: 0.11
     Significance: 0.034

Upper quantiles of permutations (null model):
  90%    95%  97.5%    99%
0.0774 0.0977 0.1214 0.1474
Number of permutations: 999

=======================================================
Mantel test - Método: Kendall
=======================================================
Mantel statistic based on Kendall's rank correlation tau
Mantel statistic r: 0.09
     Significance: 0.046

Upper quantiles of permutations (null model):
  90%    95%  97.5%    99%
0.0603 0.0835 0.1052 0.1280
Number of permutations: 999
```

Supplementary Figure S2. Mantel test comparing the matrix of genetic distances and geographic origins among Trichoderma strains

Supplementary Table S1. Telomeric repeats (TTAGGG/CCCTAA) found in the genome of *Trichoderma tlahuicanensis* sp. nov. BMH-0061

JARWZN010000001.1 Trichoderma sp. BMH	0061 Scaffold1, whole genome shotgun sequence Reverse (end of sequence) AGGGTTAGGGTTAGGGTTAGGAGTTAGGGTTAGGGTTAGGGTTAGGGTTA
JARWZN010000002.1 Trichoderma sp. BMH	0061 Scaffold2, whole genome shotgun sequence Reverse (end of sequence) tagggttagggttagggttagggttagggttagggttagggttagggtta
JARWZN010000003.1 Trichoderma sp. BMH	0061 Scaffold3, whole genome shotgun sequence Reverse (end of sequence) tagggttagggttagggttagggttagggttagggttagggttagggtta
JARWZN010000004.1 Trichoderma sp. BMH	0061 Scaffold4, whole genome shotgun sequence Forward (start of sequence) CCCTAACCCCTAACCCTAacctaaccctaaccctaaccctaaccctaacc
JARWZN010000006.1 Trichoderma sp. BMH	0061 Scaffold6, whole genome shotgun sequence Forward (start of sequence) TAACCCCTAACCTAacctaaccctaaccctaaccctaaccctaaccctaa
JARWZN010000006.1 Trichoderma sp. BMH	0061 Scaffold6, whole genome shotgun sequence Reverse (end of sequence) agggttagggttagggttagggttagggttagggttagggttagggttag
JARWZN010000007.1 Trichoderma sp. BMH	0061 Scaffold7, whole genome shotgun sequence Forward (start of sequence) aaccctaaccctaaccctaacaCCTAacctaaccctaaccctaaccctaa
*Telomeres found: 7 (3 forward, 4 reverse)	

Supplementary Table S2. KEGG orthologue annotation of the BMH-0061 genome

gene Id		UniRef100	KO	e value	bit score
augustus	g2.t1	UniRef100_	K01130	5.00E-233	812.4
augustus	g3.t1	UniRef100_	K08138	1.20E-42	179.5
augustus	g5.t1	UniRef100_	K00128	1.80E-115	421.4
augustus	g6.t1	UniRef100_	K17746	8.10E-51	205.7
augustus	g18.t1	UniRef100_	K22213	2.20E-53	214.5
augustus	g21.t1	UniRef100_	K00108	3.10E-196	689.9
augustus	g24.t1	UniRef100_	K00218	6.60E-55	219.5
augustus	g26.t1	UniRef100_	K06978	2.60E-110	404.4
augustus	g37.t1	UniRef100_	K00344	2.20E-109	400.6
augustus	g44.t1	UniRef100_	K15385	5.90E-76	289.7
augustus	g45.t1	UniRef100_	K15394	0.00E+00	2637.4
augustus	g46.t1	UniRef100_	K17645	4.60E-78	297.4
augustus	g47.t1	UniRef100_	K03929	8.60E-45	186.8
augustus	g52.t1	UniRef100_	K00108	2.10E-53	215.3
augustus	g54.t1	UniRef100_	K01312	3.70E-88	329.7
augustus	g64.t1	UniRef100_	K01126	2.20E-104	383.6
augustus	g65.t1	UniRef100_	K08141	2.70E-156	557.4
augustus	g74.t1	UniRef100_	K00218	8.50E-61	239.2
augustus	g76.t1	UniRef100_	K00354	5.90E-199	698.4
augustus	g79.t1	UniRef100_	K01539	0.00E+00	1181.8
augustus	g80.t1	UniRef100_	K08141	1.30E-239	833.9
augustus	g83.t1	UniRef100_	K01811	0.00E+00	1091.3
augustus	g84.t1	UniRef100_	K00276	0.00E+00	1101.7
augustus	g99.t1	UniRef100_	K11788	5.70E-153	545.8
augustus	g102.t1	UniRef100_	K01279	7.00E-178	629.4
augustus	g105.t1	UniRef100_	K05349	0.00E+00	1249.6
augustus	g110.t1	UniRef100_	K13953	9.50E-84	315.5
augustus	g111.t1	UniRef100_	K11153	4.60E-31	140.6
augustus	g118.t1	UniRef100_	K05986	6.00E-57	226.5
augustus	g123.t1	UniRef100_	K07078	1.80E-97	360.5
augustus	g124.t1	UniRef100_	K15419	0.00E+00	1312.7
augustus	g134.t1	UniRef100_	K01381	9.60E-86	322.4
augustus	g135.t1	UniRef100_	K15394	0.00E+00	2629.4
augustus	g137.t1	UniRef100_	K03233	4.50E-59	233
augustus	g146.t1	UniRef100_	K03418	2.10E-109	401.7
augustus	g148.t1	UniRef100_	K00272	2.40E-57	227.6
augustus	g150.t1	UniRef100_	K08141	2.40E-89	334.7
augustus	g151.t1	UniRef100_	K05882	3.10E-55	221.1
augustus	g152.t1	UniRef100_	K00826	2.40E-107	393.7
augustus	g155.t1	UniRef100_	K09456	9.60E-61	240
augustus	g164.t1	UniRef100_	K11162	1.30E-22	112.5
augustus	g166.t1	UniRef100_	K00988	2.70E-101	373.6
augustus	g171.t1	UniRef100_	K15920	0.00E+00	1467.6
augustus	g172.t1	UniRef100_	K05349	0.00E+00	1319.3
augustus	g187.t1	UniRef100_	K01046	4.20E-63	247.3
augustus	g190.t1	UniRef100_	K21159	7.80E-51	206.5
augustus	g198.t1	UniRef100_	K17754	7.00E-28	129.8
augustus	g199.t1	UniRef100_	K07248	1.40E-69	268.9
augustus	g201.t1	UniRef100_	K01915	2.40E-219	766.5

augustus	g202.t1	UniRef100_K19564	9.60E-242	840.9
augustus	g207.t1	UniRef100_K22134	6.40E-119	433
augustus	g212.t1	UniRef100_K01192	0.00E+00	1755
augustus	g217.t1	UniRef100_K08192	9.20E-32	142.9
augustus	g223.t1	UniRef100_K11808	1.40E-306	1056.6
augustus	g239.t1	UniRef100_K03790	8.80E-09	65.9
augustus	g240.t1	UniRef100_K13953	1.10E-84	318.5
augustus	g243.t1	UniRef100_K01210	1.20E-208	731.9
augustus	g247.t1	UniRef100_K19639	2.60E-63	247.7
augustus	g249.t1	UniRef100_K00108	7.70E-82	310.1
augustus	g250.t1	UniRef100_K00826	9.00E-116	421.8
augustus	g254.t1	UniRef100_K08711	0.00E+00	1682.9
augustus	g269.t1	UniRef100_K01050	4.70E-80	303.9
augustus	g281.t1	UniRef100_K03293	5.20E-274	948.3
augustus	g285.t1	UniRef100_K06016	8.60E-79	299.7
augustus	g294.t1	UniRef100_K08141	4.60E-242	842
augustus	g300.t1	UniRef100_K00218	8.10E-39	166.4
augustus	g303.t1	UniRef100_K01426	1.80E-238	830.1
augustus	g307.t1	UniRef100_K17644	2.80E-75	287
augustus	g313.t1	UniRef100_K01889	1.70E-234	817
augustus	g314.t1	UniRef100_K13137	1.90E-178	630.6
augustus	g315.t1	UniRef100_K14018	0.00E+00	1358.6
augustus	g318.t1	UniRef100_K16055	4.60E-276	955.3
augustus	g327.t1	UniRef100_K13335	1.80E-198	696.8
augustus	g330.t1	UniRef100_K17498	3.10E-178	629.8
augustus	g332.t1	UniRef100_K03018	0.00E+00	2802.7
augustus	g334.t1	UniRef100_K01563	1.20E-72	278.5
augustus	g338.t1	UniRef100_K01426	4.40E-261	905.2
augustus	g339.t1	UniRef100_K00276	0.00E+00	1249.6
augustus	g341.t1	UniRef100_K08726	6.70E-96	355.9
augustus	g342.t1	UniRef100_K05916	9.40E-225	784.3
augustus	g344.t1	UniRef100_K08832	1.30E-269	933.7
augustus	g353.t1	UniRef100_K15419	0.00E+00	1192.9
augustus	g357.t1	UniRef100_K12373	0.00E+00	1208.7
augustus	g367.t1	UniRef100_K09522	1.80E-191	673.7
augustus	g368.t1	UniRef100_K18170	3.10E-41	172.9
augustus	g369.t1	UniRef100_K12872	1.60E-177	627.1
augustus	g370.t1	UniRef100_K00559	2.70E-208	729.6
augustus	g371.t1	UniRef100_K00162	1.70E-197	693.7
augustus	g372.t1	UniRef100_K12599	0.00E+00	2219.1
augustus	g373.t1	UniRef100_K12197	6.40E-76	288.9
augustus	g375.t1	UniRef100_K16230	7.50E-132	475.7
augustus	g376.t1	UniRef100_K10998	1.00E-82	312
augustus	g378.t1	UniRef100_K03333	9.30E-141	506.9
augustus	g379.t1	UniRef100_K15385	2.20E-64	251.1
augustus	g381.t1	UniRef100_K01050	1.50E-88	332
augustus	g386.t1	UniRef100_K01176	2.50E-245	852.8
augustus	g389.t1	UniRef100_K08141	1.80E-294	1016.1
augustus	g390.t1	UniRef100_K01182	0.00E+00	1167.1
augustus	g393.t1	UniRef100_K10680	1.80E-73	281.6

augustus	g395.t1	UniRef100_K06901	1.80E-300	1036.2
augustus	g397.t1	UniRef100_K07222	4.30E-87	327.4
augustus	g405.t1	UniRef100_K21295	3.80E-141	506.9
augustus	g411.t1	UniRef100_K00838	2.70E-189	666.8
augustus	g413.t1	UniRef100_K01568	7.60E-254	881.3
augustus	g416.t1	UniRef100_K08165	3.40E-97	360.9
augustus	g428.t1	UniRef100_K15441	9.80E-135	485.7
augustus	g431.t1	UniRef100_K00621	1.00E-95	354.4
augustus	g432.t1	UniRef100_K00065	1.10E-120	438
augustus	g433.t1	UniRef100_K00008	4.00E-157	559.7
augustus	g434.t1	UniRef100_K19634	1.00E-161	574.3
augustus	g435.t1	UniRef100_K08490	3.70E-162	575.9
augustus	g439.t1	UniRef100_K01561	3.50E-69	266.9
augustus	g442.t1	UniRef100_K00728	0.00E+00	1628.2
augustus	g443.t1	UniRef100_K12825	1.80E-229	800.4
augustus	g447.t1	UniRef100_K12832	4.20E-41	171.8
augustus	g450.t1	UniRef100_K03231	4.90E-256	888.3
augustus	g457.t1	UniRef100_K11552	9.70E-134	481.5
augustus	g458.t1	UniRef100_K03372	2.90E-93	347.8
augustus	g459.t1	UniRef100_K01922	9.80E-192	674.5
augustus	g464.t1	UniRef100_K09184	2.80E-306	1056.2
augustus	g465.t1	UniRef100_K06684	6.80E-232	808.5
augustus	g469.t1	UniRef100_K15163	0.00E+00	2430.2
augustus	g470.t1	UniRef100_K20643	2.40E-301	1039.6
augustus	g471.t1	UniRef100_K02979	1.80E-29	132.9
augustus	g476.t1	UniRef100_K01164	0.00E+00	1296.2
augustus	g479.t1	UniRef100_K03681	1.00E-109	401.4
augustus	g481.t1	UniRef100_K14326	5.50E-241	839
augustus	g484.t1	UniRef100_K10706	0.00E+00	3340.1
augustus	g492.t1	UniRef100_K13711	0.00E+00	1327
augustus	g494.t1	UniRef100_K09117	2.50E-60	236.9
augustus	g497.t1	UniRef100_K13955	1.60E-41	175.3
augustus	g498.t1	UniRef100_K06961	4.70E-165	585.5
augustus	g499.t1	UniRef100_K02867	6.50E-79	298.5
augustus	g502.t1	UniRef100_K04078	2.70E-35	153.3
augustus	g504.t1	UniRef100_K07901	3.30E-111	406
augustus	g506.t1	UniRef100_K14575	0.00E+00	1338.6
augustus	g509.t1	UniRef100_K00729	2.50E-207	726.5
augustus	g510.t1	UniRef100_K00511	7.00E-285	984.2
augustus	g511.t1	UniRef100_K01195	7.70E-53	213.8
augustus	g515.t1	UniRef100_K15303	2.50E-90	337.4
augustus	g517.t1	UniRef100_K18550	9.80E-228	794.3
augustus	g518.t1	UniRef100_K20223	0.00E+00	1928.7
augustus	g521.t1	UniRef100_K03509	2.10E-279	966.5
augustus	g523.t1	UniRef100_K06101	4.60E-230	802.7
augustus	g528.t1	UniRef100_K22315	8.90E-125	451.8
augustus	g536.t1	UniRef100_K18726	1.70E-238	830.1
augustus	g538.t1	UniRef100_K12572	0.00E+00	1075.8
augustus	g542.t1	UniRef100_K15116	5.40E-209	731.9
augustus	g544.t1	UniRef100_K07836	7.90E-38	162.5

augustus	g546.t1	UniRef100_K21842	0.00E+00	1916.4
augustus	g547.t1	UniRef100_K17267	0.00E+00	1619.4
augustus	g548.t1	UniRef100_K07556	8.20E-180	634.8
augustus	g550.t1	UniRef100_K03379	1.20E-87	329.3
augustus	g561.t1	UniRef100_K08254	1.80E-219	766.9
augustus	g569.t1	UniRef100_K01720	1.60E-298	1029.6
augustus	g570.t1	UniRef100_K20522	2.10E-108	397.1
augustus	g574.t1	UniRef100_K04392	5.70E-96	355.9
augustus	g583.t1	UniRef100_K06875	4.90E-32	142.5
augustus	g584.t1	UniRef100_K18586	7.20E-134	481.9
augustus	g587.t1	UniRef100_K00485	1.50E-287	993.4
augustus	g596.t1	UniRef100_K00958	0.00E+00	1078.5
augustus	g605.t1	UniRef100_K04098	4.00E-63	246.9
augustus	g606.t1	UniRef100_K15447	2.50E-233	813.1
augustus	g607.t1	UniRef100_K03113	3.10E-44	183.3
augustus	g611.t1	UniRef100_K02350	0.00E+00	2457.9
augustus	g612.t1	UniRef100_K12819	4.00E-218	762.3
augustus	g613.t1	UniRef100_K02997	7.20E-78	295.4
augustus	g614.t1	UniRef100_K02889	1.80E-67	260.8
augustus	g615.t1	UniRef100_K20040	0.00E+00	1473.4
augustus	g616.t1	UniRef100_K03352	0.00E+00	1355.5
augustus	g617.t1	UniRef100_K02266	4.70E-38	162.5
augustus	g618.t1	UniRef100_K10772	6.90E-250	868.6
augustus	g621.t1	UniRef100_K18159	6.00E-124	448.7
augustus	g622.t1	UniRef100_K17801	2.70E-156	556.6
augustus	g624.t1	UniRef100_K14663	2.40E-304	1050
augustus	g630.t1	UniRef100_K00451	1.70E-136	491.1
augustus	g639.t1	UniRef100_K13127	6.20E-110	402.5
augustus	g640.t1	UniRef100_K08343	5.70E-171	605.5
augustus	g647.t1	UniRef100_K01951	1.80E-56	224.6
augustus	g649.t1	UniRef100_K00423	1.30E-206	724.5
augustus	g650.t1	UniRef100_K02987	9.60E-145	517.7
augustus	g655.t1	UniRef100_K08869	0.00E+00	1252.7
augustus	g660.t1	UniRef100_K00993	6.00E-217	758.4
augustus	g663.t1	UniRef100_K00108	3.50E-60	238
augustus	g666.t1	UniRef100_K09680	1.50E-207	727.2
augustus	g669.t1	UniRef100_K11699	0.00E+00	1717.6
augustus	g670.t1	UniRef100_K00102	7.70E-258	894.8
augustus	g671.t1	UniRef100_K14171	7.90E-72	275.4
augustus	g673.t1	UniRef100_K15440	4.90E-171	605.9
augustus	g683.t1	UniRef100_K03540	1.10E-52	211.5
augustus	g684.t1	UniRef100_K12812	1.40E-241	840.1
augustus	g687.t1	UniRef100_K21197	1.10E-235	821.2
augustus	g689.t1	UniRef100_K16075	5.50E-230	802
augustus	g691.t1	UniRef100_K10848	0.00E+00	1676.8
augustus	g694.t1	UniRef100_K03236	1.00E-64	251.5
augustus	g697.t1	UniRef100_K19199	7.00E-139	499.2
augustus	g701.t1	UniRef100_K01426	2.20E-184	650.2
augustus	g703.t1	UniRef100_K14569	0.00E+00	1655.6
augustus	g706.t1	UniRef100_K11566	1.10E-169	601.3

augustus	g710.t1	UniRef100_K17677	0.00E+00	1183.7
augustus	g713.t1	UniRef100_K18551	8.80E-111	404.8
augustus	g714.t1	UniRef100_K15152	8.10E-58	228
augustus	g716.t1	UniRef100_K01166	7.20E-174	614.8
augustus	g719.t1	UniRef100_K01115	0.00E+00	1336.6
augustus	g720.t1	UniRef100_K13996	0.00E+00	1164.1
augustus	g723.t1	UniRef100_K11768	3.50E-266	922.5
augustus	g727.t1	UniRef100_K17428	6.20E-109	398.7
augustus	g728.t1	UniRef100_K17255	2.20E-237	826.6
augustus	g729.t1	UniRef100_K06943	3.0e-310	1068.9
augustus	g730.t1	UniRef100_K00762	4.30E-123	445.7
augustus	g733.t1	UniRef100_K14850	3.50E-183	646.4
augustus	g736.t1	UniRef100_K02729	8.30E-122	441.8
augustus	g737.t1	UniRef100_K16569	0.00E+00	1497.3
augustus	g739.t1	UniRef100_K03107	4.20E-280	968.8
augustus	g741.t1	UniRef100_K04043	0.00E+00	1188.7
augustus	g752.t1	UniRef100_K00852	6.00E-165	585.1
augustus	g753.t1	UniRef100_K14654	6.50E-136	488.4
augustus	g755.t1	UniRef100_K13341	7.20E-195	684.9
augustus	g756.t1	UniRef100_K17679	3.80E-304	1048.5
augustus	g758.t1	UniRef100_K06674	0.00E+00	1763
augustus	g763.t1	UniRef100_K14022	1.60E-50	204.5
augustus	g767.t1	UniRef100_K19476	3.70E-127	459.5
augustus	g769.t1	UniRef100_K11827	3.10E-76	289.3
augustus	g772.t1	UniRef100_K18203	8.40E-156	555.1
augustus	g774.t1	UniRef100_K10396	0.00E+00	1545.8
augustus	g775.t1	UniRef100_K10573	9.10E-78	294.7
augustus	g776.t1	UniRef100_K00626	6.20E-213	745
augustus	g779.t1	UniRef100_K02355	0.00E+00	1491.5
augustus	g781.t1	UniRef100_K07562	4.30E-275	951.8
augustus	g782.t1	UniRef100_K14785	3.10E-116	423.3
augustus	g783.t1	UniRef100_K07248	1.60E-64	251.9
augustus	g785.t1	UniRef100_K00938	1.80E-215	753.4
augustus	g787.t1	UniRef100_K14806	0.00E+00	1271.9
augustus	g789.t1	UniRef100_K21989	0.00E+00	1830.5
augustus	g791.t1	UniRef100_K13535	1.30E-236	823.9
augustus	g794.t1	UniRef100_K11153	7.60E-27	126.3
augustus	g795.t1	UniRef100_K17991	5.80E-45	186.4
augustus	g799.t1	UniRef100_K00555	0.00E+00	1148.7
augustus	g802.t1	UniRef100_K00698	4.80E-150	537
augustus	g805.t1	UniRef100_K22153	3.00E-50	203.4
augustus	g810.t1	UniRef100_K09467	1.10E-174	617.8
augustus	g811.t1	UniRef100_K17796	2.80E-110	403.3
augustus	g812.t1	UniRef100_K12493	2.10E-149	534.3
augustus	g813.t1	UniRef100_K02371	4.40E-46	190.7
augustus	g814.t1	UniRef100_K13941	3.60E-214	749.2
augustus	g815.t1	UniRef100_K12769	1.60E-164	584.3
augustus	g819.t1	UniRef100_K08762	2.00E-23	113.2
augustus	g820.t1	UniRef100_K20048	6.90E-207	724.9
augustus	g821.t1	UniRef100_K01823	5.70E-128	462.2

augustus	g823.t1	UniRef100_K10739	3.80E-105	386.3
augustus	g824.t1	UniRef100_K01338	0.00E+00	1602
augustus	g826.t1	UniRef100_K08734	0.00E+00	1195.3
augustus	g831.t1	UniRef100_K11599	5.20E-74	282
augustus	g833.t1	UniRef100_K11825	1.80E-159	568.2
augustus	g837.t1	UniRef100_K14454	1.60E-227	793.5
augustus	g842.t1	UniRef100_K01893	4.80E-276	954.9
augustus	g844.t1	UniRef100_K03248	1.30E-153	547.4
augustus	g845.t1	UniRef100_K16369	0.00E+00	1734.5
augustus	g851.t1	UniRef100_K02892	4.50E-104	383.3
augustus	g856.t1	UniRef100_K14554	0.00E+00	1893.6
augustus	g857.t1	UniRef100_K15334	0.00E+00	1259.2
augustus	g858.t1	UniRef100_K03979	5.30E-255	885.2
augustus	g861.t1	UniRef100_K03013	1.20E-119	434.5
augustus	g866.t1	UniRef100_K21767	0.00E+00	1966.4
augustus	g869.t1	UniRef100_K19327	0.00E+00	1132.1
augustus	g872.t1	UniRef100_K13345	3.20E-222	775.8
augustus	g873.t1	UniRef100_K17362	8.30E-36	155.2
augustus	g879.t1	UniRef100_K03686	9.50E-230	801.2
augustus	g880.t1	UniRef100_K09528	1.30E-69	268.1
augustus	g881.t1	UniRef100_K14808	0.00E+00	1445.6
augustus	g884.t1	UniRef100_K14688	5.10E-117	426.4
augustus	g885.t1	UniRef100_K20404	0.00E+00	2729.9
augustus	g886.t1	UniRef100_K07300	8.50E-165	585.5
augustus	g887.t1	UniRef100_K06672	0.00E+00	2821.2
augustus	g888.t1	UniRef100_K03000	5.40E-60	235.3
augustus	g889.t1	UniRef100_K06072	5.00E-141	505.8
augustus	g890.t1	UniRef100_K03593	1.50E-121	441
augustus	g894.t1	UniRef100_K07827	3.00E-107	393.3
augustus	g897.t1	UniRef100_K15436	0.00E+00	1724.9
augustus	g900.t1	UniRef100_K14326	0.00E+00	2889
augustus	g901.t1	UniRef100_K01934	8.20E-105	385.2
augustus	g903.t1	UniRef100_K03028	0.00E+00	1609.3
augustus	g904.t1	UniRef100_K07179	4.00E-179	632.5
augustus	g906.t1	UniRef100_K00327	0.00E+00	1311.2
augustus	g912.t1	UniRef100_K19839	0.00E+00	1429.8
augustus	g913.t1	UniRef100_K16578	0.00E+00	1445.3
augustus	g919.t1	UniRef100_K14299	1.50E-209	733.8
augustus	g920.t1	UniRef100_K00507	2.40E-261	906
augustus	g922.t1	UniRef100_K08675	0.00E+00	1640.6
augustus	g925.t1	UniRef100_K17669	0.00E+00	1775
augustus	g928.t1	UniRef100_K17606	2.70E-142	510
augustus	g929.t1	UniRef100_K03164	0.00E+00	2357
augustus	g933.t1	UniRef100_K01522	1.10E-86	324.7
augustus	g935.t1	UniRef100_K12860	0.00E+00	1153.3
augustus	g943.t1	UniRef100_K12584	2.70E-164	583.2
augustus	g946.t1	UniRef100_K00121	8.80E-57	226.5
augustus	g955.t1	UniRef100_K15449	0.00E+00	1385.9
augustus	g956.t1	UniRef100_K17770	2.70E-67	260.4
augustus	g957.t1	UniRef100_K19986	0.00E+00	1224.2

augustus	g961.t1	UniRef100_K15711	0.00E+00	1398.6
augustus	g962.t1	UniRef100_K00456	1.30E-93	347.8
augustus	g964.t1	UniRef100_K18158	0.00E+00	1186
augustus	g965.t1	UniRef100_K07750	2.60E-126	456.8
augustus	g967.t1	UniRef100_K11569	1.60E-30	136.7
augustus	g968.t1	UniRef100_K11672	0.00E+00	1175.2
augustus	g970.t1	UniRef100_K01724	9.70E-58	227.6
augustus	g974.t1	UniRef100_K05759	1.50E-40	171
augustus	g976.t1	UniRef100_K08516	4.20E-94	349.4
augustus	g977.t1	UniRef100_K18664	0.00E+00	3669
augustus	g978.t1	UniRef100_K07889	1.10E-22	112.5
augustus	g981.t1	UniRef100_K03282	3.60E-78	296.6
augustus	g987.t1	UniRef100_K08257	4.80E-211	738.8
augustus	g988.t1	UniRef100_K00817	1.30E-196	690.6
augustus	g998.t1	UniRef100_K11541	0.00E+00	4144.7
augustus	g1001.t1	UniRef100_K04441	1.30E-203	713.8
augustus	g1002.t1	UniRef100_K00688	0.00E+00	1693.3
augustus	g1003.t1	UniRef100_K04630	1.80E-173	614
augustus	g1007.t1	UniRef100_K03347	0.00E+00	1340.9
augustus	g1010.t1	UniRef100_K15918	2.10E-118	430.6
augustus	g1011.t1	UniRef100_K14729	0.00E+00	1602
augustus	g1018.t1	UniRef100_K00586	1.30E-151	540.8
augustus	g1019.t1	UniRef100_K12848	3.40E-77	293.1
augustus	g1021.t1	UniRef100_K03842	2.80E-228	796.2
augustus	g1024.t1	UniRef100_K15109	4.40E-146	522.7
augustus	g1032.t1	UniRef100_K18798	9.90E-237	824.3
augustus	g1037.t1	UniRef100_K15423	1.60E-120	438
augustus	g1038.t1	UniRef100_K18174	1.30E-51	207.2
augustus	g1043.t1	UniRef100_K06628	0.00E+00	1285.8
augustus	g1045.t1	UniRef100_K12179	2.30E-204	716.5
augustus	g1048.t1	UniRef100_K15262	1.90E-149	533.5
augustus	g1049.t1	UniRef100_K01850	3.30E-123	446.4
augustus	g1057.t1	UniRef100_K10413	0.00E+00	8167
augustus	g1061.t1	UniRef100_K01937	8.60E-297	1024.2
augustus	g1067.t1	UniRef100_K00015	4.70E-87	326.2
augustus	g1070.t1	UniRef100_K20844	5.40E-240	835.1
augustus	g1071.t1	UniRef100_K05349	0.00E+00	1211.4
augustus	g1073.t1	UniRef100_K00297	0.00E+00	1184.5
augustus	g1075.t1	UniRef100_K03234	0.00E+00	1624.8
augustus	g1080.t1	UniRef100_K17637	0.00E+00	1713.4
augustus	g1081.t1	UniRef100_K00030	2.40E-187	660.2
augustus	g1086.t1	UniRef100_K01001	4.10E-237	825.5
augustus	g1089.t1	UniRef100_K02151	2.40E-49	199.9
augustus	g1092.t1	UniRef100_K01702	0.00E+00	1465.3
augustus	g1094.t1	UniRef100_K19932	5.30E-84	315.8
augustus	g1099.t1	UniRef100_K14967	5.30E-297	1024.6
augustus	g1112.t1	UniRef100_K12492	1.00E-182	644.4
augustus	g1113.t1	UniRef100_K03521	2.40E-128	463.4
augustus	g1114.t1	UniRef100_K01956	3.70E-256	888.6
augustus	g1118.t1	UniRef100_K01755	6.70E-206	721.8

augustus	g1120.t1	UniRef100_K01092	9.90E-105	385.2
augustus	g1123.t1	UniRef100_K14713	1.30E-156	558.1
augustus	g1124.t1	UniRef100_K01876	0.00E+00	1565.8
augustus	g1126.t1	UniRef100_K08051	2.20E-15	87.8
augustus	g1127.t1	UniRef100_K01115	0.00E+00	1550.8
augustus	g1129.t1	UniRef100_K00101	9.40E-266	920.6
augustus	g1130.t1	UniRef100_K11269	0.00E+00	1487.2
augustus	g1133.t1	UniRef100_K01166	1.10E-133	481.1
augustus	g1134.t1	UniRef100_K21451	0.00E+00	1514.2
augustus	g1136.t1	UniRef100_K01487	4.10E-226	788.9
augustus	g1137.t1	UniRef100_K00668	0.00E+00	4050.4
augustus	g1138.t1	UniRef100_K00667	0.00E+00	3373.9
augustus	g1141.t1	UniRef100_K08158	1.60E-276	956.8
augustus	g1143.t1	UniRef100_K10884	0.00E+00	1119.4
augustus	g1144.t1	UniRef100_K01772	4.10E-223	778.9
augustus	g1145.t1	UniRef100_K16944	2.10E-201	707.2
augustus	g1146.t1	UniRef100_K17969	9.20E-70	268.1
augustus	g1147.t1	UniRef100_K11557	1.00E-101	374.4
augustus	g1154.t1	UniRef100_K08333	0.00E+00	2809.2
augustus	g1155.t1	UniRef100_K11092	2.50E-36	157.5
augustus	g1157.t1	UniRef100_K05012	0.00E+00	1457.2
augustus	g1160.t1	UniRef100_K03661	2.70E-75	287
augustus	g1161.t1	UniRef100_K15376	0.00E+00	1129.4
augustus	g1162.t1	UniRef100_K03537	8.10E-78	295
augustus	g1163.t1	UniRef100_K06883	2.40E-171	606.7
augustus	g1164.t1	UniRef100_K14676	0.00E+00	2587.4
augustus	g1170.t1	UniRef100_K14338	0.00E+00	1891.3
augustus	g1174.t1	UniRef100_K16342	0.00E+00	1312.4
augustus	g1177.t1	UniRef100_K01092	1.70E-136	490.3
augustus	g1179.t1	UniRef100_K01083	1.20E-109	402.1
augustus	g1181.t1	UniRef100_K14861	0.00E+00	1892.5
augustus	g1182.t1	UniRef100_K00698	0.00E+00	3351.6
augustus	g1183.t1	UniRef100_K00698	0.00E+00	3159.8
augustus	g1186.t1	UniRef100_K09831	1.10E-307	1060.1
augustus	g1189.t1	UniRef100_K20298	5.20E-302	1041.6
augustus	g1193.t1	UniRef100_K03243	0.00E+00	1155.2
augustus	g1195.t1	UniRef100_K14327	0.00E+00	1850.5
augustus	g1196.t1	UniRef100_K07300	3.20E-173	613.2
augustus	g1197.t1	UniRef100_K01760	3.70E-232	808.9
augustus	g1198.t1	UniRef100_K17261	2.30E-221	773.5
augustus	g1199.t1	UniRef100_K05533	3.90E-220	769.2
augustus	g1202.t1	UniRef100_K00624	0.00E+00	1120.5
augustus	g1204.t1	UniRef100_K02541	0.00E+00	1417.1
augustus	g1208.t1	UniRef100_K01509	7.40E-74	283.1
augustus	g1209.t1	UniRef100_K05294	1.10E-155	556.2
augustus	g1210.t1	UniRef100_K21848	2.50E-157	559.7
augustus	g1214.t1	UniRef100_K06877	0.00E+00	2269.2
augustus	g1216.t1	UniRef100_K11649	0.00E+00	1120.1
augustus	g1217.t1	UniRef100_K18443	0.00E+00	2555.4
augustus	g1218.t1	UniRef100_K10246	2.30E-151	540

augustus	g1220.t1	UniRef100_K18914	2.50E-238	829.7
augustus	g1221.t1	UniRef100_K03522	2.50E-164	583.2
augustus	g1222.t1	UniRef100_K05285	0.00E+00	1669.1
augustus	g1223.t1	UniRef100_K00974	8.90E-81	306.2
augustus	g1224.t1	UniRef100_K00618	3.30E-273	945.7
augustus	g1226.t1	UniRef100_K07195	0.00E+00	1147.1
augustus	g1227.t1	UniRef100_K08741	1.70E-194	684.1
augustus	g1229.t1	UniRef100_K21864	0.00E+00	3326.2
augustus	g1230.t1	UniRef100_K00765	6.70E-145	518.5
augustus	g1231.t1	UniRef100_K06276	0.00E+00	1171.8
augustus	g1235.t1	UniRef100_K08501	2.60E-87	327
augustus	g1236.t1	UniRef100_K12782	2.00E-99	368.6
augustus	g1237.t1	UniRef100_K01183	1.20E-201	707.6
augustus	g1239.t1	UniRef100_K11650	1.10E-228	797.7
augustus	g1240.t1	UniRef100_K08272	1.20E-195	687.6
augustus	g1241.t1	UniRef100_K01870	0.00E+00	2039.2
augustus	g1242.t1	UniRef100_K01490	0.00E+00	1511.5
augustus	g1243.t1	UniRef100_K03038	6.10E-151	538.9
augustus	g1245.t1	UniRef100_K00228	2.10E-187	660.2
augustus	g1246.t1	UniRef100_K08341	2.80E-48	196.8
augustus	g1247.t1	UniRef100_K08141	8.90E-308	1060.4
augustus	g1250.t1	UniRef100_K02902	4.70E-108	396
augustus	g1251.t1	UniRef100_K01694	0.00E+00	1332.8
augustus	g1260.t1	UniRef100_K02510	3.50E-118	429.5
augustus	g1263.t1	UniRef100_K14454	2.90E-124	450.3
augustus	g1264.t1	UniRef100_K08157	9.90E-41	172.9
augustus	g1276.t1	UniRef100_K01520	1.70E-52	210.7
augustus	g1279.t1	UniRef100_K16261	1.60E-294	1016.5
augustus	g1281.t1	UniRef100_K17417	4.80E-66	255.4
augustus	g1286.t1	UniRef100_K11788	0.00E+00	1489.9
augustus	g1291.t1	UniRef100_K03305	3.40E-305	1052
augustus	g1294.t1	UniRef100_K22063	4.40E-73	279.6
augustus	g1298.t1	UniRef100_K12195	4.60E-90	335.9
augustus	g1299.t1	UniRef100_K14571	0.00E+00	1297.3
augustus	g1300.t1	UniRef100_K08738	8.70E-52	207.6
augustus	g1302.t1	UniRef100_K12589	1.90E-160	570.5
augustus	g1304.t1	UniRef100_K07766	7.10E-85	318.2
augustus	g1305.t1	UniRef100_K12620	3.10E-83	312.8
augustus	g1307.t1	UniRef100_K07874	1.20E-106	391
augustus	g1311.t1	UniRef100_K11274	0.00E+00	1456.4
augustus	g1315.t1	UniRef100_K14315	3.50E-298	1028.9
augustus	g1317.t1	UniRef100_K17797	3.30E-167	592.8
augustus	g1318.t1	UniRef100_K03844	6.60E-250	868.2
augustus	g1319.t1	UniRef100_K00681	7.30E-290	1001.1
augustus	g1320.t1	UniRef100_K15331	4.70E-237	825.5
augustus	g1321.t1	UniRef100_K21991	0.00E+00	1190.6
augustus	g1322.t1	UniRef100_K03143	9.60E-175	617.8
augustus	g1323.t1	UniRef100_K08657	1.10E-242	844.3
augustus	g1324.t1	UniRef100_K18584	2.30E-223	780
augustus	g1325.t1	UniRef100_K13953	6.00E-190	668.3

augustus	g1326.t1	UniRef100_K07213	1.40E-36	156.8
augustus	g1327.t1	UniRef100_K08764	1.10E-231	807.4
augustus	g1333.t1	UniRef100_K06954	5.60E-73	280.4
augustus	g1334.t1	UniRef100_K02258	2.90E-117	426.8
augustus	g1335.t1	UniRef100_K02898	5.80E-64	248.4
augustus	g1339.t1	UniRef100_K10582	0.00E+00	1253.4
augustus	g1340.t1	UniRef100_K00948	1.10E-228	797.3
augustus	g1343.t1	UniRef100_K04513	9.10E-44	181.8
augustus	g1344.t1	UniRef100_K01855	4.00E-228	795.8
augustus	g1345.t1	UniRef100_K03858	7.40E-67	258.8
augustus	g1351.t1	UniRef100_K12193	1.20E-75	288.1
augustus	g1353.t1	UniRef100_K12881	5.50E-80	302.8
augustus	g1356.t1	UniRef100_K01733	1.40E-229	800.8
augustus	g1358.t1	UniRef100_K20360	6.70E-290	1001.1
augustus	g1359.t1	UniRef100_K14411	1.10E-145	521.9
augustus	g1361.t1	UniRef100_K00968	4.40E-178	629.4
augustus	g1362.t1	UniRef100_K01613	0.00E+00	1696.8
augustus	g1364.t1	UniRef100_K02932	2.50E-150	536.6
augustus	g1365.t1	UniRef100_K01253	1.20E-72	278.5
augustus	g1373.t1	UniRef100_K01046	3.00E-144	516.5
augustus	g1379.t1	UniRef100_K00102	1.70E-207	727.2
augustus	g1380.t1	UniRef100_K00148	7.60E-151	538.5
augustus	g1382.t1	UniRef100_K00326	5.00E-113	413.3
augustus	g1390.t1	UniRef100_K03785	0.00E+00	1191
augustus	g1391.t1	UniRef100_K06996	4.90E-10	69.3
augustus	g1392.t1	UniRef100_K11372	2.40E-153	547.4
augustus	g1393.t1	UniRef100_K14559	9.20E-225	785
augustus	g1399.t1	UniRef100_K00793	2.40E-116	423.3
augustus	g1400.t1	UniRef100_K08991	7.40E-278	961.1
augustus	g1401.t1	UniRef100_K01836	7.80E-306	1053.9
augustus	g1402.t1	UniRef100_K11434	5.70E-166	589
augustus	g1403.t1	UniRef100_K14842	2.20E-141	506.5
augustus	g1404.t1	UniRef100_K02888	2.70E-44	183.7
augustus	g1405.t1	UniRef100_K08866	8.00E-284	981.5
augustus	g1408.t1	UniRef100_K00294	1.90E-302	1042.7
augustus	g1423.t1	UniRef100_K06018	9.10E-283	977.6
augustus	g1425.t1	UniRef100_K04102	3.90E-98	363.2
augustus	g1427.t1	UniRef100_K00480	3.10E-210	736.1
augustus	g1432.t1	UniRef100_K17741	9.40E-55	219.2
augustus	g1442.t1	UniRef100_K00605	1.30E-226	790.4
augustus	g1443.t1	UniRef100_K02144	3.10E-232	809.3
augustus	g1445.t1	UniRef100_K02940	4.90E-101	372.1
augustus	g1446.t1	UniRef100_K18466	1.90E-160	570.5
augustus	g1447.t1	UniRef100_K14303	0.00E+00	2127.4
augustus	g1448.t1	UniRef100_K04482	1.30E-180	637.5
augustus	g1452.t1	UniRef100_K02732	5.00E-149	531.9
augustus	g1453.t1	UniRef100_K00888	0.00E+00	3418.2
augustus	g1457.t1	UniRef100_K00794	8.80E-97	358.2
augustus	g1461.t1	UniRef100_K14570	0.00E+00	1149.8
augustus	g1463.t1	UniRef100_K11426	1.90E-238	830.1

augustus	g1464.t1	UniRef100_K14001	1.80E-174	617.1
augustus	g1469.t1	UniRef100_K13342	6.20E-268	928.3
augustus	g1470.t1	UniRef100_K14847	3.80E-159	565.8
augustus	g1472.t1	UniRef100_K01758	1.70E-228	796.6
augustus	g1478.t1	UniRef100_K11761	2.40E-143	514.2
augustus	g1481.t1	UniRef100_K12585	0.00E+00	1859
augustus	g1482.t1	UniRef100_K14538	2.80E-218	763.1
augustus	g1483.t1	UniRef100_K01845	2.40E-197	693.3
augustus	g1484.t1	UniRef100_K12826	4.60E-133	478.8
augustus	g1485.t1	UniRef100_K08286	0.00E+00	1095.9
augustus	g1486.t1	UniRef100_K17792	4.80E-192	675.6
augustus	g1490.t1	UniRef100_K00953	1.50E-104	384.4
augustus	g1493.t1	UniRef100_K10625	0.00E+00	3584.7
augustus	g1494.t1	UniRef100_K14294	1.10E-47	194.9
augustus	g1495.t1	UniRef100_K03064	5.00E-215	751.9
augustus	g1496.t1	UniRef100_K00820	0.00E+00	1298.9
augustus	g1500.t1	UniRef100_K07748	3.40E-199	699.1
augustus	g1506.t1	UniRef100_K10585	2.30E-163	580.1
augustus	g1507.t1	UniRef100_K10755	3.20E-172	609.8
augustus	g1509.t1	UniRef100_K12854	0.00E+00	3985.3
augustus	g1511.t1	UniRef100_K02605	2.60E-292	1009.6
augustus	g1514.t1	UniRef100_K12273	3.60E-102	376.3
augustus	g1515.t1	UniRef100_K14015	0.00E+00	1142.5
augustus	g1518.t1	UniRef100_K01955	0.00E+00	1990.7
augustus	g1524.t1	UniRef100_K03015	8.10E-89	331.6
augustus	g1525.t1	UniRef100_K02510	9.00E-133	478
augustus	g1527.t1	UniRef100_K00588	1.00E-25	122.1
augustus	g1530.t1	UniRef100_K01279	2.00E-151	541.2
augustus	g1536.t1	UniRef100_K01179	8.60E-167	591.7
augustus	g1539.t1	UniRef100_K06902	0.00E+00	1164.1
augustus	g1541.t1	UniRef100_K10365	2.30E-155	553.1
augustus	g1545.t1	UniRef100_K15102	3.90E-196	689.1
augustus	g1547.t1	UniRef100_K11872	2.40E-291	1006.1
augustus	g1548.t1	UniRef100_K03847	2.00E-224	783.5
augustus	g1549.t1	UniRef100_K01768	0.00E+00	3296.1
augustus	g1550.t1	UniRef100_K01182	3.00E-234	816.2
augustus	g1551.t1	UniRef100_K21232	1.50E-39	166.8
augustus	g1552.t1	UniRef100_K18599	5.20E-201	705.3
augustus	g1559.t1	UniRef100_K05545	3.50E-181	639.4
augustus	g1560.t1	UniRef100_K19984	0.00E+00	1523.5
augustus	g1561.t1	UniRef100_K19177	8.50E-163	578.2
augustus	g1563.t1	UniRef100_K15429	2.10E-244	849.7
augustus	g1568.t1	UniRef100_K01868	0.00E+00	1436.8
augustus	g1570.t1	UniRef100_K09580	7.50E-247	857.8
augustus	g1571.t1	UniRef100_K00219	4.30E-78	297
augustus	g1572.t1	UniRef100_K00428	3.90E-163	579.3
augustus	g1573.t1	UniRef100_K13217	4.30E-278	961.8
augustus	g1574.t1	UniRef100_K20279	0.00E+00	2213.7
augustus	g1576.t1	UniRef100_K06630	6.20E-129	465.3
augustus	g1578.t1	UniRef100_K02155	2.40E-77	293.1

augustus	g1579.t1	UniRef100_K00223	1.90E-284	983
augustus	g1580.t1	UniRef100_K01115	0.00E+00	2535
augustus	g1581.t1	UniRef100_K06134	6.50E-106	388.7
augustus	g1582.t1	UniRef100_K15178	1.90E-219	767.3
augustus	g1583.t1	UniRef100_K18694	3.80E-109	400.2
augustus	g1588.t1	UniRef100_K15069	1.90E-172	611.3
augustus	g1590.t1	UniRef100_K17768	6.90E-245	851.7
augustus	g1591.t1	UniRef100_K08486	3.70E-95	353.6
augustus	g1592.t1	UniRef100_K14561	1.50E-162	577
augustus	g1593.t1	UniRef100_K14326	0.00E+00	1812.3
augustus	g1594.t1	UniRef100_K01230	4.80E-263	912.5
augustus	g1595.t1	UniRef100_K15040	1.10E-143	514.6
augustus	g1596.t1	UniRef100_K01953	0.00E+00	1122.1
augustus	g1597.t1	UniRef100_K10577	1.20E-89	334
augustus	g1598.t1	UniRef100_K13337	9.60E-81	305.8
augustus	g1600.t1	UniRef100_K08869	1.7e-311	1072.8
augustus	g1603.t1	UniRef100_K11272	0.00E+00	1214.5
augustus	g1604.t1	UniRef100_K01858	8.10E-298	1027.3
augustus	g1623.t1	UniRef100_K10873	5.10E-230	802.7
augustus	g1644.t1	UniRef100_K12177	9.90E-186	654.8
augustus	g1647.t1	UniRef100_K01483	1.30E-82	311.2
augustus	g1649.t1	UniRef100_K02929	1.40E-45	186.8
augustus	g1650.t1	UniRef100_K17268	1.90E-132	477.2
augustus	g1652.t1	UniRef100_K03627	1.00E-68	264.6
augustus	g1672.t1	UniRef100_K20989	0.00E+00	1112.8
augustus	g1679.t1	UniRef100_K01253	2.90E-41	174.5
augustus	g1686.t1	UniRef100_K01210	5.80E-132	476.9
augustus	g1688.t1	UniRef100_K18386	1.90E-42	178.7
augustus	g1690.t1	UniRef100_K08095	7.00E-68	262.3
augustus	g1692.t1	UniRef100_K11309	1.30E-242	844
augustus	g1693.t1	UniRef100_K17437	1.40E-89	334.7
augustus	g1695.t1	UniRef100_K01061	4.10E-102	377.1
augustus	g1699.t1	UniRef100_K16833	7.00E-88	328.9
augustus	g1702.t1	UniRef100_K13281	2.50E-178	630.6
augustus	g1703.t1	UniRef100_K05302	3.20E-144	516.9
augustus	g1705.t1	UniRef100_K12393	9.20E-237	824.3
augustus	g1708.t1	UniRef100_K22211	7.20E-19	99.4
augustus	g1709.t1	UniRef100_K03131	2.90E-240	835.9
augustus	g1710.t1	UniRef100_K00236	5.30E-67	259.2
augustus	g1714.t1	UniRef100_K01279	4.10E-142	510.4
augustus	g1715.t1	UniRef100_K01279	6.70E-136	489.6
augustus	g1718.t1	UniRef100_K13524	4.90E-218	762.3
augustus	g1720.t1	UniRef100_K19245	4.00E-122	443.4
augustus	g1725.t1	UniRef100_K01530	0.00E+00	2237.2
augustus	g1730.t1	UniRef100_K05665	5.00E-189	667.5
augustus	g1732.t1	UniRef100_K10956	3.20E-261	905.6
augustus	g1733.t1	UniRef100_K00791	5.00E-216	755.4
augustus	g1734.t1	UniRef100_K17263	0.00E+00	2123.6
augustus	g1735.t1	UniRef100_K00135	1.60E-137	494.6
augustus	g1737.t1	UniRef100_K20280	1.00E-106	391.3

augustus	g1738.t1	UniRef100_K08900	2.00E-252	876.7
augustus	g1743.t1	UniRef100_K11584	2.00E-306	1056.2
augustus	g1746.t1	UniRef100_K20028	2.20E-234	816.6
augustus	g1747.t1	UniRef100_K05857	0.00E+00	1670.2
augustus	g1750.t1	UniRef100_K11578	0.00E+00	1182.2
augustus	g1752.t1	UniRef100_K06013	5.10E-83	313.5
augustus	g1754.t1	UniRef100_K06972	0.00E+00	1913.3
augustus	g1755.t1	UniRef100_K01581	1.40E-247	860.1
augustus	g1756.t1	UniRef100_K15083	0.00E+00	1250.3
augustus	g1757.t1	UniRef100_K15082	1.00E-224	784.6
augustus	g1759.t1	UniRef100_K01188	4.30E-282	975.7
augustus	g1761.t1	UniRef100_K01874	0.00E+00	1191.4
augustus	g1762.t1	UniRef100_K14285	1.30E-78	297.4
augustus	g1763.t1	UniRef100_K05955	5.40E-227	792
augustus	g1766.t1	UniRef100_K02923	3.80E-33	145.2
augustus	g1768.t1	UniRef100_K06111	0.00E+00	1856.3
augustus	g1772.t1	UniRef100_K12350	0.00E+00	1065.1
augustus	g1776.t1	UniRef100_K10777	0.00E+00	1193.7
augustus	g1781.t1	UniRef100_K18748	0.00E+00	1947.2
augustus	g1783.t1	UniRef100_K18723	3.50E-178	629.8
augustus	g1784.t1	UniRef100_K08499	6.70E-55	219.2
augustus	g1790.t1	UniRef100_K05019	2.50E-125	453.4
augustus	g1794.t1	UniRef100_K04555	5.50E-93	345.1
augustus	g1796.t1	UniRef100_K01883	0.00E+00	1404.8
augustus	g1797.t1	UniRef100_K07442	1.10E-232	810.8
augustus	g1798.t1	UniRef100_K11088	2.50E-48	196.8
augustus	g1805.t1	UniRef100_K05546	0.00E+00	1739.9
augustus	g1811.t1	UniRef100_K22048	0.00E+00	1378.2
augustus	g1817.t1	UniRef100_K20168	0.00E+00	1384
augustus	g1818.t1	UniRef100_K01715	2.70E-23	114.4
augustus	g1822.t1	UniRef100_K13950	0.00E+00	1485.3
augustus	g1823.t1	UniRef100_K20306	0.00E+00	2503.8
augustus	g1825.t1	UniRef100_K08139	2.80E-50	204.9
augustus	g1828.t1	UniRef100_K00831	1.20E-184	651
augustus	g1829.t1	UniRef100_K02725	2.90E-122	443.4
augustus	g1830.t1	UniRef100_K06966	1.10E-118	431
augustus	g1834.t1	UniRef100_K08517	6.70E-107	391.7
augustus	g1835.t1	UniRef100_K11376	4.50E-159	565.8
augustus	g1836.t1	UniRef100_K03650	1.00E-261	907.5
augustus	g1842.t1	UniRef100_K01228	0.00E+00	1562
augustus	g1844.t1	UniRef100_K11238	0.00E+00	2472.6
augustus	g1847.t1	UniRef100_K10903	1.60E-154	550.8
augustus	g1848.t1	UniRef100_K20353	0.00E+00	2043.1
augustus	g1849.t1	UniRef100_K01530	0.00E+00	2161.7
augustus	g1853.t1	UniRef100_K14429	0.00E+00	1802.7
augustus	g1857.t1	UniRef100_K01526	5.20E-286	988
augustus	g1859.t1	UniRef100_K07897	1.10E-94	351.7
augustus	g1860.t1	UniRef100_K00249	1.60E-241	840.1
augustus	g1862.t1	UniRef100_K06944	1.10E-182	644.4
augustus	g1865.t1	UniRef100_K16196	0.00E+00	2697.2

augustus	g1867.t1	UniRef100_K09510	1.50E-154	550.8
augustus	g1869.t1	UniRef100_K15463	6.30E-175	619
augustus	g1870.t1	UniRef100_K18106	5.90E-219	765
augustus	g1871.t1	UniRef100_K18103	4.90E-151	538.9
augustus	g1872.t1	UniRef100_K20984	0.00E+00	1115.5
augustus	g1880.t1	UniRef100_K14839	5.20E-82	309.3
augustus	g1881.t1	UniRef100_K00432	9.70E-85	317.8
augustus	g1885.t1	UniRef100_K00128	3.10E-106	391
augustus	g1888.t1	UniRef100_K00218	1.00E-50	205.7
augustus	g1893.t1	UniRef100_K18802	1.80E-207	726.9
augustus	g1896.t1	UniRef100_K15289	4.40E-183	646
augustus	g1900.t1	UniRef100_K03781	0.00E+00	1075.8
augustus	g1903.t1	UniRef100_K12957	8.60E-53	212.6
augustus	g1904.t1	UniRef100_K01760	1.30E-113	414.8
augustus	g1908.t1	UniRef100_K10750	5.30E-149	533.1
augustus	g1913.t1	UniRef100_K00122	1.80E-199	700.3
augustus	g1914.t1	UniRef100_K13780	3.50E-63	248.1
augustus	g1915.t1	UniRef100_K13120	2.10E-19	100.5
augustus	g1918.t1	UniRef100_K02604	5.20E-152	542.7
augustus	g1919.t1	UniRef100_K01094	3.80E-106	389.4
augustus	g1922.t1	UniRef100_K17790	1.00E-68	265
augustus	g1924.t1	UniRef100_K06063	1.90E-252	876.7
augustus	g1926.t1	UniRef100_K01113	0.00E+00	1123.6
augustus	g1930.t1	UniRef100_K22071	2.20E-85	320.1
augustus	g1933.t1	UniRef100_K11870	1.20E-296	1023.8
augustus	g1934.t1	UniRef100_K00474	1.30E-154	551.2
augustus	g1935.t1	UniRef100_K17742	3.10E-100	369.8
augustus	g1936.t1	UniRef100_K04348	0.00E+00	1080.1
augustus	g1938.t1	UniRef100_K08257	1.50E-244	850.1
augustus	g1939.t1	UniRef100_K00863	2.90E-293	1012.3
augustus	g1940.t1	UniRef100_K11096	2.90E-53	213
augustus	g1941.t1	UniRef100_K05605	3.70E-252	875.5
augustus	g1943.t1	UniRef100_K02321	1.90E-300	1036.2
augustus	g1944.t1	UniRef100_K13110	1.40E-46	192.2
augustus	g1945.t1	UniRef100_K00789	3.20E-36	157.9
augustus	g1947.t1	UniRef100_K00787	1.20E-178	630.9
augustus	g1948.t1	UniRef100_K03355	3.10E-265	919.5
augustus	g1951.t1	UniRef100_K17600	0.00E+00	1651
augustus	g1952.t1	UniRef100_K02209	0.00E+00	1351.7
augustus	g1957.t1	UniRef100_K12871	1.60E-78	297.4
augustus	g1962.t1	UniRef100_K11324	2.40E-218	763.5
augustus	g1963.t1	UniRef100_K20346	2.10E-112	410.2
augustus	g1964.t1	UniRef100_K02218	5.70E-245	851.7
augustus	g1966.t1	UniRef100_K04882	2.80E-74	284.3
augustus	g1967.t1	UniRef100_K00459	6.40E-148	528.9
augustus	g1970.t1	UniRef100_K08968	2.20E-69	267.3
augustus	g1972.t1	UniRef100_K14549	1.40E-275	953.4
augustus	g1974.t1	UniRef100_K20103	0.00E+00	2247.2
augustus	g1977.t1	UniRef100_K10570	9.60E-239	830.9
augustus	g1983.t1	UniRef100_K01469	0.00E+00	2511.9

augustus	g1985.t1	UniRef100_K19572	0.00E+00	1081.6
augustus	g1987.t1	UniRef100_K09456	1.40E-62	246.1
augustus	g1989.t1	UniRef100_K07059	1.20E-31	140.2
augustus	g1990.t1	UniRef100_K00121	3.30E-200	702.6
augustus	g1992.t1	UniRef100_K17450	0.00E+00	1513
augustus	g1994.t1	UniRef100_K08876	1.90E-264	917.1
augustus	g1995.t1	UniRef100_K01593	5.10E-36	157.5
augustus	g1998.t1	UniRef100_K09705	2.30E-83	313.5
augustus	g2000.t1	UniRef100_K10392	0.00E+00	3007.6
augustus	g2002.t1	UniRef100_K01254	0.00E+00	1140.6
augustus	g2015.t1	UniRef100_K01885	0.00E+00	1235.3
augustus	g2016.t1	UniRef100_K02863	7.70E-154	548.1
augustus	g2017.t1	UniRef100_K20289	5.80E-103	379
augustus	g2024.t1	UniRef100_K10798	4.90E-290	1001.9
augustus	g2028.t1	UniRef100_K00046	1.60E-26	125.2
augustus	g2029.t1	UniRef100_K01180	0.00E+00	1472.2
augustus	g2034.t1	UniRef100_K11446	0.00E+00	2742.6
augustus	g2035.t1	UniRef100_K15078	1.10E-142	511.1
augustus	g2036.t1	UniRef100_K12827	6.50E-265	917.9
augustus	g2037.t1	UniRef100_K13025	1.80E-227	793.1
augustus	g2040.t1	UniRef100_K03217	5.70E-171	605.9
augustus	g2042.t1	UniRef100_K14012	1.20E-166	591.3
augustus	g2043.t1	UniRef100_K16185	6.40E-182	641.7
augustus	g2044.t1	UniRef100_K20363	1.50E-130	470.7
augustus	g2046.t1	UniRef100_K08178	4.50E-190	669.5
augustus	g2049.t1	UniRef100_K15419	0.00E+00	1219.1
augustus	g2050.t1	UniRef100_K00720	2.10E-258	896.3
augustus	g2051.t1	UniRef100_K15322	1.60E-222	777.3
augustus	g2053.t1	UniRef100_K12795	1.30E-153	548.1
augustus	g2054.t1	UniRef100_K01852	3.40E-177	627.1
augustus	g2055.t1	UniRef100_K10878	5.30E-69	266.5
augustus	g2058.t1	UniRef100_K01062	6.20E-272	941.4
augustus	g2059.t1	UniRef100_K06988	9.20E-46	188.7
augustus	g2063.t1	UniRef100_K03417	2.40E-24	117.9
augustus	g2064.t1	UniRef100_K13028	3.10E-85	320.5
augustus	g2066.t1	UniRef100_K01809	6.00E-75	286.2
augustus	g2067.t1	UniRef100_K08139	9.20E-214	748
augustus	g2068.t1	UniRef100_K00467	3.50E-83	313.9
augustus	g2070.t1	UniRef100_K01426	2.30E-220	770
augustus	g2071.t1	UniRef100_K06221	3.40E-40	170.6
augustus	g2073.t1	UniRef100_K03353	4.90E-293	1011.5
augustus	g2074.t1	UniRef100_K01693	1.20E-109	401
augustus	g2075.t1	UniRef100_K00620	2.90E-242	842.4
augustus	g2079.t1	UniRef100_K15128	2.50E-124	450.3
augustus	g2080.t1	UniRef100_K14780	0.00E+00	1888.2
augustus	g2081.t1	UniRef100_K11877	3.80E-69	265.8
augustus	g2085.t1	UniRef100_K01907	2.10E-298	1029.6
augustus	g2086.t1	UniRef100_K08874	0.00E+00	7160.5
augustus	g2097.t1	UniRef100_K02371	1.90E-42	178.3
augustus	g2098.t1	UniRef100_K13179	7.00E-268	927.9

augustus	g2102.t1	UniRef100_K09828	1.40E-49	202.6
augustus	g2105.t1	UniRef100_K22322	3.50E-43	180.6
augustus	g2109.t1	UniRef100_K01433	1.40E-147	527.3
augustus	g2118.t1	UniRef100_K17741	6.70E-26	123.2
augustus	g2120.t1	UniRef100_K00943	1.30E-113	414.5
augustus	g2121.t1	UniRef100_K02437	1.00E-74	284.6
augustus	g2122.t1	UniRef100_K01749	4.80E-168	595.5
augustus	g2123.t1	UniRef100_K00323	0.00E+00	1871.7
augustus	g2124.t1	UniRef100_K18160	1.40E-29	134.8
augustus	g2125.t1	UniRef100_K00995	3.80E-263	912.1
augustus	g2132.t1	UniRef100_K02926	3.90E-137	492.7
augustus	g2133.t1	UniRef100_K17439	2.60E-212	743
augustus	g2142.t1	UniRef100_K09647	5.30E-88	328.6
augustus	g2143.t1	UniRef100_K02520	5.90E-95	352.4
augustus	g2146.t1	UniRef100_K03885	2.70E-125	453.8
augustus	g2150.t1	UniRef100_K03781	5.40E-93	346.3
augustus	g2152.t1	UniRef100_K07282	7.90E-53	213.4
augustus	g2155.t1	UniRef100_K11150	2.00E-28	131.3
augustus	g2162.t1	UniRef100_K00326	2.70E-190	669.8
augustus	g2165.t1	UniRef100_K22211	2.30E-20	104.4
augustus	g2166.t1	UniRef100_K00787	1.30E-62	245.4
augustus	g2168.t1	UniRef100_K09456	3.10E-45	188.3
augustus	g2173.t1	UniRef100_K00059	2.70E-29	134.4
augustus	g2174.t1	UniRef100_K08139	1.40E-175	621.3
augustus	g2175.t1	UniRef100_K01438	2.90E-181	640.6
augustus	g2177.t1	UniRef100_K08192	3.20E-73	281.2
augustus	g2178.t1	UniRef100_K01187	5.40E-104	384
augustus	g2180.t1	UniRef100_K15855	0.00E+00	1617.8
augustus	g2183.t1	UniRef100_K10253	2.40E-86	323.6
augustus	g2184.t1	UniRef100_K08299	2.10E-44	184.5
augustus	g2185.t1	UniRef100_K15385	1.80E-48	198.4
augustus	g2187.t1	UniRef100_K18289	4.30E-145	519.6
augustus	g2189.t1	UniRef100_K11883	2.70E-166	590.1
augustus	g2190.t1	UniRef100_K01611	7.50E-250	867.8
augustus	g2195.t1	UniRef100_K22118	0.00E+00	2922.9
augustus	g2197.t1	UniRef100_K22248	0.00E+00	1192.2
augustus	g2199.t1	UniRef100_K01078	2.80E-186	656.8
augustus	g2204.t1	UniRef100_K00387	1.30E-298	1030
augustus	g2206.t1	UniRef100_K06889	3.00E-32	144.1
augustus	g2209.t1	UniRef100_K18549	4.20E-119	433.3
augustus	g2212.t1	UniRef100_K00326	5.70E-135	486.1
augustus	g2213.t1	UniRef100_K01613	1.80E-232	810.1
augustus	g2215.t1	UniRef100_K17644	1.50E-33	148.3
augustus	g2217.t1	UniRef100_K01183	7.70E-33	146.4
augustus	g2227.t1	UniRef100_K00799	3.30E-60	236.9
augustus	g2228.t1	UniRef100_K01673	2.20E-61	240.4
augustus	g2230.t1	UniRef100_K01188	2.50E-73	281.6
augustus	g2231.t1	UniRef100_K00480	1.80E-167	594
augustus	g2232.t1	UniRef100_K00002	1.70E-174	617.1
augustus	g2233.t1	UniRef100_K01607	5.20E-26	122.9

augustus	g2234.t1	UniRef100_K00135	1.00E-130	471.9
augustus	g2236.t1	UniRef100_K07406	1.80E-127	461.1
augustus	g2243.t1	UniRef100_K00074	1.30E-31	141.7
augustus	g2246.t1	UniRef100_K09704	2.10E-79	302
augustus	g2248.t1	UniRef100_K08165	9.20E-97	359.4
augustus	g2251.t1	UniRef100_K03418	4.60E-94	350.9
augustus	g2252.t1	UniRef100_K00078	6.80E-44	183.3
augustus	g2257.t1	UniRef100_K07824	8.50E-190	668.3
augustus	g2262.t1	UniRef100_K01192	2.30E-285	986.5
augustus	g2273.t1	UniRef100_K14805	0.00E+00	1084.3
augustus	g2274.t1	UniRef100_K09291	0.00E+00	1508.8
augustus	g2277.t1	UniRef100_K03854	1.70E-155	554.3
augustus	g2278.t1	UniRef100_K15272	1.10E-163	581.6
augustus	g2279.t1	UniRef100_K00966	1.20E-177	627.9
augustus	g2291.t1	UniRef100_K15148	2.70E-97	360.1
augustus	g2300.t1	UniRef100_K10531	5.20E-273	944.9
augustus	g2302.t1	UniRef100_K22148	0.00E+00	7227.9
augustus	g2303.t1	UniRef100_K00108	2.40E-169	600.5
augustus	g2304.t1	UniRef100_K00130	1.50E-279	966.5
augustus	g2311.t1	UniRef100_K01183	3.00E-160	570.1
augustus	g2313.t1	UniRef100_K04125	2.50E-66	257.7
augustus	g2324.t1	UniRef100_K08141	2.90E-255	885.9
augustus	g2325.t1	UniRef100_K01811	0.00E+00	1379.4
augustus	g2328.t1	UniRef100_K21292	1.10E-78	299.3
augustus	g2329.t1	UniRef100_K15279	4.10E-155	552.7
augustus	g2336.t1	UniRef100_K07407	3.00E-102	377.5
augustus	g2340.t1	UniRef100_K17741	2.30E-29	134.8
augustus	g2341.t1	UniRef100_K01560	3.90E-45	186.8
augustus	g2344.t1	UniRef100_K08139	1.10E-56	226.1
augustus	g2346.t1	UniRef100_K01213	1.60E-43	182.2
augustus	g2347.t1	UniRef100_K01130	5.40E-182	642.5
augustus	g2348.t1	UniRef100_K00799	2.10E-55	220.7
augustus	g2350.t1	UniRef100_K01738	8.20E-90	335.5
augustus	g2351.t1	UniRef100_K01915	5.50E-167	592
augustus	g2352.t1	UniRef100_K08157	4.70E-54	217.6
augustus	g2354.t1	UniRef100_K02510	3.50E-27	127.1
augustus	g2356.t1	UniRef100_K14454	1.10E-179	634.4
augustus	g2358.t1	UniRef100_K02510	1.00E-25	122.5
augustus	g2359.t1	UniRef100_K15385	1.40E-43	182.2
augustus	g2360.t1	UniRef100_K15394	0.00E+00	2636.7
augustus	g2366.t1	UniRef100_K19823	1.80E-126	458
augustus	g2380.t1	UniRef100_K01181	5.20E-142	509.2
augustus	g2382.t1	UniRef100_K19564	5.70E-242	841.6
augustus	g2383.t1	UniRef100_K01480	9.20E-204	714.5
augustus	g2387.t1	UniRef100_K08712	1.30E-270	938.3
augustus	g2391.t1	UniRef100_K08141	1.10E-179	634.8
augustus	g2392.t1	UniRef100_K15924	5.20E-70	270.4
augustus	g2393.t1	UniRef100_K01235	9.80E-308	1060.8
augustus	g2404.t1	UniRef100_K01684	1.10E-173	614.4
augustus	g2415.t1	UniRef100_K06978	8.10E-94	349.7

augustus	g2420.t1	UniRef100_K08197	2.30E-58	231.9
augustus	g2421.t1	UniRef100_K22150	1.60E-134	483.8
augustus	g2422.t1	UniRef100_K22151	1.90E-220	770
augustus	g2423.t1	UniRef100_K05658	1.30E-216	758.8
augustus	g2425.t1	UniRef100_K22152	0.00E+00	2201.8
augustus	g2438.t1	UniRef100_K12957	1.20E-59	235.3
augustus	g2440.t1	UniRef100_K00354	6.70E-126	455.7
augustus	g2443.t1	UniRef100_K03232	1.40E-75	288.1
augustus	g2445.t1	UniRef100_K00108	5.40E-64	250.8
augustus	g2446.t1	UniRef100_K05665	3.40E-181	641.3
augustus	g2449.t1	UniRef100_K01253	2.10E-56	224.9
augustus	g2465.t1	UniRef100_K17649	1.10E-50	206.1
augustus	g2467.t1	UniRef100_K19200	1.40E-152	544.7
augustus	g2468.t1	UniRef100_K08192	1.90E-72	278.5
augustus	g2472.t1	UniRef100_K01286	2.70E-64	251.1
augustus	g2473.t1	UniRef100_K02510	5.20E-110	402.5
augustus	g2474.t1	UniRef100_K00140	1.00E-257	894
augustus	g2477.t1	UniRef100_K00001	1.80E-41	175.3
augustus	g2480.t1	UniRef100_K03790	1.20E-11	75.1
augustus	g2482.t1	UniRef100_K08158	8.80E-127	458.8
augustus	g2487.t1	UniRef100_K14333	1.30E-174	617.5
augustus	g2491.t1	UniRef100_K01213	3.20E-188	662.9
augustus	g2492.t1	UniRef100_K01426	6.60E-179	632.5
augustus	g2496.t1	UniRef100_K01563	7.60E-64	249.2
augustus	g2502.t1	UniRef100_K02540	0.00E+00	1424.5
augustus	g2503.t1	UniRef100_K11979	5.10E-229	798.9
augustus	g2505.t1	UniRef100_K01187	0.00E+00	1498.4
augustus	g2509.t1	UniRef100_K05349	0.00E+00	1501.1
augustus	g2514.t1	UniRef100_K15454	2.40E-265	919.5
augustus	g2515.t1	UniRef100_K07107	2.20E-75	286.6
augustus	g2518.t1	UniRef100_K17842	4.70E-288	995
augustus	g2521.t1	UniRef100_K22068	8.00E-93	344.7
augustus	g2522.t1	UniRef100_K11836	0.00E+00	1428.7
augustus	g2523.t1	UniRef100_K02537	3.60E-89	333.2
augustus	g2527.t1	UniRef100_K01563	1.60E-69	268.1
augustus	g2528.t1	UniRef100_K17645	2.60E-101	374.4
augustus	g2532.t1	UniRef100_K08832	7.50E-162	575.5
augustus	g2537.t1	UniRef100_K01652	1.40E-220	770.8
augustus	g2543.t1	UniRef100_K06316	9.60E-193	678.3
augustus	g2546.t1	UniRef100_K12249	3.20E-117	426.8
augustus	g2548.t1	UniRef100_K01941	0.00E+00	1955.6
augustus	g2550.t1	UniRef100_K07160	9.90E-127	458
augustus	g2553.t1	UniRef100_K00641	1.20E-180	637.5
augustus	g2560.t1	UniRef100_K01436	6.80E-56	223.4
augustus	g2569.t1	UniRef100_K01899	6.80E-124	448.7
augustus	g2573.t1	UniRef100_K07222	4.50E-122	443.7
augustus	g2581.t1	UniRef100_K08197	4.80E-59	234.2
augustus	g2585.t1	UniRef100_K11152	1.50E-33	148.7
augustus	g2591.t1	UniRef100_K00065	1.10E-79	301.6
augustus	g2600.t1	UniRef100_K05307	1.10E-74	284.6

augustus	g2601.t1	UniRef100_K15045	1.10E-147	527.7
augustus	g2606.t1	UniRef100_K01183	2.00E-126	457.2
augustus	g2607.t1	UniRef100_K05349	4.10E-114	417.9
augustus	g2612.t1	UniRef100_K08254	2.80E-76	291.6
augustus	g2617.t1	UniRef100_K13953	4.00E-64	250.4
augustus	g2624.t1	UniRef100_K01536	0.00E+00	1682.5
augustus	g2626.t1	UniRef100_K05882	1.90E-43	181.8
augustus	g2630.t1	UniRef100_K07824	6.30E-164	582.4
augustus	g2635.t1	UniRef100_K17649	2.70E-46	191.4
augustus	g2639.t1	UniRef100_K15388	9.80E-208	728
augustus	g2640.t1	UniRef100_K15384	0.00E+00	3681.3
augustus	g2643.t1	UniRef100_K01649	0.00E+00	1100.1
augustus	g2646.t1	UniRef100_K08137	3.90E-39	167.9
augustus	g2650.t1	UniRef100_K20302	2.00E-92	343.6
augustus	g2655.t1	UniRef100_K11213	1.40E-105	388.3
augustus	g2656.t1	UniRef100_K00463	4.00E-261	905.2
augustus	g2661.t1	UniRef100_K12183	1.20E-105	389
augustus	g2662.t1	UniRef100_K03010	0.00E+00	2470.3
augustus	g2663.t1	UniRef100_K18188	2.00E-30	136.3
augustus	g2664.t1	UniRef100_K02152	1.30E-13	80.9
augustus	g2665.t1	UniRef100_K12864	2.70E-256	889.4
augustus	g2666.t1	UniRef100_K21594	0.00E+00	1714.1
augustus	g2669.t1	UniRef100_K00253	6.60E-215	751.5
augustus	g2670.t1	UniRef100_K01969	4.00E-290	1001.9
augustus	g2675.t1	UniRef100_K03534	4.40E-13	79.7
augustus	g2685.t1	UniRef100_K00133	2.30E-185	653.3
augustus	g2687.t1	UniRef100_K05986	9.40E-93	345.5
augustus	g2690.t1	UniRef100_K10419	8.60E-28	128.3
augustus	g2691.t1	UniRef100_K14827	2.00E-167	593.6
augustus	g2692.t1	UniRef100_K11594	4.60E-254	882.1
augustus	g2693.t1	UniRef100_K00128	2.70E-108	397.9
augustus	g2694.t1	UniRef100_K01530	0.00E+00	2508
augustus	g2696.t1	UniRef100_K02150	6.10E-97	358.6
augustus	g2700.t1	UniRef100_K12412	1.20E-82	311.2
augustus	g2707.t1	UniRef100_K01638	2.70E-304	1048.9
augustus	g2711.t1	UniRef100_K07204	0.00E+00	2418.7
augustus	g2712.t1	UniRef100_K01897	0.00E+00	1243.8
augustus	g2713.t1	UniRef100_K11839	0.00E+00	1569.7
augustus	g2714.t1	UniRef100_K12182	2.40E-243	847
augustus	g2717.t1	UniRef100_K00966	6.60E-180	635.2
augustus	g2719.t1	UniRef100_K05954	4.10E-227	792.3
augustus	g2724.t1	UniRef100_K08770	0.00E+00	1409.8
augustus	g2729.t1	UniRef100_K11585	1.00E-70	272.3
augustus	g2732.t1	UniRef100_K02259	3.10E-237	825.9
augustus	g2733.t1	UniRef100_K14567	1.60E-229	801.2
augustus	g2734.t1	UniRef100_K07300	4.90E-184	649
augustus	g2735.t1	UniRef100_K01180	0.00E+00	1285
augustus	g2740.t1	UniRef100_K03379	7.20E-171	605.5
augustus	g2741.t1	UniRef100_K00354	2.20E-94	350.9
augustus	g2742.t1	UniRef100_K00799	2.10E-83	313.9

augustus	g2743.t1	UniRef100_K01426	8.60E-57	226.5
augustus	g2746.t1	UniRef100_K01673	1.20E-27	128.3
augustus	g2750.t1	UniRef100_K22134	1.10E-154	552
augustus	g2751.t1	UniRef100_K20308	0.00E+00	2050.8
augustus	g2752.t1	UniRef100_K17422	4.10E-54	215.3
augustus	g2754.t1	UniRef100_K14800	4.30E-80	302.8
augustus	g2758.t1	UniRef100_K09043	2.20E-104	384.8
augustus	g2759.t1	UniRef100_K08793	6.40E-243	844.7
augustus	g2762.t1	UniRef100_K22185	5.10E-61	239.6
augustus	g2763.t1	UniRef100_K16261	3.30E-289	998.8
augustus	g2768.t1	UniRef100_K00550	3.80E-85	319.3
augustus	g2769.t1	UniRef100_K12346	9.00E-226	788.1
augustus	g2776.t1	UniRef100_K03137	8.80E-142	508.1
augustus	g2778.t1	UniRef100_K00167	7.20E-278	961.4
augustus	g2782.t1	UniRef100_K00861	6.90E-74	282
augustus	g2786.t1	UniRef100_K00059	2.90E-23	114.4
augustus	g2794.t1	UniRef100_K01207	0.00E+00	1778.5
augustus	g2796.t1	UniRef100_K01443	8.10E-236	821.2
augustus	g2798.t1	UniRef100_K02564	9.00E-182	641.3
augustus	g2801.t1	UniRef100_K00844	1.00E-231	807.7
augustus	g2807.t1	UniRef100_K03657	0.00E+00	1218
augustus	g2808.t1	UniRef100_K12349	0.00E+00	1315.1
augustus	g2810.t1	UniRef100_K02992	1.80E-129	467.6
augustus	g2813.t1	UniRef100_K13126	0.00E+00	1109.7
augustus	g2814.t1	UniRef100_K10589	0.00E+00	1687.5
augustus	g2815.t1	UniRef100_K00311	0.00E+00	1238
augustus	g2816.t1	UniRef100_K03120	2.60E-122	443.4
augustus	g2817.t1	UniRef100_K00306	4.30E-228	795.4
augustus	g2818.t1	UniRef100_K12842	8.10E-267	924.9
augustus	g2826.t1	UniRef100_K03037	7.80E-250	867.8
augustus	g2827.t1	UniRef100_K00814	1.40E-264	916.8
augustus	g2830.t1	UniRef100_K14788	0.00E+00	1152.1
augustus	g2831.t1	UniRef100_K15165	0.00E+00	2131.7
augustus	g2835.t1	UniRef100_K00459	3.30E-95	353.6
augustus	g2838.t1	UniRef100_K00565	4.90E-227	792
augustus	g2839.t1	UniRef100_K07870	0.00E+00	1216.1
augustus	g2841.t1	UniRef100_K20062	2.60E-269	932.9
augustus	g2845.t1	UniRef100_K05355	1.40E-214	750.4
augustus	g2847.t1	UniRef100_K01466	8.10E-292	1007.3
augustus	g2856.t1	UniRef100_K01066	6.80E-37	159.8
augustus	g2859.t1	UniRef100_K00480	2.40E-217	759.6
augustus	g2860.t1	UniRef100_K08192	8.30E-73	279.6
augustus	g2861.t1	UniRef100_K17741	1.50E-55	221.9
augustus	g2864.t1	UniRef100_K09001	2.00E-65	255
augustus	g2868.t1	UniRef100_K07119	2.20E-176	623.2
augustus	g2869.t1	UniRef100_K08192	2.20E-60	238.4
augustus	g2877.t1	UniRef100_K00485	1.80E-249	866.7
augustus	g2887.t1	UniRef100_K21421	0.00E+00	1135.2
augustus	g2888.t1	UniRef100_K06689	1.60E-74	283.9
augustus	g2889.t1	UniRef100_K00252	9.60E-225	784.3

augustus	g2893.t1	UniRef100_K00889	0.00E+00	1233.8
augustus	g2894.t1	UniRef100_K17862	0.00E+00	1827.8
augustus	g2896.t1	UniRef100_K11866	2.90E-226	789.6
augustus	g2897.t1	UniRef100_K04798	1.30E-43	180.6
augustus	g2898.t1	UniRef100_K12196	5.40E-233	811.6
augustus	g2905.t1	UniRef100_K00306	3.90E-228	795.8
augustus	g2908.t1	UniRef100_K20246	3.20E-181	639.4
augustus	g2909.t1	UniRef100_K00772	4.90E-170	602.1
augustus	g2910.t1	UniRef100_K18932	0.00E+00	1389
augustus	g2911.t1	UniRef100_K02858	1.80E-114	417.2
augustus	g2912.t1	UniRef100_K00568	4.30E-93	346.3
augustus	g2917.t1	UniRef100_K01824	1.30E-107	394.4
augustus	g2919.t1	UniRef100_K06675	0.00E+00	1790.4
augustus	g2920.t1	UniRef100_K02976	3.10E-48	196.4
augustus	g2921.t1	UniRef100_K02977	2.90E-80	302.8
augustus	g2922.t1	UniRef100_K14564	1.70E-223	780.4
augustus	g2923.t1	UniRef100_K01183	5.70E-224	781.6
augustus	g2933.t1	UniRef100_K09983	5.50E-72	275.4
augustus	g2935.t1	UniRef100_K01619	1.50E-100	370.9
augustus	g2939.t1	UniRef100_K01476	1.80E-170	603.6
augustus	g2940.t1	UniRef100_K00380	0.00E+00	1804.3
augustus	g2941.t1	UniRef100_K17686	2.30E-110	405.6
augustus	g2942.t1	UniRef100_K06656	3.60E-28	131
augustus	g2943.t1	UniRef100_K04563	1.50E-185	653.7
augustus	g2946.t1	UniRef100_K17803	2.20E-164	583.6
augustus	g2949.t1	UniRef100_K06998	2.40E-25	121.3
augustus	g2950.t1	UniRef100_K12604	0.00E+00	3960.6
augustus	g2956.t1	UniRef100_K04705	4.30E-204	716.5
augustus	g2957.t1	UniRef100_K08331	0.00E+00	1206
augustus	g2959.t1	UniRef100_K15110	1.40E-151	540.8
augustus	g2962.t1	UniRef100_K12471	8.30E-187	658.7
augustus	g2963.t1	UniRef100_K16466	2.80E-108	396.4
augustus	g2964.t1	UniRef100_K14297	0.00E+00	2269.6
augustus	g2965.t1	UniRef100_K11378	0.00E+00	1271.1
augustus	g2967.t1	UniRef100_K14168	6.60E-214	748
augustus	g2968.t1	UniRef100_K02936	3.20E-119	433
augustus	g2972.t1	UniRef100_K08192	1.00E-76	292.7
augustus	g2975.t1	UniRef100_K01610	0.00E+00	1128.6
augustus	g2976.t1	UniRef100_K03267	2.00E-271	940.3
augustus	g2981.t1	UniRef100_K03115	7.20E-180	634.8
augustus	g2982.t1	UniRef100_K12571	0.00E+00	1844.3
augustus	g2987.t1	UniRef100_K10838	0.00E+00	1083.9
augustus	g2988.t1	UniRef100_K15104	9.30E-158	561.2
augustus	g2993.t1	UniRef100_K08869	0.00E+00	1200.3
augustus	g2994.t1	UniRef100_K01764	4.50E-167	592.4
augustus	g2996.t1	UniRef100_K12839	1.50E-91	341.3
augustus	g2997.t1	UniRef100_K03593	8.10E-143	511.5
augustus	g2998.t1	UniRef100_K14782	1.10E-158	565.1
augustus	g2999.t1	UniRef100_K01555	2.10E-227	793.1
augustus	g3002.t1	UniRef100_K00596	8.90E-133	478.8

augustus	g3013.t1	UniRef100_K00451	1.00E-259	900.6
augustus	g3016.t1	UniRef100_K07277	1.90E-273	946.4
augustus	g3017.t1	UniRef100_K08711	0.00E+00	1762.7
augustus	g3025.t1	UniRef100_K07513	4.60E-181	639
augustus	g3030.t1	UniRef100_K19305	3.80E-126	456.4
augustus	g3032.t1	UniRef100_K13289	2.90E-63	248.1
augustus	g3033.t1	UniRef100_K07750	8.00E-67	258.8
augustus	g3034.t1	UniRef100_K11229	0.00E+00	2102.4
augustus	g3040.t1	UniRef100_K01626	4.00E-200	702.2
augustus	g3043.t1	UniRef100_K02873	3.10E-73	280.4
augustus	g3045.t1	UniRef100_K17805	2.70E-64	249.6
augustus	g3046.t1	UniRef100_K01942	0.00E+00	1158.3
augustus	g3050.t1	UniRef100_K00549	2.60E-62	244.6
augustus	g3056.t1	UniRef100_K08712	9.60E-274	948.7
augustus	g3059.t1	UniRef100_K17362	8.60E-46	188.3
augustus	g3060.t1	UniRef100_K03144	1.80E-256	889.8
augustus	g3064.t1	UniRef100_K01230	0.00E+00	1285
augustus	g3066.t1	UniRef100_K20474	0.00E+00	1344.3
augustus	g3067.t1	UniRef100_K15109	1.40E-156	557.4
augustus	g3069.t1	UniRef100_K01130	1.70E-152	544.7
augustus	g3071.t1	UniRef100_K20290	0.00E+00	1283.9
augustus	g3073.t1	UniRef100_K01104	1.20E-228	797.3
augustus	g3077.t1	UniRef100_K11359	0.00E+00	1753.8
augustus	g3078.t1	UniRef100_K00600	1.40E-256	890.2
augustus	g3081.t1	UniRef100_K15033	3.40E-77	292.7
augustus	g3082.t1	UniRef100_K08794	2.20E-66	258.1
augustus	g3086.t1	UniRef100_K06947	0.00E+00	1109.7
augustus	g3087.t1	UniRef100_K03380	1.40E-225	788.1
augustus	g3092.t1	UniRef100_K08518	0.00E+00	1746.1
augustus	g3093.t1	UniRef100_K11593	0.00E+00	1699.5
augustus	g3094.t1	UniRef100_K14408	0.00E+00	1658.3
augustus	g3097.t1	UniRef100_K05767	0.00E+00	1403.3
augustus	g3098.t1	UniRef100_K11968	9.80E-305	1050.8
augustus	g3099.t1	UniRef100_K10746	2.00E-297	1026.5
augustus	g3100.t1	UniRef100_K14860	4.00E-73	279.3
augustus	g3101.t1	UniRef100_K15414	5.80E-122	442.2
augustus	g3102.t1	UniRef100_K20465	9.10E-243	844.3
augustus	g3105.t1	UniRef100_K21766	2.10E-139	500.7
augustus	g3107.t1	UniRef100_K15633	2.20E-290	1002.7
augustus	g3109.t1	UniRef100_K05906	3.60E-249	865.9
augustus	g3110.t1	UniRef100_K01620	4.70E-190	668.7
augustus	g3111.t1	UniRef100_K06221	4.60E-69	266.5
augustus	g3117.t1	UniRef100_K16794	3.30E-252	875.5
augustus	g3120.t1	UniRef100_K07827	2.30E-21	107.8
augustus	g3122.t1	UniRef100_K01580	1.60E-256	890.2
augustus	g3125.t1	UniRef100_K03860	0.00E+00	1272.3
augustus	g3128.t1	UniRef100_K01785	8.70E-170	601.3
augustus	g3130.t1	UniRef100_K18184	5.40E-79	298.9
augustus	g3131.t1	UniRef100_K02324	0.00E+00	4259.1
augustus	g3132.t1	UniRef100_K18667	7.60E-242	841.6

augustus	g3133.t1	UniRef100_K02969	1.20E-47	194.5
augustus	g3134.t1	UniRef100_K18716	9.60E-118	429.1
augustus	g3137.t1	UniRef100_K14557	5.40E-169	599
augustus	g3139.t1	UniRef100_K11771	0.00E+00	1557.7
augustus	g3140.t1	UniRef100_K17430	9.20E-48	194.9
augustus	g3141.t1	UniRef100_K03250	9.60E-236	820.8
augustus	g3142.t1	UniRef100_K03348	0.00E+00	3367
augustus	g3143.t1	UniRef100_K20184	0.00E+00	2060
augustus	g3147.t1	UniRef100_K02434	5.5e-311	1071.2
augustus	g3154.t1	UniRef100_K11849	0.00E+00	1766.9
augustus	g3155.t1	UniRef100_K05607	9.70E-135	485
augustus	g3156.t1	UniRef100_K12859	0.00E+00	1398.6
augustus	g3159.t1	UniRef100_K00485	1.30E-255	887.1
augustus	g3161.t1	UniRef100_K00697	1.70E-294	1016.1
augustus	g3162.t1	UniRef100_K12591	0.00E+00	1107.8
augustus	g3163.t1	UniRef100_K02128	6.00E-56	221.9
augustus	g3167.t1	UniRef100_K03033	3.90E-279	965.3
augustus	g3169.t1	UniRef100_K03126	1.50E-186	658.3
augustus	g3174.t1	UniRef100_K03031	1.10E-32	146.4
augustus	g3175.t1	UniRef100_K00558	0.00E+00	1686.4
augustus	g3177.t1	UniRef100_K03063	1.60E-199	700.7
augustus	g3179.t1	UniRef100_K11561	3.90E-85	319.7
augustus	g3183.t1	UniRef100_K01381	7.30E-201	704.9
augustus	g3185.t1	UniRef100_K11244	6.30E-79	299.3
augustus	g3187.t1	UniRef100_K04799	3.80E-194	682.6
augustus	g3189.t1	UniRef100_K14402	0.00E+00	1610.1
augustus	g3194.t1	UniRef100_K21631	3.20E-138	496.9
augustus	g3195.t1	UniRef100_K01893	1.80E-238	830.1
augustus	g3198.t1	UniRef100_K14007	0.00E+00	1795.8
augustus	g3201.t1	UniRef100_K20318	1.70E-74	284.3
augustus	g3202.t1	UniRef100_K18179	3.60E-66	255.8
augustus	g3205.t1	UniRef100_K01198	2.30E-305	1052.4
augustus	g3207.t1	UniRef100_K00128	3.50E-124	450.3
augustus	g3208.t1	UniRef100_K11153	2.00E-35	154.8
augustus	g3213.t1	UniRef100_K08637	7.20E-58	229.9
augustus	g3217.t1	UniRef100_K18278	2.00E-186	656.8
augustus	g3220.t1	UniRef100_K08176	5.20E-285	984.9
augustus	g3221.t1	UniRef100_K01539	0.00E+00	1910.6
augustus	g3228.t1	UniRef100_K00062	1.60E-63	248.4
augustus	g3237.t1	UniRef100_K08288	7.00E-250	868.2
augustus	g3239.t1	UniRef100_K07203	0.00E+00	4630.5
augustus	g3247.t1	UniRef100_K03514	3.60E-234	816.2
augustus	g3249.t1	UniRef100_K20498	1.20E-88	332.4
augustus	g3250.t1	UniRef100_K18123	2.20E-157	560.1
augustus	g3251.t1	UniRef100_K09022	7.90E-31	138.3
augustus	g3254.t1	UniRef100_K00249	5.70E-214	748.4
augustus	g3255.t1	UniRef100_K08150	1.90E-52	212.2
augustus	g3257.t1	UniRef100_K01188	1.80E-272	943
augustus	g3268.t1	UniRef100_K07101	3.30E-108	396
augustus	g3270.t1	UniRef100_K15388	1.90E-56	225.3

augustus	g3277.t1	UniRef100_K12600	0.00E+00	2360.5
augustus	g3278.t1	UniRef100_K15364	1.40E-57	228
augustus	g3279.t1	UniRef100_K20296	1.40E-103	381.3
augustus	g3282.t1	UniRef100_K12392	4.40E-177	626.7
augustus	g3283.t1	UniRef100_K10747	0.00E+00	1453.3
augustus	g3284.t1	UniRef100_K15028	3.50E-122	442.6
augustus	g3285.t1	UniRef100_K14801	1.70E-133	481.1
augustus	g3290.t1	UniRef100_K09499	3.60E-306	1055
augustus	g3292.t1	UniRef100_K01621	0.00E+00	1626.3
augustus	g3301.t1	UniRef100_K10403	6.00E-173	612.8
augustus	g3305.t1	UniRef100_K02295	0.00E+00	1110.1
augustus	g3306.t1	UniRef100_K00108	4.90E-75	287.3
augustus	g3308.t1	UniRef100_K08735	0.00E+00	1672.5
augustus	g3311.t1	UniRef100_K02684	2.40E-220	770
augustus	g3312.t1	UniRef100_K15859	2.60E-303	1045.8
augustus	g3314.t1	UniRef100_K09131	2.60E-52	209.5
augustus	g3317.t1	UniRef100_K03240	2.30E-279	966.5
augustus	g3322.t1	UniRef100_K15026	0.00E+00	1063.9
augustus	g3323.t1	UniRef100_K20826	5.10E-172	609
augustus	g3325.t1	UniRef100_K12404	1.10E-275	954.1
augustus	g3326.t1	UniRef100_K09140	9.80E-123	445.3
augustus	g3327.t1	UniRef100_K02519	2.80E-307	1059.7
augustus	g3329.t1	UniRef100_K15620	5.20E-159	565.8
augustus	g3333.t1	UniRef100_K01027	2.40E-286	989.2
augustus	g3334.t1	UniRef100_K01759	4.40E-21	105.9
augustus	g3335.t1	UniRef100_K18107	1.40E-143	514.2
augustus	g3336.t1	UniRef100_K14544	0.00E+00	1989.9
augustus	g3337.t1	UniRef100_K18725	0.00E+00	2230.3
augustus	g3338.t1	UniRef100_K05610	6.10E-179	632.5
augustus	g3339.t1	UniRef100_K10301	4.70E-305	1051.6
augustus	g3349.t1	UniRef100_K17103	1.60E-107	394.4
augustus	g3353.t1	UniRef100_K00679	0.00E+00	1146
augustus	g3354.t1	UniRef100_K00670	3.20E-87	326.2
augustus	g3356.t1	UniRef100_K17868	4.90E-186	655.6
augustus	g3357.t1	UniRef100_K14289	0.00E+00	2179.1
augustus	g3362.t1	UniRef100_K01719	2.60E-121	440.3
augustus	g3363.t1	UniRef100_K17783	5.70E-80	302.4
augustus	g3364.t1	UniRef100_K01568	1.20E-289	1000.3
augustus	g3365.t1	UniRef100_K06963	5.60E-94	349.4
augustus	g3368.t1	UniRef100_K18587	1.40E-102	377.9
augustus	g3371.t1	UniRef100_K09523	3.70E-239	832.4
augustus	g3372.t1	UniRef100_K15113	1.40E-148	531.2
augustus	g3373.t1	UniRef100_K17907	0.00E+00	1285.4
augustus	g3374.t1	UniRef100_K00382	3.00E-258	896
augustus	g3375.t1	UniRef100_K17583	0.00E+00	1154.8
augustus	g3377.t1	UniRef100_K14854	5.30E-13	78.2
augustus	g3378.t1	UniRef100_K11366	3.60E-259	899
augustus	g3380.t1	UniRef100_K13076	9.60E-297	1023.8
augustus	g3381.t1	UniRef100_K11251	8.50E-62	241.5
augustus	g3382.t1	UniRef100_K11252	1.30E-39	167.9

augustus	g3387.t1	UniRef100_K02961	2.10E-63	246.5
augustus	g3389.t1	UniRef100_K01739	1.50E-256	890.2
augustus	g3390.t1	UniRef100_K16275	2.10E-133	481.1
augustus	g3392.t1	UniRef100_K15305	0.00E+00	1469.9
augustus	g3393.t1	UniRef100_K01681	0.00E+00	1497.6
augustus	g3394.t1	UniRef100_K10875	0.00E+00	1855.1
augustus	g3395.t1	UniRef100_K15318	7.20E-61	239.2
augustus	g3396.t1	UniRef100_K03039	3.30E-195	686
augustus	g3397.t1	UniRef100_K05289	8.10E-238	828.2
augustus	g3398.t1	UniRef100_K00654	4.30E-270	935.3
augustus	g3400.t1	UniRef100_K20293	1.30E-297	1026.9
augustus	g3402.t1	UniRef100_K20222	0.00E+00	1843.2
augustus	g3403.t1	UniRef100_K14568	7.20E-132	474.9
augustus	g3404.t1	UniRef100_K14821	4.80E-35	152.5
augustus	g3405.t1	UniRef100_K02307	4.00E-42	176.4
augustus	g3406.t1	UniRef100_K09506	1.70E-183	647.5
augustus	g3407.t1	UniRef100_K08342	9.90E-194	681.4
augustus	g3408.t1	UniRef100_K00658	2.60E-176	623.2
augustus	g3412.t1	UniRef100_K11756	0.00E+00	1085.9
augustus	g3413.t1	UniRef100_K00706	0.00E+00	3441.7
augustus	g3414.t1	UniRef100_K11718	0.00E+00	2362.8
augustus	g3415.t1	UniRef100_K05236	0.00E+00	1761.5
augustus	g3416.t1	UniRef100_K03031	5.70E-140	501.9
augustus	g3425.t1	UniRef100_K14709	3.20E-69	267.7
augustus	g3430.t1	UniRef100_K12471	7.50E-116	422.9
augustus	g3432.t1	UniRef100_K03025	1.70E-173	614
augustus	g3434.t1	UniRef100_K03030	4.30E-185	652.1
augustus	g3440.t1	UniRef100_K01569	3.60E-121	440.3
augustus	g3441.t1	UniRef100_K14708	0.00E+00	1170.2
augustus	g3444.t1	UniRef100_K17645	2.10E-80	305.1
augustus	g3447.t1	UniRef100_K11153	2.30E-47	194.5
augustus	g3455.t1	UniRef100_K02971	3.60E-43	178.7
augustus	g3456.t1	UniRef100_K01669	0.00E+00	1087
augustus	g3465.t1	UniRef100_K00027	4.50E-294	1015
augustus	g3470.t1	UniRef100_K00001	1.10E-43	182.6
augustus	g3471.t1	UniRef100_K01192	0.00E+00	1424.1
augustus	g3472.t1	UniRef100_K07880	5.10E-35	153.7
augustus	g3473.t1	UniRef100_K08683	3.00E-41	174.1
augustus	g3474.t1	UniRef100_K00018	1.80E-91	341.3
augustus	g3477.t1	UniRef100_K08141	1.10E-83	315.8
augustus	g3479.t1	UniRef100_K13303	4.50E-273	945.3
augustus	g3480.t1	UniRef100_K07304	2.80E-66	256.9
augustus	g3481.t1	UniRef100_K21890	1.60E-25	122.1
augustus	g3483.t1	UniRef100_K01754	1.30E-296	1023.5
augustus	g3487.t1	UniRef100_K14833	0.00E+00	1117.1
augustus	g3489.t1	UniRef100_K12184	4.90E-97	359.4
augustus	g3490.t1	UniRef100_K17765	1.00E-74	285.8
augustus	g3491.t1	UniRef100_K00921	0.00E+00	3429.4
augustus	g3493.t1	UniRef100_K22066	2.40E-39	167.2
augustus	g3495.t1	UniRef100_K03259	2.50E-126	456.8

augustus	g3503.t1	UniRef100_K01183	2.40E-164	583.6
augustus	g3507.t1	UniRef100_K14688	4.50E-212	742.7
augustus	g3512.t1	UniRef100_K02981	1.60E-107	394.4
augustus	g3513.t1	UniRef100_K01011	5.80E-177	625.2
augustus	g3515.t1	UniRef100_K03358	1.20E-50	203.8
augustus	g3516.t1	UniRef100_K02216	8.80E-302	1040.4
augustus	g3517.t1	UniRef100_K06688	9.10E-94	347.8
augustus	g3518.t1	UniRef100_K02910	2.40E-58	229.6
augustus	g3520.t1	UniRef100_K12180	6.90E-80	302.4
augustus	g3521.t1	UniRef100_K17686	0.00E+00	1813.9
augustus	g3524.t1	UniRef100_K15280	7.10E-21	106.7
augustus	g3526.t1	UniRef100_K05767	0.00E+00	2121.7
augustus	g3527.t1	UniRef100_K01133	3.50E-170	603.2
augustus	g3533.t1	UniRef100_K03539	6.90E-121	438.7
augustus	g3536.t1	UniRef100_K09481	9.30E-20	101.3
augustus	g3537.t1	UniRef100_K15731	1.40E-213	747.3
augustus	g3540.t1	UniRef100_K15628	0.00E+00	1443.7
augustus	g3543.t1	UniRef100_K17743	7.40E-149	531.6
augustus	g3544.t1	UniRef100_K17550	1.50E-45	188.7
augustus	g3548.t1	UniRef100_K02882	2.60E-87	326.6
augustus	g3553.t1	UniRef100_K08139	4.50E-51	207.6
augustus	g3554.t1	UniRef100_K01792	7.10E-157	558.5
augustus	g3555.t1	UniRef100_K01626	4.20E-258	895.2
augustus	g3560.t1	UniRef100_K18932	2.30E-151	540.4
augustus	g3561.t1	UniRef100_K10356	0.00E+00	2088.5
augustus	g3564.t1	UniRef100_K09569	2.00E-52	210.7
augustus	g3565.t1	UniRef100_K03511	4.10E-296	1021.9
augustus	g3568.t1	UniRef100_K03809	1.40E-99	367.5
augustus	g3576.t1	UniRef100_K12816	2.30E-270	936
augustus	g3577.t1	UniRef100_K20368	2.80E-63	246.5
augustus	g3578.t1	UniRef100_K01265	2.50E-218	763.1
augustus	g3579.t1	UniRef100_K00486	2.40E-260	902.9
augustus	g3580.t1	UniRef100_K02994	1.10E-48	198
augustus	g3582.t1	UniRef100_K02355	0.00E+00	1483.4
augustus	g3586.t1	UniRef100_K05681	0.00E+00	1501.1
augustus	g3588.t1	UniRef100_K14537	1.20E-307	1060.1
augustus	g3592.t1	UniRef100_K17738	3.40E-40	170.6
augustus	g3607.t1	UniRef100_K03929	2.60E-49	201.8
augustus	g3611.t1	UniRef100_K20984	0.00E+00	1694.1
augustus	g3614.t1	UniRef100_K22134	1.60E-91	342
augustus	g3616.t1	UniRef100_K00480	3.80E-157	559.7
augustus	g3620.t1	UniRef100_K14209	3.80E-251	872.5
augustus	g3630.t1	UniRef100_K18281	6.20E-280	969.9
augustus	g3632.t1	UniRef100_K05658	0.00E+00	1883.6
augustus	g3633.t1	UniRef100_K01537	0.00E+00	1954.9
augustus	g3637.t1	UniRef100_K08157	6.10E-56	223.8
augustus	g3638.t1	UniRef100_K00873	2.50E-296	1022.3
augustus	g3643.t1	UniRef100_K11805	3.10E-308	1062
augustus	g3644.t1	UniRef100_K15172	0.00E+00	1305
augustus	g3646.t1	UniRef100_K01904	1.10E-105	389

augustus	g3647.t1	UniRef100_K00059	1.30E-38	165.2
augustus	g3651.t1	UniRef100_K00387	8.00E-305	1050.8
augustus	g3652.t1	UniRef100_K03032	0.00E+00	1899.4
augustus	g3654.t1	UniRef100_K03458	2.10E-238	830.1
augustus	g3658.t1	UniRef100_K01126	3.40E-200	702.6
augustus	g3662.t1	UniRef100_K01733	6.40E-267	924.9
augustus	g3668.t1	UniRef100_K17866	3.00E-249	865.9
augustus	g3670.t1	UniRef100_K08737	0.00E+00	1900.2
augustus	g3672.t1	UniRef100_K00616	1.10E-63	248.8
augustus	g3673.t1	UniRef100_K02257	9.70E-194	681.4
augustus	g3682.t1	UniRef100_K14674	0.00E+00	1261.5
augustus	g3683.t1	UniRef100_K03386	1.50E-75	287.7
augustus	g3686.t1	UniRef100_K00666	1.30E-89	335.9
augustus	g3689.t1	UniRef100_K19564	2.80E-69	268.1
augustus	g3695.t1	UniRef100_K01276	2.90E-251	872.5
augustus	g3707.t1	UniRef100_K13953	5.90E-177	625.2
augustus	g3709.t1	UniRef100_K03932	2.00E-31	141.7
augustus	g3710.t1	UniRef100_K05994	1.50E-208	730.7
augustus	g3718.t1	UniRef100_K08254	1.20E-104	385.2
augustus	g3720.t1	UniRef100_K08157	1.60E-110	405.2
augustus	g3721.t1	UniRef100_K14333	8.00E-198	694.5
augustus	g3723.t1	UniRef100_K00480	2.30E-233	813.1
augustus	g3724.t1	UniRef100_K03381	8.30E-151	538.1
augustus	g3727.t1	UniRef100_K17806	5.50E-126	456.4
augustus	g3729.t1	UniRef100_K00001	8.10E-38	162.9
augustus	g3732.t1	UniRef100_K15100	4.60E-127	459.1
augustus	g3733.t1	UniRef100_K17663	7.40E-17	91.7
augustus	g3735.t1	UniRef100_K00573	2.20E-98	363.6
augustus	g3740.t1	UniRef100_K03941	3.30E-65	253.8
augustus	g3741.t1	UniRef100_K03955	1.10E-51	207.6
augustus	g3748.t1	UniRef100_K11273	0.00E+00	1427.5
augustus	g3749.t1	UniRef100_K02900	5.90E-66	255.8
augustus	g3750.t1	UniRef100_K02214	1.60E-225	786.9
augustus	g3751.t1	UniRef100_K06677	0.00E+00	1968
augustus	g3752.t1	UniRef100_K15732	1.50E-291	1006.9
augustus	g3753.t1	UniRef100_K11153	1.10E-28	132.5
augustus	g3756.t1	UniRef100_K05841	0.00E+00	2159
augustus	g3757.t1	UniRef100_K00621	1.10E-76	291.2
augustus	g3764.t1	UniRef100_K19355	9.70E-165	584.7
augustus	g3765.t1	UniRef100_K18579	5.70E-252	874.8
augustus	g3768.t1	UniRef100_K02370	1.60E-146	524.2
augustus	g3775.t1	UniRef100_K10536	2.00E-143	513.8
augustus	g3777.t1	UniRef100_K01053	3.20E-176	623.2
augustus	g3782.t1	UniRef100_K01050	9.00E-132	475.7
augustus	g3793.t1	UniRef100_K17741	1.20E-23	115.9
augustus	g3795.t1	UniRef100_K01066	2.90E-40	171
augustus	g3798.t1	UniRef100_K08158	1.40E-195	687.6
augustus	g3799.t1	UniRef100_K08139	2.20E-245	853.2
augustus	g3800.t1	UniRef100_K16055	0.00E+00	1651.7
augustus	g3804.t1	UniRef100_K07755	1.80E-54	218

augustus	g3805.t1	UniRef100_K17871	4.00E-116	423.3
augustus	g3809.t1	UniRef100_K00218	2.70E-39	167.9
augustus	g3811.t1	UniRef100_K05607	2.40E-268	929.9
augustus	g3817.t1	UniRef100_K19329	6.00E-35	153.3
augustus	g3823.t1	UniRef100_K11153	9.20E-33	146
augustus	g3826.t1	UniRef100_K08141	1.20E-168	598.2
augustus	g3827.t1	UniRef100_K01130	2.20E-187	660.6
augustus	g3830.t1	UniRef100_K08022	6.30E-179	632.5
augustus	g3836.t1	UniRef100_K20985	2.10E-22	111.3
augustus	g3839.t1	UniRef100_K09553	6.40E-182	642.5
augustus	g3840.t1	UniRef100_K10215	3.00E-102	377.5
augustus	g3844.t1	UniRef100_K11099	3.90E-30	136
augustus	g3845.t1	UniRef100_K18064	2.50E-50	203.4
augustus	g3850.t1	UniRef100_K00326	2.70E-140	503.8
augustus	g3852.t1	UniRef100_K06287	3.80E-123	446
augustus	g3854.t1	UniRef100_K01868	2.30E-272	942.6
augustus	g3856.t1	UniRef100_K00344	3.30E-177	625.9
augustus	g3857.t1	UniRef100_K04523	5.20E-154	549.3
augustus	g3858.t1	UniRef100_K20405	4.70E-211	738.8
augustus	g3859.t1	UniRef100_K01866	0.00E+00	1128.6
augustus	g3860.t1	UniRef100_K00237	7.90E-79	298.1
augustus	g3861.t1	UniRef100_K13621	0.00E+00	1545.8
augustus	g3863.t1	UniRef100_K11315	2.80E-289	999.2
augustus	g3864.t1	UniRef100_K01427	0.00E+00	1547.7
augustus	g3873.t1	UniRef100_K07300	1.40E-191	674.9
augustus	g3874.t1	UniRef100_K08286	0.00E+00	1136.7
augustus	g3875.t1	UniRef100_K05283	1.60E-249	866.7
augustus	g3879.t1	UniRef100_K19639	9.80E-56	222.6
augustus	g3880.t1	UniRef100_K22122	6.60E-93	346.7
augustus	g3883.t1	UniRef100_K15866	1.70E-28	131.7
augustus	g3887.t1	UniRef100_K10398	0.00E+00	1555.8
augustus	g3888.t1	UniRef100_K10866	0.00E+00	1703.7
augustus	g3895.t1	UniRef100_K17778	6.70E-42	174.9
augustus	g3897.t1	UniRef100_K00759	2.00E-81	307.4
augustus	g3898.t1	UniRef100_K10084	3.80E-227	793.5
augustus	g3901.t1	UniRef100_K21802	9.90E-111	405.6
augustus	g3902.t1	UniRef100_K17656	1.20E-72	278.1
augustus	g3904.t1	UniRef100_K06664	3.70E-226	788.9
augustus	g3907.t1	UniRef100_K05293	8.00E-214	748
augustus	g3908.t1	UniRef100_K17714	1.30E-197	694.1
augustus	g3911.t1	UniRef100_K01870	0.00E+00	1784.2
augustus	g3913.t1	UniRef100_K21797	0.00E+00	1314.7
augustus	g3918.t1	UniRef100_K07889	1.20E-112	411
augustus	g3919.t1	UniRef100_K17785	5.50E-240	835.5
augustus	g3920.t1	UniRef100_K01930	6.00E-169	599
augustus	g3921.t1	UniRef100_K01930	6.00E-240	835.1
augustus	g3923.t1	UniRef100_K17560	1.70E-205	720.7
augustus	g3925.t1	UniRef100_K02949	1.60E-74	284.3
augustus	g3926.t1	UniRef100_K11563	0.00E+00	1483.8
augustus	g3927.t1	UniRef100_K13348	3.40E-86	323.2

augustus	g3929.t1	UniRef100_K14521	0.00E+00	1843.2
augustus	g3930.t1	UniRef100_K00863	3.80E-276	955.7
augustus	g3931.t1	UniRef100_K18123	2.40E-164	583.2
augustus	g3937.t1	UniRef100_K15544	2.30E-111	406.8
augustus	g3939.t1	UniRef100_K00326	2.20E-156	556.6
augustus	g3942.t1	UniRef100_K03283	0.00E+00	1131.3
augustus	g3949.t1	UniRef100_K01301	0.00E+00	1298.9
augustus	g3957.t1	UniRef100_K10761	2.30E-115	420.6
augustus	g3958.t1	UniRef100_K14961	1.50E-231	807
augustus	g3960.t1	UniRef100_K03006	0.00E+00	3035.4
augustus	g3967.t1	UniRef100_K14026	0.00E+00	1209.5
augustus	g3968.t1	UniRef100_K03648	9.20E-168	594.7
augustus	g3972.t1	UniRef100_K01872	0.00E+00	1785
augustus	g3974.t1	UniRef100_K01738	3.80E-226	788.9
augustus	g3975.t1	UniRef100_K03145	7.90E-130	468.4
augustus	g3976.t1	UniRef100_K10846	0.00E+00	1462.6
augustus	g3977.t1	UniRef100_K20824	4.60E-45	185.3
augustus	g3986.t1	UniRef100_K04091	1.30E-197	694.5
augustus	g3987.t1	UniRef100_K09567	9.80E-93	344.7
augustus	g3991.t1	UniRef100_K14787	0.00E+00	1200.7
augustus	g3992.t1	UniRef100_K09448	0.00E+00	1426
augustus	g3994.t1	UniRef100_K10418	5.90E-50	201.4
augustus	g3996.t1	UniRef100_K15183	1.90E-213	747.3
augustus	g4000.t1	UniRef100_K03007	1.70E-32	143.7
augustus	g4001.t1	UniRef100_K14552	0.00E+00	1514.6
augustus	g4009.t1	UniRef100_K20813	1.50E-115	421
augustus	g4010.t1	UniRef100_K00972	1.20E-247	860.5
augustus	g4011.t1	UniRef100_K00384	1.00E-167	594.3
augustus	g4012.t1	UniRef100_K20299	0.00E+00	1441.4
augustus	g4014.t1	UniRef100_K15728	0.00E+00	1152.1
augustus	g4015.t1	UniRef100_K14152	0.00E+00	1549.6
augustus	g4016.t1	UniRef100_K03133	3.90E-77	293.1
augustus	g4017.t1	UniRef100_K17732	4.60E-247	858.6
augustus	g4019.t1	UniRef100_K11400	1.40E-261	906.7
augustus	g4020.t1	UniRef100_K04567	2.20E-307	1059.3
augustus	g4021.t1	UniRef100_K14779	0.00E+00	1066.2
augustus	g4023.t1	UniRef100_K20178	0.00E+00	2683.7
augustus	g4024.t1	UniRef100_K00088	1.20E-285	986.9
augustus	g4031.t1	UniRef100_K00135	0.00E+00	1181.8
augustus	g4034.t1	UniRef100_K06269	9.60E-259	897.5
augustus	g4039.t1	UniRef100_K00008	1.40E-206	723.8
augustus	g4040.t1	UniRef100_K02140	5.60E-67	259.2
augustus	g4043.t1	UniRef100_K03122	1.70E-97	361.3
augustus	g4044.t1	UniRef100_K03363	5.40E-263	911.8
augustus	g4050.t1	UniRef100_K05544	0.00E+00	1265.4
augustus	g4051.t1	UniRef100_K13344	2.80E-142	510.4
augustus	g4053.t1	UniRef100_K10751	3.20E-294	1015.8
augustus	g4054.t1	UniRef100_K05665	4.20E-166	591.3
augustus	g4056.t1	UniRef100_K08334	5.60E-202	708.8
augustus	g4058.t1	UniRef100_K03097	9.60E-196	687.6

augustus	g4066.t1	UniRef100_K08008	0.00E+00	1104.7
augustus	g4068.t1	UniRef100_K01286	6.30E-68	263.1
augustus	g4074.t1	UniRef100_K02830	8.60E-155	551.6
augustus	g4075.t1	UniRef100_K06062	1.10E-227	793.9
augustus	g4078.t1	UniRef100_K00560	8.10E-176	621.3
augustus	g4080.t1	UniRef100_K09660	2.70E-122	443.4
augustus	g4083.t1	UniRef100_K01456	1.30E-219	767.3
augustus	g4084.t1	UniRef100_K14548	0.00E+00	1482.2
augustus	g4086.t1	UniRef100_K07034	1.60E-112	411
augustus	g4092.t1	UniRef100_K12451	5.00E-84	316.2
augustus	g4093.t1	UniRef100_K00801	5.30E-232	808.5
augustus	g4094.t1	UniRef100_K18549	1.90E-61	241.9
augustus	g4098.t1	UniRef100_K05284	3.60E-208	729.2
augustus	g4107.t1	UniRef100_K13704	9.40E-177	624.8
augustus	g4108.t1	UniRef100_K00671	6.90E-273	944.5
augustus	g4109.t1	UniRef100_K12667	6.40E-233	812
augustus	g4110.t1	UniRef100_K00683	8.40E-81	305.8
augustus	g4111.t1	UniRef100_K00993	6.90E-236	821.2
augustus	g4117.t1	UniRef100_K03128	0.00E+00	2408.3
augustus	g4118.t1	UniRef100_K00763	5.20E-258	894.8
augustus	g4119.t1	UniRef100_K11644	0.00E+00	2498
augustus	g4120.t1	UniRef100_K00626	9.10E-173	611.3
augustus	g4121.t1	UniRef100_K12852	0.00E+00	1845.5
augustus	g4128.t1	UniRef100_K13122	9.10E-104	381.7
augustus	g4130.t1	UniRef100_K15119	7.10E-180	635.2
augustus	g4131.t1	UniRef100_K10295	2.50E-206	723.4
augustus	g4141.t1	UniRef100_K09265	6.60E-211	738.8
augustus	g4142.t1	UniRef100_K03125	0.00E+00	1897.1
augustus	g4145.t1	UniRef100_K14617	9.50E-297	1023.8
augustus	g4146.t1	UniRef100_K14401	0.00E+00	2493.8
augustus	g4149.t1	UniRef100_K02219	1.50E-44	184.5
augustus	g4154.t1	UniRef100_K03574	1.30E-39	168.3
augustus	g4155.t1	UniRef100_K21249	1.20E-260	904
augustus	g4156.t1	UniRef100_K20408	0.00E+00	2221
augustus	g4157.t1	UniRef100_K12876	6.40E-57	225.3
augustus	g4158.t1	UniRef100_K01062	1.5e-311	1073.2
augustus	g4164.t1	UniRef100_K01739	5.10E-279	964.9
augustus	g4165.t1	UniRef100_K00273	4.10E-77	293.5
augustus	g4166.t1	UniRef100_K18171	1.20E-57	228
augustus	g4170.t1	UniRef100_K14769	2.50E-95	353.6
augustus	g4171.t1	UniRef100_K01876	0.00E+00	1167.1
augustus	g4172.t1	UniRef100_K20044	3.00E-214	750
augustus	g4173.t1	UniRef100_K10842	5.60E-170	602.1
augustus	g4177.t1	UniRef100_K00939	2.00E-111	407.1
augustus	g4179.t1	UniRef100_K17800	2.00E-232	810.1
augustus	g4180.t1	UniRef100_K17919	1.50E-244	850.1
augustus	g4181.t1	UniRef100_K01341	0.00E+00	1352.4
augustus	g4182.t1	UniRef100_K00053	2.60E-211	739.6
augustus	g4183.t1	UniRef100_K00698	0.00E+00	1780.4
augustus	g4184.t1	UniRef100_K15356	1.60E-184	650.6

augustus	g4185.t1	UniRef100_K19850	1.80E-162	577.4
augustus	g4186.t1	UniRef100_K07827	3.40E-128	462.6
augustus	g4188.t1	UniRef100_K01560	6.10E-110	402.1
augustus	g4190.t1	UniRef100_K18342	2.50E-94	350.5
augustus	g4192.t1	UniRef100_K00065	1.10E-46	192.2
augustus	g4197.t1	UniRef100_K00463	4.80E-265	918.3
augustus	g4202.t1	UniRef100_K18669	0.00E+00	1810
augustus	g4206.t1	UniRef100_K12194	2.10E-79	300.8
augustus	g4208.t1	UniRef100_K01247	1.00E-142	511.5
augustus	g4212.t1	UniRef100_K12840	6.00E-125	453
augustus	g4213.t1	UniRef100_K18655	4.50E-260	901.7
augustus	g4215.t1	UniRef100_K15109	6.50E-160	568.5
augustus	g4218.t1	UniRef100_K10845	2.20E-30	136
augustus	g4219.t1	UniRef100_K12663	4.30E-118	429.5
augustus	g4222.t1	UniRef100_K20463	0.00E+00	1500
augustus	g4225.t1	UniRef100_K01663	2.10E-303	1045.8
augustus	g4230.t1	UniRef100_K02866	3.30E-125	452.6
augustus	g4231.t1	UniRef100_K02908	3.90E-49	199.1
augustus	g4234.t1	UniRef100_K14191	7.30E-173	611.7
augustus	g4235.t1	UniRef100_K12855	1.60E-258	897.5
augustus	g4239.t1	UniRef100_K14803	3.50E-196	689.5
augustus	g4240.t1	UniRef100_K18637	6.90E-307	1058.1
augustus	g4249.t1	UniRef100_K01465	1.40E-170	604.4
augustus	g4250.t1	UniRef100_K01493	3.10E-165	586.3
augustus	g4251.t1	UniRef100_K03114	0.00E+00	1409
augustus	g4253.t1	UniRef100_K08257	1.60E-140	504.6
augustus	g4256.t1	UniRef100_K08337	0.00E+00	1293.5
augustus	g4266.t1	UniRef100_K20098	0.00E+00	1661.7
augustus	g4268.t1	UniRef100_K14560	2.40E-85	320.1
augustus	g4271.t1	UniRef100_K15303	2.30E-163	580.1
augustus	g4275.t1	UniRef100_K01749	8.50E-162	574.7
augustus	g4282.t1	UniRef100_K01066	9.10E-38	162.9
augustus	g4295.t1	UniRef100_K03549	2.70E-289	999.6
augustus	g4300.t1	UniRef100_K01436	9.40E-52	209.5
augustus	g4309.t1	UniRef100_K00872	2.90E-196	689.5
augustus	g4313.t1	UniRef100_K12659	1.60E-168	597.4
augustus	g4314.t1	UniRef100_K05528	2.30E-153	547
augustus	g4319.t1	UniRef100_K14298	2.00E-192	676.8
augustus	g4323.t1	UniRef100_K17906	0.00E+00	2936.7
augustus	g4324.t1	UniRef100_K00280	3.40E-102	377.5
augustus	g4325.t1	UniRef100_K15389	1.20E-69	269.2
augustus	g4326.t1	UniRef100_K15115	1.10E-200	704.1
augustus	g4330.t1	UniRef100_K03564	4.50E-72	276.6
augustus	g4333.t1	UniRef100_K01874	0.00E+00	1188.3
augustus	g4334.t1	UniRef100_K20791	1.30E-106	391
augustus	g4336.t1	UniRef100_K03754	2.50E-213	746.5
augustus	g4337.t1	UniRef100_K17617	3.30E-233	812.8
augustus	g4339.t1	UniRef100_K11129	5.50E-56	223
augustus	g4341.t1	UniRef100_K17080	1.40E-141	507.3
augustus	g4345.t1	UniRef100_K14394	1.70E-71	273.5

augustus	g4346.t1	UniRef100_K04464	1.20E-224	783.9
augustus	g4351.t1	UniRef100_K06889	2.20E-63	247.7
augustus	g4355.t1	UniRef100_K01426	3.50E-189	666.4
augustus	g4356.t1	UniRef100_K16261	3.00E-255	885.9
augustus	g4359.t1	UniRef100_K00001	1.30E-56	225.3
augustus	g4361.t1	UniRef100_K00101	2.60E-250	869.4
augustus	g4366.t1	UniRef100_K01265	2.00E-194	683.7
augustus	g4369.t1	UniRef100_K01279	1.70E-195	687.6
augustus	g4370.t1	UniRef100_K11153	2.00E-30	138.3
augustus	g4377.t1	UniRef100_K20121	5.60E-298	1028.5
augustus	g4386.t1	UniRef100_K05864	1.10E-179	634.4
augustus	g4388.t1	UniRef100_K12580	1.00E-207	728.4
augustus	g4392.t1	UniRef100_K20984	0.00E+00	1502.3
augustus	g4397.t1	UniRef100_K00059	2.30E-32	144.4
augustus	g4398.t1	UniRef100_K00031	9.10E-218	761.1
augustus	g4400.t1	UniRef100_K19998	0.00E+00	1262.7
augustus	g4414.t1	UniRef100_K20457	0.00E+00	1114.8
augustus	g4421.t1	UniRef100_K16575	1.70E-222	776.5
augustus	g4426.t1	UniRef100_K08257	3.10E-234	815.8
augustus	g4427.t1	UniRef100_K20366	5.70E-187	658.7
augustus	g4430.t1	UniRef100_K12813	0.00E+00	1574.7
augustus	g4431.t1	UniRef100_K14002	1.80E-210	736.9
augustus	g4439.t1	UniRef100_K01078	1.90E-51	208.8
augustus	g4442.t1	UniRef100_K08141	1.50E-275	953.4
augustus	g4443.t1	UniRef100_K15923	5.50E-191	672.9
augustus	g4450.t1	UniRef100_K03963	2.80E-40	169.1
augustus	g4454.t1	UniRef100_K00344	4.90E-80	303.1
augustus	g4455.t1	UniRef100_K08176	1.20E-47	196.1
augustus	g4457.t1	UniRef100_K03782	0.00E+00	1353.2
augustus	g4461.t1	UniRef100_K07407	1.10E-135	488.4
augustus	g4472.t1	UniRef100_K01253	1.00E-97	361.7
augustus	g4474.t1	UniRef100_K00273	3.30E-58	231.1
augustus	g4475.t1	UniRef100_K00681	0.00E+00	1136.7
augustus	g4476.t1	UniRef100_K07390	1.70E-75	287
augustus	g4477.t1	UniRef100_K01598	3.40E-103	379.8
augustus	g4479.t1	UniRef100_K00826	1.60E-216	756.9
augustus	g4480.t1	UniRef100_K00967	3.50E-234	815.8
augustus	g4484.t1	UniRef100_K08192	2.90E-189	666.8
augustus	g4485.t1	UniRef100_K01181	1.00E-108	397.9
augustus	g4487.t1	UniRef100_K11364	3.60E-158	562.8
augustus	g4489.t1	UniRef100_K07941	6.10E-97	358.6
augustus	g4490.t1	UniRef100_K02208	3.90E-216	755.7
augustus	g4491.t1	UniRef100_K17260	2.80E-220	769.2
augustus	g4494.t1	UniRef100_K00999	5.60E-89	332.8
augustus	g4502.t1	UniRef100_K02974	5.80E-44	182.6
augustus	g4503.t1	UniRef100_K08137	5.30E-32	144.1
augustus	g4504.t1	UniRef100_K00799	8.20E-98	361.7
augustus	g4506.t1	UniRef100_K02835	4.60E-205	718.8
augustus	g4508.t1	UniRef100_K08139	3.40E-58	231.1
augustus	g4516.t1	UniRef100_K01130	0.00E+00	1174.1

augustus	g4519.t1	UniRef100_K00001	1.40E-56	225.3
augustus	g4522.t1	UniRef100_K01497	1.90E-187	660.2
augustus	g4526.t1	UniRef100_K01809	2.80E-227	792.7
augustus	g4527.t1	UniRef100_K01876	1.20E-265	920.6
augustus	g4531.t1	UniRef100_K01502	7.20E-54	216.1
augustus	g4544.t1	UniRef100_K17741	1.90E-47	194.9
augustus	g4550.t1	UniRef100_K15924	2.20E-78	298.1
augustus	g4555.t1	UniRef100_K08192	1.30E-71	275.8
augustus	g4558.t1	UniRef100_K10534	0.00E+00	1454.1
augustus	g4559.t1	UniRef100_K17877	0.00E+00	1918.7
augustus	g4560.t1	UniRef100_K02575	2.50E-220	770
augustus	g4561.t1	UniRef100_K12256	4.20E-59	234.2
augustus	g4571.t1	UniRef100_K10675	1.60E-137	494.2
augustus	g4578.t1	UniRef100_K00001	8.40E-47	192.6
augustus	g4580.t1	UniRef100_K13955	6.60E-37	159.8
augustus	g4589.t1	UniRef100_K01050	8.50E-55	219.9
augustus	g4593.t1	UniRef100_K01426	3.70E-135	487.3
augustus	g4597.t1	UniRef100_K22134	1.20E-106	392.1
augustus	g4601.t1	UniRef100_K08157	4.10E-93	347.1
augustus	g4613.t1	UniRef100_K17362	2.20E-60	236.9
augustus	g4625.t1	UniRef100_K00863	4.40E-217	759.2
augustus	g4626.t1	UniRef100_K03441	1.30E-150	537.7
augustus	g4631.t1	UniRef100_K03781	0.00E+00	1290.4
augustus	g4633.t1	UniRef100_K00354	2.00E-143	513.8
augustus	g4636.t1	UniRef100_K13015	7.50E-47	193.4
augustus	g4639.t1	UniRef100_K01264	4.20E-142	510
augustus	g4640.t1	UniRef100_K01697	6.60E-270	934.5
augustus	g4642.t1	UniRef100_K20032	4.60E-265	919.1
augustus	g4647.t1	UniRef100_K04709	2.00E-171	607.1
augustus	g4648.t1	UniRef100_K04709	4.00E-127	460.3
augustus	g4652.t1	UniRef100_K16261	7.90E-278	961.1
augustus	g4654.t1	UniRef100_K22318	7.80E-66	255.8
augustus	g4657.t1	UniRef100_K08158	1.80E-191	674.1
augustus	g4663.t1	UniRef100_K13704	3.00E-190	669.5
augustus	g4666.t1	UniRef100_K07750	6.40E-48	196.1
augustus	g4668.t1	UniRef100_K08330	0.00E+00	1399
augustus	g4674.t1	UniRef100_K01835	5.80E-302	1041.2
augustus	g4681.t1	UniRef100_K02958	1.70E-67	260.8
augustus	g4686.t1	UniRef100_K06100	0.00E+00	1215.7
augustus	g4692.t1	UniRef100_K13237	2.60E-135	486.9
augustus	g4693.t1	UniRef100_K11230	0.00E+00	2305.4
augustus	g4698.t1	UniRef100_K14963	2.40E-107	394.4
augustus	g4700.t1	UniRef100_K00295	3.30E-172	609.4
augustus	g4702.t1	UniRef100_K06883	3.30E-157	559.3
augustus	g4703.t1	UniRef100_K00789	1.50E-213	746.9
augustus	g4706.t1	UniRef100_K01771	6.80E-245	851.3
augustus	g4709.t1	UniRef100_K21768	2.20E-259	899.8
augustus	g4710.t1	UniRef100_K09702	3.90E-97	359.4
augustus	g4711.t1	UniRef100_K05275	1.60E-156	557.4
augustus	g4712.t1	UniRef100_K10415	0.00E+00	1277.3

augustus	g4713.t1	UniRef100_K01549	2.20E-31	140.2
augustus	g4714.t1	UniRef100_K07151	0.00E+00	1441
augustus	g4717.t1	UniRef100_K12609	0.00E+00	1293.1
augustus	g4723.t1	UniRef100_K11390	2.90E-145	519.6
augustus	g4726.t1	UniRef100_K07232	2.80E-43	180.3
augustus	g4731.t1	UniRef100_K01512	1.80E-11	73.6
augustus	g4732.t1	UniRef100_K12815	0.00E+00	1702.6
augustus	g4733.t1	UniRef100_K14791	1.00E-209	734.6
augustus	g4738.t1	UniRef100_K01760	1.30E-119	434.9
augustus	g4742.t1	UniRef100_K12625	1.40E-31	140.6
augustus	g4747.t1	UniRef100_K11786	0.00E+00	2162.1
augustus	g4749.t1	UniRef100_K10703	5.20E-94	349
augustus	g4755.t1	UniRef100_K07374	2.70E-257	892.5
augustus	g4756.t1	UniRef100_K01640	1.10E-181	641
augustus	g4757.t1	UniRef100_K02147	2.30E-281	972.6
augustus	g4758.t1	UniRef100_K01785	2.40E-217	759.6
augustus	g4759.t1	UniRef100_K07561	3.30E-223	779.2
augustus	g4760.t1	UniRef100_K11270	4.20E-55	219.2
augustus	g4761.t1	UniRef100_K02991	2.70E-98	363.6
augustus	g4762.t1	UniRef100_K10592	0.00E+00	6069.2
augustus	g4764.t1	UniRef100_K02995	6.20E-95	352.1
augustus	g4766.t1	UniRef100_K10258	1.90E-146	523.9
augustus	g4769.t1	UniRef100_K10901	3.70E-222	775.8
augustus	g4774.t1	UniRef100_K01426	2.90E-134	484.2
augustus	g4790.t1	UniRef100_K00143	0.00E+00	2081.2
augustus	g4791.t1	UniRef100_K22073	5.20E-179	632.1
augustus	g4793.t1	UniRef100_K00354	3.60E-98	363.6
augustus	g4794.t1	UniRef100_K19564	7.50E-94	349.7
augustus	g4796.t1	UniRef100_K01814	2.00E-120	437.2
augustus	g4798.t1	UniRef100_K13886	4.60E-235	819.3
augustus	g4800.t1	UniRef100_K11215	0.00E+00	1115.5
augustus	g4803.t1	UniRef100_K07511	8.40E-151	538.1
augustus	g4806.t1	UniRef100_K01738	2.20E-203	713
augustus	g4811.t1	UniRef100_K21618	0.00E+00	1264.2
augustus	g4812.t1	UniRef100_K02731	1.20E-133	481.1
augustus	g4814.t1	UniRef100_K10843	0.00E+00	1521.1
augustus	g4818.t1	UniRef100_K00666	9.90E-116	422.5
augustus	g4820.t1	UniRef100_K01188	5.00E-261	904.8
augustus	g4826.t1	UniRef100_K14347	1.10E-213	747.7
augustus	g4832.t1	UniRef100_K21456	1.70E-261	906.7
augustus	g4834.t1	UniRef100_K00643	4.80E-286	988.4
augustus	g4836.t1	UniRef100_K14154	9.50E-203	711.4
augustus	g4837.t1	UniRef100_K14550	0.00E+00	3016.5
augustus	g4841.t1	UniRef100_K14572	0.00E+00	6063.4
augustus	g4842.t1	UniRef100_K02935	1.80E-36	157.5
augustus	g4843.t1	UniRef100_K15565	8.80E-127	458
augustus	g4845.t1	UniRef100_K00286	8.20E-169	598.2
augustus	g4846.t1	UniRef100_K11303	3.30E-226	789.3
augustus	g4850.t1	UniRef100_K10082	2.70E-148	530
augustus	g4851.t1	UniRef100_K17781	6.30E-32	141.7

augustus	g4854.t1	UniRef100_K12830	0.00E+00	2292.3
augustus	g4855.t1	UniRef100_K17656	0.00E+00	1548.5
augustus	g4857.t1	UniRef100_K06944	2.30E-188	663.3
augustus	g4860.t1	UniRef100_K12345	1.30E-136	491.1
augustus	g4862.t1	UniRef100_K03254	0.00E+00	1328.9
augustus	g4864.t1	UniRef100_K07973	1.10E-47	193.7
augustus	g4866.t1	UniRef100_K06269	2.40E-173	613.2
augustus	g4868.t1	UniRef100_K11380	0.00E+00	1797.7
augustus	g4874.t1	UniRef100_K03929	4.30E-44	184.5
augustus	g4876.t1	UniRef100_K00627	7.10E-67	259.6
augustus	g4879.t1	UniRef100_K09458	9.00E-213	744.6
augustus	g4880.t1	UniRef100_K17662	1.10E-137	494.6
augustus	g4883.t1	UniRef100_K13775	7.00E-37	159.8
augustus	g4900.t1	UniRef100_K09539	1.30E-42	177.6
augustus	g4902.t1	UniRef100_K10885	0.00E+00	1129.8
augustus	g4905.t1	UniRef100_K01101	1.30E-167	594
augustus	g4907.t1	UniRef100_K03165	0.00E+00	1106.7
augustus	g4911.t1	UniRef100_K13953	2.50E-84	317.4
augustus	g4912.t1	UniRef100_K03940	9.60E-110	401.4
augustus	g4913.t1	UniRef100_K10591	0.00E+00	1483.8
augustus	g4914.t1	UniRef100_K14396	2.00E-54	217.2
augustus	g4917.t1	UniRef100_K11654	0.00E+00	1939.9
augustus	g4918.t1	UniRef100_K00231	1.60E-273	946.8
augustus	g4919.t1	UniRef100_K15156	0.00E+00	1488.8
augustus	g4920.t1	UniRef100_K00036	2.60E-275	952.6
augustus	g4921.t1	UniRef100_K02917	3.10E-51	206.1
augustus	g4924.t1	UniRef100_K14439	0.00E+00	1457.6
augustus	g4928.t1	UniRef100_K00698	0.00E+00	2256.9
augustus	g4934.t1	UniRef100_K05853	0.00E+00	1762.7
augustus	g4935.t1	UniRef100_K02735	6.30E-115	418.3
augustus	g4936.t1	UniRef100_K15627	7.50E-200	701.8
augustus	g4937.t1	UniRef100_K02920	6.80E-36	155.6
augustus	g4939.t1	UniRef100_K00615	0.00E+00	1300.8
augustus	g4940.t1	UniRef100_K19308	1.20E-127	461.5
augustus	g4944.t1	UniRef100_K17987	0.00E+00	1080.9
augustus	g4946.t1	UniRef100_K10389	6.80E-264	914.4
augustus	g4949.t1	UniRef100_K11204	0.00E+00	1270.8
augustus	g4952.t1	UniRef100_K05349	0.00E+00	1252.3
augustus	g4953.t1	UniRef100_K04640	2.10E-186	656.8
augustus	g4954.t1	UniRef100_K00797	3.30E-138	496.5
augustus	g4955.t1	UniRef100_K13566	5.10E-154	548.9
augustus	g4957.t1	UniRef100_K00477	5.30E-49	199.9
augustus	g4959.t1	UniRef100_K02730	6.50E-138	495
augustus	g4960.t1	UniRef100_K07375	7.20E-238	827.8
augustus	g4964.t1	UniRef100_K08266	9.00E-175	617.8
augustus	g4971.t1	UniRef100_K14545	4.90E-135	485.7
augustus	g4975.t1	UniRef100_K08851	5.30E-127	458.8
augustus	g4976.t1	UniRef100_K11205	1.70E-148	530.4
augustus	g4977.t1	UniRef100_K12394	9.10E-213	744.6
augustus	g4981.t1	UniRef100_K04487	2.10E-265	919.5

augustus	g4982.t1	UniRef100_K12562	2.00E-210	736.9
augustus	g4984.t1	UniRef100_K01262	3.60E-305	1052.7
augustus	g4987.t1	UniRef100_K07127	5.70E-64	248.4
augustus	g4988.t1	UniRef100_K01551	4.20E-29	134
augustus	g4989.t1	UniRef100_K15541	8.70E-185	652.1
augustus	g4990.t1	UniRef100_K15428	5.20E-271	938.3
augustus	g4992.t1	UniRef100_K01194	0.00E+00	1369
augustus	g4993.t1	UniRef100_K01637	7.30E-284	981.1
augustus	g4994.t1	UniRef100_K12761	0.00E+00	1196.8
augustus	g4998.t1	UniRef100_K02325	0.00E+00	1414.1
augustus	g5001.t1	UniRef100_K11226	2.60E-243	846.3
augustus	g5003.t1	UniRef100_K11292	0.00E+00	2474.1
augustus	g5006.t1	UniRef100_K12861	1.80E-58	231.1
augustus	g5007.t1	UniRef100_K04739	4.70E-214	748.8
augustus	g5008.t1	UniRef100_K11362	4.70E-232	808.5
augustus	g5009.t1	UniRef100_K16570	0.00E+00	1492.2
augustus	g5017.t1	UniRef100_K00288	0.00E+00	1720.3
augustus	g5018.t1	UniRef100_K03246	1.00E-180	637.9
augustus	g5020.t1	UniRef100_K12373	2.80E-307	1058.9
augustus	g5024.t1	UniRef100_K14301	0.00E+00	1520.4
augustus	g5027.t1	UniRef100_K18183	5.20E-21	105.9
augustus	g5028.t1	UniRef100_K17065	0.00E+00	1338.9
augustus	g5031.t1	UniRef100_K20520	0.00E+00	1077
augustus	g5034.t1	UniRef100_K03251	1.90E-268	929.9
augustus	g5036.t1	UniRef100_K13513	9.90E-81	305.4
augustus	g5038.t1	UniRef100_K12767	0.00E+00	2993.8
augustus	g5049.t1	UniRef100_K00894	1.90E-209	733.4
augustus	g5050.t1	UniRef100_K20406	0.00E+00	1157.1
augustus	g5053.t1	UniRef100_K18757	4.70E-163	580.1
augustus	g5056.t1	UniRef100_K12869	4.50E-274	948.7
augustus	g5057.t1	UniRef100_K12844	5.30E-271	938.3
augustus	g5059.t1	UniRef100_K00925	1.00E-202	711.1
augustus	g5061.t1	UniRef100_K08176	5.60E-269	931.8
augustus	g5065.t1	UniRef100_K02886	1.80E-62	245
augustus	g5069.t1	UniRef100_K00838	1.60E-302	1043.1
augustus	g5076.t1	UniRef100_K02148	5.00E-190	669.1
augustus	g5077.t1	UniRef100_K00860	6.50E-144	516.5
augustus	g5078.t1	UniRef100_K14832	0.00E+00	1328.5
augustus	g5079.t1	UniRef100_K01881	7.10E-248	861.7
augustus	g5082.t1	UniRef100_K02603	0.00E+00	1234.9
augustus	g5083.t1	UniRef100_K12862	2.20E-79	301.6
augustus	g5084.t1	UniRef100_K06694	2.60E-66	257.3
augustus	g5085.t1	UniRef100_K20286	2.80E-164	584.3
augustus	g5087.t1	UniRef100_K21543	6.80E-278	961.4
augustus	g5090.t1	UniRef100_K01887	0.00E+00	1117.4
augustus	g5091.t1	UniRef100_K03245	4.50E-66	256.5
augustus	g5092.t1	UniRef100_K17979	1.30E-300	1036.9
augustus	g5093.t1	UniRef100_K11884	3.50E-127	459.5
augustus	g5094.t1	UniRef100_K15325	8.30E-63	244.6
augustus	g5095.t1	UniRef100_K01689	2.30E-230	803.1

augustus	g5096.t1	UniRef100_K02178	0.00E+00	1742.2
augustus	g5103.t1	UniRef100_K01736	2.10E-216	756.5
augustus	g5104.t1	UniRef100_K10771	1.50E-166	590.9
augustus	g5105.t1	UniRef100_K14457	7.70E-210	734.6
augustus	g5108.t1	UniRef100_K01238	2.20E-147	527.3
augustus	g5109.t1	UniRef100_K11483	2.70E-276	955.7
augustus	g5110.t1	UniRef100_K11319	9.50E-209	731.1
augustus	g5111.t1	UniRef100_K18803	3.00E-155	553.1
augustus	g5113.t1	UniRef100_K14781	0.00E+00	1279.6
augustus	g5114.t1	UniRef100_K19950	4.20E-99	366.7
augustus	g5117.t1	UniRef100_K01599	1.40E-186	657.5
augustus	g5122.t1	UniRef100_K16489	6.90E-107	394
augustus	g5124.t1	UniRef100_K11436	7.90E-250	867.8
augustus	g5125.t1	UniRef100_K20361	0.00E+00	1303.9
augustus	g5126.t1	UniRef100_K12619	0.00E+00	1444.9
augustus	g5127.t1	UniRef100_K09548	4.50E-49	198.7
augustus	g5128.t1	UniRef100_K04354	3.00E-252	875.9
augustus	g5129.t1	UniRef100_K02973	0.00E+00	1163.7
augustus	g5131.t1	UniRef100_K06125	8.90E-185	651.4
augustus	g5132.t1	UniRef100_K02973	2.40E-76	289.7
augustus	g5134.t1	UniRef100_K00939	3.00E-130	469.9
augustus	g5135.t1	UniRef100_K16536	1.10E-295	1020.8
augustus	g5137.t1	UniRef100_K01262	0.00E+00	1108.6
augustus	g5141.t1	UniRef100_K01867	1.00E-162	578.2
augustus	g5143.t1	UniRef100_K13989	1.80E-130	470.3
augustus	g5147.t1	UniRef100_K02905	3.50E-113	413.3
augustus	g5149.t1	UniRef100_K03255	0.00E+00	1915.2
augustus	g5151.t1	UniRef100_K14376	2.50E-253	879.8
augustus	g5154.t1	UniRef100_K06655	3.10E-111	406.8
augustus	g5155.t1	UniRef100_K13511	5.10E-184	648.7
augustus	g5156.t1	UniRef100_K13125	1.10E-144	518.1
augustus	g5157.t1	UniRef100_K11365	3.60E-254	882.9
augustus	g5159.t1	UniRef100_K03099	0.00E+00	2117.4
augustus	g5165.t1	UniRef100_K10598	1.60E-267	926.8
augustus	g5167.t1	UniRef100_K18164	1.20E-51	209.5
augustus	g5174.t1	UniRef100_K22134	1.80E-79	302
augustus	g5180.t1	UniRef100_K08818	2.20E-184	650.2
augustus	g5181.t1	UniRef100_K07734	9.90E-105	384.8
augustus	g5182.t1	UniRef100_K18740	3.20E-54	217.2
augustus	g5187.t1	UniRef100_K00059	5.90E-70	269.2
augustus	g5188.t1	UniRef100_K13333	0.00E+00	1097.8
augustus	g5194.t1	UniRef100_K21295	1.40E-156	558.1
augustus	g5198.t1	UniRef100_K07393	9.30E-185	651
augustus	g5199.t1	UniRef100_K00433	6.00E-15	86.7
augustus	g5202.t1	UniRef100_K01565	2.70E-177	626.7
augustus	g5204.t1	UniRef100_K01279	1.60E-218	764.2
augustus	g5213.t1	UniRef100_K01181	7.50E-102	375.2
augustus	g5214.t1	UniRef100_K01184	1.30E-117	428.3
augustus	g5217.t1	UniRef100_K18123	1.40E-147	527.7
augustus	g5219.t1	UniRef100_K06035	4.90E-120	435.6

augustus	g5220.t1	UniRef100_K00217	1.80E-83	314.7
augustus	g5222.t1	UniRef100_K03417	9.40E-94	348.6
augustus	g5225.t1	UniRef100_K10256	2.20E-91	341.3
augustus	g5229.t1	UniRef100_K00002	6.00E-44	183.3
augustus	g5237.t1	UniRef100_K11161	5.00E-21	107.1
augustus	g5245.t1	UniRef100_K06035	2.10E-101	373.6
augustus	g5255.t1	UniRef100_K18660	7.40E-183	645.6
augustus	g5259.t1	UniRef100_K12613	2.60E-199	700.3
augustus	g5261.t1	UniRef100_K10808	1.10E-217	760.8
augustus	g5271.t1	UniRef100_K01044	1.10E-167	595.1
augustus	g5274.t1	UniRef100_K15117	1.60E-165	587
augustus	g5275.t1	UniRef100_K14169	1.20E-160	571.2
augustus	g5278.t1	UniRef100_K13201	1.10E-232	810.8
augustus	g5279.t1	UniRef100_K18670	0.00E+00	1167.1
augustus	g5282.t1	UniRef100_K03260	0.00E+00	1558.9
augustus	g5283.t1	UniRef100_K12874	0.00E+00	2587.8
augustus	g5284.t1	UniRef100_K12878	0.00E+00	1231.1
augustus	g5285.t1	UniRef100_K03262	5.50E-188	662.1
augustus	g5289.t1	UniRef100_K03264	3.50E-126	456.1
augustus	g5291.t1	UniRef100_K03456	4.60E-224	782.7
augustus	g5293.t1	UniRef100_K02985	5.80E-137	491.9
augustus	g5296.t1	UniRef100_K20471	7.10E-233	811.6
augustus	g5297.t1	UniRef100_K00480	3.20E-32	144.4
augustus	g5311.t1	UniRef100_K17644	3.30E-86	323.2
augustus	g5312.t1	UniRef100_K22211	9.10E-89	331.6
augustus	g5314.t1	UniRef100_K22121	2.40E-171	606.7
augustus	g5315.t1	UniRef100_K21294	9.40E-207	724.5
augustus	g5316.t1	UniRef100_K22118	0.00E+00	3018
augustus	g5317.t1	UniRef100_K01273	3.40E-153	546.6
augustus	g5318.t1	UniRef100_K22119	3.90E-96	356.3
augustus	g5320.t1	UniRef100_K05658	0.00E+00	1798.9
augustus	g5322.t1	UniRef100_K21293	2.00E-71	275
augustus	g5323.t1	UniRef100_K22124	2.50E-123	447.2
augustus	g5329.t1	UniRef100_K00101	8.10E-120	435.6
augustus	g5330.t1	UniRef100_K11153	2.20E-27	128.3
augustus	g5335.t1	UniRef100_K00423	9.60E-106	389.4
augustus	g5336.t1	UniRef100_K11162	2.50E-25	121.3
augustus	g5344.t1	UniRef100_K15418	4.80E-275	953.7
augustus	g5347.t1	UniRef100_K20737	3.20E-94	350.5
augustus	g5352.t1	UniRef100_K08158	6.70E-242	841.6
augustus	g5356.t1	UniRef100_K03351	4.4e-310	1068.5
augustus	g5357.t1	UniRef100_K16186	2.80E-192	676.4
augustus	g5358.t1	UniRef100_K14400	2.00E-238	830.5
augustus	g5370.t1	UniRef100_K07297	3.50E-120	436.4
augustus	g5372.t1	UniRef100_K01011	2.50E-113	413.7
augustus	g5376.t1	UniRef100_K01253	1.80E-126	457.6
augustus	g5379.t1	UniRef100_K00799	3.20E-69	266.5
augustus	g5394.t1	UniRef100_K20656	1.20E-66	258.5
augustus	g5395.t1	UniRef100_K00803	1.80E-171	607.8
augustus	g5397.t1	UniRef100_K06210	3.60E-139	499.6

augustus	g5399.t1	UniRef100_K14997	1.10E-247	860.9
augustus	g5400.t1	UniRef100_K04513	1.10E-94	351.3
augustus	g5401.t1	UniRef100_K02895	3.10E-184	649.4
augustus	g5402.t1	UniRef100_K03189	8.10E-129	464.9
augustus	g5406.t1	UniRef100_K05972	1.30E-108	397.5
augustus	g5410.t1	UniRef100_K11153	1.80E-27	128.6
augustus	g5414.t1	UniRef100_K13953	2.50E-95	354
augustus	g5417.t1	UniRef100_K22213	8.40E-98	362.1
augustus	g5418.t1	UniRef100_K00450	2.10E-145	520.4
augustus	g5419.t1	UniRef100_K00480	1.60E-155	554.7
augustus	g5420.t1	UniRef100_K21802	6.20E-89	333.2
augustus	g5421.t1	UniRef100_K01426	1.30E-203	714.1
augustus	g5427.t1	UniRef100_K07393	2.20E-135	486.9
augustus	g5434.t1	UniRef100_K18386	2.60E-49	201.4
augustus	g5443.t1	UniRef100_K14799	0.00E+00	1384.4
augustus	g5444.t1	UniRef100_K14768	9.20E-289	997.3
augustus	g5445.t1	UniRef100_K04794	7.50E-94	348.6
augustus	g5446.t1	UniRef100_K10524	1.40E-99	367.9
augustus	g5449.t1	UniRef100_K02726	4.60E-138	495.7
augustus	g5451.t1	UniRef100_K05532	2.80E-155	553.1
augustus	g5452.t1	UniRef100_K01634	2.30E-301	1039.3
augustus	g5455.t1	UniRef100_K00898	2.10E-206	723.8
augustus	g5457.t1	UniRef100_K12765	0.00E+00	1320.4
augustus	g5461.t1	UniRef100_K16261	2.00E-272	943
augustus	g5466.t1	UniRef100_K14546	1.90E-99	367.9
augustus	g5469.t1	UniRef100_K18372	6.80E-43	179.9
augustus	g5470.t1	UniRef100_K01179	2.80E-182	643.3
augustus	g5471.t1	UniRef100_K01167	1.10E-23	114.8
augustus	g5474.t1	UniRef100_K03232	9.80E-93	344.7
augustus	g5477.t1	UniRef100_K00108	3.00E-48	198.4
augustus	g5478.t1	UniRef100_K17475	1.10E-69	269.6
augustus	g5485.t1	UniRef100_K20743	2.80E-30	137.5
augustus	g5486.t1	UniRef100_K13690	6.30E-188	661.8
augustus	g5490.t1	UniRef100_K11153	4.40E-41	173.7
augustus	g5493.t1	UniRef100_K04564	1.10E-99	368.2
augustus	g5497.t1	UniRef100_K01181	1.70E-95	354
augustus	g5508.t1	UniRef100_K21159	6.70E-55	219.9
augustus	g5509.t1	UniRef100_K11150	5.20E-22	110.2
augustus	g5515.t1	UniRef100_K10088	5.30E-190	669.1
augustus	g5518.t1	UniRef100_K10734	2.60E-89	333.2
augustus	g5524.t1	UniRef100_K22134	7.60E-224	781.6
augustus	g5526.t1	UniRef100_K01886	0.00E+00	1111.3
augustus	g5531.t1	UniRef100_K21293	8.30E-84	316.2
augustus	g5532.t1	UniRef100_K06123	1.50E-135	487.3
augustus	g5534.t1	UniRef100_K19548	9.50E-22	109
augustus	g5535.t1	UniRef100_K15419	1.40E-274	952.2
augustus	g5539.t1	UniRef100_K00001	1.10E-30	139.4
augustus	g5550.t1	UniRef100_K05658	2.50E-270	937.2
augustus	g5552.t1	UniRef100_K13076	5.00E-264	915.2
augustus	g5556.t1	UniRef100_K10675	6.00E-75	286.2

augustus	g5560.t1	UniRef100_K00838	2.90E-173	613.6
augustus	g5570.t1	UniRef100_K05986	1.60E-68	265
augustus	g5575.t1	UniRef100_K01915	1.10E-192	677.6
augustus	g5587.t1	UniRef100_K07824	2.80E-276	955.7
augustus	g5589.t1	UniRef100_K00652	1.30E-175	620.9
augustus	g5590.t1	UniRef100_K01012	3.00E-185	652.9
augustus	g5594.t1	UniRef100_K11153	6.50E-53	213
augustus	g5597.t1	UniRef100_K01952	0.00E+00	2516.5
augustus	g5599.t1	UniRef100_K20858	1.10E-194	684.5
augustus	g5602.t1	UniRef100_K01597	8.40E-201	704.5
augustus	g5604.t1	UniRef100_K01764	5.20E-105	386
augustus	g5607.t1	UniRef100_K00784	1.40E-161	573.9
augustus	g5611.t1	UniRef100_K08158	1.40E-70	272.3
augustus	g5612.t1	UniRef100_K01507	1.10E-152	544.7
augustus	g5614.t1	UniRef100_K00059	2.70E-30	137.5
augustus	g5617.t1	UniRef100_K08073	7.60E-225	784.6
augustus	g5618.t1	UniRef100_K15136	3.60E-145	519.2
augustus	g5632.t1	UniRef100_K01185	1.40E-135	487.3
augustus	g5636.t1	UniRef100_K01655	3.10E-164	583.2
augustus	g5637.t1	UniRef100_K21159	1.70E-50	205.3
augustus	g5639.t1	UniRef100_K06892	2.40E-24	118.2
augustus	g5641.t1	UniRef100_K12198	5.80E-87	325.5
augustus	g5642.t1	UniRef100_K03362	0.00E+00	1463
augustus	g5643.t1	UniRef100_K11236	0.00E+00	1509.6
augustus	g5648.t1	UniRef100_K14819	1.40E-163	581.3
augustus	g5657.t1	UniRef100_K00616	6.20E-148	529.3
augustus	g5658.t1	UniRef100_K00059	9.10E-18	96.3
augustus	g5663.t1	UniRef100_K13103	0.00E+00	1091.6
augustus	g5664.t1	UniRef100_K16803	0.00E+00	1280.4
augustus	g5666.t1	UniRef100_K19525	0.00E+00	5523
augustus	g5667.t1	UniRef100_K03317	6.70E-294	1014.6
augustus	g5671.t1	UniRef100_K15785	1.10E-61	242.7
augustus	g5675.t1	UniRef100_K14708	0.00E+00	1303.9
augustus	g5679.t1	UniRef100_K01800	6.10E-79	298.9
augustus	g5681.t1	UniRef100_K01266	1.30E-199	700.7
augustus	g5685.t1	UniRef100_K10675	1.10E-194	684.1
augustus	g5689.t1	UniRef100_K01502	2.60E-70	270.8
augustus	g5694.t1	UniRef100_K20984	0.00E+00	1444.1
augustus	g5696.t1	UniRef100_K22134	1.00E-53	216.5
augustus	g5697.t1	UniRef100_K20550	1.30E-50	206.1
augustus	g5699.t1	UniRef100_K03885	5.90E-120	436
augustus	g5704.t1	UniRef100_K01624	1.10E-136	491.1
augustus	g5705.t1	UniRef100_K22025	4.20E-57	226.9
augustus	g5708.t1	UniRef100_K00015	2.30E-120	437.2
augustus	g5710.t1	UniRef100_K14263	5.80E-23	113.2
augustus	g5713.t1	UniRef100_K00463	1.40E-139	501.5
augustus	g5715.t1	UniRef100_K15388	2.10E-140	504.2
augustus	g5716.t1	UniRef100_K00493	8.20E-68	263.1
augustus	g5717.t1	UniRef100_K05665	1.10E-146	526.6
augustus	g5721.t1	UniRef100_K18107	3.30E-62	243.8

augustus	g5723.t1	UniRef100_K15303	9.80E-141	505
augustus	g5726.t1	UniRef100_K00344	4.50E-35	153.7
augustus	g5731.t1	UniRef100_K00129	4.00E-257	892.1
augustus	g5732.t1	UniRef100_K01213	4.60E-158	562.8
augustus	g5735.t1	UniRef100_K01379	2.10E-29	135.2
augustus	g5736.t1	UniRef100_K22277	8.90E-239	830.9
augustus	g5738.t1	UniRef100_K08141	9.80E-273	944.1
augustus	g5743.t1	UniRef100_K00354	1.90E-141	507.3
augustus	g5744.t1	UniRef100_K01426	6.80E-222	775
augustus	g5747.t1	UniRef100_K08157	1.20E-79	302.4
augustus	g5749.t1	UniRef100_K00108	2.00E-65	255.4
augustus	g5750.t1	UniRef100_K03380	9.70E-183	645.6
augustus	g5753.t1	UniRef100_K12859	1.90E-175	621.7
augustus	g5758.t1	UniRef100_K03929	9.70E-123	445.7
augustus	g5759.t1	UniRef100_K22213	4.60E-145	519.2
augustus	g5761.t1	UniRef100_K00480	9.20E-116	422.2
augustus	g5764.t1	UniRef100_K00354	1.80E-136	490.7
augustus	g5765.t1	UniRef100_K22134	3.20E-185	653.3
augustus	g5773.t1	UniRef100_K18372	3.10E-45	188
augustus	g5774.t1	UniRef100_K00059	4.70E-37	160.2
augustus	g5775.t1	UniRef100_K00055	4.30E-58	230.3
augustus	g5776.t1	UniRef100_K21802	4.30E-91	340.5
augustus	g5778.t1	UniRef100_K00019	1.90E-55	221.5
augustus	g5780.t1	UniRef100_K10215	6.50E-126	456.1
augustus	g5781.t1	UniRef100_K22213	2.30E-154	550.1
augustus	g5789.t1	UniRef100_K05658	0.00E+00	1653.3
augustus	g5801.t1	UniRef100_K01907	1.50E-203	714.5
augustus	g5803.t1	UniRef100_K00135	4.10E-146	523.1
augustus	g5806.t1	UniRef100_K17741	5.30E-21	107.1
augustus	g5807.t1	UniRef100_K00306	9.80E-143	511.9
augustus	g5810.t1	UniRef100_K01750	1.30E-141	507.7
augustus	g5818.t1	UniRef100_K01192	2.60E-168	597.8
augustus	g5821.t1	UniRef100_K06889	1.30E-47	195.3
augustus	g5829.t1	UniRef100_K14541	0.00E+00	2224.5
augustus	g5833.t1	UniRef100_K05989	1.40E-227	794.7
augustus	g5834.t1	UniRef100_K05349	0.00E+00	1204.9
augustus	g5836.t1	UniRef100_K19356	2.10E-137	493.8
augustus	g5839.t1	UniRef100_K17645	5.90E-69	266.9
augustus	g5841.t1	UniRef100_K08157	6.80E-57	226.9
augustus	g5842.t1	UniRef100_K04940	5.10E-59	233.4
augustus	g5848.t1	UniRef100_K01537	0.00E+00	1516.9
augustus	g5850.t1	UniRef100_K01181	1.20E-77	295
augustus	g5853.t1	UniRef100_K10437	2.10E-164	583.9
augustus	g5854.t1	UniRef100_K00018	5.00E-96	356.3
augustus	g5856.t1	UniRef100_K00799	3.40E-72	276.6
augustus	g5857.t1	UniRef100_K01607	1.60E-50	204.1
augustus	g5858.t1	UniRef100_K13953	2.30E-107	394
augustus	g5860.t1	UniRef100_K01293	3.20E-255	885.9
augustus	g5861.t1	UniRef100_K20844	6.60E-251	871.3
augustus	g5863.t1	UniRef100_K00059	3.10E-29	134

augustus	g5867.t1	UniRef100_K17741	2.60E-65	254.2
augustus	g5870.t1	UniRef100_K13239	1.30E-67	261.5
augustus	g5872.t1	UniRef100_K01426	1.10E-161	575.1
augustus	g5876.t1	UniRef100_K00020	2.00E-66	257.7
augustus	g5877.t1	UniRef100_K00344	2.80E-48	197.6
augustus	g5881.t1	UniRef100_K03097	6.40E-85	318.9
augustus	g5883.t1	UniRef100_K17066	0.00E+00	1293.1
augustus	g5884.t1	UniRef100_K01183	2.70E-194	682.9
augustus	g5888.t1	UniRef100_K18540	5.50E-11	73.6
augustus	g5892.t1	UniRef100_K11022	8.50E-20	101.7
augustus	g5897.t1	UniRef100_K00128	2.10E-116	424.5
augustus	g5898.t1	UniRef100_K13953	1.20E-107	394.8
augustus	g5900.t1	UniRef100_K08139	1.20E-79	302.8
augustus	g5901.t1	UniRef100_K00129	3.10E-261	905.6
augustus	g5902.t1	UniRef100_K01426	2.30E-277	959.5
augustus	g5903.t1	UniRef100_K07393	2.40E-154	550.1
augustus	g5904.t1	UniRef100_K19791	3.5e-310	1068.5
augustus	g5907.t1	UniRef100_K03932	4.40E-48	196.8
augustus	g5910.t1	UniRef100_K02954	3.10E-39	166.4
augustus	g5914.t1	UniRef100_K18447	3.10E-56	223.8
augustus	g5917.t1	UniRef100_K12176	3.20E-214	749.6
augustus	g5918.t1	UniRef100_K09602	6.50E-116	423.3
augustus	g5920.t1	UniRef100_K01915	7.10E-94	349.7
augustus	g5921.t1	UniRef100_K00059	6.30E-31	139.8
augustus	g5922.t1	UniRef100_K01637	2.10E-304	1049.3
augustus	g5923.t1	UniRef100_K01647	1.20E-260	903.7
augustus	g5927.t1	UniRef100_K13953	3.70E-148	529.6
augustus	g5934.t1	UniRef100_K01829	3.80E-23	113.6
augustus	g5937.t1	UniRef100_K00059	1.40E-22	112.1
augustus	g5940.t1	UniRef100_K00091	2.60E-97	360.5
augustus	g5943.t1	UniRef100_K19572	4.30E-257	892.1
augustus	g5946.t1	UniRef100_K00799	2.80E-44	183.7
augustus	g5948.t1	UniRef100_K04097	1.40E-77	294.3
augustus	g5950.t1	UniRef100_K01501	1.60E-120	437.6
augustus	g5961.t1	UniRef100_K05349	0.00E+00	1576.6
augustus	g5962.t1	UniRef100_K01505	7.60E-164	581.6
augustus	g5965.t1	UniRef100_K03885	6.10E-112	409.5
augustus	g5971.t1	UniRef100_K05665	1.30E-127	463
augustus	g5976.t1	UniRef100_K00019	7.00E-26	122.9
augustus	g5978.t1	UniRef100_K05916	8.40E-207	724.5
augustus	g5979.t1	UniRef100_K01199	3.40E-168	596.3
augustus	g5993.t1	UniRef100_K01336	2.90E-262	909.4
augustus	g5994.t1	UniRef100_K22262	0.00E+00	4788.4
augustus	g5996.t1	UniRef100_K12670	8.00E-218	761.5
augustus	g5998.t1	UniRef100_K08139	4.60E-61	240.7
augustus	g5999.t1	UniRef100_K18102	9.40E-237	824.3
augustus	g6000.t1	UniRef100_K00799	1.20E-85	321.2
augustus	g6006.t1	UniRef100_K17724	1.70E-121	441.4
augustus	g6007.t1	UniRef100_K03417	2.50E-79	300.8
augustus	g6011.t1	UniRef100_K00641	2.30E-212	743.4

augustus	g6013.t1	UniRef100_K12870	1.40E-129	467.2
augustus	g6015.t1	UniRef100_K13566	1.00E-156	557.8
augustus	g6018.t1	UniRef100_K16261	1.30E-258	897.1
augustus	g6024.t1	UniRef100_K17740	4.40E-47	192.6
augustus	g6033.t1	UniRef100_K03119	1.70E-30	138.7
augustus	g6039.t1	UniRef100_K01253	4.80E-99	366.7
augustus	g6043.t1	UniRef100_K18549	3.90E-81	307
augustus	g6046.t1	UniRef100_K16261	3.40E-304	1048.5
augustus	g6047.t1	UniRef100_K18576	5.90E-96	355.5
augustus	g6050.t1	UniRef100_K04646	0.00E+00	3200.6
augustus	g6051.t1	UniRef100_K01282	0.00E+00	1644
augustus	g6054.t1	UniRef100_K01194	0.00E+00	1229.2
augustus	g6055.t1	UniRef100_K01620	4.10E-174	615.9
augustus	g6056.t1	UniRef100_K00737	1.40E-189	667.2
augustus	g6062.t1	UniRef100_K03002	0.00E+00	2363.6
augustus	g6063.t1	UniRef100_K00505	6.20E-239	832
augustus	g6067.t1	UniRef100_K17891	1.60E-69	267.7
augustus	g6075.t1	UniRef100_K03247	1.10E-174	617.8
augustus	g6076.t1	UniRef100_K01251	1.70E-240	836.6
augustus	g6077.t1	UniRef100_K00015	3.10E-172	609.4
augustus	g6084.t1	UniRef100_K14829	7.50E-202	708
augustus	g6087.t1	UniRef100_K12389	1.10E-142	511.5
augustus	g6092.t1	UniRef100_K01560	6.70E-50	202.6
augustus	g6095.t1	UniRef100_K15419	0.00E+00	1103.2
augustus	g6100.t1	UniRef100_K03022	9.10E-102	374.8
augustus	g6105.t1	UniRef100_K03848	5.60E-284	981.5
augustus	g6108.t1	UniRef100_K00235	1.50E-151	540.4
augustus	g6109.t1	UniRef100_K17508	4.90E-179	632.1
augustus	g6110.t1	UniRef100_K15111	3.70E-44	183.7
augustus	g6113.t1	UniRef100_K14830	2.70E-228	796.2
augustus	g6114.t1	UniRef100_K08853	0.00E+00	1312.7
augustus	g6118.t1	UniRef100_K05288	0.00E+00	1884
augustus	g6121.t1	UniRef100_K20238	2.10E-286	989.6
augustus	g6123.t1	UniRef100_K07407	4.00E-276	955.3
augustus	g6124.t1	UniRef100_K01684	4.40E-214	748.8
augustus	g6125.t1	UniRef100_K18339	1.90E-166	590.1
augustus	g6126.t1	UniRef100_K00863	3.30E-170	603.6
augustus	g6127.t1	UniRef100_K22322	4.60E-43	179.9
augustus	g6128.t1	UniRef100_K08141	2.90E-305	1052
augustus	g6131.t1	UniRef100_K03305	1.50E-118	432.2
augustus	g6135.t1	UniRef100_K03786	6.70E-69	265
augustus	g6136.t1	UniRef100_K00457	1.60E-166	590.5
augustus	g6137.t1	UniRef100_K09484	8.10E-162	574.7
augustus	g6138.t1	UniRef100_K08138	6.70E-39	167.2
augustus	g6140.t1	UniRef100_K13830	3.20E-88	331.6
augustus	g6142.t1	UniRef100_K20181	0.00E+00	1780.8
augustus	g6144.t1	UniRef100_K08192	7.80E-84	316.6
augustus	g6146.t1	UniRef100_K15339	9.80E-276	954.5
augustus	g6150.t1	UniRef100_K07478	5.60E-246	855.1
augustus	g6152.t1	UniRef100_K01078	8.60E-209	731.5

augustus	g6159.t1	UniRef100_K05012	0.00E+00	1359
augustus	g6161.t1	UniRef100_K00161	5.90E-214	748.4
augustus	g6164.t1	UniRef100_K09104	2.10E-139	500.4
augustus	g6168.t1	UniRef100_K05667	6.00E-298	1029.2
augustus	g6170.t1	UniRef100_K20403	0.00E+00	1601.3
augustus	g6172.t1	UniRef100_K17794	1.10E-110	404.4
augustus	g6174.t1	UniRef100_K00859	1.60E-142	510.4
augustus	g6179.t1	UniRef100_K00752	2.90E-304	1048.9
augustus	g6182.t1	UniRef100_K11228	0.00E+00	1430.6
augustus	g6183.t1	UniRef100_K04518	5.10E-163	578.9
augustus	g6186.t1	UniRef100_K03695	0.00E+00	1635.9
augustus	g6192.t1	UniRef100_K14685	2.40E-162	577
augustus	g6197.t1	UniRef100_K00472	1.30E-105	387.9
augustus	g6198.t1	UniRef100_K06910	9.50E-92	341.3
augustus	g6202.t1	UniRef100_K13339	0.00E+00	2271.1
augustus	g6204.t1	UniRef100_K01904	2.40E-92	344.7
augustus	g6205.t1	UniRef100_K07555	1.00E-144	518.1
augustus	g6206.t1	UniRef100_K15075	0.00E+00	1874.4
augustus	g6208.t1	UniRef100_K11991	1.30E-09	68.2
augustus	g6213.t1	UniRef100_K09788	1.30E-47	195.7
augustus	g6215.t1	UniRef100_K01824	2.50E-97	360.1
augustus	g6217.t1	UniRef100_K18719	0.00E+00	1452.2
augustus	g6218.t1	UniRef100_K12883	1.20E-79	301.2
augustus	g6220.t1	UniRef100_K20301	2.20E-86	323.2
augustus	g6221.t1	UniRef100_K11254	3.00E-49	199.1
augustus	g6222.t1	UniRef100_K03350	0.00E+00	1265.8
augustus	g6227.t1	UniRef100_K21752	1.70E-68	264.6
augustus	g6228.t1	UniRef100_K19801	0.00E+00	1728.8
augustus	g6230.t1	UniRef100_K07889	5.20E-113	412.1
augustus	g6231.t1	UniRef100_K14786	8.90E-182	642.1
augustus	g6236.t1	UniRef100_K06911	1.40E-151	540.8
augustus	g6237.t1	UniRef100_K01183	3.80E-132	478
augustus	g6238.t1	UniRef100_K00661	6.30E-28	129.4
augustus	g6239.t1	UniRef100_K20989	0.00E+00	1181.8
augustus	g6240.t1	UniRef100_K03801	1.80E-137	493.8
augustus	g6242.t1	UniRef100_K11227	2.70E-305	1052.4
augustus	g6245.t1	UniRef100_K01968	0.00E+00	1205.7
augustus	g6250.t1	UniRef100_K05609	8.10E-118	428.3
augustus	g6253.t1	UniRef100_K10352	0.00E+00	2753
augustus	g6255.t1	UniRef100_K01052	6.50E-261	904.8
augustus	g6256.t1	UniRef100_K18178	1.60E-66	256.9
augustus	g6259.t1	UniRef100_K15979	0.00E+00	1582.8
augustus	g6260.t1	UniRef100_K04565	3.30E-68	263.1
augustus	g6261.t1	UniRef100_K21295	2.30E-68	265
augustus	g6263.t1	UniRef100_K22139	2.50E-45	187.2
augustus	g6264.t1	UniRef100_K03946	1.80E-40	169.9
augustus	g6269.t1	UniRef100_K02875	8.90E-71	271.2
augustus	g6270.t1	UniRef100_K03626	5.60E-60	236.1
augustus	g6272.t1	UniRef100_K10580	2.10E-80	303.1
augustus	g6273.t1	UniRef100_K20409	0.00E+00	2491.8

augustus	g6280.t1	UniRef100_K03859	1.40E-219	767.3
augustus	g6283.t1	UniRef100_K15153	3.00E-46	189.5
augustus	g6284.t1	UniRef100_K03019	4.80E-61	238.4
augustus	g6288.t1	UniRef100_K00844	5.10E-289	999.2
augustus	g6289.t1	UniRef100_K20793	6.90E-91	339.3
augustus	g6292.t1	UniRef100_K09595	1.50E-249	867.1
augustus	g6293.t1	UniRef100_K06133	8.90E-116	421.8
augustus	g6300.t1	UniRef100_K01190	0.00E+00	1113.2
augustus	g6303.t1	UniRef100_K13431	0.00E+00	1146.3
augustus	g6310.t1	UniRef100_K15418	3.50E-292	1010.7
augustus	g6318.t1	UniRef100_K01372	5.40E-227	792
augustus	g6327.t1	UniRef100_K14758	2.60E-204	716.1
augustus	g6329.t1	UniRef100_K04345	8.00E-196	688
augustus	g6331.t1	UniRef100_K19951	0.00E+00	1944.5
augustus	g6334.t1	UniRef100_K00164	0.00E+00	2021.5
augustus	g6335.t1	UniRef100_K19842	0.00E+00	2149.4
augustus	g6336.t1	UniRef100_K02155	6.00E-61	239.2
augustus	g6342.t1	UniRef100_K02263	4.80E-60	236.1
augustus	g6343.t1	UniRef100_K03544	1.90E-268	929.9
augustus	g6345.t1	UniRef100_K04459	0.00E+00	1142.1
augustus	g6346.t1	UniRef100_K17908	5.10E-205	718.8
augustus	g6349.t1	UniRef100_K03141	9.90E-266	921
augustus	g6351.t1	UniRef100_K02607	0.00E+00	1197.6
augustus	g6353.t1	UniRef100_K17605	6.80E-171	605.1
augustus	g6355.t1	UniRef100_K03936	4.20E-149	532.3
augustus	g6356.t1	UniRef100_K10643	0.00E+00	1659
augustus	g6357.t1	UniRef100_K19983	0.00E+00	2207.2
augustus	g6358.t1	UniRef100_K03942	2.00E-274	949.5
augustus	g6359.t1	UniRef100_K17738	2.50E-44	184.9
augustus	g6360.t1	UniRef100_K02885	0.00E+00	4761.4
augustus	g6361.t1	UniRef100_K00600	1.10E-263	914.1
augustus	g6363.t1	UniRef100_K18995	0.00E+00	2213.7
augustus	g6369.t1	UniRef100_K18337	5.20E-141	505.4
augustus	g6370.t1	UniRef100_K12661	5.70E-251	871.3
augustus	g6372.t1	UniRef100_K14844	1.20E-300	1036.9
augustus	g6374.t1	UniRef100_K03012	2.20E-59	233.8
augustus	g6375.t1	UniRef100_K20303	9.70E-22	108.6
augustus	g6380.t1	UniRef100_K12158	1.00E-25	121.3
augustus	g6381.t1	UniRef100_K15077	2.10E-79	301.2
augustus	g6382.t1	UniRef100_K03139	7.60E-176	621.7
augustus	g6386.t1	UniRef100_K14004	3.80E-162	575.9
augustus	g6392.t1	UniRef100_K13123	0.00E+00	1725.7
augustus	g6395.t1	UniRef100_K13352	5.50E-92	342.4
augustus	g6396.t1	UniRef100_K03841	2.20E-172	610.1
augustus	g6403.t1	UniRef100_K12606	1.40E-153	547.7
augustus	g6404.t1	UniRef100_K17887	0.00E+00	2453.3
augustus	g6405.t1	UniRef100_K00003	3.80E-195	685.6
augustus	g6411.t1	UniRef100_K14864	1.40E-171	607.4
augustus	g6413.t1	UniRef100_K00948	2.00E-169	600.1
augustus	g6416.t1	UniRef100_K10704	4.70E-77	292

augustus	g6419.t1	UniRef100_K15271	7.10E-101	372.5
augustus	g6423.t1	UniRef100_K11415	2.80E-39	167.9
augustus	g6425.t1	UniRef100_K13115	1.00E-107	395.2
augustus	g6426.t1	UniRef100_K10576	2.70E-77	293.1
augustus	g6427.t1	UniRef100_K00606	2.40E-142	510
augustus	g6429.t1	UniRef100_K02309	2.80E-278	962.6
augustus	g6430.t1	UniRef100_K18573	3.40E-239	832.4
augustus	g6435.t1	UniRef100_K12251	4.00E-17	94
augustus	g6442.t1	UniRef100_K02933	2.40E-99	367.5
augustus	g6444.t1	UniRef100_K07375	7.00E-255	884.4
augustus	g6445.t1	UniRef100_K06158	0.00E+00	1253.4
augustus	g6448.t1	UniRef100_K07005	5.50E-106	389
augustus	g6462.t1	UniRef100_K03381	1.60E-158	563.9
augustus	g6463.t1	UniRef100_K14334	1.40E-185	654.1
augustus	g6467.t1	UniRef100_K09490	0.00E+00	1231.1
augustus	g6468.t1	UniRef100_K14416	5.5e-311	1071.6
augustus	g6469.t1	UniRef100_K09497	4.30E-288	995
augustus	g6474.t1	UniRef100_K02180	1.70E-187	660.2
augustus	g6475.t1	UniRef100_K03237	2.20E-153	547
augustus	g6479.t1	UniRef100_K02892	9.80E-84	314.7
augustus	g6481.t1	UniRef100_K15175	1.40E-204	717.2
augustus	g6482.t1	UniRef100_K15084	2.50E-182	643.3
augustus	g6487.t1	UniRef100_K14797	2.10E-254	882.9
augustus	g6493.t1	UniRef100_K11770	0.00E+00	1075.8
augustus	g6494.t1	UniRef100_K13449	3.30E-42	176.4
augustus	g6495.t1	UniRef100_K17808	9.80E-64	248.1
augustus	g6496.t1	UniRef100_K14772	0.00E+00	4608.9
augustus	g6498.t1	UniRef100_K02555	5.60E-215	752.3
augustus	g6500.t1	UniRef100_K02331	0.00E+00	1549.3
augustus	g6501.t1	UniRef100_K02727	1.50E-147	527.3
augustus	g6503.t1	UniRef100_K14319	1.20E-174	617.8
augustus	g6505.t1	UniRef100_K00413	3.30E-148	529.6
augustus	g6507.t1	UniRef100_K18740	3.30E-51	207.2
augustus	g6508.t1	UniRef100_K03061	4.20E-227	792
augustus	g6509.t1	UniRef100_K09496	1.70E-284	983
augustus	g6514.t1	UniRef100_K13993	4.10E-46	190.7
augustus	g6517.t1	UniRef100_K10901	0.00E+00	2195.6
augustus	g6522.t1	UniRef100_K14635	0.00E+00	1716.4
augustus	g6523.t1	UniRef100_K14284	4.80E-290	1001.9
augustus	g6528.t1	UniRef100_K06639	4.20E-277	958.7
augustus	g6530.t1	UniRef100_K03441	8.30E-192	675.2
augustus	g6533.t1	UniRef100_K01940	8.80E-180	634.8
augustus	g6542.t1	UniRef100_K21295	1.70E-138	498
augustus	g6544.t1	UniRef100_K12833	3.60E-59	232.3
augustus	g6553.t1	UniRef100_K01179	4.70E-225	785.4
augustus	g6559.t1	UniRef100_K00963	6.10E-266	921.4
augustus	g6563.t1	UniRef100_K01923	6.50E-126	455.7
augustus	g6564.t1	UniRef100_K01426	2.20E-175	620.5
augustus	g6571.t1	UniRef100_K21547	1.00E-77	296.2
augustus	g6578.t1	UniRef100_K01655	1.00E-204	718

augustus	g6582.t1	UniRef100_K14675	1.30E-293	1013.4
augustus	g6583.t1	UniRef100_K15177	5.90E-147	525.8
augustus	g6591.t1	UniRef100_K13176	5.20E-45	186.8
augustus	g6592.t1	UniRef100_K17292	2.00E-44	183.3
augustus	g6594.t1	UniRef100_K20291	0.00E+00	2255.7
augustus	g6595.t1	UniRef100_K07566	9.40E-217	757.7
augustus	g6596.t1	UniRef100_K20472	5.30E-92	342.4
augustus	g6597.t1	UniRef100_K02212	0.00E+00	1766.9
augustus	g6599.t1	UniRef100_K18477	5.50E-180	635.6
augustus	g6600.t1	UniRef100_K00844	4.60E-256	888.6
augustus	g6601.t1	UniRef100_K12666	2.40E-244	849.7
augustus	g6605.t1	UniRef100_K08497	4.10E-100	370.2
augustus	g6606.t1	UniRef100_K08964	5.20E-125	452.2
augustus	g6608.t1	UniRef100_K08054	6.00E-196	689.1
augustus	g6609.t1	UniRef100_K00140	1.40E-279	966.8
augustus	g6611.t1	UniRef100_K03380	1.60E-69	269.2
augustus	g6615.t1	UniRef100_K10735	1.10E-102	377.9
augustus	g6616.t1	UniRef100_K00467	3.00E-201	706.1
augustus	g6624.t1	UniRef100_K19800	0.00E+00	1158.7
augustus	g6625.t1	UniRef100_K02141	1.60E-49	200.3
augustus	g6627.t1	UniRef100_K22008	2.70E-255	885.9
augustus	g6628.t1	UniRef100_K12662	5.30E-203	712.2
augustus	g6630.t1	UniRef100_K12627	8.70E-46	187.6
augustus	g6639.t1	UniRef100_K11551	9.40E-253	877.5
augustus	g6640.t1	UniRef100_K03424	1.60E-186	657.1
augustus	g6641.t1	UniRef100_K10807	0.00E+00	1644.4
augustus	g6645.t1	UniRef100_K06911	1.20E-147	528.1
augustus	g6650.t1	UniRef100_K01050	2.70E-59	235
augustus	g6651.t1	UniRef100_K06110	0.00E+00	1421.8
augustus	g6652.t1	UniRef100_K12472	0.00E+00	1343.2
augustus	g6653.t1	UniRef100_K00383	2.50E-242	842.8
augustus	g6660.t1	UniRef100_K18187	7.80E-41	171
augustus	g6663.t1	UniRef100_K14397	1.20E-148	530.8
augustus	g6665.t1	UniRef100_K03939	5.10E-69	265.8
augustus	g6667.t1	UniRef100_K01408	0.00E+00	1648.3
augustus	g6668.t1	UniRef100_K11835	0.00E+00	2449.5
augustus	g6670.t1	UniRef100_K01120	1.80E-156	558.5
augustus	g6676.t1	UniRef100_K03781	1.90E-280	969.5
augustus	g6677.t1	UniRef100_K07942	4.50E-89	332.4
augustus	g6678.t1	UniRef100_K03094	1.30E-89	334
augustus	g6679.t1	UniRef100_K08098	6.50E-227	792
augustus	g6680.t1	UniRef100_K02865	7.90E-109	398.3
augustus	g6684.t1	UniRef100_K03265	1.70E-236	823.5
augustus	g6687.t1	UniRef100_K02938	1.90E-127	460.7
augustus	g6688.t1	UniRef100_K00856	5.50E-178	628.6
augustus	g6691.t1	UniRef100_K12873	1.20E-83	313.9
augustus	g6692.t1	UniRef100_K03950	7.30E-49	198.7
augustus	g6693.t1	UniRef100_K03020	7.50E-54	215.3
augustus	g6695.t1	UniRef100_K08286	1.50E-206	723.8
augustus	g6696.t1	UniRef100_K07359	2.1e-310	1069.3

augustus	g6697.t1	UniRef100_K20300	7.40E-66	255.4
augustus	g6698.t1	UniRef100_K13336	8.00E-247	857.8
augustus	g6700.t1	UniRef100_K09413	7.60E-97	360.1
augustus	g6704.t1	UniRef100_K00326	6.10E-156	555.4
augustus	g6707.t1	UniRef100_K21996	1.20E-298	1030.4
augustus	g6708.t1	UniRef100_K00987	1.90E-206	723.4
augustus	g6710.t1	UniRef100_K11267	0.00E+00	2201.4
augustus	g6711.t1	UniRef100_K11648	4.20E-286	988.8
augustus	g6712.t1	UniRef100_K01265	4.90E-168	595.9
augustus	g6714.t1	UniRef100_K17974	0.00E+00	1432.9
augustus	g6715.t1	UniRef100_K19833	2.60E-294	1016.5
augustus	g6717.t1	UniRef100_K18156	2.90E-135	486.5
augustus	g6718.t1	UniRef100_K08850	5.70E-149	532.3
augustus	g6719.t1	UniRef100_K17607	2.90E-132	476.5
augustus	g6721.t1	UniRef100_K17678	1.50E-276	956.8
augustus	g6726.t1	UniRef100_K15206	6.10E-120	435.6
augustus	g6727.t1	UniRef100_K10268	9.20E-280	967.6
augustus	g6728.t1	UniRef100_K08286	0.00E+00	1530.8
augustus	g6729.t1	UniRef100_K20407	0.00E+00	1787.3
augustus	g6732.t1	UniRef100_K10669	4.20E-115	419.5
augustus	g6736.t1	UniRef100_K11240	3.40E-305	1052.4
augustus	g6737.t1	UniRef100_K15275	2.10E-200	703.4
augustus	g6738.t1	UniRef100_K18738	3.40E-176	622.9
augustus	g6740.t1	UniRef100_K18469	0.00E+00	1063.9
augustus	g6741.t1	UniRef100_K01312	9.40E-99	365.2
augustus	g6746.t1	UniRef100_K01057	1.20E-139	500.7
augustus	g6748.t1	UniRef100_K20526	1.00E-184	651.7
augustus	g6749.t1	UniRef100_K18160	5.90E-63	245.7
augustus	g6751.t1	UniRef100_K01051	5.00E-106	389.4
augustus	g6753.t1	UniRef100_K21295	4.10E-158	563.1
augustus	g6757.t1	UniRef100_K08157	3.30E-85	321.2
augustus	g6758.t1	UniRef100_K15264	2.00E-257	893.3
augustus	g6765.t1	UniRef100_K05658	0.00E+00	2076.2
augustus	g6768.t1	UniRef100_K10696	1.50E-285	986.9
augustus	g6769.t1	UniRef100_K15198	2.60E-126	457.6
augustus	g6770.t1	UniRef100_K10839	2.00E-87	328.2
augustus	g6778.t1	UniRef100_K09500	1.90E-289	999.6
augustus	g6782.t1	UniRef100_K02921	4.00E-40	169.1
augustus	g6784.t1	UniRef100_K17427	1.10E-151	541.2
augustus	g6785.t1	UniRef100_K06669	0.00E+00	1775
augustus	g6786.t1	UniRef100_K15192	0.00E+00	3415.6
augustus	g6787.t1	UniRef100_K09554	5.80E-189	665.6
augustus	g6791.t1	UniRef100_K16315	1.60E-240	837
augustus	g6794.t1	UniRef100_K08726	1.90E-55	221.5
augustus	g6796.t1	UniRef100_K14802	0.00E+00	2476.8
augustus	g6802.t1	UniRef100_K11341	1.10E-122	444.5
augustus	g6803.t1	UniRef100_K17775	1.00E-260	904
augustus	g6804.t1	UniRef100_K19786	2.70E-219	766.5
augustus	g6805.t1	UniRef100_K15014	2.90E-171	606.7
augustus	g6808.t1	UniRef100_K01192	2.30E-222	777.3

augustus	g6812.t1	UniRef100_K15174	4.60E-223	778.9
augustus	g6814.t1	UniRef100_K02957	4.80E-68	261.9
augustus	g6815.t1	UniRef100_K02951	1.30E-62	244.2
augustus	g6816.t1	UniRef100_K09579	1.20E-30	138.3
augustus	g6817.t1	UniRef100_K06176	0.00E+00	1079.7
augustus	g6818.t1	UniRef100_K08294	6.30E-279	964.5
augustus	g6819.t1	UniRef100_K14778	7.70E-261	904.4
augustus	g6825.t1	UniRef100_K00077	5.30E-128	462.6
augustus	g6826.t1	UniRef100_K00826	3.60E-167	592.8
augustus	g6828.t1	UniRef100_K00219	6.20E-65	253.4
augustus	g6836.t1	UniRef100_K11121	6.80E-226	788.5
augustus	g6837.t1	UniRef100_K09650	7.30E-259	897.9
augustus	g6838.t1	UniRef100_K01190	0.00E+00	1155.6
augustus	g6844.t1	UniRef100_K13091	4.50E-204	716.1
augustus	g6845.t1	UniRef100_K19703	4.40E-202	708.8
augustus	g6847.t1	UniRef100_K19708	9.90E-255	884.4
augustus	g6849.t1	UniRef100_K07243	4.50E-168	595.9
augustus	g6850.t1	UniRef100_K19791	1.30E-305	1053.5
augustus	g6851.t1	UniRef100_K00999	6.90E-157	558.5
augustus	g6852.t1	UniRef100_K01950	1.20E-281	974.2
augustus	g6853.t1	UniRef100_K13421	7.30E-197	691.4
augustus	g6856.t1	UniRef100_K03105	2.20E-113	413.7
augustus	g6857.t1	UniRef100_K04648	0.00E+00	1802.3
augustus	g6859.t1	UniRef100_K04499	1.10E-250	870.5
augustus	g6867.t1	UniRef100_K01102	6.90E-300	1034.2
augustus	g6873.t1	UniRef100_K17411	8.00E-21	104.8
augustus	g6875.t1	UniRef100_K14005	0.00E+00	1710.7
augustus	g6877.t1	UniRef100_K01802	2.60E-38	163.3
augustus	g6879.t1	UniRef100_K02267	5.30E-41	172.2
augustus	g6881.t1	UniRef100_K02947	2.40E-88	329.7
augustus	g6885.t1	UniRef100_K11338	1.20E-249	867.1
augustus	g6889.t1	UniRef100_K00134	3.40E-158	562.8
augustus	g6891.t1	UniRef100_K14016	1.20E-183	647.5
augustus	g6902.t1	UniRef100_K04077	1.20E-305	1053.9
augustus	g6907.t1	UniRef100_K18442	0.00E+00	3038.4
augustus	g6912.t1	UniRef100_K01183	3.60E-157	559.3
augustus	g6920.t1	UniRef100_K02135	1.50E-23	113.6
augustus	g6921.t1	UniRef100_K20823	0.00E+00	1238.8
augustus	g6922.t1	UniRef100_K13354	3.10E-141	506.5
augustus	g6924.t1	UniRef100_K03036	1.20E-214	750.7
augustus	g6925.t1	UniRef100_K03687	3.60E-97	359.8
augustus	g6926.t1	UniRef100_K10754	0.00E+00	1318.1
augustus	g6928.t1	UniRef100_K10756	5.90E-181	638.6
augustus	g6930.t1	UniRef100_K01895	0.00E+00	1293.5
augustus	g6938.t1	UniRef100_K02925	1.30E-208	730.7
augustus	g6943.t1	UniRef100_K01657	4.20E-282	975.3
augustus	g6944.t1	UniRef100_K03510	6.10E-275	951.4
augustus	g6947.t1	UniRef100_K20310	1.50E-118	431
augustus	g6948.t1	UniRef100_K07025	1.10E-28	132.5
augustus	g6950.t1	UniRef100_K01653	2.20E-140	503.4

augustus	g6951.t1	UniRef100_K00611	3.90E-173	612.5
augustus	g6954.t1	UniRef100_K06221	7.70E-43	179.5
augustus	g6958.t1	UniRef100_K00627	9.30E-218	761.1
augustus	g6959.t1	UniRef100_K12232	0.00E+00	1965.7
augustus	g6960.t1	UniRef100_K17440	8.30E-70	268.5
augustus	g6961.t1	UniRef100_K19829	0.00E+00	1168.3
augustus	g6965.t1	UniRef100_K01178	0.00E+00	1148.3
augustus	g6966.t1	UniRef100_K11984	5.70E-242	842
augustus	g6970.t1	UniRef100_K18328	2.10E-240	836.6
augustus	g6971.t1	UniRef100_K03872	5.70E-54	214.9
augustus	g6973.t1	UniRef100_K11553	5.00E-09	65.1
augustus	g6975.t1	UniRef100_K00809	4.00E-200	702.2
augustus	g6977.t1	UniRef100_K00078	9.80E-41	172.9
augustus	g6978.t1	UniRef100_K15444	6.80E-107	391.7
augustus	g6981.t1	UniRef100_K17262	4.60E-103	379
augustus	g6990.t1	UniRef100_K03235	0.00E+00	1797.7
augustus	g6991.t1	UniRef100_K16261	2.90E-303	1045.4
augustus	g6994.t1	UniRef100_K18730	0.00E+00	2051.2
augustus	g6995.t1	UniRef100_K03538	1.30E-105	387.5
augustus	g6997.t1	UniRef100_K01918	3.60E-177	625.9
augustus	g6998.t1	UniRef100_K22134	3.30E-263	912.5
augustus	g7003.t1	UniRef100_K00799	3.60E-137	492.7
augustus	g7004.t1	UniRef100_K13119	9.30E-116	422.2
augustus	g7006.t1	UniRef100_K17742	1.30E-47	195.3
augustus	g7008.t1	UniRef100_K06970	5.30E-186	655.6
augustus	g7012.t1	UniRef100_K08178	8.00E-261	904.4
augustus	g7018.t1	UniRef100_K00480	7.80E-188	661.8
augustus	g7019.t1	UniRef100_K02304	1.50E-135	487.3
augustus	g7020.t1	UniRef100_K12481	0.00E+00	1110.1
augustus	g7022.t1	UniRef100_K00290	1.20E-176	624.4
augustus	g7024.t1	UniRef100_K00657	1.10E-30	138.3
augustus	g7032.t1	UniRef100_K01190	6.30E-77	293.9
augustus	g7037.t1	UniRef100_K18468	0.00E+00	1498.4
augustus	g7038.t1	UniRef100_K00469	1.10E-171	607.4
augustus	g7042.t1	UniRef100_K01652	0.00E+00	1179.5
augustus	g7047.t1	UniRef100_K00849	1.40E-267	926.8
augustus	g7050.t1	UniRef100_K07749	3.70E-44	184.5
augustus	g7055.t1	UniRef100_K18576	2.80E-70	270.4
augustus	g7058.t1	UniRef100_K07575	6.30E-110	402.1
augustus	g7061.t1	UniRef100_K07975	4.90E-74	282.7
augustus	g7062.t1	UniRef100_K17421	6.30E-74	282
augustus	g7064.t1	UniRef100_K00059	1.50E-31	141.7
augustus	g7069.t1	UniRef100_K17496	3.30E-219	766.1
augustus	g7071.t1	UniRef100_K12199	4.40E-96	356.7
augustus	g7072.t1	UniRef100_K09584	6.30E-200	701.8
augustus	g7073.t1	UniRef100_K11600	1.10E-101	374.8
augustus	g7074.t1	UniRef100_K21989	0.00E+00	1493
augustus	g7077.t1	UniRef100_K11098	8.90E-26	121.3
augustus	g7079.t1	UniRef100_K06950	8.70E-96	354.8
augustus	g7082.t1	UniRef100_K00147	2.40E-212	743

augustus	g7085.t1	UniRef100_K01754	1.80E-185	653.7
augustus	g7087.t1	UniRef100_K01440	1.70E-39	167.9
augustus	g7088.t1	UniRef100_K00021	0.00E+00	1977.6
augustus	g7089.t1	UniRef100_K21815	1.90E-97	361.3
augustus	g7099.t1	UniRef100_K09702	1.50E-89	334
augustus	g7100.t1	UniRef100_K15196	0.00E+00	1170.2
augustus	g7101.t1	UniRef100_K20737	8.60E-108	395.6
augustus	g7103.t1	UniRef100_K00015	1.10E-111	408.3
augustus	g7105.t1	UniRef100_K00306	3.90E-144	516.5
augustus	g7106.t1	UniRef100_K14859	1.30E-187	661
augustus	g7107.t1	UniRef100_K14777	1.30E-211	740.7
augustus	g7111.t1	UniRef100_K19881	0.00E+00	1197.2
augustus	g7112.t1	UniRef100_K06883	1.50E-166	590.9
augustus	g7113.t1	UniRef100_K03009	1.80E-33	146.4
augustus	g7117.t1	UniRef100_K19953	0.00E+00	1224.9
augustus	g7119.t1	UniRef100_K14849	1.40E-93	347.8
augustus	g7122.t1	UniRef100_K21989	0.00E+00	1654.4
augustus	g7124.t1	UniRef100_K00721	5.90E-129	465.3
augustus	g7125.t1	UniRef100_K00059	5.10E-139	498.8
augustus	g7126.t1	UniRef100_K20309	3.50E-211	739.2
augustus	g7127.t1	UniRef100_K15205	7.20E-294	1014.6
augustus	g7128.t1	UniRef100_K07297	2.30E-133	480.3
augustus	g7130.t1	UniRef100_K08827	3.50E-295	1019.2
augustus	g7141.t1	UniRef100_K14338	0.00E+00	1686
augustus	g7146.t1	UniRef100_K15901	3.50E-86	322.8
augustus	g7147.t1	UniRef100_K11394	1.30E-29	136
augustus	g7153.t1	UniRef100_K00480	1.90E-87	328.2
augustus	g7157.t1	UniRef100_K01569	8.40E-198	694.9
augustus	g7158.t1	UniRef100_K00219	5.90E-72	276.6
augustus	g7159.t1	UniRef100_K00354	6.20E-174	615.1
augustus	g7161.t1	UniRef100_K17742	1.80E-138	496.9
augustus	g7165.t1	UniRef100_K01561	5.60E-25	120.2
augustus	g7168.t1	UniRef100_K06893	6.90E-10	68.9
augustus	g7173.t1	UniRef100_K12957	7.10E-40	169.5
augustus	g7174.t1	UniRef100_K22134	4.50E-54	217.6
augustus	g7178.t1	UniRef100_K01495	4.70E-130	469.2
augustus	g7192.t1	UniRef100_K13796	1.10E-131	475.3
augustus	g7196.t1	UniRef100_K13282	3.70E-65	253.4
augustus	g7199.t1	UniRef100_K00799	8.10E-50	202.6
augustus	g7204.t1	UniRef100_K15877	1.90E-164	584.3
augustus	g7208.t1	UniRef100_K00001	6.40E-44	183.3
augustus	g7210.t1	UniRef100_K00015	3.70E-168	595.9
augustus	g7213.t1	UniRef100_K01502	1.00E-69	268.9
augustus	g7218.t1	UniRef100_K00022	8.60E-69	265.4
augustus	g7221.t1	UniRef100_K16164	7.90E-34	149.4
augustus	g7223.t1	UniRef100_K11169	9.40E-23	112.8
augustus	g7231.t1	UniRef100_K01198&K(	6.70E-77	293.5
augustus	g7233.t1	UniRef100_K10251	3.40E-94	350.1
augustus	g7240.t1	UniRef100_K22152	1.00E-72	279.3
augustus	g7243.t1	UniRef100_K15303	5.90E-127	459.1

augustus	g7245.t1	UniRef100_K01007	0.00E+00	1268.8
augustus	g7246.t1	UniRef100_K00016	1.20E-99	368.2
augustus	g7256.t1	UniRef100_K09958	9.70E-84	314.3
augustus	g7264.t1	UniRef100_K03575	3.20E-250	869.4
augustus	g7265.t1	UniRef100_K08987	1.30E-35	154.1
augustus	g7271.t1	UniRef100_K14213	8.60E-238	827.8
augustus	g7273.t1	UniRef100_K22075	1.30E-30	137.9
augustus	g7275.t1	UniRef100_K11262	0.00E+00	4313.5
augustus	g7279.t1	UniRef100_K10661	0.00E+00	2310.8
augustus	g7282.t1	UniRef100_K11874	6.00E-281	971.5
augustus	g7285.t1	UniRef100_K21632	5.50E-249	865.5
augustus	g7287.t1	UniRef100_K01078	6.10E-168	595.5
augustus	g7289.t1	UniRef100_K00457	3.30E-36	157.5
augustus	g7290.t1	UniRef100_K00033	3.20E-229	799.3
augustus	g7291.t1	UniRef100_K00034	1.50E-32	145.2
augustus	g7296.t1	UniRef100_K05882	1.30E-36	159.1
augustus	g7297.t1	UniRef100_K06889	1.40E-62	245
augustus	g7300.t1	UniRef100_K01190	0.00E+00	1423.3
augustus	g7302.t1	UniRef100_K15923	7.40E-177	625.9
augustus	g7315.t1	UniRef100_K20039	4.20E-184	649.4
augustus	g7316.t1	UniRef100_K03186	1.30E-78	297.7
augustus	g7321.t1	UniRef100_K00134	3.60E-140	503.1
augustus	g7323.t1	UniRef100_K03790	6.30E-09	66.2
augustus	g7324.t1	UniRef100_K03935	5.20E-248	861.7
augustus	g7325.t1	UniRef100_K06126	1.10E-259	900.6
augustus	g7326.t1	UniRef100_K02929	3.60E-78	298.1
augustus	g7327.t1	UniRef100_K02127	1.40E-106	391
augustus	g7330.t1	UniRef100_K20367	2.20E-224	783.1
augustus	g7332.t1	UniRef100_K02739	3.60E-150	535.8
augustus	g7333.t1	UniRef100_K18715	0.00E+00	1508.8
augustus	g7335.t1	UniRef100_K03027	2.60E-204	716.1
augustus	g7336.t1	UniRef100_K10686	2.00E-117	427.9
augustus	g7337.t1	UniRef100_K11137	0.00E+00	1583.5
augustus	g7340.t1	UniRef100_K15340	0.00E+00	1229.9
augustus	g7341.t1	UniRef100_K12621	2.80E-41	172.6
augustus	g7342.t1	UniRef100_K14556	0.00E+00	1554.3
augustus	g7343.t1	UniRef100_K05758	1.10E-150	537.7
augustus	g7348.t1	UniRef100_K12828	0.00E+00	2063.9
augustus	g7349.t1	UniRef100_K03660	1.90E-185	653.7
augustus	g7351.t1	UniRef100_K02953	7.90E-74	281.6
augustus	g7352.t1	UniRef100_K14968	5.10E-52	208.8
augustus	g7353.t1	UniRef100_K15363	0.00E+00	1389.8
augustus	g7355.t1	UniRef100_K08064	1.10E-62	245.4
augustus	g7358.t1	UniRef100_K19589	1.80E-111	407.1
augustus	g7360.t1	UniRef100_K03869	0.00E+00	1424.1
augustus	g7361.t1	UniRef100_K01557	3.90E-47	193.4
augustus	g7362.t1	UniRef100_K00452	3.90E-91	339.3
augustus	g7363.t1	UniRef100_K11426	5.60E-251	871.7
augustus	g7369.t1	UniRef100_K13346	1.30E-174	617.5
augustus	g7370.t1	UniRef100_K15263	4.20E-112	409.8

augustus	g7371.t1	UniRef100_K06889	1.10E-155	554.7
augustus	g7376.t1	UniRef100_K20929	0.00E+00	1629.4
augustus	g7377.t1	UniRef100_K22138	1.50E-57	227.3
augustus	g7378.t1	UniRef100_K01958	0.00E+00	2223.4
augustus	g7384.t1	UniRef100_K04564	7.20E-145	518.5
augustus	g7386.t1	UniRef100_K21547	0.00E+00	1227.2
augustus	g7394.t1	UniRef100_K20195	2.20E-301	1039.3
augustus	g7398.t1	UniRef100_K15216	0.00E+00	1183.3
augustus	g7399.t1	UniRef100_K08065	9.50E-86	322
augustus	g7400.t1	UniRef100_K09549	3.90E-47	192.6
augustus	g7401.t1	UniRef100_K18172	2.50E-29	132.5
augustus	g7403.t1	UniRef100_K12668	1.00E-45	188.3
augustus	g7405.t1	UniRef100_K14292	1.70E-140	504.2
augustus	g7408.t1	UniRef100_K00480	8.90E-150	535.4
augustus	g7409.t1	UniRef100_K06116	5.00E-118	429.1
augustus	g7415.t1	UniRef100_K12821	1.10E-271	941
augustus	g7416.t1	UniRef100_K06174	0.00E+00	1138.6
augustus	g7417.t1	UniRef100_K11367	0.00E+00	2325.1
augustus	g7418.t1	UniRef100_K01082	1.50E-114	417.9
augustus	g7419.t1	UniRef100_K13338	0.00E+00	2082.4
augustus	g7420.t1	UniRef100_K02210	0.00E+00	1434.9
augustus	g7421.t1	UniRef100_K17408	1.60E-203	713.8
augustus	g7422.t1	UniRef100_K14852	9.80E-81	304.7
augustus	g7424.t1	UniRef100_K05956	2.70E-128	463.4
augustus	g7426.t1	UniRef100_K08736	0.00E+00	1809.3
augustus	g7428.t1	UniRef100_K17497	1.80E-130	470.7
augustus	g7429.t1	UniRef100_K13754	0.00E+00	1557
augustus	g7430.t1	UniRef100_K06676	0.00E+00	1179.1
augustus	g7433.t1	UniRef100_K19691	0.00E+00	2371.3
augustus	g7435.t1	UniRef100_K13800	1.10E-138	497.7
augustus	g7448.t1	UniRef100_K02349	0.00E+00	1384.4
augustus	g7450.t1	UniRef100_K08856	6.80E-192	675.2
augustus	g7451.t1	UniRef100_K01191	0.00E+00	2058.5
augustus	g7452.t1	UniRef100_K03555	3.50E-102	378.3
augustus	g7453.t1	UniRef100_K02941	1.70E-79	301.2
augustus	g7454.t1	UniRef100_K15443	1.40E-233	813.9
augustus	g7455.t1	UniRef100_K09503	5.30E-178	629
augustus	g7458.t1	UniRef100_K14766	0.00E+00	1149.8
augustus	g7459.t1	UniRef100_K00784	0.00E+00	1518.4
augustus	g7461.t1	UniRef100_K08287	2.40E-289	999.6
augustus	g7464.t1	UniRef100_K07943	2.30E-71	273.1
augustus	g7466.t1	UniRef100_K13509	6.50E-137	491.9
augustus	g7468.t1	UniRef100_K09540	0.00E+00	1243.8
augustus	g7470.t1	UniRef100_K07238	1.80E-185	654.1
augustus	g7471.t1	UniRef100_K09191	7.10E-166	589
augustus	g7472.t1	UniRef100_K03014	7.10E-63	245.4
augustus	g7474.t1	UniRef100_K01070	1.20E-144	517.7
augustus	g7481.t1	UniRef100_K11673	0.00E+00	1354.3
augustus	g7487.t1	UniRef100_K00052	1.70E-164	583.9
augustus	g7494.t1	UniRef100_K14288	0.00E+00	1867

augustus	g7497.t1	UniRef100_K01267	3.30E-245	852.4
augustus	g7506.t1	UniRef100_K14274	4.60E-35	153.7
augustus	g7511.t1	UniRef100_K07953	1.40E-95	354.4
augustus	g7513.t1	UniRef100_K01173	1.70E-130	470.7
augustus	g7514.t1	UniRef100_K15542	1.20E-241	840.9
augustus	g7515.t1	UniRef100_K11155	4.20E-235	818.9
augustus	g7516.t1	UniRef100_K00587	4.30E-119	432.6
augustus	g7522.t1	UniRef100_K04708	5.40E-150	535.8
augustus	g7523.t1	UniRef100_K14862	0.00E+00	2124
augustus	g7524.t1	UniRef100_K17784	2.10E-38	163.3
augustus	g7529.t1	UniRef100_K01803	5.30E-125	452.2
augustus	g7530.t1	UniRef100_K17424	1.40E-66	257.3
augustus	g7532.t1	UniRef100_K14815	1.10E-115	421.4
augustus	g7537.t1	UniRef100_K17944	4.40E-136	490.7
augustus	g7542.t1	UniRef100_K09550	8.10E-46	188.3
augustus	g7548.t1	UniRef100_K00472	2.40E-95	353.6
augustus	g7553.t1	UniRef100_K14565	1.80E-245	853.6
augustus	g7554.t1	UniRef100_K02146	1.30E-194	684.1
augustus	g7558.t1	UniRef100_K06027	2.80E-296	1022.7
augustus	g7562.t1	UniRef100_K22243	9.90E-88	327.8
augustus	g7563.t1	UniRef100_K16368	3.00E-201	706.4
augustus	g7564.t1	UniRef100_K14809	0.00E+00	1089.3
augustus	g7567.t1	UniRef100_K14290	0.00E+00	2038.5
augustus	g7568.t1	UniRef100_K00830	6.00E-200	701.8
augustus	g7569.t1	UniRef100_K02516	0.00E+00	1356.7
augustus	g7570.t1	UniRef100_K12820	0.00E+00	1416.7
augustus	g7577.t1	UniRef100_K11696	7.80E-65	251.9
augustus	g7578.t1	UniRef100_K00549	0.00E+00	1418.7
augustus	g7579.t1	UniRef100_K11339	8.90E-148	528.5
augustus	g7581.t1	UniRef100_K01648	0.00E+00	1258.4
augustus	g7583.t1	UniRef100_K01648	9.90E-276	953.7
augustus	g7585.t1	UniRef100_K00942	6.30E-94	348.6
augustus	g7586.t1	UniRef100_K16938	2.30E-191	672.9
augustus	g7587.t1	UniRef100_K12601	4.10E-188	662.5
augustus	g7588.t1	UniRef100_K03505	1.80E-57	227.6
augustus	g7590.t1	UniRef100_K10685	1.30E-282	977.2
augustus	g7591.t1	UniRef100_K00059	1.20E-77	295
augustus	g7593.t1	UniRef100_K10756	0.00E+00	1208
augustus	g7599.t1	UniRef100_K05863	1.80E-167	593.6
augustus	g7600.t1	UniRef100_K02975	6.60E-43	177.9
augustus	g7601.t1	UniRef100_K02989	1.60E-100	370.9
augustus	g7603.t1	UniRef100_K05727	0.00E+00	3506.8
augustus	g7609.t1	UniRef100_K01869	0.00E+00	2135.5
augustus	g7612.t1	UniRef100_K19054	1.50E-74	284.3
augustus	g7616.t1	UniRef100_K03142	1.10E-245	854
augustus	g7617.t1	UniRef100_K00457	6.80E-225	784.6
augustus	g7620.t1	UniRef100_K02901	4.10E-70	268.9
augustus	g7622.t1	UniRef100_K01854	2.10E-86	325.1
augustus	g7628.t1	UniRef100_K01477	5.00E-166	589
augustus	g7634.t1	UniRef100_K09495	2.70E-293	1012.3

augustus	g7638.t1	UniRef100_K03639	2.10E-288	996.5
augustus	g7643.t1	UniRef100_K13289	9.30E-307	1057
augustus	g7644.t1	UniRef100_K01867	4.50E-242	842
augustus	g7645.t1	UniRef100_K14574	3.30E-144	516.2
augustus	g7646.t1	UniRef100_K02964	3.30E-76	289.7
augustus	g7650.t1	UniRef100_K15378	0.00E+00	1134.4
augustus	g7655.t1	UniRef100_K01409	1.30E-143	515
augustus	g7656.t1	UniRef100_K02737	2.10E-154	550.1
augustus	g7661.t1	UniRef100_K05658	1.90E-273	947.6
augustus	g7662.t1	UniRef100_K01892	7.40E-265	917.9
augustus	g7664.t1	UniRef100_K08794	2.70E-207	726.1
augustus	g7666.t1	UniRef100_K10688	1.50E-71	274.2
augustus	g7667.t1	UniRef100_K20182	3.40E-94	351.3
augustus	g7670.t1	UniRef100_K03163	0.00E+00	1341.3
augustus	g7673.t1	UniRef100_K21545	4.30E-224	782.3
augustus	g7677.t1	UniRef100_K01410	0.00E+00	1449.5
augustus	g7678.t1	UniRef100_K17786	1.30E-111	407.5
augustus	g7679.t1	UniRef100_K05543	6.80E-217	758.1
augustus	g7681.t1	UniRef100_K22285	1.40E-205	721.1
augustus	g7682.t1	UniRef100_K12836	5.80E-117	425.2
augustus	g7683.t1	UniRef100_K12366	7.30E-211	738.8
augustus	g7684.t1	UniRef100_K20359	7.30E-91	339.7
augustus	g7685.t1	UniRef100_K10689	6.80E-88	328.2
augustus	g7686.t1	UniRef100_K05529	4.10E-197	692.2
augustus	g7687.t1	UniRef100_K12881	2.90E-81	307.4
augustus	g7690.t1	UniRef100_K01873	0.00E+00	1931
augustus	g7691.t1	UniRef100_K12858	2.60E-304	1049.3
augustus	g7693.t1	UniRef100_K17867	6.00E-72	275.4
augustus	g7694.t1	UniRef100_K06630	1.30E-122	444.5
augustus	g7695.t1	UniRef100_K22255	4.00E-19	99.4
augustus	g7698.t1	UniRef100_K03242	7.40E-282	974.2
augustus	g7700.t1	UniRef100_K03257	2.50E-221	772.7
augustus	g7701.t1	UniRef100_K00234	0.00E+00	1142.9
augustus	g7707.t1	UniRef100_K01259	3.40E-119	433.7
augustus	g7710.t1	UniRef100_K01120	2.10E-136	491.1
augustus	g7715.t1	UniRef100_K13181	1.30E-221	774.6
augustus	g7716.t1	UniRef100_K10436	1.80E-104	384
augustus	g7717.t1	UniRef100_K08502	2.70E-177	626.3
augustus	g7720.t1	UniRef100_K00002	1.40E-205	720.3
augustus	g7722.t1	UniRef100_K03362	3.50E-250	869.4
augustus	g7723.t1	UniRef100_K01210	6.50E-237	825.5
augustus	g7729.t1	UniRef100_K10259	2.80E-269	932.9
augustus	g7731.t1	UniRef100_K15100	3.30E-159	566.2
augustus	g7732.t1	UniRef100_K08870	3.20E-159	566.2
augustus	g7734.t1	UniRef100_K02993	3.30E-93	346.3
augustus	g7735.t1	UniRef100_K10426	3.30E-302	1042
augustus	g7742.t1	UniRef100_K00001	1.20E-60	238.8
augustus	g7745.t1	UniRef100_K05349	0.00E+00	1575.8
augustus	g7746.t1	UniRef100_K00222	2.60E-261	906
augustus	g7749.t1	UniRef100_K00988	2.90E-195	686.8

augustus	g7750.t1	UniRef100_K06173	3.30E-242	842.8
augustus	g7754.t1	UniRef100_K00002	4.30E-192	675.6
augustus	g7755.t1	UniRef100_K08994	5.90E-225	785
augustus	g7767.t1	UniRef100_K06004	7.90E-70	269.6
augustus	g7768.t1	UniRef100_K07178	1.20E-211	741.1
augustus	g7774.t1	UniRef100_K16261	4.70E-271	938.3
augustus	g7775.t1	UniRef100_K14709	1.10E-160	571.2
augustus	g7777.t1	UniRef100_K03885	1.80E-225	786.9
augustus	g7778.t1	UniRef100_K08192	4.10E-256	889
augustus	g7780.t1	UniRef100_K08141	1.00E-220	771.2
augustus	g7782.t1	UniRef100_K00055	3.70E-61	240.7
augustus	g7797.t1	UniRef100_K16044	1.90E-64	251.5
augustus	g7798.t1	UniRef100_K01266	1.80E-182	643.7
augustus	g7799.t1	UniRef100_K00459	9.60E-180	634.4
augustus	g7806.t1	UniRef100_K00222	1.00E-254	884
augustus	g7812.t1	UniRef100_K09578	1.30E-83	314.3
augustus	g7813.t1	UniRef100_K03017	1.30E-48	198
augustus	g7814.t1	UniRef100_K02874	3.30E-17	93.6
augustus	g7815.t1	UniRef100_K11429	1.20E-282	978
augustus	g7816.t1	UniRef100_K00073	8.60E-51	206.1
augustus	g7817.t1	UniRef100_K07241	1.30E-173	614.4
augustus	g7819.t1	UniRef100_K08329	6.60E-174	615.5
augustus	g7821.t1	UniRef100_K08141	3.50E-290	1001.9
augustus	g7822.t1	UniRef100_K00681	6.8e-311	1070.8
augustus	g7826.t1	UniRef100_K09001	2.20E-72	278.1
augustus	g7828.t1	UniRef100_K06035	1.90E-101	374
augustus	g7835.t1	UniRef100_K08157	2.60E-89	334.3
augustus	g7840.t1	UniRef100_K01379	2.90E-140	503.4
augustus	g7843.t1	UniRef100_K14264	7.80E-176	621.7
augustus	g7845.t1	UniRef100_K08178	9.20E-177	625.2
augustus	g7846.t1	UniRef100_K03417	2.40E-111	407.1
augustus	g7847.t1	UniRef100_K13796	1.20E-103	382.1
augustus	g7852.t1	UniRef100_K18660	4.20E-143	513.5
augustus	g7853.t1	UniRef100_K00232	1.60E-106	391.3
augustus	g7860.t1	UniRef100_K21293	4.60E-66	257.3
augustus	g7864.t1	UniRef100_K15394	0.00E+00	4613.9
augustus	g7865.t1	UniRef100_K22134	9.00E-188	661.8
augustus	g7866.t1	UniRef100_K05933	2.20E-26	124.8
augustus	g7868.t1	UniRef100_K15385	2.20E-62	244.6
augustus	g7872.t1	UniRef100_K01687	2.90E-269	932.6
augustus	g7873.t1	UniRef100_K00059	1.40E-83	314.7
augustus	g7874.t1	UniRef100_K18369	2.60E-53	214.5
augustus	g7875.t1	UniRef100_K16261	2.10E-163	580.9
augustus	g7876.t1	UniRef100_K01560	9.90E-49	198.7
augustus	g7882.t1	UniRef100_K01166	3.20E-131	473.4
augustus	g7883.t1	UniRef100_K19668	8.90E-214	748
augustus	g7885.t1	UniRef100_K15532	6.10E-201	704.9
augustus	g7886.t1	UniRef100_K08192	4.70E-67	260.8
augustus	g7892.t1	UniRef100_K01179	8.10E-202	708
augustus	g7893.t1	UniRef100_K11161	1.00E-31	142.5

augustus	g7897.t1	UniRef100_K08726	9.30E-60	235.7
augustus	g7898.t1	UniRef100_K01259	1.60E-20	105.1
augustus	g7902.t1	UniRef100_K15920	5.10E-250	869
augustus	g7904.t1	UniRef100_K01738	7.90E-105	385.2
augustus	g7915.t1	UniRef100_K19564	5.70E-253	878.2
augustus	g7916.t1	UniRef100_K00286	1.00E-139	501.1
augustus	g7918.t1	UniRef100_K20989	0.00E+00	1080.5
augustus	g7919.t1	UniRef100_K20938	1.20E-132	478.4
augustus	g7922.t1	UniRef100_K07824	5.60E-109	399.8
augustus	g7924.t1	UniRef100_K03381	9.40E-49	199.1
augustus	g7925.t1	UniRef100_K00217	9.00E-83	312.4
augustus	g7926.t1	UniRef100_K07078	1.20E-49	201.4
augustus	g7928.t1	UniRef100_K03929	3.40E-65	254.6
augustus	g7937.t1	UniRef100_K15223	3.90E-55	220.3
augustus	g7939.t1	UniRef100_K08852	0.00E+00	1941.8
augustus	g7940.t1	UniRef100_K03253	0.00E+00	1417.9
augustus	g7944.t1	UniRef100_K10256	9.00E-216	754.6
augustus	g7948.t1	UniRef100_K02370	6.00E-159	565.5
augustus	g7955.t1	UniRef100_K01210	2.80E-153	547.7
augustus	g7963.t1	UniRef100_K13721	0.00E+00	1608.2
augustus	g7965.t1	UniRef100_K01490	0.00E+00	1299.3
augustus	g7978.t1	UniRef100_K17652	3.50E-59	233.8
augustus	g7981.t1	UniRef100_K13955	1.10E-34	152.5
augustus	g7985.t1	UniRef100_K08141	6.80E-250	868.2
augustus	g7986.t1	UniRef100_K09955	6.20E-290	1001.5
augustus	g7989.t1	UniRef100_K01050	4.60E-101	373.6
augustus	g7994.t1	UniRef100_K03457	6.10E-289	998
augustus	g7995.t1	UniRef100_K20224	0.00E+00	1791.5
augustus	g7997.t1	UniRef100_K13728	9.40E-83	312
augustus	g8001.t1	UniRef100_K10838	0.00E+00	1286.9
augustus	g8003.t1	UniRef100_K17981	7.10E-131	471.9
augustus	g8004.t1	UniRef100_K02143	2.30E-32	142.5
augustus	g8006.t1	UniRef100_K12190	1.10E-296	1023.8
augustus	g8008.t1	UniRef100_K11237	9.50E-270	934.1
augustus	g8012.t1	UniRef100_K06123	2.20E-46	191
augustus	g8015.t1	UniRef100_K03380	2.70E-164	583.9
augustus	g8016.t1	UniRef100_K22149	3.40E-137	493.8
augustus	g8017.t1	UniRef100_K08683	1.20E-44	185.3
augustus	g8021.t1	UniRef100_K11548	2.80E-174	616.7
augustus	g8023.t1	UniRef100_K12397	0.00E+00	1250.7
augustus	g8025.t1	UniRef100_K00020	4.50E-171	605.5
augustus	g8027.t1	UniRef100_K12462	4.10E-83	312.8
augustus	g8028.t1	UniRef100_K17615	6.50E-176	622.1
augustus	g8033.t1	UniRef100_K03671	2.40E-57	226.9
augustus	g8037.t1	UniRef100_K00273	1.50E-159	567.4
augustus	g8038.t1	UniRef100_K03845	1.10E-196	691
augustus	g8039.t1	UniRef100_K15025	4.40E-59	232.3
augustus	g8042.t1	UniRef100_K17983	5.30E-156	555.8
augustus	g8043.t1	UniRef100_K02677	0.00E+00	1587.4
augustus	g8044.t1	UniRef100_K09478	1.30E-121	441.8

augustus	g8045.t1	UniRef100_K02950	8.90E-69	265
augustus	g8046.t1	UniRef100_K10967	2.00E-227	793.1
augustus	g8048.t1	UniRef100_K12617	0.00E+00	1130.5
augustus	g8049.t1	UniRef100_K14721	9.00E-121	438.7
augustus	g8052.t1	UniRef100_K14765	2.00E-130	470.7
augustus	g8053.t1	UniRef100_K00254	2.60E-201	706.4
augustus	g8058.t1	UniRef100_K01613	3.50E-209	732.6
augustus	g8062.t1	UniRef100_K09561	1.10E-115	421.4
augustus	g8063.t1	UniRef100_K13953	3.20E-79	300.4
augustus	g8071.t1	UniRef100_K00344	1.10E-61	242.3
augustus	g8076.t1	UniRef100_K07246	1.90E-154	550.4
augustus	g8077.t1	UniRef100_K01561	9.60E-80	302
augustus	g8080.t1	UniRef100_K14731	2.30E-20	104.8
augustus	g8081.t1	UniRef100_K00059	7.80E-112	408.3
augustus	g8083.t1	UniRef100_K03850	1.70E-292	1010
augustus	g8086.t1	UniRef100_K17086	0.00E+00	1215.7
augustus	g8087.t1	UniRef100_K17815	2.50E-214	750
augustus	g8088.t1	UniRef100_K15292	0.00E+00	1129.4
augustus	g8090.t1	UniRef100_K07300	1.20E-161	575.1
augustus	g8093.t1	UniRef100_K01178	3.10E-66	257.3
augustus	g8097.t1	UniRef100_K03325	2.60E-161	573.5
augustus	g8115.t1	UniRef100_K03178	0.00E+00	2164.8
augustus	g8117.t1	UniRef100_K03778	1.20E-169	600.9
augustus	g8123.t1	UniRef100_K01563	2.90E-68	263.8
augustus	g8132.t1	UniRef100_K08165	4.90E-68	263.8
augustus	g8142.t1	UniRef100_K21989	0.00E+00	1420.2
augustus	g8144.t1	UniRef100_K01196	0.00E+00	2956.8
augustus	g8147.t1	UniRef100_K00006	1.20E-225	787.3
augustus	g8151.t1	UniRef100_K11996	3.60E-195	686
augustus	g8152.t1	UniRef100_K17806	4.40E-82	309.7
augustus	g8160.t1	UniRef100_K00218	1.50E-57	228.4
augustus	g8163.t1	UniRef100_K01293	3.30E-270	935.6
augustus	g8166.t1	UniRef100_K03781	1.70E-119	434.1
augustus	g8167.t1	UniRef100_K08157	1.40E-74	285.8
augustus	g8168.t1	UniRef100_K08139	2.30E-177	627.1
augustus	g8171.t1	UniRef100_K08681	3.40E-81	306.6
augustus	g8172.t1	UniRef100_K06215	3.40E-157	559.3
augustus	g8177.t1	UniRef100_K11886	0.00E+00	3043.4
augustus	g8178.t1	UniRef100_K01061	2.50E-135	486.9
augustus	g8180.t1	UniRef100_K22121	6.90E-50	203.4
augustus	g8181.t1	UniRef100_K04567	1.10E-278	964.1
augustus	g8182.t1	UniRef100_K19306	1.60E-132	477.2
augustus	g8191.t1	UniRef100_K00457	2.90E-114	416.8
augustus	g8192.t1	UniRef100_K08740	0.00E+00	1216.1
augustus	g8194.t1	UniRef100_K14855	7.10E-169	599
augustus	g8195.t1	UniRef100_K22337	0.00E+00	1731.5
augustus	g8196.t1	UniRef100_K00826	1.50E-192	677.2
augustus	g8198.t1	UniRef100_K08165	2.30E-66	258.5
augustus	g8199.t1	UniRef100_K13726	1.10E-119	436
augustus	g8206.t1	UniRef100_K05857	1.30E-69	269.2

augustus	g8209.t1	UniRef100_K20316	1.40E-73	280.8
augustus	g8210.t1	UniRef100_K20307	0.00E+00	2535
augustus	g8211.t1	UniRef100_K08963	5.60E-197	691.8
augustus	g8215.t1	UniRef100_K11681	0.00E+00	2394.8
augustus	g8218.t1	UniRef100_K11713	7.40E-194	681.8
augustus	g8225.t1	UniRef100_K00463	8.00E-125	452.2
augustus	g8230.t1	UniRef100_K01881	2.50E-307	1058.9
augustus	g8231.t1	UniRef100_K09493	2.40E-298	1029.2
augustus	g8236.t1	UniRef100_K10733	3.10E-108	396.4
augustus	g8238.t1	UniRef100_K05994	2.60E-192	676.4
augustus	g8239.t1	UniRef100_K14763	2.50E-182	643.7
augustus	g8240.t1	UniRef100_K03846	2.60E-306	1055.8
augustus	g8242.t1	UniRef100_K18185	3.20E-30	135.6
augustus	g8244.t1	UniRef100_K00799	1.80E-67	261.2
augustus	g8245.t1	UniRef100_K09958	3.40E-77	292.7
augustus	g8249.t1	UniRef100_K01673	2.10E-70	270.4
augustus	g8254.t1	UniRef100_K00505	9.30E-112	409.5
augustus	g8256.t1	UniRef100_K10736	0.00E+00	1077
augustus	g8261.t1	UniRef100_K01537	0.00E+00	2126.7
augustus	g8262.t1	UniRef100_K11121	2.50E-243	846.3
augustus	g8264.t1	UniRef100_K00826	3.20E-217	759.2
augustus	g8272.t1	UniRef100_K10742	0.00E+00	2082.8
augustus	g8278.t1	UniRef100_K07513	4.20E-202	709.1
augustus	g8279.t1	UniRef100_K07305	9.40E-77	291.2
augustus	g8280.t1	UniRef100_K12486	8.60E-183	645.2
augustus	g8281.t1	UniRef100_K08504	1.10E-40	170.6
augustus	g8282.t1	UniRef100_K20523	1.50E-203	713.8
augustus	g8289.t1	UniRef100_K11763	0.00E+00	1688.7
augustus	g8291.t1	UniRef100_K04409	0.00E+00	1250.3
augustus	g8293.t1	UniRef100_K09827	1.50E-227	793.9
augustus	g8295.t1	UniRef100_K19564	1.60E-237	827
augustus	g8296.t1	UniRef100_K07004	4.40E-82	310.8
augustus	g8316.t1	UniRef100_K00559	9.90E-195	684.5
augustus	g8327.t1	UniRef100_K13617	4.40E-190	669.1
augustus	g8329.t1	UniRef100_K11717	2.50E-36	158.3
augustus	g8334.t1	UniRef100_K01530	0.00E+00	2390.1
augustus	g8335.t1	UniRef100_K03124	5.10E-189	665.2
augustus	g8336.t1	UniRef100_K01783	2.10E-123	447.2
augustus	g8337.t1	UniRef100_K00766	7.80E-211	738
augustus	g8339.t1	UniRef100_K18703	6.30E-220	768.5
augustus	g8343.t1	UniRef100_K00480	3.00E-188	662.9
augustus	g8353.t1	UniRef100_K00728	0.00E+00	1435.2
augustus	g8355.t1	UniRef100_K05757	1.00E-203	714.1
augustus	g8357.t1	UniRef100_K01230	0.00E+00	1208.4
augustus	g8359.t1	UniRef100_K16261	7.50E-298	1027.7
augustus	g8363.t1	UniRef100_K15160	0.00E+00	1493.4
augustus	g8366.t1	UniRef100_K10981	2.30E-267	926.8
augustus	g8367.t1	UniRef100_K13350	5.10E-115	419.1
augustus	g8368.t1	UniRef100_K12178	2.80E-190	669.8
augustus	g8369.t1	UniRef100_K06662	0.00E+00	2052.7

augustus	g8370.t1	UniRef100_K20803	5.80E-189	665.2
augustus	g8375.t1	UniRef100_K11369	1.80E-261	906.7
augustus	g8389.t1	UniRef100_K01259	5.60E-84	316.2
augustus	g8394.t1	UniRef100_K17435	6.00E-83	312
augustus	g8398.t1	UniRef100_K05917	2.60E-285	985.7
augustus	g8401.t1	UniRef100_K03132	4.50E-179	632.9
augustus	g8403.t1	UniRef100_K11498	0.00E+00	1232.2
augustus	g8405.t1	UniRef100_K03321	0.00E+00	1808.5
augustus	g8406.t1	UniRef100_K10732	4.10E-108	396
augustus	g8407.t1	UniRef100_K06691	2.40E-189	667.5
augustus	g8410.t1	UniRef100_K20347	4.00E-103	379.4
augustus	g8412.t1	UniRef100_K07937	3.50E-93	346.3
augustus	g8413.t1	UniRef100_K00602	0.00E+00	1135.9
augustus	g8414.t1	UniRef100_K02918	6.60E-53	211.8
augustus	g8415.t1	UniRef100_K08495	9.00E-93	345.1
augustus	g8416.t1	UniRef100_K18192	3.50E-156	556.2
augustus	g8417.t1	UniRef100_K09699	1.10E-197	694.5
augustus	g8418.t1	UniRef100_K00851	3.30E-102	376.3
augustus	g8419.t1	UniRef100_K01277	0.00E+00	1312
augustus	g8420.t1	UniRef100_K02934	1.90E-99	367.1
augustus	g8421.t1	UniRef100_K03695	0.00E+00	1341.6
augustus	g8424.t1	UniRef100_K12811	0.00E+00	1574.3
augustus	g8427.t1	UniRef100_K00101	1.50E-170	604.4
augustus	g8429.t1	UniRef100_K20063	9.80E-296	1021.1
augustus	g8430.t1	UniRef100_K02213	3.30E-260	902.5
augustus	g8431.t1	UniRef100_K05770	6.50E-90	335.1
augustus	g8432.t1	UniRef100_K15114	3.40E-174	615.9
augustus	g8435.t1	UniRef100_K03515	0.00E+00	1641.7
augustus	g8437.t1	UniRef100_K01240	5.20E-181	639
augustus	g8438.t1	UniRef100_K15108	1.40E-147	527.3
augustus	g8442.t1	UniRef100_K11126	1.30E-298	1030.8
augustus	g8449.t1	UniRef100_K03349	0.00E+00	1500.7
augustus	g8455.t1	UniRef100_K04802	1.60E-139	500.4
augustus	g8456.t1	UniRef100_K00868	2.10E-164	583.6
augustus	g8457.t1	UniRef100_K01520	5.00E-62	242.7
augustus	g8458.t1	UniRef100_K11698	1.40E-26	125.6
augustus	g8459.t1	UniRef100_K18738	3.80E-162	576.6
augustus	g8463.t1	UniRef100_K12351	8.50E-243	844.3
augustus	g8464.t1	UniRef100_K00924	1.40E-144	518.8
augustus	g8465.t1	UniRef100_K14826	2.70E-123	447.6
augustus	g8469.t1	UniRef100_K11108	5.20E-222	775
augustus	g8473.t1	UniRef100_K01303	8.50E-94	350.1
augustus	g8475.t1	UniRef100_K07936	4.40E-146	523.5
augustus	g8477.t1	UniRef100_K00858	8.0e-312	1073.5
augustus	g8478.t1	UniRef100_K12590	5.30E-107	392.5
augustus	g8479.t1	UniRef100_K17605	3.10E-193	679.5
augustus	g8482.t1	UniRef100_K17757	3.70E-160	569.7
augustus	g8485.t1	UniRef100_K02132	3.20E-284	982.2
augustus	g8486.t1	UniRef100_K02868	4.70E-84	315.8
augustus	g8493.t1	UniRef100_K02966	2.10E-75	286.6

augustus	g8494.t1	UniRef100_K14709	4.40E-143	512.7
augustus	g8496.t1	UniRef100_K01253	6.30E-53	213.4
augustus	g8507.t1	UniRef100_K01621	0.00E+00	1425.2
augustus	g8510.t1	UniRef100_K00851	1.00E-78	298.1
augustus	g8514.t1	UniRef100_K03258	1.60E-107	395.2
augustus	g8516.t1	UniRef100_K22193	1.90E-225	786.9
augustus	g8518.t1	UniRef100_K15029	1.20E-268	930.2
augustus	g8519.t1	UniRef100_K14536	0.00E+00	1888.2
augustus	g8523.t1	UniRef100_K11427	1.10E-209	734.6
augustus	g8525.t1	UniRef100_K20180	0.00E+00	1380.9
augustus	g8528.t1	UniRef100_K01412	3.40E-273	945.7
augustus	g8532.t1	UniRef100_K14792	0.00E+00	2791.1
augustus	g8534.t1	UniRef100_K16732	9.20E-295	1017.7
augustus	g8540.t1	UniRef100_K00218	2.30E-42	177.9
augustus	g8541.t1	UniRef100_K01426	5.50E-249	865.1
augustus	g8545.t1	UniRef100_K03252	0.00E+00	1431
augustus	g8546.t1	UniRef100_K22072	1.80E-90	337.4
augustus	g8547.t1	UniRef100_K02937	9.50E-118	428.3
augustus	g8548.t1	UniRef100_K02955	1.90E-64	250.8
augustus	g8549.t1	UniRef100_K10841	0.00E+00	1921.7
augustus	g8550.t1	UniRef100_K00077	7.00E-186	655.2
augustus	g8554.t1	UniRef100_K15321	0.00E+00	1130.9
augustus	g8557.t1	UniRef100_K12856	0.00E+00	4658.6
augustus	g8558.t1	UniRef100_K14324	2.60E-56	223.8
augustus	g8559.t1	UniRef100_K12817	1.10E-130	471.5
augustus	g8560.t1	UniRef100_K12175	2.20E-199	700.3
augustus	g8563.t1	UniRef100_K14213	1.00E-200	704.9
augustus	g8564.t1	UniRef100_K10080	2.50E-162	577
augustus	g8565.t1	UniRef100_K03849	5.50E-262	908.3
augustus	g8574.t1	UniRef100_K01288	4.20E-121	440.3
augustus	g8591.t1	UniRef100_K11559	3.40E-121	439.9
augustus	g8595.t1	UniRef100_K04713	1.50E-181	640.6
augustus	g8599.t1	UniRef100_K17900	1.20E-265	920.6
augustus	g8612.t1	UniRef100_K14573	9.50E-286	987.6
augustus	g8613.t1	UniRef100_K06678	0.00E+00	1614.4
augustus	g8614.t1	UniRef100_K05310	0.00E+00	1382.5
augustus	g8616.t1	UniRef100_K04706	1.70E-153	548.1
augustus	g8617.t1	UniRef100_K20304	1.20E-65	255.4
augustus	g8618.t1	UniRef100_K00026	1.40E-151	541.2
augustus	g8619.t1	UniRef100_K02903	1.10E-45	188.7
augustus	g8621.t1	UniRef100_K00227	8.50E-181	637.9
augustus	g8625.t1	UniRef100_K15015	1.10E-241	841.3
augustus	g8627.t1	UniRef100_K09880	1.20E-114	417.5
augustus	g8628.t1	UniRef100_K18849	8.00E-140	501.5
augustus	g8629.t1	UniRef100_K02893	3.20E-62	243
augustus	g8630.t1	UniRef100_K17780	3.70E-46	188.7
augustus	g8631.t1	UniRef100_K16073	1.20E-286	990.3
augustus	g8632.t1	UniRef100_K12946	2.30E-43	179.5
augustus	g8639.t1	UniRef100_K13519	3.70E-274	948.7
augustus	g8641.t1	UniRef100_K08658	3.50E-140	503.1

augustus	g8642.t1	UniRef100_K17764	3.50E-194	682.6
augustus	g8643.t1	UniRef100_K12479	3.90E-266	922.2
augustus	g8644.t1	UniRef100_K03495	1.70E-287	993.4
augustus	g8645.t1	UniRef100_K13830	0.00E+00	2802.3
augustus	g8648.t1	UniRef100_K01951	1.40E-252	877.1
augustus	g8658.t1	UniRef100_K08165	1.20E-49	202.6
augustus	g8668.t1	UniRef100_K00217	2.50E-57	228
augustus	g8669.t1	UniRef100_K00451	2.30E-189	666.8
augustus	g8673.t1	UniRef100_K11254	9.40E-45	184.5
augustus	g8674.t1	UniRef100_K11253	5.40E-61	238.8
augustus	g8675.t1	UniRef100_K02999	0.00E+00	2806.9
augustus	g8676.t1	UniRef100_K12843	8.90E-227	791.2
augustus	g8681.t1	UniRef100_K02946	9.40E-110	401.4
augustus	g8682.t1	UniRef100_K15201	0.00E+00	1415.6
augustus	g8686.t1	UniRef100_K19985	0.00E+00	1382.5
augustus	g8687.t1	UniRef100_K08052	0.00E+00	4353.5
augustus	g8690.t1	UniRef100_K17275	0.00E+00	1128.6
augustus	g8693.t1	UniRef100_K10590	0.00E+00	2689.8
augustus	g8695.t1	UniRef100_K14684	2.30E-155	553.5
augustus	g8697.t1	UniRef100_K14753	8.40E-159	564.7
augustus	g8699.t1	UniRef100_K12669	1.00E-157	561.2
augustus	g8701.t1	UniRef100_K02883	1.10E-87	327.8
augustus	g8704.t1	UniRef100_K06127	2.70E-146	523.1
augustus	g8705.t1	UniRef100_K00166	1.40E-237	827
augustus	g8706.t1	UniRef100_K10428	1.10E-60	238
augustus	g8707.t1	UniRef100_K11173	8.90E-221	771.2
augustus	g8712.t1	UniRef100_K07542	1.50E-193	680.6
augustus	g8714.t1	UniRef100_K18102	4.70E-254	881.7
augustus	g8715.t1	UniRef100_K10849	4.50E-123	446.4
augustus	g8716.t1	UniRef100_K01230	2.10E-260	902.9
augustus	g8718.t1	UniRef100_K12845	4.60E-60	235.3
augustus	g8719.t1	UniRef100_K01679	9.40E-251	870.9
augustus	g8726.t1	UniRef100_K11547	0.00E+00	1211.8
augustus	g8727.t1	UniRef100_K13343	1.00E-90	339
augustus	g8730.t1	UniRef100_K01641	1.50E-236	823.5
augustus	g8731.t1	UniRef100_K08900	1.90E-258	896.7
augustus	g8737.t1	UniRef100_K00015	1.20E-26	125.9
augustus	g8741.t1	UniRef100_K15280	9.90E-244	847.8
augustus	g8745.t1	UniRef100_K01183	3.20E-148	530.4
augustus	g8746.t1	UniRef100_K15631	0.00E+00	1365.5
augustus	g8748.t1	UniRef100_K13174	2.30E-84	317
augustus	g8749.t1	UniRef100_K07297	4.70E-252	875.2
augustus	g8751.t1	UniRef100_K14708	0.00E+00	1200.3
augustus	g8759.t1	UniRef100_K11153	7.80E-42	176.4
augustus	g8768.t1	UniRef100_K01253	1.70E-53	215.3
augustus	g8771.t1	UniRef100_K11097	1.00E-43	180.6
augustus	g8773.t1	UniRef100_K20059	1.40E-245	853.6
augustus	g8775.t1	UniRef100_K01179	4.30E-122	443.4
augustus	g8781.t1	UniRef100_K15923	2.10E-124	451.8
augustus	g8782.t1	UniRef100_K05658	0.00E+00	2026.9

augustus	g8785.t1	UniRef100_K09123	3.00E-274	949.1
augustus	g8787.t1	UniRef100_K08712	9.00E-277	958.7
augustus	g8791.t1	UniRef100_K05389	3.00E-290	1002.7
augustus	g8792.t1	UniRef100_K21547	6.30E-91	340.1
augustus	g8801.t1	UniRef100_K00799	2.30E-106	390.2
augustus	g8803.t1	UniRef100_K00761	2.70E-104	383.3
augustus	g8804.t1	UniRef100_K06199	7.60E-170	602.1
augustus	g8806.t1	UniRef100_K10777	0.00E+00	1801.9
augustus	g8810.t1	UniRef100_K01756	7.30E-212	741.9
augustus	g8811.t1	UniRef100_K01077	7.10E-265	917.9
augustus	g8814.t1	UniRef100_K02915	9.90E-57	224.2
augustus	g8819.t1	UniRef100_K01183	6.20E-168	595.5
augustus	g8823.t1	UniRef100_K15462	4.20E-162	575.9
augustus	g8824.t1	UniRef100_K00211	1.60E-235	820.1
augustus	g8828.t1	UniRef100_K12188	3.10E-119	433
augustus	g8829.t1	UniRef100_K09503	5.30E-212	741.9
augustus	g8834.t1	UniRef100_K04569	1.60E-125	453.8
augustus	g8835.t1	UniRef100_K02871	3.10E-94	349.4
augustus	g8836.t1	UniRef100_K00365	1.50E-160	570.5
augustus	g8837.t1	UniRef100_K08267	0.00E+00	2438.3
augustus	g8843.t1	UniRef100_K01451	3.70E-56	224.2
augustus	g8844.t1	UniRef100_K01166	1.70E-125	454.5
augustus	g8865.t1	UniRef100_K19654	1.50E-43	181.8
augustus	g8878.t1	UniRef100_K01480	4.50E-168	595.9
augustus	g8880.t1	UniRef100_K22134	2.00E-112	411.4
augustus	g8884.t1	UniRef100_K17647	1.50E-92	344.7
augustus	g8886.t1	UniRef100_K00218	6.20E-51	206.5
augustus	g8894.t1	UniRef100_K08165	1.00E-60	239.6
augustus	g8895.t1	UniRef100_K01752	6.70E-103	380.2
augustus	g8898.t1	UniRef100_K00764	4.20E-263	912.1
augustus	g8899.t1	UniRef100_K00877	5.90E-228	795.4
augustus	g8901.t1	UniRef100_K17871	3.00E-285	985.7
augustus	g8902.t1	UniRef100_K12823	2.00E-251	873.2
augustus	g8903.t1	UniRef100_K21455	2.70E-180	636.3
augustus	g8904.t1	UniRef100_K13205	1.40E-155	554.7
augustus	g8905.t1	UniRef100_K02960	9.90E-75	284.3
augustus	g8906.t1	UniRef100_K02912	2.70E-56	223.4
augustus	g8908.t1	UniRef100_K15371	0.00E+00	1998.4
augustus	g8910.t1	UniRef100_K17769	2.90E-57	226.5
augustus	g8911.t1	UniRef100_K01488	6.80E-124	449.1
augustus	g8912.t1	UniRef100_K06030	0.00E+00	1526.1
augustus	g8914.t1	UniRef100_K19704	2.00E-212	743.8
augustus	g8917.t1	UniRef100_K03140	5.40E-105	386
augustus	g8919.t1	UniRef100_K15306	1.20E-70	271.9
augustus	g8922.t1	UniRef100_K04536	3.80E-158	562.8
augustus	g8924.t1	UniRef100_K01907	0.00E+00	1305.8
augustus	g8925.t1	UniRef100_K00654	0.00E+00	1085.5
augustus	g8927.t1	UniRef100_K19841	4.30E-227	792.3
augustus	g8935.t1	UniRef100_K03316	3.00E-298	1029.6
augustus	g8942.t1	UniRef100_K01705	0.00E+00	1096.6

augustus	g8943.t1	UniRef100_K13303	1.40E-155	555.4
augustus	g8950.t1	UniRef100_K06988	3.70E-62	243.4
augustus	g8951.t1	UniRef100_K01481	9.60E-178	628.6
augustus	g8955.t1	UniRef100_K22134	1.80E-53	215.7
augustus	g8962.t1	UniRef100_K08158	2.00E-64	251.9
augustus	g8966.t1	UniRef100_K00108	5.60E-52	210.7
augustus	g8967.t1	UniRef100_K08192	1.10E-88	332.4
augustus	g8971.t1	UniRef100_K08157	1.70E-76	292
augustus	g8976.t1	UniRef100_K20247	1.20E-204	717.6
augustus	g8977.t1	UniRef100_K09022	5.30E-27	125.6
augustus	g8979.t1	UniRef100_K22276	1.60E-252	876.7
augustus	g8990.t1	UniRef100_K03434	5.10E-121	439.5
augustus	g8991.t1	UniRef100_K14848	1.80E-206	723.8
augustus	g8992.t1	UniRef100_K19564	6.00E-67	260.4
augustus	g8997.t1	UniRef100_K00451	3.60E-139	500
augustus	g9003.t1	UniRef100_K17645	1.30E-81	308.9
augustus	g9004.t1	UniRef100_K03671	1.70E-43	180.3
augustus	g9008.t1	UniRef100_K06965	4.70E-200	702.2
augustus	g9009.t1	UniRef100_K06221	2.70E-36	157.5
augustus	g9020.t1	UniRef100_K02265	1.50E-77	294.3
augustus	g9022.t1	UniRef100_K03854	2.10E-240	836.6
augustus	g9025.t1	UniRef100_K17644	5.60E-41	172.9
augustus	g9027.t1	UniRef100_K00108	6.90E-66	256.9
augustus	g9032.t1	UniRef100_K09964	2.90E-29	133.3
augustus	g9033.t1	UniRef100_K01692	2.20E-73	280.8
augustus	g9035.t1	UniRef100_K06123	1.60E-43	181.8
augustus	g9036.t1	UniRef100_K09831	5.80E-275	951.4
augustus	g9045.t1	UniRef100_K01687	0.00E+00	1088.2
augustus	g9051.t1	UniRef100_K14810	5.40E-272	941.8
augustus	g9056.t1	UniRef100_K18107	2.50E-169	599.7
augustus	g9065.t1	UniRef100_K17741	4.60E-49	200.3
augustus	g9069.t1	UniRef100_K15505	0.00E+00	1802.3
augustus	g9070.t1	UniRef100_K18163	1.50E-157	560.8
augustus	g9071.t1	UniRef100_K02990	3.80E-50	202.6
augustus	g9073.t1	UniRef100_K03507	0.00E+00	1139.8
augustus	g9079.t1	UniRef100_K16028	3.60E-17	94
augustus	g9084.t1	UniRef100_K12622	1.80E-33	146.7
augustus	g9085.t1	UniRef100_K03231	2.40E-86	325.1
augustus	g9086.t1	UniRef100_K01535	0.00E+00	1694.9
augustus	g9090.t1	UniRef100_K07032	3.80E-16	89.7
augustus	g9093.t1	UniRef100_K05275	4.60E-62	243.4
augustus	g9094.t1	UniRef100_K00001	9.30E-58	229.2
augustus	g9096.t1	UniRef100_K05994	6.60E-160	568.9
augustus	g9099.t1	UniRef100_K15920	0.00E+00	1408.3
augustus	g9105.t1	UniRef100_K00601	3.80E-105	386
augustus	g9107.t1	UniRef100_K05349	0.00E+00	1287.7
augustus	g9109.t1	UniRef100_K10675	5.00E-69	266.5
augustus	g9111.t1	UniRef100_K00616	7.80E-176	621.7
augustus	g9114.t1	UniRef100_K13979	1.50E-43	181.8
augustus	g9117.t1	UniRef100_K01265	5.90E-182	642.1

augustus	g9120.t1	UniRef100_K00002	1.50E-136	491.1
augustus	g9124.t1	UniRef100_K14165	8.00E-53	211.8
augustus	g9125.t1	UniRef100_K01050	1.10E-71	276.2
augustus	g9128.t1	UniRef100_K17218	4.60E-243	845.1
augustus	g9134.t1	UniRef100_K06912	3.50E-131	473
augustus	g9143.t1	UniRef100_K08095	1.20E-76	291.6
augustus	g9147.t1	UniRef100_K15502	2.10E-66	258.1
augustus	g9149.t1	UniRef100_K19200	4.40E-211	738.8
augustus	g9165.t1	UniRef100_K22124	8.70E-68	262.3
augustus	g9167.t1	UniRef100_K22121	9.00E-91	339
augustus	g9169.t1	UniRef100_K22119	1.70E-67	261.2
augustus	g9170.t1	UniRef100_K22123	1.20E-61	241.9
augustus	g9172.t1	UniRef100_K01273	2.70E-64	250.8
augustus	g9178.t1	UniRef100_K00128	1.30E-144	518.1
augustus	g9184.t1	UniRef100_K00001	3.80E-59	233.8
augustus	g9185.t1	UniRef100_K14338	0.00E+00	1397.9
augustus	g9189.t1	UniRef100_K00129	1.00E-206	724.5
augustus	g9194.t1	UniRef100_K08141	7.60E-232	808.1
augustus	g9195.t1	UniRef100_K06978	1.10E-202	711.4
augustus	g9196.t1	UniRef100_K03379	2.00E-118	431.4
augustus	g9200.t1	UniRef100_K10438	1.50E-237	827
augustus	g9202.t1	UniRef100_K01183	2.80E-127	460.3
augustus	g9204.t1	UniRef100_K00128	1.20E-116	425.2
augustus	g9206.t1	UniRef100_K18581	3.30E-46	191
augustus	g9210.t1	UniRef100_K11153	1.90E-23	115.2
augustus	g9212.t1	UniRef100_K00001	8.30E-44	183
augustus	g9223.t1	UniRef100_K01054	5.10E-98	362.8
augustus	g9225.t1	UniRef100_K00293	8.30E-170	601.7
augustus	g9229.t1	UniRef100_K00078	2.20E-43	181.4
augustus	g9232.t1	UniRef100_K22134	1.70E-48	199.1
augustus	g9238.t1	UniRef100_K07824	7.50E-64	250
augustus	g9239.t1	UniRef100_K00450	3.40E-87	327
augustus	g9241.t1	UniRef100_K01679	1.80E-181	640.6
augustus	g9252.t1	UniRef100_K08158	2.80E-275	952.6
augustus	g9255.t1	UniRef100_K21159	1.10E-91	342
augustus	g9266.t1	UniRef100_K00354	1.50E-119	434.5
augustus	g9271.t1	UniRef100_K01102	4.70E-201	705.7
augustus	g9272.t1	UniRef100_K00223	1.00E-225	787.7
augustus	g9275.t1	UniRef100_K17742	1.70E-105	387.5
augustus	g9276.t1	UniRef100_K18622	7.00E-111	405.6
augustus	g9278.t1	UniRef100_K01205	0.00E+00	1154
augustus	g9281.t1	UniRef100_K00812	1.50E-50	205.7
augustus	g9292.t1	UniRef100_K08192	3.50E-64	251.1
augustus	g9293.t1	UniRef100_K00480	6.40E-204	715.3
augustus	g9295.t1	UniRef100_K03380	0.00E+00	1121.3
augustus	g9298.t1	UniRef100_K01907	2.50E-102	377.9
augustus	g9302.t1	UniRef100_K01424	1.00E-38	166
augustus	g9304.t1	UniRef100_K14640	0.00E+00	1075.1
augustus	g9310.t1	UniRef100_K15923	0.00E+00	1337.8
augustus	g9311.t1	UniRef100_K19327	0.00E+00	1228.4

augustus	g9314.t1	UniRef100_K00681	4.00E-244	849
augustus	g9315.t1	UniRef100_K01762	1.80E-63	248.4
augustus	g9317.t1	UniRef100_K07565	1.60E-97	360.5
augustus	g9318.t1	UniRef100_K00662	6.50E-75	285.8
augustus	g9319.t1	UniRef100_K15387	1.20E-105	389
augustus	g9320.t1	UniRef100_K07407	2.70E-236	822.8
augustus	g9321.t1	UniRef100_K05275	4.40E-143	512.7
augustus	g9322.t1	UniRef100_K04091	1.60E-200	703.7
augustus	g9323.t1	UniRef100_K22276	9.80E-244	847.4
augustus	g9324.t1	UniRef100_K06634	2.00E-175	620.2
augustus	g9329.t1	UniRef100_K01673	2.60E-79	299.7
augustus	g9330.t1	UniRef100_K03671	1.30E-54	217.2
augustus	g9332.t1	UniRef100_K17741	3.60E-46	190.7
augustus	g9336.t1	UniRef100_K01829	2.60E-21	107.5
augustus	g9338.t1	UniRef100_K11826	3.10E-242	842.4
augustus	g9342.t1	UniRef100_K12274	2.70E-105	386.3
augustus	g9343.t1	UniRef100_K02872	3.30E-101	372.9
augustus	g9345.t1	UniRef100_K00128	1.90E-109	401.4
augustus	g9346.t1	UniRef100_K03676	4.60E-45	185.3
augustus	g9350.t1	UniRef100_K07407	0.00E+00	1407.5
augustus	g9351.t1	UniRef100_K03360	8.80E-195	685.6
augustus	g9352.t1	UniRef100_K03320	8.90E-256	887.5
augustus	g9353.t1	UniRef100_K01056	1.70E-100	370.5
augustus	g9356.t1	UniRef100_K01099	0.00E+00	1494.6
augustus	g9363.t1	UniRef100_K01230	1.40E-247	860.5
augustus	g9367.t1	UniRef100_K01192	0.00E+00	1713.7
augustus	g9374.t1	UniRef100_K08192	1.10E-75	289.3
augustus	g9375.t1	UniRef100_K01488	2.30E-189	666.4
augustus	g9378.t1	UniRef100_K22277	1.90E-191	673.7
augustus	g9382.t1	UniRef100_K13333	0.00E+00	1175.2
augustus	g9383.t1	UniRef100_K16044	2.40E-59	234.6
augustus	g9387.t1	UniRef100_K00108	4.90E-149	533.1
augustus	g9391.t1	UniRef100_K01426	1.80E-218	763.8
augustus	g9393.t1	UniRef100_K00463	4.80E-94	350.1
augustus	g9396.t1	UniRef100_K19386	2.10E-204	716.8
augustus	g9403.t1	UniRef100_K01081	2.90E-226	789.6
augustus	g9407.t1	UniRef100_K05917	7.20E-102	376.3
augustus	g9408.t1	UniRef100_K00108	3.30E-156	557
augustus	g9410.t1	UniRef100_K01487	2.00E-212	743.4
augustus	g9420.t1	UniRef100_K01358	4.10E-98	362.8
augustus	g9421.t1	UniRef100_K01687	0.00E+00	1137.5
augustus	g9424.t1	UniRef100_K06185	0.00E+00	1100.9
augustus	g9426.t1	UniRef100_K05528	3.00E-126	456.8
augustus	g9427.t1	UniRef100_K08137	6.10E-51	207.2
augustus	g9432.t1	UniRef100_K04564	1.60E-98	364.4
augustus	g9436.t1	UniRef100_K03062	6.00E-236	821.6
augustus	g9437.t1	UniRef100_K04649	9.20E-101	371.7
augustus	g9438.t1	UniRef100_K01899	9.00E-166	588.2
augustus	g9439.t1	UniRef100_K07739	0.00E+00	1151
augustus	g9441.t1	UniRef100_K00019	8.30E-51	206.1

augustus	g9446.t1	UniRef100_K01279	2.00E-267	926.8
augustus	g9454.t1	UniRef100_K17741	3.50E-43	180.6
augustus	g9456.t1	UniRef100_K11153	4.60E-37	160.2
augustus	g9457.t1	UniRef100_K11153	1.20E-26	125.9
augustus	g9458.t1	UniRef100_K01762	2.20E-98	364.4
augustus	g9464.t1	UniRef100_K12603	0.00E+00	1160.2
augustus	g9470.t1	UniRef100_K01288	2.50E-175	620.5
augustus	g9472.t1	UniRef100_K11665	0.00E+00	2318.1
augustus	g9478.t1	UniRef100_K20828	2.60E-137	493.8
augustus	g9488.t1	UniRef100_K21403	1.00E-235	820.8
augustus	g9489.t1	UniRef100_K01426	4.40E-41	173.7
augustus	g9499.t1	UniRef100_K01873	0.00E+00	1700.6
augustus	g9502.t1	UniRef100_K00799	2.00E-122	443.7
augustus	g9507.t1	UniRef100_K00637	3.90E-259	899
augustus	g9508.t1	UniRef100_K04079	0.00E+00	1142.1
augustus	g9516.t1	UniRef100_K00141	6.60E-94	349.7
augustus	g9519.t1	UniRef100_K01100	2.30E-138	496.9
augustus	g9525.t1	UniRef100_K01027	3.60E-238	829.3
augustus	g9531.t1	UniRef100_K02685	2.80E-269	932.6
augustus	g9538.t1	UniRef100_K18732	6.80E-37	159.5
augustus	g9553.t1	UniRef100_K02979	1.50E-251	874.4
augustus	g9555.t1	UniRef100_K19787	3.10E-55	221.1
augustus	g9556.t1	UniRef100_K00046	1.40E-41	175.3
augustus	g9561.t1	UniRef100_K06901	2.60E-220	770.4
augustus	g9562.t1	UniRef100_K05610	5.30E-178	630.2
augustus	g9563.t1	UniRef100_K01613	6.70E-242	841.6
augustus	g9564.t1	UniRef100_K17878	5.80E-19	100.1
augustus	g9567.t1	UniRef100_K10601	8.50E-289	998
augustus	g9568.t1	UniRef100_K20177	0.00E+00	1810.8
augustus	g9569.t1	UniRef100_K09313	6.90E-271	938.3
augustus	g9572.t1	UniRef100_K02880	5.40E-88	328.9
augustus	g9574.t1	UniRef100_K21292	6.80E-66	256.9
augustus	g9575.t1	UniRef100_K07078	1.80E-101	373.6
augustus	g9577.t1	UniRef100_K00750	2.60E-224	783.1
augustus	g9579.t1	UniRef100_K01054	1.10E-144	517.7
augustus	g9580.t1	UniRef100_K02021	1.10E-306	1057.4
augustus	g9581.t1	UniRef100_K15559	1.70E-164	583.9
augustus	g9583.t1	UniRef100_K02269	1.20E-14	84
augustus	g9585.t1	UniRef100_K08489	1.60E-153	547.4
augustus	g9588.t1	UniRef100_K01655	2.70E-151	540.8
augustus	g9589.t1	UniRef100_K03108	9.50E-253	877.9
augustus	g9591.t1	UniRef100_K08783	5.90E-245	851.7
augustus	g9592.t1	UniRef100_K00799	8.20E-52	208.4
augustus	g9593.t1	UniRef100_K10981	1.10E-242	844.3
augustus	g9594.t1	UniRef100_K03136	5.70E-179	632.1
augustus	g9596.t1	UniRef100_K06636	0.00E+00	1540
augustus	g9598.t1	UniRef100_K03364	0.00E+00	1117.4
augustus	g9600.t1	UniRef100_K17972	2.50E-105	386.3
augustus	g9602.t1	UniRef100_K20475	0.00E+00	1299.6
augustus	g9603.t1	UniRef100_K03938	7.40E-46	188.3

augustus	g9608.t1	UniRef100_K00058	4.00E-236	822.4
augustus	g9609.t1	UniRef100_K02984	6.00E-130	468.8
augustus	g9610.t1	UniRef100_K02870	2.80E-77	293.1
augustus	g9611.t1	UniRef100_K00761	1.70E-118	430.6
augustus	g9615.t1	UniRef100_K01875	8.70E-231	804.7
augustus	g9616.t1	UniRef100_K11279	7.50E-150	535.4
augustus	g9617.t1	UniRef100_K14951	0.00E+00	2079.3
augustus	g9618.t1	UniRef100_K18532	7.00E-91	338.2
augustus	g9619.t1	UniRef100_K10357	0.00E+00	2622.4
augustus	g9621.t1	UniRef100_K14823	6.70E-135	485.7
augustus	g9625.t1	UniRef100_K19267	5.90E-39	166.4
augustus	g9629.t1	UniRef100_K00128	4.30E-215	752.3
augustus	g9633.t1	UniRef100_K15303	2.20E-108	397.1
augustus	g9636.t1	UniRef100_K03305	6.50E-87	327
augustus	g9637.t1	UniRef100_K00276	0.00E+00	1138.3
augustus	g9641.t1	UniRef100_K00274	1.20E-194	684.9
augustus	g9642.t1	UniRef100_K03379	6.50E-79	300.1
augustus	g9643.t1	UniRef100_K08192	1.10E-55	222.6
augustus	g9651.t1	UniRef100_K09241	1.20E-117	428.7
augustus	g9652.t1	UniRef100_K17742	1.30E-107	394.4
augustus	g9653.t1	UniRef100_K05351	3.40E-144	516.5
augustus	g9654.t1	UniRef100_K17742	1.20E-95	354.8
augustus	g9656.t1	UniRef100_K17742	2.70E-53	214.2
augustus	g9657.t1	UniRef100_K02173	2.20E-20	104.4
augustus	g9659.t1	UniRef100_K09788	4.40E-58	230.3
augustus	g9662.t1	UniRef100_K00799	1.30E-117	427.6
augustus	g9665.t1	UniRef100_K09022	1.70E-20	104
augustus	g9666.t1	UniRef100_K20498	1.20E-159	568.2
augustus	g9673.t1	UniRef100_K15418	0.00E+00	1377.1
augustus	g9683.t1	UniRef100_K01233	1.30E-78	298.1
augustus	g9687.t1	UniRef100_K03379	1.60E-70	272.3
augustus	g9689.t1	UniRef100_K00059	7.90E-34	149.4
augustus	g9697.t1	UniRef100_K11231	0.00E+00	1748
augustus	g9698.t1	UniRef100_K01488	6.70E-173	611.7
augustus	g9699.t1	UniRef100_K01652	8.30E-131	472.6
augustus	g9702.t1	UniRef100_K01279	7.80E-302	1040.8
augustus	g9704.t1	UniRef100_K01279	6.40E-299	1031.2
augustus	g9715.t1	UniRef100_K09252	2.60E-82	311.2
augustus	g9717.t1	UniRef100_K11153	2.70E-34	151.4
augustus	g9720.t1	UniRef100_K01593	9.70E-58	229.6
augustus	g9725.t1	UniRef100_K09885	2.50E-137	493.4
augustus	g9726.t1	UniRef100_K07443	2.80E-58	229.6
augustus	g9727.t1	UniRef100_K10395	0.00E+00	1718
augustus	g9731.t1	UniRef100_K10882	2.00E-171	607.8
augustus	g9733.t1	UniRef100_K08712	0.00E+00	1092
augustus	g9738.t1	UniRef100_K00135	1.20E-204	717.6
augustus	g9739.t1	UniRef100_K00059	1.90E-63	247.7
augustus	g9741.t1	UniRef100_K01426	4.60E-259	898.7
augustus	g9743.t1	UniRef100_K03448	2.30E-242	842.8
augustus	g9745.t1	UniRef100_K17645	9.80E-80	302.8

augustus	g9749.t1	UniRef100_K01866	2.00E-183	647.1
augustus	g9753.t1	UniRef100_K13726	7.00E-165	585.9
augustus	g9759.t1	UniRef100_K01866	5.10E-182	642.1
augustus	g9762.t1	UniRef100_K07192	1.90E-129	468
augustus	g9764.t1	UniRef100_K03932	7.50E-44	183
augustus	g9765.t1	UniRef100_K08192	3.60E-61	241.1
augustus	g9769.t1	UniRef100_K13726	9.60E-168	595.5
augustus	g9771.t1	UniRef100_K09704	1.50E-240	837
augustus	g9774.t1	UniRef100_K12200	0.00E+00	1361.3
augustus	g9780.t1	UniRef100_K01426	1.10E-174	618.2
augustus	g9784.t1	UniRef100_K01784	3.90E-85	320.1
augustus	g9785.t1	UniRef100_K01082	7.80E-177	624.8
augustus	g9795.t1	UniRef100_K14633	7.90E-24	116.3
augustus	g9797.t1	UniRef100_K15303	7.90E-137	491.9
augustus	g9798.t1	UniRef100_K10865	7.40E-275	951.4
augustus	g9800.t1	UniRef100_K01501	3.10E-141	506.9
augustus	g9803.t1	UniRef100_K00001	1.10E-37	162.5
augustus	g9806.t1	UniRef100_K00290	3.40E-46	190.7
augustus	g9808.t1	UniRef100_K03134	1.40E-88	330.9
augustus	g9809.t1	UniRef100_K10949	3.10E-104	382.9
augustus	g9810.t1	UniRef100_K06893	4.20E-11	72.8
augustus	g9812.t1	UniRef100_K01426	6.30E-162	575.9
augustus	g9817.t1	UniRef100_K15303	5.60E-142	508.8
augustus	g9824.t1	UniRef100_K00018	1.00E-96	358.6
augustus	g9825.t1	UniRef100_K00549	8.10E-71	273.1
augustus	g9828.t1	UniRef100_K12343	2.70E-133	479.9
augustus	g9831.t1	UniRef100_K19564	2.20E-69	268.5
augustus	g9833.t1	UniRef100_K14050	7.20E-149	531.9
augustus	g9835.t1	UniRef100_K00001	3.60E-35	154.5
augustus	g9836.t1	UniRef100_K21295	4.40E-144	516.5
augustus	g9842.t1	UniRef100_K01674	2.50E-48	197.6
augustus	g9847.t1	UniRef100_K03800	7.00E-137	492.3
augustus	g9853.t1	UniRef100_K21157	0.00E+00	1770.4
augustus	g9856.t1	UniRef100_K03843	4.50E-210	735.7
augustus	g9858.t1	UniRef100_K20176	0.00E+00	1500.7
augustus	g9859.t1	UniRef100_K20237	0.00E+00	2272.7
augustus	g9861.t1	UniRef100_K07756	6.20E-265	919.5
augustus	g9866.t1	UniRef100_K17402	3.50E-113	412.9
augustus	g9868.t1	UniRef100_K18045	9.50E-103	378.3
augustus	g9873.t1	UniRef100_K10847	1.30E-194	684.1
augustus	g9878.t1	UniRef100_K00480	1.60E-95	355.1
augustus	g9880.t1	UniRef100_K03379	1.10E-106	392.5
augustus	g9888.t1	UniRef100_K12598	0.00E+00	1899.8
augustus	g9889.t1	UniRef100_K02272	5.30E-35	151.4
augustus	g9892.t1	UniRef100_K01580	2.90E-261	906
augustus	g9896.t1	UniRef100_K03241	1.10E-236	824.3
augustus	g9914.t1	UniRef100_K15502	8.80E-103	379.8
augustus	g9918.t1	UniRef100_K15102	4.00E-150	536.2
augustus	g9926.t1	UniRef100_K03929	3.60E-43	181.4
augustus	g9929.t1	UniRef100_K03256	4.40E-302	1041.6

augustus	g9932.t1	UniRef100_K02429	5.40E-243	845.1
augustus	g9933.t1	UniRef100_K14856	0.00E+00	1161.4
augustus	g9939.t1	UniRef100_K08776	8.50E-143	513.1
augustus	g9943.t1	UniRef100_K19806	0.00E+00	1364.4
augustus	g9944.t1	UniRef100_K09967	1.80E-97	360.9
augustus	g9945.t1	UniRef100_K03066	1.10E-180	637.5
augustus	g9946.t1	UniRef100_K07300	0.00E+00	1783.1
augustus	g9949.t1	UniRef100_K20179	0.00E+00	1680.6
augustus	g9951.t1	UniRef100_K08739	0.00E+00	1217.2
augustus	g9964.t1	UniRef100_K00106	0.00E+00	2563.5
augustus	g9965.t1	UniRef100_K14264	5.20E-226	788.5
augustus	g9966.t1	UniRef100_K21358	4.80E-46	190.3
augustus	g9971.t1	UniRef100_K21452	3.70E-165	586.6
augustus	g9973.t1	UniRef100_K14794	0.00E+00	1978
augustus	g9974.t1	UniRef100_K18467	1.20E-99	367.9
augustus	g9982.t1	UniRef100_K13117	0.00E+00	1188.7
augustus	g9986.t1	UniRef100_K18173	2.40E-77	293.9
augustus	g9987.t1	UniRef100_K02906	2.80E-271	939.1
augustus	g9988.t1	UniRef100_K15218	0.00E+00	1354
augustus	g9989.t1	UniRef100_K11558	2.70E-124	449.9
augustus	g9990.t1	UniRef100_K18633	9.40E-32	140.6
augustus	g9992.t1	UniRef100_K00728	0.00E+00	1358.6
augustus	g9993.t1	UniRef100_K07877	1.60E-51	208
augustus	g9997.t1	UniRef100_K17069	2.50E-219	766.5
augustus	g10001.t1	UniRef100_K15560	1.00E-266	924.5
augustus	g10004.t1	UniRef100_K01535	0.00E+00	1556.6
augustus	g10006.t1	UniRef100_K14309	0.00E+00	1824.3
augustus	g10007.t1	UniRef100_K00875	5.90E-88	330.5
augustus	g10011.t1	UniRef100_K00264	0.00E+00	4165.5
augustus	g10025.t1	UniRef100_K00900	0.00E+00	1104.7
augustus	g10026.t1	UniRef100_K11806	1.30E-205	720.7
augustus	g10027.t1	UniRef100_K03011	1.00E-159	568.2
augustus	g10028.t1	UniRef100_K13577	1.50E-153	547.4
augustus	g10030.t1	UniRef100_K01889	4.10E-258	895.2
augustus	g10040.t1	UniRef100_K14328	2.50E-83	315.1
augustus	g10041.t1	UniRef100_K15427	5.40E-197	692.2
augustus	g10042.t1	UniRef100_K07055	2.20E-164	583.6
augustus	g10044.t1	UniRef100_K02608	2.00E-157	560.5
augustus	g10047.t1	UniRef100_K17741	9.10E-66	255.8
augustus	g10053.t1	UniRef100_K06874	3.10E-224	782.7
augustus	g10055.t1	UniRef100_K15326	8.10E-243	844.3
augustus	g10058.t1	UniRef100_K12734	8.80E-91	337.8
augustus	g10066.t1	UniRef100_K09419	6.50E-279	964.9
augustus	g10068.t1	UniRef100_K12737	4.60E-211	739.2
augustus	g10071.t1	UniRef100_K00128	2.10E-263	912.9
augustus	g10074.t1	UniRef100_K03857	6.80E-288	994.2
augustus	g10077.t1	UniRef100_K00415	5.10E-206	722.2
augustus	g10078.t1	UniRef100_K22074	1.70E-137	493.8
augustus	g10079.t1	UniRef100_K03023	0.00E+00	1115.1
augustus	g10091.t1	UniRef100_K02877	3.10E-97	359.4

augustus	g10095.t1	UniRef100_K03021	0.00E+00	2260
augustus	g10096.t1	UniRef100_K10958	1.10E-159	568.2
augustus	g10105.t1	UniRef100_K22062	1.70E-76	291.6
augustus	g10109.t1	UniRef100_K00624	0.00E+00	1469.1
augustus	g10110.t1	UniRef100_K03283	0.00E+00	1180.2
augustus	g10113.t1	UniRef100_K11497	1.40E-134	485.3
augustus	g10114.t1	UniRef100_K10743	5.50E-167	592
augustus	g10116.t1	UniRef100_K07407	6.70E-179	632.1
augustus	g10118.t1	UniRef100_K08141	3.30E-301	1038.5
augustus	g10120.t1	UniRef100_K07407	2.70E-228	796.6
augustus	g10122.t1	UniRef100_K22048	0.00E+00	1376.7
augustus	g10128.t1	UniRef100_K06997	3.30E-105	386.3
augustus	g10129.t1	UniRef100_K11824	0.00E+00	1791.5
augustus	g10130.t1	UniRef100_K18162	2.30E-171	606.7
augustus	g10136.t1	UniRef100_K14403	0.00E+00	1437.6
augustus	g10137.t1	UniRef100_K02154	0.00E+00	1570.8
augustus	g10139.t1	UniRef100_K02896	7.10E-81	305.4
augustus	g10141.t1	UniRef100_K11778	5.70E-181	638.6
augustus	g10143.t1	UniRef100_K02137	4.10E-168	596.7
augustus	g10144.t1	UniRef100_K00698	0.00E+00	1775.4
augustus	g10145.t1	UniRef100_K00381	0.00E+00	2741.5
augustus	g10146.t1	UniRef100_K01807	3.20E-141	506.1
augustus	g10147.t1	UniRef100_K07893	3.80E-107	392.5
augustus	g10148.t1	UniRef100_K14840	1.00E-157	561.6
augustus	g10151.t1	UniRef100_K03937	2.20E-94	350.1
augustus	g10152.t1	UniRef100_K02139	4.00E-36	156.4
augustus	g10154.t1	UniRef100_K02542	0.00E+00	1624.4
augustus	g10155.t1	UniRef100_K10745	3.90E-55	219.5
augustus	g10156.t1	UniRef100_K18327	4.80E-146	522.3
augustus	g10159.t1	UniRef100_K03504	2.80E-88	330.9
augustus	g10160.t1	UniRef100_K10627	1.90E-156	557.4
augustus	g10162.t1	UniRef100_K20305	0.00E+00	1393.6
augustus	g10163.t1	UniRef100_K13106	2.30E-87	327.4
augustus	g10164.t1	UniRef100_K03138	4.40E-258	895.6
augustus	g10165.t1	UniRef100_K13291	0.00E+00	1353.6
augustus	g10167.t1	UniRef100_K00948	7.90E-226	788.1
augustus	g10168.t1	UniRef100_K16066	8.70E-113	412.1
augustus	g10172.t1	UniRef100_K00949	2.00E-49	201.4
augustus	g10173.t1	UniRef100_K03016	2.90E-77	292.7
augustus	g10174.t1	UniRef100_K11375	2.50E-176	623.2
augustus	g10175.t1	UniRef100_K15323	5.10E-115	419.1
augustus	g10179.t1	UniRef100_K12587	6.70E-137	491.9
augustus	g10181.t1	UniRef100_K11094	1.40E-31	141.7
augustus	g10182.t1	UniRef100_K02149	2.70E-114	416.8
augustus	g10183.t1	UniRef100_K12867	0.00E+00	1387.9
augustus	g10186.t1	UniRef100_K00914	0.00E+00	1709.5
augustus	g10187.t1	UniRef100_K02607	3.30E-203	713
augustus	g10188.t1	UniRef100_K15451	0.00E+00	1241.5
augustus	g10189.t1	UniRef100_K15451	0.00E+00	1441.4
augustus	g10190.t1	UniRef100_K02607	4.40E-191	672.5

augustus	g10191.t1	UniRef100_K00914	0.00E+00	1680.6
augustus	g10194.t1	UniRef100_K19828	2.30E-170	603.2
augustus	g10199.t1	UniRef100_K12829	5.30E-250	868.6
augustus	g10200.t1	UniRef100_K15283	7.00E-223	778.1
augustus	g10204.t1	UniRef100_K03380	4.90E-92	344
augustus	g10205.t1	UniRef100_K11407	0.00E+00	1304.3
augustus	g10206.t1	UniRef100_K14820	9.80E-145	518.1
augustus	g10208.t1	UniRef100_K11595	0.00E+00	1156
augustus	g10210.t1	UniRef100_K11676	4.80E-83	313.5
augustus	g10212.t1	UniRef100_K22152	0.00E+00	2931.4
augustus	g10213.t1	UniRef100_K22149	0.00E+00	1098.6
augustus	g10214.t1	UniRef100_K22151	3.80E-253	878.6
augustus	g10215.t1	UniRef100_K08197	1.30E-69	269.2
augustus	g10222.t1	UniRef100_K06004	9.40E-50	203
augustus	g10224.t1	UniRef100_K13210	3.40E-190	669.8
augustus	g10225.t1	UniRef100_K15361	0.00E+00	1589.7
augustus	g10226.t1	UniRef100_K15111	7.70E-119	432.6
augustus	g10231.t1	UniRef100_K15161	6.80E-146	521.9
augustus	g10232.t1	UniRef100_K14684	8.70E-267	924.5
augustus	g10241.t1	UniRef100_K18666	3.30E-94	349.7
augustus	g10247.t1	UniRef100_K11373	0.00E+00	2275.7
augustus	g10249.t1	UniRef100_K08150	7.10E-219	765
augustus	g10250.t1	UniRef100_K05609	9.10E-116	421.4
augustus	g10251.t1	UniRef100_K18221	2.20E-37	161.8
augustus	g10252.t1	UniRef100_K08157	5.70E-295	1018.1
augustus	g10253.t1	UniRef100_K01875	3.10E-264	915.6
augustus	g10255.t1	UniRef100_K08286	5.20E-269	931.8
augustus	g10257.t1	UniRef100_K01533	0.00E+00	1521.1
augustus	g10260.t1	UniRef100_K22211	5.50E-22	109.8
augustus	g10262.t1	UniRef100_K19357	5.40E-213	745.3
augustus	g10270.t1	UniRef100_K01710	1.50E-219	767.3
augustus	g10273.t1	UniRef100_K14846	8.40E-167	591.7
augustus	g10274.t1	UniRef100_K14834	5.20E-304	1048.1
augustus	g10276.t1	UniRef100_K14522	1.20E-89	334.7
augustus	g10279.t1	UniRef100_K07223	8.20E-130	468.4
augustus	g10281.t1	UniRef100_K03781	1.50E-246	857.1
augustus	g10282.t1	UniRef100_K00108	2.90E-64	251.5
augustus	g10285.t1	UniRef100_K20410	0.00E+00	1211.4
augustus	g10288.t1	UniRef100_K14715	2.30E-175	620.2
augustus	g10290.t1	UniRef100_K03361	6.60E-194	682.6
augustus	g10301.t1	UniRef100_K19307	2.30E-111	407.1
augustus	g10302.t1	UniRef100_K11423	0.00E+00	1261.9
augustus	g10305.t1	UniRef100_K13524	3.00E-238	829.3
augustus	g10306.t1	UniRef100_K05012	0.00E+00	1561.2
augustus	g10307.t1	UniRef100_K14767	9.40E-199	698.4
augustus	g10308.t1	UniRef100_K20288	0.00E+00	1235.7
augustus	g10309.t1	UniRef100_K13239	4.40E-132	475.7
augustus	g10310.t1	UniRef100_K03005	5.30E-212	741.9
augustus	g10313.t1	UniRef100_K08269	0.00E+00	1559.7
augustus	g10314.t1	UniRef100_K00819	7.00E-241	837.8

augustus	g10315.t1	UniRef100_K05286	4.00E-299	1031.9
augustus	g10316.t1	UniRef100_K19845	0.00E+00	1199.1
augustus	g10318.t1	UniRef100_K17777	2.00E-41	172.9
augustus	g10319.t1	UniRef100_K14311	0.00E+00	3058.9
augustus	g10320.t1	UniRef100_K13501	0.00E+00	1338.9
augustus	g10326.t1	UniRef100_K04630	8.30E-193	677.9
augustus	g10330.t1	UniRef100_K15456	5.80E-155	552
augustus	g10331.t1	UniRef100_K13525	0.00E+00	1498
augustus	g10335.t1	UniRef100_K04709	1.30E-216	757.3
augustus	g10337.t1	UniRef100_K12615	0.00E+00	1092.8
augustus	g10339.t1	UniRef100_K15255	2.20E-262	910.2
augustus	g10341.t1	UniRef100_K10609	0.00E+00	1405.6
augustus	g10343.t1	UniRef100_K04728	0.00E+00	4142
augustus	g10344.t1	UniRef100_K14310	0.00E+00	3004.9
augustus	g10347.t1	UniRef100_K05658	0.00E+00	2127.1
augustus	g10351.t1	UniRef100_K01619	1.90E-116	423.7
augustus	g10354.t1	UniRef100_K18660	1.90E-143	514.6
augustus	g10355.t1	UniRef100_K01915	1.40E-42	179.1
augustus	g10356.t1	UniRef100_K00218	2.80E-27	127.9
augustus	g10364.t1	UniRef100_K11397	0.00E+00	1167.9
augustus	g10365.t1	UniRef100_K12041	7.10E-285	984.6
augustus	g10366.t1	UniRef100_K07952	7.50E-112	408.3
augustus	g10367.t1	UniRef100_K03678	1.90E-126	456.8
augustus	g10368.t1	UniRef100_K12863	1.30E-42	178.3
augustus	g10369.t1	UniRef100_K00854	8.90E-290	1000.7
augustus	g10371.t1	UniRef100_K11414	2.30E-36	158.3
augustus	g10373.t1	UniRef100_K02365	0.00E+00	3027.3
augustus	g10375.t1	UniRef100_K00107	5.10E-251	871.7
augustus	g10379.t1	UniRef100_K20456	0.00E+00	2215.3
augustus	g10380.t1	UniRef100_K01068	6.70E-112	409.1
augustus	g10384.t1	UniRef100_K03635	2.40E-78	296.6
augustus	g10387.t1	UniRef100_K12586	1.70E-165	587
augustus	g10389.t1	UniRef100_K00799	8.90E-86	321.6
augustus	g10390.t1	UniRef100_K00799	7.10E-64	248.8
augustus	g10394.t1	UniRef100_K12780	2.20E-66	258.8
augustus	g10396.t1	UniRef100_K14293	0.00E+00	1580.8
augustus	g10398.t1	UniRef100_K00850	0.00E+00	1480.3
augustus	g10399.t1	UniRef100_K15134	2.10E-268	929.9
augustus	g10402.t1	UniRef100_K08257	2.80E-243	846.3
augustus	g10403.t1	UniRef100_K08513	1.00E-31	141
augustus	g10404.t1	UniRef100_K12659	5.90E-237	825.1
augustus	g10411.t1	UniRef100_K03235	0.00E+00	1889
augustus	g10412.t1	UniRef100_K16675	2.90E-297	1025.8
augustus	g10414.t1	UniRef100_K01079	1.50E-247	860.1
augustus	g10417.t1	UniRef100_K00390	9.10E-155	551.2
augustus	g10418.t1	UniRef100_K15450	7.50E-137	491.9
augustus	g10421.t1	UniRef100_K00589	5.80E-254	881.7
augustus	g10423.t1	UniRef100_K01230	2.30E-274	949.5
augustus	g10425.t1	UniRef100_K09142	6.20E-168	595.1
augustus	g10426.t1	UniRef100_K03644	1.90E-219	766.5

augustus	g10428.t1	UniRef100_K01469	0.00E+00	2378.2
augustus	g10433.t1	UniRef100_K15303	1.20E-171	607.4
augustus	g10435.t1	UniRef100_K01673	2.00E-34	150.6
augustus	g10436.t1	UniRef100_K16261	1.60E-296	1023.1
augustus	g10438.t1	UniRef100_K11369	2.80E-283	979.2
augustus	g10444.t1	UniRef100_K17759	2.00E-104	383.6
augustus	g10448.t1	UniRef100_K11246	3.90E-158	562.4
augustus	g10450.t1	UniRef100_K00281	0.00E+00	1882.1
augustus	g10451.t1	UniRef100_K14308	1.70E-138	497.3
augustus	g10452.t1	UniRef100_K03952	3.80E-79	299.7
augustus	g10455.t1	UniRef100_K14526	2.10E-79	300.1
augustus	g10458.t1	UniRef100_K00287	7.80E-41	172.6
augustus	g10459.t1	UniRef100_K03035	7.20E-235	818.1
augustus	g10460.t1	UniRef100_K14818	5.90E-204	715.3
augustus	g10462.t1	UniRef100_K07152	9.80E-112	408.3
augustus	g10464.t1	UniRef100_K00866	0.00E+00	1080.9
augustus	g10465.t1	UniRef100_K06867	2.40E-70	271.9
augustus	g10466.t1	UniRef100_K20872	6.10E-230	802.4
augustus	g10468.t1	UniRef100_K07759	3.10E-125	453.8
augustus	g10475.t1	UniRef100_K22132	1.80E-259	899.8
augustus	g10479.t1	UniRef100_K06888	1.00E-98	366.3
augustus	g10480.t1	UniRef100_K10416	1.00E-247	860.9
augustus	g10483.t1	UniRef100_K16945	8.40E-165	585.1
augustus	g10484.t1	UniRef100_K06890	1.20E-137	494.2
augustus	g10485.t1	UniRef100_K04709	1.60E-215	753.8
augustus	g10486.t1	UniRef100_K00666	4.40E-87	327.4
augustus	g10487.t1	UniRef100_K05528	7.50E-183	644.8
augustus	g10488.t1	UniRef100_K00276	0.00E+00	1300
augustus	g10494.t1	UniRef100_K08141	7.70E-277	957.6
augustus	g10498.t1	UniRef100_K12849	4.90E-101	372.5
augustus	g10499.t1	UniRef100_K02433	4.70E-252	875.2
augustus	g10505.t1	UniRef100_K07342	1.60E-29	133.3
augustus	g10506.t1	UniRef100_K06999	2.50E-29	134.4
augustus	g10510.t1	UniRef100_K18182	2.90E-53	212.6
augustus	g10513.t1	UniRef100_K19791	2.90E-56	224.9
augustus	g10517.t1	UniRef100_K00547	1.80E-111	407.5
augustus	g10529.t1	UniRef100_K04486	7.30E-137	491.9
augustus	g10530.t1	UniRef100_K11484	0.00E+00	1501.1
augustus	g10532.t1	UniRef100_K00773	1.60E-231	806.6
augustus	g10534.t1	UniRef100_K02962	2.20E-64	250.4
augustus	g10535.t1	UniRef100_K20827	1.70E-120	437.2
augustus	g10536.t1	UniRef100_K20521	3.90E-252	876.7
augustus	g10543.t1	UniRef100_K19001	0.00E+00	1316.6
augustus	g10544.t1	UniRef100_K15437	1.80E-158	563.9
augustus	g10545.t1	UniRef100_K10908	0.00E+00	2197.5
augustus	g10547.t1	UniRef100_K01514	3.70E-178	629.4
augustus	g10550.t1	UniRef100_K17741	1.90E-25	121.7
augustus	g10552.t1	UniRef100_K00344	5.90E-106	389
augustus	g10553.t1	UniRef100_K01784	2.60E-192	676.4
augustus	g10555.t1	UniRef100_K14776	0.00E+00	1194.1

augustus	g10570.t1	UniRef100_K07511	1.90E-119	433.7
augustus	g10574.t1	UniRef100_K14312	0.00E+00	2489.1
augustus	g10575.t1	UniRef100_K10251	1.20E-172	610.9
augustus	g10577.t1	UniRef100_K00965	3.90E-209	732.3
augustus	g10579.t1	UniRef100_K07023	5.40E-126	455.7
augustus	g10582.t1	UniRef100_K00293	3.40E-194	683.3
augustus	g10583.t1	UniRef100_K08176	0.00E+00	1149.8
augustus	g10584.t1	UniRef100_K00641	3.00E-264	915.6
augustus	g10585.t1	UniRef100_K12879	0.00E+00	3092
augustus	g10587.t1	UniRef100_K11244	4.80E-70	270
augustus	g10589.t1	UniRef100_K01771	2.90E-168	596.3
augustus	g10591.t1	UniRef100_K21989	0.00E+00	1490.7
augustus	g10610.t1	UniRef100_K08869	4.30E-268	928.7
augustus	g10612.t1	UniRef100_K19639	1.60E-59	235
augustus	g10615.t1	UniRef100_K05824	8.50E-192	674.5
augustus	g10624.t1	UniRef100_K20069	9.70E-89	331.6
augustus	g10625.t1	UniRef100_K03239	1.40E-163	580.9
augustus	g10626.t1	UniRef100_K11153	8.60E-38	162.9
augustus	g10627.t1	UniRef100_K20246	0.00E+00	1473.8
augustus	g10629.t1	UniRef100_K19639	1.30E-57	228.8
augustus	g10631.t1	UniRef100_K15923	0.00E+00	1154.8
augustus	g10637.t1	UniRef100_K01951	3.20E-72	276.9
augustus	g10645.t1	UniRef100_K12818	0.00E+00	2036.9
augustus	g10652.t1	UniRef100_K06889	4.90E-119	432.6
augustus	g10654.t1	UniRef100_K06889	1.30E-84	318.2
augustus	g10658.t1	UniRef100_K00121	5.80E-199	698.4
augustus	g10659.t1	UniRef100_K01070	1.50E-129	467.6
augustus	g10660.t1	UniRef100_K03396	4.00E-84	315.8
augustus	g10661.t1	UniRef100_K00121	7.80E-58	229.9
augustus	g10666.t1	UniRef100_K00276	9.40E-174	615.5
augustus	g10669.t1	UniRef100_K22134	5.20E-55	220.7
augustus	g10672.t1	UniRef100_K20984	0.00E+00	1286.9
augustus	g10676.t1	UniRef100_K15317	0.00E+00	1494.2
augustus	g10678.t1	UniRef100_K01760	4.50E-125	453
augustus	g10682.t1	UniRef100_K20724	1.90E-124	450.3
augustus	g10683.t1	UniRef100_K01110	9.90E-140	502.3
augustus	g10693.t1	UniRef100_K01183	0.00E+00	1417.1
augustus	g10697.t1	UniRef100_K01568	2.80E-258	896
augustus	g10699.t1	UniRef100_K13993	7.10E-20	102.4
augustus	g10701.t1	UniRef100_K03000	7.40E-28	128.3
augustus	g10704.t1	UniRef100_K00079	6.00E-22	109.8
augustus	g10710.t1	UniRef100_K15564	1.70E-179	634
augustus	g10711.t1	UniRef100_K01074	4.60E-152	542.3
augustus	g10712.t1	UniRef100_K14775	9.60E-154	548.1
augustus	g10717.t1	UniRef100_K20042	0.00E+00	1144.4
augustus	g10718.t1	UniRef100_K08254	2.20E-81	308.1
augustus	g10719.t1	UniRef100_K06679	4.90E-187	659.8
augustus	g10721.t1	UniRef100_K20039	3.00E-222	776.2
augustus	g10723.t1	UniRef100_K03186	4.30E-90	335.9
augustus	g10729.t1	UniRef100_K01288	1.30E-167	595.1

augustus	g10731.t1	UniRef100_K11592	0.00E+00	2344.3
augustus	g10733.t1	UniRef100_K17675	0.00E+00	1224.2
augustus	g10734.t1	UniRef100_K08515	4.10E-110	402.5
augustus	g10738.t1	UniRef100_K08299	9.30E-37	159.1
augustus	g10742.t1	UniRef100_K00009	3.00E-206	722.6
augustus	g10744.t1	UniRef100_K01810	1.10E-303	1047
augustus	g10745.t1	UniRef100_K13303	0.00E+00	1138.3
augustus	g10746.t1	UniRef100_K17302	0.00E+00	1252.7
augustus	g10754.t1	UniRef100_K00306	2.70E-159	567
augustus	g10759.t1	UniRef100_K05913	1.90E-30	137.5
augustus	g10760.t1	UniRef100_K01182	1.80E-154	551.2
augustus	g10761.t1	UniRef100_K00833	2.70E-52	211.5
augustus	g10762.t1	UniRef100_K08141	3.20E-126	457.2
augustus	g10766.t1	UniRef100_K08197	2.80E-280	969.1
augustus	g10767.t1	UniRef100_K17066	0.00E+00	1077.8
augustus	g10768.t1	UniRef100_K01233	1.00E-84	318.2
augustus	g10771.t1	UniRef100_K15321	0.00E+00	1937.9
augustus	g10773.t1	UniRef100_K01738	1.50E-99	367.9
augustus	g10775.t1	UniRef100_K19245	3.40E-180	636
augustus	g10777.t1	UniRef100_K00598	1.20E-140	504.2
augustus	g10779.t1	UniRef100_K01207	4.10E-254	882.1
augustus	g10781.t1	UniRef100_K12373	0.00E+00	1088.2
augustus	g10786.t1	UniRef100_K06015	3.70E-150	537
augustus	g10794.t1	UniRef100_K08157	3.90E-183	646.4
augustus	g10795.t1	UniRef100_K00344	4.20E-98	363.2
augustus	g10797.t1	UniRef100_K16261	6.50E-252	875.2
augustus	g10799.t1	UniRef100_K01279	0.00E+00	1175.2
augustus	g10800.t1	UniRef100_K03448	3.50E-255	885.6
augustus	g10801.t1	UniRef100_K19245	8.30E-56	223
augustus	g10802.t1	UniRef100_K12957	1.50E-45	188.7
augustus	g10806.t1	UniRef100_K01563	1.60E-74	284.6
augustus	g10809.t1	UniRef100_K20743	7.20E-76	288.9
augustus	g10810.t1	UniRef100_K01225	1.40E-214	750.7
augustus	g10814.t1	UniRef100_K15924	1.00E-72	279.3
augustus	g10817.t1	UniRef100_K00480	8.20E-214	748
augustus	g10827.t1	UniRef100_K01253	2.20E-82	311.2
augustus	g10829.t1	UniRef100_K00108	5.30E-51	207.6
augustus	g10832.t1	UniRef100_K01953	6.10E-214	749.2
augustus	g10840.t1	UniRef100_K08726	8.60E-91	339
augustus	g10841.t1	UniRef100_K20550	6.90E-54	216.9
augustus	g10850.t1	UniRef100_K22152	0.00E+00	2468.3
augustus	g10852.t1	UniRef100_K05658	1.30E-216	758.8
augustus	g10853.t1	UniRef100_K22151	1.90E-220	770
augustus	g10854.t1	UniRef100_K22150	1.60E-134	483.8
augustus	g10855.t1	UniRef100_K08197	2.30E-58	231.9
augustus	g10860.t1	UniRef100_K06978	8.10E-94	349.7
augustus	g10871.t1	UniRef100_K01684	1.10E-173	614.4
augustus	g10874.t1	UniRef100_K01426	1.20E-76	292.4
augustus	g10882.t1	UniRef100_K01235	9.80E-308	1060.8
augustus	g10883.t1	UniRef100_K15924	4.00E-70	270.8

augustus	g10884.t1	UniRef100_K08141	1.10E-179	634.8
augustus	g10888.t1	UniRef100_K08712	4.00E-272	943.3
augustus	g10892.t1	UniRef100_K01480	9.20E-204	714.5
augustus	g10893.t1	UniRef100_K19564	5.70E-242	841.6
augustus	g10895.t1	UniRef100_K01181	6.80E-142	508.8
augustus	g10908.t1	UniRef100_K19823	6.70E-126	456.1
augustus	g10914.t1	UniRef100_K15394	0.00E+00	2691
augustus	g10915.t1	UniRef100_K15385	2.70E-50	204.5
augustus	g10916.t1	UniRef100_K02510	5.30E-29	133.3
augustus	g10918.t1	UniRef100_K14454	8.50E-166	588.6
augustus	g10920.t1	UniRef100_K02510	3.20E-27	127.5
augustus	g10922.t1	UniRef100_K08157	3.90E-55	221.1
augustus	g10923.t1	UniRef100_K01915	3.30E-162	576.2
augustus	g10924.t1	UniRef100_K01738	8.20E-90	335.5
augustus	g10926.t1	UniRef100_K00799	4.90E-45	186
augustus	g10927.t1	UniRef100_K01130	9.50E-193	678.3
augustus	g10930.t1	UniRef100_K08139	1.10E-56	226.1
augustus	g10933.t1	UniRef100_K01560	3.90E-45	186.8
augustus	g10934.t1	UniRef100_K17741	2.30E-29	134.8
augustus	g10938.t1	UniRef100_K07407	3.00E-102	377.5
augustus	g10945.t1	UniRef100_K15279	5.70E-157	558.9
augustus	g10946.t1	UniRef100_K21292	1.10E-78	299.3
augustus	g10949.t1	UniRef100_K01811	0.00E+00	1355.5
augustus	g10950.t1	UniRef100_K08141	2.90E-255	885.9
augustus	g10961.t1	UniRef100_K04125	2.50E-66	257.7
augustus	g10963.t1	UniRef100_K01183	2.50E-157	560.5
augustus	g10970.t1	UniRef100_K00130	1.50E-279	966.5
augustus	g10971.t1	UniRef100_K00108	2.40E-169	600.5
augustus	g10972.t1	UniRef100_K22148	0.00E+00	7227.9
augustus	g10974.t1	UniRef100_K10531	5.20E-273	944.9
augustus	g10981.t1	UniRef100_K02728	1.30E-131	474.2
augustus	g10983.t1	UniRef100_K15148	2.10E-97	360.5
augustus	g10995.t1	UniRef100_K00966	9.00E-183	644.8
augustus	g10996.t1	UniRef100_K15272	6.10E-167	592.4
augustus	g10997.t1	UniRef100_K03854	1.90E-159	567.4
augustus	g11000.t1	UniRef100_K09291	0.00E+00	1508.8
augustus	g11001.t1	UniRef100_K14805	0.00E+00	1090.9
augustus	g11008.t1	UniRef100_K19267	1.40E-67	261.5
augustus	g11015.t1	UniRef100_K08158	1.40E-119	435.6
augustus	g11018.t1	UniRef100_K00480	2.40E-86	324.7
augustus	g11020.t1	UniRef100_K07045	1.10E-49	202.2
augustus	g11021.t1	UniRef100_K15303	3.00E-111	406.8
augustus	g11025.t1	UniRef100_K12957	1.50E-41	175.3
augustus	g11031.t1	UniRef100_K00059	8.70E-38	162.5
augustus	g11032.t1	UniRef100_K01426	5.80E-299	1031.2
augustus	g11034.t1	UniRef100_K08711	0.00E+00	1343.2
augustus	g11036.t1	UniRef100_K03782	0.00E+00	1421
augustus	g11048.t1	UniRef100_K01444	1.80E-144	517.3
augustus	g11050.t1	UniRef100_K03361	0.00E+00	1344.3
augustus	g11055.t1	UniRef100_K08157	9.40E-181	638.3

augustus	g11057.t1	UniRef100_K14262	0.00E+00	1507.7
augustus	g11064.t1	UniRef100_K05292	0.00E+00	1082.8
augustus	g11065.t1	UniRef100_K01417	0.00E+00	1127.9
augustus	g11075.t1	UniRef100_K20221	0.00E+00	1994.2
augustus	g11078.t1	UniRef100_K06123	8.20E-131	471.9
augustus	g11080.t1	UniRef100_K14816	1.40E-228	797.3
augustus	g11083.t1	UniRef100_K03978	3.10E-140	503.4
augustus	g11087.t1	UniRef100_K19572	4.60E-239	832.4
augustus	g11094.t1	UniRef100_K06699	0.00E+00	3571.2
augustus	g11103.t1	UniRef100_K01424	2.10E-162	577
augustus	g11104.t1	UniRef100_K17795	6.80E-75	285.4
augustus	g11106.t1	UniRef100_K09935	4.30E-74	282.7
augustus	g11107.t1	UniRef100_K17804	1.50E-211	740.7
augustus	g11108.t1	UniRef100_K02948	2.20E-114	416.8
augustus	g11110.t1	UniRef100_K19788	5.60E-213	745
augustus	g11112.t1	UniRef100_K02948	3.10E-266	923.7
augustus	g11113.t1	UniRef100_K16261	5.10E-290	1001.5
augustus	g11116.t1	UniRef100_K07297	6.80E-138	495.4
augustus	g11118.t1	UniRef100_K21440	5.20E-270	936
augustus	g11121.t1	UniRef100_K07297	3.90E-97	359.8
augustus	g11122.t1	UniRef100_K08900	7.10E-239	831.2
augustus	g11135.t1	UniRef100_K04718	2.60E-235	819.7
augustus	g11136.t1	UniRef100_K20792	0.00E+00	1405.6
augustus	g11138.t1	UniRef100_K20465	3.00E-199	699.5
augustus	g11139.t1	UniRef100_K01624	1.90E-185	653.7
augustus	g11144.t1	UniRef100_K11419	2.70E-106	390.2
augustus	g11146.t1	UniRef100_K17301	0.00E+00	1729.1
augustus	g11147.t1	UniRef100_K12877	1.60E-66	257.7
augustus	g11149.t1	UniRef100_K15430	2.70E-208	729.6
augustus	g11150.t1	UniRef100_K14530	5.80E-136	488.8
augustus	g11151.t1	UniRef100_K06867	3.50E-100	369.4
augustus	g11152.t1	UniRef100_K14679	0.00E+00	1392.5
augustus	g11154.t1	UniRef100_K02864	1.80E-159	567
augustus	g11155.t1	UniRef100_K03065	2.10E-213	746.5
augustus	g11157.t1	UniRef100_K14824	0.00E+00	1248
augustus	g11158.t1	UniRef100_K19314	1.60E-29	134.4
augustus	g11167.t1	UniRef100_K01081	1.40E-85	322.8
augustus	g11170.t1	UniRef100_K01455	5.90E-237	824.7
augustus	g11175.t1	UniRef100_K14454	1.10E-122	444.9
augustus	g11178.t1	UniRef100_K00012	0.00E+00	1144
augustus	g11187.t1	UniRef100_K01426	9.10E-45	186.8
augustus	g11188.t1	UniRef100_K01426	5.00E-138	496.1
augustus	g11190.t1	UniRef100_K00981	1.70E-219	766.9
augustus	g11192.t1	UniRef100_K07513	6.70E-215	751.5
augustus	g11200.t1	UniRef100_K01536	0.00E+00	1813.1
augustus	g11205.t1	UniRef100_K00863	1.30E-298	1030
augustus	g11206.t1	UniRef100_K01808	2.60E-73	279.6
augustus	g11208.t1	UniRef100_K12859	1.80E-159	568.5
augustus	g11209.t1	UniRef100_K03457	0.00E+00	1728.8
augustus	g11215.t1	UniRef100_K20247	1.00E-216	757.7

augustus	g11216.t1	UniRef100_K11121	5.20E-202	709.9
augustus	g11217.t1	UniRef100_K01279	8.00E-171	605.5
augustus	g11221.t1	UniRef100_K00026	2.10E-169	600.1
augustus	g11222.t1	UniRef100_K14950	0.00E+00	2381.7
augustus	g11238.t1	UniRef100_K17623	3.70E-120	436
augustus	g11239.t1	UniRef100_K05972	1.60E-126	457.6
augustus	g11248.t1	UniRef100_K00949	2.90E-127	459.9
augustus	g11249.t1	UniRef100_K08496	1.00E-119	434.5
augustus	g11252.t1	UniRef100_K03305	0.00E+00	1135.6
augustus	g11253.t1	UniRef100_K08158	2.70E-61	241.5
augustus	g11256.t1	UniRef100_K05882	5.80E-43	180.3
augustus	g11258.t1	UniRef100_K01652	0.00E+00	1218.4
augustus	g11263.t1	UniRef100_K00108	9.90E-205	718.4
augustus	g11269.t1	UniRef100_K06889	6.10E-36	156.4
augustus	g11272.t1	UniRef100_K12957	1.40E-58	231.9
augustus	g11277.t1	UniRef100_K00864	6.00E-253	878.2
augustus	g11279.t1	UniRef100_K00111	0.00E+00	1167.1
augustus	g11280.t1	UniRef100_K18549	4.90E-72	276.9
augustus	g11282.t1	UniRef100_K07077	3.40E-211	739.6
augustus	g11284.t1	UniRef100_K01114	0.00E+00	1094
augustus	g11294.t1	UniRef100_K03781	2.70E-245	852.8
augustus	g11295.t1	UniRef100_K00387	0.00E+00	1078.5
augustus	g11296.t1	UniRef100_K01673	6.00E-21	105.9
augustus	g11297.t1	UniRef100_K01821	6.20E-24	115.5
augustus	g11300.t1	UniRef100_K17743	3.10E-127	459.9
augustus	g11301.t1	UniRef100_K17100	0.00E+00	1209.5
augustus	g11302.t1	UniRef100_K00008	1.90E-177	627.1
augustus	g11306.t1	UniRef100_K08141	1.30E-70	272.7
augustus	g11307.t1	UniRef100_K19692	0.00E+00	1097
augustus	g11308.t1	UniRef100_K00480	3.50E-76	290.8
augustus	g11309.t1	UniRef100_K01687	0.00E+00	1094.7
augustus	g11318.t1	UniRef100_K10881	6.00E-36	155.6
augustus	g11319.t1	UniRef100_K00927	2.20E-221	773.1
augustus	g11320.t1	UniRef100_K13348	1.00E-102	378.3
augustus	g11325.t1	UniRef100_K00463	9.80E-203	711.1
augustus	g11326.t1	UniRef100_K01556	3.10E-238	829.3
augustus	g11328.t1	UniRef100_K18123	1.50E-158	563.9
augustus	g11329.t1	UniRef100_K18338	5.70E-173	612.1
augustus	g11331.t1	UniRef100_K12763	1.30E-279	967.2
augustus	g11336.t1	UniRef100_K01908	0.00E+00	1302
augustus	g11338.t1	UniRef100_K00344	1.00E-101	375.2
augustus	g11340.t1	UniRef100_K07466	0.00E+00	1120.9
augustus	g11343.t1	UniRef100_K20352	1.20E-103	381.3
augustus	g11349.t1	UniRef100_K01288	0.00E+00	1131.3
augustus	g11357.t1	UniRef100_K12349	0.00E+00	1258.4
augustus	g11367.t1	UniRef100_K07432	1.40E-89	334.3
augustus	g11368.t1	UniRef100_K17413	7.00E-190	668.3
augustus	g11370.t1	UniRef100_K01182	2.40E-178	630.6
augustus	g11373.t1	UniRef100_K07430	1.40E-126	458.4
augustus	g11374.t1	UniRef100_K20984	0.00E+00	1418.7

augustus	g11376.t1	UniRef100_K17645	1.40E-56	225.7
augustus	g11382.t1	UniRef100_K00799	2.20E-118	429.9
augustus	g11384.t1	UniRef100_K01179	1.40E-89	334.7
augustus	g11387.t1	UniRef100_K21802	4.90E-97	360.1
augustus	g11388.t1	UniRef100_K00450	1.90E-106	391
augustus	g11392.t1	UniRef100_K17819	7.90E-253	877.9
augustus	g11393.t1	UniRef100_K03679	4.20E-154	549.3
augustus	g11396.t1	UniRef100_K00645	1.00E-30	139.4
augustus	g11397.t1	UniRef100_K20301	4.00E-81	306.2
augustus	g11399.t1	UniRef100_K14300	0.00E+00	2155.6
augustus	g11405.t1	UniRef100_K03238	1.70E-113	414.5
augustus	g11409.t1	UniRef100_K00799	2.70E-54	217.6
augustus	g11411.t1	UniRef100_K01698	1.60E-207	726.9
augustus	g11412.t1	UniRef100_K17787	1.80E-106	390.6
augustus	g11413.t1	UniRef100_K02879	9.50E-25	119
augustus	g11414.t1	UniRef100_K14774	0.00E+00	1064.3
augustus	g11415.t1	UniRef100_K03249	6.70E-161	572
augustus	g11416.t1	UniRef100_K14817	1.10E-59	235
augustus	g11418.t1	UniRef100_K12847	4.10E-286	988.4
augustus	g11419.t1	UniRef100_K14555	0.00E+00	1631.3
augustus	g11421.t1	UniRef100_K07019	2.20E-207	726.9
augustus	g11429.t1	UniRef100_K10752	2.60E-180	637.1
augustus	g11430.t1	UniRef100_K12618	0.00E+00	2103.2
augustus	g11431.t1	UniRef100_K12948	6.80E-27	126.3
augustus	g11432.t1	UniRef100_K08596	2.20E-221	774.6
augustus	g11433.t1	UniRef100_K14793	8.30E-211	738.4
augustus	g11434.t1	UniRef100_K02734	1.20E-101	374.8
augustus	g11435.t1	UniRef100_K11594	5.40E-250	868.6
augustus	g11444.t1	UniRef100_K00074	4.40E-156	555.8
augustus	g11445.t1	UniRef100_K10997	0.00E+00	1542.7
augustus	g11447.t1	UniRef100_K08956	0.00E+00	1613.2
augustus	g11449.t1	UniRef100_K03008	2.90E-58	229.6
augustus	g11450.t1	UniRef100_K12624	5.90E-36	155.2
augustus	g11452.t1	UniRef100_K14863	1.40E-255	886.7
augustus	g11453.t1	UniRef100_K00693	0.00E+00	1362.1
augustus	g11456.t1	UniRef100_K10246	3.90E-166	589.3
augustus	g11457.t1	UniRef100_K02264	6.60E-61	238.4
augustus	g11459.t1	UniRef100_K14845	1.40E-179	634
augustus	g11460.t1	UniRef100_K08838	8.40E-281	971.1
augustus	g11466.t1	UniRef100_K12161	1.70E-50	203.4
augustus	g11467.t1	UniRef100_K11374	0.00E+00	1525
augustus	g11470.t1	UniRef100_K05692	4.50E-213	745.3
augustus	g11473.t1	UniRef100_K02931	9.60E-186	654.4
augustus	g11475.t1	UniRef100_K11518	1.60E-191	673.7
augustus	g11476.t1	UniRef100_K19030	6.60E-248	861.7
augustus	g11477.t1	UniRef100_K03083	6.40E-225	784.6
augustus	g11478.t1	UniRef100_K12882	0.00E+00	1321.6
augustus	g11480.t1	UniRef100_K03177	4.50E-218	762.3
augustus	g11482.t1	UniRef100_K07573	1.60E-33	147.9
augustus	g11483.t1	UniRef100_K05665	3.60E-212	744.2

augustus	g11488.t1	UniRef100_K18724	5.90E-89	332.4
augustus	g11489.t1	UniRef100_K11675	1.30E-276	957.2
augustus	g11490.t1	UniRef100_K09829	5.50E-118	428.7
augustus	g11493.t1	UniRef100_K10755	1.50E-189	667.2
augustus	g11495.t1	UniRef100_K13989	1.90E-117	427.2
augustus	g11497.t1	UniRef100_K06210	2.30E-133	480.3
augustus	g11507.t1	UniRef100_K11131	3.90E-205	719.5
augustus	g11511.t1	UniRef100_K14798	2.30E-180	636.7
augustus	g11512.t1	UniRef100_K14539	5.10E-304	1048.1
augustus	g11513.t1	UniRef100_K14835	7.70E-249	865.1
augustus	g11516.t1	UniRef100_K00029	1.60E-306	1056.6
augustus	g11521.t1	UniRef100_K22324	5.30E-32	144.1
augustus	g11522.t1	UniRef100_K02930	1.50E-185	653.7
augustus	g11525.t1	UniRef100_K02358	3.00E-240	835.9
augustus	g11526.t1	UniRef100_K09584	8.90E-171	604.7
augustus	g11527.t1	UniRef100_K19765	3.90E-17	92
augustus	g11531.t1	UniRef100_K20028	1.90E-102	378.3
augustus	g11532.t1	UniRef100_K02327	0.00E+00	2114
augustus	g11533.t1	UniRef100_K00262	8.70E-228	794.7
augustus	g11540.t1	UniRef100_K01796	7.10E-180	635.2
augustus	g11541.t1	UniRef100_K17065	0.00E+00	1252.7
augustus	g11544.t1	UniRef100_K18423	0.00E+00	1707.2
augustus	g11547.t1	UniRef100_K11501	0.00E+00	1095.9
augustus	g11548.t1	UniRef100_K12608	1.00E-155	554.3
augustus	g11549.t1	UniRef100_K02328	1.40E-244	850.5
augustus	g11550.t1	UniRef100_K17989	8.60E-136	488.4
augustus	g11551.t1	UniRef100_K11511	1.40E-12	77.8
augustus	g11553.t1	UniRef100_K17989	1.20E-167	594.3
augustus	g11561.t1	UniRef100_K07252	8.70E-85	318.5
augustus	g11565.t1	UniRef100_K00858	3.50E-289	998.8
augustus	g11569.t1	UniRef100_K16912	4.50E-92	343.2
augustus	g11570.t1	UniRef100_K01069	1.70E-170	604.4
augustus	g11578.t1	UniRef100_K06671	0.00E+00	1574.7
augustus	g11579.t1	UniRef100_K03190	2.50E-153	546.6
augustus	g11589.t1	UniRef100_K15105	0.00E+00	1301.6
augustus	g11591.t1	UniRef100_K12386	5.80E-128	462.2
augustus	g11592.t1	UniRef100_K01869	0.00E+00	1702.2
augustus	g11593.t1	UniRef100_K00297	0.00E+00	1252.7
augustus	g11597.t1	UniRef100_K13124	4.50E-159	565.8
augustus	g11598.t1	UniRef100_K03854	3.10E-233	812.4
augustus	g11599.t1	UniRef100_K16572	0.00E+00	1438.3
augustus	g11602.t1	UniRef100_K00773	7.20E-213	745
augustus	g11603.t1	UniRef100_K03685	1.00E-31	141.7
augustus	g11604.t1	UniRef100_K01077	6.70E-71	273.9
augustus	g11605.t1	UniRef100_K06201	4.00E-113	412.9
augustus	g11608.t1	UniRef100_K14558	0.00E+00	1502.3
augustus	g11609.t1	UniRef100_K16948	2.50E-213	746.1
augustus	g11610.t1	UniRef100_K00905	5.30E-51	207.2
augustus	g11611.t1	UniRef100_K11838	0.00E+00	2129
augustus	g11612.t1	UniRef100_K13278	1.60E-272	943.3

augustus	g11613.t1	UniRef100_K15562	7.30E-240	834.7
augustus	g11616.t1	UniRef100_K01409	1.10E-189	667.5
augustus	g11617.t1	UniRef100_K11322	1.20E-285	986.9
augustus	g11618.t1	UniRef100_K06268	2.30E-87	326.6
augustus	g11620.t1	UniRef100_K02996	1.40E-151	540.8
augustus	g11621.t1	UniRef100_K03233	3.40E-187	659.4
augustus	g11623.t1	UniRef100_K14795	4.10E-57	226.9
augustus	g11627.t1	UniRef100_K13102	4.90E-125	452.6
augustus	g11628.t1	UniRef100_K15171	5.40E-60	235.3
augustus	g11629.t1	UniRef100_K14528	2.50E-65	253.1
augustus	g11630.t1	UniRef100_K04460	3.60E-237	825.9
augustus	g11631.t1	UniRef100_K13118	2.10E-203	713.4
augustus	g11632.t1	UniRef100_K08869	4.50E-262	909.1
augustus	g11633.t1	UniRef100_K09425	1.20E-73	282
augustus	g11635.t1	UniRef100_K15147	0.00E+00	1622.8
augustus	g11637.t1	UniRef100_K15710	0.00E+00	2259.2
augustus	g11644.t1	UniRef100_K08284	7.90E-241	838.2
augustus	g11645.t1	UniRef100_K17279	4.20E-70	269.6
augustus	g11646.t1	UniRef100_K09175	0.00E+00	1332.4
augustus	g11647.t1	UniRef100_K00928	4.10E-264	915.2
augustus	g11648.t1	UniRef100_K15071	0.00E+00	1615.5
augustus	g11649.t1	UniRef100_K02202	1.30E-215	753.8
augustus	g11654.t1	UniRef100_K00018	2.10E-92	344.4
augustus	g11655.t1	UniRef100_K01235	0.00E+00	1554.3
augustus	g11656.t1	UniRef100_K01266	9.30E-186	654.8
augustus	g11657.t1	UniRef100_K01266	4.20E-131	473
augustus	g11670.t1	UniRef100_K17053	1.40E-161	574.3
augustus	g11673.t1	UniRef100_K18588	2.30E-62	243.8
augustus	g11674.t1	UniRef100_K12396	0.00E+00	1529.2
augustus	g11676.t1	UniRef100_K01273	9.50E-199	698
augustus	g11677.t1	UniRef100_K02952	3.00E-53	212.6
augustus	g11679.t1	UniRef100_K15448	4.50E-57	225.7
augustus	g11685.t1	UniRef100_K11422	0.00E+00	1758.4
augustus	g11687.t1	UniRef100_K01519	1.70E-76	291.2
augustus	g11689.t1	UniRef100_K14857	0.00E+00	1156.4
augustus	g11690.t1	UniRef100_K22140	0.00E+00	1556.2
augustus	g11695.t1	UniRef100_K14006	0.00E+00	1503.8
augustus	g11696.t1	UniRef100_K10578	5.70E-115	419.1
augustus	g11697.t1	UniRef100_K15204	0.00E+00	2181.8

Supplementary Table S3. Genetic Diversity Metrics for MLST data

===>

Number of sequences: 48   Number of sequences used: 48

===>

Nucleotide diversity: pi = 0.026173

Number of segregating sites: 174

Number of parsimony-informative sites:122

Tajima's D statistic: D = -0.617735

p (D >= -0.617735) = 0.712829

===>

=========== Haplotype Distribution ===========

Selected region: 1-1990   Number of sites: 1990

Total number of sites (excluding sites with gaps / missing data): 1213

Sites with alignment gaps: not considered

Number of variable sites: 307

Number of haplotypes, h: 41

Haplotype diversity, Hd: 0.9929

Hap_1: 1 [Trichoderma_afarasin_DIS_314F]

Hap_2: 1 [Trichoderma_afroharzianum_GJS_04-186]

Hap_3: 1 [Trichoderma_afroharzianum_CBS_46694]

Hap_4: 1 [Trichoderma_alni_CBS_120633]

Hap_5: 1 [Trichoderma_alpinum_HMAS_248821]

Hap_6: 1 [Trichoderma_anaharzianum_YMF_100241]

Hap_7: 1 [Trichoderma_anaharzianum_YMF100383]

Hap_8: 2 [Trichoderma_asiaticum_YMF_100352 Trichoderma_asiaticum_YMF100168]

Hap_9: 3 [Trichoderma_atrobrunneum_GJS_90-254 Trichoderma_atrobrunneum_GJS_04-67 Trichoderma_atrobrunneum_T42]

Hap_10: 1 [Trichoderma_azevedoi_CEN1422]

Hap_11: 1 [Trichoderma_bannaense_HMAS_248840]

Hap_12: 1 [Trichoderma_breve_HMAS_248844]

Hap_13: 1 [Trichoderma_concentricum_HMAS_248833]

Hap_14: 1 [Trichoderma_endophyticum_549F18C-AC]

Hap_15: 1 [Trichoderma_endophyticum_23F18C-AC]

Hap_16: 2 [Trichoderma_guizhouense_HGUP0038 Trichoderma_guizhouense_HGUP0039]

Hap_17: 1 [Trichoderma_harzianum_CBS_22695]

Hap_18: 1 [Trichoderma_harzianum_GJS_05107]

Hap_19: 1 [Trichoderma_inhamatum_CBS_27378]

Hap_20: 1 [Trichoderma_lentiforme_DIS_253B]

Hap_21: 1 [Trichoderma_lentiforme_DIS_94D]

Hap_22: 1 [Trichoderma_lentiforme_DIS_218E]

Hap_23: 1 [Trichoderma_lentiforme_DIS_354A]

Hap_24: 2 [Trichoderma_lentiforme_CEN1428 Trichoderma_lentiforme_CEN1429]

Hap_25: 1 [Trichoderma_lentinulae_CGMCC_319847]

Hap_26: 1 [Trichoderma_lixii_CBS_110080]

Hap_27: 1 [Trichoderma_pollinicola_LC11686]

Hap_28: 1 [Trichoderma_pseudoasiaticum_YMF_106200]

Hap_29: 1 [Trichoderma_pseudodensum_HMAS_248828]

Hap_30: 1 [Trichoderma_pyramidale_CBS_135574]

Hap_31: 1 [Trichoderma_rifaii_DIS_337F]

Hap_32: 1 [Trichoderma_rugulosum_SFC20180301-001]

Hap_33: 2 [Trichoderma_simile_YMF_106201 Trichoderma_simile_YMF_16180]

Hap_34: 2 [Trichoderma_simmonsii_GJS_91-138 Trichoderma_simmonsii_GJS_90-127]

Hap_35: 1 [Trichoderma_vermifimicola_CGMCC_319694]

Hap_36: 1 [Trichoderma_xixiacum_CGMCC_319697]

Hap_37: 1 [Trichoderma_zayuense_HMAS_248835]

Hap_38: 1 [Trichoderma_zelobreve_CGMCC_319695]

Hap_39: 1 [Trichoderma_zeloharzianum_YMF100268]

Hap_40: 1 [Trichoderma_sp_BMH-0061]

Hap_41: 1 [Protocrea_pallida_CBS_29978]

===>

Supplementary Table S4. Variant Call Format (VCF v4.1) generated by GSAlign v1.0.22 using reference genome GCA_019097725.fna

```
##fileformat=VCFv4.1
##reference=GCA_019097725.fna
##source=GSAlign 1.0.22
##INFO=<ID=TYPE,Number=1,Type=String,Description="The type of allele, either
SUBSTITUTE, INSERT, or DELETE.">
##contig=<ID=CM032587.1,length=7693100>
##contig=<ID=CM032588.1,length=7235100>
##contig=<ID=CM032589.1,length=6422318>
##contig=<ID=CM032590.1,length=4805890>
##contig=<ID=CM032591.1,length=4727320>
##contig=<ID=CM032592.1,length=4458493>
##contig=<ID=CM032593.1,length=4295973>
##contig=<ID=JAFFQW010000014.1,length=5100>
##contig=<ID=JAFFQW010000022.1,length=1000>
##contig=<ID=JAFFQW010000012.1,length=21000>
##contig=<ID=JAFFQW010000013.1,length=6000>
##contig=<ID=JAFFQW010000023.1,length=1000>
##contig=<ID=JAFFQW010000021.1,length=1000>
##contig=<ID=JAFFQW010000016.1,length=2795>
##contig=<ID=JAFFQW010000019.1,length=1632>
##contig=<ID=JAFFQW010000017.1,length=2368>
##contig=<ID=JAFFQW010000024.1,length=1000>
##contig=<ID=JAFFQW010000009.1,length=89741>
##contig=<ID=JAFFQW010000010.1,length=83139>
##contig=<ID=JAFFQW010000020.1,length=1500>
##contig=<ID=JAFFQW010000015.1,length=2843>
##contig=<ID=JAFFQW010000018.1,length=1680>
##contig=<ID=JAFFQW010000008.1,length=119853>
##contig=<ID=JAFFQW010000011.1,length=23205>
#CHROM      POS   ID    REF   ALT   QUAL  FILTER      INFO
CM032587.1  43367 .     C     T     100   *     TYPE=SUBSTITUTE
CM032587.1  43375 .     C     T     100   *     TYPE=SUBSTITUTE
CM032587.1  43393 .     T     C     100   *     TYPE=SUBSTITUTE
CM032587.1  43398 .     G     A     100   *     TYPE=SUBSTITUTE
CM032587.1  43399 .     G     A     100   *     TYPE=SUBSTITUTE
CM032587.1  43399 .     G     A     100   *     TYPE=SUBSTITUTE
CM032587.1  43404 .     G     a     100   *     TYPE=SUBSTITUTE
CM032587.1  43404 .     G     a     100   *     TYPE=SUBSTITUTE
CM032587.1  43419 .     A     g     100   *     TYPE=SUBSTITUTE
CM032587.1  43421 .     C     t     100   *     TYPE=SUBSTITUTE
CM032587.1  43421 .     C     T     100   *     TYPE=SUBSTITUTE
CM032587.1  43427 .     C     t     100   *     TYPE=SUBSTITUTE
CM032587.1  43431 .     A     g     100   *     TYPE=SUBSTITUTE
CM032587.1  43448 .     GA    G     100   *     TYPE=DELETE
CM032587.1  43453 .     A     t     100   *     TYPE=SUBSTITUTE
CM032587.1  43455 .     G     a     100   *     TYPE=SUBSTITUTE
CM032587.1  43455 .     G     a     100   *     TYPE=SUBSTITUTE
CM032587.1  43456 .     G     a     100   *     TYPE=SUBSTITUTE
CM032587.1  43457 .     A     g     100   *     TYPE=SUBSTITUTE
CM032587.1  43457 .     A     g     100   *     TYPE=SUBSTITUTE
CM032587.1  43460 .     A     g     100   *     TYPE=SUBSTITUTE
CM032587.1  43471 .     AT    A     100   *     TYPE=DELETE
CM032587.1  43486 .     C     t     100   *     TYPE=SUBSTITUTE
CM032587.1  43486 .     C     t     100   *     TYPE=SUBSTITUTE
CM032587.1  43495 .     G     a     100   *     TYPE=SUBSTITUTE
CM032587.1  43495 .     G     A     100   *     TYPE=SUBSTITUTE
CM032587.1  43496 .     G     a     100   *     TYPE=SUBSTITUTE
CM032587.1  43497 .     GA    G     100   *     TYPE=DELETE
CM032587.1  43498 .     A     g     100   *     TYPE=SUBSTITUTE
CM032587.1  43498 .     A     g     100   *     TYPE=SUBSTITUTE
CM032587.1  43501 .     C     g     100   *     TYPE=SUBSTITUTE
CM032587.1  43501 .     C     g     100   *     TYPE=SUBSTITUTE
CM032587.1  43501 .     C     a     100   *     TYPE=SUBSTITUTE
CM032587.1  43502 .     C     t     100   *     TYPE=SUBSTITUTE
```

CM032587.1	43510	.	A	g	100	*	TYPE=SUBSTITUTE
CM032587.1	43510	.	A	g	100	*	TYPE=SUBSTITUTE
CM032587.1	43510	.	A	g	100	*	TYPE=SUBSTITUTE
CM032587.1	43511	.	C	t	100	*	TYPE=SUBSTITUTE
CM032587.1	43511	.	C	t	100	*	TYPE=SUBSTITUTE
CM032587.1	43517	.	t	tc	100	*	TYPE=INSERT
CM032587.1	43519	.	T	C	100	*	TYPE=SUBSTITUTE
CM032587.1	43524	.	T	c	100	*	TYPE=SUBSTITUTE
CM032587.1	43525	.	A	t	100	*	TYPE=SUBSTITUTE
CM032587.1	43525	.	A	T	100	*	TYPE=SUBSTITUTE
CM032587.1	43527	.	A	G	100	*	TYPE=SUBSTITUTE
CM032587.1	43527	.	A	g	100	*	TYPE=SUBSTITUTE
CM032587.1	43531	.	T	C	100	*	TYPE=SUBSTITUTE
CM032587.1	43539	.	C	t	100	*	TYPE=SUBSTITUTE
CM032587.1	43539	.	C	t	100	*	TYPE=SUBSTITUTE
CM032587.1	43540	.	C	t	100	*	TYPE=SUBSTITUTE
CM032587.1	43540	.	C	T	100	*	TYPE=SUBSTITUTE
CM032587.1	43540	.	C	t	100	*	TYPE=SUBSTITUTE
CM032587.1	43547	.	C	t	100	*	TYPE=SUBSTITUTE
CM032587.1	43548	.	C	t	100	*	TYPE=SUBSTITUTE
CM032587.1	43549	.	T	c	100	*	TYPE=SUBSTITUTE
CM032587.1	43550	.	T	c	100	*	TYPE=SUBSTITUTE
CM032587.1	43550	.	T	C	100	*	TYPE=SUBSTITUTE
CM032587.1	43555	.	a	at	100	*	TYPE=INSERT
CM032587.1	43556	.	C	t	100	*	TYPE=SUBSTITUTE
CM032587.1	43556	.	C	a	100	*	TYPE=SUBSTITUTE
CM032587.1	43560	.	G	a	100	*	TYPE=SUBSTITUTE
CM032587.1	43560	.	G	a	100	*	TYPE=SUBSTITUTE
CM032587.1	43561	.	G	a	100	*	TYPE=SUBSTITUTE
CM032587.1	43563	.	A	g	100	*	TYPE=SUBSTITUTE
CM032587.1	43569	.	T	a	100	*	TYPE=SUBSTITUTE
CM032587.1	43569	.	T	a	100	*	TYPE=SUBSTITUTE
CM032587.1	43580	.	A	g	100	*	TYPE=SUBSTITUTE
CM032587.1	43580	.	A	g	100	*	TYPE=SUBSTITUTE
CM032587.1	43580	.	A	g	100	*	TYPE=SUBSTITUTE
CM032587.1	43588	.	G	a	100	*	TYPE=SUBSTITUTE
CM032587.1	43588	.	G	a	100	*	TYPE=SUBSTITUTE
CM032587.1	43593	.	c	ct	100	*	TYPE=INSERT
CM032587.1	43594	.	C	t	100	*	TYPE=SUBSTITUTE
CM032587.1	43594	.	C	t	100	*	TYPE=SUBSTITUTE
CM032587.1	43594	.	C	t	100	*	TYPE=SUBSTITUTE
CM032587.1	43596	.	C	t	100	*	TYPE=SUBSTITUTE
CM032587.1	43596	.	C	t	100	*	TYPE=SUBSTITUTE
CM032587.1	43597	.	C	t	100	*	TYPE=SUBSTITUTE
CM032587.1	43597	.	C	t	100	*	TYPE=SUBSTITUTE
CM032587.1	43614	.	A	g	100	*	TYPE=SUBSTITUTE
CM032587.1	43615	.	G	a	100	*	TYPE=SUBSTITUTE
CM032587.1	43617	.	G	a	100	*	TYPE=SUBSTITUTE
CM032587.1	43618	.	G	a	100	*	TYPE=SUBSTITUTE
CM032587.1	43618	.	G	a	100	*	TYPE=SUBSTITUTE
CM032587.1	43621	.	G	a	100	*	TYPE=SUBSTITUTE
CM032587.1	43621	.	G	a	100	*	TYPE=SUBSTITUTE
CM032587.1	43622	.	A	g	100	*	TYPE=SUBSTITUTE
CM032587.1	43622	.	A	G	100	*	TYPE=SUBSTITUTE
CM032587.1	43623	.	G	a	100	*	TYPE=SUBSTITUTE
CM032587.1	43624	.	G	a	100	*	TYPE=SUBSTITUTE
CM032587.1	43624	.	G	A	100	*	TYPE=SUBSTITUTE
CM032587.1	43626	.	C	t	100	*	TYPE=SUBSTITUTE
CM032587.1	43629	.	T	c	100	*	TYPE=SUBSTITUTE
CM032587.1	43636	.	G	a	100	*	TYPE=SUBSTITUTE
CM032587.1	43636	.	G	a	100	*	TYPE=SUBSTITUTE
CM032587.1	43636	.	G	a	100	*	TYPE=SUBSTITUTE
CM032587.1	43639	.	T	c	100	*	TYPE=SUBSTITUTE
CM032587.1	43639	.	T	c	100	*	TYPE=SUBSTITUTE

CM032587.1	43639	.	T	c	100	*	TYPE=SUBSTITUTE
CM032587.1	43648	.	A	g	100	*	TYPE=SUBSTITUTE
CM032587.1	43648	.	A	g	100	*	TYPE=SUBSTITUTE
CM032587.1	43648	.	A	g	100	*	TYPE=SUBSTITUTE
CM032587.1	43651	.	T	c	100	*	TYPE=SUBSTITUTE
CM032587.1	43651	.	T	c	100	*	TYPE=SUBSTITUTE
CM032587.1	43651	.	T	c	100	*	TYPE=SUBSTITUTE
CM032587.1	43652	.	C	t	100	*	TYPE=SUBSTITUTE
CM032587.1	43652	.	C	t	100	*	TYPE=SUBSTITUTE
CM032587.1	43660	.	C	t	100	*	TYPE=SUBSTITUTE
CM032587.1	43660	.	C	t	100	*	TYPE=SUBSTITUTE
CM032587.1	43660	.	C	t	100	*	TYPE=SUBSTITUTE
CM032587.1	43661	.	C	t	100	*	TYPE=SUBSTITUTE
CM032587.1	43661	.	C	t	100	*	TYPE=SUBSTITUTE
CM032587.1	43661	.	C	t	100	*	TYPE=SUBSTITUTE
CM032587.1	43665	.	G	a	100	*	TYPE=SUBSTITUTE
CM032587.1	43665	.	G	a	100	*	TYPE=SUBSTITUTE
CM032587.1	43665	.	G	a	100	*	TYPE=SUBSTITUTE
CM032587.1	43666	.	T	c	100	*	TYPE=SUBSTITUTE
CM032587.1	43669	.	A	t	100	*	TYPE=SUBSTITUTE
CM032587.1	43669	.	A	t	100	*	TYPE=SUBSTITUTE
CM032587.1	43670	.	C	t	100	*	TYPE=SUBSTITUTE
CM032587.1	43670	.	C	t	100	*	TYPE=SUBSTITUTE
CM032587.1	43670	.	C	t	100	*	TYPE=SUBSTITUTE
CM032587.1	43686	.	G	a	100	*	TYPE=SUBSTITUTE
CM032587.1	43691	.	A	g	100	*	TYPE=SUBSTITUTE
CM032587.1	43691	.	A	g	100	*	TYPE=SUBSTITUTE
CM032587.1	43730	.	T	a	100	*	TYPE=SUBSTITUTE
CM032587.1	43741	.	T	c	100	*	TYPE=SUBSTITUTE
CM032587.1	43741	.	T	c	100	*	TYPE=SUBSTITUTE
CM032587.1	43741	.	T	c	100	*	TYPE=SUBSTITUTE
CM032587.1	43748	.	G	a	100	*	TYPE=SUBSTITUTE
CM032587.1	43748	.	G	a	100	*	TYPE=SUBSTITUTE
CM032587.1	43748	.	G	a	100	*	TYPE=SUBSTITUTE
CM032587.1	43749	.	C	t	100	*	TYPE=SUBSTITUTE
CM032587.1	43749	.	C	t	100	*	TYPE=SUBSTITUTE
CM032587.1	43749	.	C	t	100	*	TYPE=SUBSTITUTE
CM032587.1	43755	.	G	a	100	*	TYPE=SUBSTITUTE
CM032587.1	43755	.	G	a	100	*	TYPE=SUBSTITUTE
CM032587.1	43756	.	C	t	100	*	TYPE=SUBSTITUTE
CM032587.1	43765	.	A	g	100	*	TYPE=SUBSTITUTE
CM032587.1	43771	.	G	a	100	*	TYPE=SUBSTITUTE
CM032587.1	43771	.	G	a	100	*	TYPE=SUBSTITUTE
CM032587.1	43774	.	TA	T	100	*	TYPE=DELETE
CM032587.1	43785	.	A	g	100	*	TYPE=SUBSTITUTE
CM032587.1	43786	.	C	t	100	*	TYPE=SUBSTITUTE
CM032587.1	43791	.	A	g	100	*	TYPE=SUBSTITUTE
CM032587.1	43791	.	A	g	100	*	TYPE=SUBSTITUTE
CM032587.1	43792	.	G	a	100	*	TYPE=SUBSTITUTE
CM032587.1	43792	.	G	a	100	*	TYPE=SUBSTITUTE
CM032587.1	43793	.	A	g	100	*	TYPE=SUBSTITUTE
CM032587.1	43793	.	A	g	100	*	TYPE=SUBSTITUTE
CM032587.1	43794	.	A	g	100	*	TYPE=SUBSTITUTE
CM032587.1	43797	.	TA	T	100	*	TYPE=DELETE
CM032587.1	43802	.	A	g	100	*	TYPE=SUBSTITUTE
CM032587.1	43816	.	g	gt	100	*	TYPE=INSERT
CM032587.1	43821	.	G	a	100	*	TYPE=SUBSTITUTE
CM032587.1	43821	.	G	a	100	*	TYPE=SUBSTITUTE
CM032587.1	43821	.	G	a	100	*	TYPE=SUBSTITUTE
CM032587.1	43826	.	C	t	100	*	TYPE=SUBSTITUTE
CM032587.1	43826	.	C	t	100	*	TYPE=SUBSTITUTE
CM032587.1	43826	.	C	t	100	*	TYPE=SUBSTITUTE
CM032587.1	43829	.	G	a	100	*	TYPE=SUBSTITUTE
CM032587.1	43829	.	G	a	100	*	TYPE=SUBSTITUTE

CM032587.1	43829	.	G	a	100	*	TYPE=SUBSTITUTE
CM032587.1	43837	.	A	t	100	*	TYPE=SUBSTITUTE
CM032587.1	43840	.	T	c	100	*	TYPE=SUBSTITUTE
CM032587.1	43840	.	T	c	100	*	TYPE=SUBSTITUTE
CM032587.1	43846	.	A	g	100	*	TYPE=SUBSTITUTE
CM032587.1	43846	.	A	g	100	*	TYPE=SUBSTITUTE
CM032587.1	43849	.	A	g	100	*	TYPE=SUBSTITUTE
CM032587.1	43878	.	C	t	100	*	TYPE=SUBSTITUTE
CM032587.1	43878	.	C	t	100	*	TYPE=SUBSTITUTE
CM032587.1	43878	.	C	t	100	*	TYPE=SUBSTITUTE
CM032587.1	43879	.	C	t	100	*	TYPE=SUBSTITUTE
CM032587.1	43879	.	C	t	100	*	TYPE=SUBSTITUTE
CM032587.1	43879	.	C	t	100	*	TYPE=SUBSTITUTE
CM032587.1	43884	.	AT	A	100	*	TYPE=DELETE
CM032587.1	43884	.	AT	A	100	*	TYPE=DELETE
CM032587.1	43899	.	C	t	100	*	TYPE=SUBSTITUTE
CM032587.1	43909	.	A	t	100	*	TYPE=SUBSTITUTE
CM032587.1	43918	.	G	a	100	*	TYPE=SUBSTITUTE
CM032587.1	43923	.	A	g	100	*	TYPE=SUBSTITUTE
CM032587.1	43923	.	A	g	100	*	TYPE=SUBSTITUTE
CM032587.1	43923	.	A	g	100	*	TYPE=SUBSTITUTE
CM032587.1	43926	.	GT	G	100	*	TYPE=DELETE
CM032587.1	43926	.	G	a	100	*	TYPE=SUBSTITUTE
CM032587.1	43926	.	GT	G	100	*	TYPE=DELETE
CM032587.1	43928	.	T	a	100	*	TYPE=SUBSTITUTE
CM032587.1	43938	.	G	a	100	*	TYPE=SUBSTITUTE
CM032587.1	43938	.	G	a	100	*	TYPE=SUBSTITUTE
CM032587.1	43940	.	A	t	100	*	TYPE=SUBSTITUTE
CM032587.1	43943	.	A	g	100	*	TYPE=SUBSTITUTE
CM032587.1	43944	.	a	ag	100	*	TYPE=INSERT
CM032587.1	43944	.	A	g	100	*	TYPE=SUBSTITUTE
CM032587.1	43946	.	G	a	100	*	TYPE=SUBSTITUTE
CM032587.1	43954	.	CT	C	100	*	TYPE=DELETE
CM032587.1	43955	.	T	c	100	*	TYPE=SUBSTITUTE
CM032587.1	43958	.	T	c	100	*	TYPE=SUBSTITUTE
CM032587.1	43959	.	T	c	100	*	TYPE=SUBSTITUTE
CM032587.1	43961	.	TT	T	100	*	TYPE=DELETE
CM032587.1	43975	.	A	g	100	*	TYPE=SUBSTITUTE
CM032587.1	43976	.	C	t	100	*	TYPE=SUBSTITUTE
CM032587.1	43976	.	C	t	100	*	TYPE=SUBSTITUTE
CM032587.1	43979	.	T	c	100	*	TYPE=SUBSTITUTE
CM032587.1	43979	.	T	c	100	*	TYPE=SUBSTITUTE
CM032587.1	43987	.	T	c	100	*	TYPE=SUBSTITUTE
CM032587.1	43988	.	C	t	100	*	TYPE=SUBSTITUTE
CM032587.1	43988	.	C	t	100	*	TYPE=SUBSTITUTE
CM032587.1	43995	.	G	a	100	*	TYPE=SUBSTITUTE
CM032587.1	43995	.	G	A	100	*	TYPE=SUBSTITUTE
CM032587.1	43995	.	G	a	100	*	TYPE=SUBSTITUTE
CM032587.1	43997	.	T	C	100	*	TYPE=SUBSTITUTE
CM032587.1	44004	.	G	a	100	*	TYPE=SUBSTITUTE
CM032587.1	44006	.	C	t	100	*	TYPE=SUBSTITUTE
CM032587.1	44009	.	A	g	100	*	TYPE=SUBSTITUTE
CM032587.1	44010	.	G	A	100	*	TYPE=SUBSTITUTE
CM032587.1	44013	.	C	t	100	*	TYPE=SUBSTITUTE
CM032587.1	44013	.	C	T	100	*	TYPE=SUBSTITUTE
CM032587.1	44013	.	C	T	100	*	TYPE=SUBSTITUTE
CM032587.1	44014	.	T	TA	100	*	TYPE=INSERT
CM032587.1	44016	.	A	G	100	*	TYPE=SUBSTITUTE
CM032587.1	44017	.	G	a	100	*	TYPE=SUBSTITUTE
CM032587.1	44018	.	T	c	100	*	TYPE=SUBSTITUTE
CM032587.1	44022	.	T	c	100	*	TYPE=SUBSTITUTE
CM032587.1	44042	.	G	a	100	*	TYPE=SUBSTITUTE
CM032587.1	44045	.	G	a	100	*	TYPE=SUBSTITUTE
CM032587.1	44045	.	G	a	100	*	TYPE=SUBSTITUTE

CM032587.1	44052	.	A	g	100	*	TYPE=SUBSTITUTE
CM032587.1	44053	.	G	a	100	*	TYPE=SUBSTITUTE
CM032587.1	44055	.	A	t	100	*	TYPE=SUBSTITUTE
CM032587.1	44062	.	C	t	100	*	TYPE=SUBSTITUTE
CM032587.1	44062	.	C	t	100	*	TYPE=SUBSTITUTE
CM032587.1	44062	.	C	t	100	*	TYPE=SUBSTITUTE
CM032587.1	44063	.	G	a	100	*	TYPE=SUBSTITUTE
CM032587.1	44063	.	G	a	100	*	TYPE=SUBSTITUTE
CM032587.1	44063	.	G	a	100	*	TYPE=SUBSTITUTE
CM032587.1	44066	.	A	g	100	*	TYPE=SUBSTITUTE
CM032587.1	44068	.	c	ct	100	*	TYPE=INSERT
CM032587.1	44073	.	A	g	100	*	TYPE=SUBSTITUTE
CM032587.1	44076	.	A	g	100	*	TYPE=SUBSTITUTE
CM032587.1	44078	.	CT	C	100	*	TYPE=DELETE
CM032587.1	44080	.	T	C	100	*	TYPE=SUBSTITUTE
CM032587.1	44081	.	T	C	100	*	TYPE=SUBSTITUTE
CM032587.1	44086	.	A	G	100	*	TYPE=SUBSTITUTE
CM032587.1	44092	.	G	a	100	*	TYPE=SUBSTITUTE
CM032587.1	44093	.	C	t	100	*	TYPE=SUBSTITUTE
CM032587.1	44093	.	C	t	100	*	TYPE=SUBSTITUTE
CM032587.1	44093	.	C	T	100	*	TYPE=SUBSTITUTE
CM032587.1	44094	.	AG	A	100	*	TYPE=DELETE
CM032587.1	44095	.	G	A	100	*	TYPE=SUBSTITUTE
CM032587.1	44095	.	G	a	100	*	TYPE=SUBSTITUTE
CM032587.1	44097	.	G	a	100	*	TYPE=SUBSTITUTE
CM032587.1	44104	.	C	t	100	*	TYPE=SUBSTITUTE
CM032587.1	44104	.	C	t	100	*	TYPE=SUBSTITUTE
CM032587.1	44104	.	C	t	100	*	TYPE=SUBSTITUTE
CM032587.1	44126	.	T	c	100	*	TYPE=SUBSTITUTE
CM032587.1	44130	.	A	g	100	*	TYPE=SUBSTITUTE
CM032587.1	44146	.	AGA	A	100	*	TYPE=DELETE
CM032587.1	44147	.	G	a	100	*	TYPE=SUBSTITUTE
CM032587.1	44147	.	G	a	100	*	TYPE=SUBSTITUTE
CM032587.1	44150	.	T	a	100	*	TYPE=SUBSTITUTE
CM032587.1	44151	.	T	c	100	*	TYPE=SUBSTITUTE
CM032587.1	44165	.	G	a	100	*	TYPE=SUBSTITUTE
CM032587.1	44177	.	T	c	100	*	TYPE=SUBSTITUTE
CM032587.1	44182	.	A	G	100	*	TYPE=SUBSTITUTE
CM032587.1	44187	.	A	t	100	*	TYPE=SUBSTITUTE
CM032587.1	44188	.	G	a	100	*	TYPE=SUBSTITUTE
CM032587.1	44188	.	G	a	100	*	TYPE=SUBSTITUTE
CM032587.1	44189	.	C	t	100	*	TYPE=SUBSTITUTE
CM032587.1	44189	.	C	t	100	*	TYPE=SUBSTITUTE
CM032587.1	44189	.	C	t	100	*	TYPE=SUBSTITUTE
CM032587.1	44192	.	G	a	100	*	TYPE=SUBSTITUTE
CM032587.1	44192	.	G	a	100	*	TYPE=SUBSTITUTE
CM032587.1	44193	.	C	t	100	*	TYPE=SUBSTITUTE
CM032587.1	44204	.	TA	T	100	*	TYPE=DELETE
CM032587.1	44212	.	C	t	100	*	TYPE=SUBSTITUTE
CM032587.1	44213	.	C	t	100	*	TYPE=SUBSTITUTE
CM032587.1	44216	.	C	t	100	*	TYPE=SUBSTITUTE
CM032587.1	44216	.	C	t	100	*	TYPE=SUBSTITUTE
CM032587.1	44220	.	C	t	100	*	TYPE=SUBSTITUTE
CM032587.1	44220	.	C	t	100	*	TYPE=SUBSTITUTE
CM032587.1	44220	.	C	t	100	*	TYPE=SUBSTITUTE
CM032587.1	44224	.	G	a	100	*	TYPE=SUBSTITUTE
CM032587.1	44224	.	G	a	100	*	TYPE=SUBSTITUTE
CM032587.1	44245	.	G	a	100	*	TYPE=SUBSTITUTE
CM032587.1	44250	.	AG	A	100	*	TYPE=DELETE
CM032587.1	44251	.	G	a	100	*	TYPE=SUBSTITUTE
CM032587.1	44251	.	G	a	100	*	TYPE=SUBSTITUTE
CM032587.1	44252	.	G	a	100	*	TYPE=SUBSTITUTE
CM032587.1	44254	.	C	t	100	*	TYPE=SUBSTITUTE
CM032587.1	44259	.	TA	T	100	*	TYPE=DELETE

CM032587.1	44261	.	T	c	100	*	TYPE=SUBSTITUTE
CM032587.1	44262	.	T	a	100	*	TYPE=SUBSTITUTE
CM032587.1	44263	.	A	t	100	*	TYPE=SUBSTITUTE
CM032587.1	44266	.	A	c	100	*	TYPE=SUBSTITUTE
CM032587.1	44266	.	A	g	100	*	TYPE=SUBSTITUTE
CM032587.1	44269	.	T	c	100	*	TYPE=SUBSTITUTE
CM032587.1	44277	.	A	g	100	*	TYPE=SUBSTITUTE
CM032587.1	44277	.	A	g	100	*	TYPE=SUBSTITUTE
CM032587.1	44291	.	t	ta	100	*	TYPE=INSERT
CM032587.1	44293	.	G	t	100	*	TYPE=SUBSTITUTE
CM032587.1	44295	.	T	c	100	*	TYPE=SUBSTITUTE
CM032587.1	44299	.	T	a	100	*	TYPE=SUBSTITUTE
CM032587.1	44300	.	A	t	100	*	TYPE=SUBSTITUTE
CM032587.1	44311	.	C	t	100	*	TYPE=SUBSTITUTE
CM032587.1	44322	.	C	t	100	*	TYPE=SUBSTITUTE
CM032587.1	44322	.	C	t	100	*	TYPE=SUBSTITUTE
CM032587.1	44322	.	C	t	100	*	TYPE=SUBSTITUTE
CM032587.1	44326	.	A	g	100	*	TYPE=SUBSTITUTE
CM032587.1	44328	.	C	t	100	*	TYPE=SUBSTITUTE
CM032587.1	44328	.	C	t	100	*	TYPE=SUBSTITUTE
CM032587.1	44328	.	C	t	100	*	TYPE=SUBSTITUTE
CM032587.1	44329	.	C	t	100	*	TYPE=SUBSTITUTE
CM032587.1	44329	.	C	t	100	*	TYPE=SUBSTITUTE
CM032587.1	44329	.	C	t	100	*	TYPE=SUBSTITUTE
CM032587.1	44337	.	G	a	100	*	TYPE=SUBSTITUTE
CM032587.1	44337	.	G	a	100	*	TYPE=SUBSTITUTE
CM032587.1	44342	.	TA	T	100	*	TYPE=DELETE
CM032587.1	44344	.	A	g	100	*	TYPE=SUBSTITUTE
CM032587.1	44349	.	A	G	100	*	TYPE=SUBSTITUTE
CM032587.1	44349	.	A	g	100	*	TYPE=SUBSTITUTE
CM032587.1	44356	.	A	t	100	*	TYPE=SUBSTITUTE
CM032587.1	44356	.	A	T	100	*	TYPE=SUBSTITUTE
CM032587.1	44356	.	A	t	100	*	TYPE=SUBSTITUTE
CM032587.1	44359	.	AG	A	100	*	TYPE=DELETE
CM032587.1	44360	.	G	a	100	*	TYPE=SUBSTITUTE
CM032587.1	44360	.	G	a	100	*	TYPE=SUBSTITUTE
CM032587.1	44363	.	A	G	100	*	TYPE=SUBSTITUTE
CM032587.1	44363	.	A	g	100	*	TYPE=SUBSTITUTE
CM032587.1	44364	.	G	a	100	*	TYPE=SUBSTITUTE
CM032587.1	44364	.	G	a	100	*	TYPE=SUBSTITUTE
CM032587.1	44365	.	C	t	100	*	TYPE=SUBSTITUTE
CM032587.1	44375	.	A	g	100	*	TYPE=SUBSTITUTE
CM032587.1	44376	.	C	t	100	*	TYPE=SUBSTITUTE
CM032587.1	44378	.	C	t	100	*	TYPE=SUBSTITUTE
CM032587.1	44378	.	C	t	100	*	TYPE=SUBSTITUTE
CM032587.1	44378	.	C	t	100	*	TYPE=SUBSTITUTE
CM032587.1	44381	.	G	a	100	*	TYPE=SUBSTITUTE
CM032587.1	44381	.	G	a	100	*	TYPE=SUBSTITUTE
CM032587.1	44381	.	G	a	100	*	TYPE=SUBSTITUTE
CM032587.1	44387	.	T	a	100	*	TYPE=SUBSTITUTE
CM032587.1	44390	.	T	c	100	*	TYPE=SUBSTITUTE
CM032587.1	44390	.	T	a	100	*	TYPE=SUBSTITUTE
CM032587.1	44390	.	T	c	100	*	TYPE=SUBSTITUTE
CM032587.1	44391	.	T	c	100	*	TYPE=SUBSTITUTE
CM032587.1	44392	.	A	c	100	*	TYPE=SUBSTITUTE
CM032587.1	44393	.	T	c	100	*	TYPE=SUBSTITUTE
CM032587.1	44393	.	T	c	100	*	TYPE=SUBSTITUTE
CM032587.1	44396	.	T	a	100	*	TYPE=SUBSTITUTE
CM032587.1	44396	.	T	c	100	*	TYPE=SUBSTITUTE
CM032587.1	44397	.	T	c	100	*	TYPE=SUBSTITUTE
CM032587.1	44397	.	T	c	100	*	TYPE=SUBSTITUTE
CM032587.1	44402	.	ACTT	A	100	*	TYPE=DELETE
CM032587.1	44408	.	A	g	100	*	TYPE=SUBSTITUTE
CM032587.1	44408	.	A	g	100	*	TYPE=SUBSTITUTE

CM032587.1	44412	.	A	g	100	*	TYPE=SUBSTITUTE
CM032587.1	44412	.	A	t	100	*	TYPE=SUBSTITUTE
CM032587.1	44413	.	C	t	100	*	TYPE=SUBSTITUTE
CM032587.1	44414	.	C	t	100	*	TYPE=SUBSTITUTE
CM032587.1	44414	.	C	t	100	*	TYPE=SUBSTITUTE
CM032587.1	44414	.	C	t	100	*	TYPE=SUBSTITUTE
CM032587.1	44417	.	A	t	100	*	TYPE=SUBSTITUTE
CM032587.1	44417	.	AG	A	100	*	TYPE=DELETE
CM032587.1	44417	.	A	t	100	*	TYPE=SUBSTITUTE
CM032587.1	44418	.	G	a	100	*	TYPE=SUBSTITUTE
CM032587.1	44418	.	G	a	100	*	TYPE=SUBSTITUTE
CM032587.1	44419	.	G	a	100	*	TYPE=SUBSTITUTE
CM032587.1	44419	.	G	a	100	*	TYPE=SUBSTITUTE
CM032587.1	44419	.	G	a	100	*	TYPE=SUBSTITUTE
CM032587.1	44421	.	A	g	100	*	TYPE=SUBSTITUTE
CM032587.1	44423	.	T	c	100	*	TYPE=SUBSTITUTE
CM032587.1	44427	.	G	a	100	*	TYPE=SUBSTITUTE
CM032587.1	44428	.	C	t	100	*	TYPE=SUBSTITUTE
CM032587.1	44428	.	C	t	100	*	TYPE=SUBSTITUTE
CM032587.1	44429	.	T	c	100	*	TYPE=SUBSTITUTE
CM032587.1	44429	.	T	c	100	*	TYPE=SUBSTITUTE
CM032587.1	44432	.	C	t	100	*	TYPE=SUBSTITUTE
CM032587.1	44440	.	A	g	100	*	TYPE=SUBSTITUTE
CM032587.1	44455	.	G	a	100	*	TYPE=SUBSTITUTE
CM032587.1	44455	.	G	a	100	*	TYPE=SUBSTITUTE
CM032587.1	44462	.	A	g	100	*	TYPE=SUBSTITUTE
CM032587.1	44463	.	A	g	100	*	TYPE=SUBSTITUTE
CM032587.1	44464	.	A	g	100	*	TYPE=SUBSTITUTE
CM032587.1	44466	.	AG	A	100	*	TYPE=DELETE
CM032587.1	44467	.	G	a	100	*	TYPE=SUBSTITUTE
CM032587.1	44468	.	G	a	100	*	TYPE=SUBSTITUTE
CM032587.1	44482	.	C	t	100	*	TYPE=SUBSTITUTE
CM032587.1	44483	.	C	t	100	*	TYPE=SUBSTITUTE
CM032587.1	44486	.	C	t	100	*	TYPE=SUBSTITUTE
CM032587.1	44490	.	A	g	100	*	TYPE=SUBSTITUTE
CM032587.1	44492	.	C	t	100	*	TYPE=SUBSTITUTE
CM032587.1	44492	.	C	t	100	*	TYPE=SUBSTITUTE
CM032587.1	44497	.	G	a	100	*	TYPE=SUBSTITUTE
CM032587.1	44497	.	G	a	100	*	TYPE=SUBSTITUTE
CM032587.1	44502	.	C	t	100	*	TYPE=SUBSTITUTE
CM032587.1	44502	.	C	t	100	*	TYPE=SUBSTITUTE
CM032587.1	44502	.	C	t	100	*	TYPE=SUBSTITUTE
CM032587.1	44507	.	G	a	100	*	TYPE=SUBSTITUTE
CM032587.1	44508	.	C	t	100	*	TYPE=SUBSTITUTE
CM032587.1	44508	.	C	t	100	*	TYPE=SUBSTITUTE
CM032587.1	44508	.	C	t	100	*	TYPE=SUBSTITUTE
CM032587.1	44512	.	A	g	100	*	TYPE=SUBSTITUTE
CM032587.1	44512	.	A	g	100	*	TYPE=SUBSTITUTE
CM032587.1	44512	.	A	g	100	*	TYPE=SUBSTITUTE
CM032587.1	44513	.	TAA	T	100	*	TYPE=DELETE
CM032587.1	44516	.	A	g	100	*	TYPE=SUBSTITUTE
CM032587.1	44517	.	A	g	100	*	TYPE=SUBSTITUTE
CM032587.1	44517	.	A	G	100	*	TYPE=SUBSTITUTE
CM032587.1	44517	.	A	g	100	*	TYPE=SUBSTITUTE
CM032587.1	44518	.	A	g	100	*	TYPE=SUBSTITUTE
CM032587.1	44518	.	A	G	100	*	TYPE=SUBSTITUTE
CM032587.1	44518	.	A	g	100	*	TYPE=SUBSTITUTE
CM032587.1	44519	.	A	g	100	*	TYPE=SUBSTITUTE
CM032587.1	44523	.	C	T	100	*	TYPE=SUBSTITUTE
CM032587.1	44523	.	C	t	100	*	TYPE=SUBSTITUTE
CM032587.1	44523	.	C	t	100	*	TYPE=SUBSTITUTE
CM032587.1	44526	.	AG	A	100	*	TYPE=DELETE
CM032587.1	44526	.	AG	A	100	*	TYPE=DELETE
CM032587.1	44527	.	G	a	100	*	TYPE=SUBSTITUTE

CM032587.1	44531	.	A	g	100	*	TYPE=SUBSTITUTE
CM032587.1	44531	.	A	g	100	*	TYPE=SUBSTITUTE
CM032587.1	44532	.	A	g	100	*	TYPE=SUBSTITUTE
CM032587.1	44532	.	A	g	100	*	TYPE=SUBSTITUTE
CM032587.1	44535	.	T	c	100	*	TYPE=SUBSTITUTE
CM032587.1	44558	.	C	t	100	*	TYPE=SUBSTITUTE
CM032587.1	44558	.	C	t	100	*	TYPE=SUBSTITUTE
CM032587.1	44562	.	G	a	100	*	TYPE=SUBSTITUTE
CM032587.1	44564	.	AG	A	100	*	TYPE=DELETE
CM032587.1	44566	.	T	a	100	*	TYPE=SUBSTITUTE
CM032587.1	44569	.	T	c	100	*	TYPE=SUBSTITUTE
CM032587.1	44577	.	G	a	100	*	TYPE=SUBSTITUTE
CM032587.1	44578	.	C	t	100	*	TYPE=SUBSTITUTE
CM032587.1	44579	.	T	c	100	*	TYPE=SUBSTITUTE
CM032587.1	44590	.	G	a	100	*	TYPE=SUBSTITUTE
CM032587.1	44590	.	G	a	100	*	TYPE=SUBSTITUTE
CM032587.1	44590	.	G	a	100	*	TYPE=SUBSTITUTE
CM032587.1	44594	.	T	c	100	*	TYPE=SUBSTITUTE
CM032587.1	44594	.	T	c	100	*	TYPE=SUBSTITUTE
CM032587.1	44605	.	C	t	100	*	TYPE=SUBSTITUTE
CM032587.1	44605	.	C	t	100	*	TYPE=SUBSTITUTE
CM032587.1	44605	.	C	t	100	*	TYPE=SUBSTITUTE
CM032587.1	44608	.	G	a	100	*	TYPE=SUBSTITUTE
CM032587.1	44608	.	G	a	100	*	TYPE=SUBSTITUTE
CM032587.1	44615	.	T	c	100	*	TYPE=SUBSTITUTE
CM032587.1	44618	.	C	t	100	*	TYPE=SUBSTITUTE
CM032587.1	44618	.	C	t	100	*	TYPE=SUBSTITUTE
CM032587.1	44624	.	C	t	100	*	TYPE=SUBSTITUTE
CM032587.1	44624	.	C	t	100	*	TYPE=SUBSTITUTE
CM032587.1	44630	.	C	t	100	*	TYPE=SUBSTITUTE
CM032587.1	44631	.	t	ta	100	*	TYPE=INSERT
CM032587.1	44634	.	G	a	100	*	TYPE=SUBSTITUTE
CM032587.1	44634	.	G	a	100	*	TYPE=SUBSTITUTE
CM032587.1	44637	.	A	g	100	*	TYPE=SUBSTITUTE
CM032587.1	44637	.	A	g	100	*	TYPE=SUBSTITUTE
CM032587.1	44637	.	A	G	100	*	TYPE=SUBSTITUTE
CM032587.1	44643	.	A	c	100	*	TYPE=SUBSTITUTE
CM032587.1	44644	.	A	g	100	*	TYPE=SUBSTITUTE
CM032587.1	44647	.	t	tc	100	*	TYPE=INSERT
CM032587.1	44650	.	T	c	100	*	TYPE=SUBSTITUTE
CM032587.1	44676	.	T	c	100	*	TYPE=SUBSTITUTE
CM032587.1	44687	.	A	g	100	*	TYPE=SUBSTITUTE
CM032587.1	44688	.	A	g	100	*	TYPE=SUBSTITUTE
CM032587.1	44701	.	G	a	100	*	TYPE=SUBSTITUTE
CM032587.1	44708	.	C	t	100	*	TYPE=SUBSTITUTE
CM032587.1	44718	.	G	a	100	*	TYPE=SUBSTITUTE
CM032587.1	44718	.	G	a	100	*	TYPE=SUBSTITUTE
CM032587.1	44732	.	T	c	100	*	TYPE=SUBSTITUTE
CM032587.1	44732	.	T	c	100	*	TYPE=SUBSTITUTE
CM032587.1	44733	.	A	t	100	*	TYPE=SUBSTITUTE
CM032587.1	44733	.	A	t	100	*	TYPE=SUBSTITUTE
CM032587.1	44738	.	A	g	100	*	TYPE=SUBSTITUTE
CM032587.1	44744	.	C	t	100	*	TYPE=SUBSTITUTE
CM032587.1	44748	.	G	A	100	*	TYPE=SUBSTITUTE
CM032587.1	44749	.	T	C	100	*	TYPE=SUBSTITUTE
CM032587.1	44749	.	T	c	100	*	TYPE=SUBSTITUTE
CM032587.1	44750	.	T	c	100	*	TYPE=SUBSTITUTE
CM032587.1	44750	.	T	C	100	*	TYPE=SUBSTITUTE
CM032587.1	44756	.	T	c	100	*	TYPE=SUBSTITUTE
CM032587.1	44759	.	T	c	100	*	TYPE=SUBSTITUTE
CM032587.1	44772	.	G	a	100	*	TYPE=SUBSTITUTE
CM032587.1	44772	.	G	a	100	*	TYPE=SUBSTITUTE
CM032587.1	44782	.	C	t	100	*	TYPE=SUBSTITUTE
CM032587.1	44809	.	A	g	100	*	TYPE=SUBSTITUTE

CM032587.1	44809	.	A	g	100	*	TYPE=SUBSTITUTE
CM032587.1	44809	.	A	g	100	*	TYPE=SUBSTITUTE
CM032587.1	44811	.	C	t	100	*	TYPE=SUBSTITUTE
CM032587.1	44811	.	c	ct	100	*	TYPE=INSERT
CM032587.1	44826	.	A	g	100	*	TYPE=SUBSTITUTE
CM032587.1	44826	.	A	g	100	*	TYPE=SUBSTITUTE
CM032587.1	44826	.	A	g	100	*	TYPE=SUBSTITUTE
CM032587.1	44828	.	C	t	100	*	TYPE=SUBSTITUTE
CM032587.1	44833	.	G	GA	100	*	TYPE=INSERT
CM032587.1	44833	.	G	a	100	*	TYPE=SUBSTITUTE
CM032587.1	44834	.	A	G	100	*	TYPE=SUBSTITUTE
CM032587.1	44845	.	G	a	100	*	TYPE=SUBSTITUTE
CM032587.1	44847	.	T	c	100	*	TYPE=SUBSTITUTE
CM032587.1	44847	.	T	c	100	*	TYPE=SUBSTITUTE
CM032587.1	44851	.	G	a	100	*	TYPE=SUBSTITUTE
CM032587.1	44852	.	C	t	100	*	TYPE=SUBSTITUTE
CM032587.1	44860	.	A	g	100	*	TYPE=SUBSTITUTE
CM032587.1	44861	.	C	t	100	*	TYPE=SUBSTITUTE
CM032587.1	44861	.	C	t	100	*	TYPE=SUBSTITUTE
CM032587.1	44864	.	T	c	100	*	TYPE=SUBSTITUTE
CM032587.1	44864	.	T	c	100	*	TYPE=SUBSTITUTE
CM032587.1	44873	.	A	g	100	*	TYPE=SUBSTITUTE
CM032587.1	44873	.	A	g	100	*	TYPE=SUBSTITUTE
CM032587.1	44877	.	A	g	100	*	TYPE=SUBSTITUTE
CM032587.1	44878	.	A	g	100	*	TYPE=SUBSTITUTE
CM032587.1	44880	.	TC	T	100	*	TYPE=DELETE
CM032587.1	44902	.	A	g	100	*	TYPE=SUBSTITUTE
CM032587.1	44917	.	A	g	100	*	TYPE=SUBSTITUTE
CM032587.1	44917	.	A	g	100	*	TYPE=SUBSTITUTE
CM032587.1	44918	.	T	c	100	*	TYPE=SUBSTITUTE
CM032587.1	44918	.	T	c	100	*	TYPE=SUBSTITUTE
CM032587.1	44918	.	T	c	100	*	TYPE=SUBSTITUTE
CM032587.1	44927	.	G	a	100	*	TYPE=SUBSTITUTE
CM032587.1	44927	.	G	a	100	*	TYPE=SUBSTITUTE
CM032587.1	44934	.	TC	T	100	*	TYPE=DELETE
CM032587.1	44934	.	TC	T	100	*	TYPE=DELETE
CM032587.1	44935	.	C	t	100	*	TYPE=SUBSTITUTE
CM032587.1	44936	.	C	t	100	*	TYPE=SUBSTITUTE
CM032587.1	44946	.	C	t	100	*	TYPE=SUBSTITUTE
CM032587.1	44946	.	C	t	100	*	TYPE=SUBSTITUTE
CM032587.1	44947	.	C	t	100	*	TYPE=SUBSTITUTE
CM032587.1	44951	.	G	a	100	*	TYPE=SUBSTITUTE
CM032587.1	44951	.	G	a	100	*	TYPE=SUBSTITUTE
CM032587.1	44952	.	G	a	100	*	TYPE=SUBSTITUTE
CM032587.1	44954	.	AC	A	100	*	TYPE=DELETE
CM032587.1	44964	.	t	ta	100	*	TYPE=INSERT
CM032587.1	44969	.	G	a	100	*	TYPE=SUBSTITUTE
CM032587.1	44976	.	A	g	100	*	TYPE=SUBSTITUTE
CM032587.1	44976	.	A	g	100	*	TYPE=SUBSTITUTE
CM032587.1	44976	.	A	g	100	*	TYPE=SUBSTITUTE
CM032587.1	44978	.	C	t	100	*	TYPE=SUBSTITUTE
CM032587.1	44978	.	C	t	100	*	TYPE=SUBSTITUTE
CM032587.1	44978	.	C	t	100	*	TYPE=SUBSTITUTE
CM032587.1	44982	.	A	g	100	*	TYPE=SUBSTITUTE
CM032587.1	44982	.	A	g	100	*	TYPE=SUBSTITUTE
CM032587.1	44988	.	T	c	100	*	TYPE=SUBSTITUTE
CM032587.1	44989	.	T	c	100	*	TYPE=SUBSTITUTE
CM032587.1	44989	.	T	c	100	*	TYPE=SUBSTITUTE
CM032587.1	44991	.	T	a	100	*	TYPE=SUBSTITUTE
CM032587.1	44993	.	G	a	100	*	TYPE=SUBSTITUTE
CM032587.1	44993	.	G	c	100	*	TYPE=SUBSTITUTE
CM032587.1	44994	.	TTA	T	100	*	TYPE=DELETE
CM032587.1	45002	.	C	t	100	*	TYPE=SUBSTITUTE
CM032587.1	45002	.	C	t	100	*	TYPE=SUBSTITUTE

CM032587.1	45006	.	A	g	100	*	TYPE=SUBSTITUTE
CM032587.1	45006	.	A	g	100	*	TYPE=SUBSTITUTE
CM032587.1	45008	.	C	t	100	*	TYPE=SUBSTITUTE
CM032587.1	45008	.	C	t	100	*	TYPE=SUBSTITUTE
CM032587.1	45009	.	T	c	100	*	TYPE=SUBSTITUTE
CM032587.1	45012	.	AG	A	100	*	TYPE=DELETE
CM032587.1	45012	.	A	G	100	*	TYPE=SUBSTITUTE
CM032587.1	45013	.	G	a	100	*	TYPE=SUBSTITUTE
CM032587.1	45014	.	G	a	100	*	TYPE=SUBSTITUTE
CM032587.1	45015	.	A	G	100	*	TYPE=SUBSTITUTE
CM032587.1	45016	.	G	a	100	*	TYPE=SUBSTITUTE
CM032587.1	45016	.	G	a	100	*	TYPE=SUBSTITUTE
CM032587.1	45017	.	T	c	100	*	TYPE=SUBSTITUTE
CM032587.1	45022	.	G	a	100	*	TYPE=SUBSTITUTE
CM032587.1	45022	.	GT	G	100	*	TYPE=DELETE
CM032587.1	45024	.	T	c	100	*	TYPE=SUBSTITUTE
CM032587.1	45028	.	TC	T	100	*	TYPE=DELETE
CM032587.1	45028	.	TC	T	100	*	TYPE=DELETE
CM032587.1	45032	.	T	a	100	*	TYPE=SUBSTITUTE
CM032587.1	45036	.	G	a	100	*	TYPE=SUBSTITUTE
CM032587.1	45041	.	T	c	100	*	TYPE=SUBSTITUTE
CM032587.1	45041	.	T	c	100	*	TYPE=SUBSTITUTE
CM032587.1	45041	.	T	c	100	*	TYPE=SUBSTITUTE
CM032587.1	45043	.	T	c	100	*	TYPE=SUBSTITUTE
CM032587.1	45044	.	T	c	100	*	TYPE=SUBSTITUTE
CM032587.1	45055	.	A	g	100	*	TYPE=SUBSTITUTE
CM032587.1	45055	.	A	g	100	*	TYPE=SUBSTITUTE
CM032587.1	45060	.	G	a	100	*	TYPE=SUBSTITUTE
CM032587.1	45067	.	A	g	100	*	TYPE=SUBSTITUTE
CM032587.1	45067	.	A	g	100	*	TYPE=SUBSTITUTE
CM032587.1	45078	.	G	a	100	*	TYPE=SUBSTITUTE
CM032587.1	45086	.	A	g	100	*	TYPE=SUBSTITUTE
CM032587.1	45091	.	G	a	100	*	TYPE=SUBSTITUTE
CM032587.1	45093	.	T	c	100	*	TYPE=SUBSTITUTE
CM032587.1	45101	.	A	g	100	*	TYPE=SUBSTITUTE
CM032587.1	45101	.	A	g	100	*	TYPE=SUBSTITUTE
CM032587.1	45102	.	G	a	100	*	TYPE=SUBSTITUTE
CM032587.1	45108	.	G	a	100	*	TYPE=SUBSTITUTE
CM032587.1	45109	.	G	a	100	*	TYPE=SUBSTITUTE
CM032587.1	45109	.	G	a	100	*	TYPE=SUBSTITUTE
CM032587.1	45109	.	G	a	100	*	TYPE=SUBSTITUTE
CM032587.1	45111	.	A	g	100	*	TYPE=SUBSTITUTE
CM032587.1	45113	.	T	c	100	*	TYPE=SUBSTITUTE
CM032587.1	45128	.	T	c	100	*	TYPE=SUBSTITUTE
CM032587.1	45138	.	G	a	100	*	TYPE=SUBSTITUTE
CM032587.1	45138	.	G	a	100	*	TYPE=SUBSTITUTE
CM032587.1	45138	.	G	a	100	*	TYPE=SUBSTITUTE
CM032587.1	45139	.	G	a	100	*	TYPE=SUBSTITUTE
CM032587.1	45146	.	C	t	100	*	TYPE=SUBSTITUTE
CM032587.1	45146	.	C	t	100	*	TYPE=SUBSTITUTE
CM032587.1	45146	.	C	t	100	*	TYPE=SUBSTITUTE
CM032587.1	45156	.	C	t	100	*	TYPE=SUBSTITUTE
CM032587.1	45161	.	G	a	100	*	TYPE=SUBSTITUTE
CM032587.1	45162	.	C	t	100	*	TYPE=SUBSTITUTE
CM032587.1	45162	.	C	t	100	*	TYPE=SUBSTITUTE
CM032587.1	45169	.	G	a	100	*	TYPE=SUBSTITUTE
CM032587.1	45169	.	G	a	100	*	TYPE=SUBSTITUTE
CM032587.1	45170	.	C	t	100	*	TYPE=SUBSTITUTE
CM032587.1	45170	.	C	t	100	*	TYPE=SUBSTITUTE
CM032587.1	45170	.	C	t	100	*	TYPE=SUBSTITUTE
CM032587.1	45172	.	G	a	100	*	TYPE=SUBSTITUTE
CM032587.1	45176	.	C	t	100	*	TYPE=SUBSTITUTE
CM032587.1	45176	.	C	t	100	*	TYPE=SUBSTITUTE
CM032587.1	45177	.	C	t	100	*	TYPE=SUBSTITUTE

CM032587.1	45177	.	C	t	100	*	TYPE=SUBSTITUTE
CM032587.1	45187	.	A	g	100	*	TYPE=SUBSTITUTE
CM032587.1	45187	.	A	g	100	*	TYPE=SUBSTITUTE
CM032587.1	45191	.	C	t	100	*	TYPE=SUBSTITUTE
CM032587.1	45191	.	C	t	100	*	TYPE=SUBSTITUTE
CM032587.1	45194	.	C	t	100	*	TYPE=SUBSTITUTE
CM032587.1	45194	.	C	t	100	*	TYPE=SUBSTITUTE
CM032587.1	45194	.	C	t	100	*	TYPE=SUBSTITUTE
CM032587.1	45239	.	A	g	100	*	TYPE=SUBSTITUTE
CM032587.1	45239	.	A	g	100	*	TYPE=SUBSTITUTE
CM032587.1	45248	.	G	a	100	*	TYPE=SUBSTITUTE
CM032587.1	45254	.	G	a	100	*	TYPE=SUBSTITUTE
CM032587.1	45254	.	G	a	100	*	TYPE=SUBSTITUTE
CM032587.1	45255	.	G	a	100	*	TYPE=SUBSTITUTE
CM032587.1	45255	.	G	a	100	*	TYPE=SUBSTITUTE
CM032587.1	45256	.	C	T	100	*	TYPE=SUBSTITUTE
CM032587.1	45256	.	C	t	100	*	TYPE=SUBSTITUTE
CM032587.1	45256	.	C	t	100	*	TYPE=SUBSTITUTE
CM032587.1	45259	.	AAG	A	100	*	TYPE=DELETE
CM032587.1	45260	.	A	G	100	*	TYPE=SUBSTITUTE
CM032587.1	45261	.	G	a	100	*	TYPE=SUBSTITUTE
CM032587.1	45262	.	A	G	100	*	TYPE=SUBSTITUTE
CM032587.1	45264	.	A	g	100	*	TYPE=SUBSTITUTE
CM032587.1	45267	.	A	g	100	*	TYPE=SUBSTITUTE
CM032587.1	45267	.	A	g	100	*	TYPE=SUBSTITUTE
CM032587.1	45267	.	A	g	100	*	TYPE=SUBSTITUTE
CM032587.1	45279	.	A	g	100	*	TYPE=SUBSTITUTE
CM032587.1	45279	.	A	g	100	*	TYPE=SUBSTITUTE
CM032587.1	45287	.	AG	A	100	*	TYPE=DELETE
CM032587.1	45288	.	G	a	100	*	TYPE=SUBSTITUTE
CM032587.1	45289	.	A	g	100	*	TYPE=SUBSTITUTE
CM032587.1	45297	.	A	g	100	*	TYPE=SUBSTITUTE
CM032587.1	45298	.	A	g	100	*	TYPE=SUBSTITUTE
CM032587.1	45298	.	A	g	100	*	TYPE=SUBSTITUTE
CM032587.1	45301	.	T	C	100	*	TYPE=SUBSTITUTE
CM032587.1	45309	.	A	G	100	*	TYPE=SUBSTITUTE
CM032587.1	45310	.	G	a	100	*	TYPE=SUBSTITUTE
CM032587.1	45323	.	G	a	100	*	TYPE=SUBSTITUTE
CM032587.1	45333	.	TA	T	100	*	TYPE=DELETE
CM032587.1	45339	.	A	g	100	*	TYPE=SUBSTITUTE
CM032587.1	45340	.	G	a	100	*	TYPE=SUBSTITUTE
CM032587.1	45345	.	T	c	100	*	TYPE=SUBSTITUTE
CM032587.1	45346	.	C	t	100	*	TYPE=SUBSTITUTE
CM032587.1	45351	.	G	A	100	*	TYPE=SUBSTITUTE
CM032587.1	45352	.	C	t	100	*	TYPE=SUBSTITUTE
CM032587.1	45357	.	T	c	100	*	TYPE=SUBSTITUTE
CM032587.1	45365	.	A	g	100	*	TYPE=SUBSTITUTE
CM032587.1	45366	.	G	a	100	*	TYPE=SUBSTITUTE
CM032587.1	45366	.	G	a	100	*	TYPE=SUBSTITUTE
CM032587.1	45369	.	C	t	100	*	TYPE=SUBSTITUTE
CM032587.1	45369	.	C	t	100	*	TYPE=SUBSTITUTE
CM032587.1	45369	.	C	t	100	*	TYPE=SUBSTITUTE
CM032587.1	45379	.	C	t	100	*	TYPE=SUBSTITUTE
CM032587.1	45379	.	C	t	100	*	TYPE=SUBSTITUTE
CM032587.1	45379	.	C	t	100	*	TYPE=SUBSTITUTE
CM032587.1	45387	.	A	g	100	*	TYPE=SUBSTITUTE
CM032587.1	45390	.	A	g	100	*	TYPE=SUBSTITUTE
CM032587.1	45395	.	C	t	100	*	TYPE=SUBSTITUTE
CM032587.1	45395	.	C	t	100	*	TYPE=SUBSTITUTE
CM032587.1	45411	.	A	g	100	*	TYPE=SUBSTITUTE
CM032587.1	45415	.	AG	A	100	*	TYPE=DELETE
CM032587.1	45415	.	AG	A	100	*	TYPE=DELETE
CM032587.1	45415	.	a	aa	100	*	TYPE=INSERT
CM032587.1	45416	.	G	a	100	*	TYPE=SUBSTITUTE

CM032587.1	45417	.	C	t	100	*	TYPE=SUBSTITUTE
CM032587.1	45420	.	C	t	100	*	TYPE=SUBSTITUTE
CM032587.1	45427	.	TC	T	100	*	TYPE=DELETE
CM032587.1	45429	.	C	t	100	*	TYPE=SUBSTITUTE
CM032587.1	45429	.	C	t	100	*	TYPE=SUBSTITUTE
CM032587.1	45430	.	T	c	100	*	TYPE=SUBSTITUTE
CM032587.1	45447	.	A	g	100	*	TYPE=SUBSTITUTE
CM032587.1	45447	.	A	g	100	*	TYPE=SUBSTITUTE
CM032587.1	45471	.	A	g	100	*	TYPE=SUBSTITUTE
CM032587.1	45472	.	A	g	100	*	TYPE=SUBSTITUTE
CM032587.1	45472	.	A	g	100	*	TYPE=SUBSTITUTE
CM032587.1	45475	.	C	t	100	*	TYPE=SUBSTITUTE
CM032587.1	45475	.	C	t	100	*	TYPE=SUBSTITUTE
CM032587.1	45475	.	C	t	100	*	TYPE=SUBSTITUTE
CM032587.1	45480	.	A	g	100	*	TYPE=SUBSTITUTE
CM032587.1	45488	.	G	a	100	*	TYPE=SUBSTITUTE
CM032587.1	45488	.	G	a	100	*	TYPE=SUBSTITUTE
CM032587.1	45488	.	G	a	100	*	TYPE=SUBSTITUTE
CM032587.1	45491	.	TA	T	100	*	TYPE=DELETE
CM032587.1	45494	.	A	g	100	*	TYPE=SUBSTITUTE
CM032587.1	45495	.	A	g	100	*	TYPE=SUBSTITUTE
CM032587.1	45497	.	G	a	100	*	TYPE=SUBSTITUTE
CM032587.1	45498	.	G	a	100	*	TYPE=SUBSTITUTE
CM032587.1	45499	.	A	g	100	*	TYPE=SUBSTITUTE
CM032587.1	45501	.	A	g	100	*	TYPE=SUBSTITUTE
CM032587.1	45510	.	G	a	100	*	TYPE=SUBSTITUTE
CM032587.1	45510	.	G	a	100	*	TYPE=SUBSTITUTE
CM032587.1	45511	.	C	t	100	*	TYPE=SUBSTITUTE
CM032587.1	45512	.	T	c	100	*	TYPE=SUBSTITUTE
CM032587.1	45512	.	T	c	100	*	TYPE=SUBSTITUTE
CM032587.1	45512	.	T	c	100	*	TYPE=SUBSTITUTE
CM032587.1	45513	.	T	c	100	*	TYPE=SUBSTITUTE
CM032587.1	45518	.	C	T	100	*	TYPE=SUBSTITUTE
CM032587.1	45518	.	C	t	100	*	TYPE=SUBSTITUTE
CM032587.1	45519	.	t	ta	100	*	TYPE=INSERT
CM032587.1	45522	.	G	a	100	*	TYPE=SUBSTITUTE
CM032587.1	45522	.	G	a	100	*	TYPE=SUBSTITUTE
CM032587.1	45523	.	G	a	100	*	TYPE=SUBSTITUTE
CM032587.1	45527	.	C	t	100	*	TYPE=SUBSTITUTE
CM032587.1	45528	.	C	t	100	*	TYPE=SUBSTITUTE
CM032587.1	45528	.	C	t	100	*	TYPE=SUBSTITUTE
CM032587.1	45528	.	C	t	100	*	TYPE=SUBSTITUTE
CM032587.1	45532	.	A	g	100	*	TYPE=SUBSTITUTE
CM032587.1	45532	.	A	g	100	*	TYPE=SUBSTITUTE
CM032587.1	45532	.	A	g	100	*	TYPE=SUBSTITUTE
CM032587.1	45542	.	G	a	100	*	TYPE=SUBSTITUTE
CM032587.1	45542	.	G	a	100	*	TYPE=SUBSTITUTE
CM032587.1	45542	.	G	a	100	*	TYPE=SUBSTITUTE
CM032587.1	45546	.	G	a	100	*	TYPE=SUBSTITUTE
CM032587.1	45546	.	G	a	100	*	TYPE=SUBSTITUTE
CM032587.1	45550	.	A	g	100	*	TYPE=SUBSTITUTE
CM032587.1	45550	.	A	g	100	*	TYPE=SUBSTITUTE
CM032587.1	45550	.	A	g	100	*	TYPE=SUBSTITUTE
CM032587.1	45554	.	C	t	100	*	TYPE=SUBSTITUTE
CM032587.1	45554	.	C	t	100	*	TYPE=SUBSTITUTE
CM032587.1	45554	.	C	t	100	*	TYPE=SUBSTITUTE
CM032587.1	45554	.	C	t	100	*	TYPE=SUBSTITUTE
CM032587.1	45558	.	G	a	100	*	TYPE=SUBSTITUTE
CM032587.1	45558	.	G	a	100	*	TYPE=SUBSTITUTE
CM032587.1	45569	.	AC	A	100	*	TYPE=DELETE
CM032587.1	45570	.	c	ctt	100	*	TYPE=INSERT
CM032587.1	45573	.	T	c	100	*	TYPE=SUBSTITUTE
CM032587.1	45574	.	TT	T	100	*	TYPE=DELETE
CM032587.1	45574	.	T	c	100	*	TYPE=SUBSTITUTE

CM032587.1	45575	.	T	a	100	*	TYPE=SUBSTITUTE
CM032587.1	45576	.	T	a	100	*	TYPE=SUBSTITUTE
CM032587.1	45576	.	TA	T	100	*	TYPE=DELETE
CM032587.1	45576	.	T	a	100	*	TYPE=SUBSTITUTE
CM032587.1	45577	.	A	t	100	*	TYPE=SUBSTITUTE
CM032587.1	45582	.	A	g	100	*	TYPE=SUBSTITUTE
CM032587.1	45582	.	A	g	100	*	TYPE=SUBSTITUTE
CM032587.1	45584	.	C	t	100	*	TYPE=SUBSTITUTE
CM032587.1	45585	.	C	t	100	*	TYPE=SUBSTITUTE
CM032587.1	45585	.	C	t	100	*	TYPE=SUBSTITUTE
CM032587.1	45585	.	C	t	100	*	TYPE=SUBSTITUTE
CM032587.1	45591	.	G	a	100	*	TYPE=SUBSTITUTE
CM032587.1	45605	.	G	a	100	*	TYPE=SUBSTITUTE
CM032587.1	45629	.	ATT	A	100	*	TYPE=DELETE
CM032587.1	45629	.	ATT	A	100	*	TYPE=DELETE
CM032587.1	45630	.	T	g	100	*	TYPE=SUBSTITUTE
CM032587.1	45630	.	T	a	100	*	TYPE=SUBSTITUTE
CM032587.1	45631	.	T	g	100	*	TYPE=SUBSTITUTE
CM032587.1	45631	.	T	a	100	*	TYPE=SUBSTITUTE
CM032587.1	45632	.	T	g	100	*	TYPE=SUBSTITUTE
CM032587.1	45632	.	T	a	100	*	TYPE=SUBSTITUTE
CM032587.1	45632	.	T	a	100	*	TYPE=SUBSTITUTE
CM032587.1	45632	.	T	a	100	*	TYPE=SUBSTITUTE
CM032587.1	45633	.	T	a	100	*	TYPE=SUBSTITUTE
CM032587.1	45633	.	T	a	100	*	TYPE=SUBSTITUTE
CM032587.1	45633	.	T	a	100	*	TYPE=SUBSTITUTE
CM032587.1	45633	.	T	a	100	*	TYPE=SUBSTITUTE
CM032587.1	45634	.	T	a	100	*	TYPE=SUBSTITUTE
CM032587.1	45634	.	T	a	100	*	TYPE=SUBSTITUTE
CM032587.1	45634	.	T	g	100	*	TYPE=SUBSTITUTE
CM032587.1	45634	.	T	c	100	*	TYPE=SUBSTITUTE
CM032587.1	45635	.	T	a	100	*	TYPE=SUBSTITUTE
CM032587.1	45635	.	T	c	100	*	TYPE=SUBSTITUTE
CM032587.1	45635	.	T	c	100	*	TYPE=SUBSTITUTE
CM032587.1	45636	.	T	c	100	*	TYPE=SUBSTITUTE
CM032587.1	45636	.	T	c	100	*	TYPE=SUBSTITUTE
CM032587.1	45637	.	T	c	100	*	TYPE=SUBSTITUTE
CM032587.1	45644	.	T	c	100	*	TYPE=SUBSTITUTE
CM032587.1	45648	.	A	g	100	*	TYPE=SUBSTITUTE
CM032587.1	45650	.	A	g	100	*	TYPE=SUBSTITUTE
CM032587.1	45650	.	A	g	100	*	TYPE=SUBSTITUTE
CM032587.1	45652	.	T	c	100	*	TYPE=SUBSTITUTE
CM032587.1	45652	.	T	C	100	*	TYPE=SUBSTITUTE
CM032587.1	45653	.	TCTTA	T	100	*	TYPE=DELETE
CM032587.1	45654	.	C	t	100	*	TYPE=SUBSTITUTE
CM032587.1	45654	.	C	t	100	*	TYPE=SUBSTITUTE
CM032587.1	45658	.	A	t	100	*	TYPE=SUBSTITUTE
CM032587.1	45659	.	A	G	100	*	TYPE=SUBSTITUTE
CM032587.1	45659	.	A	c	100	*	TYPE=SUBSTITUTE
CM032587.1	45660	.	G	a	100	*	TYPE=SUBSTITUTE
CM032587.1	45661	.	T	c	100	*	TYPE=SUBSTITUTE
CM032587.1	45661	.	T	c	100	*	TYPE=SUBSTITUTE
CM032587.1	45667	.	C	t	100	*	TYPE=SUBSTITUTE
CM032587.1	45675	.	A	g	100	*	TYPE=SUBSTITUTE
CM032587.1	45678	.	A	g	100	*	TYPE=SUBSTITUTE
CM032587.1	45683	.	A	G	100	*	TYPE=SUBSTITUTE
CM032587.1	45684	.	A	g	100	*	TYPE=SUBSTITUTE
CM032587.1	45684	.	A	g	100	*	TYPE=SUBSTITUTE
CM032587.1	45685	.	A	g	100	*	TYPE=SUBSTITUTE
CM032587.1	45685	.	A	g	100	*	TYPE=SUBSTITUTE
CM032587.1	45685	.	A	g	100	*	TYPE=SUBSTITUTE
CM032587.1	45687	.	G	a	100	*	TYPE=SUBSTITUTE
CM032587.1	45687	.	G	a	100	*	TYPE=SUBSTITUTE
CM032587.1	45687	.	G	a	100	*	TYPE=SUBSTITUTE

CM032587.1	45687	.	G	a	100	*	TYPE=SUBSTITUTE
CM032587.1	45691	.	A	g	100	*	TYPE=SUBSTITUTE
CM032587.1	45692	.	G	a	100	*	TYPE=SUBSTITUTE
CM032587.1	45694	.	C	t	100	*	TYPE=SUBSTITUTE
CM032587.1	45694	.	C	t	100	*	TYPE=SUBSTITUTE
CM032587.1	45694	.	C	t	100	*	TYPE=SUBSTITUTE
CM032587.1	45694	.	C	t	100	*	TYPE=SUBSTITUTE
CM032587.1	45697	.	t	ta	100	*	TYPE=INSERT
CM032587.1	45699	.	G	a	100	*	TYPE=SUBSTITUTE
CM032587.1	45699	.	G	a	100	*	TYPE=SUBSTITUTE
CM032587.1	45700	.	C	t	100	*	TYPE=SUBSTITUTE
CM032587.1	45701	.	T	c	100	*	TYPE=SUBSTITUTE
CM032587.1	45701	.	T	c	100	*	TYPE=SUBSTITUTE
CM032587.1	45712	.	G	a	100	*	TYPE=SUBSTITUTE
CM032587.1	45712	.	G	a	100	*	TYPE=SUBSTITUTE
CM032587.1	45718	.	AC	A	100	*	TYPE=DELETE
CM032587.1	45719	.	C	t	100	*	TYPE=SUBSTITUTE
CM032587.1	45719	.	c	cttt	100	*	TYPE=INSERT
CM032587.1	45723	.	T	c	100	*	TYPE=SUBSTITUTE
CM032587.1	45723	.	T	c	100	*	TYPE=SUBSTITUTE
CM032587.1	45728	.	G	a	100	*	TYPE=SUBSTITUTE
CM032587.1	45728	.	G	a	100	*	TYPE=SUBSTITUTE
CM032587.1	45728	.	G	a	100	*	TYPE=SUBSTITUTE
CM032587.1	45729	.	T	c	100	*	TYPE=SUBSTITUTE
CM032587.1	45729	.	T	c	100	*	TYPE=SUBSTITUTE
CM032587.1	45729	.	T	c	100	*	TYPE=SUBSTITUTE
CM032587.1	45731	.	TC	T	100	*	TYPE=DELETE
CM032587.1	45732	.	C	t	100	*	TYPE=SUBSTITUTE
CM032587.1	45732	.	CT	C	100	*	TYPE=DELETE
CM032587.1	45733	.	T	C	100	*	TYPE=SUBSTITUTE
CM032587.1	45736	.	G	a	100	*	TYPE=SUBSTITUTE
CM032587.1	45736	.	G	A	100	*	TYPE=SUBSTITUTE
CM032587.1	45736	.	G	a	100	*	TYPE=SUBSTITUTE
CM032587.1	45736	.	G	a	100	*	TYPE=SUBSTITUTE
CM032587.1	45737	.	G	a	100	*	TYPE=SUBSTITUTE
CM032587.1	45742	.	A	g	100	*	TYPE=SUBSTITUTE
CM032587.1	45743	.	G	a	100	*	TYPE=SUBSTITUTE
CM032587.1	45743	.	G	a	100	*	TYPE=SUBSTITUTE
CM032587.1	45744	.	G	a	100	*	TYPE=SUBSTITUTE
CM032587.1	45744	.	G	a	100	*	TYPE=SUBSTITUTE
CM032587.1	45744	.	G	a	100	*	TYPE=SUBSTITUTE
CM032587.1	45758	.	T	c	100	*	TYPE=SUBSTITUTE
CM032587.1	45763	.	C	t	100	*	TYPE=SUBSTITUTE
CM032587.1	45763	.	C	t	100	*	TYPE=SUBSTITUTE
CM032587.1	45763	.	C	t	100	*	TYPE=SUBSTITUTE
CM032587.1	45766	.	TAG	T	100	*	TYPE=DELETE
CM032587.1	45767	.	AG	A	100	*	TYPE=DELETE
CM032587.1	45767	.	AG	A	100	*	TYPE=DELETE
CM032587.1	45773	.	A	g	100	*	TYPE=SUBSTITUTE
CM032587.1	45773	.	A	G	100	*	TYPE=SUBSTITUTE
CM032587.1	45776	.	A	g	100	*	TYPE=SUBSTITUTE
CM032587.1	45776	.	A	G	100	*	TYPE=SUBSTITUTE
CM032587.1	45779	.	A	G	100	*	TYPE=SUBSTITUTE
CM032587.1	45780	.	A	g	100	*	TYPE=SUBSTITUTE
CM032587.1	45780	.	A	G	100	*	TYPE=SUBSTITUTE
CM032587.1	45780	.	A	g	100	*	TYPE=SUBSTITUTE
CM032587.1	45783	.	A	g	100	*	TYPE=SUBSTITUTE
CM032587.1	45783	.	A	g	100	*	TYPE=SUBSTITUTE
CM032587.1	45786	.	T	c	100	*	TYPE=SUBSTITUTE
CM032587.1	45791	.	T	c	100	*	TYPE=SUBSTITUTE
CM032587.1	45791	.	T	c	100	*	TYPE=SUBSTITUTE
CM032587.1	45791	.	T	c	100	*	TYPE=SUBSTITUTE
CM032587.1	45798	.	G	a	100	*	TYPE=SUBSTITUTE
CM032587.1	45798	.	G	a	100	*	TYPE=SUBSTITUTE

CM032587.1	45810	.	A	g	100	*	TYPE=SUBSTITUTE
CM032587.1	45810	.	A	g	100	*	TYPE=SUBSTITUTE
CM032587.1	45811	.	AG	A	100	*	TYPE=DELETE
CM032587.1	45812	.	G	a	100	*	TYPE=SUBSTITUTE
CM032587.1	45813	.	A	g	100	*	TYPE=SUBSTITUTE
CM032587.1	45813	.	A	g	100	*	TYPE=SUBSTITUTE
CM032587.1	45813	.	A	g	100	*	TYPE=SUBSTITUTE
CM032587.1	45817	.	G	a	100	*	TYPE=SUBSTITUTE
CM032587.1	45817	.	G	a	100	*	TYPE=SUBSTITUTE
CM032587.1	45817	.	G	a	100	*	TYPE=SUBSTITUTE
CM032587.1	45819	.	A	g	100	*	TYPE=SUBSTITUTE
CM032587.1	45829	.	C	t	100	*	TYPE=SUBSTITUTE
CM032587.1	45829	.	C	t	100	*	TYPE=SUBSTITUTE
CM032587.1	45835	.	A	g	100	*	TYPE=SUBSTITUTE
CM032587.1	45835	.	A	g	100	*	TYPE=SUBSTITUTE
CM032587.1	45835	.	A	g	100	*	TYPE=SUBSTITUTE
CM032587.1	45838	.	A	g	100	*	TYPE=SUBSTITUTE
CM032587.1	45839	.	G	a	100	*	TYPE=SUBSTITUTE
CM032587.1	45839	.	G	a	100	*	TYPE=SUBSTITUTE
CM032587.1	45839	.	G	a	100	*	TYPE=SUBSTITUTE
CM032587.1	45840	.	C	t	100	*	TYPE=SUBSTITUTE
CM032587.1	45840	.	C	t	100	*	TYPE=SUBSTITUTE
CM032587.1	45844	.	G	a	100	*	TYPE=SUBSTITUTE
CM032587.1	45857	.	A	g	100	*	TYPE=SUBSTITUTE
CM032587.1	45857	.	A	g	100	*	TYPE=SUBSTITUTE
CM032587.1	45858	.	A	g	100	*	TYPE=SUBSTITUTE
CM032587.1	45858	.	A	g	100	*	TYPE=SUBSTITUTE
CM032587.1	45858	.	A	g	100	*	TYPE=SUBSTITUTE
CM032587.1	45858	.	A	g	100	*	TYPE=SUBSTITUTE
CM032587.1	45859	.	A	g	100	*	TYPE=SUBSTITUTE
CM032587.1	45859	.	A	g	100	*	TYPE=SUBSTITUTE
CM032587.1	45859	.	A	g	100	*	TYPE=SUBSTITUTE
CM032587.1	45859	.	A	g	100	*	TYPE=SUBSTITUTE
CM032587.1	45871	.	T	c	100	*	TYPE=SUBSTITUTE
CM032587.1	45884	.	G	a	100	*	TYPE=SUBSTITUTE
CM032587.1	45889	.	A	g	100	*	TYPE=SUBSTITUTE
CM032587.1	45895	.	C	t	100	*	TYPE=SUBSTITUTE
CM032587.1	45895	.	C	t	100	*	TYPE=SUBSTITUTE
CM032587.1	45900	.	C	t	100	*	TYPE=SUBSTITUTE
CM032587.1	45901	.	C	t	100	*	TYPE=SUBSTITUTE
CM032587.1	45901	.	C	t	100	*	TYPE=SUBSTITUTE
CM032587.1	45901	.	C	t	100	*	TYPE=SUBSTITUTE
CM032587.1	45901	.	C	t	100	*	TYPE=SUBSTITUTE
CM032587.1	45910	.	G	a	100	*	TYPE=SUBSTITUTE
CM032587.1	45910	.	G	a	100	*	TYPE=SUBSTITUTE
CM032587.1	45911	.	CT	C	100	*	TYPE=DELETE
CM032587.1	45919	.	A	g	100	*	TYPE=SUBSTITUTE
CM032587.1	45921	.	C	t	100	*	TYPE=SUBSTITUTE
CM032587.1	45921	.	C	t	100	*	TYPE=SUBSTITUTE
CM032587.1	45926	.	G	a	100	*	TYPE=SUBSTITUTE
CM032587.1	45930	.	T	a	100	*	TYPE=SUBSTITUTE
CM032587.1	45942	.	G	a	100	*	TYPE=SUBSTITUTE
CM032587.1	45942	.	G	a	100	*	TYPE=SUBSTITUTE
CM032587.1	45942	.	G	a	100	*	TYPE=SUBSTITUTE
CM032587.1	45942	.	G	a	100	*	TYPE=SUBSTITUTE
CM032587.1	45944	.	T	c	100	*	TYPE=SUBSTITUTE
CM032587.1	45944	.	T	c	100	*	TYPE=SUBSTITUTE
CM032587.1	45948	.	G	a	100	*	TYPE=SUBSTITUTE
CM032587.1	45971	.	A	g	100	*	TYPE=SUBSTITUTE
CM032587.1	45974	.	T	c	100	*	TYPE=SUBSTITUTE
CM032587.1	45974	.	T	c	100	*	TYPE=SUBSTITUTE
CM032587.1	45974	.	T	c	100	*	TYPE=SUBSTITUTE
CM032587.1	45975	.	T	c	100	*	TYPE=SUBSTITUTE
CM032587.1	45975	.	T	c	100	*	TYPE=SUBSTITUTE

CM032587.1	45980	.	T	C	100	*	TYPE=SUBSTITUTE
CM032587.1	45980	.	T	C	100	*	TYPE=SUBSTITUTE
CM032587.1	45981	.	T	C	100	*	TYPE=SUBSTITUTE
CM032587.1	45981	.	T	C	100	*	TYPE=SUBSTITUTE
CM032587.1	45986	.	A	G	100	*	TYPE=SUBSTITUTE
CM032587.1	45986	.	A	G	100	*	TYPE=SUBSTITUTE
CM032587.1	45998	.	T	C	100	*	TYPE=SUBSTITUTE
CM032587.1	45998	.	T	c	100	*	TYPE=SUBSTITUTE
CM032587.1	45998	.	T	c	100	*	TYPE=SUBSTITUTE
CM032587.1	45999	.	T	C	100	*	TYPE=SUBSTITUTE
CM032587.1	46000	.	t	tt	100	*	TYPE=INSERT
CM032587.1	46001	.	C	t	100	*	TYPE=SUBSTITUTE
CM032587.1	46001	.	C	t	100	*	TYPE=SUBSTITUTE
CM032587.1	46001	.	C	t	100	*	TYPE=SUBSTITUTE
CM032587.1	46001	.	C	t	100	*	TYPE=SUBSTITUTE
CM032587.1	46002	.	C	t	100	*	TYPE=SUBSTITUTE
CM032587.1	46002	.	C	t	100	*	TYPE=SUBSTITUTE
CM032587.1	46002	.	C	t	100	*	TYPE=SUBSTITUTE
CM032587.1	46002	.	C	t	100	*	TYPE=SUBSTITUTE
CM032587.1	46005	.	T	a	100	*	TYPE=SUBSTITUTE
CM032587.1	46005	.	T	a	100	*	TYPE=SUBSTITUTE
CM032587.1	46005	.	T	a	100	*	TYPE=SUBSTITUTE
CM032587.1	46011	.	T	c	100	*	TYPE=SUBSTITUTE
CM032587.1	46017	.	C	t	100	*	TYPE=SUBSTITUTE
CM032587.1	46022	.	G	a	100	*	TYPE=SUBSTITUTE
CM032587.1	46023	.	G	a	100	*	TYPE=SUBSTITUTE
CM032587.1	46025	.	A	g	100	*	TYPE=SUBSTITUTE
CM032587.1	46025	.	A	g	100	*	TYPE=SUBSTITUTE
CM032587.1	46025	.	A	g	100	*	TYPE=SUBSTITUTE
CM032587.1	46031	.	T	c	100	*	TYPE=SUBSTITUTE
CM032587.1	46032	.	T	c	100	*	TYPE=SUBSTITUTE
CM032587.1	46037	.	A	g	100	*	TYPE=SUBSTITUTE
CM032587.1	46040	.	AG	A	100	*	TYPE=DELETE
CM032587.1	46040	.	AG	A	100	*	TYPE=DELETE
CM032587.1	46041	.	G	a	100	*	TYPE=SUBSTITUTE
CM032587.1	46041	.	G	a	100	*	TYPE=SUBSTITUTE
CM032587.1	46046	.	T	c	100	*	TYPE=SUBSTITUTE
CM032587.1	46046	.	T	c	100	*	TYPE=SUBSTITUTE
CM032587.1	46051	.	TC	T	100	*	TYPE=DELETE
CM032587.1	46052	.	C	t	100	*	TYPE=SUBSTITUTE
CM032587.1	46052	.	C	t	100	*	TYPE=SUBSTITUTE
CM032587.1	46052	.	C	t	100	*	TYPE=SUBSTITUTE
CM032587.1	46059	.	G	a	100	*	TYPE=SUBSTITUTE
CM032587.1	46069	.	C	t	100	*	TYPE=SUBSTITUTE
CM032587.1	46078	.	T	c	100	*	TYPE=SUBSTITUTE
CM032587.1	46089	.	A	G	100	*	TYPE=SUBSTITUTE
CM032587.1	46090	.	G	a	100	*	TYPE=SUBSTITUTE
CM032587.1	46090	.	G	a	100	*	TYPE=SUBSTITUTE
CM032587.1	46090	.	G	a	100	*	TYPE=SUBSTITUTE
CM032587.1	46091	.	G	a	100	*	TYPE=SUBSTITUTE
CM032587.1	46091	.	G	a	100	*	TYPE=SUBSTITUTE
CM032587.1	46091	.	G	a	100	*	TYPE=SUBSTITUTE
CM032587.1	46092	.	G	a	100	*	TYPE=SUBSTITUTE
CM032587.1	46092	.	G	a	100	*	TYPE=SUBSTITUTE
CM032587.1	46092	.	G	a	100	*	TYPE=SUBSTITUTE
CM032587.1	46093	.	G	a	100	*	TYPE=SUBSTITUTE
CM032587.1	46093	.	G	a	100	*	TYPE=SUBSTITUTE
CM032587.1	46093	.	G	a	100	*	TYPE=SUBSTITUTE
CM032587.1	46097	.	G	a	100	*	TYPE=SUBSTITUTE
CM032587.1	46097	.	G	a	100	*	TYPE=SUBSTITUTE
CM032587.1	46097	.	G	a	100	*	TYPE=SUBSTITUTE
CM032587.1	46104	.	G	a	100	*	TYPE=SUBSTITUTE
CM032587.1	46104	.	G	a	100	*	TYPE=SUBSTITUTE
CM032587.1	46107	.	G	a	100	*	TYPE=SUBSTITUTE

CM032587.1	46107	.	G	a	100	*	TYPE=SUBSTITUTE
CM032587.1	46109	.	C	t	100	*	TYPE=SUBSTITUTE
CM032587.1	46109	.	C	t	100	*	TYPE=SUBSTITUTE
CM032587.1	46109	.	C	t	100	*	TYPE=SUBSTITUTE
CM032587.1	46115	.	C	t	100	*	TYPE=SUBSTITUTE
CM032587.1	46116	.	C	t	100	*	TYPE=SUBSTITUTE
CM032587.1	46116	.	C	t	100	*	TYPE=SUBSTITUTE
CM032587.1	46116	.	C	t	100	*	TYPE=SUBSTITUTE
CM032587.1	46116	.	C	t	100	*	TYPE=SUBSTITUTE
CM032587.1	46122	.	T	c	100	*	TYPE=SUBSTITUTE
CM032587.1	46125	.	c	ct	100	*	TYPE=INSERT
CM032587.1	46125	.	C	t	100	*	TYPE=SUBSTITUTE
CM032587.1	46129	.	A	g	100	*	TYPE=SUBSTITUTE
CM032587.1	46129	.	A	g	100	*	TYPE=SUBSTITUTE
CM032587.1	46130	.	T	c	100	*	TYPE=SUBSTITUTE
CM032587.1	46133	.	C	t	100	*	TYPE=SUBSTITUTE
CM032587.1	46133	.	C	t	100	*	TYPE=SUBSTITUTE
CM032587.1	46133	.	C	t	100	*	TYPE=SUBSTITUTE
CM032587.1	46133	.	C	t	100	*	TYPE=SUBSTITUTE
CM032587.1	46137	.	A	t	100	*	TYPE=SUBSTITUTE
CM032587.1	46137	.	A	t	100	*	TYPE=SUBSTITUTE
CM032587.1	46137	.	A	t	100	*	TYPE=SUBSTITUTE
CM032587.1	46137	.	A	t	100	*	TYPE=SUBSTITUTE
CM032587.1	46140	.	T	a	100	*	TYPE=SUBSTITUTE
CM032587.1	46140	.	T	a	100	*	TYPE=SUBSTITUTE
CM032587.1	46140	.	T	a	100	*	TYPE=SUBSTITUTE
CM032587.1	46140	.	T	a	100	*	TYPE=SUBSTITUTE
CM032587.1	46156	.	T	c	100	*	TYPE=SUBSTITUTE
CM032587.1	46159	.	G	a	100	*	TYPE=SUBSTITUTE
CM032587.1	46166	.	T	c	100	*	TYPE=SUBSTITUTE
CM032587.1	46186	.	G	a	100	*	TYPE=SUBSTITUTE
CM032587.1	46186	.	G	a	100	*	TYPE=SUBSTITUTE
CM032587.1	46187	.	C	t	100	*	TYPE=SUBSTITUTE
CM032587.1	46213	.	T	c	100	*	TYPE=SUBSTITUTE
CM032587.1	46213	.	TT	T	100	*	TYPE=DELETE
CM032587.1	46218	.	G	a	100	*	TYPE=SUBSTITUTE
CM032587.1	46218	.	G	a	100	*	TYPE=SUBSTITUTE
CM032587.1	46228	.	G	a	100	*	TYPE=SUBSTITUTE
CM032587.1	46228	.	G	a	100	*	TYPE=SUBSTITUTE
CM032587.1	46229	.	CT	C	100	*	TYPE=DELETE
CM032587.1	46229	.	CT	C	100	*	TYPE=DELETE
CM032587.1	46231	.	T	C	100	*	TYPE=SUBSTITUTE
CM032587.1	46231	.	T	c	100	*	TYPE=SUBSTITUTE
CM032587.1	46232	.	T	C	100	*	TYPE=SUBSTITUTE
CM032587.1	46240	.	A	G	100	*	TYPE=SUBSTITUTE
CM032587.1	46240	.	A	g	100	*	TYPE=SUBSTITUTE
CM032587.1	46245	.	A	g	100	*	TYPE=SUBSTITUTE
CM032587.1	46245	.	A	g	100	*	TYPE=SUBSTITUTE
CM032587.1	46245	.	A	G	100	*	TYPE=SUBSTITUTE
CM032587.1	46245	.	A	g	100	*	TYPE=SUBSTITUTE
CM032587.1	46248	.	A	g	100	*	TYPE=SUBSTITUTE
CM032587.1	46248	.	A	g	100	*	TYPE=SUBSTITUTE
CM032587.1	46249	.	T	c	100	*	TYPE=SUBSTITUTE
CM032587.1	46249	.	T	c	100	*	TYPE=SUBSTITUTE
CM032587.1	46253	.	C	t	100	*	TYPE=SUBSTITUTE
CM032587.1	46253	.	C	t	100	*	TYPE=SUBSTITUTE
CM032587.1	46253	.	C	t	100	*	TYPE=SUBSTITUTE
CM032587.1	46253	.	C	t	100	*	TYPE=SUBSTITUTE
CM032587.1	46256	.	C	t	100	*	TYPE=SUBSTITUTE
CM032587.1	46259	.	C	t	100	*	TYPE=SUBSTITUTE
CM032587.1	46259	.	C	t	100	*	TYPE=SUBSTITUTE
CM032587.1	46259	.	C	t	100	*	TYPE=SUBSTITUTE
CM032587.1	46270	.	G	a	100	*	TYPE=SUBSTITUTE
CM032587.1	46270	.	G	a	100	*	TYPE=SUBSTITUTE

CM032587.1	46270	.	G	a	100	*	TYPE=SUBSTITUTE
CM032587.1	46270	.	G	a	100	*	TYPE=SUBSTITUTE
CM032587.1	46280	.	T	c	100	*	TYPE=SUBSTITUTE
CM032587.1	46280	.	T	c	100	*	TYPE=SUBSTITUTE
CM032587.1	46283	.	C	t	100	*	TYPE=SUBSTITUTE
CM032587.1	46293	.	T	c	100	*	TYPE=SUBSTITUTE
CM032587.1	46293	.	T	c	100	*	TYPE=SUBSTITUTE
CM032587.1	46299	.	C	t	100	*	TYPE=SUBSTITUTE
CM032587.1	46302	.	C	t	100	*	TYPE=SUBSTITUTE
CM032587.1	46302	.	C	t	100	*	TYPE=SUBSTITUTE
CM032587.1	46302	.	C	t	100	*	TYPE=SUBSTITUTE
CM032587.1	46312	.	C	t	100	*	TYPE=SUBSTITUTE
CM032587.1	46312	.	C	t	100	*	TYPE=SUBSTITUTE
CM032587.1	46312	.	C	t	100	*	TYPE=SUBSTITUTE
CM032587.1	46317	.	G	a	100	*	TYPE=SUBSTITUTE
CM032587.1	46317	.	G	a	100	*	TYPE=SUBSTITUTE
CM032587.1	46318	.	C	t	100	*	TYPE=SUBSTITUTE
CM032587.1	46318	.	C	t	100	*	TYPE=SUBSTITUTE
CM032587.1	46318	.	C	t	100	*	TYPE=SUBSTITUTE
CM032587.1	46329	.	T	c	100	*	TYPE=SUBSTITUTE
CM032587.1	46329	.	T	c	100	*	TYPE=SUBSTITUTE
CM032587.1	46329	.	T	c	100	*	TYPE=SUBSTITUTE
CM032587.1	46333	.	G	a	100	*	TYPE=SUBSTITUTE
CM032587.1	46334	.	T	a	100	*	TYPE=SUBSTITUTE
CM032587.1	46339	.	C	t	100	*	TYPE=SUBSTITUTE
CM032587.1	46339	.	C	t	100	*	TYPE=SUBSTITUTE
CM032587.1	46343	.	C	t	100	*	TYPE=SUBSTITUTE
CM032587.1	46346	.	C	t	100	*	TYPE=SUBSTITUTE
CM032587.1	46355	.	T	c	100	*	TYPE=SUBSTITUTE
CM032587.1	46355	.	T	c	100	*	TYPE=SUBSTITUTE
CM032587.1	46364	.	AG	A	100	*	TYPE=DELETE
CM032587.1	46364	.	AG	A	100	*	TYPE=DELETE
CM032587.1	46365	.	G	a	100	*	TYPE=SUBSTITUTE
CM032587.1	46367	.	G	a	100	*	TYPE=SUBSTITUTE
CM032587.1	46367	.	G	a	100	*	TYPE=SUBSTITUTE
CM032587.1	46378	.	G	a	100	*	TYPE=SUBSTITUTE
CM032587.1	46378	.	G	a	100	*	TYPE=SUBSTITUTE
CM032587.1	46380	.	C	t	100	*	TYPE=SUBSTITUTE
CM032587.1	46380	.	C	t	100	*	TYPE=SUBSTITUTE
CM032587.1	46380	.	C	t	100	*	TYPE=SUBSTITUTE
CM032587.1	46397	.	A	g	100	*	TYPE=SUBSTITUTE
CM032587.1	46398	.	G	a	100	*	TYPE=SUBSTITUTE
CM032587.1	46401	.	a	ag	100	*	TYPE=INSERT
CM032587.1	46402	.	A	t	100	*	TYPE=SUBSTITUTE
CM032587.1	46403	.	C	t	100	*	TYPE=SUBSTITUTE
CM032587.1	46403	.	C	t	100	*	TYPE=SUBSTITUTE
CM032587.1	46404	.	T	c	100	*	TYPE=SUBSTITUTE
CM032587.1	46404	.	T	c	100	*	TYPE=SUBSTITUTE
CM032587.1	46404	.	T	c	100	*	TYPE=SUBSTITUTE
CM032587.1	46412	.	A	g	100	*	TYPE=SUBSTITUTE
CM032587.1	46412	.	A	g	100	*	TYPE=SUBSTITUTE
CM032587.1	46412	.	A	g	100	*	TYPE=SUBSTITUTE
CM032587.1	46416	.	A	G	100	*	TYPE=SUBSTITUTE
CM032587.1	46422	.	G	a	100	*	TYPE=SUBSTITUTE
CM032587.1	46424	.	T	A	100	*	TYPE=SUBSTITUTE
CM032587.1	46427	.	C	t	100	*	TYPE=SUBSTITUTE
CM032587.1	46428	.	TA	T	100	*	TYPE=DELETE
CM032587.1	46432	.	A	G	100	*	TYPE=SUBSTITUTE
CM032587.1	46432	.	A	G	100	*	TYPE=SUBSTITUTE
CM032587.1	46432	.	A	G	100	*	TYPE=SUBSTITUTE
CM032587.1	46432	.	AG	A	100	*	TYPE=DELETE
CM032587.1	46434	.	G	A	100	*	TYPE=SUBSTITUTE
CM032587.1	46434	.	G	a	100	*	TYPE=SUBSTITUTE
CM032587.1	46436	.	G	A	100	*	TYPE=SUBSTITUTE

CM032587.1	46436	.	G	a	100	*	TYPE=SUBSTITUTE
CM032587.1	46436	.	G	a	100	*	TYPE=SUBSTITUTE
CM032587.1	46437	.	G	a	100	*	TYPE=SUBSTITUTE
CM032587.1	46439	.	T	G	100	*	TYPE=SUBSTITUTE
CM032587.1	46443	.	A	g	100	*	TYPE=SUBSTITUTE
CM032587.1	46445	.	TA	T	100	*	TYPE=DELETE
CM032587.1	46449	.	A	g	100	*	TYPE=SUBSTITUTE
CM032587.1	46450	.	A	g	100	*	TYPE=SUBSTITUTE
CM032587.1	46452	.	G	a	100	*	TYPE=SUBSTITUTE
CM032587.1	46452	.	G	a	100	*	TYPE=SUBSTITUTE
CM032587.1	46460	.	A	g	100	*	TYPE=SUBSTITUTE
CM032587.1	46460	.	A	g	100	*	TYPE=SUBSTITUTE
CM032587.1	46464	.	C	a	100	*	TYPE=SUBSTITUTE
CM032587.1	46464	.	C	a	100	*	TYPE=SUBSTITUTE
CM032587.1	46464	.	C	a	100	*	TYPE=SUBSTITUTE
CM032587.1	46464	.	C	a	100	*	TYPE=SUBSTITUTE
CM032587.1	46465	.	C	t	100	*	TYPE=SUBSTITUTE
CM032587.1	46465	.	C	t	100	*	TYPE=SUBSTITUTE
CM032587.1	46466	.	t	ta	100	*	TYPE=INSERT
CM032587.1	46472	.	C	t	100	*	TYPE=SUBSTITUTE
CM032587.1	46472	.	C	t	100	*	TYPE=SUBSTITUTE
CM032587.1	46472	.	C	t	100	*	TYPE=SUBSTITUTE
CM032587.1	46472	.	C	t	100	*	TYPE=SUBSTITUTE
CM032587.1	46473	.	A	t	100	*	TYPE=SUBSTITUTE
CM032587.1	46473	.	A	t	100	*	TYPE=SUBSTITUTE
CM032587.1	46473	.	A	t	100	*	TYPE=SUBSTITUTE
CM032587.1	46473	.	A	t	100	*	TYPE=SUBSTITUTE
CM032587.1	46479	.	A	g	100	*	TYPE=SUBSTITUTE
CM032587.1	46487	.	A	g	100	*	TYPE=SUBSTITUTE
CM032587.1	46487	.	A	g	100	*	TYPE=SUBSTITUTE
CM032587.1	46503	.	TC	T	100	*	TYPE=DELETE
CM032587.1	46503	.	T	c	100	*	TYPE=SUBSTITUTE
CM032587.1	46503	.	T	c	100	*	TYPE=SUBSTITUTE
CM032587.1	46503	.	T	a	100	*	TYPE=SUBSTITUTE
CM032587.1	46504	.	C	t	100	*	TYPE=SUBSTITUTE
CM032587.1	46504	.	CTTA	C	100	*	TYPE=DELETE
CM032587.1	46505	.	T	c	100	*	TYPE=SUBSTITUTE
CM032587.1	46505	.	T	a	100	*	TYPE=SUBSTITUTE
CM032587.1	46508	.	T	c	100	*	TYPE=SUBSTITUTE
CM032587.1	46508	.	T	c	100	*	TYPE=SUBSTITUTE
CM032587.1	46509	.	T	c	100	*	TYPE=SUBSTITUTE
CM032587.1	46509	.	T	c	100	*	TYPE=SUBSTITUTE
CM032587.1	46510	.	T	c	100	*	TYPE=SUBSTITUTE
CM032587.1	46511	.	T	c	100	*	TYPE=SUBSTITUTE
CM032587.1	46511	.	T	c	100	*	TYPE=SUBSTITUTE
CM032587.1	46516	.	G	a	100	*	TYPE=SUBSTITUTE
CM032587.1	46526	.	A	g	100	*	TYPE=SUBSTITUTE
CM032587.1	46527	.	A	G	100	*	TYPE=SUBSTITUTE
CM032587.1	46529	.	G	a	100	*	TYPE=SUBSTITUTE
CM032587.1	46529	.	G	a	100	*	TYPE=SUBSTITUTE
CM032587.1	46530	.	G	a	100	*	TYPE=SUBSTITUTE
CM032587.1	46535	.	A	g	100	*	TYPE=SUBSTITUTE
CM032587.1	46535	.	A	g	100	*	TYPE=SUBSTITUTE
CM032587.1	46550	.	C	t	100	*	TYPE=SUBSTITUTE
CM032587.1	46550	.	C	t	100	*	TYPE=SUBSTITUTE
CM032587.1	46560	.	A	g	100	*	TYPE=SUBSTITUTE
CM032587.1	46560	.	A	g	100	*	TYPE=SUBSTITUTE
CM032587.1	46560	.	A	g	100	*	TYPE=SUBSTITUTE
CM032587.1	46560	.	A	g	100	*	TYPE=SUBSTITUTE
CM032587.1	46567	.	C	t	100	*	TYPE=SUBSTITUTE
CM032587.1	46567	.	C	t	100	*	TYPE=SUBSTITUTE
CM032587.1	46567	.	C	t	100	*	TYPE=SUBSTITUTE
CM032587.1	46567	.	C	t	100	*	TYPE=SUBSTITUTE
CM032587.1	46577	.	G	a	100	*	TYPE=SUBSTITUTE

CM032587.1	46577	.	G	a	100	*	TYPE=SUBSTITUTE
CM032587.1	46577	.	G	a	100	*	TYPE=SUBSTITUTE
CM032587.1	46577	.	G	a	100	*	TYPE=SUBSTITUTE
CM032587.1	46578	.	G	a	100	*	TYPE=SUBSTITUTE
CM032587.1	46578	.	G	a	100	*	TYPE=SUBSTITUTE
CM032587.1	46579	.	G	a	100	*	TYPE=SUBSTITUTE
CM032587.1	46594	.	G	a	100	*	TYPE=SUBSTITUTE
CM032587.1	46598	.	T	c	100	*	TYPE=SUBSTITUTE
CM032587.1	46598	.	T	c	100	*	TYPE=SUBSTITUTE
CM032587.1	46604	.	A	g	100	*	TYPE=SUBSTITUTE
CM032587.1	46608	.	A	g	100	*	TYPE=SUBSTITUTE
CM032587.1	46608	.	A	g	100	*	TYPE=SUBSTITUTE
CM032587.1	46617	.	A	g	100	*	TYPE=SUBSTITUTE
CM032587.1	46617	.	A	g	100	*	TYPE=SUBSTITUTE
CM032587.1	46619	.	T	c	100	*	TYPE=SUBSTITUTE
CM032587.1	46619	.	T	c	100	*	TYPE=SUBSTITUTE
CM032587.1	46632	.	C	t	100	*	TYPE=SUBSTITUTE
CM032587.1	46632	.	C	t	100	*	TYPE=SUBSTITUTE
CM032587.1	46632	.	C	t	100	*	TYPE=SUBSTITUTE
CM032587.1	46636	.	T	c	100	*	TYPE=SUBSTITUTE
CM032587.1	46636	.	T	c	100	*	TYPE=SUBSTITUTE
CM032587.1	46636	.	T	c	100	*	TYPE=SUBSTITUTE
CM032587.1	46636	.	T	c	100	*	TYPE=SUBSTITUTE
CM032587.1	46641	.	G	a	100	*	TYPE=SUBSTITUTE
CM032587.1	46641	.	G	a	100	*	TYPE=SUBSTITUTE
CM032587.1	46648	.	G	a	100	*	TYPE=SUBSTITUTE
CM032587.1	46648	.	G	a	100	*	TYPE=SUBSTITUTE
CM032587.1	46652	.	A	g	100	*	TYPE=SUBSTITUTE
CM032587.1	46665	.	G	a	100	*	TYPE=SUBSTITUTE
CM032587.1	46665	.	G	a	100	*	TYPE=SUBSTITUTE
CM032587.1	46665	.	G	a	100	*	TYPE=SUBSTITUTE
CM032587.1	46667	.	C	t	100	*	TYPE=SUBSTITUTE
CM032587.1	46667	.	C	t	100	*	TYPE=SUBSTITUTE
CM032587.1	46667	.	C	t	100	*	TYPE=SUBSTITUTE
CM032587.1	46672	.	AG	A	100	*	TYPE=DELETE
CM032587.1	46673	.	G	a	100	*	TYPE=SUBSTITUTE
CM032587.1	46673	.	G	a	100	*	TYPE=SUBSTITUTE
CM032587.1	46674	.	A	g	100	*	TYPE=SUBSTITUTE
CM032587.1	46674	.	A	g	100	*	TYPE=SUBSTITUTE
CM032587.1	46682	.	AC	A	100	*	TYPE=DELETE
CM032587.1	46683	.	C	t	100	*	TYPE=SUBSTITUTE
CM032587.1	46683	.	C	t	100	*	TYPE=SUBSTITUTE
CM032587.1	46683	.	C	t	100	*	TYPE=SUBSTITUTE
CM032587.1	46691	.	C	t	100	*	TYPE=SUBSTITUTE
CM032587.1	46700	.	C	t	100	*	TYPE=SUBSTITUTE
CM032587.1	46711	.	C	t	100	*	TYPE=SUBSTITUTE
CM032587.1	46711	.	C	t	100	*	TYPE=SUBSTITUTE
CM032587.1	46711	.	C	t	100	*	TYPE=SUBSTITUTE
CM032587.1	46712	.	T	c	100	*	TYPE=SUBSTITUTE
CM032587.1	46718	.	A	g	100	*	TYPE=SUBSTITUTE
CM032587.1	46719	.	G	a	100	*	TYPE=SUBSTITUTE
CM032587.1	46726	.	C	t	100	*	TYPE=SUBSTITUTE
CM032587.1	46732	.	C	t	100	*	TYPE=SUBSTITUTE
CM032587.1	46736	.	A	g	100	*	TYPE=SUBSTITUTE
CM032587.1	46739	.	C	t	100	*	TYPE=SUBSTITUTE
CM032587.1	46739	.	C	t	100	*	TYPE=SUBSTITUTE
CM032587.1	46739	.	C	t	100	*	TYPE=SUBSTITUTE
CM032587.1	46739	.	C	t	100	*	TYPE=SUBSTITUTE
CM032587.1	46745	.	A	g	100	*	TYPE=SUBSTITUTE
CM032587.1	46749	.	A	g	100	*	TYPE=SUBSTITUTE
CM032587.1	46749	.	A	g	100	*	TYPE=SUBSTITUTE
CM032587.1	46755	.	A	g	100	*	TYPE=SUBSTITUTE
CM032587.1	46755	.	A	g	100	*	TYPE=SUBSTITUTE
CM032587.1	46755	.	A	g	100	*	TYPE=SUBSTITUTE

CM032587.1	46756	.	A	g	100	*	TYPE=SUBSTITUTE
CM032587.1	46756	.	A	g	100	*	TYPE=SUBSTITUTE
CM032587.1	46756	.	A	g	100	*	TYPE=SUBSTITUTE
CM032587.1	46764	.	G	a	100	*	TYPE=SUBSTITUTE
CM032587.1	46768	.	C	t	100	*	TYPE=SUBSTITUTE
CM032587.1	46768	.	C	t	100	*	TYPE=SUBSTITUTE
CM032587.1	46768	.	C	t	100	*	TYPE=SUBSTITUTE
CM032587.1	46782	.	A	g	100	*	TYPE=SUBSTITUTE
CM032587.1	46782	.	A	g	100	*	TYPE=SUBSTITUTE
CM032587.1	46782	.	A	g	100	*	TYPE=SUBSTITUTE
CM032587.1	46788	.	A	g	100	*	TYPE=SUBSTITUTE
CM032587.1	46789	.	AG	A	100	*	TYPE=DELETE
CM032587.1	46790	.	G	a	100	*	TYPE=SUBSTITUTE
CM032587.1	46790	.	G	a	100	*	TYPE=SUBSTITUTE
CM032587.1	46790	.	G	a	100	*	TYPE=SUBSTITUTE
CM032587.1	46791	.	G	a	100	*	TYPE=SUBSTITUTE
CM032587.1	46791	.	G	a	100	*	TYPE=SUBSTITUTE
CM032587.1	46791	.	G	a	100	*	TYPE=SUBSTITUTE
CM032587.1	46791	.	GGA	G	100	*	TYPE=DELETE
CM032587.1	46792	.	G	a	100	*	TYPE=SUBSTITUTE
CM032587.1	46792	.	G	a	100	*	TYPE=SUBSTITUTE
CM032587.1	46792	.	G	a	100	*	TYPE=SUBSTITUTE
CM032587.1	46795	.	G	a	100	*	TYPE=SUBSTITUTE
CM032587.1	46795	.	G	a	100	*	TYPE=SUBSTITUTE
CM032587.1	46795	.	G	a	100	*	TYPE=SUBSTITUTE
CM032587.1	46795	.	G	a	100	*	TYPE=SUBSTITUTE
CM032587.1	46796	.	G	a	100	*	TYPE=SUBSTITUTE
CM032587.1	46798	.	T	c	100	*	TYPE=SUBSTITUTE
CM032587.1	46798	.	T	c	100	*	TYPE=SUBSTITUTE
CM032587.1	46812	.	A	g	100	*	TYPE=SUBSTITUTE
CM032587.1	46812	.	A	g	100	*	TYPE=SUBSTITUTE
CM032587.1	46813	.	G	a	100	*	TYPE=SUBSTITUTE
CM032587.1	46815	.	C	t	100	*	TYPE=SUBSTITUTE
CM032587.1	46815	.	C	t	100	*	TYPE=SUBSTITUTE
CM032587.1	46815	.	C	t	100	*	TYPE=SUBSTITUTE
CM032587.1	46822	.	A	g	100	*	TYPE=SUBSTITUTE
CM032587.1	46823	.	C	t	100	*	TYPE=SUBSTITUTE
CM032587.1	46823	.	C	t	100	*	TYPE=SUBSTITUTE
CM032587.1	46827	.	G	a	100	*	TYPE=SUBSTITUTE
CM032587.1	46832	.	C	t	100	*	TYPE=SUBSTITUTE
CM032587.1	46832	.	C	t	100	*	TYPE=SUBSTITUTE
CM032587.1	46837	.	G	a	100	*	TYPE=SUBSTITUTE
CM032587.1	46838	.	C	t	100	*	TYPE=SUBSTITUTE
CM032587.1	46838	.	C	t	100	*	TYPE=SUBSTITUTE
CM032587.1	46839	.	T	c	100	*	TYPE=SUBSTITUTE
CM032587.1	46839	.	T	c	100	*	TYPE=SUBSTITUTE
CM032587.1	46840	.	T	c	100	*	TYPE=SUBSTITUTE
CM032587.1	46841	.	A	T	100	*	TYPE=SUBSTITUTE
CM032587.1	46841	.	A	t	100	*	TYPE=SUBSTITUTE
CM032587.1	46841	.	A	t	100	*	TYPE=SUBSTITUTE
CM032587.1	46841	.	A	t	100	*	TYPE=SUBSTITUTE
CM032587.1	46843	.	A	T	100	*	TYPE=SUBSTITUTE
CM032587.1	46843	.	A	t	100	*	TYPE=SUBSTITUTE
CM032587.1	46843	.	A	t	100	*	TYPE=SUBSTITUTE
CM032587.1	46843	.	A	t	100	*	TYPE=SUBSTITUTE
CM032587.1	46845	.	A	g	100	*	TYPE=SUBSTITUTE
CM032587.1	46845	.	A	G	100	*	TYPE=SUBSTITUTE
CM032587.1	46845	.	A	g	100	*	TYPE=SUBSTITUTE
CM032587.1	46845	.	A	g	100	*	TYPE=SUBSTITUTE
CM032587.1	46851	.	A	g	100	*	TYPE=SUBSTITUTE
CM032587.1	46861	.	G	a	100	*	TYPE=SUBSTITUTE
CM032587.1	46861	.	G	a	100	*	TYPE=SUBSTITUTE
CM032587.1	46861	.	G	a	100	*	TYPE=SUBSTITUTE
CM032587.1	46861	.	G	a	100	*	TYPE=SUBSTITUTE

CM032587.1	46874	.	C	t	100	*	TYPE=SUBSTITUTE
CM032587.1	46875	.	C	t	100	*	TYPE=SUBSTITUTE
CM032587.1	46875	.	C	t	100	*	TYPE=SUBSTITUTE
CM032587.1	46875	.	C	t	100	*	TYPE=SUBSTITUTE
CM032587.1	46875	.	C	t	100	*	TYPE=SUBSTITUTE
CM032587.1	46893	.	A	g	100	*	TYPE=SUBSTITUTE
CM032587.1	46893	.	A	g	100	*	TYPE=SUBSTITUTE
CM032587.1	46894	.	T	c	100	*	TYPE=SUBSTITUTE
CM032587.1	46899	.	T	c	100	*	TYPE=SUBSTITUTE
CM032587.1	46901	.	C	t	100	*	TYPE=SUBSTITUTE
CM032587.1	46901	.	C	T	100	*	TYPE=SUBSTITUTE
CM032587.1	46901	.	C	t	100	*	TYPE=SUBSTITUTE
CM032587.1	46901	.	C	t	100	*	TYPE=SUBSTITUTE
CM032587.1	46904	.	A	g	100	*	TYPE=SUBSTITUTE
CM032587.1	46905	.	A	G	100	*	TYPE=SUBSTITUTE
CM032587.1	46905	.	A	g	100	*	TYPE=SUBSTITUTE
CM032587.1	46905	.	A	g	100	*	TYPE=SUBSTITUTE
CM032587.1	46905	.	A	g	100	*	TYPE=SUBSTITUTE
CM032587.1	46906	.	T	c	100	*	TYPE=SUBSTITUTE
CM032587.1	46906	.	T	c	100	*	TYPE=SUBSTITUTE
CM032587.1	46906	.	T	C	100	*	TYPE=SUBSTITUTE
CM032587.1	46906	.	T	c	100	*	TYPE=SUBSTITUTE
CM032587.1	46907	.	T	c	100	*	TYPE=SUBSTITUTE
CM032587.1	46907	.	T	C	100	*	TYPE=SUBSTITUTE
CM032587.1	46907	.	T	c	100	*	TYPE=SUBSTITUTE
CM032587.1	46909	.	AT	A	100	*	TYPE=DELETE
CM032587.1	46910	.	T	A	100	*	TYPE=SUBSTITUTE
CM032587.1	46910	.	T	A	100	*	TYPE=SUBSTITUTE
CM032587.1	46910	.	T	A	100	*	TYPE=SUBSTITUTE
CM032587.1	46911	.	A	G	100	*	TYPE=SUBSTITUTE
CM032587.1	46911	.	A	G	100	*	TYPE=SUBSTITUTE
CM032587.1	46912	.	A	g	100	*	TYPE=SUBSTITUTE
CM032587.1	46912	.	A	G	100	*	TYPE=SUBSTITUTE
CM032587.1	46912	.	A	g	100	*	TYPE=SUBSTITUTE
CM032587.1	46912	.	A	G	100	*	TYPE=SUBSTITUTE
CM032587.1	46913	.	A	g	100	*	TYPE=SUBSTITUTE
CM032587.1	46913	.	A	G	100	*	TYPE=SUBSTITUTE
CM032587.1	46913	.	A	g	100	*	TYPE=SUBSTITUTE
CM032587.1	46917	.	A	G	100	*	TYPE=SUBSTITUTE
CM032587.1	46917	.	A	G	100	*	TYPE=SUBSTITUTE
CM032587.1	46919	.	A	G	100	*	TYPE=SUBSTITUTE
CM032587.1	46919	.	A	G	100	*	TYPE=SUBSTITUTE
CM032587.1	46919	.	A	g	100	*	TYPE=SUBSTITUTE
CM032587.1	46919	.	A	g	100	*	TYPE=SUBSTITUTE
CM032587.1	46931	.	A	g	100	*	TYPE=SUBSTITUTE
CM032587.1	46931	.	A	G	100	*	TYPE=SUBSTITUTE
CM032587.1	46931	.	A	G	100	*	TYPE=SUBSTITUTE
CM032587.1	46934	.	A	G	100	*	TYPE=SUBSTITUTE
CM032587.1	46934	.	A	G	100	*	TYPE=SUBSTITUTE
CM032587.1	46935	.	G	A	100	*	TYPE=SUBSTITUTE
CM032587.1	46941	.	G	A	100	*	TYPE=SUBSTITUTE
CM032587.1	46942	.	A	g	100	*	TYPE=SUBSTITUTE
CM032587.1	46942	.	A	g	100	*	TYPE=SUBSTITUTE
CM032587.1	46942	.	A	g	100	*	TYPE=SUBSTITUTE
CM032587.1	46944	.	G	a	100	*	TYPE=SUBSTITUTE
CM032587.1	46961	.	G	a	100	*	TYPE=SUBSTITUTE
CM032587.1	46961	.	G	a	100	*	TYPE=SUBSTITUTE
CM032587.1	46961	.	G	a	100	*	TYPE=SUBSTITUTE
CM032587.1	46961	.	G	a	100	*	TYPE=SUBSTITUTE
CM032587.1	46964	.	C	t	100	*	TYPE=SUBSTITUTE
CM032587.1	46968	.	G	a	100	*	TYPE=SUBSTITUTE
CM032587.1	46968	.	G	a	100	*	TYPE=SUBSTITUTE
CM032587.1	46970	.	G	a	100	*	TYPE=SUBSTITUTE
CM032587.1	46975	.	G	a	100	*	TYPE=SUBSTITUTE

CM032587.1	46975	.	G	a	100	*	TYPE=SUBSTITUTE
CM032587.1	46975	.	G	a	100	*	TYPE=SUBSTITUTE
CM032587.1	46977	.	a	at	100	*	TYPE=INSERT
CM032587.1	46994	.	G	a	100	*	TYPE=SUBSTITUTE
CM032587.1	46994	.	G	a	100	*	TYPE=SUBSTITUTE
CM032587.1	46994	.	G	a	100	*	TYPE=SUBSTITUTE
CM032587.1	46994	.	G	a	100	*	TYPE=SUBSTITUTE
CM032587.1	46995	.	G	a	100	*	TYPE=SUBSTITUTE
CM032587.1	46997	.	G	a	100	*	TYPE=SUBSTITUTE
CM032587.1	46997	.	G	a	100	*	TYPE=SUBSTITUTE
CM032587.1	47006	.	C	t	100	*	TYPE=SUBSTITUTE
CM032587.1	47006	.	C	t	100	*	TYPE=SUBSTITUTE
CM032587.1	47006	.	C	t	100	*	TYPE=SUBSTITUTE
CM032587.1	47006	.	C	t	100	*	TYPE=SUBSTITUTE
CM032587.1	47017	.	G	a	100	*	TYPE=SUBSTITUTE
CM032587.1	47017	.	G	a	100	*	TYPE=SUBSTITUTE
CM032587.1	47017	.	G	a	100	*	TYPE=SUBSTITUTE
CM032587.1	47017	.	G	a	100	*	TYPE=SUBSTITUTE
CM032587.1	47019	.	A	g	100	*	TYPE=SUBSTITUTE
CM032587.1	47019	.	A	g	100	*	TYPE=SUBSTITUTE
CM032587.1	47019	.	A	g	100	*	TYPE=SUBSTITUTE
CM032587.1	47033	.	AT	A	100	*	TYPE=DELETE
CM032587.1	47047	.	G	a	100	*	TYPE=SUBSTITUTE
CM032587.1	47047	.	G	a	100	*	TYPE=SUBSTITUTE
CM032587.1	47047	.	G	a	100	*	TYPE=SUBSTITUTE
CM032587.1	47047	.	G	a	100	*	TYPE=SUBSTITUTE
CM032587.1	47048	.	T	c	100	*	TYPE=SUBSTITUTE
CM032587.1	47048	.	T	c	100	*	TYPE=SUBSTITUTE
CM032587.1	47048	.	T	c	100	*	TYPE=SUBSTITUTE
CM032587.1	47063	.	T	c	100	*	TYPE=SUBSTITUTE
CM032587.1	47063	.	T	c	100	*	TYPE=SUBSTITUTE
CM032587.1	47067	.	A	g	100	*	TYPE=SUBSTITUTE
CM032587.1	47068	.	G	a	100	*	TYPE=SUBSTITUTE
CM032587.1	47072	.	C	t	100	*	TYPE=SUBSTITUTE
CM032587.1	47072	.	C	t	100	*	TYPE=SUBSTITUTE
CM032587.1	47072	.	C	t	100	*	TYPE=SUBSTITUTE
CM032587.1	47073	.	T	c	100	*	TYPE=SUBSTITUTE
CM032587.1	47094	.	A	g	100	*	TYPE=SUBSTITUTE
CM032587.1	47095	.	T	c	100	*	TYPE=SUBSTITUTE
CM032587.1	47098	.	CCT	C	100	*	TYPE=DELETE
CM032587.1	47099	.	C	t	100	*	TYPE=SUBSTITUTE
CM032587.1	47099	.	C	t	100	*	TYPE=SUBSTITUTE
CM032587.1	47099	.	C	t	100	*	TYPE=SUBSTITUTE
CM032587.1	47111	.	A	g	100	*	TYPE=SUBSTITUTE
CM032587.1	47119	.	A	g	100	*	TYPE=SUBSTITUTE
CM032587.1	47130	.	G	a	100	*	TYPE=SUBSTITUTE
CM032587.1	47130	.	G	a	100	*	TYPE=SUBSTITUTE
CM032587.1	47130	.	G	a	100	*	TYPE=SUBSTITUTE
CM032587.1	47130	.	G	a	100	*	TYPE=SUBSTITUTE
CM032587.1	47137	.	C	t	100	*	TYPE=SUBSTITUTE
CM032587.1	47137	.	C	t	100	*	TYPE=SUBSTITUTE
CM032587.1	47137	.	C	t	100	*	TYPE=SUBSTITUTE
CM032587.1	47140	.	C	t	100	*	TYPE=SUBSTITUTE
CM032587.1	47140	.	C	t	100	*	TYPE=SUBSTITUTE
CM032587.1	47144	.	TCC	T	100	*	TYPE=DELETE
CM032587.1	47145	.	C	t	100	*	TYPE=SUBSTITUTE
CM032587.1	47145	.	C	t	100	*	TYPE=SUBSTITUTE
CM032587.1	47146	.	C	t	100	*	TYPE=SUBSTITUTE
CM032587.1	47146	.	C	t	100	*	TYPE=SUBSTITUTE
CM032587.1	47155	.	A	g	100	*	TYPE=SUBSTITUTE
CM032587.1	47155	.	A	g	100	*	TYPE=SUBSTITUTE
CM032587.1	47160	.	G	a	100	*	TYPE=SUBSTITUTE
CM032587.1	47160	.	G	a	100	*	TYPE=SUBSTITUTE
CM032587.1	47160	.	G	a	100	*	TYPE=SUBSTITUTE

CM032587.1	47160	.	G	a	100	*	TYPE=SUBSTITUTE
CM032587.1	47163	.	A	g	100	*	TYPE=SUBSTITUTE
CM032587.1	47163	.	A	g	100	*	TYPE=SUBSTITUTE
CM032587.1	47163	.	A	g	100	*	TYPE=SUBSTITUTE
CM032587.1	47163	.	A	g	100	*	TYPE=SUBSTITUTE
CM032587.1	47172	.	G	a	100	*	TYPE=SUBSTITUTE
CM032587.1	47172	.	G	a	100	*	TYPE=SUBSTITUTE
CM032587.1	47173	.	T	c	100	*	TYPE=SUBSTITUTE
CM032587.1	47176	.	A	g	100	*	TYPE=SUBSTITUTE
CM032587.1	47176	.	A	g	100	*	TYPE=SUBSTITUTE
CM032587.1	47176	.	A	g	100	*	TYPE=SUBSTITUTE
CM032587.1	47182	.	C	t	100	*	TYPE=SUBSTITUTE
CM032587.1	47182	.	C	t	100	*	TYPE=SUBSTITUTE
CM032587.1	47182	.	C	t	100	*	TYPE=SUBSTITUTE
CM032587.1	47187	.	A	g	100	*	TYPE=SUBSTITUTE
CM032587.1	47187	.	A	g	100	*	TYPE=SUBSTITUTE
CM032587.1	47187	.	A	g	100	*	TYPE=SUBSTITUTE
CM032587.1	47191	.	T	c	100	*	TYPE=SUBSTITUTE
CM032587.1	47191	.	T	c	100	*	TYPE=SUBSTITUTE
CM032587.1	47200	.	A	g	100	*	TYPE=SUBSTITUTE
CM032587.1	47201	.	G	a	100	*	TYPE=SUBSTITUTE
CM032587.1	47202	.	C	t	100	*	TYPE=SUBSTITUTE
CM032587.1	47202	.	C	t	100	*	TYPE=SUBSTITUTE
CM032587.1	47202	.	C	t	100	*	TYPE=SUBSTITUTE
CM032587.1	47203	.	T	c	100	*	TYPE=SUBSTITUTE
CM032587.1	47228	.	A	g	100	*	TYPE=SUBSTITUTE
CM032587.1	47228	.	A	g	100	*	TYPE=SUBSTITUTE
CM032587.1	47229	.	G	a	100	*	TYPE=SUBSTITUTE
CM032587.1	47229	.	G	a	100	*	TYPE=SUBSTITUTE
CM032587.1	47229	.	G	a	100	*	TYPE=SUBSTITUTE
CM032587.1	47230	.	G	a	100	*	TYPE=SUBSTITUTE
CM032587.1	47241	.	C	t	100	*	TYPE=SUBSTITUTE
CM032587.1	47241	.	C	t	100	*	TYPE=SUBSTITUTE
CM032587.1	47242	.	C	t	100	*	TYPE=SUBSTITUTE
CM032587.1	47242	.	C	t	100	*	TYPE=SUBSTITUTE
CM032587.1	47246	.	A	G	100	*	TYPE=SUBSTITUTE
CM032587.1	47247	.	G	a	100	*	TYPE=SUBSTITUTE
CM032587.1	47248	.	G	a	100	*	TYPE=SUBSTITUTE
CM032587.1	47249	.	G	a	100	*	TYPE=SUBSTITUTE
CM032587.1	47249	.	G	a	100	*	TYPE=SUBSTITUTE
CM032587.1	47270	.	TTC	T	100	*	TYPE=DELETE
CM032587.1	47272	.	C	t	100	*	TYPE=SUBSTITUTE
CM032587.1	47272	.	C	t	100	*	TYPE=SUBSTITUTE
CM032587.1	47274	.	T	c	100	*	TYPE=SUBSTITUTE
CM032587.1	47277	.	G	a	100	*	TYPE=SUBSTITUTE
CM032587.1	47277	.	G	a	100	*	TYPE=SUBSTITUTE
CM032587.1	47278	.	C	t	100	*	TYPE=SUBSTITUTE
CM032587.1	47278	.	C	t	100	*	TYPE=SUBSTITUTE
CM032587.1	47281	.	T	c	100	*	TYPE=SUBSTITUTE
CM032587.1	47281	.	T	c	100	*	TYPE=SUBSTITUTE
CM032587.1	47284	.	C	t	100	*	TYPE=SUBSTITUTE
CM032587.1	47284	.	C	t	100	*	TYPE=SUBSTITUTE
CM032587.1	47285	.	T	c	100	*	TYPE=SUBSTITUTE
CM032587.1	47285	.	T	c	100	*	TYPE=SUBSTITUTE
CM032587.1	47287	.	T	c	100	*	TYPE=SUBSTITUTE
CM032587.1	47300	.	G	a	100	*	TYPE=SUBSTITUTE
CM032587.1	47304	.	G	a	100	*	TYPE=SUBSTITUTE
CM032587.1	47304	.	G	a	100	*	TYPE=SUBSTITUTE
CM032587.1	47305	.	C	t	100	*	TYPE=SUBSTITUTE
CM032587.1	47305	.	C	t	100	*	TYPE=SUBSTITUTE
CM032587.1	47305	.	C	t	100	*	TYPE=SUBSTITUTE
CM032587.1	47312	.	A	g	100	*	TYPE=SUBSTITUTE
CM032587.1	47313	.	A	g	100	*	TYPE=SUBSTITUTE
CM032587.1	47314	.	C	t	100	*	TYPE=SUBSTITUTE

CM032587.1	47317	.	C	t	100	*	TYPE=SUBSTITUTE
CM032587.1	47317	.	C	t	100	*	TYPE=SUBSTITUTE
CM032587.1	47325	.	G	a	100	*	TYPE=SUBSTITUTE
CM032587.1	47330	.	A	g	100	*	TYPE=SUBSTITUTE
CM032587.1	47330	.	A	g	100	*	TYPE=SUBSTITUTE
CM032587.1	47331	.	G	a	100	*	TYPE=SUBSTITUTE
CM032587.1	47333	.	A	g	100	*	TYPE=SUBSTITUTE
CM032587.1	47333	.	A	g	100	*	TYPE=SUBSTITUTE
CM032587.1	47333	.	A	g	100	*	TYPE=SUBSTITUTE
CM032587.1	47334	.	G	a	100	*	TYPE=SUBSTITUTE
CM032587.1	47335	.	T	c	100	*	TYPE=SUBSTITUTE
CM032587.1	47337	.	c	ct	100	*	TYPE=INSERT
CM032587.1	47337	.	C	t	100	*	TYPE=SUBSTITUTE
CM032587.1	47337	.	C	t	100	*	TYPE=SUBSTITUTE
CM032587.1	47337	.	C	t	100	*	TYPE=SUBSTITUTE
CM032587.1	47339	.	T	a	100	*	TYPE=SUBSTITUTE
CM032587.1	47347	.	A	g	100	*	TYPE=SUBSTITUTE
CM032587.1	47350	.	C	t	100	*	TYPE=SUBSTITUTE
CM032587.1	47350	.	C	t	100	*	TYPE=SUBSTITUTE
CM032587.1	47353	.	C	t	100	*	TYPE=SUBSTITUTE
CM032587.1	47353	.	C	t	100	*	TYPE=SUBSTITUTE
CM032587.1	47353	.	C	t	100	*	TYPE=SUBSTITUTE
CM032587.1	47354	.	T	c	100	*	TYPE=SUBSTITUTE
CM032587.1	47363	.	A	g	100	*	TYPE=SUBSTITUTE
CM032587.1	47380	.	CT	C	100	*	TYPE=DELETE
CM032587.1	47380	.	C	t	100	*	TYPE=SUBSTITUTE
CM032587.1	47380	.	C	t	100	*	TYPE=SUBSTITUTE
CM032587.1	47381	.	T	c	100	*	TYPE=SUBSTITUTE
CM032587.1	47381	.	T	c	100	*	TYPE=SUBSTITUTE
CM032587.1	47382	.	T	c	100	*	TYPE=SUBSTITUTE
CM032587.1	47388	.	G	a	100	*	TYPE=SUBSTITUTE
CM032587.1	47388	.	G	a	100	*	TYPE=SUBSTITUTE
CM032587.1	47388	.	G	a	100	*	TYPE=SUBSTITUTE
CM032587.1	47388	.	G	a	100	*	TYPE=SUBSTITUTE
CM032587.1	47389	.	C	t	100	*	TYPE=SUBSTITUTE
CM032587.1	47395	.	C	t	100	*	TYPE=SUBSTITUTE
CM032587.1	47401	.	A	g	100	*	TYPE=SUBSTITUTE
CM032587.1	47401	.	A	g	100	*	TYPE=SUBSTITUTE
CM032587.1	47401	.	A	g	100	*	TYPE=SUBSTITUTE
CM032587.1	47402	.	A	G	100	*	TYPE=SUBSTITUTE
CM032587.1	47403	.	G	a	100	*	TYPE=SUBSTITUTE
CM032587.1	47404	.	G	a	100	*	TYPE=SUBSTITUTE
CM032587.1	47405	.	A	G	100	*	TYPE=SUBSTITUTE
CM032587.1	47406	.	AG	A	100	*	TYPE=DELETE
CM032587.1	47407	.	G	a	100	*	TYPE=SUBSTITUTE
CM032587.1	47407	.	G	A	100	*	TYPE=SUBSTITUTE
CM032587.1	47408	.	G	A	100	*	TYPE=SUBSTITUTE
CM032587.1	47414	.	A	T	100	*	TYPE=SUBSTITUTE
CM032587.1	47415	.	G	A	100	*	TYPE=SUBSTITUTE
CM032587.1	47420	.	G	a	100	*	TYPE=SUBSTITUTE
CM032587.1	47421	.	T	C	100	*	TYPE=SUBSTITUTE
CM032587.1	47424	.	A	g	100	*	TYPE=SUBSTITUTE
CM032587.1	47424	.	A	g	100	*	TYPE=SUBSTITUTE
CM032587.1	47426	.	A	g	100	*	TYPE=SUBSTITUTE
CM032587.1	47431	.	A	g	100	*	TYPE=SUBSTITUTE
CM032587.1	47434	.	A	g	100	*	TYPE=SUBSTITUTE
CM032587.1	47435	.	C	t	100	*	TYPE=SUBSTITUTE
CM032587.1	47435	.	C	t	100	*	TYPE=SUBSTITUTE
CM032587.1	47435	.	C	t	100	*	TYPE=SUBSTITUTE
CM032587.1	47445	.	A	g	100	*	TYPE=SUBSTITUTE
CM032587.1	47445	.	A	g	100	*	TYPE=SUBSTITUTE
CM032587.1	47446	.	A	g	100	*	TYPE=SUBSTITUTE
CM032587.1	47446	.	A	g	100	*	TYPE=SUBSTITUTE
CM032587.1	47446	.	A	g	100	*	TYPE=SUBSTITUTE

CM032587.1	47448	.	C	t	100	*	TYPE=SUBSTITUTE
CM032587.1	47448	.	C	t	100	*	TYPE=SUBSTITUTE
CM032587.1	47451	.	A	G	100	*	TYPE=SUBSTITUTE
CM032587.1	47456	.	A	g	100	*	TYPE=SUBSTITUTE
CM032587.1	47460	.	C	t	100	*	TYPE=SUBSTITUTE
CM032587.1	47460	.	C	t	100	*	TYPE=SUBSTITUTE
CM032587.1	47461	.	C	t	100	*	TYPE=SUBSTITUTE
CM032587.1	47461	.	C	t	100	*	TYPE=SUBSTITUTE
CM032587.1	47461	.	C	t	100	*	TYPE=SUBSTITUTE
CM032587.1	47464	.	G	a	100	*	TYPE=SUBSTITUTE
CM032587.1	47464	.	G	a	100	*	TYPE=SUBSTITUTE
CM032587.1	47464	.	G	a	100	*	TYPE=SUBSTITUTE
CM032587.1	47465	.	G	a	100	*	TYPE=SUBSTITUTE
CM032587.1	47465	.	G	a	100	*	TYPE=SUBSTITUTE
CM032587.1	47469	.	A	g	100	*	TYPE=SUBSTITUTE
CM032587.1	47470	.	A	c	100	*	TYPE=SUBSTITUTE
CM032587.1	47470	.	A	g	100	*	TYPE=SUBSTITUTE
CM032587.1	47470	.	A	t	100	*	TYPE=SUBSTITUTE
CM032587.1	47471	.	C	t	100	*	TYPE=SUBSTITUTE
CM032587.1	47471	.	C	t	100	*	TYPE=SUBSTITUTE
CM032587.1	47471	.	C	t	100	*	TYPE=SUBSTITUTE
CM032587.1	47471	.	C	t	100	*	TYPE=SUBSTITUTE
CM032587.1	47472	.	G	a	100	*	TYPE=SUBSTITUTE
CM032587.1	47472	.	G	a	100	*	TYPE=SUBSTITUTE
CM032587.1	47472	.	G	a	100	*	TYPE=SUBSTITUTE
CM032587.1	47481	.	A	g	100	*	TYPE=SUBSTITUTE
CM032587.1	47486	.	AAAAACT	A	100	*	TYPE=DELETE
CM032587.1	47488	.	a	aggt	100	*	TYPE=INSERT
CM032587.1	47489	.	A	g	100	*	TYPE=SUBSTITUTE
CM032587.1	47489	.	A	g	100	*	TYPE=SUBSTITUTE
CM032587.1	47490	.	A	g	100	*	TYPE=SUBSTITUTE
CM032587.1	47490	.	A	g	100	*	TYPE=SUBSTITUTE
CM032587.1	47490	.	a	aGGG	100	*	TYPE=INSERT
CM032587.1	47491	.	C	t	100	*	TYPE=SUBSTITUTE
CM032587.1	47491	.	C	T	100	*	TYPE=SUBSTITUTE
CM032587.1	47492	.	T	A	100	*	TYPE=SUBSTITUTE
CM032587.1	47495	.	G	a	100	*	TYPE=SUBSTITUTE
CM032587.1	47496	.	G	a	100	*	TYPE=SUBSTITUTE
CM032587.1	47497	.	t	ta	100	*	TYPE=INSERT
CM032587.1	47499	.	G	A	100	*	TYPE=SUBSTITUTE
CM032587.1	47499	.	G	a	100	*	TYPE=SUBSTITUTE
CM032587.1	47499	.	G	A	100	*	TYPE=SUBSTITUTE
CM032587.1	47501	.	A	G	100	*	TYPE=SUBSTITUTE
CM032587.1	47501	.	A	g	100	*	TYPE=SUBSTITUTE
CM032587.1	47506	.	G	A	100	*	TYPE=SUBSTITUTE
CM032587.1	47507	.	T	G	100	*	TYPE=SUBSTITUTE
CM032587.1	47510	.	A	T	100	*	TYPE=SUBSTITUTE
CM032587.1	47511	.	A	G	100	*	TYPE=SUBSTITUTE
CM032587.1	47517	.	A	G	100	*	TYPE=SUBSTITUTE
CM032587.1	47518	.	G	a	100	*	TYPE=SUBSTITUTE
CM032587.1	47520	.	A	AGG	100	*	TYPE=INSERT
CM032587.1	47521	.	A	g	100	*	TYPE=SUBSTITUTE
CM032587.1	47526	.	A	g	100	*	TYPE=SUBSTITUTE
CM032587.1	47543	.	C	t	100	*	TYPE=SUBSTITUTE
CM032587.1	47543	.	C	t	100	*	TYPE=SUBSTITUTE
CM032587.1	47546	.	A	g	100	*	TYPE=SUBSTITUTE
CM032587.1	47547	.	C	t	100	*	TYPE=SUBSTITUTE
CM032587.1	47547	.	C	t	100	*	TYPE=SUBSTITUTE
CM032587.1	47554	.	t	tc	100	*	TYPE=INSERT
CM032587.1	47555	.	T	c	100	*	TYPE=SUBSTITUTE
CM032587.1	47560	.	T	c	100	*	TYPE=SUBSTITUTE
CM032587.1	47563	.	C	t	100	*	TYPE=SUBSTITUTE
CM032587.1	47564	.	C	t	100	*	TYPE=SUBSTITUTE
CM032587.1	47564	.	C	t	100	*	TYPE=SUBSTITUTE

CM032587.1	47564	.	C	t	100	*	TYPE=SUBSTITUTE
CM032587.1	47564	.	C	t	100	*	TYPE=SUBSTITUTE
CM032587.1	47569	.	G	a	100	*	TYPE=SUBSTITUTE
CM032587.1	47580	.	G	a	100	*	TYPE=SUBSTITUTE
CM032587.1	47580	.	G	a	100	*	TYPE=SUBSTITUTE
CM032587.1	47588	.	T	a	100	*	TYPE=SUBSTITUTE
CM032587.1	47590	.	C	t	100	*	TYPE=SUBSTITUTE
CM032587.1	47590	.	CTT	C	100	*	TYPE=DELETE
CM032587.1	47594	.	T	c	100	*	TYPE=SUBSTITUTE
CM032587.1	47594	.	T	c	100	*	TYPE=SUBSTITUTE
CM032587.1	47596	.	T	c	100	*	TYPE=SUBSTITUTE
CM032587.1	47596	.	T	c	100	*	TYPE=SUBSTITUTE
CM032587.1	47610	.	A	g	100	*	TYPE=SUBSTITUTE
CM032587.1	47611	.	T	c	100	*	TYPE=SUBSTITUTE
CM032587.1	47614	.	G	a	100	*	TYPE=SUBSTITUTE
CM032587.1	47614	.	G	a	100	*	TYPE=SUBSTITUTE
CM032587.1	47619	.	A	g	100	*	TYPE=SUBSTITUTE
CM032587.1	47623	.	A	g	100	*	TYPE=SUBSTITUTE
CM032587.1	47624	.	G	a	100	*	TYPE=SUBSTITUTE
CM032587.1	47624	.	G	a	100	*	TYPE=SUBSTITUTE
CM032587.1	47627	.	C	a	100	*	TYPE=SUBSTITUTE
CM032587.1	47627	.	C	t	100	*	TYPE=SUBSTITUTE
CM032587.1	47627	.	C	g	100	*	TYPE=SUBSTITUTE
CM032587.1	47633	.	G	a	100	*	TYPE=SUBSTITUTE
CM032587.1	47633	.	G	a	100	*	TYPE=SUBSTITUTE
CM032587.1	47633	.	G	a	100	*	TYPE=SUBSTITUTE
CM032587.1	47634	.	G	a	100	*	TYPE=SUBSTITUTE
CM032587.1	47634	.	G	a	100	*	TYPE=SUBSTITUTE
CM032587.1	47634	.	G	a	100	*	TYPE=SUBSTITUTE
CM032587.1	47636	.	G	t	100	*	TYPE=SUBSTITUTE
CM032587.1	47636	.	G	t	100	*	TYPE=SUBSTITUTE
CM032587.1	47638	.	T	c	100	*	TYPE=SUBSTITUTE
CM032587.1	47638	.	T	c	100	*	TYPE=SUBSTITUTE
CM032587.1	47646	.	t	ta	100	*	TYPE=INSERT
CM032587.1	47649	.	G	a	100	*	TYPE=SUBSTITUTE
CM032587.1	47649	.	G	a	100	*	TYPE=SUBSTITUTE
CM032587.1	47650	.	G	a	100	*	TYPE=SUBSTITUTE
CM032587.1	47650	.	G	a	100	*	TYPE=SUBSTITUTE
CM032587.1	47651	.	G	a	100	*	TYPE=SUBSTITUTE
CM032587.1	47651	.	G	a	100	*	TYPE=SUBSTITUTE
CM032587.1	47651	.	G	a	100	*	TYPE=SUBSTITUTE
CM032587.1	47652	.	T	c	100	*	TYPE=SUBSTITUTE
CM032587.1	47660	.	A	g	100	*	TYPE=SUBSTITUTE
CM032587.1	47660	.	A	g	100	*	TYPE=SUBSTITUTE
CM032587.1	47660	.	A	g	100	*	TYPE=SUBSTITUTE
CM032587.1	47660	.	A	g	100	*	TYPE=SUBSTITUTE
CM032587.1	47669	.	C	t	100	*	TYPE=SUBSTITUTE
CM032587.1	47672	.	AAGC	A	100	*	TYPE=DELETE
CM032587.1	47675	.	C	t	100	*	TYPE=SUBSTITUTE
CM032587.1	47675	.	C	t	100	*	TYPE=SUBSTITUTE
CM032587.1	47675	.	C	t	100	*	TYPE=SUBSTITUTE
CM032587.1	47676	.	G	a	100	*	TYPE=SUBSTITUTE
CM032587.1	47676	.	G	a	100	*	TYPE=SUBSTITUTE
CM032587.1	47676	.	G	a	100	*	TYPE=SUBSTITUTE
CM032587.1	47677	.	G	a	100	*	TYPE=SUBSTITUTE
CM032587.1	47677	.	G	a	100	*	TYPE=SUBSTITUTE
CM032587.1	47677	.	G	a	100	*	TYPE=SUBSTITUTE
CM032587.1	47678	.	A	t	100	*	TYPE=SUBSTITUTE
CM032587.1	47680	.	AT	A	100	*	TYPE=DELETE
CM032587.1	47684	.	A	g	100	*	TYPE=SUBSTITUTE
CM032587.1	47687	.	G	a	100	*	TYPE=SUBSTITUTE
CM032587.1	47687	.	G	a	100	*	TYPE=SUBSTITUTE
CM032587.1	47688	.	T	g	100	*	TYPE=SUBSTITUTE
CM032587.1	47690	.	A	t	100	*	TYPE=SUBSTITUTE

CM032587.1	47690	.	A	t	100	*	TYPE=SUBSTITUTE
CM032587.1	47692	.	T	c	100	*	TYPE=SUBSTITUTE
CM032587.1	47692	.	T	c	100	*	TYPE=SUBSTITUTE
CM032587.1	47697	.	G	a	100	*	TYPE=SUBSTITUTE
CM032587.1	47697	.	G	c	100	*	TYPE=SUBSTITUTE
CM032587.1	47697	.	G	a	100	*	TYPE=SUBSTITUTE
CM032587.1	47700	.	TTATTATATTTAAAA	T	100	*	TYPE=DELETE
CM032587.1	47710	.	TA	T	100	*	TYPE=DELETE
CM032587.1	47715	.	A	g	100	*	TYPE=SUBSTITUTE
CM032587.1	47715	.	A	g	100	*	TYPE=SUBSTITUTE
CM032587.1	47716	.	A	c	100	*	TYPE=SUBSTITUTE
CM032587.1	47716	.	A	c	100	*	TYPE=SUBSTITUTE
CM032587.1	47718	.	AAT	A	100	*	TYPE=DELETE
CM032587.1	47719	.	A	g	100	*	TYPE=SUBSTITUTE
CM032587.1	47719	.	A	g	100	*	TYPE=SUBSTITUTE
CM032587.1	47724	.	C	t	100	*	TYPE=SUBSTITUTE
CM032587.1	47724	.	C	t	100	*	TYPE=SUBSTITUTE
CM032587.1	47724	.	C	t	100	*	TYPE=SUBSTITUTE
CM032587.1	47724	.	C	t	100	*	TYPE=SUBSTITUTE
CM032587.1	47738	.	C	t	100	*	TYPE=SUBSTITUTE
CM032587.1	47739	.	T	c	100	*	TYPE=SUBSTITUTE
CM032587.1	47744	.	A	t	100	*	TYPE=SUBSTITUTE
CM032587.1	47744	.	AG	A	100	*	TYPE=DELETE
CM032587.1	47745	.	G	a	100	*	TYPE=SUBSTITUTE
CM032587.1	47745	.	G	a	100	*	TYPE=SUBSTITUTE
CM032587.1	47745	.	G	a	100	*	TYPE=SUBSTITUTE
CM032587.1	47747	.	C	t	100	*	TYPE=SUBSTITUTE
CM032587.1	47747	.	C	t	100	*	TYPE=SUBSTITUTE
CM032587.1	47747	.	C	t	100	*	TYPE=SUBSTITUTE
CM032587.1	47747	.	C	t	100	*	TYPE=SUBSTITUTE
CM032587.1	47748	.	C	t	100	*	TYPE=SUBSTITUTE
CM032587.1	47748	.	C	t	100	*	TYPE=SUBSTITUTE
CM032587.1	47748	.	C	t	100	*	TYPE=SUBSTITUTE
CM032587.1	47748	.	C	t	100	*	TYPE=SUBSTITUTE
CM032587.1	47751	.	G	a	100	*	TYPE=SUBSTITUTE
CM032587.1	47751	.	G	a	100	*	TYPE=SUBSTITUTE
CM032587.1	47756	.	A	g	100	*	TYPE=SUBSTITUTE
CM032587.1	47759	.	C	t	100	*	TYPE=SUBSTITUTE
CM032587.1	47764	.	G	a	100	*	TYPE=SUBSTITUTE
CM032587.1	47764	.	G	a	100	*	TYPE=SUBSTITUTE
CM032587.1	47765	.	G	a	100	*	TYPE=SUBSTITUTE
CM032587.1	47775	.	C	t	100	*	TYPE=SUBSTITUTE
CM032587.1	47780	.	G	a	100	*	TYPE=SUBSTITUTE
CM032587.1	47786	.	T	a	100	*	TYPE=SUBSTITUTE
CM032587.1	47786	.	T	a	100	*	TYPE=SUBSTITUTE
CM032587.1	47789	.	G	a	100	*	TYPE=SUBSTITUTE
CM032587.1	47789	.	G	a	100	*	TYPE=SUBSTITUTE
CM032587.1	47789	.	G	a	100	*	TYPE=SUBSTITUTE
CM032587.1	47794	.	a	ag	100	*	TYPE=INSERT
CM032587.1	47795	.	A	g	100	*	TYPE=SUBSTITUTE
CM032587.1	47795	.	A	g	100	*	TYPE=SUBSTITUTE
CM032587.1	47796	.	A	G	100	*	TYPE=SUBSTITUTE
CM032587.1	47796	.	A	g	100	*	TYPE=SUBSTITUTE
CM032587.1	47796	.	A	g	100	*	TYPE=SUBSTITUTE
CM032587.1	47796	.	A	g	100	*	TYPE=SUBSTITUTE
CM032587.1	47800	.	C	t	100	*	TYPE=SUBSTITUTE
CM032587.1	47800	.	C	t	100	*	TYPE=SUBSTITUTE
CM032587.1	47800	.	C	T	100	*	TYPE=SUBSTITUTE
CM032587.1	47801	.	C	t	100	*	TYPE=SUBSTITUTE
CM032587.1	47801	.	C	a	100	*	TYPE=SUBSTITUTE
CM032587.1	47801	.	C	A	100	*	TYPE=SUBSTITUTE
CM032587.1	47804	.	A	t	100	*	TYPE=SUBSTITUTE
CM032587.1	47805	.	G	a	100	*	TYPE=SUBSTITUTE
CM032587.1	47805	.	G	a	100	*	TYPE=SUBSTITUTE

CM032587.1	47806	.	G	a	100	*	TYPE=SUBSTITUTE
CM032587.1	47806	.	G	a	100	*	TYPE=SUBSTITUTE
CM032587.1	47807	.	G	a	100	*	TYPE=SUBSTITUTE
CM032587.1	47807	.	G	A	100	*	TYPE=SUBSTITUTE
CM032587.1	47807	.	G	a	100	*	TYPE=SUBSTITUTE
CM032587.1	47808	.	A	G	100	*	TYPE=SUBSTITUTE
CM032587.1	47809	.	G	a	100	*	TYPE=SUBSTITUTE
CM032587.1	47819	.	G	a	100	*	TYPE=SUBSTITUTE
CM032587.1	47824	.	T	C	100	*	TYPE=SUBSTITUTE
CM032587.1	47827	.	T	c	100	*	TYPE=SUBSTITUTE
CM032587.1	47833	.	A	G	100	*	TYPE=SUBSTITUTE
CM032587.1	47833	.	A	g	100	*	TYPE=SUBSTITUTE
CM032587.1	47834	.	A	g	100	*	TYPE=SUBSTITUTE
CM032587.1	47834	.	A	g	100	*	TYPE=SUBSTITUTE
CM032587.1	47834	.	A	g	100	*	TYPE=SUBSTITUTE
CM032587.1	47834	.	A	g	100	*	TYPE=SUBSTITUTE
CM032587.1	47835	.	A	g	100	*	TYPE=SUBSTITUTE
CM032587.1	47836	.	AG	A	100	*	TYPE=DELETE
CM032587.1	47836	.	AG	A	100	*	TYPE=DELETE
CM032587.1	47838	.	C	t	100	*	TYPE=SUBSTITUTE
CM032587.1	47838	.	C	t	100	*	TYPE=SUBSTITUTE
CM032587.1	47838	.	C	t	100	*	TYPE=SUBSTITUTE
CM032587.1	47838	.	C	t	100	*	TYPE=SUBSTITUTE
CM032587.1	47850	.	C	t	100	*	TYPE=SUBSTITUTE
CM032587.1	47850	.	C	t	100	*	TYPE=SUBSTITUTE
CM032587.1	47850	.	C	t	100	*	TYPE=SUBSTITUTE
CM032587.1	47856	.	C	t	100	*	TYPE=SUBSTITUTE
CM032587.1	47856	.	C	t	100	*	TYPE=SUBSTITUTE
CM032587.1	47859	.	G	a	100	*	TYPE=SUBSTITUTE
CM032587.1	47859	.	G	a	100	*	TYPE=SUBSTITUTE
CM032587.1	47859	.	G	a	100	*	TYPE=SUBSTITUTE
CM032587.1	47859	.	G	a	100	*	TYPE=SUBSTITUTE
CM032587.1	47860	.	G	a	100	*	TYPE=SUBSTITUTE
CM032587.1	47861	.	T	c	100	*	TYPE=SUBSTITUTE
CM032587.1	47861	.	T	c	100	*	TYPE=SUBSTITUTE
CM032587.1	47867	.	A	g	100	*	TYPE=SUBSTITUTE
CM032587.1	47872	.	T	c	100	*	TYPE=SUBSTITUTE
CM032587.1	47873	.	A	c	100	*	TYPE=SUBSTITUTE
CM032587.1	47873	.	A	t	100	*	TYPE=SUBSTITUTE
CM032587.1	47878	.	AG	A	100	*	TYPE=DELETE
CM032587.1	47879	.	G	a	100	*	TYPE=SUBSTITUTE
CM032587.1	47879	.	G	a	100	*	TYPE=SUBSTITUTE
CM032587.1	47880	.	A	g	100	*	TYPE=SUBSTITUTE
CM032587.1	47880	.	A	t	100	*	TYPE=SUBSTITUTE
CM032587.1	47882	.	A	g	100	*	TYPE=SUBSTITUTE
CM032587.1	47891	.	A	g	100	*	TYPE=SUBSTITUTE
CM032587.1	47891	.	A	g	100	*	TYPE=SUBSTITUTE
CM032587.1	47893	.	T	a	100	*	TYPE=SUBSTITUTE
CM032587.1	47893	.	T	a	100	*	TYPE=SUBSTITUTE
CM032587.1	47894	.	A	g	100	*	TYPE=SUBSTITUTE
CM032587.1	47894	.	A	g	100	*	TYPE=SUBSTITUTE
CM032587.1	47906	.	A	g	100	*	TYPE=SUBSTITUTE
CM032587.1	47909	.	C	t	100	*	TYPE=SUBSTITUTE
CM032587.1	47911	.	T	c	100	*	TYPE=SUBSTITUTE
CM032587.1	47911	.	T	c	100	*	TYPE=SUBSTITUTE
CM032587.1	47912	.	T	c	100	*	TYPE=SUBSTITUTE
CM032587.1	47913	.	C	t	100	*	TYPE=SUBSTITUTE
CM032587.1	47913	.	C	t	100	*	TYPE=SUBSTITUTE
CM032587.1	47913	.	C	t	100	*	TYPE=SUBSTITUTE
CM032587.1	47918	.	G	a	100	*	TYPE=SUBSTITUTE
CM032587.1	47918	.	G	a	100	*	TYPE=SUBSTITUTE
CM032587.1	47918	.	G	a	100	*	TYPE=SUBSTITUTE
CM032587.1	47919	.	T	a	100	*	TYPE=SUBSTITUTE
CM032587.1	47919	.	T	a	100	*	TYPE=SUBSTITUTE

CM032587.1	47920	.	T	a	100	*	TYPE=SUBSTITUTE
CM032587.1	47920	.	T	a	100	*	TYPE=SUBSTITUTE
CM032587.1	47922	.	G	a	100	*	TYPE=SUBSTITUTE
CM032587.1	47922	.	G	a	100	*	TYPE=SUBSTITUTE
CM032587.1	47922	.	G	a	100	*	TYPE=SUBSTITUTE
CM032587.1	47931	.	TA	T	100	*	TYPE=DELETE
CM032587.1	47934	.	A	g	100	*	TYPE=SUBSTITUTE
CM032587.1	47935	.	A	t	100	*	TYPE=SUBSTITUTE
CM032587.1	47935	.	A	t	100	*	TYPE=SUBSTITUTE
CM032587.1	47937	.	A	g	100	*	TYPE=SUBSTITUTE
CM032587.1	47938	.	T	c	100	*	TYPE=SUBSTITUTE
CM032587.1	47939	.	A	t	100	*	TYPE=SUBSTITUTE
CM032587.1	47939	.	A	t	100	*	TYPE=SUBSTITUTE
CM032587.1	47944	.	A	G	100	*	TYPE=SUBSTITUTE
CM032587.1	47945	.	G	A	100	*	TYPE=SUBSTITUTE
CM032587.1	47945	.	G	a	100	*	TYPE=SUBSTITUTE
CM032587.1	47945	.	G	a	100	*	TYPE=SUBSTITUTE
CM032587.1	47945	.	G	a	100	*	TYPE=SUBSTITUTE
CM032587.1	47946	.	T	c	100	*	TYPE=SUBSTITUTE
CM032587.1	47946	.	T	c	100	*	TYPE=SUBSTITUTE
CM032587.1	47947	.	T	c	100	*	TYPE=SUBSTITUTE
CM032587.1	47952	.	G	A	100	*	TYPE=SUBSTITUTE
CM032587.1	47954	.	C	t	100	*	TYPE=SUBSTITUTE
CM032587.1	47955	.	C	t	100	*	TYPE=SUBSTITUTE
CM032587.1	47955	.	C	t	100	*	TYPE=SUBSTITUTE
CM032587.1	47956	.	C	t	100	*	TYPE=SUBSTITUTE
CM032587.1	47956	.	C	t	100	*	TYPE=SUBSTITUTE
CM032587.1	47964	.	A	g	100	*	TYPE=SUBSTITUTE
CM032587.1	47964	.	A	g	100	*	TYPE=SUBSTITUTE
CM032587.1	47964	.	A	g	100	*	TYPE=SUBSTITUTE
CM032587.1	47970	.	AC	A	100	*	TYPE=DELETE
CM032587.1	47970	.	AC	A	100	*	TYPE=DELETE
CM032587.1	47971	.	C	t	100	*	TYPE=SUBSTITUTE
CM032587.1	47972	.	T	c	100	*	TYPE=SUBSTITUTE
CM032587.1	47974	.	T	c	100	*	TYPE=SUBSTITUTE
CM032587.1	47974	.	T	c	100	*	TYPE=SUBSTITUTE
CM032587.1	47978	.	T	a	100	*	TYPE=SUBSTITUTE
CM032587.1	47979	.	a	ag	100	*	TYPE=INSERT
CM032587.1	47980	.	A	g	100	*	TYPE=SUBSTITUTE
CM032587.1	47980	.	A	g	100	*	TYPE=SUBSTITUTE
CM032587.1	47980	.	A	g	100	*	TYPE=SUBSTITUTE
CM032587.1	47981	.	G	a	100	*	TYPE=SUBSTITUTE
CM032587.1	47986	.	A	g	100	*	TYPE=SUBSTITUTE
CM032587.1	47986	.	A	g	100	*	TYPE=SUBSTITUTE
CM032587.1	47987	.	t	ta	100	*	TYPE=INSERT
CM032587.1	47993	.	T	a	100	*	TYPE=SUBSTITUTE
CM032587.1	47993	.	T	a	100	*	TYPE=SUBSTITUTE
CM032587.1	47994	.	A	g	100	*	TYPE=SUBSTITUTE
CM032587.1	48003	.	A	g	100	*	TYPE=SUBSTITUTE
CM032587.1	48003	.	A	g	100	*	TYPE=SUBSTITUTE
CM032587.1	48005	.	C	t	100	*	TYPE=SUBSTITUTE
CM032587.1	48005	.	C	t	100	*	TYPE=SUBSTITUTE
CM032587.1	48005	.	C	t	100	*	TYPE=SUBSTITUTE
CM032587.1	48005	.	C	t	100	*	TYPE=SUBSTITUTE
CM032587.1	48006	.	C	t	100	*	TYPE=SUBSTITUTE
CM032587.1	48006	.	C	t	100	*	TYPE=SUBSTITUTE
CM032587.1	48006	.	C	t	100	*	TYPE=SUBSTITUTE
CM032587.1	48009	.	C	t	100	*	TYPE=SUBSTITUTE
CM032587.1	48009	.	C	t	100	*	TYPE=SUBSTITUTE
CM032587.1	48017	.	A	t	100	*	TYPE=SUBSTITUTE
CM032587.1	48017	.	A	t	100	*	TYPE=SUBSTITUTE
CM032587.1	48023	.	T	a	100	*	TYPE=SUBSTITUTE
CM032587.1	48023	.	T	a	100	*	TYPE=SUBSTITUTE
CM032587.1	48024	.	A	g	100	*	TYPE=SUBSTITUTE

CM032587.1	48024	.	A	g	100	*	TYPE=SUBSTITUTE
CM032587.1	48050	.	AA	A	100	*	TYPE=DELETE
CM032587.1	48053	.	C	t	100	*	TYPE=SUBSTITUTE
CM032587.1	48053	.	C	t	100	*	TYPE=SUBSTITUTE
CM032587.1	48059	.	T	a	100	*	TYPE=SUBSTITUTE
CM032587.1	48059	.	T	a	100	*	TYPE=SUBSTITUTE
CM032587.1	48060	.	A	g	100	*	TYPE=SUBSTITUTE
CM032587.1	48061	.	A	t	100	*	TYPE=SUBSTITUTE
CM032587.1	48061	.	A	t	100	*	TYPE=SUBSTITUTE
CM032587.1	48067	.	C	t	100	*	TYPE=SUBSTITUTE
CM032587.1	48067	.	C	t	100	*	TYPE=SUBSTITUTE
CM032587.1	48069	.	T	a	100	*	TYPE=SUBSTITUTE
CM032587.1	48070	.	C	t	100	*	TYPE=SUBSTITUTE
CM032587.1	48070	.	C	t	100	*	TYPE=SUBSTITUTE
CM032587.1	48075	.	a	at	100	*	TYPE=INSERT
CM032587.1	48082	.	A	g	100	*	TYPE=SUBSTITUTE
CM032587.1	48082	.	A	g	100	*	TYPE=SUBSTITUTE
CM032587.1	48083	.	A	t	100	*	TYPE=SUBSTITUTE
CM032587.1	48083	.	A	t	100	*	TYPE=SUBSTITUTE
CM032587.1	48086	.	A	t	100	*	TYPE=SUBSTITUTE
CM032587.1	48086	.	A	t	100	*	TYPE=SUBSTITUTE
CM032587.1	48090	.	T	c	100	*	TYPE=SUBSTITUTE
CM032587.1	48090	.	T	c	100	*	TYPE=SUBSTITUTE
CM032587.1	48094	.	C	t	100	*	TYPE=SUBSTITUTE
CM032587.1	48094	.	C	t	100	*	TYPE=SUBSTITUTE
CM032587.1	48094	.	C	t	100	*	TYPE=SUBSTITUTE
CM032587.1	48097	.	C	t	100	*	TYPE=SUBSTITUTE
CM032587.1	48097	.	C	t	100	*	TYPE=SUBSTITUTE
CM032587.1	48099	.	A	t	100	*	TYPE=SUBSTITUTE
CM032587.1	48099	.	A	t	100	*	TYPE=SUBSTITUTE
CM032587.1	48100	.	C	a	100	*	TYPE=SUBSTITUTE
CM032587.1	48100	.	C	a	100	*	TYPE=SUBSTITUTE
CM032587.1	48100	.	C	t	100	*	TYPE=SUBSTITUTE
CM032587.1	48100	.	C	t	100	*	TYPE=SUBSTITUTE
CM032587.1	48103	.	G	a	100	*	TYPE=SUBSTITUTE
CM032587.1	48104	.	C	t	100	*	TYPE=SUBSTITUTE
CM032587.1	48104	.	C	t	100	*	TYPE=SUBSTITUTE
CM032587.1	48104	.	C	t	100	*	TYPE=SUBSTITUTE
CM032587.1	48109	.	G	a	100	*	TYPE=SUBSTITUTE
CM032587.1	48109	.	G	a	100	*	TYPE=SUBSTITUTE
CM032587.1	48127	.	T	a	100	*	TYPE=SUBSTITUTE
CM032587.1	48127	.	T	a	100	*	TYPE=SUBSTITUTE
CM032587.1	48136	.	A	t	100	*	TYPE=SUBSTITUTE
CM032587.1	48137	.	A	g	100	*	TYPE=SUBSTITUTE
CM032587.1	48143	.	a	at	100	*	TYPE=INSERT
CM032587.1	48144	.	C	t	100	*	TYPE=SUBSTITUTE
CM032587.1	48145	.	C	t	100	*	TYPE=SUBSTITUTE
CM032587.1	48146	.	T	c	100	*	TYPE=SUBSTITUTE
CM032587.1	48147	.	C	T	100	*	TYPE=SUBSTITUTE
CM032587.1	48147	.	C	t	100	*	TYPE=SUBSTITUTE
CM032587.1	48151	.	G	a	100	*	TYPE=SUBSTITUTE
CM032587.1	48151	.	G	A	100	*	TYPE=SUBSTITUTE
CM032587.1	48152	.	G	a	100	*	TYPE=SUBSTITUTE
CM032587.1	48153	.	G	a	100	*	TYPE=SUBSTITUTE
CM032587.1	48169	.	T	c	100	*	TYPE=SUBSTITUTE
CM032587.1	48169	.	T	c	100	*	TYPE=SUBSTITUTE
CM032587.1	48169	.	T	c	100	*	TYPE=SUBSTITUTE
CM032587.1	48169	.	T	c	100	*	TYPE=SUBSTITUTE
CM032587.1	48170	.	T	c	100	*	TYPE=SUBSTITUTE
CM032587.1	48170	.	T	c	100	*	TYPE=SUBSTITUTE
CM032587.1	48172	.	T	c	100	*	TYPE=SUBSTITUTE
CM032587.1	48179	.	G	a	100	*	TYPE=SUBSTITUTE
CM032587.1	48183	.	G	a	100	*	TYPE=SUBSTITUTE
CM032587.1	48188	.	a	at	100	*	TYPE=INSERT

CM032587.1	48190	.	C	t	100	*	TYPE=SUBSTITUTE
CM032587.1	48190	.	C	t	100	*	TYPE=SUBSTITUTE
CM032587.1	48203	.	C	t	100	*	TYPE=SUBSTITUTE
CM032587.1	48203	.	C	t	100	*	TYPE=SUBSTITUTE
CM032587.1	48213	.	C	t	100	*	TYPE=SUBSTITUTE
CM032587.1	48213	.	C	t	100	*	TYPE=SUBSTITUTE
CM032587.1	48213	.	C	t	100	*	TYPE=SUBSTITUTE
CM032587.1	48213	.	C	t	100	*	TYPE=SUBSTITUTE
CM032587.1	48220	.	A	g	100	*	TYPE=SUBSTITUTE
CM032587.1	48220	.	A	g	100	*	TYPE=SUBSTITUTE
CM032587.1	48221	.	T	c	100	*	TYPE=SUBSTITUTE
CM032587.1	48223	.	C	t	100	*	TYPE=SUBSTITUTE
CM032587.1	48223	.	C	t	100	*	TYPE=SUBSTITUTE
CM032587.1	48223	.	C	t	100	*	TYPE=SUBSTITUTE
CM032587.1	48223	.	C	t	100	*	TYPE=SUBSTITUTE
CM032587.1	48231	.	A	g	100	*	TYPE=SUBSTITUTE
CM032587.1	48231	.	A	g	100	*	TYPE=SUBSTITUTE
CM032587.1	48231	.	A	g	100	*	TYPE=SUBSTITUTE
CM032587.1	48233	.	C	t	100	*	TYPE=SUBSTITUTE
CM032587.1	48233	.	C	t	100	*	TYPE=SUBSTITUTE
CM032587.1	48233	.	C	t	100	*	TYPE=SUBSTITUTE
CM032587.1	48233	.	C	t	100	*	TYPE=SUBSTITUTE
CM032587.1	48241	.	C	t	100	*	TYPE=SUBSTITUTE
CM032587.1	48241	.	C	t	100	*	TYPE=SUBSTITUTE
CM032587.1	48241	.	C	t	100	*	TYPE=SUBSTITUTE
CM032587.1	48246	.	AACT	A	100	*	TYPE=DELETE
CM032587.1	48247	.	A	g	100	*	TYPE=SUBSTITUTE
CM032587.1	48247	.	A	g	100	*	TYPE=SUBSTITUTE
CM032587.1	48247	.	A	g	100	*	TYPE=SUBSTITUTE
CM032587.1	48248	.	C	t	100	*	TYPE=SUBSTITUTE
CM032587.1	48250	.	T	g	100	*	TYPE=SUBSTITUTE
CM032587.1	48252	.	C	t	100	*	TYPE=SUBSTITUTE
CM032587.1	48252	.	C	t	100	*	TYPE=SUBSTITUTE
CM032587.1	48252	.	C	t	100	*	TYPE=SUBSTITUTE
CM032587.1	48263	.	A	g	100	*	TYPE=SUBSTITUTE
CM032587.1	48263	.	A	g	100	*	TYPE=SUBSTITUTE
CM032587.1	48263	.	A	g	100	*	TYPE=SUBSTITUTE
CM032587.1	48265	.	C	t	100	*	TYPE=SUBSTITUTE
CM032587.1	48265	.	C	t	100	*	TYPE=SUBSTITUTE
CM032587.1	48265	.	C	t	100	*	TYPE=SUBSTITUTE
CM032587.1	48265	.	C	t	100	*	TYPE=SUBSTITUTE
CM032587.1	48268	.	A	g	100	*	TYPE=SUBSTITUTE
CM032587.1	48268	.	A	g	100	*	TYPE=SUBSTITUTE
CM032587.1	48269	.	G	t	100	*	TYPE=SUBSTITUTE
CM032587.1	48269	.	GAA	G	100	*	TYPE=DELETE
CM032587.1	48269	.	G	a	100	*	TYPE=SUBSTITUTE
CM032587.1	48269	.	G	t	100	*	TYPE=SUBSTITUTE
CM032587.1	48271	.	A	g	100	*	TYPE=SUBSTITUTE
CM032587.1	48272	.	A	g	100	*	TYPE=SUBSTITUTE
CM032587.1	48274	.	C	t	100	*	TYPE=SUBSTITUTE
CM032587.1	48274	.	C	t	100	*	TYPE=SUBSTITUTE
CM032587.1	48284	.	A	g	100	*	TYPE=SUBSTITUTE
CM032587.1	48290	.	G	a	100	*	TYPE=SUBSTITUTE
CM032587.1	48290	.	G	a	100	*	TYPE=SUBSTITUTE
CM032587.1	48290	.	G	a	100	*	TYPE=SUBSTITUTE
CM032587.1	48290	.	G	a	100	*	TYPE=SUBSTITUTE
CM032587.1	48295	.	TA	T	100	*	TYPE=DELETE
CM032587.1	48298	.	A	g	100	*	TYPE=SUBSTITUTE
CM032587.1	48299	.	A	t	100	*	TYPE=SUBSTITUTE
CM032587.1	48299	.	A	g	100	*	TYPE=SUBSTITUTE
CM032587.1	48300	.	C	t	100	*	TYPE=SUBSTITUTE
CM032587.1	48304	.	C	t	100	*	TYPE=SUBSTITUTE
CM032587.1	48304	.	C	t	100	*	TYPE=SUBSTITUTE
CM032587.1	48305	.	C	t	100	*	TYPE=SUBSTITUTE

CM032587.1	48305	.	C	t	100	*	TYPE=SUBSTITUTE
CM032587.1	48305	.	C	t	100	*	TYPE=SUBSTITUTE
CM032587.1	48309	.	A	g	100	*	TYPE=SUBSTITUTE
CM032587.1	48309	.	A	g	100	*	TYPE=SUBSTITUTE
CM032587.1	48314	.	A	t	100	*	TYPE=SUBSTITUTE
CM032587.1	48314	.	A	t	100	*	TYPE=SUBSTITUTE
CM032587.1	48317	.	a	ag	100	*	TYPE=INSERT
CM032587.1	48317	.	a	ag	100	*	TYPE=INSERT
CM032587.1	48318	.	A	t	100	*	TYPE=SUBSTITUTE
CM032587.1	48318	.	A	t	100	*	TYPE=SUBSTITUTE
CM032587.1	48318	.	a	at	100	*	TYPE=INSERT
CM032587.1	48321	.	t	tt	100	*	TYPE=INSERT
CM032587.1	48325	.	TATA	T	100	*	TYPE=DELETE
CM032587.1	48325	.	TATA	T	100	*	TYPE=DELETE
CM032587.1	48328	.	a	at	100	*	TYPE=INSERT
CM032587.1	48332	.	T	a	100	*	TYPE=SUBSTITUTE
CM032587.1	48332	.	T	a	100	*	TYPE=SUBSTITUTE
CM032587.1	48333	.	T	a	100	*	TYPE=SUBSTITUTE
CM032587.1	48333	.	T	a	100	*	TYPE=SUBSTITUTE
CM032587.1	48335	.	T	c	100	*	TYPE=SUBSTITUTE
CM032587.1	48336	.	T	c	100	*	TYPE=SUBSTITUTE
CM032587.1	48337	.	A	t	100	*	TYPE=SUBSTITUTE
CM032587.1	48347	.	A	t	100	*	TYPE=SUBSTITUTE
CM032587.1	48347	.	A	t	100	*	TYPE=SUBSTITUTE
CM032587.1	48349	.	A	g	100	*	TYPE=SUBSTITUTE
CM032587.1	48349	.	A	g	100	*	TYPE=SUBSTITUTE
CM032587.1	48351	.	C	t	100	*	TYPE=SUBSTITUTE
CM032587.1	48354	.	G	a	100	*	TYPE=SUBSTITUTE
CM032587.1	48357	.	T	C	100	*	TYPE=SUBSTITUTE
CM032587.1	48359	.	T	C	100	*	TYPE=SUBSTITUTE
CM032587.1	48359	.	t	ta	100	*	TYPE=INSERT
CM032587.1	48365	.	C	t	100	*	TYPE=SUBSTITUTE
CM032587.1	48365	.	C	t	100	*	TYPE=SUBSTITUTE
CM032587.1	48365	.	C	t	100	*	TYPE=SUBSTITUTE
CM032587.1	48367	.	T	a	100	*	TYPE=SUBSTITUTE
CM032587.1	48367	.	T	a	100	*	TYPE=SUBSTITUTE
CM032587.1	48369	.	T	a	100	*	TYPE=SUBSTITUTE
CM032587.1	48369	.	T	a	100	*	TYPE=SUBSTITUTE
CM032587.1	48371	.	A	g	100	*	TYPE=SUBSTITUTE
CM032587.1	48371	.	A	g	100	*	TYPE=SUBSTITUTE
CM032587.1	48371	.	A	G	100	*	TYPE=SUBSTITUTE
CM032587.1	48372	.	A	c	100	*	TYPE=SUBSTITUTE
CM032587.1	48372	.	A	t	100	*	TYPE=SUBSTITUTE
CM032587.1	48373	.	A	t	100	*	TYPE=SUBSTITUTE
CM032587.1	48373	.	A	g	100	*	TYPE=SUBSTITUTE
CM032587.1	48373	.	A	t	100	*	TYPE=SUBSTITUTE
CM032587.1	48373	.	A	g	100	*	TYPE=SUBSTITUTE
CM032587.1	48374	.	A	g	100	*	TYPE=SUBSTITUTE
CM032587.1	48375	.	A	g	100	*	TYPE=SUBSTITUTE
CM032587.1	48376	.	A	g	100	*	TYPE=SUBSTITUTE
CM032587.1	48376	.	A	g	100	*	TYPE=SUBSTITUTE
CM032587.1	48378	.	A	t	100	*	TYPE=SUBSTITUTE
CM032587.1	48378	.	A	t	100	*	TYPE=SUBSTITUTE
CM032587.1	48379	.	C	t	100	*	TYPE=SUBSTITUTE
CM032587.1	48379	.	C	t	100	*	TYPE=SUBSTITUTE
CM032587.1	48379	.	C	t	100	*	TYPE=SUBSTITUTE
CM032587.1	48382	.	t	ta	100	*	TYPE=INSERT
CM032587.1	48382	.	t	ta	100	*	TYPE=INSERT
CM032587.1	48384	.	G	a	100	*	TYPE=SUBSTITUTE
CM032587.1	48384	.	G	a	100	*	TYPE=SUBSTITUTE
CM032587.1	48384	.	G	a	100	*	TYPE=SUBSTITUTE
CM032587.1	48384	.	G	a	100	*	TYPE=SUBSTITUTE
CM032587.1	48385	.	G	a	100	*	TYPE=SUBSTITUTE
CM032587.1	48385	.	G	a	100	*	TYPE=SUBSTITUTE

CM032587.1	48388	.	AT	A	100	*	TYPE=DELETE
CM032587.1	48390	.	T	a	100	*	TYPE=SUBSTITUTE
CM032587.1	48392	.	A	g	100	*	TYPE=SUBSTITUTE
CM032587.1	48393	.	T	g	100	*	TYPE=SUBSTITUTE
CM032587.1	48394	.	TCC	T	100	*	TYPE=DELETE
CM032587.1	48395	.	C	t	100	*	TYPE=SUBSTITUTE
CM032587.1	48396	.	C	t	100	*	TYPE=SUBSTITUTE
CM032587.1	48403	.	A	t	100	*	TYPE=SUBSTITUTE
CM032587.1	48403	.	A	t	100	*	TYPE=SUBSTITUTE
CM032587.1	48404	.	A	g	100	*	TYPE=SUBSTITUTE
CM032587.1	48405	.	G	t	100	*	TYPE=SUBSTITUTE
CM032587.1	48405	.	G	t	100	*	TYPE=SUBSTITUTE
CM032587.1	48406	.	G	t	100	*	TYPE=SUBSTITUTE
CM032587.1	48406	.	G	t	100	*	TYPE=SUBSTITUTE
CM032587.1	48409	.	TTTA	T	100	*	TYPE=DELETE
CM032587.1	48419	.	G	a	100	*	TYPE=SUBSTITUTE
CM032587.1	48419	.	G	a	100	*	TYPE=SUBSTITUTE
CM032587.1	48419	.	G	a	100	*	TYPE=SUBSTITUTE
CM032587.1	48419	.	G	a	100	*	TYPE=SUBSTITUTE
CM032587.1	48423	.	T	c	100	*	TYPE=SUBSTITUTE
CM032587.1	48426	.	C	t	100	*	TYPE=SUBSTITUTE
CM032587.1	48426	.	C	t	100	*	TYPE=SUBSTITUTE
CM032587.1	48426	.	C	t	100	*	TYPE=SUBSTITUTE
CM032587.1	48426	.	C	t	100	*	TYPE=SUBSTITUTE
CM032587.1	48427	.	T	a	100	*	TYPE=SUBSTITUTE
CM032587.1	48427	.	T	a	100	*	TYPE=SUBSTITUTE
CM032587.1	48438	.	T	c	100	*	TYPE=SUBSTITUTE
CM032587.1	48438	.	T	c	100	*	TYPE=SUBSTITUTE
CM032587.1	48444	.	C	t	100	*	TYPE=SUBSTITUTE
CM032587.1	48444	.	C	t	100	*	TYPE=SUBSTITUTE
CM032587.1	48444	.	C	t	100	*	TYPE=SUBSTITUTE
CM032587.1	48453	.	C	t	100	*	TYPE=SUBSTITUTE
CM032587.1	48454	.	C	t	100	*	TYPE=SUBSTITUTE
CM032587.1	48455	.	C	t	100	*	TYPE=SUBSTITUTE
CM032587.1	48455	.	C	t	100	*	TYPE=SUBSTITUTE
CM032587.1	48458	.	A	G	100	*	TYPE=SUBSTITUTE
CM032587.1	48458	.	A	G	100	*	TYPE=SUBSTITUTE
CM032587.1	48459	.	G	a	100	*	TYPE=SUBSTITUTE
CM032587.1	48461	.	T	C	100	*	TYPE=SUBSTITUTE
CM032587.1	48462	.	T	c	100	*	TYPE=SUBSTITUTE
CM032587.1	48462	.	T	C	100	*	TYPE=SUBSTITUTE
CM032587.1	48463	.	T	a	100	*	TYPE=SUBSTITUTE
CM032587.1	48463	.	T	a	100	*	TYPE=SUBSTITUTE
CM032587.1	48463	.	T	C	100	*	TYPE=SUBSTITUTE
CM032587.1	48464	.	T	c	100	*	TYPE=SUBSTITUTE
CM032587.1	48464	.	T	c	100	*	TYPE=SUBSTITUTE
CM032587.1	48469	.	A	g	100	*	TYPE=SUBSTITUTE
CM032587.1	48469	.	A	G	100	*	TYPE=SUBSTITUTE
CM032587.1	48470	.	T	c	100	*	TYPE=SUBSTITUTE
CM032587.1	48471	.	T	c	100	*	TYPE=SUBSTITUTE
CM032587.1	48479	.	T	c	100	*	TYPE=SUBSTITUTE
CM032587.1	48485	.	A	g	100	*	TYPE=SUBSTITUTE
CM032587.1	48486	.	A	t	100	*	TYPE=SUBSTITUTE
CM032587.1	48486	.	A	t	100	*	TYPE=SUBSTITUTE
CM032587.1	48493	.	A	t	100	*	TYPE=SUBSTITUTE
CM032587.1	48493	.	a	aa	100	*	TYPE=INSERT
CM032587.1	48493	.	A	t	100	*	TYPE=SUBSTITUTE
CM032587.1	48494	.	G	a	100	*	TYPE=SUBSTITUTE
CM032587.1	48494	.	G	a	100	*	TYPE=SUBSTITUTE
CM032587.1	48495	.	c	ct	100	*	TYPE=INSERT
CM032587.1	48498	.	T	C	100	*	TYPE=SUBSTITUTE
CM032587.1	48498	.	T	c	100	*	TYPE=SUBSTITUTE
CM032587.1	48498	.	T	c	100	*	TYPE=SUBSTITUTE
CM032587.1	48500	.	A	G	100	*	TYPE=SUBSTITUTE

CM032587.1	48501	.	C	t	100	*	TYPE=SUBSTITUTE
CM032587.1	48503	.	A	t	100	*	TYPE=SUBSTITUTE
CM032587.1	48503	.	A	T	100	*	TYPE=SUBSTITUTE
CM032587.1	48504	.	T	a	100	*	TYPE=SUBSTITUTE
CM032587.1	48506	.	A	g	100	*	TYPE=SUBSTITUTE
CM032587.1	48506	.	A	g	100	*	TYPE=SUBSTITUTE
CM032587.1	48506	.	A	g	100	*	TYPE=SUBSTITUTE
CM032587.1	48509	.	G	a	100	*	TYPE=SUBSTITUTE
CM032587.1	48509	.	G	a	100	*	TYPE=SUBSTITUTE
CM032587.1	48509	.	G	a	100	*	TYPE=SUBSTITUTE
CM032587.1	48509	.	G	a	100	*	TYPE=SUBSTITUTE
CM032587.1	48510	.	G	a	100	*	TYPE=SUBSTITUTE
CM032587.1	48510	.	G	a	100	*	TYPE=SUBSTITUTE
CM032587.1	48510	.	G	a	100	*	TYPE=SUBSTITUTE
CM032587.1	48511	.	G	a	100	*	TYPE=SUBSTITUTE
CM032587.1	48512	.	G	t	100	*	TYPE=SUBSTITUTE
CM032587.1	48512	.	G	t	100	*	TYPE=SUBSTITUTE
CM032587.1	48512	.	G	a	100	*	TYPE=SUBSTITUTE
CM032587.1	48513	.	G	t	100	*	TYPE=SUBSTITUTE
CM032587.1	48513	.	G	t	100	*	TYPE=SUBSTITUTE
CM032587.1	48514	.	C	g	100	*	TYPE=SUBSTITUTE
CM032587.1	48514	.	C	t	100	*	TYPE=SUBSTITUTE
CM032587.1	48514	.	C	t	100	*	TYPE=SUBSTITUTE
CM032587.1	48515	.	T	c	100	*	TYPE=SUBSTITUTE
CM032587.1	48515	.	T	a	100	*	TYPE=SUBSTITUTE
CM032587.1	48518	.	G	a	100	*	TYPE=SUBSTITUTE
CM032587.1	48518	.	G	a	100	*	TYPE=SUBSTITUTE
CM032587.1	48521	.	A	g	100	*	TYPE=SUBSTITUTE
CM032587.1	48522	.	T	a	100	*	TYPE=SUBSTITUTE
CM032587.1	48522	.	T	a	100	*	TYPE=SUBSTITUTE
CM032587.1	48523	.	A	t	100	*	TYPE=SUBSTITUTE
CM032587.1	48523	.	A	t	100	*	TYPE=SUBSTITUTE
CM032587.1	48528	.	A	g	100	*	TYPE=SUBSTITUTE
CM032587.1	48530	.	t	tg	100	*	TYPE=INSERT
CM032587.1	48532	.	AT	A	100	*	TYPE=DELETE
CM032587.1	48534	.	T	c	100	*	TYPE=SUBSTITUTE
CM032587.1	48535	.	T	c	100	*	TYPE=SUBSTITUTE
CM032587.1	48535	.	T	a	100	*	TYPE=SUBSTITUTE
CM032587.1	48535	.	T	a	100	*	TYPE=SUBSTITUTE
CM032587.1	48536	.	T	c	100	*	TYPE=SUBSTITUTE
CM032587.1	48542	.	T	c	100	*	TYPE=SUBSTITUTE
CM032587.1	48545	.	t	ta	100	*	TYPE=INSERT
CM032587.1	48551	.	A	c	100	*	TYPE=SUBSTITUTE
CM032587.1	48557	.	G	a	100	*	TYPE=SUBSTITUTE
CM032587.1	48557	.	G	a	100	*	TYPE=SUBSTITUTE
CM032587.1	48557	.	G	a	100	*	TYPE=SUBSTITUTE
CM032587.1	48557	.	G	a	100	*	TYPE=SUBSTITUTE
CM032587.1	48558	.	C	t	100	*	TYPE=SUBSTITUTE
CM032587.1	48558	.	C	t	100	*	TYPE=SUBSTITUTE
CM032587.1	48558	.	C	t	100	*	TYPE=SUBSTITUTE
CM032587.1	48568	.	C	t	100	*	TYPE=SUBSTITUTE
CM032587.1	48568	.	C	t	100	*	TYPE=SUBSTITUTE
CM032587.1	48568	.	C	t	100	*	TYPE=SUBSTITUTE
CM032587.1	48568	.	C	t	100	*	TYPE=SUBSTITUTE
CM032587.1	48572	.	G	t	100	*	TYPE=SUBSTITUTE
CM032587.1	48572	.	G	a	100	*	TYPE=SUBSTITUTE
CM032587.1	48572	.	G	t	100	*	TYPE=SUBSTITUTE
CM032587.1	48572	.	G	a	100	*	TYPE=SUBSTITUTE
CM032587.1	48574	.	T	a	100	*	TYPE=SUBSTITUTE
CM032587.1	48574	.	T	a	100	*	TYPE=SUBSTITUTE
CM032587.1	48581	.	G	a	100	*	TYPE=SUBSTITUTE
CM032587.1	48582	.	C	t	100	*	TYPE=SUBSTITUTE
CM032587.1	48582	.	C	t	100	*	TYPE=SUBSTITUTE
CM032587.1	48582	.	C	t	100	*	TYPE=SUBSTITUTE

CM032587.1	48582	.	C	t	100	*	TYPE=SUBSTITUTE
CM032587.1	48584	.	A	g	100	*	TYPE=SUBSTITUTE
CM032587.1	48584	.	A	g	100	*	TYPE=SUBSTITUTE
CM032587.1	48585	.	C	t	100	*	TYPE=SUBSTITUTE
CM032587.1	48585	.	C	t	100	*	TYPE=SUBSTITUTE
CM032587.1	48585	.	C	t	100	*	TYPE=SUBSTITUTE
CM032587.1	48586	.	G	t	100	*	TYPE=SUBSTITUTE
CM032587.1	48586	.	G	t	100	*	TYPE=SUBSTITUTE
CM032587.1	48586	.	G	a	100	*	TYPE=SUBSTITUTE
CM032587.1	48586	.	G	a	100	*	TYPE=SUBSTITUTE
CM032587.1	48587	.	C	t	100	*	TYPE=SUBSTITUTE
CM032587.1	48587	.	C	t	100	*	TYPE=SUBSTITUTE
CM032587.1	48587	.	C	t	100	*	TYPE=SUBSTITUTE
CM032587.1	48589	.	T	a	100	*	TYPE=SUBSTITUTE
CM032587.1	48589	.	T	a	100	*	TYPE=SUBSTITUTE
CM032587.1	48591	.	C	t	100	*	TYPE=SUBSTITUTE
CM032587.1	48591	.	C	t	100	*	TYPE=SUBSTITUTE
CM032587.1	48591	.	C	t	100	*	TYPE=SUBSTITUTE
CM032587.1	48591	.	C	t	100	*	TYPE=SUBSTITUTE
CM032587.1	48596	.	C	t	100	*	TYPE=SUBSTITUTE
CM032587.1	48596	.	C	t	100	*	TYPE=SUBSTITUTE
CM032587.1	48599	.	C	t	100	*	TYPE=SUBSTITUTE
CM032587.1	48603	.	G	A	100	*	TYPE=SUBSTITUTE
CM032587.1	48603	.	G	a	100	*	TYPE=SUBSTITUTE
CM032587.1	48603	.	G	a	100	*	TYPE=SUBSTITUTE
CM032587.1	48607	.	T	c	100	*	TYPE=SUBSTITUTE
CM032587.1	48607	.	T	c	100	*	TYPE=SUBSTITUTE
CM032587.1	48612	.	A	g	100	*	TYPE=SUBSTITUTE
CM032587.1	48621	.	G	a	100	*	TYPE=SUBSTITUTE
CM032587.1	48621	.	GCC	G	100	*	TYPE=DELETE
CM032587.1	48623	.	C	t	100	*	TYPE=SUBSTITUTE
CM032587.1	48625	.	C	t	100	*	TYPE=SUBSTITUTE
CM032587.1	48625	.	C	t	100	*	TYPE=SUBSTITUTE
CM032587.1	48625	.	C	t	100	*	TYPE=SUBSTITUTE
CM032587.1	48625	.	C	t	100	*	TYPE=SUBSTITUTE
CM032587.1	48626	.	C	t	100	*	TYPE=SUBSTITUTE
CM032587.1	48626	.	C	t	100	*	TYPE=SUBSTITUTE
CM032587.1	48626	.	C	t	100	*	TYPE=SUBSTITUTE
CM032587.1	48627	.	T	c	100	*	TYPE=SUBSTITUTE
CM032587.1	48627	.	T	c	100	*	TYPE=SUBSTITUTE
CM032587.1	48632	.	G	a	100	*	TYPE=SUBSTITUTE
CM032587.1	48632	.	G	a	100	*	TYPE=SUBSTITUTE
CM032587.1	48632	.	G	a	100	*	TYPE=SUBSTITUTE
CM032587.1	48632	.	G	a	100	*	TYPE=SUBSTITUTE
CM032587.1	48633	.	G	a	100	*	TYPE=SUBSTITUTE
CM032587.1	48633	.	G	a	100	*	TYPE=SUBSTITUTE
CM032587.1	48660	.	t	ta	100	*	TYPE=INSERT
CM032587.1	48660	.	t	tt	100	*	TYPE=INSERT
CM032587.1	48661	.	A	t	100	*	TYPE=SUBSTITUTE
CM032587.1	48661	.	A	t	100	*	TYPE=SUBSTITUTE
CM032587.1	48664	.	A	t	100	*	TYPE=SUBSTITUTE
CM032587.1	48664	.	A	t	100	*	TYPE=SUBSTITUTE
CM032587.1	48665	.	G	a	100	*	TYPE=SUBSTITUTE
CM032587.1	48665	.	G	a	100	*	TYPE=SUBSTITUTE
CM032587.1	48665	.	G	a	100	*	TYPE=SUBSTITUTE
CM032587.1	48665	.	G	a	100	*	TYPE=SUBSTITUTE
CM032587.1	48668	.	G	a	100	*	TYPE=SUBSTITUTE
CM032587.1	48682	.	A	t	100	*	TYPE=SUBSTITUTE
CM032587.1	48682	.	A	c	100	*	TYPE=SUBSTITUTE
CM032587.1	48688	.	T	c	100	*	TYPE=SUBSTITUTE
CM032587.1	48696	.	T	c	100	*	TYPE=SUBSTITUTE
CM032587.1	48696	.	T	c	100	*	TYPE=SUBSTITUTE
CM032587.1	48699	.	T	c	100	*	TYPE=SUBSTITUTE
CM032587.1	48701	.	AC	A	100	*	TYPE=DELETE

CM032587.1	48702	.	C	t	100	*	TYPE=SUBSTITUTE
CM032587.1	48702	.	C	t	100	*	TYPE=SUBSTITUTE
CM032587.1	48702	.	C	t	100	*	TYPE=SUBSTITUTE
CM032587.1	48704	.	A	t	100	*	TYPE=SUBSTITUTE
CM032587.1	48706	.	A	g	100	*	TYPE=SUBSTITUTE
CM032587.1	48707	.	A	g	100	*	TYPE=SUBSTITUTE
CM032587.1	48707	.	A	g	100	*	TYPE=SUBSTITUTE
CM032587.1	48711	.	A	g	100	*	TYPE=SUBSTITUTE
CM032587.1	48711	.	a	ag	100	*	TYPE=INSERT
CM032587.1	48711	.	A	g	100	*	TYPE=SUBSTITUTE
CM032587.1	48712	.	C	t	100	*	TYPE=SUBSTITUTE
CM032587.1	48712	.	C	t	100	*	TYPE=SUBSTITUTE
CM032587.1	48712	.	C	t	100	*	TYPE=SUBSTITUTE
CM032587.1	48715	.	A	g	100	*	TYPE=SUBSTITUTE
CM032587.1	48718	.	G	a	100	*	TYPE=SUBSTITUTE
CM032587.1	48718	.	G	a	100	*	TYPE=SUBSTITUTE
CM032587.1	48718	.	G	a	100	*	TYPE=SUBSTITUTE
CM032587.1	48718	.	G	a	100	*	TYPE=SUBSTITUTE
CM032587.1	48719	.	G	a	100	*	TYPE=SUBSTITUTE
CM032587.1	48727	.	C	t	100	*	TYPE=SUBSTITUTE
CM032587.1	48727	.	C	t	100	*	TYPE=SUBSTITUTE
CM032587.1	48727	.	C	t	100	*	TYPE=SUBSTITUTE
CM032587.1	48727	.	C	t	100	*	TYPE=SUBSTITUTE
CM032587.1	48728	.	G	a	100	*	TYPE=SUBSTITUTE
CM032587.1	48728	.	G	a	100	*	TYPE=SUBSTITUTE
CM032587.1	48728	.	G	a	100	*	TYPE=SUBSTITUTE
CM032587.1	48728	.	G	a	100	*	TYPE=SUBSTITUTE
CM032587.1	48729	.	C	t	100	*	TYPE=SUBSTITUTE
CM032587.1	48729	.	C	t	100	*	TYPE=SUBSTITUTE
CM032587.1	48729	.	C	t	100	*	TYPE=SUBSTITUTE
CM032587.1	48732	.	a	aat	100	*	TYPE=INSERT
CM032587.1	48733	.	G	a	100	*	TYPE=SUBSTITUTE
CM032587.1	48734	.	C	a	100	*	TYPE=SUBSTITUTE
CM032587.1	48734	.	C	t	100	*	TYPE=SUBSTITUTE
CM032587.1	48734	.	C	t	100	*	TYPE=SUBSTITUTE
CM032587.1	48734	.	C	t	100	*	TYPE=SUBSTITUTE
CM032587.1	48736	.	G	a	100	*	TYPE=SUBSTITUTE
CM032587.1	48736	.	G	a	100	*	TYPE=SUBSTITUTE
CM032587.1	48736	.	G	a	100	*	TYPE=SUBSTITUTE
CM032587.1	48736	.	G	a	100	*	TYPE=SUBSTITUTE
CM032587.1	48745	.	A	t	100	*	TYPE=SUBSTITUTE
CM032587.1	48745	.	A	t	100	*	TYPE=SUBSTITUTE
CM032587.1	48746	.	A	g	100	*	TYPE=SUBSTITUTE
CM032587.1	48752	.	G	a	100	*	TYPE=SUBSTITUTE
CM032587.1	48752	.	G	a	100	*	TYPE=SUBSTITUTE
CM032587.1	48760	.	TA	T	100	*	TYPE=DELETE
CM032587.1	48760	.	TA	T	100	*	TYPE=DELETE
CM032587.1	48760	.	TA	T	100	*	TYPE=DELETE
CM032587.1	48760	.	TA	T	100	*	TYPE=DELETE
CM032587.1	48780	.	A	g	100	*	TYPE=SUBSTITUTE
CM032587.1	48781	.	C	t	100	*	TYPE=SUBSTITUTE
CM032587.1	48781	.	C	t	100	*	TYPE=SUBSTITUTE
CM032587.1	48781	.	C	t	100	*	TYPE=SUBSTITUTE
CM032587.1	48785	.	T	c	100	*	TYPE=SUBSTITUTE
CM032587.1	48809	.	T	a	100	*	TYPE=SUBSTITUTE
CM032587.1	48822	.	T	c	100	*	TYPE=SUBSTITUTE
CM032587.1	48825	.	T	c	100	*	TYPE=SUBSTITUTE
CM032587.1	48825	.	T	c	100	*	TYPE=SUBSTITUTE
CM032587.1	48836	.	A	g	100	*	TYPE=SUBSTITUTE
CM032587.1	48837	.	A	g	100	*	TYPE=SUBSTITUTE
CM032587.1	48837	.	A	g	100	*	TYPE=SUBSTITUTE
CM032587.1	48841	.	TCC	T	100	*	TYPE=DELETE
CM032587.1	48842	.	CCT	C	100	*	TYPE=DELETE
CM032587.1	48842	.	C	t	100	*	TYPE=SUBSTITUTE

CM032587.1	48842	.	CC	C	100	*	TYPE=DELETE
CM032587.1	48848	.	T	a	100	*	TYPE=SUBSTITUTE
CM032587.1	48849	.	A	t	100	*	TYPE=SUBSTITUTE
CM032587.1	48849	.	A	t	100	*	TYPE=SUBSTITUTE
CM032587.1	48849	.	A	t	100	*	TYPE=SUBSTITUTE
CM032587.1	48851	.	A	t	100	*	TYPE=SUBSTITUTE
CM032587.1	48852	.	T	a	100	*	TYPE=SUBSTITUTE
CM032587.1	48852	.	T	a	100	*	TYPE=SUBSTITUTE
CM032587.1	48855	.	A	t	100	*	TYPE=SUBSTITUTE
CM032587.1	48857	.	A	g	100	*	TYPE=SUBSTITUTE
CM032587.1	48857	.	A	g	100	*	TYPE=SUBSTITUTE
CM032587.1	48858	.	T	g	100	*	TYPE=SUBSTITUTE
CM032587.1	48861	.	G	a	100	*	TYPE=SUBSTITUTE
CM032587.1	48861	.	G	a	100	*	TYPE=SUBSTITUTE
CM032587.1	48861	.	G	a	100	*	TYPE=SUBSTITUTE
CM032587.1	48861	.	G	a	100	*	TYPE=SUBSTITUTE
CM032587.1	48864	.	G	a	100	*	TYPE=SUBSTITUTE
CM032587.1	48864	.	G	a	100	*	TYPE=SUBSTITUTE
CM032587.1	48865	.	T	c	100	*	TYPE=SUBSTITUTE
CM032587.1	48865	.	T	c	100	*	TYPE=SUBSTITUTE
CM032587.1	48865	.	T	c	100	*	TYPE=SUBSTITUTE
CM032587.1	48868	.	T	c	100	*	TYPE=SUBSTITUTE
CM032587.1	48879	.	C	t	100	*	TYPE=SUBSTITUTE
CM032587.1	48879	.	C	t	100	*	TYPE=SUBSTITUTE
CM032587.1	48879	.	C	t	100	*	TYPE=SUBSTITUTE
CM032587.1	48879	.	C	t	100	*	TYPE=SUBSTITUTE
CM032587.1	48884	.	G	a	100	*	TYPE=SUBSTITUTE
CM032587.1	48884	.	G	a	100	*	TYPE=SUBSTITUTE
CM032587.1	48899	.	G	a	100	*	TYPE=SUBSTITUTE
CM032587.1	48899	.	G	a	100	*	TYPE=SUBSTITUTE
CM032587.1	48899	.	G	a	100	*	TYPE=SUBSTITUTE
CM032587.1	48901	.	T	c	100	*	TYPE=SUBSTITUTE
CM032587.1	48901	.	T	c	100	*	TYPE=SUBSTITUTE
CM032587.1	48909	.	A	g	100	*	TYPE=SUBSTITUTE
CM032587.1	48909	.	A	g	100	*	TYPE=SUBSTITUTE
CM032587.1	48909	.	A	g	100	*	TYPE=SUBSTITUTE
CM032587.1	48923	.	AAT	A	100	*	TYPE=DELETE
CM032587.1	48923	.	AAT	A	100	*	TYPE=DELETE
CM032587.1	48923	.	AATT	A	100	*	TYPE=DELETE
CM032587.1	48927	.	AT	A	100	*	TYPE=DELETE
CM032587.1	48927	.	ATTA	A	100	*	TYPE=DELETE
CM032587.1	48927	.	AT	A	100	*	TYPE=DELETE
CM032587.1	48931	.	T	a	100	*	TYPE=SUBSTITUTE
CM032587.1	48931	.	T	c	100	*	TYPE=SUBSTITUTE
CM032587.1	48931	.	T	c	100	*	TYPE=SUBSTITUTE
CM032587.1	48931	.	T	a	100	*	TYPE=SUBSTITUTE
CM032587.1	48934	.	T	c	100	*	TYPE=SUBSTITUTE
CM032587.1	48934	.	T	c	100	*	TYPE=SUBSTITUTE
CM032587.1	48937	.	T	c	100	*	TYPE=SUBSTITUTE
CM032587.1	48937	.	T	c	100	*	TYPE=SUBSTITUTE
CM032587.1	48940	.	T	c	100	*	TYPE=SUBSTITUTE
CM032587.1	48941	.	T	a	100	*	TYPE=SUBSTITUTE
CM032587.1	48941	.	T	a	100	*	TYPE=SUBSTITUTE
CM032587.1	48945	.	T	c	100	*	TYPE=SUBSTITUTE
CM032587.1	48945	.	T	c	100	*	TYPE=SUBSTITUTE
CM032587.1	48945	.	T	c	100	*	TYPE=SUBSTITUTE
CM032587.1	48949	.	A	g	100	*	TYPE=SUBSTITUTE
CM032587.1	48949	.	A	g	100	*	TYPE=SUBSTITUTE
CM032587.1	48955	.	C	t	100	*	TYPE=SUBSTITUTE
CM032587.1	48955	.	C	t	100	*	TYPE=SUBSTITUTE
CM032587.1	48965	.	G	a	100	*	TYPE=SUBSTITUTE
CM032587.1	48966	.	C	t	100	*	TYPE=SUBSTITUTE
CM032587.1	48966	.	C	t	100	*	TYPE=SUBSTITUTE
CM032587.1	48970	.	G	a	100	*	TYPE=SUBSTITUTE

CM032587.1	48975	.	A	g	100	*	TYPE=SUBSTITUTE
CM032587.1	48975	.	A	g	100	*	TYPE=SUBSTITUTE
CM032587.1	48975	.	A	g	100	*	TYPE=SUBSTITUTE
CM032587.1	48989	.	G	a	100	*	TYPE=SUBSTITUTE
CM032587.1	48989	.	G	a	100	*	TYPE=SUBSTITUTE
CM032587.1	48989	.	G	a	100	*	TYPE=SUBSTITUTE
CM032587.1	48989	.	G	a	100	*	TYPE=SUBSTITUTE
CM032587.1	48990	.	C	t	100	*	TYPE=SUBSTITUTE
CM032587.1	48990	.	C	t	100	*	TYPE=SUBSTITUTE
CM032587.1	48994	.	G	a	100	*	TYPE=SUBSTITUTE
CM032587.1	48994	.	G	a	100	*	TYPE=SUBSTITUTE
CM032587.1	48994	.	G	a	100	*	TYPE=SUBSTITUTE
CM032587.1	48995	.	G	a	100	*	TYPE=SUBSTITUTE
CM032587.1	48997	.	T	c	100	*	TYPE=SUBSTITUTE
CM032587.1	48997	.	T	c	100	*	TYPE=SUBSTITUTE
CM032587.1	49007	.	G	a	100	*	TYPE=SUBSTITUTE
CM032587.1	49007	.	G	a	100	*	TYPE=SUBSTITUTE
CM032587.1	49009	.	a	aatt	100	*	TYPE=INSERT
CM032587.1	49018	.	t	tta	100	*	TYPE=INSERT
CM032587.1	49018	.	t	tta	100	*	TYPE=INSERT
CM032587.1	49021	.	G	a	100	*	TYPE=SUBSTITUTE
CM032587.1	49021	.	G	a	100	*	TYPE=SUBSTITUTE
CM032587.1	49021	.	G	a	100	*	TYPE=SUBSTITUTE
CM032587.1	49021	.	G	a	100	*	TYPE=SUBSTITUTE
CM032587.1	49022	.	G	a	100	*	TYPE=SUBSTITUTE
CM032587.1	49022	.	G	a	100	*	TYPE=SUBSTITUTE
CM032587.1	49022	.	G	a	100	*	TYPE=SUBSTITUTE
CM032587.1	49023	.	G	a	100	*	TYPE=SUBSTITUTE
CM032587.1	49023	.	G	t	100	*	TYPE=SUBSTITUTE
CM032587.1	49023	.	G	t	100	*	TYPE=SUBSTITUTE
CM032587.1	49024	.	T	a	100	*	TYPE=SUBSTITUTE
CM032587.1	49024	.	T	a	100	*	TYPE=SUBSTITUTE
CM032587.1	49028	.	G	a	100	*	TYPE=SUBSTITUTE
CM032587.1	49028	.	G	a	100	*	TYPE=SUBSTITUTE
CM032587.1	49028	.	G	a	100	*	TYPE=SUBSTITUTE
CM032587.1	49028	.	G	a	100	*	TYPE=SUBSTITUTE
CM032587.1	49029	.	G	a	100	*	TYPE=SUBSTITUTE
CM032587.1	49029	.	G	a	100	*	TYPE=SUBSTITUTE
CM032587.1	49030	.	G	a	100	*	TYPE=SUBSTITUTE
CM032587.1	49030	.	G	a	100	*	TYPE=SUBSTITUTE
CM032587.1	49031	.	G	a	100	*	TYPE=SUBSTITUTE
CM032587.1	49031	.	G	a	100	*	TYPE=SUBSTITUTE
CM032587.1	49031	.	G	a	100	*	TYPE=SUBSTITUTE
CM032587.1	49031	.	G	a	100	*	TYPE=SUBSTITUTE
CM032587.1	49033	.	G	a	100	*	TYPE=SUBSTITUTE
CM032587.1	49033	.	G	a	100	*	TYPE=SUBSTITUTE
CM032587.1	49033	.	G	a	100	*	TYPE=SUBSTITUTE
CM032587.1	49034	.	G	a	100	*	TYPE=SUBSTITUTE
CM032587.1	49034	.	G	a	100	*	TYPE=SUBSTITUTE
CM032587.1	49034	.	G	a	100	*	TYPE=SUBSTITUTE
CM032587.1	49034	.	G	a	100	*	TYPE=SUBSTITUTE
CM032587.1	49036	.	T	c	100	*	TYPE=SUBSTITUTE
CM032587.1	49041	.	AC	A	100	*	TYPE=DELETE
CM032587.1	49042	.	C	t	100	*	TYPE=SUBSTITUTE
CM032587.1	49042	.	C	t	100	*	TYPE=SUBSTITUTE
CM032587.1	49042	.	C	t	100	*	TYPE=SUBSTITUTE
CM032587.1	49058	.	G	a	100	*	TYPE=SUBSTITUTE
CM032587.1	49058	.	G	a	100	*	TYPE=SUBSTITUTE
CM032587.1	49058	.	G	a	100	*	TYPE=SUBSTITUTE
CM032587.1	49059	.	C	t	100	*	TYPE=SUBSTITUTE
CM032587.1	49059	.	C	t	100	*	TYPE=SUBSTITUTE
CM032587.1	49060	.	T	c	100	*	TYPE=SUBSTITUTE
CM032587.1	49060	.	t	ta	100	*	TYPE=INSERT
CM032587.1	49061	.	T	a	100	*	TYPE=SUBSTITUTE

CM032587.1	49061	.	T	a	100	*	TYPE=SUBSTITUTE
CM032587.1	49063	.	AG	A	100	*	TYPE=DELETE
CM032587.1	49063	.	AG	A	100	*	TYPE=DELETE
CM032587.1	49065	.	G	a	100	*	TYPE=SUBSTITUTE
CM032587.1	49065	.	G	a	100	*	TYPE=SUBSTITUTE
CM032587.1	49066	.	G	a	100	*	TYPE=SUBSTITUTE
CM032587.1	49066	.	G	a	100	*	TYPE=SUBSTITUTE
CM032587.1	49066	.	G	a	100	*	TYPE=SUBSTITUTE
CM032587.1	49067	.	G	A	100	*	TYPE=SUBSTITUTE
CM032587.1	49072	.	C	t	100	*	TYPE=SUBSTITUTE
CM032587.1	49073	.	C	t	100	*	TYPE=SUBSTITUTE
CM032587.1	49073	.	C	t	100	*	TYPE=SUBSTITUTE
CM032587.1	49075	.	TC	T	100	*	TYPE=DELETE
CM032587.1	49076	.	C	t	100	*	TYPE=SUBSTITUTE
CM032587.1	49076	.	C	t	100	*	TYPE=SUBSTITUTE
CM032587.1	49076	.	C	t	100	*	TYPE=SUBSTITUTE
CM032587.1	49080	.	A	t	100	*	TYPE=SUBSTITUTE
CM032587.1	49080	.	A	t	100	*	TYPE=SUBSTITUTE
CM032587.1	49082	.	AG	A	100	*	TYPE=DELETE
CM032587.1	49083	.	G	a	100	*	TYPE=SUBSTITUTE
CM032587.1	49083	.	G	a	100	*	TYPE=SUBSTITUTE
CM032587.1	49083	.	G	a	100	*	TYPE=SUBSTITUTE
CM032587.1	49084	.	G	a	100	*	TYPE=SUBSTITUTE
CM032587.1	49085	.	T	c	100	*	TYPE=SUBSTITUTE
CM032587.1	49085	.	T	c	100	*	TYPE=SUBSTITUTE
CM032587.1	49086	.	TA	T	100	*	TYPE=DELETE
CM032587.1	49088	.	A	g	100	*	TYPE=SUBSTITUTE
CM032587.1	49089	.	A	G	100	*	TYPE=SUBSTITUTE
CM032587.1	49089	.	A	g	100	*	TYPE=SUBSTITUTE
CM032587.1	49090	.	A	G	100	*	TYPE=SUBSTITUTE
CM032587.1	49090	.	A	G	100	*	TYPE=SUBSTITUTE
CM032587.1	49091	.	A	G	100	*	TYPE=SUBSTITUTE
CM032587.1	49091	.	A	G	100	*	TYPE=SUBSTITUTE
CM032587.1	49091	.	A	g	100	*	TYPE=SUBSTITUTE
CM032587.1	49093	.	C	T	100	*	TYPE=SUBSTITUTE
CM032587.1	49099	.	T	C	100	*	TYPE=SUBSTITUTE
CM032587.1	49100	.	T	A	100	*	TYPE=SUBSTITUTE
CM032587.1	49100	.	T	A	100	*	TYPE=SUBSTITUTE
CM032587.1	49102	.	G	a	100	*	TYPE=SUBSTITUTE
CM032587.1	49102	.	G	A	100	*	TYPE=SUBSTITUTE
CM032587.1	49102	.	G	A	100	*	TYPE=SUBSTITUTE
CM032587.1	49104	.	C	t	100	*	TYPE=SUBSTITUTE
CM032587.1	49108	.	G	a	100	*	TYPE=SUBSTITUTE
CM032587.1	49108	.	G	a	100	*	TYPE=SUBSTITUTE
CM032587.1	49116	.	G	a	100	*	TYPE=SUBSTITUTE
CM032587.1	49116	.	G	a	100	*	TYPE=SUBSTITUTE
CM032587.1	49117	.	G	a	100	*	TYPE=SUBSTITUTE
CM032587.1	49117	.	G	a	100	*	TYPE=SUBSTITUTE
CM032587.1	49117	.	G	a	100	*	TYPE=SUBSTITUTE
CM032587.1	49118	.	T	g	100	*	TYPE=SUBSTITUTE
CM032587.1	49118	.	T	g	100	*	TYPE=SUBSTITUTE
CM032587.1	49123	.	T	g	100	*	TYPE=SUBSTITUTE
CM032587.1	49123	.	T	g	100	*	TYPE=SUBSTITUTE
CM032587.1	49130	.	T	c	100	*	TYPE=SUBSTITUTE
CM032587.1	49133	.	G	a	100	*	TYPE=SUBSTITUTE
CM032587.1	49133	.	G	a	100	*	TYPE=SUBSTITUTE
CM032587.1	49133	.	G	a	100	*	TYPE=SUBSTITUTE
CM032587.1	49134	.	T	a	100	*	TYPE=SUBSTITUTE
CM032587.1	49134	.	T	a	100	*	TYPE=SUBSTITUTE
CM032587.1	49135	.	T	a	100	*	TYPE=SUBSTITUTE
CM032587.1	49135	.	T	a	100	*	TYPE=SUBSTITUTE
CM032587.1	49136	.	A	g	100	*	TYPE=SUBSTITUTE
CM032587.1	49136	.	A	g	100	*	TYPE=SUBSTITUTE
CM032587.1	49138	.	T	c	100	*	TYPE=SUBSTITUTE

CM032587.1	49139	.	T	C	100	*	TYPE=SUBSTITUTE
CM032587.1	49141	.	A	G	100	*	TYPE=SUBSTITUTE
CM032587.1	49143	.	G	a	100	*	TYPE=SUBSTITUTE
CM032587.1	49143	.	G	a	100	*	TYPE=SUBSTITUTE
CM032587.1	49144	.	G	a	100	*	TYPE=SUBSTITUTE
CM032587.1	49159	.	T	c	100	*	TYPE=SUBSTITUTE
CM032587.1	49165	.	T	c	100	*	TYPE=SUBSTITUTE
CM032587.1	49167	.	T	c	100	*	TYPE=SUBSTITUTE
CM032587.1	49167	.	TT	T	100	*	TYPE=DELETE
CM032587.1	49175	.	C	t	100	*	TYPE=SUBSTITUTE
CM032587.1	49175	.	C	t	100	*	TYPE=SUBSTITUTE
CM032587.1	49179	.	G	a	100	*	TYPE=SUBSTITUTE
CM032587.1	49179	.	G	a	100	*	TYPE=SUBSTITUTE
CM032587.1	49179	.	G	a	100	*	TYPE=SUBSTITUTE
CM032587.1	49183	.	A	g	100	*	TYPE=SUBSTITUTE
CM032587.1	49186	.	C	t	100	*	TYPE=SUBSTITUTE
CM032587.1	49188	.	G	a	100	*	TYPE=SUBSTITUTE
CM032587.1	49188	.	G	a	100	*	TYPE=SUBSTITUTE
CM032587.1	49193	.	T	a	100	*	TYPE=SUBSTITUTE
CM032587.1	49193	.	T	a	100	*	TYPE=SUBSTITUTE
CM032587.1	49194	.	A	g	100	*	TYPE=SUBSTITUTE
CM032587.1	49194	.	A	g	100	*	TYPE=SUBSTITUTE
CM032587.1	49195	.	A	t	100	*	TYPE=SUBSTITUTE
CM032587.1	49195	.	A	t	100	*	TYPE=SUBSTITUTE
CM032587.1	49204	.	T	a	100	*	TYPE=SUBSTITUTE
CM032587.1	49204	.	T	a	100	*	TYPE=SUBSTITUTE
CM032587.1	49222	.	G	a	100	*	TYPE=SUBSTITUTE
CM032587.1	49223	.	C	t	100	*	TYPE=SUBSTITUTE
CM032587.1	49223	.	C	t	100	*	TYPE=SUBSTITUTE
CM032587.1	49226	.	G	a	100	*	TYPE=SUBSTITUTE
CM032587.1	49232	.	t	ta	100	*	TYPE=INSERT
CM032587.1	49233	.	A	AA	100	*	TYPE=INSERT
CM032587.1	49234	.	G	A	100	*	TYPE=SUBSTITUTE
CM032587.1	49234	.	G	a	100	*	TYPE=SUBSTITUTE
CM032587.1	49234	.	G	a	100	*	TYPE=SUBSTITUTE
CM032587.1	49236	.	T	A	100	*	TYPE=SUBSTITUTE
CM032587.1	49236	.	T	a	100	*	TYPE=SUBSTITUTE
CM032587.1	49237	.	A	g	100	*	TYPE=SUBSTITUTE
CM032587.1	49237	.	A	G	100	*	TYPE=SUBSTITUTE
CM032587.1	49239	.	A	G	100	*	TYPE=SUBSTITUTE
CM032587.1	49239	.	A	G	100	*	TYPE=SUBSTITUTE
CM032587.1	49239	.	A	G	100	*	TYPE=SUBSTITUTE
CM032587.1	49240	.	A	G	100	*	TYPE=SUBSTITUTE
CM032587.1	49240	.	A	G	100	*	TYPE=SUBSTITUTE
CM032587.1	49248	.	G	A	100	*	TYPE=SUBSTITUTE
CM032587.1	49248	.	G	A	100	*	TYPE=SUBSTITUTE
CM032587.1	49250	.	G	A	100	*	TYPE=SUBSTITUTE
CM032587.1	49250	.	G	A	100	*	TYPE=SUBSTITUTE
CM032587.1	49250	.	G	A	100	*	TYPE=SUBSTITUTE
CM032587.1	49255	.	G	A	100	*	TYPE=SUBSTITUTE
CM032587.1	49255	.	G	A	100	*	TYPE=SUBSTITUTE
CM032587.1	49256	.	G	A	100	*	TYPE=SUBSTITUTE
CM032587.1	49260	.	C	T	100	*	TYPE=SUBSTITUTE
CM032587.1	49269	.	A	AG	100	*	TYPE=INSERT
CM032587.1	475892	.	G	A	100	*	TYPE=SUBSTITUTE
CM032587.1	475909	.	C	T	100	*	TYPE=SUBSTITUTE
CM032587.1	475914	.	A	G	100	*	TYPE=SUBSTITUTE
CM032587.1	475915	.	T	G	100	*	TYPE=SUBSTITUTE
CM032587.1	475919	.	C	T	100	*	TYPE=SUBSTITUTE
CM032587.1	475949	.	T	G	100	*	TYPE=SUBSTITUTE
CM032587.1	476011	.	A	G	100	*	TYPE=SUBSTITUTE
CM032587.1	476015	.	A	T	100	*	TYPE=SUBSTITUTE
CM032587.1	476016	.	A	C	100	*	TYPE=SUBSTITUTE
CM032587.1	476017	.	T	G	100	*	TYPE=SUBSTITUTE

CM032587.1	476025	.	G	A	100	*	TYPE=SUBSTITUTE
CM032587.1	476027	.	C	T	100	*	TYPE=SUBSTITUTE
CM032587.1	476028	.	G	T	100	*	TYPE=SUBSTITUTE
CM032587.1	476047	.	G	A	100	*	TYPE=SUBSTITUTE
CM032587.1	476055	.	A	G	100	*	TYPE=SUBSTITUTE
CM032587.1	476058	.	G	C	100	*	TYPE=SUBSTITUTE
CM032587.1	476094	.	C	T	100	*	TYPE=SUBSTITUTE
CM032587.1	476105	.	C	T	100	*	TYPE=SUBSTITUTE
CM032587.1	476106	.	G	T	100	*	TYPE=SUBSTITUTE
CM032587.1	476112	.	C	T	100	*	TYPE=SUBSTITUTE
CM032587.1	476119	.	T	A	100	*	TYPE=SUBSTITUTE
CM032587.1	476120	.	A	G	100	*	TYPE=SUBSTITUTE
CM032587.1	476121	.	G	C	100	*	TYPE=SUBSTITUTE
CM032587.1	476124	.	T	C	100	*	TYPE=SUBSTITUTE
CM032587.1	476128	.	A	G	100	*	TYPE=SUBSTITUTE
CM032587.1	476129	.	T	A	100	*	TYPE=SUBSTITUTE
CM032587.1	476130	.	G	A	100	*	TYPE=SUBSTITUTE
CM032587.1	476132	.	T	C	100	*	TYPE=SUBSTITUTE
CM032587.1	476134	.	G	T	100	*	TYPE=SUBSTITUTE
CM032587.1	476136	.	A	C	100	*	TYPE=SUBSTITUTE
CM032587.1	476138	.	T	A	100	*	TYPE=SUBSTITUTE
CM032587.1	476139	.	G	A	100	*	TYPE=SUBSTITUTE
CM032587.1	476146	.	G	A	100	*	TYPE=SUBSTITUTE
CM032587.1	476159	.	G	T	100	*	TYPE=SUBSTITUTE
CM032587.1	476161	.	T	C	100	*	TYPE=SUBSTITUTE
CM032587.1	476170	.	A	G	100	*	TYPE=SUBSTITUTE
CM032587.1	476184	.	T	C	100	*	TYPE=SUBSTITUTE
CM032587.1	476187	.	C	T	100	*	TYPE=SUBSTITUTE
CM032587.1	476215	.	G	A	100	*	TYPE=SUBSTITUTE
CM032587.1	476216	.	G	A	100	*	TYPE=SUBSTITUTE
CM032587.1	476217	.	G	C	100	*	TYPE=SUBSTITUTE
CM032587.1	476227	.	A	G	100	*	TYPE=SUBSTITUTE
CM032587.1	476235	.	T	C	100	*	TYPE=SUBSTITUTE
CM032587.1	476257	.	A	C	100	*	TYPE=SUBSTITUTE
CM032587.1	476259	.	G	T	100	*	TYPE=SUBSTITUTE
CM032587.1	476260	.	C	T	100	*	TYPE=SUBSTITUTE
CM032587.1	476261	.	C	T	100	*	TYPE=SUBSTITUTE
CM032587.1	476263	.	T	C	100	*	TYPE=SUBSTITUTE
CM032587.1	476264	.	G	T	100	*	TYPE=SUBSTITUTE
CM032587.1	476265	.	G	T	100	*	TYPE=SUBSTITUTE
CM032587.1	476266	.	A	G	100	*	TYPE=SUBSTITUTE
CM032587.1	476268	.	C	A	100	*	TYPE=SUBSTITUTE
CM032587.1	476269	.	G	A	100	*	TYPE=SUBSTITUTE
CM032587.1	476271	.	A	G	100	*	TYPE=SUBSTITUTE
CM032587.1	476278	.	C	G	100	*	TYPE=SUBSTITUTE
CM032587.1	476283	.	A	T	100	*	TYPE=SUBSTITUTE
CM032587.1	476297	.	T	C	100	*	TYPE=SUBSTITUTE
CM032587.1	476298	.	C	G	100	*	TYPE=SUBSTITUTE
CM032587.1	476300	.	G	A	100	*	TYPE=SUBSTITUTE
CM032587.1	476301	.	A	T	100	*	TYPE=SUBSTITUTE
CM032587.1	476303	.	C	T	100	*	TYPE=SUBSTITUTE
CM032587.1	476305	.	C	A	100	*	TYPE=SUBSTITUTE
CM032587.1	476306	.	A	T	100	*	TYPE=SUBSTITUTE
CM032587.1	476307	.	G	C	100	*	TYPE=SUBSTITUTE
CM032587.1	476309	.	T	TTCA	100	*	TYPE=INSERT
CM032587.1	476313	.	C	CATGGTA	100	*	TYPE=INSERT
CM032587.1	476319	.	C	T	100	*	TYPE=SUBSTITUTE
CM032587.1	476320	.	A	G	100	*	TYPE=SUBSTITUTE
CM032587.1	476326	.	C	A	100	*	TYPE=SUBSTITUTE
CM032587.1	476329	.	G	A	100	*	TYPE=SUBSTITUTE
CM032587.1	476330	.	G	A	100	*	TYPE=SUBSTITUTE
CM032587.1	476339	.	T	G	100	*	TYPE=SUBSTITUTE
CM032587.1	476341	.	T	G	100	*	TYPE=SUBSTITUTE
CM032587.1	476342	.	T	C	100	*	TYPE=SUBSTITUTE

CM032587.1	476353	.	T	C	100	*	TYPE=SUBSTITUTE
CM032587.1	476356	.	G	A	100	*	TYPE=SUBSTITUTE
CM032587.1	476360	.	C	T	100	*	TYPE=SUBSTITUTE
CM032587.1	476361	.	T	C	100	*	TYPE=SUBSTITUTE
CM032587.1	476362	.	T	A	100	*	TYPE=SUBSTITUTE
CM032587.1	476364	.	G	T	100	*	TYPE=SUBSTITUTE
CM032587.1	476365	.	A	G	100	*	TYPE=SUBSTITUTE
CM032587.1	476380	.	C	T	100	*	TYPE=SUBSTITUTE
CM032587.1	476383	.	A	G	100	*	TYPE=SUBSTITUTE
CM032587.1	476390	.	C	T	100	*	TYPE=SUBSTITUTE
CM032587.1	476392	.	T	C	100	*	TYPE=SUBSTITUTE
CM032587.1	476396	.	A	C	100	*	TYPE=SUBSTITUTE
CM032587.1	476398	.	A	T	100	*	TYPE=SUBSTITUTE
CM032587.1	476404	.	T	A	100	*	TYPE=SUBSTITUTE
CM032587.1	476406	.	C	T	100	*	TYPE=SUBSTITUTE
CM032587.1	476421	.	C	T	100	*	TYPE=SUBSTITUTE
CM032587.1	476439	.	T	C	100	*	TYPE=SUBSTITUTE
CM032587.1	476442	.	C	A	100	*	TYPE=SUBSTITUTE
CM032587.1	476446	.	C	T	100	*	TYPE=SUBSTITUTE
CM032587.1	476448	.	T	C	100	*	TYPE=SUBSTITUTE
CM032587.1	476451	.	A	T	100	*	TYPE=SUBSTITUTE
CM032587.1	476461	.	A	T	100	*	TYPE=SUBSTITUTE
CM032587.1	476463	.	A	G	100	*	TYPE=SUBSTITUTE
CM032587.1	476466	.	G	C	100	*	TYPE=SUBSTITUTE
CM032587.1	476476	.	A	G	100	*	TYPE=SUBSTITUTE
CM032587.1	476499	.	A	G	100	*	TYPE=SUBSTITUTE
CM032587.1	476510	.	A	G	100	*	TYPE=SUBSTITUTE
CM032587.1	476521	.	G	a	100	*	TYPE=SUBSTITUTE
CM032587.1	476527	.	G	a	100	*	TYPE=SUBSTITUTE
CM032587.1	929977	.	G	A	100	*	TYPE=SUBSTITUTE
CM032587.1	929997	.	A	G	100	*	TYPE=SUBSTITUTE
CM032587.1	929998	.	C	T	100	*	TYPE=SUBSTITUTE
CM032587.1	929999	.	C	T	100	*	TYPE=SUBSTITUTE
CM032587.1	930000	.	A	C	100	*	TYPE=SUBSTITUTE
CM032587.1	930001	.	A	C	100	*	TYPE=SUBSTITUTE
CM032587.1	930002	.	C	T	100	*	TYPE=SUBSTITUTE
CM032587.1	930008	.	AC	A	100	*	TYPE=DELETE
CM032587.1	930010	.	T	A	100	*	TYPE=SUBSTITUTE
CM032587.1	930139	.	G	T	100	*	TYPE=SUBSTITUTE
CM032587.1	930238	.	A	G	100	*	TYPE=SUBSTITUTE
CM032587.1	930256	.	C	T	100	*	TYPE=SUBSTITUTE
CM032587.1	930311	.	A	G	100	*	TYPE=SUBSTITUTE
CM032587.1	930322	.	AC	A	100	*	TYPE=DELETE
CM032587.1	930331	.	T	TC	100	*	TYPE=INSERT
CM032587.1	930352	.	C	T	100	*	TYPE=SUBSTITUTE
CM032587.1	930363	.	T	C	100	*	TYPE=SUBSTITUTE
CM032587.1	930368	.	TA	T	100	*	TYPE=DELETE
CM032587.1	930536	.	G	A	100	*	TYPE=SUBSTITUTE
CM032587.1	930539	.	T	C	100	*	TYPE=SUBSTITUTE
CM032587.1	930551	.	T	A	100	*	TYPE=SUBSTITUTE
CM032587.1	930645	.	A	G	100	*	TYPE=SUBSTITUTE
CM032587.1	930650	.	C	T	100	*	TYPE=SUBSTITUTE
CM032587.1	930651	.	A	G	100	*	TYPE=SUBSTITUTE
CM032587.1	930688	.	T	A	100	*	TYPE=SUBSTITUTE
CM032587.1	930689	.	G	A	100	*	TYPE=SUBSTITUTE
CM032587.1	930692	.	A	C	100	*	TYPE=SUBSTITUTE
CM032587.1	930693	.	T	C	100	*	TYPE=SUBSTITUTE
CM032587.1	930700	.	C	T	100	*	TYPE=SUBSTITUTE
CM032587.1	930708	.	A	T	100	*	TYPE=SUBSTITUTE
CM032587.1	930729	.	AAAG	A	100	*	TYPE=DELETE
CM032587.1	930734	.	A	G	100	*	TYPE=SUBSTITUTE
CM032587.1	930741	.	G	T	100	*	TYPE=SUBSTITUTE
CM032587.1	930765	.	T	G	100	*	TYPE=SUBSTITUTE
CM032587.1	930766	.	C	T	100	*	TYPE=SUBSTITUTE

CM032587.1	930771	.	G	A	100	*	TYPE=SUBSTITUTE
CM032587.1	930775	.	A	C	100	*	TYPE=SUBSTITUTE
CM032587.1	930801	.	C	T	100	*	TYPE=SUBSTITUTE
CM032587.1	930812	.	C	T	100	*	TYPE=SUBSTITUTE
CM032587.1	930887	.	A	c	100	*	TYPE=SUBSTITUTE
CM032587.1	930894	.	CATTA	C	100	*	TYPE=DELETE
CM032587.1	930902	.	A	g	100	*	TYPE=SUBSTITUTE
CM032587.1	930916	.	A	G	100	*	TYPE=SUBSTITUTE
CM032587.1	930929	.	G	A	100	*	TYPE=SUBSTITUTE
CM032587.1	930932	.	G	A	100	*	TYPE=SUBSTITUTE
CM032587.1	930941	.	AT	A	100	*	TYPE=DELETE
CM032587.1	930947	.	G	A	100	*	TYPE=SUBSTITUTE
CM032587.1	930962	.	G	A	100	*	TYPE=SUBSTITUTE
CM032587.1	930963	.	G	T	100	*	TYPE=SUBSTITUTE
CM032587.1	930966	.	A	G	100	*	TYPE=SUBSTITUTE
CM032587.1	930967	.	A	G	100	*	TYPE=SUBSTITUTE
CM032587.1	930979	.	A	G	100	*	TYPE=SUBSTITUTE
CM032587.1	930995	.	C	T	100	*	TYPE=SUBSTITUTE
CM032587.1	930998	.	C	A	100	*	TYPE=SUBSTITUTE
CM032587.1	931015	.	t	ttcttgt	100	*	TYPE=INSERT
CM032587.1	931019	.	T	A	100	*	TYPE=SUBSTITUTE
CM032587.1	931039	.	A	T	100	*	TYPE=SUBSTITUTE
CM032587.1	931055	.	TT	T	100	*	TYPE=DELETE
CM032587.1	931092	.	T	C	100	*	TYPE=SUBSTITUTE
CM032587.1	931174	.	T	G	100	*	TYPE=SUBSTITUTE
CM032587.1	931243	.	G	GG	100	*	TYPE=INSERT
CM032587.1	931245	.	C	A	100	*	TYPE=SUBSTITUTE
CM032587.1	931246	.	G	A	100	*	TYPE=SUBSTITUTE
CM032587.1	931264	.	A	T	100	*	TYPE=SUBSTITUTE
CM032587.1	931265	.	C	G	100	*	TYPE=SUBSTITUTE
CM032587.1	931267	.	A	T	100	*	TYPE=SUBSTITUTE
CM032587.1	931269	.	G	T	100	*	TYPE=SUBSTITUTE
CM032587.1	931278	.	G	C	100	*	TYPE=SUBSTITUTE
CM032587.1	931280	.	TG	T	100	*	TYPE=DELETE
CM032587.1	931283	.	C	T	100	*	TYPE=SUBSTITUTE
CM032587.1	931284	.	A	C	100	*	TYPE=SUBSTITUTE
CM032587.1	931375	.	T	TT	100	*	TYPE=INSERT
CM032587.1	931379	.	T	C	100	*	TYPE=SUBSTITUTE
CM032587.1	931400	.	A	C	100	*	TYPE=SUBSTITUTE
CM032587.1	931413	.	CG	C	100	*	TYPE=DELETE
CM032587.1	931441	.	A	G	100	*	TYPE=SUBSTITUTE
CM032587.1	931445	.	G	A	100	*	TYPE=SUBSTITUTE
CM032587.1	931475	.	C	CC	100	*	TYPE=INSERT
CM032587.1	931524	.	T	TC	100	*	TYPE=INSERT
CM032587.1	931581	.	C	T	100	*	TYPE=SUBSTITUTE
CM032587.1	931584	.	G	A	100	*	TYPE=SUBSTITUTE
CM032587.1	931585	.	C	A	100	*	TYPE=SUBSTITUTE
CM032587.1	931587	.	T	G	100	*	TYPE=SUBSTITUTE
CM032587.1	931588	.	T	G	100	*	TYPE=SUBSTITUTE
CM032587.1	931589	.	C	G	100	*	TYPE=SUBSTITUTE
CM032587.1	931640	.	T	C	100	*	TYPE=SUBSTITUTE
CM032587.1	931663	.	T	TGACGGCAA	100	*	TYPE=INSERT
CM032587.1	931877	.	A	T	100	*	TYPE=SUBSTITUTE
CM032587.1	931879	.	G	A	100	*	TYPE=SUBSTITUTE
CM032587.1	931925	.	T	C	100	*	TYPE=SUBSTITUTE
CM032587.1	931947	.	C	T	100	*	TYPE=SUBSTITUTE
CM032587.1	931971	.	T	C	100	*	TYPE=SUBSTITUTE
CM032587.1	931989	.	T	TTT	100	*	TYPE=INSERT
CM032587.1	931993	.	CT	C	100	*	TYPE=DELETE
CM032587.1	932006	.	C	T	100	*	TYPE=SUBSTITUTE
CM032587.1	932074	.	A	G	100	*	TYPE=SUBSTITUTE
CM032587.1	932099	.	C	A	100	*	TYPE=SUBSTITUTE
CM032587.1	932116	.	C	T	100	*	TYPE=SUBSTITUTE
CM032587.1	932118	.	G	C	100	*	TYPE=SUBSTITUTE

CM032587.1	932122	.	T	C	100	*	TYPE=SUBSTITUTE
CM032587.1	932125	.	C	T	100	*	TYPE=SUBSTITUTE
CM032587.1	932164	.	G	A	100	*	TYPE=SUBSTITUTE
CM032587.1	932167	.	C	T	100	*	TYPE=SUBSTITUTE
CM032587.1	932191	.	T	C	100	*	TYPE=SUBSTITUTE
CM032587.1	932197	.	C	T	100	*	TYPE=SUBSTITUTE
CM032587.1	932215	.	T	C	100	*	TYPE=SUBSTITUTE
CM032587.1	932293	.	A	G	100	*	TYPE=SUBSTITUTE
CM032587.1	932347	.	T	C	100	*	TYPE=SUBSTITUTE
CM032587.1	932366	.	A	G	100	*	TYPE=SUBSTITUTE
CM032587.1	932367	.	C	T	100	*	TYPE=SUBSTITUTE
CM032587.1	932368	.	T	G	100	*	TYPE=SUBSTITUTE
CM032587.1	932407	.	T	C	100	*	TYPE=SUBSTITUTE
CM032587.1	932426	.	A	C	100	*	TYPE=SUBSTITUTE
CM032587.1	932428	.	G	C	100	*	TYPE=SUBSTITUTE
CM032587.1	932435	.	A	G	100	*	TYPE=SUBSTITUTE
CM032587.1	932452	.	A	AACT	100	*	TYPE=INSERT
CM032587.1	932461	.	C	T	100	*	TYPE=SUBSTITUTE
CM032587.1	932476	.	A	G	100	*	TYPE=SUBSTITUTE
CM032587.1	932482	.	C	G	100	*	TYPE=SUBSTITUTE
CM032587.1	932497	.	C	T	100	*	TYPE=SUBSTITUTE
CM032587.1	932519	.	C	T	100	*	TYPE=SUBSTITUTE
CM032587.1	932530	.	C	T	100	*	TYPE=SUBSTITUTE
CM032587.1	932563	.	T	C	100	*	TYPE=SUBSTITUTE
CM032587.1	932599	.	C	T	100	*	TYPE=SUBSTITUTE
CM032587.1	932671	.	C	T	100	*	TYPE=SUBSTITUTE
CM032587.1	932719	.	T	C	100	*	TYPE=SUBSTITUTE
CM032587.1	932737	.	C	T	100	*	TYPE=SUBSTITUTE
CM032587.1	932755	.	T	G	100	*	TYPE=SUBSTITUTE
CM032587.1	932758	.	C	A	100	*	TYPE=SUBSTITUTE
CM032587.1	932773	.	T	C	100	*	TYPE=SUBSTITUTE
CM032587.1	932791	.	T	C	100	*	TYPE=SUBSTITUTE
CM032587.1	932810	.	C	A	100	*	TYPE=SUBSTITUTE
CM032587.1	932811	.	G	C	100	*	TYPE=SUBSTITUTE
CM032587.1	932814	.	A	G	100	*	TYPE=SUBSTITUTE
CM032587.1	932816	.	A	G	100	*	TYPE=SUBSTITUTE
CM032587.1	932821	.	C	T	100	*	TYPE=SUBSTITUTE
CM032587.1	932824	.	C	T	100	*	TYPE=SUBSTITUTE
CM032587.1	932830	.	T	A	100	*	TYPE=SUBSTITUTE
CM032587.1	932840	.	G	A	100	*	TYPE=SUBSTITUTE
CM032587.1	932858	.	C	A	100	*	TYPE=SUBSTITUTE
CM032587.1	932859	.	G	C	100	*	TYPE=SUBSTITUTE
CM032587.1	932862	.	A	G	100	*	TYPE=SUBSTITUTE
CM032587.1	932899	.	G	A	100	*	TYPE=SUBSTITUTE
CM032587.1	932902	.	TGGTACCGGCACCTATGGTTCAACC	T	100	*	TYPE=DELETE
CM032587.1	932932	.	TGATACCTTGGGCTCGTCTGGTACC	T	100	*	TYPE=DELETE
CM032587.1	932960	.	A	G	100	*	TYPE=SUBSTITUTE
CM032587.1	933054	.	CCAC	C	100	*	TYPE=DELETE
CM032587.1	933090	.	T	C	100	*	TYPE=SUBSTITUTE
CM032587.1	933136	.	C	G	100	*	TYPE=SUBSTITUTE
CM032587.1	933217	.	C	T	100	*	TYPE=SUBSTITUTE
CM032587.1	933257	.	A	C	100	*	TYPE=SUBSTITUTE
CM032587.1	933260	.	T	C	100	*	TYPE=SUBSTITUTE
CM032587.1	933261	.	C	T	100	*	TYPE=SUBSTITUTE
CM032587.1	933326	.	A	G	100	*	TYPE=SUBSTITUTE
CM032587.1	933328	.	G	A	100	*	TYPE=SUBSTITUTE
CM032587.1	933364	.	G	T	100	*	TYPE=SUBSTITUTE
CM032587.1	933376	.	G	A	100	*	TYPE=SUBSTITUTE
CM032587.1	933424	.	C	T	100	*	TYPE=SUBSTITUTE
CM032587.1	933569	.	t	tgaaat	100	*	TYPE=INSERT
CM032587.1	933576	.	c	ct	100	*	TYPE=INSERT
CM032587.1	933579	.	G	a	100	*	TYPE=SUBSTITUTE

CM032587.1	933600	.	G	A	100	*	TYPE=SUBSTITUTE
CM032587.1	933604	.	A	ATT	100	*	TYPE=INSERT
CM032587.1	933605	.	G	C	100	*	TYPE=SUBSTITUTE
CM032587.1	933606	.	C	G	100	*	TYPE=SUBSTITUTE
CM032587.1	933608	.	C	A	100	*	TYPE=SUBSTITUTE
CM032587.1	933609	.	T	G	100	*	TYPE=SUBSTITUTE
CM032587.1	933612	.	T	TC	100	*	TYPE=INSERT
CM032587.1	933620	.	T	C	100	*	TYPE=SUBSTITUTE
CM032587.1	933687	.	T	C	100	*	TYPE=SUBSTITUTE
CM032587.1	933694	.	G	A	100	*	TYPE=SUBSTITUTE
CM032587.1	933734	.	T	C	100	*	TYPE=SUBSTITUTE
CM032587.1	933748	.	G	T	100	*	TYPE=SUBSTITUTE
CM032587.1	933766	.	C	T	100	*	TYPE=SUBSTITUTE
CM032587.1	933767	.	T	G	100	*	TYPE=SUBSTITUTE
CM032587.1	933866	.	T	TT	100	*	TYPE=INSERT
CM032587.1	933884	.	C	CG	100	*	TYPE=INSERT
CM032587.1	933975	.	G	A	100	*	TYPE=SUBSTITUTE
CM032587.1	934004	.	T	TT	100	*	TYPE=INSERT
CM032587.1	934041	.	T	A	100	*	TYPE=SUBSTITUTE
CM032587.1	934043	.	A	AGCT	100	*	TYPE=INSERT
CM032587.1	934044	.	A	G	100	*	TYPE=SUBSTITUTE
CM032587.1	934045	.	G	T	100	*	TYPE=SUBSTITUTE
CM032587.1	934114	.	T	TGGAG	100	*	TYPE=INSERT
CM032587.1	934124	.	C	A	100	*	TYPE=SUBSTITUTE
CM032587.1	934144	.	G	A	100	*	TYPE=SUBSTITUTE
CM032587.1	934146	.	C	G	100	*	TYPE=SUBSTITUTE
CM032587.1	934147	.	TG	T	100	*	TYPE=DELETE
CM032587.1	934157	.	AGATGGTTG	A	100	*	TYPE=DELETE
CM032587.1	934175	.	AG	A	100	*	TYPE=DELETE
CM032587.1	934186	.	G	A	100	*	TYPE=SUBSTITUTE
CM032587.1	934191	.	T	TCC	100	*	TYPE=INSERT
CM032587.1	934194	.	A	G	100	*	TYPE=SUBSTITUTE
CM032587.1	934195	.	C	G	100	*	TYPE=SUBSTITUTE
CM032587.1	934200	.	G	C	100	*	TYPE=SUBSTITUTE
CM032587.1	934202	.	T	A	100	*	TYPE=SUBSTITUTE
CM032587.1	934226	.	T	C	100	*	TYPE=SUBSTITUTE
CM032587.1	934227	.	G	A	100	*	TYPE=SUBSTITUTE
CM032587.1	934228	.	T	G	100	*	TYPE=SUBSTITUTE
CM032587.1	934239	.	T	C	100	*	TYPE=SUBSTITUTE
CM032587.1	934245	.	A	G	100	*	TYPE=SUBSTITUTE
CM032587.1	934250	.	C	T	100	*	TYPE=SUBSTITUTE
CM032587.1	934266	.	C	T	100	*	TYPE=SUBSTITUTE
CM032587.1	934276	.	T	G	100	*	TYPE=SUBSTITUTE
CM032587.1	934305	.	T	C	100	*	TYPE=SUBSTITUTE
CM032587.1	934330	.	G	C	100	*	TYPE=SUBSTITUTE
CM032587.1	934349	.	C	T	100	*	TYPE=SUBSTITUTE
CM032587.1	934357	.	A	G	100	*	TYPE=SUBSTITUTE
CM032587.1	934376	.	a	actc	100	*	TYPE=INSERT
CM032587.1	934377	.	G	c	100	*	TYPE=SUBSTITUTE
CM032587.1	934421	.	AG	A	100	*	TYPE=DELETE
CM032587.1	934476	.	T	A	100	*	TYPE=SUBSTITUTE
CM032587.1	934504	.	G	A	100	*	TYPE=SUBSTITUTE
CM032587.1	934518	.	G	A	100	*	TYPE=SUBSTITUTE
CM032587.1	934566	.	G	A	100	*	TYPE=SUBSTITUTE
CM032587.1	934576	.	G	A	100	*	TYPE=SUBSTITUTE
CM032587.1	934579	.	C	T	100	*	TYPE=SUBSTITUTE
CM032587.1	934581	.	A	C	100	*	TYPE=SUBSTITUTE
CM032587.1	934587	.	T	C	100	*	TYPE=SUBSTITUTE
CM032587.1	934590	.	T	C	100	*	TYPE=SUBSTITUTE
CM032587.1	934614	.	G	A	100	*	TYPE=SUBSTITUTE
CM032587.1	934617	.	T	C	100	*	TYPE=SUBSTITUTE
CM032587.1	934618	.	C	T	100	*	TYPE=SUBSTITUTE
CM032587.1	934674	.	ATAT	A	100	*	TYPE=DELETE
CM032587.1	934703	.	T	C	100	*	TYPE=SUBSTITUTE

CM032587.1	934881	.	A	T	100	*	TYPE=SUBSTITUTE
CM032587.1	935143	.	TT	T	100	*	TYPE=DELETE
CM032587.1	935317	.	T	C	100	*	TYPE=SUBSTITUTE
CM032587.1	935543	.	T	C	100	*	TYPE=SUBSTITUTE
CM032587.1	935545	.	G	A	100	*	TYPE=SUBSTITUTE
CM032587.1	935578	.	G	T	100	*	TYPE=SUBSTITUTE
CM032587.1	935617	.	C	T	100	*	TYPE=SUBSTITUTE
CM032587.1	935677	.	T	C	100	*	TYPE=SUBSTITUTE
CM032587.1	935686	.	C	T	100	*	TYPE=SUBSTITUTE
CM032587.1	935689	.	C	T	100	*	TYPE=SUBSTITUTE
CM032587.1	935705	.	A	G	100	*	TYPE=SUBSTITUTE
CM032587.1	935708	.	A	T	100	*	TYPE=SUBSTITUTE
CM032587.1	935716	.	G	GT	100	*	TYPE=INSERT
CM032587.1	935737	.	T	G	100	*	TYPE=SUBSTITUTE
CM032587.1	935739	.	C	A	100	*	TYPE=SUBSTITUTE
CM032587.1	935740	.	C	A	100	*	TYPE=SUBSTITUTE
CM032587.1	935745	.	C	T	100	*	TYPE=SUBSTITUTE
CM032587.1	935752	.	C	T	100	*	TYPE=SUBSTITUTE
CM032587.1	935781	.	TT	T	100	*	TYPE=DELETE
CM032587.1	935783	.	T	G	100	*	TYPE=SUBSTITUTE
CM032587.1	935788	.	A	C	100	*	TYPE=SUBSTITUTE
CM032587.1	935804	.	A	G	100	*	TYPE=SUBSTITUTE
CM032587.1	935811	.	A	C	100	*	TYPE=SUBSTITUTE
CM032587.1	935816	.	T	A	100	*	TYPE=SUBSTITUTE
CM032587.1	935822	.	T	G	100	*	TYPE=SUBSTITUTE
CM032587.1	935824	.	A	T	100	*	TYPE=SUBSTITUTE
CM032587.1	935838	.	CA	C	100	*	TYPE=DELETE
CM032587.1	935841	.	G	A	100	*	TYPE=SUBSTITUTE
CM032587.1	935868	.	A	G	100	*	TYPE=SUBSTITUTE
CM032587.1	935869	.	A	G	100	*	TYPE=SUBSTITUTE
CM032587.1	935870	.	G	A	100	*	TYPE=SUBSTITUTE
CM032587.1	935871	.	C	A	100	*	TYPE=SUBSTITUTE
CM032587.1	935873	.	G	A	100	*	TYPE=SUBSTITUTE
CM032587.1	935879	.	G	T	100	*	TYPE=SUBSTITUTE
CM032587.1	935882	.	G	A	100	*	TYPE=SUBSTITUTE
CM032587.1	935933	.	C	t	100	*	TYPE=SUBSTITUTE
CM032587.1	935939	.	C	t	100	*	TYPE=SUBSTITUTE
CM032587.1	935942	.	C	t	100	*	TYPE=SUBSTITUTE
CM032587.1	935975	.	G	a	100	*	TYPE=SUBSTITUTE
CM032587.1	935987	.	G	A	100	*	TYPE=SUBSTITUTE
CM032587.1	935990	.	C	T	100	*	TYPE=SUBSTITUTE
CM032587.1	935992	.	C	T	100	*	TYPE=SUBSTITUTE
CM032587.1	936080	.	A	G	100	*	TYPE=SUBSTITUTE
CM032587.1	936224	.	A	G	100	*	TYPE=SUBSTITUTE
CM032587.1	936512	.	G	C	100	*	TYPE=SUBSTITUTE
CM032587.1	936596	.	C	A	100	*	TYPE=SUBSTITUTE
CM032587.1	936647	.	A	T	100	*	TYPE=SUBSTITUTE
CM032587.1	936662	.	G	A	100	*	TYPE=SUBSTITUTE
CM032587.1	936707	.	C	G	100	*	TYPE=SUBSTITUTE
CM032587.1	936722	.	T	C	100	*	TYPE=SUBSTITUTE
CM032587.1	936809	.	C	T	100	*	TYPE=SUBSTITUTE
CM032587.1	936992	.	A	G	100	*	TYPE=SUBSTITUTE
CM032587.1	937025	.	T	C	100	*	TYPE=SUBSTITUTE
CM032587.1	937029	.	C	T	100	*	TYPE=SUBSTITUTE
CM032587.1	937046	.	A	G	100	*	TYPE=SUBSTITUTE
CM032587.1	937054	.	G	A	100	*	TYPE=SUBSTITUTE
CM032587.1	937064	.	T	C	100	*	TYPE=SUBSTITUTE
CM032587.1	937076	.	A	G	100	*	TYPE=SUBSTITUTE
CM032587.1	937142	.	G	C	100	*	TYPE=SUBSTITUTE
CM032587.1	937191	.	G	A	100	*	TYPE=SUBSTITUTE
CM032587.1	937196	.	G	T	100	*	TYPE=SUBSTITUTE
CM032587.1	937202	.	T	C	100	*	TYPE=SUBSTITUTE
CM032587.1	937208	.	G	A	100	*	TYPE=SUBSTITUTE
CM032587.1	937211	.	G	A	100	*	TYPE=SUBSTITUTE

CM032587.1	937226	.	A	T	100	*	TYPE=SUBSTITUTE
CM032587.1	937286	.	T	C	100	*	TYPE=SUBSTITUTE
CM032587.1	937304	.	A	G	100	*	TYPE=SUBSTITUTE
CM032587.1	937345	.	G	A	100	*	TYPE=SUBSTITUTE
CM032587.1	937355	.	T	C	100	*	TYPE=SUBSTITUTE
CM032587.1	937426	.	A	T	100	*	TYPE=SUBSTITUTE
CM032587.1	937582	.	T	tt	100	*	TYPE=INSERT
CM032587.1	937631	.	t	tc	100	*	TYPE=INSERT
CM032587.1	937636	.	T	c	100	*	TYPE=SUBSTITUTE
CM032587.1	937691	.	C	T	100	*	TYPE=SUBSTITUTE
CM032587.1	937739	.	G	GCAAGG	100	*	TYPE=INSERT
CM032587.1	937740	.	G	T	100	*	TYPE=SUBSTITUTE
CM032587.1	937741	.	C	T	100	*	TYPE=SUBSTITUTE
CM032587.1	937922	.	G	A	100	*	TYPE=SUBSTITUTE
CM032587.1	938054	.	CAA	C	100	*	TYPE=DELETE
CM032587.1	938093	.	G	T	100	*	TYPE=SUBSTITUTE
CM032587.1	938118	.	A	C	100	*	TYPE=SUBSTITUTE
CM032587.1	938330	.	C	T	100	*	TYPE=SUBSTITUTE
CM032587.1	938377	.	G	A	100	*	TYPE=SUBSTITUTE
CM032587.1	938422	.	T	g	100	*	TYPE=SUBSTITUTE
CM032587.1	938425	.	A	g	100	*	TYPE=SUBSTITUTE
CM032587.1	938446	.	A	G	100	*	TYPE=SUBSTITUTE
CM032587.1	938449	.	T	C	100	*	TYPE=SUBSTITUTE
CM032587.1	938535	.	A	G	100	*	TYPE=SUBSTITUTE
CM032587.1	938536	.	T	C	100	*	TYPE=SUBSTITUTE
CM032587.1	938685	.	G	A	100	*	TYPE=SUBSTITUTE
CM032587.1	938752	.	G	T	100	*	TYPE=SUBSTITUTE
CM032587.1	938840	.	G	A	100	*	TYPE=SUBSTITUTE
CM032587.1	938927	.	T	C	100	*	TYPE=SUBSTITUTE
CM032587.1	938975	.	C	T	100	*	TYPE=SUBSTITUTE
CM032587.1	939034	.	A	G	100	*	TYPE=SUBSTITUTE
CM032587.1	939040	.	A	C	100	*	TYPE=SUBSTITUTE
CM032587.1	939071	.	G	T	100	*	TYPE=SUBSTITUTE
CM032587.1	939072	.	T	A	100	*	TYPE=SUBSTITUTE
CM032587.1	939074	.	T	C	100	*	TYPE=SUBSTITUTE
CM032587.1	939082	.	G	A	100	*	TYPE=SUBSTITUTE
CM032587.1	939088	.	C	T	100	*	TYPE=SUBSTITUTE
CM032587.1	939149	.	G	A	100	*	TYPE=SUBSTITUTE
CM032587.1	939174	.	T	G	100	*	TYPE=SUBSTITUTE
CM032587.1	939213	.	C	T	100	*	TYPE=SUBSTITUTE
CM032587.1	939223	.	T	A	100	*	TYPE=SUBSTITUTE
CM032587.1	939273	.	G	A	100	*	TYPE=SUBSTITUTE
CM032587.1	939305	.	C	t	100	*	TYPE=SUBSTITUTE
CM032587.1	939328	.	A	AA	100	*	TYPE=INSERT
CM032587.1	939366	.	AA	A	100	*	TYPE=DELETE
CM032587.1	939456	.	A	C	100	*	TYPE=SUBSTITUTE
CM032587.1	939460	.	G	T	100	*	TYPE=SUBSTITUTE
CM032587.1	939464	.	G	GA	100	*	TYPE=INSERT
CM032587.1	939466	.	G	GGAA	100	*	TYPE=INSERT
CM032587.1	939473	.	C	T	100	*	TYPE=SUBSTITUTE
CM032587.1	939475	.	GA	G	100	*	TYPE=DELETE
CM032587.1	939487	.	A	AT	100	*	TYPE=INSERT
CM032587.1	939504	.	A	AAGTA	100	*	TYPE=INSERT
CM032587.1	939505	.	T	A	100	*	TYPE=SUBSTITUTE
CM032587.1	939516	.	AGT	A	100	*	TYPE=DELETE
CM032587.1	939527	.	G	A	100	*	TYPE=SUBSTITUTE
CM032587.1	939557	.	AA	A	100	*	TYPE=DELETE
CM032587.1	939559	.	C	T	100	*	TYPE=SUBSTITUTE
CM032587.1	939583	.	A	G	100	*	TYPE=SUBSTITUTE
CM032587.1	939597	.	T	tacaa	100	*	TYPE=INSERT
CM032587.1	939613	.	CC	C	100	*	TYPE=DELETE
CM032587.1	939619	.	T	C	100	*	TYPE=SUBSTITUTE
CM032587.1	939625	.	TTAG	T	100	*	TYPE=DELETE
CM032587.1	939642	.	A	G	100	*	TYPE=SUBSTITUTE

CM032587.1	939695	.	A	C	100	*	TYPE=SUBSTITUTE
CM032587.1	939764	.	A	AA	100	*	TYPE=INSERT
CM032587.1	939771	.	A	T	100	*	TYPE=SUBSTITUTE
CM032587.1	939783	.	C	CAA	100	*	TYPE=INSERT
CM032587.1	939784	.	T	C	100	*	TYPE=SUBSTITUTE
CM032587.1	939786	.	A	AT	100	*	TYPE=INSERT
CM032587.1	939787	.	C	A	100	*	TYPE=SUBSTITUTE
CM032587.1	939802	.	a	aga	100	*	TYPE=INSERT
CM032587.1	939804	.	G	a	100	*	TYPE=SUBSTITUTE
CM032587.1	939805	.	A	g	100	*	TYPE=SUBSTITUTE
CM032587.1	939822	.	a	ag	100	*	TYPE=INSERT
CM032587.1	939889	.	t	tttt	100	*	TYPE=INSERT
CM032587.1	939890	.	C	t	100	*	TYPE=SUBSTITUTE
CM032587.1	939891	.	C	t	100	*	TYPE=SUBSTITUTE
CM032587.1	939971	.	C	CC	100	*	TYPE=INSERT
CM032587.1	940005	.	G	gg	100	*	TYPE=INSERT
CM032587.1	940033	.	G	A	100	*	TYPE=SUBSTITUTE
CM032587.1	940040	.	GGAAA	G	100	*	TYPE=DELETE
CM032587.1	940150	.	AGA	A	100	*	TYPE=DELETE
CM032587.1	940170	.	G	a	100	*	TYPE=SUBSTITUTE
CM032587.1	940171	.	a	ag	100	*	TYPE=INSERT
CM032587.1	940177	.	a	agaaagttATTCTAC	100	*	TYPE=INSERT
CM032587.1	940187	.	CA	C	100	*	TYPE=DELETE
CM032587.1	940194	.	C	a	100	*	TYPE=SUBSTITUTE
CM032587.1	940212	.	TACCTA	T	100	*	TYPE=DELETE
CM032587.1	940221	.	TGAGAGAGAGAAAAA	T	100	*	TYPE=DELETE
CM032587.1	940240	.	G	A	100	*	TYPE=SUBSTITUTE
CM032587.1	940249	.	T	C	100	*	TYPE=SUBSTITUTE
CM032587.1	940331	.	C	CCA	100	*	TYPE=INSERT
CM032587.1	940332	.	G	C	100	*	TYPE=SUBSTITUTE
CM032587.1	940342	.	GTAA	G	100	*	TYPE=DELETE
CM032587.1	940353	.	AT	A	100	*	TYPE=DELETE
CM032587.1	940356	.	CAA	C	100	*	TYPE=DELETE
CM032587.1	940374	.	C	t	100	*	TYPE=SUBSTITUTE
CM032587.1	940377	.	A	T	100	*	TYPE=SUBSTITUTE
CM032587.1	940379	.	C	A	100	*	TYPE=SUBSTITUTE
CM032587.1	940395	.	CTGGC	C	100	*	TYPE=DELETE
CM032587.1	940495	.	GGCCTAC	G	100	*	TYPE=DELETE
CM032587.1	940502	.	A	T	100	*	TYPE=SUBSTITUTE
CM032587.1	940505	.	AACAAGCGCG	A	100	*	TYPE=DELETE
CM032587.1	940516	.	GAGTC	G	100	*	TYPE=DELETE
CM032587.1	940551	.	GAG	G	100	*	TYPE=DELETE
CM032587.1	940562	.	T	TCG	100	*	TYPE=INSERT
CM032587.1	940577	.	T	C	100	*	TYPE=SUBSTITUTE
CM032587.1	940588	.	C	G	100	*	TYPE=SUBSTITUTE
CM032587.1	940590	.	T	TC	100	*	TYPE=INSERT
CM032587.1	940593	.	T	A	100	*	TYPE=SUBSTITUTE
CM032587.1	940594	.	C	A	100	*	TYPE=SUBSTITUTE
CM032587.1	940596	.	C	t	100	*	TYPE=SUBSTITUTE
CM032587.1	940605	.	T	c	100	*	TYPE=SUBSTITUTE
CM032587.1	940614	.	C	t	100	*	TYPE=SUBSTITUTE
CM032587.1	940617	.	C	T	100	*	TYPE=SUBSTITUTE
CM032587.1	940644	.	G	A	100	*	TYPE=SUBSTITUTE
CM032587.1	940675	.	C	g	100	*	TYPE=SUBSTITUTE
CM032587.1	940683	.	C	T	100	*	TYPE=SUBSTITUTE
CM032587.1	940800	.	G	C	100	*	TYPE=SUBSTITUTE
CM032587.1	940801	.	T	C	100	*	TYPE=SUBSTITUTE
CM032587.1	940804	.	A	G	100	*	TYPE=SUBSTITUTE
CM032587.1	940852	.	AC	A	100	*	TYPE=DELETE
CM032587.1	940883	.	a	aga	100	*	TYPE=INSERT
CM032587.1	940887	.	C	t	100	*	TYPE=SUBSTITUTE
CM032587.1	940889	.	A	t	100	*	TYPE=SUBSTITUTE
CM032587.1	941216	.	C	G	100	*	TYPE=SUBSTITUTE
CM032587.1	941367	.	A	T	100	*	TYPE=SUBSTITUTE

CM032587.1	941368	.	T	A	100	*	TYPE=SUBSTITUTE
CM032587.1	941369	.	A	C	100	*	TYPE=SUBSTITUTE
CM032587.1	941393	.	G	GAATAGCCG	100	*	TYPE=INSERT
CM032587.1	941407	.	T	C	100	*	TYPE=SUBSTITUTE
CM032587.1	941450	.	TTCT	T	100	*	TYPE=DELETE
CM032587.1	941462	.	a	at	100	*	TYPE=INSERT
CM032587.1	941498	.	TT	T	100	*	TYPE=DELETE
CM032587.1	941633	.	T	C	100	*	TYPE=SUBSTITUTE
CM032587.1	941651	.	C	T	100	*	TYPE=SUBSTITUTE
CM032587.1	941691	.	C	G	100	*	TYPE=SUBSTITUTE
CM032587.1	941868	.	A	C	100	*	TYPE=SUBSTITUTE
CM032587.1	941882	.	A	G	100	*	TYPE=SUBSTITUTE
CM032587.1	941883	.	G	A	100	*	TYPE=SUBSTITUTE
CM032587.1	941909	.	a	aa	100	*	TYPE=INSERT
CM032587.1	941919	.	T	C	100	*	TYPE=SUBSTITUTE
CM032587.1	941969	.	A	aa	100	*	TYPE=INSERT
CM032587.1	942069	.	TT	T	100	*	TYPE=DELETE
CM032587.1	942243	.	A	C	100	*	TYPE=SUBSTITUTE
CM032587.1	942308	.	G	GC	100	*	TYPE=INSERT
CM032587.1	942328	.	G	T	100	*	TYPE=SUBSTITUTE
CM032587.1	942355	.	GAGG	G	100	*	TYPE=DELETE
CM032587.1	942359	.	T	C	100	*	TYPE=SUBSTITUTE
CM032587.1	942415	.	A	G	100	*	TYPE=SUBSTITUTE
CM032587.1	942426	.	C	A	100	*	TYPE=SUBSTITUTE
CM032587.1	942428	.	G	C	100	*	TYPE=SUBSTITUTE
CM032587.1	942430	.	C	G	100	*	TYPE=SUBSTITUTE
CM032587.1	942431	.	A	T	100	*	TYPE=SUBSTITUTE
CM032587.1	942434	.	GCCCGT	G	100	*	TYPE=DELETE
CM032587.1	942440	.	T	A	100	*	TYPE=SUBSTITUTE
CM032587.1	942441	.	G	T	100	*	TYPE=SUBSTITUTE
CM032587.1	942475	.	C	T	100	*	TYPE=SUBSTITUTE
CM032587.1	942481	.	C	T	100	*	TYPE=SUBSTITUTE
CM032587.1	942487	.	G	A	100	*	TYPE=SUBSTITUTE
CM032587.1	942495	.	T	C	100	*	TYPE=SUBSTITUTE
CM032587.1	942514	.	G	A	100	*	TYPE=SUBSTITUTE
CM032587.1	942580	.	G	C	100	*	TYPE=SUBSTITUTE
CM032587.1	942589	.	C	G	100	*	TYPE=SUBSTITUTE
CM032587.1	942596	.	G	A	100	*	TYPE=SUBSTITUTE
CM032587.1	942647	.	G	A	100	*	TYPE=SUBSTITUTE
CM032587.1	942668	.	A	G	100	*	TYPE=SUBSTITUTE
CM032587.1	942671	.	C	T	100	*	TYPE=SUBSTITUTE
CM032587.1	942786	.	TT	T	100	*	TYPE=DELETE
CM032587.1	942827	.	G	C	100	*	TYPE=SUBSTITUTE
CM032587.1	943013	.	A	G	100	*	TYPE=SUBSTITUTE
CM032587.1	943046	.	A	G	100	*	TYPE=SUBSTITUTE
CM032587.1	943068	.	A	G	100	*	TYPE=SUBSTITUTE
CM032587.1	943111	.	G	A	100	*	TYPE=SUBSTITUTE
CM032587.1	943124	.	G	A	100	*	TYPE=SUBSTITUTE
CM032587.1	943125	.	A	C	100	*	TYPE=SUBSTITUTE
CM032587.1	943145	.	C	T	100	*	TYPE=SUBSTITUTE
CM032587.1	943147	.	T	C	100	*	TYPE=SUBSTITUTE
CM032587.1	943180	.	T	G	100	*	TYPE=SUBSTITUTE
CM032587.1	943189	.	T	G	100	*	TYPE=SUBSTITUTE
CM032587.1	943217	.	C	T	100	*	TYPE=SUBSTITUTE
CM032587.1	943279	.	T	G	100	*	TYPE=SUBSTITUTE
CM032587.1	943295	.	G	A	100	*	TYPE=SUBSTITUTE
CM032587.1	943298	.	G	A	100	*	TYPE=SUBSTITUTE
CM032587.1	943384	.	A	T	100	*	TYPE=SUBSTITUTE
CM032587.1	943392	.	A	T	100	*	TYPE=SUBSTITUTE
CM032587.1	943417	.	A	G	100	*	TYPE=SUBSTITUTE
CM032587.1	943471	.	GGTAG	G	100	*	TYPE=DELETE
CM032587.1	943536	.	C	A	100	*	TYPE=SUBSTITUTE
CM032587.1	943556	.	A	G	100	*	TYPE=SUBSTITUTE
CM032587.1	943621	.	A	g	100	*	TYPE=SUBSTITUTE

CM032587.1	943641	.	G	a	100	*	TYPE=SUBSTITUTE
CM032587.1	943652	.	T	a	100	*	TYPE=SUBSTITUTE
CM032587.1	943654	.	t	ta	100	*	TYPE=INSERT
CM032587.1	943656	.	T	C	100	*	TYPE=SUBSTITUTE
CM032587.1	943784	.	A	G	100	*	TYPE=SUBSTITUTE
CM032587.1	943797	.	A	T	100	*	TYPE=SUBSTITUTE
CM032587.1	943841	.	C	T	100	*	TYPE=SUBSTITUTE
CM032587.1	943904	.	T	c	100	*	TYPE=SUBSTITUTE
CM032587.1	944013	.	CCTC	C	100	*	TYPE=DELETE
CM032587.1	944244	.	A	C	100	*	TYPE=SUBSTITUTE
CM032587.1	944273	.	G	a	100	*	TYPE=SUBSTITUTE
CM032587.1	944281	.	C	t	100	*	TYPE=SUBSTITUTE
CM032587.1	944291	.	G	A	100	*	TYPE=SUBSTITUTE
CM032587.1	944292	.	A	G	100	*	TYPE=SUBSTITUTE
CM032587.1	944322	.	GGA	G	100	*	TYPE=DELETE
CM032587.1	944330	.	GA	G	100	*	TYPE=DELETE
CM032587.1	944355	.	G	A	100	*	TYPE=SUBSTITUTE
CM032587.1	944359	.	AG	A	100	*	TYPE=DELETE
CM032587.1	944402	.	T	A	100	*	TYPE=SUBSTITUTE
CM032587.1	944403	.	G	A	100	*	TYPE=SUBSTITUTE
CM032587.1	944407	.	T	C	100	*	TYPE=SUBSTITUTE
CM032587.1	944460	.	A	C	100	*	TYPE=SUBSTITUTE
CM032587.1	944461	.	G	A	100	*	TYPE=SUBSTITUTE
CM032587.1	944486	.	GG	G	100	*	TYPE=DELETE
CM032587.1	944529	.	A	T	100	*	TYPE=SUBSTITUTE
CM032587.1	944540	.	C	T	100	*	TYPE=SUBSTITUTE
CM032587.1	944556	.	GC	G	100	*	TYPE=DELETE
CM032587.1	944585	.	G	A	100	*	TYPE=SUBSTITUTE
CM032587.1	944621	.	T	C	100	*	TYPE=SUBSTITUTE
CM032587.1	944631	.	C	T	100	*	TYPE=SUBSTITUTE
CM032587.1	944649	.	A	T	100	*	TYPE=SUBSTITUTE
CM032587.1	944650	.	C	T	100	*	TYPE=SUBSTITUTE
CM032587.1	944676	.	G	A	100	*	TYPE=SUBSTITUTE
CM032587.1	944679	.	G	a	100	*	TYPE=SUBSTITUTE
CM032587.1	944680	.	G	a	100	*	TYPE=SUBSTITUTE
CM032587.1	944691	.	G	c	100	*	TYPE=SUBSTITUTE
CM032587.1	944693	.	T	c	100	*	TYPE=SUBSTITUTE
CM032587.1	944694	.	C	t	100	*	TYPE=SUBSTITUTE
CM032587.1	944695	.	T	c	100	*	TYPE=SUBSTITUTE
CM032587.1	944766	.	G	A	100	*	TYPE=SUBSTITUTE
CM032587.1	944869	.	T	G	100	*	TYPE=SUBSTITUTE
CM032587.1	944920	.	C	T	100	*	TYPE=SUBSTITUTE
CM032587.1	944925	.	A	G	100	*	TYPE=SUBSTITUTE
CM032587.1	944977	.	A	G	100	*	TYPE=SUBSTITUTE
CM032587.1	945070	.	T	TCTACG	100	*	TYPE=INSERT
CM032587.1	945116	.	T	C	100	*	TYPE=SUBSTITUTE
CM032587.1	945178	.	A	G	100	*	TYPE=SUBSTITUTE
CM032587.1	945247	.	T	TACTA	100	*	TYPE=INSERT
CM032587.1	945331	.	A	G	100	*	TYPE=SUBSTITUTE
CM032587.1	945334	.	A	G	100	*	TYPE=SUBSTITUTE
CM032587.1	945355	.	GC	G	100	*	TYPE=DELETE
CM032587.1	945363	.	CA	C	100	*	TYPE=DELETE
CM032587.1	945464	.	G	A	100	*	TYPE=SUBSTITUTE
CM032587.1	945486	.	T	C	100	*	TYPE=SUBSTITUTE
CM032587.1	945501	.	CC	C	100	*	TYPE=DELETE
CM032587.1	945503	.	A	T	100	*	TYPE=SUBSTITUTE
CM032587.1	945564	.	T	tt	100	*	TYPE=INSERT
CM032587.1	945700	.	C	T	100	*	TYPE=SUBSTITUTE
CM032587.1	945763	.	TT	T	100	*	TYPE=DELETE
CM032587.1	945773	.	A	G	100	*	TYPE=SUBSTITUTE
CM032587.1	945775	.	A	AT	100	*	TYPE=INSERT
CM032587.1	945782	.	C	A	100	*	TYPE=SUBSTITUTE
CM032587.1	945833	.	CGC	C	100	*	TYPE=DELETE
CM032587.1	945936	.	t	tt	100	*	TYPE=INSERT

CM032587.1	945937	.	C	t	100	*	TYPE=SUBSTITUTE
CM032587.1	945983	.	T	TA	100	*	TYPE=INSERT
CM032587.1	945997	.	T	G	100	*	TYPE=SUBSTITUTE
CM032587.1	946063	.	A	G	100	*	TYPE=SUBSTITUTE
CM032587.1	946307	.	A	ag	100	*	TYPE=INSERT
CM032587.1	946379	.	T	G	100	*	TYPE=SUBSTITUTE
CM032587.1	946533	.	A	AA	100	*	TYPE=INSERT
CM032587.1	946552	.	T	c	100	*	TYPE=SUBSTITUTE
CM032587.1	946574	.	G	A	100	*	TYPE=SUBSTITUTE
CM032587.1	946576	.	T	A	100	*	TYPE=SUBSTITUTE
CM032587.1	946598	.	T	A	100	*	TYPE=SUBSTITUTE
CM032587.1	946640	.	C	G	100	*	TYPE=SUBSTITUTE
CM032587.1	946698	.	AGTACGA	A	100	*	TYPE=DELETE
CM032587.1	946705	.	G	A	100	*	TYPE=SUBSTITUTE
CM032587.1	946716	.	T	A	100	*	TYPE=SUBSTITUTE
CM032587.1	946718	.	A	G	100	*	TYPE=SUBSTITUTE
CM032587.1	946737	.	AA	A	100	*	TYPE=DELETE
CM032587.1	946745	.	TA	T	100	*	TYPE=DELETE
CM032587.1	946829	.	AA	A	100	*	TYPE=DELETE
CM032587.1	946939	.	AA	A	100	*	TYPE=DELETE
CM032587.1	946955	.	A	AATGGCC	100	*	TYPE=INSERT
CM032587.1	946956	.	T	A	100	*	TYPE=SUBSTITUTE
CM032587.1	946967	.	G	A	100	*	TYPE=SUBSTITUTE
CM032587.1	946981	.	GC	G	100	*	TYPE=DELETE
CM032587.1	947034	.	C	T	100	*	TYPE=SUBSTITUTE
CM032587.1	947084	.	A	atag	100	*	TYPE=INSERT
CM032587.1	947177	.	AA	A	100	*	TYPE=DELETE
CM032587.1	947200	.	A	G	100	*	TYPE=SUBSTITUTE
CM032587.1	947202	.	C	A	100	*	TYPE=SUBSTITUTE
CM032587.1	947204	.	A	C	100	*	TYPE=SUBSTITUTE
CM032587.1	947205	.	C	T	100	*	TYPE=SUBSTITUTE
CM032587.1	947207	.	T	C	100	*	TYPE=SUBSTITUTE
CM032587.1	947269	.	T	TT	100	*	TYPE=INSERT
CM032587.1	947317	.	A	G	100	*	TYPE=SUBSTITUTE
CM032587.1	947376	.	C	A	100	*	TYPE=SUBSTITUTE
CM032587.1	947383	.	C	T	100	*	TYPE=SUBSTITUTE
CM032587.1	947571	.	C	T	100	*	TYPE=SUBSTITUTE
CM032587.1	947581	.	CGTACGA	C	100	*	TYPE=DELETE
CM032587.1	947595	.	C	T	100	*	TYPE=SUBSTITUTE
CM032587.1	947596	.	C	T	100	*	TYPE=SUBSTITUTE
CM032587.1	947791	.	A	G	100	*	TYPE=SUBSTITUTE
CM032587.1	947793	.	T	G	100	*	TYPE=SUBSTITUTE
CM032587.1	947811	.	C	T	100	*	TYPE=SUBSTITUTE
CM032587.1	947912	.	C	G	100	*	TYPE=SUBSTITUTE
CM032587.1	947925	.	C	T	100	*	TYPE=SUBSTITUTE
CM032587.1	947926	.	C	T	100	*	TYPE=SUBSTITUTE
CM032587.1	947959	.	GA	G	100	*	TYPE=DELETE
CM032587.1	948009	.	G	GTAG	100	*	TYPE=INSERT
CM032587.1	948010	.	C	T	100	*	TYPE=SUBSTITUTE
CM032587.1	948093	.	TT	T	100	*	TYPE=DELETE
CM032587.1	948370	.	CC	C	100	*	TYPE=DELETE
CM032587.1	948496	.	G	C	100	*	TYPE=SUBSTITUTE
CM032587.1	948612	.	A	T	100	*	TYPE=SUBSTITUTE
CM032587.1	948723	.	a	aa	100	*	TYPE=INSERT
CM032587.1	948733	.	TTACCGCGAGA	T	100	*	TYPE=DELETE
CM032587.1	948770	.	G	A	100	*	TYPE=SUBSTITUTE
CM032587.1	948775	.	C	T	100	*	TYPE=SUBSTITUTE
CM032587.1	948805	.	T	A	100	*	TYPE=SUBSTITUTE
CM032587.1	948823	.	T	TCG	100	*	TYPE=INSERT
CM032587.1	948825	.	G	T	100	*	TYPE=SUBSTITUTE
CM032587.1	948826	.	A	G	100	*	TYPE=SUBSTITUTE
CM032587.1	948842	.	C	G	100	*	TYPE=SUBSTITUTE
CM032587.1	948849	.	T	C	100	*	TYPE=SUBSTITUTE
CM032587.1	948874	.	G	GA	100	*	TYPE=INSERT

CM032587.1	948875	.	T	G	100	*	TYPE=SUBSTITUTE
CM032587.1	948878	.	CAT	C	100	*	TYPE=DELETE
CM032587.1	948881	.	G	T	100	*	TYPE=SUBSTITUTE
CM032587.1	948882	.	A	C	100	*	TYPE=SUBSTITUTE
CM032587.1	948890	.	C	A	100	*	TYPE=SUBSTITUTE
CM032587.1	948899	.	A	ACAAG	100	*	TYPE=INSERT
CM032587.1	949137	.	A	C	100	*	TYPE=SUBSTITUTE
CM032587.1	949148	.	C	T	100	*	TYPE=SUBSTITUTE
CM032587.1	949153	.	A	AGTC	100	*	TYPE=INSERT
CM032587.1	949232	.	C	CC	100	*	TYPE=INSERT
CM032587.1	949244	.	A	C	100	*	TYPE=SUBSTITUTE
CM032587.1	949245	.	A	T	100	*	TYPE=SUBSTITUTE
CM032587.1	949253	.	C	g	100	*	TYPE=SUBSTITUTE
CM032587.1	949273	.	CT	C	100	*	TYPE=DELETE
CM032587.1	949291	.	T	C	100	*	TYPE=SUBSTITUTE
CM032587.1	949454	.	GGAA	G	100	*	TYPE=DELETE
CM032587.1	949467	.	G	a	100	*	TYPE=SUBSTITUTE
CM032587.1	949474	.	A	g	100	*	TYPE=SUBSTITUTE
CM032587.1	949628	.	a	aaaat	100	*	TYPE=INSERT
CM032587.1	949642	.	C	T	100	*	TYPE=SUBSTITUTE
CM032587.1	949830	.	A	G	100	*	TYPE=SUBSTITUTE
CM032587.1	949927	.	GG	G	100	*	TYPE=DELETE
CM032587.1	950058	.	GG	G	100	*	TYPE=DELETE
CM032587.1	950078	.	G	A	100	*	TYPE=SUBSTITUTE
CM032587.1	950080	.	G	T	100	*	TYPE=SUBSTITUTE
CM032587.1	950081	.	T	TG	100	*	TYPE=INSERT
CM032587.1	950777	.	TT	T	100	*	TYPE=DELETE
CM032587.1	950910	.	A	G	100	*	TYPE=SUBSTITUTE
CM032587.1	950961	.	CC	C	100	*	TYPE=DELETE
CM032587.1	951015	.	G	A	100	*	TYPE=SUBSTITUTE
CM032587.1	951190	.	C	G	100	*	TYPE=SUBSTITUTE
CM032587.1	951220	.	GG	G	100	*	TYPE=DELETE
CM032587.1	951387	.	C	T	100	*	TYPE=SUBSTITUTE
CM032587.1	951430	.	C	CT	100	*	TYPE=INSERT
CM032587.1	951473	.	TTT	T	100	*	TYPE=DELETE
CM032587.1	951571	.	G	T	100	*	TYPE=SUBSTITUTE
CM032587.1	951580	.	T	TG	100	*	TYPE=INSERT
CM032587.1	951581	.	A	C	100	*	TYPE=SUBSTITUTE
CM032587.1	951582	.	G	T	100	*	TYPE=SUBSTITUTE
CM032587.1	951603	.	CCATC	C	100	*	TYPE=DELETE
CM032587.1	951611	.	T	A	100	*	TYPE=SUBSTITUTE
CM032587.1	951612	.	A	T	100	*	TYPE=SUBSTITUTE
CM032587.1	951652	.	TAT	T	100	*	TYPE=DELETE
CM032587.1	951702	.	AA	A	100	*	TYPE=DELETE
CM032587.1	952210	.	CC	C	100	*	TYPE=DELETE
CM032587.1	952410	.	G	C	100	*	TYPE=SUBSTITUTE
CM032587.1	952411	.	G	GAC	100	*	TYPE=INSERT
CM032587.1	952438	.	TCTT	T	100	*	TYPE=DELETE
CM032587.1	952461	.	C	CTGGC	100	*	TYPE=INSERT
CM032587.1	952462	.	C	T	100	*	TYPE=SUBSTITUTE
CM032587.1	952463	.	A	G	100	*	TYPE=SUBSTITUTE
CM032587.1	952465	.	A	C	100	*	TYPE=SUBSTITUTE
CM032587.1	952468	.	G	T	100	*	TYPE=SUBSTITUTE
CM032587.1	952469	.	C	A	100	*	TYPE=SUBSTITUTE
CM032587.1	952470	.	A	C	100	*	TYPE=SUBSTITUTE
CM032587.1	952471	.	T	G	100	*	TYPE=SUBSTITUTE
CM032587.1	952472	.	C	G	100	*	TYPE=SUBSTITUTE
CM032587.1	952491	.	CGT	C	100	*	TYPE=DELETE
CM032587.1	952526	.	CTC	C	100	*	TYPE=DELETE
CM032587.1	952563	.	C	CCCCCCC	100	*	TYPE=INSERT
CM032587.1	952789	.	t	tc	100	*	TYPE=INSERT
CM032587.1	952792	.	C	a	100	*	TYPE=SUBSTITUTE
CM032587.1	952794	.	T	c	100	*	TYPE=SUBSTITUTE
CM032587.1	952797	.	CT	C	100	*	TYPE=DELETE

CM032587.1	952803	.	c	ct	100	*	TYPE=INSERT
CM032587.1	952816	.	T	C	100	*	TYPE=SUBSTITUTE
CM032587.1	953251	.	C	T	100	*	TYPE=SUBSTITUTE
CM032587.1	953513	.	CCC	C	100	*	TYPE=DELETE
CM032587.1	953570	.	AG	A	100	*	TYPE=DELETE
CM032587.1	953845	.	C	T	100	*	TYPE=SUBSTITUTE
CM032587.1	953985	.	G	A	100	*	TYPE=SUBSTITUTE
CM032587.1	954027	.	C	T	100	*	TYPE=SUBSTITUTE
CM032587.1	954030	.	A	G	100	*	TYPE=SUBSTITUTE
CM032587.1	954120	.	G	A	100	*	TYPE=SUBSTITUTE
CM032587.1	954153	.	T	C	100	*	TYPE=SUBSTITUTE
CM032587.1	954234	.	A	C	100	*	TYPE=SUBSTITUTE
CM032587.1	954289	.	A	C	100	*	TYPE=SUBSTITUTE
CM032587.1	954297	.	ATG	A	100	*	TYPE=DELETE
CM032587.1	954303	.	T	C	100	*	TYPE=SUBSTITUTE
CM032587.1	954332	.	A	G	100	*	TYPE=SUBSTITUTE
CM032587.1	954383	.	A	G	100	*	TYPE=SUBSTITUTE
CM032587.1	954410	.	A	G	100	*	TYPE=SUBSTITUTE
CM032587.1	954430	.	A	G	100	*	TYPE=SUBSTITUTE
CM032587.1	954450	.	T	TT	100	*	TYPE=INSERT
CM032587.1	954485	.	A	G	100	*	TYPE=SUBSTITUTE
CM032587.1	954502	.	C	A	100	*	TYPE=SUBSTITUTE
CM032587.1	954506	.	G	A	100	*	TYPE=SUBSTITUTE
CM032587.1	954511	.	AT	A	100	*	TYPE=DELETE
CM032587.1	954581	.	T	C	100	*	TYPE=SUBSTITUTE
CM032587.1	954596	.	A	G	100	*	TYPE=SUBSTITUTE
CM032587.1	954608	.	C	T	100	*	TYPE=SUBSTITUTE
CM032587.1	954645	.	C	T	100	*	TYPE=SUBSTITUTE
CM032587.1	954650	.	T	C	100	*	TYPE=SUBSTITUTE
CM032587.1	954734	.	A	G	100	*	TYPE=SUBSTITUTE
CM032587.1	954761	.	T	C	100	*	TYPE=SUBSTITUTE
CM032587.1	954788	.	G	A	100	*	TYPE=SUBSTITUTE
CM032587.1	954792	.	C	T	100	*	TYPE=SUBSTITUTE
CM032587.1	954800	.	C	T	100	*	TYPE=SUBSTITUTE
CM032587.1	954821	.	A	G	100	*	TYPE=SUBSTITUTE
CM032587.1	954863	.	T	C	100	*	TYPE=SUBSTITUTE
CM032587.1	954872	.	G	A	100	*	TYPE=SUBSTITUTE
CM032587.1	954899	.	A	G	100	*	TYPE=SUBSTITUTE
CM032587.1	954926	.	C	T	100	*	TYPE=SUBSTITUTE
CM032587.1	954974	.	A	T	100	*	TYPE=SUBSTITUTE
CM032587.1	955028	.	C	T	100	*	TYPE=SUBSTITUTE
CM032587.1	955043	.	T	C	100	*	TYPE=SUBSTITUTE
CM032587.1	955049	.	A	T	100	*	TYPE=SUBSTITUTE
CM032587.1	955058	.	G	A	100	*	TYPE=SUBSTITUTE
CM032587.1	955061	.	T	G	100	*	TYPE=SUBSTITUTE
CM032587.1	955094	.	C	T	100	*	TYPE=SUBSTITUTE
CM032587.1	955097	.	C	T	100	*	TYPE=SUBSTITUTE
CM032587.1	955151	.	C	G	100	*	TYPE=SUBSTITUTE
CM032587.1	955154	.	C	T	100	*	TYPE=SUBSTITUTE
CM032587.1	955157	.	T	c	100	*	TYPE=SUBSTITUTE
CM032587.1	955223	.	A	G	100	*	TYPE=SUBSTITUTE
CM032587.1	955430	.	T	C	100	*	TYPE=SUBSTITUTE
CM032587.1	955511	.	T	C	100	*	TYPE=SUBSTITUTE
CM032587.1	955625	.	G	C	100	*	TYPE=SUBSTITUTE
CM032587.1	955637	.	T	C	100	*	TYPE=SUBSTITUTE
CM032587.1	955640	.	C	T	100	*	TYPE=SUBSTITUTE
CM032587.1	955667	.	G	A	100	*	TYPE=SUBSTITUTE
CM032587.1	955691	.	T	A	100	*	TYPE=SUBSTITUTE
CM032587.1	956061	.	A	C	100	*	TYPE=SUBSTITUTE
CM032587.1	956064	.	A	t	100	*	TYPE=SUBSTITUTE
CM032587.1	956093	.	AT	A	100	*	TYPE=DELETE
CM032587.1	956136	.	G	A	100	*	TYPE=SUBSTITUTE
CM032587.1	956250	.	CAT	C	100	*	TYPE=DELETE
CM032587.1	957143	.	G	A	100	*	TYPE=SUBSTITUTE

CM032587.1	957272	.	A	g	100	*	TYPE=SUBSTITUTE
CM032587.1	957399	.	C	A	100	*	TYPE=SUBSTITUTE
CM032587.1	957753	.	AA	A	100	*	TYPE=DELETE
CM032587.1	957898	.	TT	T	100	*	TYPE=DELETE
CM032587.1	957922	.	T	TT	100	*	TYPE=INSERT
CM032587.1	958028	.	GG	G	100	*	TYPE=DELETE
CM032587.1	958038	.	AG	A	100	*	TYPE=DELETE
CM032587.1	958046	.	G	C	100	*	TYPE=SUBSTITUTE
CM032587.1	958048	.	A	G	100	*	TYPE=SUBSTITUTE
CM032587.1	958159	.	t	tt	100	*	TYPE=INSERT
CM032587.1	958171	.	C	CT	100	*	TYPE=INSERT
CM032587.1	958208	.	A	G	100	*	TYPE=SUBSTITUTE
CM032587.1	958211	.	G	GA	100	*	TYPE=INSERT
CM032587.1	958212	.	G	C	100	*	TYPE=SUBSTITUTE
CM032587.1	958240	.	TT	T	100	*	TYPE=DELETE
CM032587.1	958304	.	C	T	100	*	TYPE=SUBSTITUTE
CM032587.1	958310	.	T	c	100	*	TYPE=SUBSTITUTE
CM032587.1	958339	.	C	T	100	*	TYPE=SUBSTITUTE
CM032587.1	958397	.	AA	A	100	*	TYPE=DELETE
CM032587.1	958413	.	C	CG	100	*	TYPE=INSERT
CM032587.1	958457	.	A	T	100	*	TYPE=SUBSTITUTE
CM032587.1	958461	.	A	G	100	*	TYPE=SUBSTITUTE
CM032587.1	958470	.	C	CT	100	*	TYPE=INSERT
CM032587.1	958481	.	A	G	100	*	TYPE=SUBSTITUTE
CM032587.1	958491	.	G	GC	100	*	TYPE=INSERT
CM032587.1	958508	.	G	A	100	*	TYPE=SUBSTITUTE
CM032587.1	958510	.	A	T	100	*	TYPE=SUBSTITUTE
CM032587.1	958524	.	G	GGGG	100	*	TYPE=INSERT
CM032587.1	958545	.	CCTGGAAC	C	100	*	TYPE=DELETE
CM032587.1	958561	.	C	A	100	*	TYPE=SUBSTITUTE
CM032587.1	958599	.	T	C	100	*	TYPE=SUBSTITUTE
CM032587.1	958606	.	T	C	100	*	TYPE=SUBSTITUTE
CM032587.1	958640	.	T	TT	100	*	TYPE=INSERT
CM032587.1	958655	.	A	G	100	*	TYPE=SUBSTITUTE
CM032587.1	958657	.	C	CT	100	*	TYPE=INSERT
CM032587.1	958666	.	A	ATACT	100	*	TYPE=INSERT
CM032587.1	958674	.	A	c	100	*	TYPE=SUBSTITUTE
CM032587.1	958683	.	C	t	100	*	TYPE=SUBSTITUTE
CM032587.1	958691	.	C	T	100	*	TYPE=SUBSTITUTE
CM032587.1	958697	.	G	A	100	*	TYPE=SUBSTITUTE
CM032587.1	958706	.	T	C	100	*	TYPE=SUBSTITUTE
CM032587.1	958718	.	C	G	100	*	TYPE=SUBSTITUTE
CM032587.1	958725	.	C	T	100	*	TYPE=SUBSTITUTE
CM032587.1	958734	.	G	A	100	*	TYPE=SUBSTITUTE
CM032587.1	958741	.	ACTTGTTTG	A	100	*	TYPE=DELETE
CM032587.1	958754	.	C	G	100	*	TYPE=SUBSTITUTE
CM032587.1	958755	.	T	C	100	*	TYPE=SUBSTITUTE
CM032587.1	958783	.	C	T	100	*	TYPE=SUBSTITUTE
CM032587.1	958791	.	A	G	100	*	TYPE=SUBSTITUTE
CM032587.1	958804	.	A	T	100	*	TYPE=SUBSTITUTE
CM032587.1	958888	.	GG	G	100	*	TYPE=DELETE
CM032587.1	958947	.	T	TTCGGAGT	100	*	TYPE=INSERT
CM032587.1	958949	.	G	A	100	*	TYPE=SUBSTITUTE
CM032587.1	958964	.	G	A	100	*	TYPE=SUBSTITUTE
CM032587.1	959058	.	T	TT	100	*	TYPE=INSERT
CM032587.1	959059	.	A	G	100	*	TYPE=SUBSTITUTE
CM032587.1	959107	.	TGTACGGAGTATGCGTGTTAGTGTGT	T	100	*	TYPE=DELETE
CM032587.1	959134	.	C	T	100	*	TYPE=SUBSTITUTE
CM032587.1	959140	.	GC	G	100	*	TYPE=DELETE
CM032587.1	959144	.	A	G	100	*	TYPE=SUBSTITUTE
CM032587.1	959156	.	TG	T	100	*	TYPE=DELETE
CM032587.1	959180	.	A	G	100	*	TYPE=SUBSTITUTE
CM032587.1	959217	.	T	G	100	*	TYPE=SUBSTITUTE

CM032587.1	959266	.	GG	G	100	*	TYPE=DELETE
CM032587.1	959272	.	A	g	100	*	TYPE=SUBSTITUTE
CM032587.1	959302	.	G	T	100	*	TYPE=SUBSTITUTE
CM032587.1	959303	.	C	T	100	*	TYPE=SUBSTITUTE
CM032587.1	959307	.	AG	A	100	*	TYPE=DELETE
CM032587.1	959313	.	G	T	100	*	TYPE=SUBSTITUTE
CM032587.1	959319	.	GA	G	100	*	TYPE=DELETE
CM032587.1	959328	.	G	GTA	100	*	TYPE=INSERT
CM032587.1	959366	.	ATGGATTCGAT	A	100	*	TYPE=DELETE
CM032587.1	959380	.	TG	T	100	*	TYPE=DELETE
CM032587.1	959432	.	T	C	100	*	TYPE=SUBSTITUTE
CM032587.1	959437	.	T	C	100	*	TYPE=SUBSTITUTE
CM032587.1	959461	.	G	T	100	*	TYPE=SUBSTITUTE
CM032587.1	959541	.	T	A	100	*	TYPE=SUBSTITUTE
CM032587.1	959651	.	T	A	100	*	TYPE=SUBSTITUTE
CM032587.1	959658	.	TG	T	100	*	TYPE=DELETE
CM032587.1	959678	.	A	ACAGTA	100	*	TYPE=INSERT
CM032587.1	959684	.	CA	C	100	*	TYPE=DELETE
CM032587.1	959720	.	C	T	100	*	TYPE=SUBSTITUTE
CM032587.1	959730	.	AG	A	100	*	TYPE=DELETE
CM032587.1	959736	.	A	T	100	*	TYPE=SUBSTITUTE
CM032587.1	959745	.	G	A	100	*	TYPE=SUBSTITUTE
CM032587.1	959773	.	T	C	100	*	TYPE=SUBSTITUTE
CM032587.1	959801	.	G	A	100	*	TYPE=SUBSTITUTE
CM032587.1	959844	.	T	C	100	*	TYPE=SUBSTITUTE
CM032587.1	959864	.	A	G	100	*	TYPE=SUBSTITUTE
CM032587.1	959882	.	C	G	100	*	TYPE=SUBSTITUTE
CM032587.1	959885	.	A	G	100	*	TYPE=SUBSTITUTE
CM032587.1	959895	.	C	CT	100	*	TYPE=INSERT
CM032587.1	959899	.	C	G	100	*	TYPE=SUBSTITUTE
CM032587.1	959902	.	G	T	100	*	TYPE=SUBSTITUTE
CM032587.1	959913	.	CT	C	100	*	TYPE=DELETE
CM032587.1	960026	.	A	g	100	*	TYPE=SUBSTITUTE
CM032587.1	960029	.	G	a	100	*	TYPE=SUBSTITUTE
CM032587.1	960031	.	T	a	100	*	TYPE=SUBSTITUTE
CM032587.1	960057	.	A	C	100	*	TYPE=SUBSTITUTE
CM032587.1	960079	.	C	G	100	*	TYPE=SUBSTITUTE
CM032587.1	960093	.	G	A	100	*	TYPE=SUBSTITUTE
CM032587.1	960099	.	C	T	100	*	TYPE=SUBSTITUTE
CM032587.1	960100	.	A	G	100	*	TYPE=SUBSTITUTE
CM032587.1	960119	.	G	A	100	*	TYPE=SUBSTITUTE
CM032587.1	960185	.	G	A	100	*	TYPE=SUBSTITUTE
CM032587.1	960192	.	T	A	100	*	TYPE=SUBSTITUTE
CM032587.1	960234	.	C	G	100	*	TYPE=SUBSTITUTE
CM032587.1	960235	.	C	G	100	*	TYPE=SUBSTITUTE
CM032587.1	960363	.	G	A	100	*	TYPE=SUBSTITUTE
CM032587.1	960366	.	G	A	100	*	TYPE=SUBSTITUTE
CM032587.1	960367	.	G	A	100	*	TYPE=SUBSTITUTE
CM032587.1	960417	.	C	T	100	*	TYPE=SUBSTITUTE
CM032587.1	960532	.	G	A	100	*	TYPE=SUBSTITUTE
CM032587.1	960570	.	A	G	100	*	TYPE=SUBSTITUTE
CM032587.1	960612	.	A	G	100	*	TYPE=SUBSTITUTE
CM032587.1	960642	.	C	G	100	*	TYPE=SUBSTITUTE
CM032587.1	960643	.	C	T	100	*	TYPE=SUBSTITUTE
CM032587.1	960658	.	A	C	100	*	TYPE=SUBSTITUTE
CM032587.1	960680	.	C	T	100	*	TYPE=SUBSTITUTE
CM032587.1	961001	.	G	A	100	*	TYPE=SUBSTITUTE
CM032587.1	961047	.	TT	T	100	*	TYPE=DELETE
CM032587.1	961054	.	GC	G	100	*	TYPE=DELETE
CM032587.1	961057	.	G	A	100	*	TYPE=SUBSTITUTE
CM032587.1	961064	.	C	T	100	*	TYPE=SUBSTITUTE
CM032587.1	961071	.	T	G	100	*	TYPE=SUBSTITUTE
CM032587.1	961101	.	A	G	100	*	TYPE=SUBSTITUTE
CM032587.1	961130	.	G	A	100	*	TYPE=SUBSTITUTE

CM032587.1	961139	.	G	C	100	*	TYPE=SUBSTITUTE
CM032587.1	961166	.	G	GTG	100	*	TYPE=INSERT
CM032587.1	961168	.	C	T	100	*	TYPE=SUBSTITUTE
CM032587.1	961171	.	T	C	100	*	TYPE=SUBSTITUTE
CM032587.1	961201	.	T	TT	100	*	TYPE=INSERT
CM032587.1	961231	.	G	A	100	*	TYPE=SUBSTITUTE
CM032587.1	961233	.	T	G	100	*	TYPE=SUBSTITUTE
CM032587.1	961237	.	T	G	100	*	TYPE=SUBSTITUTE
CM032587.1	961239	.	GTAT	G	100	*	TYPE=DELETE
CM032587.1	961287	.	A	T	100	*	TYPE=SUBSTITUTE
CM032587.1	961288	.	C	G	100	*	TYPE=SUBSTITUTE
CM032587.1	961289	.	G	C	100	*	TYPE=SUBSTITUTE
CM032587.1	961299	.	C	A	100	*	TYPE=SUBSTITUTE
CM032587.1	961304	.	AC	A	100	*	TYPE=DELETE
CM032587.1	961312	.	A	G	100	*	TYPE=SUBSTITUTE
CM032587.1	961327	.	C	T	100	*	TYPE=SUBSTITUTE
CM032587.1	961348	.	A	T	100	*	TYPE=SUBSTITUTE
CM032587.1	961368	.	A	C	100	*	TYPE=SUBSTITUTE
CM032587.1	961419	.	A	G	100	*	TYPE=SUBSTITUTE
CM032587.1	961434	.	A	G	100	*	TYPE=SUBSTITUTE
CM032587.1	961522	.	TT	T	100	*	TYPE=DELETE
CM032587.1	961564	.	C	t	100	*	TYPE=SUBSTITUTE
CM032587.1	961569	.	GA	G	100	*	TYPE=DELETE
CM032587.1	961579	.	G	t	100	*	TYPE=SUBSTITUTE
CM032587.1	961581	.	T	c	100	*	TYPE=SUBSTITUTE
CM032587.1	961627	.	C	a	100	*	TYPE=SUBSTITUTE
CM032587.1	961682	.	GAG	G	100	*	TYPE=DELETE
CM032587.1	961760	.	T	A	100	*	TYPE=SUBSTITUTE
CM032587.1	961762	.	A	C	100	*	TYPE=SUBSTITUTE
CM032587.1	961763	.	G	GT	100	*	TYPE=INSERT
CM032587.1	961857	.	T	G	100	*	TYPE=SUBSTITUTE
CM032587.1	961861	.	C	T	100	*	TYPE=SUBSTITUTE
CM032587.1	961882	.	T	C	100	*	TYPE=SUBSTITUTE
CM032587.1	961897	.	A	T	100	*	TYPE=SUBSTITUTE
CM032587.1	961922	.	T	C	100	*	TYPE=SUBSTITUTE
CM032587.1	962027	.	A	G	100	*	TYPE=SUBSTITUTE
CM032587.1	962092	.	G	A	100	*	TYPE=SUBSTITUTE
CM032587.1	962116	.	T	c	100	*	TYPE=SUBSTITUTE
CM032587.1	962120	.	GT	G	100	*	TYPE=DELETE
CM032587.1	962127	.	A	g	100	*	TYPE=SUBSTITUTE
CM032587.1	962146	.	G	A	100	*	TYPE=SUBSTITUTE
CM032587.1	962228	.	T	C	100	*	TYPE=SUBSTITUTE
CM032587.1	962295	.	C	T	100	*	TYPE=SUBSTITUTE
CM032587.1	962303	.	C	T	100	*	TYPE=SUBSTITUTE
CM032587.1	962307	.	A	T	100	*	TYPE=SUBSTITUTE
CM032587.1	962310	.	G	C	100	*	TYPE=SUBSTITUTE
CM032587.1	962312	.	A	G	100	*	TYPE=SUBSTITUTE
CM032587.1	962316	.	A	G	100	*	TYPE=SUBSTITUTE
CM032587.1	962325	.	C	T	100	*	TYPE=SUBSTITUTE
CM032587.1	962328	.	C	T	100	*	TYPE=SUBSTITUTE
CM032587.1	962342	.	G	A	100	*	TYPE=SUBSTITUTE
CM032587.1	962344	.	C	A	100	*	TYPE=SUBSTITUTE
CM032587.1	962348	.	A	G	100	*	TYPE=SUBSTITUTE
CM032587.1	962594	.	C	T	100	*	TYPE=SUBSTITUTE
CM032587.1	962600	.	A	G	100	*	TYPE=SUBSTITUTE
CM032587.1	962646	.	T	C	100	*	TYPE=SUBSTITUTE
CM032587.1	962694	.	T	C	100	*	TYPE=SUBSTITUTE
CM032587.1	962700	.	T	C	100	*	TYPE=SUBSTITUTE
CM032587.1	962714	.	C	T	100	*	TYPE=SUBSTITUTE
CM032587.1	962718	.	C	T	100	*	TYPE=SUBSTITUTE
CM032587.1	962732	.	C	G	100	*	TYPE=SUBSTITUTE
CM032587.1	962776	.	G	T	100	*	TYPE=SUBSTITUTE
CM032587.1	962778	.	A	G	100	*	TYPE=SUBSTITUTE
CM032587.1	962835	.	T	A	100	*	TYPE=SUBSTITUTE

CM032587.1	962847	.	T	C	100	*	TYPE=SUBSTITUTE
CM032587.1	962855	.	AG	A	100	*	TYPE=DELETE
CM032587.1	962857	.	T	A	100	*	TYPE=SUBSTITUTE
CM032587.1	962878	.	T	C	100	*	TYPE=SUBSTITUTE
CM032587.1	962880	.	C	A	100	*	TYPE=SUBSTITUTE
CM032587.1	962881	.	A	G	100	*	TYPE=SUBSTITUTE
CM032587.1	962882	.	T	C	100	*	TYPE=SUBSTITUTE
CM032587.1	962886	.	T	A	100	*	TYPE=SUBSTITUTE
CM032587.1	962900	.	T	C	100	*	TYPE=SUBSTITUTE
CM032587.1	962901	.	T	A	100	*	TYPE=SUBSTITUTE
CM032587.1	962902	.	A	G	100	*	TYPE=SUBSTITUTE
CM032587.1	962995	.	G	GTC	100	*	TYPE=INSERT
CM032587.1	962996	.	C	G	100	*	TYPE=SUBSTITUTE
CM032587.1	962997	.	A	T	100	*	TYPE=SUBSTITUTE
CM032587.1	963017	.	A	G	100	*	TYPE=SUBSTITUTE
CM032587.1	963023	.	C	A	100	*	TYPE=SUBSTITUTE
CM032587.1	963024	.	C	T	100	*	TYPE=SUBSTITUTE
CM032587.1	963112	.	T	G	100	*	TYPE=SUBSTITUTE
CM032587.1	963184	.	A	G	100	*	TYPE=SUBSTITUTE
CM032587.1	963202	.	A	T	100	*	TYPE=SUBSTITUTE
CM032587.1	963241	.	C	T	100	*	TYPE=SUBSTITUTE
CM032587.1	963252	.	G	A	100	*	TYPE=SUBSTITUTE
CM032587.1	963259	.	A	T	100	*	TYPE=SUBSTITUTE
CM032587.1	963288	.	C	G	100	*	TYPE=SUBSTITUTE
CM032587.1	963316	.	A	G	100	*	TYPE=SUBSTITUTE
CM032587.1	963322	.	C	T	100	*	TYPE=SUBSTITUTE
CM032587.1	963325	.	C	G	100	*	TYPE=SUBSTITUTE
CM032587.1	963368	.	C	T	100	*	TYPE=SUBSTITUTE
CM032587.1	963382	.	C	T	100	*	TYPE=SUBSTITUTE
CM032587.1	963417	.	AA	A	100	*	TYPE=DELETE
CM032587.1	963420	.	C	A	100	*	TYPE=SUBSTITUTE
CM032587.1	963429	.	C	T	100	*	TYPE=SUBSTITUTE
CM032587.1	963434	.	T	C	100	*	TYPE=SUBSTITUTE
CM032587.1	1568511	.	A	G	100	*	TYPE=SUBSTITUTE
CM032587.1	1568531	.	C	T	100	*	TYPE=SUBSTITUTE
CM032587.1	1568536	.	G	A	100	*	TYPE=SUBSTITUTE
CM032587.1	1568537	.	C	G	100	*	TYPE=SUBSTITUTE
CM032587.1	1568562	.	A	C	100	*	TYPE=SUBSTITUTE
CM032587.1	1568586	.	G	A	100	*	TYPE=SUBSTITUTE
CM032587.1	1568592	.	A	G	100	*	TYPE=SUBSTITUTE
CM032587.1	1568594	.	G	A	100	*	TYPE=SUBSTITUTE
CM032587.1	1568622	.	T	C	100	*	TYPE=SUBSTITUTE
CM032587.1	1568627	.	G	A	100	*	TYPE=SUBSTITUTE
CM032587.1	1568629	.	G	A	100	*	TYPE=SUBSTITUTE
CM032587.1	1568631	.	A	G	100	*	TYPE=SUBSTITUTE
CM032587.1	1568649	.	G	A	100	*	TYPE=SUBSTITUTE
CM032587.1	1568652	.	C	T	100	*	TYPE=SUBSTITUTE
CM032587.1	1568715	.	T	C	100	*	TYPE=SUBSTITUTE
CM032587.1	1568717	.	A	C	100	*	TYPE=SUBSTITUTE
CM032587.1	1568745	.	T	C	100	*	TYPE=SUBSTITUTE
CM032587.1	1568752	.	G	C	100	*	TYPE=SUBSTITUTE
CM032587.1	1568769	.	CGAAACA	C	100	*	TYPE=DELETE
CM032587.1	1568773	.	G	T	100	*	TYPE=SUBSTITUTE
CM032587.1	1568781	.	G	A	100	*	TYPE=SUBSTITUTE
CM032587.1	1568818	.	T	C	100	*	TYPE=SUBSTITUTE
CM032587.1	1568837	.	C	A	100	*	TYPE=SUBSTITUTE
CM032587.1	1568844	.	T	G	100	*	TYPE=SUBSTITUTE
CM032587.1	1568849	.	G	T	100	*	TYPE=SUBSTITUTE
CM032587.1	1568861	.	G	A	100	*	TYPE=SUBSTITUTE
CM032587.1	1568865	.	T	c	100	*	TYPE=SUBSTITUTE
CM032587.1	1568891	.	C	A	100	*	TYPE=SUBSTITUTE
CM032587.1	1568911	.	G	A	100	*	TYPE=SUBSTITUTE
CM032587.1	1568915	.	A	G	100	*	TYPE=SUBSTITUTE
CM032587.1	1568921	.	A	G	100	*	TYPE=SUBSTITUTE

CM032587.1	1568927	.	G	A	100	*	TYPE=SUBSTITUTE
CM032587.1	1568930	.	G	A	100	*	TYPE=SUBSTITUTE
CM032587.1	1568932	.	C	A	100	*	TYPE=SUBSTITUTE
CM032587.1	1568935	.	A	G	100	*	TYPE=SUBSTITUTE
CM032587.1	1568939	.	A	G	100	*	TYPE=SUBSTITUTE
CM032587.1	1568940	.	A	G	100	*	TYPE=SUBSTITUTE
CM032587.1	1568942	.	T	C	100	*	TYPE=SUBSTITUTE
CM032587.1	1568944	.	C	A	100	*	TYPE=SUBSTITUTE
CM032587.1	1568948	.	C	A	100	*	TYPE=SUBSTITUTE
CM032587.1	1568949	.	C	A	100	*	TYPE=SUBSTITUTE
CM032587.1	1568953	.	CCTT	C	100	*	TYPE=DELETE
CM032587.1	1568964	.	CTG	C	100	*	TYPE=DELETE
CM032587.1	1568970	.	GGCGT	G	100	*	TYPE=DELETE
CM032587.1	1568976	.	CAGG	C	100	*	TYPE=DELETE
CM032587.1	1568977	.	C	T	100	*	TYPE=SUBSTITUTE
CM032587.1	1568980	.	C	G	100	*	TYPE=SUBSTITUTE
CM032587.1	1568985	.	C	A	100	*	TYPE=SUBSTITUTE
CM032587.1	1568986	.	G	A	100	*	TYPE=SUBSTITUTE
CM032587.1	1568990	.	A	G	100	*	TYPE=SUBSTITUTE
CM032587.1	1569025	.	C	G	100	*	TYPE=SUBSTITUTE
CM032587.1	1569045	.	C	T	100	*	TYPE=SUBSTITUTE
CM032587.1	1569055	.	G	A	100	*	TYPE=SUBSTITUTE
CM032587.1	1569061	.	C	G	100	*	TYPE=SUBSTITUTE
CM032587.1	1569067	.	T	C	100	*	TYPE=SUBSTITUTE
CM032587.1	1569073	.	C	A	100	*	TYPE=SUBSTITUTE
CM032587.1	1569078	.	G	C	100	*	TYPE=SUBSTITUTE
CM032587.1	1569079	.	G	A	100	*	TYPE=SUBSTITUTE
CM032587.1	1569090	.	G	A	100	*	TYPE=SUBSTITUTE
CM032587.1	1569112	.	G	A	100	*	TYPE=SUBSTITUTE
CM032587.1	1569126	.	C	T	100	*	TYPE=SUBSTITUTE
CM032587.1	1569132	.	C	T	100	*	TYPE=SUBSTITUTE
CM032587.1	1569136	.	G	a	100	*	TYPE=SUBSTITUTE
CM032587.1	1569144	.	C	a	100	*	TYPE=SUBSTITUTE
CM032587.1	1569145	.	T	a	100	*	TYPE=SUBSTITUTE
CM032587.1	1569148	.	A	g	100	*	TYPE=SUBSTITUTE
CM032587.1	1569151	.	C	t	100	*	TYPE=SUBSTITUTE
CM032587.1	1569152	.	C	a	100	*	TYPE=SUBSTITUTE
CM032587.1	1569153	.	T	g	100	*	TYPE=SUBSTITUTE
CM032587.1	1569154	.	T	a	100	*	TYPE=SUBSTITUTE
CM032587.1	1569155	.	T	C	100	*	TYPE=SUBSTITUTE
CM032587.1	1569156	.	G	A	100	*	TYPE=SUBSTITUTE
CM032587.1	1569157	.	A	C	100	*	TYPE=SUBSTITUTE
CM032587.1	1569158	.	G	A	100	*	TYPE=SUBSTITUTE
CM032587.1	1569163	.	T	C	100	*	TYPE=SUBSTITUTE
CM032587.1	1569164	.	G	A	100	*	TYPE=SUBSTITUTE
CM032587.1	1569165	.	G	A	100	*	TYPE=SUBSTITUTE
CM032587.1	1569169	.	G	T	100	*	TYPE=SUBSTITUTE
CM032587.1	1569172	.	A	G	100	*	TYPE=SUBSTITUTE
CM032587.1	1569177	.	AA	A	100	*	TYPE=DELETE
CM032587.1	1569211	.	T	C	100	*	TYPE=SUBSTITUTE
CM032587.1	1569225	.	G	T	100	*	TYPE=SUBSTITUTE
CM032587.1	1569245	.	A	C	100	*	TYPE=SUBSTITUTE
CM032587.1	1569246	.	G	T	100	*	TYPE=SUBSTITUTE
CM032587.1	1569259	.	C	T	100	*	TYPE=SUBSTITUTE
CM032587.1	1569283	.	C	G	100	*	TYPE=SUBSTITUTE
CM032587.1	1569292	.	A	G	100	*	TYPE=SUBSTITUTE
CM032587.1	1569294	.	G	A	100	*	TYPE=SUBSTITUTE
CM032587.1	1569300	.	A	c	100	*	TYPE=SUBSTITUTE
CM032587.1	1569301	.	T	a	100	*	TYPE=SUBSTITUTE
CM032587.1	1569311	.	T	c	100	*	TYPE=SUBSTITUTE
CM032587.1	1569314	.	G	c	100	*	TYPE=SUBSTITUTE
CM032587.1	1569319	.	A	G	100	*	TYPE=SUBSTITUTE
CM032587.1	1569326	.	A	G	100	*	TYPE=SUBSTITUTE
CM032587.1	1569327	.	A	C	100	*	TYPE=SUBSTITUTE

CM032587.1	1569362	.	G	A	100	*	TYPE=SUBSTITUTE
CM032587.1	1569372	.	G	C	100	*	TYPE=SUBSTITUTE
CM032587.1	1569374	.	A	T	100	*	TYPE=SUBSTITUTE
CM032587.1	1569377	.	C	A	100	*	TYPE=SUBSTITUTE
CM032587.1	1569385	.	C	G	100	*	TYPE=SUBSTITUTE
CM032587.1	1569386	.	A	G	100	*	TYPE=SUBSTITUTE
CM032587.1	1569387	.	G	T	100	*	TYPE=SUBSTITUTE
CM032587.1	1569402	.	C	A	100	*	TYPE=SUBSTITUTE
CM032587.1	1569404	.	C	G	100	*	TYPE=SUBSTITUTE
CM032587.1	1569411	.	T	A	100	*	TYPE=SUBSTITUTE
CM032587.1	1569415	.	G	GGGTACT	100	*	TYPE=INSERT
CM032587.1	1569424	.	C	T	100	*	TYPE=SUBSTITUTE
CM032587.1	1569469	.	A	G	100	*	TYPE=SUBSTITUTE
CM032587.1	1569486	.	A	T	100	*	TYPE=SUBSTITUTE
CM032587.1	1569501	.	G	A	100	*	TYPE=SUBSTITUTE
CM032587.1	1569521	.	C	T	100	*	TYPE=SUBSTITUTE
CM032587.1	1569523	.	T	C	100	*	TYPE=SUBSTITUTE
CM032587.1	1569559	.	C	T	100	*	TYPE=SUBSTITUTE
CM032587.1	1569560	.	G	C	100	*	TYPE=SUBSTITUTE
CM032587.1	1569562	.	T	C	100	*	TYPE=SUBSTITUTE
CM032587.1	1569565	.	G	C	100	*	TYPE=SUBSTITUTE
CM032587.1	1569581	.	A	G	100	*	TYPE=SUBSTITUTE
CM032587.1	1569584	.	C	T	100	*	TYPE=SUBSTITUTE
CM032587.1	1569603	.	A	C	100	*	TYPE=SUBSTITUTE
CM032587.1	1569615	.	G	A	100	*	TYPE=SUBSTITUTE
CM032587.1	1569616	.	C	T	100	*	TYPE=SUBSTITUTE
CM032587.1	1569617	.	C	T	100	*	TYPE=SUBSTITUTE
CM032587.1	1569623	.	G	C	100	*	TYPE=SUBSTITUTE
CM032587.1	1569635	.	G	A	100	*	TYPE=SUBSTITUTE
CM032587.1	1569636	.	A	G	100	*	TYPE=SUBSTITUTE
CM032587.1	1569638	.	G	C	100	*	TYPE=SUBSTITUTE
CM032587.1	1569647	.	G	A	100	*	TYPE=SUBSTITUTE
CM032587.1	1569655	.	C	T	100	*	TYPE=SUBSTITUTE
CM032587.1	1569660	.	C	G	100	*	TYPE=SUBSTITUTE
CM032587.1	1569675	.	C	T	100	*	TYPE=SUBSTITUTE
CM032587.1	1569682	.	C	T	100	*	TYPE=SUBSTITUTE
CM032587.1	1569691	.	C	T	100	*	TYPE=SUBSTITUTE
CM032587.1	1569706	.	C	A	100	*	TYPE=SUBSTITUTE
CM032587.1	1569733	.	C	CGACGATTTATCACTGCAAGTATCTTGTCTC	100	*	TYPE=INSERT
CM032587.1	1569748	.	G	A	100	*	TYPE=SUBSTITUTE
CM032587.1	1569785	.	G	T	100	*	TYPE=SUBSTITUTE
CM032587.1	1569817	.	T	C	100	*	TYPE=SUBSTITUTE
CM032587.1	1569836	.	G	A	100	*	TYPE=SUBSTITUTE
CM032587.1	1569837	.	T	C	100	*	TYPE=SUBSTITUTE
CM032587.1	1569839	.	G	A	100	*	TYPE=SUBSTITUTE
CM032587.1	1569846	.	C	G	100	*	TYPE=SUBSTITUTE
CM032587.1	1569849	.	A	G	100	*	TYPE=SUBSTITUTE
CM032587.1	1569859	.	T	G	100	*	TYPE=SUBSTITUTE
CM032587.1	1569860	.	T	G	100	*	TYPE=SUBSTITUTE
CM032587.1	1569883	.	C	T	100	*	TYPE=SUBSTITUTE
CM032587.1	1569885	.	T	A	100	*	TYPE=SUBSTITUTE
CM032587.1	1569894	.	T	C	100	*	TYPE=SUBSTITUTE
CM032587.1	1569899	.	GACC	G	100	*	TYPE=DELETE
CM032587.1	1569931	.	G	A	100	*	TYPE=SUBSTITUTE
CM032587.1	1569938	.	G	T	100	*	TYPE=SUBSTITUTE
CM032587.1	1569950	.	A	G	100	*	TYPE=SUBSTITUTE
CM032587.1	1569972	.	A	C	100	*	TYPE=SUBSTITUTE
CM032587.1	1569973	.	T	C	100	*	TYPE=SUBSTITUTE
CM032587.1	1569976	.	A	G	100	*	TYPE=SUBSTITUTE
CM032587.1	1570012	.	A	C	100	*	TYPE=SUBSTITUTE
CM032587.1	1570022	.	T	c	100	*	TYPE=SUBSTITUTE
CM032587.1	1570060	.	C	T	100	*	TYPE=SUBSTITUTE
CM032587.1	1570101	.	A	C	100	*	TYPE=SUBSTITUTE

CM032587.1	1570118	.	T	G	100	*	TYPE=SUBSTITUTE
CM032587.1	1570141	.	C	G	100	*	TYPE=SUBSTITUTE
CM032587.1	1570156	.	G	A	100	*	TYPE=SUBSTITUTE
CM032587.1	1570159	.	G	A	100	*	TYPE=SUBSTITUTE
CM032587.1	1570207	.	AAGGTTCGTGCTCGAT	A	100	*	TYPE=DELETE
CM032587.1	1570210	.	G	A	100	*	TYPE=SUBSTITUTE
CM032587.1	1570212	.	A	G	100	*	TYPE=SUBSTITUTE
CM032587.1	1570220	.	T	C	100	*	TYPE=SUBSTITUTE
CM032587.1	1570223	.	C	CTA	100	*	TYPE=INSERT
CM032587.1	1570228	.	g	gata	100	*	TYPE=INSERT
CM032587.1	1570232	.	A	g	100	*	TYPE=SUBSTITUTE
CM032587.1	1570273	.	T	C	100	*	TYPE=SUBSTITUTE
CM032587.1	1570290	.	T	A	100	*	TYPE=SUBSTITUTE
CM032587.1	1570291	.	C	CAGGT	100	*	TYPE=INSERT
CM032587.1	1570293	.	A	G	100	*	TYPE=SUBSTITUTE
CM032587.1	1570307	.	A	G	100	*	TYPE=SUBSTITUTE
CM032587.1	1570310	.	T	C	100	*	TYPE=SUBSTITUTE
CM032587.1	1570324	.	C	T	100	*	TYPE=SUBSTITUTE
CM032587.1	1570343	.	A	G	100	*	TYPE=SUBSTITUTE
CM032587.1	1570344	.	T	C	100	*	TYPE=SUBSTITUTE
CM032587.1	1570353	.	A	g	100	*	TYPE=SUBSTITUTE
CM032587.1	1570372	.	T	C	100	*	TYPE=SUBSTITUTE
CM032587.1	1570373	.	G	A	100	*	TYPE=SUBSTITUTE
CM032587.1	1570406	.	A	T	100	*	TYPE=SUBSTITUTE
CM032587.1	1570407	.	A	G	100	*	TYPE=SUBSTITUTE
CM032587.1	1570411	.	A	C	100	*	TYPE=SUBSTITUTE
CM032587.1	1570414	.	A	C	100	*	TYPE=SUBSTITUTE
CM032587.1	1570418	.	G	C	100	*	TYPE=SUBSTITUTE
CM032587.1	1570461	.	G	T	100	*	TYPE=SUBSTITUTE
CM032587.1	1570463	.	A	G	100	*	TYPE=SUBSTITUTE
CM032587.1	1570468	.	T	C	100	*	TYPE=SUBSTITUTE
CM032587.1	1570478	.	A	C	100	*	TYPE=SUBSTITUTE
CM032587.1	1570481	.	T	C	100	*	TYPE=SUBSTITUTE
CM032587.1	1570486	.	A	C	100	*	TYPE=SUBSTITUTE
CM032587.1	1570495	.	A	G	100	*	TYPE=SUBSTITUTE
CM032587.1	1570496	.	A	T	100	*	TYPE=SUBSTITUTE
CM032587.1	1570500	.	C	G	100	*	TYPE=SUBSTITUTE
CM032587.1	1570521	.	A	G	100	*	TYPE=SUBSTITUTE
CM032587.1	1570535	.	C	A	100	*	TYPE=SUBSTITUTE
CM032587.1	1570539	.	A	G	100	*	TYPE=SUBSTITUTE
CM032587.1	1570541	.	T	C	100	*	TYPE=SUBSTITUTE
CM032587.1	1570552	.	T	TCT	100	*	TYPE=INSERT
CM032587.1	1570572	.	A	G	100	*	TYPE=SUBSTITUTE
CM032587.1	1570574	.	C	T	100	*	TYPE=SUBSTITUTE
CM032587.1	1570576	.	C	T	100	*	TYPE=SUBSTITUTE
CM032587.1	1570581	.	C	A	100	*	TYPE=SUBSTITUTE
CM032587.1	1570594	.	G	T	100	*	TYPE=SUBSTITUTE
CM032587.1	1570598	.	A	G	100	*	TYPE=SUBSTITUTE
CM032587.1	1570611	.	G	T	100	*	TYPE=SUBSTITUTE
CM032587.1	1570615	.	T	C	100	*	TYPE=SUBSTITUTE
CM032587.1	1570633	.	C	T	100	*	TYPE=SUBSTITUTE
CM032587.1	1570649	.	C	A	100	*	TYPE=SUBSTITUTE
CM032587.1	1570656	.	T	C	100	*	TYPE=SUBSTITUTE
CM032587.1	1570660	.	A	G	100	*	TYPE=SUBSTITUTE
CM032587.1	1570682	.	A	G	100	*	TYPE=SUBSTITUTE
CM032587.1	1570685	.	A	G	100	*	TYPE=SUBSTITUTE
CM032587.1	1570689	.	G	A	100	*	TYPE=SUBSTITUTE
CM032587.1	1570690	.	T	C	100	*	TYPE=SUBSTITUTE
CM032587.1	1570691	.	A	G	100	*	TYPE=SUBSTITUTE
CM032587.1	1570693	.	G	T	100	*	TYPE=SUBSTITUTE
CM032587.1	1570708	.	T	C	100	*	TYPE=SUBSTITUTE
CM032587.1	1570710	.	C	T	100	*	TYPE=SUBSTITUTE
CM032587.1	1570712	.	T	C	100	*	TYPE=SUBSTITUTE
CM032587.1	1570713	.	A	G	100	*	TYPE=SUBSTITUTE

CM032587.1 1570714 . C T 100 * TYPE=SUBSTITUTE
CM032587.1 1570720 . A G 100 * TYPE=SUBSTITUTE
CM032587.1 1570765 . GAGGCATCCTCAATGTGGGAAGACGGGGCCA G 100 *
TYPE=DELETE
CM032587.1 1570768 . G T 100 * TYPE=SUBSTITUTE
CM032587.1 1570772 . G A 100 * TYPE=SUBSTITUTE
CM032587.1 1570778 . T C 100 * TYPE=SUBSTITUTE
CM032587.1 1570790 . T G 100 * TYPE=SUBSTITUTE
CM032587.1 1570793 . G A 100 * TYPE=SUBSTITUTE
CM032587.1 1570806 . C G 100 * TYPE=SUBSTITUTE
CM032587.1 1570812 . C T 100 * TYPE=SUBSTITUTE
CM032587.1 1570832 . A T 100 * TYPE=SUBSTITUTE
CM032587.1 1570833 . C G 100 * TYPE=SUBSTITUTE
CM032587.1 1570842 . A T 100 * TYPE=SUBSTITUTE
CM032587.1 1570843 . A G 100 * TYPE=SUBSTITUTE
CM032587.1 1570902 . CC C 100 * TYPE=DELETE
CM032587.1 1570937 . T A 100 * TYPE=SUBSTITUTE
CM032587.1 1570941 . G A 100 * TYPE=SUBSTITUTE
CM032587.1 1570959 . T C 100 * TYPE=SUBSTITUTE
CM032587.1 1571010 . G C 100 * TYPE=SUBSTITUTE
CM032587.1 1571011 . G A 100 * TYPE=SUBSTITUTE
CM032587.1 1571015 . T A 100 * TYPE=SUBSTITUTE
CM032587.1 1571042 . G a 100 * TYPE=SUBSTITUTE
CM032587.1 1571044 . A g 100 * TYPE=SUBSTITUTE
CM032587.1 1571046 . T c 100 * TYPE=SUBSTITUTE
CM032587.1 1571081 . G T 100 * TYPE=SUBSTITUTE
CM032587.1 1571089 . A C 100 * TYPE=SUBSTITUTE
CM032587.1 1571090 . C CCAGTAT 100 * TYPE=INSERT
CM032587.1 1571091 . T C 100 * TYPE=SUBSTITUTE
CM032587.1 1571095 . A C 100 * TYPE=SUBSTITUTE
CM032587.1 1571097 . G T 100 * TYPE=SUBSTITUTE
CM032587.1 1571098 . C CT 100 * TYPE=INSERT
CM032587.1 1571121 . C T 100 * TYPE=SUBSTITUTE
CM032587.1 1571124 . A C 100 * TYPE=SUBSTITUTE
CM032587.1 1571127 . AT A 100 * TYPE=DELETE
CM032587.1 1571130 . G T 100 * TYPE=SUBSTITUTE
CM032587.1 1571137 . C T 100 * TYPE=SUBSTITUTE
CM032587.1 1571171 . A G 100 * TYPE=SUBSTITUTE
CM032587.1 1571175 . G A 100 * TYPE=SUBSTITUTE
CM032587.1 1571178 . G A 100 * TYPE=SUBSTITUTE
CM032587.1 1571184 . A T 100 * TYPE=SUBSTITUTE
CM032587.1 1571197 . A G 100 * TYPE=SUBSTITUTE
CM032587.1 1571218 . CATGG C 100 * TYPE=DELETE
CM032587.1 1571225 . G A 100 * TYPE=SUBSTITUTE
CM032587.1 1571233 . C T 100 * TYPE=SUBSTITUTE
CM032587.1 1571235 . T C 100 * TYPE=SUBSTITUTE
CM032587.1 1571242 . AG A 100 * TYPE=DELETE
CM032587.1 1571247 . C G 100 * TYPE=SUBSTITUTE
CM032587.1 1571248 . C A 100 * TYPE=SUBSTITUTE
CM032587.1 1571249 . A AG 100 * TYPE=INSERT
CM032587.1 1571253 . A T 100 * TYPE=SUBSTITUTE
CM032587.1 1571256 . G C 100 * TYPE=SUBSTITUTE
CM032587.1 1571257 . A T 100 * TYPE=SUBSTITUTE
CM032587.1 1571258 . T C 100 * TYPE=SUBSTITUTE
CM032587.1 1571259 . G C 100 * TYPE=SUBSTITUTE
CM032587.1 1571260 . G A 100 * TYPE=SUBSTITUTE
CM032587.1 1571264 . G T 100 * TYPE=SUBSTITUTE
CM032587.1 1571277 . TGAG T 100 * TYPE=DELETE
CM032587.1 1571311 . G GTTTGCA 100 * TYPE=INSERT
CM032587.1 1571314 . A G 100 * TYPE=SUBSTITUTE
CM032587.1 1571315 . C T 100 * TYPE=SUBSTITUTE
CM032587.1 1571328 . A G 100 * TYPE=SUBSTITUTE
CM032587.1 1571336 . A G 100 * TYPE=SUBSTITUTE
CM032587.1 1571344 . T TAA 100 * TYPE=INSERT

CM032587.1	1571346	.	T	TATG	100	*	TYPE=INSERT
CM032587.1	1571348	.	C	T	100	*	TYPE=SUBSTITUTE
CM032587.1	1571350	.	T	C	100	*	TYPE=SUBSTITUTE
CM032587.1	1571351	.	T	C	100	*	TYPE=SUBSTITUTE
CM032587.1	1571354	.	G	A	100	*	TYPE=SUBSTITUTE
CM032587.1	1571355	.	G	A	100	*	TYPE=SUBSTITUTE
CM032587.1	1571380	.	T	C	100	*	TYPE=SUBSTITUTE
CM032587.1	1571392	.	T	TGC	100	*	TYPE=INSERT
CM032587.1	1571406	.	G	C	100	*	TYPE=SUBSTITUTE
CM032587.1	1571409	.	G	A	100	*	TYPE=SUBSTITUTE
CM032587.1	1571411	.	T	C	100	*	TYPE=SUBSTITUTE
CM032587.1	1571432	.	T	TT	100	*	TYPE=INSERT
CM032587.1	1571478	.	T	C	100	*	TYPE=SUBSTITUTE
CM032587.1	1571493	.	GA	G	100	*	TYPE=DELETE
CM032587.1	1571502	.	A	G	100	*	TYPE=SUBSTITUTE
CM032587.1	1571559	.	GA	G	100	*	TYPE=DELETE
CM032587.1	1571732	.	C	T	100	*	TYPE=SUBSTITUTE
CM032587.1	1571753	.	T	C	100	*	TYPE=SUBSTITUTE
CM032587.1	1571757	.	C	T	100	*	TYPE=SUBSTITUTE
CM032587.1	1571897	.	C	T	100	*	TYPE=SUBSTITUTE
CM032587.1	1571979	.	G	A	100	*	TYPE=SUBSTITUTE
CM032587.1	1571982	.	A	C	100	*	TYPE=SUBSTITUTE
CM032587.1	1572030	.	G	T	100	*	TYPE=SUBSTITUTE
CM032587.1	1572044	.	T	a	100	*	TYPE=SUBSTITUTE
CM032587.1	1572167	.	T	C	100	*	TYPE=SUBSTITUTE
CM032587.1	1572177	.	C	A	100	*	TYPE=SUBSTITUTE
CM032587.1	1572181	.	A	T	100	*	TYPE=SUBSTITUTE
CM032587.1	1572192	.	AG	A	100	*	TYPE=DELETE
CM032587.1	1572194	.	A	C	100	*	TYPE=SUBSTITUTE
CM032587.1	1572200	.	G	A	100	*	TYPE=SUBSTITUTE
CM032587.1	1572204	.	A	G	100	*	TYPE=SUBSTITUTE
CM032587.1	1572220	.	A	AA	100	*	TYPE=INSERT
CM032587.1	1572241	.	T	A	100	*	TYPE=SUBSTITUTE
CM032587.1	1572279	.	C	t	100	*	TYPE=SUBSTITUTE
CM032587.1	1572346	.	C	G	100	*	TYPE=SUBSTITUTE
CM032587.1	1572358	.	G	A	100	*	TYPE=SUBSTITUTE
CM032587.1	1572377	.	G	T	100	*	TYPE=SUBSTITUTE
CM032587.1	1572391	.	G	A	100	*	TYPE=SUBSTITUTE
CM032587.1	1572403	.	C	T	100	*	TYPE=SUBSTITUTE
CM032587.1	1572424	.	G	A	100	*	TYPE=SUBSTITUTE
CM032587.1	1572425	.	A	G	100	*	TYPE=SUBSTITUTE
CM032587.1	1572463	.	A	T	100	*	TYPE=SUBSTITUTE
CM032587.1	1572505	.	AGTCC	A	100	*	TYPE=DELETE
CM032587.1	1572520	.	GA	G	100	*	TYPE=DELETE
CM032587.1	1572523	.	A	G	100	*	TYPE=SUBSTITUTE
CM032587.1	1572524	.	G	A	100	*	TYPE=SUBSTITUTE
CM032587.1	1572525	.	G	A	100	*	TYPE=SUBSTITUTE
CM032587.1	1572532	.	A	G	100	*	TYPE=SUBSTITUTE
CM032587.1	1572549	.	A	G	100	*	TYPE=SUBSTITUTE
CM032587.1	1572552	.	T	C	100	*	TYPE=SUBSTITUTE
CM032587.1	1572567	.	C	A	100	*	TYPE=SUBSTITUTE
CM032587.1	1572591	.	C	T	100	*	TYPE=SUBSTITUTE
CM032587.1	1572606	.	A	G	100	*	TYPE=SUBSTITUTE
CM032587.1	1572636	.	G	A	100	*	TYPE=SUBSTITUTE
CM032587.1	1572726	.	G	A	100	*	TYPE=SUBSTITUTE
CM032587.1	1572789	.	G	A	100	*	TYPE=SUBSTITUTE
CM032587.1	1572801	.	T	A	100	*	TYPE=SUBSTITUTE
CM032587.1	1572846	.	A	G	100	*	TYPE=SUBSTITUTE
CM032587.1	1572858	.	A	G	100	*	TYPE=SUBSTITUTE
CM032587.1	1572864	.	G	A	100	*	TYPE=SUBSTITUTE
CM032587.1	1572913	.	G	C	100	*	TYPE=SUBSTITUTE
CM032587.1	1572975	.	G	a	100	*	TYPE=SUBSTITUTE
CM032587.1	1572993	.	A	G	100	*	TYPE=SUBSTITUTE
CM032587.1	1573045	.	A	C	100	*	TYPE=SUBSTITUTE

CM032587.1	1573059	.	A	G	100	*	TYPE=SUBSTITUTE
CM032587.1	1573060	.	G	T	100	*	TYPE=SUBSTITUTE
CM032587.1	1573061	.	G	T	100	*	TYPE=SUBSTITUTE
CM032587.1	1573062	.	G	C	100	*	TYPE=SUBSTITUTE
CM032587.1	1573212	.	G	A	100	*	TYPE=SUBSTITUTE
CM032587.1	1573269	.	A	G	100	*	TYPE=SUBSTITUTE
CM032587.1	1573319	.	T	C	100	*	TYPE=SUBSTITUTE
CM032587.1	1573348	.	G	A	100	*	TYPE=SUBSTITUTE
CM032587.1	1573445	.	T	G	100	*	TYPE=SUBSTITUTE
CM032587.1	1573446	.	G	A	100	*	TYPE=SUBSTITUTE
CM032587.1	1573447	.	C	T	100	*	TYPE=SUBSTITUTE
CM032587.1	1573455	.	C	T	100	*	TYPE=SUBSTITUTE
CM032587.1	1573459	.	T	C	100	*	TYPE=SUBSTITUTE
CM032587.1	1573464	.	G	GTA	100	*	TYPE=INSERT
CM032587.1	1573468	.	T	G	100	*	TYPE=SUBSTITUTE
CM032587.1	1573475	.	G	A	100	*	TYPE=SUBSTITUTE
CM032587.1	1573476	.	A	T	100	*	TYPE=SUBSTITUTE
CM032587.1	1573477	.	G	A	100	*	TYPE=SUBSTITUTE
CM032587.1	1573486	.	C	T	100	*	TYPE=SUBSTITUTE
CM032587.1	1573488	.	C	CT	100	*	TYPE=INSERT
CM032587.1	1573489	.	A	G	100	*	TYPE=SUBSTITUTE
CM032587.1	1573490	.	C	T	100	*	TYPE=SUBSTITUTE
CM032587.1	1573493	.	C	a	100	*	TYPE=SUBSTITUTE
CM032587.1	1573494	.	A	g	100	*	TYPE=SUBSTITUTE
CM032587.1	1573495	.	t	ttta	100	*	TYPE=INSERT
CM032587.1	1573496	.	G	c	100	*	TYPE=SUBSTITUTE
CM032587.1	1573498	.	G	t	100	*	TYPE=SUBSTITUTE
CM032587.1	1573503	.	a	aacct	100	*	TYPE=INSERT
CM032587.1	1573509	.	C	A	100	*	TYPE=SUBSTITUTE
CM032587.1	1573517	.	G	A	100	*	TYPE=SUBSTITUTE
CM032587.1	1573519	.	A	T	100	*	TYPE=SUBSTITUTE
CM032587.1	1573520	.	C	T	100	*	TYPE=SUBSTITUTE
CM032587.1	1573524	.	T	C	100	*	TYPE=SUBSTITUTE
CM032587.1	1573525	.	T	TGTGCTCAAAAGAAC	100	*	TYPE=INSERT
CM032587.1	1573527	.	C	T	100	*	TYPE=SUBSTITUTE
CM032587.1	1573533	.	G	A	100	*	TYPE=SUBSTITUTE
CM032587.1	1573534	.	G	A	100	*	TYPE=SUBSTITUTE
CM032587.1	1573541	.	CT	C	100	*	TYPE=DELETE
CM032587.1	1573553	.	T	C	100	*	TYPE=SUBSTITUTE
CM032587.1	1573566	.	A	T	100	*	TYPE=SUBSTITUTE
CM032587.1	1573571	.	T	A	100	*	TYPE=SUBSTITUTE
CM032587.1	1573575	.	G	A	100	*	TYPE=SUBSTITUTE
CM032587.1	1573587	.	G	A	100	*	TYPE=SUBSTITUTE
CM032587.1	1573589	.	A	G	100	*	TYPE=SUBSTITUTE
CM032587.1	1573598	.	G	T	100	*	TYPE=SUBSTITUTE
CM032587.1	1573599	.	T	C	100	*	TYPE=SUBSTITUTE
CM032587.1	1573606	.	G	A	100	*	TYPE=SUBSTITUTE
CM032587.1	1573628	.	C	G	100	*	TYPE=SUBSTITUTE
CM032587.1	1573632	.	G	A	100	*	TYPE=SUBSTITUTE
CM032587.1	1573637	.	A	AG	100	*	TYPE=INSERT
CM032587.1	1573640	.	T	A	100	*	TYPE=SUBSTITUTE
CM032587.1	1573641	.	A	G	100	*	TYPE=SUBSTITUTE
CM032587.1	1573644	.	T	TTA	100	*	TYPE=INSERT
CM032587.1	1573658	.	G	A	100	*	TYPE=SUBSTITUTE
CM032587.1	1573662	.	A	G	100	*	TYPE=SUBSTITUTE
CM032587.1	1573674	.	C	T	100	*	TYPE=SUBSTITUTE
CM032587.1	1573678	.	G	A	100	*	TYPE=SUBSTITUTE
CM032587.1	1573685	.	A	G	100	*	TYPE=SUBSTITUTE
CM032587.1	1573701	.	AT	A	100	*	TYPE=DELETE
CM032587.1	1573705	.	C	a	100	*	TYPE=SUBSTITUTE
CM032587.1	1573753	.	G	A	100	*	TYPE=SUBSTITUTE
CM032587.1	1573755	.	G	T	100	*	TYPE=SUBSTITUTE
CM032587.1	1573761	.	G	A	100	*	TYPE=SUBSTITUTE
CM032587.1	1573773	.	A	ATTGG	100	*	TYPE=INSERT

CM032587.1	1573787	.	GG	G	100	*	TYPE=DELETE
CM032587.1	1573880	.	T	TC	100	*	TYPE=INSERT
CM032587.1	1573891	.	C	G	100	*	TYPE=SUBSTITUTE
CM032587.1	1573905	.	C	T	100	*	TYPE=SUBSTITUTE
CM032587.1	1573913	.	T	C	100	*	TYPE=SUBSTITUTE
CM032587.1	1573916	.	C	T	100	*	TYPE=SUBSTITUTE
CM032587.1	1573918	.	C	A	100	*	TYPE=SUBSTITUTE
CM032587.1	1573919	.	T	C	100	*	TYPE=SUBSTITUTE
CM032587.1	1573921	.	T	A	100	*	TYPE=SUBSTITUTE
CM032587.1	1573922	.	T	C	100	*	TYPE=SUBSTITUTE
CM032587.1	1573924	.	C	G	100	*	TYPE=SUBSTITUTE
CM032587.1	1573927	.	TAC	T	100	*	TYPE=DELETE
CM032587.1	1573932	.	T	C	100	*	TYPE=SUBSTITUTE
CM032587.1	1573940	.	AGGG	A	100	*	TYPE=DELETE
CM032587.1	1573955	.	TT	T	100	*	TYPE=DELETE
CM032587.1	1574045	.	A	G	100	*	TYPE=SUBSTITUTE
CM032587.1	1574059	.	A	G	100	*	TYPE=SUBSTITUTE
CM032587.1	1574085	.	GG	G	100	*	TYPE=DELETE
CM032587.1	1574115	.	A	G	100	*	TYPE=SUBSTITUTE
CM032587.1	1574174	.	A	C	100	*	TYPE=SUBSTITUTE
CM032587.1	1574204	.	GG	G	100	*	TYPE=DELETE
CM032587.1	1574226	.	T	C	100	*	TYPE=SUBSTITUTE
CM032587.1	1574382	.	A	G	100	*	TYPE=SUBSTITUTE
CM032587.1	1574389	.	A	C	100	*	TYPE=SUBSTITUTE
CM032587.1	1574395	.	A	c	100	*	TYPE=SUBSTITUTE
CM032587.1	1574405	.	GA	G	100	*	TYPE=DELETE
CM032587.1	1574412	.	C	T	100	*	TYPE=SUBSTITUTE
CM032587.1	1574460	.	T	C	100	*	TYPE=SUBSTITUTE
CM032587.1	1574485	.	T	C	100	*	TYPE=SUBSTITUTE
CM032587.1	1574617	.	G	C	100	*	TYPE=SUBSTITUTE
CM032587.1	1574632	.	G	A	100	*	TYPE=SUBSTITUTE
CM032587.1	1574644	.	G	A	100	*	TYPE=SUBSTITUTE
CM032587.1	1574661	.	G	A	100	*	TYPE=SUBSTITUTE
CM032587.1	1574677	.	T	c	100	*	TYPE=SUBSTITUTE
CM032587.1	1574683	.	C	g	100	*	TYPE=SUBSTITUTE
CM032587.1	1574740	.	T	C	100	*	TYPE=SUBSTITUTE
CM032587.1	1574751	.	AG	A	100	*	TYPE=DELETE
CM032587.1	1574772	.	T	C	100	*	TYPE=SUBSTITUTE
CM032587.1	1574782	.	A	C	100	*	TYPE=SUBSTITUTE
CM032587.1	1574783	.	A	T	100	*	TYPE=SUBSTITUTE
CM032587.1	1574787	.	C	CC	100	*	TYPE=INSERT
CM032587.1	1574827	.	T	C	100	*	TYPE=SUBSTITUTE
CM032587.1	1574986	.	T	C	100	*	TYPE=SUBSTITUTE
CM032587.1	1574995	.	T	C	100	*	TYPE=SUBSTITUTE
CM032587.1	1575019	.	T	C	100	*	TYPE=SUBSTITUTE
CM032587.1	1575022	.	A	G	100	*	TYPE=SUBSTITUTE
CM032587.1	1575265	.	G	C	100	*	TYPE=SUBSTITUTE
CM032587.1	1575302	.	C	T	100	*	TYPE=SUBSTITUTE
CM032587.1	1575310	.	G	A	100	*	TYPE=SUBSTITUTE
CM032587.1	1575315	.	C	G	100	*	TYPE=SUBSTITUTE
CM032587.1	1575331	.	T	C	100	*	TYPE=SUBSTITUTE
CM032587.1	1575508	.	A	G	100	*	TYPE=SUBSTITUTE
CM032587.1	1575523	.	C	T	100	*	TYPE=SUBSTITUTE
CM032587.1	1575531	.	A	G	100	*	TYPE=SUBSTITUTE
CM032587.1	1575532	.	A	G	100	*	TYPE=SUBSTITUTE
CM032587.1	1575537	.	C	A	100	*	TYPE=SUBSTITUTE
CM032587.1	1575538	.	C	T	100	*	TYPE=SUBSTITUTE
CM032587.1	1575613	.	A	G	100	*	TYPE=SUBSTITUTE
CM032587.1	1575616	.	T	G	100	*	TYPE=SUBSTITUTE
CM032587.1	1575634	.	A	G	100	*	TYPE=SUBSTITUTE
CM032587.1	1575925	.	T	A	100	*	TYPE=SUBSTITUTE
CM032587.1	1575980	.	T	C	100	*	TYPE=SUBSTITUTE
CM032587.1	1576054	.	G	A	100	*	TYPE=SUBSTITUTE
CM032587.1	1576227	.	AAG	A	100	*	TYPE=DELETE

CM032587.1	1576241	.	G	GT	100	*	TYPE=INSERT
CM032587.1	1576245	.	T	C	100	*	TYPE=SUBSTITUTE
CM032587.1	1576408	.	G	A	100	*	TYPE=SUBSTITUTE
CM032587.1	1576437	.	G	A	100	*	TYPE=SUBSTITUTE
CM032587.1	1576478	.	G	C	100	*	TYPE=SUBSTITUTE
CM032587.1	1576481	.	C	T	100	*	TYPE=SUBSTITUTE
CM032587.1	1576751	.	T	C	100	*	TYPE=SUBSTITUTE
CM032587.1	1576794	.	TC	T	100	*	TYPE=DELETE
CM032587.1	1576796	.	G	C	100	*	TYPE=SUBSTITUTE
CM032587.1	1576942	.	G	a	100	*	TYPE=SUBSTITUTE
CM032587.1	1576982	.	C	T	100	*	TYPE=SUBSTITUTE
CM032587.1	1577009	.	T	TT	100	*	TYPE=INSERT
CM032587.1	1577033	.	C	T	100	*	TYPE=SUBSTITUTE
CM032587.1	1577061	.	T	C	100	*	TYPE=SUBSTITUTE
CM032587.1	1577121	.	G	C	100	*	TYPE=SUBSTITUTE
CM032587.1	1577134	.	TG	T	100	*	TYPE=DELETE
CM032587.1	1577141	.	T	C	100	*	TYPE=SUBSTITUTE
CM032587.1	1577200	.	C	T	100	*	TYPE=SUBSTITUTE
CM032587.1	1577220	.	T	c	100	*	TYPE=SUBSTITUTE
CM032587.1	1577225	.	C	t	100	*	TYPE=SUBSTITUTE
CM032587.1	1577399	.	T	C	100	*	TYPE=SUBSTITUTE
CM032587.1	1577473	.	C	t	100	*	TYPE=SUBSTITUTE
CM032587.1	1577533	.	AT	A	100	*	TYPE=DELETE
CM032587.1	1577534	.	C	t	100	*	TYPE=SUBSTITUTE
CM032587.1	1577560	.	TGT	T	100	*	TYPE=DELETE
CM032587.1	1577741	.	C	T	100	*	TYPE=SUBSTITUTE
CM032587.1	1577763	.	A	G	100	*	TYPE=SUBSTITUTE
CM032587.1	1577854	.	a	aacctG	100	*	TYPE=INSERT
CM032587.1	1577862	.	G	c	100	*	TYPE=SUBSTITUTE
CM032587.1	1577882	.	G	C	100	*	TYPE=SUBSTITUTE
CM032587.1	1577899	.	AC	A	100	*	TYPE=DELETE
CM032587.1	1577904	.	TT	T	100	*	TYPE=DELETE
CM032587.1	1577920	.	T	A	100	*	TYPE=SUBSTITUTE
CM032587.1	1577926	.	ATCCA	A	100	*	TYPE=DELETE
CM032587.1	1577944	.	A	G	100	*	TYPE=SUBSTITUTE
CM032587.1	1577948	.	C	T	100	*	TYPE=SUBSTITUTE
CM032587.1	1577977	.	C	T	100	*	TYPE=SUBSTITUTE
CM032587.1	1577987	.	A	G	100	*	TYPE=SUBSTITUTE
CM032587.1	1577989	.	T	C	100	*	TYPE=SUBSTITUTE
CM032587.1	1578028	.	G	c	100	*	TYPE=SUBSTITUTE
CM032587.1	1578039	.	TTTGCTGTGCT	T	100	*	TYPE=DELETE
CM032587.1	1578059	.	A	ACTGTG	100	*	TYPE=INSERT
CM032587.1	1578069	.	G	C	100	*	TYPE=SUBSTITUTE
CM032587.1	1578095	.	G	T	100	*	TYPE=SUBSTITUTE
CM032587.1	1578096	.	T	G	100	*	TYPE=SUBSTITUTE
CM032587.1	1578110	.	TGGAGATTCAGAC	T	100	*	TYPE=DELETE
CM032587.1	1578119	.	TG	T	100	*	TYPE=DELETE
CM032587.1	1578124	.	T	C	100	*	TYPE=SUBSTITUTE
CM032587.1	1578125	.	G	A	100	*	TYPE=SUBSTITUTE
CM032587.1	1578131	.	T	C	100	*	TYPE=SUBSTITUTE
CM032587.1	1578136	.	C	CAGAT	100	*	TYPE=INSERT
CM032587.1	1578144	.	G	A	100	*	TYPE=SUBSTITUTE
CM032587.1	1578145	.	A	G	100	*	TYPE=SUBSTITUTE
CM032587.1	1578151	.	G	GAAAT	100	*	TYPE=INSERT
CM032587.1	1578153	.	A	G	100	*	TYPE=SUBSTITUTE
CM032587.1	1578155	.	C	A	100	*	TYPE=SUBSTITUTE
CM032587.1	1578161	.	A	G	100	*	TYPE=SUBSTITUTE
CM032587.1	1578163	.	A	G	100	*	TYPE=SUBSTITUTE
CM032587.1	1578167	.	C	A	100	*	TYPE=SUBSTITUTE
CM032587.1	1578177	.	T	C	100	*	TYPE=SUBSTITUTE
CM032587.1	1578178	.	G	T	100	*	TYPE=SUBSTITUTE
CM032587.1	1578179	.	G	C	100	*	TYPE=SUBSTITUTE
CM032587.1	1578181	.	GA	G	100	*	TYPE=DELETE
CM032587.1	1578193	.	ACTGTAG	A	100	*	TYPE=DELETE

CM032587.1	1578196	.	T	G	100	*	TYPE=SUBSTITUTE
CM032587.1	1578202	.	C	G	100	*	TYPE=SUBSTITUTE
CM032587.1	1578208	.	G	GCGGTAGGCGTAC	100	*	TYPE=INSERT
CM032587.1	1578231	.	A	T	100	*	TYPE=SUBSTITUTE
CM032587.1	1578232	.	T	G	100	*	TYPE=SUBSTITUTE
CM032587.1	1578233	.	C	T	100	*	TYPE=SUBSTITUTE
CM032587.1	1578242	.	TA	T	100	*	TYPE=DELETE
CM032587.1	1578255	.	GGA	G	100	*	TYPE=DELETE
CM032587.1	1578265	.	C	G	100	*	TYPE=SUBSTITUTE
CM032587.1	1578282	.	AC	A	100	*	TYPE=DELETE
CM032587.1	1578296	.	T	C	100	*	TYPE=SUBSTITUTE
CM032587.1	1578298	.	C	T	100	*	TYPE=SUBSTITUTE
CM032587.1	1578376	.	A	AGCACCATGTGC	100	*	TYPE=INSERT
CM032587.1	1578378	.	A	G	100	*	TYPE=SUBSTITUTE
CM032587.1	1578413	.	C	CT	100	*	TYPE=INSERT
CM032587.1	1578423	.	G	A	100	*	TYPE=SUBSTITUTE
CM032587.1	1578438	.	A	AT	100	*	TYPE=INSERT
CM032587.1	1578457	.	T	C	100	*	TYPE=SUBSTITUTE
CM032587.1	1578460	.	c	cA	100	*	TYPE=INSERT
CM032587.1	1578474	.	T	a	100	*	TYPE=SUBSTITUTE
CM032587.1	1578475	.	t	ta	100	*	TYPE=INSERT
CM032587.1	1578478	.	G	A	100	*	TYPE=SUBSTITUTE
CM032587.1	1578481	.	A	G	100	*	TYPE=SUBSTITUTE
CM032587.1	1578483	.	A	G	100	*	TYPE=SUBSTITUTE
CM032587.1	1578492	.	T	G	100	*	TYPE=SUBSTITUTE
CM032587.1	1578507	.	T	C	100	*	TYPE=SUBSTITUTE
CM032587.1	1578518	.	c	ctttg	100	*	TYPE=INSERT
CM032587.1	1578523	.	G	c	100	*	TYPE=SUBSTITUTE
CM032587.1	1578524	.	G	t	100	*	TYPE=SUBSTITUTE
CM032587.1	1578535	.	CG	C	100	*	TYPE=DELETE
CM032587.1	1578550	.	t	tagc	100	*	TYPE=INSERT
CM032587.1	1578555	.	G	t	100	*	TYPE=SUBSTITUTE
CM032587.1	1578558	.	A	g	100	*	TYPE=SUBSTITUTE
CM032587.1	1578561	.	A	g	100	*	TYPE=SUBSTITUTE
CM032587.1	1578603	.	C	T	100	*	TYPE=SUBSTITUTE
CM032587.1	1578624	.	C	G	100	*	TYPE=SUBSTITUTE
CM032587.1	1578648	.	T	C	100	*	TYPE=SUBSTITUTE
CM032587.1	1578666	.	G	T	100	*	TYPE=SUBSTITUTE
CM032587.1	1578672	.	A	G	100	*	TYPE=SUBSTITUTE
CM032587.1	1578687	.	T	C	100	*	TYPE=SUBSTITUTE
CM032587.1	1578690	.	G	A	100	*	TYPE=SUBSTITUTE
CM032587.1	1578712	.	A	G	100	*	TYPE=SUBSTITUTE
CM032587.1	1578716	.	A	C	100	*	TYPE=SUBSTITUTE
CM032587.1	1578719	.	G	C	100	*	TYPE=SUBSTITUTE
CM032587.1	1578720	.	G	C	100	*	TYPE=SUBSTITUTE
CM032587.1	1578730	.	A	T	100	*	TYPE=SUBSTITUTE
CM032587.1	1578735	.	T	C	100	*	TYPE=SUBSTITUTE
CM032587.1	1578736	.	A	G	100	*	TYPE=SUBSTITUTE
CM032587.1	1578741	.	C	T	100	*	TYPE=SUBSTITUTE
CM032587.1	1578743	.	G	A	100	*	TYPE=SUBSTITUTE
CM032587.1	1578749	.	C	T	100	*	TYPE=SUBSTITUTE
CM032587.1	1578753	.	C	CN	100	*	TYPE=INSERT
CM032587.1	1578757	.	G	A	100	*	TYPE=SUBSTITUTE
CM032587.1	1578760	.	T	C	100	*	TYPE=SUBSTITUTE
CM032587.1	1578766	.	AC	A	100	*	TYPE=DELETE
CM032587.1	1578771	.	G	C	100	*	TYPE=SUBSTITUTE
CM032587.1	1578801	.	T	C	100	*	TYPE=SUBSTITUTE
CM032587.1	1578807	.	C	T	100	*	TYPE=SUBSTITUTE
CM032587.1	1578816	.	A	G	100	*	TYPE=SUBSTITUTE
CM032587.1	1578822	.	T	C	100	*	TYPE=SUBSTITUTE
CM032587.1	1578825	.	A	G	100	*	TYPE=SUBSTITUTE
CM032587.1	1578896	.	A	G	100	*	TYPE=SUBSTITUTE
CM032587.1	1578897	.	G	A	100	*	TYPE=SUBSTITUTE
CM032587.1	1578903	.	A	G	100	*	TYPE=SUBSTITUTE

CM032587.1	1578904	.	T	A	100	*	TYPE=SUBSTITUTE
CM032587.1	1578929	.	T	G	100	*	TYPE=SUBSTITUTE
CM032587.1	1578962	.	A	G	100	*	TYPE=SUBSTITUTE
CM032587.1	1578963	.	T	C	100	*	TYPE=SUBSTITUTE
CM032587.1	1578966	.	G	A	100	*	TYPE=SUBSTITUTE
CM032587.1	1578968	.	A	T	100	*	TYPE=SUBSTITUTE
CM032587.1	1578974	.	A	C	100	*	TYPE=SUBSTITUTE
CM032587.1	1578976	.	T	C	100	*	TYPE=SUBSTITUTE
CM032587.1	1578979	.	T	C	100	*	TYPE=SUBSTITUTE
CM032587.1	1578984	.	T	C	100	*	TYPE=SUBSTITUTE
CM032587.1	1578985	.	C	T	100	*	TYPE=SUBSTITUTE
CM032587.1	1578990	.	G	GA	100	*	TYPE=INSERT
CM032587.1	1578994	.	T	A	100	*	TYPE=SUBSTITUTE
CM032587.1	1579003	.	T	G	100	*	TYPE=SUBSTITUTE
CM032587.1	1579020	.	TT	T	100	*	TYPE=DELETE
CM032587.1	1579065	.	C	T	100	*	TYPE=SUBSTITUTE
CM032587.1	1579074	.	C	T	100	*	TYPE=SUBSTITUTE
CM032587.1	1579098	.	A	G	100	*	TYPE=SUBSTITUTE
CM032587.1	1579110	.	A	T	100	*	TYPE=SUBSTITUTE
CM032587.1	1579113	.	G	A	100	*	TYPE=SUBSTITUTE
CM032587.1	1579122	.	C	T	100	*	TYPE=SUBSTITUTE
CM032587.1	1579130	.	C	T	100	*	TYPE=SUBSTITUTE
CM032587.1	1579155	.	C	A	100	*	TYPE=SUBSTITUTE
CM032587.1	1579176	.	T	C	100	*	TYPE=SUBSTITUTE
CM032587.1	1579177	.	T	G	100	*	TYPE=SUBSTITUTE
CM032587.1	1579181	.	C	T	100	*	TYPE=SUBSTITUTE
CM032587.1	1579183	.	A	G	100	*	TYPE=SUBSTITUTE
CM032587.1	1579190	.	A	G	100	*	TYPE=SUBSTITUTE
CM032587.1	1579193	.	A	G	100	*	TYPE=SUBSTITUTE
CM032587.1	1579194	.	C	T	100	*	TYPE=SUBSTITUTE
CM032587.1	1579196	.	A	G	100	*	TYPE=SUBSTITUTE
CM032587.1	1579197	.	C	G	100	*	TYPE=SUBSTITUTE
CM032587.1	1579200	.	C	T	100	*	TYPE=SUBSTITUTE
CM032587.1	1579208	.	TGAG	T	100	*	TYPE=DELETE
CM032587.1	1579211	.	G	C	100	*	TYPE=SUBSTITUTE
CM032587.1	1579212	.	T	A	100	*	TYPE=SUBSTITUTE
CM032587.1	1579214	.	T	G	100	*	TYPE=SUBSTITUTE
CM032587.1	1579221	.	G	A	100	*	TYPE=SUBSTITUTE
CM032587.1	1579222	.	A	C	100	*	TYPE=SUBSTITUTE
CM032587.1	1579232	.	CA	C	100	*	TYPE=DELETE
CM032587.1	1579239	.	G	T	100	*	TYPE=SUBSTITUTE
CM032587.1	1579243	.	A	C	100	*	TYPE=SUBSTITUTE
CM032587.1	1579250	.	A	G	100	*	TYPE=SUBSTITUTE
CM032587.1	1579251	.	C	A	100	*	TYPE=SUBSTITUTE
CM032587.1	1579252	.	G	A	100	*	TYPE=SUBSTITUTE
CM032587.1	1579260	.	CC	C	100	*	TYPE=DELETE
CM032587.1	1579433	.	A	T	100	*	TYPE=SUBSTITUTE
CM032587.1	1579509	.	T	G	100	*	TYPE=SUBSTITUTE
CM032587.1	1579510	.	C	T	100	*	TYPE=SUBSTITUTE
CM032587.1	1579511	.	G	C	100	*	TYPE=SUBSTITUTE
CM032587.1	1579524	.	C	T	100	*	TYPE=SUBSTITUTE
CM032587.1	1579562	.	T	G	100	*	TYPE=SUBSTITUTE
CM032587.1	1579571	.	C	T	100	*	TYPE=SUBSTITUTE
CM032587.1	1579595	.	A	G	100	*	TYPE=SUBSTITUTE
CM032587.1	1579604	.	A	G	100	*	TYPE=SUBSTITUTE
CM032587.1	1579676	.	A	C	100	*	TYPE=SUBSTITUTE
CM032587.1	1579760	.	T	C	100	*	TYPE=SUBSTITUTE
CM032587.1	1579778	.	C	G	100	*	TYPE=SUBSTITUTE
CM032587.1	1579787	.	C	T	100	*	TYPE=SUBSTITUTE
CM032587.1	1579799	.	A	G	100	*	TYPE=SUBSTITUTE
CM032587.1	1579802	.	G	A	100	*	TYPE=SUBSTITUTE
CM032587.1	1579817	.	G	A	100	*	TYPE=SUBSTITUTE
CM032587.1	1579862	.	T	C	100	*	TYPE=SUBSTITUTE
CM032587.1	1579865	.	C	T	100	*	TYPE=SUBSTITUTE

CM032587.1	1579905	.	G	A	100	*	TYPE=SUBSTITUTE
CM032587.1	1579922	.	G	A	100	*	TYPE=SUBSTITUTE
CM032587.1	1579932	.	T	G	100	*	TYPE=SUBSTITUTE
CM032587.1	1579935	.	G	A	100	*	TYPE=SUBSTITUTE
CM032587.1	1579971	.	C	T	100	*	TYPE=SUBSTITUTE
CM032587.1	1579986	.	T	A	100	*	TYPE=SUBSTITUTE
CM032587.1	1580016	.	C	T	100	*	TYPE=SUBSTITUTE
CM032587.1	1580021	.	C	T	100	*	TYPE=SUBSTITUTE
CM032587.1	1580025	.	G	A	100	*	TYPE=SUBSTITUTE
CM032587.1	1580085	.	A	G	100	*	TYPE=SUBSTITUTE
CM032587.1	1580099	.	T	C	100	*	TYPE=SUBSTITUTE
CM032587.1	1580103	.	G	C	100	*	TYPE=SUBSTITUTE
CM032587.1	1580109	.	G	a	100	*	TYPE=SUBSTITUTE
CM032587.1	1580115	.	A	g	100	*	TYPE=SUBSTITUTE
CM032587.1	1580117	.	C	g	100	*	TYPE=SUBSTITUTE
CM032587.1	1580130	.	A	G	100	*	TYPE=SUBSTITUTE
CM032587.1	1580139	.	C	T	100	*	TYPE=SUBSTITUTE
CM032587.1	1580144	.	C	T	100	*	TYPE=SUBSTITUTE
CM032587.1	1580159	.	G	A	100	*	TYPE=SUBSTITUTE
CM032587.1	1580175	.	G	A	100	*	TYPE=SUBSTITUTE
CM032587.1	1580193	.	G	A	100	*	TYPE=SUBSTITUTE
CM032587.1	1580198	.	A	G	100	*	TYPE=SUBSTITUTE
CM032587.1	1580274	.	C	T	100	*	TYPE=SUBSTITUTE
CM032587.1	1580313	.	T	A	100	*	TYPE=SUBSTITUTE
CM032587.1	1580315	.	A	G	100	*	TYPE=SUBSTITUTE
CM032587.1	1580325	.	T	A	100	*	TYPE=SUBSTITUTE
CM032587.1	1580349	.	T	C	100	*	TYPE=SUBSTITUTE
CM032587.1	1580368	.	G	A	100	*	TYPE=SUBSTITUTE
CM032587.1	1580369	.	T	A	100	*	TYPE=SUBSTITUTE
CM032587.1	1580421	.	A	T	100	*	TYPE=SUBSTITUTE
CM032587.1	1580427	.	C	T	100	*	TYPE=SUBSTITUTE
CM032587.1	1580445	.	A	G	100	*	TYPE=SUBSTITUTE
CM032587.1	1580462	.	A	G	100	*	TYPE=SUBSTITUTE
CM032587.1	1580514	.	G	A	100	*	TYPE=SUBSTITUTE
CM032587.1	1580517	.	C	a	100	*	TYPE=SUBSTITUTE
CM032587.1	1580534	.	T	c	100	*	TYPE=SUBSTITUTE
CM032587.1	1580548	.	A	G	100	*	TYPE=SUBSTITUTE
CM032587.1	1580559	.	G	T	100	*	TYPE=SUBSTITUTE
CM032587.1	1580574	.	T	C	100	*	TYPE=SUBSTITUTE
CM032587.1	1580580	.	G	T	100	*	TYPE=SUBSTITUTE
CM032587.1	1580588	.	A	G	100	*	TYPE=SUBSTITUTE
CM032587.1	1580613	.	T	A	100	*	TYPE=SUBSTITUTE
CM032587.1	1580618	.	G	A	100	*	TYPE=SUBSTITUTE
CM032587.1	1580623	.	C	T	100	*	TYPE=SUBSTITUTE
CM032587.1	1580626	.	T	A	100	*	TYPE=SUBSTITUTE
CM032587.1	1580627	.	A	G	100	*	TYPE=SUBSTITUTE
CM032587.1	1580628	.	A	G	100	*	TYPE=SUBSTITUTE
CM032587.1	1580660	.	T	C	100	*	TYPE=SUBSTITUTE
CM032587.1	1580674	.	C	A	100	*	TYPE=SUBSTITUTE
CM032587.1	1580697	.	A	C	100	*	TYPE=SUBSTITUTE
CM032587.1	1580715	.	A	G	100	*	TYPE=SUBSTITUTE
CM032587.1	1580727	.	T	A	100	*	TYPE=SUBSTITUTE
CM032587.1	1580735	.	A	G	100	*	TYPE=SUBSTITUTE
CM032587.1	1580736	.	C	G	100	*	TYPE=SUBSTITUTE
CM032587.1	1580770	.	G	A	100	*	TYPE=SUBSTITUTE
CM032587.1	1580774	.	G	C	100	*	TYPE=SUBSTITUTE
CM032587.1	1580784	.	A	G	100	*	TYPE=SUBSTITUTE
CM032587.1	1580788	.	A	G	100	*	TYPE=SUBSTITUTE
CM032587.1	1580805	.	C	T	100	*	TYPE=SUBSTITUTE
CM032587.1	1580827	.	G	C	100	*	TYPE=SUBSTITUTE
CM032587.1	1580852	.	T	C	100	*	TYPE=SUBSTITUTE
CM032587.1	1580857	.	C	T	100	*	TYPE=SUBSTITUTE
CM032587.1	1580871	.	A	G	100	*	TYPE=SUBSTITUTE
CM032587.1	1580873	.	C	T	100	*	TYPE=SUBSTITUTE

CM032587.1	1580881	.	C	G	100	*	TYPE=SUBSTITUTE
CM032587.1	1580922	.	A	T	100	*	TYPE=SUBSTITUTE
CM032587.1	1581046	.	T	C	100	*	TYPE=SUBSTITUTE
CM032587.1	1581182	.	G	A	100	*	TYPE=SUBSTITUTE
CM032587.1	1581200	.	G	A	100	*	TYPE=SUBSTITUTE
CM032587.1	1581234	.	T	C	100	*	TYPE=SUBSTITUTE
CM032587.1	1581315	.	G	A	100	*	TYPE=SUBSTITUTE
CM032587.1	1581408	.	G	A	100	*	TYPE=SUBSTITUTE
CM032587.1	1581430	.	A	T	100	*	TYPE=SUBSTITUTE
CM032587.1	1581442	.	A	G	100	*	TYPE=SUBSTITUTE
CM032587.1	1581534	.	A	T	100	*	TYPE=SUBSTITUTE
CM032587.1	1581838	.	GT	G	100	*	TYPE=DELETE
CM032587.1	1581962	.	G	A	100	*	TYPE=SUBSTITUTE
CM032587.1	1581997	.	T	G	100	*	TYPE=SUBSTITUTE
CM032587.1	1582042	.	A	T	100	*	TYPE=SUBSTITUTE
CM032587.1	1582049	.	A	G	100	*	TYPE=SUBSTITUTE
CM032587.1	1582127	.	A	G	100	*	TYPE=SUBSTITUTE
CM032587.1	1582274	.	T	C	100	*	TYPE=SUBSTITUTE
CM032587.1	1582294	.	A	G	100	*	TYPE=SUBSTITUTE
CM032587.1	1582396	.	T	C	100	*	TYPE=SUBSTITUTE
CM032587.1	1582541	.	A	G	100	*	TYPE=SUBSTITUTE
CM032587.1	1582618	.	T	C	100	*	TYPE=SUBSTITUTE
CM032587.1	1582648	.	A	g	100	*	TYPE=SUBSTITUTE
CM032587.1	1582708	.	T	C	100	*	TYPE=SUBSTITUTE
CM032587.1	1582717	.	C	G	100	*	TYPE=SUBSTITUTE
CM032587.1	1582729	.	T	A	100	*	TYPE=SUBSTITUTE
CM032587.1	1582748	.	G	A	100	*	TYPE=SUBSTITUTE
CM032587.1	1582750	.	A	G	100	*	TYPE=SUBSTITUTE
CM032587.1	1582759	.	A	G	100	*	TYPE=SUBSTITUTE
CM032587.1	1582790	.	G	C	100	*	TYPE=SUBSTITUTE
CM032587.1	1582818	.	C	T	100	*	TYPE=SUBSTITUTE
CM032587.1	1582828	.	T	C	100	*	TYPE=SUBSTITUTE
CM032587.1	1582846	.	G	A	100	*	TYPE=SUBSTITUTE
CM032587.1	1582873	.	T	G	100	*	TYPE=SUBSTITUTE
CM032587.1	1582885	.	A	G	100	*	TYPE=SUBSTITUTE
CM032587.1	1582904	.	G	A	100	*	TYPE=SUBSTITUTE
CM032587.1	1582913	.	T	G	100	*	TYPE=SUBSTITUTE
CM032587.1	1582921	.	T	C	100	*	TYPE=SUBSTITUTE
CM032587.1	1582928	.	AG	A	100	*	TYPE=DELETE
CM032587.1	1582959	.	C	T	100	*	TYPE=SUBSTITUTE
CM032587.1	1582960	.	A	G	100	*	TYPE=SUBSTITUTE
CM032587.1	1582963	.	A	G	100	*	TYPE=SUBSTITUTE
CM032587.1	1582991	.	T	C	100	*	TYPE=SUBSTITUTE
CM032587.1	1583024	.	G	T	100	*	TYPE=SUBSTITUTE
CM032587.1	1583060	.	T	C	100	*	TYPE=SUBSTITUTE
CM032587.1	1583069	.	A	T	100	*	TYPE=SUBSTITUTE
CM032587.1	1583084	.	C	T	100	*	TYPE=SUBSTITUTE
CM032587.1	1583120	.	T	C	100	*	TYPE=SUBSTITUTE
CM032587.1	1583126	.	G	T	100	*	TYPE=SUBSTITUTE
CM032587.1	1583129	.	G	A	100	*	TYPE=SUBSTITUTE
CM032587.1	1583132	.	T	C	100	*	TYPE=SUBSTITUTE
CM032587.1	1583135	.	G	A	100	*	TYPE=SUBSTITUTE
CM032587.1	1583147	.	T	G	100	*	TYPE=SUBSTITUTE
CM032587.1	1583156	.	A	T	100	*	TYPE=SUBSTITUTE
CM032587.1	1583162	.	A	G	100	*	TYPE=SUBSTITUTE
CM032587.1	1583180	.	C	G	100	*	TYPE=SUBSTITUTE
CM032587.1	1583183	.	C	G	100	*	TYPE=SUBSTITUTE
CM032587.1	1583189	.	A	G	100	*	TYPE=SUBSTITUTE
CM032587.1	1583195	.	C	T	100	*	TYPE=SUBSTITUTE
CM032587.1	1583201	.	C	T	100	*	TYPE=SUBSTITUTE
CM032587.1	1583202	.	A	G	100	*	TYPE=SUBSTITUTE
CM032587.1	1583203	.	A	G	100	*	TYPE=SUBSTITUTE
CM032587.1	1583205	.	G	A	100	*	TYPE=SUBSTITUTE
CM032587.1	1583210	.	G	A	100	*	TYPE=SUBSTITUTE

CM032587.1	1583216	.	T	C	100	*	TYPE=SUBSTITUTE
CM032587.1	1583227	.	G	A	100	*	TYPE=SUBSTITUTE
CM032587.1	1583231	.	T	A	100	*	TYPE=SUBSTITUTE
CM032587.1	1583243	.	T	C	100	*	TYPE=SUBSTITUTE
CM032587.1	1583244	.	C	G	100	*	TYPE=SUBSTITUTE
CM032587.1	1583246	.	A	G	100	*	TYPE=SUBSTITUTE
CM032587.1	1583267	.	T	C	100	*	TYPE=SUBSTITUTE
CM032587.1	1583276	.	G	C	100	*	TYPE=SUBSTITUTE
CM032587.1	1583303	.	C	A	100	*	TYPE=SUBSTITUTE
CM032587.1	1583306	.	T	C	100	*	TYPE=SUBSTITUTE
CM032587.1	1583325	.	A	T	100	*	TYPE=SUBSTITUTE
CM032587.1	1583326	.	T	C	100	*	TYPE=SUBSTITUTE
CM032587.1	1583328	.	T	C	100	*	TYPE=SUBSTITUTE
CM032587.1	1583331	.	G	A	100	*	TYPE=SUBSTITUTE
CM032587.1	1583332	.	T	C	100	*	TYPE=SUBSTITUTE
CM032587.1	1583350	.	A	AG	100	*	TYPE=INSERT
CM032587.1	1583352	.	G	T	100	*	TYPE=SUBSTITUTE
CM032587.1	1583354	.	A	T	100	*	TYPE=SUBSTITUTE
CM032587.1	1583356	.	C	T	100	*	TYPE=SUBSTITUTE
CM032587.1	1583358	.	A	T	100	*	TYPE=SUBSTITUTE
CM032587.1	1583359	.	A	C	100	*	TYPE=SUBSTITUTE
CM032587.1	1583360	.	G	T	100	*	TYPE=SUBSTITUTE
CM032587.1	1583361	.	A	G	100	*	TYPE=SUBSTITUTE
CM032587.1	1583362	.	A	T	100	*	TYPE=SUBSTITUTE
CM032587.1	1583363	.	A	C	100	*	TYPE=SUBSTITUTE
CM032587.1	1583364	.	C	T	100	*	TYPE=SUBSTITUTE
CM032587.1	1583365	.	C	G	100	*	TYPE=SUBSTITUTE
CM032587.1	1583366	.	A	T	100	*	TYPE=SUBSTITUTE
CM032587.1	1583367	.	C	T	100	*	TYPE=SUBSTITUTE
CM032587.1	1583369	.	T	G	100	*	TYPE=SUBSTITUTE
CM032587.1	1583371	.	C	T	100	*	TYPE=SUBSTITUTE
CM032587.1	1583374	.	A	AC	100	*	TYPE=INSERT
CM032587.1	1583376	.	G	GACTCTCC	100	*	TYPE=INSERT
CM032587.1	1583380	.	G	GAGCAGTTA	100	*	TYPE=INSERT
CM032587.1	1583383	.	C	T	100	*	TYPE=SUBSTITUTE
CM032587.1	1583386	.	G	A	100	*	TYPE=SUBSTITUTE
CM032587.1	1583389	.	T	C	100	*	TYPE=SUBSTITUTE
CM032587.1	1583398	.	C	T	100	*	TYPE=SUBSTITUTE
CM032587.1	1583399	.	G	A	100	*	TYPE=SUBSTITUTE
CM032587.1	1583400	.	T	C	100	*	TYPE=SUBSTITUTE
CM032587.1	1583401	.	G	A	100	*	TYPE=SUBSTITUTE
CM032587.1	1583402	.	A	G	100	*	TYPE=SUBSTITUTE
CM032587.1	1583405	.	G	A	100	*	TYPE=SUBSTITUTE
CM032587.1	1583418	.	C	T	100	*	TYPE=SUBSTITUTE
CM032587.1	1583421	.	G	A	100	*	TYPE=SUBSTITUTE
CM032587.1	1583422	.	G	C	100	*	TYPE=SUBSTITUTE
CM032587.1	1583430	.	GTAC	G	100	*	TYPE=DELETE
CM032587.1	1583437	.	C	G	100	*	TYPE=SUBSTITUTE
CM032587.1	1583447	.	C	G	100	*	TYPE=SUBSTITUTE
CM032587.1	1583458	.	T	C	100	*	TYPE=SUBSTITUTE
CM032587.1	1583459	.	C	G	100	*	TYPE=SUBSTITUTE
CM032587.1	1583460	.	T	C	100	*	TYPE=SUBSTITUTE
CM032587.1	1583464	.	C	G	100	*	TYPE=SUBSTITUTE
CM032587.1	1583529	.	A	G	100	*	TYPE=SUBSTITUTE
CM032587.1	1583567	.	T	C	100	*	TYPE=SUBSTITUTE
CM032587.1	1583751	.	T	C	100	*	TYPE=SUBSTITUTE
CM032587.1	1583769	.	T	C	100	*	TYPE=SUBSTITUTE
CM032587.1	1583770	.	C	T	100	*	TYPE=SUBSTITUTE
CM032587.1	1583784	.	GA	G	100	*	TYPE=DELETE
CM032587.1	1583786	.	T	C	100	*	TYPE=SUBSTITUTE
CM032587.1	1583792	.	T	C	100	*	TYPE=SUBSTITUTE
CM032587.1	1583814	.	C	T	100	*	TYPE=SUBSTITUTE
CM032587.1	1583815	.	G	GT	100	*	TYPE=INSERT
CM032587.1	1583826	.	A	G	100	*	TYPE=SUBSTITUTE

CM032587.1	1583829	.	G	A	100	*	TYPE=SUBSTITUTE
CM032587.1	1583902	.	G	A	100	*	TYPE=SUBSTITUTE
CM032587.1	1583943	.	C	T	100	*	TYPE=SUBSTITUTE
CM032587.1	1583954	.	T	G	100	*	TYPE=SUBSTITUTE
CM032587.1	1583955	.	C	T	100	*	TYPE=SUBSTITUTE
CM032587.1	1583957	.	TA	T	100	*	TYPE=DELETE
CM032587.1	1583965	.	CCTACGA	C	100	*	TYPE=DELETE
CM032587.1	1583969	.	A	T	100	*	TYPE=SUBSTITUTE
CM032587.1	1583981	.	GTACTTTGG	G	100	*	TYPE=DELETE
CM032587.1	1584036	.	C	T	100	*	TYPE=SUBSTITUTE
CM032587.1	1584107	.	C	A	100	*	TYPE=SUBSTITUTE
CM032587.1	1584134	.	A	C	100	*	TYPE=SUBSTITUTE
CM032587.1	1584141	.	C	G	100	*	TYPE=SUBSTITUTE
CM032587.1	1584143	.	T	C	100	*	TYPE=SUBSTITUTE
CM032587.1	1584179	.	AACCTG	A	100	*	TYPE=DELETE
CM032587.1	1584194	.	G	T	100	*	TYPE=SUBSTITUTE
CM032587.1	1584210	.	A	G	100	*	TYPE=SUBSTITUTE
CM032587.1	1584213	.	T	C	100	*	TYPE=SUBSTITUTE
CM032587.1	1584214	.	T	G	100	*	TYPE=SUBSTITUTE
CM032587.1	1584234	.	C	T	100	*	TYPE=SUBSTITUTE
CM032587.1	1584236	.	C	CTTA	100	*	TYPE=INSERT
CM032587.1	1584244	.	C	T	100	*	TYPE=SUBSTITUTE
CM032587.1	1584309	.	T	C	100	*	TYPE=SUBSTITUTE
CM032587.1	1584398	.	T	TT	100	*	TYPE=INSERT
CM032587.1	1584597	.	C	A	100	*	TYPE=SUBSTITUTE
CM032587.1	1584601	.	A	G	100	*	TYPE=SUBSTITUTE
CM032587.1	1584611	.	G	A	100	*	TYPE=SUBSTITUTE
CM032587.1	1584619	.	A	G	100	*	TYPE=SUBSTITUTE
CM032587.1	1584622	.	G	A	100	*	TYPE=SUBSTITUTE
CM032587.1	1584627	.	G	A	100	*	TYPE=SUBSTITUTE
CM032587.1	1584631	.	T	C	100	*	TYPE=SUBSTITUTE
CM032587.1	1584643	.	C	CA	100	*	TYPE=INSERT
CM032587.1	1584645	.	A	ACCAAC	100	*	TYPE=INSERT
CM032587.1	1584742	.	CAGCG	C	100	*	TYPE=DELETE
CM032587.1	1584756	.	TA	T	100	*	TYPE=DELETE
CM032587.1	1584788	.	G	GT	100	*	TYPE=INSERT
CM032587.1	1584795	.	A	G	100	*	TYPE=SUBSTITUTE
CM032587.1	1584838	.	A	AA	100	*	TYPE=INSERT
CM032587.1	1585098	.	C	G	100	*	TYPE=SUBSTITUTE
CM032587.1	1585174	.	T	G	100	*	TYPE=SUBSTITUTE
CM032587.1	1585195	.	G	A	100	*	TYPE=SUBSTITUTE
CM032587.1	1585200	.	A	G	100	*	TYPE=SUBSTITUTE
CM032587.1	1585255	.	C	G	100	*	TYPE=SUBSTITUTE
CM032587.1	1585617	.	G	A	100	*	TYPE=SUBSTITUTE
CM032587.1	1585803	.	G	A	100	*	TYPE=SUBSTITUTE
CM032587.1	1585914	.	T	C	100	*	TYPE=SUBSTITUTE
CM032587.1	1585924	.	G	A	100	*	TYPE=SUBSTITUTE
CM032587.1	1585926	.	A	C	100	*	TYPE=SUBSTITUTE
CM032587.1	1585945	.	CCAT	C	100	*	TYPE=DELETE
CM032587.1	1585953	.	T	C	100	*	TYPE=SUBSTITUTE
CM032587.1	1585980	.	A	C	100	*	TYPE=SUBSTITUTE
CM032587.1	1586016	.	C	T	100	*	TYPE=SUBSTITUTE
CM032587.1	1586019	.	A	G	100	*	TYPE=SUBSTITUTE
CM032587.1	1586034	.	A	C	100	*	TYPE=SUBSTITUTE
CM032587.1	1586052	.	G	C	100	*	TYPE=SUBSTITUTE
CM032587.1	1586082	.	A	G	100	*	TYPE=SUBSTITUTE
CM032587.1	1586175	.	A	G	100	*	TYPE=SUBSTITUTE
CM032587.1	1586193	.	G	a	100	*	TYPE=SUBSTITUTE
CM032587.1	1586238	.	A	G	100	*	TYPE=SUBSTITUTE
CM032587.1	1586241	.	C	T	100	*	TYPE=SUBSTITUTE
CM032587.1	1586271	.	G	A	100	*	TYPE=SUBSTITUTE
CM032587.1	1586307	.	G	A	100	*	TYPE=SUBSTITUTE
CM032587.1	1586337	.	C	T	100	*	TYPE=SUBSTITUTE
CM032587.1	1586437	.	A	G	100	*	TYPE=SUBSTITUTE

CM032587.1	1586445	.	C	T	100	*	TYPE=SUBSTITUTE
CM032587.1	1586565	.	A	G	100	*	TYPE=SUBSTITUTE
CM032587.1	1586583	.	C	T	100	*	TYPE=SUBSTITUTE
CM032587.1	1586586	.	C	T	100	*	TYPE=SUBSTITUTE
CM032587.1	1586610	.	G	C	100	*	TYPE=SUBSTITUTE
CM032587.1	1586619	.	G	A	100	*	TYPE=SUBSTITUTE
CM032587.1	1586925	.	T	c	100	*	TYPE=SUBSTITUTE
CM032587.1	1586938	.	C	t	100	*	TYPE=SUBSTITUTE
CM032587.1	1586953	.	A	G	100	*	TYPE=SUBSTITUTE
CM032587.1	1586962	.	T	C	100	*	TYPE=SUBSTITUTE
CM032587.1	1586987	.	T	C	100	*	TYPE=SUBSTITUTE
CM032587.1	1587011	.	T	C	100	*	TYPE=SUBSTITUTE
CM032587.1	1587116	.	C	t	100	*	TYPE=SUBSTITUTE
CM032587.1	1587138	.	A	G	100	*	TYPE=SUBSTITUTE
CM032587.1	1587161	.	G	C	100	*	TYPE=SUBSTITUTE
CM032587.1	1587162	.	A	G	100	*	TYPE=SUBSTITUTE
CM032587.1	1587244	.	TA	T	100	*	TYPE=DELETE
CM032587.1	1587251	.	t	tttat	100	*	TYPE=INSERT
CM032587.1	1587327	.	TT	T	100	*	TYPE=DELETE
CM032587.1	1587357	.	T	C	100	*	TYPE=SUBSTITUTE
CM032587.1	1587482	.	A	G	100	*	TYPE=SUBSTITUTE
CM032587.1	1587822	.	A	c	100	*	TYPE=SUBSTITUTE
CM032587.1	1587827	.	C	t	100	*	TYPE=SUBSTITUTE
CM032587.1	1587828	.	a	at	100	*	TYPE=INSERT
CM032587.1	1587840	.	T	c	100	*	TYPE=SUBSTITUTE
CM032587.1	1587868	.	A	g	100	*	TYPE=SUBSTITUTE
CM032587.1	1587869	.	G	a	100	*	TYPE=SUBSTITUTE
CM032587.1	1587876	.	T	c	100	*	TYPE=SUBSTITUTE
CM032587.1	1587879	.	G	a	100	*	TYPE=SUBSTITUTE
CM032587.1	1588027	.	G	T	100	*	TYPE=SUBSTITUTE
CM032587.1	1588042	.	C	T	100	*	TYPE=SUBSTITUTE
CM032587.1	1588048	.	T	G	100	*	TYPE=SUBSTITUTE
CM032587.1	1588063	.	A	G	100	*	TYPE=SUBSTITUTE
CM032587.1	1588074	.	C	T	100	*	TYPE=SUBSTITUTE
CM032587.1	1588077	.	C	T	100	*	TYPE=SUBSTITUTE
CM032587.1	1588087	.	T	G	100	*	TYPE=SUBSTITUTE
CM032587.1	1588090	.	C	T	100	*	TYPE=SUBSTITUTE
CM032587.1	1588132	.	A	G	100	*	TYPE=SUBSTITUTE
CM032587.1	1588201	.	A	G	100	*	TYPE=SUBSTITUTE
CM032587.1	1588210	.	G	A	100	*	TYPE=SUBSTITUTE
CM032587.1	1588219	.	G	A	100	*	TYPE=SUBSTITUTE
CM032587.1	1588230	.	A	G	100	*	TYPE=SUBSTITUTE
CM032587.1	1588237	.	A	G	100	*	TYPE=SUBSTITUTE
CM032587.1	1588249	.	C	T	100	*	TYPE=SUBSTITUTE
CM032587.1	1588264	.	G	C	100	*	TYPE=SUBSTITUTE
CM032587.1	1588267	.	G	A	100	*	TYPE=SUBSTITUTE
CM032587.1	1588276	.	G	A	100	*	TYPE=SUBSTITUTE
CM032587.1	1588285	.	T	C	100	*	TYPE=SUBSTITUTE
CM032587.1	1588314	.	A	G	100	*	TYPE=SUBSTITUTE
CM032587.1	1588320	.	G	T	100	*	TYPE=SUBSTITUTE
CM032587.1	1588363	.	T	C	100	*	TYPE=SUBSTITUTE
CM032587.1	1588366	.	A	G	100	*	TYPE=SUBSTITUTE
CM032587.1	1588381	.	C	t	100	*	TYPE=SUBSTITUTE
CM032587.1	1588382	.	G	c	100	*	TYPE=SUBSTITUTE
CM032587.1	1588387	.	G	c	100	*	TYPE=SUBSTITUTE
CM032587.1	1588390	.	T	a	100	*	TYPE=SUBSTITUTE
CM032587.1	1588400	.	AGGGTGATGC	A	100	*	TYPE=DELETE
CM032587.1	1588405	.	G	a	100	*	TYPE=SUBSTITUTE
CM032587.1	1588408	.	G	a	100	*	TYPE=SUBSTITUTE
CM032587.1	1588447	.	G	a	100	*	TYPE=SUBSTITUTE
CM032587.1	1588449	.	C	a	100	*	TYPE=SUBSTITUTE
CM032587.1	1588462	.	A	G	100	*	TYPE=SUBSTITUTE
CM032587.1	1588492	.	G	A	100	*	TYPE=SUBSTITUTE
CM032587.1	1588499	.	A	T	100	*	TYPE=SUBSTITUTE

CM032587.1	1588512	.	C	T	100	*	TYPE=SUBSTITUTE
CM032587.1	1588525	.	A	G	100	*	TYPE=SUBSTITUTE
CM032587.1	1588651	.	T	C	100	*	TYPE=SUBSTITUTE
CM032587.1	1588686	.	A	G	100	*	TYPE=SUBSTITUTE
CM032587.1	1588784	.	C	A	100	*	TYPE=SUBSTITUTE
CM032587.1	1588785	.	T	TG	100	*	TYPE=INSERT
CM032587.1	1588791	.	C	A	100	*	TYPE=SUBSTITUTE
CM032587.1	1588793	.	CT	C	100	*	TYPE=DELETE
CM032587.1	1588798	.	T	C	100	*	TYPE=SUBSTITUTE
CM032587.1	1588800	.	G	C	100	*	TYPE=SUBSTITUTE
CM032587.1	1588802	.	T	G	100	*	TYPE=SUBSTITUTE
CM032587.1	1588804	.	T	C	100	*	TYPE=SUBSTITUTE
CM032587.1	1588805	.	A	C	100	*	TYPE=SUBSTITUTE
CM032587.1	1588807	.	C	A	100	*	TYPE=SUBSTITUTE
CM032587.1	1588808	.	A	T	100	*	TYPE=SUBSTITUTE
CM032587.1	1588831	.	C	A	100	*	TYPE=SUBSTITUTE
CM032587.1	1588834	.	T	C	100	*	TYPE=SUBSTITUTE
CM032587.1	1588849	.	G	T	100	*	TYPE=SUBSTITUTE
CM032587.1	1588881	.	A	C	100	*	TYPE=SUBSTITUTE
CM032587.1	1588887	.	G	T	100	*	TYPE=SUBSTITUTE
CM032587.1	1588888	.	G	A	100	*	TYPE=SUBSTITUTE
CM032587.1	1588889	.	C	T	100	*	TYPE=SUBSTITUTE
CM032587.1	1588891	.	A	G	100	*	TYPE=SUBSTITUTE
CM032587.1	1588921	.	C	T	100	*	TYPE=SUBSTITUTE
CM032587.1	1588933	.	T	C	100	*	TYPE=SUBSTITUTE
CM032587.1	1588937	.	G	A	100	*	TYPE=SUBSTITUTE
CM032587.1	1588941	.	G	C	100	*	TYPE=SUBSTITUTE
CM032587.1	1588953	.	T	C	100	*	TYPE=SUBSTITUTE
CM032587.1	1589139	.	G	t	100	*	TYPE=SUBSTITUTE
CM032587.1	1589188	.	A	ATGTCTCTAGTAtatgctactatagctata	100	*	TYPE=INSERT
CM032587.1	1589194	.	G	A	100	*	TYPE=SUBSTITUTE
CM032587.1	1589213	.	T	C	100	*	TYPE=SUBSTITUTE
CM032587.1	1589266	.	T	c	100	*	TYPE=SUBSTITUTE
CM032587.1	1589280	.	C	T	100	*	TYPE=SUBSTITUTE
CM032587.1	1589319	.	GA	G	100	*	TYPE=DELETE
CM032587.1	1589324	.	A	G	100	*	TYPE=SUBSTITUTE
CM032587.1	1589704	.	AG	A	100	*	TYPE=DELETE
CM032587.1	1589729	.	T	C	100	*	TYPE=SUBSTITUTE
CM032587.1	1589738	.	A	G	100	*	TYPE=SUBSTITUTE
CM032587.1	1590079	.	C	T	100	*	TYPE=SUBSTITUTE
CM032587.1	1590562	.	C	T	100	*	TYPE=SUBSTITUTE
CM032587.1	1590994	.	C	T	100	*	TYPE=SUBSTITUTE
CM032587.1	1591243	.	C	T	100	*	TYPE=SUBSTITUTE
CM032587.1	1591337	.	TA	T	100	*	TYPE=DELETE
CM032587.1	1591384	.	A	G	100	*	TYPE=SUBSTITUTE
CM032587.1	1591408	.	TT	T	100	*	TYPE=DELETE
CM032587.1	1591529	.	TAG	T	100	*	TYPE=DELETE
CM032587.1	1591537	.	C	T	100	*	TYPE=SUBSTITUTE
CM032587.1	1591539	.	T	C	100	*	TYPE=SUBSTITUTE
CM032587.1	1591540	.	T	C	100	*	TYPE=SUBSTITUTE
CM032587.1	1591541	.	A	G	100	*	TYPE=SUBSTITUTE
CM032587.1	1591550	.	T	C	100	*	TYPE=SUBSTITUTE
CM032587.1	1591556	.	C	T	100	*	TYPE=SUBSTITUTE
CM032587.1	1591562	.	ACATAA	A	100	*	TYPE=DELETE
CM032587.1	1591648	.	C	T	100	*	TYPE=SUBSTITUTE
CM032587.1	1591657	.	T	C	100	*	TYPE=SUBSTITUTE
CM032587.1	1591662	.	A	G	100	*	TYPE=SUBSTITUTE
CM032587.1	1591664	.	A	C	100	*	TYPE=SUBSTITUTE
CM032587.1	1591665	.	T	C	100	*	TYPE=SUBSTITUTE
CM032587.1	1591672	.	C	T	100	*	TYPE=SUBSTITUTE
CM032587.1	1591676	.	A	G	100	*	TYPE=SUBSTITUTE
CM032587.1	1591716	.	T	A	100	*	TYPE=SUBSTITUTE
CM032587.1	1591742	.	G	A	100	*	TYPE=SUBSTITUTE

CM032587.1	1591770	.	G	a	100	*	TYPE=SUBSTITUTE
CM032587.1	1591776	.	A	AG	100	*	TYPE=INSERT
CM032587.1	1591781	.	C	T	100	*	TYPE=SUBSTITUTE
CM032587.1	1591842	.	GG	G	100	*	TYPE=DELETE
CM032587.1	1591911	.	A	AA	100	*	TYPE=INSERT
CM032587.1	1591931	.	T	C	100	*	TYPE=SUBSTITUTE
CM032587.1	1591933	.	A	G	100	*	TYPE=SUBSTITUTE
CM032587.1	1591943	.	A	C	100	*	TYPE=SUBSTITUTE
CM032587.1	1591944	.	A	T	100	*	TYPE=SUBSTITUTE
CM032587.1	1591949	.	C	T	100	*	TYPE=SUBSTITUTE
CM032587.1	1591956	.	A	T	100	*	TYPE=SUBSTITUTE
CM032587.1	1591965	.	T	TT	100	*	TYPE=INSERT
CM032587.1	1592051	.	T	C	100	*	TYPE=SUBSTITUTE
CM032587.1	1592102	.	T	C	100	*	TYPE=SUBSTITUTE
CM032587.1	1592245	.	G	A	100	*	TYPE=SUBSTITUTE
CM032587.1	1592246	.	A	AAT	100	*	TYPE=INSERT
CM032587.1	1592247	.	C	G	100	*	TYPE=SUBSTITUTE
CM032587.1	1592251	.	A	ACGGC	100	*	TYPE=INSERT
CM032587.1	1592253	.	C	CTCCAGCCGCTGG	100	*	TYPE=INSERT
CM032587.1	1592441	.	C	T	100	*	TYPE=SUBSTITUTE
CM032587.1	1592523	.	TT	T	100	*	TYPE=DELETE
CM032587.1	1592538	.	T	C	100	*	TYPE=SUBSTITUTE
CM032587.1	1592574	.	A	ACA	100	*	TYPE=INSERT
CM032587.1	1592853	.	G	T	100	*	TYPE=SUBSTITUTE
CM032587.1	1592983	.	C	T	100	*	TYPE=SUBSTITUTE
CM032587.1	1593128	.	T	C	100	*	TYPE=SUBSTITUTE
CM032587.1	1593129	.	C	T	100	*	TYPE=SUBSTITUTE
CM032587.1	1593163	.	T	c	100	*	TYPE=SUBSTITUTE
CM032587.1	1593179	.	A	G	100	*	TYPE=SUBSTITUTE
CM032587.1	1593215	.	G	A	100	*	TYPE=SUBSTITUTE
CM032587.1	1593261	.	A	G	100	*	TYPE=SUBSTITUTE
CM032587.1	1593270	.	T	TGCATGA	100	*	TYPE=INSERT
CM032587.1	1593288	.	A	G	100	*	TYPE=SUBSTITUTE
CM032587.1	1593293	.	T	C	100	*	TYPE=SUBSTITUTE
CM032587.1	1593386	.	C	T	100	*	TYPE=SUBSTITUTE
CM032587.1	1593389	.	T	C	100	*	TYPE=SUBSTITUTE
CM032587.1	1593419	.	T	C	100	*	TYPE=SUBSTITUTE
CM032587.1	1593473	.	T	C	100	*	TYPE=SUBSTITUTE
CM032587.1	1593518	.	G	A	100	*	TYPE=SUBSTITUTE
CM032587.1	1593710	.	C	A	100	*	TYPE=SUBSTITUTE
CM032587.1	1593734	.	G	A	100	*	TYPE=SUBSTITUTE
CM032587.1	1593741	.	G	A	100	*	TYPE=SUBSTITUTE
CM032587.1	1593761	.	A	G	100	*	TYPE=SUBSTITUTE
CM032587.1	1593770	.	T	C	100	*	TYPE=SUBSTITUTE
CM032587.1	1593785	.	C	T	100	*	TYPE=SUBSTITUTE
CM032587.1	1593797	.	C	T	100	*	TYPE=SUBSTITUTE
CM032587.1	1593836	.	T	C	100	*	TYPE=SUBSTITUTE
CM032587.1	1593848	.	G	A	100	*	TYPE=SUBSTITUTE
CM032587.1	1593857	.	T	C	100	*	TYPE=SUBSTITUTE
CM032587.1	1593875	.	G	T	100	*	TYPE=SUBSTITUTE
CM032587.1	1593899	.	A	T	100	*	TYPE=SUBSTITUTE
CM032587.1	1593902	.	A	G	100	*	TYPE=SUBSTITUTE
CM032587.1	1593904	.	C	A	100	*	TYPE=SUBSTITUTE
CM032587.1	1593913	.	A	G	100	*	TYPE=SUBSTITUTE
CM032587.1	1593936	.	A	G	100	*	TYPE=SUBSTITUTE
CM032587.1	1593962	.	T	C	100	*	TYPE=SUBSTITUTE
CM032587.1	1593965	.	C	T	100	*	TYPE=SUBSTITUTE
CM032587.1	1594052	.	T	C	100	*	TYPE=SUBSTITUTE
CM032587.1	1594082	.	A	G	100	*	TYPE=SUBSTITUTE
CM032587.1	1594118	.	A	G	100	*	TYPE=SUBSTITUTE
CM032587.1	1594130	.	T	C	100	*	TYPE=SUBSTITUTE
CM032587.1	1594154	.	A	G	100	*	TYPE=SUBSTITUTE
CM032587.1	1594226	.	T	C	100	*	TYPE=SUBSTITUTE
CM032587.1	1594232	.	C	T	100	*	TYPE=SUBSTITUTE

CM032587.1	1594241	.	A	G	100	*	TYPE=SUBSTITUTE
CM032587.1	1594256	.	T	C	100	*	TYPE=SUBSTITUTE
CM032587.1	1594259	.	A	T	100	*	TYPE=SUBSTITUTE
CM032587.1	1594272	.	CA	C	100	*	TYPE=DELETE
CM032587.1	1594277	.	A	G	100	*	TYPE=SUBSTITUTE
CM032587.1	1594281	.	T	G	100	*	TYPE=SUBSTITUTE
CM032587.1	1594284	.	T	A	100	*	TYPE=SUBSTITUTE
CM032587.1	1594304	.	C	T	100	*	TYPE=SUBSTITUTE
CM032587.1	1594306	.	T	A	100	*	TYPE=SUBSTITUTE
CM032587.1	1594312	.	C	T	100	*	TYPE=SUBSTITUTE
CM032587.1	1594318	.	C	A	100	*	TYPE=SUBSTITUTE
CM032587.1	1594321	.	A	AATAC	100	*	TYPE=INSERT
CM032587.1	1594331	.	C	T	100	*	TYPE=SUBSTITUTE
CM032587.1	1594333	.	C	T	100	*	TYPE=SUBSTITUTE
CM032587.1	1594352	.	C	T	100	*	TYPE=SUBSTITUTE
CM032587.1	1594364	.	G	A	100	*	TYPE=SUBSTITUTE
CM032587.1	1594370	.	C	T	100	*	TYPE=SUBSTITUTE
CM032587.1	1594379	.	A	G	100	*	TYPE=SUBSTITUTE
CM032587.1	1594382	.	C	T	100	*	TYPE=SUBSTITUTE
CM032587.1	1594397	.	T	C	100	*	TYPE=SUBSTITUTE
CM032587.1	1594406	.	C	A	100	*	TYPE=SUBSTITUTE
CM032587.1	1594412	.	A	C	100	*	TYPE=SUBSTITUTE
CM032587.1	1594414	.	T	C	100	*	TYPE=SUBSTITUTE
CM032587.1	1594442	.	C	T	100	*	TYPE=SUBSTITUTE
CM032587.1	1594469	.	T	C	100	*	TYPE=SUBSTITUTE
CM032587.1	1594502	.	G	A	100	*	TYPE=SUBSTITUTE
CM032587.1	1594538	.	C	T	100	*	TYPE=SUBSTITUTE
CM032587.1	1594547	.	A	T	100	*	TYPE=SUBSTITUTE
CM032587.1	1594555	.	TA	T	100	*	TYPE=DELETE
CM032587.1	1594558	.	T	C	100	*	TYPE=SUBSTITUTE
CM032587.1	1594563	.	T	C	100	*	TYPE=SUBSTITUTE
CM032587.1	1594568	.	G	A	100	*	TYPE=SUBSTITUTE
CM032587.1	1594575	.	C	T	100	*	TYPE=SUBSTITUTE
CM032587.1	1594577	.	A	G	100	*	TYPE=SUBSTITUTE
CM032587.1	1594583	.	A	T	100	*	TYPE=SUBSTITUTE
CM032587.1	1594584	.	A	T	100	*	TYPE=SUBSTITUTE
CM032587.1	1594589	.	T	g	100	*	TYPE=SUBSTITUTE
CM032587.1	1594590	.	A	g	100	*	TYPE=SUBSTITUTE
CM032587.1	1594594	.	C	a	100	*	TYPE=SUBSTITUTE
CM032587.1	1594598	.	G	a	100	*	TYPE=SUBSTITUTE
CM032587.1	1594604	.	A	AG	100	*	TYPE=INSERT
CM032587.1	1594607	.	C	A	100	*	TYPE=SUBSTITUTE
CM032587.1	1594649	.	T	C	100	*	TYPE=SUBSTITUTE
CM032587.1	1594694	.	G	A	100	*	TYPE=SUBSTITUTE
CM032587.1	1594730	.	A	G	100	*	TYPE=SUBSTITUTE
CM032587.1	1594760	.	T	C	100	*	TYPE=SUBSTITUTE
CM032587.1	1594769	.	A	C	100	*	TYPE=SUBSTITUTE
CM032587.1	1594790	.	T	A	100	*	TYPE=SUBSTITUTE
CM032587.1	1594802	.	T	c	100	*	TYPE=SUBSTITUTE
CM032587.1	1594832	.	A	G	100	*	TYPE=SUBSTITUTE
CM032587.1	1594838	.	G	A	100	*	TYPE=SUBSTITUTE
CM032587.1	1594847	.	A	G	100	*	TYPE=SUBSTITUTE
CM032587.1	1594850	.	T	C	100	*	TYPE=SUBSTITUTE
CM032587.1	1594859	.	T	C	100	*	TYPE=SUBSTITUTE
CM032587.1	1594862	.	G	A	100	*	TYPE=SUBSTITUTE
CM032587.1	1594898	.	A	G	100	*	TYPE=SUBSTITUTE
CM032587.1	1594950	.	C	A	100	*	TYPE=SUBSTITUTE
CM032587.1	1594952	.	C	T	100	*	TYPE=SUBSTITUTE
CM032587.1	1594954	.	G	A	100	*	TYPE=SUBSTITUTE
CM032587.1	1594961	.	T	C	100	*	TYPE=SUBSTITUTE
CM032587.1	1594965	.	G	C	100	*	TYPE=SUBSTITUTE
CM032587.1	1594977	.	C	T	100	*	TYPE=SUBSTITUTE
CM032587.1	1595021	.	C	T	100	*	TYPE=SUBSTITUTE
CM032587.1	1595027	.	C	T	100	*	TYPE=SUBSTITUTE

CM032587.1	1595036	.	T	C	100	*	TYPE=SUBSTITUTE
CM032587.1	1595072	.	C	T	100	*	TYPE=SUBSTITUTE
CM032587.1	1595080	.	T	C	100	*	TYPE=SUBSTITUTE
CM032587.1	1595084	.	GCT	G	100	*	TYPE=DELETE
CM032587.1	1595085	.	G	A	100	*	TYPE=SUBSTITUTE
CM032587.1	1595087	.	G	A	100	*	TYPE=SUBSTITUTE
CM032587.1	1595092	.	T	C	100	*	TYPE=SUBSTITUTE
CM032587.1	1595094	.	T	G	100	*	TYPE=SUBSTITUTE
CM032587.1	1595100	.	G	A	100	*	TYPE=SUBSTITUTE
CM032587.1	1595101	.	A	G	100	*	TYPE=SUBSTITUTE
CM032587.1	1595103	.	A	G	100	*	TYPE=SUBSTITUTE
CM032587.1	1595105	.	G	A	100	*	TYPE=SUBSTITUTE
CM032587.1	1595112	.	A	T	100	*	TYPE=SUBSTITUTE
CM032587.1	1595113	.	G	A	100	*	TYPE=SUBSTITUTE
CM032587.1	1595114	.	T	C	100	*	TYPE=SUBSTITUTE
CM032587.1	1595118	.	G	C	100	*	TYPE=SUBSTITUTE
CM032587.1	1595152	.	A	G	100	*	TYPE=SUBSTITUTE
CM032587.1	1595188	.	T	C	100	*	TYPE=SUBSTITUTE
CM032587.1	1595220	.	A	T	100	*	TYPE=SUBSTITUTE
CM032587.1	1595222	.	G	A	100	*	TYPE=SUBSTITUTE
CM032587.1	1595236	.	T	C	100	*	TYPE=SUBSTITUTE
CM032587.1	1595269	.	C	T	100	*	TYPE=SUBSTITUTE
CM032587.1	1595275	.	T	C	100	*	TYPE=SUBSTITUTE
CM032587.1	1595295	.	A	C	100	*	TYPE=SUBSTITUTE
CM032587.1	1595330	.	T	G	100	*	TYPE=SUBSTITUTE
CM032587.1	1595345	.	C	T	100	*	TYPE=SUBSTITUTE
CM032587.1	1595369	.	CA	C	100	*	TYPE=DELETE
CM032587.1	1595373	.	C	T	100	*	TYPE=SUBSTITUTE
CM032587.1	1595376	.	T	TGATCCC	100	*	TYPE=INSERT
CM032587.1	1595402	.	A	G	100	*	TYPE=SUBSTITUTE
CM032587.1	1595441	.	A	G	100	*	TYPE=SUBSTITUTE
CM032587.1	1595457	.	A	G	100	*	TYPE=SUBSTITUTE
CM032587.1	1595472	.	G	A	100	*	TYPE=SUBSTITUTE
CM032587.1	1595496	.	G	GG	100	*	TYPE=INSERT
CM032587.1	1595530	.	A	AA	100	*	TYPE=INSERT
CM032587.1	1595552	.	T	C	100	*	TYPE=SUBSTITUTE
CM032587.1	1595554	.	GT	G	100	*	TYPE=DELETE
CM032587.1	1595567	.	T	C	100	*	TYPE=SUBSTITUTE
CM032587.1	1595696	.	T	G	100	*	TYPE=SUBSTITUTE
CM032587.1	1595720	.	C	A	100	*	TYPE=SUBSTITUTE
CM032587.1	1595721	.	T	C	100	*	TYPE=SUBSTITUTE
CM032587.1	1595755	.	C	T	100	*	TYPE=SUBSTITUTE
CM032587.1	1595756	.	C	T	100	*	TYPE=SUBSTITUTE
CM032587.1	1595880	.	CA	C	100	*	TYPE=DELETE
CM032587.1	1595893	.	T	G	100	*	TYPE=SUBSTITUTE
CM032587.1	1595894	.	T	G	100	*	TYPE=SUBSTITUTE
CM032587.1	1595896	.	T	A	100	*	TYPE=SUBSTITUTE
CM032587.1	1595909	.	T	c	100	*	TYPE=SUBSTITUTE
CM032587.1	1595937	.	C	G	100	*	TYPE=SUBSTITUTE
CM032587.1	1595978	.	C	T	100	*	TYPE=SUBSTITUTE
CM032587.1	1595982	.	T	C	100	*	TYPE=SUBSTITUTE
CM032587.1	1595994	.	T	C	100	*	TYPE=SUBSTITUTE
CM032587.1	1596051	.	C	A	100	*	TYPE=SUBSTITUTE
CM032587.1	1596057	.	T	TCA	100	*	TYPE=INSERT
CM032587.1	1596059	.	G	A	100	*	TYPE=SUBSTITUTE
CM032587.1	1596061	.	A	G	100	*	TYPE=SUBSTITUTE
CM032587.1	1596073	.	C	T	100	*	TYPE=SUBSTITUTE
CM032587.1	1596100	.	C	T	100	*	TYPE=SUBSTITUTE
CM032587.1	1596104	.	CGT	C	100	*	TYPE=DELETE
CM032587.1	1596105	.	A	T	100	*	TYPE=SUBSTITUTE
CM032587.1	1596106	.	A	G	100	*	TYPE=SUBSTITUTE
CM032587.1	1596108	.	G	T	100	*	TYPE=SUBSTITUTE
CM032587.1	1596109	.	T	A	100	*	TYPE=SUBSTITUTE
CM032587.1	1596125	.	T	C	100	*	TYPE=SUBSTITUTE

CM032587.1	1596130	.	A	C	100	*	TYPE=SUBSTITUTE
CM032587.1	1596154	.	G	T	100	*	TYPE=SUBSTITUTE
CM032587.1	1596166	.	TTAGAC	T	100	*	TYPE=DELETE
CM032587.1	1596176	.	G	T	100	*	TYPE=SUBSTITUTE
CM032587.1	1596178	.	C	T	100	*	TYPE=SUBSTITUTE
CM032587.1	1596187	.	T	A	100	*	TYPE=SUBSTITUTE
CM032587.1	1596243	.	A	G	100	*	TYPE=SUBSTITUTE
CM032587.1	1596260	.	T	C	100	*	TYPE=SUBSTITUTE
CM032587.1	1596342	.	T	C	100	*	TYPE=SUBSTITUTE
CM032587.1	1596362	.	AG	A	100	*	TYPE=DELETE
CM032587.1	1596432	.	C	T	100	*	TYPE=SUBSTITUTE
CM032587.1	1596457	.	A	G	100	*	TYPE=SUBSTITUTE
CM032587.1	1596461	.	GA	G	100	*	TYPE=DELETE
CM032587.1	1596469	.	C	T	100	*	TYPE=SUBSTITUTE
CM032587.1	1596508	.	T	C	100	*	TYPE=SUBSTITUTE
CM032587.1	1596519	.	G	A	100	*	TYPE=SUBSTITUTE
CM032587.1	1596534	.	A	G	100	*	TYPE=SUBSTITUTE
CM032587.1	1596583	.	A	G	100	*	TYPE=SUBSTITUTE
CM032587.1	1596593	.	C	T	100	*	TYPE=SUBSTITUTE
CM032587.1	1596608	.	T	C	100	*	TYPE=SUBSTITUTE
CM032587.1	1596622	.	T	C	100	*	TYPE=SUBSTITUTE
CM032587.1	1596630	.	C	T	100	*	TYPE=SUBSTITUTE
CM032587.1	1596642	.	C	G	100	*	TYPE=SUBSTITUTE
CM032587.1	1596648	.	C	T	100	*	TYPE=SUBSTITUTE
CM032587.1	1596650	.	G	A	100	*	TYPE=SUBSTITUTE
CM032587.1	1596703	.	ATGTT	A	100	*	TYPE=DELETE
CM032587.1	1596710	.	C	T	100	*	TYPE=SUBSTITUTE
CM032587.1	1596724	.	AC	A	100	*	TYPE=DELETE
CM032587.1	1596726	.	A	C	100	*	TYPE=SUBSTITUTE
CM032587.1	1596730	.	G	A	100	*	TYPE=SUBSTITUTE
CM032587.1	1596739	.	C	T	100	*	TYPE=SUBSTITUTE
CM032587.1	1596766	.	T	A	100	*	TYPE=SUBSTITUTE
CM032587.1	1596770	.	C	T	100	*	TYPE=SUBSTITUTE
CM032587.1	1596776	.	G	C	100	*	TYPE=SUBSTITUTE
CM032587.1	1596777	.	C	T	100	*	TYPE=SUBSTITUTE
CM032587.1	1596783	.	T	A	100	*	TYPE=SUBSTITUTE
CM032587.1	1596799	.	G	A	100	*	TYPE=SUBSTITUTE
CM032587.1	1596804	.	C	T	100	*	TYPE=SUBSTITUTE
CM032587.1	1596805	.	T	C	100	*	TYPE=SUBSTITUTE
CM032587.1	1596844	.	A	G	100	*	TYPE=SUBSTITUTE
CM032587.1	1596860	.	C	T	100	*	TYPE=SUBSTITUTE
CM032587.1	1596913	.	T	G	100	*	TYPE=SUBSTITUTE
CM032587.1	1596937	.	C	T	100	*	TYPE=SUBSTITUTE
CM032587.1	1597024	.	C	T	100	*	TYPE=SUBSTITUTE
CM032587.1	1597033	.	T	C	100	*	TYPE=SUBSTITUTE
CM032587.1	1597100	.	C	G	100	*	TYPE=SUBSTITUTE
CM032587.1	1597133	.	A	G	100	*	TYPE=SUBSTITUTE
CM032587.1	1597160	.	T	C	100	*	TYPE=SUBSTITUTE
CM032587.1	1597166	.	C	T	100	*	TYPE=SUBSTITUTE
CM032587.1	1597187	.	A	g	100	*	TYPE=SUBSTITUTE
CM032587.1	1597203	.	C	T	100	*	TYPE=SUBSTITUTE
CM032587.1	1597223	.	T	C	100	*	TYPE=SUBSTITUTE
CM032587.1	1597268	.	T	C	100	*	TYPE=SUBSTITUTE
CM032587.1	1597280	.	T	G	100	*	TYPE=SUBSTITUTE
CM032587.1	1597301	.	T	C	100	*	TYPE=SUBSTITUTE
CM032587.1	1597303	.	G	C	100	*	TYPE=SUBSTITUTE
CM032587.1	1597307	.	G	A	100	*	TYPE=SUBSTITUTE
CM032587.1	1597311	.	A	g	100	*	TYPE=SUBSTITUTE
CM032587.1	1597333	.	T	g	100	*	TYPE=SUBSTITUTE
CM032587.1	1597373	.	A	G	100	*	TYPE=SUBSTITUTE
CM032587.1	1597400	.	C	T	100	*	TYPE=SUBSTITUTE
CM032587.1	1597402	.	A	G	100	*	TYPE=SUBSTITUTE
CM032587.1	1597454	.	G	A	100	*	TYPE=SUBSTITUTE
CM032587.1	1597467	.	G	A	100	*	TYPE=SUBSTITUTE

CM032587.1	1597487	.	T	C	100	*	TYPE=SUBSTITUTE
CM032587.1	1597495	.	T	C	100	*	TYPE=SUBSTITUTE
CM032587.1	1597496	.	T	C	100	*	TYPE=SUBSTITUTE
CM032587.1	1597520	.	A	G	100	*	TYPE=SUBSTITUTE
CM032587.1	1597556	.	A	G	100	*	TYPE=SUBSTITUTE
CM032587.1	1597560	.	T	C	100	*	TYPE=SUBSTITUTE
CM032587.1	1597572	.	G	A	100	*	TYPE=SUBSTITUTE
CM032587.1	1597578	.	C	T	100	*	TYPE=SUBSTITUTE
CM032587.1	1597579	.	G	A	100	*	TYPE=SUBSTITUTE
CM032587.1	1597596	.	G	A	100	*	TYPE=SUBSTITUTE
CM032587.1	1597636	.	C	A	100	*	TYPE=SUBSTITUTE
CM032587.1	1597706	.	A	G	100	*	TYPE=SUBSTITUTE
CM032587.1	1597736	.	A	G	100	*	TYPE=SUBSTITUTE
CM032587.1	1597751	.	T	C	100	*	TYPE=SUBSTITUTE
CM032587.1	1597898	.	C	T	100	*	TYPE=SUBSTITUTE
CM032587.1	1597903	.	C	T	100	*	TYPE=SUBSTITUTE
CM032587.1	1597916	.	A	T	100	*	TYPE=SUBSTITUTE
CM032587.1	1597920	.	A	G	100	*	TYPE=SUBSTITUTE
CM032587.1	1597935	.	T	C	100	*	TYPE=SUBSTITUTE
CM032587.1	1597936	.	C	T	100	*	TYPE=SUBSTITUTE
CM032587.1	1597943	.	G	A	100	*	TYPE=SUBSTITUTE
CM032587.1	1597949	.	T	C	100	*	TYPE=SUBSTITUTE
CM032587.1	1597971	.	A	G	100	*	TYPE=SUBSTITUTE
CM032587.1	1597972	.	A	C	100	*	TYPE=SUBSTITUTE
CM032587.1	1597979	.	T	C	100	*	TYPE=SUBSTITUTE
CM032587.1	1598006	.	G	A	100	*	TYPE=SUBSTITUTE
CM032587.1	1598036	.	G	A	100	*	TYPE=SUBSTITUTE
CM032587.1	1598096	.	C	T	100	*	TYPE=SUBSTITUTE
CM032587.1	1598099	.	T	C	100	*	TYPE=SUBSTITUTE
CM032587.1	1598105	.	G	A	100	*	TYPE=SUBSTITUTE
CM032587.1	1598111	.	C	T	100	*	TYPE=SUBSTITUTE
CM032587.1	1598129	.	G	A	100	*	TYPE=SUBSTITUTE
CM032587.1	1598144	.	T	G	100	*	TYPE=SUBSTITUTE
CM032587.1	1598189	.	C	T	100	*	TYPE=SUBSTITUTE
CM032587.1	1598240	.	T	C	100	*	TYPE=SUBSTITUTE
CM032587.1	1598279	.	C	T	100	*	TYPE=SUBSTITUTE
CM032587.1	1598321	.	C	T	100	*	TYPE=SUBSTITUTE
CM032587.1	1598330	.	T	C	100	*	TYPE=SUBSTITUTE
CM032587.1	1598393	.	A	G	100	*	TYPE=SUBSTITUTE
CM032587.1	1598417	.	T	C	100	*	TYPE=SUBSTITUTE
CM032587.1	1598456	.	C	T	100	*	TYPE=SUBSTITUTE
CM032587.1	1598660	.	G	A	100	*	TYPE=SUBSTITUTE
CM032587.1	1598668	.	T	C	100	*	TYPE=SUBSTITUTE
CM032587.1	1598744	.	G	A	100	*	TYPE=SUBSTITUTE
CM032587.1	1598846	.	C	T	100	*	TYPE=SUBSTITUTE
CM032587.1	1599047	.	T	C	100	*	TYPE=SUBSTITUTE
CM032587.1	1599122	.	T	C	100	*	TYPE=SUBSTITUTE
CM032587.1	1599212	.	A	G	100	*	TYPE=SUBSTITUTE
CM032587.1	1599214	.	C	T	100	*	TYPE=SUBSTITUTE
CM032587.1	1599274	.	A	G	100	*	TYPE=SUBSTITUTE
CM032587.1	1599305	.	T	C	100	*	TYPE=SUBSTITUTE
CM032587.1	1599352	.	A	G	100	*	TYPE=SUBSTITUTE
CM032587.1	1599377	.	A	G	100	*	TYPE=SUBSTITUTE
CM032587.1	1599398	.	T	G	100	*	TYPE=SUBSTITUTE
CM032587.1	1599420	.	G	C	100	*	TYPE=SUBSTITUTE
CM032587.1	1599432	.	C	T	100	*	TYPE=SUBSTITUTE
CM032587.1	1599449	.	A	T	100	*	TYPE=SUBSTITUTE
CM032587.1	1599462	.	T	C	100	*	TYPE=SUBSTITUTE
CM032587.1	1599485	.	T	C	100	*	TYPE=SUBSTITUTE
CM032587.1	1599505	.	C	T	100	*	TYPE=SUBSTITUTE
CM032587.1	1599566	.	T	C	100	*	TYPE=SUBSTITUTE
CM032587.1	1599605	.	G	A	100	*	TYPE=SUBSTITUTE
CM032587.1	1599617	.	T	C	100	*	TYPE=SUBSTITUTE
CM032587.1	1599620	.	T	G	100	*	TYPE=SUBSTITUTE

CM032587.1	1599623	.	A	G	100	*	TYPE=SUBSTITUTE
CM032587.1	1599632	.	A	G	100	*	TYPE=SUBSTITUTE
CM032587.1	1599652	.	C	A	100	*	TYPE=SUBSTITUTE
CM032587.1	1599653	.	T	C	100	*	TYPE=SUBSTITUTE
CM032587.1	1599654	.	T	A	100	*	TYPE=SUBSTITUTE
CM032587.1	1599696	.	C	A	100	*	TYPE=SUBSTITUTE
CM032587.1	1599700	.	CGGA	C	100	*	TYPE=DELETE
CM032587.1	1599701	.	T	C	100	*	TYPE=SUBSTITUTE
CM032587.1	1599741	.	C	T	100	*	TYPE=SUBSTITUTE
CM032587.1	1599760	.	T	G	100	*	TYPE=SUBSTITUTE
CM032587.1	1599791	.	T	C	100	*	TYPE=SUBSTITUTE
CM032587.1	1599792	.	C	A	100	*	TYPE=SUBSTITUTE
CM032587.1	1599795	.	A	G	100	*	TYPE=SUBSTITUTE
CM032587.1	1599796	.	T	C	100	*	TYPE=SUBSTITUTE
CM032587.1	1599807	.	TC	T	100	*	TYPE=DELETE
CM032587.1	1599810	.	G	A	100	*	TYPE=SUBSTITUTE
CM032587.1	1599814	.	T	C	100	*	TYPE=SUBSTITUTE
CM032587.1	1599815	.	C	A	100	*	TYPE=SUBSTITUTE
CM032587.1	1599820	.	G	A	100	*	TYPE=SUBSTITUTE
CM032587.1	1599838	.	A	G	100	*	TYPE=SUBSTITUTE
CM032587.1	1599854	.	c	ct	100	*	TYPE=INSERT
CM032587.1	1599863	.	G	a	100	*	TYPE=SUBSTITUTE
CM032587.1	1599897	.	A	C	100	*	TYPE=SUBSTITUTE
CM032587.1	1599943	.	C	T	100	*	TYPE=SUBSTITUTE
CM032587.1	1600099	.	G	A	100	*	TYPE=SUBSTITUTE
CM032587.1	1600102	.	C	T	100	*	TYPE=SUBSTITUTE
CM032587.1	1600211	.	A	C	100	*	TYPE=SUBSTITUTE
CM032587.1	1600215	.	G	C	100	*	TYPE=SUBSTITUTE
CM032587.1	1600230	.	G	T	100	*	TYPE=SUBSTITUTE
CM032587.1	1600322	.	T	G	100	*	TYPE=SUBSTITUTE
CM032587.1	1600331	.	G	A	100	*	TYPE=SUBSTITUTE
CM032587.1	1600344	.	G	A	100	*	TYPE=SUBSTITUTE
CM032587.1	1600382	.	C	T	100	*	TYPE=SUBSTITUTE
CM032587.1	1600422	.	C	G	100	*	TYPE=SUBSTITUTE
CM032587.1	1600436	.	G	T	100	*	TYPE=SUBSTITUTE
CM032587.1	1600448	.	A	G	100	*	TYPE=SUBSTITUTE
CM032587.1	1600536	.	C	T	100	*	TYPE=SUBSTITUTE
CM032587.1	1600538	.	A	G	100	*	TYPE=SUBSTITUTE
CM032587.1	1600550	.	T	A	100	*	TYPE=SUBSTITUTE
CM032587.1	1600568	.	T	C	100	*	TYPE=SUBSTITUTE
CM032587.1	1600601	.	C	T	100	*	TYPE=SUBSTITUTE
CM032587.1	1600638	.	C	T	100	*	TYPE=SUBSTITUTE
CM032587.1	1600652	.	C	A	100	*	TYPE=SUBSTITUTE
CM032587.1	1600727	.	C	T	100	*	TYPE=SUBSTITUTE
CM032587.1	1600733	.	A	G	100	*	TYPE=SUBSTITUTE
CM032587.1	1600787	.	G	T	100	*	TYPE=SUBSTITUTE
CM032587.1	1600853	.	C	T	100	*	TYPE=SUBSTITUTE
CM032587.1	1600964	.	T	C	100	*	TYPE=SUBSTITUTE
CM032587.1	1600982	.	T	C	100	*	TYPE=SUBSTITUTE
CM032587.1	1600985	.	G	A	100	*	TYPE=SUBSTITUTE
CM032587.1	1600993	.	T	C	100	*	TYPE=SUBSTITUTE
CM032587.1	1601008	.	C	T	100	*	TYPE=SUBSTITUTE
CM032587.1	1601011	.	C	T	100	*	TYPE=SUBSTITUTE
CM032587.1	1601024	.	AG	A	100	*	TYPE=DELETE
CM032587.1	1601025	.	G	C	100	*	TYPE=SUBSTITUTE
CM032587.1	1601064	.	A	T	100	*	TYPE=SUBSTITUTE
CM032587.1	1601071	.	A	T	100	*	TYPE=SUBSTITUTE
CM032587.1	1601073	.	A	G	100	*	TYPE=SUBSTITUTE
CM032587.1	1601124	.	G	A	100	*	TYPE=SUBSTITUTE
CM032587.1	1601289	.	T	a	100	*	TYPE=SUBSTITUTE
CM032587.1	1601346	.	G	A	100	*	TYPE=SUBSTITUTE
CM032587.1	1601353	.	G	A	100	*	TYPE=SUBSTITUTE
CM032587.1	1601367	.	A	G	100	*	TYPE=SUBSTITUTE
CM032587.1	1601379	.	C	A	100	*	TYPE=SUBSTITUTE

CM032587.1	1601382	.	G	A	100	*	TYPE=SUBSTITUTE
CM032587.1	1601403	.	C	T	100	*	TYPE=SUBSTITUTE
CM032587.1	1601415	.	A	G	100	*	TYPE=SUBSTITUTE
CM032587.1	1601448	.	G	A	100	*	TYPE=SUBSTITUTE
CM032587.1	1601496	.	A	G	100	*	TYPE=SUBSTITUTE
CM032587.1	1601523	.	A	G	100	*	TYPE=SUBSTITUTE
CM032587.1	1601535	.	G	A	100	*	TYPE=SUBSTITUTE
CM032587.1	1601607	.	A	G	100	*	TYPE=SUBSTITUTE
CM032587.1	1601661	.	A	G	100	*	TYPE=SUBSTITUTE
CM032587.1	1601688	.	T	C	100	*	TYPE=SUBSTITUTE
CM032587.1	1601715	.	G	A	100	*	TYPE=SUBSTITUTE
CM032587.1	1601760	.	C	T	100	*	TYPE=SUBSTITUTE
CM032587.1	1601763	.	G	A	100	*	TYPE=SUBSTITUTE
CM032587.1	1601778	.	C	G	100	*	TYPE=SUBSTITUTE
CM032587.1	1601808	.	G	A	100	*	TYPE=SUBSTITUTE
CM032587.1	1601820	.	T	C	100	*	TYPE=SUBSTITUTE
CM032587.1	1601838	.	T	C	100	*	TYPE=SUBSTITUTE
CM032587.1	1601883	.	T	A	100	*	TYPE=SUBSTITUTE
CM032587.1	1601982	.	T	C	100	*	TYPE=SUBSTITUTE
CM032587.1	1601994	.	C	T	100	*	TYPE=SUBSTITUTE
CM032587.1	1602070	.	C	T	100	*	TYPE=SUBSTITUTE
CM032587.1	1602075	.	C	T	100	*	TYPE=SUBSTITUTE
CM032587.1	1602108	.	C	T	100	*	TYPE=SUBSTITUTE
CM032587.1	1602135	.	A	g	100	*	TYPE=SUBSTITUTE
CM032587.1	1602171	.	C	G	100	*	TYPE=SUBSTITUTE
CM032587.1	1602183	.	C	T	100	*	TYPE=SUBSTITUTE
CM032587.1	1602192	.	G	A	100	*	TYPE=SUBSTITUTE
CM032587.1	1602201	.	G	A	100	*	TYPE=SUBSTITUTE
CM032587.1	1602207	.	C	A	100	*	TYPE=SUBSTITUTE
CM032587.1	1602225	.	G	A	100	*	TYPE=SUBSTITUTE
CM032587.1	1602228	.	G	A	100	*	TYPE=SUBSTITUTE
CM032587.1	1602231	.	A	G	100	*	TYPE=SUBSTITUTE
CM032587.1	1602246	.	A	G	100	*	TYPE=SUBSTITUTE
CM032587.1	1602258	.	T	C	100	*	TYPE=SUBSTITUTE
CM032587.1	1602261	.	T	C	100	*	TYPE=SUBSTITUTE
CM032587.1	1602312	.	G	A	100	*	TYPE=SUBSTITUTE
CM032587.1	1602414	.	C	T	100	*	TYPE=SUBSTITUTE
CM032587.1	1602468	.	A	G	100	*	TYPE=SUBSTITUTE
CM032587.1	1602516	.	T	C	100	*	TYPE=SUBSTITUTE
CM032587.1	1602540	.	G	A	100	*	TYPE=SUBSTITUTE
CM032587.1	1602549	.	T	C	100	*	TYPE=SUBSTITUTE
CM032587.1	1602651	.	T	G	100	*	TYPE=SUBSTITUTE
CM032587.1	1602657	.	A	G	100	*	TYPE=SUBSTITUTE
CM032587.1	1602870	.	A	T	100	*	TYPE=SUBSTITUTE
CM032587.1	1602904	.	C	T	100	*	TYPE=SUBSTITUTE
CM032587.1	1602905	.	A	C	100	*	TYPE=SUBSTITUTE
CM032587.1	1602926	.	T	C	100	*	TYPE=SUBSTITUTE
CM032587.1	1602971	.	C	A	100	*	TYPE=SUBSTITUTE
CM032587.1	1602975	.	G	A	100	*	TYPE=SUBSTITUTE
CM032587.1	1603059	.	GG	G	100	*	TYPE=DELETE
CM032587.1	1603112	.	A	G	100	*	TYPE=SUBSTITUTE
CM032587.1	1603137	.	T	C	100	*	TYPE=SUBSTITUTE
CM032587.1	1603157	.	ATGAAGAGGAAG	A	100	*	TYPE=DELETE
CM032587.1	1603158	.	A	C	100	*	TYPE=SUBSTITUTE
CM032587.1	1603264	.	A	T	100	*	TYPE=SUBSTITUTE
CM032587.1	1603312	.	T	C	100	*	TYPE=SUBSTITUTE
CM032587.1	1603334	.	A	C	100	*	TYPE=SUBSTITUTE
CM032587.1	1603455	.	AA	A	100	*	TYPE=DELETE
CM032587.1	1603565	.	G	a	100	*	TYPE=SUBSTITUTE
CM032587.1	1603630	.	C	T	100	*	TYPE=SUBSTITUTE
CM032587.1	1603717	.	G	A	100	*	TYPE=SUBSTITUTE
CM032587.1	1603718	.	T	G	100	*	TYPE=SUBSTITUTE
CM032587.1	1603719	.	C	G	100	*	TYPE=SUBSTITUTE
CM032587.1	1603914	.	A	G	100	*	TYPE=SUBSTITUTE

CM032587.1	1603982	.	G	A	100	*	TYPE=SUBSTITUTE
CM032587.1	1604030	.	G	A	100	*	TYPE=SUBSTITUTE
CM032587.1	1604045	.	TCCAGT	T	100	*	TYPE=DELETE
CM032587.1	1604062	.	A	AAGCA	100	*	TYPE=INSERT
CM032587.1	1604084	.	CAC	C	100	*	TYPE=DELETE
CM032587.1	1604128	.	A	G	100	*	TYPE=SUBSTITUTE
CM032587.1	1604156	.	A	G	100	*	TYPE=SUBSTITUTE
CM032587.1	1604213	.	A	C	100	*	TYPE=SUBSTITUTE
CM032587.1	1604220	.	G	A	100	*	TYPE=SUBSTITUTE
CM032587.1	1604227	.	C	A	100	*	TYPE=SUBSTITUTE
CM032587.1	1604231	.	C	T	100	*	TYPE=SUBSTITUTE
CM032587.1	1604232	.	T	G	100	*	TYPE=SUBSTITUTE
CM032587.1	1604236	.	C	T	100	*	TYPE=SUBSTITUTE
CM032587.1	1604251	.	T	A	100	*	TYPE=SUBSTITUTE
CM032587.1	1604260	.	A	T	100	*	TYPE=SUBSTITUTE
CM032587.1	1604264	.	TTG	T	100	*	TYPE=DELETE
CM032587.1	1604284	.	A	G	100	*	TYPE=SUBSTITUTE
CM032587.1	1604305	.	G	A	100	*	TYPE=SUBSTITUTE
CM032587.1	1604308	.	C	T	100	*	TYPE=SUBSTITUTE
CM032587.1	1604383	.	C	T	100	*	TYPE=SUBSTITUTE
CM032587.1	1604395	.	A	G	100	*	TYPE=SUBSTITUTE
CM032587.1	1604410	.	G	A	100	*	TYPE=SUBSTITUTE
CM032587.1	1604417	.	G	A	100	*	TYPE=SUBSTITUTE
CM032587.1	1604450	.	T	G	100	*	TYPE=SUBSTITUTE
CM032587.1	1604455	.	TGG	T	100	*	TYPE=DELETE
CM032587.1	1604655	.	A	G	100	*	TYPE=SUBSTITUTE
CM032587.1	1604739	.	G	A	100	*	TYPE=SUBSTITUTE
CM032587.1	1604817	.	T	C	100	*	TYPE=SUBSTITUTE
CM032587.1	1604898	.	T	C	100	*	TYPE=SUBSTITUTE
CM032587.1	1604923	.	T	G	100	*	TYPE=SUBSTITUTE
CM032587.1	1604979	.	C	T	100	*	TYPE=SUBSTITUTE
CM032587.1	1605084	.	A	G	100	*	TYPE=SUBSTITUTE
CM032587.1	1605087	.	T	C	100	*	TYPE=SUBSTITUTE
CM032587.1	1605098	.	A	G	100	*	TYPE=SUBSTITUTE
CM032587.1	1605111	.	G	C	100	*	TYPE=SUBSTITUTE
CM032587.1	1605159	.	A	G	100	*	TYPE=SUBSTITUTE
CM032587.1	1605165	.	A	G	100	*	TYPE=SUBSTITUTE
CM032587.1	1605177	.	C	T	100	*	TYPE=SUBSTITUTE
CM032587.1	1605180	.	G	A	100	*	TYPE=SUBSTITUTE
CM032587.1	1605183	.	T	C	100	*	TYPE=SUBSTITUTE
CM032587.1	1605189	.	T	C	100	*	TYPE=SUBSTITUTE
CM032587.1	1605210	.	G	A	100	*	TYPE=SUBSTITUTE
CM032587.1	1605213	.	C	T	100	*	TYPE=SUBSTITUTE
CM032587.1	1605214	.	T	C	100	*	TYPE=SUBSTITUTE
CM032587.1	1605217	.	G	A	100	*	TYPE=SUBSTITUTE
CM032587.1	1605310	.	C	T	100	*	TYPE=SUBSTITUTE
CM032587.1	1605319	.	G	C	100	*	TYPE=SUBSTITUTE
CM032587.1	1605333	.	T	C	100	*	TYPE=SUBSTITUTE
CM032587.1	1605340	.	G	T	100	*	TYPE=SUBSTITUTE
CM032587.1	1605353	.	T	G	100	*	TYPE=SUBSTITUTE
CM032587.1	1605406	.	T	C	100	*	TYPE=SUBSTITUTE
CM032587.1	1605537	.	C	T	100	*	TYPE=SUBSTITUTE
CM032587.1	1605594	.	T	G	100	*	TYPE=SUBSTITUTE
CM032587.1	1605764	.	T	A	100	*	TYPE=SUBSTITUTE
CM032587.1	1605797	.	T	G	100	*	TYPE=SUBSTITUTE
CM032587.1	1605836	.	C	T	100	*	TYPE=SUBSTITUTE
CM032587.1	1605872	.	G	T	100	*	TYPE=SUBSTITUTE
CM032587.1	1605905	.	A	G	100	*	TYPE=SUBSTITUTE
CM032587.1	1605936	.	G	A	100	*	TYPE=SUBSTITUTE
CM032587.1	1605988	.	G	T	100	*	TYPE=SUBSTITUTE
CM032587.1	1605992	.	C	CA	100	*	TYPE=INSERT
CM032587.1	1605995	.	T	A	100	*	TYPE=SUBSTITUTE
CM032587.1	1605996	.	T	TG	100	*	TYPE=INSERT
CM032587.1	1606108	.	G	A	100	*	TYPE=SUBSTITUTE

CM032587.1	1608417	.	G	T	100	*	TYPE=SUBSTITUTE
CM032587.1	1608715	.	C	A	100	*	TYPE=SUBSTITUTE
CM032587.1	1608909	.	TT	T	100	*	TYPE=DELETE
CM032587.1	1609163	.	C	T	100	*	TYPE=SUBSTITUTE
CM032587.1	1609212	.	A	G	100	*	TYPE=SUBSTITUTE
CM032587.1	1609213	.	G	A	100	*	TYPE=SUBSTITUTE
CM032587.1	1609223	.	A	G	100	*	TYPE=SUBSTITUTE
CM032587.1	1609761	.	C	T	100	*	TYPE=SUBSTITUTE
CM032587.1	1609934	.	G	A	100	*	TYPE=SUBSTITUTE
CM032587.1	1610179	.	G	A	100	*	TYPE=SUBSTITUTE
CM032587.1	1610542	.	T	C	100	*	TYPE=SUBSTITUTE
CM032587.1	1610721	.	C	G	100	*	TYPE=SUBSTITUTE
CM032587.1	1611083	.	G	T	100	*	TYPE=SUBSTITUTE
CM032587.1	1611295	.	T	C	100	*	TYPE=SUBSTITUTE
CM032587.1	1611296	.	G	T	100	*	TYPE=SUBSTITUTE
CM032587.1	1611299	.	C	T	100	*	TYPE=SUBSTITUTE
CM032587.1	1611303	.	G	T	100	*	TYPE=SUBSTITUTE
CM032587.1	1611307	.	GA	G	100	*	TYPE=DELETE
CM032587.1	1611311	.	C	T	100	*	TYPE=SUBSTITUTE
CM032587.1	1611366	.	G	A	100	*	TYPE=SUBSTITUTE
CM032587.1	1611533	.	TT	T	100	*	TYPE=DELETE
CM032587.1	1611577	.	G	T	100	*	TYPE=SUBSTITUTE
CM032587.1	1611578	.	T	G	100	*	TYPE=SUBSTITUTE
CM032587.1	1611582	.	A	AT	100	*	TYPE=INSERT
CM032587.1	1611590	.	C	T	100	*	TYPE=SUBSTITUTE
CM032587.1	1611600	.	A	G	100	*	TYPE=SUBSTITUTE
CM032587.1	1611639	.	A	G	100	*	TYPE=SUBSTITUTE
CM032587.1	1611652	.	C	T	100	*	TYPE=SUBSTITUTE
CM032587.1	1611658	.	AT	A	100	*	TYPE=DELETE
CM032587.1	1611662	.	A	ATA	100	*	TYPE=INSERT
CM032587.1	1611887	.	G	A	100	*	TYPE=SUBSTITUTE
CM032587.1	1611888	.	A	G	100	*	TYPE=SUBSTITUTE
CM032587.1	1611938	.	T	C	100	*	TYPE=SUBSTITUTE
CM032587.1	1611983	.	A	G	100	*	TYPE=SUBSTITUTE
CM032587.1	1611995	.	T	C	100	*	TYPE=SUBSTITUTE
CM032587.1	1612001	.	A	G	100	*	TYPE=SUBSTITUTE
CM032587.1	1612003	.	A	T	100	*	TYPE=SUBSTITUTE
CM032587.1	1612007	.	C	T	100	*	TYPE=SUBSTITUTE
CM032587.1	1612019	.	T	G	100	*	TYPE=SUBSTITUTE
CM032587.1	1612058	.	C	T	100	*	TYPE=SUBSTITUTE
CM032587.1	1612061	.	T	C	100	*	TYPE=SUBSTITUTE
CM032587.1	1612064	.	C	T	100	*	TYPE=SUBSTITUTE
CM032587.1	1612088	.	G	A	100	*	TYPE=SUBSTITUTE
CM032587.1	1612097	.	A	G	100	*	TYPE=SUBSTITUTE
CM032587.1	1612112	.	G	A	100	*	TYPE=SUBSTITUTE
CM032587.1	1612118	.	G	C	100	*	TYPE=SUBSTITUTE
CM032587.1	1612136	.	A	T	100	*	TYPE=SUBSTITUTE
CM032587.1	1612145	.	A	G	100	*	TYPE=SUBSTITUTE
CM032587.1	1612190	.	G	A	100	*	TYPE=SUBSTITUTE
CM032587.1	1612214	.	G	A	100	*	TYPE=SUBSTITUTE
CM032587.1	1612238	.	A	G	100	*	TYPE=SUBSTITUTE
CM032587.1	1612289	.	T	c	100	*	TYPE=SUBSTITUTE
CM032587.1	1612315	.	A	C	100	*	TYPE=SUBSTITUTE
CM032587.1	1612318	.	C	A	100	*	TYPE=SUBSTITUTE
CM032587.1	1612344	.	A	T	100	*	TYPE=SUBSTITUTE
CM032587.1	1612349	.	A	T	100	*	TYPE=SUBSTITUTE
CM032587.1	1612358	.	C	T	100	*	TYPE=SUBSTITUTE
CM032587.1	1612360	.	T	C	100	*	TYPE=SUBSTITUTE
CM032587.1	1612369	.	C	A	100	*	TYPE=SUBSTITUTE
CM032587.1	1612371	.	G	A	100	*	TYPE=SUBSTITUTE
CM032587.1	1612410	.	A	T	100	*	TYPE=SUBSTITUTE
CM032587.1	1612429	.	C	T	100	*	TYPE=SUBSTITUTE
CM032587.1	1612434	.	A	G	100	*	TYPE=SUBSTITUTE
CM032587.1	1612439	.	CA	C	100	*	TYPE=DELETE

CM032587.1	1612654	.	T	TT	100	*	TYPE=INSERT
CM032587.1	1612709	.	G	A	100	*	TYPE=SUBSTITUTE
CM032587.1	1612715	.	A	AAAAGCA	100	*	TYPE=INSERT
CM032587.1	1612862	.	TG	T	100	*	TYPE=DELETE
CM032587.1	1612865	.	G	A	100	*	TYPE=SUBSTITUTE
CM032587.1	1612876	.	G	A	100	*	TYPE=SUBSTITUTE
CM032587.1	1613027	.	G	T	100	*	TYPE=SUBSTITUTE
CM032587.1	1613335	.	G	gtcg	100	*	TYPE=INSERT
CM032587.1	1614006	.	A	G	100	*	TYPE=SUBSTITUTE
CM032587.1	1614166	.	T	C	100	*	TYPE=SUBSTITUTE
CM032587.1	1614185	.	G	C	100	*	TYPE=SUBSTITUTE
CM032587.1	1614216	.	A	AG	100	*	TYPE=INSERT
CM032587.1	1614218	.	G	A	100	*	TYPE=SUBSTITUTE
CM032587.1	1614256	.	T	C	100	*	TYPE=SUBSTITUTE
CM032587.1	1614257	.	G	GGT	100	*	TYPE=INSERT
CM032587.1	1614271	.	C	T	100	*	TYPE=SUBSTITUTE
CM032587.1	1614370	.	T	C	100	*	TYPE=SUBSTITUTE
CM032587.1	1614380	.	T	C	100	*	TYPE=SUBSTITUTE
CM032587.1	1614424	.	G	C	100	*	TYPE=SUBSTITUTE
CM032587.1	1614486	.	A	G	100	*	TYPE=SUBSTITUTE
CM032587.1	1614504	.	A	T	100	*	TYPE=SUBSTITUTE
CM032587.1	1614506	.	A	G	100	*	TYPE=SUBSTITUTE
CM032587.1	1614517	.	A	AC	100	*	TYPE=INSERT
CM032587.1	1614518	.	T	C	100	*	TYPE=SUBSTITUTE
CM032587.1	1614534	.	CAA	C	100	*	TYPE=DELETE
CM032587.1	1614553	.	T	C	100	*	TYPE=SUBSTITUTE
CM032587.1	1614578	.	G	A	100	*	TYPE=SUBSTITUTE
CM032587.1	1614579	.	T	C	100	*	TYPE=SUBSTITUTE
CM032587.1	1614583	.	A	G	100	*	TYPE=SUBSTITUTE
CM032587.1	1614585	.	T	c	100	*	TYPE=SUBSTITUTE
CM032587.1	1614593	.	G	a	100	*	TYPE=SUBSTITUTE
CM032587.1	1614603	.	C	a	100	*	TYPE=SUBSTITUTE
CM032587.1	1614616	.	T	C	100	*	TYPE=SUBSTITUTE
CM032587.1	1614618	.	C	T	100	*	TYPE=SUBSTITUTE
CM032587.1	1614621	.	C	T	100	*	TYPE=SUBSTITUTE
CM032587.1	1614625	.	T	C	100	*	TYPE=SUBSTITUTE
CM032587.1	1614630	.	ACCT	A	100	*	TYPE=DELETE
CM032587.1	1614640	.	T	C	100	*	TYPE=SUBSTITUTE
CM032587.1	1614654	.	T	C	100	*	TYPE=SUBSTITUTE
CM032587.1	1614655	.	G	A	100	*	TYPE=SUBSTITUTE
CM032587.1	1614666	.	C	A	100	*	TYPE=SUBSTITUTE
CM032587.1	1614672	.	A	G	100	*	TYPE=SUBSTITUTE
CM032587.1	1614675	.	G	A	100	*	TYPE=SUBSTITUTE
CM032587.1	1614692	.	GT	G	100	*	TYPE=DELETE
CM032587.1	1614700	.	G	GACTGGAGAA	100	*	TYPE=INSERT
CM032587.1	1614708	.	T	C	100	*	TYPE=SUBSTITUTE
CM032587.1	1614762	.	C	cc	100	*	TYPE=INSERT
CM032587.1	1614891	.	A	G	100	*	TYPE=SUBSTITUTE
CM032587.1	1614911	.	A	C	100	*	TYPE=SUBSTITUTE
CM032587.1	1614931	.	A	G	100	*	TYPE=SUBSTITUTE
CM032587.1	1614935	.	C	CA	100	*	TYPE=INSERT
CM032587.1	1614944	.	C	T	100	*	TYPE=SUBSTITUTE
CM032587.1	1614957	.	C	T	100	*	TYPE=SUBSTITUTE
CM032587.1	1614979	.	G	A	100	*	TYPE=SUBSTITUTE
CM032587.1	1614981	.	C	T	100	*	TYPE=SUBSTITUTE
CM032587.1	1614986	.	A	G	100	*	TYPE=SUBSTITUTE
CM032587.1	1614988	.	C	G	100	*	TYPE=SUBSTITUTE
CM032587.1	1614996	.	C	G	100	*	TYPE=SUBSTITUTE
CM032587.1	1615011	.	C	A	100	*	TYPE=SUBSTITUTE
CM032587.1	1615014	.	A	G	100	*	TYPE=SUBSTITUTE
CM032587.1	1615023	.	A	G	100	*	TYPE=SUBSTITUTE
CM032587.1	1615026	.	C	T	100	*	TYPE=SUBSTITUTE
CM032587.1	1615027	.	C	A	100	*	TYPE=SUBSTITUTE
CM032587.1	1615029	.	A	G	100	*	TYPE=SUBSTITUTE

CM032587.1	1615034	.	G	GT	100	*	TYPE=INSERT
CM032587.1	1615049	.	A	T	100	*	TYPE=SUBSTITUTE
CM032587.1	1615058	.	T	C	100	*	TYPE=SUBSTITUTE
CM032587.1	1615063	.	G	A	100	*	TYPE=SUBSTITUTE
CM032587.1	1615065	.	T	C	100	*	TYPE=SUBSTITUTE
CM032587.1	1615084	.	T	C	100	*	TYPE=SUBSTITUTE
CM032587.1	1615101	.	G	A	100	*	TYPE=SUBSTITUTE
CM032587.1	1615123	.	G	C	100	*	TYPE=SUBSTITUTE
CM032587.1	1615133	.	T	C	100	*	TYPE=SUBSTITUTE
CM032587.1	1615135	.	A	G	100	*	TYPE=SUBSTITUTE
CM032587.1	1615137	.	TA	T	100	*	TYPE=DELETE
CM032587.1	1615181	.	A	G	100	*	TYPE=SUBSTITUTE
CM032587.1	1615182	.	G	A	100	*	TYPE=SUBSTITUTE
CM032587.1	1615186	.	C	T	100	*	TYPE=SUBSTITUTE
CM032587.1	1615188	.	A	G	100	*	TYPE=SUBSTITUTE
CM032587.1	1615189	.	A	T	100	*	TYPE=SUBSTITUTE
CM032587.1	1615190	.	G	A	100	*	TYPE=SUBSTITUTE
CM032587.1	1615192	.	T	C	100	*	TYPE=SUBSTITUTE
CM032587.1	1615216	.	A	T	100	*	TYPE=SUBSTITUTE
CM032587.1	1615225	.	G	A	100	*	TYPE=SUBSTITUTE
CM032587.1	1615228	.	C	G	100	*	TYPE=SUBSTITUTE
CM032587.1	1615229	.	T	G	100	*	TYPE=SUBSTITUTE
CM032587.1	1615314	.	C	T	100	*	TYPE=SUBSTITUTE
CM032587.1	1615317	.	G	A	100	*	TYPE=SUBSTITUTE
CM032587.1	1615320	.	G	A	100	*	TYPE=SUBSTITUTE
CM032587.1	1615329	.	G	C	100	*	TYPE=SUBSTITUTE
CM032587.1	1615338	.	A	G	100	*	TYPE=SUBSTITUTE
CM032587.1	1615420	.	T	C	100	*	TYPE=SUBSTITUTE
CM032587.1	1615421	.	C	T	100	*	TYPE=SUBSTITUTE
CM032587.1	1615426	.	C	T	100	*	TYPE=SUBSTITUTE
CM032587.1	1615429	.	G	A	100	*	TYPE=SUBSTITUTE
CM032587.1	1615436	.	G	C	100	*	TYPE=SUBSTITUTE
CM032587.1	1615437	.	T	C	100	*	TYPE=SUBSTITUTE
CM032587.1	1615448	.	A	G	100	*	TYPE=SUBSTITUTE
CM032587.1	1615452	.	G	A	100	*	TYPE=SUBSTITUTE
CM032587.1	1615464	.	TA	T	100	*	TYPE=DELETE
CM032587.1	1615513	.	C	T	100	*	TYPE=SUBSTITUTE
CM032587.1	1615549	.	T	G	100	*	TYPE=SUBSTITUTE
CM032587.1	1615567	.	G	T	100	*	TYPE=SUBSTITUTE
CM032587.1	1615573	.	G	A	100	*	TYPE=SUBSTITUTE
CM032587.1	1615591	.	T	A	100	*	TYPE=SUBSTITUTE
CM032587.1	1615594	.	G	A	100	*	TYPE=SUBSTITUTE
CM032587.1	1615603	.	T	C	100	*	TYPE=SUBSTITUTE
CM032587.1	1615633	.	C	t	100	*	TYPE=SUBSTITUTE
CM032587.1	1615678	.	G	A	100	*	TYPE=SUBSTITUTE
CM032587.1	1615759	.	G	A	100	*	TYPE=SUBSTITUTE
CM032587.1	1615885	.	G	a	100	*	TYPE=SUBSTITUTE
CM032587.1	1615909	.	A	C	100	*	TYPE=SUBSTITUTE
CM032587.1	1615951	.	G	A	100	*	TYPE=SUBSTITUTE
CM032587.1	1615972	.	C	G	100	*	TYPE=SUBSTITUTE
CM032587.1	1616062	.	T	C	100	*	TYPE=SUBSTITUTE
CM032587.1	1616101	.	A	C	100	*	TYPE=SUBSTITUTE
CM032587.1	1616161	.	A	C	100	*	TYPE=SUBSTITUTE
CM032587.1	1616182	.	A	T	100	*	TYPE=SUBSTITUTE
CM032587.1	1616206	.	T	C	100	*	TYPE=SUBSTITUTE
CM032587.1	1616236	.	A	G	100	*	TYPE=SUBSTITUTE
CM032587.1	1616323	.	C	T	100	*	TYPE=SUBSTITUTE
CM032587.1	1616344	.	A	G	100	*	TYPE=SUBSTITUTE
CM032587.1	1616345	.	A	G	100	*	TYPE=SUBSTITUTE
CM032587.1	1616398	.	G	A	100	*	TYPE=SUBSTITUTE
CM032587.1	1616413	.	G	A	100	*	TYPE=SUBSTITUTE
CM032587.1	1616491	.	C	T	100	*	TYPE=SUBSTITUTE
CM032587.1	1616500	.	G	C	100	*	TYPE=SUBSTITUTE
CM032587.1	1616512	.	G	C	100	*	TYPE=SUBSTITUTE

CM032587.1	1616539	.	A	G	100	*	TYPE=SUBSTITUTE
CM032587.1	1616560	.	A	G	100	*	TYPE=SUBSTITUTE
CM032587.1	1616584	.	A	G	100	*	TYPE=SUBSTITUTE
CM032587.1	1616695	.	G	A	100	*	TYPE=SUBSTITUTE
CM032587.1	1616698	.	G	A	100	*	TYPE=SUBSTITUTE
CM032587.1	1616707	.	G	A	100	*	TYPE=SUBSTITUTE
CM032587.1	1616728	.	G	A	100	*	TYPE=SUBSTITUTE
CM032587.1	1616737	.	A	T	100	*	TYPE=SUBSTITUTE
CM032587.1	1616751	.	A	G	100	*	TYPE=SUBSTITUTE
CM032587.1	1616754	.	G	A	100	*	TYPE=SUBSTITUTE
CM032587.1	1616755	.	G	C	100	*	TYPE=SUBSTITUTE
CM032587.1	1616756	.	C	T	100	*	TYPE=SUBSTITUTE
CM032587.1	1616757	.	T	C	100	*	TYPE=SUBSTITUTE
CM032587.1	1616765	.	ACA	A	100	*	TYPE=DELETE
CM032587.1	1616794	.	C	G	100	*	TYPE=SUBSTITUTE
CM032587.1	1616799	.	T	C	100	*	TYPE=SUBSTITUTE
CM032587.1	1616815	.	G	A	100	*	TYPE=SUBSTITUTE
CM032587.1	1616817	.	C	T	100	*	TYPE=SUBSTITUTE
CM032587.1	1616833	.	A	T	100	*	TYPE=SUBSTITUTE
CM032587.1	1616858	.	C	A	100	*	TYPE=SUBSTITUTE
CM032587.1	1616881	.	CC	C	100	*	TYPE=DELETE
CM032587.1	1616907	.	AA	A	100	*	TYPE=DELETE
CM032587.1	1616993	.	C	A	100	*	TYPE=SUBSTITUTE
CM032587.1	1617032	.	G	A	100	*	TYPE=SUBSTITUTE
CM032587.1	1617040	.	A	G	100	*	TYPE=SUBSTITUTE
CM032587.1	1617041	.	G	A	100	*	TYPE=SUBSTITUTE
CM032587.1	1617043	.	C	T	100	*	TYPE=SUBSTITUTE
CM032587.1	1617049	.	A	T	100	*	TYPE=SUBSTITUTE
CM032587.1	1617061	.	C	T	100	*	TYPE=SUBSTITUTE
CM032587.1	1617074	.	T	TA	100	*	TYPE=INSERT
CM032587.1	1617087	.	G	A	100	*	TYPE=SUBSTITUTE
CM032587.1	1617100	.	TGTATCA	T	100	*	TYPE=DELETE
CM032587.1	1617101	.	A	T	100	*	TYPE=SUBSTITUTE
CM032587.1	1617122	.	C	T	100	*	TYPE=SUBSTITUTE
CM032587.1	1617205	.	G	A	100	*	TYPE=SUBSTITUTE
CM032587.1	1617256	.	C	T	100	*	TYPE=SUBSTITUTE
CM032587.1	1617270	.	A	C	100	*	TYPE=SUBSTITUTE
CM032587.1	1617279	.	C	T	100	*	TYPE=SUBSTITUTE
CM032587.1	1617280	.	T	A	100	*	TYPE=SUBSTITUTE
CM032587.1	1617286	.	T	C	100	*	TYPE=SUBSTITUTE
CM032587.1	1617301	.	G	a	100	*	TYPE=SUBSTITUTE
CM032587.1	1617337	.	C	T	100	*	TYPE=SUBSTITUTE
CM032587.1	1617340	.	T	C	100	*	TYPE=SUBSTITUTE
CM032587.1	1617346	.	C	T	100	*	TYPE=SUBSTITUTE
CM032587.1	1617364	.	T	C	100	*	TYPE=SUBSTITUTE
CM032587.1	1617367	.	A	T	100	*	TYPE=SUBSTITUTE
CM032587.1	1617370	.	C	T	100	*	TYPE=SUBSTITUTE
CM032587.1	1617379	.	T	G	100	*	TYPE=SUBSTITUTE
CM032587.1	1617430	.	T	c	100	*	TYPE=SUBSTITUTE
CM032587.1	1617448	.	C	G	100	*	TYPE=SUBSTITUTE
CM032587.1	1617460	.	T	C	100	*	TYPE=SUBSTITUTE
CM032587.1	1617478	.	C	A	100	*	TYPE=SUBSTITUTE
CM032587.1	1617490	.	T	C	100	*	TYPE=SUBSTITUTE
CM032587.1	1617496	.	G	T	100	*	TYPE=SUBSTITUTE
CM032587.1	1617517	.	A	G	100	*	TYPE=SUBSTITUTE
CM032587.1	1617523	.	G	C	100	*	TYPE=SUBSTITUTE
CM032587.1	1617532	.	T	c	100	*	TYPE=SUBSTITUTE
CM032587.1	1617562	.	T	C	100	*	TYPE=SUBSTITUTE
CM032587.1	1617568	.	C	T	100	*	TYPE=SUBSTITUTE
CM032587.1	1617832	.	A	C	100	*	TYPE=SUBSTITUTE
CM032587.1	1617840	.	A	G	100	*	TYPE=SUBSTITUTE
CM032587.1	1618099	.	G	A	100	*	TYPE=SUBSTITUTE
CM032587.1	1618138	.	G	A	100	*	TYPE=SUBSTITUTE
CM032587.1	1618183	.	G	T	100	*	TYPE=SUBSTITUTE

CM032587.1	1618218	.	T	C	100	*	TYPE=SUBSTITUTE
CM032587.1	1618228	.	A	G	100	*	TYPE=SUBSTITUTE
CM032587.1	1618278	.	A	G	100	*	TYPE=SUBSTITUTE
CM032587.1	1618345	.	G	A	100	*	TYPE=SUBSTITUTE
CM032587.1	1618365	.	A	c	100	*	TYPE=SUBSTITUTE
CM032587.1	1618399	.	T	C	100	*	TYPE=SUBSTITUTE
CM032587.1	1618427	.	A	G	100	*	TYPE=SUBSTITUTE
CM032587.1	1618516	.	T	C	100	*	TYPE=SUBSTITUTE
CM032587.1	1618588	.	T	C	100	*	TYPE=SUBSTITUTE
CM032587.1	1618590	.	C	T	100	*	TYPE=SUBSTITUTE
CM032587.1	1618591	.	T	C	100	*	TYPE=SUBSTITUTE
CM032587.1	1618606	.	A	C	100	*	TYPE=SUBSTITUTE
CM032587.1	1618689	.	G	C	100	*	TYPE=SUBSTITUTE
CM032587.1	1618700	.	CTCCCGCTGCT	C	100	*	TYPE=DELETE
CM032587.1	1618719	.	G	A	100	*	TYPE=SUBSTITUTE
CM032587.1	1618723	.	G	T	100	*	TYPE=SUBSTITUTE
CM032587.1	1618727	.	G	GGAAACCTA	100	*	TYPE=INSERT
CM032587.1	1618734	.	T	C	100	*	TYPE=SUBSTITUTE
CM032587.1	1618742	.	C	T	100	*	TYPE=SUBSTITUTE
CM032587.1	1618743	.	T	C	100	*	TYPE=SUBSTITUTE
CM032587.1	1618744	.	C	G	100	*	TYPE=SUBSTITUTE
CM032587.1	1618745	.	C	A	100	*	TYPE=SUBSTITUTE
CM032587.1	1618749	.	A	C	100	*	TYPE=SUBSTITUTE
CM032587.1	1618764	.	C	A	100	*	TYPE=SUBSTITUTE
CM032587.1	1618770	.	T	C	100	*	TYPE=SUBSTITUTE
CM032587.1	1618847	.	C	A	100	*	TYPE=SUBSTITUTE
CM032587.1	1618858	.	C	t	100	*	TYPE=SUBSTITUTE
CM032587.1	1618888	.	T	TGGTG	100	*	TYPE=INSERT
CM032587.1	1618894	.	C	CT	100	*	TYPE=INSERT
CM032587.1	1618967	.	C	T	100	*	TYPE=SUBSTITUTE
CM032587.1	1618999	.	G	A	100	*	TYPE=SUBSTITUTE
CM032587.1	1619015	.	A	C	100	*	TYPE=SUBSTITUTE
CM032587.1	1619028	.	C	T	100	*	TYPE=SUBSTITUTE
CM032587.1	1619030	.	C	A	100	*	TYPE=SUBSTITUTE
CM032587.1	1619041	.	T	C	100	*	TYPE=SUBSTITUTE
CM032587.1	1619050	.	A	G	100	*	TYPE=SUBSTITUTE
CM032587.1	1619056	.	GTACA	G	100	*	TYPE=DELETE
CM032587.1	1619059	.	A	C	100	*	TYPE=SUBSTITUTE
CM032587.1	1619062	.	T	C	100	*	TYPE=SUBSTITUTE
CM032587.1	1619065	.	A	G	100	*	TYPE=SUBSTITUTE
CM032587.1	1619120	.	A	G	100	*	TYPE=SUBSTITUTE
CM032587.1	1619129	.	T	C	100	*	TYPE=SUBSTITUTE
CM032587.1	1619131	.	G	A	100	*	TYPE=SUBSTITUTE
CM032587.1	1619133	.	A	ATAGC	100	*	TYPE=INSERT
CM032587.1	1619140	.	G	A	100	*	TYPE=SUBSTITUTE
CM032587.1	1619141	.	C	A	100	*	TYPE=SUBSTITUTE
CM032587.1	1619143	.	A	G	100	*	TYPE=SUBSTITUTE
CM032587.1	1619164	.	A	G	100	*	TYPE=SUBSTITUTE
CM032587.1	1619181	.	T	A	100	*	TYPE=SUBSTITUTE
CM032587.1	1619183	.	C	T	100	*	TYPE=SUBSTITUTE
CM032587.1	1619188	.	C	T	100	*	TYPE=SUBSTITUTE
CM032587.1	1619201	.	A	T	100	*	TYPE=SUBSTITUTE
CM032587.1	1619231	.	CAT	C	100	*	TYPE=DELETE
CM032587.1	1619236	.	A	G	100	*	TYPE=SUBSTITUTE
CM032587.1	1619269	.	G	A	100	*	TYPE=SUBSTITUTE
CM032587.1	1619341	.	A	T	100	*	TYPE=SUBSTITUTE
CM032587.1	1619364	.	A	G	100	*	TYPE=SUBSTITUTE
CM032587.1	1619370	.	G	T	100	*	TYPE=SUBSTITUTE
CM032587.1	1619409	.	C	A	100	*	TYPE=SUBSTITUTE
CM032587.1	1619410	.	A	C	100	*	TYPE=SUBSTITUTE
CM032587.1	1619418	.	GA	G	100	*	TYPE=DELETE
CM032587.1	1619466	.	C	T	100	*	TYPE=SUBSTITUTE
CM032587.1	1619478	.	A	G	100	*	TYPE=SUBSTITUTE
CM032587.1	1619513	.	C	A	100	*	TYPE=SUBSTITUTE

CM032587.1	1619525	.	T	C	100	*	TYPE=SUBSTITUTE
CM032587.1	1619526	.	G	A	100	*	TYPE=SUBSTITUTE
CM032587.1	1619583	.	T	C	100	*	TYPE=SUBSTITUTE
CM032587.1	1619592	.	C	G	100	*	TYPE=SUBSTITUTE
CM032587.1	1619601	.	C	T	100	*	TYPE=SUBSTITUTE
CM032587.1	1619612	.	A	T	100	*	TYPE=SUBSTITUTE
CM032587.1	1619615	.	C	T	100	*	TYPE=SUBSTITUTE
CM032587.1	1619630	.	T	C	100	*	TYPE=SUBSTITUTE
CM032587.1	1619637	.	C	T	100	*	TYPE=SUBSTITUTE
CM032587.1	1619711	.	T	C	100	*	TYPE=SUBSTITUTE
CM032587.1	1619735	.	T	C	100	*	TYPE=SUBSTITUTE
CM032587.1	1619768	.	A	G	100	*	TYPE=SUBSTITUTE
CM032587.1	1619786	.	A	G	100	*	TYPE=SUBSTITUTE
CM032587.1	1619816	.	G	T	100	*	TYPE=SUBSTITUTE
CM032587.1	1619822	.	G	A	100	*	TYPE=SUBSTITUTE
CM032587.1	1619825	.	T	C	100	*	TYPE=SUBSTITUTE
CM032587.1	1619831	.	A	G	100	*	TYPE=SUBSTITUTE
CM032587.1	1619846	.	A	G	100	*	TYPE=SUBSTITUTE
CM032587.1	1619849	.	T	C	100	*	TYPE=SUBSTITUTE
CM032587.1	1619867	.	T	A	100	*	TYPE=SUBSTITUTE
CM032587.1	1619870	.	C	T	100	*	TYPE=SUBSTITUTE
CM032587.1	1619884	.	A	C	100	*	TYPE=SUBSTITUTE
CM032587.1	1619918	.	T	C	100	*	TYPE=SUBSTITUTE
CM032587.1	1619930	.	G	A	100	*	TYPE=SUBSTITUTE
CM032587.1	1619954	.	T	C	100	*	TYPE=SUBSTITUTE
CM032587.1	1619963	.	G	A	100	*	TYPE=SUBSTITUTE
CM032587.1	1619981	.	T	C	100	*	TYPE=SUBSTITUTE
CM032587.1	1620011	.	G	A	100	*	TYPE=SUBSTITUTE
CM032587.1	1620038	.	T	C	100	*	TYPE=SUBSTITUTE
CM032587.1	1620050	.	G	A	100	*	TYPE=SUBSTITUTE
CM032587.1	1620053	.	A	C	100	*	TYPE=SUBSTITUTE
CM032587.1	1620071	.	A	G	100	*	TYPE=SUBSTITUTE
CM032587.1	1620089	.	C	T	100	*	TYPE=SUBSTITUTE
CM032587.1	1620093	.	T	A	100	*	TYPE=SUBSTITUTE
CM032587.1	1620123	.	A	T	100	*	TYPE=SUBSTITUTE
CM032587.1	1620127	.	C	T	100	*	TYPE=SUBSTITUTE
CM032587.1	1620128	.	T	C	100	*	TYPE=SUBSTITUTE
CM032587.1	1620133	.	A	T	100	*	TYPE=SUBSTITUTE
CM032587.1	1620138	.	A	G	100	*	TYPE=SUBSTITUTE
CM032587.1	1620149	.	T	C	100	*	TYPE=SUBSTITUTE
CM032587.1	1620150	.	A	C	100	*	TYPE=SUBSTITUTE
CM032587.1	1620164	.	G	A	100	*	TYPE=SUBSTITUTE
CM032587.1	1620179	.	C	T	100	*	TYPE=SUBSTITUTE
CM032587.1	1620194	.	C	T	100	*	TYPE=SUBSTITUTE
CM032587.1	1620200	.	T	G	100	*	TYPE=SUBSTITUTE
CM032587.1	1620203	.	A	T	100	*	TYPE=SUBSTITUTE
CM032587.1	1620215	.	G	A	100	*	TYPE=SUBSTITUTE
CM032587.1	1620241	.	T	A	100	*	TYPE=SUBSTITUTE
CM032587.1	1620243	.	A	G	100	*	TYPE=SUBSTITUTE
CM032587.1	1620251	.	T	A	100	*	TYPE=SUBSTITUTE
CM032587.1	1620274	.	A	G	100	*	TYPE=SUBSTITUTE
CM032587.1	1620287	.	C	T	100	*	TYPE=SUBSTITUTE
CM032587.1	1620291	.	C	T	100	*	TYPE=SUBSTITUTE
CM032587.1	1620320	.	A	T	100	*	TYPE=SUBSTITUTE
CM032587.1	1620345	.	TA	T	100	*	TYPE=DELETE
CM032587.1	1620349	.	A	G	100	*	TYPE=SUBSTITUTE
CM032587.1	1620381	.	C	T	100	*	TYPE=SUBSTITUTE
CM032587.1	1620406	.	C	T	100	*	TYPE=SUBSTITUTE
CM032587.1	1620422	.	A	AA	100	*	TYPE=INSERT
CM032587.1	1620474	.	A	G	100	*	TYPE=SUBSTITUTE
CM032587.1	1620502	.	T	C	100	*	TYPE=SUBSTITUTE
CM032587.1	1620514	.	C	T	100	*	TYPE=SUBSTITUTE
CM032587.1	1620515	.	G	A	100	*	TYPE=SUBSTITUTE
CM032587.1	1620536	.	T	C	100	*	TYPE=SUBSTITUTE

CM032587.1	1620589	.	A	G	100	*	TYPE=SUBSTITUTE
CM032587.1	1620613	.	C	T	100	*	TYPE=SUBSTITUTE
CM032587.1	1620662	.	C	A	100	*	TYPE=SUBSTITUTE
CM032587.1	1620670	.	T	A	100	*	TYPE=SUBSTITUTE
CM032587.1	1620673	.	T	C	100	*	TYPE=SUBSTITUTE
CM032587.1	1620675	.	G	A	100	*	TYPE=SUBSTITUTE
CM032587.1	1620677	.	C	T	100	*	TYPE=SUBSTITUTE
CM032587.1	1620681	.	A	G	100	*	TYPE=SUBSTITUTE
CM032587.1	1620682	.	C	T	100	*	TYPE=SUBSTITUTE
CM032587.1	1620683	.	A	G	100	*	TYPE=SUBSTITUTE
CM032587.1	1620684	.	G	A	100	*	TYPE=SUBSTITUTE
CM032587.1	1620685	.	T	C	100	*	TYPE=SUBSTITUTE
CM032587.1	1620691	.	T	C	100	*	TYPE=SUBSTITUTE
CM032587.1	1620700	.	A	T	100	*	TYPE=SUBSTITUTE
CM032587.1	1620710	.	TT	T	100	*	TYPE=DELETE
CM032587.1	1620758	.	G	C	100	*	TYPE=SUBSTITUTE
CM032587.1	1620791	.	T	C	100	*	TYPE=SUBSTITUTE
CM032587.1	1620796	.	A	T	100	*	TYPE=SUBSTITUTE
CM032587.1	1620797	.	A	G	100	*	TYPE=SUBSTITUTE
CM032587.1	1620798	.	T	G	100	*	TYPE=SUBSTITUTE
CM032587.1	1620905	.	T	C	100	*	TYPE=SUBSTITUTE
CM032587.1	1620910	.	G	A	100	*	TYPE=SUBSTITUTE
CM032587.1	1620915	.	T	C	100	*	TYPE=SUBSTITUTE
CM032587.1	1620916	.	A	G	100	*	TYPE=SUBSTITUTE
CM032587.1	1620926	.	G	A	100	*	TYPE=SUBSTITUTE
CM032587.1	1620929	.	T	C	100	*	TYPE=SUBSTITUTE
CM032587.1	1620959	.	C	T	100	*	TYPE=SUBSTITUTE
CM032587.1	1620980	.	G	A	100	*	TYPE=SUBSTITUTE
CM032587.1	1620984	.	T	C	100	*	TYPE=SUBSTITUTE
CM032587.1	1622087	.	GAT	G	100	*	TYPE=DELETE
CM032587.1	1622606	.	C	A	100	*	TYPE=SUBSTITUTE
CM032587.1	1622648	.	A	G	100	*	TYPE=SUBSTITUTE
CM032587.1	1623015	.	G	A	100	*	TYPE=SUBSTITUTE
CM032587.1	1623424	.	G	C	100	*	TYPE=SUBSTITUTE
CM032587.1	1623468	.	G	A	100	*	TYPE=SUBSTITUTE
CM032587.1	1623511	.	A	C	100	*	TYPE=SUBSTITUTE
CM032587.1	1623528	.	G	A	100	*	TYPE=SUBSTITUTE
CM032587.1	1623555	.	C	T	100	*	TYPE=SUBSTITUTE
CM032587.1	1623585	.	C	T	100	*	TYPE=SUBSTITUTE
CM032587.1	1623589	.	G	A	100	*	TYPE=SUBSTITUTE
CM032587.1	1623591	.	A	C	100	*	TYPE=SUBSTITUTE
CM032587.1	1623640	.	G	A	100	*	TYPE=SUBSTITUTE
CM032587.1	1623645	.	A	G	100	*	TYPE=SUBSTITUTE
CM032587.1	1623662	.	C	T	100	*	TYPE=SUBSTITUTE
CM032587.1	1623663	.	T	C	100	*	TYPE=SUBSTITUTE
CM032587.1	1623675	.	T	C	100	*	TYPE=SUBSTITUTE
CM032587.1	1623684	.	G	A	100	*	TYPE=SUBSTITUTE
CM032587.1	1623690	.	T	C	100	*	TYPE=SUBSTITUTE
CM032587.1	1623710	.	C	t	100	*	TYPE=SUBSTITUTE
CM032587.1	1623723	.	A	G	100	*	TYPE=SUBSTITUTE
CM032587.1	1623728	.	C	G	100	*	TYPE=SUBSTITUTE
CM032587.1	1623729	.	T	A	100	*	TYPE=SUBSTITUTE
CM032587.1	1623778	.	A	C	100	*	TYPE=SUBSTITUTE
CM032587.1	1623785	.	A	T	100	*	TYPE=SUBSTITUTE
CM032587.1	1623787	.	C	t	100	*	TYPE=SUBSTITUTE
CM032587.1	1623789	.	C	t	100	*	TYPE=SUBSTITUTE
CM032587.1	1623793	.	a	aaat	100	*	TYPE=INSERT
CM032587.1	1623798	.	C	t	100	*	TYPE=SUBSTITUTE
CM032587.1	1623799	.	G	a	100	*	TYPE=SUBSTITUTE
CM032587.1	1623806	.	A	G	100	*	TYPE=SUBSTITUTE
CM032587.1	1623811	.	C	T	100	*	TYPE=SUBSTITUTE
CM032587.1	1623814	.	A	g	100	*	TYPE=SUBSTITUTE
CM032587.1	1623842	.	TC	T	100	*	TYPE=DELETE
CM032587.1	1623848	.	TCTA	T	100	*	TYPE=DELETE

CM032587.1	1623853	.	C	A	100	*	TYPE=SUBSTITUTE
CM032587.1	1623854	.	A	C	100	*	TYPE=SUBSTITUTE
CM032587.1	1623862	.	G	A	100	*	TYPE=SUBSTITUTE
CM032587.1	1623867	.	GT	G	100	*	TYPE=DELETE
CM032587.1	1623869	.	G	T	100	*	TYPE=SUBSTITUTE
CM032587.1	1623870	.	A	T	100	*	TYPE=SUBSTITUTE
CM032587.1	1623873	.	G	A	100	*	TYPE=SUBSTITUTE
CM032587.1	1623877	.	C	t	100	*	TYPE=SUBSTITUTE
CM032587.1	1623885	.	A	g	100	*	TYPE=SUBSTITUTE
CM032587.1	1623894	.	T	g	100	*	TYPE=SUBSTITUTE
CM032587.1	1623895	.	T	a	100	*	TYPE=SUBSTITUTE
CM032587.1	1623905	.	A	T	100	*	TYPE=SUBSTITUTE
CM032587.1	1623913	.	C	T	100	*	TYPE=SUBSTITUTE
CM032587.1	1623914	.	T	C	100	*	TYPE=SUBSTITUTE
CM032587.1	1623917	.	T	C	100	*	TYPE=SUBSTITUTE
CM032587.1	1623918	.	A	G	100	*	TYPE=SUBSTITUTE
CM032587.1	1623922	.	T	C	100	*	TYPE=SUBSTITUTE
CM032587.1	1623925	.	A	C	100	*	TYPE=SUBSTITUTE
CM032587.1	1623927	.	T	C	100	*	TYPE=SUBSTITUTE
CM032587.1	1623929	.	G	A	100	*	TYPE=SUBSTITUTE
CM032587.1	1623933	.	A	C	100	*	TYPE=SUBSTITUTE
CM032587.1	1623935	.	A	G	100	*	TYPE=SUBSTITUTE
CM032587.1	1623938	.	A	G	100	*	TYPE=SUBSTITUTE
CM032587.1	1623939	.	G	A	100	*	TYPE=SUBSTITUTE
CM032587.1	1623940	.	G	T	100	*	TYPE=SUBSTITUTE
CM032587.1	1623949	.	A	G	100	*	TYPE=SUBSTITUTE
CM032587.1	1623950	.	T	C	100	*	TYPE=SUBSTITUTE
CM032587.1	1623951	.	C	T	100	*	TYPE=SUBSTITUTE
CM032587.1	1623960	.	G	GGTT	100	*	TYPE=INSERT
CM032587.1	1623973	.	C	T	100	*	TYPE=SUBSTITUTE
CM032587.1	1623975	.	T	C	100	*	TYPE=SUBSTITUTE
CM032587.1	1623979	.	G	A	100	*	TYPE=SUBSTITUTE
CM032587.1	1623983	.	T	a	100	*	TYPE=SUBSTITUTE
CM032587.1	1623987	.	G	a	100	*	TYPE=SUBSTITUTE
CM032587.1	1623988	.	A	g	100	*	TYPE=SUBSTITUTE
CM032587.1	1624014	.	A	G	100	*	TYPE=SUBSTITUTE
CM032587.1	1624043	.	C	G	100	*	TYPE=SUBSTITUTE
CM032587.1	1624045	.	A	G	100	*	TYPE=SUBSTITUTE
CM032587.1	1624049	.	T	C	100	*	TYPE=SUBSTITUTE
CM032587.1	1624050	.	G	T	100	*	TYPE=SUBSTITUTE
CM032587.1	1624089	.	A	AA	100	*	TYPE=INSERT
CM032587.1	1624115	.	G	A	100	*	TYPE=SUBSTITUTE
CM032587.1	1624120	.	C	T	100	*	TYPE=SUBSTITUTE
CM032587.1	1624131	.	A	T	100	*	TYPE=SUBSTITUTE
CM032587.1	1624137	.	G	A	100	*	TYPE=SUBSTITUTE
CM032587.1	1624173	.	G	A	100	*	TYPE=SUBSTITUTE
CM032587.1	1624176	.	C	G	100	*	TYPE=SUBSTITUTE
CM032587.1	1624177	.	C	A	100	*	TYPE=SUBSTITUTE
CM032587.1	1624178	.	A	T	100	*	TYPE=SUBSTITUTE
CM032587.1	1624185	.	A	G	100	*	TYPE=SUBSTITUTE
CM032587.1	1624219	.	G	A	100	*	TYPE=SUBSTITUTE
CM032587.1	1624270	.	G	GT	100	*	TYPE=INSERT
CM032587.1	1624273	.	T	C	100	*	TYPE=SUBSTITUTE
CM032587.1	1624281	.	T	G	100	*	TYPE=SUBSTITUTE
CM032587.1	1624284	.	TAG	T	100	*	TYPE=DELETE
CM032587.1	1624286	.	T	G	100	*	TYPE=SUBSTITUTE
CM032587.1	1624295	.	ATGCT	A	100	*	TYPE=DELETE
CM032587.1	1624310	.	A	AC	100	*	TYPE=INSERT
CM032587.1	1624311	.	G	A	100	*	TYPE=SUBSTITUTE
CM032587.1	1624322	.	c	ct	100	*	TYPE=INSERT
CM032587.1	1624328	.	AAA	A	100	*	TYPE=DELETE
CM032587.1	1624389	.	G	A	100	*	TYPE=SUBSTITUTE
CM032587.1	1624419	.	TGTGAGAGAGGGC	T	100	*	TYPE=DELETE
CM032587.1	1624435	.	t	ttgc	100	*	TYPE=INSERT

CM032587.1	1624441	.	G	C	100	*	TYPE=SUBSTITUTE
CM032587.1	1624454	.	T	G	100	*	TYPE=SUBSTITUTE
CM032587.1	1624455	.	A	T	100	*	TYPE=SUBSTITUTE
CM032587.1	1624457	.	A	AGGT	100	*	TYPE=INSERT
CM032587.1	1624458	.	A	G	100	*	TYPE=SUBSTITUTE
CM032587.1	1624462	.	A	G	100	*	TYPE=SUBSTITUTE
CM032587.1	1624464	.	A	G	100	*	TYPE=SUBSTITUTE
CM032587.1	1624476	.	C	G	100	*	TYPE=SUBSTITUTE
CM032587.1	1624497	.	C	T	100	*	TYPE=SUBSTITUTE
CM032587.1	1624576	.	G	a	100	*	TYPE=SUBSTITUTE
CM032587.1	1624605	.	C	T	100	*	TYPE=SUBSTITUTE
CM032587.1	1624657	.	A	C	100	*	TYPE=SUBSTITUTE
CM032587.1	1624658	.	A	G	100	*	TYPE=SUBSTITUTE
CM032587.1	1624660	.	T	C	100	*	TYPE=SUBSTITUTE
CM032587.1	1624663	.	C	T	100	*	TYPE=SUBSTITUTE
CM032587.1	1624666	.	T	C	100	*	TYPE=SUBSTITUTE
CM032587.1	1624670	.	A	C	100	*	TYPE=SUBSTITUTE
CM032587.1	1624674	.	TTG	T	100	*	TYPE=DELETE
CM032587.1	1624675	.	T	G	100	*	TYPE=SUBSTITUTE
CM032587.1	1624677	.	C	A	100	*	TYPE=SUBSTITUTE
CM032587.1	1624680	.	T	A	100	*	TYPE=SUBSTITUTE
CM032587.1	1624683	.	C	T	100	*	TYPE=SUBSTITUTE
CM032587.1	1624685	.	G	A	100	*	TYPE=SUBSTITUTE
CM032587.1	1624686	.	C	T	100	*	TYPE=SUBSTITUTE
CM032587.1	1624687	.	C	T	100	*	TYPE=SUBSTITUTE
CM032587.1	1624692	.	TT	T	100	*	TYPE=DELETE
CM032587.1	1624714	.	CAT	C	100	*	TYPE=DELETE
CM032587.1	1624715	.	G	T	100	*	TYPE=SUBSTITUTE
CM032587.1	1624726	.	A	G	100	*	TYPE=SUBSTITUTE
CM032587.1	1624728	.	G	T	100	*	TYPE=SUBSTITUTE
CM032587.1	1624746	.	C	T	100	*	TYPE=SUBSTITUTE
CM032587.1	1624803	.	A	G	100	*	TYPE=SUBSTITUTE
CM032587.1	1624815	.	T	C	100	*	TYPE=SUBSTITUTE
CM032587.1	1624834	.	G	c	100	*	TYPE=SUBSTITUTE
CM032587.1	1624843	.	G	A	100	*	TYPE=SUBSTITUTE
CM032587.1	1624863	.	G	GTCA	100	*	TYPE=INSERT
CM032587.1	1624875	.	C	T	100	*	TYPE=SUBSTITUTE
CM032587.1	1624887	.	G	A	100	*	TYPE=SUBSTITUTE
CM032587.1	1624891	.	T	C	100	*	TYPE=SUBSTITUTE
CM032587.1	1624907	.	C	t	100	*	TYPE=SUBSTITUTE
CM032587.1	1624927	.	T	C	100	*	TYPE=SUBSTITUTE
CM032587.1	1624963	.	T	C	100	*	TYPE=SUBSTITUTE
CM032587.1	1624978	.	G	C	100	*	TYPE=SUBSTITUTE
CM032587.1	1625023	.	A	C	100	*	TYPE=SUBSTITUTE
CM032587.1	1625047	.	A	G	100	*	TYPE=SUBSTITUTE
CM032587.1	1625050	.	A	G	100	*	TYPE=SUBSTITUTE
CM032587.1	1625062	.	T	C	100	*	TYPE=SUBSTITUTE
CM032587.1	1625065	.	C	T	100	*	TYPE=SUBSTITUTE
CM032587.1	1625077	.	G	A	100	*	TYPE=SUBSTITUTE
CM032587.1	1625083	.	A	G	100	*	TYPE=SUBSTITUTE
CM032587.1	1625110	.	C	G	100	*	TYPE=SUBSTITUTE
CM032587.1	1625113	.	A	G	100	*	TYPE=SUBSTITUTE
CM032587.1	1625140	.	T	C	100	*	TYPE=SUBSTITUTE
CM032587.1	1625158	.	G	A	100	*	TYPE=SUBSTITUTE
CM032587.1	1625188	.	A	G	100	*	TYPE=SUBSTITUTE
CM032587.1	1625200	.	G	A	100	*	TYPE=SUBSTITUTE
CM032587.1	1625206	.	T	C	100	*	TYPE=SUBSTITUTE
CM032587.1	1625215	.	C	A	100	*	TYPE=SUBSTITUTE
CM032587.1	1625224	.	C	T	100	*	TYPE=SUBSTITUTE
CM032587.1	1625239	.	G	A	100	*	TYPE=SUBSTITUTE
CM032587.1	1625242	.	A	G	100	*	TYPE=SUBSTITUTE
CM032587.1	1625248	.	G	T	100	*	TYPE=SUBSTITUTE
CM032587.1	1625251	.	C	T	100	*	TYPE=SUBSTITUTE
CM032587.1	1625272	.	C	G	100	*	TYPE=SUBSTITUTE

CM032587.1	1625276	.	C	T	100	*	TYPE=SUBSTITUTE
CM032587.1	1625284	.	C	t	100	*	TYPE=SUBSTITUTE
CM032587.1	1625289	.	GG	G	100	*	TYPE=DELETE
CM032587.1	1625325	.	G	A	100	*	TYPE=SUBSTITUTE
CM032587.1	1625327	.	C	G	100	*	TYPE=SUBSTITUTE
CM032587.1	1625335	.	C	T	100	*	TYPE=SUBSTITUTE
CM032587.1	1625338	.	C	T	100	*	TYPE=SUBSTITUTE
CM032587.1	1625341	.	C	T	100	*	TYPE=SUBSTITUTE
CM032587.1	1625401	.	A	T	100	*	TYPE=SUBSTITUTE
CM032587.1	1625422	.	C	T	100	*	TYPE=SUBSTITUTE
CM032587.1	1625463	.	C	G	100	*	TYPE=SUBSTITUTE
CM032587.1	1625491	.	C	T	100	*	TYPE=SUBSTITUTE
CM032587.1	1625506	.	T	C	100	*	TYPE=SUBSTITUTE
CM032587.1	1625519	.	CT	C	100	*	TYPE=DELETE
CM032587.1	1625521	.	C	T	100	*	TYPE=SUBSTITUTE
CM032587.1	1625530	.	G	A	100	*	TYPE=SUBSTITUTE
CM032587.1	1625533	.	G	A	100	*	TYPE=SUBSTITUTE
CM032587.1	1625551	.	G	A	100	*	TYPE=SUBSTITUTE
CM032587.1	1625557	.	G	A	100	*	TYPE=SUBSTITUTE
CM032587.1	1625584	.	C	G	100	*	TYPE=SUBSTITUTE
CM032587.1	1625716	.	C	T	100	*	TYPE=SUBSTITUTE
CM032587.1	1625719	.	C	A	100	*	TYPE=SUBSTITUTE
CM032587.1	1625734	.	A	G	100	*	TYPE=SUBSTITUTE
CM032587.1	1625764	.	T	C	100	*	TYPE=SUBSTITUTE
CM032587.1	1625782	.	C	T	100	*	TYPE=SUBSTITUTE
CM032587.1	1625809	.	A	T	100	*	TYPE=SUBSTITUTE
CM032587.1	1625818	.	T	C	100	*	TYPE=SUBSTITUTE
CM032587.1	1625826	.	C	T	100	*	TYPE=SUBSTITUTE
CM032587.1	1625851	.	T	C	100	*	TYPE=SUBSTITUTE
CM032587.1	1625953	.	C	T	100	*	TYPE=SUBSTITUTE
CM032587.1	1625957	.	G	A	100	*	TYPE=SUBSTITUTE
CM032587.1	1625958	.	G	A	100	*	TYPE=SUBSTITUTE
CM032587.1	1626004	.	A	G	100	*	TYPE=SUBSTITUTE
CM032587.1	1626016	.	C	T	100	*	TYPE=SUBSTITUTE
CM032587.1	1626025	.	C	T	100	*	TYPE=SUBSTITUTE
CM032587.1	1626069	.	T	G	100	*	TYPE=SUBSTITUTE
CM032587.1	1626079	.	A	C	100	*	TYPE=SUBSTITUTE
CM032587.1	1626124	.	C	A	100	*	TYPE=SUBSTITUTE
CM032587.1	1626166	.	T	C	100	*	TYPE=SUBSTITUTE
CM032587.1	1626172	.	T	C	100	*	TYPE=SUBSTITUTE
CM032587.1	1626175	.	C	A	100	*	TYPE=SUBSTITUTE
CM032587.1	1626181	.	T	C	100	*	TYPE=SUBSTITUTE
CM032587.1	1626199	.	C	G	100	*	TYPE=SUBSTITUTE
CM032587.1	1626244	.	G	A	100	*	TYPE=SUBSTITUTE
CM032587.1	1626262	.	C	T	100	*	TYPE=SUBSTITUTE
CM032587.1	1626274	.	A	G	100	*	TYPE=SUBSTITUTE
CM032587.1	1626313	.	C	T	100	*	TYPE=SUBSTITUTE
CM032587.1	1626327	.	A	T	100	*	TYPE=SUBSTITUTE
CM032587.1	1626331	.	C	T	100	*	TYPE=SUBSTITUTE
CM032587.1	1626360	.	G	A	100	*	TYPE=SUBSTITUTE
CM032587.1	1626364	.	T	C	100	*	TYPE=SUBSTITUTE
CM032587.1	1626366	.	G	A	100	*	TYPE=SUBSTITUTE
CM032587.1	1626379	.	C	T	100	*	TYPE=SUBSTITUTE
CM032587.1	1626406	.	A	G	100	*	TYPE=SUBSTITUTE
CM032587.1	1626412	.	A	G	100	*	TYPE=SUBSTITUTE
CM032587.1	1626418	.	G	A	100	*	TYPE=SUBSTITUTE
CM032587.1	1626427	.	C	T	100	*	TYPE=SUBSTITUTE
CM032587.1	1626430	.	C	T	100	*	TYPE=SUBSTITUTE
CM032587.1	1626438	.	C	T	100	*	TYPE=SUBSTITUTE
CM032587.1	1626448	.	A	G	100	*	TYPE=SUBSTITUTE
CM032587.1	1626481	.	A	G	100	*	TYPE=SUBSTITUTE
CM032587.1	1626514	.	G	A	100	*	TYPE=SUBSTITUTE
CM032587.1	1626523	.	T	C	100	*	TYPE=SUBSTITUTE
CM032587.1	1626568	.	T	C	100	*	TYPE=SUBSTITUTE

CM032587.1	1626577	.	G	C	100	*	TYPE=SUBSTITUTE
CM032587.1	1626654	.	C	t	100	*	TYPE=SUBSTITUTE
CM032587.1	1626655	.	G	A	100	*	TYPE=SUBSTITUTE
CM032587.1	1626676	.	A	T	100	*	TYPE=SUBSTITUTE
CM032587.1	1626733	.	A	G	100	*	TYPE=SUBSTITUTE
CM032587.1	1626742	.	G	C	100	*	TYPE=SUBSTITUTE
CM032587.1	1626769	.	C	T	100	*	TYPE=SUBSTITUTE
CM032587.1	1626772	.	G	A	100	*	TYPE=SUBSTITUTE
CM032587.1	1626789	.	T	C	100	*	TYPE=SUBSTITUTE
CM032587.1	1626790	.	G	A	100	*	TYPE=SUBSTITUTE
CM032587.1	1626792	.	A	C	100	*	TYPE=SUBSTITUTE
CM032587.1	1626796	.	T	G	100	*	TYPE=SUBSTITUTE
CM032587.1	1626800	.	C	T	100	*	TYPE=SUBSTITUTE
CM032587.1	1626812	.	CTAATGATT	C	100	*	TYPE=DELETE
CM032587.1	1626813	.	A	G	100	*	TYPE=SUBSTITUTE
CM032587.1	1626817	.	C	T	100	*	TYPE=SUBSTITUTE
CM032587.1	1626819	.	C	T	100	*	TYPE=SUBSTITUTE
CM032587.1	1626842	.	A	T	100	*	TYPE=SUBSTITUTE
CM032587.1	1626843	.	T	C	100	*	TYPE=SUBSTITUTE
CM032587.1	1626883	.	T	C	100	*	TYPE=SUBSTITUTE
CM032587.1	1626931	.	A	G	100	*	TYPE=SUBSTITUTE
CM032587.1	1626940	.	C	T	100	*	TYPE=SUBSTITUTE
CM032587.1	1626947	.	T	G	100	*	TYPE=SUBSTITUTE
CM032587.1	1626952	.	T	G	100	*	TYPE=SUBSTITUTE
CM032587.1	1626954	.	T	G	100	*	TYPE=SUBSTITUTE
CM032587.1	1626964	.	T	C	100	*	TYPE=SUBSTITUTE
CM032587.1	1626974	.	A	G	100	*	TYPE=SUBSTITUTE
CM032587.1	1626977	.	T	C	100	*	TYPE=SUBSTITUTE
CM032587.1	1626978	.	G	A	100	*	TYPE=SUBSTITUTE
CM032587.1	1626983	.	A	G	100	*	TYPE=SUBSTITUTE
CM032587.1	1626987	.	G	T	100	*	TYPE=SUBSTITUTE
CM032587.1	1626995	.	T	A	100	*	TYPE=SUBSTITUTE
CM032587.1	1626997	.	T	A	100	*	TYPE=SUBSTITUTE
CM032587.1	1626998	.	T	C	100	*	TYPE=SUBSTITUTE
CM032587.1	1626999	.	C	T	100	*	TYPE=SUBSTITUTE
CM032587.1	1627000	.	G	A	100	*	TYPE=SUBSTITUTE
CM032587.1	1627002	.	T	C	100	*	TYPE=SUBSTITUTE
CM032587.1	1627006	.	T	A	100	*	TYPE=SUBSTITUTE
CM032587.1	1627007	.	T	G	100	*	TYPE=SUBSTITUTE
CM032587.1	1627017	.	C	A	100	*	TYPE=SUBSTITUTE
CM032587.1	1627024	.	T	C	100	*	TYPE=SUBSTITUTE
CM032587.1	1627030	.	T	C	100	*	TYPE=SUBSTITUTE
CM032587.1	1627042	.	T	A	100	*	TYPE=SUBSTITUTE
CM032587.1	1627045	.	T	C	100	*	TYPE=SUBSTITUTE
CM032587.1	1627048	.	C	T	100	*	TYPE=SUBSTITUTE
CM032587.1	1627057	.	C	T	100	*	TYPE=SUBSTITUTE
CM032587.1	1627076	.	C	G	100	*	TYPE=SUBSTITUTE
CM032587.1	1627084	.	C	T	100	*	TYPE=SUBSTITUTE
CM032587.1	1627093	.	A	G	100	*	TYPE=SUBSTITUTE
CM032587.1	1627138	.	C	A	100	*	TYPE=SUBSTITUTE
CM032587.1	1627141	.	C	A	100	*	TYPE=SUBSTITUTE
CM032587.1	1627144	.	A	G	100	*	TYPE=SUBSTITUTE
CM032587.1	1627159	.	A	T	100	*	TYPE=SUBSTITUTE
CM032587.1	1627174	.	T	C	100	*	TYPE=SUBSTITUTE
CM032587.1	1627177	.	G	A	100	*	TYPE=SUBSTITUTE
CM032587.1	1627195	.	A	G	100	*	TYPE=SUBSTITUTE
CM032587.1	1627249	.	A	G	100	*	TYPE=SUBSTITUTE
CM032587.1	1627276	.	A	G	100	*	TYPE=SUBSTITUTE
CM032587.1	1627282	.	C	T	100	*	TYPE=SUBSTITUTE
CM032587.1	1627303	.	T	C	100	*	TYPE=SUBSTITUTE
CM032587.1	1627309	.	T	C	100	*	TYPE=SUBSTITUTE
CM032587.1	1627345	.	T	C	100	*	TYPE=SUBSTITUTE
CM032587.1	1627369	.	C	G	100	*	TYPE=SUBSTITUTE
CM032587.1	1627390	.	G	A	100	*	TYPE=SUBSTITUTE

CM032587.1	1627393	.	G	A	100	*	TYPE=SUBSTITUTE
CM032587.1	1627396	.	T	C	100	*	TYPE=SUBSTITUTE
CM032587.1	1627432	.	C	T	100	*	TYPE=SUBSTITUTE
CM032587.1	1627450	.	A	T	100	*	TYPE=SUBSTITUTE
CM032587.1	1627533	.	T	C	100	*	TYPE=SUBSTITUTE
CM032587.1	1627676	.	T	TT	100	*	TYPE=INSERT
CM032587.1	1628168	.	A	G	100	*	TYPE=SUBSTITUTE
CM032587.1	1628392	.	A	G	100	*	TYPE=SUBSTITUTE
CM032587.1	1628452	.	G	A	100	*	TYPE=SUBSTITUTE
CM032587.1	1628589	.	AA	A	100	*	TYPE=DELETE
CM032587.1	1628656	.	G	GTCT	100	*	TYPE=INSERT
CM032587.1	1628663	.	TG	T	100	*	TYPE=DELETE
CM032587.1	1628750	.	GG	G	100	*	TYPE=DELETE
CM032587.1	1628869	.	A	g	100	*	TYPE=SUBSTITUTE
CM032587.1	1628938	.	C	cc	100	*	TYPE=INSERT
CM032587.1	1628964	.	AAC	A	100	*	TYPE=DELETE
CM032587.1	1629018	.	CC	C	100	*	TYPE=DELETE
CM032587.1	1629061	.	CT	C	100	*	TYPE=DELETE
CM032587.1	1629062	.	C	T	100	*	TYPE=SUBSTITUTE
CM032587.1	1629098	.	G	GG	100	*	TYPE=INSERT
CM032587.1	1629140	.	T	A	100	*	TYPE=SUBSTITUTE
CM032587.1	1629141	.	A	G	100	*	TYPE=SUBSTITUTE
CM032587.1	1629201	.	G	A	100	*	TYPE=SUBSTITUTE
CM032587.1	1629224	.	A	C	100	*	TYPE=SUBSTITUTE
CM032587.1	1629226	.	C	T	100	*	TYPE=SUBSTITUTE
CM032587.1	1629227	.	G	A	100	*	TYPE=SUBSTITUTE
CM032587.1	1629279	.	T	C	100	*	TYPE=SUBSTITUTE
CM032587.1	1629280	.	C	T	100	*	TYPE=SUBSTITUTE
CM032587.1	1629281	.	T	C	100	*	TYPE=SUBSTITUTE
CM032587.1	1629345	.	T	C	100	*	TYPE=SUBSTITUTE
CM032587.1	1629388	.	C	T	100	*	TYPE=SUBSTITUTE
CM032587.1	1629389	.	G	GGAT	100	*	TYPE=INSERT
CM032587.1	1629400	.	T	C	100	*	TYPE=SUBSTITUTE
CM032587.1	1629407	.	ATAC	A	100	*	TYPE=DELETE
CM032587.1	1629412	.	T	C	100	*	TYPE=SUBSTITUTE
CM032587.1	1629413	.	A	G	100	*	TYPE=SUBSTITUTE
CM032587.1	1629432	.	A	C	100	*	TYPE=SUBSTITUTE
CM032587.1	1629438	.	C	T	100	*	TYPE=SUBSTITUTE
CM032587.1	1629491	.	G	A	100	*	TYPE=SUBSTITUTE
CM032587.1	1629493	.	T	C	100	*	TYPE=SUBSTITUTE
CM032587.1	1629495	.	T	C	100	*	TYPE=SUBSTITUTE
CM032587.1	1629500	.	C	T	100	*	TYPE=SUBSTITUTE
CM032587.1	1629506	.	C	A	100	*	TYPE=SUBSTITUTE
CM032587.1	1629521	.	G	A	100	*	TYPE=SUBSTITUTE
CM032587.1	1629522	.	C	G	100	*	TYPE=SUBSTITUTE
CM032587.1	1629524	.	C	T	100	*	TYPE=SUBSTITUTE
CM032587.1	1629586	.	AT	A	100	*	TYPE=DELETE
CM032587.1	1629589	.	T	C	100	*	TYPE=SUBSTITUTE
CM032587.1	1629592	.	T	C	100	*	TYPE=SUBSTITUTE
CM032587.1	1629603	.	C	A	100	*	TYPE=SUBSTITUTE
CM032587.1	1629605	.	A	G	100	*	TYPE=SUBSTITUTE
CM032587.1	1629613	.	G	A	100	*	TYPE=SUBSTITUTE
CM032587.1	1629616	.	T	C	100	*	TYPE=SUBSTITUTE
CM032587.1	1629645	.	G	A	100	*	TYPE=SUBSTITUTE
CM032587.1	1629669	.	GCTT	G	100	*	TYPE=DELETE
CM032587.1	1629683	.	G	C	100	*	TYPE=SUBSTITUTE
CM032587.1	1629684	.	G	T	100	*	TYPE=SUBSTITUTE
CM032587.1	1629687	.	C	T	100	*	TYPE=SUBSTITUTE
CM032587.1	1629770	.	A	G	100	*	TYPE=SUBSTITUTE
CM032587.1	1629775	.	T	C	100	*	TYPE=SUBSTITUTE
CM032587.1	1629782	.	G	C	100	*	TYPE=SUBSTITUTE
CM032587.1	1629783	.	A	G	100	*	TYPE=SUBSTITUTE
CM032587.1	1629789	.	T	C	100	*	TYPE=SUBSTITUTE
CM032587.1	1629832	.	C	a	100	*	TYPE=SUBSTITUTE

CM032587.1	1629872	.	G	T	100	*	TYPE=SUBSTITUTE
CM032587.1	1629883	.	A	G	100	*	TYPE=SUBSTITUTE
CM032587.1	1629888	.	G	T	100	*	TYPE=SUBSTITUTE
CM032587.1	1629930	.	G	A	100	*	TYPE=SUBSTITUTE
CM032587.1	1629937	.	G	t	100	*	TYPE=SUBSTITUTE
CM032587.1	1629942	.	A	g	100	*	TYPE=SUBSTITUTE
CM032587.1	1629953	.	C	T	100	*	TYPE=SUBSTITUTE
CM032587.1	1629995	.	TGA	T	100	*	TYPE=DELETE
CM032587.1	1630006	.	A	G	100	*	TYPE=SUBSTITUTE
CM032587.1	1630033	.	C	CC	100	*	TYPE=INSERT
CM032587.1	1630105	.	T	TC	100	*	TYPE=INSERT
CM032587.1	1630130	.	A	T	100	*	TYPE=SUBSTITUTE
CM032587.1	1630180	.	T	A	100	*	TYPE=SUBSTITUTE
CM032587.1	1630197	.	C	T	100	*	TYPE=SUBSTITUTE
CM032587.1	1630213	.	G	C	100	*	TYPE=SUBSTITUTE
CM032587.1	1630214	.	T	G	100	*	TYPE=SUBSTITUTE
CM032587.1	1630216	.	C	T	100	*	TYPE=SUBSTITUTE
CM032587.1	1630219	.	C	T	100	*	TYPE=SUBSTITUTE
CM032587.1	1630220	.	C	G	100	*	TYPE=SUBSTITUTE
CM032587.1	1630256	.	T	A	100	*	TYPE=SUBSTITUTE
CM032587.1	1630260	.	GGCC	G	100	*	TYPE=DELETE
CM032587.1	1630272	.	T	C	100	*	TYPE=SUBSTITUTE
CM032587.1	1630275	.	C	G	100	*	TYPE=SUBSTITUTE
CM032587.1	1630282	.	C	t	100	*	TYPE=SUBSTITUTE
CM032587.1	1630283	.	A	g	100	*	TYPE=SUBSTITUTE
CM032587.1	1630286	.	C	t	100	*	TYPE=SUBSTITUTE
CM032587.1	1630293	.	G	c	100	*	TYPE=SUBSTITUTE
CM032587.1	1630301	.	G	a	100	*	TYPE=SUBSTITUTE
CM032587.1	1630312	.	G	A	100	*	TYPE=SUBSTITUTE
CM032587.1	1630316	.	G	C	100	*	TYPE=SUBSTITUTE
CM032587.1	1630319	.	C	T	100	*	TYPE=SUBSTITUTE
CM032587.1	1630322	.	T	C	100	*	TYPE=SUBSTITUTE
CM032587.1	1630324	.	T	C	100	*	TYPE=SUBSTITUTE
CM032587.1	1630326	.	TA	T	100	*	TYPE=DELETE
CM032587.1	1630327	.	T	A	100	*	TYPE=SUBSTITUTE
CM032587.1	1630329	.	T	C	100	*	TYPE=SUBSTITUTE
CM032587.1	1630341	.	C	T	100	*	TYPE=SUBSTITUTE
CM032587.1	1630349	.	C	T	100	*	TYPE=SUBSTITUTE
CM032587.1	1630353	.	A	G	100	*	TYPE=SUBSTITUTE
CM032587.1	1630357	.	T	C	100	*	TYPE=SUBSTITUTE
CM032587.1	1630366	.	G	A	100	*	TYPE=SUBSTITUTE
CM032587.1	1630381	.	T	C	100	*	TYPE=SUBSTITUTE
CM032587.1	1630385	.	C	A	100	*	TYPE=SUBSTITUTE
CM032587.1	1630390	.	T	C	100	*	TYPE=SUBSTITUTE
CM032587.1	1630392	.	T	A	100	*	TYPE=SUBSTITUTE
CM032587.1	1630393	.	C	G	100	*	TYPE=SUBSTITUTE
CM032587.1	1630394	.	A	T	100	*	TYPE=SUBSTITUTE
CM032587.1	1630395	.	C	T	100	*	TYPE=SUBSTITUTE
CM032587.1	1630398	.	A	AT	100	*	TYPE=INSERT
CM032587.1	1630399	.	G	C	100	*	TYPE=SUBSTITUTE
CM032587.1	1630450	.	G	GTAGCA	100	*	TYPE=INSERT
CM032587.1	1630457	.	C	T	100	*	TYPE=SUBSTITUTE
CM032587.1	1630477	.	G	C	100	*	TYPE=SUBSTITUTE
CM032587.1	1630486	.	T	C	100	*	TYPE=SUBSTITUTE
CM032587.1	1630499	.	T	C	100	*	TYPE=SUBSTITUTE
CM032587.1	1630508	.	C	T	100	*	TYPE=SUBSTITUTE
CM032587.1	1630617	.	G	A	100	*	TYPE=SUBSTITUTE
CM032587.1	1630637	.	G	a	100	*	TYPE=SUBSTITUTE
CM032587.1	1630643	.	c	ctca	100	*	TYPE=INSERT
CM032587.1	1630649	.	C	A	100	*	TYPE=SUBSTITUTE
CM032587.1	1630651	.	T	C	100	*	TYPE=SUBSTITUTE
CM032587.1	1630671	.	T	C	100	*	TYPE=SUBSTITUTE
CM032587.1	1630692	.	T	C	100	*	TYPE=SUBSTITUTE
CM032587.1	1630714	.	C	T	100	*	TYPE=SUBSTITUTE

CM032587.1	1630716	.	T	C	100	*	TYPE=SUBSTITUTE
CM032587.1	1630717	.	C	T	100	*	TYPE=SUBSTITUTE
CM032587.1	1630724	.	A	G	100	*	TYPE=SUBSTITUTE
CM032587.1	1630727	.	TAC	T	100	*	TYPE=DELETE
CM032587.1	1630728	.	A	G	100	*	TYPE=SUBSTITUTE
CM032587.1	1630732	.	T	A	100	*	TYPE=SUBSTITUTE
CM032587.1	1630733	.	A	G	100	*	TYPE=SUBSTITUTE
CM032587.1	1630737	.	TA	T	100	*	TYPE=DELETE
CM032587.1	1630739	.	A	T	100	*	TYPE=SUBSTITUTE
CM032587.1	1630741	.	G	C	100	*	TYPE=SUBSTITUTE
CM032587.1	1630749	.	AG	A	100	*	TYPE=DELETE
CM032587.1	1630752	.	A	G	100	*	TYPE=SUBSTITUTE
CM032587.1	1630775	.	C	G	100	*	TYPE=SUBSTITUTE
CM032587.1	1630796	.	A	C	100	*	TYPE=SUBSTITUTE
CM032587.1	1630823	.	C	T	100	*	TYPE=SUBSTITUTE
CM032587.1	1630831	.	G	T	100	*	TYPE=SUBSTITUTE
CM032587.1	1630859	.	A	G	100	*	TYPE=SUBSTITUTE
CM032587.1	1630880	.	A	C	100	*	TYPE=SUBSTITUTE
CM032587.1	1630895	.	A	G	100	*	TYPE=SUBSTITUTE
CM032587.1	1630927	.	T	C	100	*	TYPE=SUBSTITUTE
CM032587.1	1630981	.	TAGA	T	100	*	TYPE=DELETE
CM032587.1	1631005	.	G	A	100	*	TYPE=SUBSTITUTE
CM032587.1	1631130	.	T	C	100	*	TYPE=SUBSTITUTE
CM032587.1	1631131	.	G	A	100	*	TYPE=SUBSTITUTE
CM032587.1	1631136	.	T	C	100	*	TYPE=SUBSTITUTE
CM032587.1	1631170	.	C	A	100	*	TYPE=SUBSTITUTE
CM032587.1	1631269	.	G	A	100	*	TYPE=SUBSTITUTE
CM032587.1	1631394	.	A	G	100	*	TYPE=SUBSTITUTE
CM032587.1	1631395	.	G	A	100	*	TYPE=SUBSTITUTE
CM032587.1	1631458	.	TT	T	100	*	TYPE=DELETE
CM032587.1	1631481	.	G	c	100	*	TYPE=SUBSTITUTE
CM032587.1	1631482	.	A	t	100	*	TYPE=SUBSTITUTE
CM032587.1	1631484	.	CA	C	100	*	TYPE=DELETE
CM032587.1	1631503	.	T	A	100	*	TYPE=SUBSTITUTE
CM032587.1	1631528	.	G	A	100	*	TYPE=SUBSTITUTE
CM032587.1	1631575	.	G	A	100	*	TYPE=SUBSTITUTE
CM032587.1	1631588	.	T	G	100	*	TYPE=SUBSTITUTE
CM032587.1	1631639	.	A	G	100	*	TYPE=SUBSTITUTE
CM032587.1	1631677	.	A	C	100	*	TYPE=SUBSTITUTE
CM032587.1	1631682	.	G	a	100	*	TYPE=SUBSTITUTE
CM032587.1	1631683	.	a	ac	100	*	TYPE=INSERT
CM032587.1	1631700	.	T	g	100	*	TYPE=SUBSTITUTE
CM032587.1	1631701	.	G	C	100	*	TYPE=SUBSTITUTE
CM032587.1	1631739	.	G	A	100	*	TYPE=SUBSTITUTE
CM032587.1	1631743	.	CGA	C	100	*	TYPE=DELETE
CM032587.1	1631750	.	C	A	100	*	TYPE=SUBSTITUTE
CM032587.1	1631751	.	C	A	100	*	TYPE=SUBSTITUTE
CM032587.1	1631752	.	C	CA	100	*	TYPE=INSERT
CM032587.1	1631758	.	T	C	100	*	TYPE=SUBSTITUTE
CM032587.1	1631783	.	T	C	100	*	TYPE=SUBSTITUTE
CM032587.1	1631787	.	A	G	100	*	TYPE=SUBSTITUTE
CM032587.1	1631799	.	A	G	100	*	TYPE=SUBSTITUTE
CM032587.1	1631811	.	T	C	100	*	TYPE=SUBSTITUTE
CM032587.1	1631814	.	G	A	100	*	TYPE=SUBSTITUTE
CM032587.1	1631844	.	A	G	100	*	TYPE=SUBSTITUTE
CM032587.1	1631901	.	C	G	100	*	TYPE=SUBSTITUTE
CM032587.1	1631914	.	T	G	100	*	TYPE=SUBSTITUTE
CM032587.1	1631928	.	C	G	100	*	TYPE=SUBSTITUTE
CM032587.1	1631934	.	G	A	100	*	TYPE=SUBSTITUTE
CM032587.1	1631991	.	A	g	100	*	TYPE=SUBSTITUTE
CM032587.1	1631992	.	C	t	100	*	TYPE=SUBSTITUTE
CM032587.1	1632021	.	A	T	100	*	TYPE=SUBSTITUTE
CM032587.1	1632054	.	G	T	100	*	TYPE=SUBSTITUTE
CM032587.1	1632063	.	G	A	100	*	TYPE=SUBSTITUTE

CM032587.1	1632064	.	C	A	100	*	TYPE=SUBSTITUTE
CM032587.1	1632084	.	A	G	100	*	TYPE=SUBSTITUTE
CM032587.1	1632099	.	G	A	100	*	TYPE=SUBSTITUTE
CM032587.1	1632125	.	G	A	100	*	TYPE=SUBSTITUTE
CM032587.1	1632129	.	A	T	100	*	TYPE=SUBSTITUTE
CM032587.1	1632133	.	GGTA	G	100	*	TYPE=DELETE
CM032587.1	1632135	.	T	A	100	*	TYPE=SUBSTITUTE
CM032587.1	1632137	.	G	C	100	*	TYPE=SUBSTITUTE
CM032587.1	1632139	.	TG	T	100	*	TYPE=DELETE
CM032587.1	1632149	.	T	C	100	*	TYPE=SUBSTITUTE
CM032587.1	1632154	.	T	A	100	*	TYPE=SUBSTITUTE
CM032587.1	1632155	.	A	G	100	*	TYPE=SUBSTITUTE
CM032587.1	1632164	.	T	C	100	*	TYPE=SUBSTITUTE
CM032587.1	1632166	.	G	A	100	*	TYPE=SUBSTITUTE
CM032587.1	1632191	.	G	A	100	*	TYPE=SUBSTITUTE
CM032587.1	1632209	.	A	T	100	*	TYPE=SUBSTITUTE
CM032587.1	1632216	.	C	T	100	*	TYPE=SUBSTITUTE
CM032587.1	1632224	.	C	T	100	*	TYPE=SUBSTITUTE
CM032587.1	1632239	.	T	G	100	*	TYPE=SUBSTITUTE
CM032587.1	1632281	.	G	A	100	*	TYPE=SUBSTITUTE
CM032587.1	1632380	.	G	A	100	*	TYPE=SUBSTITUTE
CM032587.1	1632404	.	G	A	100	*	TYPE=SUBSTITUTE
CM032587.1	1632443	.	A	G	100	*	TYPE=SUBSTITUTE
CM032587.1	1632470	.	T	A	100	*	TYPE=SUBSTITUTE
CM032587.1	1632485	.	C	T	100	*	TYPE=SUBSTITUTE
CM032587.1	1632515	.	T	C	100	*	TYPE=SUBSTITUTE
CM032587.1	1632569	.	G	A	100	*	TYPE=SUBSTITUTE
CM032587.1	1632572	.	A	G	100	*	TYPE=SUBSTITUTE
CM032587.1	1632590	.	G	A	100	*	TYPE=SUBSTITUTE
CM032587.1	1632623	.	A	G	100	*	TYPE=SUBSTITUTE
CM032587.1	1632671	.	G	t	100	*	TYPE=SUBSTITUTE
CM032587.1	1632683	.	A	G	100	*	TYPE=SUBSTITUTE
CM032587.1	1632698	.	G	C	100	*	TYPE=SUBSTITUTE
CM032587.1	1632704	.	C	T	100	*	TYPE=SUBSTITUTE
CM032587.1	1632713	.	G	A	100	*	TYPE=SUBSTITUTE
CM032587.1	1632722	.	G	A	100	*	TYPE=SUBSTITUTE
CM032587.1	1632764	.	G	A	100	*	TYPE=SUBSTITUTE
CM032587.1	1632770	.	A	G	100	*	TYPE=SUBSTITUTE
CM032587.1	1632791	.	G	A	100	*	TYPE=SUBSTITUTE
CM032587.1	1632839	.	A	G	100	*	TYPE=SUBSTITUTE
CM032587.1	1632845	.	G	A	100	*	TYPE=SUBSTITUTE
CM032587.1	1632851	.	G	A	100	*	TYPE=SUBSTITUTE
CM032587.1	1632860	.	G	A	100	*	TYPE=SUBSTITUTE
CM032587.1	1632902	.	G	A	100	*	TYPE=SUBSTITUTE
CM032587.1	1632915	.	C	T	100	*	TYPE=SUBSTITUTE
CM032587.1	1632946	.	C	T	100	*	TYPE=SUBSTITUTE
CM032587.1	1632947	.	G	A	100	*	TYPE=SUBSTITUTE
CM032587.1	1632959	.	A	T	100	*	TYPE=SUBSTITUTE
CM032587.1	1632976	.	A	C	100	*	TYPE=SUBSTITUTE
CM032587.1	1632985	.	C	T	100	*	TYPE=SUBSTITUTE
CM032587.1	1632998	.	C	t	100	*	TYPE=SUBSTITUTE
CM032587.1	1633005	.	A	t	100	*	TYPE=SUBSTITUTE
CM032587.1	1633009	.	C	t	100	*	TYPE=SUBSTITUTE
CM032587.1	1633035	.	G	T	100	*	TYPE=SUBSTITUTE
CM032587.1	1633051	.	T	G	100	*	TYPE=SUBSTITUTE
CM032587.1	1633069	.	G	A	100	*	TYPE=SUBSTITUTE
CM032587.1	1633075	.	T	C	100	*	TYPE=SUBSTITUTE
CM032587.1	1633076	.	A	G	100	*	TYPE=SUBSTITUTE
CM032587.1	1633077	.	A	C	100	*	TYPE=SUBSTITUTE
CM032587.1	1633113	.	G	T	100	*	TYPE=SUBSTITUTE
CM032587.1	1633118	.	C	T	100	*	TYPE=SUBSTITUTE
CM032587.1	1633120	.	G	A	100	*	TYPE=SUBSTITUTE
CM032587.1	1633125	.	T	C	100	*	TYPE=SUBSTITUTE
CM032587.1	1633161	.	C	T	100	*	TYPE=SUBSTITUTE

CM032587.1	1633183	.	G	T	100	*	TYPE=SUBSTITUTE
CM032587.1	1633184	.	C	G	100	*	TYPE=SUBSTITUTE
CM032587.1	1633191	.	C	T	100	*	TYPE=SUBSTITUTE
CM032587.1	1633233	.	T	C	100	*	TYPE=SUBSTITUTE
CM032587.1	1633263	.	CA	C	100	*	TYPE=DELETE
CM032587.1	1633285	.	T	C	100	*	TYPE=SUBSTITUTE
CM032587.1	1633330	.	A	C	100	*	TYPE=SUBSTITUTE
CM032587.1	1633346	.	C	T	100	*	TYPE=SUBSTITUTE
CM032587.1	1633349	.	G	A	100	*	TYPE=SUBSTITUTE
CM032587.1	1633352	.	C	T	100	*	TYPE=SUBSTITUTE
CM032587.1	1633366	.	C	t	100	*	TYPE=SUBSTITUTE
CM032587.1	1633368	.	c	ctt	100	*	TYPE=INSERT
CM032587.1	1633372	.	T	a	100	*	TYPE=SUBSTITUTE
CM032587.1	1633375	.	t	tg	100	*	TYPE=INSERT
CM032587.1	1633382	.	C	G	100	*	TYPE=SUBSTITUTE
CM032587.1	1633383	.	C	T	100	*	TYPE=SUBSTITUTE
CM032587.1	1633427	.	T	C	100	*	TYPE=SUBSTITUTE
CM032587.1	1633470	.	T	C	100	*	TYPE=SUBSTITUTE
CM032587.1	1633485	.	A	G	100	*	TYPE=SUBSTITUTE
CM032587.1	1633503	.	T	C	100	*	TYPE=SUBSTITUTE
CM032587.1	1633518	.	A	G	100	*	TYPE=SUBSTITUTE
CM032587.1	1633521	.	C	T	100	*	TYPE=SUBSTITUTE
CM032587.1	1633524	.	T	C	100	*	TYPE=SUBSTITUTE
CM032587.1	1633548	.	C	T	100	*	TYPE=SUBSTITUTE
CM032587.1	1633556	.	A	G	100	*	TYPE=SUBSTITUTE
CM032587.1	1633564	.	A	T	100	*	TYPE=SUBSTITUTE
CM032587.1	1633569	.	G	A	100	*	TYPE=SUBSTITUTE
CM032587.1	1633571	.	T	C	100	*	TYPE=SUBSTITUTE
CM032587.1	1633572	.	A	G	100	*	TYPE=SUBSTITUTE
CM032587.1	1633585	.	A	G	100	*	TYPE=SUBSTITUTE
CM032587.1	1633589	.	T	A	100	*	TYPE=SUBSTITUTE
CM032587.1	1633671	.	C	T	100	*	TYPE=SUBSTITUTE
CM032587.1	1633686	.	A	G	100	*	TYPE=SUBSTITUTE
CM032587.1	1633719	.	C	G	100	*	TYPE=SUBSTITUTE
CM032587.1	1633752	.	A	G	100	*	TYPE=SUBSTITUTE
CM032587.1	1633779	.	A	G	100	*	TYPE=SUBSTITUTE
CM032587.1	1633783	.	C	G	100	*	TYPE=SUBSTITUTE
CM032587.1	1633784	.	C	G	100	*	TYPE=SUBSTITUTE
CM032587.1	1633787	.	C	A	100	*	TYPE=SUBSTITUTE
CM032587.1	1633788	.	T	C	100	*	TYPE=SUBSTITUTE
CM032587.1	1633806	.	C	T	100	*	TYPE=SUBSTITUTE
CM032587.1	1633809	.	G	A	100	*	TYPE=SUBSTITUTE
CM032587.1	1633884	.	T	C	100	*	TYPE=SUBSTITUTE
CM032587.1	1633926	.	T	G	100	*	TYPE=SUBSTITUTE
CM032587.1	1633974	.	C	T	100	*	TYPE=SUBSTITUTE
CM032587.1	1633991	.	C	G	100	*	TYPE=SUBSTITUTE
CM032587.1	1634001	.	T	C	100	*	TYPE=SUBSTITUTE
CM032587.1	1634034	.	C	T	100	*	TYPE=SUBSTITUTE
CM032587.1	1634043	.	C	T	100	*	TYPE=SUBSTITUTE
CM032587.1	1634163	.	T	C	100	*	TYPE=SUBSTITUTE
CM032587.1	1634217	.	A	T	100	*	TYPE=SUBSTITUTE
CM032587.1	1634226	.	A	G	100	*	TYPE=SUBSTITUTE
CM032587.1	1634271	.	G	A	100	*	TYPE=SUBSTITUTE
CM032587.1	1634277	.	G	C	100	*	TYPE=SUBSTITUTE
CM032587.1	1634298	.	C	T	100	*	TYPE=SUBSTITUTE
CM032587.1	1634349	.	T	G	100	*	TYPE=SUBSTITUTE
CM032587.1	1634388	.	A	C	100	*	TYPE=SUBSTITUTE
CM032587.1	1634394	.	C	T	100	*	TYPE=SUBSTITUTE
CM032587.1	1634418	.	T	C	100	*	TYPE=SUBSTITUTE
CM032587.1	1634481	.	T	C	100	*	TYPE=SUBSTITUTE
CM032587.1	1634487	.	A	G	100	*	TYPE=SUBSTITUTE
CM032587.1	1634508	.	A	G	100	*	TYPE=SUBSTITUTE
CM032587.1	1634577	.	G	A	100	*	TYPE=SUBSTITUTE
CM032587.1	1634670	.	T	C	100	*	TYPE=SUBSTITUTE

CM032587.1	1634676	.	A	C	100	*	TYPE=SUBSTITUTE
CM032587.1	1634679	.	A	G	100	*	TYPE=SUBSTITUTE
CM032587.1	1634715	.	T	C	100	*	TYPE=SUBSTITUTE
CM032587.1	1634729	.	T	C	100	*	TYPE=SUBSTITUTE
CM032587.1	1634820	.	T	C	100	*	TYPE=SUBSTITUTE
CM032587.1	1634850	.	C	t	100	*	TYPE=SUBSTITUTE
CM032587.1	1634853	.	C	t	100	*	TYPE=SUBSTITUTE
CM032587.1	1634856	.	T	c	100	*	TYPE=SUBSTITUTE
CM032587.1	1634881	.	A	G	100	*	TYPE=SUBSTITUTE
CM032587.1	1634910	.	A	C	100	*	TYPE=SUBSTITUTE
CM032587.1	1635006	.	T	C	100	*	TYPE=SUBSTITUTE
CM032587.1	1635024	.	G	A	100	*	TYPE=SUBSTITUTE
CM032587.1	1635032	.	A	G	100	*	TYPE=SUBSTITUTE
CM032587.1	1635038	.	G	C	100	*	TYPE=SUBSTITUTE
CM032587.1	1635049	.	G	A	100	*	TYPE=SUBSTITUTE
CM032587.1	1635084	.	C	T	100	*	TYPE=SUBSTITUTE
CM032587.1	1635114	.	A	G	100	*	TYPE=SUBSTITUTE
CM032587.1	1635117	.	G	T	100	*	TYPE=SUBSTITUTE
CM032587.1	1635120	.	TCA	T	100	*	TYPE=DELETE
CM032587.1	1635136	.	T	A	100	*	TYPE=SUBSTITUTE
CM032587.1	1635142	.	T	G	100	*	TYPE=SUBSTITUTE
CM032587.1	1635213	.	G	A	100	*	TYPE=SUBSTITUTE
CM032587.1	1635224	.	A	T	100	*	TYPE=SUBSTITUTE
CM032587.1	1635226	.	C	G	100	*	TYPE=SUBSTITUTE
CM032587.1	1635228	.	G	GT	100	*	TYPE=INSERT
CM032587.1	1635231	.	T	C	100	*	TYPE=SUBSTITUTE
CM032587.1	1635330	.	C	T	100	*	TYPE=SUBSTITUTE
CM032587.1	1635332	.	C	T	100	*	TYPE=SUBSTITUTE
CM032587.1	1635345	.	T	G	100	*	TYPE=SUBSTITUTE
CM032587.1	1635359	.	A	T	100	*	TYPE=SUBSTITUTE
CM032587.1	1635360	.	A	C	100	*	TYPE=SUBSTITUTE
CM032587.1	1635375	.	C	CC	100	*	TYPE=INSERT
CM032587.1	1635415	.	G	A	100	*	TYPE=SUBSTITUTE
CM032587.1	1635429	.	A	G	100	*	TYPE=SUBSTITUTE
CM032587.1	1635434	.	A	C	100	*	TYPE=SUBSTITUTE
CM032587.1	1635438	.	C	A	100	*	TYPE=SUBSTITUTE
CM032587.1	1635439	.	G	A	100	*	TYPE=SUBSTITUTE
CM032587.1	1635445	.	A	C	100	*	TYPE=SUBSTITUTE
CM032587.1	1635452	.	G	T	100	*	TYPE=SUBSTITUTE
CM032587.1	1635459	.	T	G	100	*	TYPE=SUBSTITUTE
CM032587.1	1635477	.	C	T	100	*	TYPE=SUBSTITUTE
CM032587.1	1635536	.	T	A	100	*	TYPE=SUBSTITUTE
CM032587.1	1635563	.	A	G	100	*	TYPE=SUBSTITUTE
CM032587.1	1635565	.	C	T	100	*	TYPE=SUBSTITUTE
CM032587.1	1635639	.	G	C	100	*	TYPE=SUBSTITUTE
CM032587.1	1635653	.	T	A	100	*	TYPE=SUBSTITUTE
CM032587.1	1635663	.	C	T	100	*	TYPE=SUBSTITUTE
CM032587.1	1635664	.	T	G	100	*	TYPE=SUBSTITUTE
CM032587.1	1635669	.	A	C	100	*	TYPE=SUBSTITUTE
CM032587.1	1635670	.	T	G	100	*	TYPE=SUBSTITUTE
CM032587.1	1635673	.	C	G	100	*	TYPE=SUBSTITUTE
CM032587.1	1635690	.	G	A	100	*	TYPE=SUBSTITUTE
CM032587.1	1635691	.	C	G	100	*	TYPE=SUBSTITUTE
CM032587.1	1635730	.	T	A	100	*	TYPE=SUBSTITUTE
CM032587.1	1635738	.	G	A	100	*	TYPE=SUBSTITUTE
CM032587.1	1635828	.	C	T	100	*	TYPE=SUBSTITUTE
CM032587.1	1635873	.	G	A	100	*	TYPE=SUBSTITUTE
CM032587.1	1635907	.	AAGAAGAAGAAG	A	100	*	TYPE=DELETE
CM032587.1	1635909	.	A	g	100	*	TYPE=SUBSTITUTE
CM032587.1	1635910	.	A	g	100	*	TYPE=SUBSTITUTE
CM032587.1	1635918	.	G	a	100	*	TYPE=SUBSTITUTE
CM032587.1	1635968	.	A	G	100	*	TYPE=SUBSTITUTE
CM032587.1	1635992	.	T	C	100	*	TYPE=SUBSTITUTE
CM032587.1	1636014	.	A	AA	100	*	TYPE=INSERT

CM032587.1	1636048	.	A	AA	100	*	TYPE=INSERT
CM032587.1	1636093	.	C	G	100	*	TYPE=SUBSTITUTE
CM032587.1	1636094	.	G	GTTG	100	*	TYPE=INSERT
CM032587.1	1636112	.	G	A	100	*	TYPE=SUBSTITUTE
CM032587.1	1636117	.	G	A	100	*	TYPE=SUBSTITUTE
CM032587.1	1636144	.	C	t	100	*	TYPE=SUBSTITUTE
CM032587.1	1636147	.	G	t	100	*	TYPE=SUBSTITUTE
CM032587.1	1636152	.	A	g	100	*	TYPE=SUBSTITUTE
CM032587.1	1636160	.	CAA	C	100	*	TYPE=DELETE
CM032587.1	1636164	.	G	A	100	*	TYPE=SUBSTITUTE
CM032587.1	1636167	.	A	G	100	*	TYPE=SUBSTITUTE
CM032587.1	1636174	.	T	C	100	*	TYPE=SUBSTITUTE
CM032587.1	1636178	.	A	T	100	*	TYPE=SUBSTITUTE
CM032587.1	1636181	.	G	T	100	*	TYPE=SUBSTITUTE
CM032587.1	1636189	.	G	A	100	*	TYPE=SUBSTITUTE
CM032587.1	1636190	.	T	G	100	*	TYPE=SUBSTITUTE
CM032587.1	1636289	.	A	ATGGATG	100	*	TYPE=INSERT
CM032587.1	1636374	.	A	G	100	*	TYPE=SUBSTITUTE
CM032587.1	1636386	.	C	T	100	*	TYPE=SUBSTITUTE
CM032587.1	1636395	.	A	G	100	*	TYPE=SUBSTITUTE
CM032587.1	1636401	.	C	G	100	*	TYPE=SUBSTITUTE
CM032587.1	1636426	.	C	A	100	*	TYPE=SUBSTITUTE
CM032587.1	1636431	.	T	C	100	*	TYPE=SUBSTITUTE
CM032587.1	1636461	.	G	A	100	*	TYPE=SUBSTITUTE
CM032587.1	1636475	.	C	A	100	*	TYPE=SUBSTITUTE
CM032587.1	1636480	.	T	C	100	*	TYPE=SUBSTITUTE
CM032587.1	1636549	.	T	A	100	*	TYPE=SUBSTITUTE
CM032587.1	1636550	.	G	A	100	*	TYPE=SUBSTITUTE
CM032587.1	1636572	.	T	C	100	*	TYPE=SUBSTITUTE
CM032587.1	1636707	.	T	C	100	*	TYPE=SUBSTITUTE
CM032587.1	1636715	.	A	T	100	*	TYPE=SUBSTITUTE
CM032587.1	1636731	.	G	GGCGGTT	100	*	TYPE=INSERT
CM032587.1	1636734	.	C	T	100	*	TYPE=SUBSTITUTE
CM032587.1	1636742	.	C	T	100	*	TYPE=SUBSTITUTE
CM032587.1	1636752	.	T	C	100	*	TYPE=SUBSTITUTE
CM032587.1	1636787	.	G	C	100	*	TYPE=SUBSTITUTE
CM032587.1	1636792	.	C	A	100	*	TYPE=SUBSTITUTE
CM032587.1	1636794	.	G	A	100	*	TYPE=SUBSTITUTE
CM032587.1	1636827	.	C	T	100	*	TYPE=SUBSTITUTE
CM032587.1	1636837	.	T	C	100	*	TYPE=SUBSTITUTE
CM032587.1	1636896	.	C	T	100	*	TYPE=SUBSTITUTE
CM032587.1	1636918	.	A	G	100	*	TYPE=SUBSTITUTE
CM032587.1	1636942	.	C	G	100	*	TYPE=SUBSTITUTE
CM032587.1	1636947	.	T	A	100	*	TYPE=SUBSTITUTE
CM032587.1	1636976	.	G	a	100	*	TYPE=SUBSTITUTE
CM032587.1	1636977	.	A	g	100	*	TYPE=SUBSTITUTE
CM032587.1	1636997	.	G	A	100	*	TYPE=SUBSTITUTE
CM032587.1	1637013	.	C	T	100	*	TYPE=SUBSTITUTE
CM032587.1	1637022	.	A	G	100	*	TYPE=SUBSTITUTE
CM032587.1	1637025	.	T	A	100	*	TYPE=SUBSTITUTE
CM032587.1	1637049	.	T	C	100	*	TYPE=SUBSTITUTE
CM032587.1	1637064	.	T	C	100	*	TYPE=SUBSTITUTE
CM032587.1	1637067	.	G	A	100	*	TYPE=SUBSTITUTE
CM032587.1	1637073	.	T	C	100	*	TYPE=SUBSTITUTE
CM032587.1	1637088	.	A	G	100	*	TYPE=SUBSTITUTE
CM032587.1	1637136	.	T	c	100	*	TYPE=SUBSTITUTE
CM032587.1	1637148	.	T	a	100	*	TYPE=SUBSTITUTE
CM032587.1	1637151	.	A	c	100	*	TYPE=SUBSTITUTE
CM032587.1	1637160	.	T	a	100	*	TYPE=SUBSTITUTE
CM032587.1	1637178	.	GTCGTCGTCAACA	G	100	*	TYPE=DELETE
CM032587.1	1637181	.	G	A	100	*	TYPE=SUBSTITUTE
CM032587.1	1637190	.	G	A	100	*	TYPE=SUBSTITUTE
CM032587.1	1637191	.	C	T	100	*	TYPE=SUBSTITUTE
CM032587.1	1637205	.	T	G	100	*	TYPE=SUBSTITUTE

CM032587.1	1637235	.	C	T	100	*	TYPE=SUBSTITUTE
CM032587.1	1637237	.	G	a	100	*	TYPE=SUBSTITUTE
CM032587.1	1637241	.	T	c	100	*	TYPE=SUBSTITUTE
CM032587.1	1637247	.	CTTC	C	100	*	TYPE=DELETE
CM032587.1	1637269	.	A	G	100	*	TYPE=SUBSTITUTE
CM032587.1	1637271	.	A	T	100	*	TYPE=SUBSTITUTE
CM032587.1	1637272	.	C	T	100	*	TYPE=SUBSTITUTE
CM032587.1	1637274	.	A	G	100	*	TYPE=SUBSTITUTE
CM032587.1	1637280	.	G	C	100	*	TYPE=SUBSTITUTE
CM032587.1	1637285	.	A	G	100	*	TYPE=SUBSTITUTE
CM032587.1	1637307	.	G	A	100	*	TYPE=SUBSTITUTE
CM032587.1	1637328	.	G	A	100	*	TYPE=SUBSTITUTE
CM032587.1	1637526	.	C	T	100	*	TYPE=SUBSTITUTE
CM032587.1	1637541	.	A	G	100	*	TYPE=SUBSTITUTE
CM032587.1	1637562	.	C	T	100	*	TYPE=SUBSTITUTE
CM032587.1	1637685	.	A	G	100	*	TYPE=SUBSTITUTE
CM032587.1	1637694	.	A	G	100	*	TYPE=SUBSTITUTE
CM032587.1	1637715	.	A	T	100	*	TYPE=SUBSTITUTE
CM032587.1	1637802	.	A	G	100	*	TYPE=SUBSTITUTE
CM032587.1	1637886	.	A	G	100	*	TYPE=SUBSTITUTE
CM032587.1	1637937	.	A	G	100	*	TYPE=SUBSTITUTE
CM032587.1	1638204	.	C	T	100	*	TYPE=SUBSTITUTE
CM032587.1	1638306	.	C	T	100	*	TYPE=SUBSTITUTE
CM032587.1	1638412	.	G	A	100	*	TYPE=SUBSTITUTE
CM032587.1	1638501	.	A	G	100	*	TYPE=SUBSTITUTE
CM032587.1	1638519	.	C	t	100	*	TYPE=SUBSTITUTE
CM032587.1	1638681	.	C	t	100	*	TYPE=SUBSTITUTE
CM032587.1	1638735	.	G	A	100	*	TYPE=SUBSTITUTE
CM032587.1	1638774	.	C	T	100	*	TYPE=SUBSTITUTE
CM032587.1	1638795	.	T	C	100	*	TYPE=SUBSTITUTE
CM032587.1	1638810	.	C	T	100	*	TYPE=SUBSTITUTE
CM032587.1	1638813	.	A	G	100	*	TYPE=SUBSTITUTE
CM032587.1	1638819	.	T	C	100	*	TYPE=SUBSTITUTE
CM032587.1	1638820	.	T	G	100	*	TYPE=SUBSTITUTE
CM032587.1	1638828	.	G	A	100	*	TYPE=SUBSTITUTE
CM032587.1	1638837	.	C	T	100	*	TYPE=SUBSTITUTE
CM032587.1	1638840	.	A	G	100	*	TYPE=SUBSTITUTE
CM032587.1	1638846	.	G	A	100	*	TYPE=SUBSTITUTE
CM032587.1	1638879	.	T	C	100	*	TYPE=SUBSTITUTE
CM032587.1	1638894	.	A	G	100	*	TYPE=SUBSTITUTE
CM032587.1	1638900	.	G	A	100	*	TYPE=SUBSTITUTE
CM032587.1	1638906	.	G	A	100	*	TYPE=SUBSTITUTE
CM032587.1	1638909	.	A	T	100	*	TYPE=SUBSTITUTE
CM032587.1	1638930	.	C	T	100	*	TYPE=SUBSTITUTE
CM032587.1	1638951	.	A	C	100	*	TYPE=SUBSTITUTE
CM032587.1	1639011	.	A	G	100	*	TYPE=SUBSTITUTE
CM032587.1	1639041	.	A	G	100	*	TYPE=SUBSTITUTE
CM032587.1	1639086	.	G	A	100	*	TYPE=SUBSTITUTE
CM032587.1	1639194	.	G	A	100	*	TYPE=SUBSTITUTE
CM032587.1	1639206	.	T	C	100	*	TYPE=SUBSTITUTE
CM032587.1	1639235	.	A	G	100	*	TYPE=SUBSTITUTE
CM032587.1	1639237	.	C	A	100	*	TYPE=SUBSTITUTE
CM032587.1	1639258	.	A	T	100	*	TYPE=SUBSTITUTE
CM032587.1	1639269	.	A	G	100	*	TYPE=SUBSTITUTE
CM032587.1	1639282	.	A	G	100	*	TYPE=SUBSTITUTE
CM032587.1	1639288	.	A	G	100	*	TYPE=SUBSTITUTE
CM032587.1	1639354	.	C	T	100	*	TYPE=SUBSTITUTE
CM032587.1	1639363	.	A	G	100	*	TYPE=SUBSTITUTE
CM032587.1	1639369	.	T	C	100	*	TYPE=SUBSTITUTE
CM032587.1	1639390	.	G	A	100	*	TYPE=SUBSTITUTE
CM032587.1	1639414	.	A	g	100	*	TYPE=SUBSTITUTE
CM032587.1	1639432	.	G	a	100	*	TYPE=SUBSTITUTE
CM032587.1	1639438	.	A	G	100	*	TYPE=SUBSTITUTE
CM032587.1	1639486	.	G	A	100	*	TYPE=SUBSTITUTE

CM032587.1	1639528	.	G	T	100	*	TYPE=SUBSTITUTE
CM032587.1	1639561	.	T	C	100	*	TYPE=SUBSTITUTE
CM032587.1	1639565	.	G	A	100	*	TYPE=SUBSTITUTE
CM032587.1	1639568	.	A	G	100	*	TYPE=SUBSTITUTE
CM032587.1	1639571	.	A	G	100	*	TYPE=SUBSTITUTE
CM032587.1	1639573	.	A	T	100	*	TYPE=SUBSTITUTE
CM032587.1	1639580	.	C	A	100	*	TYPE=SUBSTITUTE
CM032587.1	1639581	.	T	A	100	*	TYPE=SUBSTITUTE
CM032587.1	1639582	.	C	T	100	*	TYPE=SUBSTITUTE
CM032587.1	1639595	.	A	G	100	*	TYPE=SUBSTITUTE
CM032587.1	1639638	.	T	C	100	*	TYPE=SUBSTITUTE
CM032587.1	1639655	.	G	GT	100	*	TYPE=INSERT
CM032587.1	1639668	.	A	C	100	*	TYPE=SUBSTITUTE
CM032587.1	1639674	.	A	t	100	*	TYPE=SUBSTITUTE
CM032587.1	1639681	.	AG	A	100	*	TYPE=DELETE
CM032587.1	1639702	.	A	C	100	*	TYPE=SUBSTITUTE
CM032587.1	1639718	.	C	T	100	*	TYPE=SUBSTITUTE
CM032587.1	1639728	.	C	T	100	*	TYPE=SUBSTITUTE
CM032587.1	1639736	.	A	G	100	*	TYPE=SUBSTITUTE
CM032587.1	1639756	.	A	G	100	*	TYPE=SUBSTITUTE
CM032587.1	1639770	.	T	A	100	*	TYPE=SUBSTITUTE
CM032587.1	1639779	.	A	G	100	*	TYPE=SUBSTITUTE
CM032587.1	1639785	.	A	G	100	*	TYPE=SUBSTITUTE
CM032587.1	1639786	.	T	G	100	*	TYPE=SUBSTITUTE
CM032587.1	1639789	.	T	A	100	*	TYPE=SUBSTITUTE
CM032587.1	1639804	.	C	t	100	*	TYPE=SUBSTITUTE
CM032587.1	1639829	.	G	t	100	*	TYPE=SUBSTITUTE
CM032587.1	1639844	.	T	C	100	*	TYPE=SUBSTITUTE
CM032587.1	1639848	.	G	A	100	*	TYPE=SUBSTITUTE
CM032587.1	1639851	.	T	C	100	*	TYPE=SUBSTITUTE
CM032587.1	1639854	.	T	C	100	*	TYPE=SUBSTITUTE
CM032587.1	1639859	.	T	C	100	*	TYPE=SUBSTITUTE
CM032587.1	1639863	.	T	G	100	*	TYPE=SUBSTITUTE
CM032587.1	1639866	.	T	G	100	*	TYPE=SUBSTITUTE
CM032587.1	1639867	.	G	T	100	*	TYPE=SUBSTITUTE
CM032587.1	1639869	.	A	G	100	*	TYPE=SUBSTITUTE
CM032587.1	1639877	.	ATTAATC	A	100	*	TYPE=DELETE
CM032587.1	1639880	.	G	C	100	*	TYPE=SUBSTITUTE
CM032587.1	1639885	.	C	T	100	*	TYPE=SUBSTITUTE
CM032587.1	1639893	.	A	G	100	*	TYPE=SUBSTITUTE
CM032587.1	1639896	.	C	T	100	*	TYPE=SUBSTITUTE
CM032587.1	1639900	.	C	A	100	*	TYPE=SUBSTITUTE
CM032587.1	1639901	.	A	AT	100	*	TYPE=INSERT
CM032587.1	1639914	.	C	G	100	*	TYPE=SUBSTITUTE
CM032587.1	1639917	.	A	G	100	*	TYPE=SUBSTITUTE
CM032587.1	1639927	.	G	A	100	*	TYPE=SUBSTITUTE
CM032587.1	1639936	.	A	G	100	*	TYPE=SUBSTITUTE
CM032587.1	1639947	.	A	T	100	*	TYPE=SUBSTITUTE
CM032587.1	1639952	.	ACATT	A	100	*	TYPE=DELETE
CM032587.1	1639953	.	G	A	100	*	TYPE=SUBSTITUTE
CM032587.1	1639958	.	C	A	100	*	TYPE=SUBSTITUTE
CM032587.1	1639970	.	T	C	100	*	TYPE=SUBSTITUTE
CM032587.1	1639977	.	A	G	100	*	TYPE=SUBSTITUTE
CM032587.1	1639981	.	A	G	100	*	TYPE=SUBSTITUTE
CM032587.1	1639983	.	A	G	100	*	TYPE=SUBSTITUTE
CM032587.1	1639984	.	T	A	100	*	TYPE=SUBSTITUTE
CM032587.1	1639985	.	G	T	100	*	TYPE=SUBSTITUTE
CM032587.1	1639986	.	C	T	100	*	TYPE=SUBSTITUTE
CM032587.1	1639988	.	T	A	100	*	TYPE=SUBSTITUTE
CM032587.1	1639990	.	G	A	100	*	TYPE=SUBSTITUTE
CM032587.1	1639991	.	A	C	100	*	TYPE=SUBSTITUTE
CM032587.1	1640011	.	G	a	100	*	TYPE=SUBSTITUTE
CM032587.1	1640018	.	A	g	100	*	TYPE=SUBSTITUTE
CM032587.1	1640019	.	A	T	100	*	TYPE=SUBSTITUTE

CM032587.1	1640032	.	T	C	100	*	TYPE=SUBSTITUTE
CM032587.1	1640034	.	G	A	100	*	TYPE=SUBSTITUTE
CM032587.1	1640048	.	G	A	100	*	TYPE=SUBSTITUTE
CM032587.1	1640052	.	T	C	100	*	TYPE=SUBSTITUTE
CM032587.1	1640106	.	G	a	100	*	TYPE=SUBSTITUTE
CM032587.1	1640115	.	T	c	100	*	TYPE=SUBSTITUTE
CM032587.1	1640144	.	C	a	100	*	TYPE=SUBSTITUTE
CM032587.1	1640148	.	C	G	100	*	TYPE=SUBSTITUTE
CM032587.1	1640155	.	CTA	C	100	*	TYPE=DELETE
CM032587.1	1640158	.	T	C	100	*	TYPE=SUBSTITUTE
CM032587.1	1640168	.	C	T	100	*	TYPE=SUBSTITUTE
CM032587.1	1640169	.	T	A	100	*	TYPE=SUBSTITUTE
CM032587.1	1640179	.	AA	A	100	*	TYPE=DELETE
CM032587.1	1640214	.	GA	G	100	*	TYPE=DELETE
CM032587.1	1640220	.	TT	T	100	*	TYPE=DELETE
CM032587.1	1640241	.	T	G	100	*	TYPE=SUBSTITUTE
CM032587.1	1640242	.	T	G	100	*	TYPE=SUBSTITUTE
CM032587.1	1640247	.	T	A	100	*	TYPE=SUBSTITUTE
CM032587.1	1640255	.	A	C	100	*	TYPE=SUBSTITUTE
CM032587.1	1640259	.	G	A	100	*	TYPE=SUBSTITUTE
CM032587.1	1640269	.	C	T	100	*	TYPE=SUBSTITUTE
CM032587.1	1640272	.	T	G	100	*	TYPE=SUBSTITUTE
CM032587.1	1640301	.	T	C	100	*	TYPE=SUBSTITUTE
CM032587.1	1640304	.	G	A	100	*	TYPE=SUBSTITUTE
CM032587.1	1640309	.	T	C	100	*	TYPE=SUBSTITUTE
CM032587.1	1640313	.	G	A	100	*	TYPE=SUBSTITUTE
CM032587.1	1640315	.	C	T	100	*	TYPE=SUBSTITUTE
CM032587.1	1640316	.	C	T	100	*	TYPE=SUBSTITUTE
CM032587.1	1640321	.	A	G	100	*	TYPE=SUBSTITUTE
CM032587.1	1640333	.	T	C	100	*	TYPE=SUBSTITUTE
CM032587.1	1640423	.	T	C	100	*	TYPE=SUBSTITUTE
CM032587.1	1640447	.	C	T	100	*	TYPE=SUBSTITUTE
CM032587.1	1640456	.	T	C	100	*	TYPE=SUBSTITUTE
CM032587.1	1640480	.	C	T	100	*	TYPE=SUBSTITUTE
CM032587.1	1640489	.	T	C	100	*	TYPE=SUBSTITUTE
CM032587.1	1640492	.	T	C	100	*	TYPE=SUBSTITUTE
CM032587.1	1640531	.	T	C	100	*	TYPE=SUBSTITUTE
CM032587.1	1640663	.	A	G	100	*	TYPE=SUBSTITUTE
CM032587.1	1640684	.	A	G	100	*	TYPE=SUBSTITUTE
CM032587.1	1640687	.	T	C	100	*	TYPE=SUBSTITUTE
CM032587.1	1640717	.	T	C	100	*	TYPE=SUBSTITUTE
CM032587.1	1640734	.	A	G	100	*	TYPE=SUBSTITUTE
CM032587.1	1640750	.	C	T	100	*	TYPE=SUBSTITUTE
CM032587.1	1640756	.	T	C	100	*	TYPE=SUBSTITUTE
CM032587.1	1640834	.	T	C	100	*	TYPE=SUBSTITUTE
CM032587.1	1640843	.	A	T	100	*	TYPE=SUBSTITUTE
CM032587.1	1640846	.	T	G	100	*	TYPE=SUBSTITUTE
CM032587.1	1640855	.	C	T	100	*	TYPE=SUBSTITUTE
CM032587.1	1640915	.	T	C	100	*	TYPE=SUBSTITUTE
CM032587.1	1640918	.	C	T	100	*	TYPE=SUBSTITUTE
CM032587.1	1640924	.	T	C	100	*	TYPE=SUBSTITUTE
CM032587.1	1640936	.	A	T	100	*	TYPE=SUBSTITUTE
CM032587.1	1640945	.	T	C	100	*	TYPE=SUBSTITUTE
CM032587.1	1641011	.	T	C	100	*	TYPE=SUBSTITUTE
CM032587.1	1641062	.	G	C	100	*	TYPE=SUBSTITUTE
CM032587.1	1641065	.	G	A	100	*	TYPE=SUBSTITUTE
CM032587.1	1641067	.	T	C	100	*	TYPE=SUBSTITUTE
CM032587.1	1641095	.	C	T	100	*	TYPE=SUBSTITUTE
CM032587.1	1641128	.	T	C	100	*	TYPE=SUBSTITUTE
CM032587.1	1641161	.	C	T	100	*	TYPE=SUBSTITUTE
CM032587.1	1641179	.	T	C	100	*	TYPE=SUBSTITUTE
CM032587.1	1641224	.	C	T	100	*	TYPE=SUBSTITUTE
CM032587.1	1641269	.	T	C	100	*	TYPE=SUBSTITUTE
CM032587.1	1641305	.	C	T	100	*	TYPE=SUBSTITUTE

CM032587.1	1641668	.	A	G	100	*	TYPE=SUBSTITUTE
CM032587.1	1641723	.	CC	C	100	*	TYPE=DELETE
CM032587.1	1641765	.	T	C	100	*	TYPE=SUBSTITUTE
CM032587.1	1642023	.	C	A	100	*	TYPE=SUBSTITUTE
CM032587.1	1642034	.	A	C	100	*	TYPE=SUBSTITUTE
CM032587.1	1642039	.	A	G	100	*	TYPE=SUBSTITUTE
CM032587.1	1642041	.	CC	C	100	*	TYPE=DELETE
CM032587.1	1642063	.	T	g	100	*	TYPE=SUBSTITUTE
CM032587.1	1642066	.	A	C	100	*	TYPE=SUBSTITUTE
CM032587.1	1642075	.	T	G	100	*	TYPE=SUBSTITUTE
CM032587.1	1642126	.	T	C	100	*	TYPE=SUBSTITUTE
CM032587.1	1642144	.	GGT	G	100	*	TYPE=DELETE
CM032587.1	1642157	.	CT	C	100	*	TYPE=DELETE
CM032587.1	1642170	.	T	C	100	*	TYPE=SUBSTITUTE
CM032587.1	1642189	.	C	T	100	*	TYPE=SUBSTITUTE
CM032587.1	1642193	.	T	C	100	*	TYPE=SUBSTITUTE
CM032587.1	1642221	.	T	C	100	*	TYPE=SUBSTITUTE
CM032587.1	1642242	.	G	t	100	*	TYPE=SUBSTITUTE
CM032587.1	1642244	.	C	a	100	*	TYPE=SUBSTITUTE
CM032587.1	1642250	.	G	a	100	*	TYPE=SUBSTITUTE
CM032587.1	1642273	.	C	T	100	*	TYPE=SUBSTITUTE
CM032587.1	1642275	.	T	G	100	*	TYPE=SUBSTITUTE
CM032587.1	1642281	.	C	T	100	*	TYPE=SUBSTITUTE
CM032587.1	1642286	.	T	G	100	*	TYPE=SUBSTITUTE
CM032587.1	1642287	.	T	G	100	*	TYPE=SUBSTITUTE
CM032587.1	1642295	.	c	ct	100	*	TYPE=INSERT
CM032587.1	1642303	.	T	g	100	*	TYPE=SUBSTITUTE
CM032587.1	1642307	.	C	T	100	*	TYPE=SUBSTITUTE
CM032587.1	1642309	.	T	A	100	*	TYPE=SUBSTITUTE
CM032587.1	1642339	.	C	A	100	*	TYPE=SUBSTITUTE
CM032587.1	1642411	.	A	T	100	*	TYPE=SUBSTITUTE
CM032587.1	1642442	.	T	G	100	*	TYPE=SUBSTITUTE
CM032587.1	1642458	.	T	a	100	*	TYPE=SUBSTITUTE
CM032587.1	1642459	.	A	t	100	*	TYPE=SUBSTITUTE
CM032587.1	1642460	.	C	t	100	*	TYPE=SUBSTITUTE
CM032587.1	1642464	.	T	c	100	*	TYPE=SUBSTITUTE
CM032587.1	1642471	.	T	G	100	*	TYPE=SUBSTITUTE
CM032587.1	1642504	.	C	T	100	*	TYPE=SUBSTITUTE
CM032587.1	1642506	.	A	G	100	*	TYPE=SUBSTITUTE
CM032587.1	1642510	.	C	CA	100	*	TYPE=INSERT
CM032587.1	1642534	.	T	C	100	*	TYPE=SUBSTITUTE
CM032587.1	1642535	.	T	A	100	*	TYPE=SUBSTITUTE
CM032587.1	1642536	.	A	T	100	*	TYPE=SUBSTITUTE
CM032587.1	1642556	.	T	C	100	*	TYPE=SUBSTITUTE
CM032587.1	1642558	.	T	c	100	*	TYPE=SUBSTITUTE
CM032587.1	1642559	.	T	c	100	*	TYPE=SUBSTITUTE
CM032587.1	1642560	.	t	tc	100	*	TYPE=INSERT
CM032587.1	1642628	.	G	C	100	*	TYPE=SUBSTITUTE
CM032587.1	1642659	.	TT	T	100	*	TYPE=DELETE
CM032587.1	1642696	.	C	T	100	*	TYPE=SUBSTITUTE
CM032587.1	1642702	.	T	A	100	*	TYPE=SUBSTITUTE
CM032587.1	1642710	.	T	C	100	*	TYPE=SUBSTITUTE
CM032587.1	1642734	.	A	T	100	*	TYPE=SUBSTITUTE
CM032587.1	1642739	.	C	CAACCTTGG	100	*	TYPE=INSERT
CM032587.1	1642745	.	C	T	100	*	TYPE=SUBSTITUTE
CM032587.1	1642762	.	G	A	100	*	TYPE=SUBSTITUTE
CM032587.1	1642767	.	CC	C	100	*	TYPE=DELETE
CM032587.1	1642796	.	A	C	100	*	TYPE=SUBSTITUTE
CM032587.1	1642832	.	G	A	100	*	TYPE=SUBSTITUTE
CM032587.1	1642841	.	G	T	100	*	TYPE=SUBSTITUTE
CM032587.1	1642842	.	T	C	100	*	TYPE=SUBSTITUTE
CM032587.1	1642843	.	T	A	100	*	TYPE=SUBSTITUTE
CM032587.1	1642844	.	C	A	100	*	TYPE=SUBSTITUTE
CM032587.1	1642846	.	T	A	100	*	TYPE=SUBSTITUTE

CM032587.1	1642847	.	C	G	100	*	TYPE=SUBSTITUTE
CM032587.1	1642848	.	G	T	100	*	TYPE=SUBSTITUTE
CM032587.1	1642851	.	G	C	100	*	TYPE=SUBSTITUTE
CM032587.1	1642852	.	G	T	100	*	TYPE=SUBSTITUTE
CM032587.1	1642854	.	G	A	100	*	TYPE=SUBSTITUTE
CM032587.1	1642855	.	T	A	100	*	TYPE=SUBSTITUTE
CM032587.1	1642857	.	C	T	100	*	TYPE=SUBSTITUTE
CM032587.1	1642860	.	A	G	100	*	TYPE=SUBSTITUTE
CM032587.1	1642861	.	T	TC	100	*	TYPE=INSERT
CM032587.1	1642866	.	A	C	100	*	TYPE=SUBSTITUTE
CM032587.1	1642870	.	CGTG	C	100	*	TYPE=DELETE
CM032587.1	1642877	.	TTAA	T	100	*	TYPE=DELETE
CM032587.1	1642878	.	A	G	100	*	TYPE=SUBSTITUTE
CM032587.1	1642881	.	A	T	100	*	TYPE=SUBSTITUTE
CM032587.1	1642882	.	G	C	100	*	TYPE=SUBSTITUTE
CM032587.1	1642883	.	C	T	100	*	TYPE=SUBSTITUTE
CM032587.1	1642884	.	T	C	100	*	TYPE=SUBSTITUTE
CM032587.1	1642887	.	A	G	100	*	TYPE=SUBSTITUTE
CM032587.1	1642888	.	C	T	100	*	TYPE=SUBSTITUTE
CM032587.1	1642890	.	A	T	100	*	TYPE=SUBSTITUTE
CM032587.1	1642892	.	A	T	100	*	TYPE=SUBSTITUTE
CM032587.1	1642894	.	C	A	100	*	TYPE=SUBSTITUTE
CM032587.1	1642897	.	T	G	100	*	TYPE=SUBSTITUTE
CM032587.1	1642900	.	TCC	T	100	*	TYPE=DELETE
CM032587.1	1642902	.	T	G	100	*	TYPE=SUBSTITUTE
CM032587.1	1642904	.	C	T	100	*	TYPE=SUBSTITUTE
CM032587.1	1642907	.	AC	A	100	*	TYPE=DELETE
CM032587.1	1642908	.	C	t	100	*	TYPE=SUBSTITUTE
CM032587.1	1642909	.	G	t	100	*	TYPE=SUBSTITUTE
CM032587.1	1642911	.	C	t	100	*	TYPE=SUBSTITUTE
CM032587.1	1642912	.	A	g	100	*	TYPE=SUBSTITUTE
CM032587.1	1642917	.	g	gt	100	*	TYPE=INSERT
CM032587.1	1642918	.	C	t	100	*	TYPE=SUBSTITUTE
CM032587.1	1642920	.	A	c	100	*	TYPE=SUBSTITUTE
CM032587.1	1642921	.	T	g	100	*	TYPE=SUBSTITUTE
CM032587.1	1642922	.	A	t	100	*	TYPE=SUBSTITUTE
CM032587.1	1642925	.	T	C	100	*	TYPE=SUBSTITUTE
CM032587.1	1642927	.	AG	A	100	*	TYPE=DELETE
CM032587.1	1642940	.	G	A	100	*	TYPE=SUBSTITUTE
CM032587.1	1642950	.	G	GGATAGC	100	*	TYPE=INSERT
CM032587.1	1642955	.	C	A	100	*	TYPE=SUBSTITUTE
CM032587.1	1642956	.	A	G	100	*	TYPE=SUBSTITUTE
CM032587.1	1642957	.	A	T	100	*	TYPE=SUBSTITUTE
CM032587.1	1642961	.	C	T	100	*	TYPE=SUBSTITUTE
CM032587.1	1642963	.	T	TG	100	*	TYPE=INSERT
CM032587.1	1642967	.	C	CAGAGTAGTATT	100	*	TYPE=INSERT
CM032587.1	1642976	.	T	C	100	*	TYPE=SUBSTITUTE
CM032587.1	1642980	.	T	TACTCA	100	*	TYPE=INSERT
CM032587.1	1642984	.	A	C	100	*	TYPE=SUBSTITUTE
CM032587.1	1642986	.	T	G	100	*	TYPE=SUBSTITUTE
CM032587.1	1642996	.	G	A	100	*	TYPE=SUBSTITUTE
CM032587.1	1642998	.	T	C	100	*	TYPE=SUBSTITUTE
CM032587.1	1643017	.	T	A	100	*	TYPE=SUBSTITUTE
CM032587.1	1643019	.	A	AC	100	*	TYPE=INSERT
CM032587.1	1643026	.	A	G	100	*	TYPE=SUBSTITUTE
CM032587.1	1643027	.	G	T	100	*	TYPE=SUBSTITUTE
CM032587.1	1643064	.	A	T	100	*	TYPE=SUBSTITUTE
CM032587.1	1643159	.	C	CC	100	*	TYPE=INSERT
CM032587.1	1643183	.	T	C	100	*	TYPE=SUBSTITUTE
CM032587.1	1643187	.	GTAC	G	100	*	TYPE=DELETE
CM032587.1	1643194	.	G	T	100	*	TYPE=SUBSTITUTE
CM032587.1	1643209	.	T	C	100	*	TYPE=SUBSTITUTE
CM032587.1	1643225	.	TT	T	100	*	TYPE=DELETE
CM032587.1	1643256	.	GAGTACT	G	100	*	TYPE=DELETE

CM032587.1	1643281	.	GGCA	G	100	*	TYPE=DELETE
CM032587.1	1643288	.	A	G	100	*	TYPE=SUBSTITUTE
CM032587.1	1643289	.	C	A	100	*	TYPE=SUBSTITUTE
CM032587.1	1643290	.	A	ATG	100	*	TYPE=INSERT
CM032587.1	1643352	.	A	G	100	*	TYPE=SUBSTITUTE
CM032587.1	1643358	.	ATGCAC	A	100	*	TYPE=DELETE
CM032587.1	1643372	.	GAAACTGGGACTA	G	100	*	TYPE=DELETE
CM032587.1	1643378	.	A	T	100	*	TYPE=SUBSTITUTE
CM032587.1	1643382	.	AGAG	A	100	*	TYPE=DELETE
CM032587.1	1643407	.	C	t	100	*	TYPE=SUBSTITUTE
CM032587.1	1643415	.	GGCT	G	100	*	TYPE=DELETE
CM032587.1	1643426	.	G	a	100	*	TYPE=SUBSTITUTE
CM032587.1	1643434	.	AG	A	100	*	TYPE=DELETE
CM032587.1	1643437	.	G	a	100	*	TYPE=SUBSTITUTE
CM032587.1	1643440	.	A	g	100	*	TYPE=SUBSTITUTE
CM032587.1	1643445	.	T	c	100	*	TYPE=SUBSTITUTE
CM032587.1	1643451	.	G	A	100	*	TYPE=SUBSTITUTE
CM032587.1	1643462	.	g	gA	100	*	TYPE=INSERT
CM032587.1	1643467	.	CT	C	100	*	TYPE=DELETE
CM032587.1	1643469	.	G	t	100	*	TYPE=SUBSTITUTE
CM032587.1	1643493	.	T	c	100	*	TYPE=SUBSTITUTE
CM032587.1	1643531	.	GG	G	100	*	TYPE=DELETE
CM032587.1	1643557	.	A	C	100	*	TYPE=SUBSTITUTE
CM032587.1	1643570	.	C	CC	100	*	TYPE=INSERT
CM032587.1	1643651	.	A	AC	100	*	TYPE=INSERT
CM032587.1	1643652	.	C	T	100	*	TYPE=SUBSTITUTE
CM032587.1	1643672	.	TT	T	100	*	TYPE=DELETE
CM032587.1	1643701	.	T	C	100	*	TYPE=SUBSTITUTE
CM032587.1	1643726	.	C	T	100	*	TYPE=SUBSTITUTE
CM032587.1	1643728	.	C	T	100	*	TYPE=SUBSTITUTE
CM032587.1	1643730	.	A	G	100	*	TYPE=SUBSTITUTE
CM032587.1	1643747	.	G	A	100	*	TYPE=SUBSTITUTE
CM032587.1	1643749	.	G	C	100	*	TYPE=SUBSTITUTE
CM032587.1	1643750	.	A	T	100	*	TYPE=SUBSTITUTE
CM032587.1	1643753	.	T	A	100	*	TYPE=SUBSTITUTE
CM032587.1	1643754	.	T	A	100	*	TYPE=SUBSTITUTE
CM032587.1	1643755	.	T	G	100	*	TYPE=SUBSTITUTE
CM032587.1	1643759	.	G	C	100	*	TYPE=SUBSTITUTE
CM032587.1	1643765	.	TCAG	T	100	*	TYPE=DELETE
CM032587.1	1643766	.	T	C	100	*	TYPE=SUBSTITUTE
CM032587.1	1643775	.	C	T	100	*	TYPE=SUBSTITUTE
CM032587.1	1643783	.	T	TGCATGAAA	100	*	TYPE=INSERT
CM032587.1	1643790	.	C	T	100	*	TYPE=SUBSTITUTE
CM032587.1	1643800	.	AA	A	100	*	TYPE=DELETE
CM032587.1	1643825	.	AAG	A	100	*	TYPE=DELETE
CM032587.1	1643841	.	T	A	100	*	TYPE=SUBSTITUTE
CM032587.1	1643842	.	A	C	100	*	TYPE=SUBSTITUTE
CM032587.1	1643843	.	A	C	100	*	TYPE=SUBSTITUTE
CM032587.1	1643845	.	A	C	100	*	TYPE=SUBSTITUTE
CM032587.1	1643849	.	G	A	100	*	TYPE=SUBSTITUTE
CM032587.1	1643850	.	G	C	100	*	TYPE=SUBSTITUTE
CM032587.1	1643853	.	T	TG	100	*	TYPE=INSERT
CM032587.1	1643906	.	C	A	100	*	TYPE=SUBSTITUTE
CM032587.1	1643908	.	AG	A	100	*	TYPE=DELETE
CM032587.1	1643924	.	C	T	100	*	TYPE=SUBSTITUTE
CM032587.1	1643938	.	TTAG	T	100	*	TYPE=DELETE
CM032587.1	1643947	.	T	A	100	*	TYPE=SUBSTITUTE
CM032587.1	1643957	.	T	C	100	*	TYPE=SUBSTITUTE
CM032587.1	1643994	.	ACT	A	100	*	TYPE=DELETE
CM032587.1	1643995	.	G	T	100	*	TYPE=SUBSTITUTE
CM032587.1	1643999	.	T	G	100	*	TYPE=SUBSTITUTE
CM032587.1	1644000	.	T	G	100	*	TYPE=SUBSTITUTE
CM032587.1	1644003	.	A	G	100	*	TYPE=SUBSTITUTE
CM032587.1	1644004	.	G	A	100	*	TYPE=SUBSTITUTE

CM032587.1 1644008 . GT G 100 * TYPE=DELETE
CM032587.1 1644031 . CAGTGCCG C 100 * TYPE=DELETE
CM032587.1 1644050 . G C 100 * TYPE=SUBSTITUTE
CM032587.1 1644051 . A C 100 * TYPE=SUBSTITUTE
CM032587.1 1644053 . C T 100 * TYPE=SUBSTITUTE
CM032587.1 1644055 . T G 100 * TYPE=SUBSTITUTE
CM032587.1 1644073 . AAAGGAGAGAGAGAGAGC A 100 *
TYPE=DELETE
CM032587.1 1644094 . A G 100 * TYPE=SUBSTITUTE
CM032587.1 1644096 . CA C 100 * TYPE=DELETE
CM032587.1 1644099 . G A 100 * TYPE=SUBSTITUTE
CM032587.1 1644113 . T G 100 * TYPE=SUBSTITUTE
CM032587.1 1644121 . T C 100 * TYPE=SUBSTITUTE
CM032587.1 1644123 . A G 100 * TYPE=SUBSTITUTE
CM032587.1 1644127 . G T 100 * TYPE=SUBSTITUTE
CM032587.1 1644139 . G A 100 * TYPE=SUBSTITUTE
CM032587.1 1644141 . T TTACA 100 * TYPE=INSERT
CM032587.1 1644156 . G GC 100 * TYPE=INSERT
CM032587.1 1644164 . C CTTGAGGACAAAACAGGACTCGAAGAG 100 *
TYPE=INSERT
CM032587.1 1644280 . TTG T 100 * TYPE=DELETE
CM032587.1 1644287 . A t 100 * TYPE=SUBSTITUTE
CM032587.1 1644294 . G A 100 * TYPE=SUBSTITUTE
CM032587.1 1644297 . C T 100 * TYPE=SUBSTITUTE
CM032587.1 1644306 . T C 100 * TYPE=SUBSTITUTE
CM032587.1 1644314 . G T 100 * TYPE=SUBSTITUTE
CM032587.1 1644324 . A C 100 * TYPE=SUBSTITUTE
CM032587.1 1644332 . TCGAG T 100 * TYPE=DELETE
CM032587.1 1644361 . T C 100 * TYPE=SUBSTITUTE
CM032587.1 1644367 . GAATTG G 100 * TYPE=DELETE
CM032587.1 1644395 . TTGACACAA T 100 * TYPE=DELETE
CM032587.1 1644406 . AG A 100 * TYPE=DELETE
CM032587.1 1644429 . CT C 100 * TYPE=DELETE
CM032587.1 1644435 . G A 100 * TYPE=SUBSTITUTE
CM032587.1 1644492 . TTTTGTAAGATATATGCCGGGCAAGGGACTAAAAAGCTGAGCAAA T
100 * TYPE=DELETE
CM032587.1 1644588 . T C 100 * TYPE=SUBSTITUTE
CM032587.1 1644639 . CG C 100 * TYPE=DELETE
CM032587.1 1644654 . A G 100 * TYPE=SUBSTITUTE
CM032587.1 1644667 . CT C 100 * TYPE=DELETE
CM032587.1 1644676 . C CT 100 * TYPE=INSERT
CM032587.1 1644678 . T C 100 * TYPE=SUBSTITUTE
CM032587.1 1644692 . C T 100 * TYPE=SUBSTITUTE
CM032587.1 1644746 . A G 100 * TYPE=SUBSTITUTE
CM032587.1 1644747 . G A 100 * TYPE=SUBSTITUTE
CM032587.1 1644762 . T C 100 * TYPE=SUBSTITUTE
CM032587.1 1644763 . C T 100 * TYPE=SUBSTITUTE
CM032587.1 1644792 . GGAC G 100 * TYPE=DELETE
CM032587.1 1644798 . C T 100 * TYPE=SUBSTITUTE
CM032587.1 1644844 . AT A 100 * TYPE=DELETE
CM032587.1 1644906 . T G 100 * TYPE=SUBSTITUTE
CM032587.1 1644920 . A G 100 * TYPE=SUBSTITUTE
CM032587.1 1644944 . A G 100 * TYPE=SUBSTITUTE
CM032587.1 1644947 . GAC G 100 * TYPE=DELETE
CM032587.1 1644949 . T C 100 * TYPE=SUBSTITUTE
CM032587.1 1644971 . TGCAT T 100 * TYPE=DELETE
CM032587.1 1644992 . T C 100 * TYPE=SUBSTITUTE
CM032587.1 1645019 . G C 100 * TYPE=SUBSTITUTE
CM032587.1 1645020 . C G 100 * TYPE=SUBSTITUTE
CM032587.1 1645025 . A G 100 * TYPE=SUBSTITUTE
CM032587.1 1645028 . C A 100 * TYPE=SUBSTITUTE
CM032587.1 1645083 . A c 100 * TYPE=SUBSTITUTE
CM032587.1 1645118 . G A 100 * TYPE=SUBSTITUTE
CM032587.1 1645124 . A G 100 * TYPE=SUBSTITUTE

CM032587.1	1645144	.	T	C	100	*	TYPE=SUBSTITUTE
CM032587.1	1645175	.	T	c	100	*	TYPE=SUBSTITUTE
CM032587.1	1645217	.	A	G	100	*	TYPE=SUBSTITUTE
CM032587.1	1645241	.	G	A	100	*	TYPE=SUBSTITUTE
CM032587.1	1645298	.	T	G	100	*	TYPE=SUBSTITUTE
CM032587.1	1645310	.	A	G	100	*	TYPE=SUBSTITUTE
CM032587.1	1645313	.	T	C	100	*	TYPE=SUBSTITUTE
CM032587.1	1645340	.	A	G	100	*	TYPE=SUBSTITUTE
CM032587.1	1645367	.	A	C	100	*	TYPE=SUBSTITUTE
CM032587.1	1645394	.	C	T	100	*	TYPE=SUBSTITUTE
CM032587.1	1645403	.	C	T	100	*	TYPE=SUBSTITUTE
CM032587.1	1645409	.	T	C	100	*	TYPE=SUBSTITUTE
CM032587.1	1645424	.	A	G	100	*	TYPE=SUBSTITUTE
CM032587.1	1645433	.	A	G	100	*	TYPE=SUBSTITUTE
CM032587.1	1645462	.	C	T	100	*	TYPE=SUBSTITUTE
CM032587.1	1645481	.	G	T	100	*	TYPE=SUBSTITUTE
CM032587.1	1645499	.	C	T	100	*	TYPE=SUBSTITUTE
CM032587.1	1645505	.	C	A	100	*	TYPE=SUBSTITUTE
CM032587.1	1645648	.	T	C	100	*	TYPE=SUBSTITUTE
CM032587.1	1645649	.	C	T	100	*	TYPE=SUBSTITUTE
CM032587.1	1645781	.	A	C	100	*	TYPE=SUBSTITUTE
CM032587.1	1645886	.	T	C	100	*	TYPE=SUBSTITUTE
CM032587.1	1645901	.	C	T	100	*	TYPE=SUBSTITUTE
CM032587.1	1645940	.	G	A	100	*	TYPE=SUBSTITUTE
CM032587.1	1645952	.	A	G	100	*	TYPE=SUBSTITUTE
CM032587.1	1645994	.	G	A	100	*	TYPE=SUBSTITUTE
CM032587.1	1646030	.	C	T	100	*	TYPE=SUBSTITUTE
CM032587.1	1646033	.	G	C	100	*	TYPE=SUBSTITUTE
CM032587.1	1646057	.	C	T	100	*	TYPE=SUBSTITUTE
CM032587.1	1646090	.	T	C	100	*	TYPE=SUBSTITUTE
CM032587.1	1646099	.	C	T	100	*	TYPE=SUBSTITUTE
CM032587.1	1646117	.	T	C	100	*	TYPE=SUBSTITUTE
CM032587.1	1646135	.	T	A	100	*	TYPE=SUBSTITUTE
CM032587.1	1646138	.	C	T	100	*	TYPE=SUBSTITUTE
CM032587.1	1646144	.	G	a	100	*	TYPE=SUBSTITUTE
CM032587.1	1646147	.	C	t	100	*	TYPE=SUBSTITUTE
CM032587.1	1646174	.	C	T	100	*	TYPE=SUBSTITUTE
CM032587.1	1646177	.	A	G	100	*	TYPE=SUBSTITUTE
CM032587.1	1646233	.	T	C	100	*	TYPE=SUBSTITUTE
CM032587.1	1646246	.	T	C	100	*	TYPE=SUBSTITUTE
CM032587.1	1646249	.	A	C	100	*	TYPE=SUBSTITUTE
CM032587.1	1646252	.	T	C	100	*	TYPE=SUBSTITUTE
CM032587.1	1646257	.	C	A	100	*	TYPE=SUBSTITUTE
CM032587.1	1646297	.	C	T	100	*	TYPE=SUBSTITUTE
CM032587.1	1646300	.	G	A	100	*	TYPE=SUBSTITUTE
CM032587.1	1646307	.	G	A	100	*	TYPE=SUBSTITUTE
CM032587.1	1646308	.	A	C	100	*	TYPE=SUBSTITUTE
CM032587.1	1646310	.	A	G	100	*	TYPE=SUBSTITUTE
CM032587.1	1646327	.	G	A	100	*	TYPE=SUBSTITUTE
CM032587.1	1646330	.	A	T	100	*	TYPE=SUBSTITUTE
CM032587.1	1646342	.	G	A	100	*	TYPE=SUBSTITUTE
CM032587.1	1646354	.	A	T	100	*	TYPE=SUBSTITUTE
CM032587.1	1646378	.	A	G	100	*	TYPE=SUBSTITUTE
CM032587.1	1646390	.	C	T	100	*	TYPE=SUBSTITUTE
CM032587.1	1646399	.	C	T	100	*	TYPE=SUBSTITUTE
CM032587.1	1646422	.	T	C	100	*	TYPE=SUBSTITUTE
CM032587.1	1646441	.	G	A	100	*	TYPE=SUBSTITUTE
CM032587.1	1646465	.	G	a	100	*	TYPE=SUBSTITUTE
CM032587.1	1646474	.	C	g	100	*	TYPE=SUBSTITUTE
CM032587.1	1646480	.	C	T	100	*	TYPE=SUBSTITUTE
CM032587.1	1646492	.	A	G	100	*	TYPE=SUBSTITUTE
CM032587.1	1646507	.	A	G	100	*	TYPE=SUBSTITUTE
CM032587.1	1646510	.	A	C	100	*	TYPE=SUBSTITUTE
CM032587.1	1646533	.	C	t	100	*	TYPE=SUBSTITUTE

CM032587.1	1646534	.	C	t	100	*	TYPE=SUBSTITUTE
CM032587.1	1646537	.	C	t	100	*	TYPE=SUBSTITUTE
CM032587.1	1646558	.	G	A	100	*	TYPE=SUBSTITUTE
CM032587.1	1646567	.	T	C	100	*	TYPE=SUBSTITUTE
CM032587.1	1646648	.	T	A	100	*	TYPE=SUBSTITUTE
CM032587.1	1646738	.	C	T	100	*	TYPE=SUBSTITUTE
CM032587.1	1646744	.	C	T	100	*	TYPE=SUBSTITUTE
CM032587.1	1646762	.	C	T	100	*	TYPE=SUBSTITUTE
CM032587.1	1646783	.	A	G	100	*	TYPE=SUBSTITUTE
CM032587.1	1646795	.	G	A	100	*	TYPE=SUBSTITUTE
CM032587.1	1646804	.	T	C	100	*	TYPE=SUBSTITUTE
CM032587.1	1646819	.	G	A	100	*	TYPE=SUBSTITUTE
CM032587.1	1646840	.	G	A	100	*	TYPE=SUBSTITUTE
CM032587.1	1646849	.	C	A	100	*	TYPE=SUBSTITUTE
CM032587.1	1646867	.	T	C	100	*	TYPE=SUBSTITUTE
CM032587.1	1646876	.	G	A	100	*	TYPE=SUBSTITUTE
CM032587.1	1646891	.	C	T	100	*	TYPE=SUBSTITUTE
CM032587.1	1646894	.	T	C	100	*	TYPE=SUBSTITUTE
CM032587.1	1646897	.	A	G	100	*	TYPE=SUBSTITUTE
CM032587.1	1646906	.	T	C	100	*	TYPE=SUBSTITUTE
CM032587.1	1646921	.	C	T	100	*	TYPE=SUBSTITUTE
CM032587.1	1646942	.	C	T	100	*	TYPE=SUBSTITUTE
CM032587.1	1646951	.	C	T	100	*	TYPE=SUBSTITUTE
CM032587.1	1646972	.	G	A	100	*	TYPE=SUBSTITUTE
CM032587.1	1646975	.	C	T	100	*	TYPE=SUBSTITUTE
CM032587.1	1646981	.	C	T	100	*	TYPE=SUBSTITUTE
CM032587.1	1647014	.	G	A	100	*	TYPE=SUBSTITUTE
CM032587.1	1647017	.	C	T	100	*	TYPE=SUBSTITUTE
CM032587.1	1647025	.	T	C	100	*	TYPE=SUBSTITUTE
CM032587.1	1647029	.	C	G	100	*	TYPE=SUBSTITUTE
CM032587.1	1647032	.	C	T	100	*	TYPE=SUBSTITUTE
CM032587.1	1647038	.	C	G	100	*	TYPE=SUBSTITUTE
CM032587.1	1647041	.	G	A	100	*	TYPE=SUBSTITUTE
CM032587.1	1647062	.	C	T	100	*	TYPE=SUBSTITUTE
CM032587.1	1647116	.	T	C	100	*	TYPE=SUBSTITUTE
CM032587.1	1647119	.	G	A	100	*	TYPE=SUBSTITUTE
CM032587.1	1647164	.	A	G	100	*	TYPE=SUBSTITUTE
CM032587.1	1647167	.	A	G	100	*	TYPE=SUBSTITUTE
CM032587.1	1647212	.	A	G	100	*	TYPE=SUBSTITUTE
CM032587.1	1647227	.	A	G	100	*	TYPE=SUBSTITUTE
CM032587.1	1647257	.	T	C	100	*	TYPE=SUBSTITUTE
CM032587.1	1647305	.	T	C	100	*	TYPE=SUBSTITUTE
CM032587.1	1647313	.	C	T	100	*	TYPE=SUBSTITUTE
CM032587.1	1647326	.	A	G	100	*	TYPE=SUBSTITUTE
CM032587.1	1647332	.	C	G	100	*	TYPE=SUBSTITUTE
CM032587.1	1647362	.	T	C	100	*	TYPE=SUBSTITUTE
CM032587.1	1647370	.	G	A	100	*	TYPE=SUBSTITUTE
CM032587.1	1647380	.	T	C	100	*	TYPE=SUBSTITUTE
CM032587.1	1647389	.	T	C	100	*	TYPE=SUBSTITUTE
CM032587.1	1647413	.	A	G	100	*	TYPE=SUBSTITUTE
CM032587.1	1647428	.	A	G	100	*	TYPE=SUBSTITUTE
CM032587.1	1647431	.	G	A	100	*	TYPE=SUBSTITUTE
CM032587.1	1647440	.	T	C	100	*	TYPE=SUBSTITUTE
CM032587.1	1647452	.	G	T	100	*	TYPE=SUBSTITUTE
CM032587.1	1647467	.	G	A	100	*	TYPE=SUBSTITUTE
CM032587.1	1647479	.	C	A	100	*	TYPE=SUBSTITUTE
CM032587.1	1647527	.	A	G	100	*	TYPE=SUBSTITUTE
CM032587.1	1647584	.	A	C	100	*	TYPE=SUBSTITUTE
CM032587.1	1647586	.	T	C	100	*	TYPE=SUBSTITUTE
CM032587.1	1647596	.	T	C	100	*	TYPE=SUBSTITUTE
CM032587.1	1647614	.	A	C	100	*	TYPE=SUBSTITUTE
CM032587.1	1647680	.	A	g	100	*	TYPE=SUBSTITUTE
CM032587.1	1647704	.	C	T	100	*	TYPE=SUBSTITUTE
CM032587.1	1647707	.	A	T	100	*	TYPE=SUBSTITUTE

CM032587.1	1647714	.	GA	G	100	*	TYPE=DELETE
CM032587.1	1647804	.	A	T	100	*	TYPE=SUBSTITUTE
CM032587.1	1647822	.	C	A	100	*	TYPE=SUBSTITUTE
CM032587.1	1647843	.	T	C	100	*	TYPE=SUBSTITUTE
CM032587.1	1647867	.	T	C	100	*	TYPE=SUBSTITUTE
CM032587.1	1647930	.	A	g	100	*	TYPE=SUBSTITUTE
CM032587.1	1647939	.	A	g	100	*	TYPE=SUBSTITUTE
CM032587.1	1647942	.	T	c	100	*	TYPE=SUBSTITUTE
CM032587.1	1647989	.	C	G	100	*	TYPE=SUBSTITUTE
CM032587.1	1648058	.	T	C	100	*	TYPE=SUBSTITUTE
CM032587.1	1648060	.	C	A	100	*	TYPE=SUBSTITUTE
CM032587.1	1648125	.	A	C	100	*	TYPE=SUBSTITUTE
CM032587.1	1648141	.	T	C	100	*	TYPE=SUBSTITUTE
CM032587.1	1648146	.	A	G	100	*	TYPE=SUBSTITUTE
CM032587.1	1648149	.	T	C	100	*	TYPE=SUBSTITUTE
CM032587.1	1648208	.	A	G	100	*	TYPE=SUBSTITUTE
CM032587.1	1648211	.	G	A	100	*	TYPE=SUBSTITUTE
CM032587.1	1648282	.	G	A	100	*	TYPE=SUBSTITUTE
CM032587.1	1648352	.	T	G	100	*	TYPE=SUBSTITUTE
CM032587.1	1648504	.	C	A	100	*	TYPE=SUBSTITUTE
CM032587.1	1648521	.	A	G	100	*	TYPE=SUBSTITUTE
CM032587.1	1648529	.	A	G	100	*	TYPE=SUBSTITUTE
CM032587.1	1648583	.	T	C	100	*	TYPE=SUBSTITUTE
CM032587.1	1648584	.	A	C	100	*	TYPE=SUBSTITUTE
CM032587.1	1648669	.	T	G	100	*	TYPE=SUBSTITUTE
CM032587.1	1649076	.	T	C	100	*	TYPE=SUBSTITUTE
CM032587.1	1649151	.	TTT	T	100	*	TYPE=DELETE
CM032587.1	1649490	.	GGG	G	100	*	TYPE=DELETE
CM032587.1	1649720	.	CT	C	100	*	TYPE=DELETE
CM032587.1	1649958	.	G	GN	100	*	TYPE=INSERT
CM032587.1	1650644	.	T	G	100	*	TYPE=SUBSTITUTE
CM032587.1	1650735	.	CA	C	100	*	TYPE=DELETE
CM032587.1	1650738	.	T	C	100	*	TYPE=SUBSTITUTE
CM032587.1	1650763	.	A	G	100	*	TYPE=SUBSTITUTE
CM032587.1	1651084	.	A	g	100	*	TYPE=SUBSTITUTE
CM032587.1	1651286	.	CAACATGC	C	100	*	TYPE=DELETE
CM032587.1	1651471	.	A	T	100	*	TYPE=SUBSTITUTE
CM032587.1	1651472	.	A	G	100	*	TYPE=SUBSTITUTE
CM032587.1	1651543	.	TTT	T	100	*	TYPE=DELETE
CM032587.1	1651977	.	C	T	100	*	TYPE=SUBSTITUTE
CM032587.1	1651980	.	A	G	100	*	TYPE=SUBSTITUTE
CM032587.1	1652023	.	G	T	100	*	TYPE=SUBSTITUTE
CM032587.1	1652088	.	G	A	100	*	TYPE=SUBSTITUTE
CM032587.1	1652097	.	A	G	100	*	TYPE=SUBSTITUTE
CM032587.1	1652173	.	A	G	100	*	TYPE=SUBSTITUTE
CM032587.1	1652175	.	T	C	100	*	TYPE=SUBSTITUTE
CM032587.1	1652223	.	A	G	100	*	TYPE=SUBSTITUTE
CM032587.1	1652261	.	C	g	100	*	TYPE=SUBSTITUTE
CM032587.1	1652262	.	G	C	100	*	TYPE=SUBSTITUTE
CM032587.1	1652295	.	C	A	100	*	TYPE=SUBSTITUTE
CM032587.1	1652298	.	G	A	100	*	TYPE=SUBSTITUTE
CM032587.1	1652301	.	A	G	100	*	TYPE=SUBSTITUTE
CM032587.1	1652313	.	A	C	100	*	TYPE=SUBSTITUTE
CM032587.1	1652354	.	G	A	100	*	TYPE=SUBSTITUTE
CM032587.1	1652359	.	A	G	100	*	TYPE=SUBSTITUTE
CM032587.1	1652370	.	A	G	100	*	TYPE=SUBSTITUTE
CM032587.1	1652388	.	A	G	100	*	TYPE=SUBSTITUTE
CM032587.1	1652400	.	G	A	100	*	TYPE=SUBSTITUTE
CM032587.1	1652406	.	G	C	100	*	TYPE=SUBSTITUTE
CM032587.1	1652469	.	A	G	100	*	TYPE=SUBSTITUTE
CM032587.1	1652529	.	G	A	100	*	TYPE=SUBSTITUTE
CM032587.1	1652539	.	A	G	100	*	TYPE=SUBSTITUTE
CM032587.1	1652554	.	A	G	100	*	TYPE=SUBSTITUTE
CM032587.1	1652555	.	G	A	100	*	TYPE=SUBSTITUTE

CM032587.1	1652559	.	G	A	100	*	TYPE=SUBSTITUTE
CM032587.1	1652568	.	A	G	100	*	TYPE=SUBSTITUTE
CM032587.1	1652628	.	C	T	100	*	TYPE=SUBSTITUTE
CM032587.1	1652772	.	T	C	100	*	TYPE=SUBSTITUTE
CM032587.1	1652778	.	A	G	100	*	TYPE=SUBSTITUTE
CM032587.1	1652850	.	G	A	100	*	TYPE=SUBSTITUTE
CM032587.1	1652854	.	A	G	100	*	TYPE=SUBSTITUTE
CM032587.1	1652857	.	T	G	100	*	TYPE=SUBSTITUTE
CM032587.1	1652875	.	G	A	100	*	TYPE=SUBSTITUTE
CM032587.1	1652879	.	T	C	100	*	TYPE=SUBSTITUTE
CM032587.1	1652880	.	A	G	100	*	TYPE=SUBSTITUTE
CM032587.1	1652884	.	A	G	100	*	TYPE=SUBSTITUTE
CM032587.1	1652907	.	A	G	100	*	TYPE=SUBSTITUTE
CM032587.1	1652919	.	G	a	100	*	TYPE=SUBSTITUTE
CM032587.1	1652940	.	T	G	100	*	TYPE=SUBSTITUTE
CM032587.1	1652943	.	C	G	100	*	TYPE=SUBSTITUTE
CM032587.1	1652946	.	A	G	100	*	TYPE=SUBSTITUTE
CM032587.1	1652949	.	T	A	100	*	TYPE=SUBSTITUTE
CM032587.1	1652952	.	G	T	100	*	TYPE=SUBSTITUTE
CM032587.1	1652968	.	C	T	100	*	TYPE=SUBSTITUTE
CM032587.1	1653018	.	G	T	100	*	TYPE=SUBSTITUTE
CM032587.1	1653111	.	A	G	100	*	TYPE=SUBSTITUTE
CM032587.1	1653123	.	T	C	100	*	TYPE=SUBSTITUTE
CM032587.1	1653150	.	G	A	100	*	TYPE=SUBSTITUTE
CM032587.1	1653213	.	T	C	100	*	TYPE=SUBSTITUTE
CM032587.1	1653228	.	G	A	100	*	TYPE=SUBSTITUTE
CM032587.1	1653238	.	T	C	100	*	TYPE=SUBSTITUTE
CM032587.1	1653246	.	C	T	100	*	TYPE=SUBSTITUTE
CM032587.1	1653247	.	G	T	100	*	TYPE=SUBSTITUTE
CM032587.1	1653257	.	C	t	100	*	TYPE=SUBSTITUTE
CM032587.1	1653258	.	G	c	100	*	TYPE=SUBSTITUTE
CM032587.1	1653259	.	C	t	100	*	TYPE=SUBSTITUTE
CM032587.1	1653262	.	T	c	100	*	TYPE=SUBSTITUTE
CM032587.1	1653334	.	G	A	100	*	TYPE=SUBSTITUTE
CM032587.1	1653337	.	C	T	100	*	TYPE=SUBSTITUTE
CM032587.1	1653342	.	T	C	100	*	TYPE=SUBSTITUTE
CM032587.1	1653348	.	A	T	100	*	TYPE=SUBSTITUTE
CM032587.1	1653349	.	T	C	100	*	TYPE=SUBSTITUTE
CM032587.1	1653368	.	C	T	100	*	TYPE=SUBSTITUTE
CM032587.1	1653391	.	G	A	100	*	TYPE=SUBSTITUTE
CM032587.1	1653393	.	G	A	100	*	TYPE=SUBSTITUTE
CM032587.1	1653400	.	C	A	100	*	TYPE=SUBSTITUTE
CM032587.1	1653402	.	A	C	100	*	TYPE=SUBSTITUTE
CM032587.1	1653403	.	G	A	100	*	TYPE=SUBSTITUTE
CM032587.1	1653404	.	A	G	100	*	TYPE=SUBSTITUTE
CM032587.1	1653407	.	G	T	100	*	TYPE=SUBSTITUTE
CM032587.1	1653408	.	C	A	100	*	TYPE=SUBSTITUTE
CM032587.1	1653409	.	T	G	100	*	TYPE=SUBSTITUTE
CM032587.1	1653412	.	C	A	100	*	TYPE=SUBSTITUTE
CM032587.1	1653414	.	C	T	100	*	TYPE=SUBSTITUTE
CM032587.1	1653415	.	A	C	100	*	TYPE=SUBSTITUTE
CM032587.1	1653416	.	G	A	100	*	TYPE=SUBSTITUTE
CM032587.1	1653418	.	A	C	100	*	TYPE=SUBSTITUTE
CM032587.1	1653420	.	A	G	100	*	TYPE=SUBSTITUTE
CM032587.1	1653423	.	G	A	100	*	TYPE=SUBSTITUTE
CM032587.1	1653424	.	A	AAC	100	*	TYPE=INSERT
CM032587.1	1653430	.	C	T	100	*	TYPE=SUBSTITUTE
CM032587.1	1653432	.	G	T	100	*	TYPE=SUBSTITUTE
CM032587.1	1653433	.	T	TCG	100	*	TYPE=INSERT
CM032587.1	1653435	.	T	G	100	*	TYPE=SUBSTITUTE
CM032587.1	1653436	.	A	G	100	*	TYPE=SUBSTITUTE
CM032587.1	1653437	.	C	A	100	*	TYPE=SUBSTITUTE
CM032587.1	1653440	.	A	G	100	*	TYPE=SUBSTITUTE
CM032587.1	1653441	.	A	G	100	*	TYPE=SUBSTITUTE

CM032587.1	1653442	.	T	A	100	*	TYPE=SUBSTITUTE
CM032587.1	1653443	.	C	G	100	*	TYPE=SUBSTITUTE
CM032587.1	1653445	.	A	T	100	*	TYPE=SUBSTITUTE
CM032587.1	1653447	.	G	T	100	*	TYPE=SUBSTITUTE
CM032587.1	1653449	.	A	T	100	*	TYPE=SUBSTITUTE
CM032587.1	1653450	.	T	A	100	*	TYPE=SUBSTITUTE
CM032587.1	1653453	.	A	G	100	*	TYPE=SUBSTITUTE
CM032587.1	1653454	.	A	T	100	*	TYPE=SUBSTITUTE
CM032587.1	1653456	.	AC	A	100	*	TYPE=DELETE
CM032587.1	1653460	.	TA	T	100	*	TYPE=DELETE
CM032587.1	1653463	.	GT	G	100	*	TYPE=DELETE
CM032587.1	1653464	.	T	A	100	*	TYPE=SUBSTITUTE
CM032587.1	1653471	.	A	AATC	100	*	TYPE=INSERT
CM032587.1	1653472	.	G	A	100	*	TYPE=SUBSTITUTE
CM032587.1	1653475	.	G	A	100	*	TYPE=SUBSTITUTE
CM032587.1	1653490	.	T	G	100	*	TYPE=SUBSTITUTE
CM032587.1	1653499	.	T	TA	100	*	TYPE=INSERT
CM032587.1	1653523	.	C	g	100	*	TYPE=SUBSTITUTE
CM032587.1	1653526	.	C	t	100	*	TYPE=SUBSTITUTE
CM032587.1	1653547	.	C	t	100	*	TYPE=SUBSTITUTE
CM032587.1	1653574	.	T	c	100	*	TYPE=SUBSTITUTE
CM032587.1	1653580	.	C	t	100	*	TYPE=SUBSTITUTE
CM032587.1	1653585	.	A	g	100	*	TYPE=SUBSTITUTE
CM032587.1	1653595	.	T	g	100	*	TYPE=SUBSTITUTE
CM032587.1	1653612	.	A	G	100	*	TYPE=SUBSTITUTE
CM032587.1	1653619	.	A	G	100	*	TYPE=SUBSTITUTE
CM032587.1	1653622	.	C	T	100	*	TYPE=SUBSTITUTE
CM032587.1	1653675	.	A	C	100	*	TYPE=SUBSTITUTE
CM032587.1	1653685	.	A	T	100	*	TYPE=SUBSTITUTE
CM032587.1	1653709	.	A	T	100	*	TYPE=SUBSTITUTE
CM032587.1	1653723	.	G	A	100	*	TYPE=SUBSTITUTE
CM032587.1	1653729	.	C	T	100	*	TYPE=SUBSTITUTE
CM032587.1	1653730	.	T	A	100	*	TYPE=SUBSTITUTE
CM032587.1	1653741	.	A	ACTTAT	100	*	TYPE=INSERT
CM032587.1	1653748	.	A	G	100	*	TYPE=SUBSTITUTE
CM032587.1	1653763	.	G	A	100	*	TYPE=SUBSTITUTE
CM032587.1	1653764	.	T	TTATT	100	*	TYPE=INSERT
CM032587.1	1653799	.	T	A	100	*	TYPE=SUBSTITUTE
CM032587.1	1653809	.	C	A	100	*	TYPE=SUBSTITUTE
CM032587.1	1653818	.	C	A	100	*	TYPE=SUBSTITUTE
CM032587.1	1653883	.	C	T	100	*	TYPE=SUBSTITUTE
CM032587.1	1653891	.	T	C	100	*	TYPE=SUBSTITUTE
CM032587.1	1653892	.	C	T	100	*	TYPE=SUBSTITUTE
CM032587.1	1653895	.	G	C	100	*	TYPE=SUBSTITUTE
CM032587.1	1653961	.	T	C	100	*	TYPE=SUBSTITUTE
CM032587.1	1653976	.	A	G	100	*	TYPE=SUBSTITUTE
CM032587.1	1653982	.	C	A	100	*	TYPE=SUBSTITUTE
CM032587.1	1653997	.	C	T	100	*	TYPE=SUBSTITUTE
CM032587.1	1654012	.	T	C	100	*	TYPE=SUBSTITUTE
CM032587.1	1654018	.	T	C	100	*	TYPE=SUBSTITUTE
CM032587.1	1654030	.	C	T	100	*	TYPE=SUBSTITUTE
CM032587.1	1654042	.	T	C	100	*	TYPE=SUBSTITUTE
CM032587.1	1654084	.	C	T	100	*	TYPE=SUBSTITUTE
CM032587.1	1654114	.	C	G	100	*	TYPE=SUBSTITUTE
CM032587.1	1654117	.	A	G	100	*	TYPE=SUBSTITUTE
CM032587.1	1654123	.	A	C	100	*	TYPE=SUBSTITUTE
CM032587.1	1654126	.	T	A	100	*	TYPE=SUBSTITUTE
CM032587.1	1654135	.	C	T	100	*	TYPE=SUBSTITUTE
CM032587.1	1654144	.	T	C	100	*	TYPE=SUBSTITUTE
CM032587.1	1654147	.	T	C	100	*	TYPE=SUBSTITUTE
CM032587.1	1654156	.	T	C	100	*	TYPE=SUBSTITUTE
CM032587.1	1654186	.	T	C	100	*	TYPE=SUBSTITUTE
CM032587.1	1654204	.	A	G	100	*	TYPE=SUBSTITUTE
CM032587.1	1654210	.	A	T	100	*	TYPE=SUBSTITUTE

CM032587.1	1654222	.	T	C	100	*	TYPE=SUBSTITUTE
CM032587.1	1654333	.	T	c	100	*	TYPE=SUBSTITUTE
CM032587.1	1654348	.	C	T	100	*	TYPE=SUBSTITUTE
CM032587.1	1654366	.	T	C	100	*	TYPE=SUBSTITUTE
CM032587.1	1654378	.	G	C	100	*	TYPE=SUBSTITUTE
CM032587.1	1654411	.	A	G	100	*	TYPE=SUBSTITUTE
CM032587.1	1654486	.	C	T	100	*	TYPE=SUBSTITUTE
CM032587.1	1654491	.	A	G	100	*	TYPE=SUBSTITUTE
CM032587.1	1654570	.	C	g	100	*	TYPE=SUBSTITUTE
CM032587.1	1654636	.	G	A	100	*	TYPE=SUBSTITUTE
CM032587.1	1654648	.	T	C	100	*	TYPE=SUBSTITUTE
CM032587.1	1654660	.	A	G	100	*	TYPE=SUBSTITUTE
CM032587.1	1654687	.	C	T	100	*	TYPE=SUBSTITUTE
CM032587.1	1654729	.	C	T	100	*	TYPE=SUBSTITUTE
CM032587.1	1654804	.	A	C	100	*	TYPE=SUBSTITUTE
CM032587.1	1654831	.	C	T	100	*	TYPE=SUBSTITUTE
CM032587.1	1654840	.	G	A	100	*	TYPE=SUBSTITUTE
CM032587.1	1654855	.	A	G	100	*	TYPE=SUBSTITUTE
CM032587.1	1654870	.	T	C	100	*	TYPE=SUBSTITUTE
CM032587.1	1654882	.	T	C	100	*	TYPE=SUBSTITUTE
CM032587.1	1654909	.	A	G	100	*	TYPE=SUBSTITUTE
CM032587.1	1654930	.	A	G	100	*	TYPE=SUBSTITUTE
CM032587.1	1654948	.	C	A	100	*	TYPE=SUBSTITUTE
CM032587.1	1654951	.	T	C	100	*	TYPE=SUBSTITUTE
CM032587.1	1654960	.	C	T	100	*	TYPE=SUBSTITUTE
CM032587.1	1654969	.	T	C	100	*	TYPE=SUBSTITUTE
CM032587.1	1654981	.	T	C	100	*	TYPE=SUBSTITUTE
CM032587.1	1655005	.	A	G	100	*	TYPE=SUBSTITUTE
CM032587.1	1655053	.	T	C	100	*	TYPE=SUBSTITUTE
CM032587.1	1655122	.	T	C	100	*	TYPE=SUBSTITUTE
CM032587.1	1655125	.	T	A	100	*	TYPE=SUBSTITUTE
CM032587.1	1655176	.	T	C	100	*	TYPE=SUBSTITUTE
CM032587.1	1655179	.	T	C	100	*	TYPE=SUBSTITUTE
CM032587.1	1655197	.	C	T	100	*	TYPE=SUBSTITUTE
CM032587.1	1655269	.	A	G	100	*	TYPE=SUBSTITUTE
CM032587.1	1655293	.	A	C	100	*	TYPE=SUBSTITUTE
CM032587.1	1655295	.	A	G	100	*	TYPE=SUBSTITUTE
CM032587.1	1655308	.	G	A	100	*	TYPE=SUBSTITUTE
CM032587.1	1655350	.	T	C	100	*	TYPE=SUBSTITUTE
CM032587.1	1655431	.	A	G	100	*	TYPE=SUBSTITUTE
CM032587.1	1655497	.	C	T	100	*	TYPE=SUBSTITUTE
CM032587.1	1655518	.	C	T	100	*	TYPE=SUBSTITUTE
CM032587.1	1655539	.	T	C	100	*	TYPE=SUBSTITUTE
CM032587.1	1655587	.	A	G	100	*	TYPE=SUBSTITUTE
CM032587.1	1655602	.	G	A	100	*	TYPE=SUBSTITUTE
CM032587.1	1655641	.	C	T	100	*	TYPE=SUBSTITUTE
CM032587.1	1655645	.	A	G	100	*	TYPE=SUBSTITUTE
CM032587.1	1655659	.	A	G	100	*	TYPE=SUBSTITUTE
CM032587.1	1655680	.	T	G	100	*	TYPE=SUBSTITUTE
CM032587.1	1655683	.	G	A	100	*	TYPE=SUBSTITUTE
CM032587.1	1655707	.	A	G	100	*	TYPE=SUBSTITUTE
CM032587.1	1655774	.	A	G	100	*	TYPE=SUBSTITUTE
CM032587.1	1655776	.	A	G	100	*	TYPE=SUBSTITUTE
CM032587.1	1655788	.	T	C	100	*	TYPE=SUBSTITUTE
CM032587.1	1655800	.	T	C	100	*	TYPE=SUBSTITUTE
CM032587.1	1655884	.	T	C	100	*	TYPE=SUBSTITUTE
CM032587.1	1655917	.	A	G	100	*	TYPE=SUBSTITUTE
CM032587.1	1655920	.	A	C	100	*	TYPE=SUBSTITUTE
CM032587.1	1656046	.	A	G	100	*	TYPE=SUBSTITUTE
CM032587.1	1656162	.	T	C	100	*	TYPE=SUBSTITUTE
CM032587.1	1656412	.	T	C	100	*	TYPE=SUBSTITUTE
CM032587.1	1656470	.	G	A	100	*	TYPE=SUBSTITUTE
CM032587.1	1656523	.	C	T	100	*	TYPE=SUBSTITUTE
CM032587.1	1656655	.	C	T	100	*	TYPE=SUBSTITUTE

CM032587.1	1656727	.	A	G	100	*	TYPE=SUBSTITUTE
CM032587.1	1656733	.	G	A	100	*	TYPE=SUBSTITUTE
CM032587.1	1656778	.	T	C	100	*	TYPE=SUBSTITUTE
CM032587.1	1656797	.	G	A	100	*	TYPE=SUBSTITUTE
CM032587.1	1656820	.	CT	C	100	*	TYPE=DELETE
CM032587.1	1656821	.	G	A	100	*	TYPE=SUBSTITUTE
CM032587.1	1656924	.	G	A	100	*	TYPE=SUBSTITUTE
CM032587.1	1656988	.	GC	G	100	*	TYPE=DELETE
CM032587.1	1656991	.	A	C	100	*	TYPE=SUBSTITUTE
CM032587.1	1657041	.	T	C	100	*	TYPE=SUBSTITUTE
CM032587.1	1657126	.	G	A	100	*	TYPE=SUBSTITUTE
CM032587.1	1657147	.	G	A	100	*	TYPE=SUBSTITUTE
CM032587.1	1657154	.	G	A	100	*	TYPE=SUBSTITUTE
CM032587.1	1657185	.	T	G	100	*	TYPE=SUBSTITUTE
CM032587.1	1657192	.	T	A	100	*	TYPE=SUBSTITUTE
CM032587.1	1657220	.	T	C	100	*	TYPE=SUBSTITUTE
CM032587.1	1657225	.	G	A	100	*	TYPE=SUBSTITUTE
CM032587.1	1657247	.	C	CT	100	*	TYPE=INSERT
CM032587.1	1657255	.	T	C	100	*	TYPE=SUBSTITUTE
CM032587.1	1657281	.	T	TG	100	*	TYPE=INSERT
CM032587.1	1657288	.	G	A	100	*	TYPE=SUBSTITUTE
CM032587.1	1657291	.	CCA	C	100	*	TYPE=DELETE
CM032587.1	1657331	.	A	C	100	*	TYPE=SUBSTITUTE
CM032587.1	1657339	.	C	A	100	*	TYPE=SUBSTITUTE
CM032587.1	1657343	.	TC	T	100	*	TYPE=DELETE
CM032587.1	1657346	.	A	T	100	*	TYPE=SUBSTITUTE
CM032587.1	1657349	.	A	G	100	*	TYPE=SUBSTITUTE
CM032587.1	1657391	.	T	C	100	*	TYPE=SUBSTITUTE
CM032587.1	1657418	.	G	T	100	*	TYPE=SUBSTITUTE
CM032587.1	1657426	.	C	A	100	*	TYPE=SUBSTITUTE
CM032587.1	1657431	.	G	T	100	*	TYPE=SUBSTITUTE
CM032587.1	1657435	.	T	C	100	*	TYPE=SUBSTITUTE
CM032587.1	1657443	.	G	A	100	*	TYPE=SUBSTITUTE
CM032587.1	1657465	.	G	A	100	*	TYPE=SUBSTITUTE
CM032587.1	1657466	.	G	A	100	*	TYPE=SUBSTITUTE
CM032587.1	1657470	.	A	G	100	*	TYPE=SUBSTITUTE
CM032587.1	1657508	.	C	a	100	*	TYPE=SUBSTITUTE
CM032587.1	1657510	.	C	t	100	*	TYPE=SUBSTITUTE
CM032587.1	1657516	.	CT	C	100	*	TYPE=DELETE
CM032587.1	1657517	.	C	t	100	*	TYPE=SUBSTITUTE
CM032587.1	1657525	.	T	c	100	*	TYPE=SUBSTITUTE
CM032587.1	1657526	.	C	t	100	*	TYPE=SUBSTITUTE
CM032587.1	1657548	.	A	T	100	*	TYPE=SUBSTITUTE
CM032587.1	1657567	.	CC	C	100	*	TYPE=DELETE
CM032587.1	1657664	.	C	CTAAGG	100	*	TYPE=INSERT
CM032587.1	1657698	.	C	T	100	*	TYPE=SUBSTITUTE
CM032587.1	1657708	.	A	C	100	*	TYPE=SUBSTITUTE
CM032587.1	1657873	.	C	T	100	*	TYPE=SUBSTITUTE
CM032587.1	1657890	.	G	A	100	*	TYPE=SUBSTITUTE
CM032587.1	1657919	.	A	g	100	*	TYPE=SUBSTITUTE
CM032587.1	1657923	.	G	c	100	*	TYPE=SUBSTITUTE
CM032587.1	1657927	.	G	c	100	*	TYPE=SUBSTITUTE
CM032587.1	1657945	.	C	A	100	*	TYPE=SUBSTITUTE
CM032587.1	1657948	.	GG	G	100	*	TYPE=DELETE
CM032587.1	1657986	.	C	T	100	*	TYPE=SUBSTITUTE
CM032587.1	1658041	.	G	a	100	*	TYPE=SUBSTITUTE
CM032587.1	1658054	.	C	t	100	*	TYPE=SUBSTITUTE
CM032587.1	1658055	.	G	a	100	*	TYPE=SUBSTITUTE
CM032587.1	1658092	.	A	C	100	*	TYPE=SUBSTITUTE
CM032587.1	1658109	.	T	A	100	*	TYPE=SUBSTITUTE
CM032587.1	1658131	.	T	A	100	*	TYPE=SUBSTITUTE
CM032587.1	1658164	.	A	G	100	*	TYPE=SUBSTITUTE
CM032587.1	1658184	.	A	G	100	*	TYPE=SUBSTITUTE
CM032587.1	1658212	.	T	C	100	*	TYPE=SUBSTITUTE

CM032587.1	1658264	.	T	C	100	*	TYPE=SUBSTITUTE
CM032587.1	1658311	.	T	C	100	*	TYPE=SUBSTITUTE
CM032587.1	1658319	.	A	G	100	*	TYPE=SUBSTITUTE
CM032587.1	1658354	.	G	C	100	*	TYPE=SUBSTITUTE
CM032587.1	1658364	.	T	TA	100	*	TYPE=INSERT
CM032587.1	1658366	.	T	A	100	*	TYPE=SUBSTITUTE
CM032587.1	1658409	.	TA	T	100	*	TYPE=DELETE
CM032587.1	1658422	.	G	A	100	*	TYPE=SUBSTITUTE
CM032587.1	1658491	.	G	A	100	*	TYPE=SUBSTITUTE
CM032587.1	1658527	.	A	G	100	*	TYPE=SUBSTITUTE
CM032587.1	1658540	.	T	C	100	*	TYPE=SUBSTITUTE
CM032587.1	1658545	.	T	C	100	*	TYPE=SUBSTITUTE
CM032587.1	1658578	.	T	C	100	*	TYPE=SUBSTITUTE
CM032587.1	1658686	.	C	T	100	*	TYPE=SUBSTITUTE
CM032587.1	1658713	.	A	G	100	*	TYPE=SUBSTITUTE
CM032587.1	1658732	.	C	T	100	*	TYPE=SUBSTITUTE
CM032587.1	1658740	.	A	G	100	*	TYPE=SUBSTITUTE
CM032587.1	1658751	.	C	A	100	*	TYPE=SUBSTITUTE
CM032587.1	1658758	.	T	G	100	*	TYPE=SUBSTITUTE
CM032587.1	1658779	.	TC	T	100	*	TYPE=DELETE
CM032587.1	1658894	.	A	G	100	*	TYPE=SUBSTITUTE
CM032587.1	1658906	.	C	T	100	*	TYPE=SUBSTITUTE
CM032587.1	1658925	.	G	A	100	*	TYPE=SUBSTITUTE
CM032587.1	1658927	.	C	A	100	*	TYPE=SUBSTITUTE
CM032587.1	1658936	.	G	A	100	*	TYPE=SUBSTITUTE
CM032587.1	1658988	.	C	T	100	*	TYPE=SUBSTITUTE
CM032587.1	1658996	.	T	C	100	*	TYPE=SUBSTITUTE
CM032587.1	1659094	.	G	A	100	*	TYPE=SUBSTITUTE
CM032587.1	1659198	.	C	A	100	*	TYPE=SUBSTITUTE
CM032587.1	1659221	.	C	T	100	*	TYPE=SUBSTITUTE
CM032587.1	1659242	.	G	A	100	*	TYPE=SUBSTITUTE
CM032587.1	1659249	.	C	T	100	*	TYPE=SUBSTITUTE
CM032587.1	1659269	.	A	G	100	*	TYPE=SUBSTITUTE
CM032587.1	1659272	.	A	G	100	*	TYPE=SUBSTITUTE
CM032587.1	1659287	.	A	G	100	*	TYPE=SUBSTITUTE
CM032587.1	1659314	.	G	A	100	*	TYPE=SUBSTITUTE
CM032587.1	1659326	.	G	A	100	*	TYPE=SUBSTITUTE
CM032587.1	1659329	.	C	T	100	*	TYPE=SUBSTITUTE
CM032587.1	1659331	.	T	A	100	*	TYPE=SUBSTITUTE
CM032587.1	1659346	.	A	G	100	*	TYPE=SUBSTITUTE
CM032587.1	1659362	.	G	T	100	*	TYPE=SUBSTITUTE
CM032587.1	1659363	.	T	C	100	*	TYPE=SUBSTITUTE
CM032587.1	1659365	.	C	T	100	*	TYPE=SUBSTITUTE
CM032587.1	1659371	.	C	T	100	*	TYPE=SUBSTITUTE
CM032587.1	1659380	.	C	T	100	*	TYPE=SUBSTITUTE
CM032587.1	1659392	.	G	A	100	*	TYPE=SUBSTITUTE
CM032587.1	1659418	.	T	C	100	*	TYPE=SUBSTITUTE
CM032587.1	1659488	.	A	G	100	*	TYPE=SUBSTITUTE
CM032587.1	1659512	.	C	T	100	*	TYPE=SUBSTITUTE
CM032587.1	1659536	.	A	G	100	*	TYPE=SUBSTITUTE
CM032587.1	1659569	.	G	A	100	*	TYPE=SUBSTITUTE
CM032587.1	1659572	.	A	G	100	*	TYPE=SUBSTITUTE
CM032587.1	1659581	.	A	G	100	*	TYPE=SUBSTITUTE
CM032587.1	1659593	.	T	C	100	*	TYPE=SUBSTITUTE
CM032587.1	1659605	.	G	A	100	*	TYPE=SUBSTITUTE
CM032587.1	1659620	.	T	C	100	*	TYPE=SUBSTITUTE
CM032587.1	1659635	.	T	G	100	*	TYPE=SUBSTITUTE
CM032587.1	1659644	.	c	ctcaccc	100	*	TYPE=INSERT
CM032587.1	1659668	.	G	C	100	*	TYPE=SUBSTITUTE
CM032587.1	1659680	.	T	G	100	*	TYPE=SUBSTITUTE
CM032587.1	1659689	.	A	g	100	*	TYPE=SUBSTITUTE
CM032587.1	1659698	.	G	a	100	*	TYPE=SUBSTITUTE
CM032587.1	1659716	.	C	T	100	*	TYPE=SUBSTITUTE
CM032587.1	1659748	.	T	C	100	*	TYPE=SUBSTITUTE

CM032587.1	1659755	.	A	G	100	*	TYPE=SUBSTITUTE
CM032587.1	1659806	.	G	A	100	*	TYPE=SUBSTITUTE
CM032587.1	1659873	.	C	T	100	*	TYPE=SUBSTITUTE
CM032587.1	1659880	.	G	A	100	*	TYPE=SUBSTITUTE
CM032587.1	1659881	.	C	T	100	*	TYPE=SUBSTITUTE
CM032587.1	1659887	.	T	a	100	*	TYPE=SUBSTITUTE
CM032587.1	1659985	.	A	G	100	*	TYPE=SUBSTITUTE
CM032587.1	1660017	.	T	C	100	*	TYPE=SUBSTITUTE
CM032587.1	1660058	.	T	A	100	*	TYPE=SUBSTITUTE
CM032587.1	1660070	.	T	C	100	*	TYPE=SUBSTITUTE
CM032587.1	1660130	.	T	C	100	*	TYPE=SUBSTITUTE
CM032587.1	1660181	.	T	G	100	*	TYPE=SUBSTITUTE
CM032587.1	1660196	.	G	A	100	*	TYPE=SUBSTITUTE
CM032587.1	1660199	.	T	C	100	*	TYPE=SUBSTITUTE
CM032587.1	1660205	.	C	T	100	*	TYPE=SUBSTITUTE
CM032587.1	1660208	.	A	G	100	*	TYPE=SUBSTITUTE
CM032587.1	1660220	.	A	G	100	*	TYPE=SUBSTITUTE
CM032587.1	1660232	.	A	G	100	*	TYPE=SUBSTITUTE
CM032587.1	1660246	.	C	T	100	*	TYPE=SUBSTITUTE
CM032587.1	1660255	.	C	T	100	*	TYPE=SUBSTITUTE
CM032587.1	1660263	.	G	GC	100	*	TYPE=INSERT
CM032587.1	1660269	.	G	A	100	*	TYPE=SUBSTITUTE
CM032587.1	1660283	.	C	T	100	*	TYPE=SUBSTITUTE
CM032587.1	1660287	.	C	T	100	*	TYPE=SUBSTITUTE
CM032587.1	1660303	.	A	G	100	*	TYPE=SUBSTITUTE
CM032587.1	1660318	.	C	T	100	*	TYPE=SUBSTITUTE
CM032587.1	1660321	.	C	T	100	*	TYPE=SUBSTITUTE
CM032587.1	1660342	.	A	T	100	*	TYPE=SUBSTITUTE
CM032587.1	1660345	.	C	T	100	*	TYPE=SUBSTITUTE
CM032587.1	1660347	.	T	G	100	*	TYPE=SUBSTITUTE
CM032587.1	1660348	.	A	G	100	*	TYPE=SUBSTITUTE
CM032587.1	1660369	.	T	C	100	*	TYPE=SUBSTITUTE
CM032587.1	1660411	.	G	A	100	*	TYPE=SUBSTITUTE
CM032587.1	1660435	.	A	G	100	*	TYPE=SUBSTITUTE
CM032587.1	1660471	.	T	A	100	*	TYPE=SUBSTITUTE
CM032587.1	1660486	.	A	G	100	*	TYPE=SUBSTITUTE
CM032587.1	1660492	.	C	T	100	*	TYPE=SUBSTITUTE
CM032587.1	1660510	.	T	C	100	*	TYPE=SUBSTITUTE
CM032587.1	1660567	.	T	A	100	*	TYPE=SUBSTITUTE
CM032587.1	1660573	.	T	C	100	*	TYPE=SUBSTITUTE
CM032587.1	1660630	.	T	C	100	*	TYPE=SUBSTITUTE
CM032587.1	1660675	.	T	c	100	*	TYPE=SUBSTITUTE
CM032587.1	1660678	.	G	A	100	*	TYPE=SUBSTITUTE
CM032587.1	1660735	.	A	G	100	*	TYPE=SUBSTITUTE
CM032587.1	1660744	.	C	T	100	*	TYPE=SUBSTITUTE
CM032587.1	1660760	.	A	G	100	*	TYPE=SUBSTITUTE
CM032587.1	1660807	.	C	T	100	*	TYPE=SUBSTITUTE
CM032587.1	1660834	.	C	T	100	*	TYPE=SUBSTITUTE
CM032587.1	1660855	.	T	C	100	*	TYPE=SUBSTITUTE
CM032587.1	1660888	.	G	A	100	*	TYPE=SUBSTITUTE
CM032587.1	1660904	.	G	A	100	*	TYPE=SUBSTITUTE
CM032587.1	1660906	.	T	C	100	*	TYPE=SUBSTITUTE
CM032587.1	1660972	.	C	t	100	*	TYPE=SUBSTITUTE
CM032587.1	1661014	.	C	T	100	*	TYPE=SUBSTITUTE
CM032587.1	1661030	.	A	G	100	*	TYPE=SUBSTITUTE
CM032587.1	1661033	.	C	T	100	*	TYPE=SUBSTITUTE
CM032587.1	1661039	.	TAA	T	100	*	TYPE=DELETE
CM032587.1	1661042	.	C	T	100	*	TYPE=SUBSTITUTE
CM032587.1	1661058	.	T	A	100	*	TYPE=SUBSTITUTE
CM032587.1	1661070	.	T	TC	100	*	TYPE=INSERT
CM032587.1	1661074	.	C	G	100	*	TYPE=SUBSTITUTE
CM032587.1	1661075	.	G	A	100	*	TYPE=SUBSTITUTE
CM032587.1	1661089	.	C	G	100	*	TYPE=SUBSTITUTE
CM032587.1	1661090	.	A	G	100	*	TYPE=SUBSTITUTE

CM032587.1	1661091	.	G	A	100	*	TYPE=SUBSTITUTE
CM032587.1	1661092	.	A	G	100	*	TYPE=SUBSTITUTE
CM032587.1	1661094	.	C	T	100	*	TYPE=SUBSTITUTE
CM032587.1	1661103	.	C	G	100	*	TYPE=SUBSTITUTE
CM032587.1	1661104	.	C	T	100	*	TYPE=SUBSTITUTE
CM032587.1	1661110	.	G	GAATCCA	100	*	TYPE=INSERT
CM032587.1	1661114	.	C	G	100	*	TYPE=SUBSTITUTE
CM032587.1	1661115	.	C	G	100	*	TYPE=SUBSTITUTE
CM032587.1	1661116	.	G	T	100	*	TYPE=SUBSTITUTE
CM032587.1	1661117	.	C	CA	100	*	TYPE=INSERT
CM032587.1	1661118	.	A	C	100	*	TYPE=SUBSTITUTE
CM032587.1	1661119	.	A	G	100	*	TYPE=SUBSTITUTE
CM032587.1	1661124	.	A	G	100	*	TYPE=SUBSTITUTE
CM032587.1	1661133	.	A	G	100	*	TYPE=SUBSTITUTE
CM032587.1	1661138	.	C	G	100	*	TYPE=SUBSTITUTE
CM032587.1	1661146	.	ACGATTAT	A	100	*	TYPE=DELETE
CM032587.1	1661148	.	C	G	100	*	TYPE=SUBSTITUTE
CM032587.1	1661149	.	A	G	100	*	TYPE=SUBSTITUTE
CM032587.1	1661155	.	AG	A	100	*	TYPE=DELETE
CM032587.1	1661157	.	T	C	100	*	TYPE=SUBSTITUTE
CM032587.1	1661160	.	G	C	100	*	TYPE=SUBSTITUTE
CM032587.1	1661192	.	A	G	100	*	TYPE=SUBSTITUTE
CM032587.1	1661266	.	G	A	100	*	TYPE=SUBSTITUTE
CM032587.1	1661329	.	CA	C	100	*	TYPE=DELETE
CM032587.1	1661340	.	G	C	100	*	TYPE=SUBSTITUTE
CM032587.1	1661347	.	T	C	100	*	TYPE=SUBSTITUTE
CM032587.1	1661384	.	ATAGTAAAGTATG	A	100	*	TYPE=DELETE
CM032587.1	1661387	.	T	C	100	*	TYPE=SUBSTITUTE
CM032587.1	1661390	.	A	G	100	*	TYPE=SUBSTITUTE
CM032587.1	1661417	.	T	G	100	*	TYPE=SUBSTITUTE
CM032587.1	1661422	.	G	A	100	*	TYPE=SUBSTITUTE
CM032587.1	1661428	.	T	A	100	*	TYPE=SUBSTITUTE
CM032587.1	1661432	.	G	A	100	*	TYPE=SUBSTITUTE
CM032587.1	1661443	.	A	G	100	*	TYPE=SUBSTITUTE
CM032587.1	1661448	.	A	C	100	*	TYPE=SUBSTITUTE
CM032587.1	1661466	.	G	t	100	*	TYPE=SUBSTITUTE
CM032587.1	1661470	.	A	g	100	*	TYPE=SUBSTITUTE
CM032587.1	1661487	.	G	GT	100	*	TYPE=INSERT
CM032587.1	1661488	.	A	G	100	*	TYPE=SUBSTITUTE
CM032587.1	1661489	.	C	T	100	*	TYPE=SUBSTITUTE
CM032587.1	1661490	.	G	A	100	*	TYPE=SUBSTITUTE
CM032587.1	1661509	.	A	g	100	*	TYPE=SUBSTITUTE
CM032587.1	1661510	.	G	a	100	*	TYPE=SUBSTITUTE
CM032587.1	1661517	.	G	a	100	*	TYPE=SUBSTITUTE
CM032587.1	1661542	.	A	g	100	*	TYPE=SUBSTITUTE
CM032587.1	1661545	.	G	a	100	*	TYPE=SUBSTITUTE
CM032587.1	1661553	.	g	gaaa	100	*	TYPE=INSERT
CM032587.1	1661564	.	A	G	100	*	TYPE=SUBSTITUTE
CM032587.1	1661568	.	A	ACCGGTTG	100	*	TYPE=INSERT
CM032587.1	1661570	.	G	A	100	*	TYPE=SUBSTITUTE
CM032587.1	1661574	.	A	T	100	*	TYPE=SUBSTITUTE
CM032587.1	1661575	.	G	A	100	*	TYPE=SUBSTITUTE
CM032587.1	1661659	.	A	G	100	*	TYPE=SUBSTITUTE
CM032587.1	1661677	.	G	t	100	*	TYPE=SUBSTITUTE
CM032587.1	1661679	.	CA	C	100	*	TYPE=DELETE
CM032587.1	1661690	.	TT	T	100	*	TYPE=DELETE
CM032587.1	1661832	.	A	G	100	*	TYPE=SUBSTITUTE
CM032587.1	1661838	.	A	T	100	*	TYPE=SUBSTITUTE
CM032587.1	1661839	.	A	G	100	*	TYPE=SUBSTITUTE
CM032587.1	1661840	.	A	T	100	*	TYPE=SUBSTITUTE
CM032587.1	1661844	.	GA	G	100	*	TYPE=DELETE
CM032587.1	1661849	.	A	G	100	*	TYPE=SUBSTITUTE
CM032587.1	1661856	.	A	G	100	*	TYPE=SUBSTITUTE
CM032587.1	1661887	.	G	GTAC	100	*	TYPE=INSERT

CM032587.1	1661891	.	A	G	100	*	TYPE=SUBSTITUTE
CM032587.1	1661899	.	T	A	100	*	TYPE=SUBSTITUTE
CM032587.1	1661900	.	T	C	100	*	TYPE=SUBSTITUTE
CM032587.1	1661912	.	GG	G	100	*	TYPE=DELETE
CM032587.1	1661932	.	CTAAGG	C	100	*	TYPE=DELETE
CM032587.1	1661933	.	C	T	100	*	TYPE=SUBSTITUTE
CM032587.1	1661959	.	C	CG	100	*	TYPE=INSERT
CM032587.1	1661961	.	C	T	100	*	TYPE=SUBSTITUTE
CM032587.1	1661990	.	T	G	100	*	TYPE=SUBSTITUTE
CM032587.1	1662011	.	A	T	100	*	TYPE=SUBSTITUTE
CM032587.1	1662014	.	G	C	100	*	TYPE=SUBSTITUTE
CM032587.1	1662019	.	T	C	100	*	TYPE=SUBSTITUTE
CM032587.1	1662027	.	G	A	100	*	TYPE=SUBSTITUTE
CM032587.1	1662031	.	TG	T	100	*	TYPE=DELETE
CM032587.1	1662035	.	C	G	100	*	TYPE=SUBSTITUTE
CM032587.1	1662045	.	A	C	100	*	TYPE=SUBSTITUTE
CM032587.1	1662067	.	G	c	100	*	TYPE=SUBSTITUTE
CM032587.1	1662069	.	A	t	100	*	TYPE=SUBSTITUTE
CM032587.1	1662081	.	C	t	100	*	TYPE=SUBSTITUTE
CM032587.1	1662093	.	A	G	100	*	TYPE=SUBSTITUTE
CM032587.1	1662139	.	T	tt	100	*	TYPE=INSERT
CM032587.1	1662214	.	AT	A	100	*	TYPE=DELETE
CM032587.1	1662221	.	A	ACC	100	*	TYPE=INSERT
CM032587.1	1662232	.	G	A	100	*	TYPE=SUBSTITUTE
CM032587.1	1662236	.	GGA	G	100	*	TYPE=DELETE
CM032587.1	1662285	.	A	AA	100	*	TYPE=INSERT
CM032587.1	1662306	.	G	a	100	*	TYPE=SUBSTITUTE
CM032587.1	1662309	.	C	t	100	*	TYPE=SUBSTITUTE
CM032587.1	1662313	.	GGC	G	100	*	TYPE=DELETE
CM032587.1	1662323	.	C	T	100	*	TYPE=SUBSTITUTE
CM032587.1	1662356	.	T	a	100	*	TYPE=SUBSTITUTE
CM032587.1	1662363	.	T	a	100	*	TYPE=SUBSTITUTE
CM032587.1	1662378	.	GTTTTTTTTTTTTTT	G	100	*	TYPE=DELETE
CM032587.1	1662391	.	a	aa	100	*	TYPE=INSERT
CM032587.1	1662417	.	T	TT	100	*	TYPE=INSERT
CM032587.1	1662476	.	C	A	100	*	TYPE=SUBSTITUTE
CM032587.1	1662484	.	C	T	100	*	TYPE=SUBSTITUTE
CM032587.1	1662485	.	T	TG	100	*	TYPE=INSERT
CM032587.1	1662506	.	c	ca	100	*	TYPE=INSERT
CM032587.1	1662513	.	a	aa	100	*	TYPE=INSERT
CM032587.1	1662530	.	T	C	100	*	TYPE=SUBSTITUTE
CM032587.1	1662547	.	GG	G	100	*	TYPE=DELETE
CM032587.1	1662585	.	G	GTCGCA	100	*	TYPE=INSERT
CM032587.1	1662800	.	A	G	100	*	TYPE=SUBSTITUTE
CM032587.1	1662889	.	T	tt	100	*	TYPE=INSERT
CM032587.1	1663068	.	A	G	100	*	TYPE=SUBSTITUTE
CM032587.1	1663175	.	T	A	100	*	TYPE=SUBSTITUTE
CM032587.1	1663187	.	A	G	100	*	TYPE=SUBSTITUTE
CM032587.1	1663197	.	T	A	100	*	TYPE=SUBSTITUTE
CM032587.1	1663201	.	G	A	100	*	TYPE=SUBSTITUTE
CM032587.1	1663242	.	A	G	100	*	TYPE=SUBSTITUTE
CM032587.1	1663282	.	G	C	100	*	TYPE=SUBSTITUTE
CM032587.1	1663352	.	G	T	100	*	TYPE=SUBSTITUTE
CM032587.1	1663353	.	T	G	100	*	TYPE=SUBSTITUTE
CM032587.1	1663391	.	T	C	100	*	TYPE=SUBSTITUTE
CM032587.1	1663531	.	TCC	T	100	*	TYPE=DELETE
CM032587.1	1663537	.	G	A	100	*	TYPE=SUBSTITUTE
CM032587.1	1663565	.	C	G	100	*	TYPE=SUBSTITUTE
CM032587.1	1663592	.	G	GCG	100	*	TYPE=INSERT
CM032587.1	1663692	.	G	A	100	*	TYPE=SUBSTITUTE
CM032587.1	1663697	.	A	C	100	*	TYPE=SUBSTITUTE
CM032587.1	1663719	.	T	g	100	*	TYPE=SUBSTITUTE
CM032587.1	1663728	.	C	T	100	*	TYPE=SUBSTITUTE
CM032587.1	1663731	.	C	A	100	*	TYPE=SUBSTITUTE

CM032587.1	1663735	.	GGTG	G	100	*	TYPE=DELETE
CM032587.1	1663776	.	T	G	100	*	TYPE=SUBSTITUTE
CM032587.1	1663790	.	T	C	100	*	TYPE=SUBSTITUTE
CM032587.1	1663795	.	A	C	100	*	TYPE=SUBSTITUTE
CM032587.1	1663874	.	A	G	100	*	TYPE=SUBSTITUTE
CM032587.1	1663886	.	G	A	100	*	TYPE=SUBSTITUTE
CM032587.1	1663943	.	TCG	T	100	*	TYPE=DELETE
CM032587.1	1663950	.	A	G	100	*	TYPE=SUBSTITUTE
CM032587.1	1663960	.	T	C	100	*	TYPE=SUBSTITUTE
CM032587.1	1663961	.	A	T	100	*	TYPE=SUBSTITUTE
CM032587.1	1664048	.	A	C	100	*	TYPE=SUBSTITUTE
CM032587.1	1664117	.	A	G	100	*	TYPE=SUBSTITUTE
CM032587.1	1664149	.	T	C	100	*	TYPE=SUBSTITUTE
CM032587.1	1664310	.	A	G	100	*	TYPE=SUBSTITUTE
CM032587.1	1664355	.	T	A	100	*	TYPE=SUBSTITUTE
CM032587.1	1664379	.	G	A	100	*	TYPE=SUBSTITUTE
CM032587.1	1664380	.	T	G	100	*	TYPE=SUBSTITUTE
CM032587.1	1664381	.	A	C	100	*	TYPE=SUBSTITUTE
CM032587.1	1664404	.	C	A	100	*	TYPE=SUBSTITUTE
CM032587.1	1664407	.	T	G	100	*	TYPE=SUBSTITUTE
CM032587.1	1664434	.	T	C	100	*	TYPE=SUBSTITUTE
CM032587.1	1664440	.	T	C	100	*	TYPE=SUBSTITUTE
CM032587.1	1664455	.	GGTGTAA	G	100	*	TYPE=DELETE
CM032587.1	1664462	.	CTCTG	C	100	*	TYPE=DELETE
CM032587.1	1664473	.	G	c	100	*	TYPE=SUBSTITUTE
CM032587.1	1664555	.	T	C	100	*	TYPE=SUBSTITUTE
CM032587.1	1664581	.	T	C	100	*	TYPE=SUBSTITUTE
CM032587.1	1664625	.	A	G	100	*	TYPE=SUBSTITUTE
CM032587.1	1664639	.	G	T	100	*	TYPE=SUBSTITUTE
CM032587.1	1664641	.	C	T	100	*	TYPE=SUBSTITUTE
CM032587.1	1664661	.	A	T	100	*	TYPE=SUBSTITUTE
CM032587.1	1664677	.	A	g	100	*	TYPE=SUBSTITUTE
CM032587.1	1664678	.	a	agaaag	100	*	TYPE=INSERT
CM032587.1	1664699	.	A	G	100	*	TYPE=SUBSTITUTE
CM032587.1	1664704	.	T	C	100	*	TYPE=SUBSTITUTE
CM032587.1	1664708	.	C	T	100	*	TYPE=SUBSTITUTE
CM032587.1	1664715	.	A	T	100	*	TYPE=SUBSTITUTE
CM032587.1	1664721	.	G	A	100	*	TYPE=SUBSTITUTE
CM032587.1	1664729	.	C	G	100	*	TYPE=SUBSTITUTE
CM032587.1	1664735	.	T	C	100	*	TYPE=SUBSTITUTE
CM032587.1	1664772	.	C	T	100	*	TYPE=SUBSTITUTE
CM032587.1	1664815	.	C	T	100	*	TYPE=SUBSTITUTE
CM032587.1	1664836	.	CC	C	100	*	TYPE=DELETE
CM032587.1	1664881	.	C	CA	100	*	TYPE=INSERT
CM032587.1	1664889	.	T	G	100	*	TYPE=SUBSTITUTE
CM032587.1	1664890	.	A	AGCAAT	100	*	TYPE=INSERT
CM032587.1	1664894	.	A	C	100	*	TYPE=SUBSTITUTE
CM032587.1	1664995	.	C	T	100	*	TYPE=SUBSTITUTE
CM032587.1	1665005	.	T	A	100	*	TYPE=SUBSTITUTE
CM032587.1	1665040	.	A	G	100	*	TYPE=SUBSTITUTE
CM032587.1	1665146	.	T	C	100	*	TYPE=SUBSTITUTE
CM032587.1	1665184	.	C	G	100	*	TYPE=SUBSTITUTE
CM032587.1	1665386	.	A	G	100	*	TYPE=SUBSTITUTE
CM032587.1	1665389	.	T	C	100	*	TYPE=SUBSTITUTE
CM032587.1	1665390	.	G	A	100	*	TYPE=SUBSTITUTE
CM032587.1	1665402	.	C	T	100	*	TYPE=SUBSTITUTE
CM032587.1	1665411	.	G	A	100	*	TYPE=SUBSTITUTE
CM032587.1	1665574	.	G	C	100	*	TYPE=SUBSTITUTE
CM032587.1	1665604	.	G	A	100	*	TYPE=SUBSTITUTE
CM032587.1	1665612	.	GGAGGGGA	G	100	*	TYPE=DELETE
CM032587.1	1665635	.	G	T	100	*	TYPE=SUBSTITUTE
CM032587.1	1665651	.	G	A	100	*	TYPE=SUBSTITUTE
CM032587.1	1665739	.	A	G	100	*	TYPE=SUBSTITUTE
CM032587.1	1665910	.	T	A	100	*	TYPE=SUBSTITUTE

CM032587.1	1665991	.	G	A	100	*	TYPE=SUBSTITUTE
CM032587.1	1666078	.	CGGTGC	C	100	*	TYPE=DELETE
CM032587.1	1666098	.	T	C	100	*	TYPE=SUBSTITUTE
CM032587.1	1666106	.	A	G	100	*	TYPE=SUBSTITUTE
CM032587.1	1666166	.	C	T	100	*	TYPE=SUBSTITUTE
CM032587.1	1666177	.	A	G	100	*	TYPE=SUBSTITUTE
CM032587.1	1666184	.	C	T	100	*	TYPE=SUBSTITUTE
CM032587.1	1666218	.	C	t	100	*	TYPE=SUBSTITUTE
CM032587.1	1666226	.	C	t	100	*	TYPE=SUBSTITUTE
CM032587.1	1666269	.	G	A	100	*	TYPE=SUBSTITUTE
CM032587.1	1666270	.	G	GA	100	*	TYPE=INSERT
CM032587.1	1666341	.	G	C	100	*	TYPE=SUBSTITUTE
CM032587.1	1666342	.	G	A	100	*	TYPE=SUBSTITUTE
CM032587.1	1666354	.	C	CTGCC	100	*	TYPE=INSERT
CM032587.1	1666385	.	T	C	100	*	TYPE=SUBSTITUTE
CM032587.1	1666397	.	A	T	100	*	TYPE=SUBSTITUTE
CM032587.1	1666403	.	ATACC	A	100	*	TYPE=DELETE
CM032587.1	1666406	.	A	G	100	*	TYPE=SUBSTITUTE
CM032587.1	1666407	.	A	G	100	*	TYPE=SUBSTITUTE
CM032587.1	1666408	.	G	A	100	*	TYPE=SUBSTITUTE
CM032587.1	1666438	.	G	A	100	*	TYPE=SUBSTITUTE
CM032587.1	1666530	.	T	C	100	*	TYPE=SUBSTITUTE
CM032587.1	1666531	.	T	C	100	*	TYPE=SUBSTITUTE
CM032587.1	1666546	.	A	G	100	*	TYPE=SUBSTITUTE
CM032587.1	1666547	.	A	G	100	*	TYPE=SUBSTITUTE
CM032587.1	1666555	.	T	G	100	*	TYPE=SUBSTITUTE
CM032587.1	1666578	.	T	C	100	*	TYPE=SUBSTITUTE
CM032587.1	1666624	.	G	A	100	*	TYPE=SUBSTITUTE
CM032587.1	1666636	.	G	A	100	*	TYPE=SUBSTITUTE
CM032587.1	1666646	.	A	G	100	*	TYPE=SUBSTITUTE
CM032587.1	1666667	.	A	G	100	*	TYPE=SUBSTITUTE
CM032587.1	1666682	.	T	G	100	*	TYPE=SUBSTITUTE
CM032587.1	1666749	.	GG	G	100	*	TYPE=DELETE
CM032587.1	1666849	.	A	G	100	*	TYPE=SUBSTITUTE
CM032587.1	1666860	.	T	G	100	*	TYPE=SUBSTITUTE
CM032587.1	1666863	.	C	T	100	*	TYPE=SUBSTITUTE
CM032587.1	1666874	.	T	C	100	*	TYPE=SUBSTITUTE
CM032587.1	1666880	.	C	T	100	*	TYPE=SUBSTITUTE
CM032587.1	1667003	.	C	G	100	*	TYPE=SUBSTITUTE
CM032587.1	1667016	.	G	C	100	*	TYPE=SUBSTITUTE
CM032587.1	1667045	.	A	G	100	*	TYPE=SUBSTITUTE
CM032587.1	1667058	.	C	T	100	*	TYPE=SUBSTITUTE
CM032587.1	1667067	.	C	G	100	*	TYPE=SUBSTITUTE
CM032587.1	1667122	.	A	G	100	*	TYPE=SUBSTITUTE
CM032587.1	1667137	.	C	T	100	*	TYPE=SUBSTITUTE
CM032587.1	1667144	.	T	C	100	*	TYPE=SUBSTITUTE
CM032587.1	1667166	.	A	C	100	*	TYPE=SUBSTITUTE
CM032587.1	1667186	.	A	T	100	*	TYPE=SUBSTITUTE
CM032587.1	1667200	.	TT	T	100	*	TYPE=DELETE
CM032587.1	1667222	.	G	GAG	100	*	TYPE=INSERT
CM032587.1	1667237	.	C	t	100	*	TYPE=SUBSTITUTE
CM032587.1	1667274	.	A	g	100	*	TYPE=SUBSTITUTE
CM032587.1	1667285	.	T	G	100	*	TYPE=SUBSTITUTE
CM032587.1	1667310	.	T	C	100	*	TYPE=SUBSTITUTE
CM032587.1	1667313	.	T	C	100	*	TYPE=SUBSTITUTE
CM032587.1	1667322	.	T	C	100	*	TYPE=SUBSTITUTE
CM032587.1	1667338	.	C	CCCTCCATC	100	*	TYPE=INSERT
CM032587.1	1667472	.	A	G	100	*	TYPE=SUBSTITUTE
CM032587.1	1667516	.	C	T	100	*	TYPE=SUBSTITUTE
CM032587.1	1667517	.	T	C	100	*	TYPE=SUBSTITUTE
CM032587.1	1667518	.	G	A	100	*	TYPE=SUBSTITUTE
CM032587.1	1667519	.	C	T	100	*	TYPE=SUBSTITUTE
CM032587.1	1667536	.	CC	C	100	*	TYPE=DELETE
CM032587.1	1667561	.	CC	C	100	*	TYPE=DELETE

CM032587.1	1667613	.	C	T	100	*	TYPE=SUBSTITUTE
CM032587.1	1667636	.	C	T	100	*	TYPE=SUBSTITUTE
CM032587.1	1668021	.	a	at	100	*	TYPE=INSERT
CM032587.1	1668026	.	A	c	100	*	TYPE=SUBSTITUTE
CM032587.1	1668147	.	G	A	100	*	TYPE=SUBSTITUTE
CM032587.1	1668213	.	TC	T	100	*	TYPE=DELETE
CM032587.1	1668272	.	G	C	100	*	TYPE=SUBSTITUTE
CM032587.1	1668339	.	A	G	100	*	TYPE=SUBSTITUTE
CM032587.1	1668342	.	AA	A	100	*	TYPE=DELETE
CM032587.1	1668398	.	GA	G	100	*	TYPE=DELETE
CM032587.1	1668399	.	G	A	100	*	TYPE=SUBSTITUTE
CM032587.1	1668494	.	C	CACC	100	*	TYPE=INSERT
CM032587.1	1668553	.	A	ACA	100	*	TYPE=INSERT
CM032587.1	1668569	.	T	C	100	*	TYPE=SUBSTITUTE
CM032587.1	1668583	.	GG	G	100	*	TYPE=DELETE
CM032587.1	1668692	.	G	GCG	100	*	TYPE=INSERT
CM032587.1	1668805	.	G	A	100	*	TYPE=SUBSTITUTE
CM032587.1	1668842	.	C	T	100	*	TYPE=SUBSTITUTE
CM032587.1	1668858	.	G	C	100	*	TYPE=SUBSTITUTE
CM032587.1	1668963	.	T	C	100	*	TYPE=SUBSTITUTE
CM032587.1	1668993	.	C	G	100	*	TYPE=SUBSTITUTE
CM032587.1	1669031	.	C	A	100	*	TYPE=SUBSTITUTE
CM032587.1	1669032	.	A	T	100	*	TYPE=SUBSTITUTE
CM032587.1	1669038	.	C	T	100	*	TYPE=SUBSTITUTE
CM032587.1	1669039	.	A	G	100	*	TYPE=SUBSTITUTE
CM032587.1	1669067	.	T	C	100	*	TYPE=SUBSTITUTE
CM032587.1	1669088	.	T	C	100	*	TYPE=SUBSTITUTE
CM032587.1	1669123	.	C	A	100	*	TYPE=SUBSTITUTE
CM032587.1	1669164	.	A	G	100	*	TYPE=SUBSTITUTE
CM032587.1	1669204	.	C	T	100	*	TYPE=SUBSTITUTE
CM032587.1	1669223	.	T	C	100	*	TYPE=SUBSTITUTE
CM032587.1	1669248	.	C	T	100	*	TYPE=SUBSTITUTE
CM032587.1	1669252	.	G	T	100	*	TYPE=SUBSTITUTE
CM032587.1	1669270	.	C	T	100	*	TYPE=SUBSTITUTE
CM032587.1	1669291	.	A	T	100	*	TYPE=SUBSTITUTE
CM032587.1	1669308	.	A	G	100	*	TYPE=SUBSTITUTE
CM032587.1	1669311	.	C	G	100	*	TYPE=SUBSTITUTE
CM032587.1	1669326	.	C	T	100	*	TYPE=SUBSTITUTE
CM032587.1	1669329	.	G	A	100	*	TYPE=SUBSTITUTE
CM032587.1	1669343	.	T	G	100	*	TYPE=SUBSTITUTE
CM032587.1	1669345	.	C	T	100	*	TYPE=SUBSTITUTE
CM032587.1	1669358	.	T	C	100	*	TYPE=SUBSTITUTE
CM032587.1	1669373	.	T	C	100	*	TYPE=SUBSTITUTE
CM032587.1	1669410	.	T	TT	100	*	TYPE=INSERT
CM032587.1	1669446	.	C	T	100	*	TYPE=SUBSTITUTE
CM032587.1	1669475	.	G	T	100	*	TYPE=SUBSTITUTE
CM032587.1	1669476	.	C	CATTTCG	100	*	TYPE=INSERT
CM032587.1	1669496	.	T	C	100	*	TYPE=SUBSTITUTE
CM032587.1	1669504	.	C	T	100	*	TYPE=SUBSTITUTE
CM032587.1	1669506	.	C	T	100	*	TYPE=SUBSTITUTE
CM032587.1	1669549	.	G	C	100	*	TYPE=SUBSTITUTE
CM032587.1	1669555	.	ATC	A	100	*	TYPE=DELETE
CM032587.1	1669565	.	C	T	100	*	TYPE=SUBSTITUTE
CM032587.1	1669566	.	C	T	100	*	TYPE=SUBSTITUTE
CM032587.1	1669571	.	C	A	100	*	TYPE=SUBSTITUTE
CM032587.1	1669581	.	C	T	100	*	TYPE=SUBSTITUTE
CM032587.1	1669591	.	G	a	100	*	TYPE=SUBSTITUTE
CM032587.1	1669592	.	G	a	100	*	TYPE=SUBSTITUTE
CM032587.1	1669596	.	G	a	100	*	TYPE=SUBSTITUTE
CM032587.1	1669615	.	G	a	100	*	TYPE=SUBSTITUTE
CM032587.1	1669616	.	C	g	100	*	TYPE=SUBSTITUTE
CM032587.1	1669622	.	GA	G	100	*	TYPE=DELETE
CM032587.1	1669631	.	AAGATT	A	100	*	TYPE=DELETE
CM032587.1	1669635	.	G	a	100	*	TYPE=SUBSTITUTE

CM032587.1	1669658	.	C	T	100	*	TYPE=SUBSTITUTE
CM032587.1	1669674	.	C	T	100	*	TYPE=SUBSTITUTE
CM032587.1	1669686	.	AT	A	100	*	TYPE=DELETE
CM032587.1	1669691	.	A	G	100	*	TYPE=SUBSTITUTE
CM032587.1	1669704	.	C	T	100	*	TYPE=SUBSTITUTE
CM032587.1	1669705	.	G	A	100	*	TYPE=SUBSTITUTE
CM032587.1	1669709	.	C	G	100	*	TYPE=SUBSTITUTE
CM032587.1	1669711	.	A	G	100	*	TYPE=SUBSTITUTE
CM032587.1	1669713	.	G	T	100	*	TYPE=SUBSTITUTE
CM032587.1	1669714	.	A	G	100	*	TYPE=SUBSTITUTE
CM032587.1	1669728	.	A	G	100	*	TYPE=SUBSTITUTE
CM032587.1	1669743	.	C	T	100	*	TYPE=SUBSTITUTE
CM032587.1	1669745	.	C	T	100	*	TYPE=SUBSTITUTE
CM032587.1	1669756	.	C	G	100	*	TYPE=SUBSTITUTE
CM032587.1	1669764	.	T	A	100	*	TYPE=SUBSTITUTE
CM032587.1	1669794	.	G	C	100	*	TYPE=SUBSTITUTE
CM032587.1	1669949	.	A	AA	100	*	TYPE=INSERT
CM032587.1	1670149	.	C	T	100	*	TYPE=SUBSTITUTE
CM032587.1	1670204	.	G	C	100	*	TYPE=SUBSTITUTE
CM032587.1	1670210	.	A	G	100	*	TYPE=SUBSTITUTE
CM032587.1	1670214	.	A	G	100	*	TYPE=SUBSTITUTE
CM032587.1	1670215	.	A	G	100	*	TYPE=SUBSTITUTE
CM032587.1	1670233	.	G	T	100	*	TYPE=SUBSTITUTE
CM032587.1	1670234	.	A	G	100	*	TYPE=SUBSTITUTE
CM032587.1	1670248	.	C	T	100	*	TYPE=SUBSTITUTE
CM032587.1	1670603	.	G	A	100	*	TYPE=SUBSTITUTE
CM032587.1	1670610	.	A	C	100	*	TYPE=SUBSTITUTE
CM032587.1	1670679	.	C	T	100	*	TYPE=SUBSTITUTE
CM032587.1	1670691	.	C	T	100	*	TYPE=SUBSTITUTE
CM032587.1	1670736	.	T	A	100	*	TYPE=SUBSTITUTE
CM032587.1	1670737	.	C	A	100	*	TYPE=SUBSTITUTE
CM032587.1	1670738	.	G	C	100	*	TYPE=SUBSTITUTE
CM032587.1	1670750	.	C	T	100	*	TYPE=SUBSTITUTE
CM032587.1	1670770	.	T	C	100	*	TYPE=SUBSTITUTE
CM032587.1	1670778	.	C	T	100	*	TYPE=SUBSTITUTE
CM032587.1	1670791	.	T	C	100	*	TYPE=SUBSTITUTE
CM032587.1	1670798	.	G	A	100	*	TYPE=SUBSTITUTE
CM032587.1	1670814	.	GA	G	100	*	TYPE=DELETE
CM032587.1	1670826	.	C	G	100	*	TYPE=SUBSTITUTE
CM032587.1	1670836	.	C	T	100	*	TYPE=SUBSTITUTE
CM032587.1	1670837	.	G	A	100	*	TYPE=SUBSTITUTE
CM032587.1	1670845	.	A	C	100	*	TYPE=SUBSTITUTE
CM032587.1	1670858	.	A	T	100	*	TYPE=SUBSTITUTE
CM032587.1	1670863	.	T	C	100	*	TYPE=SUBSTITUTE
CM032587.1	1670868	.	C	T	100	*	TYPE=SUBSTITUTE
CM032587.1	1670879	.	A	T	100	*	TYPE=SUBSTITUTE
CM032587.1	1670882	.	C	G	100	*	TYPE=SUBSTITUTE
CM032587.1	1670883	.	T	G	100	*	TYPE=SUBSTITUTE
CM032587.1	1670884	.	C	T	100	*	TYPE=SUBSTITUTE
CM032587.1	1670885	.	G	A	100	*	TYPE=SUBSTITUTE
CM032587.1	1670886	.	C	A	100	*	TYPE=SUBSTITUTE
CM032587.1	1670892	.	A	G	100	*	TYPE=SUBSTITUTE
CM032587.1	1670894	.	A	T	100	*	TYPE=SUBSTITUTE
CM032587.1	1670895	.	G	C	100	*	TYPE=SUBSTITUTE
CM032587.1	1670909	.	C	T	100	*	TYPE=SUBSTITUTE
CM032587.1	1670935	.	C	CGGGAGCA	100	*	TYPE=INSERT
CM032587.1	1670936	.	A	G	100	*	TYPE=SUBSTITUTE
CM032587.1	1670946	.	A	G	100	*	TYPE=SUBSTITUTE
CM032587.1	1670958	.	TGC	T	100	*	TYPE=DELETE
CM032587.1	1670960	.	C	T	100	*	TYPE=SUBSTITUTE
CM032587.1	1671006	.	C	T	100	*	TYPE=SUBSTITUTE
CM032587.1	1671058	.	T	C	100	*	TYPE=SUBSTITUTE
CM032587.1	1671351	.	G	A	100	*	TYPE=SUBSTITUTE
CM032587.1	1671358	.	C	T	100	*	TYPE=SUBSTITUTE

CM032587.1	1671386	.	A	G	100	*	TYPE=SUBSTITUTE
CM032587.1	1671416	.	C	T	100	*	TYPE=SUBSTITUTE
CM032587.1	1671432	.	C	A	100	*	TYPE=SUBSTITUTE
CM032587.1	1671454	.	G	A	100	*	TYPE=SUBSTITUTE
CM032587.1	1671468	.	A	G	100	*	TYPE=SUBSTITUTE
CM032587.1	1671482	.	G	A	100	*	TYPE=SUBSTITUTE
CM032587.1	1671487	.	C	A	100	*	TYPE=SUBSTITUTE
CM032587.1	1671501	.	G	a	100	*	TYPE=SUBSTITUTE
CM032587.1	1671523	.	a	atC	100	*	TYPE=INSERT
CM032587.1	1671526	.	C	a	100	*	TYPE=SUBSTITUTE
CM032587.1	1671534	.	G	c	100	*	TYPE=SUBSTITUTE
CM032587.1	1671540	.	T	c	100	*	TYPE=SUBSTITUTE
CM032587.1	1671541	.	A	g	100	*	TYPE=SUBSTITUTE
CM032587.1	1671546	.	C	CAa	100	*	TYPE=INSERT
CM032587.1	1671549	.	G	A	100	*	TYPE=SUBSTITUTE
CM032587.1	1671558	.	A	G	100	*	TYPE=SUBSTITUTE
CM032587.1	1671561	.	C	T	100	*	TYPE=SUBSTITUTE
CM032587.1	1671563	.	G	C	100	*	TYPE=SUBSTITUTE
CM032587.1	1671565	.	C	A	100	*	TYPE=SUBSTITUTE
CM032587.1	1671570	.	A	T	100	*	TYPE=SUBSTITUTE
CM032587.1	1671573	.	G	A	100	*	TYPE=SUBSTITUTE
CM032587.1	1671580	.	G	A	100	*	TYPE=SUBSTITUTE
CM032587.1	1671608	.	C	T	100	*	TYPE=SUBSTITUTE
CM032587.1	1671610	.	A	G	100	*	TYPE=SUBSTITUTE
CM032587.1	1671618	.	G	A	100	*	TYPE=SUBSTITUTE
CM032587.1	1671620	.	C	T	100	*	TYPE=SUBSTITUTE
CM032587.1	1671630	.	T	A	100	*	TYPE=SUBSTITUTE
CM032587.1	1671648	.	A	G	100	*	TYPE=SUBSTITUTE
CM032587.1	1671662	.	A	G	100	*	TYPE=SUBSTITUTE
CM032587.1	1671663	.	T	C	100	*	TYPE=SUBSTITUTE
CM032587.1	1671666	.	C	T	100	*	TYPE=SUBSTITUTE
CM032587.1	1671676	.	G	A	100	*	TYPE=SUBSTITUTE
CM032587.1	1671680	.	T	G	100	*	TYPE=SUBSTITUTE
CM032587.1	1671684	.	A	G	100	*	TYPE=SUBSTITUTE
CM032587.1	1671685	.	C	T	100	*	TYPE=SUBSTITUTE
CM032587.1	1671686	.	A	C	100	*	TYPE=SUBSTITUTE
CM032587.1	1671696	.	G	A	100	*	TYPE=SUBSTITUTE
CM032587.1	1671701	.	C	A	100	*	TYPE=SUBSTITUTE
CM032587.1	1671702	.	T	C	100	*	TYPE=SUBSTITUTE
CM032587.1	1671703	.	A	G	100	*	TYPE=SUBSTITUTE
CM032587.1	1671710	.	A	t	100	*	TYPE=SUBSTITUTE
CM032587.1	1671711	.	A	g	100	*	TYPE=SUBSTITUTE
CM032587.1	1671712	.	C	a	100	*	TYPE=SUBSTITUTE
CM032587.1	1671715	.	c	ctacata	100	*	TYPE=INSERT
CM032587.1	1671742	.	G	C	100	*	TYPE=SUBSTITUTE
CM032587.1	1671759	.	A	G	100	*	TYPE=SUBSTITUTE
CM032587.1	1671777	.	G	A	100	*	TYPE=SUBSTITUTE
CM032587.1	1671784	.	G	A	100	*	TYPE=SUBSTITUTE
CM032587.1	1671785	.	A	T	100	*	TYPE=SUBSTITUTE
CM032587.1	1671851	.	G	A	100	*	TYPE=SUBSTITUTE
CM032587.1	1671892	.	C	T	100	*	TYPE=SUBSTITUTE
CM032587.1	1671901	.	G	A	100	*	TYPE=SUBSTITUTE
CM032587.1	1672002	.	A	C	100	*	TYPE=SUBSTITUTE
CM032587.1	1672008	.	C	G	100	*	TYPE=SUBSTITUTE
CM032587.1	1672013	.	A	AT	100	*	TYPE=INSERT
CM032587.1	1672021	.	A	C	100	*	TYPE=SUBSTITUTE
CM032587.1	1672039	.	C	t	100	*	TYPE=SUBSTITUTE
CM032587.1	1672049	.	T	c	100	*	TYPE=SUBSTITUTE
CM032587.1	1672080	.	T	C	100	*	TYPE=SUBSTITUTE
CM032587.1	1672085	.	AA	A	100	*	TYPE=DELETE
CM032587.1	1672115	.	G	A	100	*	TYPE=SUBSTITUTE
CM032587.1	1672158	.	C	t	100	*	TYPE=SUBSTITUTE
CM032587.1	1672185	.	G	A	100	*	TYPE=SUBSTITUTE
CM032587.1	1672224	.	A	G	100	*	TYPE=SUBSTITUTE

CM032587.1	1672239	.	T	C	100	*	TYPE=SUBSTITUTE
CM032587.1	1672244	.	T	c	100	*	TYPE=SUBSTITUTE
CM032587.1	1672252	.	A	g	100	*	TYPE=SUBSTITUTE
CM032587.1	1672265	.	G	A	100	*	TYPE=SUBSTITUTE
CM032587.1	1672267	.	G	C	100	*	TYPE=SUBSTITUTE
CM032587.1	1672278	.	T	A	100	*	TYPE=SUBSTITUTE
CM032587.1	1672280	.	TG	T	100	*	TYPE=DELETE
CM032587.1	1672284	.	T	C	100	*	TYPE=SUBSTITUTE
CM032587.1	1672312	.	C	T	100	*	TYPE=SUBSTITUTE
CM032587.1	1672327	.	ACC	A	100	*	TYPE=DELETE
CM032587.1	1672334	.	G	A	100	*	TYPE=SUBSTITUTE
CM032587.1	1672429	.	A	G	100	*	TYPE=SUBSTITUTE
CM032587.1	1672431	.	A	T	100	*	TYPE=SUBSTITUTE
CM032587.1	1672461	.	TCT	T	100	*	TYPE=DELETE
CM032587.1	1672523	.	G	T	100	*	TYPE=SUBSTITUTE
CM032587.1	1672536	.	A	G	100	*	TYPE=SUBSTITUTE
CM032587.1	1672545	.	T	G	100	*	TYPE=SUBSTITUTE
CM032587.1	1672546	.	G	GCG	100	*	TYPE=INSERT
CM032587.1	1672563	.	G	A	100	*	TYPE=SUBSTITUTE
CM032587.1	1672607	.	T	C	100	*	TYPE=SUBSTITUTE
CM032587.1	1672625	.	A	C	100	*	TYPE=SUBSTITUTE
CM032587.1	1672649	.	G	A	100	*	TYPE=SUBSTITUTE
CM032587.1	1672667	.	G	T	100	*	TYPE=SUBSTITUTE
CM032587.1	1672679	.	A	C	100	*	TYPE=SUBSTITUTE
CM032587.1	1672704	.	C	T	100	*	TYPE=SUBSTITUTE
CM032587.1	1672715	.	C	T	100	*	TYPE=SUBSTITUTE
CM032587.1	1672785	.	A	C	100	*	TYPE=SUBSTITUTE
CM032587.1	1672790	.	C	T	100	*	TYPE=SUBSTITUTE
CM032587.1	1672953	.	T	G	100	*	TYPE=SUBSTITUTE
CM032587.1	1673008	.	CTGC	C	100	*	TYPE=DELETE
CM032587.1	1673051	.	G	GG	100	*	TYPE=INSERT
CM032587.1	1673138	.	C	T	100	*	TYPE=SUBSTITUTE
CM032587.1	1673151	.	G	C	100	*	TYPE=SUBSTITUTE
CM032587.1	1673185	.	G	A	100	*	TYPE=SUBSTITUTE
CM032587.1	1673212	.	C	T	100	*	TYPE=SUBSTITUTE
CM032587.1	1673320	.	A	G	100	*	TYPE=SUBSTITUTE
CM032587.1	1673343	.	G	A	100	*	TYPE=SUBSTITUTE
CM032587.1	1673525	.	CT	C	100	*	TYPE=DELETE
CM032587.1	1673529	.	C	T	100	*	TYPE=SUBSTITUTE
CM032587.1	1673530	.	G	C	100	*	TYPE=SUBSTITUTE
CM032587.1	1673544	.	C	T	100	*	TYPE=SUBSTITUTE
CM032587.1	1673608	.	G	A	100	*	TYPE=SUBSTITUTE
CM032587.1	1673673	.	GA	G	100	*	TYPE=DELETE
CM032587.1	1673690	.	T	C	100	*	TYPE=SUBSTITUTE
CM032587.1	1673694	.	TT	T	100	*	TYPE=DELETE
CM032587.1	1673857	.	GG	G	100	*	TYPE=DELETE
CM032587.1	1673949	.	C	T	100	*	TYPE=SUBSTITUTE
CM032587.1	1674004	.	C	CA	100	*	TYPE=INSERT
CM032587.1	1674043	.	GGACCA	G	100	*	TYPE=DELETE
CM032587.1	1674059	.	C	CTGCACTGCCTGGC	100	*	TYPE=INSERT
CM032587.1	1674074	.	T	G	100	*	TYPE=SUBSTITUTE
CM032587.1	1674090	.	T	TT	100	*	TYPE=INSERT
CM032587.1	1674115	.	C	g	100	*	TYPE=SUBSTITUTE
CM032587.1	1674136	.	A	G	100	*	TYPE=SUBSTITUTE
CM032587.1	1674197	.	A	G	100	*	TYPE=SUBSTITUTE
CM032587.1	1674295	.	A	G	100	*	TYPE=SUBSTITUTE
CM032587.1	1674296	.	G	A	100	*	TYPE=SUBSTITUTE
CM032587.1	1674307	.	G	C	100	*	TYPE=SUBSTITUTE
CM032587.1	1674398	.	G	GTG	100	*	TYPE=INSERT
CM032587.1	1674443	.	AA	A	100	*	TYPE=DELETE
CM032587.1	1674480	.	A	T	100	*	TYPE=SUBSTITUTE
CM032587.1	1674481	.	C	A	100	*	TYPE=SUBSTITUTE
CM032587.1	1674488	.	a	at	100	*	TYPE=INSERT
CM032587.1	1674496	.	C	t	100	*	TYPE=SUBSTITUTE

CM032587.1	1674497	.	C	t	100	*	TYPE=SUBSTITUTE
CM032587.1	1674499	.	T	c	100	*	TYPE=SUBSTITUTE
CM032587.1	1674590	.	C	T	100	*	TYPE=SUBSTITUTE
CM032587.1	1674664	.	T	G	100	*	TYPE=SUBSTITUTE
CM032587.1	1674676	.	C	T	100	*	TYPE=SUBSTITUTE
CM032587.1	1674713	.	C	T	100	*	TYPE=SUBSTITUTE
CM032587.1	1675027	.	C	T	100	*	TYPE=SUBSTITUTE
CM032587.1	1675056	.	G	GA	100	*	TYPE=INSERT
CM032587.1	1675070	.	A	C	100	*	TYPE=SUBSTITUTE
CM032587.1	1675097	.	A	G	100	*	TYPE=SUBSTITUTE
CM032587.1	1675099	.	C	G	100	*	TYPE=SUBSTITUTE
CM032587.1	1675164	.	AATATGGA	A	100	*	TYPE=DELETE
CM032587.1	1675208	.	GTA	G	100	*	TYPE=DELETE
CM032587.1	1675223	.	G	T	100	*	TYPE=SUBSTITUTE
CM032587.1	1675228	.	G	GGT	100	*	TYPE=INSERT
CM032587.1	1675230	.	G	GGTA	100	*	TYPE=INSERT
CM032587.1	1675233	.	G	A	100	*	TYPE=SUBSTITUTE
CM032587.1	1675257	.	T	TC	100	*	TYPE=INSERT
CM032587.1	1675277	.	G	A	100	*	TYPE=SUBSTITUTE
CM032587.1	1675292	.	C	T	100	*	TYPE=SUBSTITUTE
CM032587.1	1675296	.	A	T	100	*	TYPE=SUBSTITUTE
CM032587.1	1675311	.	TGACA	T	100	*	TYPE=DELETE
CM032587.1	1675316	.	T	g	100	*	TYPE=SUBSTITUTE
CM032587.1	1675319	.	A	T	100	*	TYPE=SUBSTITUTE
CM032587.1	1675321	.	A	C	100	*	TYPE=SUBSTITUTE
CM032587.1	1675327	.	C	T	100	*	TYPE=SUBSTITUTE
CM032587.1	1675329	.	T	TG	100	*	TYPE=INSERT
CM032587.1	1675332	.	C	T	100	*	TYPE=SUBSTITUTE
CM032587.1	1675358	.	CTTTAC	C	100	*	TYPE=DELETE
CM032587.1	1675418	.	G	T	100	*	TYPE=SUBSTITUTE
CM032587.1	1675465	.	T	G	100	*	TYPE=SUBSTITUTE
CM032587.1	1675466	.	G	A	100	*	TYPE=SUBSTITUTE
CM032587.1	1675549	.	TA	T	100	*	TYPE=DELETE
CM032587.1	1675571	.	G	A	100	*	TYPE=SUBSTITUTE
CM032587.1	1675576	.	T	C	100	*	TYPE=SUBSTITUTE
CM032587.1	1675688	.	A	AA	100	*	TYPE=INSERT
CM032587.1	1675716	.	C	T	100	*	TYPE=SUBSTITUTE
CM032587.1	1675731	.	G	A	100	*	TYPE=SUBSTITUTE
CM032587.1	1675788	.	A	G	100	*	TYPE=SUBSTITUTE
CM032587.1	1675931	.	G	a	100	*	TYPE=SUBSTITUTE
CM032587.1	1675939	.	A	g	100	*	TYPE=SUBSTITUTE
CM032587.1	1676025	.	G	C	100	*	TYPE=SUBSTITUTE
CM032587.1	1676046	.	C	T	100	*	TYPE=SUBSTITUTE
CM032587.1	1676088	.	CA	C	100	*	TYPE=DELETE
CM032587.1	1676122	.	T	C	100	*	TYPE=SUBSTITUTE
CM032587.1	1676130	.	C	t	100	*	TYPE=SUBSTITUTE
CM032587.1	1676134	.	T	c	100	*	TYPE=SUBSTITUTE
CM032587.1	1676135	.	C	t	100	*	TYPE=SUBSTITUTE
CM032587.1	1676167	.	C	T	100	*	TYPE=SUBSTITUTE
CM032587.1	1676206	.	G	A	100	*	TYPE=SUBSTITUTE
CM032587.1	1676237	.	G	A	100	*	TYPE=SUBSTITUTE
CM032587.1	1676287	.	GGG	G	100	*	TYPE=DELETE
CM032587.1	1676316	.	C	G	100	*	TYPE=SUBSTITUTE
CM032587.1	1676334	.	G	T	100	*	TYPE=SUBSTITUTE
CM032587.1	1676335	.	A	G	100	*	TYPE=SUBSTITUTE
CM032587.1	1676387	.	C	G	100	*	TYPE=SUBSTITUTE
CM032587.1	1676488	.	T	G	100	*	TYPE=SUBSTITUTE
CM032587.1	1676489	.	A	G	100	*	TYPE=SUBSTITUTE
CM032587.1	1676633	.	C	CC	100	*	TYPE=INSERT
CM032587.1	1676737	.	GGTCTAG	G	100	*	TYPE=DELETE
CM032587.1	1676780	.	CT	C	100	*	TYPE=DELETE
CM032587.1	1676795	.	C	T	100	*	TYPE=SUBSTITUTE
CM032587.1	1676833	.	C	CA	100	*	TYPE=INSERT
CM032587.1	1676927	.	C	G	100	*	TYPE=SUBSTITUTE

CM032587.1	1676943	.	T	G	100	*	TYPE=SUBSTITUTE
CM032587.1	1676965	.	A	G	100	*	TYPE=SUBSTITUTE
CM032587.1	1676998	.	T	C	100	*	TYPE=SUBSTITUTE
CM032587.1	1677046	.	T	G	100	*	TYPE=SUBSTITUTE
CM032587.1	1677052	.	C	A	100	*	TYPE=SUBSTITUTE
CM032587.1	1677237	.	C	T	100	*	TYPE=SUBSTITUTE
CM032587.1	1677277	.	TA	T	100	*	TYPE=DELETE
CM032587.1	1677349	.	G	A	100	*	TYPE=SUBSTITUTE
CM032587.1	1677354	.	A	G	100	*	TYPE=SUBSTITUTE
CM032587.1	1677391	.	C	T	100	*	TYPE=SUBSTITUTE
CM032587.1	1677399	.	G	a	100	*	TYPE=SUBSTITUTE
CM032587.1	1677400	.	T	c	100	*	TYPE=SUBSTITUTE
CM032587.1	1677409	.	g	gc	100	*	TYPE=INSERT
CM032587.1	1677414	.	G	A	100	*	TYPE=SUBSTITUTE
CM032587.1	1677415	.	T	C	100	*	TYPE=SUBSTITUTE
CM032587.1	1677416	.	C	G	100	*	TYPE=SUBSTITUTE
CM032587.1	1677418	.	T	C	100	*	TYPE=SUBSTITUTE
CM032587.1	1677419	.	C	G	100	*	TYPE=SUBSTITUTE
CM032587.1	1677420	.	G	A	100	*	TYPE=SUBSTITUTE
CM032587.1	1677424	.	TGC	T	100	*	TYPE=DELETE
CM032587.1	1677478	.	CTTTT	C	100	*	TYPE=DELETE
CM032587.1	1677526	.	TT	T	100	*	TYPE=DELETE
CM032587.1	1677549	.	G	C	100	*	TYPE=SUBSTITUTE
CM032587.1	1677550	.	C	G	100	*	TYPE=SUBSTITUTE
CM032587.1	1677571	.	G	A	100	*	TYPE=SUBSTITUTE
CM032587.1	1677572	.	A	C	100	*	TYPE=SUBSTITUTE
CM032587.1	1677576	.	T	C	100	*	TYPE=SUBSTITUTE
CM032587.1	1677577	.	C	T	100	*	TYPE=SUBSTITUTE
CM032587.1	1677578	.	G	C	100	*	TYPE=SUBSTITUTE
CM032587.1	1677601	.	G	GC	100	*	TYPE=INSERT
CM032587.1	1677609	.	C	A	100	*	TYPE=SUBSTITUTE
CM032587.1	1677615	.	T	C	100	*	TYPE=SUBSTITUTE
CM032587.1	1677624	.	CTGTA	C	100	*	TYPE=DELETE
CM032587.1	1677625	.	C	G	100	*	TYPE=SUBSTITUTE
CM032587.1	1677631	.	A	G	100	*	TYPE=SUBSTITUTE
CM032587.1	1677641	.	G	A	100	*	TYPE=SUBSTITUTE
CM032587.1	1677642	.	A	G	100	*	TYPE=SUBSTITUTE
CM032587.1	1677687	.	TG	T	100	*	TYPE=DELETE
CM032587.1	1677688	.	A	G	100	*	TYPE=SUBSTITUTE
CM032587.1	1677691	.	A	G	100	*	TYPE=SUBSTITUTE
CM032587.1	1677701	.	CCAGAATCCA	C	100	*	TYPE=DELETE
CM032587.1	1677771	.	T	A	100	*	TYPE=SUBSTITUTE
CM032587.1	1677772	.	C	G	100	*	TYPE=SUBSTITUTE
CM032587.1	1677780	.	T	C	100	*	TYPE=SUBSTITUTE
CM032587.1	1677792	.	C	G	100	*	TYPE=SUBSTITUTE
CM032587.1	1677795	.	C	CT	100	*	TYPE=INSERT
CM032587.1	1677806	.	A	T	100	*	TYPE=SUBSTITUTE
CM032587.1	1677849	.	A	t	100	*	TYPE=SUBSTITUTE
CM032587.1	1677851	.	AC	A	100	*	TYPE=DELETE
CM032587.1	1677858	.	T	a	100	*	TYPE=SUBSTITUTE
CM032587.1	1677862	.	A	g	100	*	TYPE=SUBSTITUTE
CM032587.1	1677864	.	A	t	100	*	TYPE=SUBSTITUTE
CM032587.1	1677867	.	A	G	100	*	TYPE=SUBSTITUTE
CM032587.1	1677875	.	AACCTTGG	A	100	*	TYPE=DELETE
CM032587.1	1677879	.	T	A	100	*	TYPE=SUBSTITUTE
CM032587.1	1677894	.	c	ca	100	*	TYPE=INSERT
CM032587.1	1677903	.	AGGATG	A	100	*	TYPE=DELETE
CM032587.1	1677909	.	C	T	100	*	TYPE=SUBSTITUTE
CM032587.1	1677915	.	AACAC	A	100	*	TYPE=DELETE
CM032587.1	1677920	.	G	A	100	*	TYPE=SUBSTITUTE
CM032587.1	1677945	.	G	A	100	*	TYPE=SUBSTITUTE
CM032587.1	1677953	.	A	G	100	*	TYPE=SUBSTITUTE
CM032587.1	1677966	.	G	A	100	*	TYPE=SUBSTITUTE
CM032587.1	1677986	.	C	G	100	*	TYPE=SUBSTITUTE

CM032587.1	1678010	.	G	A	100	*	TYPE=SUBSTITUTE
CM032587.1	1678042	.	T	C	100	*	TYPE=SUBSTITUTE
CM032587.1	1678047	.	T	G	100	*	TYPE=SUBSTITUTE
CM032587.1	1678048	.	A	T	100	*	TYPE=SUBSTITUTE
CM032587.1	1678057	.	C	T	100	*	TYPE=SUBSTITUTE
CM032587.1	1678076	.	T	A	100	*	TYPE=SUBSTITUTE
CM032587.1	1678093	.	C	T	100	*	TYPE=SUBSTITUTE
CM032587.1	1678121	.	A	C	100	*	TYPE=SUBSTITUTE
CM032587.1	1678122	.	C	CC	100	*	TYPE=INSERT
CM032587.1	1678155	.	TT	T	100	*	TYPE=DELETE
CM032587.1	1678188	.	C	T	100	*	TYPE=SUBSTITUTE
CM032587.1	1678189	.	T	A	100	*	TYPE=SUBSTITUTE
CM032587.1	1678299	.	G	A	100	*	TYPE=SUBSTITUTE
CM032587.1	1678451	.	AT	A	100	*	TYPE=DELETE
CM032587.1	1678473	.	GCTA	G	100	*	TYPE=DELETE
CM032587.1	1678489	.	TC	T	100	*	TYPE=DELETE
CM032587.1	1678509	.	T	g	100	*	TYPE=SUBSTITUTE
CM032587.1	1678521	.	T	c	100	*	TYPE=SUBSTITUTE
CM032587.1	1678523	.	TT	T	100	*	TYPE=DELETE
CM032587.1	1678548	.	C	T	100	*	TYPE=SUBSTITUTE
CM032587.1	1678570	.	ACTG	A	100	*	TYPE=DELETE
CM032587.1	1678575	.	A	G	100	*	TYPE=SUBSTITUTE
CM032587.1	1678580	.	C	A	100	*	TYPE=SUBSTITUTE
CM032587.1	1678585	.	G	A	100	*	TYPE=SUBSTITUTE
CM032587.1	1678607	.	CCTGCG	C	100	*	TYPE=DELETE
CM032587.1	1678626	.	C	A	100	*	TYPE=SUBSTITUTE
CM032587.1	1678637	.	G	c	100	*	TYPE=SUBSTITUTE
CM032587.1	1678638	.	A	t	100	*	TYPE=SUBSTITUTE
CM032587.1	1678645	.	G	GGTgt	100	*	TYPE=INSERT
CM032587.1	1678653	.	T	TG	100	*	TYPE=INSERT
CM032587.1	1678681	.	G	A	100	*	TYPE=SUBSTITUTE
CM032587.1	1678684	.	T	C	100	*	TYPE=SUBSTITUTE
CM032587.1	1678723	.	C	T	100	*	TYPE=SUBSTITUTE
CM032587.1	1678728	.	G	A	100	*	TYPE=SUBSTITUTE
CM032587.1	1678729	.	A	G	100	*	TYPE=SUBSTITUTE
CM032587.1	1678731	.	T	C	100	*	TYPE=SUBSTITUTE
CM032587.1	1678775	.	G	a	100	*	TYPE=SUBSTITUTE
CM032587.1	1678933	.	A	g	100	*	TYPE=SUBSTITUTE
CM032587.1	1678935	.	AA	A	100	*	TYPE=DELETE
CM032587.1	1678955	.	G	A	100	*	TYPE=SUBSTITUTE
CM032587.1	1679126	.	GG	G	100	*	TYPE=DELETE
CM032587.1	1679509	.	C	T	100	*	TYPE=SUBSTITUTE
CM032587.1	1679554	.	C	CTAC	100	*	TYPE=INSERT
CM032587.1	1679698	.	A	G	100	*	TYPE=SUBSTITUTE
CM032587.1	1679704	.	A	G	100	*	TYPE=SUBSTITUTE
CM032587.1	1679747	.	A	G	100	*	TYPE=SUBSTITUTE
CM032587.1	1679811	.	G	A	100	*	TYPE=SUBSTITUTE
CM032587.1	1680010	.	G	A	100	*	TYPE=SUBSTITUTE
CM032587.1	1680014	.	G	C	100	*	TYPE=SUBSTITUTE
CM032587.1	1680040	.	C	T	100	*	TYPE=SUBSTITUTE
CM032587.1	1680041	.	A	T	100	*	TYPE=SUBSTITUTE
CM032587.1	1680045	.	GCCA	G	100	*	TYPE=DELETE
CM032587.1	1680066	.	G	A	100	*	TYPE=SUBSTITUTE
CM032587.1	1680075	.	G	A	100	*	TYPE=SUBSTITUTE
CM032587.1	1680096	.	A	G	100	*	TYPE=SUBSTITUTE
CM032587.1	1680126	.	G	a	100	*	TYPE=SUBSTITUTE
CM032587.1	1680140	.	T	g	100	*	TYPE=SUBSTITUTE
CM032587.1	1680145	.	G	t	100	*	TYPE=SUBSTITUTE
CM032587.1	1680152	.	G	A	100	*	TYPE=SUBSTITUTE
CM032587.1	1680153	.	T	C	100	*	TYPE=SUBSTITUTE
CM032587.1	1680157	.	T	G	100	*	TYPE=SUBSTITUTE
CM032587.1	1680159	.	A	G	100	*	TYPE=SUBSTITUTE
CM032587.1	1680168	.	G	A	100	*	TYPE=SUBSTITUTE
CM032587.1	1680171	.	G	T	100	*	TYPE=SUBSTITUTE

CM032587.1	1680183	.	GGCT	G	100	*	TYPE=DELETE
CM032587.1	1680190	.	G	C	100	*	TYPE=SUBSTITUTE
CM032587.1	1680208	.	G	C	100	*	TYPE=SUBSTITUTE
CM032587.1	1680217	.	C	T	100	*	TYPE=SUBSTITUTE
CM032587.1	1680243	.	G	A	100	*	TYPE=SUBSTITUTE
CM032587.1	1680249	.	A	G	100	*	TYPE=SUBSTITUTE
CM032587.1	1680264	.	C	T	100	*	TYPE=SUBSTITUTE
CM032587.1	1680372	.	T	c	100	*	TYPE=SUBSTITUTE
CM032587.1	1680399	.	C	T	100	*	TYPE=SUBSTITUTE
CM032587.1	1680405	.	C	A	100	*	TYPE=SUBSTITUTE
CM032587.1	1680462	.	C	T	100	*	TYPE=SUBSTITUTE
CM032587.1	1680532	.	G	A	100	*	TYPE=SUBSTITUTE
CM032587.1	1680609	.	G	A	100	*	TYPE=SUBSTITUTE
CM032587.1	1680645	.	C	G	100	*	TYPE=SUBSTITUTE
CM032587.1	1680657	.	G	A	100	*	TYPE=SUBSTITUTE
CM032587.1	1680660	.	G	A	100	*	TYPE=SUBSTITUTE
CM032587.1	1680675	.	G	A	100	*	TYPE=SUBSTITUTE
CM032587.1	1680726	.	A	G	100	*	TYPE=SUBSTITUTE
CM032587.1	1680804	.	G	A	100	*	TYPE=SUBSTITUTE
CM032587.1	1680828	.	T	C	100	*	TYPE=SUBSTITUTE
CM032587.1	1680867	.	G	C	100	*	TYPE=SUBSTITUTE
CM032587.1	1680960	.	G	A	100	*	TYPE=SUBSTITUTE
CM032587.1	1680981	.	A	G	100	*	TYPE=SUBSTITUTE
CM032587.1	1681011	.	G	A	100	*	TYPE=SUBSTITUTE
CM032587.1	1681038	.	G	A	100	*	TYPE=SUBSTITUTE
CM032587.1	1681095	.	G	C	100	*	TYPE=SUBSTITUTE
CM032587.1	1681098	.	T	C	100	*	TYPE=SUBSTITUTE
CM032587.1	1681101	.	A	G	100	*	TYPE=SUBSTITUTE
CM032587.1	1681110	.	G	A	100	*	TYPE=SUBSTITUTE
CM032587.1	1681159	.	C	T	100	*	TYPE=SUBSTITUTE
CM032587.1	1681182	.	T	C	100	*	TYPE=SUBSTITUTE
CM032587.1	1681205	.	C	T	100	*	TYPE=SUBSTITUTE
CM032587.1	1681245	.	T	C	100	*	TYPE=SUBSTITUTE
CM032587.1	1681362	.	A	G	100	*	TYPE=SUBSTITUTE
CM032587.1	1681386	.	T	G	100	*	TYPE=SUBSTITUTE
CM032587.1	1681407	.	T	C	100	*	TYPE=SUBSTITUTE
CM032587.1	1681434	.	T	C	100	*	TYPE=SUBSTITUTE
CM032587.1	1681470	.	A	G	100	*	TYPE=SUBSTITUTE
CM032587.1	1681476	.	C	T	100	*	TYPE=SUBSTITUTE
CM032587.1	1681485	.	T	C	100	*	TYPE=SUBSTITUTE
CM032587.1	1681530	.	G	A	100	*	TYPE=SUBSTITUTE
CM032587.1	1681614	.	A	G	100	*	TYPE=SUBSTITUTE
CM032587.1	1681642	.	T	C	100	*	TYPE=SUBSTITUTE
CM032587.1	1681644	.	A	C	100	*	TYPE=SUBSTITUTE
CM032587.1	1681650	.	A	G	100	*	TYPE=SUBSTITUTE
CM032587.1	1681677	.	G	A	100	*	TYPE=SUBSTITUTE
CM032587.1	1681686	.	G	A	100	*	TYPE=SUBSTITUTE
CM032587.1	1681689	.	C	T	100	*	TYPE=SUBSTITUTE
CM032587.1	1681719	.	A	G	100	*	TYPE=SUBSTITUTE
CM032587.1	1681746	.	G	C	100	*	TYPE=SUBSTITUTE
CM032587.1	1681752	.	A	G	100	*	TYPE=SUBSTITUTE
CM032587.1	1681767	.	C	T	100	*	TYPE=SUBSTITUTE
CM032587.1	1681773	.	T	C	100	*	TYPE=SUBSTITUTE
CM032587.1	1681774	.	A	G	100	*	TYPE=SUBSTITUTE
CM032587.1	1681776	.	T	C	100	*	TYPE=SUBSTITUTE
CM032587.1	1681779	.	G	A	100	*	TYPE=SUBSTITUTE
CM032587.1	1681786	.	C	A	100	*	TYPE=SUBSTITUTE
CM032587.1	1681788	.	C	T	100	*	TYPE=SUBSTITUTE
CM032587.1	1681789	.	A	C	100	*	TYPE=SUBSTITUTE
CM032587.1	1681790	.	G	T	100	*	TYPE=SUBSTITUTE
CM032587.1	1681791	.	G	T	100	*	TYPE=SUBSTITUTE
CM032587.1	1681803	.	G	a	100	*	TYPE=SUBSTITUTE
CM032587.1	1681812	.	C	T	100	*	TYPE=SUBSTITUTE
CM032587.1	1681827	.	A	G	100	*	TYPE=SUBSTITUTE

CM032587.1	1681871	.	A	G	100	*	TYPE=SUBSTITUTE
CM032587.1	1681881	.	G	A	100	*	TYPE=SUBSTITUTE
CM032587.1	1681896	.	C	T	100	*	TYPE=SUBSTITUTE
CM032587.1	1681902	.	G	A	100	*	TYPE=SUBSTITUTE
CM032587.1	1681926	.	G	A	100	*	TYPE=SUBSTITUTE
CM032587.1	1681954	.	C	t	100	*	TYPE=SUBSTITUTE
CM032587.1	1681968	.	T	C	100	*	TYPE=SUBSTITUTE
CM032587.1	1681977	.	G	A	100	*	TYPE=SUBSTITUTE
CM032587.1	1681986	.	G	A	100	*	TYPE=SUBSTITUTE
CM032587.1	1682004	.	A	G	100	*	TYPE=SUBSTITUTE
CM032587.1	1682069	.	C	G	100	*	TYPE=SUBSTITUTE
CM032587.1	1682070	.	G	A	100	*	TYPE=SUBSTITUTE
CM032587.1	1682097	.	A	G	100	*	TYPE=SUBSTITUTE
CM032587.1	1682106	.	G	A	100	*	TYPE=SUBSTITUTE
CM032587.1	1682133	.	G	A	100	*	TYPE=SUBSTITUTE
CM032587.1	1682142	.	T	C	100	*	TYPE=SUBSTITUTE
CM032587.1	1682148	.	C	T	100	*	TYPE=SUBSTITUTE
CM032587.1	1682157	.	T	C	100	*	TYPE=SUBSTITUTE
CM032587.1	1682202	.	T	C	100	*	TYPE=SUBSTITUTE
CM032587.1	1682247	.	G	A	100	*	TYPE=SUBSTITUTE
CM032587.1	1682274	.	C	t	100	*	TYPE=SUBSTITUTE
CM032587.1	1682293	.	G	T	100	*	TYPE=SUBSTITUTE
CM032587.1	1682322	.	C	A	100	*	TYPE=SUBSTITUTE
CM032587.1	1682355	.	G	A	100	*	TYPE=SUBSTITUTE
CM032587.1	1682415	.	G	T	100	*	TYPE=SUBSTITUTE
CM032587.1	1682457	.	G	A	100	*	TYPE=SUBSTITUTE
CM032587.1	1682472	.	A	G	100	*	TYPE=SUBSTITUTE
CM032587.1	1682481	.	A	G	100	*	TYPE=SUBSTITUTE
CM032587.1	1682484	.	C	G	100	*	TYPE=SUBSTITUTE
CM032587.1	1682520	.	T	C	100	*	TYPE=SUBSTITUTE
CM032587.1	1682524	.	C	T	100	*	TYPE=SUBSTITUTE
CM032587.1	1682568	.	T	C	100	*	TYPE=SUBSTITUTE
CM032587.1	1682634	.	A	G	100	*	TYPE=SUBSTITUTE
CM032587.1	1682643	.	T	C	100	*	TYPE=SUBSTITUTE
CM032587.1	1682658	.	G	A	100	*	TYPE=SUBSTITUTE
CM032587.1	1682713	.	A	g	100	*	TYPE=SUBSTITUTE
CM032587.1	1682715	.	G	A	100	*	TYPE=SUBSTITUTE
CM032587.1	1682787	.	A	G	100	*	TYPE=SUBSTITUTE
CM032587.1	1682795	.	C	T	100	*	TYPE=SUBSTITUTE
CM032587.1	1682808	.	G	A	100	*	TYPE=SUBSTITUTE
CM032587.1	1682890	.	T	C	100	*	TYPE=SUBSTITUTE
CM032587.1	1682922	.	G	A	100	*	TYPE=SUBSTITUTE
CM032587.1	1682973	.	A	G	100	*	TYPE=SUBSTITUTE
CM032587.1	1683009	.	G	a	100	*	TYPE=SUBSTITUTE
CM032587.1	1683034	.	T	C	100	*	TYPE=SUBSTITUTE
CM032587.1	1683051	.	T	C	100	*	TYPE=SUBSTITUTE
CM032587.1	1683110	.	A	ACGC	100	*	TYPE=INSERT
CM032587.1	1683112	.	G	A	100	*	TYPE=SUBSTITUTE
CM032587.1	1683147	.	A	G	100	*	TYPE=SUBSTITUTE
CM032587.1	1683162	.	A	G	100	*	TYPE=SUBSTITUTE
CM032587.1	1683202	.	TG	T	100	*	TYPE=DELETE
CM032587.1	1683204	.	G	A	100	*	TYPE=SUBSTITUTE
CM032587.1	1683224	.	A	G	100	*	TYPE=SUBSTITUTE
CM032587.1	1683274	.	A	G	100	*	TYPE=SUBSTITUTE
CM032587.1	1683282	.	TG	T	100	*	TYPE=DELETE
CM032587.1	1683283	.	T	C	100	*	TYPE=SUBSTITUTE
CM032587.1	1683328	.	G	A	100	*	TYPE=SUBSTITUTE
CM032587.1	1683365	.	G	A	100	*	TYPE=SUBSTITUTE
CM032587.1	1683394	.	A	T	100	*	TYPE=SUBSTITUTE
CM032587.1	1683406	.	A	g	100	*	TYPE=SUBSTITUTE
CM032587.1	1683431	.	G	T	100	*	TYPE=SUBSTITUTE
CM032587.1	1683478	.	G	C	100	*	TYPE=SUBSTITUTE
CM032587.1	1683544	.	A	g	100	*	TYPE=SUBSTITUTE
CM032587.1	1683545	.	T	a	100	*	TYPE=SUBSTITUTE

CM032587.1	1683576	.	A	C	100	*	TYPE=SUBSTITUTE
CM032587.1	1683577	.	G	A	100	*	TYPE=SUBSTITUTE
CM032587.1	1683618	.	G	T	100	*	TYPE=SUBSTITUTE
CM032587.1	1683630	.	G	A	100	*	TYPE=SUBSTITUTE
CM032587.1	1683677	.	G	t	100	*	TYPE=SUBSTITUTE
CM032587.1	1683713	.	C	T	100	*	TYPE=SUBSTITUTE
CM032587.1	1683730	.	C	T	100	*	TYPE=SUBSTITUTE
CM032587.1	1683746	.	G	T	100	*	TYPE=SUBSTITUTE
CM032587.1	1683817	.	C	T	100	*	TYPE=SUBSTITUTE
CM032587.1	1683847	.	AAA	A	100	*	TYPE=DELETE
CM032587.1	1683864	.	T	a	100	*	TYPE=SUBSTITUTE
CM032587.1	1683870	.	A	g	100	*	TYPE=SUBSTITUTE
CM032587.1	1683871	.	T	a	100	*	TYPE=SUBSTITUTE
CM032587.1	1683872	.	A	g	100	*	TYPE=SUBSTITUTE
CM032587.1	1683876	.	A	g	100	*	TYPE=SUBSTITUTE
CM032587.1	1683880	.	A	g	100	*	TYPE=SUBSTITUTE
CM032587.1	1683883	.	G	a	100	*	TYPE=SUBSTITUTE
CM032587.1	1683907	.	C	T	100	*	TYPE=SUBSTITUTE
CM032587.1	1683926	.	A	G	100	*	TYPE=SUBSTITUTE
CM032587.1	1683937	.	G	t	100	*	TYPE=SUBSTITUTE
CM032587.1	1683941	.	a	aag	100	*	TYPE=INSERT
CM032587.1	1683953	.	A	g	100	*	TYPE=SUBSTITUTE
CM032587.1	1683977	.	G	a	100	*	TYPE=SUBSTITUTE
CM032587.1	1683978	.	a	aagaaaag	100	*	TYPE=INSERT
CM032587.1	1684031	.	T	G	100	*	TYPE=SUBSTITUTE
CM032587.1	1684033	.	A	T	100	*	TYPE=SUBSTITUTE
CM032587.1	1684034	.	T	A	100	*	TYPE=SUBSTITUTE
CM032587.1	1684051	.	C	T	100	*	TYPE=SUBSTITUTE
CM032587.1	1684070	.	C	T	100	*	TYPE=SUBSTITUTE
CM032587.1	1684072	.	C	T	100	*	TYPE=SUBSTITUTE
CM032587.1	1684105	.	A	T	100	*	TYPE=SUBSTITUTE
CM032587.1	1684110	.	CATT	C	100	*	TYPE=DELETE
CM032587.1	1684120	.	AA	A	100	*	TYPE=DELETE
CM032587.1	1684171	.	A	T	100	*	TYPE=SUBSTITUTE
CM032587.1	1684172	.	T	A	100	*	TYPE=SUBSTITUTE
CM032587.1	1684203	.	C	T	100	*	TYPE=SUBSTITUTE
CM032587.1	1684254	.	CGC	C	100	*	TYPE=DELETE
CM032587.1	1684442	.	A	g	100	*	TYPE=SUBSTITUTE
CM032587.1	1684477	.	A	T	100	*	TYPE=SUBSTITUTE
CM032587.1	1684478	.	C	G	100	*	TYPE=SUBSTITUTE
CM032587.1	1684516	.	G	A	100	*	TYPE=SUBSTITUTE
CM032587.1	1684522	.	CAA	C	100	*	TYPE=DELETE
CM032587.1	1684524	.	A	G	100	*	TYPE=SUBSTITUTE
CM032587.1	1684550	.	G	a	100	*	TYPE=SUBSTITUTE
CM032587.1	1684551	.	g	gtc	100	*	TYPE=INSERT
CM032587.1	1684566	.	A	G	100	*	TYPE=SUBSTITUTE
CM032587.1	1684576	.	G	A	100	*	TYPE=SUBSTITUTE
CM032587.1	1684579	.	A	G	100	*	TYPE=SUBSTITUTE
CM032587.1	1684581	.	T	A	100	*	TYPE=SUBSTITUTE
CM032587.1	1684586	.	C	T	100	*	TYPE=SUBSTITUTE
CM032587.1	1684599	.	T	C	100	*	TYPE=SUBSTITUTE
CM032587.1	1684609	.	A	T	100	*	TYPE=SUBSTITUTE
CM032587.1	1684617	.	A	G	100	*	TYPE=SUBSTITUTE
CM032587.1	1684618	.	A	T	100	*	TYPE=SUBSTITUTE
CM032587.1	1684647	.	C	T	100	*	TYPE=SUBSTITUTE
CM032587.1	1684675	.	A	T	100	*	TYPE=SUBSTITUTE
CM032587.1	1684709	.	T	TTGCC	100	*	TYPE=INSERT
CM032587.1	1684743	.	G	A	100	*	TYPE=SUBSTITUTE
CM032587.1	1684761	.	T	G	100	*	TYPE=SUBSTITUTE
CM032587.1	1684762	.	T	TG	100	*	TYPE=INSERT
CM032587.1	1684772	.	G	A	100	*	TYPE=SUBSTITUTE
CM032587.1	1684784	.	T	G	100	*	TYPE=SUBSTITUTE
CM032587.1	1684884	.	A	G	100	*	TYPE=SUBSTITUTE
CM032587.1	1684899	.	CCACC	C	100	*	TYPE=DELETE

CM032587.1	1684914	.	A	G	100	*	TYPE=SUBSTITUTE
CM032587.1	1684936	.	T	A	100	*	TYPE=SUBSTITUTE
CM032587.1	1684938	.	A	G	100	*	TYPE=SUBSTITUTE
CM032587.1	1684948	.	GT	G	100	*	TYPE=DELETE
CM032587.1	1684956	.	GG	G	100	*	TYPE=DELETE
CM032587.1	1684997	.	A	G	100	*	TYPE=SUBSTITUTE
CM032587.1	1685018	.	TT	T	100	*	TYPE=DELETE
CM032587.1	1685042	.	T	A	100	*	TYPE=SUBSTITUTE
CM032587.1	1685043	.	T	C	100	*	TYPE=SUBSTITUTE
CM032587.1	1685060	.	T	C	100	*	TYPE=SUBSTITUTE
CM032587.1	1685066	.	T	A	100	*	TYPE=SUBSTITUTE
CM032587.1	1685184	.	C	T	100	*	TYPE=SUBSTITUTE
CM032587.1	1685223	.	T	C	100	*	TYPE=SUBSTITUTE
CM032587.1	1685235	.	CA	C	100	*	TYPE=DELETE
CM032587.1	1685248	.	T	C	100	*	TYPE=SUBSTITUTE
CM032587.1	1685249	.	T	a	100	*	TYPE=SUBSTITUTE
CM032587.1	1685251	.	T	a	100	*	TYPE=SUBSTITUTE
CM032587.1	1685262	.	CTCGT	C	100	*	TYPE=DELETE
CM032587.1	1685284	.	G	A	100	*	TYPE=SUBSTITUTE
CM032587.1	1685286	.	a	ac	100	*	TYPE=INSERT
CM032587.1	1685287	.	T	a	100	*	TYPE=SUBSTITUTE
CM032587.1	1685292	.	a	at	100	*	TYPE=INSERT
CM032587.1	1685312	.	G	a	100	*	TYPE=SUBSTITUTE
CM032587.1	1685315	.	g	ga	100	*	TYPE=INSERT
CM032587.1	1685328	.	TA	T	100	*	TYPE=DELETE
CM032587.1	1685337	.	T	C	100	*	TYPE=SUBSTITUTE
CM032587.1	1685349	.	G	A	100	*	TYPE=SUBSTITUTE
CM032587.1	1685363	.	A	T	100	*	TYPE=SUBSTITUTE
CM032587.1	1685394	.	C	T	100	*	TYPE=SUBSTITUTE
CM032587.1	1685406	.	TCC	T	100	*	TYPE=DELETE
CM032587.1	1685412	.	A	G	100	*	TYPE=SUBSTITUTE
CM032587.1	1685415	.	A	T	100	*	TYPE=SUBSTITUTE
CM032587.1	1685446	.	G	GA	100	*	TYPE=INSERT
CM032587.1	1685454	.	TT	T	100	*	TYPE=DELETE
CM032587.1	1685491	.	G	C	100	*	TYPE=SUBSTITUTE
CM032587.1	1685522	.	C	T	100	*	TYPE=SUBSTITUTE
CM032587.1	1685524	.	A	T	100	*	TYPE=SUBSTITUTE
CM032587.1	1685525	.	G	A	100	*	TYPE=SUBSTITUTE
CM032587.1	1685543	.	G	GGAT	100	*	TYPE=INSERT
CM032587.1	1685554	.	T	TCCGCAGAGA	100	*	TYPE=INSERT
CM032587.1	1685565	.	T	C	100	*	TYPE=SUBSTITUTE
CM032587.1	1685569	.	A	G	100	*	TYPE=SUBSTITUTE
CM032587.1	1685603	.	A	C	100	*	TYPE=SUBSTITUTE
CM032587.1	1685607	.	GC	G	100	*	TYPE=DELETE
CM032587.1	1685632	.	C	T	100	*	TYPE=SUBSTITUTE
CM032587.1	1685644	.	C	A	100	*	TYPE=SUBSTITUTE
CM032587.1	1685680	.	G	GT	100	*	TYPE=INSERT
CM032587.1	1685681	.	C	T	100	*	TYPE=SUBSTITUTE
CM032587.1	1685683	.	T	G	100	*	TYPE=SUBSTITUTE
CM032587.1	1685713	.	A	G	100	*	TYPE=SUBSTITUTE
CM032587.1	1685718	.	C	T	100	*	TYPE=SUBSTITUTE
CM032587.1	1685724	.	TGC	T	100	*	TYPE=DELETE
CM032587.1	1685727	.	T	A	100	*	TYPE=SUBSTITUTE
CM032587.1	1685731	.	C	T	100	*	TYPE=SUBSTITUTE
CM032587.1	1685755	.	T	G	100	*	TYPE=SUBSTITUTE
CM032587.1	1685817	.	T	c	100	*	TYPE=SUBSTITUTE
CM032587.1	1685828	.	TTACTCGCTCG	T	100	*	TYPE=DELETE
CM032587.1	1685839	.	T	c	100	*	TYPE=SUBSTITUTE
CM032587.1	1685840	.	C	T	100	*	TYPE=SUBSTITUTE
CM032587.1	1685851	.	CC	C	100	*	TYPE=DELETE
CM032587.1	1685873	.	C	t	100	*	TYPE=SUBSTITUTE
CM032587.1	1685923	.	c	ct	100	*	TYPE=INSERT
CM032587.1	1685931	.	C	t	100	*	TYPE=SUBSTITUTE
CM032587.1	1685934	.	T	c	100	*	TYPE=SUBSTITUTE

CM032587.1	1685972	.	T	G	100	*	TYPE=SUBSTITUTE
CM032587.1	1685977	.	AC	A	100	*	TYPE=DELETE
CM032587.1	1685988	.	A	G	100	*	TYPE=SUBSTITUTE
CM032587.1	1686016	.	CG	C	100	*	TYPE=DELETE
CM032587.1	1686018	.	A	G	100	*	TYPE=SUBSTITUTE
CM032587.1	1686073	.	T	C	100	*	TYPE=SUBSTITUTE
CM032587.1	1686155	.	T	G	100	*	TYPE=SUBSTITUTE
CM032587.1	1686193	.	G	A	100	*	TYPE=SUBSTITUTE
CM032587.1	1686194	.	G	GC	100	*	TYPE=INSERT
CM032587.1	1686224	.	G	T	100	*	TYPE=SUBSTITUTE
CM032587.1	1686244	.	G	A	100	*	TYPE=SUBSTITUTE
CM032587.1	1686246	.	C	A	100	*	TYPE=SUBSTITUTE
CM032587.1	1686248	.	T	C	100	*	TYPE=SUBSTITUTE
CM032587.1	1686249	.	A	c	100	*	TYPE=SUBSTITUTE
CM032587.1	1686253	.	CA	C	100	*	TYPE=DELETE
CM032587.1	1686263	.	GA	G	100	*	TYPE=DELETE
CM032587.1	1686273	.	T	C	100	*	TYPE=SUBSTITUTE
CM032587.1	1686297	.	G	A	100	*	TYPE=SUBSTITUTE
CM032587.1	1686298	.	A	ACA	100	*	TYPE=INSERT
CM032587.1	1686351	.	C	A	100	*	TYPE=SUBSTITUTE
CM032587.1	1686355	.	TGC	T	100	*	TYPE=DELETE
CM032587.1	1686357	.	C	G	100	*	TYPE=SUBSTITUTE
CM032587.1	1686391	.	TTTT	T	100	*	TYPE=DELETE
CM032587.1	1686410	.	C	A	100	*	TYPE=SUBSTITUTE
CM032587.1	1686427	.	C	CAAG	100	*	TYPE=INSERT
CM032587.1	1686431	.	C	CA	100	*	TYPE=INSERT
CM032587.1	1686433	.	C	G	100	*	TYPE=SUBSTITUTE
CM032587.1	1686453	.	G	A	100	*	TYPE=SUBSTITUTE
CM032587.1	1686455	.	C	A	100	*	TYPE=SUBSTITUTE
CM032587.1	1686460	.	T	C	100	*	TYPE=SUBSTITUTE
CM032587.1	1686489	.	G	A	100	*	TYPE=SUBSTITUTE
CM032587.1	1686539	.	C	T	100	*	TYPE=SUBSTITUTE
CM032587.1	1686578	.	AA	A	100	*	TYPE=DELETE
CM032587.1	1686642	.	TC	T	100	*	TYPE=DELETE
CM032587.1	1686645	.	TT	T	100	*	TYPE=DELETE
CM032587.1	1686673	.	T	g	100	*	TYPE=SUBSTITUTE
CM032587.1	1686674	.	G	t	100	*	TYPE=SUBSTITUTE
CM032587.1	1686691	.	TA	T	100	*	TYPE=DELETE
CM032587.1	1686702	.	A	C	100	*	TYPE=SUBSTITUTE
CM032587.1	1686709	.	AG	A	100	*	TYPE=DELETE
CM032587.1	1686712	.	G	a	100	*	TYPE=SUBSTITUTE
CM032587.1	1686713	.	G	c	100	*	TYPE=SUBSTITUTE
CM032587.1	1686721	.	C	g	100	*	TYPE=SUBSTITUTE
CM032587.1	1686725	.	C	g	100	*	TYPE=SUBSTITUTE
CM032587.1	1686727	.	A	c	100	*	TYPE=SUBSTITUTE
CM032587.1	1686732	.	GACA	G	100	*	TYPE=DELETE
CM032587.1	1686764	.	C	G	100	*	TYPE=SUBSTITUTE
CM032587.1	1686836	.	A	G	100	*	TYPE=SUBSTITUTE
CM032587.1	1686840	.	A	C	100	*	TYPE=SUBSTITUTE
CM032587.1	1686846	.	G	GTGCATTC	100	*	TYPE=INSERT
CM032587.1	1686848	.	G	C	100	*	TYPE=SUBSTITUTE
CM032587.1	1686861	.	C	t	100	*	TYPE=SUBSTITUTE
CM032587.1	1686863	.	T	c	100	*	TYPE=SUBSTITUTE
CM032587.1	1686872	.	C	t	100	*	TYPE=SUBSTITUTE
CM032587.1	1686875	.	T	C	100	*	TYPE=SUBSTITUTE
CM032587.1	1686881	.	A	T	100	*	TYPE=SUBSTITUTE
CM032587.1	1686887	.	G	A	100	*	TYPE=SUBSTITUTE
CM032587.1	1686888	.	A	C	100	*	TYPE=SUBSTITUTE
CM032587.1	1686892	.	G	A	100	*	TYPE=SUBSTITUTE
CM032587.1	1686895	.	GT	G	100	*	TYPE=DELETE
CM032587.1	1686896	.	A	T	100	*	TYPE=SUBSTITUTE
CM032587.1	1686901	.	T	A	100	*	TYPE=SUBSTITUTE
CM032587.1	1686902	.	A	AG	100	*	TYPE=INSERT
CM032587.1	1686907	.	T	G	100	*	TYPE=SUBSTITUTE

CM032587.1	1686910	.	G	A	100	*	TYPE=SUBSTITUTE
CM032587.1	1686916	.	T	A	100	*	TYPE=SUBSTITUTE
CM032587.1	1686917	.	A	T	100	*	TYPE=SUBSTITUTE
CM032587.1	1686920	.	T	TAGTA	100	*	TYPE=INSERT
CM032587.1	1686935	.	C	T	100	*	TYPE=SUBSTITUTE
CM032587.1	1686947	.	T	C	100	*	TYPE=SUBSTITUTE
CM032587.1	1686958	.	CT	C	100	*	TYPE=DELETE
CM032587.1	1686961	.	C	T	100	*	TYPE=SUBSTITUTE
CM032587.1	1686964	.	A	C	100	*	TYPE=SUBSTITUTE
CM032587.1	1686966	.	C	t	100	*	TYPE=SUBSTITUTE
CM032587.1	1686967	.	t	tac	100	*	TYPE=INSERT
CM032587.1	1686971	.	T	c	100	*	TYPE=SUBSTITUTE
CM032587.1	1686976	.	t	tctc	100	*	TYPE=INSERT
CM032587.1	1686986	.	T	g	100	*	TYPE=SUBSTITUTE
CM032587.1	1686993	.	T	C	100	*	TYPE=SUBSTITUTE
CM032587.1	1686995	.	G	A	100	*	TYPE=SUBSTITUTE
CM032587.1	1687000	.	CA	C	100	*	TYPE=DELETE
CM032587.1	1687001	.	A	T	100	*	TYPE=SUBSTITUTE
CM032587.1	1687030	.	T	C	100	*	TYPE=SUBSTITUTE
CM032587.1	1687102	.	CG	C	100	*	TYPE=DELETE
CM032587.1	1687168	.	C	T	100	*	TYPE=SUBSTITUTE
CM032587.1	1687169	.	A	T	100	*	TYPE=SUBSTITUTE
CM032587.1	1687173	.	C	CT	100	*	TYPE=INSERT
CM032587.1	1687176	.	A	T	100	*	TYPE=SUBSTITUTE
CM032587.1	1687180	.	A	G	100	*	TYPE=SUBSTITUTE
CM032587.1	1687196	.	GT	G	100	*	TYPE=DELETE
CM032587.1	1687201	.	C	T	100	*	TYPE=SUBSTITUTE
CM032587.1	1687203	.	A	G	100	*	TYPE=SUBSTITUTE
CM032587.1	1687220	.	T	C	100	*	TYPE=SUBSTITUTE
CM032587.1	1687221	.	C	G	100	*	TYPE=SUBSTITUTE
CM032587.1	1687223	.	C	T	100	*	TYPE=SUBSTITUTE
CM032587.1	1687228	.	T	A	100	*	TYPE=SUBSTITUTE
CM032587.1	1687250	.	G	c	100	*	TYPE=SUBSTITUTE
CM032587.1	1687279	.	A	G	100	*	TYPE=SUBSTITUTE
CM032587.1	1687328	.	T	C	100	*	TYPE=SUBSTITUTE
CM032587.1	1687335	.	C	G	100	*	TYPE=SUBSTITUTE
CM032587.1	1687338	.	TCT	T	100	*	TYPE=DELETE
CM032587.1	1687368	.	T	TA	100	*	TYPE=INSERT
CM032587.1	1687380	.	T	TT	100	*	TYPE=INSERT
CM032587.1	1687425	.	C	A	100	*	TYPE=SUBSTITUTE
CM032587.1	1687428	.	T	C	100	*	TYPE=SUBSTITUTE
CM032587.1	1687446	.	C	CC	100	*	TYPE=INSERT
CM032587.1	1687502	.	T	C	100	*	TYPE=SUBSTITUTE
CM032587.1	1687504	.	GT	G	100	*	TYPE=DELETE
CM032587.1	1687524	.	c	ct	100	*	TYPE=INSERT
CM032587.1	1687534	.	A	c	100	*	TYPE=SUBSTITUTE
CM032587.1	1687563	.	C	CTGT	100	*	TYPE=INSERT
CM032587.1	1687567	.	C	CTACATTCT	100	*	TYPE=INSERT
CM032587.1	1687577	.	C	G	100	*	TYPE=SUBSTITUTE
CM032587.1	1687584	.	T	G	100	*	TYPE=SUBSTITUTE
CM032587.1	1687594	.	T	C	100	*	TYPE=SUBSTITUTE
CM032587.1	1687609	.	G	A	100	*	TYPE=SUBSTITUTE
CM032587.1	1687610	.	T	G	100	*	TYPE=SUBSTITUTE
CM032587.1	1687726	.	T	TC	100	*	TYPE=INSERT
CM032587.1	1687738	.	CC	C	100	*	TYPE=DELETE
CM032587.1	1687757	.	G	A	100	*	TYPE=SUBSTITUTE
CM032587.1	1687758	.	A	G	100	*	TYPE=SUBSTITUTE
CM032587.1	1687759	.	G	A	100	*	TYPE=SUBSTITUTE
CM032587.1	1687803	.	T	C	100	*	TYPE=SUBSTITUTE
CM032587.1	1687811	.	TCAAT	T	100	*	TYPE=DELETE
CM032587.1	1687829	.	a	atttttt	100	*	TYPE=INSERT
CM032587.1	1687838	.	AT	A	100	*	TYPE=DELETE
CM032587.1	1687842	.	A	G	100	*	TYPE=SUBSTITUTE
CM032587.1	1687843	.	A	AGA	100	*	TYPE=INSERT

CM032587.1	1687933	.	C	A	100	*	TYPE=SUBSTITUTE
CM032587.1	1687934	.	T	TG	100	*	TYPE=INSERT
CM032587.1	1687974	.	T	c	100	*	TYPE=SUBSTITUTE
CM032587.1	1687975	.	A	g	100	*	TYPE=SUBSTITUTE
CM032587.1	1687977	.	G	c	100	*	TYPE=SUBSTITUTE
CM032587.1	1688006	.	A	G	100	*	TYPE=SUBSTITUTE
CM032587.1	1688007	.	G	GG	100	*	TYPE=INSERT
CM032587.1	1688046	.	C	G	100	*	TYPE=SUBSTITUTE
CM032587.1	1688049	.	A	G	100	*	TYPE=SUBSTITUTE
CM032587.1	1688050	.	C	CG	100	*	TYPE=INSERT
CM032587.1	1688100	.	T	C	100	*	TYPE=SUBSTITUTE
CM032587.1	1688101	.	G	A	100	*	TYPE=SUBSTITUTE
CM032587.1	1688136	.	A	g	100	*	TYPE=SUBSTITUTE
CM032587.1	1688138	.	G	a	100	*	TYPE=SUBSTITUTE
CM032587.1	1688139	.	A	g	100	*	TYPE=SUBSTITUTE
CM032587.1	1688181	.	GA	G	100	*	TYPE=DELETE
CM032587.1	1688196	.	G	A	100	*	TYPE=SUBSTITUTE
CM032587.1	1688216	.	C	T	100	*	TYPE=SUBSTITUTE
CM032587.1	1688218	.	GG	G	100	*	TYPE=DELETE
CM032587.1	1688258	.	T	A	100	*	TYPE=SUBSTITUTE
CM032587.1	1688294	.	A	C	100	*	TYPE=SUBSTITUTE
CM032587.1	1688297	.	CTA	C	100	*	TYPE=DELETE
CM032587.1	1688300	.	C	T	100	*	TYPE=SUBSTITUTE
CM032587.1	1688301	.	C	T	100	*	TYPE=SUBSTITUTE
CM032587.1	1688304	.	T	TC	100	*	TYPE=INSERT
CM032587.1	1688305	.	A	C	100	*	TYPE=SUBSTITUTE
CM032587.1	1688307	.	C	T	100	*	TYPE=SUBSTITUTE
CM032587.1	1688308	.	T	G	100	*	TYPE=SUBSTITUTE
CM032587.1	1688309	.	C	T	100	*	TYPE=SUBSTITUTE
CM032587.1	1688328	.	AA	A	100	*	TYPE=DELETE
CM032587.1	1688361	.	G	A	100	*	TYPE=SUBSTITUTE
CM032587.1	1688457	.	G	gag	100	*	TYPE=INSERT
CM032587.1	1688484	.	T	C	100	*	TYPE=SUBSTITUTE
CM032587.1	1688492	.	GGTACTC	G	100	*	TYPE=DELETE
CM032587.1	1688497	.	A	G	100	*	TYPE=SUBSTITUTE
CM032587.1	1688504	.	A	G	100	*	TYPE=SUBSTITUTE
CM032587.1	1688508	.	A	T	100	*	TYPE=SUBSTITUTE
CM032587.1	1688521	.	T	C	100	*	TYPE=SUBSTITUTE
CM032587.1	1688522	.	G	A	100	*	TYPE=SUBSTITUTE
CM032587.1	1688524	.	C	G	100	*	TYPE=SUBSTITUTE
CM032587.1	1688525	.	G	A	100	*	TYPE=SUBSTITUTE
CM032587.1	1688527	.	A	AACG	100	*	TYPE=INSERT
CM032587.1	1688564	.	A	G	100	*	TYPE=SUBSTITUTE
CM032587.1	1688565	.	G	C	100	*	TYPE=SUBSTITUTE
CM032587.1	1688574	.	GT	G	100	*	TYPE=DELETE
CM032587.1	1688579	.	G	A	100	*	TYPE=SUBSTITUTE
CM032587.1	1688581	.	T	C	100	*	TYPE=SUBSTITUTE
CM032587.1	1688584	.	T	G	100	*	TYPE=SUBSTITUTE
CM032587.1	1688588	.	CCTT	C	100	*	TYPE=DELETE
CM032587.1	1688601	.	G	C	100	*	TYPE=SUBSTITUTE
CM032587.1	1688604	.	C	T	100	*	TYPE=SUBSTITUTE
CM032587.1	1688610	.	A	G	100	*	TYPE=SUBSTITUTE
CM032587.1	1688613	.	T	TC	100	*	TYPE=INSERT
CM032587.1	1688685	.	T	c	100	*	TYPE=SUBSTITUTE
CM032587.1	1688697	.	G	C	100	*	TYPE=SUBSTITUTE
CM032587.1	1688757	.	A	T	100	*	TYPE=SUBSTITUTE
CM032587.1	1688771	.	G	A	100	*	TYPE=SUBSTITUTE
CM032587.1	1688826	.	TT	T	100	*	TYPE=DELETE
CM032587.1	1688877	.	A	G	100	*	TYPE=SUBSTITUTE
CM032587.1	1688880	.	G	GA	100	*	TYPE=INSERT
CM032587.1	1688894	.	T	g	100	*	TYPE=SUBSTITUTE
CM032587.1	1688925	.	AA	A	100	*	TYPE=DELETE
CM032587.1	1688950	.	T	G	100	*	TYPE=SUBSTITUTE
CM032587.1	1688958	.	T	TT	100	*	TYPE=INSERT

CM032587.1	1689034	.	A	G	100	*	TYPE=SUBSTITUTE
CM032587.1	1689036	.	GA	G	100	*	TYPE=DELETE
CM032587.1	1689043	.	C	T	100	*	TYPE=SUBSTITUTE
CM032587.1	1689064	.	CC	C	100	*	TYPE=DELETE
CM032587.1	1689117	.	T	tt	100	*	TYPE=INSERT
CM032587.1	1689171	.	GG	G	100	*	TYPE=DELETE
CM032587.1	1689189	.	T	C	100	*	TYPE=SUBSTITUTE
CM032587.1	1689191	.	C	A	100	*	TYPE=SUBSTITUTE
CM032587.1	1689250	.	T	C	100	*	TYPE=SUBSTITUTE
CM032587.1	1689257	.	G	GA	100	*	TYPE=INSERT
CM032587.1	1689258	.	C	T	100	*	TYPE=SUBSTITUTE
CM032587.1	1689263	.	T	C	100	*	TYPE=SUBSTITUTE
CM032587.1	1689280	.	t	tcttccttg	100	*	TYPE=INSERT
CM032587.1	1689291	.	CT	C	100	*	TYPE=DELETE
CM032587.1	1689326	.	A	t	100	*	TYPE=SUBSTITUTE
CM032587.1	1689354	.	A	c	100	*	TYPE=SUBSTITUTE
CM032587.1	1689357	.	c	cgtc	100	*	TYPE=INSERT
CM032587.1	1689385	.	A	T	100	*	TYPE=SUBSTITUTE
CM032587.1	1689389	.	TCGA	T	100	*	TYPE=DELETE
CM032587.1	1689393	.	C	t	100	*	TYPE=SUBSTITUTE
CM032587.1	1689567	.	C	T	100	*	TYPE=SUBSTITUTE
CM032587.1	1689708	.	G	A	100	*	TYPE=SUBSTITUTE
CM032587.1	1689762	.	G	C	100	*	TYPE=SUBSTITUTE
CM032587.1	1689763	.	G	C	100	*	TYPE=SUBSTITUTE
CM032587.1	1689764	.	G	C	100	*	TYPE=SUBSTITUTE
CM032587.1	1689812	.	T	G	100	*	TYPE=SUBSTITUTE
CM032587.1	1689813	.	G	A	100	*	TYPE=SUBSTITUTE
CM032587.1	1689855	.	G	A	100	*	TYPE=SUBSTITUTE
CM032587.1	1689857	.	C	A	100	*	TYPE=SUBSTITUTE
CM032587.1	1689864	.	A	G	100	*	TYPE=SUBSTITUTE
CM032587.1	1689900	.	G	A	100	*	TYPE=SUBSTITUTE
CM032587.1	1689942	.	A	C	100	*	TYPE=SUBSTITUTE
CM032587.1	1689948	.	C	T	100	*	TYPE=SUBSTITUTE
CM032587.1	1690059	.	C	T	100	*	TYPE=SUBSTITUTE
CM032587.1	1690125	.	G	C	100	*	TYPE=SUBSTITUTE
CM032587.1	1690142	.	T	A	100	*	TYPE=SUBSTITUTE
CM032587.1	1690152	.	A	G	100	*	TYPE=SUBSTITUTE
CM032587.1	1690158	.	G	A	100	*	TYPE=SUBSTITUTE
CM032587.1	1690164	.	A	G	100	*	TYPE=SUBSTITUTE
CM032587.1	1690179	.	T	C	100	*	TYPE=SUBSTITUTE
CM032587.1	1690197	.	C	T	100	*	TYPE=SUBSTITUTE
CM032587.1	1690214	.	A	C	100	*	TYPE=SUBSTITUTE
CM032587.1	1690215	.	G	C	100	*	TYPE=SUBSTITUTE
CM032587.1	1690248	.	A	G	100	*	TYPE=SUBSTITUTE
CM032587.1	1690308	.	G	A	100	*	TYPE=SUBSTITUTE
CM032587.1	1690332	.	C	T	100	*	TYPE=SUBSTITUTE
CM032587.1	1690341	.	G	A	100	*	TYPE=SUBSTITUTE
CM032587.1	1690410	.	G	A	100	*	TYPE=SUBSTITUTE
CM032587.1	1690431	.	C	T	100	*	TYPE=SUBSTITUTE
CM032587.1	1690443	.	C	T	100	*	TYPE=SUBSTITUTE
CM032587.1	1690463	.	A	T	100	*	TYPE=SUBSTITUTE
CM032587.1	1690476	.	T	C	100	*	TYPE=SUBSTITUTE
CM032587.1	1690497	.	C	A	100	*	TYPE=SUBSTITUTE
CM032587.1	1690517	.	C	T	100	*	TYPE=SUBSTITUTE
CM032587.1	1690599	.	C	T	100	*	TYPE=SUBSTITUTE
CM032587.1	1690623	.	C	T	100	*	TYPE=SUBSTITUTE
CM032587.1	1690644	.	A	G	100	*	TYPE=SUBSTITUTE
CM032587.1	1690661	.	A	G	100	*	TYPE=SUBSTITUTE
CM032587.1	1690663	.	A	G	100	*	TYPE=SUBSTITUTE
CM032587.1	1690670	.	A	G	100	*	TYPE=SUBSTITUTE
CM032587.1	1690697	.	A	G	100	*	TYPE=SUBSTITUTE
CM032587.1	1690725	.	C	T	100	*	TYPE=SUBSTITUTE
CM032587.1	1690806	.	C	T	100	*	TYPE=SUBSTITUTE
CM032587.1	1690814	.	T	C	100	*	TYPE=SUBSTITUTE

CM032587.1	1690815	.	C	T	100	*	TYPE=SUBSTITUTE
CM032587.1	1690821	.	T	C	100	*	TYPE=SUBSTITUTE
CM032587.1	1690844	.	GCA	G	100	*	TYPE=DELETE
CM032587.1	1690847	.	C	T	100	*	TYPE=SUBSTITUTE
CM032587.1	1690850	.	ACA	A	100	*	TYPE=DELETE
CM032587.1	1690880	.	T	g	100	*	TYPE=SUBSTITUTE
CM032587.1	1690884	.	A	g	100	*	TYPE=SUBSTITUTE
CM032587.1	1690887	.	T	C	100	*	TYPE=SUBSTITUTE
CM032587.1	1690889	.	A	G	100	*	TYPE=SUBSTITUTE
CM032587.1	1690899	.	GA	G	100	*	TYPE=DELETE
CM032587.1	1690900	.	C	A	100	*	TYPE=SUBSTITUTE
CM032587.1	1690910	.	A	g	100	*	TYPE=SUBSTITUTE
CM032587.1	1690918	.	a	aagagagagaaagag	100	*	TYPE=INSERT
CM032587.1	1690925	.	N	NNa	100	*	TYPE=INSERT
CM032587.1	1690934	.	T	G	100	*	TYPE=SUBSTITUTE
CM032587.1	1690940	.	G	A	100	*	TYPE=SUBSTITUTE
CM032587.1	1690946	.	A	G	100	*	TYPE=SUBSTITUTE
CM032587.1	1690947	.	C	G	100	*	TYPE=SUBSTITUTE
CM032587.1	1690948	.	T	C	100	*	TYPE=SUBSTITUTE
CM032587.1	1691172	.	G	C	100	*	TYPE=SUBSTITUTE
CM032587.1	1691174	.	G	A	100	*	TYPE=SUBSTITUTE
CM032587.1	1691186	.	g	gaccaac	100	*	TYPE=INSERT
CM032587.1	1691230	.	AGAGGGCCAACAAG	A	100	*	TYPE=DELETE
CM032587.1	1691241	.	AA	A	100	*	TYPE=DELETE
CM032587.1	1691336	.	A	aa	100	*	TYPE=INSERT
CM032587.1	1691525	.	A	G	100	*	TYPE=SUBSTITUTE
CM032587.1	1691531	.	AGT	A	100	*	TYPE=DELETE
CM032587.1	1691545	.	G	A	100	*	TYPE=SUBSTITUTE
CM032587.1	1691551	.	T	C	100	*	TYPE=SUBSTITUTE
CM032587.1	1691578	.	TGTCTGG	T	100	*	TYPE=DELETE
CM032587.1	1691587	.	T	C	100	*	TYPE=SUBSTITUTE
CM032587.1	1691599	.	C	T	100	*	TYPE=SUBSTITUTE
CM032587.1	1691612	.	C	T	100	*	TYPE=SUBSTITUTE
CM032587.1	1691617	.	T	A	100	*	TYPE=SUBSTITUTE
CM032587.1	1691618	.	A	G	100	*	TYPE=SUBSTITUTE
CM032587.1	1691620	.	A	G	100	*	TYPE=SUBSTITUTE
CM032587.1	1691633	.	A	G	100	*	TYPE=SUBSTITUTE
CM032587.1	1691635	.	T	A	100	*	TYPE=SUBSTITUTE
CM032587.1	1691639	.	T	C	100	*	TYPE=SUBSTITUTE
CM032587.1	1691643	.	TC	T	100	*	TYPE=DELETE
CM032587.1	1691673	.	G	A	100	*	TYPE=SUBSTITUTE
CM032587.1	1691679	.	A	G	100	*	TYPE=SUBSTITUTE
CM032587.1	1691690	.	C	T	100	*	TYPE=SUBSTITUTE
CM032587.1	1691695	.	G	A	100	*	TYPE=SUBSTITUTE
CM032587.1	1691714	.	G	A	100	*	TYPE=SUBSTITUTE
CM032587.1	1691722	.	T	C	100	*	TYPE=SUBSTITUTE
CM032587.1	1691723	.	T	C	100	*	TYPE=SUBSTITUTE
CM032587.1	1691732	.	A	G	100	*	TYPE=SUBSTITUTE
CM032587.1	1691734	.	G	A	100	*	TYPE=SUBSTITUTE
CM032587.1	1691751	.	TAAGA	T	100	*	TYPE=DELETE
CM032587.1	1691753	.	C	T	100	*	TYPE=SUBSTITUTE
CM032587.1	1691803	.	G	t	100	*	TYPE=SUBSTITUTE
CM032587.1	1691809	.	C	T	100	*	TYPE=SUBSTITUTE
CM032587.1	1691813	.	G	A	100	*	TYPE=SUBSTITUTE
CM032587.1	1691814	.	A	G	100	*	TYPE=SUBSTITUTE
CM032587.1	1691823	.	A	T	100	*	TYPE=SUBSTITUTE
CM032587.1	1691825	.	T	C	100	*	TYPE=SUBSTITUTE
CM032587.1	1691829	.	A	C	100	*	TYPE=SUBSTITUTE
CM032587.1	1691835	.	C	A	100	*	TYPE=SUBSTITUTE
CM032587.1	1691837	.	A	G	100	*	TYPE=SUBSTITUTE
CM032587.1	1691838	.	C	G	100	*	TYPE=SUBSTITUTE
CM032587.1	1691846	.	C	T	100	*	TYPE=SUBSTITUTE
CM032587.1	1691852	.	A	G	100	*	TYPE=SUBSTITUTE
CM032587.1	1691855	.	A	G	100	*	TYPE=SUBSTITUTE

CM032587.1	1691861	.	T	C	100	*	TYPE=SUBSTITUTE
CM032587.1	1691862	.	G	A	100	*	TYPE=SUBSTITUTE
CM032587.1	1691864	.	T	C	100	*	TYPE=SUBSTITUTE
CM032587.1	1691865	.	T	C	100	*	TYPE=SUBSTITUTE
CM032587.1	1691866	.	G	A	100	*	TYPE=SUBSTITUTE
CM032587.1	1691868	.	A	T	100	*	TYPE=SUBSTITUTE
CM032587.1	1691869	.	G	A	100	*	TYPE=SUBSTITUTE
CM032587.1	1691884	.	T	A	100	*	TYPE=SUBSTITUTE
CM032587.1	1691922	.	C	T	100	*	TYPE=SUBSTITUTE
CM032587.1	1691942	.	C	A	100	*	TYPE=SUBSTITUTE
CM032587.1	1691954	.	C	T	100	*	TYPE=SUBSTITUTE
CM032587.1	1691981	.	T	C	100	*	TYPE=SUBSTITUTE
CM032587.1	1692204	.	C	G	100	*	TYPE=SUBSTITUTE
CM032587.1	1692209	.	G	A	100	*	TYPE=SUBSTITUTE
CM032587.1	1692212	.	T	C	100	*	TYPE=SUBSTITUTE
CM032587.1	1692215	.	CC	C	100	*	TYPE=DELETE
CM032587.1	1692231	.	C	t	100	*	TYPE=SUBSTITUTE
CM032587.1	1692264	.	C	T	100	*	TYPE=SUBSTITUTE
CM032587.1	1692294	.	C	T	100	*	TYPE=SUBSTITUTE
CM032587.1	1692306	.	T	C	100	*	TYPE=SUBSTITUTE
CM032587.1	1692324	.	A	G	100	*	TYPE=SUBSTITUTE
CM032587.1	1692343	.	G	GCAT	100	*	TYPE=INSERT
CM032587.1	1692345	.	T	G	100	*	TYPE=SUBSTITUTE
CM032587.1	1692349	.	T	C	100	*	TYPE=SUBSTITUTE
CM032587.1	1692353	.	G	A	100	*	TYPE=SUBSTITUTE
CM032587.1	1692363	.	T	C	100	*	TYPE=SUBSTITUTE
CM032587.1	1692380	.	C	G	100	*	TYPE=SUBSTITUTE
CM032587.1	1692387	.	A	G	100	*	TYPE=SUBSTITUTE
CM032587.1	1692396	.	C	T	100	*	TYPE=SUBSTITUTE
CM032587.1	1692402	.	G	A	100	*	TYPE=SUBSTITUTE
CM032587.1	1692404	.	T	C	100	*	TYPE=SUBSTITUTE
CM032587.1	1692405	.	G	C	100	*	TYPE=SUBSTITUTE
CM032587.1	1692408	.	A	G	100	*	TYPE=SUBSTITUTE
CM032587.1	1692412	.	T	C	100	*	TYPE=SUBSTITUTE
CM032587.1	1692443	.	T	A	100	*	TYPE=SUBSTITUTE
CM032587.1	1692445	.	A	G	100	*	TYPE=SUBSTITUTE
CM032587.1	1692463	.	T	C	100	*	TYPE=SUBSTITUTE
CM032587.1	1692466	.	G	T	100	*	TYPE=SUBSTITUTE
CM032587.1	1692467	.	G	A	100	*	TYPE=SUBSTITUTE
CM032587.1	1692483	.	C	T	100	*	TYPE=SUBSTITUTE
CM032587.1	1692494	.	A	G	100	*	TYPE=SUBSTITUTE
CM032587.1	1692567	.	C	T	100	*	TYPE=SUBSTITUTE
CM032587.1	1692693	.	A	G	100	*	TYPE=SUBSTITUTE
CM032587.1	1692708	.	G	A	100	*	TYPE=SUBSTITUTE
CM032587.1	1692716	.	T	G	100	*	TYPE=SUBSTITUTE
CM032587.1	1692731	.	T	C	100	*	TYPE=SUBSTITUTE
CM032587.1	1692774	.	T	C	100	*	TYPE=SUBSTITUTE
CM032587.1	1692779	.	C	T	100	*	TYPE=SUBSTITUTE
CM032587.1	1692873	.	T	C	100	*	TYPE=SUBSTITUTE
CM032587.1	1692882	.	T	G	100	*	TYPE=SUBSTITUTE
CM032587.1	1692890	.	T	C	100	*	TYPE=SUBSTITUTE
CM032587.1	1692894	.	A	G	100	*	TYPE=SUBSTITUTE
CM032587.1	1692897	.	C	T	100	*	TYPE=SUBSTITUTE
CM032587.1	1692927	.	T	C	100	*	TYPE=SUBSTITUTE
CM032587.1	1692958	.	C	CA	100	*	TYPE=INSERT
CM032587.1	1692966	.	G	T	100	*	TYPE=SUBSTITUTE
CM032587.1	1692968	.	T	C	100	*	TYPE=SUBSTITUTE
CM032587.1	1692975	.	A	C	100	*	TYPE=SUBSTITUTE
CM032587.1	1692982	.	C	T	100	*	TYPE=SUBSTITUTE
CM032587.1	1692983	.	A	T	100	*	TYPE=SUBSTITUTE
CM032587.1	1692989	.	A	G	100	*	TYPE=SUBSTITUTE
CM032587.1	1692994	.	C	T	100	*	TYPE=SUBSTITUTE
CM032587.1	1692996	.	C	T	100	*	TYPE=SUBSTITUTE
CM032587.1	1693019	.	G	A	100	*	TYPE=SUBSTITUTE

CM032587.1	1693022	.	G	A	100	*	TYPE=SUBSTITUTE
CM032587.1	1693034	.	A	G	100	*	TYPE=SUBSTITUTE
CM032587.1	1693051	.	C	T	100	*	TYPE=SUBSTITUTE
CM032587.1	1693058	.	C	T	100	*	TYPE=SUBSTITUTE
CM032587.1	1693067	.	T	C	100	*	TYPE=SUBSTITUTE
CM032587.1	1693070	.	T	C	100	*	TYPE=SUBSTITUTE
CM032587.1	1693109	.	G	A	100	*	TYPE=SUBSTITUTE
CM032587.1	1693127	.	A	G	100	*	TYPE=SUBSTITUTE
CM032587.1	1693135	.	T	C	100	*	TYPE=SUBSTITUTE
CM032587.1	1693151	.	C	T	100	*	TYPE=SUBSTITUTE
CM032587.1	1693190	.	C	T	100	*	TYPE=SUBSTITUTE
CM032587.1	1693221	.	A	c	100	*	TYPE=SUBSTITUTE
CM032587.1	1693222	.	A	t	100	*	TYPE=SUBSTITUTE
CM032587.1	1693232	.	G	A	100	*	TYPE=SUBSTITUTE
CM032587.1	1693233	.	T	C	100	*	TYPE=SUBSTITUTE
CM032587.1	1693257	.	C	T	100	*	TYPE=SUBSTITUTE
CM032587.1	1693261	.	G	A	100	*	TYPE=SUBSTITUTE
CM032587.1	1693262	.	A	G	100	*	TYPE=SUBSTITUTE
CM032587.1	1693294	.	T	C	100	*	TYPE=SUBSTITUTE
CM032587.1	1693327	.	T	C	100	*	TYPE=SUBSTITUTE
CM032587.1	1693328	.	T	C	100	*	TYPE=SUBSTITUTE
CM032587.1	1693424	.	A	G	100	*	TYPE=SUBSTITUTE
CM032587.1	1693439	.	T	C	100	*	TYPE=SUBSTITUTE
CM032587.1	1693443	.	T	C	100	*	TYPE=SUBSTITUTE
CM032587.1	1693459	.	C	T	100	*	TYPE=SUBSTITUTE
CM032587.1	1693504	.	G	A	100	*	TYPE=SUBSTITUTE
CM032587.1	1693514	.	T	A	100	*	TYPE=SUBSTITUTE
CM032587.1	1693528	.	A	G	100	*	TYPE=SUBSTITUTE
CM032587.1	1693532	.	C	T	100	*	TYPE=SUBSTITUTE
CM032587.1	1693551	.	T	C	100	*	TYPE=SUBSTITUTE
CM032587.1	1693553	.	A	C	100	*	TYPE=SUBSTITUTE
CM032587.1	1693555	.	A	T	100	*	TYPE=SUBSTITUTE
CM032587.1	1693556	.	C	T	100	*	TYPE=SUBSTITUTE
CM032587.1	1693563	.	C	T	100	*	TYPE=SUBSTITUTE
CM032587.1	1693586	.	A	G	100	*	TYPE=SUBSTITUTE
CM032587.1	1693589	.	T	C	100	*	TYPE=SUBSTITUTE
CM032587.1	1693594	.	T	C	100	*	TYPE=SUBSTITUTE
CM032587.1	1693607	.	G	A	100	*	TYPE=SUBSTITUTE
CM032587.1	1693628	.	CCACTAGA	C	100	*	TYPE=DELETE
CM032587.1	1693645	.	T	C	100	*	TYPE=SUBSTITUTE
CM032587.1	1693647	.	A	C	100	*	TYPE=SUBSTITUTE
CM032587.1	1693649	.	G	A	100	*	TYPE=SUBSTITUTE
CM032587.1	1693652	.	A	G	100	*	TYPE=SUBSTITUTE
CM032587.1	1693675	.	C	T	100	*	TYPE=SUBSTITUTE
CM032587.1	1693687	.	T	C	100	*	TYPE=SUBSTITUTE
CM032587.1	1693694	.	G	A	100	*	TYPE=SUBSTITUTE
CM032587.1	1693709	.	A	G	100	*	TYPE=SUBSTITUTE
CM032587.1	1693714	.	A	g	100	*	TYPE=SUBSTITUTE
CM032587.1	1693716	.	A	g	100	*	TYPE=SUBSTITUTE
CM032587.1	1693721	.	A	g	100	*	TYPE=SUBSTITUTE
CM032587.1	1693729	.	C	t	100	*	TYPE=SUBSTITUTE
CM032587.1	1693744	.	G	t	100	*	TYPE=SUBSTITUTE
CM032587.1	1693747	.	C	g	100	*	TYPE=SUBSTITUTE
CM032587.1	1693759	.	C	t	100	*	TYPE=SUBSTITUTE
CM032587.1	1693765	.	C	t	100	*	TYPE=SUBSTITUTE
CM032587.1	1693771	.	T	c	100	*	TYPE=SUBSTITUTE
CM032587.1	1693786	.	T	C	100	*	TYPE=SUBSTITUTE
CM032587.1	1693801	.	C	T	100	*	TYPE=SUBSTITUTE
CM032587.1	1693813	.	A	G	100	*	TYPE=SUBSTITUTE
CM032587.1	1693834	.	G	A	100	*	TYPE=SUBSTITUTE
CM032587.1	1693837	.	G	C	100	*	TYPE=SUBSTITUTE
CM032587.1	1693870	.	T	c	100	*	TYPE=SUBSTITUTE
CM032587.1	1693957	.	G	a	100	*	TYPE=SUBSTITUTE
CM032587.1	1693990	.	G	C	100	*	TYPE=SUBSTITUTE

CM032587.1	1693999	.	T	C	100	*	TYPE=SUBSTITUTE
CM032587.1	1694035	.	T	C	100	*	TYPE=SUBSTITUTE
CM032587.1	1694053	.	C	A	100	*	TYPE=SUBSTITUTE
CM032587.1	1694062	.	T	A	100	*	TYPE=SUBSTITUTE
CM032587.1	1694065	.	C	G	100	*	TYPE=SUBSTITUTE
CM032587.1	1694167	.	G	A	100	*	TYPE=SUBSTITUTE
CM032587.1	1694194	.	A	G	100	*	TYPE=SUBSTITUTE
CM032587.1	1694224	.	A	G	100	*	TYPE=SUBSTITUTE
CM032587.1	1694242	.	A	G	100	*	TYPE=SUBSTITUTE
CM032587.1	1694269	.	T	C	100	*	TYPE=SUBSTITUTE
CM032587.1	1694272	.	G	A	100	*	TYPE=SUBSTITUTE
CM032587.1	1694302	.	A	G	100	*	TYPE=SUBSTITUTE
CM032587.1	1694368	.	C	T	100	*	TYPE=SUBSTITUTE
CM032587.1	1694371	.	A	G	100	*	TYPE=SUBSTITUTE
CM032587.1	1694374	.	C	G	100	*	TYPE=SUBSTITUTE
CM032587.1	1694377	.	A	C	100	*	TYPE=SUBSTITUTE
CM032587.1	1694379	.	T	C	100	*	TYPE=SUBSTITUTE
CM032587.1	1694383	.	G	A	100	*	TYPE=SUBSTITUTE
CM032587.1	1694389	.	G	T	100	*	TYPE=SUBSTITUTE
CM032587.1	1694437	.	A	C	100	*	TYPE=SUBSTITUTE
CM032587.1	1694455	.	G	A	100	*	TYPE=SUBSTITUTE
CM032587.1	1694461	.	C	T	100	*	TYPE=SUBSTITUTE
CM032587.1	1694473	.	C	T	100	*	TYPE=SUBSTITUTE
CM032587.1	1694479	.	G	A	100	*	TYPE=SUBSTITUTE
CM032587.1	1694530	.	A	G	100	*	TYPE=SUBSTITUTE
CM032587.1	1694563	.	T	C	100	*	TYPE=SUBSTITUTE
CM032587.1	1694592	.	T	C	100	*	TYPE=SUBSTITUTE
CM032587.1	1694593	.	G	A	100	*	TYPE=SUBSTITUTE
CM032587.1	1694596	.	A	T	100	*	TYPE=SUBSTITUTE
CM032587.1	1694629	.	G	A	100	*	TYPE=SUBSTITUTE
CM032587.1	1694668	.	T	C	100	*	TYPE=SUBSTITUTE
CM032587.1	1694686	.	C	T	100	*	TYPE=SUBSTITUTE
CM032587.1	1694689	.	A	G	100	*	TYPE=SUBSTITUTE
CM032587.1	1694699	.	T	C	100	*	TYPE=SUBSTITUTE
CM032587.1	1694716	.	A	G	100	*	TYPE=SUBSTITUTE
CM032587.1	1694725	.	C	T	100	*	TYPE=SUBSTITUTE
CM032587.1	1694746	.	G	A	100	*	TYPE=SUBSTITUTE
CM032587.1	1694785	.	C	T	100	*	TYPE=SUBSTITUTE
CM032587.1	1694803	.	T	C	100	*	TYPE=SUBSTITUTE
CM032587.1	1694857	.	T	C	100	*	TYPE=SUBSTITUTE
CM032587.1	1694901	.	C	g	100	*	TYPE=SUBSTITUTE
CM032587.1	1694925	.	A	G	100	*	TYPE=SUBSTITUTE
CM032587.1	1694934	.	A	T	100	*	TYPE=SUBSTITUTE
CM032587.1	1694937	.	G	A	100	*	TYPE=SUBSTITUTE
CM032587.1	1694943	.	C	T	100	*	TYPE=SUBSTITUTE
CM032587.1	1694951	.	C	T	100	*	TYPE=SUBSTITUTE
CM032587.1	1694977	.	T	C	100	*	TYPE=SUBSTITUTE
CM032587.1	1694983	.	G	A	100	*	TYPE=SUBSTITUTE
CM032587.1	1695001	.	A	G	100	*	TYPE=SUBSTITUTE
CM032587.1	1695004	.	G	A	100	*	TYPE=SUBSTITUTE
CM032587.1	1695070	.	C	T	100	*	TYPE=SUBSTITUTE
CM032587.1	1695091	.	C	T	100	*	TYPE=SUBSTITUTE
CM032587.1	1695166	.	C	CTCAGAAAAT	100	*	TYPE=INSERT
CM032587.1	1695177	.	T	C	100	*	TYPE=SUBSTITUTE
CM032587.1	1695181	.	C	T	100	*	TYPE=SUBSTITUTE
CM032587.1	1695189	.	G	A	100	*	TYPE=SUBSTITUTE
CM032587.1	1695208	.	T	C	100	*	TYPE=SUBSTITUTE
CM032587.1	1695221	.	A	G	100	*	TYPE=SUBSTITUTE
CM032587.1	1695272	.	AG	A	100	*	TYPE=DELETE
CM032587.1	1695292	.	G	A	100	*	TYPE=SUBSTITUTE
CM032587.1	1695310	.	C	A	100	*	TYPE=SUBSTITUTE
CM032587.1	1695313	.	C	T	100	*	TYPE=SUBSTITUTE
CM032587.1	1695334	.	A	C	100	*	TYPE=SUBSTITUTE
CM032587.1	1695361	.	G	A	100	*	TYPE=SUBSTITUTE

CM032587.1	1695376	.	T	C	100	*	TYPE=SUBSTITUTE
CM032587.1	1695387	.	T	G	100	*	TYPE=SUBSTITUTE
CM032587.1	1695396	.	ATGATTTG	A	100	*	TYPE=DELETE
CM032587.1	1695425	.	C	CA	100	*	TYPE=INSERT
CM032587.1	1695447	.	C	A	100	*	TYPE=SUBSTITUTE
CM032587.1	1695448	.	G	A	100	*	TYPE=SUBSTITUTE
CM032587.1	1695482	.	A	C	100	*	TYPE=SUBSTITUTE
CM032587.1	1695491	.	A	T	100	*	TYPE=SUBSTITUTE
CM032587.1	1695658	.	A	G	100	*	TYPE=SUBSTITUTE
CM032587.1	1695664	.	C	T	100	*	TYPE=SUBSTITUTE
CM032587.1	1695665	.	C	A	100	*	TYPE=SUBSTITUTE
CM032587.1	1695673	.	A	G	100	*	TYPE=SUBSTITUTE
CM032587.1	1695683	.	C	T	100	*	TYPE=SUBSTITUTE
CM032587.1	1695690	.	A	AAGGTATTG	100	*	TYPE=INSERT
CM032587.1	1695707	.	A	AT	100	*	TYPE=INSERT
CM032587.1	1695708	.	G	T	100	*	TYPE=SUBSTITUTE
CM032587.1	1695720	.	T	C	100	*	TYPE=SUBSTITUTE
CM032587.1	1695834	.	C	G	100	*	TYPE=SUBSTITUTE
CM032587.1	1695969	.	A	G	100	*	TYPE=SUBSTITUTE
CM032587.1	1696001	.	T	C	100	*	TYPE=SUBSTITUTE
CM032587.1	1696010	.	A	G	100	*	TYPE=SUBSTITUTE
CM032587.1	1696116	.	G	C	100	*	TYPE=SUBSTITUTE
CM032587.1	1696140	.	C	T	100	*	TYPE=SUBSTITUTE
CM032587.1	1696197	.	T	TAGCGAGCTGAGGACGG	100	*	TYPE=INSERT
CM032587.1	1696239	.	G	A	100	*	TYPE=SUBSTITUTE
CM032587.1	1696251	.	C	G	100	*	TYPE=SUBSTITUTE
CM032587.1	1696252	.	A	G	100	*	TYPE=SUBSTITUTE
CM032587.1	1696315	.	T	C	100	*	TYPE=SUBSTITUTE
CM032587.1	1696811	.	G	A	100	*	TYPE=SUBSTITUTE
CM032587.1	1697155	.	GA	G	100	*	TYPE=DELETE
CM032587.1	1697188	.	G	C	100	*	TYPE=SUBSTITUTE
CM032587.1	1697646	.	GGGAGA	G	100	*	TYPE=DELETE
CM032587.1	1697703	.	C	T	100	*	TYPE=SUBSTITUTE
CM032587.1	1697704	.	G	C	100	*	TYPE=SUBSTITUTE
CM032587.1	1697807	.	T	G	100	*	TYPE=SUBSTITUTE
CM032587.1	1698335	.	C	T	100	*	TYPE=SUBSTITUTE
CM032587.1	1698710	.	TTG	T	100	*	TYPE=DELETE
CM032587.1	1699033	.	CAC	C	100	*	TYPE=DELETE
CM032587.1	1699119	.	A	G	100	*	TYPE=SUBSTITUTE
CM032587.1	1699727	.	AAA	A	100	*	TYPE=DELETE
CM032587.1	1699812	.	AC	A	100	*	TYPE=DELETE
CM032587.1	1699828	.	T	a	100	*	TYPE=SUBSTITUTE
CM032587.1	1699830	.	G	a	100	*	TYPE=SUBSTITUTE
CM032587.1	1699836	.	G	a	100	*	TYPE=SUBSTITUTE
CM032587.1	1699845	.	a	ag	100	*	TYPE=INSERT
CM032587.1	1699847	.	G	a	100	*	TYPE=SUBSTITUTE
CM032587.1	1699849	.	G	a	100	*	TYPE=SUBSTITUTE
CM032587.1	1699858	.	T	a	100	*	TYPE=SUBSTITUTE
CM032587.1	1699860	.	a	aaaat	100	*	TYPE=INSERT
CM032587.1	1699872	.	A	C	100	*	TYPE=SUBSTITUTE
CM032587.1	1699873	.	A	G	100	*	TYPE=SUBSTITUTE
CM032587.1	1699876	.	C	A	100	*	TYPE=SUBSTITUTE
CM032587.1	1699881	.	GGGA	G	100	*	TYPE=DELETE
CM032587.1	1699882	.	T	A	100	*	TYPE=SUBSTITUTE
CM032587.1	1699896	.	ATCCCC	A	100	*	TYPE=DELETE
CM032587.1	1699899	.	G	A	100	*	TYPE=SUBSTITUTE
CM032587.1	1699909	.	T	C	100	*	TYPE=SUBSTITUTE
CM032587.1	1699913	.	AT	A	100	*	TYPE=DELETE
CM032587.1	1699915	.	T	C	100	*	TYPE=SUBSTITUTE
CM032587.1	1699924	.	T	C	100	*	TYPE=SUBSTITUTE
CM032587.1	1700079	.	AA	A	100	*	TYPE=DELETE
CM032587.1	1700119	.	g	ga	100	*	TYPE=INSERT
CM032587.1	1700123	.	G	a	100	*	TYPE=SUBSTITUTE
CM032587.1	1700143	.	C	g	100	*	TYPE=SUBSTITUTE

CM032587.1	1700149	.	G	a	100	*	TYPE=SUBSTITUTE
CM032587.1	1700155	.	CC	C	100	*	TYPE=DELETE
CM032587.1	1700198	.	T	TG	100	*	TYPE=INSERT
CM032587.1	1700209	.	A	AA	100	*	TYPE=INSERT
CM032587.1	1700275	.	T	a	100	*	TYPE=SUBSTITUTE
CM032587.1	1700294	.	T	G	100	*	TYPE=SUBSTITUTE
CM032587.1	1700308	.	T	C	100	*	TYPE=SUBSTITUTE
CM032587.1	1700312	.	A	G	100	*	TYPE=SUBSTITUTE
CM032587.1	1700318	.	C	A	100	*	TYPE=SUBSTITUTE
CM032587.1	1700327	.	T	C	100	*	TYPE=SUBSTITUTE
CM032587.1	1700328	.	A	C	100	*	TYPE=SUBSTITUTE
CM032587.1	1700329	.	G	A	100	*	TYPE=SUBSTITUTE
CM032587.1	1700334	.	C	T	100	*	TYPE=SUBSTITUTE
CM032587.1	1700338	.	C	T	100	*	TYPE=SUBSTITUTE
CM032587.1	1700341	.	G	T	100	*	TYPE=SUBSTITUTE
CM032587.1	1700354	.	T	C	100	*	TYPE=SUBSTITUTE
CM032587.1	1700356	.	C	G	100	*	TYPE=SUBSTITUTE
CM032587.1	1700361	.	G	GCTA	100	*	TYPE=INSERT
CM032587.1	1700396	.	C	T	100	*	TYPE=SUBSTITUTE
CM032587.1	1700441	.	C	T	100	*	TYPE=SUBSTITUTE
CM032587.1	1700447	.	A	AT	100	*	TYPE=INSERT
CM032587.1	1700449	.	A	G	100	*	TYPE=SUBSTITUTE
CM032587.1	1700478	.	A	T	100	*	TYPE=SUBSTITUTE
CM032587.1	1700479	.	A	G	100	*	TYPE=SUBSTITUTE
CM032587.1	1700482	.	T	A	100	*	TYPE=SUBSTITUTE
CM032587.1	1700483	.	C	CA	100	*	TYPE=INSERT
CM032587.1	1700490	.	A	G	100	*	TYPE=SUBSTITUTE
CM032587.1	1700512	.	TA	T	100	*	TYPE=DELETE
CM032587.1	1700584	.	C	T	100	*	TYPE=SUBSTITUTE
CM032587.1	1700594	.	C	T	100	*	TYPE=SUBSTITUTE
CM032587.1	1700608	.	A	C	100	*	TYPE=SUBSTITUTE
CM032587.1	1700672	.	T	A	100	*	TYPE=SUBSTITUTE
CM032587.1	1700699	.	A	G	100	*	TYPE=SUBSTITUTE
CM032587.1	1700705	.	C	T	100	*	TYPE=SUBSTITUTE
CM032587.1	1700765	.	C	T	100	*	TYPE=SUBSTITUTE
CM032587.1	1700838	.	CCC	C	100	*	TYPE=DELETE
CM032587.1	1700858	.	T	C	100	*	TYPE=SUBSTITUTE
CM032587.1	1700893	.	C	CNN	100	*	TYPE=INSERT
CM032587.1	1700939	.	G	A	100	*	TYPE=SUBSTITUTE
CM032587.1	1701161	.	C	G	100	*	TYPE=SUBSTITUTE
CM032587.1	1701167	.	A	G	100	*	TYPE=SUBSTITUTE
CM032587.1	1701187	.	T	A	100	*	TYPE=SUBSTITUTE
CM032587.1	1701200	.	C	T	100	*	TYPE=SUBSTITUTE
CM032587.1	1701209	.	C	T	100	*	TYPE=SUBSTITUTE
CM032587.1	1701218	.	C	a	100	*	TYPE=SUBSTITUTE
CM032587.1	1701228	.	A	g	100	*	TYPE=SUBSTITUTE
CM032587.1	1701245	.	A	T	100	*	TYPE=SUBSTITUTE
CM032587.1	1701269	.	C	T	100	*	TYPE=SUBSTITUTE
CM032587.1	1701284	.	C	T	100	*	TYPE=SUBSTITUTE
CM032587.1	1701287	.	A	G	100	*	TYPE=SUBSTITUTE
CM032587.1	1701297	.	G	A	100	*	TYPE=SUBSTITUTE
CM032587.1	1701311	.	T	C	100	*	TYPE=SUBSTITUTE
CM032587.1	1701329	.	C	T	100	*	TYPE=SUBSTITUTE
CM032587.1	1701377	.	C	T	100	*	TYPE=SUBSTITUTE
CM032587.1	1701395	.	T	C	100	*	TYPE=SUBSTITUTE
CM032587.1	1701410	.	C	A	100	*	TYPE=SUBSTITUTE
CM032587.1	1701422	.	G	A	100	*	TYPE=SUBSTITUTE
CM032587.1	1701425	.	C	T	100	*	TYPE=SUBSTITUTE
CM032587.1	1701431	.	T	C	100	*	TYPE=SUBSTITUTE
CM032587.1	1701458	.	G	A	100	*	TYPE=SUBSTITUTE
CM032587.1	1701467	.	G	A	100	*	TYPE=SUBSTITUTE
CM032587.1	1701473	.	C	G	100	*	TYPE=SUBSTITUTE
CM032587.1	1701476	.	T	C	100	*	TYPE=SUBSTITUTE
CM032587.1	1701485	.	T	C	100	*	TYPE=SUBSTITUTE

CM032587.1	1701503	.	G	A	100	*	TYPE=SUBSTITUTE
CM032587.1	1701512	.	G	A	100	*	TYPE=SUBSTITUTE
CM032587.1	1701518	.	T	C	100	*	TYPE=SUBSTITUTE
CM032587.1	1701530	.	G	A	100	*	TYPE=SUBSTITUTE
CM032587.1	1701533	.	C	T	100	*	TYPE=SUBSTITUTE
CM032587.1	1701536	.	C	T	100	*	TYPE=SUBSTITUTE
CM032587.1	1701539	.	C	A	100	*	TYPE=SUBSTITUTE
CM032587.1	1701635	.	G	A	100	*	TYPE=SUBSTITUTE
CM032587.1	1701638	.	T	C	100	*	TYPE=SUBSTITUTE
CM032587.1	1701653	.	G	A	100	*	TYPE=SUBSTITUTE
CM032587.1	1701695	.	T	C	100	*	TYPE=SUBSTITUTE
CM032587.1	1701721	.	A	G	100	*	TYPE=SUBSTITUTE
CM032587.1	1701745	.	A	G	100	*	TYPE=SUBSTITUTE
CM032587.1	1701767	.	G	A	100	*	TYPE=SUBSTITUTE
CM032587.1	1701907	.	G	A	100	*	TYPE=SUBSTITUTE
CM032587.1	1701923	.	T	C	100	*	TYPE=SUBSTITUTE
CM032587.1	1701932	.	C	T	100	*	TYPE=SUBSTITUTE
CM032587.1	1701947	.	A	G	100	*	TYPE=SUBSTITUTE
CM032587.1	1701950	.	A	G	100	*	TYPE=SUBSTITUTE
CM032587.1	1701952	.	A	C	100	*	TYPE=SUBSTITUTE
CM032587.1	1702030	.	C	T	100	*	TYPE=SUBSTITUTE
CM032587.1	1702049	.	C	T	100	*	TYPE=SUBSTITUTE
CM032587.1	1702088	.	G	T	100	*	TYPE=SUBSTITUTE
CM032587.1	1702089	.	A	G	100	*	TYPE=SUBSTITUTE
CM032587.1	1702091	.	TT	T	100	*	TYPE=DELETE
CM032587.1	1702245	.	C	T	100	*	TYPE=SUBSTITUTE
CM032587.1	1702329	.	T	C	100	*	TYPE=SUBSTITUTE
CM032587.1	1702349	.	T	C	100	*	TYPE=SUBSTITUTE
CM032587.1	1702396	.	T	C	100	*	TYPE=SUBSTITUTE
CM032587.1	1702428	.	G	A	100	*	TYPE=SUBSTITUTE
CM032587.1	1702461	.	T	C	100	*	TYPE=SUBSTITUTE
CM032587.1	1702495	.	G	A	100	*	TYPE=SUBSTITUTE
CM032587.1	1702521	.	T	G	100	*	TYPE=SUBSTITUTE
CM032587.1	1702533	.	G	A	100	*	TYPE=SUBSTITUTE
CM032587.1	1702538	.	C	A	100	*	TYPE=SUBSTITUTE
CM032587.1	1702540	.	G	C	100	*	TYPE=SUBSTITUTE
CM032587.1	1702542	.	T	A	100	*	TYPE=SUBSTITUTE
CM032587.1	1702547	.	A	T	100	*	TYPE=SUBSTITUTE
CM032587.1	1702554	.	T	C	100	*	TYPE=SUBSTITUTE
CM032587.1	1702565	.	A	G	100	*	TYPE=SUBSTITUTE
CM032587.1	1702569	.	T	C	100	*	TYPE=SUBSTITUTE
CM032587.1	1702580	.	AC	A	100	*	TYPE=DELETE
CM032587.1	1702585	.	T	C	100	*	TYPE=SUBSTITUTE
CM032587.1	1702588	.	A	C	100	*	TYPE=SUBSTITUTE
CM032587.1	1702592	.	G	A	100	*	TYPE=SUBSTITUTE
CM032587.1	1702596	.	T	A	100	*	TYPE=SUBSTITUTE
CM032587.1	1702597	.	G	A	100	*	TYPE=SUBSTITUTE
CM032587.1	1702600	.	T	A	100	*	TYPE=SUBSTITUTE
CM032587.1	1702603	.	G	A	100	*	TYPE=SUBSTITUTE
CM032587.1	1702611	.	A	G	100	*	TYPE=SUBSTITUTE
CM032587.1	1702613	.	C	T	100	*	TYPE=SUBSTITUTE
CM032587.1	1702622	.	C	T	100	*	TYPE=SUBSTITUTE
CM032587.1	1702630	.	T	C	100	*	TYPE=SUBSTITUTE
CM032587.1	1702635	.	G	A	100	*	TYPE=SUBSTITUTE
CM032587.1	1702637	.	T	C	100	*	TYPE=SUBSTITUTE
CM032587.1	1702639	.	T	C	100	*	TYPE=SUBSTITUTE
CM032587.1	1702640	.	C	T	100	*	TYPE=SUBSTITUTE
CM032587.1	1702648	.	T	C	100	*	TYPE=SUBSTITUTE
CM032587.1	1702661	.	TTTT	T	100	*	TYPE=DELETE
CM032587.1	1702677	.	T	C	100	*	TYPE=SUBSTITUTE
CM032587.1	1702678	.	A	T	100	*	TYPE=SUBSTITUTE
CM032587.1	1702679	.	G	A	100	*	TYPE=SUBSTITUTE
CM032587.1	1702691	.	C	T	100	*	TYPE=SUBSTITUTE
CM032587.1	1702702	.	C	T	100	*	TYPE=SUBSTITUTE

CM032587.1	1702730	.	C	G	100	*	TYPE=SUBSTITUTE
CM032587.1	1702731	.	C	G	100	*	TYPE=SUBSTITUTE
CM032587.1	1702735	.	T	C	100	*	TYPE=SUBSTITUTE
CM032587.1	1702742	.	G	A	100	*	TYPE=SUBSTITUTE
CM032587.1	1702755	.	T	G	100	*	TYPE=SUBSTITUTE
CM032587.1	1702769	.	A	T	100	*	TYPE=SUBSTITUTE
CM032587.1	1702852	.	T	TAC	100	*	TYPE=INSERT
CM032587.1	1702965	.	A	c	100	*	TYPE=SUBSTITUTE
CM032587.1	1703058	.	G	T	100	*	TYPE=SUBSTITUTE
CM032587.1	1703220	.	G	A	100	*	TYPE=SUBSTITUTE
CM032587.1	1703229	.	G	A	100	*	TYPE=SUBSTITUTE
CM032587.1	1703256	.	A	G	100	*	TYPE=SUBSTITUTE
CM032587.1	1703300	.	A	G	100	*	TYPE=SUBSTITUTE
CM032587.1	1703337	.	TTACACT	T	100	*	TYPE=DELETE
CM032587.1	1703496	.	T	C	100	*	TYPE=SUBSTITUTE
CM032587.1	1703497	.	C	T	100	*	TYPE=SUBSTITUTE
CM032587.1	1703527	.	C	T	100	*	TYPE=SUBSTITUTE
CM032587.1	1703553	.	T	C	100	*	TYPE=SUBSTITUTE
CM032587.1	1703561	.	A	ATTGTA	100	*	TYPE=INSERT
CM032587.1	1703704	.	AC	A	100	*	TYPE=DELETE
CM032587.1	1703706	.	A	G	100	*	TYPE=SUBSTITUTE
CM032587.1	1703728	.	C	T	100	*	TYPE=SUBSTITUTE
CM032587.1	1703779	.	C	T	100	*	TYPE=SUBSTITUTE
CM032587.1	1703795	.	T	G	100	*	TYPE=SUBSTITUTE
CM032587.1	1703798	.	G	A	100	*	TYPE=SUBSTITUTE
CM032587.1	1703817	.	C	T	100	*	TYPE=SUBSTITUTE
CM032587.1	1703846	.	G	A	100	*	TYPE=SUBSTITUTE
CM032587.1	1703858	.	G	C	100	*	TYPE=SUBSTITUTE
CM032587.1	1703940	.	GA	G	100	*	TYPE=DELETE
CM032587.1	1703947	.	C	G	100	*	TYPE=SUBSTITUTE
CM032587.1	1703973	.	A	T	100	*	TYPE=SUBSTITUTE
CM032587.1	1703977	.	G	A	100	*	TYPE=SUBSTITUTE
CM032587.1	1704009	.	G	A	100	*	TYPE=SUBSTITUTE
CM032587.1	1704011	.	G	A	100	*	TYPE=SUBSTITUTE
CM032587.1	1704087	.	A	G	100	*	TYPE=SUBSTITUTE
CM032587.1	1704089	.	T	C	100	*	TYPE=SUBSTITUTE
CM032587.1	1704107	.	A	AGA	100	*	TYPE=INSERT
CM032587.1	1704132	.	GG	G	100	*	TYPE=DELETE
CM032587.1	1704169	.	G	C	100	*	TYPE=SUBSTITUTE
CM032587.1	1704170	.	G	C	100	*	TYPE=SUBSTITUTE
CM032587.1	1704171	.	C	T	100	*	TYPE=SUBSTITUTE
CM032587.1	1704173	.	C	G	100	*	TYPE=SUBSTITUTE
CM032587.1	1704174	.	A	G	100	*	TYPE=SUBSTITUTE
CM032587.1	1704177	.	A	G	100	*	TYPE=SUBSTITUTE
CM032587.1	1704178	.	T	C	100	*	TYPE=SUBSTITUTE
CM032587.1	1704182	.	A	G	100	*	TYPE=SUBSTITUTE
CM032587.1	1704201	.	G	A	100	*	TYPE=SUBSTITUTE
CM032587.1	1704202	.	T	C	100	*	TYPE=SUBSTITUTE
CM032587.1	1704206	.	C	T	100	*	TYPE=SUBSTITUTE
CM032587.1	1704211	.	A	G	100	*	TYPE=SUBSTITUTE
CM032587.1	1704212	.	G	A	100	*	TYPE=SUBSTITUTE
CM032587.1	1704213	.	T	G	100	*	TYPE=SUBSTITUTE
CM032587.1	1704270	.	A	T	100	*	TYPE=SUBSTITUTE
CM032587.1	1704293	.	C	T	100	*	TYPE=SUBSTITUTE
CM032587.1	1704294	.	T	C	100	*	TYPE=SUBSTITUTE
CM032587.1	1704389	.	T	C	100	*	TYPE=SUBSTITUTE
CM032587.1	1704398	.	C	A	100	*	TYPE=SUBSTITUTE
CM032587.1	1704401	.	C	T	100	*	TYPE=SUBSTITUTE
CM032587.1	1704410	.	A	G	100	*	TYPE=SUBSTITUTE
CM032587.1	1704428	.	G	A	100	*	TYPE=SUBSTITUTE
CM032587.1	1704438	.	GGA	G	100	*	TYPE=DELETE
CM032587.1	1704449	.	G	A	100	*	TYPE=SUBSTITUTE
CM032587.1	1704470	.	A	G	100	*	TYPE=SUBSTITUTE
CM032587.1	1704495	.	G	A	100	*	TYPE=SUBSTITUTE

CM032587.1	1704507	.	G	A	100	*	TYPE=SUBSTITUTE
CM032587.1	1704614	.	G	A	100	*	TYPE=SUBSTITUTE
CM032587.1	1704771	.	G	T	100	*	TYPE=SUBSTITUTE
CM032587.1	1704861	.	A	G	100	*	TYPE=SUBSTITUTE
CM032587.1	1704872	.	A	G	100	*	TYPE=SUBSTITUTE
CM032587.1	1704884	.	A	G	100	*	TYPE=SUBSTITUTE
CM032587.1	1704887	.	C	G	100	*	TYPE=SUBSTITUTE
CM032587.1	1704903	.	A	G	100	*	TYPE=SUBSTITUTE
CM032587.1	1704905	.	A	T	100	*	TYPE=SUBSTITUTE
CM032587.1	1704962	.	A	G	100	*	TYPE=SUBSTITUTE
CM032587.1	1704965	.	G	A	100	*	TYPE=SUBSTITUTE
CM032587.1	1704974	.	T	C	100	*	TYPE=SUBSTITUTE
CM032587.1	1704977	.	G	C	100	*	TYPE=SUBSTITUTE
CM032587.1	1705028	.	C	T	100	*	TYPE=SUBSTITUTE
CM032587.1	1705052	.	C	T	100	*	TYPE=SUBSTITUTE
CM032587.1	1705060	.	A	C	100	*	TYPE=SUBSTITUTE
CM032587.1	1705061	.	G	C	100	*	TYPE=SUBSTITUTE
CM032587.1	1705067	.	G	a	100	*	TYPE=SUBSTITUTE
CM032587.1	1705079	.	G	c	100	*	TYPE=SUBSTITUTE
CM032587.1	1705088	.	A	G	100	*	TYPE=SUBSTITUTE
CM032587.1	1705091	.	C	T	100	*	TYPE=SUBSTITUTE
CM032587.1	1705115	.	C	T	100	*	TYPE=SUBSTITUTE
CM032587.1	1705158	.	T	G	100	*	TYPE=SUBSTITUTE
CM032587.1	1705160	.	T	C	100	*	TYPE=SUBSTITUTE
CM032587.1	1705172	.	A	G	100	*	TYPE=SUBSTITUTE
CM032587.1	1705188	.	A	C	100	*	TYPE=SUBSTITUTE
CM032587.1	1705208	.	A	G	100	*	TYPE=SUBSTITUTE
CM032587.1	1705220	.	T	C	100	*	TYPE=SUBSTITUTE
CM032587.1	1705226	.	G	T	100	*	TYPE=SUBSTITUTE
CM032587.1	1705235	.	C	A	100	*	TYPE=SUBSTITUTE
CM032587.1	1705243	.	A	G	100	*	TYPE=SUBSTITUTE
CM032587.1	1705274	.	C	T	100	*	TYPE=SUBSTITUTE
CM032587.1	1705317	.	T	c	100	*	TYPE=SUBSTITUTE
CM032587.1	1705325	.	C	t	100	*	TYPE=SUBSTITUTE
CM032587.1	1705391	.	G	A	100	*	TYPE=SUBSTITUTE
CM032587.1	1705424	.	C	A	100	*	TYPE=SUBSTITUTE
CM032587.1	1705470	.	T	C	100	*	TYPE=SUBSTITUTE
CM032587.1	1705481	.	C	T	100	*	TYPE=SUBSTITUTE
CM032587.1	1705487	.	T	C	100	*	TYPE=SUBSTITUTE
CM032587.1	1705506	.	C	G	100	*	TYPE=SUBSTITUTE
CM032587.1	1705508	.	C	T	100	*	TYPE=SUBSTITUTE
CM032587.1	1705511	.	G	A	100	*	TYPE=SUBSTITUTE
CM032587.1	1705514	.	A	G	100	*	TYPE=SUBSTITUTE
CM032587.1	1705532	.	G	A	100	*	TYPE=SUBSTITUTE
CM032587.1	1705538	.	T	C	100	*	TYPE=SUBSTITUTE
CM032587.1	1705562	.	A	G	100	*	TYPE=SUBSTITUTE
CM032587.1	1705563	.	C	A	100	*	TYPE=SUBSTITUTE
CM032587.1	1705569	.	C	G	100	*	TYPE=SUBSTITUTE
CM032587.1	1705575	.	A	G	100	*	TYPE=SUBSTITUTE
CM032587.1	1705601	.	G	A	100	*	TYPE=SUBSTITUTE
CM032587.1	1705613	.	G	T	100	*	TYPE=SUBSTITUTE
CM032587.1	1705619	.	C	T	100	*	TYPE=SUBSTITUTE
CM032587.1	1705620	.	T	A	100	*	TYPE=SUBSTITUTE
CM032587.1	1705621	.	C	G	100	*	TYPE=SUBSTITUTE
CM032587.1	1705622	.	G	A	100	*	TYPE=SUBSTITUTE
CM032587.1	1705653	.	T	G	100	*	TYPE=SUBSTITUTE
CM032587.1	1705658	.	G	A	100	*	TYPE=SUBSTITUTE
CM032587.1	1705685	.	G	A	100	*	TYPE=SUBSTITUTE
CM032587.1	1705692	.	C	CGAT	100	*	TYPE=INSERT
CM032587.1	1705702	.	G	A	100	*	TYPE=SUBSTITUTE
CM032587.1	1705707	.	G	C	100	*	TYPE=SUBSTITUTE
CM032587.1	1705728	.	C	T	100	*	TYPE=SUBSTITUTE
CM032587.1	1705733	.	A	G	100	*	TYPE=SUBSTITUTE
CM032587.1	1705735	.	A	G	100	*	TYPE=SUBSTITUTE

CM032587.1	1705788	.	G	C	100	*	TYPE=SUBSTITUTE
CM032587.1	1705815	.	T	C	100	*	TYPE=SUBSTITUTE
CM032587.1	1705874	.	G	A	100	*	TYPE=SUBSTITUTE
CM032587.1	1705943	.	A	G	100	*	TYPE=SUBSTITUTE
CM032587.1	1705995	.	G	A	100	*	TYPE=SUBSTITUTE
CM032587.1	1706057	.	G	A	100	*	TYPE=SUBSTITUTE
CM032587.1	1706087	.	A	T	100	*	TYPE=SUBSTITUTE
CM032587.1	1706108	.	C	T	100	*	TYPE=SUBSTITUTE
CM032587.1	1706117	.	G	A	100	*	TYPE=SUBSTITUTE
CM032587.1	1706165	.	T	C	100	*	TYPE=SUBSTITUTE
CM032587.1	1706171	.	G	A	100	*	TYPE=SUBSTITUTE
CM032587.1	1706228	.	A	G	100	*	TYPE=SUBSTITUTE
CM032587.1	1706243	.	T	G	100	*	TYPE=SUBSTITUTE
CM032587.1	1706290	.	T	A	100	*	TYPE=SUBSTITUTE
CM032587.1	1706294	.	C	T	100	*	TYPE=SUBSTITUTE
CM032587.1	1706295	.	C	T	100	*	TYPE=SUBSTITUTE
CM032587.1	1706297	.	C	T	100	*	TYPE=SUBSTITUTE
CM032587.1	1706300	.	A	G	100	*	TYPE=SUBSTITUTE
CM032587.1	1706303	.	T	C	100	*	TYPE=SUBSTITUTE
CM032587.1	1706351	.	G	A	100	*	TYPE=SUBSTITUTE
CM032587.1	1706460	.	A	G	100	*	TYPE=SUBSTITUTE
CM032587.1	1706477	.	A	G	100	*	TYPE=SUBSTITUTE
CM032587.1	1706490	.	A	G	100	*	TYPE=SUBSTITUTE
CM032587.1	1706492	.	C	T	100	*	TYPE=SUBSTITUTE
CM032587.1	1706504	.	G	A	100	*	TYPE=SUBSTITUTE
CM032587.1	1706522	.	G	A	100	*	TYPE=SUBSTITUTE
CM032587.1	1706525	.	G	A	100	*	TYPE=SUBSTITUTE
CM032587.1	1706549	.	C	G	100	*	TYPE=SUBSTITUTE
CM032587.1	1706576	.	G	A	100	*	TYPE=SUBSTITUTE
CM032587.1	1706579	.	A	G	100	*	TYPE=SUBSTITUTE
CM032587.1	1706585	.	C	A	100	*	TYPE=SUBSTITUTE
CM032587.1	1706756	.	A	G	100	*	TYPE=SUBSTITUTE
CM032587.1	1706765	.	T	C	100	*	TYPE=SUBSTITUTE
CM032587.1	1706852	.	G	A	100	*	TYPE=SUBSTITUTE
CM032587.1	1706969	.	A	G	100	*	TYPE=SUBSTITUTE
CM032587.1	1707101	.	T	C	100	*	TYPE=SUBSTITUTE
CM032587.1	1707107	.	A	T	100	*	TYPE=SUBSTITUTE
CM032587.1	1707134	.	C	T	100	*	TYPE=SUBSTITUTE
CM032587.1	1707270	.	T	G	100	*	TYPE=SUBSTITUTE
CM032587.1	1707341	.	T	C	100	*	TYPE=SUBSTITUTE
CM032587.1	1707356	.	G	A	100	*	TYPE=SUBSTITUTE
CM032587.1	1707365	.	G	T	100	*	TYPE=SUBSTITUTE
CM032587.1	1707371	.	G	A	100	*	TYPE=SUBSTITUTE
CM032587.1	1707416	.	G	A	100	*	TYPE=SUBSTITUTE
CM032587.1	1707476	.	G	a	100	*	TYPE=SUBSTITUTE
CM032587.1	1707680	.	T	C	100	*	TYPE=SUBSTITUTE
CM032587.1	1707719	.	G	T	100	*	TYPE=SUBSTITUTE
CM032587.1	1707758	.	T	C	100	*	TYPE=SUBSTITUTE
CM032587.1	1707779	.	A	G	100	*	TYPE=SUBSTITUTE
CM032587.1	1707843	.	G	T	100	*	TYPE=SUBSTITUTE
CM032587.1	1707869	.	A	G	100	*	TYPE=SUBSTITUTE
CM032587.1	1707893	.	T	C	100	*	TYPE=SUBSTITUTE
CM032587.1	1707899	.	C	T	100	*	TYPE=SUBSTITUTE
CM032587.1	1707926	.	T	C	100	*	TYPE=SUBSTITUTE
CM032587.1	1707950	.	C	T	100	*	TYPE=SUBSTITUTE
CM032587.1	1707963	.	C	T	100	*	TYPE=SUBSTITUTE
CM032587.1	1707981	.	G	T	100	*	TYPE=SUBSTITUTE
CM032587.1	1707983	.	A	G	100	*	TYPE=SUBSTITUTE
CM032587.1	1707984	.	C	T	100	*	TYPE=SUBSTITUTE
CM032587.1	1707985	.	A	G	100	*	TYPE=SUBSTITUTE
CM032587.1	1707990	.	A	G	100	*	TYPE=SUBSTITUTE
CM032587.1	1707997	.	AT	A	100	*	TYPE=DELETE
CM032587.1	1708001	.	A	G	100	*	TYPE=SUBSTITUTE
CM032587.1	1708006	.	A	G	100	*	TYPE=SUBSTITUTE

CM032587.1	1708008	.	G	T	100	*	TYPE=SUBSTITUTE
CM032587.1	1708010	.	C	G	100	*	TYPE=SUBSTITUTE
CM032587.1	1708027	.	C	A	100	*	TYPE=SUBSTITUTE
CM032587.1	1708028	.	C	T	100	*	TYPE=SUBSTITUTE
CM032587.1	1708029	.	A	G	100	*	TYPE=SUBSTITUTE
CM032587.1	1708038	.	T	C	100	*	TYPE=SUBSTITUTE
CM032587.1	1708047	.	T	C	100	*	TYPE=SUBSTITUTE
CM032587.1	1708077	.	C	T	100	*	TYPE=SUBSTITUTE
CM032587.1	1708087	.	G	A	100	*	TYPE=SUBSTITUTE
CM032587.1	1708098	.	T	C	100	*	TYPE=SUBSTITUTE
CM032587.1	1708261	.	GT	G	100	*	TYPE=DELETE
CM032587.1	1708272	.	C	CC	100	*	TYPE=INSERT
CM032587.1	1708298	.	T	A	100	*	TYPE=SUBSTITUTE
CM032587.1	1708299	.	C	T	100	*	TYPE=SUBSTITUTE
CM032587.1	1708326	.	T	c	100	*	TYPE=SUBSTITUTE
CM032587.1	1708407	.	T	C	100	*	TYPE=SUBSTITUTE
CM032587.1	1708468	.	CC	C	100	*	TYPE=DELETE
CM032587.1	1708526	.	C	CA	100	*	TYPE=INSERT
CM032587.1	1708527	.	A	T	100	*	TYPE=SUBSTITUTE
CM032587.1	1708535	.	CA	C	100	*	TYPE=DELETE
CM032587.1	1708540	.	A	g	100	*	TYPE=SUBSTITUTE
CM032587.1	1708556	.	g	gt	100	*	TYPE=INSERT
CM032587.1	1708564	.	C	T	100	*	TYPE=SUBSTITUTE
CM032587.1	1708597	.	G	C	100	*	TYPE=SUBSTITUTE
CM032587.1	1708599	.	A	G	100	*	TYPE=SUBSTITUTE
CM032587.1	1708609	.	T	C	100	*	TYPE=SUBSTITUTE
CM032587.1	1708631	.	C	T	100	*	TYPE=SUBSTITUTE
CM032587.1	1708642	.	C	T	100	*	TYPE=SUBSTITUTE
CM032587.1	1708644	.	G	A	100	*	TYPE=SUBSTITUTE
CM032587.1	1708647	.	T	C	100	*	TYPE=SUBSTITUTE
CM032587.1	1708660	.	C	CA	100	*	TYPE=INSERT
CM032587.1	1708663	.	T	C	100	*	TYPE=SUBSTITUTE
CM032587.1	1708677	.	A	T	100	*	TYPE=SUBSTITUTE
CM032587.1	1708693	.	A	C	100	*	TYPE=SUBSTITUTE
CM032587.1	1708694	.	C	T	100	*	TYPE=SUBSTITUTE
CM032587.1	1708700	.	A	C	100	*	TYPE=SUBSTITUTE
CM032587.1	1708708	.	T	C	100	*	TYPE=SUBSTITUTE
CM032587.1	1708709	.	G	T	100	*	TYPE=SUBSTITUTE
CM032587.1	1708720	.	C	T	100	*	TYPE=SUBSTITUTE
CM032587.1	1708739	.	T	A	100	*	TYPE=SUBSTITUTE
CM032587.1	1708743	.	A	G	100	*	TYPE=SUBSTITUTE
CM032587.1	1708746	.	G	A	100	*	TYPE=SUBSTITUTE
CM032587.1	1708755	.	G	T	100	*	TYPE=SUBSTITUTE
CM032587.1	1708777	.	A	ACC	100	*	TYPE=INSERT
CM032587.1	1708793	.	G	A	100	*	TYPE=SUBSTITUTE
CM032587.1	1708796	.	T	C	100	*	TYPE=SUBSTITUTE
CM032587.1	1708823	.	A	ATATAGCC	100	*	TYPE=INSERT
CM032587.1	1708829	.	T	G	100	*	TYPE=SUBSTITUTE
CM032587.1	1708850	.	T	G	100	*	TYPE=SUBSTITUTE
CM032587.1	1708851	.	T	C	100	*	TYPE=SUBSTITUTE
CM032587.1	1708852	.	G	A	100	*	TYPE=SUBSTITUTE
CM032587.1	1708863	.	C	T	100	*	TYPE=SUBSTITUTE
CM032587.1	1708894	.	G	A	100	*	TYPE=SUBSTITUTE
CM032587.1	1708911	.	A	T	100	*	TYPE=SUBSTITUTE
CM032587.1	1708912	.	G	A	100	*	TYPE=SUBSTITUTE
CM032587.1	1708914	.	A	C	100	*	TYPE=SUBSTITUTE
CM032587.1	1708947	.	TATTTTACCTAG	T	100	*	TYPE=DELETE
CM032587.1	1708955	.	CATT	C	100	*	TYPE=DELETE
CM032587.1	1708964	.	G	A	100	*	TYPE=SUBSTITUTE
CM032587.1	1708994	.	ACGTATT	A	100	*	TYPE=DELETE
CM032587.1	1709004	.	A	G	100	*	TYPE=SUBSTITUTE
CM032587.1	1709007	.	A	G	100	*	TYPE=SUBSTITUTE
CM032587.1	1709008	.	A	C	100	*	TYPE=SUBSTITUTE
CM032587.1	1709016	.	G	A	100	*	TYPE=SUBSTITUTE

CM032587.1	1709019	.	T	C	100	*	TYPE=SUBSTITUTE
CM032587.1	1709037	.	T	C	100	*	TYPE=SUBSTITUTE
CM032587.1	1709038	.	T	C	100	*	TYPE=SUBSTITUTE
CM032587.1	1709061	.	C	CA	100	*	TYPE=INSERT
CM032587.1	1709067	.	G	a	100	*	TYPE=SUBSTITUTE
CM032587.1	1709068	.	G	c	100	*	TYPE=SUBSTITUTE
CM032587.1	1709069	.	t	tctttt	100	*	TYPE=INSERT
CM032587.1	1709133	.	T	a	100	*	TYPE=SUBSTITUTE
CM032587.1	1709134	.	T	a	100	*	TYPE=SUBSTITUTE
CM032587.1	1709135	.	A	g	100	*	TYPE=SUBSTITUTE
CM032587.1	1709137	.	G	t	100	*	TYPE=SUBSTITUTE
CM032587.1	1709141	.	C	t	100	*	TYPE=SUBSTITUTE
CM032587.1	1709142	.	T	c	100	*	TYPE=SUBSTITUTE
CM032587.1	1709149	.	C	t	100	*	TYPE=SUBSTITUTE
CM032587.1	1709151	.	T	a	100	*	TYPE=SUBSTITUTE
CM032587.1	1709153	.	c	ca	100	*	TYPE=INSERT
CM032587.1	1709173	.	TC	T	100	*	TYPE=DELETE
CM032587.1	1709178	.	GT	G	100	*	TYPE=DELETE
CM032587.1	1709183	.	C	CC	100	*	TYPE=INSERT
CM032587.1	1709210	.	T	C	100	*	TYPE=SUBSTITUTE
CM032587.1	1709241	.	C	CTGGCCCTGAAGTCT	100	*	TYPE=INSERT
CM032587.1	1709256	.	C	T	100	*	TYPE=SUBSTITUTE
CM032587.1	1709259	.	GA	G	100	*	TYPE=DELETE
CM032587.1	1709264	.	G	GA	100	*	TYPE=INSERT
CM032587.1	1709270	.	T	C	100	*	TYPE=SUBSTITUTE
CM032587.1	1709272	.	T	A	100	*	TYPE=SUBSTITUTE
CM032587.1	1709281	.	A	C	100	*	TYPE=SUBSTITUTE
CM032587.1	1709298	.	G	A	100	*	TYPE=SUBSTITUTE
CM032587.1	1709299	.	A	G	100	*	TYPE=SUBSTITUTE
CM032587.1	1709302	.	A	G	100	*	TYPE=SUBSTITUTE
CM032587.1	1709305	.	C	T	100	*	TYPE=SUBSTITUTE
CM032587.1	1709306	.	C	G	100	*	TYPE=SUBSTITUTE
CM032587.1	1709307	.	A	AG	100	*	TYPE=INSERT
CM032587.1	1709311	.	G	A	100	*	TYPE=SUBSTITUTE
CM032587.1	1709319	.	A	C	100	*	TYPE=SUBSTITUTE
CM032587.1	1709323	.	AAG	A	100	*	TYPE=DELETE
CM032587.1	1709326	.	G	C	100	*	TYPE=SUBSTITUTE
CM032587.1	1709331	.	A	G	100	*	TYPE=SUBSTITUTE
CM032587.1	1709333	.	T	A	100	*	TYPE=SUBSTITUTE
CM032587.1	1709334	.	C	G	100	*	TYPE=SUBSTITUTE
CM032587.1	1709337	.	C	A	100	*	TYPE=SUBSTITUTE
CM032587.1	1709340	.	T	C	100	*	TYPE=SUBSTITUTE
CM032587.1	1709341	.	A	G	100	*	TYPE=SUBSTITUTE
CM032587.1	1709343	.	C	A	100	*	TYPE=SUBSTITUTE
CM032587.1	1709347	.	T	C	100	*	TYPE=SUBSTITUTE
CM032587.1	1709351	.	C	t	100	*	TYPE=SUBSTITUTE
CM032587.1	1709352	.	C	t	100	*	TYPE=SUBSTITUTE
CM032587.1	1709354	.	C	t	100	*	TYPE=SUBSTITUTE
CM032587.1	1709362	.	C	t	100	*	TYPE=SUBSTITUTE
CM032587.1	1709364	.	T	c	100	*	TYPE=SUBSTITUTE
CM032587.1	1709382	.	TATCTTTTCTCTTC	T	100	*	TYPE=DELETE
CM032587.1	1709385	.	T	C	100	*	TYPE=SUBSTITUTE
CM032587.1	1709395	.	G	a	100	*	TYPE=SUBSTITUTE
CM032587.1	1709407	.	T	c	100	*	TYPE=SUBSTITUTE
CM032587.1	1709408	.	A	t	100	*	TYPE=SUBSTITUTE
CM032587.1	1709421	.	C	T	100	*	TYPE=SUBSTITUTE
CM032587.1	1709442	.	AA	A	100	*	TYPE=DELETE
CM032587.1	1709517	.	A	G	100	*	TYPE=SUBSTITUTE
CM032587.1	1709520	.	T	C	100	*	TYPE=SUBSTITUTE
CM032587.1	1709526	.	C	T	100	*	TYPE=SUBSTITUTE
CM032587.1	1709531	.	G	T	100	*	TYPE=SUBSTITUTE
CM032587.1	1709532	.	G	GT	100	*	TYPE=INSERT
CM032587.1	1709534	.	A	G	100	*	TYPE=SUBSTITUTE
CM032587.1	1709536	.	T	A	100	*	TYPE=SUBSTITUTE

CM032587.1	1709566	.	G	GC	100	*	TYPE=INSERT
CM032587.1	1709578	.	GA	G	100	*	TYPE=DELETE
CM032587.1	1709595	.	C	T	100	*	TYPE=SUBSTITUTE
CM032587.1	1709597	.	C	T	100	*	TYPE=SUBSTITUTE
CM032587.1	1709600	.	T	TT	100	*	TYPE=INSERT
CM032587.1	1709624	.	TT	T	100	*	TYPE=DELETE
CM032587.1	1709664	.	G	C	100	*	TYPE=SUBSTITUTE
CM032587.1	1709700	.	C	T	100	*	TYPE=SUBSTITUTE
CM032587.1	1709703	.	C	G	100	*	TYPE=SUBSTITUTE
CM032587.1	1709911	.	G	A	100	*	TYPE=SUBSTITUTE
CM032587.1	1709943	.	A	G	100	*	TYPE=SUBSTITUTE
CM032587.1	1709952	.	G	A	100	*	TYPE=SUBSTITUTE
CM032587.1	1709964	.	G	C	100	*	TYPE=SUBSTITUTE
CM032587.1	1709979	.	A	C	100	*	TYPE=SUBSTITUTE
CM032587.1	1710138	.	G	A	100	*	TYPE=SUBSTITUTE
CM032587.1	1710174	.	G	A	100	*	TYPE=SUBSTITUTE
CM032587.1	1710180	.	T	C	100	*	TYPE=SUBSTITUTE
CM032587.1	1710279	.	A	G	100	*	TYPE=SUBSTITUTE
CM032587.1	1710310	.	G	A	100	*	TYPE=SUBSTITUTE
CM032587.1	1710313	.	C	G	100	*	TYPE=SUBSTITUTE
CM032587.1	1710314	.	G	A	100	*	TYPE=SUBSTITUTE
CM032587.1	1710319	.	A	AT	100	*	TYPE=INSERT
CM032587.1	1710321	.	A	C	100	*	TYPE=SUBSTITUTE
CM032587.1	1710325	.	T	TCA	100	*	TYPE=INSERT
CM032587.1	1710334	.	AA	A	100	*	TYPE=DELETE
CM032587.1	1710371	.	C	CT	100	*	TYPE=INSERT
CM032587.1	1710374	.	T	C	100	*	TYPE=SUBSTITUTE
CM032587.1	1710391	.	A	G	100	*	TYPE=SUBSTITUTE
CM032587.1	1710570	.	C	A	100	*	TYPE=SUBSTITUTE
CM032587.1	1710597	.	A	G	100	*	TYPE=SUBSTITUTE
CM032587.1	1710639	.	G	A	100	*	TYPE=SUBSTITUTE
CM032587.1	1710645	.	C	T	100	*	TYPE=SUBSTITUTE
CM032587.1	1710660	.	T	C	100	*	TYPE=SUBSTITUTE
CM032587.1	1710731	.	G	A	100	*	TYPE=SUBSTITUTE
CM032587.1	1710838	.	A	G	100	*	TYPE=SUBSTITUTE
CM032587.1	1710846	.	T	C	100	*	TYPE=SUBSTITUTE
CM032587.1	1710847	.	G	A	100	*	TYPE=SUBSTITUTE
CM032587.1	1710853	.	A	T	100	*	TYPE=SUBSTITUTE
CM032587.1	1710884	.	T	C	100	*	TYPE=SUBSTITUTE
CM032587.1	1710886	.	T	C	100	*	TYPE=SUBSTITUTE
CM032587.1	1710887	.	G	A	100	*	TYPE=SUBSTITUTE
CM032587.1	1710897	.	C	T	100	*	TYPE=SUBSTITUTE
CM032587.1	1710903	.	A	ATTCC	100	*	TYPE=INSERT
CM032587.1	1710905	.	T	C	100	*	TYPE=SUBSTITUTE
CM032587.1	1710960	.	G	A	100	*	TYPE=SUBSTITUTE
CM032587.1	1710980	.	T	C	100	*	TYPE=SUBSTITUTE
CM032587.1	1711012	.	T	C	100	*	TYPE=SUBSTITUTE
CM032587.1	1711028	.	G	A	100	*	TYPE=SUBSTITUTE
CM032587.1	1711079	.	A	G	100	*	TYPE=SUBSTITUTE
CM032587.1	1711082	.	G	A	100	*	TYPE=SUBSTITUTE
CM032587.1	1711253	.	A	C	100	*	TYPE=SUBSTITUTE
CM032587.1	1711473	.	T	C	100	*	TYPE=SUBSTITUTE
CM032587.1	1711757	.	G	C	100	*	TYPE=SUBSTITUTE
CM032587.1	1711779	.	G	T	100	*	TYPE=SUBSTITUTE
CM032587.1	1711792	.	G	A	100	*	TYPE=SUBSTITUTE
CM032587.1	1711898	.	CAGCTTGCC	C	100	*	TYPE=DELETE
CM032587.1	1711989	.	A	G	100	*	TYPE=SUBSTITUTE
CM032587.1	1712039	.	T	G	100	*	TYPE=SUBSTITUTE
CM032587.1	1712048	.	TT	T	100	*	TYPE=DELETE
CM032587.1	1712136	.	A	C	100	*	TYPE=SUBSTITUTE
CM032587.1	1712141	.	AG	A	100	*	TYPE=DELETE
CM032587.1	1712145	.	c	cggagtac	100	*	TYPE=INSERT
CM032587.1	1712166	.	A	G	100	*	TYPE=SUBSTITUTE
CM032587.1	1712300	.	G	A	100	*	TYPE=SUBSTITUTE

CM032587.1	1712303	.	C	T	100	*	TYPE=SUBSTITUTE
CM032587.1	1712323	.	A	G	100	*	TYPE=SUBSTITUTE
CM032587.1	1712338	.	ACT	A	100	*	TYPE=DELETE
CM032587.1	1712340	.	T	G	100	*	TYPE=SUBSTITUTE
CM032587.1	1712341	.	T	A	100	*	TYPE=SUBSTITUTE
CM032587.1	1712348	.	T	C	100	*	TYPE=SUBSTITUTE
CM032587.1	1712368	.	AC	A	100	*	TYPE=DELETE
CM032587.1	1712372	.	T	C	100	*	TYPE=SUBSTITUTE
CM032587.1	1712376	.	TGT	T	100	*	TYPE=DELETE
CM032587.1	1712399	.	CC	C	100	*	TYPE=DELETE
CM032587.1	1712456	.	T	C	100	*	TYPE=SUBSTITUTE
CM032587.1	1712461	.	A	G	100	*	TYPE=SUBSTITUTE
CM032587.1	1712495	.	A	G	100	*	TYPE=SUBSTITUTE
CM032587.1	1712499	.	G	A	100	*	TYPE=SUBSTITUTE
CM032587.1	1712501	.	A	G	100	*	TYPE=SUBSTITUTE
CM032587.1	1712508	.	A	G	100	*	TYPE=SUBSTITUTE
CM032587.1	1712531	.	C	T	100	*	TYPE=SUBSTITUTE
CM032587.1	1712545	.	T	G	100	*	TYPE=SUBSTITUTE
CM032587.1	1712548	.	CCT	C	100	*	TYPE=DELETE
CM032587.1	1712590	.	T	C	100	*	TYPE=SUBSTITUTE
CM032587.1	1712591	.	G	T	100	*	TYPE=SUBSTITUTE
CM032587.1	1712641	.	T	C	100	*	TYPE=SUBSTITUTE
CM032587.1	1712710	.	C	CT	100	*	TYPE=INSERT
CM032587.1	1712720	.	G	A	100	*	TYPE=SUBSTITUTE
CM032587.1	1712751	.	C	T	100	*	TYPE=SUBSTITUTE
CM032587.1	1712753	.	T	A	100	*	TYPE=SUBSTITUTE
CM032587.1	1712755	.	T	C	100	*	TYPE=SUBSTITUTE
CM032587.1	1712756	.	G	A	100	*	TYPE=SUBSTITUTE
CM032587.1	1712758	.	C	T	100	*	TYPE=SUBSTITUTE
CM032587.1	1712765	.	C	G	100	*	TYPE=SUBSTITUTE
CM032587.1	1712772	.	C	T	100	*	TYPE=SUBSTITUTE
CM032587.1	1712781	.	C	T	100	*	TYPE=SUBSTITUTE
CM032587.1	1712783	.	G	A	100	*	TYPE=SUBSTITUTE
CM032587.1	1712787	.	T	A	100	*	TYPE=SUBSTITUTE
CM032587.1	1712796	.	CG	C	100	*	TYPE=DELETE
CM032587.1	1712803	.	A	T	100	*	TYPE=SUBSTITUTE
CM032587.1	1712816	.	C	A	100	*	TYPE=SUBSTITUTE
CM032587.1	1712818	.	T	A	100	*	TYPE=SUBSTITUTE
CM032587.1	1712826	.	G	A	100	*	TYPE=SUBSTITUTE
CM032587.1	1712838	.	AACAA	A	100	*	TYPE=DELETE
CM032587.1	1712853	.	C	T	100	*	TYPE=SUBSTITUTE
CM032587.1	1712868	.	T	G	100	*	TYPE=SUBSTITUTE
CM032587.1	1712873	.	G	A	100	*	TYPE=SUBSTITUTE
CM032587.1	1712875	.	A	AAAG	100	*	TYPE=INSERT
CM032587.1	1712887	.	G	T	100	*	TYPE=SUBSTITUTE
CM032587.1	1712896	.	G	C	100	*	TYPE=SUBSTITUTE
CM032587.1	1712899	.	G	GA	100	*	TYPE=INSERT
CM032587.1	1712918	.	T	G	100	*	TYPE=SUBSTITUTE
CM032587.1	1712926	.	C	T	100	*	TYPE=SUBSTITUTE
CM032587.1	1712992	.	A	G	100	*	TYPE=SUBSTITUTE
CM032587.1	1713021	.	G	c	100	*	TYPE=SUBSTITUTE
CM032587.1	1713027	.	G	a	100	*	TYPE=SUBSTITUTE
CM032587.1	1713078	.	T	C	100	*	TYPE=SUBSTITUTE
CM032587.1	1713106	.	A	T	100	*	TYPE=SUBSTITUTE
CM032587.1	1713131	.	A	T	100	*	TYPE=SUBSTITUTE
CM032587.1	1713133	.	C	A	100	*	TYPE=SUBSTITUTE
CM032587.1	1713163	.	A	G	100	*	TYPE=SUBSTITUTE
CM032587.1	1713187	.	A	G	100	*	TYPE=SUBSTITUTE
CM032587.1	1713188	.	G	A	100	*	TYPE=SUBSTITUTE
CM032587.1	1713193	.	T	C	100	*	TYPE=SUBSTITUTE
CM032587.1	1713265	.	C	t	100	*	TYPE=SUBSTITUTE
CM032587.1	1713274	.	C	t	100	*	TYPE=SUBSTITUTE
CM032587.1	1713374	.	A	c	100	*	TYPE=SUBSTITUTE
CM032587.1	1713400	.	G	A	100	*	TYPE=SUBSTITUTE

CM032587.1	1713413	.	A	G	100	*	TYPE=SUBSTITUTE
CM032587.1	1713415	.	C	T	100	*	TYPE=SUBSTITUTE
CM032587.1	1713475	.	C	A	100	*	TYPE=SUBSTITUTE
CM032587.1	1713667	.	G	a	100	*	TYPE=SUBSTITUTE
CM032587.1	1713764	.	A	G	100	*	TYPE=SUBSTITUTE
CM032587.1	1713768	.	G	T	100	*	TYPE=SUBSTITUTE
CM032587.1	1713776	.	C	T	100	*	TYPE=SUBSTITUTE
CM032587.1	1713810	.	A	T	100	*	TYPE=SUBSTITUTE
CM032587.1	1713812	.	TC	T	100	*	TYPE=DELETE
CM032587.1	1713818	.	AT	A	100	*	TYPE=DELETE
CM032587.1	1713850	.	A	G	100	*	TYPE=SUBSTITUTE
CM032587.1	1713880	.	G	A	100	*	TYPE=SUBSTITUTE
CM032587.1	1713946	.	G	A	100	*	TYPE=SUBSTITUTE
CM032587.1	1714006	.	TA	T	100	*	TYPE=DELETE
CM032587.1	1714011	.	A	T	100	*	TYPE=SUBSTITUTE
CM032587.1	1714030	.	CAA	C	100	*	TYPE=DELETE
CM032587.1	1714038	.	A	G	100	*	TYPE=SUBSTITUTE
CM032587.1	1714113	.	T	C	100	*	TYPE=SUBSTITUTE
CM032587.1	1714212	.	C	T	100	*	TYPE=SUBSTITUTE
CM032587.1	1714362	.	C	T	100	*	TYPE=SUBSTITUTE
CM032587.1	1714422	.	C	T	100	*	TYPE=SUBSTITUTE
CM032587.1	1714428	.	G	C	100	*	TYPE=SUBSTITUTE
CM032587.1	1714431	.	T	G	100	*	TYPE=SUBSTITUTE
CM032587.1	1714541	.	G	C	100	*	TYPE=SUBSTITUTE
CM032587.1	1714548	.	G	A	100	*	TYPE=SUBSTITUTE
CM032587.1	1714590	.	A	g	100	*	TYPE=SUBSTITUTE
CM032587.1	1714609	.	C	T	100	*	TYPE=SUBSTITUTE
CM032587.1	1714644	.	G	A	100	*	TYPE=SUBSTITUTE
CM032587.1	1714647	.	C	T	100	*	TYPE=SUBSTITUTE
CM032587.1	1714656	.	T	c	100	*	TYPE=SUBSTITUTE
CM032587.1	1714659	.	T	c	100	*	TYPE=SUBSTITUTE
CM032587.1	1714675	.	G	a	100	*	TYPE=SUBSTITUTE
CM032587.1	1714676	.	A	g	100	*	TYPE=SUBSTITUTE
CM032587.1	1714677	.	A	G	100	*	TYPE=SUBSTITUTE
CM032587.1	1714746	.	G	A	100	*	TYPE=SUBSTITUTE
CM032587.1	1714758	.	G	A	100	*	TYPE=SUBSTITUTE
CM032587.1	1714779	.	G	A	100	*	TYPE=SUBSTITUTE
CM032587.1	1714788	.	G	A	100	*	TYPE=SUBSTITUTE
CM032587.1	1714857	.	C	T	100	*	TYPE=SUBSTITUTE
CM032587.1	1714869	.	G	A	100	*	TYPE=SUBSTITUTE
CM032587.1	1714920	.	A	G	100	*	TYPE=SUBSTITUTE
CM032587.1	1715028	.	T	g	100	*	TYPE=SUBSTITUTE
CM032587.1	1715079	.	C	G	100	*	TYPE=SUBSTITUTE
CM032587.1	1715088	.	G	T	100	*	TYPE=SUBSTITUTE
CM032587.1	1715091	.	A	G	100	*	TYPE=SUBSTITUTE
CM032587.1	1715106	.	T	G	100	*	TYPE=SUBSTITUTE
CM032587.1	1715221	.	C	T	100	*	TYPE=SUBSTITUTE
CM032587.1	1715227	.	T	C	100	*	TYPE=SUBSTITUTE
CM032587.1	1715283	.	G	A	100	*	TYPE=SUBSTITUTE
CM032587.1	1715293	.	AT	A	100	*	TYPE=DELETE
CM032587.1	1715394	.	G	A	100	*	TYPE=SUBSTITUTE
CM032587.1	1715395	.	G	A	100	*	TYPE=SUBSTITUTE
CM032587.1	1715407	.	T	C	100	*	TYPE=SUBSTITUTE
CM032587.1	1715469	.	T	C	100	*	TYPE=SUBSTITUTE
CM032587.1	1715503	.	G	a	100	*	TYPE=SUBSTITUTE
CM032587.1	1715572	.	C	A	100	*	TYPE=SUBSTITUTE
CM032587.1	1715619	.	T	C	100	*	TYPE=SUBSTITUTE
CM032587.1	1715662	.	G	GCTGTATGGACGG	100	*	TYPE=INSERT
CM032587.1	1715702	.	A	G	100	*	TYPE=SUBSTITUTE
CM032587.1	1715707	.	A	G	100	*	TYPE=SUBSTITUTE
CM032587.1	1715711	.	G	GGA	100	*	TYPE=INSERT
CM032587.1	1715714	.	C	T	100	*	TYPE=SUBSTITUTE
CM032587.1	1715715	.	G	C	100	*	TYPE=SUBSTITUTE
CM032587.1	1715760	.	A	G	100	*	TYPE=SUBSTITUTE

CM032587.1	1715799	.	G	A	100	*	TYPE=SUBSTITUTE
CM032587.1	1715847	.	A	G	100	*	TYPE=SUBSTITUTE
CM032587.1	1715886	.	A	G	100	*	TYPE=SUBSTITUTE
CM032587.1	1715904	.	T	G	100	*	TYPE=SUBSTITUTE
CM032587.1	1715908	.	A	G	100	*	TYPE=SUBSTITUTE
CM032587.1	1715961	.	T	C	100	*	TYPE=SUBSTITUTE
CM032587.1	1715989	.	C	T	100	*	TYPE=SUBSTITUTE
CM032587.1	1716011	.	G	A	100	*	TYPE=SUBSTITUTE
CM032587.1	1716015	.	G	GC	100	*	TYPE=INSERT
CM032587.1	1716016	.	A	G	100	*	TYPE=SUBSTITUTE
CM032587.1	1716017	.	C	A	100	*	TYPE=SUBSTITUTE
CM032587.1	1716019	.	G	GTA	100	*	TYPE=INSERT
CM032587.1	1716031	.	C	T	100	*	TYPE=SUBSTITUTE
CM032587.1	1716033	.	A	G	100	*	TYPE=SUBSTITUTE
CM032587.1	1716034	.	G	T	100	*	TYPE=SUBSTITUTE
CM032587.1	1716035	.	T	C	100	*	TYPE=SUBSTITUTE
CM032587.1	1716038	.	A	G	100	*	TYPE=SUBSTITUTE
CM032587.1	1716040	.	A	T	100	*	TYPE=SUBSTITUTE
CM032587.1	1716052	.	C	G	100	*	TYPE=SUBSTITUTE
CM032587.1	1716072	.	A	G	100	*	TYPE=SUBSTITUTE
CM032587.1	1716076	.	G	A	100	*	TYPE=SUBSTITUTE
CM032587.1	1716084	.	GA	G	100	*	TYPE=DELETE
CM032587.1	1716124	.	C	A	100	*	TYPE=SUBSTITUTE
CM032587.1	1716130	.	A	G	100	*	TYPE=SUBSTITUTE
CM032587.1	1716175	.	C	T	100	*	TYPE=SUBSTITUTE
CM032587.1	1716190	.	G	A	100	*	TYPE=SUBSTITUTE
CM032587.1	1716250	.	C	T	100	*	TYPE=SUBSTITUTE
CM032587.1	1716262	.	T	C	100	*	TYPE=SUBSTITUTE
CM032587.1	1716301	.	G	A	100	*	TYPE=SUBSTITUTE
CM032587.1	1716325	.	A	G	100	*	TYPE=SUBSTITUTE
CM032587.1	1716328	.	G	A	100	*	TYPE=SUBSTITUTE
CM032587.1	1716340	.	C	T	100	*	TYPE=SUBSTITUTE
CM032587.1	1716400	.	G	A	100	*	TYPE=SUBSTITUTE
CM032587.1	1716421	.	C	T	100	*	TYPE=SUBSTITUTE
CM032587.1	1716478	.	A	G	100	*	TYPE=SUBSTITUTE
CM032587.1	1716484	.	G	A	100	*	TYPE=SUBSTITUTE
CM032587.1	1716537	.	G	T	100	*	TYPE=SUBSTITUTE
CM032587.1	1716544	.	G	GTA	100	*	TYPE=INSERT
CM032587.1	1716548	.	G	A	100	*	TYPE=SUBSTITUTE
CM032587.1	1716554	.	C	T	100	*	TYPE=SUBSTITUTE
CM032587.1	1716563	.	T	A	100	*	TYPE=SUBSTITUTE
CM032587.1	1716616	.	T	C	100	*	TYPE=SUBSTITUTE
CM032587.1	1716668	.	G	T	100	*	TYPE=SUBSTITUTE
CM032587.1	1716672	.	A	G	100	*	TYPE=SUBSTITUTE
CM032587.1	1716673	.	G	T	100	*	TYPE=SUBSTITUTE
CM032587.1	1716692	.	C	G	100	*	TYPE=SUBSTITUTE
CM032587.1	1716708	.	T	A	100	*	TYPE=SUBSTITUTE
CM032587.1	1716711	.	G	A	100	*	TYPE=SUBSTITUTE
CM032587.1	1716712	.	C	A	100	*	TYPE=SUBSTITUTE
CM032587.1	1716713	.	G	A	100	*	TYPE=SUBSTITUTE
CM032587.1	1716725	.	A	G	100	*	TYPE=SUBSTITUTE
CM032587.1	1716734	.	G	C	100	*	TYPE=SUBSTITUTE
CM032587.1	1716735	.	C	T	100	*	TYPE=SUBSTITUTE
CM032587.1	1716742	.	T	C	100	*	TYPE=SUBSTITUTE
CM032587.1	1716748	.	A	G	100	*	TYPE=SUBSTITUTE
CM032587.1	1716749	.	C	T	100	*	TYPE=SUBSTITUTE
CM032587.1	1716760	.	A	C	100	*	TYPE=SUBSTITUTE
CM032587.1	1716762	.	C	T	100	*	TYPE=SUBSTITUTE
CM032587.1	1716767	.	GT	G	100	*	TYPE=DELETE
CM032587.1	1716791	.	T	G	100	*	TYPE=SUBSTITUTE
CM032587.1	1716793	.	A	G	100	*	TYPE=SUBSTITUTE
CM032587.1	1716795	.	C	G	100	*	TYPE=SUBSTITUTE
CM032587.1	1716796	.	T	C	100	*	TYPE=SUBSTITUTE
CM032587.1	1716797	.	A	AATA	100	*	TYPE=INSERT

CM032587.1	1716817	.	C	T	100	*	TYPE=SUBSTITUTE
CM032587.1	1716818	.	A	T	100	*	TYPE=SUBSTITUTE
CM032587.1	1716826	.	T	C	100	*	TYPE=SUBSTITUTE
CM032587.1	1716837	.	G	A	100	*	TYPE=SUBSTITUTE
CM032587.1	1716843	.	G	A	100	*	TYPE=SUBSTITUTE
CM032587.1	1716856	.	A	G	100	*	TYPE=SUBSTITUTE
CM032587.1	1716890	.	G	A	100	*	TYPE=SUBSTITUTE
CM032587.1	1716898	.	G	A	100	*	TYPE=SUBSTITUTE
CM032587.1	1716899	.	C	G	100	*	TYPE=SUBSTITUTE
CM032587.1	1716904	.	C	G	100	*	TYPE=SUBSTITUTE
CM032587.1	1716912	.	A	C	100	*	TYPE=SUBSTITUTE
CM032587.1	1716982	.	A	G	100	*	TYPE=SUBSTITUTE
CM032587.1	1716991	.	C	A	100	*	TYPE=SUBSTITUTE
CM032587.1	1717036	.	A	T	100	*	TYPE=SUBSTITUTE
CM032587.1	1717054	.	C	G	100	*	TYPE=SUBSTITUTE
CM032587.1	1717057	.	T	C	100	*	TYPE=SUBSTITUTE
CM032587.1	1717102	.	A	C	100	*	TYPE=SUBSTITUTE
CM032587.1	1717108	.	T	C	100	*	TYPE=SUBSTITUTE
CM032587.1	1717114	.	C	T	100	*	TYPE=SUBSTITUTE
CM032587.1	1717138	.	C	T	100	*	TYPE=SUBSTITUTE
CM032587.1	1717161	.	C	T	100	*	TYPE=SUBSTITUTE
CM032587.1	1717171	.	A	ATG	100	*	TYPE=INSERT
CM032587.1	1717181	.	A	G	100	*	TYPE=SUBSTITUTE
CM032587.1	1717189	.	G	A	100	*	TYPE=SUBSTITUTE
CM032587.1	1717195	.	G	A	100	*	TYPE=SUBSTITUTE
CM032587.1	1717199	.	T	C	100	*	TYPE=SUBSTITUTE
CM032587.1	1717201	.	T	C	100	*	TYPE=SUBSTITUTE
CM032587.1	1717205	.	A	G	100	*	TYPE=SUBSTITUTE
CM032587.1	1717209	.	C	A	100	*	TYPE=SUBSTITUTE
CM032587.1	1717210	.	T	C	100	*	TYPE=SUBSTITUTE
CM032587.1	1717220	.	C	G	100	*	TYPE=SUBSTITUTE
CM032587.1	1717249	.	G	A	100	*	TYPE=SUBSTITUTE
CM032587.1	1717251	.	C	T	100	*	TYPE=SUBSTITUTE
CM032587.1	1717252	.	A	T	100	*	TYPE=SUBSTITUTE
CM032587.1	1717253	.	C	T	100	*	TYPE=SUBSTITUTE
CM032587.1	1717264	.	T	C	100	*	TYPE=SUBSTITUTE
CM032587.1	1717279	.	C	A	100	*	TYPE=SUBSTITUTE
CM032587.1	1717289	.	T	C	100	*	TYPE=SUBSTITUTE
CM032587.1	1717291	.	T	C	100	*	TYPE=SUBSTITUTE
CM032587.1	1717295	.	T	C	100	*	TYPE=SUBSTITUTE
CM032587.1	1717306	.	A	C	100	*	TYPE=SUBSTITUTE
CM032587.1	1717309	.	C	T	100	*	TYPE=SUBSTITUTE
CM032587.1	1717310	.	C	T	100	*	TYPE=SUBSTITUTE
CM032587.1	1717324	.	A	G	100	*	TYPE=SUBSTITUTE
CM032587.1	1717348	.	G	C	100	*	TYPE=SUBSTITUTE
CM032587.1	1717364	.	C	T	100	*	TYPE=SUBSTITUTE
CM032587.1	1717366	.	A	ACAAAC	100	*	TYPE=INSERT
CM032587.1	1717372	.	T	C	100	*	TYPE=SUBSTITUTE
CM032587.1	1717395	.	GCTGC	G	100	*	TYPE=DELETE
CM032587.1	1717398	.	G	A	100	*	TYPE=SUBSTITUTE
CM032587.1	1717399	.	G	A	100	*	TYPE=SUBSTITUTE
CM032587.1	1717404	.	G	A	100	*	TYPE=SUBSTITUTE
CM032587.1	1717405	.	T	C	100	*	TYPE=SUBSTITUTE
CM032587.1	1717407	.	A	G	100	*	TYPE=SUBSTITUTE
CM032587.1	1717411	.	C	T	100	*	TYPE=SUBSTITUTE
CM032587.1	1717413	.	A	G	100	*	TYPE=SUBSTITUTE
CM032587.1	1717414	.	G	A	100	*	TYPE=SUBSTITUTE
CM032587.1	1717418	.	AC	A	100	*	TYPE=DELETE
CM032587.1	1717429	.	T	G	100	*	TYPE=SUBSTITUTE
CM032587.1	1717443	.	T	C	100	*	TYPE=SUBSTITUTE
CM032587.1	1717446	.	G	A	100	*	TYPE=SUBSTITUTE
CM032587.1	1717448	.	G	GCT	100	*	TYPE=INSERT
CM032587.1	1717449	.	T	G	100	*	TYPE=SUBSTITUTE
CM032587.1	1717450	.	A	T	100	*	TYPE=SUBSTITUTE

CM032587.1	1717452	.	T	G	100	*	TYPE=SUBSTITUTE
CM032587.1	1717455	.	A	C	100	*	TYPE=SUBSTITUTE
CM032587.1	1717456	.	T	A	100	*	TYPE=SUBSTITUTE
CM032587.1	1717463	.	T	A	100	*	TYPE=SUBSTITUTE
CM032587.1	1717464	.	C	T	100	*	TYPE=SUBSTITUTE
CM032587.1	1717470	.	A	G	100	*	TYPE=SUBSTITUTE
CM032587.1	1717473	.	G	A	100	*	TYPE=SUBSTITUTE
CM032587.1	1717474	.	C	T	100	*	TYPE=SUBSTITUTE
CM032587.1	1717477	.	G	A	100	*	TYPE=SUBSTITUTE
CM032587.1	1717478	.	A	C	100	*	TYPE=SUBSTITUTE
CM032587.1	1717480	.	G	A	100	*	TYPE=SUBSTITUTE
CM032587.1	1717486	.	T	C	100	*	TYPE=SUBSTITUTE
CM032587.1	1717487	.	C	T	100	*	TYPE=SUBSTITUTE
CM032587.1	1717494	.	C	A	100	*	TYPE=SUBSTITUTE
CM032587.1	1717495	.	T	C	100	*	TYPE=SUBSTITUTE
CM032587.1	1717498	.	C	A	100	*	TYPE=SUBSTITUTE
CM032587.1	1717501	.	T	C	100	*	TYPE=SUBSTITUTE
CM032587.1	1717503	.	T	C	100	*	TYPE=SUBSTITUTE
CM032587.1	1717505	.	C	A	100	*	TYPE=SUBSTITUTE
CM032587.1	1717511	.	T	C	100	*	TYPE=SUBSTITUTE
CM032587.1	1717514	.	A	G	100	*	TYPE=SUBSTITUTE
CM032587.1	1717517	.	C	T	100	*	TYPE=SUBSTITUTE
CM032587.1	1717518	.	T	TTATA	100	*	TYPE=INSERT
CM032587.1	1717523	.	C	G	100	*	TYPE=SUBSTITUTE
CM032587.1	1717530	.	G	T	100	*	TYPE=SUBSTITUTE
CM032587.1	1717531	.	T	A	100	*	TYPE=SUBSTITUTE
CM032587.1	1717532	.	G	GC	100	*	TYPE=INSERT
CM032587.1	1717533	.	C	T	100	*	TYPE=SUBSTITUTE
CM032587.1	1717535	.	G	C	100	*	TYPE=SUBSTITUTE
CM032587.1	1717536	.	T	C	100	*	TYPE=SUBSTITUTE
CM032587.1	1717549	.	A	G	100	*	TYPE=SUBSTITUTE
CM032587.1	1717555	.	G	A	100	*	TYPE=SUBSTITUTE
CM032587.1	1717575	.	C	CA	100	*	TYPE=INSERT
CM032587.1	1717581	.	A	G	100	*	TYPE=SUBSTITUTE
CM032587.1	1717599	.	ATACCTACC	A	100	*	TYPE=DELETE
CM032587.1	1717604	.	T	C	100	*	TYPE=SUBSTITUTE
CM032587.1	1717612	.	T	C	100	*	TYPE=SUBSTITUTE
CM032587.1	1717624	.	CCTTAGTTT	C	100	*	TYPE=DELETE
CM032587.1	1717635	.	ACCG	A	100	*	TYPE=DELETE
CM032587.1	1717663	.	T	A	100	*	TYPE=SUBSTITUTE
CM032587.1	1717664	.	G	T	100	*	TYPE=SUBSTITUTE
CM032587.1	1717669	.	TG	T	100	*	TYPE=DELETE
CM032587.1	1717670	.	C	G	100	*	TYPE=SUBSTITUTE
CM032587.1	1717675	.	T	C	100	*	TYPE=SUBSTITUTE
CM032587.1	1717680	.	G	A	100	*	TYPE=SUBSTITUTE
CM032587.1	1717703	.	G	T	100	*	TYPE=SUBSTITUTE
CM032587.1	1717704	.	C	T	100	*	TYPE=SUBSTITUTE
CM032587.1	1717708	.	A	C	100	*	TYPE=SUBSTITUTE
CM032587.1	1717709	.	A	T	100	*	TYPE=SUBSTITUTE
CM032587.1	1717747	.	G	GG	100	*	TYPE=INSERT
CM032587.1	1717769	.	A	C	100	*	TYPE=SUBSTITUTE
CM032587.1	1717781	.	A	AT	100	*	TYPE=INSERT
CM032587.1	1717787	.	A	T	100	*	TYPE=SUBSTITUTE
CM032587.1	1717812	.	G	A	100	*	TYPE=SUBSTITUTE
CM032587.1	1717875	.	G	A	100	*	TYPE=SUBSTITUTE
CM032587.1	1717893	.	C	A	100	*	TYPE=SUBSTITUTE
CM032587.1	1717923	.	T	C	100	*	TYPE=SUBSTITUTE
CM032587.1	1717950	.	T	C	100	*	TYPE=SUBSTITUTE
CM032587.1	1717989	.	C	T	100	*	TYPE=SUBSTITUTE
CM032587.1	1718004	.	A	G	100	*	TYPE=SUBSTITUTE
CM032587.1	1718010	.	C	T	100	*	TYPE=SUBSTITUTE
CM032587.1	1718052	.	T	C	100	*	TYPE=SUBSTITUTE
CM032587.1	1718070	.	G	A	100	*	TYPE=SUBSTITUTE
CM032587.1	1718114	.	A	G	100	*	TYPE=SUBSTITUTE

CM032587.1	1718115	.	G	A	100	*	TYPE=SUBSTITUTE
CM032587.1	1718148	.	T	C	100	*	TYPE=SUBSTITUTE
CM032587.1	1718163	.	C	T	100	*	TYPE=SUBSTITUTE
CM032587.1	1718166	.	A	C	100	*	TYPE=SUBSTITUTE
CM032587.1	1718181	.	G	A	100	*	TYPE=SUBSTITUTE
CM032587.1	1718256	.	T	C	100	*	TYPE=SUBSTITUTE
CM032587.1	1718277	.	A	T	100	*	TYPE=SUBSTITUTE
CM032587.1	1718283	.	C	T	100	*	TYPE=SUBSTITUTE
CM032587.1	1718319	.	T	C	100	*	TYPE=SUBSTITUTE
CM032587.1	1718352	.	T	c	100	*	TYPE=SUBSTITUTE
CM032587.1	1718372	.	C	A	100	*	TYPE=SUBSTITUTE
CM032587.1	1718409	.	C	T	100	*	TYPE=SUBSTITUTE
CM032587.1	1718433	.	T	C	100	*	TYPE=SUBSTITUTE
CM032587.1	1718442	.	C	T	100	*	TYPE=SUBSTITUTE
CM032587.1	1718469	.	C	A	100	*	TYPE=SUBSTITUTE
CM032587.1	1718529	.	A	G	100	*	TYPE=SUBSTITUTE
CM032587.1	1718544	.	A	T	100	*	TYPE=SUBSTITUTE
CM032587.1	1718604	.	C	G	100	*	TYPE=SUBSTITUTE
CM032587.1	1718622	.	C	T	100	*	TYPE=SUBSTITUTE
CM032587.1	1718706	.	G	A	100	*	TYPE=SUBSTITUTE
CM032587.1	1718714	.	C	T	100	*	TYPE=SUBSTITUTE
CM032587.1	1718715	.	T	C	100	*	TYPE=SUBSTITUTE
CM032587.1	1718718	.	C	G	100	*	TYPE=SUBSTITUTE
CM032587.1	1718733	.	G	A	100	*	TYPE=SUBSTITUTE
CM032587.1	1718773	.	C	G	100	*	TYPE=SUBSTITUTE
CM032587.1	1718778	.	G	A	100	*	TYPE=SUBSTITUTE
CM032587.1	1718802	.	G	A	100	*	TYPE=SUBSTITUTE
CM032587.1	1718809	.	A	T	100	*	TYPE=SUBSTITUTE
CM032587.1	1718934	.	G	C	100	*	TYPE=SUBSTITUTE
CM032587.1	1718961	.	C	A	100	*	TYPE=SUBSTITUTE
CM032587.1	1718997	.	C	T	100	*	TYPE=SUBSTITUTE
CM032587.1	1719072	.	G	A	100	*	TYPE=SUBSTITUTE
CM032587.1	1719150	.	A	C	100	*	TYPE=SUBSTITUTE
CM032587.1	1719219	.	A	ACAA	100	*	TYPE=INSERT
CM032587.1	1719232	.	A	C	100	*	TYPE=SUBSTITUTE
CM032587.1	1719254	.	T	C	100	*	TYPE=SUBSTITUTE
CM032587.1	1719312	.	A	C	100	*	TYPE=SUBSTITUTE
CM032587.1	1719324	.	C	T	100	*	TYPE=SUBSTITUTE
CM032587.1	1719330	.	G	A	100	*	TYPE=SUBSTITUTE
CM032587.1	1719334	.	C	T	100	*	TYPE=SUBSTITUTE
CM032587.1	1719352	.	A	G	100	*	TYPE=SUBSTITUTE
CM032587.1	1719354	.	C	T	100	*	TYPE=SUBSTITUTE
CM032587.1	1719355	.	T	C	100	*	TYPE=SUBSTITUTE
CM032587.1	1719369	.	C	t	100	*	TYPE=SUBSTITUTE
CM032587.1	1719371	.	A	t	100	*	TYPE=SUBSTITUTE
CM032587.1	1719381	.	C	a	100	*	TYPE=SUBSTITUTE
CM032587.1	1719382	.	c	caa	100	*	TYPE=INSERT
CM032587.1	1719394	.	C	CATCAATCCAATCAGCA	100	*	TYPE=INSERT
CM032587.1	1719399	.	A	AATCAGCAAAACATCAATCCAATCAGC	100	*	TYPE=INSERT
CM032587.1	1719423	.	A	C	100	*	TYPE=SUBSTITUTE
CM032587.1	1719461	.	C	T	100	*	TYPE=SUBSTITUTE
CM032587.1	1719468	.	T	TC	100	*	TYPE=INSERT
CM032587.1	1719475	.	G	A	100	*	TYPE=SUBSTITUTE
CM032587.1	1719483	.	T	G	100	*	TYPE=SUBSTITUTE
CM032587.1	1719493	.	T	TA	100	*	TYPE=INSERT
CM032587.1	1719504	.	G	A	100	*	TYPE=SUBSTITUTE
CM032587.1	1719515	.	C	CA	100	*	TYPE=INSERT
CM032587.1	1719519	.	T	C	100	*	TYPE=SUBSTITUTE
CM032587.1	1719521	.	CT	C	100	*	TYPE=DELETE
CM032587.1	1719542	.	C	A	100	*	TYPE=SUBSTITUTE
CM032587.1	1719544	.	A	G	100	*	TYPE=SUBSTITUTE
CM032587.1	1719545	.	G	A	100	*	TYPE=SUBSTITUTE
CM032587.1	1719548	.	A	C	100	*	TYPE=SUBSTITUTE

CM032587.1	1719553	.	A	T	100	*	TYPE=SUBSTITUTE
CM032587.1	1719561	.	A	C	100	*	TYPE=SUBSTITUTE
CM032587.1	1719581	.	A	G	100	*	TYPE=SUBSTITUTE
CM032587.1	1719589	.	AG	A	100	*	TYPE=DELETE
CM032587.1	1719615	.	A	G	100	*	TYPE=SUBSTITUTE
CM032587.1	1719623	.	A	G	100	*	TYPE=SUBSTITUTE
CM032587.1	1719624	.	G	A	100	*	TYPE=SUBSTITUTE
CM032587.1	1719628	.	T	C	100	*	TYPE=SUBSTITUTE
CM032587.1	1719660	.	T	TTCTTTCCGTCAGGG	100	*	TYPE=INSERT
CM032587.1	1719754	.	G	T	100	*	TYPE=SUBSTITUTE
CM032587.1	1719761	.	G	A	100	*	TYPE=SUBSTITUTE
CM032587.1	1719767	.	G	A	100	*	TYPE=SUBSTITUTE
CM032587.1	1719777	.	C	T	100	*	TYPE=SUBSTITUTE
CM032587.1	1719780	.	G	C	100	*	TYPE=SUBSTITUTE
CM032587.1	1719790	.	AT	A	100	*	TYPE=DELETE
CM032587.1	1719862	.	T	C	100	*	TYPE=SUBSTITUTE
CM032587.1	1719873	.	T	C	100	*	TYPE=SUBSTITUTE
CM032587.1	1719878	.	A	T	100	*	TYPE=SUBSTITUTE
CM032587.1	1719885	.	G	A	100	*	TYPE=SUBSTITUTE
CM032587.1	1719888	.	G	A	100	*	TYPE=SUBSTITUTE
CM032587.1	1719898	.	A	C	100	*	TYPE=SUBSTITUTE
CM032587.1	1719914	.	T	C	100	*	TYPE=SUBSTITUTE
CM032587.1	1719919	.	T	C	100	*	TYPE=SUBSTITUTE
CM032587.1	1719933	.	GA	G	100	*	TYPE=DELETE
CM032587.1	1719939	.	C	T	100	*	TYPE=SUBSTITUTE
CM032587.1	1719941	.	T	C	100	*	TYPE=SUBSTITUTE
CM032587.1	1719942	.	G	C	100	*	TYPE=SUBSTITUTE
CM032587.1	1719948	.	A	AC	100	*	TYPE=INSERT
CM032587.1	1719955	.	G	A	100	*	TYPE=SUBSTITUTE
CM032587.1	1719956	.	T	C	100	*	TYPE=SUBSTITUTE
CM032587.1	1719961	.	G	C	100	*	TYPE=SUBSTITUTE
CM032587.1	1720012	.	G	GC	100	*	TYPE=INSERT
CM032587.1	1720020	.	T	C	100	*	TYPE=SUBSTITUTE
CM032587.1	1720052	.	C	T	100	*	TYPE=SUBSTITUTE
CM032587.1	1720056	.	G	C	100	*	TYPE=SUBSTITUTE
CM032587.1	1720058	.	G	A	100	*	TYPE=SUBSTITUTE
CM032587.1	1720075	.	T	TC	100	*	TYPE=INSERT
CM032587.1	1720131	.	C	T	100	*	TYPE=SUBSTITUTE
CM032587.1	1720143	.	T	C	100	*	TYPE=SUBSTITUTE
CM032587.1	1720147	.	C	T	100	*	TYPE=SUBSTITUTE
CM032587.1	1720152	.	C	G	100	*	TYPE=SUBSTITUTE
CM032587.1	1720156	.	T	A	100	*	TYPE=SUBSTITUTE
CM032587.1	1720173	.	A	G	100	*	TYPE=SUBSTITUTE
CM032587.1	1720181	.	C	T	100	*	TYPE=SUBSTITUTE
CM032587.1	1720200	.	C	T	100	*	TYPE=SUBSTITUTE
CM032587.1	1720205	.	C	T	100	*	TYPE=SUBSTITUTE
CM032587.1	1720206	.	T	C	100	*	TYPE=SUBSTITUTE
CM032587.1	1720236	.	T	C	100	*	TYPE=SUBSTITUTE
CM032587.1	1720264	.	T	C	100	*	TYPE=SUBSTITUTE
CM032587.1	1720282	.	C	T	100	*	TYPE=SUBSTITUTE
CM032587.1	1720297	.	G	A	100	*	TYPE=SUBSTITUTE
CM032587.1	1720299	.	A	G	100	*	TYPE=SUBSTITUTE
CM032587.1	1720300	.	C	T	100	*	TYPE=SUBSTITUTE
CM032587.1	1720303	.	G	T	100	*	TYPE=SUBSTITUTE
CM032587.1	1720307	.	A	c	100	*	TYPE=SUBSTITUTE
CM032587.1	1720308	.	C	a	100	*	TYPE=SUBSTITUTE
CM032587.1	1720310	.	A	g	100	*	TYPE=SUBSTITUTE
CM032587.1	1720318	.	A	g	100	*	TYPE=SUBSTITUTE
CM032587.1	1720320	.	G	a	100	*	TYPE=SUBSTITUTE
CM032587.1	1720325	.	G	a	100	*	TYPE=SUBSTITUTE
CM032587.1	1720330	.	A	G	100	*	TYPE=SUBSTITUTE
CM032587.1	1720332	.	G	A	100	*	TYPE=SUBSTITUTE
CM032587.1	1720334	.	T	G	100	*	TYPE=SUBSTITUTE
CM032587.1	1720337	.	A	G	100	*	TYPE=SUBSTITUTE

CM032587.1	1720340	.	G	C	100	*	TYPE=SUBSTITUTE
CM032587.1	1720341	.	G	A	100	*	TYPE=SUBSTITUTE
CM032587.1	1720344	.	G	A	100	*	TYPE=SUBSTITUTE
CM032587.1	1720352	.	A	G	100	*	TYPE=SUBSTITUTE
CM032587.1	1720358	.	T	C	100	*	TYPE=SUBSTITUTE
CM032587.1	1720359	.	A	G	100	*	TYPE=SUBSTITUTE
CM032587.1	1720375	.	T	C	100	*	TYPE=SUBSTITUTE
CM032587.1	1720388	.	G	A	100	*	TYPE=SUBSTITUTE
CM032587.1	1720391	.	C	T	100	*	TYPE=SUBSTITUTE
CM032587.1	1720408	.	GTA	G	100	*	TYPE=DELETE
CM032587.1	1720411	.	T	C	100	*	TYPE=SUBSTITUTE
CM032587.1	1720417	.	T	C	100	*	TYPE=SUBSTITUTE
CM032587.1	1720433	.	C	G	100	*	TYPE=SUBSTITUTE
CM032587.1	1720447	.	A	T	100	*	TYPE=SUBSTITUTE
CM032587.1	1720451	.	A	G	100	*	TYPE=SUBSTITUTE
CM032587.1	1720457	.	A	G	100	*	TYPE=SUBSTITUTE
CM032587.1	1720470	.	G	A	100	*	TYPE=SUBSTITUTE
CM032587.1	1720478	.	GA	G	100	*	TYPE=DELETE
CM032587.1	1720485	.	A	G	100	*	TYPE=SUBSTITUTE
CM032587.1	1720495	.	T	A	100	*	TYPE=SUBSTITUTE
CM032587.1	1720505	.	T	C	100	*	TYPE=SUBSTITUTE
CM032587.1	1720511	.	A	G	100	*	TYPE=SUBSTITUTE
CM032587.1	1720523	.	T	C	100	*	TYPE=SUBSTITUTE
CM032587.1	1720531	.	T	C	100	*	TYPE=SUBSTITUTE
CM032587.1	1720539	.	C	T	100	*	TYPE=SUBSTITUTE
CM032587.1	1720543	.	C	T	100	*	TYPE=SUBSTITUTE
CM032587.1	1720550	.	T	A	100	*	TYPE=SUBSTITUTE
CM032587.1	1720566	.	A	G	100	*	TYPE=SUBSTITUTE
CM032587.1	1720574	.	T	c	100	*	TYPE=SUBSTITUTE
CM032587.1	1720577	.	G	a	100	*	TYPE=SUBSTITUTE
CM032587.1	1720586	.	C	T	100	*	TYPE=SUBSTITUTE
CM032587.1	1720600	.	G	C	100	*	TYPE=SUBSTITUTE
CM032587.1	1720601	.	G	T	100	*	TYPE=SUBSTITUTE
CM032587.1	1720611	.	G	c	100	*	TYPE=SUBSTITUTE
CM032587.1	1720617	.	G	a	100	*	TYPE=SUBSTITUTE
CM032587.1	1720620	.	G	a	100	*	TYPE=SUBSTITUTE
CM032587.1	1720624	.	A	g	100	*	TYPE=SUBSTITUTE
CM032587.1	1720635	.	T	C	100	*	TYPE=SUBSTITUTE
CM032587.1	1720641	.	C	T	100	*	TYPE=SUBSTITUTE
CM032587.1	1720649	.	T	A	100	*	TYPE=SUBSTITUTE
CM032587.1	1720651	.	C	T	100	*	TYPE=SUBSTITUTE
CM032587.1	1720657	.	T	TAGGTTTAA	100	*	TYPE=INSERT
CM032587.1	1720674	.	A	G	100	*	TYPE=SUBSTITUTE
CM032587.1	1720698	.	TA	T	100	*	TYPE=DELETE
CM032587.1	1720715	.	C	CTAGAGCAG	100	*	TYPE=INSERT
CM032587.1	1720751	.	A	C	100	*	TYPE=SUBSTITUTE
CM032587.1	1720775	.	CG	C	100	*	TYPE=DELETE
CM032587.1	1720833	.	CTAGG	C	100	*	TYPE=DELETE
CM032587.1	1720844	.	C	T	100	*	TYPE=SUBSTITUTE
CM032587.1	1720869	.	T	G	100	*	TYPE=SUBSTITUTE
CM032587.1	1720886	.	A	G	100	*	TYPE=SUBSTITUTE
CM032587.1	1720887	.	G	C	100	*	TYPE=SUBSTITUTE
CM032587.1	1720907	.	G	A	100	*	TYPE=SUBSTITUTE
CM032587.1	1720912	.	G	A	100	*	TYPE=SUBSTITUTE
CM032587.1	1720939	.	A	T	100	*	TYPE=SUBSTITUTE
CM032587.1	1720971	.	A	ACCTA	100	*	TYPE=INSERT
CM032587.1	1721005	.	T	c	100	*	TYPE=SUBSTITUTE
CM032587.1	1721100	.	T	c	100	*	TYPE=SUBSTITUTE
CM032587.1	1721106	.	T	c	100	*	TYPE=SUBSTITUTE
CM032587.1	1721121	.	G	A	100	*	TYPE=SUBSTITUTE
CM032587.1	1721124	.	T	C	100	*	TYPE=SUBSTITUTE
CM032587.1	1721136	.	C	G	100	*	TYPE=SUBSTITUTE
CM032587.1	1721154	.	G	A	100	*	TYPE=SUBSTITUTE
CM032587.1	1721157	.	A	T	100	*	TYPE=SUBSTITUTE

CM032587.1	1721160	.	T	A	100	*	TYPE=SUBSTITUTE
CM032587.1	1721181	.	A	g	100	*	TYPE=SUBSTITUTE
CM032587.1	1721246	.	A	G	100	*	TYPE=SUBSTITUTE
CM032587.1	1721249	.	C	T	100	*	TYPE=SUBSTITUTE
CM032587.1	1721256	.	T	C	100	*	TYPE=SUBSTITUTE
CM032587.1	1721288	.	G	A	100	*	TYPE=SUBSTITUTE
CM032587.1	1721349	.	G	A	100	*	TYPE=SUBSTITUTE
CM032587.1	1721418	.	T	C	100	*	TYPE=SUBSTITUTE
CM032587.1	1721517	.	G	A	100	*	TYPE=SUBSTITUTE
CM032587.1	1721589	.	A	T	100	*	TYPE=SUBSTITUTE
CM032587.1	1721592	.	C	T	100	*	TYPE=SUBSTITUTE
CM032587.1	1721598	.	T	C	100	*	TYPE=SUBSTITUTE
CM032587.1	1721619	.	C	G	100	*	TYPE=SUBSTITUTE
CM032587.1	1721646	.	G	T	100	*	TYPE=SUBSTITUTE
CM032587.1	1721655	.	A	G	100	*	TYPE=SUBSTITUTE
CM032587.1	1721667	.	A	G	100	*	TYPE=SUBSTITUTE
CM032587.1	1721688	.	C	T	100	*	TYPE=SUBSTITUTE
CM032587.1	1721712	.	C	G	100	*	TYPE=SUBSTITUTE
CM032587.1	1721727	.	T	C	100	*	TYPE=SUBSTITUTE
CM032587.1	1721748	.	C	G	100	*	TYPE=SUBSTITUTE
CM032587.1	1721751	.	T	A	100	*	TYPE=SUBSTITUTE
CM032587.1	1721760	.	T	C	100	*	TYPE=SUBSTITUTE
CM032587.1	1721787	.	T	G	100	*	TYPE=SUBSTITUTE
CM032587.1	1721793	.	G	A	100	*	TYPE=SUBSTITUTE
CM032587.1	1721794	.	A	G	100	*	TYPE=SUBSTITUTE
CM032587.1	1721823	.	G	A	100	*	TYPE=SUBSTITUTE
CM032587.1	1721826	.	G	A	100	*	TYPE=SUBSTITUTE
CM032587.1	1721841	.	A	G	100	*	TYPE=SUBSTITUTE
CM032587.1	1721857	.	T	G	100	*	TYPE=SUBSTITUTE
CM032587.1	1721858	.	A	T	100	*	TYPE=SUBSTITUTE
CM032587.1	1721859	.	G	A	100	*	TYPE=SUBSTITUTE
CM032587.1	1721877	.	G	A	100	*	TYPE=SUBSTITUTE
CM032587.1	1721889	.	A	C	100	*	TYPE=SUBSTITUTE
CM032587.1	1721943	.	T	C	100	*	TYPE=SUBSTITUTE
CM032587.1	1721958	.	G	A	100	*	TYPE=SUBSTITUTE
CM032587.1	1721962	.	A	G	100	*	TYPE=SUBSTITUTE
CM032587.1	1721973	.	C	T	100	*	TYPE=SUBSTITUTE
CM032587.1	1721997	.	G	A	100	*	TYPE=SUBSTITUTE
CM032587.1	1721998	.	A	G	100	*	TYPE=SUBSTITUTE
CM032587.1	1722012	.	A	g	100	*	TYPE=SUBSTITUTE
CM032587.1	1722018	.	A	g	100	*	TYPE=SUBSTITUTE
CM032587.1	1722030	.	A	T	100	*	TYPE=SUBSTITUTE
CM032587.1	1722042	.	G	A	100	*	TYPE=SUBSTITUTE
CM032587.1	1722060	.	G	A	100	*	TYPE=SUBSTITUTE
CM032587.1	1722063	.	T	G	100	*	TYPE=SUBSTITUTE
CM032587.1	1722066	.	A	G	100	*	TYPE=SUBSTITUTE
CM032587.1	1722082	.	A	G	100	*	TYPE=SUBSTITUTE
CM032587.1	1722135	.	G	A	100	*	TYPE=SUBSTITUTE
CM032587.1	1722150	.	C	G	100	*	TYPE=SUBSTITUTE
CM032587.1	1722156	.	G	A	100	*	TYPE=SUBSTITUTE
CM032587.1	1722159	.	G	A	100	*	TYPE=SUBSTITUTE
CM032587.1	1722306	.	G	C	100	*	TYPE=SUBSTITUTE
CM032587.1	1722318	.	G	A	100	*	TYPE=SUBSTITUTE
CM032587.1	1722339	.	G	A	100	*	TYPE=SUBSTITUTE
CM032587.1	1722345	.	G	A	100	*	TYPE=SUBSTITUTE
CM032587.1	1722369	.	G	A	100	*	TYPE=SUBSTITUTE
CM032587.1	1722426	.	G	A	100	*	TYPE=SUBSTITUTE
CM032587.1	1722448	.	A	G	100	*	TYPE=SUBSTITUTE
CM032587.1	1722490	.	A	G	100	*	TYPE=SUBSTITUTE
CM032587.1	1722513	.	A	G	100	*	TYPE=SUBSTITUTE
CM032587.1	1722540	.	A	G	100	*	TYPE=SUBSTITUTE
CM032587.1	1722570	.	A	C	100	*	TYPE=SUBSTITUTE
CM032587.1	1722597	.	A	T	100	*	TYPE=SUBSTITUTE
CM032587.1	1722601	.	T	A	100	*	TYPE=SUBSTITUTE

CM032587.1	1722603	.	A	G	100	*	TYPE=SUBSTITUTE
CM032587.1	1722639	.	A	G	100	*	TYPE=SUBSTITUTE
CM032587.1	1722648	.	T	G	100	*	TYPE=SUBSTITUTE
CM032587.1	1722657	.	A	G	100	*	TYPE=SUBSTITUTE
CM032587.1	1722678	.	G	C	100	*	TYPE=SUBSTITUTE
CM032587.1	1722693	.	T	C	100	*	TYPE=SUBSTITUTE
CM032587.1	1722765	.	G	A	100	*	TYPE=SUBSTITUTE
CM032587.1	1722777	.	A	G	100	*	TYPE=SUBSTITUTE
CM032587.1	1722789	.	C	T	100	*	TYPE=SUBSTITUTE
CM032587.1	1722826	.	T	C	100	*	TYPE=SUBSTITUTE
CM032587.1	1722879	.	A	G	100	*	TYPE=SUBSTITUTE
CM032587.1	1722885	.	G	A	100	*	TYPE=SUBSTITUTE
CM032587.1	1722888	.	C	T	100	*	TYPE=SUBSTITUTE
CM032587.1	1722897	.	T	C	100	*	TYPE=SUBSTITUTE
CM032587.1	1722919	.	G	A	100	*	TYPE=SUBSTITUTE
CM032587.1	1722945	.	T	C	100	*	TYPE=SUBSTITUTE
CM032587.1	1722972	.	C	T	100	*	TYPE=SUBSTITUTE
CM032587.1	1723047	.	A	G	100	*	TYPE=SUBSTITUTE
CM032587.1	1723050	.	A	G	100	*	TYPE=SUBSTITUTE
CM032587.1	1723065	.	C	G	100	*	TYPE=SUBSTITUTE
CM032587.1	1723074	.	T	C	100	*	TYPE=SUBSTITUTE
CM032587.1	1723080	.	A	G	100	*	TYPE=SUBSTITUTE
CM032587.1	1723083	.	G	A	100	*	TYPE=SUBSTITUTE
CM032587.1	1723134	.	G	A	100	*	TYPE=SUBSTITUTE
CM032587.1	1723143	.	A	G	100	*	TYPE=SUBSTITUTE
CM032587.1	1723164	.	A	G	100	*	TYPE=SUBSTITUTE
CM032587.1	1723167	.	A	G	100	*	TYPE=SUBSTITUTE
CM032587.1	1723218	.	G	A	100	*	TYPE=SUBSTITUTE
CM032587.1	1723257	.	C	T	100	*	TYPE=SUBSTITUTE
CM032587.1	1723263	.	A	G	100	*	TYPE=SUBSTITUTE
CM032587.1	1723353	.	A	G	100	*	TYPE=SUBSTITUTE
CM032587.1	1723401	.	T	C	100	*	TYPE=SUBSTITUTE
CM032587.1	1723408	.	C	T	100	*	TYPE=SUBSTITUTE
CM032587.1	1723443	.	G	A	100	*	TYPE=SUBSTITUTE
CM032587.1	1723467	.	A	G	100	*	TYPE=SUBSTITUTE
CM032587.1	1723554	.	G	T	100	*	TYPE=SUBSTITUTE
CM032587.1	1723575	.	A	G	100	*	TYPE=SUBSTITUTE
CM032587.1	1723584	.	A	G	100	*	TYPE=SUBSTITUTE
CM032587.1	1723608	.	G	A	100	*	TYPE=SUBSTITUTE
CM032587.1	1723632	.	T	C	100	*	TYPE=SUBSTITUTE
CM032587.1	1723665	.	G	C	100	*	TYPE=SUBSTITUTE
CM032587.1	1723713	.	T	c	100	*	TYPE=SUBSTITUTE
CM032587.1	1723722	.	A	g	100	*	TYPE=SUBSTITUTE
CM032587.1	1723737	.	G	A	100	*	TYPE=SUBSTITUTE
CM032587.1	1723755	.	G	A	100	*	TYPE=SUBSTITUTE
CM032587.1	1723761	.	G	C	100	*	TYPE=SUBSTITUTE
CM032587.1	1723821	.	C	T	100	*	TYPE=SUBSTITUTE
CM032587.1	1723827	.	A	G	100	*	TYPE=SUBSTITUTE
CM032587.1	1723839	.	G	A	100	*	TYPE=SUBSTITUTE
CM032587.1	1723845	.	C	T	100	*	TYPE=SUBSTITUTE
CM032587.1	1723860	.	T	C	100	*	TYPE=SUBSTITUTE
CM032587.1	1723872	.	A	G	100	*	TYPE=SUBSTITUTE
CM032587.1	1723902	.	A	G	100	*	TYPE=SUBSTITUTE
CM032587.1	1723920	.	G	C	100	*	TYPE=SUBSTITUTE
CM032587.1	1723923	.	A	G	100	*	TYPE=SUBSTITUTE
CM032587.1	1723932	.	G	A	100	*	TYPE=SUBSTITUTE
CM032587.1	1723941	.	G	A	100	*	TYPE=SUBSTITUTE
CM032587.1	1723948	.	A	G	100	*	TYPE=SUBSTITUTE
CM032587.1	1723992	.	T	c	100	*	TYPE=SUBSTITUTE
CM032587.1	1724010	.	T	C	100	*	TYPE=SUBSTITUTE
CM032587.1	1724022	.	C	T	100	*	TYPE=SUBSTITUTE
CM032587.1	1724034	.	T	C	100	*	TYPE=SUBSTITUTE
CM032587.1	1724052	.	A	G	100	*	TYPE=SUBSTITUTE
CM032587.1	1724064	.	A	G	100	*	TYPE=SUBSTITUTE

CM032587.1	1724065	.	G	T	100	*	TYPE=SUBSTITUTE
CM032587.1	1724067	.	T	C	100	*	TYPE=SUBSTITUTE
CM032587.1	1724085	.	C	A	100	*	TYPE=SUBSTITUTE
CM032587.1	1724121	.	C	T	100	*	TYPE=SUBSTITUTE
CM032587.1	1724127	.	C	T	100	*	TYPE=SUBSTITUTE
CM032587.1	1724143	.	A	G	100	*	TYPE=SUBSTITUTE
CM032587.1	1724145	.	T	C	100	*	TYPE=SUBSTITUTE
CM032587.1	1724163	.	T	C	100	*	TYPE=SUBSTITUTE
CM032587.1	1724172	.	T	C	100	*	TYPE=SUBSTITUTE
CM032587.1	1724199	.	A	G	100	*	TYPE=SUBSTITUTE
CM032587.1	1724220	.	G	T	100	*	TYPE=SUBSTITUTE
CM032587.1	1724268	.	A	G	100	*	TYPE=SUBSTITUTE
CM032587.1	1724301	.	T	C	100	*	TYPE=SUBSTITUTE
CM032587.1	1724352	.	A	G	100	*	TYPE=SUBSTITUTE
CM032587.1	1724390	.	TC	T	100	*	TYPE=DELETE
CM032587.1	1724437	.	C	T	100	*	TYPE=SUBSTITUTE
CM032587.1	1724441	.	T	C	100	*	TYPE=SUBSTITUTE
CM032587.1	1724444	.	G	T	100	*	TYPE=SUBSTITUTE
CM032587.1	1724479	.	G	A	100	*	TYPE=SUBSTITUTE
CM032587.1	1724506	.	C	T	100	*	TYPE=SUBSTITUTE
CM032587.1	1724567	.	G	a	100	*	TYPE=SUBSTITUTE
CM032587.1	1724569	.	T	c	100	*	TYPE=SUBSTITUTE
CM032587.1	1724626	.	C	T	100	*	TYPE=SUBSTITUTE
CM032587.1	1724629	.	A	G	100	*	TYPE=SUBSTITUTE
CM032587.1	1724644	.	C	T	100	*	TYPE=SUBSTITUTE
CM032587.1	1724653	.	G	A	100	*	TYPE=SUBSTITUTE
CM032587.1	1724707	.	G	A	100	*	TYPE=SUBSTITUTE
CM032587.1	1724756	.	G	A	100	*	TYPE=SUBSTITUTE
CM032587.1	1724761	.	C	CT	100	*	TYPE=INSERT
CM032587.1	1724778	.	CTA	C	100	*	TYPE=DELETE
CM032587.1	1724816	.	A	C	100	*	TYPE=SUBSTITUTE
CM032587.1	1724861	.	A	G	100	*	TYPE=SUBSTITUTE
CM032587.1	1724873	.	G	A	100	*	TYPE=SUBSTITUTE
CM032587.1	1724879	.	A	G	100	*	TYPE=SUBSTITUTE
CM032587.1	1724913	.	G	A	100	*	TYPE=SUBSTITUTE
CM032587.1	1724939	.	A	G	100	*	TYPE=SUBSTITUTE
CM032587.1	1724987	.	A	G	100	*	TYPE=SUBSTITUTE
CM032587.1	1724996	.	G	C	100	*	TYPE=SUBSTITUTE
CM032587.1	1725047	.	T	c	100	*	TYPE=SUBSTITUTE
CM032587.1	1725056	.	A	G	100	*	TYPE=SUBSTITUTE
CM032587.1	1725069	.	A	G	100	*	TYPE=SUBSTITUTE
CM032587.1	1725071	.	C	G	100	*	TYPE=SUBSTITUTE
CM032587.1	1725077	.	T	C	100	*	TYPE=SUBSTITUTE
CM032587.1	1725086	.	A	G	100	*	TYPE=SUBSTITUTE
CM032587.1	1725089	.	C	T	100	*	TYPE=SUBSTITUTE
CM032587.1	1725107	.	C	t	100	*	TYPE=SUBSTITUTE
CM032587.1	1725119	.	G	A	100	*	TYPE=SUBSTITUTE
CM032587.1	1725125	.	T	A	100	*	TYPE=SUBSTITUTE
CM032587.1	1725128	.	G	C	100	*	TYPE=SUBSTITUTE
CM032587.1	1725134	.	G	A	100	*	TYPE=SUBSTITUTE
CM032587.1	1725140	.	A	G	100	*	TYPE=SUBSTITUTE
CM032587.1	1725143	.	T	C	100	*	TYPE=SUBSTITUTE
CM032587.1	1725146	.	C	T	100	*	TYPE=SUBSTITUTE
CM032587.1	1725436	.	TT	T	100	*	TYPE=DELETE
CM032587.1	1725504	.	C	G	100	*	TYPE=SUBSTITUTE
CM032587.1	1725615	.	C	CT	100	*	TYPE=INSERT
CM032587.1	1725628	.	CA	C	100	*	TYPE=DELETE
CM032587.1	1725651	.	T	C	100	*	TYPE=SUBSTITUTE
CM032587.1	1725653	.	T	G	100	*	TYPE=SUBSTITUTE
CM032587.1	1725721	.	TGTATCGTACTACT	T	100	*	TYPE=DELETE
CM032587.1	1725751	.	G	GGA	100	*	TYPE=INSERT
CM032587.1	1725756	.	AT	A	100	*	TYPE=DELETE
CM032587.1	1725757	.	C	t	100	*	TYPE=SUBSTITUTE
CM032587.1	1725768	.	A	t	100	*	TYPE=SUBSTITUTE

CM032587.1	1725774	.	c	ct	100	*	TYPE=INSERT
CM032587.1	1725777	.	t	tca	100	*	TYPE=INSERT
CM032587.1	1725796	.	A	g	100	*	TYPE=SUBSTITUTE
CM032587.1	1725797	.	G	t	100	*	TYPE=SUBSTITUTE
CM032587.1	1725799	.	A	g	100	*	TYPE=SUBSTITUTE
CM032587.1	1725805	.	TGCTA	T	100	*	TYPE=DELETE
CM032587.1	1725818	.	TACTGA	T	100	*	TYPE=DELETE
CM032587.1	1725826	.	GCTGCCT	G	100	*	TYPE=DELETE
CM032587.1	1725866	.	T	G	100	*	TYPE=SUBSTITUTE
CM032587.1	1725896	.	C	T	100	*	TYPE=SUBSTITUTE
CM032587.1	1725930	.	T	TACG	100	*	TYPE=INSERT
CM032587.1	1725938	.	T	TA	100	*	TYPE=INSERT
CM032587.1	1725951	.	A	AT	100	*	TYPE=INSERT
CM032587.1	1725952	.	C	A	100	*	TYPE=SUBSTITUTE
CM032587.1	1725958	.	A	C	100	*	TYPE=SUBSTITUTE
CM032587.1	1725962	.	T	A	100	*	TYPE=SUBSTITUTE
CM032587.1	1725964	.	C	A	100	*	TYPE=SUBSTITUTE
CM032587.1	1725965	.	A	C	100	*	TYPE=SUBSTITUTE
CM032587.1	1725966	.	A	T	100	*	TYPE=SUBSTITUTE
CM032587.1	1725967	.	C	T	100	*	TYPE=SUBSTITUTE
CM032587.1	1725968	.	A	G	100	*	TYPE=SUBSTITUTE
CM032587.1	1725970	.	C	T	100	*	TYPE=SUBSTITUTE
CM032587.1	1725971	.	A	G	100	*	TYPE=SUBSTITUTE
CM032587.1	1725972	.	A	T	100	*	TYPE=SUBSTITUTE
CM032587.1	1725973	.	A	C	100	*	TYPE=SUBSTITUTE
CM032587.1	1725975	.	A	T	100	*	TYPE=SUBSTITUTE
CM032587.1	1725978	.	TT	T	100	*	TYPE=DELETE
CM032587.1	1726012	.	G	GAG	100	*	TYPE=INSERT
CM032587.1	1726041	.	A	acgcaa	100	*	TYPE=INSERT
CM032587.1	1726077	.	A	G	100	*	TYPE=SUBSTITUTE
CM032587.1	1726095	.	c	ct	100	*	TYPE=INSERT
CM032587.1	1726099	.	A	g	100	*	TYPE=SUBSTITUTE
CM032587.1	1726100	.	C	g	100	*	TYPE=SUBSTITUTE
CM032587.1	1726104	.	T	g	100	*	TYPE=SUBSTITUTE
CM032587.1	1726105	.	T	a	100	*	TYPE=SUBSTITUTE
CM032587.1	1726106	.	C	t	100	*	TYPE=SUBSTITUTE
CM032587.1	1726110	.	C	T	100	*	TYPE=SUBSTITUTE
CM032587.1	1726111	.	A	AT	100	*	TYPE=INSERT
CM032587.1	1726113	.	C	G	100	*	TYPE=SUBSTITUTE
CM032587.1	1726115	.	A	T	100	*	TYPE=SUBSTITUTE
CM032587.1	1726116	.	T	G	100	*	TYPE=SUBSTITUTE
CM032587.1	1726117	.	T	G	100	*	TYPE=SUBSTITUTE
CM032587.1	1726118	.	A	C	100	*	TYPE=SUBSTITUTE
CM032587.1	1726121	.	C	T	100	*	TYPE=SUBSTITUTE
CM032587.1	1726123	.	T	A	100	*	TYPE=SUBSTITUTE
CM032587.1	1726218	.	T	C	100	*	TYPE=SUBSTITUTE
CM032587.1	1726253	.	C	CT	100	*	TYPE=INSERT
CM032587.1	1726287	.	T	C	100	*	TYPE=SUBSTITUTE
CM032587.1	1726291	.	A	T	100	*	TYPE=SUBSTITUTE
CM032587.1	1726293	.	C	A	100	*	TYPE=SUBSTITUTE
CM032587.1	1726297	.	CGTA	C	100	*	TYPE=DELETE
CM032587.1	1726329	.	T	C	100	*	TYPE=SUBSTITUTE
CM032587.1	1726332	.	C	T	100	*	TYPE=SUBSTITUTE
CM032587.1	1726337	.	G	GAG	100	*	TYPE=INSERT
CM032587.1	1726393	.	G	A	100	*	TYPE=SUBSTITUTE
CM032587.1	1726487	.	TTCC	T	100	*	TYPE=DELETE
CM032587.1	1726495	.	G	GTGC	100	*	TYPE=INSERT
CM032587.1	1726506	.	G	A	100	*	TYPE=SUBSTITUTE
CM032587.1	1726523	.	G	c	100	*	TYPE=SUBSTITUTE
CM032587.1	1726538	.	TTCCTCT	T	100	*	TYPE=DELETE
CM032587.1	1726569	.	T	C	100	*	TYPE=SUBSTITUTE
CM032587.1	1726573	.	T	A	100	*	TYPE=SUBSTITUTE
CM032587.1	1726619	.	G	C	100	*	TYPE=SUBSTITUTE
CM032587.1	1726672	.	T	TT	100	*	TYPE=INSERT

CM032587.1	1726723	.	C	CAC	100	*	TYPE=INSERT
CM032587.1	1726746	.	TG	T	100	*	TYPE=DELETE
CM032587.1	1726752	.	G	c	100	*	TYPE=SUBSTITUTE
CM032587.1	1726753	.	C	g	100	*	TYPE=SUBSTITUTE
CM032587.1	1726754	.	A	T	100	*	TYPE=SUBSTITUTE
CM032587.1	1726788	.	C	T	100	*	TYPE=SUBSTITUTE
CM032587.1	1726789	.	T	C	100	*	TYPE=SUBSTITUTE
CM032587.1	1726790	.	C	T	100	*	TYPE=SUBSTITUTE
CM032587.1	1726891	.	C	A	100	*	TYPE=SUBSTITUTE
CM032587.1	1726904	.	CCAGCTTTCCCAT	C	100	*	TYPE=DELETE
CM032587.1	1726946	.	CCAA	C	100	*	TYPE=DELETE
CM032587.1	1726956	.	G	GA	100	*	TYPE=INSERT
CM032587.1	1726962	.	A	t	100	*	TYPE=SUBSTITUTE
CM032587.1	1726969	.	A	g	100	*	TYPE=SUBSTITUTE
CM032587.1	1726971	.	A	g	100	*	TYPE=SUBSTITUTE
CM032587.1	1726994	.	A	T	100	*	TYPE=SUBSTITUTE
CM032587.1	1726996	.	A	G	100	*	TYPE=SUBSTITUTE
CM032587.1	1727002	.	A	G	100	*	TYPE=SUBSTITUTE
CM032587.1	1727021	.	A	G	100	*	TYPE=SUBSTITUTE
CM032587.1	1727027	.	A	G	100	*	TYPE=SUBSTITUTE
CM032587.1	1727040	.	ATATG	A	100	*	TYPE=DELETE
CM032587.1	1727052	.	g	ga	100	*	TYPE=INSERT
CM032587.1	1727056	.	G	A	100	*	TYPE=SUBSTITUTE
CM032587.1	1727058	.	T	A	100	*	TYPE=SUBSTITUTE
CM032587.1	1727059	.	C	G	100	*	TYPE=SUBSTITUTE
CM032587.1	1727060	.	G	T	100	*	TYPE=SUBSTITUTE
CM032587.1	1727066	.	ATAG	A	100	*	TYPE=DELETE
CM032587.1	1727071	.	G	A	100	*	TYPE=SUBSTITUTE
CM032587.1	1727080	.	A	G	100	*	TYPE=SUBSTITUTE
CM032587.1	1727084	.	A	G	100	*	TYPE=SUBSTITUTE
CM032587.1	1727087	.	AC	A	100	*	TYPE=DELETE
CM032587.1	1727093	.	C	T	100	*	TYPE=SUBSTITUTE
CM032587.1	1727099	.	A	G	100	*	TYPE=SUBSTITUTE
CM032587.1	1727100	.	C	T	100	*	TYPE=SUBSTITUTE
CM032587.1	1727111	.	c	ctt	100	*	TYPE=INSERT
CM032587.1	1727130	.	GTT	G	100	*	TYPE=DELETE
CM032587.1	1727131	.	T	C	100	*	TYPE=SUBSTITUTE
CM032587.1	1727140	.	G	T	100	*	TYPE=SUBSTITUTE
CM032587.1	1727144	.	G	A	100	*	TYPE=SUBSTITUTE
CM032587.1	1727150	.	TGATG	T	100	*	TYPE=DELETE
CM032587.1	1727168	.	T	G	100	*	TYPE=SUBSTITUTE
CM032587.1	1727192	.	A	G	100	*	TYPE=SUBSTITUTE
CM032587.1	1727198	.	A	G	100	*	TYPE=SUBSTITUTE
CM032587.1	1727201	.	A	G	100	*	TYPE=SUBSTITUTE
CM032587.1	1727213	.	T	G	100	*	TYPE=SUBSTITUTE
CM032587.1	1727225	.	G	A	100	*	TYPE=SUBSTITUTE
CM032587.1	1727228	.	A	G	100	*	TYPE=SUBSTITUTE
CM032587.1	1727276	.	G	C	100	*	TYPE=SUBSTITUTE
CM032587.1	1727303	.	T	C	100	*	TYPE=SUBSTITUTE
CM032587.1	1727335	.	C	T	100	*	TYPE=SUBSTITUTE
CM032587.1	1727337	.	G	A	100	*	TYPE=SUBSTITUTE
CM032587.1	1727338	.	G	A	100	*	TYPE=SUBSTITUTE
CM032587.1	1727347	.	C	CCGATGTGTTCCCCATGACCTCTCAAGCGAGA	100	*	TYPE=INSERT
CM032587.1	1727356	.	T	C	100	*	TYPE=SUBSTITUTE
CM032587.1	1727359	.	C	T	100	*	TYPE=SUBSTITUTE
CM032587.1	1727362	.	A	G	100	*	TYPE=SUBSTITUTE
CM032587.1	1727363	.	G	A	100	*	TYPE=SUBSTITUTE
CM032587.1	1727375	.	A	g	100	*	TYPE=SUBSTITUTE
CM032587.1	1727377	.	C	g	100	*	TYPE=SUBSTITUTE
CM032587.1	1727392	.	c	caa	100	*	TYPE=INSERT
CM032587.1	1727401	.	GA	G	100	*	TYPE=DELETE
CM032587.1	1727430	.	G	a	100	*	TYPE=SUBSTITUTE
CM032587.1	1727434	.	C	t	100	*	TYPE=SUBSTITUTE

CM032587.1	1727448	.	A	G	100	*	TYPE=SUBSTITUTE
CM032587.1	1727457	.	C	G	100	*	TYPE=SUBSTITUTE
CM032587.1	1727502	.	C	T	100	*	TYPE=SUBSTITUTE
CM032587.1	1727524	.	T	C	100	*	TYPE=SUBSTITUTE
CM032587.1	1727525	.	C	G	100	*	TYPE=SUBSTITUTE
CM032587.1	1727536	.	G	A	100	*	TYPE=SUBSTITUTE
CM032587.1	1727545	.	C	T	100	*	TYPE=SUBSTITUTE
CM032587.1	1727548	.	G	A	100	*	TYPE=SUBSTITUTE
CM032587.1	1727561	.	G	C	100	*	TYPE=SUBSTITUTE
CM032587.1	1727564	.	C	T	100	*	TYPE=SUBSTITUTE
CM032587.1	1727568	.	A	G	100	*	TYPE=SUBSTITUTE
CM032587.1	1727569	.	G	A	100	*	TYPE=SUBSTITUTE
CM032587.1	1727571	.	A	G	100	*	TYPE=SUBSTITUTE
CM032587.1	1727574	.	T	C	100	*	TYPE=SUBSTITUTE
CM032587.1	1727580	.	G	A	100	*	TYPE=SUBSTITUTE
CM032587.1	1727596	.	C	T	100	*	TYPE=SUBSTITUTE
CM032587.1	1727603	.	T	A	100	*	TYPE=SUBSTITUTE
CM032587.1	1727611	.	C	T	100	*	TYPE=SUBSTITUTE
CM032587.1	1727620	.	T	C	100	*	TYPE=SUBSTITUTE
CM032587.1	1727660	.	A	T	100	*	TYPE=SUBSTITUTE
CM032587.1	1727663	.	G	A	100	*	TYPE=SUBSTITUTE
CM032587.1	1727669	.	C	T	100	*	TYPE=SUBSTITUTE
CM032587.1	1727673	.	A	G	100	*	TYPE=SUBSTITUTE
CM032587.1	1727704	.	ACCCCAAAAGGGAGATAGTAGTTTGATAAA	A	100	*	TYPE=DELETE
CM032587.1	1727725	.	T	C	100	*	TYPE=SUBSTITUTE
CM032587.1	1727753	.	G	A	100	*	TYPE=SUBSTITUTE
CM032587.1	1727761	.	AA	A	100	*	TYPE=DELETE
CM032587.1	1727797	.	T	C	100	*	TYPE=SUBSTITUTE
CM032587.1	1727823	.	A	T	100	*	TYPE=SUBSTITUTE
CM032587.1	1727841	.	C	A	100	*	TYPE=SUBSTITUTE
CM032587.1	1727848	.	G	A	100	*	TYPE=SUBSTITUTE
CM032587.1	1727854	.	A	G	100	*	TYPE=SUBSTITUTE
CM032587.1	1727860	.	C	T	100	*	TYPE=SUBSTITUTE
CM032587.1	1727873	.	T	G	100	*	TYPE=SUBSTITUTE
CM032587.1	1727878	.	G	A	100	*	TYPE=SUBSTITUTE
CM032587.1	1727880	.	G	A	100	*	TYPE=SUBSTITUTE
CM032587.1	1727895	.	A	T	100	*	TYPE=SUBSTITUTE
CM032587.1	1727896	.	A	T	100	*	TYPE=SUBSTITUTE
CM032587.1	1727898	.	C	G	100	*	TYPE=SUBSTITUTE
CM032587.1	1727899	.	A	AAGAGACGGGAGAC	100	*	TYPE=INSERT
CM032587.1	1727903	.	G	A	100	*	TYPE=SUBSTITUTE
CM032587.1	1727906	.	A	T	100	*	TYPE=SUBSTITUTE
CM032587.1	1727908	.	A	C	100	*	TYPE=SUBSTITUTE
CM032587.1	1727919	.	T	A	100	*	TYPE=SUBSTITUTE
CM032587.1	1727921	.	G	A	100	*	TYPE=SUBSTITUTE
CM032587.1	1727929	.	T	A	100	*	TYPE=SUBSTITUTE
CM032587.1	1727931	.	G	T	100	*	TYPE=SUBSTITUTE
CM032587.1	1727946	.	C	T	100	*	TYPE=SUBSTITUTE
CM032587.1	1727947	.	A	G	100	*	TYPE=SUBSTITUTE
CM032587.1	1727948	.	C	T	100	*	TYPE=SUBSTITUTE
CM032587.1	1727950	.	A	G	100	*	TYPE=SUBSTITUTE
CM032587.1	1727960	.	G	A	100	*	TYPE=SUBSTITUTE
CM032587.1	1727965	.	A	G	100	*	TYPE=SUBSTITUTE
CM032587.1	1727971	.	T	C	100	*	TYPE=SUBSTITUTE
CM032587.1	1727976	.	G	T	100	*	TYPE=SUBSTITUTE
CM032587.1	1727978	.	C	T	100	*	TYPE=SUBSTITUTE
CM032587.1	1727987	.	A	C	100	*	TYPE=SUBSTITUTE
CM032587.1	1727994	.	C	T	100	*	TYPE=SUBSTITUTE
CM032587.1	1727997	.	C	T	100	*	TYPE=SUBSTITUTE
CM032587.1	1728000	.	T	C	100	*	TYPE=SUBSTITUTE
CM032587.1	1728001	.	A	G	100	*	TYPE=SUBSTITUTE
CM032587.1	1728019	.	G	A	100	*	TYPE=SUBSTITUTE
CM032587.1	1728024	.	T	C	100	*	TYPE=SUBSTITUTE

CM032587.1	1728058	.	T	G	100	*	TYPE=SUBSTITUTE
CM032587.1	1728061	.	A	G	100	*	TYPE=SUBSTITUTE
CM032587.1	1728064	.	G	A	100	*	TYPE=SUBSTITUTE
CM032587.1	1728087	.	G	C	100	*	TYPE=SUBSTITUTE
CM032587.1	1728132	.	A	G	100	*	TYPE=SUBSTITUTE
CM032587.1	1728166	.	G	A	100	*	TYPE=SUBSTITUTE
CM032587.1	1728173	.	C	G	100	*	TYPE=SUBSTITUTE
CM032587.1	1728209	.	T	G	100	*	TYPE=SUBSTITUTE
CM032587.1	1728210	.	C	T	100	*	TYPE=SUBSTITUTE
CM032587.1	1728216	.	G	A	100	*	TYPE=SUBSTITUTE
CM032587.1	1728224	.	T	C	100	*	TYPE=SUBSTITUTE
CM032587.1	1728228	.	T	A	100	*	TYPE=SUBSTITUTE
CM032587.1	1728229	.	T	C	100	*	TYPE=SUBSTITUTE
CM032587.1	1728250	.	C	T	100	*	TYPE=SUBSTITUTE
CM032587.1	1728268	.	T	G	100	*	TYPE=SUBSTITUTE
CM032587.1	1728269	.	A	G	100	*	TYPE=SUBSTITUTE
CM032587.1	1728277	.	A	G	100	*	TYPE=SUBSTITUTE
CM032587.1	1728278	.	G	A	100	*	TYPE=SUBSTITUTE
CM032587.1	1728284	.	T	A	100	*	TYPE=SUBSTITUTE
CM032587.1	1728285	.	T	A	100	*	TYPE=SUBSTITUTE
CM032587.1	1728287	.	A	C	100	*	TYPE=SUBSTITUTE
CM032587.1	1728290	.	A	AG	100	*	TYPE=INSERT
CM032587.1	1728292	.	G	A	100	*	TYPE=SUBSTITUTE
CM032587.1	1728300	.	G	A	100	*	TYPE=SUBSTITUTE
CM032587.1	1728302	.	A	G	100	*	TYPE=SUBSTITUTE
CM032587.1	1728306	.	CA	C	100	*	TYPE=DELETE
CM032587.1	1728311	.	A	G	100	*	TYPE=SUBSTITUTE
CM032587.1	1728312	.	T	C	100	*	TYPE=SUBSTITUTE
CM032587.1	1728351	.	G	A	100	*	TYPE=SUBSTITUTE
CM032587.1	1728369	.	T	G	100	*	TYPE=SUBSTITUTE
CM032587.1	1728372	.	G	A	100	*	TYPE=SUBSTITUTE
CM032587.1	1728375	.	G	C	100	*	TYPE=SUBSTITUTE
CM032587.1	1728377	.	C	T	100	*	TYPE=SUBSTITUTE
CM032587.1	1728378	.	T	C	100	*	TYPE=SUBSTITUTE
CM032587.1	1728388	.	A	G	100	*	TYPE=SUBSTITUTE
CM032587.1	1728432	.	T	C	100	*	TYPE=SUBSTITUTE
CM032587.1	1728441	.	A	T	100	*	TYPE=SUBSTITUTE
CM032587.1	1728464	.	A	G	100	*	TYPE=SUBSTITUTE
CM032587.1	1728486	.	G	a	100	*	TYPE=SUBSTITUTE
CM032587.1	1728487	.	A	g	100	*	TYPE=SUBSTITUTE
CM032587.1	1728492	.	A	g	100	*	TYPE=SUBSTITUTE
CM032587.1	1728507	.	C	T	100	*	TYPE=SUBSTITUTE
CM032587.1	1728516	.	C	T	100	*	TYPE=SUBSTITUTE
CM032587.1	1728519	.	G	A	100	*	TYPE=SUBSTITUTE
CM032587.1	1728528	.	G	C	100	*	TYPE=SUBSTITUTE
CM032587.1	1728537	.	T	C	100	*	TYPE=SUBSTITUTE
CM032587.1	1728666	.	G	A	100	*	TYPE=SUBSTITUTE
CM032587.1	1728669	.	G	A	100	*	TYPE=SUBSTITUTE
CM032587.1	1728678	.	T	C	100	*	TYPE=SUBSTITUTE
CM032587.1	1728729	.	T	C	100	*	TYPE=SUBSTITUTE
CM032587.1	1728752	.	G	A	100	*	TYPE=SUBSTITUTE
CM032587.1	1728771	.	T	C	100	*	TYPE=SUBSTITUTE
CM032587.1	1728816	.	A	G	100	*	TYPE=SUBSTITUTE
CM032587.1	1728846	.	G	T	100	*	TYPE=SUBSTITUTE
CM032587.1	1728870	.	A	G	100	*	TYPE=SUBSTITUTE
CM032587.1	1728897	.	C	T	100	*	TYPE=SUBSTITUTE
CM032587.1	1728906	.	G	a	100	*	TYPE=SUBSTITUTE
CM032587.1	1728931	.	T	C	100	*	TYPE=SUBSTITUTE
CM032587.1	1728978	.	G	A	100	*	TYPE=SUBSTITUTE
CM032587.1	1728999	.	C	t	100	*	TYPE=SUBSTITUTE
CM032587.1	1729015	.	G	a	100	*	TYPE=SUBSTITUTE
CM032587.1	1729020	.	A	g	100	*	TYPE=SUBSTITUTE
CM032587.1	1729061	.	A	G	100	*	TYPE=SUBSTITUTE
CM032587.1	1729064	.	A	G	100	*	TYPE=SUBSTITUTE

CM032587.1	1729248	.	A	G	100	*	TYPE=SUBSTITUTE
CM032587.1	1729353	.	A	C	100	*	TYPE=SUBSTITUTE
CM032587.1	1729452	.	G	A	100	*	TYPE=SUBSTITUTE
CM032587.1	1729465	.	G	A	100	*	TYPE=SUBSTITUTE
CM032587.1	1729494	.	G	A	100	*	TYPE=SUBSTITUTE
CM032587.1	1729530	.	T	C	100	*	TYPE=SUBSTITUTE
CM032587.1	1729533	.	G	A	100	*	TYPE=SUBSTITUTE
CM032587.1	1729554	.	T	C	100	*	TYPE=SUBSTITUTE
CM032587.1	1729569	.	C	T	100	*	TYPE=SUBSTITUTE
CM032587.1	1729644	.	C	T	100	*	TYPE=SUBSTITUTE
CM032587.1	1729684	.	AG	A	100	*	TYPE=DELETE
CM032587.1	1729692	.	A	T	100	*	TYPE=SUBSTITUTE
CM032587.1	1729735	.	A	T	100	*	TYPE=SUBSTITUTE
CM032587.1	1729737	.	C	T	100	*	TYPE=SUBSTITUTE
CM032587.1	1729789	.	G	A	100	*	TYPE=SUBSTITUTE
CM032587.1	1729796	.	C	T	100	*	TYPE=SUBSTITUTE
CM032587.1	1729802	.	T	C	100	*	TYPE=SUBSTITUTE
CM032587.1	1729811	.	C	T	100	*	TYPE=SUBSTITUTE
CM032587.1	1729833	.	A	T	100	*	TYPE=SUBSTITUTE
CM032587.1	1729840	.	ACTGTCT	A	100	*	TYPE=DELETE
CM032587.1	1729846	.	C	T	100	*	TYPE=SUBSTITUTE
CM032587.1	1729899	.	A	G	100	*	TYPE=SUBSTITUTE
CM032587.1	1729907	.	G	A	100	*	TYPE=SUBSTITUTE
CM032587.1	1729916	.	A	C	100	*	TYPE=SUBSTITUTE
CM032587.1	1729922	.	G	A	100	*	TYPE=SUBSTITUTE
CM032587.1	1729923	.	G	A	100	*	TYPE=SUBSTITUTE
CM032587.1	1729937	.	C	G	100	*	TYPE=SUBSTITUTE
CM032587.1	1729946	.	TA	T	100	*	TYPE=DELETE
CM032587.1	1729949	.	T	C	100	*	TYPE=SUBSTITUTE
CM032587.1	1729982	.	C	T	100	*	TYPE=SUBSTITUTE
CM032587.1	1730054	.	TG	T	100	*	TYPE=DELETE
CM032587.1	1730059	.	T	C	100	*	TYPE=SUBSTITUTE
CM032587.1	1730067	.	A	T	100	*	TYPE=SUBSTITUTE
CM032587.1	1730071	.	GT	G	100	*	TYPE=DELETE
CM032587.1	1730115	.	A	T	100	*	TYPE=SUBSTITUTE
CM032587.1	1730146	.	C	G	100	*	TYPE=SUBSTITUTE
CM032587.1	1730160	.	A	G	100	*	TYPE=SUBSTITUTE
CM032587.1	1730162	.	AC	A	100	*	TYPE=DELETE
CM032587.1	1730181	.	A	T	100	*	TYPE=SUBSTITUTE
CM032587.1	1730186	.	C	CC	100	*	TYPE=INSERT
CM032587.1	1730219	.	T	G	100	*	TYPE=SUBSTITUTE
CM032587.1	1730264	.	A	G	100	*	TYPE=SUBSTITUTE
CM032587.1	1730299	.	A	g	100	*	TYPE=SUBSTITUTE
CM032587.1	1730364	.	A	T	100	*	TYPE=SUBSTITUTE
CM032587.1	1730391	.	G	a	100	*	TYPE=SUBSTITUTE
CM032587.1	1730394	.	AGG	A	100	*	TYPE=DELETE
CM032587.1	1730398	.	A	g	100	*	TYPE=SUBSTITUTE
CM032587.1	1730436	.	G	A	100	*	TYPE=SUBSTITUTE
CM032587.1	1730502	.	G	A	100	*	TYPE=SUBSTITUTE
CM032587.1	1730503	.	T	C	100	*	TYPE=SUBSTITUTE
CM032587.1	1730642	.	A	T	100	*	TYPE=SUBSTITUTE
CM032587.1	1730684	.	A	G	100	*	TYPE=SUBSTITUTE
CM032587.1	1730760	.	A	G	100	*	TYPE=SUBSTITUTE
CM032587.1	1730769	.	G	A	100	*	TYPE=SUBSTITUTE
CM032587.1	1730996	.	G	A	100	*	TYPE=SUBSTITUTE
CM032587.1	1731104	.	C	T	100	*	TYPE=SUBSTITUTE
CM032587.1	1731272	.	A	G	100	*	TYPE=SUBSTITUTE
CM032587.1	1731323	.	A	G	100	*	TYPE=SUBSTITUTE
CM032587.1	1731365	.	T	A	100	*	TYPE=SUBSTITUTE
CM032587.1	1731461	.	G	C	100	*	TYPE=SUBSTITUTE
CM032587.1	1731506	.	G	C	100	*	TYPE=SUBSTITUTE
CM032587.1	1731543	.	C	T	100	*	TYPE=SUBSTITUTE
CM032587.1	1731551	.	A	G	100	*	TYPE=SUBSTITUTE
CM032587.1	1731599	.	G	A	100	*	TYPE=SUBSTITUTE

CM032587.1	1731627	.	T	C	100	*	TYPE=SUBSTITUTE
CM032587.1	1731629	.	G	T	100	*	TYPE=SUBSTITUTE
CM032587.1	1731638	.	G	A	100	*	TYPE=SUBSTITUTE
CM032587.1	1731665	.	G	a	100	*	TYPE=SUBSTITUTE
CM032587.1	1731713	.	G	C	100	*	TYPE=SUBSTITUTE
CM032587.1	1731719	.	G	A	100	*	TYPE=SUBSTITUTE
CM032587.1	1731749	.	G	A	100	*	TYPE=SUBSTITUTE
CM032587.1	1731872	.	A	G	100	*	TYPE=SUBSTITUTE
CM032587.1	1731903	.	A	AG	100	*	TYPE=INSERT
CM032587.1	1731911	.	C	G	100	*	TYPE=SUBSTITUTE
CM032587.1	1731914	.	A	T	100	*	TYPE=SUBSTITUTE
CM032587.1	1731915	.	A	G	100	*	TYPE=SUBSTITUTE
CM032587.1	1731916	.	T	C	100	*	TYPE=SUBSTITUTE
CM032587.1	1731925	.	A	C	100	*	TYPE=SUBSTITUTE
CM032587.1	1731937	.	A	AGTTT	100	*	TYPE=INSERT
CM032587.1	1731940	.	T	G	100	*	TYPE=SUBSTITUTE
CM032587.1	1731941	.	G	T	100	*	TYPE=SUBSTITUTE
CM032587.1	1731943	.	T	A	100	*	TYPE=SUBSTITUTE
CM032587.1	1731948	.	G	A	100	*	TYPE=SUBSTITUTE
CM032587.1	1731993	.	G	A	100	*	TYPE=SUBSTITUTE
CM032587.1	1732041	.	CC	C	100	*	TYPE=DELETE
CM032587.1	1732066	.	A	AA	100	*	TYPE=INSERT
CM032587.1	1732086	.	T	A	100	*	TYPE=SUBSTITUTE
CM032587.1	1732094	.	A	G	100	*	TYPE=SUBSTITUTE
CM032587.1	1732195	.	AA	A	100	*	TYPE=DELETE
CM032587.1	1732232	.	C	T	100	*	TYPE=SUBSTITUTE
CM032587.1	1732242	.	A	G	100	*	TYPE=SUBSTITUTE
CM032587.1	1732243	.	G	C	100	*	TYPE=SUBSTITUTE
CM032587.1	1732258	.	AGGT	A	100	*	TYPE=DELETE
CM032587.1	1732269	.	ACCT	A	100	*	TYPE=DELETE
CM032587.1	1732313	.	A	G	100	*	TYPE=SUBSTITUTE
CM032587.1	1732341	.	T	A	100	*	TYPE=SUBSTITUTE
CM032587.1	1732346	.	A	G	100	*	TYPE=SUBSTITUTE
CM032587.1	1732347	.	T	TCGCGAGTTTACTCGTA	100	*	TYPE=INSERT
CM032587.1	1732352	.	G	A	100	*	TYPE=SUBSTITUTE
CM032587.1	1732353	.	T	A	100	*	TYPE=SUBSTITUTE
CM032587.1	1732369	.	G	A	100	*	TYPE=SUBSTITUTE
CM032587.1	1732414	.	T	TT	100	*	TYPE=INSERT
CM032587.1	1732452	.	T	TGCTCCAGG	100	*	TYPE=INSERT
CM032587.1	1732465	.	T	G	100	*	TYPE=SUBSTITUTE
CM032587.1	1732531	.	TCCTGGCATG	T	100	*	TYPE=DELETE
CM032587.1	1732536	.	A	T	100	*	TYPE=SUBSTITUTE
CM032587.1	1732538	.	T	C	100	*	TYPE=SUBSTITUTE
CM032587.1	1732543	.	G	C	100	*	TYPE=SUBSTITUTE
CM032587.1	1732544	.	T	A	100	*	TYPE=SUBSTITUTE
CM032587.1	1732564	.	G	A	100	*	TYPE=SUBSTITUTE
CM032587.1	1732565	.	C	G	100	*	TYPE=SUBSTITUTE
CM032587.1	1732572	.	A	C	100	*	TYPE=SUBSTITUTE
CM032587.1	1732587	.	G	C	100	*	TYPE=SUBSTITUTE
CM032587.1	1732592	.	CCC	C	100	*	TYPE=DELETE
CM032587.1	1732654	.	C	T	100	*	TYPE=SUBSTITUTE
CM032587.1	1732674	.	T	C	100	*	TYPE=SUBSTITUTE
CM032587.1	1732891	.	A	G	100	*	TYPE=SUBSTITUTE
CM032587.1	1732913	.	A	G	100	*	TYPE=SUBSTITUTE
CM032587.1	1732933	.	C	T	100	*	TYPE=SUBSTITUTE
CM032587.1	1732955	.	T	C	100	*	TYPE=SUBSTITUTE
CM032587.1	1732970	.	T	C	100	*	TYPE=SUBSTITUTE
CM032587.1	1732974	.	G	A	100	*	TYPE=SUBSTITUTE
CM032587.1	1732977	.	A	G	100	*	TYPE=SUBSTITUTE
CM032587.1	1732980	.	A	C	100	*	TYPE=SUBSTITUTE
CM032587.1	1732983	.	T	C	100	*	TYPE=SUBSTITUTE
CM032587.1	1732986	.	G	A	100	*	TYPE=SUBSTITUTE
CM032587.1	1732989	.	G	C	100	*	TYPE=SUBSTITUTE
CM032587.1	1733001	.	G	A	100	*	TYPE=SUBSTITUTE

CM032587.1	1733049	.	G	A	100	*	TYPE=SUBSTITUTE
CM032587.1	1733061	.	T	C	100	*	TYPE=SUBSTITUTE
CM032587.1	1733289	.	TG	T	100	*	TYPE=DELETE
CM032587.1	1733413	.	A	G	100	*	TYPE=SUBSTITUTE
CM032587.1	1733443	.	T	C	100	*	TYPE=SUBSTITUTE
CM032587.1	1733475	.	A	G	100	*	TYPE=SUBSTITUTE
CM032587.1	1733492	.	C	T	100	*	TYPE=SUBSTITUTE
CM032587.1	1733524	.	A	G	100	*	TYPE=SUBSTITUTE
CM032587.1	1733527	.	G	A	100	*	TYPE=SUBSTITUTE
CM032587.1	1733594	.	G	T	100	*	TYPE=SUBSTITUTE
CM032587.1	1733596	.	G	A	100	*	TYPE=SUBSTITUTE
CM032587.1	1733609	.	T	C	100	*	TYPE=SUBSTITUTE
CM032587.1	1733611	.	T	A	100	*	TYPE=SUBSTITUTE
CM032587.1	1733612	.	G	A	100	*	TYPE=SUBSTITUTE
CM032587.1	1733616	.	G	C	100	*	TYPE=SUBSTITUTE
CM032587.1	1733620	.	A	G	100	*	TYPE=SUBSTITUTE
CM032587.1	1733622	.	T	A	100	*	TYPE=SUBSTITUTE
CM032587.1	1733722	.	A	G	100	*	TYPE=SUBSTITUTE
CM032587.1	1733737	.	A	G	100	*	TYPE=SUBSTITUTE
CM032587.1	1733764	.	C	T	100	*	TYPE=SUBSTITUTE
CM032587.1	1733803	.	G	A	100	*	TYPE=SUBSTITUTE
CM032587.1	1733806	.	A	G	100	*	TYPE=SUBSTITUTE
CM032587.1	1733818	.	A	G	100	*	TYPE=SUBSTITUTE
CM032587.1	1733845	.	G	A	100	*	TYPE=SUBSTITUTE
CM032587.1	1733941	.	A	G	100	*	TYPE=SUBSTITUTE
CM032587.1	1733968	.	A	G	100	*	TYPE=SUBSTITUTE
CM032587.1	1733975	.	G	A	100	*	TYPE=SUBSTITUTE
CM032587.1	1733992	.	G	A	100	*	TYPE=SUBSTITUTE
CM032587.1	1734001	.	T	C	100	*	TYPE=SUBSTITUTE
CM032587.1	1734007	.	C	T	100	*	TYPE=SUBSTITUTE
CM032587.1	1734095	.	T	c	100	*	TYPE=SUBSTITUTE
CM032587.1	1734101	.	T	c	100	*	TYPE=SUBSTITUTE
CM032587.1	1734130	.	G	A	100	*	TYPE=SUBSTITUTE
CM032587.1	1734181	.	C	T	100	*	TYPE=SUBSTITUTE
CM032587.1	1734220	.	A	G	100	*	TYPE=SUBSTITUTE
CM032587.1	1734236	.	G	C	100	*	TYPE=SUBSTITUTE
CM032587.1	1734262	.	C	A	100	*	TYPE=SUBSTITUTE
CM032587.1	1734263	.	A	AAACCAA	100	*	TYPE=INSERT
CM032587.1	1734280	.	T	C	100	*	TYPE=SUBSTITUTE
CM032587.1	1734298	.	TC	T	100	*	TYPE=DELETE
CM032587.1	1734308	.	T	TA	100	*	TYPE=INSERT
CM032587.1	1734312	.	AA	A	100	*	TYPE=DELETE
CM032587.1	1734392	.	A	G	100	*	TYPE=SUBSTITUTE
CM032587.1	1734415	.	C	T	100	*	TYPE=SUBSTITUTE
CM032587.1	1734416	.	G	A	100	*	TYPE=SUBSTITUTE
CM032587.1	1734432	.	C	T	100	*	TYPE=SUBSTITUTE
CM032587.1	1734450	.	G	A	100	*	TYPE=SUBSTITUTE
CM032587.1	1734486	.	G	GAA	100	*	TYPE=INSERT
CM032587.1	1734488	.	A	G	100	*	TYPE=SUBSTITUTE
CM032587.1	1734492	.	C	CA	100	*	TYPE=INSERT
CM032587.1	1734503	.	A	G	100	*	TYPE=SUBSTITUTE
CM032587.1	1734504	.	T	C	100	*	TYPE=SUBSTITUTE
CM032587.1	1734509	.	A	G	100	*	TYPE=SUBSTITUTE
CM032587.1	1734561	.	A	G	100	*	TYPE=SUBSTITUTE
CM032587.1	1734576	.	G	A	100	*	TYPE=SUBSTITUTE
CM032587.1	1734579	.	C	G	100	*	TYPE=SUBSTITUTE
CM032587.1	1734580	.	C	G	100	*	TYPE=SUBSTITUTE
CM032587.1	1734606	.	C	CCAA	100	*	TYPE=INSERT
CM032587.1	1734620	.	GA	G	100	*	TYPE=DELETE
CM032587.1	1734623	.	T	C	100	*	TYPE=SUBSTITUTE
CM032587.1	1734627	.	T	C	100	*	TYPE=SUBSTITUTE
CM032587.1	1734638	.	T	C	100	*	TYPE=SUBSTITUTE
CM032587.1	1734639	.	C	T	100	*	TYPE=SUBSTITUTE
CM032587.1	1734644	.	C	T	100	*	TYPE=SUBSTITUTE

CM032587.1	1734649	.	C	G	100	*	TYPE=SUBSTITUTE
CM032587.1	1734650	.	G	A	100	*	TYPE=SUBSTITUTE
CM032587.1	1734667	.	C	T	100	*	TYPE=SUBSTITUTE
CM032587.1	1734672	.	G	T	100	*	TYPE=SUBSTITUTE
CM032587.1	1734690	.	T	C	100	*	TYPE=SUBSTITUTE
CM032587.1	1734691	.	A	C	100	*	TYPE=SUBSTITUTE
CM032587.1	1734692	.	A	T	100	*	TYPE=SUBSTITUTE
CM032587.1	1734696	.	A	G	100	*	TYPE=SUBSTITUTE
CM032587.1	1734698	.	C	CGACCCTG	100	*	TYPE=INSERT
CM032587.1	1734702	.	T	TGGATGCGCTCC	100	*	TYPE=INSERT
CM032587.1	1734708	.	T	G	100	*	TYPE=SUBSTITUTE
CM032587.1	1734728	.	T	TC	100	*	TYPE=INSERT
CM032587.1	1734754	.	T	G	100	*	TYPE=SUBSTITUTE
CM032587.1	1734756	.	C	CGT	100	*	TYPE=INSERT
CM032587.1	1734759	.	G	GAA	100	*	TYPE=INSERT
CM032587.1	1734768	.	G	T	100	*	TYPE=SUBSTITUTE
CM032587.1	1734780	.	T	C	100	*	TYPE=SUBSTITUTE
CM032587.1	1734785	.	A	G	100	*	TYPE=SUBSTITUTE
CM032587.1	1734793	.	A	G	100	*	TYPE=SUBSTITUTE
CM032587.1	1734795	.	G	C	100	*	TYPE=SUBSTITUTE
CM032587.1	1734803	.	T	C	100	*	TYPE=SUBSTITUTE
CM032587.1	1734818	.	A	G	100	*	TYPE=SUBSTITUTE
CM032587.1	1734821	.	A	G	100	*	TYPE=SUBSTITUTE
CM032587.1	1734830	.	A	C	100	*	TYPE=SUBSTITUTE
CM032587.1	1734832	.	CG	C	100	*	TYPE=DELETE
CM032587.1	1734833	.	G	A	100	*	TYPE=SUBSTITUTE
CM032587.1	1734834	.	G	T	100	*	TYPE=SUBSTITUTE
CM032587.1	1734835	.	C	A	100	*	TYPE=SUBSTITUTE
CM032587.1	1734836	.	C	A	100	*	TYPE=SUBSTITUTE
CM032587.1	1734837	.	G	T	100	*	TYPE=SUBSTITUTE
CM032587.1	1734838	.	C	A	100	*	TYPE=SUBSTITUTE
CM032587.1	1734839	.	A	G	100	*	TYPE=SUBSTITUTE
CM032587.1	1734840	.	T	C	100	*	TYPE=SUBSTITUTE
CM032587.1	1734841	.	G	C	100	*	TYPE=SUBSTITUTE
CM032587.1	1734860	.	G	A	100	*	TYPE=SUBSTITUTE
CM032587.1	1734872	.	A	G	100	*	TYPE=SUBSTITUTE
CM032587.1	1734876	.	C	T	100	*	TYPE=SUBSTITUTE
CM032587.1	1734888	.	AACC	A	100	*	TYPE=DELETE
CM032587.1	1734892	.	A	C	100	*	TYPE=SUBSTITUTE
CM032587.1	1734895	.	C	G	100	*	TYPE=SUBSTITUTE
CM032587.1	1734896	.	C	G	100	*	TYPE=SUBSTITUTE
CM032587.1	1734897	.	T	A	100	*	TYPE=SUBSTITUTE
CM032587.1	1734899	.	AC	A	100	*	TYPE=DELETE
CM032587.1	1734901	.	G	C	100	*	TYPE=SUBSTITUTE
CM032587.1	1734902	.	C	G	100	*	TYPE=SUBSTITUTE
CM032587.1	1734903	.	C	T	100	*	TYPE=SUBSTITUTE
CM032587.1	1734904	.	T	G	100	*	TYPE=SUBSTITUTE
CM032587.1	1734909	.	G	T	100	*	TYPE=SUBSTITUTE
CM032587.1	1734910	.	G	C	100	*	TYPE=SUBSTITUTE
CM032587.1	1734913	.	T	A	100	*	TYPE=SUBSTITUTE
CM032587.1	1734917	.	A	AACATATTTCATCCAT	100	*	TYPE=INSERT
CM032587.1	1734921	.	C	A	100	*	TYPE=SUBSTITUTE
CM032587.1	1734922	.	G	T	100	*	TYPE=SUBSTITUTE
CM032587.1	1734924	.	A	C	100	*	TYPE=SUBSTITUTE
CM032587.1	1734943	.	A	AT	100	*	TYPE=INSERT
CM032587.1	1734953	.	T	C	100	*	TYPE=SUBSTITUTE
CM032587.1	1734955	.	C	T	100	*	TYPE=SUBSTITUTE
CM032587.1	1735014	.	TTACCTG	T	100	*	TYPE=DELETE
CM032587.1	1735016	.	A	T	100	*	TYPE=SUBSTITUTE
CM032587.1	1735020	.	A	G	100	*	TYPE=SUBSTITUTE
CM032587.1	1735036	.	C	T	100	*	TYPE=SUBSTITUTE
CM032587.1	1735042	.	AT	A	100	*	TYPE=DELETE
CM032587.1	1735044	.	T	G	100	*	TYPE=SUBSTITUTE
CM032587.1	1735055	.	C	T	100	*	TYPE=SUBSTITUTE

CM032587.1	1735119	.	C	T	100	*	TYPE=SUBSTITUTE
CM032587.1	1735120	.	T	C	100	*	TYPE=SUBSTITUTE
CM032587.1	1735121	.	T	C	100	*	TYPE=SUBSTITUTE
CM032587.1	1735201	.	T	A	100	*	TYPE=SUBSTITUTE
CM032587.1	1735202	.	A	G	100	*	TYPE=SUBSTITUTE
CM032587.1	1735205	.	G	A	100	*	TYPE=SUBSTITUTE
CM032587.1	1735218	.	T	C	100	*	TYPE=SUBSTITUTE
CM032587.1	1735220	.	C	G	100	*	TYPE=SUBSTITUTE
CM032587.1	1735221	.	G	C	100	*	TYPE=SUBSTITUTE
CM032587.1	1735223	.	A	G	100	*	TYPE=SUBSTITUTE
CM032587.1	1735229	.	G	A	100	*	TYPE=SUBSTITUTE
CM032587.1	1735232	.	G	A	100	*	TYPE=SUBSTITUTE
CM032587.1	1735234	.	C	T	100	*	TYPE=SUBSTITUTE
CM032587.1	1735239	.	C	T	100	*	TYPE=SUBSTITUTE
CM032587.1	1735250	.	T	C	100	*	TYPE=SUBSTITUTE
CM032587.1	1735270	.	C	T	100	*	TYPE=SUBSTITUTE
CM032587.1	1735275	.	C	T	100	*	TYPE=SUBSTITUTE
CM032587.1	1735290	.	TTG	T	100	*	TYPE=DELETE
CM032587.1	1735292	.	A	g	100	*	TYPE=SUBSTITUTE
CM032587.1	1735313	.	A	G	100	*	TYPE=SUBSTITUTE
CM032587.1	1735330	.	G	C	100	*	TYPE=SUBSTITUTE
CM032587.1	1735344	.	A	G	100	*	TYPE=SUBSTITUTE
CM032587.1	1735351	.	A	G	100	*	TYPE=SUBSTITUTE
CM032587.1	1735354	.	C	CGTA	100	*	TYPE=INSERT
CM032587.1	1735355	.	A	T	100	*	TYPE=SUBSTITUTE
CM032587.1	1735362	.	G	A	100	*	TYPE=SUBSTITUTE
CM032587.1	1735380	.	T	TAC	100	*	TYPE=INSERT
CM032587.1	1735383	.	G	A	100	*	TYPE=SUBSTITUTE
CM032587.1	1735385	.	G	GATGATCC	100	*	TYPE=INSERT
CM032587.1	1735399	.	T	G	100	*	TYPE=SUBSTITUTE
CM032587.1	1735415	.	CT	C	100	*	TYPE=DELETE
CM032587.1	1735424	.	T	TTGG	100	*	TYPE=INSERT
CM032587.1	1735430	.	G	A	100	*	TYPE=SUBSTITUTE
CM032587.1	1735431	.	T	C	100	*	TYPE=SUBSTITUTE
CM032587.1	1735432	.	A	G	100	*	TYPE=SUBSTITUTE
CM032587.1	1735435	.	G	A	100	*	TYPE=SUBSTITUTE
CM032587.1	1735436	.	C	CAG	100	*	TYPE=INSERT
CM032587.1	1735441	.	A	AACTACG	100	*	TYPE=INSERT
CM032587.1	1735451	.	C	CT	100	*	TYPE=INSERT
CM032587.1	1735455	.	C	T	100	*	TYPE=SUBSTITUTE
CM032587.1	1735465	.	A	G	100	*	TYPE=SUBSTITUTE
CM032587.1	1735466	.	T	A	100	*	TYPE=SUBSTITUTE
CM032587.1	1735469	.	T	A	100	*	TYPE=SUBSTITUTE
CM032587.1	1735471	.	T	C	100	*	TYPE=SUBSTITUTE
CM032587.1	1735472	.	G	A	100	*	TYPE=SUBSTITUTE
CM032587.1	1735477	.	AA	A	100	*	TYPE=DELETE
CM032587.1	1735498	.	C	T	100	*	TYPE=SUBSTITUTE
CM032587.1	1735501	.	C	CC	100	*	TYPE=INSERT
CM032587.1	1735523	.	C	CC	100	*	TYPE=INSERT
CM032587.1	1735723	.	G	GG	100	*	TYPE=INSERT
CM032587.1	1735750	.	T	A	100	*	TYPE=SUBSTITUTE
CM032587.1	1735751	.	C	A	100	*	TYPE=SUBSTITUTE
CM032587.1	1735752	.	T	C	100	*	TYPE=SUBSTITUTE
CM032587.1	1735754	.	C	CACAG	100	*	TYPE=INSERT
CM032587.1	1735755	.	T	C	100	*	TYPE=SUBSTITUTE
CM032587.1	1735757	.	C	T	100	*	TYPE=SUBSTITUTE
CM032587.1	1735759	.	G	T	100	*	TYPE=SUBSTITUTE
CM032587.1	1735767	.	t	tt	100	*	TYPE=INSERT
CM032587.1	1735782	.	C	T	100	*	TYPE=SUBSTITUTE
CM032587.1	1735784	.	C	CTT	100	*	TYPE=INSERT
CM032587.1	1735809	.	T	C	100	*	TYPE=SUBSTITUTE
CM032587.1	1735819	.	A	G	100	*	TYPE=SUBSTITUTE
CM032587.1	1735830	.	TGCTG	T	100	*	TYPE=DELETE
CM032587.1	1735833	.	T	C	100	*	TYPE=SUBSTITUTE

CM032587.1	1735836	.	C	T	100	*	TYPE=SUBSTITUTE
CM032587.1	1735842	.	C	T	100	*	TYPE=SUBSTITUTE
CM032587.1	1735852	.	G	C	100	*	TYPE=SUBSTITUTE
CM032587.1	1735889	.	C	G	100	*	TYPE=SUBSTITUTE
CM032587.1	1735890	.	A	g	100	*	TYPE=SUBSTITUTE
CM032587.1	1735891	.	T	a	100	*	TYPE=SUBSTITUTE
CM032587.1	1735898	.	C	a	100	*	TYPE=SUBSTITUTE
CM032587.1	1735899	.	T	g	100	*	TYPE=SUBSTITUTE
CM032587.1	1735907	.	C	t	100	*	TYPE=SUBSTITUTE
CM032587.1	1735909	.	A	g	100	*	TYPE=SUBSTITUTE
CM032587.1	1735913	.	C	CT	100	*	TYPE=INSERT
CM032587.1	1735922	.	A	G	100	*	TYPE=SUBSTITUTE
CM032587.1	1735954	.	T	C	100	*	TYPE=SUBSTITUTE
CM032587.1	1735956	.	TT	T	100	*	TYPE=DELETE
CM032587.1	1735997	.	A	AGATG	100	*	TYPE=INSERT
CM032587.1	1736058	.	A	G	100	*	TYPE=SUBSTITUTE
CM032587.1	1736067	.	G	C	100	*	TYPE=SUBSTITUTE
CM032587.1	1736068	.	C	CT	100	*	TYPE=INSERT
CM032587.1	1736104	.	CT	C	100	*	TYPE=DELETE
CM032587.1	1736105	.	T	A	100	*	TYPE=SUBSTITUTE
CM032587.1	1736106	.	T	A	100	*	TYPE=SUBSTITUTE
CM032587.1	1736107	.	T	C	100	*	TYPE=SUBSTITUTE
CM032587.1	1736140	.	A	G	100	*	TYPE=SUBSTITUTE
CM032587.1	1736141	.	G	A	100	*	TYPE=SUBSTITUTE
CM032587.1	1736142	.	T	A	100	*	TYPE=SUBSTITUTE
CM032587.1	1736144	.	C	T	100	*	TYPE=SUBSTITUTE
CM032587.1	1736212	.	A	G	100	*	TYPE=SUBSTITUTE
CM032587.1	1736237	.	T	G	100	*	TYPE=SUBSTITUTE
CM032587.1	1736250	.	A	G	100	*	TYPE=SUBSTITUTE
CM032587.1	1736321	.	G	A	100	*	TYPE=SUBSTITUTE
CM032587.1	1736371	.	T	C	100	*	TYPE=SUBSTITUTE
CM032587.1	1736385	.	C	T	100	*	TYPE=SUBSTITUTE
CM032587.1	1736423	.	T	C	100	*	TYPE=SUBSTITUTE
CM032587.1	1736425	.	T	TC	100	*	TYPE=INSERT
CM032587.1	1736433	.	A	G	100	*	TYPE=SUBSTITUTE
CM032587.1	1736436	.	A	C	100	*	TYPE=SUBSTITUTE
CM032587.1	1736471	.	C	ccaatc	100	*	TYPE=INSERT
CM032587.1	1736505	.	T	C	100	*	TYPE=SUBSTITUTE
CM032587.1	1736507	.	G	T	100	*	TYPE=SUBSTITUTE
CM032587.1	1736508	.	G	A	100	*	TYPE=SUBSTITUTE
CM032587.1	1736509	.	A	G	100	*	TYPE=SUBSTITUTE
CM032587.1	1736566	.	A	G	100	*	TYPE=SUBSTITUTE
CM032587.1	1736585	.	A	C	100	*	TYPE=SUBSTITUTE
CM032587.1	1736606	.	C	T	100	*	TYPE=SUBSTITUTE
CM032587.1	1736654	.	C	T	100	*	TYPE=SUBSTITUTE
CM032587.1	1736698	.	G	A	100	*	TYPE=SUBSTITUTE
CM032587.1	1736765	.	A	C	100	*	TYPE=SUBSTITUTE
CM032587.1	1736766	.	G	A	100	*	TYPE=SUBSTITUTE
CM032587.1	1736768	.	A	G	100	*	TYPE=SUBSTITUTE
CM032587.1	1736782	.	G	A	100	*	TYPE=SUBSTITUTE
CM032587.1	1736799	.	G	C	100	*	TYPE=SUBSTITUTE
CM032587.1	1736802	.	G	T	100	*	TYPE=SUBSTITUTE
CM032587.1	1736810	.	G	C	100	*	TYPE=SUBSTITUTE
CM032587.1	1736812	.	T	A	100	*	TYPE=SUBSTITUTE
CM032587.1	1736813	.	C	CGA	100	*	TYPE=INSERT
CM032587.1	1736814	.	C	T	100	*	TYPE=SUBSTITUTE
CM032587.1	1736953	.	A	G	100	*	TYPE=SUBSTITUTE
CM032587.1	1737078	.	T	A	100	*	TYPE=SUBSTITUTE
CM032587.1	1737079	.	C	T	100	*	TYPE=SUBSTITUTE
CM032587.1	1737085	.	G	C	100	*	TYPE=SUBSTITUTE
CM032587.1	1737086	.	A	T	100	*	TYPE=SUBSTITUTE
CM032587.1	1737125	.	AT	A	100	*	TYPE=DELETE
CM032587.1	1737224	.	AA	A	100	*	TYPE=DELETE
CM032587.1	1737314	.	T	a	100	*	TYPE=SUBSTITUTE

CM032587.1	1737315	.	T	c	100	*	TYPE=SUBSTITUTE
CM032587.1	1737323	.	T	c	100	*	TYPE=SUBSTITUTE
CM032587.1	1737325	.	TT	T	100	*	TYPE=DELETE
CM032587.1	1737396	.	A	G	100	*	TYPE=SUBSTITUTE
CM032587.1	1737399	.	G	GC	100	*	TYPE=INSERT
CM032587.1	1737403	.	A	C	100	*	TYPE=SUBSTITUTE
CM032587.1	1737404	.	A	T	100	*	TYPE=SUBSTITUTE
CM032587.1	1737428	.	T	C	100	*	TYPE=SUBSTITUTE
CM032587.1	1737429	.	G	A	100	*	TYPE=SUBSTITUTE
CM032587.1	1737431	.	A	T	100	*	TYPE=SUBSTITUTE
CM032587.1	1737437	.	ATG	A	100	*	TYPE=DELETE
CM032587.1	1737439	.	A	T	100	*	TYPE=SUBSTITUTE
CM032587.1	1737486	.	A	C	100	*	TYPE=SUBSTITUTE
CM032587.1	1737722	.	C	T	100	*	TYPE=SUBSTITUTE
CM032587.1	1737759	.	G	A	100	*	TYPE=SUBSTITUTE
CM032587.1	1737798	.	G	A	100	*	TYPE=SUBSTITUTE
CM032587.1	1737819	.	A	G	100	*	TYPE=SUBSTITUTE
CM032587.1	1737855	.	G	A	100	*	TYPE=SUBSTITUTE
CM032587.1	1737884	.	A	G	100	*	TYPE=SUBSTITUTE
CM032587.1	1737893	.	T	C	100	*	TYPE=SUBSTITUTE
CM032587.1	1737911	.	G	C	100	*	TYPE=SUBSTITUTE
CM032587.1	1737912	.	A	G	100	*	TYPE=SUBSTITUTE
CM032587.1	1737954	.	G	T	100	*	TYPE=SUBSTITUTE
CM032587.1	1738008	.	G	a	100	*	TYPE=SUBSTITUTE
CM032587.1	1738055	.	T	A	100	*	TYPE=SUBSTITUTE
CM032587.1	1738057	.	C	T	100	*	TYPE=SUBSTITUTE
CM032587.1	1738069	.	AA	A	100	*	TYPE=DELETE
CM032587.1	1738086	.	G	A	100	*	TYPE=SUBSTITUTE
CM032587.1	1738101	.	G	A	100	*	TYPE=SUBSTITUTE
CM032587.1	1738191	.	G	A	100	*	TYPE=SUBSTITUTE
CM032587.1	1738215	.	G	A	100	*	TYPE=SUBSTITUTE
CM032587.1	1738227	.	A	G	100	*	TYPE=SUBSTITUTE
CM032587.1	1738233	.	C	T	100	*	TYPE=SUBSTITUTE
CM032587.1	1738237	.	T	G	100	*	TYPE=SUBSTITUTE
CM032587.1	1738238	.	A	T	100	*	TYPE=SUBSTITUTE
CM032587.1	1738239	.	G	A	100	*	TYPE=SUBSTITUTE
CM032587.1	1738251	.	G	T	100	*	TYPE=SUBSTITUTE
CM032587.1	1738290	.	G	A	100	*	TYPE=SUBSTITUTE
CM032587.1	1738335	.	A	C	100	*	TYPE=SUBSTITUTE
CM032587.1	1738356	.	T	A	100	*	TYPE=SUBSTITUTE
CM032587.1	1738467	.	G	A	100	*	TYPE=SUBSTITUTE
CM032587.1	1738479	.	A	G	100	*	TYPE=SUBSTITUTE
CM032587.1	1738497	.	G	A	100	*	TYPE=SUBSTITUTE
CM032587.1	1738509	.	G	A	100	*	TYPE=SUBSTITUTE
CM032587.1	1738625	.	A	G	100	*	TYPE=SUBSTITUTE
CM032587.1	1738875	.	G	A	100	*	TYPE=SUBSTITUTE
CM032587.1	1739112	.	A	G	100	*	TYPE=SUBSTITUTE
CM032587.1	1739133	.	C	T	100	*	TYPE=SUBSTITUTE
CM032587.1	1739202	.	G	A	100	*	TYPE=SUBSTITUTE
CM032587.1	1739211	.	G	A	100	*	TYPE=SUBSTITUTE
CM032587.1	1739467	.	A	G	100	*	TYPE=SUBSTITUTE
CM032587.1	1739478	.	G	A	100	*	TYPE=SUBSTITUTE
CM032587.1	1739847	.	C	T	100	*	TYPE=SUBSTITUTE
CM032587.1	1739865	.	G	A	100	*	TYPE=SUBSTITUTE
CM032587.1	1739988	.	G	A	100	*	TYPE=SUBSTITUTE
CM032587.1	1740066	.	A	G	100	*	TYPE=SUBSTITUTE
CM032587.1	1740102	.	A	G	100	*	TYPE=SUBSTITUTE
CM032587.1	1740117	.	T	C	100	*	TYPE=SUBSTITUTE
CM032587.1	1740138	.	G	A	100	*	TYPE=SUBSTITUTE
CM032587.1	1740243	.	G	A	100	*	TYPE=SUBSTITUTE
CM032587.1	1740351	.	G	A	100	*	TYPE=SUBSTITUTE
CM032587.1	1740417	.	C	A	100	*	TYPE=SUBSTITUTE
CM032587.1	1740588	.	T	G	100	*	TYPE=SUBSTITUTE
CM032587.1	1740609	.	T	G	100	*	TYPE=SUBSTITUTE

CM032587.1	1740630	.	T	G	100	*	TYPE=SUBSTITUTE
CM032587.1	1740636	.	T	G	100	*	TYPE=SUBSTITUTE
CM032587.1	1740645	.	C	G	100	*	TYPE=SUBSTITUTE
CM032587.1	1740651	.	A	G	100	*	TYPE=SUBSTITUTE
CM032587.1	1740660	.	G	A	100	*	TYPE=SUBSTITUTE
CM032587.1	1740669	.	A	G	100	*	TYPE=SUBSTITUTE
CM032587.1	1740750	.	A	G	100	*	TYPE=SUBSTITUTE
CM032587.1	1740783	.	A	C	100	*	TYPE=SUBSTITUTE
CM032587.1	1740792	.	G	A	100	*	TYPE=SUBSTITUTE
CM032587.1	1740802	.	A	G	100	*	TYPE=SUBSTITUTE
CM032587.1	1740816	.	C	T	100	*	TYPE=SUBSTITUTE
CM032587.1	1740822	.	A	G	100	*	TYPE=SUBSTITUTE
CM032587.1	1740825	.	A	T	100	*	TYPE=SUBSTITUTE
CM032587.1	1740831	.	T	C	100	*	TYPE=SUBSTITUTE
CM032587.1	1740834	.	C	G	100	*	TYPE=SUBSTITUTE
CM032587.1	1740837	.	A	G	100	*	TYPE=SUBSTITUTE
CM032587.1	1740840	.	T	A	100	*	TYPE=SUBSTITUTE
CM032587.1	1740876	.	T	C	100	*	TYPE=SUBSTITUTE
CM032587.1	1740885	.	G	A	100	*	TYPE=SUBSTITUTE
CM032587.1	1740897	.	A	G	100	*	TYPE=SUBSTITUTE
CM032587.1	1740945	.	G	A	100	*	TYPE=SUBSTITUTE
CM032587.1	1740957	.	G	A	100	*	TYPE=SUBSTITUTE
CM032587.1	1740982	.	C	T	100	*	TYPE=SUBSTITUTE
CM032587.1	1741023	.	C	T	100	*	TYPE=SUBSTITUTE
CM032587.1	1741038	.	A	G	100	*	TYPE=SUBSTITUTE
CM032587.1	1741125	.	A	G	100	*	TYPE=SUBSTITUTE
CM032587.1	1741137	.	G	A	100	*	TYPE=SUBSTITUTE
CM032587.1	1741167	.	G	T	100	*	TYPE=SUBSTITUTE
CM032587.1	1741206	.	A	G	100	*	TYPE=SUBSTITUTE
CM032587.1	1741212	.	C	T	100	*	TYPE=SUBSTITUTE
CM032587.1	1741233	.	T	C	100	*	TYPE=SUBSTITUTE
CM032587.1	1741242	.	C	T	100	*	TYPE=SUBSTITUTE
CM032587.1	1741245	.	T	C	100	*	TYPE=SUBSTITUTE
CM032587.1	1741272	.	A	G	100	*	TYPE=SUBSTITUTE
CM032587.1	1741290	.	G	A	100	*	TYPE=SUBSTITUTE
CM032587.1	1741299	.	G	A	100	*	TYPE=SUBSTITUTE
CM032587.1	1741306	.	C	T	100	*	TYPE=SUBSTITUTE
CM032587.1	1741307	.	C	T	100	*	TYPE=SUBSTITUTE
CM032587.1	1741308	.	A	G	100	*	TYPE=SUBSTITUTE
CM032587.1	1741309	.	T	G	100	*	TYPE=SUBSTITUTE
CM032587.1	1741313	.	C	T	100	*	TYPE=SUBSTITUTE
CM032587.1	1741314	.	A	T	100	*	TYPE=SUBSTITUTE
CM032587.1	1741326	.	T	C	100	*	TYPE=SUBSTITUTE
CM032587.1	1741344	.	C	T	100	*	TYPE=SUBSTITUTE
CM032587.1	1741372	.	C	T	100	*	TYPE=SUBSTITUTE
CM032587.1	1741422	.	G	A	100	*	TYPE=SUBSTITUTE
CM032587.1	1741542	.	C	T	100	*	TYPE=SUBSTITUTE
CM032587.1	1741557	.	A	G	100	*	TYPE=SUBSTITUTE
CM032587.1	1741728	.	A	G	100	*	TYPE=SUBSTITUTE
CM032587.1	1741743	.	G	A	100	*	TYPE=SUBSTITUTE
CM032587.1	1741851	.	G	A	100	*	TYPE=SUBSTITUTE
CM032587.1	1741917	.	C	T	100	*	TYPE=SUBSTITUTE
CM032587.1	1742067	.	G	C	100	*	TYPE=SUBSTITUTE
CM032587.1	1742142	.	T	C	100	*	TYPE=SUBSTITUTE
CM032587.1	1742148	.	G	C	100	*	TYPE=SUBSTITUTE
CM032587.1	1742158	.	A	G	100	*	TYPE=SUBSTITUTE
CM032587.1	1742160	.	T	G	100	*	TYPE=SUBSTITUTE
CM032587.1	1742175	.	C	T	100	*	TYPE=SUBSTITUTE
CM032587.1	1742212	.	C	T	100	*	TYPE=SUBSTITUTE
CM032587.1	1742220	.	A	C	100	*	TYPE=SUBSTITUTE
CM032587.1	1742223	.	C	A	100	*	TYPE=SUBSTITUTE
CM032587.1	1742251	.	T	A	100	*	TYPE=SUBSTITUTE
CM032587.1	1742262	.	A	G	100	*	TYPE=SUBSTITUTE
CM032587.1	1742295	.	G	T	100	*	TYPE=SUBSTITUTE

CM032587.1	1742361	.	C	T	100	*	TYPE=SUBSTITUTE
CM032587.1	1742385	.	A	G	100	*	TYPE=SUBSTITUTE
CM032587.1	1742424	.	G	A	100	*	TYPE=SUBSTITUTE
CM032587.1	1742469	.	G	T	100	*	TYPE=SUBSTITUTE
CM032587.1	1742478	.	A	G	100	*	TYPE=SUBSTITUTE
CM032587.1	1742484	.	G	A	100	*	TYPE=SUBSTITUTE
CM032587.1	1742574	.	G	A	100	*	TYPE=SUBSTITUTE
CM032587.1	1742625	.	C	T	100	*	TYPE=SUBSTITUTE
CM032587.1	1742652	.	T	C	100	*	TYPE=SUBSTITUTE
CM032587.1	1742697	.	A	G	100	*	TYPE=SUBSTITUTE
CM032587.1	1742823	.	A	G	100	*	TYPE=SUBSTITUTE
CM032587.1	1742847	.	C	T	100	*	TYPE=SUBSTITUTE
CM032587.1	1742853	.	C	T	100	*	TYPE=SUBSTITUTE
CM032587.1	1742892	.	C	G	100	*	TYPE=SUBSTITUTE
CM032587.1	1742961	.	T	C	100	*	TYPE=SUBSTITUTE
CM032587.1	1742989	.	A	G	100	*	TYPE=SUBSTITUTE
CM032587.1	1743048	.	G	a	100	*	TYPE=SUBSTITUTE
CM032587.1	1743107	.	T	C	100	*	TYPE=SUBSTITUTE
CM032587.1	1743110	.	C	T	100	*	TYPE=SUBSTITUTE
CM032587.1	1743149	.	G	a	100	*	TYPE=SUBSTITUTE
CM032587.1	2019311	.	A	G	100	*	TYPE=SUBSTITUTE
CM032587.1	2019316	.	A	T	100	*	TYPE=SUBSTITUTE
CM032587.1	2019318	.	A	G	100	*	TYPE=SUBSTITUTE
CM032587.1	2019320	.	T	A	100	*	TYPE=SUBSTITUTE
CM032587.1	2019321	.	A	T	100	*	TYPE=SUBSTITUTE
CM032587.1	2019329	.	G	A	100	*	TYPE=SUBSTITUTE
CM032587.1	2019333	.	C	T	100	*	TYPE=SUBSTITUTE
CM032587.1	2019334	.	A	G	100	*	TYPE=SUBSTITUTE
CM032587.1	2019335	.	A	G	100	*	TYPE=SUBSTITUTE
CM032587.1	2019344	.	G	T	100	*	TYPE=SUBSTITUTE
CM032587.1	2019352	.	A	T	100	*	TYPE=SUBSTITUTE
CM032587.1	2019359	.	C	T	100	*	TYPE=SUBSTITUTE
CM032587.1	2019361	.	A	C	100	*	TYPE=SUBSTITUTE
CM032587.1	2019364	.	AC	A	100	*	TYPE=DELETE
CM032587.1	2019374	.	TCA	T	100	*	TYPE=DELETE
CM032587.1	2019377	.	G	A	100	*	TYPE=SUBSTITUTE
CM032587.1	2019379	.	T	C	100	*	TYPE=SUBSTITUTE
CM032587.1	2019382	.	TACAC	T	100	*	TYPE=DELETE
CM032587.1	2019389	.	G	T	100	*	TYPE=SUBSTITUTE
CM032587.1	2019390	.	C	T	100	*	TYPE=SUBSTITUTE
CM032587.1	2019403	.	T	C	100	*	TYPE=SUBSTITUTE
CM032587.1	2019404	.	A	G	100	*	TYPE=SUBSTITUTE
CM032587.1	2019408	.	G	GA	100	*	TYPE=INSERT
CM032587.1	2019416	.	T	C	100	*	TYPE=SUBSTITUTE
CM032587.1	2019436	.	T	C	100	*	TYPE=SUBSTITUTE
CM032587.1	2019450	.	G	A	100	*	TYPE=SUBSTITUTE
CM032587.1	2019469	.	G	GA	100	*	TYPE=INSERT
CM032587.1	2019470	.	C	G	100	*	TYPE=SUBSTITUTE
CM032587.1	2019471	.	C	T	100	*	TYPE=SUBSTITUTE
CM032587.1	2019477	.	A	G	100	*	TYPE=SUBSTITUTE
CM032587.1	2019484	.	g	ggga	100	*	TYPE=INSERT
CM032587.1	2019489	.	A	g	100	*	TYPE=SUBSTITUTE
CM032587.1	2019494	.	G	a	100	*	TYPE=SUBSTITUTE
CM032587.1	2019497	.	AACGTC	A	100	*	TYPE=DELETE
CM032587.1	2019517	.	AAT	A	100	*	TYPE=DELETE
CM032587.1	2019520	.	A	g	100	*	TYPE=SUBSTITUTE
CM032587.1	2019579	.	ACA	A	100	*	TYPE=DELETE
CM032587.1	2019591	.	A	c	100	*	TYPE=SUBSTITUTE
CM032587.1	2019592	.	A	t	100	*	TYPE=SUBSTITUTE
CM032587.1	2019593	.	A	c	100	*	TYPE=SUBSTITUTE
CM032587.1	2019594	.	AAAAAAAT	A	100	*	TYPE=DELETE
CM032587.1	2019626	.	AA	A	100	*	TYPE=DELETE
CM032587.1	2019650	.	T	G	100	*	TYPE=SUBSTITUTE
CM032587.1	2019651	.	G	A	100	*	TYPE=SUBSTITUTE

CM032587.1	2019654	.	G	A	100	*	TYPE=SUBSTITUTE
CM032587.1	2019659	.	CA	C	100	*	TYPE=DELETE
CM032587.1	2019721	.	AAA	A	100	*	TYPE=DELETE
CM032587.1	2019729	.	A	g	100	*	TYPE=SUBSTITUTE
CM032587.1	2019731	.	AG	A	100	*	TYPE=DELETE
CM032587.1	2019765	.	A	g	100	*	TYPE=SUBSTITUTE
CM032587.1	2019772	.	A	g	100	*	TYPE=SUBSTITUTE
CM032587.1	2019786	.	A	T	100	*	TYPE=SUBSTITUTE
CM032587.1	2019788	.	G	GA	100	*	TYPE=INSERT
CM032587.1	2019827	.	G	T	100	*	TYPE=SUBSTITUTE
CM032587.1	2019849	.	A	G	100	*	TYPE=SUBSTITUTE
CM032587.1	2019871	.	G	A	100	*	TYPE=SUBSTITUTE
CM032587.1	2019891	.	G	A	100	*	TYPE=SUBSTITUTE
CM032587.1	2019892	.	A	G	100	*	TYPE=SUBSTITUTE
CM032587.1	2019904	.	A	T	100	*	TYPE=SUBSTITUTE
CM032587.1	2019908	.	T	C	100	*	TYPE=SUBSTITUTE
CM032587.1	2019916	.	G	A	100	*	TYPE=SUBSTITUTE
CM032587.1	2019940	.	A	G	100	*	TYPE=SUBSTITUTE
CM032587.1	2019950	.	TA	T	100	*	TYPE=DELETE
CM032587.1	2019962	.	A	G	100	*	TYPE=SUBSTITUTE
CM032587.1	2019963	.	G	A	100	*	TYPE=SUBSTITUTE
CM032587.1	2019981	.	G	A	100	*	TYPE=SUBSTITUTE
CM032587.1	2019990	.	T	G	100	*	TYPE=SUBSTITUTE
CM032587.1	2020000	.	CA	C	100	*	TYPE=DELETE
CM032587.1	2020007	.	G	a	100	*	TYPE=SUBSTITUTE
CM032587.1	2020008	.	C	g	100	*	TYPE=SUBSTITUTE
CM032587.1	2020012	.	G	a	100	*	TYPE=SUBSTITUTE
CM032587.1	2020015	.	A	g	100	*	TYPE=SUBSTITUTE
CM032587.1	2020018	.	C	a	100	*	TYPE=SUBSTITUTE
CM032587.1	2020022	.	G	a	100	*	TYPE=SUBSTITUTE
CM032587.1	2020030	.	A	G	100	*	TYPE=SUBSTITUTE
CM032587.1	2020095	.	G	A	100	*	TYPE=SUBSTITUTE
CM032587.1	2020125	.	C	T	100	*	TYPE=SUBSTITUTE
CM032587.1	2020131	.	T	C	100	*	TYPE=SUBSTITUTE
CM032587.1	2020143	.	G	a	100	*	TYPE=SUBSTITUTE
CM032587.1	2020146	.	C	t	100	*	TYPE=SUBSTITUTE
CM032587.1	2020155	.	C	t	100	*	TYPE=SUBSTITUTE
CM032587.1	2020188	.	T	C	100	*	TYPE=SUBSTITUTE
CM032587.1	2020209	.	A	G	100	*	TYPE=SUBSTITUTE
CM032587.1	2020215	.	A	G	100	*	TYPE=SUBSTITUTE
CM032587.1	2020218	.	G	A	100	*	TYPE=SUBSTITUTE
CM032587.1	2020221	.	T	G	100	*	TYPE=SUBSTITUTE
CM032587.1	2020236	.	A	G	100	*	TYPE=SUBSTITUTE
CM032587.1	2020269	.	A	g	100	*	TYPE=SUBSTITUTE
CM032587.1	2020278	.	T	c	100	*	TYPE=SUBSTITUTE
CM032587.1	2020317	.	C	G	100	*	TYPE=SUBSTITUTE
CM032587.1	2020319	.	G	C	100	*	TYPE=SUBSTITUTE
CM032587.1	2020335	.	C	T	100	*	TYPE=SUBSTITUTE
CM032587.1	2020350	.	A	G	100	*	TYPE=SUBSTITUTE
CM032587.1	2020359	.	A	G	100	*	TYPE=SUBSTITUTE
CM032587.1	2020416	.	A	G	100	*	TYPE=SUBSTITUTE
CM032587.1	2020509	.	T	C	100	*	TYPE=SUBSTITUTE
CM032587.1	2020515	.	G	A	100	*	TYPE=SUBSTITUTE
CM032587.1	2020518	.	G	A	100	*	TYPE=SUBSTITUTE
CM032587.1	2020519	.	C	G	100	*	TYPE=SUBSTITUTE
CM032587.1	2020551	.	C	t	100	*	TYPE=SUBSTITUTE
CM032587.1	2020576	.	G	C	100	*	TYPE=SUBSTITUTE
CM032587.1	2020611	.	A	T	100	*	TYPE=SUBSTITUTE
CM032587.1	2020614	.	C	T	100	*	TYPE=SUBSTITUTE
CM032587.1	2020617	.	C	G	100	*	TYPE=SUBSTITUTE
CM032587.1	2020626	.	T	A	100	*	TYPE=SUBSTITUTE
CM032587.1	2020635	.	A	G	100	*	TYPE=SUBSTITUTE
CM032587.1	2020647	.	C	G	100	*	TYPE=SUBSTITUTE
CM032587.1	2020648	.	G	T	100	*	TYPE=SUBSTITUTE

CM032587.1	2020649	.	T	A	100	*	TYPE=SUBSTITUTE
CM032587.1	2020674	.	T	C	100	*	TYPE=SUBSTITUTE
CM032587.1	2020680	.	T	C	100	*	TYPE=SUBSTITUTE
CM032587.1	2020686	.	A	G	100	*	TYPE=SUBSTITUTE
CM032587.1	2020725	.	A	g	100	*	TYPE=SUBSTITUTE
CM032587.1	2020728	.	A	g	100	*	TYPE=SUBSTITUTE
CM032587.1	2020758	.	C	T	100	*	TYPE=SUBSTITUTE
CM032587.1	2020770	.	A	G	100	*	TYPE=SUBSTITUTE
CM032587.1	2020771	.	T	C	100	*	TYPE=SUBSTITUTE
CM032587.1	2020782	.	C	T	100	*	TYPE=SUBSTITUTE
CM032587.1	2020788	.	C	T	100	*	TYPE=SUBSTITUTE
CM032587.1	2020797	.	G	C	100	*	TYPE=SUBSTITUTE
CM032587.1	2020812	.	T	c	100	*	TYPE=SUBSTITUTE
CM032587.1	2020845	.	G	A	100	*	TYPE=SUBSTITUTE
CM032587.1	2020917	.	A	G	100	*	TYPE=SUBSTITUTE
CM032587.1	2020992	.	T	C	100	*	TYPE=SUBSTITUTE
CM032587.1	2020995	.	T	C	100	*	TYPE=SUBSTITUTE
CM032587.1	2021007	.	A	G	100	*	TYPE=SUBSTITUTE
CM032587.1	2021067	.	G	T	100	*	TYPE=SUBSTITUTE
CM032587.1	2021130	.	A	C	100	*	TYPE=SUBSTITUTE
CM032587.1	2021154	.	T	G	100	*	TYPE=SUBSTITUTE
CM032587.1	2021159	.	G	A	100	*	TYPE=SUBSTITUTE
CM032587.1	2021175	.	C	T	100	*	TYPE=SUBSTITUTE
CM032587.1	2021198	.	GTAGTTGTTGTTG	G	100	*	TYPE=DELETE
CM032587.1	2021211	.	T	c	100	*	TYPE=SUBSTITUTE
CM032587.1	2021214	.	T	c	100	*	TYPE=SUBSTITUTE
CM032587.1	2021226	.	C	t	100	*	TYPE=SUBSTITUTE
CM032587.1	2021232	.	C	t	100	*	TYPE=SUBSTITUTE
CM032587.1	2021251	.	C	T	100	*	TYPE=SUBSTITUTE
CM032587.1	2021355	.	G	A	100	*	TYPE=SUBSTITUTE
CM032587.1	2021427	.	T	C	100	*	TYPE=SUBSTITUTE
CM032587.1	2021851	.	C	G	100	*	TYPE=SUBSTITUTE
CM032587.1	2021852	.	T	A	100	*	TYPE=SUBSTITUTE
CM032587.1	2021908	.	T	A	100	*	TYPE=SUBSTITUTE
CM032587.1	2021995	.	TT	T	100	*	TYPE=DELETE
CM032587.1	2022032	.	C	T	100	*	TYPE=SUBSTITUTE
CM032587.1	2022039	.	TG	T	100	*	TYPE=DELETE
CM032587.1	2022060	.	A	G	100	*	TYPE=SUBSTITUTE
CM032587.1	2022090	.	T	C	100	*	TYPE=SUBSTITUTE
CM032587.1	2022145	.	A	t	100	*	TYPE=SUBSTITUTE
CM032587.1	2022225	.	A	G	100	*	TYPE=SUBSTITUTE
CM032587.1	2022286	.	a	aaacca	100	*	TYPE=INSERT
CM032587.1	2022287	.	C	a	100	*	TYPE=SUBSTITUTE
CM032587.1	2022468	.	A	T	100	*	TYPE=SUBSTITUTE
CM032587.1	2022529	.	C	T	100	*	TYPE=SUBSTITUTE
CM032587.1	2022624	.	AT	A	100	*	TYPE=DELETE
CM032587.1	2022950	.	C	A	100	*	TYPE=SUBSTITUTE
CM032587.1	2022951	.	G	C	100	*	TYPE=SUBSTITUTE
CM032587.1	2022971	.	C	T	100	*	TYPE=SUBSTITUTE
CM032587.1	2023060	.	G	A	100	*	TYPE=SUBSTITUTE
CM032587.1	2023302	.	G	C	100	*	TYPE=SUBSTITUTE
CM032587.1	2023357	.	C	T	100	*	TYPE=SUBSTITUTE
CM032587.1	2023506	.	C	G	100	*	TYPE=SUBSTITUTE
CM032587.1	2023557	.	G	A	100	*	TYPE=SUBSTITUTE
CM032587.1	2023569	.	T	G	100	*	TYPE=SUBSTITUTE
CM032587.1	2023573	.	C	G	100	*	TYPE=SUBSTITUTE
CM032587.1	2023575	.	A	C	100	*	TYPE=SUBSTITUTE
CM032587.1	2023657	.	GT	G	100	*	TYPE=DELETE
CM032587.1	2023665	.	A	T	100	*	TYPE=SUBSTITUTE
CM032587.1	2023739	.	A	G	100	*	TYPE=SUBSTITUTE
CM032587.1	2023975	.	G	A	100	*	TYPE=SUBSTITUTE
CM032587.1	2024258	.	C	G	100	*	TYPE=SUBSTITUTE
CM032587.1	2024274	.	GA	G	100	*	TYPE=DELETE
CM032587.1	2024314	.	A	g	100	*	TYPE=SUBSTITUTE

CM032587.1	2024315	.	G	a	100	*	TYPE=SUBSTITUTE
CM032587.1	2024327	.	T	a	100	*	TYPE=SUBSTITUTE
CM032587.1	2024329	.	T	a	100	*	TYPE=SUBSTITUTE
CM032587.1	2024337	.	C	A	100	*	TYPE=SUBSTITUTE
CM032587.1	2024338	.	A	G	100	*	TYPE=SUBSTITUTE
CM032587.1	2024403	.	T	C	100	*	TYPE=SUBSTITUTE
CM032587.1	2024624	.	C	T	100	*	TYPE=SUBSTITUTE
CM032587.1	2024780	.	CT	C	100	*	TYPE=DELETE
CM032587.1	2024806	.	C	A	100	*	TYPE=SUBSTITUTE
CM032587.1	2024840	.	C	t	100	*	TYPE=SUBSTITUTE
CM032587.1	2024841	.	G	c	100	*	TYPE=SUBSTITUTE
CM032587.1	2024845	.	A	c	100	*	TYPE=SUBSTITUTE
CM032587.1	2024850	.	G	a	100	*	TYPE=SUBSTITUTE
CM032587.1	2024863	.	ATG	A	100	*	TYPE=DELETE
CM032587.1	2024868	.	G	c	100	*	TYPE=SUBSTITUTE
CM032587.1	2024869	.	A	C	100	*	TYPE=SUBSTITUTE
CM032587.1	2024872	.	ATGAACCAG	A	100	*	TYPE=DELETE
CM032587.1	2024884	.	A	G	100	*	TYPE=SUBSTITUTE
CM032587.1	2024933	.	TT	T	100	*	TYPE=DELETE
CM032587.1	2025030	.	A	G	100	*	TYPE=SUBSTITUTE
CM032587.1	2025033	.	G	C	100	*	TYPE=SUBSTITUTE
CM032587.1	2025034	.	T	C	100	*	TYPE=SUBSTITUTE
CM032587.1	2025035	.	C	T	100	*	TYPE=SUBSTITUTE
CM032587.1	2025116	.	T	C	100	*	TYPE=SUBSTITUTE
CM032587.1	2025127	.	G	A	100	*	TYPE=SUBSTITUTE
CM032587.1	2025128	.	T	A	100	*	TYPE=SUBSTITUTE
CM032587.1	2025144	.	C	A	100	*	TYPE=SUBSTITUTE
CM032587.1	2025158	.	G	T	100	*	TYPE=SUBSTITUTE
CM032587.1	2025162	.	T	G	100	*	TYPE=SUBSTITUTE
CM032587.1	2025225	.	C	T	100	*	TYPE=SUBSTITUTE
CM032587.1	2025231	.	C	G	100	*	TYPE=SUBSTITUTE
CM032587.1	2025315	.	G	A	100	*	TYPE=SUBSTITUTE
CM032587.1	2025505	.	G	A	100	*	TYPE=SUBSTITUTE
CM032587.1	2025651	.	A	G	100	*	TYPE=SUBSTITUTE
CM032587.1	2025684	.	A	C	100	*	TYPE=SUBSTITUTE
CM032587.1	2025686	.	A	G	100	*	TYPE=SUBSTITUTE
CM032587.1	2025720	.	G	A	100	*	TYPE=SUBSTITUTE
CM032587.1	2025721	.	A	G	100	*	TYPE=SUBSTITUTE
CM032587.1	2025738	.	C	T	100	*	TYPE=SUBSTITUTE
CM032587.1	2025780	.	T	C	100	*	TYPE=SUBSTITUTE
CM032587.1	2025846	.	T	C	100	*	TYPE=SUBSTITUTE
CM032587.1	2026072	.	G	A	100	*	TYPE=SUBSTITUTE
CM032587.1	2026074	.	C	T	100	*	TYPE=SUBSTITUTE
CM032587.1	2026099	.	T	C	100	*	TYPE=SUBSTITUTE
CM032587.1	2026101	.	A	G	100	*	TYPE=SUBSTITUTE
CM032587.1	2026115	.	T	C	100	*	TYPE=SUBSTITUTE
CM032587.1	2026150	.	A	G	100	*	TYPE=SUBSTITUTE
CM032587.1	2026153	.	C	A	100	*	TYPE=SUBSTITUTE
CM032587.1	2026163	.	G	A	100	*	TYPE=SUBSTITUTE
CM032587.1	2026165	.	T	C	100	*	TYPE=SUBSTITUTE
CM032587.1	2026180	.	G	a	100	*	TYPE=SUBSTITUTE
CM032587.1	2026185	.	C	t	100	*	TYPE=SUBSTITUTE
CM032587.1	2026190	.	G	a	100	*	TYPE=SUBSTITUTE
CM032587.1	2026223	.	T	C	100	*	TYPE=SUBSTITUTE
CM032587.1	2026237	.	A	G	100	*	TYPE=SUBSTITUTE
CM032587.1	2026319	.	G	A	100	*	TYPE=SUBSTITUTE
CM032587.1	2026346	.	T	C	100	*	TYPE=SUBSTITUTE
CM032587.1	2026348	.	G	A	100	*	TYPE=SUBSTITUTE
CM032587.1	2026353	.	T	C	100	*	TYPE=SUBSTITUTE
CM032587.1	2026511	.	G	GCTTGTCAGG	100	*	TYPE=INSERT
CM032587.1	2026545	.	G	GCTGTA	100	*	TYPE=INSERT
CM032587.1	2026555	.	G	A	100	*	TYPE=SUBSTITUTE
CM032587.1	2026558	.	G	GCGACA	100	*	TYPE=INSERT
CM032587.1	2026560	.	T	G	100	*	TYPE=SUBSTITUTE

CM032587.1	2026562	.	G	C	100	*	TYPE=SUBSTITUTE
CM032587.1	2026564	.	G	GCTGC	100	*	TYPE=INSERT
CM032587.1	2026565	.	G	C	100	*	TYPE=SUBSTITUTE
CM032587.1	2026569	.	G	T	100	*	TYPE=SUBSTITUTE
CM032587.1	2026571	.	C	CATCA	100	*	TYPE=INSERT
CM032587.1	2026679	.	C	CTGGCT	100	*	TYPE=INSERT
CM032587.1	2026721	.	C	T	100	*	TYPE=SUBSTITUTE
CM032587.1	2026727	.	GGC	G	100	*	TYPE=DELETE
CM032587.1	2026739	.	AG	A	100	*	TYPE=DELETE
CM032587.1	2026920	.	T	C	100	*	TYPE=SUBSTITUTE
CM032587.1	2027112	.	G	C	100	*	TYPE=SUBSTITUTE
CM032587.1	2027203	.	G	C	100	*	TYPE=SUBSTITUTE
CM032587.1	2027222	.	T	C	100	*	TYPE=SUBSTITUTE
CM032587.1	2027227	.	GA	G	100	*	TYPE=DELETE
CM032587.1	2027229	.	A	G	100	*	TYPE=SUBSTITUTE
CM032587.1	2027246	.	C	CA	100	*	TYPE=INSERT
CM032587.1	2027295	.	CCCGTACAGTA	C	100	*	TYPE=DELETE
CM032587.1	2027307	.	A	C	100	*	TYPE=SUBSTITUTE
CM032587.1	2027325	.	AG	A	100	*	TYPE=DELETE
CM032587.1	2027327	.	G	T	100	*	TYPE=SUBSTITUTE
CM032587.1	2027328	.	T	A	100	*	TYPE=SUBSTITUTE
CM032587.1	2027344	.	A	ACGTAA	100	*	TYPE=INSERT
CM032587.1	2027352	.	AG	A	100	*	TYPE=DELETE
CM032587.1	2027360	.	A	C	100	*	TYPE=SUBSTITUTE
CM032587.1	2027364	.	A	G	100	*	TYPE=SUBSTITUTE
CM032587.1	2027376	.	ATACTG	A	100	*	TYPE=DELETE
CM032587.1	2027385	.	C	T	100	*	TYPE=SUBSTITUTE
CM032587.1	2027439	.	A	C	100	*	TYPE=SUBSTITUTE
CM032587.1	2027449	.	G	GA	100	*	TYPE=INSERT
CM032587.1	2027502	.	C	A	100	*	TYPE=SUBSTITUTE
CM032587.1	2027574	.	A	g	100	*	TYPE=SUBSTITUTE
CM032587.1	2027592	.	C	T	100	*	TYPE=SUBSTITUTE
CM032587.1	2027626	.	G	A	100	*	TYPE=SUBSTITUTE
CM032587.1	2027657	.	A	g	100	*	TYPE=SUBSTITUTE
CM032587.1	2027695	.	A	ATCA	100	*	TYPE=INSERT
CM032587.1	2027700	.	A	G	100	*	TYPE=SUBSTITUTE
CM032587.1	2027740	.	AGCCTG	A	100	*	TYPE=DELETE
CM032587.1	2027748	.	T	a	100	*	TYPE=SUBSTITUTE
CM032587.1	2027750	.	G	t	100	*	TYPE=SUBSTITUTE
CM032587.1	2027751	.	G	a	100	*	TYPE=SUBSTITUTE
CM032587.1	2027752	.	A	t	100	*	TYPE=SUBSTITUTE
CM032587.1	2027753	.	G	a	100	*	TYPE=SUBSTITUTE
CM032587.1	2027755	.	TAG	T	100	*	TYPE=DELETE
CM032587.1	2027759	.	A	T	100	*	TYPE=SUBSTITUTE
CM032587.1	2027760	.	AT	A	100	*	TYPE=DELETE
CM032587.1	2027762	.	A	G	100	*	TYPE=SUBSTITUTE
CM032587.1	2027766	.	C	G	100	*	TYPE=SUBSTITUTE
CM032587.1	2027768	.	A	G	100	*	TYPE=SUBSTITUTE
CM032587.1	2027769	.	T	TGG	100	*	TYPE=INSERT
CM032587.1	2027771	.	T	A	100	*	TYPE=SUBSTITUTE
CM032587.1	2027775	.	G	A	100	*	TYPE=SUBSTITUTE
CM032587.1	2027778	.	C	A	100	*	TYPE=SUBSTITUTE
CM032587.1	2027779	.	T	a	100	*	TYPE=SUBSTITUTE
CM032587.1	2027780	.	G	t	100	*	TYPE=SUBSTITUTE
CM032587.1	2027781	.	T	g	100	*	TYPE=SUBSTITUTE
CM032587.1	2027782	.	G	c	100	*	TYPE=SUBSTITUTE
CM032587.1	2027801	.	C	T	100	*	TYPE=SUBSTITUTE
CM032587.1	2027994	.	T	C	100	*	TYPE=SUBSTITUTE
CM032587.1	2028013	.	A	G	100	*	TYPE=SUBSTITUTE
CM032587.1	2028021	.	ACTACTACCGTCGTG	A	100	*	TYPE=DELETE
CM032587.1	2028040	.	T	g	100	*	TYPE=SUBSTITUTE
CM032587.1	2028067	.	T	C	100	*	TYPE=SUBSTITUTE
CM032587.1	2028077	.	A	ATTGCTTTACTTAGCGCATCCAGGTTGG	100	*	TYPE=INSERT

CM032587.1	2028093	.	G	A	100	*	TYPE=SUBSTITUTE
CM032587.1	2028117	.	G	C	100	*	TYPE=SUBSTITUTE
CM032587.1	2028160	.	TTTCCT	T	100	*	TYPE=DELETE
CM032587.1	2028215	.	G	A	100	*	TYPE=SUBSTITUTE
CM032587.1	2028242	.	T	G	100	*	TYPE=SUBSTITUTE
CM032587.1	2028261	.	T	C	100	*	TYPE=SUBSTITUTE
CM032587.1	2028264	.	A	AT	100	*	TYPE=INSERT
CM032587.1	2028265	.	C	T	100	*	TYPE=SUBSTITUTE
CM032587.1	2028266	.	C	T	100	*	TYPE=SUBSTITUTE
CM032587.1	2028268	.	T	C	100	*	TYPE=SUBSTITUTE
CM032587.1	2028269	.	G	A	100	*	TYPE=SUBSTITUTE
CM032587.1	2028276	.	TGTACAAGTGCTC	T	100	*	TYPE=DELETE
CM032587.1	2028290	.	T	C	100	*	TYPE=SUBSTITUTE
CM032587.1	2028307	.	TCTCTAGTG	T	100	*	TYPE=DELETE
CM032587.1	2028461	.	A	t	100	*	TYPE=SUBSTITUTE
CM032587.1	2028677	.	A	G	100	*	TYPE=SUBSTITUTE
CM032587.1	2028863	.	C	T	100	*	TYPE=SUBSTITUTE
CM032587.1	2028870	.	CG	C	100	*	TYPE=DELETE
CM032587.1	2028893	.	TT	T	100	*	TYPE=DELETE
CM032587.1	2029029	.	C	A	100	*	TYPE=SUBSTITUTE
CM032587.1	2029065	.	C	T	100	*	TYPE=SUBSTITUTE
CM032587.1	2029083	.	G	T	100	*	TYPE=SUBSTITUTE
CM032587.1	2029084	.	A	G	100	*	TYPE=SUBSTITUTE
CM032587.1	2029106	.	T	C	100	*	TYPE=SUBSTITUTE
CM032587.1	2029111	.	C	T	100	*	TYPE=SUBSTITUTE
CM032587.1	2029121	.	G	A	100	*	TYPE=SUBSTITUTE
CM032587.1	2029123	.	G	T	100	*	TYPE=SUBSTITUTE
CM032587.1	2029160	.	G	C	100	*	TYPE=SUBSTITUTE
CM032587.1	2029161	.	G	A	100	*	TYPE=SUBSTITUTE
CM032587.1	2029166	.	C	T	100	*	TYPE=SUBSTITUTE
CM032587.1	2029173	.	A	G	100	*	TYPE=SUBSTITUTE
CM032587.1	2029180	.	CTAT	C	100	*	TYPE=DELETE
CM032587.1	2029184	.	G	A	100	*	TYPE=SUBSTITUTE
CM032587.1	2029243	.	G	A	100	*	TYPE=SUBSTITUTE
CM032587.1	2029252	.	T	C	100	*	TYPE=SUBSTITUTE
CM032587.1	2029273	.	G	C	100	*	TYPE=SUBSTITUTE
CM032587.1	2029280	.	G	A	100	*	TYPE=SUBSTITUTE
CM032587.1	2029284	.	C	CTACAG	100	*	TYPE=INSERT
CM032587.1	2029285	.	C	T	100	*	TYPE=SUBSTITUTE
CM032587.1	2029351	.	C	A	100	*	TYPE=SUBSTITUTE
CM032587.1	2029366	.	G	A	100	*	TYPE=SUBSTITUTE
CM032587.1	2029385	.	G	A	100	*	TYPE=SUBSTITUTE
CM032587.1	2029391	.	A	C	100	*	TYPE=SUBSTITUTE
CM032587.1	2029406	.	C	T	100	*	TYPE=SUBSTITUTE
CM032587.1	2029473	.	G	A	100	*	TYPE=SUBSTITUTE
CM032587.1	2029497	.	C	T	100	*	TYPE=SUBSTITUTE
CM032587.1	2029522	.	A	T	100	*	TYPE=SUBSTITUTE
CM032587.1	2029525	.	G	A	100	*	TYPE=SUBSTITUTE
CM032587.1	2029544	.	C	T	100	*	TYPE=SUBSTITUTE
CM032587.1	2029617	.	GG	G	100	*	TYPE=DELETE
CM032587.1	2029619	.	T	C	100	*	TYPE=SUBSTITUTE
CM032587.1	2029649	.	T	C	100	*	TYPE=SUBSTITUTE
CM032587.1	2029666	.	C	t	100	*	TYPE=SUBSTITUTE
CM032587.1	2029697	.	C	T	100	*	TYPE=SUBSTITUTE
CM032587.1	2029698	.	C	G	100	*	TYPE=SUBSTITUTE
CM032587.1	2029706	.	C	T	100	*	TYPE=SUBSTITUTE
CM032587.1	2029707	.	C	T	100	*	TYPE=SUBSTITUTE
CM032587.1	2029718	.	C	T	100	*	TYPE=SUBSTITUTE
CM032587.1	2029723	.	A	T	100	*	TYPE=SUBSTITUTE
CM032587.1	2029816	.	GA	G	100	*	TYPE=DELETE
CM032587.1	2029861	.	A	g	100	*	TYPE=SUBSTITUTE
CM032587.1	2029877	.	C	T	100	*	TYPE=SUBSTITUTE
CM032587.1	2029899	.	T	TT	100	*	TYPE=INSERT
CM032587.1	2029906	.	C	G	100	*	TYPE=SUBSTITUTE

CM032587.1	2029907	.	A	G	100	*	TYPE=SUBSTITUTE
CM032587.1	2029933	.	TT	T	100	*	TYPE=DELETE
CM032587.1	2029960	.	C	T	100	*	TYPE=SUBSTITUTE
CM032587.1	2030061	.	C	A	100	*	TYPE=SUBSTITUTE
CM032587.1	2030098	.	C	T	100	*	TYPE=SUBSTITUTE
CM032587.1	2030110	.	C	T	100	*	TYPE=SUBSTITUTE
CM032587.1	2030142	.	G	T	100	*	TYPE=SUBSTITUTE
CM032587.1	2030170	.	A	G	100	*	TYPE=SUBSTITUTE
CM032587.1	2030172	.	A	T	100	*	TYPE=SUBSTITUTE
CM032587.1	2030173	.	C	A	100	*	TYPE=SUBSTITUTE
CM032587.1	2030177	.	G	A	100	*	TYPE=SUBSTITUTE
CM032587.1	2030232	.	C	CT	100	*	TYPE=INSERT
CM032587.1	2030235	.	T	A	100	*	TYPE=SUBSTITUTE
CM032587.1	2030384	.	T	C	100	*	TYPE=SUBSTITUTE
CM032587.1	2030417	.	G	T	100	*	TYPE=SUBSTITUTE
CM032587.1	2030418	.	T	C	100	*	TYPE=SUBSTITUTE
CM032587.1	2030437	.	G	A	100	*	TYPE=SUBSTITUTE
CM032587.1	2030529	.	T	C	100	*	TYPE=SUBSTITUTE
CM032587.1	2030598	.	T	C	100	*	TYPE=SUBSTITUTE
CM032587.1	2030627	.	C	G	100	*	TYPE=SUBSTITUTE
CM032587.1	2030635	.	G	A	100	*	TYPE=SUBSTITUTE
CM032587.1	2030667	.	A	G	100	*	TYPE=SUBSTITUTE
CM032587.1	2030676	.	T	G	100	*	TYPE=SUBSTITUTE
CM032587.1	2030694	.	T	C	100	*	TYPE=SUBSTITUTE
CM032587.1	2030697	.	G	A	100	*	TYPE=SUBSTITUTE
CM032587.1	2030708	.	C	A	100	*	TYPE=SUBSTITUTE
CM032587.1	2030743	.	C	T	100	*	TYPE=SUBSTITUTE
CM032587.1	2030819	.	A	G	100	*	TYPE=SUBSTITUTE
CM032587.1	2030957	.	G	C	100	*	TYPE=SUBSTITUTE
CM032587.1	2031132	.	T	C	100	*	TYPE=SUBSTITUTE
CM032587.1	2031162	.	T	G	100	*	TYPE=SUBSTITUTE
CM032587.1	2031167	.	A	G	100	*	TYPE=SUBSTITUTE
CM032587.1	2031205	.	T	C	100	*	TYPE=SUBSTITUTE
CM032587.1	2031221	.	C	G	100	*	TYPE=SUBSTITUTE
CM032587.1	2031318	.	G	C	100	*	TYPE=SUBSTITUTE
CM032587.1	2031322	.	A	G	100	*	TYPE=SUBSTITUTE
CM032587.1	2031360	.	C	T	100	*	TYPE=SUBSTITUTE
CM032587.1	2031385	.	C	T	100	*	TYPE=SUBSTITUTE
CM032587.1	2031677	.	G	C	100	*	TYPE=SUBSTITUTE
CM032587.1	2031786	.	A	G	100	*	TYPE=SUBSTITUTE
CM032587.1	2031861	.	G	T	100	*	TYPE=SUBSTITUTE
CM032587.1	2031874	.	A	G	100	*	TYPE=SUBSTITUTE
CM032587.1	2031882	.	G	A	100	*	TYPE=SUBSTITUTE
CM032587.1	2031894	.	G	A	100	*	TYPE=SUBSTITUTE
CM032587.1	2031898	.	A	C	100	*	TYPE=SUBSTITUTE
CM032587.1	2031903	.	G	A	100	*	TYPE=SUBSTITUTE
CM032587.1	2031907	.	G	A	100	*	TYPE=SUBSTITUTE
CM032587.1	2031993	.	G	C	100	*	TYPE=SUBSTITUTE
CM032587.1	2032023	.	T	C	100	*	TYPE=SUBSTITUTE
CM032587.1	2032024	.	G	T	100	*	TYPE=SUBSTITUTE
CM032587.1	2032046	.	T	G	100	*	TYPE=SUBSTITUTE
CM032587.1	2032051	.	A	G	100	*	TYPE=SUBSTITUTE
CM032587.1	2032120	.	G	A	100	*	TYPE=SUBSTITUTE
CM032587.1	2032127	.	G	C	100	*	TYPE=SUBSTITUTE
CM032587.1	2032133	.	C	T	100	*	TYPE=SUBSTITUTE
CM032587.1	2032174	.	T	C	100	*	TYPE=SUBSTITUTE
CM032587.1	2032196	.	T	C	100	*	TYPE=SUBSTITUTE
CM032587.1	2032233	.	C	T	100	*	TYPE=SUBSTITUTE
CM032587.1	2032276	.	G	A	100	*	TYPE=SUBSTITUTE
CM032587.1	2032320	.	T	C	100	*	TYPE=SUBSTITUTE
CM032587.1	2032427	.	G	GG	100	*	TYPE=INSERT
CM032587.1	2032428	.	C	T	100	*	TYPE=SUBSTITUTE
CM032587.1	2032446	.	G	A	100	*	TYPE=SUBSTITUTE
CM032587.1	2032506	.	A	G	100	*	TYPE=SUBSTITUTE

CM032587.1	2032593	.	A	G	100	*	TYPE=SUBSTITUTE
CM032587.1	2032607	.	G	A	100	*	TYPE=SUBSTITUTE
CM032587.1	2032627	.	T	C	100	*	TYPE=SUBSTITUTE
CM032587.1	2032655	.	A	G	100	*	TYPE=SUBSTITUTE
CM032587.1	2032715	.	T	A	100	*	TYPE=SUBSTITUTE
CM032587.1	2032740	.	ATTA	A	100	*	TYPE=DELETE
CM032587.1	2032767	.	C	T	100	*	TYPE=SUBSTITUTE
CM032587.1	2032792	.	A	C	100	*	TYPE=SUBSTITUTE
CM032587.1	2032810	.	G	a	100	*	TYPE=SUBSTITUTE
CM032587.1	2032813	.	t	tGTTCAA	100	*	TYPE=INSERT
CM032587.1	2032829	.	A	G	100	*	TYPE=SUBSTITUTE
CM032587.1	2032833	.	C	T	100	*	TYPE=SUBSTITUTE
CM032587.1	2032856	.	A	G	100	*	TYPE=SUBSTITUTE
CM032587.1	2032997	.	C	T	100	*	TYPE=SUBSTITUTE
CM032587.1	2033052	.	C	T	100	*	TYPE=SUBSTITUTE
CM032587.1	2033053	.	G	T	100	*	TYPE=SUBSTITUTE
CM032587.1	2033065	.	C	T	100	*	TYPE=SUBSTITUTE
CM032587.1	2033117	.	T	C	100	*	TYPE=SUBSTITUTE
CM032587.1	2033129	.	G	T	100	*	TYPE=SUBSTITUTE
CM032587.1	2033131	.	C	T	100	*	TYPE=SUBSTITUTE
CM032587.1	2033141	.	A	C	100	*	TYPE=SUBSTITUTE
CM032587.1	2033142	.	C	T	100	*	TYPE=SUBSTITUTE
CM032587.1	2033210	.	T	G	100	*	TYPE=SUBSTITUTE
CM032587.1	2033225	.	T	A	100	*	TYPE=SUBSTITUTE
CM032587.1	2033228	.	G	A	100	*	TYPE=SUBSTITUTE
CM032587.1	2033421	.	T	C	100	*	TYPE=SUBSTITUTE
CM032587.1	2033422	.	T	C	100	*	TYPE=SUBSTITUTE
CM032587.1	2033485	.	T	G	100	*	TYPE=SUBSTITUTE
CM032587.1	2033486	.	G	A	100	*	TYPE=SUBSTITUTE
CM032587.1	2033590	.	T	C	100	*	TYPE=SUBSTITUTE
CM032587.1	2033591	.	T	C	100	*	TYPE=SUBSTITUTE
CM032587.1	2033664	.	C	T	100	*	TYPE=SUBSTITUTE
CM032587.1	2033665	.	C	G	100	*	TYPE=SUBSTITUTE
CM032587.1	2033719	.	A	G	100	*	TYPE=SUBSTITUTE
CM032587.1	2033722	.	G	A	100	*	TYPE=SUBSTITUTE
CM032587.1	2033723	.	G	A	100	*	TYPE=SUBSTITUTE
CM032587.1	2033726	.	G	T	100	*	TYPE=SUBSTITUTE
CM032587.1	2033727	.	T	C	100	*	TYPE=SUBSTITUTE
CM032587.1	2033728	.	G	T	100	*	TYPE=SUBSTITUTE
CM032587.1	2033756	.	G	A	100	*	TYPE=SUBSTITUTE
CM032587.1	2033788	.	A	G	100	*	TYPE=SUBSTITUTE
CM032587.1	2033800	.	G	T	100	*	TYPE=SUBSTITUTE
CM032587.1	2033816	.	G	A	100	*	TYPE=SUBSTITUTE
CM032587.1	2033825	.	A	G	100	*	TYPE=SUBSTITUTE
CM032587.1	2033828	.	G	A	100	*	TYPE=SUBSTITUTE
CM032587.1	2033880	.	C	T	100	*	TYPE=SUBSTITUTE
CM032587.1	2033889	.	G	T	100	*	TYPE=SUBSTITUTE
CM032587.1	2033912	.	G	A	100	*	TYPE=SUBSTITUTE
CM032587.1	2033927	.	A	G	100	*	TYPE=SUBSTITUTE
CM032587.1	2033945	.	T	C	100	*	TYPE=SUBSTITUTE
CM032587.1	2033970	.	G	T	100	*	TYPE=SUBSTITUTE
CM032587.1	2034014	.	A	G	100	*	TYPE=SUBSTITUTE
CM032587.1	2034035	.	C	T	100	*	TYPE=SUBSTITUTE
CM032587.1	2034044	.	T	C	100	*	TYPE=SUBSTITUTE
CM032587.1	2034047	.	A	G	100	*	TYPE=SUBSTITUTE
CM032587.1	2034102	.	T	C	100	*	TYPE=SUBSTITUTE
CM032587.1	2034167	.	G	A	100	*	TYPE=SUBSTITUTE
CM032587.1	2034168	.	T	C	100	*	TYPE=SUBSTITUTE
CM032587.1	2034180	.	T	G	100	*	TYPE=SUBSTITUTE
CM032587.1	2034183	.	T	A	100	*	TYPE=SUBSTITUTE
CM032587.1	2034216	.	G	T	100	*	TYPE=SUBSTITUTE
CM032587.1	2034251	.	C	T	100	*	TYPE=SUBSTITUTE
CM032587.1	2034255	.	A	C	100	*	TYPE=SUBSTITUTE
CM032587.1	2034257	.	C	T	100	*	TYPE=SUBSTITUTE

CM032587.1	2034295	.	A	G	100	*	TYPE=SUBSTITUTE
CM032587.1	2034299	.	G	A	100	*	TYPE=SUBSTITUTE
CM032587.1	2034301	.	T	C	100	*	TYPE=SUBSTITUTE
CM032587.1	2034316	.	A	G	100	*	TYPE=SUBSTITUTE
CM032587.1	2034318	.	T	G	100	*	TYPE=SUBSTITUTE
CM032587.1	2034323	.	T	C	100	*	TYPE=SUBSTITUTE
CM032587.1	2034326	.	G	A	100	*	TYPE=SUBSTITUTE
CM032587.1	2034349	.	G	T	100	*	TYPE=SUBSTITUTE
CM032587.1	2034356	.	G	A	100	*	TYPE=SUBSTITUTE
CM032587.1	2034365	.	A	G	100	*	TYPE=SUBSTITUTE
CM032587.1	2034395	.	A	C	100	*	TYPE=SUBSTITUTE
CM032587.1	2034430	.	T	G	100	*	TYPE=SUBSTITUTE
CM032587.1	2034435	.	A	T	100	*	TYPE=SUBSTITUTE
CM032587.1	2034443	.	A	T	100	*	TYPE=SUBSTITUTE
CM032587.1	2034444	.	G	A	100	*	TYPE=SUBSTITUTE
CM032587.1	2034446	.	C	T	100	*	TYPE=SUBSTITUTE
CM032587.1	2034462	.	A	G	100	*	TYPE=SUBSTITUTE
CM032587.1	2034464	.	T	C	100	*	TYPE=SUBSTITUTE
CM032587.1	2034467	.	T	G	100	*	TYPE=SUBSTITUTE
CM032587.1	2034480	.	T	G	100	*	TYPE=SUBSTITUTE
CM032587.1	2034484	.	G	A	100	*	TYPE=SUBSTITUTE
CM032587.1	2034485	.	G	T	100	*	TYPE=SUBSTITUTE
CM032587.1	2034495	.	G	A	100	*	TYPE=SUBSTITUTE
CM032587.1	2034503	.	T	G	100	*	TYPE=SUBSTITUTE
CM032587.1	2034504	.	C	T	100	*	TYPE=SUBSTITUTE
CM032587.1	2034553	.	T	G	100	*	TYPE=SUBSTITUTE
CM032587.1	2034559	.	C	T	100	*	TYPE=SUBSTITUTE
CM032587.1	2034569	.	C	T	100	*	TYPE=SUBSTITUTE
CM032587.1	2034574	.	T	G	100	*	TYPE=SUBSTITUTE
CM032587.1	2034578	.	C	t	100	*	TYPE=SUBSTITUTE
CM032587.1	2034585	.	G	a	100	*	TYPE=SUBSTITUTE
CM032587.1	2034589	.	G	t	100	*	TYPE=SUBSTITUTE
CM032587.1	2034590	.	A	g	100	*	TYPE=SUBSTITUTE
CM032587.1	2034592	.	G	t	100	*	TYPE=SUBSTITUTE
CM032587.1	2034617	.	g	gagatggagatg	100	*	TYPE=INSERT
CM032587.1	2034619	.	a	ag	100	*	TYPE=INSERT
CM032587.1	2034630	.	C	G	100	*	TYPE=SUBSTITUTE
CM032587.1	2034676	.	C	G	100	*	TYPE=SUBSTITUTE
CM032587.1	2034682	.	G	A	100	*	TYPE=SUBSTITUTE
CM032587.1	2034689	.	G	T	100	*	TYPE=SUBSTITUTE
CM032587.1	2034702	.	T	C	100	*	TYPE=SUBSTITUTE
CM032587.1	2034708	.	G	A	100	*	TYPE=SUBSTITUTE
CM032587.1	2034709	.	A	G	100	*	TYPE=SUBSTITUTE
CM032587.1	2034711	.	TA	T	100	*	TYPE=DELETE
CM032587.1	2034718	.	G	A	100	*	TYPE=SUBSTITUTE
CM032587.1	2034732	.	T	C	100	*	TYPE=SUBSTITUTE
CM032587.1	2034735	.	A	T	100	*	TYPE=SUBSTITUTE
CM032587.1	2034766	.	T	TT	100	*	TYPE=INSERT
CM032587.1	2034794	.	A	G	100	*	TYPE=SUBSTITUTE
CM032587.1	2034796	.	A	ATATCGTTGCtctgtgtacatg	100	*	TYPE=INSERT
CM032587.1	2034807	.	C	CCACCA	100	*	TYPE=INSERT
CM032587.1	2034837	.	A	C	100	*	TYPE=SUBSTITUTE
CM032587.1	2034870	.	a	acctagta	100	*	TYPE=INSERT
CM032587.1	2034882	.	C	T	100	*	TYPE=SUBSTITUTE
CM032587.1	2034902	.	G	T	100	*	TYPE=SUBSTITUTE
CM032587.1	2034951	.	G	A	100	*	TYPE=SUBSTITUTE
CM032587.1	2034990	.	CCTTAGTAGGTGT	C	100	*	TYPE=DELETE
CM032587.1	2035015	.	A	ATAGG	100	*	TYPE=INSERT
CM032587.1	2035036	.	G	GACAATG	100	*	TYPE=INSERT
CM032587.1	2035054	.	C	T	100	*	TYPE=SUBSTITUTE
CM032587.1	2035057	.	G	A	100	*	TYPE=SUBSTITUTE
CM032587.1	2035068	.	T	c	100	*	TYPE=SUBSTITUTE
CM032587.1	2035111	.	A	AC	100	*	TYPE=INSERT

CM032587.1	2035126	.	A	G	100	*	TYPE=SUBSTITUTE
CM032587.1	2035129	.	T	G	100	*	TYPE=SUBSTITUTE
CM032587.1	2035132	.	G	A	100	*	TYPE=SUBSTITUTE
CM032587.1	2035137	.	G	A	100	*	TYPE=SUBSTITUTE
CM032587.1	2035138	.	T	C	100	*	TYPE=SUBSTITUTE
CM032587.1	2035150	.	T	C	100	*	TYPE=SUBSTITUTE
CM032587.1	2035163	.	T	C	100	*	TYPE=SUBSTITUTE
CM032587.1	2035280	.	T	C	100	*	TYPE=SUBSTITUTE
CM032587.1	2035281	.	C	T	100	*	TYPE=SUBSTITUTE
CM032587.1	2035300	.	C	T	100	*	TYPE=SUBSTITUTE
CM032587.1	2035318	.	A	G	100	*	TYPE=SUBSTITUTE
CM032587.1	2035333	.	T	A	100	*	TYPE=SUBSTITUTE
CM032587.1	2035371	.	T	C	100	*	TYPE=SUBSTITUTE
CM032587.1	2035391	.	A	C	100	*	TYPE=SUBSTITUTE
CM032587.1	2035411	.	G	C	100	*	TYPE=SUBSTITUTE
CM032587.1	2035427	.	C	G	100	*	TYPE=SUBSTITUTE
CM032587.1	2035461	.	T	C	100	*	TYPE=SUBSTITUTE
CM032587.1	2035478	.	G	A	100	*	TYPE=SUBSTITUTE
CM032587.1	2035481	.	A	G	100	*	TYPE=SUBSTITUTE
CM032587.1	2035492	.	C	T	100	*	TYPE=SUBSTITUTE
CM032587.1	2035493	.	G	GGAGTATA	100	*	TYPE=INSERT
CM032587.1	2035496	.	C	A	100	*	TYPE=SUBSTITUTE
CM032587.1	2035497	.	A	G	100	*	TYPE=SUBSTITUTE
CM032587.1	2035527	.	T	A	100	*	TYPE=SUBSTITUTE
CM032587.1	2035534	.	G	A	100	*	TYPE=SUBSTITUTE
CM032587.1	2035558	.	G	A	100	*	TYPE=SUBSTITUTE
CM032587.1	2035571	.	T	C	100	*	TYPE=SUBSTITUTE
CM032587.1	2035573	.	G	A	100	*	TYPE=SUBSTITUTE
CM032587.1	2035576	.	T	TATA	100	*	TYPE=INSERT
CM032587.1	2035577	.	G	C	100	*	TYPE=SUBSTITUTE
CM032587.1	2035579	.	G	T	100	*	TYPE=SUBSTITUTE
CM032587.1	2035580	.	T	A	100	*	TYPE=SUBSTITUTE
CM032587.1	2035582	.	T	G	100	*	TYPE=SUBSTITUTE
CM032587.1	2035583	.	G	T	100	*	TYPE=SUBSTITUTE
CM032587.1	2035590	.	T	g	100	*	TYPE=SUBSTITUTE
CM032587.1	2035591	.	c	catacatgtatacat	100	*	TYPE=INSERT
CM032587.1	2035592	.	T	g	100	*	TYPE=SUBSTITUTE
CM032587.1	2035594	.	C	T	100	*	TYPE=SUBSTITUTE
CM032587.1	2035640	.	T	G	100	*	TYPE=SUBSTITUTE
CM032587.1	2035641	.	A	G	100	*	TYPE=SUBSTITUTE
CM032587.1	2035663	.	T	C	100	*	TYPE=SUBSTITUTE
CM032587.1	2035664	.	G	C	100	*	TYPE=SUBSTITUTE
CM032587.1	2035669	.	G	A	100	*	TYPE=SUBSTITUTE
CM032587.1	2035684	.	CACAGTACT	C	100	*	TYPE=DELETE
CM032587.1	2035693	.	C	G	100	*	TYPE=SUBSTITUTE
CM032587.1	2035696	.	T	C	100	*	TYPE=SUBSTITUTE
CM032587.1	2035698	.	T	G	100	*	TYPE=SUBSTITUTE
CM032587.1	2035703	.	G	GA	100	*	TYPE=INSERT
CM032587.1	2035704	.	G	C	100	*	TYPE=SUBSTITUTE
CM032587.1	2035721	.	A	C	100	*	TYPE=SUBSTITUTE
CM032587.1	2035746	.	CA	C	100	*	TYPE=DELETE
CM032587.1	2035752	.	G	A	100	*	TYPE=SUBSTITUTE
CM032587.1	2035763	.	T	TG	100	*	TYPE=INSERT
CM032587.1	2035766	.	A	C	100	*	TYPE=SUBSTITUTE
CM032587.1	2035771	.	C	G	100	*	TYPE=SUBSTITUTE
CM032587.1	2035775	.	AGAGGCCT	A	100	*	TYPE=DELETE
CM032587.1	2035785	.	GA	G	100	*	TYPE=DELETE
CM032587.1	2035790	.	G	C	100	*	TYPE=SUBSTITUTE
CM032587.1	2035793	.	GACA	G	100	*	TYPE=DELETE
CM032587.1	2035798	.	T	A	100	*	TYPE=SUBSTITUTE
CM032587.1	2035799	.	A	G	100	*	TYPE=SUBSTITUTE
CM032587.1	2035815	.	T	C	100	*	TYPE=SUBSTITUTE
CM032587.1	2035816	.	C	T	100	*	TYPE=SUBSTITUTE
CM032587.1	2035832	.	C	T	100	*	TYPE=SUBSTITUTE

CM032587.1	2035837	.	C	T	100	*	TYPE=SUBSTITUTE
CM032587.1	2035838	.	G	A	100	*	TYPE=SUBSTITUTE
CM032587.1	2035914	.	A	T	100	*	TYPE=SUBSTITUTE
CM032587.1	2035915	.	T	C	100	*	TYPE=SUBSTITUTE
CM032587.1	2035916	.	G	T	100	*	TYPE=SUBSTITUTE
CM032587.1	2035918	.	TTG	T	100	*	TYPE=DELETE
CM032587.1	2035928	.	T	TA	100	*	TYPE=INSERT
CM032587.1	2035945	.	C	G	100	*	TYPE=SUBSTITUTE
CM032587.1	2035951	.	G	GTTGCAC	100	*	TYPE=INSERT
CM032587.1	2035960	.	G	GGGATGTGACC	100	*	TYPE=INSERT
CM032587.1	2035963	.	G	A	100	*	TYPE=SUBSTITUTE
CM032587.1	2035969	.	T	TTGAG	100	*	TYPE=INSERT
CM032587.1	2035989	.	TTG	T	100	*	TYPE=DELETE
CM032587.1	2035992	.	A	G	100	*	TYPE=SUBSTITUTE
CM032587.1	2035999	.	C	T	100	*	TYPE=SUBSTITUTE
CM032587.1	2036046	.	C	G	100	*	TYPE=SUBSTITUTE
CM032587.1	2036049	.	T	C	100	*	TYPE=SUBSTITUTE
CM032587.1	2036052	.	A	AT	100	*	TYPE=INSERT
CM032587.1	2036109	.	A	G	100	*	TYPE=SUBSTITUTE
CM032587.1	2036110	.	G	C	100	*	TYPE=SUBSTITUTE
CM032587.1	2036112	.	A	AAT	100	*	TYPE=INSERT
CM032587.1	2036117	.	T	C	100	*	TYPE=SUBSTITUTE
CM032587.1	2036124	.	ATTCTTCCT	A	100	*	TYPE=DELETE
CM032587.1	2036139	.	G	A	100	*	TYPE=SUBSTITUTE
CM032587.1	2036160	.	T	A	100	*	TYPE=SUBSTITUTE
CM032587.1	2036161	.	T	A	100	*	TYPE=SUBSTITUTE
CM032587.1	2036164	.	C	CCATTTG	100	*	TYPE=INSERT
CM032587.1	2036177	.	TTTTG	T	100	*	TYPE=DELETE
CM032587.1	2036182	.	T	A	100	*	TYPE=SUBSTITUTE
CM032587.1	2036251	.	T	TT	100	*	TYPE=INSERT
CM032587.1	2036265	.	G	c	100	*	TYPE=SUBSTITUTE
CM032587.1	2036267	.	t	ttcttcctcttaaC	100	*	TYPE=INSERT
CM032587.1	2036279	.	A	G	100	*	TYPE=SUBSTITUTE
CM032587.1	2036316	.	T	C	100	*	TYPE=SUBSTITUTE
CM032587.1	2036394	.	G	a	100	*	TYPE=SUBSTITUTE
CM032587.1	2036397	.	TC	T	100	*	TYPE=DELETE
CM032587.1	2036421	.	A	G	100	*	TYPE=SUBSTITUTE
CM032587.1	2036423	.	C	A	100	*	TYPE=SUBSTITUTE
CM032587.1	2036458	.	T	C	100	*	TYPE=SUBSTITUTE
CM032587.1	2036473	.	C	t	100	*	TYPE=SUBSTITUTE
CM032587.1	2036485	.	T	C	100	*	TYPE=SUBSTITUTE
CM032587.1	2036512	.	T	C	100	*	TYPE=SUBSTITUTE
CM032587.1	2036535	.	A	G	100	*	TYPE=SUBSTITUTE
CM032587.1	2036608	.	A	g	100	*	TYPE=SUBSTITUTE
CM032587.1	2036701	.	C	T	100	*	TYPE=SUBSTITUTE
CM032587.1	2036713	.	T	C	100	*	TYPE=SUBSTITUTE
CM032587.1	2036839	.	G	A	100	*	TYPE=SUBSTITUTE
CM032587.1	2036871	.	a	aaca	100	*	TYPE=INSERT
CM032587.1	2036881	.	A	g	100	*	TYPE=SUBSTITUTE
CM032587.1	2036884	.	G	a	100	*	TYPE=SUBSTITUTE
CM032587.1	2036910	.	AGCAGCAACAACA	A	100	*	TYPE=DELETE
CM032587.1	2036929	.	A	g	100	*	TYPE=SUBSTITUTE
CM032587.1	2036935	.	T	C	100	*	TYPE=SUBSTITUTE
CM032587.1	2037034	.	A	G	100	*	TYPE=SUBSTITUTE
CM032587.1	2037076	.	G	A	100	*	TYPE=SUBSTITUTE
CM032587.1	2037145	.	T	C	100	*	TYPE=SUBSTITUTE
CM032587.1	2037160	.	G	C	100	*	TYPE=SUBSTITUTE
CM032587.1	2037163	.	T	C	100	*	TYPE=SUBSTITUTE
CM032587.1	2037204	.	C	T	100	*	TYPE=SUBSTITUTE
CM032587.1	2037211	.	C	T	100	*	TYPE=SUBSTITUTE
CM032587.1	2037215	.	T	C	100	*	TYPE=SUBSTITUTE
CM032587.1	2037216	.	A	G	100	*	TYPE=SUBSTITUTE
CM032587.1	2037220	.	A	G	100	*	TYPE=SUBSTITUTE
CM032587.1	2037226	.	G	A	100	*	TYPE=SUBSTITUTE

CM032587.1	2037229	.	T	A	100	*	TYPE=SUBSTITUTE
CM032587.1	2037232	.	T	G	100	*	TYPE=SUBSTITUTE
CM032587.1	2037235	.	G	A	100	*	TYPE=SUBSTITUTE
CM032587.1	2037247	.	T	C	100	*	TYPE=SUBSTITUTE
CM032587.1	2037251	.	G	A	100	*	TYPE=SUBSTITUTE
CM032587.1	2037261	.	T	A	100	*	TYPE=SUBSTITUTE
CM032587.1	2037265	.	TA	T	100	*	TYPE=DELETE
CM032587.1	2037359	.	C	T	100	*	TYPE=SUBSTITUTE
CM032587.1	2037374	.	G	C	100	*	TYPE=SUBSTITUTE
CM032587.1	2037389	.	C	T	100	*	TYPE=SUBSTITUTE
CM032587.1	2037471	.	C	T	100	*	TYPE=SUBSTITUTE
CM032587.1	2037476	.	C	T	100	*	TYPE=SUBSTITUTE
CM032587.1	2037482	.	T	C	100	*	TYPE=SUBSTITUTE
CM032587.1	2037515	.	C	T	100	*	TYPE=SUBSTITUTE
CM032587.1	2037542	.	G	A	100	*	TYPE=SUBSTITUTE
CM032587.1	2037548	.	A	G	100	*	TYPE=SUBSTITUTE
CM032587.1	2037602	.	C	T	100	*	TYPE=SUBSTITUTE
CM032587.1	2037605	.	T	C	100	*	TYPE=SUBSTITUTE
CM032587.1	2037608	.	C	T	100	*	TYPE=SUBSTITUTE
CM032587.1	2037627	.	C	A	100	*	TYPE=SUBSTITUTE
CM032587.1	2037630	.	T	C	100	*	TYPE=SUBSTITUTE
CM032587.1	2037635	.	G	C	100	*	TYPE=SUBSTITUTE
CM032587.1	2037636	.	T	C	100	*	TYPE=SUBSTITUTE
CM032587.1	2037637	.	T	C	100	*	TYPE=SUBSTITUTE
CM032587.1	2037641	.	C	T	100	*	TYPE=SUBSTITUTE
CM032587.1	2037647	.	T	C	100	*	TYPE=SUBSTITUTE
CM032587.1	2037649	.	T	A	100	*	TYPE=SUBSTITUTE
CM032587.1	2037650	.	T	C	100	*	TYPE=SUBSTITUTE
CM032587.1	2037651	.	T	C	100	*	TYPE=SUBSTITUTE
CM032587.1	2037652	.	A	T	100	*	TYPE=SUBSTITUTE
CM032587.1	2037655	.	T	G	100	*	TYPE=SUBSTITUTE
CM032587.1	2037664	.	C	T	100	*	TYPE=SUBSTITUTE
CM032587.1	2037668	.	C	T	100	*	TYPE=SUBSTITUTE
CM032587.1	2037674	.	AG	A	100	*	TYPE=DELETE
CM032587.1	2037684	.	A	G	100	*	TYPE=SUBSTITUTE
CM032587.1	2037711	.	C	T	100	*	TYPE=SUBSTITUTE
CM032587.1	2037714	.	C	T	100	*	TYPE=SUBSTITUTE
CM032587.1	2037729	.	C	T	100	*	TYPE=SUBSTITUTE
CM032587.1	2037750	.	C	T	100	*	TYPE=SUBSTITUTE
CM032587.1	2037795	.	T	C	100	*	TYPE=SUBSTITUTE
CM032587.1	2037825	.	C	T	100	*	TYPE=SUBSTITUTE
CM032587.1	2037843	.	G	A	100	*	TYPE=SUBSTITUTE
CM032587.1	2037861	.	C	T	100	*	TYPE=SUBSTITUTE
CM032587.1	2037870	.	C	T	100	*	TYPE=SUBSTITUTE
CM032587.1	2037881	.	CT	C	100	*	TYPE=DELETE
CM032587.1	2037909	.	G	A	100	*	TYPE=SUBSTITUTE
CM032587.1	2037911	.	G	A	100	*	TYPE=SUBSTITUTE
CM032587.1	2037931	.	T	C	100	*	TYPE=SUBSTITUTE
CM032587.1	2037955	.	G	A	100	*	TYPE=SUBSTITUTE
CM032587.1	2037965	.	T	C	100	*	TYPE=SUBSTITUTE
CM032587.1	2037968	.	C	A	100	*	TYPE=SUBSTITUTE
CM032587.1	2037976	.	A	G	100	*	TYPE=SUBSTITUTE
CM032587.1	2037990	.	T	tttg	100	*	TYPE=INSERT
CM032587.1	2038009	.	A	C	100	*	TYPE=SUBSTITUTE
CM032587.1	2038053	.	C	T	100	*	TYPE=SUBSTITUTE
CM032587.1	2038070	.	A	G	100	*	TYPE=SUBSTITUTE
CM032587.1	2038089	.	C	T	100	*	TYPE=SUBSTITUTE
CM032587.1	2038105	.	A	G	100	*	TYPE=SUBSTITUTE
CM032587.1	2038112	.	A	G	100	*	TYPE=SUBSTITUTE
CM032587.1	2038158	.	A	G	100	*	TYPE=SUBSTITUTE
CM032587.1	2038169	.	G	C	100	*	TYPE=SUBSTITUTE
CM032587.1	2038171	.	C	T	100	*	TYPE=SUBSTITUTE
CM032587.1	2038177	.	C	T	100	*	TYPE=SUBSTITUTE
CM032587.1	2038181	.	C	T	100	*	TYPE=SUBSTITUTE

CM032587.1	2038183	.	C	A	100	*	TYPE=SUBSTITUTE
CM032587.1	2038185	.	C	g	100	*	TYPE=SUBSTITUTE
CM032587.1	2038188	.	T	g	100	*	TYPE=SUBSTITUTE
CM032587.1	2038194	.	TCTCTTTTCTCTC	T	100	*	TYPE=DELETE
CM032587.1	2038208	.	T	c	100	*	TYPE=SUBSTITUTE
CM032587.1	2038223	.	g	ga	100	*	TYPE=INSERT
CM032587.1	2038225	.	G	c	100	*	TYPE=SUBSTITUTE
CM032587.1	2038227	.	A	t	100	*	TYPE=SUBSTITUTE
CM032587.1	2038234	.	t	ttctccccc	100	*	TYPE=INSERT
CM032587.1	2038237	.	C	a	100	*	TYPE=SUBSTITUTE
CM032587.1	2038256	.	A	T	100	*	TYPE=SUBSTITUTE
CM032587.1	2038262	.	GTTGACGGAGGGAGCAAAAAGCCTTAACAAATCAC	G	100	*	TYPE=DELETE
CM032587.1	2038308	.	G	A	100	*	TYPE=SUBSTITUTE
CM032587.1	2038309	.	A	G	100	*	TYPE=SUBSTITUTE
CM032587.1	2038320	.	C	T	100	*	TYPE=SUBSTITUTE
CM032587.1	2038346	.	T	C	100	*	TYPE=SUBSTITUTE
CM032587.1	2038372	.	A	G	100	*	TYPE=SUBSTITUTE
CM032587.1	2038382	.	A	T	100	*	TYPE=SUBSTITUTE
CM032587.1	2038386	.	G	T	100	*	TYPE=SUBSTITUTE
CM032587.1	2038390	.	C	A	100	*	TYPE=SUBSTITUTE
CM032587.1	2038394	.	C	T	100	*	TYPE=SUBSTITUTE
CM032587.1	2038396	.	A	C	100	*	TYPE=SUBSTITUTE
CM032587.1	2038408	.	G	A	100	*	TYPE=SUBSTITUTE
CM032587.1	2038426	.	T	C	100	*	TYPE=SUBSTITUTE
CM032587.1	2038439	.	A	G	100	*	TYPE=SUBSTITUTE
CM032587.1	2038444	.	T	C	100	*	TYPE=SUBSTITUTE
CM032587.1	2038446	.	T	G	100	*	TYPE=SUBSTITUTE
CM032587.1	2038456	.	A	G	100	*	TYPE=SUBSTITUTE
CM032587.1	2038479	.	G	A	100	*	TYPE=SUBSTITUTE
CM032587.1	2038480	.	T	A	100	*	TYPE=SUBSTITUTE
CM032587.1	2038481	.	ATCTC	A	100	*	TYPE=DELETE
CM032587.1	2038535	.	A	AA	100	*	TYPE=INSERT
CM032587.1	2038581	.	G	a	100	*	TYPE=SUBSTITUTE
CM032587.1	2038590	.	GC	G	100	*	TYPE=DELETE
CM032587.1	2038599	.	GCCTT	G	100	*	TYPE=DELETE
CM032587.1	2038605	.	G	C	100	*	TYPE=SUBSTITUTE
CM032587.1	2038661	.	G	A	100	*	TYPE=SUBSTITUTE
CM032587.1	2038674	.	C	T	100	*	TYPE=SUBSTITUTE
CM032587.1	2038686	.	T	C	100	*	TYPE=SUBSTITUTE
CM032587.1	2038705	.	G	A	100	*	TYPE=SUBSTITUTE
CM032587.1	2038721	.	C	T	100	*	TYPE=SUBSTITUTE
CM032587.1	2038735	.	C	A	100	*	TYPE=SUBSTITUTE
CM032587.1	2038749	.	C	T	100	*	TYPE=SUBSTITUTE
CM032587.1	2038757	.	C	T	100	*	TYPE=SUBSTITUTE
CM032587.1	2038762	.	C	T	100	*	TYPE=SUBSTITUTE
CM032587.1	2038774	.	T	C	100	*	TYPE=SUBSTITUTE
CM032587.1	2038794	.	T	G	100	*	TYPE=SUBSTITUTE
CM032587.1	2038843	.	CG	C	100	*	TYPE=DELETE
CM032587.1	2038845	.	T	A	100	*	TYPE=SUBSTITUTE
CM032587.1	2038853	.	A	G	100	*	TYPE=SUBSTITUTE
CM032587.1	2038905	.	A	g	100	*	TYPE=SUBSTITUTE
CM032587.1	2038917	.	T	C	100	*	TYPE=SUBSTITUTE
CM032587.1	2038927	.	C	A	100	*	TYPE=SUBSTITUTE
CM032587.1	2038938	.	A	G	100	*	TYPE=SUBSTITUTE
CM032587.1	2038955	.	A	G	100	*	TYPE=SUBSTITUTE
CM032587.1	2038971	.	T	C	100	*	TYPE=SUBSTITUTE
CM032587.1	2038976	.	C	T	100	*	TYPE=SUBSTITUTE
CM032587.1	2038981	.	C	G	100	*	TYPE=SUBSTITUTE
CM032587.1	2039003	.	C	A	100	*	TYPE=SUBSTITUTE
CM032587.1	2039006	.	T	A	100	*	TYPE=SUBSTITUTE
CM032587.1	2039010	.	A	G	100	*	TYPE=SUBSTITUTE
CM032587.1	2039032	.	G	C	100	*	TYPE=SUBSTITUTE
CM032587.1	2039054	.	G	A	100	*	TYPE=SUBSTITUTE

CM032587.1	2039081	.	C	T	100	*	TYPE=SUBSTITUTE
CM032587.1	2039120	.	A	G	100	*	TYPE=SUBSTITUTE
CM032587.1	2039145	.	A	G	100	*	TYPE=SUBSTITUTE
CM032587.1	2039212	.	G	A	100	*	TYPE=SUBSTITUTE
CM032587.1	2039218	.	G	A	100	*	TYPE=SUBSTITUTE
CM032587.1	2039226	.	G	A	100	*	TYPE=SUBSTITUTE
CM032587.1	2039230	.	A	G	100	*	TYPE=SUBSTITUTE
CM032587.1	2039243	.	C	T	100	*	TYPE=SUBSTITUTE
CM032587.1	2039248	.	A	G	100	*	TYPE=SUBSTITUTE
CM032587.1	2039249	.	G	A	100	*	TYPE=SUBSTITUTE
CM032587.1	2039309	.	A	C	100	*	TYPE=SUBSTITUTE
CM032587.1	2039345	.	G	A	100	*	TYPE=SUBSTITUTE
CM032587.1	2039346	.	A	G	100	*	TYPE=SUBSTITUTE
CM032587.1	2039354	.	G	A	100	*	TYPE=SUBSTITUTE
CM032587.1	2039372	.	A	G	100	*	TYPE=SUBSTITUTE
CM032587.1	2039393	.	T	C	100	*	TYPE=SUBSTITUTE
CM032587.1	2039414	.	C	T	100	*	TYPE=SUBSTITUTE
CM032587.1	2039423	.	A	G	100	*	TYPE=SUBSTITUTE
CM032587.1	2039432	.	C	T	100	*	TYPE=SUBSTITUTE
CM032587.1	2039434	.	G	A	100	*	TYPE=SUBSTITUTE
CM032587.1	2039441	.	T	TCTCCAG	100	*	TYPE=INSERT
CM032587.1	2039444	.	A	G	100	*	TYPE=SUBSTITUTE
CM032587.1	2039451	.	G	A	100	*	TYPE=SUBSTITUTE
CM032587.1	2039457	.	C	T	100	*	TYPE=SUBSTITUTE
CM032587.1	2039468	.	T	C	100	*	TYPE=SUBSTITUTE
CM032587.1	2039470	.	C	T	100	*	TYPE=SUBSTITUTE
CM032587.1	2039472	.	A	G	100	*	TYPE=SUBSTITUTE
CM032587.1	2039475	.	A	G	100	*	TYPE=SUBSTITUTE
CM032587.1	2039476	.	T	C	100	*	TYPE=SUBSTITUTE
CM032587.1	2039477	.	T	A	100	*	TYPE=SUBSTITUTE
CM032587.1	2039483	.	T	C	100	*	TYPE=SUBSTITUTE
CM032587.1	2039495	.	C	T	100	*	TYPE=SUBSTITUTE
CM032587.1	2039514	.	A	G	100	*	TYPE=SUBSTITUTE
CM032587.1	2039516	.	C	A	100	*	TYPE=SUBSTITUTE
CM032587.1	2039539	.	A	G	100	*	TYPE=SUBSTITUTE
CM032587.1	2039553	.	A	c	100	*	TYPE=SUBSTITUTE
CM032587.1	2039558	.	A	g	100	*	TYPE=SUBSTITUTE
CM032587.1	2039564	.	A	g	100	*	TYPE=SUBSTITUTE
CM032587.1	2039571	.	A	G	100	*	TYPE=SUBSTITUTE
CM032587.1	2039573	.	C	T	100	*	TYPE=SUBSTITUTE
CM032587.1	2039582	.	C	A	100	*	TYPE=SUBSTITUTE
CM032587.1	2039586	.	T	C	100	*	TYPE=SUBSTITUTE
CM032587.1	2039594	.	G	A	100	*	TYPE=SUBSTITUTE
CM032587.1	2039603	.	T	G	100	*	TYPE=SUBSTITUTE
CM032587.1	2039606	.	G	A	100	*	TYPE=SUBSTITUTE
CM032587.1	2039636	.	T	G	100	*	TYPE=SUBSTITUTE
CM032587.1	2039652	.	A	G	100	*	TYPE=SUBSTITUTE
CM032587.1	2039678	.	A	G	100	*	TYPE=SUBSTITUTE
CM032587.1	2039696	.	A	G	100	*	TYPE=SUBSTITUTE
CM032587.1	2039707	.	G	C	100	*	TYPE=SUBSTITUTE
CM032587.1	2039724	.	A	C	100	*	TYPE=SUBSTITUTE
CM032587.1	2039729	.	G	A	100	*	TYPE=SUBSTITUTE
CM032587.1	2039735	.	C	T	100	*	TYPE=SUBSTITUTE
CM032587.1	2039756	.	C	G	100	*	TYPE=SUBSTITUTE
CM032587.1	2039814	.	A	C	100	*	TYPE=SUBSTITUTE
CM032587.1	2039827	.	A	G	100	*	TYPE=SUBSTITUTE
CM032587.1	2039838	.	T	A	100	*	TYPE=SUBSTITUTE
CM032587.1	2039839	.	T	C	100	*	TYPE=SUBSTITUTE
CM032587.1	2039855	.	A	G	100	*	TYPE=SUBSTITUTE
CM032587.1	2039877	.	G	A	100	*	TYPE=SUBSTITUTE
CM032587.1	2039907	.	G	A	100	*	TYPE=SUBSTITUTE
CM032587.1	2039924	.	C	T	100	*	TYPE=SUBSTITUTE
CM032587.1	2039936	.	C	T	100	*	TYPE=SUBSTITUTE
CM032587.1	2039954	.	C	T	100	*	TYPE=SUBSTITUTE

CM032587.1	2039969	.	C	A	100	*	TYPE=SUBSTITUTE
CM032587.1	2039991	.	T	C	100	*	TYPE=SUBSTITUTE
CM032587.1	2039996	.	T	G	100	*	TYPE=SUBSTITUTE
CM032587.1	2040000	.	T	G	100	*	TYPE=SUBSTITUTE
CM032587.1	2040001	.	C	T	100	*	TYPE=SUBSTITUTE
CM032587.1	2040008	.	G	A	100	*	TYPE=SUBSTITUTE
CM032587.1	2040012	.	G	C	100	*	TYPE=SUBSTITUTE
CM032587.1	2040020	.	T	C	100	*	TYPE=SUBSTITUTE
CM032587.1	2040026	.	C	T	100	*	TYPE=SUBSTITUTE
CM032587.1	2040034	.	A	G	100	*	TYPE=SUBSTITUTE
CM032587.1	2040058	.	A	G	100	*	TYPE=SUBSTITUTE
CM032587.1	2040064	.	G	A	100	*	TYPE=SUBSTITUTE
CM032587.1	2040068	.	C	T	100	*	TYPE=SUBSTITUTE
CM032587.1	2040092	.	C	A	100	*	TYPE=SUBSTITUTE
CM032587.1	2040155	.	G	A	100	*	TYPE=SUBSTITUTE
CM032587.1	2040164	.	T	C	100	*	TYPE=SUBSTITUTE
CM032587.1	2040185	.	C	T	100	*	TYPE=SUBSTITUTE
CM032587.1	2040186	.	A	G	100	*	TYPE=SUBSTITUTE
CM032587.1	2040190	.	C	G	100	*	TYPE=SUBSTITUTE
CM032587.1	2040203	.	A	G	100	*	TYPE=SUBSTITUTE
CM032587.1	2040207	.	G	C	100	*	TYPE=SUBSTITUTE
CM032587.1	2040218	.	T	G	100	*	TYPE=SUBSTITUTE
CM032587.1	2040229	.	A	G	100	*	TYPE=SUBSTITUTE
CM032587.1	2040230	.	C	T	100	*	TYPE=SUBSTITUTE
CM032587.1	2040237	.	G	A	100	*	TYPE=SUBSTITUTE
CM032587.1	2040244	.	C	G	100	*	TYPE=SUBSTITUTE
CM032587.1	2040246	.	T	G	100	*	TYPE=SUBSTITUTE
CM032587.1	2040286	.	G	A	100	*	TYPE=SUBSTITUTE
CM032587.1	2040341	.	T	C	100	*	TYPE=SUBSTITUTE
CM032587.1	2040362	.	C	T	100	*	TYPE=SUBSTITUTE
CM032587.1	2040392	.	G	A	100	*	TYPE=SUBSTITUTE
CM032587.1	2040393	.	C	G	100	*	TYPE=SUBSTITUTE
CM032587.1	2040446	.	T	C	100	*	TYPE=SUBSTITUTE
CM032587.1	2040469	.	A	G	100	*	TYPE=SUBSTITUTE
CM032587.1	2040544	.	T	C	100	*	TYPE=SUBSTITUTE
CM032587.1	2040561	.	A	C	100	*	TYPE=SUBSTITUTE
CM032587.1	2040585	.	C	T	100	*	TYPE=SUBSTITUTE
CM032587.1	2040596	.	A	C	100	*	TYPE=SUBSTITUTE
CM032587.1	2040607	.	T	C	100	*	TYPE=SUBSTITUTE
CM032587.1	2040630	.	C	G	100	*	TYPE=SUBSTITUTE
CM032587.1	2040674	.	G	T	100	*	TYPE=SUBSTITUTE
CM032587.1	2040684	.	A	G	100	*	TYPE=SUBSTITUTE
CM032587.1	2040720	.	T	C	100	*	TYPE=SUBSTITUTE
CM032587.1	2040724	.	C	T	100	*	TYPE=SUBSTITUTE
CM032587.1	2040737	.	C	T	100	*	TYPE=SUBSTITUTE
CM032587.1	2040764	.	T	C	100	*	TYPE=SUBSTITUTE
CM032587.1	2040777	.	G	A	100	*	TYPE=SUBSTITUTE
CM032587.1	2040791	.	A	G	100	*	TYPE=SUBSTITUTE
CM032587.1	2040792	.	C	T	100	*	TYPE=SUBSTITUTE
CM032587.1	2040794	.	T	C	100	*	TYPE=SUBSTITUTE
CM032587.1	2040795	.	G	A	100	*	TYPE=SUBSTITUTE
CM032587.1	2040815	.	C	T	100	*	TYPE=SUBSTITUTE
CM032587.1	2040849	.	G	A	100	*	TYPE=SUBSTITUTE
CM032587.1	2040878	.	G	A	100	*	TYPE=SUBSTITUTE
CM032587.1	2040953	.	T	C	100	*	TYPE=SUBSTITUTE
CM032587.1	2041019	.	G	A	100	*	TYPE=SUBSTITUTE
CM032587.1	2041021	.	C	T	100	*	TYPE=SUBSTITUTE
CM032587.1	2041032	.	C	T	100	*	TYPE=SUBSTITUTE
CM032587.1	2041062	.	A	G	100	*	TYPE=SUBSTITUTE
CM032587.1	2041087	.	A	C	100	*	TYPE=SUBSTITUTE
CM032587.1	2041091	.	G	C	100	*	TYPE=SUBSTITUTE
CM032587.1	2041092	.	A	G	100	*	TYPE=SUBSTITUTE
CM032587.1	2041093	.	G	A	100	*	TYPE=SUBSTITUTE
CM032587.1	2041109	.	C	T	100	*	TYPE=SUBSTITUTE

CM032587.1	2041114	.	C	T	100	*	TYPE=SUBSTITUTE
CM032587.1	2041120	.	G	A	100	*	TYPE=SUBSTITUTE
CM032587.1	2041125	.	A	G	100	*	TYPE=SUBSTITUTE
CM032587.1	2041128	.	A	C	100	*	TYPE=SUBSTITUTE
CM032587.1	2041147	.	G	GTGTCGTATAATGCAG	100	*	TYPE=INSERT
CM032587.1	2041151	.	C	T	100	*	TYPE=SUBSTITUTE
CM032587.1	2041153	.	C	T	100	*	TYPE=SUBSTITUTE
CM032587.1	2041155	.	C	G	100	*	TYPE=SUBSTITUTE
CM032587.1	2041190	.	CC	C	100	*	TYPE=DELETE
CM032587.1	2041199	.	CT	C	100	*	TYPE=DELETE
CM032587.1	2041224	.	A	G	100	*	TYPE=SUBSTITUTE
CM032587.1	2041225	.	A	G	100	*	TYPE=SUBSTITUTE
CM032587.1	2041228	.	A	T	100	*	TYPE=SUBSTITUTE
CM032587.1	2041243	.	CT	C	100	*	TYPE=DELETE
CM032587.1	2041254	.	T	c	100	*	TYPE=SUBSTITUTE
CM032587.1	2041255	.	C	t	100	*	TYPE=SUBSTITUTE
CM032587.1	2041257	.	G	C	100	*	TYPE=SUBSTITUTE
CM032587.1	2041264	.	A	G	100	*	TYPE=SUBSTITUTE
CM032587.1	2041265	.	A	G	100	*	TYPE=SUBSTITUTE
CM032587.1	2041278	.	G	C	100	*	TYPE=SUBSTITUTE
CM032587.1	2041307	.	T	A	100	*	TYPE=SUBSTITUTE
CM032587.1	2041312	.	C	T	100	*	TYPE=SUBSTITUTE
CM032587.1	2041342	.	T	C	100	*	TYPE=SUBSTITUTE
CM032587.1	2041346	.	T	C	100	*	TYPE=SUBSTITUTE
CM032587.1	2041349	.	T	C	100	*	TYPE=SUBSTITUTE
CM032587.1	2041355	.	T	C	100	*	TYPE=SUBSTITUTE
CM032587.1	2041382	.	A	G	100	*	TYPE=SUBSTITUTE
CM032587.1	2041389	.	C	g	100	*	TYPE=SUBSTITUTE
CM032587.1	2041418	.	G	a	100	*	TYPE=SUBSTITUTE
CM032587.1	2041436	.	A	G	100	*	TYPE=SUBSTITUTE
CM032587.1	2041465	.	C	A	100	*	TYPE=SUBSTITUTE
CM032587.1	2041467	.	T	C	100	*	TYPE=SUBSTITUTE
CM032587.1	2041478	.	T	C	100	*	TYPE=SUBSTITUTE
CM032587.1	2041486	.	A	T	100	*	TYPE=SUBSTITUTE
CM032587.1	2041503	.	A	G	100	*	TYPE=SUBSTITUTE
CM032587.1	2041504	.	G	A	100	*	TYPE=SUBSTITUTE
CM032587.1	2041505	.	C	G	100	*	TYPE=SUBSTITUTE
CM032587.1	2041508	.	A	G	100	*	TYPE=SUBSTITUTE
CM032587.1	2041594	.	T	C	100	*	TYPE=SUBSTITUTE
CM032587.1	2041645	.	ATAGAA	A	100	*	TYPE=DELETE
CM032587.1	2041656	.	C	T	100	*	TYPE=SUBSTITUTE
CM032587.1	2041703	.	T	C	100	*	TYPE=SUBSTITUTE
CM032587.1	2041724	.	G	A	100	*	TYPE=SUBSTITUTE
CM032587.1	2041771	.	T	c	100	*	TYPE=SUBSTITUTE
CM032587.1	2041775	.	G	T	100	*	TYPE=SUBSTITUTE
CM032587.1	2041809	.	T	c	100	*	TYPE=SUBSTITUTE
CM032587.1	2041863	.	G	a	100	*	TYPE=SUBSTITUTE
CM032587.1	2041865	.	t	ta	100	*	TYPE=INSERT
CM032587.1	2041886	.	G	GTGA	100	*	TYPE=INSERT
CM032587.1	2041888	.	T	g	100	*	TYPE=SUBSTITUTE
CM032587.1	2041889	.	A	g	100	*	TYPE=SUBSTITUTE
CM032587.1	2041893	.	t	tg	100	*	TYPE=INSERT
CM032587.1	2041902	.	C	a	100	*	TYPE=SUBSTITUTE
CM032587.1	2041908	.	T	a	100	*	TYPE=SUBSTITUTE
CM032587.1	2041917	.	c	ctagg	100	*	TYPE=INSERT
CM032587.1	2041960	.	A	AA	100	*	TYPE=INSERT
CM032587.1	2041977	.	G	GG	100	*	TYPE=INSERT
CM032587.1	2042003	.	C	a	100	*	TYPE=SUBSTITUTE
CM032587.1	2042050	.	T	c	100	*	TYPE=SUBSTITUTE
CM032587.1	2042065	.	C	T	100	*	TYPE=SUBSTITUTE
CM032587.1	2042079	.	T	A	100	*	TYPE=SUBSTITUTE
CM032587.1	2042083	.	T	A	100	*	TYPE=SUBSTITUTE
CM032587.1	2042085	.	T	C	100	*	TYPE=SUBSTITUTE
CM032587.1	2042086	.	C	T	100	*	TYPE=SUBSTITUTE

CM032587.1	2042145	.	A	T	100	*	TYPE=SUBSTITUTE
CM032587.1	2042168	.	G	T	100	*	TYPE=SUBSTITUTE
CM032587.1	2042178	.	C	G	100	*	TYPE=SUBSTITUTE
CM032587.1	2042179	.	T	G	100	*	TYPE=SUBSTITUTE
CM032587.1	2042180	.	A	T	100	*	TYPE=SUBSTITUTE
CM032587.1	2042241	.	ACCTA	A	100	*	TYPE=DELETE
CM032587.1	2042251	.	G	T	100	*	TYPE=SUBSTITUTE
CM032587.1	2042259	.	T	C	100	*	TYPE=SUBSTITUTE
CM032587.1	2042278	.	A	G	100	*	TYPE=SUBSTITUTE
CM032587.1	2042283	.	A	T	100	*	TYPE=SUBSTITUTE
CM032587.1	2042300	.	TT	T	100	*	TYPE=DELETE
CM032587.1	2042305	.	T	G	100	*	TYPE=SUBSTITUTE
CM032587.1	2042306	.	A	G	100	*	TYPE=SUBSTITUTE
CM032587.1	2042319	.	G	GGC	100	*	TYPE=INSERT
CM032587.1	2042339	.	C	T	100	*	TYPE=SUBSTITUTE
CM032587.1	2042341	.	T	A	100	*	TYPE=SUBSTITUTE
CM032587.1	2042372	.	G	A	100	*	TYPE=SUBSTITUTE
CM032587.1	2042374	.	T	C	100	*	TYPE=SUBSTITUTE
CM032587.1	2042378	.	C	T	100	*	TYPE=SUBSTITUTE
CM032587.1	2042380	.	T	C	100	*	TYPE=SUBSTITUTE
CM032587.1	2042382	.	A	G	100	*	TYPE=SUBSTITUTE
CM032587.1	2042384	.	A	C	100	*	TYPE=SUBSTITUTE
CM032587.1	2042385	.	G	C	100	*	TYPE=SUBSTITUTE
CM032587.1	2042417	.	G	t	100	*	TYPE=SUBSTITUTE
CM032587.1	2042420	.	A	c	100	*	TYPE=SUBSTITUTE
CM032587.1	2042471	.	A	t	100	*	TYPE=SUBSTITUTE
CM032587.1	2042490	.	GG	G	100	*	TYPE=DELETE
CM032587.1	2042499	.	C	G	100	*	TYPE=SUBSTITUTE
CM032587.1	2042504	.	G	A	100	*	TYPE=SUBSTITUTE
CM032587.1	2042511	.	T	C	100	*	TYPE=SUBSTITUTE
CM032587.1	2042515	.	CT	C	100	*	TYPE=DELETE
CM032587.1	2042521	.	T	C	100	*	TYPE=SUBSTITUTE
CM032587.1	2042524	.	T	C	100	*	TYPE=SUBSTITUTE
CM032587.1	2042538	.	T	C	100	*	TYPE=SUBSTITUTE
CM032587.1	2042598	.	C	T	100	*	TYPE=SUBSTITUTE
CM032587.1	2042611	.	C	T	100	*	TYPE=SUBSTITUTE
CM032587.1	2042638	.	T	c	100	*	TYPE=SUBSTITUTE
CM032587.1	2042639	.	T	a	100	*	TYPE=SUBSTITUTE
CM032587.1	2042640	.	A	t	100	*	TYPE=SUBSTITUTE
CM032587.1	2042681	.	TATAC	T	100	*	TYPE=DELETE
CM032587.1	2042686	.	C	G	100	*	TYPE=SUBSTITUTE
CM032587.1	2042687	.	A	G	100	*	TYPE=SUBSTITUTE
CM032587.1	2042689	.	C	T	100	*	TYPE=SUBSTITUTE
CM032587.1	2042691	.	G	C	100	*	TYPE=SUBSTITUTE
CM032587.1	2042754	.	A	AAACTACT	100	*	TYPE=INSERT
CM032587.1	2042757	.	A	AGC	100	*	TYPE=INSERT
CM032587.1	2042759	.	A	G	100	*	TYPE=SUBSTITUTE
CM032587.1	2042765	.	C	G	100	*	TYPE=SUBSTITUTE
CM032587.1	2042780	.	C	A	100	*	TYPE=SUBSTITUTE
CM032587.1	2042782	.	A	G	100	*	TYPE=SUBSTITUTE
CM032587.1	2042832	.	T	G	100	*	TYPE=SUBSTITUTE
CM032587.1	2042836	.	A	C	100	*	TYPE=SUBSTITUTE
CM032587.1	2042862	.	C	T	100	*	TYPE=SUBSTITUTE
CM032587.1	2042863	.	T	TC	100	*	TYPE=INSERT
CM032587.1	2042875	.	A	G	100	*	TYPE=SUBSTITUTE
CM032587.1	2042888	.	T	c	100	*	TYPE=SUBSTITUTE
CM032587.1	2042899	.	CT	C	100	*	TYPE=DELETE
CM032587.1	2042960	.	T	C	100	*	TYPE=SUBSTITUTE
CM032587.1	2043010	.	TCAATG	T	100	*	TYPE=DELETE
CM032587.1	2043023	.	A	T	100	*	TYPE=SUBSTITUTE
CM032587.1	2043030	.	A	AGCATG	100	*	TYPE=INSERT
CM032587.1	2043053	.	A	t	100	*	TYPE=SUBSTITUTE
CM032587.1	2043084	.	CC	C	100	*	TYPE=DELETE
CM032587.1	2043092	.	G	A	100	*	TYPE=SUBSTITUTE

CM032587.1	2043110	.	G	T	100	*	TYPE=SUBSTITUTE
CM032587.1	2043159	.	CC	C	100	*	TYPE=DELETE
CM032587.1	2043171	.	T	C	100	*	TYPE=SUBSTITUTE
CM032587.1	2043194	.	A	t	100	*	TYPE=SUBSTITUTE
CM032587.1	2043195	.	c	cagt	100	*	TYPE=INSERT
CM032587.1	2043201	.	G	a	100	*	TYPE=SUBSTITUTE
CM032587.1	2043209	.	C	t	100	*	TYPE=SUBSTITUTE
CM032587.1	2043214	.	T	G	100	*	TYPE=SUBSTITUTE
CM032587.1	2043215	.	A	G	100	*	TYPE=SUBSTITUTE
CM032587.1	2043227	.	A	T	100	*	TYPE=SUBSTITUTE
CM032587.1	2043233	.	T	A	100	*	TYPE=SUBSTITUTE
CM032587.1	2043235	.	A	G	100	*	TYPE=SUBSTITUTE
CM032587.1	2043245	.	T	G	100	*	TYPE=SUBSTITUTE
CM032587.1	2043277	.	C	A	100	*	TYPE=SUBSTITUTE
CM032587.1	2043278	.	T	C	100	*	TYPE=SUBSTITUTE
CM032587.1	2043294	.	C	T	100	*	TYPE=SUBSTITUTE
CM032587.1	2043318	.	C	A	100	*	TYPE=SUBSTITUTE
CM032587.1	2043321	.	C	A	100	*	TYPE=SUBSTITUTE
CM032587.1	2043361	.	C	A	100	*	TYPE=SUBSTITUTE
CM032587.1	2043364	.	T	A	100	*	TYPE=SUBSTITUTE
CM032587.1	2043437	.	C	T	100	*	TYPE=SUBSTITUTE
CM032587.1	2043446	.	G	a	100	*	TYPE=SUBSTITUTE
CM032587.1	2043459	.	G	t	100	*	TYPE=SUBSTITUTE
CM032587.1	2043532	.	G	T	100	*	TYPE=SUBSTITUTE
CM032587.1	2043537	.	T	A	100	*	TYPE=SUBSTITUTE
CM032587.1	2043538	.	T	G	100	*	TYPE=SUBSTITUTE
CM032587.1	2043560	.	G	A	100	*	TYPE=SUBSTITUTE
CM032587.1	2043568	.	G	C	100	*	TYPE=SUBSTITUTE
CM032587.1	2043597	.	G	A	100	*	TYPE=SUBSTITUTE
CM032587.1	2043619	.	G	T	100	*	TYPE=SUBSTITUTE
CM032587.1	2043661	.	G	A	100	*	TYPE=SUBSTITUTE
CM032587.1	2043662	.	T	G	100	*	TYPE=SUBSTITUTE
CM032587.1	2043664	.	G	A	100	*	TYPE=SUBSTITUTE
CM032587.1	2043665	.	A	G	100	*	TYPE=SUBSTITUTE
CM032587.1	2043677	.	T	G	100	*	TYPE=SUBSTITUTE
CM032587.1	2043679	.	C	T	100	*	TYPE=SUBSTITUTE
CM032587.1	2043693	.	C	T	100	*	TYPE=SUBSTITUTE
CM032587.1	2043703	.	G	A	100	*	TYPE=SUBSTITUTE
CM032587.1	2043716	.	G	T	100	*	TYPE=SUBSTITUTE
CM032587.1	2043733	.	T	G	100	*	TYPE=SUBSTITUTE
CM032587.1	2043768	.	c	catcc	100	*	TYPE=INSERT
CM032587.1	2043769	.	T	a	100	*	TYPE=SUBSTITUTE
CM032587.1	2043789	.	A	G	100	*	TYPE=SUBSTITUTE
CM032587.1	2043801	.	G	A	100	*	TYPE=SUBSTITUTE
CM032587.1	2043803	.	A	G	100	*	TYPE=SUBSTITUTE
CM032587.1	2043885	.	AA	A	100	*	TYPE=DELETE
CM032587.1	2043928	.	C	a	100	*	TYPE=SUBSTITUTE
CM032587.1	2043999	.	C	G	100	*	TYPE=SUBSTITUTE
CM032587.1	2044000	.	A	G	100	*	TYPE=SUBSTITUTE
CM032587.1	2044002	.	A	G	100	*	TYPE=SUBSTITUTE
CM032587.1	2044007	.	C	T	100	*	TYPE=SUBSTITUTE
CM032587.1	2044023	.	T	C	100	*	TYPE=SUBSTITUTE
CM032587.1	2044032	.	T	C	100	*	TYPE=SUBSTITUTE
CM032587.1	2044039	.	T	C	100	*	TYPE=SUBSTITUTE
CM032587.1	2044041	.	G	A	100	*	TYPE=SUBSTITUTE
CM032587.1	2044055	.	G	a	100	*	TYPE=SUBSTITUTE
CM032587.1	2044069	.	A	g	100	*	TYPE=SUBSTITUTE
CM032587.1	2044116	.	CCTTC	C	100	*	TYPE=DELETE
CM032587.1	2044262	.	T	C	100	*	TYPE=SUBSTITUTE
CM032587.1	2044332	.	T	C	100	*	TYPE=SUBSTITUTE
CM032587.1	2044353	.	G	A	100	*	TYPE=SUBSTITUTE
CM032587.1	2044425	.	A	G	100	*	TYPE=SUBSTITUTE
CM032587.1	2044886	.	A	C	100	*	TYPE=SUBSTITUTE
CM032587.1	2044893	.	G	C	100	*	TYPE=SUBSTITUTE

CM032587.1	2044943	.	C	G	100	*	TYPE=SUBSTITUTE
CM032587.1	2044971	.	G	A	100	*	TYPE=SUBSTITUTE
CM032587.1	2044980	.	T	C	100	*	TYPE=SUBSTITUTE
CM032587.1	2045068	.	A	G	100	*	TYPE=SUBSTITUTE
CM032587.1	2045106	.	A	G	100	*	TYPE=SUBSTITUTE
CM032587.1	2045107	.	T	G	100	*	TYPE=SUBSTITUTE
CM032587.1	2045108	.	G	A	100	*	TYPE=SUBSTITUTE
CM032587.1	2045538	.	C	G	100	*	TYPE=SUBSTITUTE
CM032587.1	2045583	.	GCCAACGCTG	G	100	*	TYPE=DELETE
CM032587.1	2045595	.	G	C	100	*	TYPE=SUBSTITUTE
CM032587.1	2045613	.	C	a	100	*	TYPE=SUBSTITUTE
CM032587.1	2045616	.	A	g	100	*	TYPE=SUBSTITUTE
CM032587.1	2045663	.	T	G	100	*	TYPE=SUBSTITUTE
CM032587.1	2045690	.	TTT	T	100	*	TYPE=DELETE
CM032587.1	2045755	.	G	T	100	*	TYPE=SUBSTITUTE
CM032587.1	2045788	.	A	G	100	*	TYPE=SUBSTITUTE
CM032587.1	2045826	.	A	G	100	*	TYPE=SUBSTITUTE
CM032587.1	2045862	.	T	C	100	*	TYPE=SUBSTITUTE
CM032587.1	2045894	.	A	g	100	*	TYPE=SUBSTITUTE
CM032587.1	2045916	.	G	A	100	*	TYPE=SUBSTITUTE
CM032587.1	2045975	.	A	g	100	*	TYPE=SUBSTITUTE
CM032587.1	2045992	.	A	g	100	*	TYPE=SUBSTITUTE
CM032587.1	2045994	.	A	t	100	*	TYPE=SUBSTITUTE
CM032587.1	2046003	.	g	gga	100	*	TYPE=INSERT
CM032587.1	2046007	.	T	a	100	*	TYPE=SUBSTITUTE
CM032587.1	2046088	.	C	t	100	*	TYPE=SUBSTITUTE
CM032587.1	2046222	.	AT	A	100	*	TYPE=DELETE
CM032587.1	2046224	.	A	G	100	*	TYPE=SUBSTITUTE
CM032587.1	2046226	.	G	A	100	*	TYPE=SUBSTITUTE
CM032587.1	2046246	.	A	aga	100	*	TYPE=INSERT
CM032587.1	2046281	.	G	GATT	100	*	TYPE=INSERT
CM032587.1	2046283	.	T	G	100	*	TYPE=SUBSTITUTE
CM032587.1	2046326	.	A	G	100	*	TYPE=SUBSTITUTE
CM032587.1	2046334	.	TACCTACTCGC	T	100	*	TYPE=DELETE
CM032587.1	2046363	.	T	a	100	*	TYPE=SUBSTITUTE
CM032587.1	2046374	.	G	A	100	*	TYPE=SUBSTITUTE
CM032587.1	2046389	.	C	A	100	*	TYPE=SUBSTITUTE
CM032587.1	2046390	.	A	C	100	*	TYPE=SUBSTITUTE
CM032587.1	2046400	.	TGTCTGTCC	T	100	*	TYPE=DELETE
CM032587.1	2046423	.	A	G	100	*	TYPE=SUBSTITUTE
CM032587.1	2046432	.	TG	T	100	*	TYPE=DELETE
CM032587.1	2046502	.	G	GG	100	*	TYPE=INSERT
CM032587.1	2046574	.	C	A	100	*	TYPE=SUBSTITUTE
CM032587.1	2046584	.	G	T	100	*	TYPE=SUBSTITUTE
CM032587.1	2046588	.	T	C	100	*	TYPE=SUBSTITUTE
CM032587.1	2046604	.	TGC	T	100	*	TYPE=DELETE
CM032587.1	2046614	.	T	C	100	*	TYPE=SUBSTITUTE
CM032587.1	2046697	.	GTTG	G	100	*	TYPE=DELETE
CM032587.1	2046746	.	T	C	100	*	TYPE=SUBSTITUTE
CM032587.1	2046751	.	A	G	100	*	TYPE=SUBSTITUTE
CM032587.1	2046753	.	A	G	100	*	TYPE=SUBSTITUTE
CM032587.1	2046778	.	A	G	100	*	TYPE=SUBSTITUTE
CM032587.1	2046779	.	T	C	100	*	TYPE=SUBSTITUTE
CM032587.1	2046789	.	G	A	100	*	TYPE=SUBSTITUTE
CM032587.1	2046806	.	A	G	100	*	TYPE=SUBSTITUTE
CM032587.1	2046807	.	A	G	100	*	TYPE=SUBSTITUTE
CM032587.1	2046829	.	TT	T	100	*	TYPE=DELETE
CM032587.1	2046852	.	C	G	100	*	TYPE=SUBSTITUTE
CM032587.1	2046935	.	A	G	100	*	TYPE=SUBSTITUTE
CM032587.1	2046949	.	A	G	100	*	TYPE=SUBSTITUTE
CM032587.1	2047032	.	A	g	100	*	TYPE=SUBSTITUTE
CM032587.1	2047080	.	C	T	100	*	TYPE=SUBSTITUTE
CM032587.1	2047159	.	C	G	100	*	TYPE=SUBSTITUTE
CM032587.1	2047205	.	C	A	100	*	TYPE=SUBSTITUTE

CM032587.1	2047206	.	G	C	100	*	TYPE=SUBSTITUTE
CM032587.1	2047439	.	AA	A	100	*	TYPE=DELETE
CM032587.1	2047514	.	C	t	100	*	TYPE=SUBSTITUTE
CM032587.1	2047527	.	AGAGG	A	100	*	TYPE=DELETE
CM032587.1	2047578	.	C	T	100	*	TYPE=SUBSTITUTE
CM032587.1	2047585	.	C	A	100	*	TYPE=SUBSTITUTE
CM032587.1	2047634	.	GGACTCG	G	100	*	TYPE=DELETE
CM032587.1	2047648	.	C	G	100	*	TYPE=SUBSTITUTE
CM032587.1	2047699	.	T	C	100	*	TYPE=SUBSTITUTE
CM032587.1	2047701	.	CT	C	100	*	TYPE=DELETE
CM032587.1	2047766	.	G	A	100	*	TYPE=SUBSTITUTE
CM032587.1	2047801	.	G	A	100	*	TYPE=SUBSTITUTE
CM032587.1	2047817	.	A	G	100	*	TYPE=SUBSTITUTE
CM032587.1	2047824	.	A	G	100	*	TYPE=SUBSTITUTE
CM032587.1	2047825	.	C	T	100	*	TYPE=SUBSTITUTE
CM032587.1	2047831	.	C	A	100	*	TYPE=SUBSTITUTE
CM032587.1	2047877	.	C	T	100	*	TYPE=SUBSTITUTE
CM032587.1	2047925	.	T	TGAGTGAGTCCTCGTAT	100	*	TYPE=INSERT
CM032587.1	2047952	.	A	G	100	*	TYPE=SUBSTITUTE
CM032587.1	2047970	.	A	T	100	*	TYPE=SUBSTITUTE
CM032587.1	2048014	.	A	G	100	*	TYPE=SUBSTITUTE
CM032587.1	2048017	.	C	T	100	*	TYPE=SUBSTITUTE
CM032587.1	2048117	.	G	T	100	*	TYPE=SUBSTITUTE
CM032587.1	2048133	.	A	aa	100	*	TYPE=INSERT
CM032587.1	2048154	.	CTC	C	100	*	TYPE=DELETE
CM032587.1	2048169	.	C	CG	100	*	TYPE=INSERT
CM032587.1	2048170	.	T	A	100	*	TYPE=SUBSTITUTE
CM032587.1	2048172	.	TGC	T	100	*	TYPE=DELETE
CM032587.1	2048186	.	G	A	100	*	TYPE=SUBSTITUTE
CM032587.1	2048189	.	C	A	100	*	TYPE=SUBSTITUTE
CM032587.1	2048191	.	T	C	100	*	TYPE=SUBSTITUTE
CM032587.1	2048193	.	A	T	100	*	TYPE=SUBSTITUTE
CM032587.1	2048194	.	A	C	100	*	TYPE=SUBSTITUTE
CM032587.1	2048195	.	C	T	100	*	TYPE=SUBSTITUTE
CM032587.1	2048196	.	C	A	100	*	TYPE=SUBSTITUTE
CM032587.1	2048197	.	T	G	100	*	TYPE=SUBSTITUTE
CM032587.1	2048216	.	G	C	100	*	TYPE=SUBSTITUTE
CM032587.1	2048224	.	C	T	100	*	TYPE=SUBSTITUTE
CM032587.1	2048226	.	G	C	100	*	TYPE=SUBSTITUTE
CM032587.1	2048228	.	A	G	100	*	TYPE=SUBSTITUTE
CM032587.1	2048376	.	A	G	100	*	TYPE=SUBSTITUTE
CM032587.1	2048377	.	T	C	100	*	TYPE=SUBSTITUTE
CM032587.1	2048431	.	GG	G	100	*	TYPE=DELETE
CM032587.1	2048481	.	T	C	100	*	TYPE=SUBSTITUTE
CM032587.1	2048589	.	A	G	100	*	TYPE=SUBSTITUTE
CM032587.1	2048623	.	T	c	100	*	TYPE=SUBSTITUTE
CM032587.1	2048624	.	G	a	100	*	TYPE=SUBSTITUTE
CM032587.1	2048632	.	G	a	100	*	TYPE=SUBSTITUTE
CM032587.1	2048639	.	c	ctcaaca	100	*	TYPE=INSERT
CM032587.1	2048644	.	t	tgc	100	*	TYPE=INSERT
CM032587.1	2048888	.	C	A	100	*	TYPE=SUBSTITUTE
CM032587.1	2048889	.	T	G	100	*	TYPE=SUBSTITUTE
CM032587.1	2048892	.	G	A	100	*	TYPE=SUBSTITUTE
CM032587.1	2048893	.	C	CT	100	*	TYPE=INSERT
CM032587.1	2048899	.	G	A	100	*	TYPE=SUBSTITUTE
CM032587.1	2048902	.	T	A	100	*	TYPE=SUBSTITUTE
CM032587.1	2048903	.	C	A	100	*	TYPE=SUBSTITUTE
CM032587.1	2048904	.	G	T	100	*	TYPE=SUBSTITUTE
CM032587.1	2048905	.	T	G	100	*	TYPE=SUBSTITUTE
CM032587.1	2048906	.	A	G	100	*	TYPE=SUBSTITUTE
CM032587.1	2048911	.	A	G	100	*	TYPE=SUBSTITUTE
CM032587.1	2048916	.	T	C	100	*	TYPE=SUBSTITUTE
CM032587.1	2048918	.	C	T	100	*	TYPE=SUBSTITUTE
CM032587.1	2048923	.	T	A	100	*	TYPE=SUBSTITUTE

CM032587.1	2048924	.	GA	G	100	*	TYPE=DELETE
CM032587.1	2048926	.	G	T	100	*	TYPE=SUBSTITUTE
CM032587.1	2048930	.	A	G	100	*	TYPE=SUBSTITUTE
CM032587.1	2048936	.	A	C	100	*	TYPE=SUBSTITUTE
CM032587.1	2048938	.	T	A	100	*	TYPE=SUBSTITUTE
CM032587.1	2048940	.	C	A	100	*	TYPE=SUBSTITUTE
CM032587.1	2048941	.	T	C	100	*	TYPE=SUBSTITUTE
CM032587.1	2048946	.	C	T	100	*	TYPE=SUBSTITUTE
CM032587.1	2048948	.	G	C	100	*	TYPE=SUBSTITUTE
CM032587.1	2048951	.	T	C	100	*	TYPE=SUBSTITUTE
CM032587.1	2049087	.	T	C	100	*	TYPE=SUBSTITUTE
CM032587.1	2049145	.	G	A	100	*	TYPE=SUBSTITUTE
CM032587.1	2049152	.	C	T	100	*	TYPE=SUBSTITUTE
CM032587.1	2049215	.	C	t	100	*	TYPE=SUBSTITUTE
CM032587.1	2049220	.	G	A	100	*	TYPE=SUBSTITUTE
CM032587.1	2049303	.	T	c	100	*	TYPE=SUBSTITUTE
CM032587.1	2049307	.	t	ttcc	100	*	TYPE=INSERT
CM032587.1	2049336	.	A	G	100	*	TYPE=SUBSTITUTE
CM032587.1	2049463	.	A	T	100	*	TYPE=SUBSTITUTE
CM032587.1	2049473	.	C	G	100	*	TYPE=SUBSTITUTE
CM032587.1	2049505	.	C	T	100	*	TYPE=SUBSTITUTE
CM032587.1	2049508	.	A	G	100	*	TYPE=SUBSTITUTE
CM032587.1	2049558	.	A	C	100	*	TYPE=SUBSTITUTE
CM032587.1	2049563	.	C	A	100	*	TYPE=SUBSTITUTE
CM032587.1	2049574	.	C	T	100	*	TYPE=SUBSTITUTE
CM032587.1	2049581	.	T	A	100	*	TYPE=SUBSTITUTE
CM032587.1	2049588	.	C	T	100	*	TYPE=SUBSTITUTE
CM032587.1	2049592	.	T	G	100	*	TYPE=SUBSTITUTE
CM032587.1	2049606	.	T	G	100	*	TYPE=SUBSTITUTE
CM032587.1	2049618	.	G	a	100	*	TYPE=SUBSTITUTE
CM032587.1	2049624	.	t	tcttcttctg	100	*	TYPE=INSERT
CM032587.1	2049633	.	T	g	100	*	TYPE=SUBSTITUTE
CM032587.1	2049651	.	GC	G	100	*	TYPE=DELETE
CM032587.1	2049707	.	T	A	100	*	TYPE=SUBSTITUTE
CM032587.1	2049748	.	G	A	100	*	TYPE=SUBSTITUTE
CM032587.1	2049762	.	TGCC	T	100	*	TYPE=DELETE
CM032587.1	2049773	.	c	cCATCGT	100	*	TYPE=INSERT
CM032587.1	2049774	.	A	C	100	*	TYPE=SUBSTITUTE
CM032587.1	2049777	.	A	G	100	*	TYPE=SUBSTITUTE
CM032587.1	2049784	.	G	C	100	*	TYPE=SUBSTITUTE
CM032587.1	2049798	.	C	A	100	*	TYPE=SUBSTITUTE
CM032587.1	2049836	.	G	A	100	*	TYPE=SUBSTITUTE
CM032587.1	2049874	.	T	C	100	*	TYPE=SUBSTITUTE
CM032587.1	2049889	.	C	T	100	*	TYPE=SUBSTITUTE
CM032587.1	2049901	.	CG	C	100	*	TYPE=DELETE
CM032587.1	2049933	.	C	T	100	*	TYPE=SUBSTITUTE
CM032587.1	2049946	.	G	GC	100	*	TYPE=INSERT
CM032587.1	2049948	.	C	G	100	*	TYPE=SUBSTITUTE
CM032587.1	2049950	.	C	T	100	*	TYPE=SUBSTITUTE
CM032587.1	2049962	.	CTCA	C	100	*	TYPE=DELETE
CM032587.1	2049966	.	A	G	100	*	TYPE=SUBSTITUTE
CM032587.1	2049970	.	C	A	100	*	TYPE=SUBSTITUTE
CM032587.1	2050087	.	T	C	100	*	TYPE=SUBSTITUTE
CM032587.1	2050095	.	CG	C	100	*	TYPE=DELETE
CM032587.1	2050103	.	T	C	100	*	TYPE=SUBSTITUTE
CM032587.1	2050207	.	A	G	100	*	TYPE=SUBSTITUTE
CM032587.1	2050241	.	C	t	100	*	TYPE=SUBSTITUTE
CM032587.1	2050242	.	A	g	100	*	TYPE=SUBSTITUTE
CM032587.1	2050286	.	G	A	100	*	TYPE=SUBSTITUTE
CM032587.1	2050307	.	CC	C	100	*	TYPE=DELETE
CM032587.1	2050312	.	C	a	100	*	TYPE=SUBSTITUTE
CM032587.1	2050313	.	T	a	100	*	TYPE=SUBSTITUTE
CM032587.1	2050314	.	T	a	100	*	TYPE=SUBSTITUTE
CM032587.1	2050315	.	T	a	100	*	TYPE=SUBSTITUTE

CM032587.1	2050316	.	T	a	100	*	TYPE=SUBSTITUTE
CM032587.1	2050317	.	T	a	100	*	TYPE=SUBSTITUTE
CM032587.1	2050318	.	T	a	100	*	TYPE=SUBSTITUTE
CM032587.1	2050319	.	T	a	100	*	TYPE=SUBSTITUTE
CM032587.1	2050344	.	t	tcac	100	*	TYPE=INSERT
CM032587.1	2050345	.	A	t	100	*	TYPE=SUBSTITUTE
CM032587.1	2050347	.	c	ct	100	*	TYPE=INSERT
CM032587.1	2050365	.	G	A	100	*	TYPE=SUBSTITUTE
CM032587.1	2050368	.	A	G	100	*	TYPE=SUBSTITUTE
CM032587.1	2050377	.	T	G	100	*	TYPE=SUBSTITUTE
CM032587.1	2050379	.	T	C	100	*	TYPE=SUBSTITUTE
CM032587.1	2050420	.	TT	T	100	*	TYPE=DELETE
CM032587.1	2050443	.	C	a	100	*	TYPE=SUBSTITUTE
CM032587.1	2050449	.	A	c	100	*	TYPE=SUBSTITUTE
CM032587.1	2050450	.	T	c	100	*	TYPE=SUBSTITUTE
CM032587.1	2050451	.	A	g	100	*	TYPE=SUBSTITUTE
CM032587.1	2050471	.	T	C	100	*	TYPE=SUBSTITUTE
CM032587.1	2050525	.	C	T	100	*	TYPE=SUBSTITUTE
CM032587.1	2050570	.	A	T	100	*	TYPE=SUBSTITUTE
CM032587.1	2050648	.	G	A	100	*	TYPE=SUBSTITUTE
CM032587.1	2050655	.	G	A	100	*	TYPE=SUBSTITUTE
CM032587.1	2050659	.	G	A	100	*	TYPE=SUBSTITUTE
CM032587.1	2050717	.	A	G	100	*	TYPE=SUBSTITUTE
CM032587.1	2050721	.	T	C	100	*	TYPE=SUBSTITUTE
CM032587.1	2050762	.	AA	A	100	*	TYPE=DELETE
CM032587.1	2050764	.	A	G	100	*	TYPE=SUBSTITUTE
CM032587.1	2050838	.	C	T	100	*	TYPE=SUBSTITUTE
CM032587.1	2050852	.	C	T	100	*	TYPE=SUBSTITUTE
CM032587.1	2050865	.	A	G	100	*	TYPE=SUBSTITUTE
CM032587.1	2050902	.	G	A	100	*	TYPE=SUBSTITUTE
CM032587.1	2050915	.	T	C	100	*	TYPE=SUBSTITUTE
CM032587.1	2050916	.	T	C	100	*	TYPE=SUBSTITUTE
CM032587.1	2050945	.	C	T	100	*	TYPE=SUBSTITUTE
CM032587.1	2050946	.	C	T	100	*	TYPE=SUBSTITUTE
CM032587.1	2050979	.	G	A	100	*	TYPE=SUBSTITUTE
CM032587.1	2051037	.	T	G	100	*	TYPE=SUBSTITUTE
CM032587.1	2051038	.	T	C	100	*	TYPE=SUBSTITUTE
CM032587.1	2051062	.	T	tt	100	*	TYPE=INSERT
CM032587.1	2051167	.	T	C	100	*	TYPE=SUBSTITUTE
CM032587.1	2051270	.	T	g	100	*	TYPE=SUBSTITUTE
CM032587.1	2051271	.	T	c	100	*	TYPE=SUBSTITUTE
CM032587.1	2051278	.	TC	T	100	*	TYPE=DELETE
CM032587.1	2051340	.	C	T	100	*	TYPE=SUBSTITUTE
CM032587.1	2051366	.	A	G	100	*	TYPE=SUBSTITUTE
CM032587.1	2051368	.	C	A	100	*	TYPE=SUBSTITUTE
CM032587.1	2051388	.	A	G	100	*	TYPE=SUBSTITUTE
CM032587.1	2051429	.	T	C	100	*	TYPE=SUBSTITUTE
CM032587.1	2051430	.	C	T	100	*	TYPE=SUBSTITUTE
CM032587.1	2051445	.	C	T	100	*	TYPE=SUBSTITUTE
CM032587.1	2051458	.	A	G	100	*	TYPE=SUBSTITUTE
CM032587.1	2051466	.	G	A	100	*	TYPE=SUBSTITUTE
CM032587.1	2051469	.	GA	G	100	*	TYPE=DELETE
CM032587.1	2051475	.	G	C	100	*	TYPE=SUBSTITUTE
CM032587.1	2051552	.	T	C	100	*	TYPE=SUBSTITUTE
CM032587.1	2051743	.	A	G	100	*	TYPE=SUBSTITUTE
CM032587.1	2051767	.	GT	G	100	*	TYPE=DELETE
CM032587.1	2051797	.	T	G	100	*	TYPE=SUBSTITUTE
CM032587.1	2051804	.	A	G	100	*	TYPE=SUBSTITUTE
CM032587.1	2051848	.	CT	C	100	*	TYPE=DELETE
CM032587.1	2051855	.	C	A	100	*	TYPE=SUBSTITUTE
CM032587.1	2051870	.	C	t	100	*	TYPE=SUBSTITUTE
CM032587.1	2051913	.	GG	G	100	*	TYPE=DELETE
CM032587.1	2051930	.	A	G	100	*	TYPE=SUBSTITUTE
CM032587.1	2051940	.	A	T	100	*	TYPE=SUBSTITUTE

CM032587.1	2051956	.	C	G	100	*	TYPE=SUBSTITUTE
CM032587.1	2051958	.	G	T	100	*	TYPE=SUBSTITUTE
CM032587.1	2051987	.	CCC	C	100	*	TYPE=DELETE
CM032587.1	2052072	.	G	A	100	*	TYPE=SUBSTITUTE
CM032587.1	2052217	.	C	T	100	*	TYPE=SUBSTITUTE
CM032587.1	2052284	.	C	G	100	*	TYPE=SUBSTITUTE
CM032587.1	2052421	.	C	T	100	*	TYPE=SUBSTITUTE
CM032587.1	2052531	.	T	TT	100	*	TYPE=INSERT
CM032587.1	2052568	.	C	T	100	*	TYPE=SUBSTITUTE
CM032587.1	2052571	.	A	C	100	*	TYPE=SUBSTITUTE
CM032587.1	2052572	.	G	C	100	*	TYPE=SUBSTITUTE
CM032587.1	2052582	.	T	G	100	*	TYPE=SUBSTITUTE
CM032587.1	2052586	.	G	A	100	*	TYPE=SUBSTITUTE
CM032587.1	2052587	.	C	T	100	*	TYPE=SUBSTITUTE
CM032587.1	2052588	.	G	T	100	*	TYPE=SUBSTITUTE
CM032587.1	2052594	.	A	G	100	*	TYPE=SUBSTITUTE
CM032587.1	2052595	.	A	G	100	*	TYPE=SUBSTITUTE
CM032587.1	2052602	.	T	A	100	*	TYPE=SUBSTITUTE
CM032587.1	2052605	.	C	T	100	*	TYPE=SUBSTITUTE
CM032587.1	2052609	.	T	G	100	*	TYPE=SUBSTITUTE
CM032587.1	2052613	.	G	A	100	*	TYPE=SUBSTITUTE
CM032587.1	2052627	.	A	C	100	*	TYPE=SUBSTITUTE
CM032587.1	2052645	.	C	T	100	*	TYPE=SUBSTITUTE
CM032587.1	2052647	.	T	A	100	*	TYPE=SUBSTITUTE
CM032587.1	2052654	.	G	A	100	*	TYPE=SUBSTITUTE
CM032587.1	2052687	.	A	G	100	*	TYPE=SUBSTITUTE
CM032587.1	2052700	.	A	G	100	*	TYPE=SUBSTITUTE
CM032587.1	2052710	.	T	G	100	*	TYPE=SUBSTITUTE
CM032587.1	2052814	.	A	G	100	*	TYPE=SUBSTITUTE
CM032587.1	2052850	.	CT	C	100	*	TYPE=DELETE
CM032587.1	2052863	.	A	C	100	*	TYPE=SUBSTITUTE
CM032587.1	2052886	.	T	C	100	*	TYPE=SUBSTITUTE
CM032587.1	2052887	.	C	T	100	*	TYPE=SUBSTITUTE
CM032587.1	2052896	.	AC	A	100	*	TYPE=DELETE
CM032587.1	2052913	.	G	GAC	100	*	TYPE=INSERT
CM032587.1	2052931	.	GCAAT	G	100	*	TYPE=DELETE
CM032587.1	2052936	.	C	T	100	*	TYPE=SUBSTITUTE
CM032587.1	2052939	.	T	C	100	*	TYPE=SUBSTITUTE
CM032587.1	2052951	.	T	C	100	*	TYPE=SUBSTITUTE
CM032587.1	2052957	.	A	G	100	*	TYPE=SUBSTITUTE
CM032587.1	2052987	.	T	C	100	*	TYPE=SUBSTITUTE
CM032587.1	2053011	.	A	G	100	*	TYPE=SUBSTITUTE
CM032587.1	2053017	.	C	T	100	*	TYPE=SUBSTITUTE
CM032587.1	2053022	.	C	T	100	*	TYPE=SUBSTITUTE
CM032587.1	2053023	.	A	G	100	*	TYPE=SUBSTITUTE
CM032587.1	2053027	.	G	T	100	*	TYPE=SUBSTITUTE
CM032587.1	2053044	.	G	C	100	*	TYPE=SUBSTITUTE
CM032587.1	2053060	.	T	C	100	*	TYPE=SUBSTITUTE
CM032587.1	2053074	.	A	G	100	*	TYPE=SUBSTITUTE
CM032587.1	2053078	.	C	G	100	*	TYPE=SUBSTITUTE
CM032587.1	2053079	.	C	T	100	*	TYPE=SUBSTITUTE
CM032587.1	2053080	.	T	C	100	*	TYPE=SUBSTITUTE
CM032587.1	2053085	.	A	G	100	*	TYPE=SUBSTITUTE
CM032587.1	2053119	.	A	G	100	*	TYPE=SUBSTITUTE
CM032587.1	2053123	.	C	T	100	*	TYPE=SUBSTITUTE
CM032587.1	2053125	.	C	T	100	*	TYPE=SUBSTITUTE
CM032587.1	2053129	.	A	C	100	*	TYPE=SUBSTITUTE
CM032587.1	2053134	.	T	G	100	*	TYPE=SUBSTITUTE
CM032587.1	2053135	.	C	T	100	*	TYPE=SUBSTITUTE
CM032587.1	2053148	.	C	T	100	*	TYPE=SUBSTITUTE
CM032587.1	2053163	.	C	T	100	*	TYPE=SUBSTITUTE
CM032587.1	2053167	.	T	C	100	*	TYPE=SUBSTITUTE
CM032587.1	2053170	.	C	T	100	*	TYPE=SUBSTITUTE
CM032587.1	2053172	.	A	G	100	*	TYPE=SUBSTITUTE

CM032587.1	2053175	.	GAT	G	100	*	TYPE=DELETE
CM032587.1	2053179	.	G	T	100	*	TYPE=SUBSTITUTE
CM032587.1	2053180	.	T	C	100	*	TYPE=SUBSTITUTE
CM032587.1	2053182	.	CT	C	100	*	TYPE=DELETE
CM032587.1	2053195	.	A	G	100	*	TYPE=SUBSTITUTE
CM032587.1	2053197	.	G	GAGCCC	100	*	TYPE=INSERT
CM032587.1	2053198	.	C	T	100	*	TYPE=SUBSTITUTE
CM032587.1	2053199	.	A	T	100	*	TYPE=SUBSTITUTE
CM032587.1	2053200	.	A	G	100	*	TYPE=SUBSTITUTE
CM032587.1	2053204	.	C	T	100	*	TYPE=SUBSTITUTE
CM032587.1	2053207	.	G	A	100	*	TYPE=SUBSTITUTE
CM032587.1	2053211	.	T	C	100	*	TYPE=SUBSTITUTE
CM032587.1	2053228	.	A	T	100	*	TYPE=SUBSTITUTE
CM032587.1	2053237	.	TAG	T	100	*	TYPE=DELETE
CM032587.1	2053240	.	C	T	100	*	TYPE=SUBSTITUTE
CM032587.1	2053241	.	A	T	100	*	TYPE=SUBSTITUTE
CM032587.1	2053242	.	G	A	100	*	TYPE=SUBSTITUTE
CM032587.1	2053252	.	G	GTAACGGGCCAC	100	*	TYPE=INSERT
CM032587.1	2053257	.	A	G	100	*	TYPE=SUBSTITUTE
CM032587.1	2053259	.	T	C	100	*	TYPE=SUBSTITUTE
CM032587.1	2053262	.	T	C	100	*	TYPE=SUBSTITUTE
CM032587.1	2053270	.	G	A	100	*	TYPE=SUBSTITUTE
CM032587.1	2053271	.	T	C	100	*	TYPE=SUBSTITUTE
CM032587.1	2053275	.	G	A	100	*	TYPE=SUBSTITUTE
CM032587.1	2053276	.	TACTA	T	100	*	TYPE=DELETE
CM032587.1	2053281	.	A	C	100	*	TYPE=SUBSTITUTE
CM032587.1	2053301	.	C	T	100	*	TYPE=SUBSTITUTE
CM032587.1	2053302	.	C	CAATGG	100	*	TYPE=INSERT
CM032587.1	2053303	.	G	C	100	*	TYPE=SUBSTITUTE
CM032587.1	2053306	.	G	GA	100	*	TYPE=INSERT
CM032587.1	2053308	.	C	T	100	*	TYPE=SUBSTITUTE
CM032587.1	2053310	.	C	A	100	*	TYPE=SUBSTITUTE
CM032587.1	2053312	.	A	T	100	*	TYPE=SUBSTITUTE
CM032587.1	2053322	.	T	G	100	*	TYPE=SUBSTITUTE
CM032587.1	2053329	.	C	T	100	*	TYPE=SUBSTITUTE
CM032587.1	2053330	.	C	T	100	*	TYPE=SUBSTITUTE
CM032587.1	2053334	.	T	TAC	100	*	TYPE=INSERT
CM032587.1	2053336	.	T	A	100	*	TYPE=SUBSTITUTE
CM032587.1	2053340	.	CGT	C	100	*	TYPE=DELETE
CM032587.1	2053350	.	T	g	100	*	TYPE=SUBSTITUTE
CM032587.1	2053355	.	c	ctg	100	*	TYPE=INSERT
CM032587.1	2053362	.	C	G	100	*	TYPE=SUBSTITUTE
CM032587.1	2053363	.	G	A	100	*	TYPE=SUBSTITUTE
CM032587.1	2053370	.	CT	C	100	*	TYPE=DELETE
CM032587.1	2053375	.	TCTTTG	T	100	*	TYPE=DELETE
CM032587.1	2053382	.	T	C	100	*	TYPE=SUBSTITUTE
CM032587.1	2053383	.	C	A	100	*	TYPE=SUBSTITUTE
CM032587.1	2053387	.	G	A	100	*	TYPE=SUBSTITUTE
CM032587.1	2053402	.	C	A	100	*	TYPE=SUBSTITUTE
CM032587.1	2053408	.	T	A	100	*	TYPE=SUBSTITUTE
CM032587.1	2053412	.	AATTCAC	A	100	*	TYPE=DELETE
CM032587.1	2053422	.	TCA	T	100	*	TYPE=DELETE
CM032587.1	2053432	.	A	G	100	*	TYPE=SUBSTITUTE
CM032587.1	2053440	.	C	CCAT	100	*	TYPE=INSERT
CM032587.1	2053441	.	G	C	100	*	TYPE=SUBSTITUTE
CM032587.1	2053444	.	C	CTT	100	*	TYPE=INSERT
CM032587.1	2053450	.	C	G	100	*	TYPE=SUBSTITUTE
CM032587.1	2053460	.	G	A	100	*	TYPE=SUBSTITUTE
CM032587.1	2053470	.	A	G	100	*	TYPE=SUBSTITUTE
CM032587.1	2053475	.	G	GT	100	*	TYPE=INSERT
CM032587.1	2053482	.	C	T	100	*	TYPE=SUBSTITUTE
CM032587.1	2053503	.	A	c	100	*	TYPE=SUBSTITUTE
CM032587.1	2053510	.	A	g	100	*	TYPE=SUBSTITUTE
CM032587.1	2053523	.	C	T	100	*	TYPE=SUBSTITUTE

CM032587.1	2053543	.	C	T	100	*	TYPE=SUBSTITUTE
CM032587.1	2053560	.	C	T	100	*	TYPE=SUBSTITUTE
CM032587.1	2053578	.	G	A	100	*	TYPE=SUBSTITUTE
CM032587.1	2053591	.	A	G	100	*	TYPE=SUBSTITUTE
CM032587.1	2053608	.	T	C	100	*	TYPE=SUBSTITUTE
CM032587.1	2053619	.	G	A	100	*	TYPE=SUBSTITUTE
CM032587.1	2053631	.	C	T	100	*	TYPE=SUBSTITUTE
CM032587.1	2053636	.	A	G	100	*	TYPE=SUBSTITUTE
CM032587.1	2053637	.	C	T	100	*	TYPE=SUBSTITUTE
CM032587.1	2053642	.	C	T	100	*	TYPE=SUBSTITUTE
CM032587.1	2053646	.	A	AATG	100	*	TYPE=INSERT
CM032587.1	2053661	.	A	G	100	*	TYPE=SUBSTITUTE
CM032587.1	2053670	.	C	T	100	*	TYPE=SUBSTITUTE
CM032587.1	2053672	.	A	G	100	*	TYPE=SUBSTITUTE
CM032587.1	2053674	.	C	G	100	*	TYPE=SUBSTITUTE
CM032587.1	2053682	.	G	t	100	*	TYPE=SUBSTITUTE
CM032587.1	2053686	.	T	c	100	*	TYPE=SUBSTITUTE
CM032587.1	2053692	.	T	c	100	*	TYPE=SUBSTITUTE
CM032587.1	2053693	.	C	t	100	*	TYPE=SUBSTITUTE
CM032587.1	2053704	.	C	T	100	*	TYPE=SUBSTITUTE
CM032587.1	2053710	.	T	C	100	*	TYPE=SUBSTITUTE
CM032587.1	2053713	.	T	C	100	*	TYPE=SUBSTITUTE
CM032587.1	2053731	.	T	A	100	*	TYPE=SUBSTITUTE
CM032587.1	2053733	.	T	C	100	*	TYPE=SUBSTITUTE
CM032587.1	2053740	.	C	T	100	*	TYPE=SUBSTITUTE
CM032587.1	2053747	.	T	C	100	*	TYPE=SUBSTITUTE
CM032587.1	2053751	.	A	G	100	*	TYPE=SUBSTITUTE
CM032587.1	2053752	.	T	C	100	*	TYPE=SUBSTITUTE
CM032587.1	2053760	.	G	A	100	*	TYPE=SUBSTITUTE
CM032587.1	2053784	.	A	G	100	*	TYPE=SUBSTITUTE
CM032587.1	2053791	.	A	G	100	*	TYPE=SUBSTITUTE
CM032587.1	2053872	.	GGCAAG	G	100	*	TYPE=DELETE
CM032587.1	2053902	.	A	G	100	*	TYPE=SUBSTITUTE
CM032587.1	2053903	.	C	T	100	*	TYPE=SUBSTITUTE
CM032587.1	2053923	.	T	C	100	*	TYPE=SUBSTITUTE
CM032587.1	2053949	.	G	A	100	*	TYPE=SUBSTITUTE
CM032587.1	2053950	.	C	T	100	*	TYPE=SUBSTITUTE
CM032587.1	2053952	.	T	C	100	*	TYPE=SUBSTITUTE
CM032587.1	2054144	.	C	t	100	*	TYPE=SUBSTITUTE
CM032587.1	2054184	.	A	G	100	*	TYPE=SUBSTITUTE
CM032587.1	2054201	.	G	T	100	*	TYPE=SUBSTITUTE
CM032587.1	2054204	.	A	T	100	*	TYPE=SUBSTITUTE
CM032587.1	2054236	.	GG	G	100	*	TYPE=DELETE
CM032587.1	2054239	.	C	T	100	*	TYPE=SUBSTITUTE
CM032587.1	2054246	.	C	T	100	*	TYPE=SUBSTITUTE
CM032587.1	2054279	.	G	A	100	*	TYPE=SUBSTITUTE
CM032587.1	2054283	.	T	A	100	*	TYPE=SUBSTITUTE
CM032587.1	2054284	.	G	A	100	*	TYPE=SUBSTITUTE
CM032587.1	2054285	.	A	G	100	*	TYPE=SUBSTITUTE
CM032587.1	2054312	.	G	A	100	*	TYPE=SUBSTITUTE
CM032587.1	2054338	.	T	TCT	100	*	TYPE=INSERT
CM032587.1	2054339	.	G	A	100	*	TYPE=SUBSTITUTE
CM032587.1	2054380	.	A	C	100	*	TYPE=SUBSTITUTE
CM032587.1	2054395	.	G	C	100	*	TYPE=SUBSTITUTE
CM032587.1	2054439	.	C	CAA	100	*	TYPE=INSERT
CM032587.1	2054443	.	C	T	100	*	TYPE=SUBSTITUTE
CM032587.1	2054447	.	G	C	100	*	TYPE=SUBSTITUTE
CM032587.1	2054512	.	C	T	100	*	TYPE=SUBSTITUTE
CM032587.1	2054524	.	C	T	100	*	TYPE=SUBSTITUTE
CM032587.1	2054526	.	T	A	100	*	TYPE=SUBSTITUTE
CM032587.1	2054529	.	T	A	100	*	TYPE=SUBSTITUTE
CM032587.1	2054540	.	A	G	100	*	TYPE=SUBSTITUTE
CM032587.1	2054543	.	T	C	100	*	TYPE=SUBSTITUTE
CM032587.1	2054554	.	G	A	100	*	TYPE=SUBSTITUTE

CM032587.1	2054567	.	C	T	100	*	TYPE=SUBSTITUTE
CM032587.1	2054575	.	A	G	100	*	TYPE=SUBSTITUTE
CM032587.1	2054637	.	A	G	100	*	TYPE=SUBSTITUTE
CM032587.1	2054661	.	C	T	100	*	TYPE=SUBSTITUTE
CM032587.1	2054680	.	T	G	100	*	TYPE=SUBSTITUTE
CM032587.1	2054727	.	CCTGGC	C	100	*	TYPE=DELETE
CM032587.1	2054736	.	A	C	100	*	TYPE=SUBSTITUTE
CM032587.1	2054762	.	C	T	100	*	TYPE=SUBSTITUTE
CM032587.1	2054801	.	G	A	100	*	TYPE=SUBSTITUTE
CM032587.1	2054818	.	G	GAGAGGAG	100	*	TYPE=INSERT
CM032587.1	2054819	.	T	G	100	*	TYPE=SUBSTITUTE
CM032587.1	2054822	.	C	A	100	*	TYPE=SUBSTITUTE
CM032587.1	2054855	.	A	g	100	*	TYPE=SUBSTITUTE
CM032587.1	2054860	.	A	C	100	*	TYPE=SUBSTITUTE
CM032587.1	2054868	.	G	C	100	*	TYPE=SUBSTITUTE
CM032587.1	2054904	.	A	aa	100	*	TYPE=INSERT
CM032587.1	2054941	.	C	T	100	*	TYPE=SUBSTITUTE
CM032587.1	2054971	.	A	G	100	*	TYPE=SUBSTITUTE
CM032587.1	2054975	.	T	C	100	*	TYPE=SUBSTITUTE
CM032587.1	2054984	.	T	C	100	*	TYPE=SUBSTITUTE
CM032587.1	2054985	.	G	A	100	*	TYPE=SUBSTITUTE
CM032587.1	2055006	.	G	A	100	*	TYPE=SUBSTITUTE
CM032587.1	2055016	.	AGTATG	A	100	*	TYPE=DELETE
CM032587.1	2055027	.	C	T	100	*	TYPE=SUBSTITUTE
CM032587.1	2055059	.	AA	A	100	*	TYPE=DELETE
CM032587.1	2055071	.	C	T	100	*	TYPE=SUBSTITUTE
CM032587.1	2055078	.	CT	C	100	*	TYPE=DELETE
CM032587.1	2055084	.	t	tcc	100	*	TYPE=INSERT
CM032587.1	2055085	.	T	c	100	*	TYPE=SUBSTITUTE
CM032587.1	2055177	.	t	tc	100	*	TYPE=INSERT
CM032587.1	2055179	.	C	t	100	*	TYPE=SUBSTITUTE
CM032587.1	2055180	.	T	c	100	*	TYPE=SUBSTITUTE
CM032587.1	2055273	.	T	C	100	*	TYPE=SUBSTITUTE
CM032587.1	2055290	.	G	A	100	*	TYPE=SUBSTITUTE
CM032587.1	2055349	.	A	G	100	*	TYPE=SUBSTITUTE
CM032587.1	2055356	.	C	t	100	*	TYPE=SUBSTITUTE
CM032587.1	2055361	.	C	t	100	*	TYPE=SUBSTITUTE
CM032587.1	2055381	.	T	C	100	*	TYPE=SUBSTITUTE
CM032587.1	2055504	.	C	T	100	*	TYPE=SUBSTITUTE
CM032587.1	2055525	.	G	a	100	*	TYPE=SUBSTITUTE
CM032587.1	2055564	.	T	G	100	*	TYPE=SUBSTITUTE
CM032587.1	2055569	.	TA	T	100	*	TYPE=DELETE
CM032587.1	2055582	.	C	T	100	*	TYPE=SUBSTITUTE
CM032587.1	2055674	.	TT	T	100	*	TYPE=DELETE
CM032587.1	2055683	.	C	G	100	*	TYPE=SUBSTITUTE
CM032587.1	2055700	.	C	T	100	*	TYPE=SUBSTITUTE
CM032587.1	2055703	.	C	G	100	*	TYPE=SUBSTITUTE
CM032587.1	2055726	.	C	G	100	*	TYPE=SUBSTITUTE
CM032587.1	2055818	.	A	aaaaa	100	*	TYPE=INSERT
CM032587.1	2055862	.	C	T	100	*	TYPE=SUBSTITUTE
CM032587.1	2055865	.	A	G	100	*	TYPE=SUBSTITUTE
CM032587.1	2055867	.	C	T	100	*	TYPE=SUBSTITUTE
CM032587.1	2055869	.	T	A	100	*	TYPE=SUBSTITUTE
CM032587.1	2055895	.	C	t	100	*	TYPE=SUBSTITUTE
CM032587.1	2055900	.	A	g	100	*	TYPE=SUBSTITUTE
CM032587.1	2055902	.	CT	C	100	*	TYPE=DELETE
CM032587.1	2055908	.	T	C	100	*	TYPE=SUBSTITUTE
CM032587.1	2055947	.	G	C	100	*	TYPE=SUBSTITUTE
CM032587.1	2055984	.	T	C	100	*	TYPE=SUBSTITUTE
CM032587.1	2056010	.	T	C	100	*	TYPE=SUBSTITUTE
CM032587.1	2056013	.	T	C	100	*	TYPE=SUBSTITUTE
CM032587.1	2056019	.	C	T	100	*	TYPE=SUBSTITUTE
CM032587.1	2056024	.	A	G	100	*	TYPE=SUBSTITUTE
CM032587.1	2056026	.	C	G	100	*	TYPE=SUBSTITUTE

CM032587.1	2056045	.	C	A	100	*	TYPE=SUBSTITUTE
CM032587.1	2056048	.	T	TA	100	*	TYPE=INSERT
CM032587.1	2056057	.	T	C	100	*	TYPE=SUBSTITUTE
CM032587.1	2056059	.	C	T	100	*	TYPE=SUBSTITUTE
CM032587.1	2056063	.	T	TAAC	100	*	TYPE=INSERT
CM032587.1	2056077	.	A	g	100	*	TYPE=SUBSTITUTE
CM032587.1	2056079	.	A	g	100	*	TYPE=SUBSTITUTE
CM032587.1	2056101	.	C	t	100	*	TYPE=SUBSTITUTE
CM032587.1	2056103	.	G	A	100	*	TYPE=SUBSTITUTE
CM032587.1	2056105	.	A	C	100	*	TYPE=SUBSTITUTE
CM032587.1	2056108	.	G	A	100	*	TYPE=SUBSTITUTE
CM032587.1	2056112	.	T	C	100	*	TYPE=SUBSTITUTE
CM032587.1	2056118	.	A	C	100	*	TYPE=SUBSTITUTE
CM032587.1	2056137	.	A	T	100	*	TYPE=SUBSTITUTE
CM032587.1	2056141	.	T	C	100	*	TYPE=SUBSTITUTE
CM032587.1	2056142	.	TTTGA	T	100	*	TYPE=DELETE
CM032587.1	2056158	.	GCC	G	100	*	TYPE=DELETE
CM032587.1	2056169	.	C	t	100	*	TYPE=SUBSTITUTE
CM032587.1	2056276	.	c	cc	100	*	TYPE=INSERT
CM032587.1	2056285	.	C	T	100	*	TYPE=SUBSTITUTE
CM032587.1	2056320	.	GGG	G	100	*	TYPE=DELETE
CM032587.1	2056389	.	A	G	100	*	TYPE=SUBSTITUTE
CM032587.1	2056423	.	A	G	100	*	TYPE=SUBSTITUTE
CM032587.1	2056429	.	AGC	A	100	*	TYPE=DELETE
CM032587.1	2056533	.	T	ttt	100	*	TYPE=INSERT
CM032587.1	2056548	.	TT	T	100	*	TYPE=DELETE
CM032587.1	2056557	.	CT	C	100	*	TYPE=DELETE
CM032587.1	2056604	.	A	G	100	*	TYPE=SUBSTITUTE
CM032587.1	2056720	.	A	G	100	*	TYPE=SUBSTITUTE
CM032587.1	2056740	.	C	T	100	*	TYPE=SUBSTITUTE
CM032587.1	2056751	.	C	T	100	*	TYPE=SUBSTITUTE
CM032587.1	2056781	.	C	T	100	*	TYPE=SUBSTITUTE
CM032587.1	2056867	.	A	G	100	*	TYPE=SUBSTITUTE
CM032587.1	2056981	.	A	G	100	*	TYPE=SUBSTITUTE
CM032587.1	2057031	.	T	C	100	*	TYPE=SUBSTITUTE
CM032587.1	2057035	.	C	T	100	*	TYPE=SUBSTITUTE
CM032587.1	2057081	.	CGTC	C	100	*	TYPE=DELETE
CM032587.1	2057089	.	A	G	100	*	TYPE=SUBSTITUTE
CM032587.1	2057110	.	C	G	100	*	TYPE=SUBSTITUTE
CM032587.1	2057119	.	C	T	100	*	TYPE=SUBSTITUTE
CM032587.1	2057136	.	A	G	100	*	TYPE=SUBSTITUTE
CM032587.1	2057140	.	T	A	100	*	TYPE=SUBSTITUTE
CM032587.1	2057141	.	C	T	100	*	TYPE=SUBSTITUTE
CM032587.1	2057143	.	A	T	100	*	TYPE=SUBSTITUTE
CM032587.1	2057148	.	G	A	100	*	TYPE=SUBSTITUTE
CM032587.1	2057158	.	G	A	100	*	TYPE=SUBSTITUTE
CM032587.1	2057177	.	A	G	100	*	TYPE=SUBSTITUTE
CM032587.1	2057179	.	C	T	100	*	TYPE=SUBSTITUTE
CM032587.1	2057180	.	C	T	100	*	TYPE=SUBSTITUTE
CM032587.1	2057188	.	C	T	100	*	TYPE=SUBSTITUTE
CM032587.1	2057189	.	A	G	100	*	TYPE=SUBSTITUTE
CM032587.1	2057198	.	A	C	100	*	TYPE=SUBSTITUTE
CM032587.1	2057207	.	A	T	100	*	TYPE=SUBSTITUTE
CM032587.1	2057211	.	T	C	100	*	TYPE=SUBSTITUTE
CM032587.1	2057215	.	C	G	100	*	TYPE=SUBSTITUTE
CM032587.1	2057229	.	C	T	100	*	TYPE=SUBSTITUTE
CM032587.1	2057230	.	T	C	100	*	TYPE=SUBSTITUTE
CM032587.1	2057231	.	G	A	100	*	TYPE=SUBSTITUTE
CM032587.1	2057236	.	G	A	100	*	TYPE=SUBSTITUTE
CM032587.1	2057239	.	A	G	100	*	TYPE=SUBSTITUTE
CM032587.1	2057252	.	T	A	100	*	TYPE=SUBSTITUTE
CM032587.1	2057253	.	C	T	100	*	TYPE=SUBSTITUTE
CM032587.1	2057258	.	C	T	100	*	TYPE=SUBSTITUTE
CM032587.1	2057260	.	A	G	100	*	TYPE=SUBSTITUTE

CM032587.1	2057263	.	A	G	100	*	TYPE=SUBSTITUTE
CM032587.1	2057268	.	G	T	100	*	TYPE=SUBSTITUTE
CM032587.1	2057277	.	A	C	100	*	TYPE=SUBSTITUTE
CM032587.1	2057286	.	A	G	100	*	TYPE=SUBSTITUTE
CM032587.1	2057287	.	A	G	100	*	TYPE=SUBSTITUTE
CM032587.1	2057333	.	C	A	100	*	TYPE=SUBSTITUTE
CM032587.1	2057344	.	T	C	100	*	TYPE=SUBSTITUTE
CM032587.1	2057360	.	C	G	100	*	TYPE=SUBSTITUTE
CM032587.1	2057397	.	A	T	100	*	TYPE=SUBSTITUTE
CM032587.1	2057406	.	T	C	100	*	TYPE=SUBSTITUTE
CM032587.1	2057409	.	G	T	100	*	TYPE=SUBSTITUTE
CM032587.1	2057425	.	A	G	100	*	TYPE=SUBSTITUTE
CM032587.1	2057428	.	G	A	100	*	TYPE=SUBSTITUTE
CM032587.1	2057433	.	G	A	100	*	TYPE=SUBSTITUTE
CM032587.1	2057455	.	T	C	100	*	TYPE=SUBSTITUTE
CM032587.1	2057553	.	A	G	100	*	TYPE=SUBSTITUTE
CM032587.1	2057560	.	C	T	100	*	TYPE=SUBSTITUTE
CM032587.1	2057565	.	G	A	100	*	TYPE=SUBSTITUTE
CM032587.1	2057572	.	A	G	100	*	TYPE=SUBSTITUTE
CM032587.1	2057589	.	T	G	100	*	TYPE=SUBSTITUTE
CM032587.1	2057591	.	T	A	100	*	TYPE=SUBSTITUTE
CM032587.1	2057617	.	C	T	100	*	TYPE=SUBSTITUTE
CM032587.1	2057647	.	A	G	100	*	TYPE=SUBSTITUTE
CM032587.1	2057650	.	G	A	100	*	TYPE=SUBSTITUTE
CM032587.1	2057656	.	C	G	100	*	TYPE=SUBSTITUTE
CM032587.1	2057657	.	G	T	100	*	TYPE=SUBSTITUTE
CM032587.1	2057658	.	G	C	100	*	TYPE=SUBSTITUTE
CM032587.1	2057659	.	A	C	100	*	TYPE=SUBSTITUTE
CM032587.1	2057661	.	T	G	100	*	TYPE=SUBSTITUTE
CM032587.1	2057725	.	A	G	100	*	TYPE=SUBSTITUTE
CM032587.1	2057742	.	C	A	100	*	TYPE=SUBSTITUTE
CM032587.1	2057755	.	T	G	100	*	TYPE=SUBSTITUTE
CM032587.1	2057761	.	T	C	100	*	TYPE=SUBSTITUTE
CM032587.1	2057764	.	A	G	100	*	TYPE=SUBSTITUTE
CM032587.1	2057802	.	C	T	100	*	TYPE=SUBSTITUTE
CM032587.1	2057817	.	G	C	100	*	TYPE=SUBSTITUTE
CM032587.1	2057820	.	T	C	100	*	TYPE=SUBSTITUTE
CM032587.1	2057825	.	A	G	100	*	TYPE=SUBSTITUTE
CM032587.1	2057829	.	C	G	100	*	TYPE=SUBSTITUTE
CM032587.1	2057840	.	C	T	100	*	TYPE=SUBSTITUTE
CM032587.1	2057847	.	G	T	100	*	TYPE=SUBSTITUTE
CM032587.1	2057855	.	C	T	100	*	TYPE=SUBSTITUTE
CM032587.1	2057856	.	G	A	100	*	TYPE=SUBSTITUTE
CM032587.1	2057863	.	T	A	100	*	TYPE=SUBSTITUTE
CM032587.1	2057865	.	A	G	100	*	TYPE=SUBSTITUTE
CM032587.1	2057872	.	A	C	100	*	TYPE=SUBSTITUTE
CM032587.1	2057873	.	T	A	100	*	TYPE=SUBSTITUTE
CM032587.1	2057881	.	A	G	100	*	TYPE=SUBSTITUTE
CM032587.1	2057884	.	T	A	100	*	TYPE=SUBSTITUTE
CM032587.1	2057892	.	C	T	100	*	TYPE=SUBSTITUTE
CM032587.1	2057893	.	A	G	100	*	TYPE=SUBSTITUTE
CM032587.1	2057896	.	T	C	100	*	TYPE=SUBSTITUTE
CM032587.1	2057902	.	G	A	100	*	TYPE=SUBSTITUTE
CM032587.1	2057907	.	G	T	100	*	TYPE=SUBSTITUTE
CM032587.1	2057911	.	T	C	100	*	TYPE=SUBSTITUTE
CM032587.1	2057931	.	C	T	100	*	TYPE=SUBSTITUTE
CM032587.1	2057935	.	G	A	100	*	TYPE=SUBSTITUTE
CM032587.1	2057966	.	T	G	100	*	TYPE=SUBSTITUTE
CM032587.1	2057995	.	C	T	100	*	TYPE=SUBSTITUTE
CM032587.1	2057998	.	C	A	100	*	TYPE=SUBSTITUTE
CM032587.1	2058001	.	C	T	100	*	TYPE=SUBSTITUTE
CM032587.1	2058008	.	A	ATGT	100	*	TYPE=INSERT
CM032587.1	2058028	.	A	G	100	*	TYPE=SUBSTITUTE
CM032587.1	2058030	.	G	T	100	*	TYPE=SUBSTITUTE

CM032587.1	2058031	.	G	T	100	*	TYPE=SUBSTITUTE
CM032587.1	2058033	.	C	G	100	*	TYPE=SUBSTITUTE
CM032587.1	2058038	.	G	A	100	*	TYPE=SUBSTITUTE
CM032587.1	2058044	.	G	A	100	*	TYPE=SUBSTITUTE
CM032587.1	2058046	.	G	A	100	*	TYPE=SUBSTITUTE
CM032587.1	2058051	.	T	C	100	*	TYPE=SUBSTITUTE
CM032587.1	2058058	.	A	T	100	*	TYPE=SUBSTITUTE
CM032587.1	2058066	.	A	G	100	*	TYPE=SUBSTITUTE
CM032587.1	2058068	.	T	C	100	*	TYPE=SUBSTITUTE
CM032587.1	2058082	.	G	A	100	*	TYPE=SUBSTITUTE
CM032587.1	2058084	.	A	G	100	*	TYPE=SUBSTITUTE
CM032587.1	2058091	.	T	A	100	*	TYPE=SUBSTITUTE
CM032587.1	2058095	.	A	G	100	*	TYPE=SUBSTITUTE
CM032587.1	2058110	.	G	A	100	*	TYPE=SUBSTITUTE
CM032587.1	2058126	.	T	G	100	*	TYPE=SUBSTITUTE
CM032587.1	2058133	.	G	A	100	*	TYPE=SUBSTITUTE
CM032587.1	2058145	.	C	T	100	*	TYPE=SUBSTITUTE
CM032587.1	2058161	.	G	A	100	*	TYPE=SUBSTITUTE
CM032587.1	2058176	.	T	A	100	*	TYPE=SUBSTITUTE
CM032587.1	2058195	.	A	C	100	*	TYPE=SUBSTITUTE
CM032587.1	2058207	.	T	C	100	*	TYPE=SUBSTITUTE
CM032587.1	2058224	.	G	T	100	*	TYPE=SUBSTITUTE
CM032587.1	2058226	.	G	A	100	*	TYPE=SUBSTITUTE
CM032587.1	2058234	.	G	C	100	*	TYPE=SUBSTITUTE
CM032587.1	2058251	.	T	C	100	*	TYPE=SUBSTITUTE
CM032587.1	2058253	.	G	A	100	*	TYPE=SUBSTITUTE
CM032587.1	2058272	.	C	t	100	*	TYPE=SUBSTITUTE
CM032587.1	2058292	.	G	A	100	*	TYPE=SUBSTITUTE
CM032587.1	2058303	.	C	G	100	*	TYPE=SUBSTITUTE
CM032587.1	2058314	.	T	A	100	*	TYPE=SUBSTITUTE
CM032587.1	2058323	.	C	T	100	*	TYPE=SUBSTITUTE
CM032587.1	2058340	.	G	A	100	*	TYPE=SUBSTITUTE
CM032587.1	2058343	.	T	C	100	*	TYPE=SUBSTITUTE
CM032587.1	2058356	.	C	A	100	*	TYPE=SUBSTITUTE
CM032587.1	2058358	.	T	G	100	*	TYPE=SUBSTITUTE
CM032587.1	2058371	.	A	G	100	*	TYPE=SUBSTITUTE
CM032587.1	2058382	.	G	C	100	*	TYPE=SUBSTITUTE
CM032587.1	2058403	.	T	G	100	*	TYPE=SUBSTITUTE
CM032587.1	2058414	.	A	G	100	*	TYPE=SUBSTITUTE
CM032587.1	2058416	.	G	A	100	*	TYPE=SUBSTITUTE
CM032587.1	2058421	.	A	T	100	*	TYPE=SUBSTITUTE
CM032587.1	2058423	.	G	A	100	*	TYPE=SUBSTITUTE
CM032587.1	2058426	.	A	T	100	*	TYPE=SUBSTITUTE
CM032587.1	2058437	.	C	T	100	*	TYPE=SUBSTITUTE
CM032587.1	2058438	.	T	C	100	*	TYPE=SUBSTITUTE
CM032587.1	2058450	.	G	A	100	*	TYPE=SUBSTITUTE
CM032587.1	2058465	.	A	G	100	*	TYPE=SUBSTITUTE
CM032587.1	2058483	.	T	G	100	*	TYPE=SUBSTITUTE
CM032587.1	2058504	.	A	G	100	*	TYPE=SUBSTITUTE
CM032587.1	2058507	.	T	C	100	*	TYPE=SUBSTITUTE
CM032587.1	2058528	.	C	T	100	*	TYPE=SUBSTITUTE
CM032587.1	2058651	.	C	T	100	*	TYPE=SUBSTITUTE
CM032587.1	2058661	.	T	C	100	*	TYPE=SUBSTITUTE
CM032587.1	2058670	.	G	T	100	*	TYPE=SUBSTITUTE
CM032587.1	2058676	.	AT	A	100	*	TYPE=DELETE
CM032587.1	2058681	.	C	G	100	*	TYPE=SUBSTITUTE
CM032587.1	2058693	.	G	C	100	*	TYPE=SUBSTITUTE
CM032587.1	2058701	.	C	T	100	*	TYPE=SUBSTITUTE
CM032587.1	2058708	.	C	A	100	*	TYPE=SUBSTITUTE
CM032587.1	2058712	.	T	C	100	*	TYPE=SUBSTITUTE
CM032587.1	2058714	.	G	A	100	*	TYPE=SUBSTITUTE
CM032587.1	2058744	.	C	T	100	*	TYPE=SUBSTITUTE
CM032587.1	2058747	.	C	A	100	*	TYPE=SUBSTITUTE
CM032587.1	2058772	.	A	G	100	*	TYPE=SUBSTITUTE

CM032587.1	2058773	.	A	G	100	*	TYPE=SUBSTITUTE
CM032587.1	2058779	.	G	A	100	*	TYPE=SUBSTITUTE
CM032587.1	2058780	.	G	T	100	*	TYPE=SUBSTITUTE
CM032587.1	2058785	.	G	A	100	*	TYPE=SUBSTITUTE
CM032587.1	2058794	.	G	A	100	*	TYPE=SUBSTITUTE
CM032587.1	2058806	.	A	G	100	*	TYPE=SUBSTITUTE
CM032587.1	2058810	.	G	A	100	*	TYPE=SUBSTITUTE
CM032587.1	2058811	.	C	G	100	*	TYPE=SUBSTITUTE
CM032587.1	2058812	.	C	T	100	*	TYPE=SUBSTITUTE
CM032587.1	2058816	.	A	G	100	*	TYPE=SUBSTITUTE
CM032587.1	2058833	.	T	C	100	*	TYPE=SUBSTITUTE
CM032587.1	2058835	.	C	T	100	*	TYPE=SUBSTITUTE
CM032587.1	2058845	.	T	C	100	*	TYPE=SUBSTITUTE
CM032587.1	2058856	.	G	T	100	*	TYPE=SUBSTITUTE
CM032587.1	2058870	.	A	G	100	*	TYPE=SUBSTITUTE
CM032587.1	2058896	.	T	C	100	*	TYPE=SUBSTITUTE
CM032587.1	2058905	.	C	G	100	*	TYPE=SUBSTITUTE
CM032587.1	2058913	.	C	T	100	*	TYPE=SUBSTITUTE
CM032587.1	2058914	.	A	T	100	*	TYPE=SUBSTITUTE
CM032587.1	2058920	.	A	C	100	*	TYPE=SUBSTITUTE
CM032587.1	2058940	.	T	G	100	*	TYPE=SUBSTITUTE
CM032587.1	2058942	.	A	ATTC	100	*	TYPE=INSERT
CM032587.1	2058952	.	A	G	100	*	TYPE=SUBSTITUTE
CM032587.1	2058980	.	A	G	100	*	TYPE=SUBSTITUTE
CM032587.1	2058991	.	A	G	100	*	TYPE=SUBSTITUTE
CM032587.1	2058996	.	A	C	100	*	TYPE=SUBSTITUTE
CM032587.1	2059009	.	C	G	100	*	TYPE=SUBSTITUTE
CM032587.1	2059017	.	A	C	100	*	TYPE=SUBSTITUTE
CM032587.1	2059024	.	C	t	100	*	TYPE=SUBSTITUTE
CM032587.1	2059098	.	A	G	100	*	TYPE=SUBSTITUTE
CM032587.1	2059104	.	G	A	100	*	TYPE=SUBSTITUTE
CM032587.1	2059122	.	C	A	100	*	TYPE=SUBSTITUTE
CM032587.1	2059167	.	G	a	100	*	TYPE=SUBSTITUTE
CM032587.1	2059194	.	A	G	100	*	TYPE=SUBSTITUTE
CM032587.1	2059212	.	A	G	100	*	TYPE=SUBSTITUTE
CM032587.1	2059239	.	G	A	100	*	TYPE=SUBSTITUTE
CM032587.1	2059251	.	T	C	100	*	TYPE=SUBSTITUTE
CM032587.1	2059268	.	T	A	100	*	TYPE=SUBSTITUTE
CM032587.1	2059274	.	T	C	100	*	TYPE=SUBSTITUTE
CM032587.1	2059314	.	A	G	100	*	TYPE=SUBSTITUTE
CM032587.1	2059326	.	T	A	100	*	TYPE=SUBSTITUTE
CM032587.1	2059342	.	A	G	100	*	TYPE=SUBSTITUTE
CM032587.1	2059353	.	C	A	100	*	TYPE=SUBSTITUTE
CM032587.1	2059354	.	T	C	100	*	TYPE=SUBSTITUTE
CM032587.1	2059355	.	GGAA	G	100	*	TYPE=DELETE
CM032587.1	2059361	.	A	G	100	*	TYPE=SUBSTITUTE
CM032587.1	2059367	.	C	T	100	*	TYPE=SUBSTITUTE
CM032587.1	2059395	.	G	C	100	*	TYPE=SUBSTITUTE
CM032587.1	2059419	.	G	T	100	*	TYPE=SUBSTITUTE
CM032587.1	2059488	.	A	T	100	*	TYPE=SUBSTITUTE
CM032587.1	2059551	.	T	C	100	*	TYPE=SUBSTITUTE
CM032587.1	2059743	.	G	A	100	*	TYPE=SUBSTITUTE
CM032587.1	2059791	.	G	A	100	*	TYPE=SUBSTITUTE
CM032587.1	2059903	.	G	A	100	*	TYPE=SUBSTITUTE
CM032587.1	2059968	.	A	G	100	*	TYPE=SUBSTITUTE
CM032587.1	2059976	.	G	T	100	*	TYPE=SUBSTITUTE
CM032587.1	2060035	.	T	g	100	*	TYPE=SUBSTITUTE
CM032587.1	2060085	.	GC	G	100	*	TYPE=DELETE
CM032587.1	2060123	.	C	A	100	*	TYPE=SUBSTITUTE
CM032587.1	2060134	.	A	G	100	*	TYPE=SUBSTITUTE
CM032587.1	2060149	.	T	A	100	*	TYPE=SUBSTITUTE
CM032587.1	2060166	.	A	AA	100	*	TYPE=INSERT
CM032587.1	2060168	.	T	A	100	*	TYPE=SUBSTITUTE
CM032587.1	2060171	.	A	G	100	*	TYPE=SUBSTITUTE

CM032587.1	2060191	.	A	C	100	*	TYPE=SUBSTITUTE
CM032587.1	2060195	.	G	GA	100	*	TYPE=INSERT
CM032587.1	2060196	.	C	G	100	*	TYPE=SUBSTITUTE
CM032587.1	2060226	.	AGCA	A	100	*	TYPE=DELETE
CM032587.1	2060236	.	G	A	100	*	TYPE=SUBSTITUTE
CM032587.1	2060242	.	T	C	100	*	TYPE=SUBSTITUTE
CM032587.1	2060248	.	T	C	100	*	TYPE=SUBSTITUTE
CM032587.1	2060273	.	G	A	100	*	TYPE=SUBSTITUTE
CM032587.1	2060297	.	G	c	100	*	TYPE=SUBSTITUTE
CM032587.1	2060312	.	A	G	100	*	TYPE=SUBSTITUTE
CM032587.1	2060318	.	A	T	100	*	TYPE=SUBSTITUTE
CM032587.1	2060336	.	G	A	100	*	TYPE=SUBSTITUTE
CM032587.1	2060366	.	A	G	100	*	TYPE=SUBSTITUTE
CM032587.1	2060384	.	G	A	100	*	TYPE=SUBSTITUTE
CM032587.1	2060464	.	T	C	100	*	TYPE=SUBSTITUTE
CM032587.1	2060606	.	A	g	100	*	TYPE=SUBSTITUTE
CM032587.1	2060650	.	A	G	100	*	TYPE=SUBSTITUTE
CM032587.1	2060651	.	T	C	100	*	TYPE=SUBSTITUTE
CM032587.1	2060686	.	C	G	100	*	TYPE=SUBSTITUTE
CM032587.1	2060715	.	A	G	100	*	TYPE=SUBSTITUTE
CM032587.1	2060719	.	G	A	100	*	TYPE=SUBSTITUTE
CM032587.1	2060722	.	T	G	100	*	TYPE=SUBSTITUTE
CM032587.1	2060723	.	GCCCAAGAACC	G	100	*	TYPE=DELETE
CM032587.1	2060777	.	A	C	100	*	TYPE=SUBSTITUTE
CM032587.1	2060905	.	A	AC	100	*	TYPE=INSERT
CM032587.1	2060910	.	T	G	100	*	TYPE=SUBSTITUTE
CM032587.1	2060922	.	C	T	100	*	TYPE=SUBSTITUTE
CM032587.1	2060970	.	C	G	100	*	TYPE=SUBSTITUTE
CM032587.1	2060984	.	A	C	100	*	TYPE=SUBSTITUTE
CM032587.1	2060992	.	T	TCCATTC	100	*	TYPE=INSERT
CM032587.1	2061000	.	C	T	100	*	TYPE=SUBSTITUTE
CM032587.1	2061001	.	T	G	100	*	TYPE=SUBSTITUTE
CM032587.1	2061003	.	C	T	100	*	TYPE=SUBSTITUTE
CM032587.1	2061005	.	c	cccgtact	100	*	TYPE=INSERT
CM032587.1	2061007	.	T	c	100	*	TYPE=SUBSTITUTE
CM032587.1	2061126	.	A	G	100	*	TYPE=SUBSTITUTE
CM032587.1	2061129	.	CA	C	100	*	TYPE=DELETE
CM032587.1	2061153	.	T	G	100	*	TYPE=SUBSTITUTE
CM032587.1	2061310	.	t	tt	100	*	TYPE=INSERT
CM032587.1	2061312	.	C	t	100	*	TYPE=SUBSTITUTE
CM032587.1	2061367	.	G	A	100	*	TYPE=SUBSTITUTE
CM032587.1	2061388	.	C	t	100	*	TYPE=SUBSTITUTE
CM032587.1	2061391	.	T	c	100	*	TYPE=SUBSTITUTE
CM032587.1	2061395	.	T	C	100	*	TYPE=SUBSTITUTE
CM032587.1	2061406	.	T	C	100	*	TYPE=SUBSTITUTE
CM032587.1	2061419	.	T	G	100	*	TYPE=SUBSTITUTE
CM032587.1	2061470	.	C	T	100	*	TYPE=SUBSTITUTE
CM032587.1	2061504	.	G	A	100	*	TYPE=SUBSTITUTE
CM032587.1	2061538	.	A	G	100	*	TYPE=SUBSTITUTE
CM032587.1	2061608	.	T	TT	100	*	TYPE=INSERT
CM032587.1	2061641	.	A	C	100	*	TYPE=SUBSTITUTE
CM032587.1	2061666	.	C	T	100	*	TYPE=SUBSTITUTE
CM032587.1	2061679	.	A	G	100	*	TYPE=SUBSTITUTE
CM032587.1	2061688	.	C	T	100	*	TYPE=SUBSTITUTE
CM032587.1	2061710	.	C	T	100	*	TYPE=SUBSTITUTE
CM032587.1	2061727	.	C	T	100	*	TYPE=SUBSTITUTE
CM032587.1	2061905	.	A	G	100	*	TYPE=SUBSTITUTE
CM032587.1	2061928	.	T	C	100	*	TYPE=SUBSTITUTE
CM032587.1	2061958	.	C	T	100	*	TYPE=SUBSTITUTE
CM032587.1	2061962	.	C	A	100	*	TYPE=SUBSTITUTE
CM032587.1	2061975	.	G	A	100	*	TYPE=SUBSTITUTE
CM032587.1	2062025	.	T	G	100	*	TYPE=SUBSTITUTE
CM032587.1	2062030	.	G	A	100	*	TYPE=SUBSTITUTE
CM032587.1	2062037	.	A	G	100	*	TYPE=SUBSTITUTE

CM032587.1	2062057	.	T	C	100	*	TYPE=SUBSTITUTE
CM032587.1	2062094	.	G	A	100	*	TYPE=SUBSTITUTE
CM032587.1	2062142	.	G	A	100	*	TYPE=SUBSTITUTE
CM032587.1	2062143	.	A	G	100	*	TYPE=SUBSTITUTE
CM032587.1	2062201	.	C	T	100	*	TYPE=SUBSTITUTE
CM032587.1	2062245	.	C	G	100	*	TYPE=SUBSTITUTE
CM032587.1	2062253	.	C	T	100	*	TYPE=SUBSTITUTE
CM032587.1	2062269	.	C	A	100	*	TYPE=SUBSTITUTE
CM032587.1	2062275	.	GT	G	100	*	TYPE=DELETE
CM032587.1	2062283	.	T	C	100	*	TYPE=SUBSTITUTE
CM032587.1	2062287	.	G	A	100	*	TYPE=SUBSTITUTE
CM032587.1	2062307	.	C	A	100	*	TYPE=SUBSTITUTE
CM032587.1	2062308	.	A	G	100	*	TYPE=SUBSTITUTE
CM032587.1	2062325	.	A	AC	100	*	TYPE=INSERT
CM032587.1	2062341	.	A	G	100	*	TYPE=SUBSTITUTE
CM032587.1	2062347	.	C	T	100	*	TYPE=SUBSTITUTE
CM032587.1	2062390	.	C	T	100	*	TYPE=SUBSTITUTE
CM032587.1	2062420	.	A	G	100	*	TYPE=SUBSTITUTE
CM032587.1	2062436	.	G	T	100	*	TYPE=SUBSTITUTE
CM032587.1	2062493	.	TTTT	T	100	*	TYPE=DELETE
CM032587.1	2062504	.	A	G	100	*	TYPE=SUBSTITUTE
CM032587.1	2062526	.	C	T	100	*	TYPE=SUBSTITUTE
CM032587.1	2062529	.	A	G	100	*	TYPE=SUBSTITUTE
CM032587.1	2062535	.	G	A	100	*	TYPE=SUBSTITUTE
CM032587.1	2062547	.	C	A	100	*	TYPE=SUBSTITUTE
CM032587.1	2062566	.	A	g	100	*	TYPE=SUBSTITUTE
CM032587.1	2062575	.	G	C	100	*	TYPE=SUBSTITUTE
CM032587.1	2062579	.	T	C	100	*	TYPE=SUBSTITUTE
CM032587.1	2062622	.	C	T	100	*	TYPE=SUBSTITUTE
CM032587.1	2062631	.	A	G	100	*	TYPE=SUBSTITUTE
CM032587.1	2062694	.	T	C	100	*	TYPE=SUBSTITUTE
CM032587.1	2062727	.	G	T	100	*	TYPE=SUBSTITUTE
CM032587.1	2062747	.	A	C	100	*	TYPE=SUBSTITUTE
CM032587.1	2062767	.	C	T	100	*	TYPE=SUBSTITUTE
CM032587.1	2062796	.	C	T	100	*	TYPE=SUBSTITUTE
CM032587.1	2062800	.	T	C	100	*	TYPE=SUBSTITUTE
CM032587.1	2062811	.	T	C	100	*	TYPE=SUBSTITUTE
CM032587.1	2062826	.	T	C	100	*	TYPE=SUBSTITUTE
CM032587.1	2062834	.	T	C	100	*	TYPE=SUBSTITUTE
CM032587.1	2062839	.	A	G	100	*	TYPE=SUBSTITUTE
CM032587.1	2062840	.	T	C	100	*	TYPE=SUBSTITUTE
CM032587.1	2062863	.	T	C	100	*	TYPE=SUBSTITUTE
CM032587.1	2062895	.	G	A	100	*	TYPE=SUBSTITUTE
CM032587.1	2062910	.	C	T	100	*	TYPE=SUBSTITUTE
CM032587.1	2062917	.	TA	T	100	*	TYPE=DELETE
CM032587.1	2062929	.	C	T	100	*	TYPE=SUBSTITUTE
CM032587.1	2062936	.	C	T	100	*	TYPE=SUBSTITUTE
CM032587.1	2062939	.	A	G	100	*	TYPE=SUBSTITUTE
CM032587.1	2062942	.	A	T	100	*	TYPE=SUBSTITUTE
CM032587.1	2063032	.	T	TCT	100	*	TYPE=INSERT
CM032587.1	2063036	.	G	T	100	*	TYPE=SUBSTITUTE
CM032587.1	2063037	.	A	T	100	*	TYPE=SUBSTITUTE
CM032587.1	2063039	.	T	A	100	*	TYPE=SUBSTITUTE
CM032587.1	2063046	.	A	G	100	*	TYPE=SUBSTITUTE
CM032587.1	2063062	.	T	G	100	*	TYPE=SUBSTITUTE
CM032587.1	2063063	.	C	T	100	*	TYPE=SUBSTITUTE
CM032587.1	2063109	.	C	CC	100	*	TYPE=INSERT
CM032587.1	2063121	.	T	C	100	*	TYPE=SUBSTITUTE
CM032587.1	2063137	.	G	C	100	*	TYPE=SUBSTITUTE
CM032587.1	2063139	.	G	A	100	*	TYPE=SUBSTITUTE
CM032587.1	2063238	.	A	G	100	*	TYPE=SUBSTITUTE
CM032587.1	2063252	.	A	G	100	*	TYPE=SUBSTITUTE
CM032587.1	2063259	.	C	T	100	*	TYPE=SUBSTITUTE
CM032587.1	2063260	.	G	A	100	*	TYPE=SUBSTITUTE

CM032587.1	2063297	.	C	T	100	*	TYPE=SUBSTITUTE
CM032587.1	2063321	.	C	T	100	*	TYPE=SUBSTITUTE
CM032587.1	2063326	.	C	A	100	*	TYPE=SUBSTITUTE
CM032587.1	2063350	.	T	C	100	*	TYPE=SUBSTITUTE
CM032587.1	2063430	.	CC	C	100	*	TYPE=DELETE
CM032587.1	2063435	.	A	C	100	*	TYPE=SUBSTITUTE
CM032587.1	2063461	.	G	C	100	*	TYPE=SUBSTITUTE
CM032587.1	2063467	.	A	G	100	*	TYPE=SUBSTITUTE
CM032587.1	2063479	.	A	G	100	*	TYPE=SUBSTITUTE
CM032587.1	2063507	.	C	G	100	*	TYPE=SUBSTITUTE
CM032587.1	2063541	.	A	C	100	*	TYPE=SUBSTITUTE
CM032587.1	2063559	.	T	C	100	*	TYPE=SUBSTITUTE
CM032587.1	2063569	.	C	T	100	*	TYPE=SUBSTITUTE
CM032587.1	2063602	.	A	G	100	*	TYPE=SUBSTITUTE
CM032587.1	2063605	.	T	TG	100	*	TYPE=INSERT
CM032587.1	2063677	.	G	A	100	*	TYPE=SUBSTITUTE
CM032587.1	2063706	.	A	G	100	*	TYPE=SUBSTITUTE
CM032587.1	2063709	.	C	CA	100	*	TYPE=INSERT
CM032587.1	2063741	.	G	t	100	*	TYPE=SUBSTITUTE
CM032587.1	2063761	.	t	tt	100	*	TYPE=INSERT
CM032587.1	2063773	.	C	T	100	*	TYPE=SUBSTITUTE
CM032587.1	2063791	.	T	C	100	*	TYPE=SUBSTITUTE
CM032587.1	2063799	.	T	C	100	*	TYPE=SUBSTITUTE
CM032587.1	2063807	.	T	A	100	*	TYPE=SUBSTITUTE
CM032587.1	2063854	.	C	CCT	100	*	TYPE=INSERT
CM032587.1	2063908	.	C	T	100	*	TYPE=SUBSTITUTE
CM032587.1	2063911	.	T	C	100	*	TYPE=SUBSTITUTE
CM032587.1	2063936	.	GG	G	100	*	TYPE=DELETE
CM032587.1	2064023	.	C	T	100	*	TYPE=SUBSTITUTE
CM032587.1	2064030	.	G	A	100	*	TYPE=SUBSTITUTE
CM032587.1	2064130	.	G	C	100	*	TYPE=SUBSTITUTE
CM032587.1	2064135	.	C	T	100	*	TYPE=SUBSTITUTE
CM032587.1	2064152	.	G	GA	100	*	TYPE=INSERT
CM032587.1	2064259	.	T	TT	100	*	TYPE=INSERT
CM032587.1	2064304	.	A	C	100	*	TYPE=SUBSTITUTE
CM032587.1	2064338	.	C	T	100	*	TYPE=SUBSTITUTE
CM032587.1	2064364	.	T	C	100	*	TYPE=SUBSTITUTE
CM032587.1	2064370	.	T	C	100	*	TYPE=SUBSTITUTE
CM032587.1	2064408	.	A	G	100	*	TYPE=SUBSTITUTE
CM032587.1	2064413	.	T	C	100	*	TYPE=SUBSTITUTE
CM032587.1	2064467	.	AA	A	100	*	TYPE=DELETE
CM032587.1	2064544	.	A	C	100	*	TYPE=SUBSTITUTE
CM032587.1	2064546	.	A	G	100	*	TYPE=SUBSTITUTE
CM032587.1	2064579	.	C	T	100	*	TYPE=SUBSTITUTE
CM032587.1	2064580	.	G	C	100	*	TYPE=SUBSTITUTE
CM032587.1	2064581	.	T	G	100	*	TYPE=SUBSTITUTE
CM032587.1	2064604	.	C	CTCGAATCGTTGCCCAC	100	*	TYPE=INSERT
CM032587.1	2064621	.	A	G	100	*	TYPE=SUBSTITUTE
CM032587.1	2064699	.	C	A	100	*	TYPE=SUBSTITUTE
CM032587.1	2064762	.	C	T	100	*	TYPE=SUBSTITUTE
CM032587.1	2064822	.	G	A	100	*	TYPE=SUBSTITUTE
CM032587.1	2064840	.	T	TT	100	*	TYPE=INSERT
CM032587.1	2064900	.	G	C	100	*	TYPE=SUBSTITUTE
CM032587.1	2064940	.	T	TT	100	*	TYPE=INSERT
CM032587.1	2064950	.	G	T	100	*	TYPE=SUBSTITUTE
CM032587.1	2064954	.	C	T	100	*	TYPE=SUBSTITUTE
CM032587.1	2064955	.	T	C	100	*	TYPE=SUBSTITUTE
CM032587.1	2064997	.	C	T	100	*	TYPE=SUBSTITUTE
CM032587.1	2064999	.	C	T	100	*	TYPE=SUBSTITUTE
CM032587.1	2065000	.	G	GGAT	100	*	TYPE=INSERT
CM032587.1	2065044	.	T	C	100	*	TYPE=SUBSTITUTE
CM032587.1	2065048	.	A	G	100	*	TYPE=SUBSTITUTE
CM032587.1	2065062	.	A	G	100	*	TYPE=SUBSTITUTE
CM032587.1	2065143	.	G	A	100	*	TYPE=SUBSTITUTE

CM032587.1	2065171	.	T	G	100	*	TYPE=SUBSTITUTE
CM032587.1	2065173	.	G	A	100	*	TYPE=SUBSTITUTE
CM032587.1	2065184	.	A	G	100	*	TYPE=SUBSTITUTE
CM032587.1	2065199	.	C	A	100	*	TYPE=SUBSTITUTE
CM032587.1	2065230	.	T	C	100	*	TYPE=SUBSTITUTE
CM032587.1	2065233	.	C	G	100	*	TYPE=SUBSTITUTE
CM032587.1	2065250	.	GG	G	100	*	TYPE=DELETE
CM032587.1	2065294	.	G	A	100	*	TYPE=SUBSTITUTE
CM032587.1	2065311	.	T	TGGTCTAT	100	*	TYPE=INSERT
CM032587.1	2065312	.	T	C	100	*	TYPE=SUBSTITUTE
CM032587.1	2065319	.	A	G	100	*	TYPE=SUBSTITUTE
CM032587.1	2065327	.	T	C	100	*	TYPE=SUBSTITUTE
CM032587.1	2065340	.	C	T	100	*	TYPE=SUBSTITUTE
CM032587.1	2065380	.	T	G	100	*	TYPE=SUBSTITUTE
CM032587.1	2065384	.	T	C	100	*	TYPE=SUBSTITUTE
CM032587.1	2065385	.	GT	G	100	*	TYPE=DELETE
CM032587.1	2065387	.	G	C	100	*	TYPE=SUBSTITUTE
CM032587.1	2065396	.	C	T	100	*	TYPE=SUBSTITUTE
CM032587.1	2065422	.	A	G	100	*	TYPE=SUBSTITUTE
CM032587.1	2065534	.	T	c	100	*	TYPE=SUBSTITUTE
CM032587.1	2065555	.	G	a	100	*	TYPE=SUBSTITUTE
CM032587.1	2065567	.	C	A	100	*	TYPE=SUBSTITUTE
CM032587.1	2065590	.	C	A	100	*	TYPE=SUBSTITUTE
CM032587.1	2065642	.	T	C	100	*	TYPE=SUBSTITUTE
CM032587.1	2065648	.	A	G	100	*	TYPE=SUBSTITUTE
CM032587.1	2065650	.	T	C	100	*	TYPE=SUBSTITUTE
CM032587.1	2065654	.	G	T	100	*	TYPE=SUBSTITUTE
CM032587.1	2065723	.	A	G	100	*	TYPE=SUBSTITUTE
CM032587.1	2065755	.	A	G	100	*	TYPE=SUBSTITUTE
CM032587.1	2065777	.	C	T	100	*	TYPE=SUBSTITUTE
CM032587.1	2065782	.	A	G	100	*	TYPE=SUBSTITUTE
CM032587.1	2065826	.	C	T	100	*	TYPE=SUBSTITUTE
CM032587.1	2065828	.	C	T	100	*	TYPE=SUBSTITUTE
CM032587.1	2065829	.	C	T	100	*	TYPE=SUBSTITUTE
CM032587.1	2065846	.	C	T	100	*	TYPE=SUBSTITUTE
CM032587.1	2065869	.	T	C	100	*	TYPE=SUBSTITUTE
CM032587.1	2065873	.	G	A	100	*	TYPE=SUBSTITUTE
CM032587.1	2065887	.	C	T	100	*	TYPE=SUBSTITUTE
CM032587.1	2065899	.	A	G	100	*	TYPE=SUBSTITUTE
CM032587.1	2065920	.	T	G	100	*	TYPE=SUBSTITUTE
CM032587.1	2065924	.	T	A	100	*	TYPE=SUBSTITUTE
CM032587.1	2065956	.	C	G	100	*	TYPE=SUBSTITUTE
CM032587.1	2065973	.	A	G	100	*	TYPE=SUBSTITUTE
CM032587.1	2065979	.	A	G	100	*	TYPE=SUBSTITUTE
CM032587.1	2065980	.	T	C	100	*	TYPE=SUBSTITUTE
CM032587.1	2066055	.	A	G	100	*	TYPE=SUBSTITUTE
CM032587.1	2066073	.	C	T	100	*	TYPE=SUBSTITUTE
CM032587.1	2066097	.	C	T	100	*	TYPE=SUBSTITUTE
CM032587.1	2066112	.	A	G	100	*	TYPE=SUBSTITUTE
CM032587.1	2066151	.	C	T	100	*	TYPE=SUBSTITUTE
CM032587.1	2066154	.	T	C	100	*	TYPE=SUBSTITUTE
CM032587.1	2066187	.	T	A	100	*	TYPE=SUBSTITUTE
CM032587.1	2066199	.	A	G	100	*	TYPE=SUBSTITUTE
CM032587.1	2066225	.	T	G	100	*	TYPE=SUBSTITUTE
CM032587.1	2066244	.	C	T	100	*	TYPE=SUBSTITUTE
CM032587.1	2066270	.	A	G	100	*	TYPE=SUBSTITUTE
CM032587.1	2066277	.	C	G	100	*	TYPE=SUBSTITUTE
CM032587.1	2066286	.	A	G	100	*	TYPE=SUBSTITUTE
CM032587.1	2066322	.	A	G	100	*	TYPE=SUBSTITUTE
CM032587.1	2066361	.	TGA	T	100	*	TYPE=DELETE
CM032587.1	2066364	.	G	A	100	*	TYPE=SUBSTITUTE
CM032587.1	2066374	.	A	G	100	*	TYPE=SUBSTITUTE
CM032587.1	2066389	.	G	A	100	*	TYPE=SUBSTITUTE
CM032587.1	2066390	.	T	C	100	*	TYPE=SUBSTITUTE

CM032587.1	2066402	.	G	A	100	*	TYPE=SUBSTITUTE
CM032587.1	2066414	.	T	TG	100	*	TYPE=INSERT
CM032587.1	2066419	.	C	T	100	*	TYPE=SUBSTITUTE
CM032587.1	2066461	.	A	T	100	*	TYPE=SUBSTITUTE
CM032587.1	2066462	.	G	GA	100	*	TYPE=INSERT
CM032587.1	2066464	.	A	G	100	*	TYPE=SUBSTITUTE
CM032587.1	2066485	.	G	A	100	*	TYPE=SUBSTITUTE
CM032587.1	2066512	.	G	A	100	*	TYPE=SUBSTITUTE
CM032587.1	2066518	.	G	A	100	*	TYPE=SUBSTITUTE
CM032587.1	2066523	.	A	C	100	*	TYPE=SUBSTITUTE
CM032587.1	2066533	.	G	A	100	*	TYPE=SUBSTITUTE
CM032587.1	2066538	.	G	A	100	*	TYPE=SUBSTITUTE
CM032587.1	2066546	.	A	T	100	*	TYPE=SUBSTITUTE
CM032587.1	2066549	.	C	A	100	*	TYPE=SUBSTITUTE
CM032587.1	2066565	.	A	G	100	*	TYPE=SUBSTITUTE
CM032587.1	2066566	.	A	G	100	*	TYPE=SUBSTITUTE
CM032587.1	2066573	.	C	T	100	*	TYPE=SUBSTITUTE
CM032587.1	2066574	.	A	G	100	*	TYPE=SUBSTITUTE
CM032587.1	2066582	.	A	G	100	*	TYPE=SUBSTITUTE
CM032587.1	2066584	.	T	G	100	*	TYPE=SUBSTITUTE
CM032587.1	2066596	.	G	A	100	*	TYPE=SUBSTITUTE
CM032587.1	2066609	.	C	T	100	*	TYPE=SUBSTITUTE
CM032587.1	2066613	.	T	C	100	*	TYPE=SUBSTITUTE
CM032587.1	2066618	.	G	C	100	*	TYPE=SUBSTITUTE
CM032587.1	2066638	.	G	A	100	*	TYPE=SUBSTITUTE
CM032587.1	2066643	.	A	G	100	*	TYPE=SUBSTITUTE
CM032587.1	2066657	.	A	G	100	*	TYPE=SUBSTITUTE
CM032587.1	2066663	.	C	G	100	*	TYPE=SUBSTITUTE
CM032587.1	2066672	.	A	T	100	*	TYPE=SUBSTITUTE
CM032587.1	2066673	.	C	G	100	*	TYPE=SUBSTITUTE
CM032587.1	2066676	.	A	G	100	*	TYPE=SUBSTITUTE
CM032587.1	2066686	.	AGAAT	A	100	*	TYPE=DELETE
CM032587.1	2066691	.	C	A	100	*	TYPE=SUBSTITUTE
CM032587.1	2066701	.	C	T	100	*	TYPE=SUBSTITUTE
CM032587.1	2066799	.	TT	T	100	*	TYPE=DELETE
CM032587.1	2066814	.	A	C	100	*	TYPE=SUBSTITUTE
CM032587.1	2066827	.	A	AG	100	*	TYPE=INSERT
CM032587.1	2066828	.	A	G	100	*	TYPE=SUBSTITUTE
CM032587.1	2066843	.	C	G	100	*	TYPE=SUBSTITUTE
CM032587.1	2066845	.	C	CCACTAGG	100	*	TYPE=INSERT
CM032587.1	2066875	.	A	T	100	*	TYPE=SUBSTITUTE
CM032587.1	2066886	.	T	A	100	*	TYPE=SUBSTITUTE
CM032587.1	2066887	.	C	A	100	*	TYPE=SUBSTITUTE
CM032587.1	2066894	.	T	TCC	100	*	TYPE=INSERT
CM032587.1	2066896	.	G	C	100	*	TYPE=SUBSTITUTE
CM032587.1	2066897	.	C	G	100	*	TYPE=SUBSTITUTE
CM032587.1	2066898	.	C	G	100	*	TYPE=SUBSTITUTE
CM032587.1	2066902	.	C	G	100	*	TYPE=SUBSTITUTE
CM032587.1	2066903	.	T	G	100	*	TYPE=SUBSTITUTE
CM032587.1	2066904	.	C	G	100	*	TYPE=SUBSTITUTE
CM032587.1	2066905	.	G	T	100	*	TYPE=SUBSTITUTE
CM032587.1	2066907	.	C	A	100	*	TYPE=SUBSTITUTE
CM032587.1	2066908	.	A	AAGC	100	*	TYPE=INSERT
CM032587.1	2066914	.	T	C	100	*	TYPE=SUBSTITUTE
CM032587.1	2066925	.	G	A	100	*	TYPE=SUBSTITUTE
CM032587.1	2066940	.	G	C	100	*	TYPE=SUBSTITUTE
CM032587.1	2066948	.	A	AGC	100	*	TYPE=INSERT
CM032587.1	2066958	.	G	A	100	*	TYPE=SUBSTITUTE
CM032587.1	2066962	.	A	G	100	*	TYPE=SUBSTITUTE
CM032587.1	2067028	.	G	A	100	*	TYPE=SUBSTITUTE
CM032587.1	2067030	.	T	A	100	*	TYPE=SUBSTITUTE
CM032587.1	2067055	.	A	G	100	*	TYPE=SUBSTITUTE
CM032587.1	2067063	.	G	T	100	*	TYPE=SUBSTITUTE
CM032587.1	2067108	.	C	G	100	*	TYPE=SUBSTITUTE

CM032587.1	2067120	.	T	C	100	*	TYPE=SUBSTITUTE
CM032587.1	2067123	.	T	C	100	*	TYPE=SUBSTITUTE
CM032587.1	2067150	.	A	G	100	*	TYPE=SUBSTITUTE
CM032587.1	2067153	.	A	C	100	*	TYPE=SUBSTITUTE
CM032587.1	2067159	.	C	G	100	*	TYPE=SUBSTITUTE
CM032587.1	2067186	.	C	T	100	*	TYPE=SUBSTITUTE
CM032587.1	2067224	.	A	C	100	*	TYPE=SUBSTITUTE
CM032587.1	2067226	.	A	G	100	*	TYPE=SUBSTITUTE
CM032587.1	2067243	.	T	a	100	*	TYPE=SUBSTITUTE
CM032587.1	2067255	.	G	T	100	*	TYPE=SUBSTITUTE
CM032587.1	2067258	.	G	A	100	*	TYPE=SUBSTITUTE
CM032587.1	2067276	.	T	G	100	*	TYPE=SUBSTITUTE
CM032587.1	2067282	.	C	T	100	*	TYPE=SUBSTITUTE
CM032587.1	2067285	.	G	A	100	*	TYPE=SUBSTITUTE
CM032587.1	2067288	.	G	A	100	*	TYPE=SUBSTITUTE
CM032587.1	2067330	.	A	G	100	*	TYPE=SUBSTITUTE
CM032587.1	2067333	.	C	T	100	*	TYPE=SUBSTITUTE
CM032587.1	2067351	.	A	G	100	*	TYPE=SUBSTITUTE
CM032587.1	2067369	.	A	C	100	*	TYPE=SUBSTITUTE
CM032587.1	2067403	.	T	TT	100	*	TYPE=INSERT
CM032587.1	2067411	.	T	C	100	*	TYPE=SUBSTITUTE
CM032587.1	2067414	.	G	A	100	*	TYPE=SUBSTITUTE
CM032587.1	2067426	.	T	A	100	*	TYPE=SUBSTITUTE
CM032587.1	2067428	.	T	G	100	*	TYPE=SUBSTITUTE
CM032587.1	2067429	.	A	C	100	*	TYPE=SUBSTITUTE
CM032587.1	2067440	.	T	C	100	*	TYPE=SUBSTITUTE
CM032587.1	2067445	.	CTAG	C	100	*	TYPE=DELETE
CM032587.1	2067449	.	T	A	100	*	TYPE=SUBSTITUTE
CM032587.1	2067455	.	A	G	100	*	TYPE=SUBSTITUTE
CM032587.1	2067499	.	A	G	100	*	TYPE=SUBSTITUTE
CM032587.1	2067509	.	T	C	100	*	TYPE=SUBSTITUTE
CM032587.1	2067521	.	C	T	100	*	TYPE=SUBSTITUTE
CM032587.1	2067533	.	C	G	100	*	TYPE=SUBSTITUTE
CM032587.1	2067563	.	T	C	100	*	TYPE=SUBSTITUTE
CM032587.1	2067635	.	C	T	100	*	TYPE=SUBSTITUTE
CM032587.1	2067641	.	A	G	100	*	TYPE=SUBSTITUTE
CM032587.1	2067650	.	A	G	100	*	TYPE=SUBSTITUTE
CM032587.1	2067662	.	T	C	100	*	TYPE=SUBSTITUTE
CM032587.1	2067672	.	T	C	100	*	TYPE=SUBSTITUTE
CM032587.1	2067683	.	G	A	100	*	TYPE=SUBSTITUTE
CM032587.1	2067743	.	T	C	100	*	TYPE=SUBSTITUTE
CM032587.1	2067752	.	T	C	100	*	TYPE=SUBSTITUTE
CM032587.1	2067758	.	A	G	100	*	TYPE=SUBSTITUTE
CM032587.1	2067765	.	A	C	100	*	TYPE=SUBSTITUTE
CM032587.1	2067766	.	A	G	100	*	TYPE=SUBSTITUTE
CM032587.1	2067767	.	A	G	100	*	TYPE=SUBSTITUTE
CM032587.1	2067776	.	C	T	100	*	TYPE=SUBSTITUTE
CM032587.1	2067851	.	C	T	100	*	TYPE=SUBSTITUTE
CM032587.1	2067852	.	G	A	100	*	TYPE=SUBSTITUTE
CM032587.1	2067861	.	A	G	100	*	TYPE=SUBSTITUTE
CM032587.1	2067886	.	A	G	100	*	TYPE=SUBSTITUTE
CM032587.1	2067896	.	T	A	100	*	TYPE=SUBSTITUTE
CM032587.1	2067899	.	T	C	100	*	TYPE=SUBSTITUTE
CM032587.1	2067906	.	G	A	100	*	TYPE=SUBSTITUTE
CM032587.1	2067944	.	G	A	100	*	TYPE=SUBSTITUTE
CM032587.1	2067965	.	C	T	100	*	TYPE=SUBSTITUTE
CM032587.1	2068028	.	A	G	100	*	TYPE=SUBSTITUTE
CM032587.1	2068058	.	A	G	100	*	TYPE=SUBSTITUTE
CM032587.1	2068076	.	C	T	100	*	TYPE=SUBSTITUTE
CM032587.1	2068089	.	G	A	100	*	TYPE=SUBSTITUTE
CM032587.1	2068154	.	G	A	100	*	TYPE=SUBSTITUTE
CM032587.1	2068172	.	C	T	100	*	TYPE=SUBSTITUTE
CM032587.1	2068180	.	G	A	100	*	TYPE=SUBSTITUTE
CM032587.1	2068184	.	C	T	100	*	TYPE=SUBSTITUTE

CM032587.1	2068199	.	C	T	100	*	TYPE=SUBSTITUTE
CM032587.1	2068241	.	G	A	100	*	TYPE=SUBSTITUTE
CM032587.1	2068244	.	T	C	100	*	TYPE=SUBSTITUTE
CM032587.1	2068274	.	G	A	100	*	TYPE=SUBSTITUTE
CM032587.1	2068277	.	A	G	100	*	TYPE=SUBSTITUTE
CM032587.1	2068296	.	T	C	100	*	TYPE=SUBSTITUTE
CM032587.1	2068302	.	A	G	100	*	TYPE=SUBSTITUTE
CM032587.1	2068304	.	A	C	100	*	TYPE=SUBSTITUTE
CM032587.1	2068311	.	A	G	100	*	TYPE=SUBSTITUTE
CM032587.1	2068352	.	A	c	100	*	TYPE=SUBSTITUTE
CM032587.1	2068388	.	G	T	100	*	TYPE=SUBSTITUTE
CM032587.1	2068406	.	G	A	100	*	TYPE=SUBSTITUTE
CM032587.1	2068430	.	T	C	100	*	TYPE=SUBSTITUTE
CM032587.1	2068439	.	G	C	100	*	TYPE=SUBSTITUTE
CM032587.1	2068463	.	T	C	100	*	TYPE=SUBSTITUTE
CM032587.1	2068484	.	T	G	100	*	TYPE=SUBSTITUTE
CM032587.1	2068487	.	C	T	100	*	TYPE=SUBSTITUTE
CM032587.1	2068505	.	A	C	100	*	TYPE=SUBSTITUTE
CM032587.1	2068547	.	A	G	100	*	TYPE=SUBSTITUTE
CM032587.1	2068559	.	A	G	100	*	TYPE=SUBSTITUTE
CM032587.1	2068575	.	C	G	100	*	TYPE=SUBSTITUTE
CM032587.1	2068637	.	G	A	100	*	TYPE=SUBSTITUTE
CM032587.1	2068654	.	G	A	100	*	TYPE=SUBSTITUTE
CM032587.1	2068666	.	A	T	100	*	TYPE=SUBSTITUTE
CM032587.1	2068668	.	G	GT	100	*	TYPE=INSERT
CM032587.1	2068674	.	A	G	100	*	TYPE=SUBSTITUTE
CM032587.1	2068676	.	A	T	100	*	TYPE=SUBSTITUTE
CM032587.1	2068685	.	G	A	100	*	TYPE=SUBSTITUTE
CM032587.1	2068686	.	A	G	100	*	TYPE=SUBSTITUTE
CM032587.1	2068690	.	T	C	100	*	TYPE=SUBSTITUTE
CM032587.1	2068698	.	C	T	100	*	TYPE=SUBSTITUTE
CM032587.1	2068699	.	T	A	100	*	TYPE=SUBSTITUTE
CM032587.1	2068700	.	G	C	100	*	TYPE=SUBSTITUTE
CM032587.1	2068702	.	T	A	100	*	TYPE=SUBSTITUTE
CM032587.1	2068704	.	G	A	100	*	TYPE=SUBSTITUTE
CM032587.1	2068705	.	A	T	100	*	TYPE=SUBSTITUTE
CM032587.1	2068707	.	A	G	100	*	TYPE=SUBSTITUTE
CM032587.1	2068778	.	C	T	100	*	TYPE=SUBSTITUTE
CM032587.1	2068789	.	A	G	100	*	TYPE=SUBSTITUTE
CM032587.1	2068791	.	G	T	100	*	TYPE=SUBSTITUTE
CM032587.1	2068806	.	A	T	100	*	TYPE=SUBSTITUTE
CM032587.1	2068824	.	A	T	100	*	TYPE=SUBSTITUTE
CM032587.1	2068836	.	C	T	100	*	TYPE=SUBSTITUTE
CM032587.1	2068847	.	T	C	100	*	TYPE=SUBSTITUTE
CM032587.1	2068849	.	C	T	100	*	TYPE=SUBSTITUTE
CM032587.1	2068867	.	A	G	100	*	TYPE=SUBSTITUTE
CM032587.1	2068879	.	C	T	100	*	TYPE=SUBSTITUTE
CM032587.1	2068928	.	TC	T	100	*	TYPE=DELETE
CM032587.1	2068943	.	C	T	100	*	TYPE=SUBSTITUTE
CM032587.1	2068944	.	G	C	100	*	TYPE=SUBSTITUTE
CM032587.1	2068973	.	T	C	100	*	TYPE=SUBSTITUTE
CM032587.1	2068986	.	G	T	100	*	TYPE=SUBSTITUTE
CM032587.1	2068987	.	A	G	100	*	TYPE=SUBSTITUTE
CM032587.1	2069004	.	C	T	100	*	TYPE=SUBSTITUTE
CM032587.1	2069011	.	T	C	100	*	TYPE=SUBSTITUTE
CM032587.1	2069014	.	T	G	100	*	TYPE=SUBSTITUTE
CM032587.1	2069016	.	G	C	100	*	TYPE=SUBSTITUTE
CM032587.1	2069021	.	A	G	100	*	TYPE=SUBSTITUTE
CM032587.1	2069065	.	T	C	100	*	TYPE=SUBSTITUTE
CM032587.1	2069069	.	G	GC	100	*	TYPE=INSERT
CM032587.1	2069079	.	CCAAAAAAAAA	C	100	*	TYPE=DELETE
CM032587.1	2069093	.	A	C	100	*	TYPE=SUBSTITUTE
CM032587.1	2069106	.	T	G	100	*	TYPE=SUBSTITUTE
CM032587.1	2069108	.	T	C	100	*	TYPE=SUBSTITUTE

CM032587.1	2069119	.	C	T	100	*	TYPE=SUBSTITUTE
CM032587.1	2069128	.	A	G	100	*	TYPE=SUBSTITUTE
CM032587.1	2069136	.	T	C	100	*	TYPE=SUBSTITUTE
CM032587.1	2069144	.	C	T	100	*	TYPE=SUBSTITUTE
CM032587.1	2069157	.	T	C	100	*	TYPE=SUBSTITUTE
CM032587.1	2069169	.	T	C	100	*	TYPE=SUBSTITUTE
CM032587.1	2069173	.	T	A	100	*	TYPE=SUBSTITUTE
CM032587.1	2069208	.	T	c	100	*	TYPE=SUBSTITUTE
CM032587.1	2069214	.	G	a	100	*	TYPE=SUBSTITUTE
CM032587.1	2069223	.	T	C	100	*	TYPE=SUBSTITUTE
CM032587.1	2069226	.	G	C	100	*	TYPE=SUBSTITUTE
CM032587.1	2069233	.	A	C	100	*	TYPE=SUBSTITUTE
CM032587.1	2069253	.	T	C	100	*	TYPE=SUBSTITUTE
CM032587.1	2069274	.	T	a	100	*	TYPE=SUBSTITUTE
CM032587.1	2069277	.	T	c	100	*	TYPE=SUBSTITUTE
CM032587.1	2069295	.	C	t	100	*	TYPE=SUBSTITUTE
CM032587.1	2069301	.	A	T	100	*	TYPE=SUBSTITUTE
CM032587.1	2069302	.	A	T	100	*	TYPE=SUBSTITUTE
CM032587.1	2069319	.	C	G	100	*	TYPE=SUBSTITUTE
CM032587.1	2069328	.	T	A	100	*	TYPE=SUBSTITUTE
CM032587.1	2069350	.	A	G	100	*	TYPE=SUBSTITUTE
CM032587.1	2069355	.	T	C	100	*	TYPE=SUBSTITUTE
CM032587.1	2069358	.	G	A	100	*	TYPE=SUBSTITUTE
CM032587.1	2069361	.	G	A	100	*	TYPE=SUBSTITUTE
CM032587.1	2069363	.	T	C	100	*	TYPE=SUBSTITUTE
CM032587.1	2069377	.	C	T	100	*	TYPE=SUBSTITUTE
CM032587.1	2069380	.	C	T	100	*	TYPE=SUBSTITUTE
CM032587.1	2069385	.	C	T	100	*	TYPE=SUBSTITUTE
CM032587.1	2069400	.	C	T	100	*	TYPE=SUBSTITUTE
CM032587.1	2069403	.	G	A	100	*	TYPE=SUBSTITUTE
CM032587.1	2069412	.	T	C	100	*	TYPE=SUBSTITUTE
CM032587.1	2069436	.	T	G	100	*	TYPE=SUBSTITUTE
CM032587.1	2069477	.	A	G	100	*	TYPE=SUBSTITUTE
CM032587.1	2069493	.	T	G	100	*	TYPE=SUBSTITUTE
CM032587.1	2069505	.	G	A	100	*	TYPE=SUBSTITUTE
CM032587.1	2069521	.	A	G	100	*	TYPE=SUBSTITUTE
CM032587.1	2069526	.	C	T	100	*	TYPE=SUBSTITUTE
CM032587.1	2069541	.	C	T	100	*	TYPE=SUBSTITUTE
CM032587.1	2069553	.	A	C	100	*	TYPE=SUBSTITUTE
CM032587.1	2069559	.	A	g	100	*	TYPE=SUBSTITUTE
CM032587.1	2069580	.	C	G	100	*	TYPE=SUBSTITUTE
CM032587.1	2069598	.	A	G	100	*	TYPE=SUBSTITUTE
CM032587.1	2932792	.	TT	T	100	*	TYPE=DELETE
CM032587.1	2932807	.	C	T	100	*	TYPE=SUBSTITUTE
CM032587.1	2932818	.	C	T	100	*	TYPE=SUBSTITUTE
CM032587.1	2932857	.	A	G	100	*	TYPE=SUBSTITUTE
CM032587.1	2932885	.	G	t	100	*	TYPE=SUBSTITUTE
CM032587.1	2932965	.	C	T	100	*	TYPE=SUBSTITUTE
CM032587.1	2932991	.	T	g	100	*	TYPE=SUBSTITUTE
CM032587.1	3498561	.	A	t	100	*	TYPE=SUBSTITUTE
CM032587.1	3498563	.	G	a	100	*	TYPE=SUBSTITUTE
CM032587.1	3498598	.	A	t	100	*	TYPE=SUBSTITUTE
CM032587.1	3498627	.	C	t	100	*	TYPE=SUBSTITUTE
CM032587.1	3498631	.	AATAATACTTTTATTT	A	100	*	TYPE=DELETE
CM032587.1	3498653	.	C	t	100	*	TYPE=SUBSTITUTE
CM032587.1	3498659	.	C	t	100	*	TYPE=SUBSTITUTE
CM032587.1	3498667	.	C	t	100	*	TYPE=SUBSTITUTE
CM032587.1	3498677	.	G	a	100	*	TYPE=SUBSTITUTE
CM032587.1	3498678	.	C	t	100	*	TYPE=SUBSTITUTE
CM032587.1	3498683	.	G	a	100	*	TYPE=SUBSTITUTE
CM032587.1	3498700	.	T	c	100	*	TYPE=SUBSTITUTE
CM032587.1	3498702	.	TC	T	100	*	TYPE=DELETE
CM032587.1	3498722	.	C	t	100	*	TYPE=SUBSTITUTE
CM032587.1	3498732	.	G	a	100	*	TYPE=SUBSTITUTE

CM032587.1	3498742	.	C	t	100	*	TYPE=SUBSTITUTE
CM032587.1	3498773	.	T	c	100	*	TYPE=SUBSTITUTE
CM032587.1	3498781	.	G	a	100	*	TYPE=SUBSTITUTE
CM032587.1	3498782	.	C	t	100	*	TYPE=SUBSTITUTE
CM032587.1	3498785	.	T	a	100	*	TYPE=SUBSTITUTE
CM032587.1	3498793	.	T	a	100	*	TYPE=SUBSTITUTE
CM032587.1	3498808	.	TA	T	100	*	TYPE=DELETE
CM032587.1	3498811	.	T	g	100	*	TYPE=SUBSTITUTE
CM032587.1	3498845	.	TC	T	100	*	TYPE=DELETE
CM032587.1	3498847	.	C	t	100	*	TYPE=SUBSTITUTE
CM032587.1	3498853	.	C	t	100	*	TYPE=SUBSTITUTE
CM032587.1	3498857	.	A	g	100	*	TYPE=SUBSTITUTE
CM032587.1	3498860	.	t	ta	100	*	TYPE=INSERT
CM032587.1	3498862	.	T	a	100	*	TYPE=SUBSTITUTE
CM032587.1	3498869	.	A	c	100	*	TYPE=SUBSTITUTE
CM032587.1	3498870	.	C	t	100	*	TYPE=SUBSTITUTE
CM032587.1	3498878	.	A	t	100	*	TYPE=SUBSTITUTE
CM032587.1	3498879	.	G	a	100	*	TYPE=SUBSTITUTE
CM032587.1	3498881	.	AG	A	100	*	TYPE=DELETE
CM032587.1	3498883	.	T	a	100	*	TYPE=SUBSTITUTE
CM032587.1	3498889	.	T	c	100	*	TYPE=SUBSTITUTE
CM032587.1	3498897	.	T	c	100	*	TYPE=SUBSTITUTE
CM032587.1	3498906	.	C	t	100	*	TYPE=SUBSTITUTE
CM032587.1	3498914	.	C	t	100	*	TYPE=SUBSTITUTE
CM032587.1	3498939	.	T	a	100	*	TYPE=SUBSTITUTE
CM032587.1	3498948	.	A	t	100	*	TYPE=SUBSTITUTE
CM032587.1	3498951	.	TC	T	100	*	TYPE=DELETE
CM032587.1	3498957	.	G	a	100	*	TYPE=SUBSTITUTE
CM032587.1	3498965	.	C	t	100	*	TYPE=SUBSTITUTE
CM032587.1	3498978	.	T	a	100	*	TYPE=SUBSTITUTE
CM032587.1	3498988	.	T	a	100	*	TYPE=SUBSTITUTE
CM032587.1	3499008	.	A	t	100	*	TYPE=SUBSTITUTE
CM032587.1	3499009	.	T	a	100	*	TYPE=SUBSTITUTE
CM032587.1	3499027	.	T	c	100	*	TYPE=SUBSTITUTE
CM032587.1	3499034	.	C	t	100	*	TYPE=SUBSTITUTE
CM032587.1	3499036	.	T	a	100	*	TYPE=SUBSTITUTE
CM032587.1	3499049	.	C	t	100	*	TYPE=SUBSTITUTE
CM032587.1	3499082	.	A	g	100	*	TYPE=SUBSTITUTE
CM032587.1	3499105	.	TT	T	100	*	TYPE=DELETE
CM032587.1	3499107	.	T	a	100	*	TYPE=SUBSTITUTE
CM032587.1	3499108	.	T	a	100	*	TYPE=SUBSTITUTE
CM032587.1	3499121	.	A	t	100	*	TYPE=SUBSTITUTE
CM032587.1	3499135	.	A	t	100	*	TYPE=SUBSTITUTE
CM032587.1	3499136	.	G	a	100	*	TYPE=SUBSTITUTE
CM032587.1	3499157	.	TA	T	100	*	TYPE=DELETE
CM032587.1	3499177	.	G	a	100	*	TYPE=SUBSTITUTE
CM032587.1	3499189	.	A	g	100	*	TYPE=SUBSTITUTE
CM032587.1	3499208	.	C	t	100	*	TYPE=SUBSTITUTE
CM032587.1	3499213	.	G	a	100	*	TYPE=SUBSTITUTE
CM032587.1	3499225	.	T	a	100	*	TYPE=SUBSTITUTE
CM032587.1	3499239	.	T	a	100	*	TYPE=SUBSTITUTE
CM032587.1	3499248	.	AT	A	100	*	TYPE=DELETE
CM032587.1	3499270	.	C	t	100	*	TYPE=SUBSTITUTE
CM032587.1	3499275	.	G	a	100	*	TYPE=SUBSTITUTE
CM032587.1	3499276	.	G	a	100	*	TYPE=SUBSTITUTE
CM032587.1	3499295	.	A	t	100	*	TYPE=SUBSTITUTE
CM032587.1	3499296	.	G	a	100	*	TYPE=SUBSTITUTE
CM032587.1	3499304	.	C	t	100	*	TYPE=SUBSTITUTE
CM032587.1	3499318	.	C	t	100	*	TYPE=SUBSTITUTE
CM032587.1	3499323	.	T	a	100	*	TYPE=SUBSTITUTE
CM032587.1	3499359	.	A	g	100	*	TYPE=SUBSTITUTE
CM032587.1	3499391	.	A	g	100	*	TYPE=SUBSTITUTE
CM032587.1	3499410	.	C	t	100	*	TYPE=SUBSTITUTE
CM032587.1	3499428	.	A	g	100	*	TYPE=SUBSTITUTE

CM032587.1	3499435	.	A	t	100	*	TYPE=SUBSTITUTE
CM032587.1	3546149	.	a	aaa	100	*	TYPE=INSERT
CM032587.1	3546160	.	t	ta	100	*	TYPE=INSERT
CM032587.1	3546167	.	T	a	100	*	TYPE=SUBSTITUTE
CM032587.1	3546185	.	ACT	A	100	*	TYPE=DELETE
CM032587.1	3546191	.	A	t	100	*	TYPE=SUBSTITUTE
CM032587.1	3546214	.	T	a	100	*	TYPE=SUBSTITUTE
CM032587.1	3546224	.	t	ta	100	*	TYPE=INSERT
CM032587.1	3546228	.	C	t	100	*	TYPE=SUBSTITUTE
CM032587.1	3546237	.	C	a	100	*	TYPE=SUBSTITUTE
CM032587.1	3546241	.	A	t	100	*	TYPE=SUBSTITUTE
CM032587.1	3546242	.	G	a	100	*	TYPE=SUBSTITUTE
CM032587.1	3546273	.	A	t	100	*	TYPE=SUBSTITUTE
CM032587.1	3546276	.	A	t	100	*	TYPE=SUBSTITUTE
CM032587.1	3546282	.	A	t	100	*	TYPE=SUBSTITUTE
CM032587.1	3546283	.	G	t	100	*	TYPE=SUBSTITUTE
CM032587.1	3546285	.	T	a	100	*	TYPE=SUBSTITUTE
CM032587.1	3546290	.	a	at	100	*	TYPE=INSERT
CM032587.1	3546294	.	T	a	100	*	TYPE=SUBSTITUTE
CM032587.1	3546302	.	A	t	100	*	TYPE=SUBSTITUTE
CM032587.1	3546306	.	TA	T	100	*	TYPE=DELETE
CM032587.1	3546315	.	A	t	100	*	TYPE=SUBSTITUTE
CM032587.1	3546321	.	G	a	100	*	TYPE=SUBSTITUTE
CM032587.1	3546329	.	C	t	100	*	TYPE=SUBSTITUTE
CM032587.1	3546332	.	A	t	100	*	TYPE=SUBSTITUTE
CM032587.1	3546333	.	AG	A	100	*	TYPE=DELETE
CM032587.1	3546342	.	T	a	100	*	TYPE=SUBSTITUTE
CM032587.1	3546360	.	AG	A	100	*	TYPE=DELETE
CM032587.1	3546366	.	C	a	100	*	TYPE=SUBSTITUTE
CM032587.1	3546368	.	T	a	100	*	TYPE=SUBSTITUTE
CM032587.1	3546369	.	AGG	A	100	*	TYPE=DELETE
CM032587.1	3546374	.	T	a	100	*	TYPE=SUBSTITUTE
CM032587.1	3546379	.	A	t	100	*	TYPE=SUBSTITUTE
CM032587.1	3546388	.	T	a	100	*	TYPE=SUBSTITUTE
CM032587.1	3546396	.	A	t	100	*	TYPE=SUBSTITUTE
CM032587.1	3546398	.	T	a	100	*	TYPE=SUBSTITUTE
CM032587.1	3546403	.	A	t	100	*	TYPE=SUBSTITUTE
CM032587.1	3546409	.	a	at	100	*	TYPE=INSERT
CM032587.1	3546413	.	G	a	100	*	TYPE=SUBSTITUTE
CM032587.1	3546425	.	G	a	100	*	TYPE=SUBSTITUTE
CM032587.1	3546439	.	C	t	100	*	TYPE=SUBSTITUTE
CM032587.1	3546445	.	C	t	100	*	TYPE=SUBSTITUTE
CM032587.1	3546448	.	TAA	T	100	*	TYPE=DELETE
CM032587.1	3546452	.	A	c	100	*	TYPE=SUBSTITUTE
CM032587.1	3546453	.	A	t	100	*	TYPE=SUBSTITUTE
CM032587.1	3546454	.	A	t	100	*	TYPE=SUBSTITUTE
CM032587.1	3546457	.	G	t	100	*	TYPE=SUBSTITUTE
CM032587.1	3546460	.	A	t	100	*	TYPE=SUBSTITUTE
CM032587.1	3546465	.	C	a	100	*	TYPE=SUBSTITUTE
CM032587.1	3546467	.	t	ta	100	*	TYPE=INSERT
CM032587.1	3546484	.	AT	A	100	*	TYPE=DELETE
CM032587.1	3546518	.	C	t	100	*	TYPE=SUBSTITUTE
CM032587.1	3546546	.	G	t	100	*	TYPE=SUBSTITUTE
CM032587.1	3546552	.	C	t	100	*	TYPE=SUBSTITUTE
CM032587.1	3546569	.	G	a	100	*	TYPE=SUBSTITUTE
CM032587.1	3546601	.	G	a	100	*	TYPE=SUBSTITUTE
CM032587.1	3546641	.	G	a	100	*	TYPE=SUBSTITUTE
CM032587.1	3546642	.	C	t	100	*	TYPE=SUBSTITUTE
CM032587.1	3546687	.	G	a	100	*	TYPE=SUBSTITUTE
CM032587.1	3546719	.	G	t	100	*	TYPE=SUBSTITUTE
CM032587.1	3546721	.	T	c	100	*	TYPE=SUBSTITUTE
CM032587.1	3546743	.	G	a	100	*	TYPE=SUBSTITUTE
CM032587.1	3546752	.	TA	T	100	*	TYPE=DELETE
CM032587.1	3546783	.	G	a	100	*	TYPE=SUBSTITUTE

CM032587.1	3546811	.	G	a	100	*	TYPE=SUBSTITUTE
CM032587.1	3546822	.	AC	A	100	*	TYPE=DELETE
CM032587.1	3546905	.	GCT	G	100	*	TYPE=DELETE
CM032587.1	3546941	.	A	g	100	*	TYPE=SUBSTITUTE
CM032587.1	3546987	.	AAAA	A	100	*	TYPE=DELETE
CM032587.1	3546997	.	AT	A	100	*	TYPE=DELETE
CM032587.1	3547022	.	C	t	100	*	TYPE=SUBSTITUTE
CM032587.1	3547048	.	G	a	100	*	TYPE=SUBSTITUTE
CM032587.1	3547050	.	C	t	100	*	TYPE=SUBSTITUTE
CM032587.1	3547054	.	G	a	100	*	TYPE=SUBSTITUTE
CM032587.1	3547088	.	G	a	100	*	TYPE=SUBSTITUTE
CM032587.1	3547089	.	C	t	100	*	TYPE=SUBSTITUTE
CM032587.1	3547108	.	C	t	100	*	TYPE=SUBSTITUTE
CM032587.1	3547127	.	T	c	100	*	TYPE=SUBSTITUTE
CM032587.1	3547178	.	t	taaataa	100	*	TYPE=INSERT
CM032587.1	3547180	.	T	a	100	*	TYPE=SUBSTITUTE
CM032587.1	3547197	.	G	a	100	*	TYPE=SUBSTITUTE
CM032587.1	3547206	.	G	a	100	*	TYPE=SUBSTITUTE
CM032587.1	3547226	.	C	a	100	*	TYPE=SUBSTITUTE
CM032587.1	3547228	.	C	t	100	*	TYPE=SUBSTITUTE
CM032587.1	3547237	.	G	a	100	*	TYPE=SUBSTITUTE
CM032587.1	3547249	.	G	a	100	*	TYPE=SUBSTITUTE
CM032587.1	3547283	.	G	a	100	*	TYPE=SUBSTITUTE
CM032587.1	3547312	.	C	t	100	*	TYPE=SUBSTITUTE
CM032587.1	3547317	.	G	a	100	*	TYPE=SUBSTITUTE
CM032587.1	3547319	.	GT	G	100	*	TYPE=DELETE
CM032587.1	3547360	.	G	a	100	*	TYPE=SUBSTITUTE
CM032587.1	3547429	.	C	t	100	*	TYPE=SUBSTITUTE
CM032587.1	3547444	.	C	t	100	*	TYPE=SUBSTITUTE
CM032587.1	3547457	.	A	g	100	*	TYPE=SUBSTITUTE
CM032587.1	3547486	.	T	c	100	*	TYPE=SUBSTITUTE
CM032587.1	3547500	.	G	a	100	*	TYPE=SUBSTITUTE
CM032587.1	3547518	.	G	a	100	*	TYPE=SUBSTITUTE
CM032587.1	3547552	.	T	c	100	*	TYPE=SUBSTITUTE
CM032587.1	3547584	.	C	t	100	*	TYPE=SUBSTITUTE
CM032587.1	3547627	.	C	t	100	*	TYPE=SUBSTITUTE
CM032587.1	3547638	.	C	t	100	*	TYPE=SUBSTITUTE
CM032587.1	3547655	.	G	a	100	*	TYPE=SUBSTITUTE
CM032587.1	3547706	.	T	c	100	*	TYPE=SUBSTITUTE
CM032587.1	3547771	.	A	g	100	*	TYPE=SUBSTITUTE
CM032587.1	3547791	.	C	t	100	*	TYPE=SUBSTITUTE
CM032587.1	3547792	.	C	t	100	*	TYPE=SUBSTITUTE
CM032587.1	3547796	.	T	a	100	*	TYPE=SUBSTITUTE
CM032587.1	3547802	.	C	t	100	*	TYPE=SUBSTITUTE
CM032587.1	3547835	.	AT	A	100	*	TYPE=DELETE
CM032587.1	3547847	.	C	t	100	*	TYPE=SUBSTITUTE
CM032587.1	3547852	.	G	a	100	*	TYPE=SUBSTITUTE
CM032587.1	3547855	.	T	c	100	*	TYPE=SUBSTITUTE
CM032587.1	3547871	.	G	a	100	*	TYPE=SUBSTITUTE
CM032587.1	3547882	.	G	a	100	*	TYPE=SUBSTITUTE
CM032587.1	3547900	.	C	t	100	*	TYPE=SUBSTITUTE
CM032587.1	3547973	.	T	c	100	*	TYPE=SUBSTITUTE
CM032587.1	3547989	.	C	t	100	*	TYPE=SUBSTITUTE
CM032587.1	3548000	.	TA	T	100	*	TYPE=DELETE
CM032587.1	3548009	.	T	c	100	*	TYPE=SUBSTITUTE
CM032587.1	3548017	.	A	t	100	*	TYPE=SUBSTITUTE
CM032587.1	3548023	.	G	a	100	*	TYPE=SUBSTITUTE
CM032587.1	3548058	.	G	a	100	*	TYPE=SUBSTITUTE
CM032587.1	3548081	.	G	a	100	*	TYPE=SUBSTITUTE
CM032587.1	3548082	.	C	t	100	*	TYPE=SUBSTITUTE
CM032587.1	3548090	.	G	a	100	*	TYPE=SUBSTITUTE
CM032587.1	3548102	.	G	a	100	*	TYPE=SUBSTITUTE
CM032587.1	3548113	.	G	a	100	*	TYPE=SUBSTITUTE
CM032587.1	3548158	.	G	a	100	*	TYPE=SUBSTITUTE

CM032587.1	3548177	.	A	g	100	*	TYPE=SUBSTITUTE
CM032587.1	3548185	.	A	g	100	*	TYPE=SUBSTITUTE
CM032587.1	3548195	.	TAAA	T	100	*	TYPE=DELETE
CM032587.1	3548208	.	C	t	100	*	TYPE=SUBSTITUTE
CM032587.1	3548212	.	T	c	100	*	TYPE=SUBSTITUTE
CM032587.1	3548232	.	G	a	100	*	TYPE=SUBSTITUTE
CM032587.1	3548247	.	G	a	100	*	TYPE=SUBSTITUTE
CM032587.1	3548248	.	C	t	100	*	TYPE=SUBSTITUTE
CM032587.1	3548252	.	A	g	100	*	TYPE=SUBSTITUTE
CM032587.1	3548310	.	G	a	100	*	TYPE=SUBSTITUTE
CM032587.1	3548311	.	C	t	100	*	TYPE=SUBSTITUTE
CM032587.1	3548334	.	T	c	100	*	TYPE=SUBSTITUTE
CM032587.1	3548355	.	a	aat	100	*	TYPE=INSERT
CM032587.1	3548375	.	A	g	100	*	TYPE=SUBSTITUTE
CM032587.1	3548379	.	A	g	100	*	TYPE=SUBSTITUTE
CM032587.1	3548411	.	G	a	100	*	TYPE=SUBSTITUTE
CM032587.1	3548429	.	AAATTAAAAT	A	100	*	TYPE=DELETE
CM032587.1	3548487	.	G	a	100	*	TYPE=SUBSTITUTE
CM032587.1	3548493	.	g	gta	100	*	TYPE=INSERT
CM032587.1	3548494	.	C	a	100	*	TYPE=SUBSTITUTE
CM032587.1	3548497	.	a	att	100	*	TYPE=INSERT
CM032587.1	3548499	.	G	a	100	*	TYPE=SUBSTITUTE
CM032587.1	3548528	.	G	a	100	*	TYPE=SUBSTITUTE
CM032587.1	3548529	.	C	t	100	*	TYPE=SUBSTITUTE
CM032587.1	3548538	.	C	t	100	*	TYPE=SUBSTITUTE
CM032587.1	3548541	.	C	t	100	*	TYPE=SUBSTITUTE
CM032587.1	3548547	.	C	t	100	*	TYPE=SUBSTITUTE
CM032587.1	3548554	.	G	a	100	*	TYPE=SUBSTITUTE
CM032587.1	3548565	.	G	a	100	*	TYPE=SUBSTITUTE
CM032587.1	3548568	.	ATT	A	100	*	TYPE=DELETE
CM032587.1	3889215	.	T	TGGGCTTGACATAT	100	*	TYPE=INSERT
CM032587.1	3889221	.	T	G	100	*	TYPE=SUBSTITUTE
CM032587.1	3889230	.	CTA	C	100	*	TYPE=DELETE
CM032587.1	3889287	.	G	A	100	*	TYPE=SUBSTITUTE
CM032587.1	3889313	.	A	T	100	*	TYPE=SUBSTITUTE
CM032587.1	3889320	.	G	A	100	*	TYPE=SUBSTITUTE
CM032587.1	3889323	.	C	T	100	*	TYPE=SUBSTITUTE
CM032587.1	3889332	.	G	A	100	*	TYPE=SUBSTITUTE
CM032587.1	3889335	.	C	T	100	*	TYPE=SUBSTITUTE
CM032587.1	3889341	.	C	T	100	*	TYPE=SUBSTITUTE
CM032587.1	3889356	.	A	G	100	*	TYPE=SUBSTITUTE
CM032587.1	3889368	.	G	T	100	*	TYPE=SUBSTITUTE
CM032587.1	3889370	.	G	A	100	*	TYPE=SUBSTITUTE
CM032587.1	3889374	.	C	G	100	*	TYPE=SUBSTITUTE
CM032587.1	3889398	.	G	A	100	*	TYPE=SUBSTITUTE
CM032587.1	3889404	.	A	G	100	*	TYPE=SUBSTITUTE
CM032587.1	3889419	.	G	A	100	*	TYPE=SUBSTITUTE
CM032587.1	3889425	.	T	C	100	*	TYPE=SUBSTITUTE
CM032587.1	3889452	.	AG	A	100	*	TYPE=DELETE
CM032587.1	3889455	.	T	A	100	*	TYPE=SUBSTITUTE
CM032587.1	3889464	.	A	G	100	*	TYPE=SUBSTITUTE
CM032587.1	3889471	.	T	C	100	*	TYPE=SUBSTITUTE
CM032587.1	3889472	.	C	A	100	*	TYPE=SUBSTITUTE
CM032587.1	3889476	.	T	A	100	*	TYPE=SUBSTITUTE
CM032587.1	3889479	.	T	A	100	*	TYPE=SUBSTITUTE
CM032587.1	3889481	.	A	C	100	*	TYPE=SUBSTITUTE
CM032587.1	3889482	.	C	T	100	*	TYPE=SUBSTITUTE
CM032587.1	3889487	.	T	A	100	*	TYPE=SUBSTITUTE
CM032587.1	3889490	.	C	A	100	*	TYPE=SUBSTITUTE
CM032587.1	3889492	.	T	A	100	*	TYPE=SUBSTITUTE
CM032587.1	3889493	.	A	T	100	*	TYPE=SUBSTITUTE
CM032587.1	3889495	.	A	C	100	*	TYPE=SUBSTITUTE
CM032587.1	3889498	.	A	ACATG	100	*	TYPE=INSERT
CM032587.1	3889502	.	T	g	100	*	TYPE=SUBSTITUTE

CM032587.1	3889503	.	C	a	100	*	TYPE=SUBSTITUTE
CM032587.1	3889504	.	g	ga	100	*	TYPE=INSERT
CM032587.1	3889505	.	T	a	100	*	TYPE=SUBSTITUTE
CM032587.1	3889507	.	G	a	100	*	TYPE=SUBSTITUTE
CM032587.1	3889509	.	G	a	100	*	TYPE=SUBSTITUTE
CM032587.1	3889513	.	g	gt	100	*	TYPE=INSERT
CM032587.1	3889518	.	t	taGGG	100	*	TYPE=INSERT
CM032587.1	3889520	.	A	G	100	*	TYPE=SUBSTITUTE
CM032587.1	3889521	.	T	A	100	*	TYPE=SUBSTITUTE
CM032587.1	3889523	.	C	G	100	*	TYPE=SUBSTITUTE
CM032587.1	3889525	.	A	G	100	*	TYPE=SUBSTITUTE
CM032587.1	3889526	.	A	T	100	*	TYPE=SUBSTITUTE
CM032587.1	3889529	.	G	T	100	*	TYPE=SUBSTITUTE
CM032587.1	3889532	.	G	A	100	*	TYPE=SUBSTITUTE
CM032587.1	3889545	.	C	T	100	*	TYPE=SUBSTITUTE
CM032587.1	3889556	.	A	G	100	*	TYPE=SUBSTITUTE
CM032587.1	3889557	.	C	A	100	*	TYPE=SUBSTITUTE
CM032587.1	3889560	.	C	G	100	*	TYPE=SUBSTITUTE
CM032587.1	3889566	.	C	G	100	*	TYPE=SUBSTITUTE
CM032587.1	3889568	.	T	G	100	*	TYPE=SUBSTITUTE
CM032587.1	3889569	.	C	A	100	*	TYPE=SUBSTITUTE
CM032587.1	3889581	.	C	G	100	*	TYPE=SUBSTITUTE
CM032587.1	3889584	.	A	G	100	*	TYPE=SUBSTITUTE
CM032587.1	3889588	.	T	C	100	*	TYPE=SUBSTITUTE
CM032587.1	3889593	.	A	G	100	*	TYPE=SUBSTITUTE
CM032587.1	3889595	.	C	T	100	*	TYPE=SUBSTITUTE
CM032587.1	3889596	.	A	G	100	*	TYPE=SUBSTITUTE
CM032587.1	3889602	.	C	A	100	*	TYPE=SUBSTITUTE
CM032587.1	3889662	.	A	G	100	*	TYPE=SUBSTITUTE
CM032587.1	3889668	.	C	G	100	*	TYPE=SUBSTITUTE
CM032587.1	3889671	.	A	G	100	*	TYPE=SUBSTITUTE
CM032587.1	3889680	.	G	T	100	*	TYPE=SUBSTITUTE
CM032587.1	3889731	.	G	A	100	*	TYPE=SUBSTITUTE
CM032587.1	3889743	.	G	a	100	*	TYPE=SUBSTITUTE
CM032587.1	3889848	.	GG	G	100	*	TYPE=DELETE
CM032587.1	3889851	.	A	G	100	*	TYPE=SUBSTITUTE
CM032587.1	3889852	.	T	G	100	*	TYPE=SUBSTITUTE
CM032587.1	3889855	.	A	G	100	*	TYPE=SUBSTITUTE
CM032587.1	3889857	.	G	T	100	*	TYPE=SUBSTITUTE
CM032587.1	3889862	.	C	T	100	*	TYPE=SUBSTITUTE
CM032587.1	3889865	.	T	A	100	*	TYPE=SUBSTITUTE
CM032587.1	3889866	.	G	T	100	*	TYPE=SUBSTITUTE
CM032587.1	3889867	.	A	G	100	*	TYPE=SUBSTITUTE
CM032587.1	3889868	.	T	G	100	*	TYPE=SUBSTITUTE
CM032587.1	3889871	.	A	G	100	*	TYPE=SUBSTITUTE
CM032587.1	3889872	.	A	G	100	*	TYPE=SUBSTITUTE
CM032587.1	3889880	.	A	G	100	*	TYPE=SUBSTITUTE
CM032587.1	3889883	.	G	A	100	*	TYPE=SUBSTITUTE
CM032587.1	3889890	.	T	C	100	*	TYPE=SUBSTITUTE
CM032587.1	3889892	.	T	G	100	*	TYPE=SUBSTITUTE
CM032587.1	3889893	.	G	A	100	*	TYPE=SUBSTITUTE
CM032587.1	3889895	.	G	A	100	*	TYPE=SUBSTITUTE
CM032587.1	3889897	.	A	G	100	*	TYPE=SUBSTITUTE
CM032587.1	3889921	.	G	A	100	*	TYPE=SUBSTITUTE
CM032587.1	3889948	.	C	T	100	*	TYPE=SUBSTITUTE
CM032587.1	3889957	.	C	T	100	*	TYPE=SUBSTITUTE
CM032587.1	3889995	.	C	t	100	*	TYPE=SUBSTITUTE
CM032587.1	3890018	.	T	C	100	*	TYPE=SUBSTITUTE
CM032587.1	3890056	.	T	C	100	*	TYPE=SUBSTITUTE
CM032587.1	3890068	.	ATGT	A	100	*	TYPE=DELETE
CM032587.1	3890074	.	T	C	100	*	TYPE=SUBSTITUTE
CM032587.1	3890110	.	C	T	100	*	TYPE=SUBSTITUTE
CM032587.1	3890119	.	C	A	100	*	TYPE=SUBSTITUTE
CM032587.1	3890122	.	A	C	100	*	TYPE=SUBSTITUTE

CM032587.1	3890158	.	C	T	100	*	TYPE=SUBSTITUTE
CM032587.1	3890176	.	G	A	100	*	TYPE=SUBSTITUTE
CM032587.1	3890179	.	G	A	100	*	TYPE=SUBSTITUTE
CM032587.1	3890184	.	A	G	100	*	TYPE=SUBSTITUTE
CM032587.1	3890206	.	C	T	100	*	TYPE=SUBSTITUTE
CM032587.1	3890227	.	A	G	100	*	TYPE=SUBSTITUTE
CM032587.1	3890236	.	C	T	100	*	TYPE=SUBSTITUTE
CM032587.1	3890296	.	G	C	100	*	TYPE=SUBSTITUTE
CM032587.1	3890326	.	C	T	100	*	TYPE=SUBSTITUTE
CM032587.1	3890329	.	A	C	100	*	TYPE=SUBSTITUTE
CM032587.1	3890365	.	G	T	100	*	TYPE=SUBSTITUTE
CM032587.1	3890407	.	G	A	100	*	TYPE=SUBSTITUTE
CM032587.1	3890476	.	T	C	100	*	TYPE=SUBSTITUTE
CM032587.1	3890491	.	C	T	100	*	TYPE=SUBSTITUTE
CM032587.1	3890518	.	G	T	100	*	TYPE=SUBSTITUTE
CM032587.1	3890521	.	A	G	100	*	TYPE=SUBSTITUTE
CM032587.1	3890528	.	TA	T	100	*	TYPE=DELETE
CM032587.1	3890530	.	T	G	100	*	TYPE=SUBSTITUTE
CM032587.1	3890533	.	C	g	100	*	TYPE=SUBSTITUTE
CM032587.1	3890536	.	c	ca	100	*	TYPE=INSERT
CM032587.1	3890544	.	A	g	100	*	TYPE=SUBSTITUTE
CM032587.1	3890545	.	G	a	100	*	TYPE=SUBSTITUTE
CM032587.1	3890547	.	A	g	100	*	TYPE=SUBSTITUTE
CM032587.1	3890552	.	T	C	100	*	TYPE=SUBSTITUTE
CM032587.1	3890556	.	C	T	100	*	TYPE=SUBSTITUTE
CM032587.1	3890565	.	T	A	100	*	TYPE=SUBSTITUTE
CM032587.1	3890568	.	T	A	100	*	TYPE=SUBSTITUTE
CM032587.1	3890574	.	C	T	100	*	TYPE=SUBSTITUTE
CM032587.1	3890577	.	G	T	100	*	TYPE=SUBSTITUTE
CM032587.1	3890582	.	T	C	100	*	TYPE=SUBSTITUTE
CM032587.1	3890583	.	T	C	100	*	TYPE=SUBSTITUTE
CM032587.1	3890584	.	C	T	100	*	TYPE=SUBSTITUTE
CM032587.1	3890585	.	T	C	100	*	TYPE=SUBSTITUTE
CM032587.1	3890591	.	A	G	100	*	TYPE=SUBSTITUTE
CM032587.1	3890592	.	A	G	100	*	TYPE=SUBSTITUTE
CM032587.1	3890598	.	T	C	100	*	TYPE=SUBSTITUTE
CM032587.1	3890613	.	T	TN	100	*	TYPE=INSERT
CM032587.1	3890618	.	C	T	100	*	TYPE=SUBSTITUTE
CM032587.1	3890620	.	G	GC	100	*	TYPE=INSERT
CM032587.1	3890660	.	C	T	100	*	TYPE=SUBSTITUTE
CM032587.1	3890669	.	G	T	100	*	TYPE=SUBSTITUTE
CM032587.1	3890678	.	T	C	100	*	TYPE=SUBSTITUTE
CM032587.1	3890682	.	G	A	100	*	TYPE=SUBSTITUTE
CM032587.1	3890717	.	C	T	100	*	TYPE=SUBSTITUTE
CM032587.1	3890723	.	A	G	100	*	TYPE=SUBSTITUTE
CM032587.1	3890735	.	C	T	100	*	TYPE=SUBSTITUTE
CM032587.1	3890756	.	T	G	100	*	TYPE=SUBSTITUTE
CM032587.1	3890773	.	T	C	100	*	TYPE=SUBSTITUTE
CM032587.1	3890779	.	G	A	100	*	TYPE=SUBSTITUTE
CM032587.1	3890780	.	C	G	100	*	TYPE=SUBSTITUTE
CM032587.1	3890787	.	G	A	100	*	TYPE=SUBSTITUTE
CM032587.1	3890795	.	T	C	100	*	TYPE=SUBSTITUTE
CM032587.1	3890798	.	T	C	100	*	TYPE=SUBSTITUTE
CM032587.1	3890810	.	A	g	100	*	TYPE=SUBSTITUTE
CM032587.1	3890812	.	C	a	100	*	TYPE=SUBSTITUTE
CM032587.1	3890814	.	A	g	100	*	TYPE=SUBSTITUTE
CM032587.1	3890821	.	a	aagg	100	*	TYPE=INSERT
CM032587.1	3890824	.	A	g	100	*	TYPE=SUBSTITUTE
CM032587.1	3890827	.	A	g	100	*	TYPE=SUBSTITUTE
CM032587.1	3890828	.	A	g	100	*	TYPE=SUBSTITUTE
CM032587.1	3890842	.	G	A	100	*	TYPE=SUBSTITUTE
CM032587.1	3890843	.	A	G	100	*	TYPE=SUBSTITUTE
CM032587.1	3890850	.	G	A	100	*	TYPE=SUBSTITUTE
CM032587.1	3890911	.	C	T	100	*	TYPE=SUBSTITUTE

CM032587.1	3890916	.	A	G	100	*	TYPE=SUBSTITUTE
CM032587.1	3890924	.	T	TC	100	*	TYPE=INSERT
CM032587.1	3890929	.	A	G	100	*	TYPE=SUBSTITUTE
CM032587.1	3890934	.	C	T	100	*	TYPE=SUBSTITUTE
CM032587.1	3890944	.	T	C	100	*	TYPE=SUBSTITUTE
CM032587.1	3890957	.	G	A	100	*	TYPE=SUBSTITUTE
CM032587.1	3890961	.	A	G	100	*	TYPE=SUBSTITUTE
CM032587.1	3890968	.	T	C	100	*	TYPE=SUBSTITUTE
CM032587.1	3890984	.	T	C	100	*	TYPE=SUBSTITUTE
CM032587.1	3890991	.	A	G	100	*	TYPE=SUBSTITUTE
CM032587.1	4530584	.	A	C	100	*	TYPE=SUBSTITUTE
CM032587.1	4530592	.	G	C	100	*	TYPE=SUBSTITUTE
CM032587.1	4530621	.	A	G	100	*	TYPE=SUBSTITUTE
CM032587.1	4530621	.	A	G	100	*	TYPE=SUBSTITUTE
CM032587.1	4530634	.	A	G	100	*	TYPE=SUBSTITUTE
CM032587.1	4530634	.	A	G	100	*	TYPE=SUBSTITUTE
CM032587.1	4530636	.	A	C	100	*	TYPE=SUBSTITUTE
CM032587.1	4530637	.	A	G	100	*	TYPE=SUBSTITUTE
CM032587.1	4530638	.	G	T	100	*	TYPE=SUBSTITUTE
CM032587.1	4530638	.	G	T	100	*	TYPE=SUBSTITUTE
CM032587.1	4530641	.	G	A	100	*	TYPE=SUBSTITUTE
CM032587.1	4530641	.	G	A	100	*	TYPE=SUBSTITUTE
CM032587.1	4530642	.	A	G	100	*	TYPE=SUBSTITUTE
CM032587.1	4530642	.	A	G	100	*	TYPE=SUBSTITUTE
CM032587.1	4530643	.	T	A	100	*	TYPE=SUBSTITUTE
CM032587.1	4530643	.	T	A	100	*	TYPE=SUBSTITUTE
CM032587.1	4530677	.	A	G	100	*	TYPE=SUBSTITUTE
CM032587.1	4530704	.	A	G	100	*	TYPE=SUBSTITUTE
CM032587.1	4530704	.	A	G	100	*	TYPE=SUBSTITUTE
CM032587.1	4530731	.	G	A	100	*	TYPE=SUBSTITUTE
CM032587.1	6931441	.	C	A	100	*	TYPE=SUBSTITUTE
CM032587.1	6931445	.	C	T	100	*	TYPE=SUBSTITUTE
CM032587.1	6931488	.	T	C	100	*	TYPE=SUBSTITUTE
CM032587.1	6931496	.	G	A	100	*	TYPE=SUBSTITUTE
CM032587.1	6931516	.	G	A	100	*	TYPE=SUBSTITUTE
CM032587.1	6931517	.	G	C	100	*	TYPE=SUBSTITUTE
CM032587.1	6931520	.	A	G	100	*	TYPE=SUBSTITUTE
CM032587.1	6931521	.	C	G	100	*	TYPE=SUBSTITUTE
CM032587.1	6931524	.	G	T	100	*	TYPE=SUBSTITUTE
CM032587.1	6931530	.	T	C	100	*	TYPE=SUBSTITUTE
CM032587.1	6931533	.	G	C	100	*	TYPE=SUBSTITUTE
CM032587.1	6931559	.	A	G	100	*	TYPE=SUBSTITUTE
CM032587.1	6931571	.	T	C	100	*	TYPE=SUBSTITUTE
CM032587.1	6931580	.	A	G	100	*	TYPE=SUBSTITUTE
CM032587.1	6931589	.	G	T	100	*	TYPE=SUBSTITUTE
CM032587.1	6931623	.	A	C	100	*	TYPE=SUBSTITUTE
CM032587.1	6931633	.	A	G	100	*	TYPE=SUBSTITUTE
CM032587.1	6931634	.	A	G	100	*	TYPE=SUBSTITUTE
CM032587.1	6931652	.	C	A	100	*	TYPE=SUBSTITUTE
CM032587.1	6931663	.	G	A	100	*	TYPE=SUBSTITUTE
CM032587.1	6931668	.	C	T	100	*	TYPE=SUBSTITUTE
CM032587.1	6931669	.	G	A	100	*	TYPE=SUBSTITUTE
CM032587.1	6931688	.	C	CC	100	*	TYPE=INSERT
CM032587.1	6931707	.	C	G	100	*	TYPE=SUBSTITUTE
CM032587.1	6931766	.	T	c	100	*	TYPE=SUBSTITUTE
CM032587.1	6931771	.	T	c	100	*	TYPE=SUBSTITUTE
CM032587.1	6931776	.	C	t	100	*	TYPE=SUBSTITUTE
CM032587.1	6931785	.	G	A	100	*	TYPE=SUBSTITUTE
CM032587.1	6931818	.	T	C	100	*	TYPE=SUBSTITUTE
CM032587.1	6931906	.	T	C	100	*	TYPE=SUBSTITUTE
CM032587.1	6931927	.	G	C	100	*	TYPE=SUBSTITUTE
CM032587.1	6931930	.	A	T	100	*	TYPE=SUBSTITUTE
CM032587.1	6931941	.	A	G	100	*	TYPE=SUBSTITUTE
CM032587.1	6931960	.	G	T	100	*	TYPE=SUBSTITUTE

CM032587.1	6931962	.	C	T	100	*	TYPE=SUBSTITUTE
CM032587.1	6931976	.	G	A	100	*	TYPE=SUBSTITUTE
CM032587.1	6931993	.	A	G	100	*	TYPE=SUBSTITUTE
CM032587.1	6932001	.	C	T	100	*	TYPE=SUBSTITUTE
CM032587.1	6932020	.	G	A	100	*	TYPE=SUBSTITUTE
CM032587.1	6932037	.	T	C	100	*	TYPE=SUBSTITUTE
CM032587.1	6932065	.	A	T	100	*	TYPE=SUBSTITUTE
CM032587.1	6932066	.	T	TG	100	*	TYPE=INSERT
CM032587.1	6932067	.	T	A	100	*	TYPE=SUBSTITUTE
CM032587.1	6932097	.	TCT	T	100	*	TYPE=DELETE
CM032587.1	6932105	.	A	G	100	*	TYPE=SUBSTITUTE
CM032587.1	6932107	.	C	A	100	*	TYPE=SUBSTITUTE
CM032587.1	6932110	.	G	T	100	*	TYPE=SUBSTITUTE
CM032587.1	6932113	.	T	C	100	*	TYPE=SUBSTITUTE
CM032587.1	6932117	.	C	A	100	*	TYPE=SUBSTITUTE
CM032587.1	6932136	.	T	C	100	*	TYPE=SUBSTITUTE
CM032587.1	6932146	.	A	C	100	*	TYPE=SUBSTITUTE
CM032587.1	6932149	.	CA	C	100	*	TYPE=DELETE
CM032587.1	6932177	.	G	A	100	*	TYPE=SUBSTITUTE
CM032587.1	6932199	.	A	G	100	*	TYPE=SUBSTITUTE
CM032587.1	6932201	.	G	A	100	*	TYPE=SUBSTITUTE
CM032587.1	6932202	.	A	G	100	*	TYPE=SUBSTITUTE
CM032587.1	6932213	.	A	G	100	*	TYPE=SUBSTITUTE
CM032587.1	6932215	.	G	A	100	*	TYPE=SUBSTITUTE
CM032587.1	6932219	.	T	C	100	*	TYPE=SUBSTITUTE
CM032587.1	6932224	.	T	C	100	*	TYPE=SUBSTITUTE
CM032587.1	6932230	.	TA	T	100	*	TYPE=DELETE
CM032587.1	6932234	.	A	G	100	*	TYPE=SUBSTITUTE
CM032587.1	6932260	.	A	C	100	*	TYPE=SUBSTITUTE
CM032587.1	6932281	.	C	T	100	*	TYPE=SUBSTITUTE
CM032587.1	6932288	.	A	G	100	*	TYPE=SUBSTITUTE
CM032587.1	6932301	.	G	A	100	*	TYPE=SUBSTITUTE
CM032587.1	6932335	.	T	C	100	*	TYPE=SUBSTITUTE
CM032587.1	6932338	.	C	A	100	*	TYPE=SUBSTITUTE
CM032587.1	6932349	.	G	C	100	*	TYPE=SUBSTITUTE
CM032587.1	6932356	.	A	G	100	*	TYPE=SUBSTITUTE
CM032587.1	6932374	.	T	C	100	*	TYPE=SUBSTITUTE
CM032587.1	6932378	.	T	A	100	*	TYPE=SUBSTITUTE
CM032587.1	6932395	.	C	T	100	*	TYPE=SUBSTITUTE
CM032587.1	6932401	.	T	G	100	*	TYPE=SUBSTITUTE
CM032587.1	6932418	.	T	C	100	*	TYPE=SUBSTITUTE
CM032587.1	6932423	.	A	t	100	*	TYPE=SUBSTITUTE
CM032587.1	6932425	.	C	t	100	*	TYPE=SUBSTITUTE
CM032587.1	6932430	.	C	t	100	*	TYPE=SUBSTITUTE
CM032587.1	6932435	.	T	a	100	*	TYPE=SUBSTITUTE
CM032587.1	6932438	.	A	G	100	*	TYPE=SUBSTITUTE
CM032587.1	6932473	.	C	T	100	*	TYPE=SUBSTITUTE
CM032587.1	6932476	.	A	C	100	*	TYPE=SUBSTITUTE
CM032587.1	6932479	.	C	T	100	*	TYPE=SUBSTITUTE
CM032587.1	6932491	.	C	T	100	*	TYPE=SUBSTITUTE
CM032587.1	6932494	.	T	C	100	*	TYPE=SUBSTITUTE
CM032587.1	6932515	.	C	T	100	*	TYPE=SUBSTITUTE
CM032587.1	6932569	.	A	G	100	*	TYPE=SUBSTITUTE
CM032587.1	6932572	.	A	G	100	*	TYPE=SUBSTITUTE
CM032587.1	6932593	.	C	T	100	*	TYPE=SUBSTITUTE
CM032587.1	6932599	.	G	A	100	*	TYPE=SUBSTITUTE
CM032587.1	6932614	.	T	C	100	*	TYPE=SUBSTITUTE
CM032587.1	6932641	.	A	G	100	*	TYPE=SUBSTITUTE
CM032587.1	6932644	.	C	A	100	*	TYPE=SUBSTITUTE
CM032587.1	6932654	.	C	G	100	*	TYPE=SUBSTITUTE
CM032587.1	6932655	.	T	G	100	*	TYPE=SUBSTITUTE
CM032587.1	6932680	.	G	A	100	*	TYPE=SUBSTITUTE
CM032587.1	6932686	.	T	C	100	*	TYPE=SUBSTITUTE
CM032587.1	6932689	.	C	T	100	*	TYPE=SUBSTITUTE

CM032587.1	6932695	.	C	T	100	*	TYPE=SUBSTITUTE
CM032587.1	6932707	.	A	C	100	*	TYPE=SUBSTITUTE
CM032587.1	6932714	.	A	C	100	*	TYPE=SUBSTITUTE
CM032587.1	6932715	.	A	G	100	*	TYPE=SUBSTITUTE
CM032587.1	6932746	.	C	A	100	*	TYPE=SUBSTITUTE
CM032587.1	6932767	.	A	G	100	*	TYPE=SUBSTITUTE
CM032587.1	6932856	.	G	C	100	*	TYPE=SUBSTITUTE
CM032587.1	6932998	.	C	T	100	*	TYPE=SUBSTITUTE
CM032587.1	6933034	.	C	T	100	*	TYPE=SUBSTITUTE
CM032587.1	6933043	.	C	T	100	*	TYPE=SUBSTITUTE
CM032587.1	6933070	.	A	C	100	*	TYPE=SUBSTITUTE
CM032587.1	6933106	.	T	C	100	*	TYPE=SUBSTITUTE
CM032587.1	6933112	.	C	G	100	*	TYPE=SUBSTITUTE
CM032587.1	6933118	.	C	T	100	*	TYPE=SUBSTITUTE
CM032587.1	6933124	.	T	C	100	*	TYPE=SUBSTITUTE
CM032587.1	6933133	.	C	T	100	*	TYPE=SUBSTITUTE
CM032587.1	6933139	.	C	T	100	*	TYPE=SUBSTITUTE
CM032587.1	6933141	.	A	G	100	*	TYPE=SUBSTITUTE
CM032587.1	6933142	.	C	T	100	*	TYPE=SUBSTITUTE
CM032587.1	6933143	.	T	C	100	*	TYPE=SUBSTITUTE
CM032587.1	6933145	.	A	G	100	*	TYPE=SUBSTITUTE
CM032587.1	6933152	.	C	A	100	*	TYPE=SUBSTITUTE
CM032587.1	6933154	.	A	G	100	*	TYPE=SUBSTITUTE
CM032587.1	6933163	.	T	C	100	*	TYPE=SUBSTITUTE
CM032587.1	6933169	.	C	T	100	*	TYPE=SUBSTITUTE
CM032587.1	6933178	.	A	G	100	*	TYPE=SUBSTITUTE
CM032587.1	6933202	.	C	T	100	*	TYPE=SUBSTITUTE
CM032587.1	6933217	.	A	G	100	*	TYPE=SUBSTITUTE
CM032587.1	6933241	.	A	G	100	*	TYPE=SUBSTITUTE
CM032587.1	6933254	.	A	G	100	*	TYPE=SUBSTITUTE
CM032587.1	6933264	.	C	A	100	*	TYPE=SUBSTITUTE
CM032587.1	6933268	.	T	C	100	*	TYPE=SUBSTITUTE
CM032587.1	6933277	.	T	C	100	*	TYPE=SUBSTITUTE
CM032587.1	6933283	.	T	C	100	*	TYPE=SUBSTITUTE
CM032587.1	6933290	.	A	G	100	*	TYPE=SUBSTITUTE
CM032587.1	6933292	.	C	T	100	*	TYPE=SUBSTITUTE
CM032587.1	6933300	.	A	C	100	*	TYPE=SUBSTITUTE
CM032587.1	6933301	.	C	T	100	*	TYPE=SUBSTITUTE
CM032587.1	6933304	.	T	C	100	*	TYPE=SUBSTITUTE
CM032587.1	6933370	.	G	C	100	*	TYPE=SUBSTITUTE
CM032587.1	6933397	.	C	T	100	*	TYPE=SUBSTITUTE
CM032587.1	6933400	.	C	T	100	*	TYPE=SUBSTITUTE
CM032587.1	6933403	.	T	C	100	*	TYPE=SUBSTITUTE
CM032587.1	6933416	.	C	A	100	*	TYPE=SUBSTITUTE
CM032587.1	6933436	.	T	G	100	*	TYPE=SUBSTITUTE
CM032587.1	6933490	.	T	C	100	*	TYPE=SUBSTITUTE
CM032587.1	6933523	.	T	C	100	*	TYPE=SUBSTITUTE
CM032587.1	6933532	.	A	C	100	*	TYPE=SUBSTITUTE
CM032587.1	6933589	.	T	C	100	*	TYPE=SUBSTITUTE
CM032587.1	6933601	.	C	T	100	*	TYPE=SUBSTITUTE
CM032587.1	6933610	.	C	T	100	*	TYPE=SUBSTITUTE
CM032587.1	6933626	.	T	A	100	*	TYPE=SUBSTITUTE
CM032587.1	6933627	.	A	C	100	*	TYPE=SUBSTITUTE
CM032587.1	6933628	.	T	C	100	*	TYPE=SUBSTITUTE
CM032587.1	6933634	.	C	T	100	*	TYPE=SUBSTITUTE
CM032587.1	6933661	.	A	G	100	*	TYPE=SUBSTITUTE
CM032587.1	6933668	.	G	T	100	*	TYPE=SUBSTITUTE
CM032587.1	6933700	.	C	T	100	*	TYPE=SUBSTITUTE
CM032587.1	6933706	.	G	A	100	*	TYPE=SUBSTITUTE
CM032587.1	6933715	.	G	T	100	*	TYPE=SUBSTITUTE
CM032587.1	6933727	.	G	T	100	*	TYPE=SUBSTITUTE
CM032587.1	6933730	.	A	G	100	*	TYPE=SUBSTITUTE
CM032587.1	6933736	.	G	T	100	*	TYPE=SUBSTITUTE
CM032587.1	6933739	.	A	C	100	*	TYPE=SUBSTITUTE

CM032587.1	6933748	.	T	G	100	*	TYPE=SUBSTITUTE
CM032587.1	6933751	.	A	G	100	*	TYPE=SUBSTITUTE
CM032587.1	6933772	.	CAAG	C	100	*	TYPE=DELETE
CM032587.1	6933776	.	A	G	100	*	TYPE=SUBSTITUTE
CM032587.1	6933779	.	A	G	100	*	TYPE=SUBSTITUTE
CM032587.1	6933783	.	C	T	100	*	TYPE=SUBSTITUTE
CM032587.1	6933785	.	C	T	100	*	TYPE=SUBSTITUTE
CM032587.1	6933793	.	A	C	100	*	TYPE=SUBSTITUTE
CM032587.1	6933836	.	C	T	100	*	TYPE=SUBSTITUTE
CM032587.1	6933837	.	G	C	100	*	TYPE=SUBSTITUTE
CM032587.1	6933841	.	C	T	100	*	TYPE=SUBSTITUTE
CM032587.1	6933849	.	C	G	100	*	TYPE=SUBSTITUTE
CM032587.1	6933871	.	T	C	100	*	TYPE=SUBSTITUTE
CM032587.1	6933874	.	A	G	100	*	TYPE=SUBSTITUTE
CM032587.1	6933877	.	A	G	100	*	TYPE=SUBSTITUTE
CM032587.1	6933901	.	C	T	100	*	TYPE=SUBSTITUTE
CM032587.1	6933902	.	T	C	100	*	TYPE=SUBSTITUTE
CM032587.1	6933919	.	T	C	100	*	TYPE=SUBSTITUTE
CM032587.1	6933964	.	A	G	100	*	TYPE=SUBSTITUTE
CM032587.1	6933968	.	C	T	100	*	TYPE=SUBSTITUTE
CM032587.1	6933988	.	C	T	100	*	TYPE=SUBSTITUTE
CM032587.1	6933991	.	C	T	100	*	TYPE=SUBSTITUTE
CM032587.1	6933995	.	C	T	100	*	TYPE=SUBSTITUTE
CM032587.1	6934026	.	C	T	100	*	TYPE=SUBSTITUTE
CM032587.1	6934037	.	T	G	100	*	TYPE=SUBSTITUTE
CM032587.1	6934040	.	A	G	100	*	TYPE=SUBSTITUTE
CM032587.1	6934045	.	T	C	100	*	TYPE=SUBSTITUTE
CM032587.1	6934058	.	A	C	100	*	TYPE=SUBSTITUTE
CM032587.1	6934059	.	A	G	100	*	TYPE=SUBSTITUTE
CM032587.1	6934060	.	G	A	100	*	TYPE=SUBSTITUTE
CM032587.1	6934072	.	AG	A	100	*	TYPE=DELETE
CM032587.1	6934084	.	T	C	100	*	TYPE=SUBSTITUTE
CM032587.1	6934092	.	G	A	100	*	TYPE=SUBSTITUTE
CM032587.1	6934095	.	C	T	100	*	TYPE=SUBSTITUTE
CM032587.1	6934096	.	C	T	100	*	TYPE=SUBSTITUTE
CM032587.1	6934101	.	c	cgggat	100	*	TYPE=INSERT
CM032587.1	6934113	.	A	g	100	*	TYPE=SUBSTITUTE
CM032587.1	6934116	.	T	A	100	*	TYPE=SUBSTITUTE
CM032587.1	6934128	.	GGCTGTTATGAGACACCCCCT	G	100	*	TYPE=DELETE
CM032587.1	6934150	.	A	T	100	*	TYPE=SUBSTITUTE
CM032587.1	6934152	.	T	A	100	*	TYPE=SUBSTITUTE
CM032587.1	6934156	.	C	T	100	*	TYPE=SUBSTITUTE
CM032587.1	6934160	.	T	C	100	*	TYPE=SUBSTITUTE
CM032587.1	6934161	.	G	T	100	*	TYPE=SUBSTITUTE
CM032587.1	6934171	.	A	AG	100	*	TYPE=INSERT
CM032587.1	6934178	.	T	G	100	*	TYPE=SUBSTITUTE
CM032587.1	6934179	.	C	A	100	*	TYPE=SUBSTITUTE
CM032587.1	6934181	.	T	C	100	*	TYPE=SUBSTITUTE
CM032587.1	6934183	.	G	A	100	*	TYPE=SUBSTITUTE
CM032587.1	6934184	.	C	T	100	*	TYPE=SUBSTITUTE
CM032587.1	6934211	.	A	T	100	*	TYPE=SUBSTITUTE
CM032587.1	6934212	.	T	C	100	*	TYPE=SUBSTITUTE
CM032587.1	6934213	.	A	G	100	*	TYPE=SUBSTITUTE
CM032587.1	6934239	.	T	C	100	*	TYPE=SUBSTITUTE
CM032587.1	6934357	.	A	G	100	*	TYPE=SUBSTITUTE
CM032587.1	6934358	.	A	T	100	*	TYPE=SUBSTITUTE
CM032587.1	6934360	.	A	G	100	*	TYPE=SUBSTITUTE
CM032587.1	6934376	.	C	CT	100	*	TYPE=INSERT
CM032587.1	6934379	.	G	A	100	*	TYPE=SUBSTITUTE
CM032587.1	6934403	.	A	G	100	*	TYPE=SUBSTITUTE
CM032587.1	6934409	.	CT	C	100	*	TYPE=DELETE
CM032587.1	6934411	.	T	A	100	*	TYPE=SUBSTITUTE
CM032587.1	6934416	.	A	AGCGCCCTGCTTT	100	*	TYPE=INSERT

CM032587.1	6934440	.	G	t	100	*	TYPE=SUBSTITUTE
CM032587.1	6934441	.	T	a	100	*	TYPE=SUBSTITUTE
CM032587.1	6934447	.	A	g	100	*	TYPE=SUBSTITUTE
CM032587.1	6934452	.	A	t	100	*	TYPE=SUBSTITUTE
CM032587.1	6934480	.	a	agaa	100	*	TYPE=INSERT
CM032587.1	6934485	.	g	ggtatcCTGAAGGA	100	*	TYPE=INSERT
CM032587.1	6934487	.	T	G	100	*	TYPE=SUBSTITUTE
CM032587.1	6934505	.	A	T	100	*	TYPE=SUBSTITUTE
CM032587.1	6934515	.	A	G	100	*	TYPE=SUBSTITUTE
CM032587.1	6934519	.	A	G	100	*	TYPE=SUBSTITUTE
CM032587.1	6934520	.	G	A	100	*	TYPE=SUBSTITUTE
CM032587.1	6934531	.	ATGTGAAATGTAGATGCATCTTGGT	A	100	*	TYPE=DELETE
CM032587.1	6934556	.	G	A	100	*	TYPE=SUBSTITUTE
CM032587.1	6934574	.	C	T	100	*	TYPE=SUBSTITUTE
CM032587.1	6934575	.	A	T	100	*	TYPE=SUBSTITUTE
CM032587.1	6934576	.	C	T	100	*	TYPE=SUBSTITUTE
CM032587.1	6934577	.	C	G	100	*	TYPE=SUBSTITUTE
CM032587.1	6934581	.	G	A	100	*	TYPE=SUBSTITUTE
CM032587.1	6934584	.	G	T	100	*	TYPE=SUBSTITUTE
CM032587.1	6934588	.	G	A	100	*	TYPE=SUBSTITUTE
CM032587.1	6934594	.	C	CGA	100	*	TYPE=INSERT
CM032587.1	6934618	.	T	A	100	*	TYPE=SUBSTITUTE
CM032587.1	6934629	.	G	A	100	*	TYPE=SUBSTITUTE
CM032587.1	6934631	.	C	A	100	*	TYPE=SUBSTITUTE
CM032587.1	6934648	.	T	C	100	*	TYPE=SUBSTITUTE
CM032587.1	6934651	.	A	g	100	*	TYPE=SUBSTITUTE
CM032587.1	6934684	.	C	T	100	*	TYPE=SUBSTITUTE
CM032587.1	6934705	.	C	T	100	*	TYPE=SUBSTITUTE
CM032587.1	6934723	.	A	g	100	*	TYPE=SUBSTITUTE
CM032587.1	6934726	.	A	g	100	*	TYPE=SUBSTITUTE
CM032587.1	6934774	.	C	G	100	*	TYPE=SUBSTITUTE
CM032587.1	6934783	.	A	G	100	*	TYPE=SUBSTITUTE
CM032587.1	6934792	.	A	G	100	*	TYPE=SUBSTITUTE
CM032587.1	6934801	.	T	G	100	*	TYPE=SUBSTITUTE
CM032587.1	6934807	.	G	A	100	*	TYPE=SUBSTITUTE
CM032587.1	6934810	.	A	G	100	*	TYPE=SUBSTITUTE
CM032587.1	6934828	.	T	G	100	*	TYPE=SUBSTITUTE
CM032587.1	6934831	.	C	A	100	*	TYPE=SUBSTITUTE
CM032587.1	6934832	.	T	C	100	*	TYPE=SUBSTITUTE
CM032587.1	6934833	.	G	T	100	*	TYPE=SUBSTITUTE
CM032587.1	6934834	.	T	C	100	*	TYPE=SUBSTITUTE
CM032587.1	6934845	.	G	C	100	*	TYPE=SUBSTITUTE
CM032587.1	6934867	.	A	G	100	*	TYPE=SUBSTITUTE
CM032587.1	6934876	.	A	G	100	*	TYPE=SUBSTITUTE
CM032587.1	6934879	.	G	A	100	*	TYPE=SUBSTITUTE
CM032587.1	6934882	.	A	G	100	*	TYPE=SUBSTITUTE
CM032587.1	6934900	.	G	A	100	*	TYPE=SUBSTITUTE
CM032587.1	6934903	.	C	T	100	*	TYPE=SUBSTITUTE
CM032587.1	6934927	.	A	G	100	*	TYPE=SUBSTITUTE
CM032587.1	6934930	.	C	G	100	*	TYPE=SUBSTITUTE
CM032587.1	6934933	.	T	C	100	*	TYPE=SUBSTITUTE
CM032587.1	6934969	.	G	A	100	*	TYPE=SUBSTITUTE
CM032587.1	6934972	.	G	C	100	*	TYPE=SUBSTITUTE
CM032587.1	6934975	.	G	A	100	*	TYPE=SUBSTITUTE
CM032587.1	6935005	.	A	G	100	*	TYPE=SUBSTITUTE
CM032587.1	6935011	.	C	G	100	*	TYPE=SUBSTITUTE
CM032587.1	6935013	.	A	G	100	*	TYPE=SUBSTITUTE
CM032587.1	6935038	.	T	G	100	*	TYPE=SUBSTITUTE
CM032587.1	6935068	.	A	G	100	*	TYPE=SUBSTITUTE
CM032587.1	6935077	.	A	G	100	*	TYPE=SUBSTITUTE
CM032587.1	6935080	.	A	G	100	*	TYPE=SUBSTITUTE
CM032587.1	6935107	.	G	A	100	*	TYPE=SUBSTITUTE
CM032587.1	6935122	.	G	A	100	*	TYPE=SUBSTITUTE

CM032587.1	6935140	.	G	A	100	*	TYPE=SUBSTITUTE
CM032587.1	6935161	.	A	G	100	*	TYPE=SUBSTITUTE
CM032587.1	6935167	.	A	G	100	*	TYPE=SUBSTITUTE
CM032587.1	6935176	.	G	A	100	*	TYPE=SUBSTITUTE
CM032587.1	6935206	.	T	G	100	*	TYPE=SUBSTITUTE
CM032587.1	6935298	.	A	T	100	*	TYPE=SUBSTITUTE
CM032587.1	6935353	.	C	G	100	*	TYPE=SUBSTITUTE
CM032587.1	6935356	.	C	T	100	*	TYPE=SUBSTITUTE
CM032587.1	6935371	.	A	G	100	*	TYPE=SUBSTITUTE
CM032587.1	6935386	.	C	T	100	*	TYPE=SUBSTITUTE
CM032587.1	6935404	.	A	G	100	*	TYPE=SUBSTITUTE
CM032587.1	6935488	.	A	G	100	*	TYPE=SUBSTITUTE
CM032587.1	6935491	.	C	G	100	*	TYPE=SUBSTITUTE
CM032587.1	6935493	.	A	G	100	*	TYPE=SUBSTITUTE
CM032587.1	6935497	.	C	A	100	*	TYPE=SUBSTITUTE
CM032587.1	6935524	.	C	T	100	*	TYPE=SUBSTITUTE
CM032587.1	6935533	.	A	G	100	*	TYPE=SUBSTITUTE
CM032587.1	6935536	.	G	T	100	*	TYPE=SUBSTITUTE
CM032587.1	6935542	.	C	G	100	*	TYPE=SUBSTITUTE
CM032587.1	6935544	.	A	G	100	*	TYPE=SUBSTITUTE
CM032587.1	6935545	.	A	G	100	*	TYPE=SUBSTITUTE
CM032587.1	6935554	.	A	T	100	*	TYPE=SUBSTITUTE
CM032587.1	6935593	.	G	C	100	*	TYPE=SUBSTITUTE
CM032587.1	6935632	.	C	G	100	*	TYPE=SUBSTITUTE
CM032587.1	6935656	.	C	T	100	*	TYPE=SUBSTITUTE
CM032587.1	6935659	.	G	A	100	*	TYPE=SUBSTITUTE
CM032587.1	6935689	.	A	T	100	*	TYPE=SUBSTITUTE
CM032587.1	6935695	.	G	A	100	*	TYPE=SUBSTITUTE
CM032587.1	6935704	.	G	A	100	*	TYPE=SUBSTITUTE
CM032587.1	6935712	.	G	A	100	*	TYPE=SUBSTITUTE
CM032587.1	6935722	.	C	A	100	*	TYPE=SUBSTITUTE
CM032587.1	6935725	.	A	C	100	*	TYPE=SUBSTITUTE
CM032587.1	6935737	.	C	A	100	*	TYPE=SUBSTITUTE
CM032587.1	6935740	.	G	A	100	*	TYPE=SUBSTITUTE
CM032587.1	6935746	.	G	A	100	*	TYPE=SUBSTITUTE
CM032587.1	6935800	.	G	C	100	*	TYPE=SUBSTITUTE
CM032587.1	6935833	.	A	G	100	*	TYPE=SUBSTITUTE
CM032587.1	6935839	.	A	G	100	*	TYPE=SUBSTITUTE
CM032587.1	6935870	.	G	T	100	*	TYPE=SUBSTITUTE
CM032587.1	6935932	.	C	T	100	*	TYPE=SUBSTITUTE
CM032587.1	6935935	.	G	A	100	*	TYPE=SUBSTITUTE
CM032587.1	6935968	.	A	G	100	*	TYPE=SUBSTITUTE
CM032587.1	6935974	.	T	A	100	*	TYPE=SUBSTITUTE
CM032587.1	6936019	.	C	T	100	*	TYPE=SUBSTITUTE
CM032587.1	6936061	.	A	G	100	*	TYPE=SUBSTITUTE
CM032587.1	6936100	.	G	T	100	*	TYPE=SUBSTITUTE
CM032587.1	6936109	.	G	T	100	*	TYPE=SUBSTITUTE
CM032587.1	6936127	.	T	G	100	*	TYPE=SUBSTITUTE
CM032587.1	6936148	.	G	A	100	*	TYPE=SUBSTITUTE
CM032587.1	6936151	.	G	A	100	*	TYPE=SUBSTITUTE
CM032587.1	6936154	.	A	G	100	*	TYPE=SUBSTITUTE
CM032587.1	6936157	.	A	G	100	*	TYPE=SUBSTITUTE
CM032587.1	6936166	.	A	G	100	*	TYPE=SUBSTITUTE
CM032587.1	6936169	.	C	T	100	*	TYPE=SUBSTITUTE
CM032587.1	6936175	.	G	A	100	*	TYPE=SUBSTITUTE
CM032587.1	6936208	.	T	C	100	*	TYPE=SUBSTITUTE
CM032587.1	6936220	.	T	C	100	*	TYPE=SUBSTITUTE
CM032587.1	6936229	.	G	A	100	*	TYPE=SUBSTITUTE
CM032587.1	6936244	.	G	A	100	*	TYPE=SUBSTITUTE
CM032587.1	6936247	.	C	T	100	*	TYPE=SUBSTITUTE
CM032587.1	6936249	.	T	G	100	*	TYPE=SUBSTITUTE
CM032587.1	6936250	.	G	A	100	*	TYPE=SUBSTITUTE
CM032587.1	6936274	.	T	C	100	*	TYPE=SUBSTITUTE
CM032587.1	6936280	.	A	G	100	*	TYPE=SUBSTITUTE

CM032587.1	6936337	.	C	G	100	*	TYPE=SUBSTITUTE
CM032587.1	6936340	.	A	G	100	*	TYPE=SUBSTITUTE
CM032587.1	6936367	.	A	G	100	*	TYPE=SUBSTITUTE
CM032587.1	6936385	.	T	C	100	*	TYPE=SUBSTITUTE
CM032587.1	6936394	.	T	C	100	*	TYPE=SUBSTITUTE
CM032587.1	6936400	.	T	C	100	*	TYPE=SUBSTITUTE
CM032587.1	6936403	.	T	A	100	*	TYPE=SUBSTITUTE
CM032587.1	6936415	.	A	G	100	*	TYPE=SUBSTITUTE
CM032587.1	6936424	.	G	A	100	*	TYPE=SUBSTITUTE
CM032587.1	6936432	.	G	A	100	*	TYPE=SUBSTITUTE
CM032587.1	6936448	.	A	G	100	*	TYPE=SUBSTITUTE
CM032587.1	6936478	.	T	C	100	*	TYPE=SUBSTITUTE
CM032587.1	6936484	.	G	A	100	*	TYPE=SUBSTITUTE
CM032587.1	6936493	.	A	G	100	*	TYPE=SUBSTITUTE
CM032587.1	6936508	.	G	A	100	*	TYPE=SUBSTITUTE
CM032587.1	6936541	.	T	c	100	*	TYPE=SUBSTITUTE
CM032587.1	6936544	.	A	g	100	*	TYPE=SUBSTITUTE
CM032587.1	6936580	.	T	C	100	*	TYPE=SUBSTITUTE
CM032587.1	6936601	.	G	A	100	*	TYPE=SUBSTITUTE
CM032587.1	6936637	.	T	C	100	*	TYPE=SUBSTITUTE
CM032587.1	6936643	.	G	A	100	*	TYPE=SUBSTITUTE
CM032587.1	6936667	.	A	G	100	*	TYPE=SUBSTITUTE
CM032587.1	6936673	.	C	T	100	*	TYPE=SUBSTITUTE
CM032587.1	6936697	.	C	G	100	*	TYPE=SUBSTITUTE
CM032587.1	6936700	.	G	A	100	*	TYPE=SUBSTITUTE
CM032587.1	6936702	.	T	G	100	*	TYPE=SUBSTITUTE
CM032587.1	6936708	.	G	A	100	*	TYPE=SUBSTITUTE
CM032587.1	6936730	.	A	g	100	*	TYPE=SUBSTITUTE
CM032587.1	6936742	.	A	G	100	*	TYPE=SUBSTITUTE
CM032587.1	6936743	.	C	G	100	*	TYPE=SUBSTITUTE
CM032587.1	6936744	.	T	A	100	*	TYPE=SUBSTITUTE
CM032587.1	6936748	.	C	T	100	*	TYPE=SUBSTITUTE
CM032587.1	6936749	.	T	C	100	*	TYPE=SUBSTITUTE
CM032587.1	6936766	.	G	C	100	*	TYPE=SUBSTITUTE
CM032587.1	6936784	.	A	C	100	*	TYPE=SUBSTITUTE
CM032587.1	6936799	.	G	T	100	*	TYPE=SUBSTITUTE
CM032587.1	6936802	.	G	A	100	*	TYPE=SUBSTITUTE
CM032587.1	6936805	.	C	G	100	*	TYPE=SUBSTITUTE
CM032587.1	6936808	.	G	A	100	*	TYPE=SUBSTITUTE
CM032587.1	6936817	.	T	G	100	*	TYPE=SUBSTITUTE
CM032587.1	6936838	.	T	G	100	*	TYPE=SUBSTITUTE
CM032587.1	6936841	.	A	G	100	*	TYPE=SUBSTITUTE
CM032587.1	6936880	.	A	G	100	*	TYPE=SUBSTITUTE
CM032587.1	6936889	.	A	C	100	*	TYPE=SUBSTITUTE
CM032587.1	6936958	.	A	G	100	*	TYPE=SUBSTITUTE
CM032587.1	6936961	.	G	A	100	*	TYPE=SUBSTITUTE
CM032587.1	6936964	.	G	A	100	*	TYPE=SUBSTITUTE
CM032587.1	6936970	.	C	T	100	*	TYPE=SUBSTITUTE
CM032587.1	6937021	.	G	A	100	*	TYPE=SUBSTITUTE
CM032587.1	6937024	.	G	A	100	*	TYPE=SUBSTITUTE
CM032587.1	6937039	.	A	G	100	*	TYPE=SUBSTITUTE
CM032587.1	6937045	.	G	A	100	*	TYPE=SUBSTITUTE
CM032587.1	6937069	.	G	A	100	*	TYPE=SUBSTITUTE
CM032587.1	6937075	.	G	C	100	*	TYPE=SUBSTITUTE
CM032587.1	6937096	.	G	A	100	*	TYPE=SUBSTITUTE
CM032587.1	6937102	.	A	G	100	*	TYPE=SUBSTITUTE
CM032587.1	6937144	.	G	T	100	*	TYPE=SUBSTITUTE
CM032587.1	6937187	.	C	T	100	*	TYPE=SUBSTITUTE
CM032587.1	6937188	.	G	C	100	*	TYPE=SUBSTITUTE
CM032587.1	6937208	.	A	G	100	*	TYPE=SUBSTITUTE
CM032587.1	6937214	.	G	A	100	*	TYPE=SUBSTITUTE
CM032587.1	6937224	.	C	G	100	*	TYPE=SUBSTITUTE
CM032587.1	6937226	.	T	C	100	*	TYPE=SUBSTITUTE
CM032587.1	6937242	.	T	G	100	*	TYPE=SUBSTITUTE

CM032587.1	6937281	.	G	A	100	*	TYPE=SUBSTITUTE
CM032587.1	6937288	.	A	G	100	*	TYPE=SUBSTITUTE
CM032587.1	6937294	.	G	C	100	*	TYPE=SUBSTITUTE
CM032587.1	6937309	.	G	a	100	*	TYPE=SUBSTITUTE
CM032587.1	6937312	.	G	a	100	*	TYPE=SUBSTITUTE
CM032587.1	6937315	.	A	g	100	*	TYPE=SUBSTITUTE
CM032587.1	6937318	.	G	a	100	*	TYPE=SUBSTITUTE
CM032587.1	6937321	.	T	c	100	*	TYPE=SUBSTITUTE
CM032587.1	6937324	.	G	T	100	*	TYPE=SUBSTITUTE
CM032587.1	6937327	.	G	C	100	*	TYPE=SUBSTITUTE
CM032587.1	6937333	.	A	G	100	*	TYPE=SUBSTITUTE
CM032587.1	6937336	.	G	T	100	*	TYPE=SUBSTITUTE
CM032587.1	6937342	.	G	A	100	*	TYPE=SUBSTITUTE
CM032587.1	6937357	.	G	C	100	*	TYPE=SUBSTITUTE
CM032587.1	6937362	.	A	G	100	*	TYPE=SUBSTITUTE
CM032587.1	6937372	.	G	A	100	*	TYPE=SUBSTITUTE
CM032587.1	6937375	.	T	C	100	*	TYPE=SUBSTITUTE
CM032587.1	6937414	.	A	C	100	*	TYPE=SUBSTITUTE
CM032587.1	6937420	.	C	G	100	*	TYPE=SUBSTITUTE
CM032587.1	6937429	.	C	A	100	*	TYPE=SUBSTITUTE
CM032587.1	6937474	.	C	G	100	*	TYPE=SUBSTITUTE
CM032587.1	6937486	.	T	C	100	*	TYPE=SUBSTITUTE
CM032587.1	6937494	.	A	G	100	*	TYPE=SUBSTITUTE
CM032587.1	6937495	.	A	G	100	*	TYPE=SUBSTITUTE
CM032587.1	6937498	.	C	T	100	*	TYPE=SUBSTITUTE
CM032587.1	6937510	.	G	A	100	*	TYPE=SUBSTITUTE
CM032587.1	6937540	.	G	A	100	*	TYPE=SUBSTITUTE
CM032587.1	6937543	.	G	A	100	*	TYPE=SUBSTITUTE
CM032587.1	6937555	.	T	C	100	*	TYPE=SUBSTITUTE
CM032587.1	6937596	.	A	G	100	*	TYPE=SUBSTITUTE
CM032587.1	6937632	.	G	A	100	*	TYPE=SUBSTITUTE
CM032587.1	6937668	.	A	g	100	*	TYPE=SUBSTITUTE
CM032587.1	6937687	.	T	G	100	*	TYPE=SUBSTITUTE
CM032587.1	6937696	.	G	A	100	*	TYPE=SUBSTITUTE
CM032587.1	6937705	.	T	C	100	*	TYPE=SUBSTITUTE
CM032587.1	6937738	.	A	G	100	*	TYPE=SUBSTITUTE
CM032587.1	6937795	.	G	T	100	*	TYPE=SUBSTITUTE
CM032587.1	6937798	.	G	A	100	*	TYPE=SUBSTITUTE
CM032587.1	6937815	.	A	C	100	*	TYPE=SUBSTITUTE
CM032587.1	6937834	.	A	G	100	*	TYPE=SUBSTITUTE
CM032587.1	6937837	.	C	T	100	*	TYPE=SUBSTITUTE
CM032587.1	6937840	.	A	C	100	*	TYPE=SUBSTITUTE
CM032587.1	6937843	.	A	G	100	*	TYPE=SUBSTITUTE
CM032587.1	6937873	.	G	A	100	*	TYPE=SUBSTITUTE
CM032587.1	6937903	.	G	A	100	*	TYPE=SUBSTITUTE
CM032587.1	6937906	.	G	A	100	*	TYPE=SUBSTITUTE
CM032587.1	6937909	.	C	T	100	*	TYPE=SUBSTITUTE
CM032587.1	6937942	.	C	G	100	*	TYPE=SUBSTITUTE
CM032587.1	6937969	.	T	C	100	*	TYPE=SUBSTITUTE
CM032587.1	6937987	.	G	A	100	*	TYPE=SUBSTITUTE
CM032587.1	6937990	.	T	C	100	*	TYPE=SUBSTITUTE
CM032587.1	6937993	.	A	G	100	*	TYPE=SUBSTITUTE
CM032587.1	6937996	.	G	C	100	*	TYPE=SUBSTITUTE
CM032587.1	6938003	.	C	T	100	*	TYPE=SUBSTITUTE
CM032587.1	6938005	.	C	T	100	*	TYPE=SUBSTITUTE
CM032587.1	6938017	.	T	C	100	*	TYPE=SUBSTITUTE
CM032587.1	6938020	.	A	G	100	*	TYPE=SUBSTITUTE
CM032587.1	6938023	.	C	T	100	*	TYPE=SUBSTITUTE
CM032587.1	6938026	.	G	A	100	*	TYPE=SUBSTITUTE
CM032587.1	6938029	.	C	T	100	*	TYPE=SUBSTITUTE
CM032587.1	6938032	.	C	A	100	*	TYPE=SUBSTITUTE
CM032587.1	6938044	.	A	G	100	*	TYPE=SUBSTITUTE
CM032587.1	6938053	.	C	T	100	*	TYPE=SUBSTITUTE
CM032587.1	6938092	.	G	A	100	*	TYPE=SUBSTITUTE

CM032587.1	6938098	.	C	T	100	*	TYPE=SUBSTITUTE
CM032587.1	6938101	.	A	C	100	*	TYPE=SUBSTITUTE
CM032587.1	6938107	.	C	T	100	*	TYPE=SUBSTITUTE
CM032587.1	6938122	.	A	G	100	*	TYPE=SUBSTITUTE
CM032587.1	6938128	.	C	T	100	*	TYPE=SUBSTITUTE
CM032587.1	6938140	.	A	G	100	*	TYPE=SUBSTITUTE
CM032587.1	6938143	.	G	A	100	*	TYPE=SUBSTITUTE
CM032587.1	6938146	.	A	G	100	*	TYPE=SUBSTITUTE
CM032587.1	6938155	.	G	A	100	*	TYPE=SUBSTITUTE
CM032587.1	6938167	.	G	A	100	*	TYPE=SUBSTITUTE
CM032587.1	6938176	.	A	G	100	*	TYPE=SUBSTITUTE
CM032587.1	6938185	.	G	A	100	*	TYPE=SUBSTITUTE
CM032587.1	6938191	.	G	A	100	*	TYPE=SUBSTITUTE
CM032587.1	6938194	.	G	A	100	*	TYPE=SUBSTITUTE
CM032587.1	6938227	.	A	G	100	*	TYPE=SUBSTITUTE
CM032587.1	6938231	.	G	C	100	*	TYPE=SUBSTITUTE
CM032587.1	6938232	.	C	G	100	*	TYPE=SUBSTITUTE
CM032587.1	6938308	.	G	A	100	*	TYPE=SUBSTITUTE
CM032587.1	6938332	.	G	A	100	*	TYPE=SUBSTITUTE
CM032587.1	6938335	.	G	A	100	*	TYPE=SUBSTITUTE
CM032587.1	6938362	.	G	A	100	*	TYPE=SUBSTITUTE
CM032587.1	6938371	.	T	C	100	*	TYPE=SUBSTITUTE
CM032587.1	6938395	.	A	G	100	*	TYPE=SUBSTITUTE
CM032587.1	6938400	.	G	A	100	*	TYPE=SUBSTITUTE
CM032587.1	6938428	.	A	G	100	*	TYPE=SUBSTITUTE
CM032587.1	6938506	.	G	A	100	*	TYPE=SUBSTITUTE
CM032587.1	6938509	.	T	C	100	*	TYPE=SUBSTITUTE
CM032587.1	6938521	.	C	T	100	*	TYPE=SUBSTITUTE
CM032587.1	6938533	.	A	G	100	*	TYPE=SUBSTITUTE
CM032587.1	6938584	.	T	C	100	*	TYPE=SUBSTITUTE
CM032587.1	6938596	.	C	T	100	*	TYPE=SUBSTITUTE
CM032587.1	6938611	.	G	C	100	*	TYPE=SUBSTITUTE
CM032587.1	6938623	.	G	A	100	*	TYPE=SUBSTITUTE
CM032587.1	6938626	.	T	C	100	*	TYPE=SUBSTITUTE
CM032587.1	6938629	.	C	A	100	*	TYPE=SUBSTITUTE
CM032587.1	6938638	.	A	C	100	*	TYPE=SUBSTITUTE
CM032587.1	6938641	.	G	A	100	*	TYPE=SUBSTITUTE
CM032587.1	6938644	.	A	G	100	*	TYPE=SUBSTITUTE
CM032587.1	6938656	.	C	A	100	*	TYPE=SUBSTITUTE
CM032587.1	6938659	.	T	C	100	*	TYPE=SUBSTITUTE
CM032587.1	6938662	.	G	C	100	*	TYPE=SUBSTITUTE
CM032587.1	6938664	.	G	A	100	*	TYPE=SUBSTITUTE
CM032587.1	6938674	.	A	G	100	*	TYPE=SUBSTITUTE
CM032587.1	6938685	.	A	G	100	*	TYPE=SUBSTITUTE
CM032587.1	6938686	.	G	A	100	*	TYPE=SUBSTITUTE
CM032587.1	6938704	.	A	G	100	*	TYPE=SUBSTITUTE
CM032587.1	6938705	.	C	G	100	*	TYPE=SUBSTITUTE
CM032587.1	6938706	.	T	C	100	*	TYPE=SUBSTITUTE
CM032587.1	6938707	.	G	A	100	*	TYPE=SUBSTITUTE
CM032587.1	6938709	.	T	A	100	*	TYPE=SUBSTITUTE
CM032587.1	6938716	.	A	G	100	*	TYPE=SUBSTITUTE
CM032587.1	6938719	.	C	G	100	*	TYPE=SUBSTITUTE
CM032587.1	6938746	.	C	T	100	*	TYPE=SUBSTITUTE
CM032587.1	6938752	.	A	C	100	*	TYPE=SUBSTITUTE
CM032587.1	6938755	.	G	A	100	*	TYPE=SUBSTITUTE
CM032587.1	6938758	.	G	C	100	*	TYPE=SUBSTITUTE
CM032587.1	6938833	.	A	T	100	*	TYPE=SUBSTITUTE
CM032587.1	6938878	.	C	T	100	*	TYPE=SUBSTITUTE
CM032587.1	6938911	.	C	G	100	*	TYPE=SUBSTITUTE
CM032587.1	6938947	.	A	G	100	*	TYPE=SUBSTITUTE
CM032587.1	6938956	.	G	C	100	*	TYPE=SUBSTITUTE
CM032587.1	6938959	.	C	T	100	*	TYPE=SUBSTITUTE
CM032587.1	6938965	.	C	T	100	*	TYPE=SUBSTITUTE
CM032587.1	6939001	.	G	A	100	*	TYPE=SUBSTITUTE

CM032587.1	6939004	.	G	C	100	*	TYPE=SUBSTITUTE
CM032587.1	6939044	.	G	C	100	*	TYPE=SUBSTITUTE
CM032587.1	6939045	.	C	G	100	*	TYPE=SUBSTITUTE
CM032587.1	6939076	.	g	gccgtcgttg	100	*	TYPE=INSERT
CM032587.1	6939094	.	C	g	100	*	TYPE=SUBSTITUTE
CM032587.1	6939095	.	T	g	100	*	TYPE=SUBSTITUTE
CM032587.1	6939106	.	A	G	100	*	TYPE=SUBSTITUTE
CM032587.1	6939112	.	G	A	100	*	TYPE=SUBSTITUTE
CM032587.1	6939113	.	G	T	100	*	TYPE=SUBSTITUTE
CM032587.1	6939114	.	A	C	100	*	TYPE=SUBSTITUTE
CM032587.1	6939117	.	A	G	100	*	TYPE=SUBSTITUTE
CM032587.1	6939126	.	TTTGTTG	T	100	*	TYPE=DELETE
CM032587.1	6939145	.	G	A	100	*	TYPE=SUBSTITUTE
CM032587.1	6939196	.	C	T	100	*	TYPE=SUBSTITUTE
CM032587.1	6939223	.	A	G	100	*	TYPE=SUBSTITUTE
CM032587.1	6939228	.	G	C	100	*	TYPE=SUBSTITUTE
CM032587.1	6939238	.	G	A	100	*	TYPE=SUBSTITUTE
CM032587.1	6939242	.	G	GTCGA	100	*	TYPE=INSERT
CM032587.1	6939243	.	C	G	100	*	TYPE=SUBSTITUTE
CM032587.1	6939252	.	A	G	100	*	TYPE=SUBSTITUTE
CM032587.1	6939255	.	G	A	100	*	TYPE=SUBSTITUTE
CM032587.1	6939256	.	T	C	100	*	TYPE=SUBSTITUTE
CM032587.1	6939259	.	T	G	100	*	TYPE=SUBSTITUTE
CM032587.1	6939261	.	T	C	100	*	TYPE=SUBSTITUTE
CM032587.1	6939262	.	T	C	100	*	TYPE=SUBSTITUTE
CM032587.1	6939263	.	G	T	100	*	TYPE=SUBSTITUTE
CM032587.1	6939265	.	C	T	100	*	TYPE=SUBSTITUTE
CM032587.1	6939296	.	A	G	100	*	TYPE=SUBSTITUTE
CM032587.1	6939301	.	C	G	100	*	TYPE=SUBSTITUTE
CM032587.1	6939310	.	T	C	100	*	TYPE=SUBSTITUTE
CM032587.1	6939313	.	AACC	A	100	*	TYPE=DELETE
CM032587.1	6939320	.	AC	A	100	*	TYPE=DELETE
CM032587.1	6939322	.	T	G	100	*	TYPE=SUBSTITUTE
CM032587.1	6939326	.	T	C	100	*	TYPE=SUBSTITUTE
CM032587.1	6939329	.	G	C	100	*	TYPE=SUBSTITUTE
CM032587.1	6939364	.	TT	T	100	*	TYPE=DELETE
CM032587.1	6939384	.	T	A	100	*	TYPE=SUBSTITUTE
CM032587.1	6939428	.	G	C	100	*	TYPE=SUBSTITUTE
CM032587.1	6939444	.	G	A	100	*	TYPE=SUBSTITUTE
CM032587.1	6939447	.	T	C	100	*	TYPE=SUBSTITUTE
CM032587.1	6939476	.	AA	A	100	*	TYPE=DELETE
CM032587.1	6939479	.	T	A	100	*	TYPE=SUBSTITUTE
CM032587.1	6939484	.	T	A	100	*	TYPE=SUBSTITUTE
CM032587.1	6939560	.	TA	T	100	*	TYPE=DELETE
CM032587.1	6939581	.	C	CGTC	100	*	TYPE=INSERT
CM032587.1	6939586	.	C	T	100	*	TYPE=SUBSTITUTE
CM032587.1	6939593	.	A	C	100	*	TYPE=SUBSTITUTE
CM032587.1	6939613	.	T	C	100	*	TYPE=SUBSTITUTE
CM032587.1	6939631	.	G	T	100	*	TYPE=SUBSTITUTE
CM032587.1	6939632	.	T	C	100	*	TYPE=SUBSTITUTE
CM032587.1	6939633	.	C	A	100	*	TYPE=SUBSTITUTE
CM032587.1	6939635	.	G	C	100	*	TYPE=SUBSTITUTE
CM032587.1	6939643	.	GAA	G	100	*	TYPE=DELETE
CM032587.1	6939648	.	C	G	100	*	TYPE=SUBSTITUTE
CM032587.1	6939651	.	C	G	100	*	TYPE=SUBSTITUTE
CM032587.1	6939665	.	C	CT	100	*	TYPE=INSERT
CM032587.1	6939679	.	C	CCAG	100	*	TYPE=INSERT
CM032587.1	6939682	.	A	G	100	*	TYPE=SUBSTITUTE
CM032587.1	6939684	.	C	G	100	*	TYPE=SUBSTITUTE
CM032587.1	6939717	.	C	T	100	*	TYPE=SUBSTITUTE
CM032587.1	6939769	.	T	C	100	*	TYPE=SUBSTITUTE
CM032587.1	6939893	.	T	G	100	*	TYPE=SUBSTITUTE
CM032587.1	6939988	.	TT	T	100	*	TYPE=DELETE
CM032587.1	6940041	.	C	T	100	*	TYPE=SUBSTITUTE

CM032587.1	6940070	.	C	CC	100	*	TYPE=INSERT
CM032587.1	6940073	.	T	G	100	*	TYPE=SUBSTITUTE
CM032587.1	6940078	.	T	C	100	*	TYPE=SUBSTITUTE
CM032587.1	6940089	.	G	A	100	*	TYPE=SUBSTITUTE
CM032587.1	6940093	.	T	A	100	*	TYPE=SUBSTITUTE
CM032587.1	6940094	.	C	G	100	*	TYPE=SUBSTITUTE
CM032587.1	6940101	.	T	C	100	*	TYPE=SUBSTITUTE
CM032587.1	6940104	.	A	T	100	*	TYPE=SUBSTITUTE
CM032587.1	6940105	.	T	A	100	*	TYPE=SUBSTITUTE
CM032587.1	6940129	.	A	G	100	*	TYPE=SUBSTITUTE
CM032587.1	6940171	.	C	T	100	*	TYPE=SUBSTITUTE
CM032587.1	6940196	.	C	t	100	*	TYPE=SUBSTITUTE
CM032587.1	6940199	.	C	t	100	*	TYPE=SUBSTITUTE
CM032587.1	6940200	.	T	c	100	*	TYPE=SUBSTITUTE
CM032587.1	6940208	.	T	g	100	*	TYPE=SUBSTITUTE
CM032587.1	6940216	.	T	c	100	*	TYPE=SUBSTITUTE
CM032587.1	6940226	.	A	C	100	*	TYPE=SUBSTITUTE
CM032587.1	6940230	.	G	C	100	*	TYPE=SUBSTITUTE
CM032587.1	6940232	.	C	G	100	*	TYPE=SUBSTITUTE
CM032587.1	6940233	.	A	T	100	*	TYPE=SUBSTITUTE
CM032587.1	6940243	.	G	A	100	*	TYPE=SUBSTITUTE
CM032587.1	6940244	.	G	A	100	*	TYPE=SUBSTITUTE
CM032587.1	6940248	.	CCATTTTGAAA	C	100	*	TYPE=DELETE
CM032587.1	6940261	.	C	T	100	*	TYPE=SUBSTITUTE
CM032587.1	6940275	.	C	cacata	100	*	TYPE=INSERT
CM032587.1	6940303	.	A	G	100	*	TYPE=SUBSTITUTE
CM032587.1	6940323	.	T	C	100	*	TYPE=SUBSTITUTE
CM032587.1	6940428	.	G	A	100	*	TYPE=SUBSTITUTE
CM032587.1	6940440	.	T	C	100	*	TYPE=SUBSTITUTE
CM032587.1	6940568	.	A	G	100	*	TYPE=SUBSTITUTE
CM032587.1	6940598	.	A	G	100	*	TYPE=SUBSTITUTE
CM032587.1	6940614	.	T	C	100	*	TYPE=SUBSTITUTE
CM032587.1	6940718	.	A	T	100	*	TYPE=SUBSTITUTE
CM032587.1	6940733	.	A	T	100	*	TYPE=SUBSTITUTE
CM032587.1	6940757	.	C	T	100	*	TYPE=SUBSTITUTE
CM032587.1	6940758	.	T	A	100	*	TYPE=SUBSTITUTE
CM032587.1	6940760	.	A	T	100	*	TYPE=SUBSTITUTE
CM032587.1	6940763	.	C	T	100	*	TYPE=SUBSTITUTE
CM032587.1	6940764	.	A	T	100	*	TYPE=SUBSTITUTE
CM032587.1	6940772	.	T	C	100	*	TYPE=SUBSTITUTE
CM032587.1	6940778	.	G	A	100	*	TYPE=SUBSTITUTE
CM032587.1	6940801	.	T	A	100	*	TYPE=SUBSTITUTE
CM032587.1	6940808	.	A	T	100	*	TYPE=SUBSTITUTE
CM032587.1	6940816	.	T	G	100	*	TYPE=SUBSTITUTE
CM032587.1	6940875	.	C	t	100	*	TYPE=SUBSTITUTE
CM032587.1	6940879	.	CT	C	100	*	TYPE=DELETE
CM032587.1	6940885	.	T	c	100	*	TYPE=SUBSTITUTE
CM032587.1	6940892	.	a	att	100	*	TYPE=INSERT
CM032587.1	6940914	.	T	C	100	*	TYPE=SUBSTITUTE
CM032587.1	6940917	.	C	T	100	*	TYPE=SUBSTITUTE
CM032587.1	6940918	.	A	G	100	*	TYPE=SUBSTITUTE
CM032587.1	6940919	.	G	GT	100	*	TYPE=INSERT
CM032587.1	6940920	.	C	G	100	*	TYPE=SUBSTITUTE
CM032587.1	6940921	.	C	G	100	*	TYPE=SUBSTITUTE
CM032587.1	6940924	.	G	T	100	*	TYPE=SUBSTITUTE
CM032587.1	6940925	.	T	A	100	*	TYPE=SUBSTITUTE
CM032587.1	6940931	.	A	C	100	*	TYPE=SUBSTITUTE
CM032587.1	6940932	.	G	T	100	*	TYPE=SUBSTITUTE
CM032587.1	6940933	.	A	G	100	*	TYPE=SUBSTITUTE
CM032587.1	6940934	.	T	G	100	*	TYPE=SUBSTITUTE
CM032587.1	6940935	.	T	C	100	*	TYPE=SUBSTITUTE
CM032587.1	6940936	.	T	A	100	*	TYPE=SUBSTITUTE
CM032587.1	6940937	.	G	A	100	*	TYPE=SUBSTITUTE
CM032587.1	6940938	.	A	C	100	*	TYPE=SUBSTITUTE

CM032587.1	6940939	.	A	C	100	*	TYPE=SUBSTITUTE
CM032587.1	6940940	.	G	A	100	*	TYPE=SUBSTITUTE
CM032587.1	6940944	.	C	T	100	*	TYPE=SUBSTITUTE
CM032587.1	6940946	.	C	G	100	*	TYPE=SUBSTITUTE
CM032587.1	6940950	.	C	CT	100	*	TYPE=INSERT
CM032587.1	6940955	.	CT	C	100	*	TYPE=DELETE
CM032587.1	6940958	.	C	T	100	*	TYPE=SUBSTITUTE
CM032587.1	6940959	.	C	T	100	*	TYPE=SUBSTITUTE
CM032587.1	6940962	.	GA	G	100	*	TYPE=DELETE
CM032587.1	6941004	.	AA	A	100	*	TYPE=DELETE
CM032587.1	6941047	.	A	C	100	*	TYPE=SUBSTITUTE
CM032587.1	6941053	.	T	A	100	*	TYPE=SUBSTITUTE
CM032587.1	6941063	.	C	T	100	*	TYPE=SUBSTITUTE
CM032587.1	6941065	.	C	T	100	*	TYPE=SUBSTITUTE
CM032587.1	6941101	.	T	G	100	*	TYPE=SUBSTITUTE
CM032587.1	6941104	.	G	T	100	*	TYPE=SUBSTITUTE
CM032587.1	6941105	.	A	G	100	*	TYPE=SUBSTITUTE
CM032587.1	6941138	.	A	G	100	*	TYPE=SUBSTITUTE
CM032587.1	6941170	.	G	A	100	*	TYPE=SUBSTITUTE
CM032587.1	6941218	.	G	GG	100	*	TYPE=INSERT
CM032587.1	6941227	.	TG	T	100	*	TYPE=DELETE
CM032587.1	6941275	.	G	T	100	*	TYPE=SUBSTITUTE
CM032587.1	6941276	.	T	G	100	*	TYPE=SUBSTITUTE
CM032587.1	6941311	.	G	GA	100	*	TYPE=INSERT
CM032587.1	6941329	.	A	g	100	*	TYPE=SUBSTITUTE
CM032587.1	6941333	.	CA	C	100	*	TYPE=DELETE
CM032587.1	6941335	.	G	t	100	*	TYPE=SUBSTITUTE
CM032587.1	6941341	.	c	ctaT	100	*	TYPE=INSERT
CM032587.1	6941345	.	C	G	100	*	TYPE=SUBSTITUTE
CM032587.1	6941377	.	T	C	100	*	TYPE=SUBSTITUTE
CM032587.1	6941429	.	ACT	A	100	*	TYPE=DELETE
CM032587.1	6941472	.	G	c	100	*	TYPE=SUBSTITUTE
CM032587.1	6941516	.	T	A	100	*	TYPE=SUBSTITUTE
CM032587.1	6941524	.	C	CA	100	*	TYPE=INSERT
CM032587.1	6941539	.	G	C	100	*	TYPE=SUBSTITUTE
CM032587.1	6941544	.	GAGTTTCTA	G	100	*	TYPE=DELETE
CM032587.1	6941566	.	T	C	100	*	TYPE=SUBSTITUTE
CM032587.1	6941571	.	G	T	100	*	TYPE=SUBSTITUTE
CM032587.1	6941573	.	A	G	100	*	TYPE=SUBSTITUTE
CM032587.1	6941574	.	C	T	100	*	TYPE=SUBSTITUTE
CM032587.1	6941575	.	T	A	100	*	TYPE=SUBSTITUTE
CM032587.1	6941589	.	T	C	100	*	TYPE=SUBSTITUTE
CM032587.1	6941591	.	C	G	100	*	TYPE=SUBSTITUTE
CM032587.1	6941615	.	T	G	100	*	TYPE=SUBSTITUTE
CM032587.1	6941629	.	C	G	100	*	TYPE=SUBSTITUTE
CM032587.1	6941631	.	T	C	100	*	TYPE=SUBSTITUTE
CM032587.1	6941634	.	A	G	100	*	TYPE=SUBSTITUTE
CM032587.1	6941647	.	C	T	100	*	TYPE=SUBSTITUTE
CM032587.1	6941662	.	T	C	100	*	TYPE=SUBSTITUTE
CM032587.1	6941663	.	G	A	100	*	TYPE=SUBSTITUTE
CM032587.1	6941693	.	C	T	100	*	TYPE=SUBSTITUTE
CM032587.1	6941701	.	T	A	100	*	TYPE=SUBSTITUTE
CM032587.1	6941730	.	T	C	100	*	TYPE=SUBSTITUTE
CM032587.1	6941740	.	A	c	100	*	TYPE=SUBSTITUTE
CM032587.1	6941767	.	C	A	100	*	TYPE=SUBSTITUTE
CM032587.1	6941785	.	A	C	100	*	TYPE=SUBSTITUTE
CM032587.1	6941788	.	T	A	100	*	TYPE=SUBSTITUTE
CM032587.1	6941800	.	C	T	100	*	TYPE=SUBSTITUTE
CM032587.1	6941806	.	T	C	100	*	TYPE=SUBSTITUTE
CM032587.1	6941816	.	C	T	100	*	TYPE=SUBSTITUTE
CM032587.1	6941818	.	G	A	100	*	TYPE=SUBSTITUTE
CM032587.1	6941837	.	T	C	100	*	TYPE=SUBSTITUTE
CM032587.1	6941878	.	T	c	100	*	TYPE=SUBSTITUTE
CM032587.1	6941894	.	T	A	100	*	TYPE=SUBSTITUTE

CM032587.1	6941895	.	C	A	100	*	TYPE=SUBSTITUTE
CM032587.1	6941896	.	C	A	100	*	TYPE=SUBSTITUTE
CM032587.1	6941901	.	C	T	100	*	TYPE=SUBSTITUTE
CM032587.1	6941914	.	A	G	100	*	TYPE=SUBSTITUTE
CM032587.1	6941915	.	G	T	100	*	TYPE=SUBSTITUTE
CM032587.1	6941922	.	C	A	100	*	TYPE=SUBSTITUTE
CM032587.1	6941928	.	C	T	100	*	TYPE=SUBSTITUTE
CM032587.1	6941929	.	A	T	100	*	TYPE=SUBSTITUTE
CM032587.1	6941935	.	TC	T	100	*	TYPE=DELETE
CM032587.1	6941958	.	T	G	100	*	TYPE=SUBSTITUTE
CM032587.1	6941982	.	C	T	100	*	TYPE=SUBSTITUTE
CM032587.1	6942024	.	T	G	100	*	TYPE=SUBSTITUTE
CM032587.1	6942027	.	A	G	100	*	TYPE=SUBSTITUTE
CM032587.1	6942046	.	T	C	100	*	TYPE=SUBSTITUTE
CM032587.1	6942069	.	G	T	100	*	TYPE=SUBSTITUTE
CM032587.1	6942078	.	G	A	100	*	TYPE=SUBSTITUTE
CM032587.1	6942087	.	A	G	100	*	TYPE=SUBSTITUTE
CM032587.1	6942099	.	G	A	100	*	TYPE=SUBSTITUTE
CM032587.1	6942107	.	G	A	100	*	TYPE=SUBSTITUTE
CM032587.1	6942142	.	C	T	100	*	TYPE=SUBSTITUTE
CM032587.1	6942159	.	T	G	100	*	TYPE=SUBSTITUTE
CM032587.1	6942168	.	G	A	100	*	TYPE=SUBSTITUTE
CM032587.1	6942169	.	A	C	100	*	TYPE=SUBSTITUTE
CM032587.1	6942195	.	G	T	100	*	TYPE=SUBSTITUTE
CM032587.1	6942203	.	C	T	100	*	TYPE=SUBSTITUTE
CM032587.1	6942215	.	C	g	100	*	TYPE=SUBSTITUTE
CM032587.1	6942217	.	G	a	100	*	TYPE=SUBSTITUTE
CM032587.1	6942273	.	C	T	100	*	TYPE=SUBSTITUTE
CM032587.1	6942281	.	A	G	100	*	TYPE=SUBSTITUTE
CM032587.1	6942283	.	AG	A	100	*	TYPE=DELETE
CM032587.1	6942285	.	A	T	100	*	TYPE=SUBSTITUTE
CM032587.1	6942288	.	C	T	100	*	TYPE=SUBSTITUTE
CM032587.1	6942289	.	T	C	100	*	TYPE=SUBSTITUTE
CM032587.1	6942294	.	A	G	100	*	TYPE=SUBSTITUTE
CM032587.1	6942295	.	G	A	100	*	TYPE=SUBSTITUTE
CM032587.1	6942303	.	A	G	100	*	TYPE=SUBSTITUTE
CM032587.1	6942306	.	C	A	100	*	TYPE=SUBSTITUTE
CM032587.1	6942307	.	C	T	100	*	TYPE=SUBSTITUTE
CM032587.1	6942311	.	T	G	100	*	TYPE=SUBSTITUTE
CM032587.1	6942313	.	TAATGAAGCCTACACGGCGGA	T	100	*	TYPE=DELETE
CM032587.1	6942334	.	T	C	100	*	TYPE=SUBSTITUTE
CM032587.1	6942338	.	A	G	100	*	TYPE=SUBSTITUTE
CM032587.1	6942345	.	T	C	100	*	TYPE=SUBSTITUTE
CM032587.1	6942354	.	T	C	100	*	TYPE=SUBSTITUTE
CM032587.1	6942358	.	G	A	100	*	TYPE=SUBSTITUTE
CM032587.1	6942359	.	G	T	100	*	TYPE=SUBSTITUTE
CM032587.1	6942364	.	T	C	100	*	TYPE=SUBSTITUTE
CM032587.1	6942368	.	C	A	100	*	TYPE=SUBSTITUTE
CM032587.1	6942389	.	T	C	100	*	TYPE=SUBSTITUTE
CM032587.1	6942391	.	C	T	100	*	TYPE=SUBSTITUTE
CM032587.1	6942393	.	G	A	100	*	TYPE=SUBSTITUTE
CM032587.1	6942398	.	C	A	100	*	TYPE=SUBSTITUTE
CM032587.1	6942399	.	C	A	100	*	TYPE=SUBSTITUTE
CM032587.1	6942407	.	T	C	100	*	TYPE=SUBSTITUTE
CM032587.1	6942410	.	A	T	100	*	TYPE=SUBSTITUTE
CM032587.1	6942417	.	A	G	100	*	TYPE=SUBSTITUTE
CM032587.1	6942425	.	T	A	100	*	TYPE=SUBSTITUTE
CM032587.1	6942426	.	C	T	100	*	TYPE=SUBSTITUTE
CM032587.1	6942429	.	A	G	100	*	TYPE=SUBSTITUTE
CM032587.1	6942431	.	G	A	100	*	TYPE=SUBSTITUTE
CM032587.1	6942432	.	C	A	100	*	TYPE=SUBSTITUTE
CM032587.1	6942434	.	G	A	100	*	TYPE=SUBSTITUTE
CM032587.1	6942435	.	C	T	100	*	TYPE=SUBSTITUTE

CM032587.1	6942438	.	C	G	100	*	TYPE=SUBSTITUTE
CM032587.1	6942442	.	T	A	100	*	TYPE=SUBSTITUTE
CM032587.1	6942443	.	T	C	100	*	TYPE=SUBSTITUTE
CM032587.1	6942445	.	C	T	100	*	TYPE=SUBSTITUTE
CM032587.1	6942446	.	T	A	100	*	TYPE=SUBSTITUTE
CM032587.1	6942447	.	A	G	100	*	TYPE=SUBSTITUTE
CM032587.1	6942452	.	G	T	100	*	TYPE=SUBSTITUTE
CM032587.1	6942453	.	C	A	100	*	TYPE=SUBSTITUTE
CM032587.1	6942455	.	T	G	100	*	TYPE=SUBSTITUTE
CM032587.1	6942456	.	C	A	100	*	TYPE=SUBSTITUTE
CM032587.1	6942467	.	T	C	100	*	TYPE=SUBSTITUTE
CM032587.1	6942469	.	T	C	100	*	TYPE=SUBSTITUTE
CM032587.1	6942471	.	A	G	100	*	TYPE=SUBSTITUTE
CM032587.1	6942472	.	A	G	100	*	TYPE=SUBSTITUTE
CM032587.1	6942473	.	TAATATC	T	100	*	TYPE=DELETE
CM032587.1	6942483	.	GT	G	100	*	TYPE=DELETE
CM032587.1	6942487	.	CAATAA	C	100	*	TYPE=DELETE
CM032587.1	6942496	.	C	T	100	*	TYPE=SUBSTITUTE
CM032587.1	6942499	.	AGTGT	A	100	*	TYPE=DELETE
CM032587.1	6942506	.	ATAATATCAGTGT	A	100	*	TYPE=DELETE
CM032587.1	6942521	.	T	A	100	*	TYPE=SUBSTITUTE
CM032587.1	6942523	.	A	C	100	*	TYPE=SUBSTITUTE
CM032587.1	6942524	.	T	C	100	*	TYPE=SUBSTITUTE
CM032587.1	6942525	.	AGGGGT	A	100	*	TYPE=DELETE
CM032587.1	6942531	.	A	C	100	*	TYPE=SUBSTITUTE
CM032587.1	6942534	.	T	A	100	*	TYPE=SUBSTITUTE
CM032587.1	6942537	.	C	A	100	*	TYPE=SUBSTITUTE
CM032587.1	6942539	.	G	C	100	*	TYPE=SUBSTITUTE
CM032587.1	6942540	.	TGGC	T	100	*	TYPE=DELETE
CM032587.1	6942546	.	A	T	100	*	TYPE=SUBSTITUTE
CM032587.1	6942548	.	A	G	100	*	TYPE=SUBSTITUTE
CM032587.1	6942549	.	A	G	100	*	TYPE=SUBSTITUTE
CM032587.1	6942550	.	A	T	100	*	TYPE=SUBSTITUTE
CM032587.1	6942556	.	C	CTG	100	*	TYPE=INSERT
CM032587.1	6942558	.	G	A	100	*	TYPE=SUBSTITUTE
CM032587.1	6942561	.	G	T	100	*	TYPE=SUBSTITUTE
CM032587.1	6942562	.	C	T	100	*	TYPE=SUBSTITUTE
CM032587.1	6942563	.	A	G	100	*	TYPE=SUBSTITUTE
CM032587.1	6942566	.	G	C	100	*	TYPE=SUBSTITUTE
CM032587.1	6942568	.	AGC	A	100	*	TYPE=DELETE
CM032587.1	6942572	.	C	A	100	*	TYPE=SUBSTITUTE
CM032587.1	6942573	.	G	C	100	*	TYPE=SUBSTITUTE
CM032587.1	6942575	.	AAG	A	100	*	TYPE=DELETE
CM032587.1	6942578	.	T	C	100	*	TYPE=SUBSTITUTE
CM032587.1	6942580	.	A	t	100	*	TYPE=SUBSTITUTE
CM032587.1	6942583	.	TCGAAC	T	100	*	TYPE=DELETE
CM032587.1	6942594	.	A	t	100	*	TYPE=SUBSTITUTE
CM032587.1	6942595	.	A	t	100	*	TYPE=SUBSTITUTE
CM032587.1	6942596	.	C	t	100	*	TYPE=SUBSTITUTE
CM032587.1	6942599	.	A	t	100	*	TYPE=SUBSTITUTE
CM032587.1	6942601	.	A	t	100	*	TYPE=SUBSTITUTE
CM032587.1	6942602	.	C	a	100	*	TYPE=SUBSTITUTE
CM032587.1	6942603	.	A	t	100	*	TYPE=SUBSTITUTE
CM032587.1	6942605	.	A	g	100	*	TYPE=SUBSTITUTE
CM032587.1	6942606	.	C	g	100	*	TYPE=SUBSTITUTE
CM032587.1	6942610	.	G	a	100	*	TYPE=SUBSTITUTE
CM032587.1	6942615	.	G	a	100	*	TYPE=SUBSTITUTE
CM032587.1	6942620	.	A	c	100	*	TYPE=SUBSTITUTE
CM032587.1	6942624	.	G	t	100	*	TYPE=SUBSTITUTE
CM032587.1	6942626	.	C	t	100	*	TYPE=SUBSTITUTE
CM032587.1	6942633	.	T	g	100	*	TYPE=SUBSTITUTE
CM032587.1	6942638	.	G	A	100	*	TYPE=SUBSTITUTE
CM032587.1	6942641	.	C	T	100	*	TYPE=SUBSTITUTE
CM032587.1	6942643	.	T	G	100	*	TYPE=SUBSTITUTE

CM032587.1	6942644	.	G	GTTCAGAGa	100	*	TYPE=INSERT
CM032587.1	6942645	.	C	t	100	*	TYPE=SUBSTITUTE
CM032587.1	6942646	.	C	a	100	*	TYPE=SUBSTITUTE
CM032587.1	6942653	.	G	a	100	*	TYPE=SUBSTITUTE
CM032587.1	6942658	.	C	t	100	*	TYPE=SUBSTITUTE
CM032587.1	6942661	.	T	C	100	*	TYPE=SUBSTITUTE
CM032587.1	6942662	.	A	ACT	100	*	TYPE=INSERT
CM032587.1	6942663	.	T	A	100	*	TYPE=SUBSTITUTE
CM032587.1	6942664	.	G	A	100	*	TYPE=SUBSTITUTE
CM032587.1	6942667	.	C	T	100	*	TYPE=SUBSTITUTE
CM032587.1	6942668	.	T	A	100	*	TYPE=SUBSTITUTE
CM032587.1	6942669	.	C	A	100	*	TYPE=SUBSTITUTE
CM032587.1	6942671	.	A	AG	100	*	TYPE=INSERT
CM032587.1	6942672	.	T	G	100	*	TYPE=SUBSTITUTE
CM032587.1	6942676	.	A	T	100	*	TYPE=SUBSTITUTE
CM032587.1	6942677	.	T	A	100	*	TYPE=SUBSTITUTE
CM032587.1	6942679	.	G	A	100	*	TYPE=SUBSTITUTE
CM032587.1	6942682	.	G	T	100	*	TYPE=SUBSTITUTE
CM032587.1	6942684	.	G	GA	100	*	TYPE=INSERT
CM032587.1	6942688	.	C	G	100	*	TYPE=SUBSTITUTE
CM032587.1	6942690	.	T	C	100	*	TYPE=SUBSTITUTE
CM032587.1	6942691	.	G	C	100	*	TYPE=SUBSTITUTE
CM032587.1	6942692	.	A	AT	100	*	TYPE=INSERT
CM032587.1	6942693	.	G	C	100	*	TYPE=SUBSTITUTE
CM032587.1	6942696	.	A	C	100	*	TYPE=SUBSTITUTE
CM032587.1	6942697	.	G	C	100	*	TYPE=SUBSTITUTE
CM032587.1	6942699	.	C	t	100	*	TYPE=SUBSTITUTE
CM032587.1	6942700	.	A	t	100	*	TYPE=SUBSTITUTE
CM032587.1	6942705	.	c	ctta	100	*	TYPE=INSERT
CM032587.1	6942706	.	C	t	100	*	TYPE=SUBSTITUTE
CM032587.1	6942709	.	a	ac	100	*	TYPE=INSERT
CM032587.1	6942716	.	A	T	100	*	TYPE=SUBSTITUTE
CM032587.1	6942718	.	A	G	100	*	TYPE=SUBSTITUTE
CM032587.1	6942721	.	G	A	100	*	TYPE=SUBSTITUTE
CM032587.1	6942722	.	A	C	100	*	TYPE=SUBSTITUTE
CM032587.1	6942724	.	C	A	100	*	TYPE=SUBSTITUTE
CM032587.1	6942727	.	T	G	100	*	TYPE=SUBSTITUTE
CM032587.1	6942729	.	G	A	100	*	TYPE=SUBSTITUTE
CM032587.1	6942731	.	C	T	100	*	TYPE=SUBSTITUTE
CM032587.1	6942733	.	T	C	100	*	TYPE=SUBSTITUTE
CM032587.1	6942734	.	C	CAGAT	100	*	TYPE=INSERT
CM032587.1	6942736	.	C	T	100	*	TYPE=SUBSTITUTE
CM032587.1	6942738	.	G	GC	100	*	TYPE=INSERT
CM032587.1	6942739	.	G	T	100	*	TYPE=SUBSTITUTE
CM032587.1	6942740	.	A	G	100	*	TYPE=SUBSTITUTE
CM032587.1	6942742	.	A	G	100	*	TYPE=SUBSTITUTE
CM032587.1	6942743	.	A	G	100	*	TYPE=SUBSTITUTE
CM032587.1	6942749	.	C	T	100	*	TYPE=SUBSTITUTE
CM032587.1	6942750	.	T	A	100	*	TYPE=SUBSTITUTE
CM032587.1	6942759	.	T	G	100	*	TYPE=SUBSTITUTE
CM032587.1	6942770	.	A	G	100	*	TYPE=SUBSTITUTE
CM032587.1	6942771	.	C	A	100	*	TYPE=SUBSTITUTE
CM032587.1	6942776	.	A	G	100	*	TYPE=SUBSTITUTE
CM032587.1	6942786	.	A	G	100	*	TYPE=SUBSTITUTE
CM032587.1	6942789	.	A	G	100	*	TYPE=SUBSTITUTE
CM032587.1	6942792	.	C	T	100	*	TYPE=SUBSTITUTE
CM032587.1	6942793	.	G	T	100	*	TYPE=SUBSTITUTE
CM032587.1	6942798	.	C	A	100	*	TYPE=SUBSTITUTE
CM032587.1	6942800	.	A	C	100	*	TYPE=SUBSTITUTE
CM032587.1	6942801	.	G	A	100	*	TYPE=SUBSTITUTE
CM032587.1	6942805	.	C	T	100	*	TYPE=SUBSTITUTE
CM032587.1	6942808	.	A	G	100	*	TYPE=SUBSTITUTE
CM032587.1	6942810	.	G	A	100	*	TYPE=SUBSTITUTE
CM032587.1	6942812	.	A	g	100	*	TYPE=SUBSTITUTE

CM032587.1	6942818	.	t	ttttttattaaaagaagaaaagaaaagatgggcTGTAG	100	*	TYPE=INSERT
CM032587.1	6942821	.	C	T	100	*	TYPE=SUBSTITUTE
CM032587.1	6942824	.	T	G	100	*	TYPE=SUBSTITUTE
CM032587.1	6942825	.	T	A	100	*	TYPE=SUBSTITUTE
CM032587.1	6942826	.	G	A	100	*	TYPE=SUBSTITUTE
CM032587.1	6942828	.	G	A	100	*	TYPE=SUBSTITUTE
CM032587.1	6942831	.	C	T	100	*	TYPE=SUBSTITUTE
CM032587.1	6942832	.	A	G	100	*	TYPE=SUBSTITUTE
CM032587.1	6942836	.	G	A	100	*	TYPE=SUBSTITUTE
CM032587.1	6942839	.	T	G	100	*	TYPE=SUBSTITUTE
CM032587.1	6942843	.	C	A	100	*	TYPE=SUBSTITUTE
CM032587.1	6942846	.	T	G	100	*	TYPE=SUBSTITUTE
CM032587.1	6942851	.	G	T	100	*	TYPE=SUBSTITUTE
CM032587.1	6942867	.	C	T	100	*	TYPE=SUBSTITUTE
CM032587.1	6942870	.	C	T	100	*	TYPE=SUBSTITUTE
CM032587.1	6942873	.	C	T	100	*	TYPE=SUBSTITUTE
CM032587.1	6942874	.	A	T	100	*	TYPE=SUBSTITUTE
CM032587.1	6942878	.	A	C	100	*	TYPE=SUBSTITUTE
CM032587.1	6942879	.	T	C	100	*	TYPE=SUBSTITUTE
CM032587.1	6942890	.	A	C	100	*	TYPE=SUBSTITUTE
CM032587.1	6942892	.	G	GCACATTGAGTT	100	*	TYPE=INSERT
CM032587.1	6942895	.	T	TA	100	*	TYPE=INSERT
CM032587.1	6942897	.	C	G	100	*	TYPE=SUBSTITUTE
CM032587.1	6942900	.	G	A	100	*	TYPE=SUBSTITUTE
CM032587.1	6942902	.	C	T	100	*	TYPE=SUBSTITUTE
CM032587.1	6942904	.	C	G	100	*	TYPE=SUBSTITUTE
CM032587.1	6942909	.	G	A	100	*	TYPE=SUBSTITUTE
CM032587.1	6942910	.	G	T	100	*	TYPE=SUBSTITUTE
CM032587.1	6942917	.	C	CCT	100	*	TYPE=INSERT
CM032587.1	6942921	.	G	A	100	*	TYPE=SUBSTITUTE
CM032587.1	6942922	.	G	C	100	*	TYPE=SUBSTITUTE
CM032587.1	6942925	.	T	C	100	*	TYPE=SUBSTITUTE
CM032587.1	6942930	.	C	T	100	*	TYPE=SUBSTITUTE
CM032587.1	6942932	.	TGTA	T	100	*	TYPE=DELETE
CM032587.1	6942936	.	T	A	100	*	TYPE=SUBSTITUTE
CM032587.1	6942939	.	C	T	100	*	TYPE=SUBSTITUTE
CM032587.1	6942940	.	T	C	100	*	TYPE=SUBSTITUTE
CM032587.1	6942942	.	G	T	100	*	TYPE=SUBSTITUTE
CM032587.1	6942943	.	G	A	100	*	TYPE=SUBSTITUTE
CM032587.1	6942947	.	T	C	100	*	TYPE=SUBSTITUTE
CM032587.1	6942949	.	G	a	100	*	TYPE=SUBSTITUTE
CM032587.1	6942950	.	G	c	100	*	TYPE=SUBSTITUTE
CM032587.1	6942952	.	A	t	100	*	TYPE=SUBSTITUTE
CM032587.1	6942954	.	C	a	100	*	TYPE=SUBSTITUTE
CM032587.1	6942955	.	T	a	100	*	TYPE=SUBSTITUTE
CM032587.1	6942956	.	C	a	100	*	TYPE=SUBSTITUTE
CM032587.1	6942958	.	G	a	100	*	TYPE=SUBSTITUTE
CM032587.1	6942959	.	G	a	100	*	TYPE=SUBSTITUTE
CM032587.1	6942960	.	G	c	100	*	TYPE=SUBSTITUTE
CM032587.1	6942961	.	G	t	100	*	TYPE=SUBSTITUTE
CM032587.1	6942962	.	G	a	100	*	TYPE=SUBSTITUTE
CM032587.1	6942963	.	G	t	100	*	TYPE=SUBSTITUTE
CM032587.1	6942965	.	G	A	100	*	TYPE=SUBSTITUTE
CM032587.1	6942967	.	C	G	100	*	TYPE=SUBSTITUTE
CM032587.1	6942968	.	A	G	100	*	TYPE=SUBSTITUTE
CM032587.1	6942969	.	C	T	100	*	TYPE=SUBSTITUTE
CM032587.1	6942974	.	A	T	100	*	TYPE=SUBSTITUTE
CM032587.1	6942977	.	T	a	100	*	TYPE=SUBSTITUTE
CM032587.1	6942978	.	A	g	100	*	TYPE=SUBSTITUTE
CM032587.1	6942981	.	G	a	100	*	TYPE=SUBSTITUTE
CM032587.1	6942983	.	G	a	100	*	TYPE=SUBSTITUTE
CM032587.1	6942988	.	G	a	100	*	TYPE=SUBSTITUTE
CM032587.1	6942989	.	G	a	100	*	TYPE=SUBSTITUTE

CM032587.1	6942991	.	T	c	100	*	TYPE=SUBSTITUTE
CM032587.1	6942992	.	A	C	100	*	TYPE=SUBSTITUTE
CM032587.1	6942993	.	T	C	100	*	TYPE=SUBSTITUTE
CM032587.1	6942994	.	T	C	100	*	TYPE=SUBSTITUTE
CM032587.1	6942995	.	A	G	100	*	TYPE=SUBSTITUTE
CM032587.1	6942997	.	T	C	100	*	TYPE=SUBSTITUTE
CM032587.1	6943001	.	A	G	100	*	TYPE=SUBSTITUTE
CM032587.1	6943002	.	C	G	100	*	TYPE=SUBSTITUTE
CM032587.1	6943003	.	T	G	100	*	TYPE=SUBSTITUTE
CM032587.1	6943004	.	G	C	100	*	TYPE=SUBSTITUTE
CM032587.1	6943007	.	T	A	100	*	TYPE=SUBSTITUTE
CM032587.1	6943010	.	A	T	100	*	TYPE=SUBSTITUTE
CM032587.1	6943011	.	A	T	100	*	TYPE=SUBSTITUTE
CM032587.1	6943012	.	G	A	100	*	TYPE=SUBSTITUTE
CM032587.1	6943013	.	G	A	100	*	TYPE=SUBSTITUTE
CM032587.1	6943017	.	A	C	100	*	TYPE=SUBSTITUTE
CM032587.1	6943019	.	C	G	100	*	TYPE=SUBSTITUTE
CM032587.1	6943020	.	C	G	100	*	TYPE=SUBSTITUTE
CM032587.1	6943028	.	C	G	100	*	TYPE=SUBSTITUTE
CM032587.1	6943030	.	A	G	100	*	TYPE=SUBSTITUTE
CM032587.1	6943031	.	T	G	100	*	TYPE=SUBSTITUTE
CM032587.1	6943032	.	A	C	100	*	TYPE=SUBSTITUTE
CM032587.1	6943034	.	G	T	100	*	TYPE=SUBSTITUTE
CM032587.1	6943036	.	T	A	100	*	TYPE=SUBSTITUTE
CM032587.1	6943043	.	A	G	100	*	TYPE=SUBSTITUTE
CM032587.1	6943044	.	G	A	100	*	TYPE=SUBSTITUTE
CM032587.1	6943048	.	T	G	100	*	TYPE=SUBSTITUTE
CM032587.1	6943050	.	G	C	100	*	TYPE=SUBSTITUTE
CM032587.1	6943053	.	G	C	100	*	TYPE=SUBSTITUTE
CM032587.1	6943058	.	A	G	100	*	TYPE=SUBSTITUTE
CM032587.1	6943060	.	CTAG	C	100	*	TYPE=DELETE
CM032587.1	6943064	.	T	G	100	*	TYPE=SUBSTITUTE
CM032587.1	6943067	.	TGA	T	100	*	TYPE=DELETE
CM032587.1	6943070	.	G	T	100	*	TYPE=SUBSTITUTE
CM032587.1	6943072	.	T	C	100	*	TYPE=SUBSTITUTE
CM032587.1	6943074	.	A	C	100	*	TYPE=SUBSTITUTE
CM032587.1	6943079	.	A	T	100	*	TYPE=SUBSTITUTE
CM032587.1	6943080	.	C	G	100	*	TYPE=SUBSTITUTE
CM032587.1	6943081	.	T	C	100	*	TYPE=SUBSTITUTE
CM032587.1	6943083	.	TAG	T	100	*	TYPE=DELETE
CM032587.1	6943086	.	G	C	100	*	TYPE=SUBSTITUTE
CM032587.1	6943087	.	A	C	100	*	TYPE=SUBSTITUTE
CM032587.1	6943094	.	C	G	100	*	TYPE=SUBSTITUTE
CM032587.1	6943095	.	A	C	100	*	TYPE=SUBSTITUTE
CM032587.1	6943099	.	A	G	100	*	TYPE=SUBSTITUTE
CM032587.1	6943102	.	A	G	100	*	TYPE=SUBSTITUTE
CM032587.1	6943103	.	C	T	100	*	TYPE=SUBSTITUTE
CM032587.1	6943104	.	T	C	100	*	TYPE=SUBSTITUTE
CM032587.1	6943105	.	G	A	100	*	TYPE=SUBSTITUTE
CM032587.1	6943106	.	A	G	100	*	TYPE=SUBSTITUTE
CM032587.1	6943107	.	C	T	100	*	TYPE=SUBSTITUTE
CM032587.1	6943111	.	A	C	100	*	TYPE=SUBSTITUTE
CM032587.1	6943112	.	T	G	100	*	TYPE=SUBSTITUTE
CM032587.1	6943115	.	G	A	100	*	TYPE=SUBSTITUTE
CM032587.1	6943116	.	T	C	100	*	TYPE=SUBSTITUTE
CM032587.1	6943122	.	T	G	100	*	TYPE=SUBSTITUTE
CM032587.1	6943125	.	T	A	100	*	TYPE=SUBSTITUTE
CM032587.1	6943130	.	CATTTGTACCTATACGATATCGGTCTACTTATTA	C	100	*	TYPE=DELETE
CM032587.1	6943170	.	A	T	100	*	TYPE=SUBSTITUTE
CM032587.1	6943175	.	A	G	100	*	TYPE=SUBSTITUTE
CM032587.1	6943176	.	A	G	100	*	TYPE=SUBSTITUTE
CM032587.1	6943178	.	T	TGC	100	*	TYPE=INSERT
CM032587.1	6943180	.	T	C	100	*	TYPE=SUBSTITUTE

CM032587.1	6943181	.	T	G	100	*	TYPE=SUBSTITUTE
CM032587.1	6943184	.	G	A	100	*	TYPE=SUBSTITUTE
CM032587.1	6943188	.	C	G	100	*	TYPE=SUBSTITUTE
CM032587.1	6943189	.	T	A	100	*	TYPE=SUBSTITUTE
CM032587.1	6943206	.	G	A	100	*	TYPE=SUBSTITUTE
CM032587.1	6943207	.	A	AG	100	*	TYPE=INSERT
CM032587.1	6943208	.	A	C	100	*	TYPE=SUBSTITUTE
CM032587.1	6943209	.	T	C	100	*	TYPE=SUBSTITUTE
CM032587.1	6943212	.	C	A	100	*	TYPE=SUBSTITUTE
CM032587.1	6943216	.	C	G	100	*	TYPE=SUBSTITUTE
CM032587.1	6943221	.	A	AG	100	*	TYPE=INSERT
CM032587.1	6943223	.	C	A	100	*	TYPE=SUBSTITUTE
CM032587.1	6943224	.	C	A	100	*	TYPE=SUBSTITUTE
CM032587.1	6943225	.	T	G	100	*	TYPE=SUBSTITUTE
CM032587.1	6943226	.	T	G	100	*	TYPE=SUBSTITUTE
CM032587.1	6943228	.	C	T	100	*	TYPE=SUBSTITUTE
CM032587.1	6943229	.	G	A	100	*	TYPE=SUBSTITUTE
CM032587.1	6943232	.	C	T	100	*	TYPE=SUBSTITUTE
CM032587.1	6943235	.	G	A	100	*	TYPE=SUBSTITUTE
CM032587.1	6943237	.	A	C	100	*	TYPE=SUBSTITUTE
CM032587.1	6943240	.	CTAGGGGGAA	C	100	*	TYPE=DELETE
CM032587.1	6943250	.	A	C	100	*	TYPE=SUBSTITUTE
CM032587.1	6943251	.	A	C	100	*	TYPE=SUBSTITUTE
CM032587.1	6943252	.	G	T	100	*	TYPE=SUBSTITUTE
CM032587.1	6943257	.	G	A	100	*	TYPE=SUBSTITUTE
CM032587.1	6943258	.	T	G	100	*	TYPE=SUBSTITUTE
CM032587.1	6943260	.	G	A	100	*	TYPE=SUBSTITUTE
CM032587.1	6943262	.	AGTGGTGTCG	A	100	*	TYPE=DELETE
CM032587.1	6943273	.	G	A	100	*	TYPE=SUBSTITUTE
CM032587.1	6943275	.	G	A	100	*	TYPE=SUBSTITUTE
CM032587.1	6943277	.	A	T	100	*	TYPE=SUBSTITUTE
CM032587.1	6943278	.	A	T	100	*	TYPE=SUBSTITUTE
CM032587.1	6943282	.	T	C	100	*	TYPE=SUBSTITUTE
CM032587.1	6943283	.	G	C	100	*	TYPE=SUBSTITUTE
CM032587.1	6943284	.	A	C	100	*	TYPE=SUBSTITUTE
CM032587.1	6943285	.	G	A	100	*	TYPE=SUBSTITUTE
CM032587.1	6943287	.	A	T	100	*	TYPE=SUBSTITUTE
CM032587.1	6943288	.	A	C	100	*	TYPE=SUBSTITUTE
CM032587.1	6943289	.	A	T	100	*	TYPE=SUBSTITUTE
CM032587.1	6943290	.	G	T	100	*	TYPE=SUBSTITUTE
CM032587.1	6943292	.	A	T	100	*	TYPE=SUBSTITUTE
CM032587.1	6943293	.	A	T	100	*	TYPE=SUBSTITUTE
CM032587.1	6943294	.	GCA	G	100	*	TYPE=DELETE
CM032587.1	6943301	.	C	T	100	*	TYPE=SUBSTITUTE
CM032587.1	6943302	.	A	G	100	*	TYPE=SUBSTITUTE
CM032587.1	6943304	.	T	C	100	*	TYPE=SUBSTITUTE
CM032587.1	6943305	.	T	C	100	*	TYPE=SUBSTITUTE
CM032587.1	6943309	.	T	C	100	*	TYPE=SUBSTITUTE
CM032587.1	6943310	.	G	T	100	*	TYPE=SUBSTITUTE
CM032587.1	6943312	.	G	T	100	*	TYPE=SUBSTITUTE
CM032587.1	6943313	.	T	G	100	*	TYPE=SUBSTITUTE
CM032587.1	6943314	.	T	A	100	*	TYPE=SUBSTITUTE
CM032587.1	6943315	.	A	G	100	*	TYPE=SUBSTITUTE
CM032587.1	6943320	.	C	G	100	*	TYPE=SUBSTITUTE
CM032587.1	6943321	.	G	C	100	*	TYPE=SUBSTITUTE
CM032587.1	6943322	.	T	C	100	*	TYPE=SUBSTITUTE
CM032587.1	6943327	.	G	T	100	*	TYPE=SUBSTITUTE
CM032587.1	6943328	.	G	GCC	100	*	TYPE=INSERT
CM032587.1	6943332	.	G	A	100	*	TYPE=SUBSTITUTE
CM032587.1	6943333	.	A	AC	100	*	TYPE=INSERT
CM032587.1	6943334	.	T	G	100	*	TYPE=SUBSTITUTE
CM032587.1	6943336	.	G	A	100	*	TYPE=SUBSTITUTE
CM032587.1	6943339	.	a	ac	100	*	TYPE=INSERT
CM032587.1	6943344	.	G	a	100	*	TYPE=SUBSTITUTE

CM032587.1	6943345	.	C	a	100	*	TYPE=SUBSTITUTE
CM032587.1	6943347	.	C	a	100	*	TYPE=SUBSTITUTE
CM032587.1	6943348	.	TGG	T	100	*	TYPE=DELETE
CM032587.1	6943352	.	C	a	100	*	TYPE=SUBSTITUTE
CM032587.1	6943353	.	A	t	100	*	TYPE=SUBSTITUTE
CM032587.1	6943357	.	A	t	100	*	TYPE=SUBSTITUTE
CM032587.1	6943359	.	T	a	100	*	TYPE=SUBSTITUTE
CM032587.1	6943360	.	T	g	100	*	TYPE=SUBSTITUTE
CM032587.1	6943361	.	T	A	100	*	TYPE=SUBSTITUTE
CM032587.1	6943362	.	T	A	100	*	TYPE=SUBSTITUTE
CM032587.1	6943365	.	T	G	100	*	TYPE=SUBSTITUTE
CM032587.1	6943368	.	C	T	100	*	TYPE=SUBSTITUTE
CM032587.1	6943372	.	T	C	100	*	TYPE=SUBSTITUTE
CM032587.1	6943374	.	T	A	100	*	TYPE=SUBSTITUTE
CM032587.1	6943375	.	T	C	100	*	TYPE=SUBSTITUTE
CM032587.1	6943377	.	T	A	100	*	TYPE=SUBSTITUTE
CM032587.1	6943382	.	C	A	100	*	TYPE=SUBSTITUTE
CM032587.1	6943386	.	T	C	100	*	TYPE=SUBSTITUTE
CM032587.1	6943388	.	G	C	100	*	TYPE=SUBSTITUTE
CM032587.1	6943389	.	A	C	100	*	TYPE=SUBSTITUTE
CM032587.1	6943392	.	C	T	100	*	TYPE=SUBSTITUTE
CM032587.1	6943395	.	G	A	100	*	TYPE=SUBSTITUTE
CM032587.1	6943399	.	C	T	100	*	TYPE=SUBSTITUTE
CM032587.1	6943400	.	G	A	100	*	TYPE=SUBSTITUTE
CM032587.1	6943401	.	T	A	100	*	TYPE=SUBSTITUTE
CM032587.1	6943402	.	T	C	100	*	TYPE=SUBSTITUTE
CM032587.1	6943404	.	A	G	100	*	TYPE=SUBSTITUTE
CM032587.1	6943409	.	G	A	100	*	TYPE=SUBSTITUTE
CM032587.1	6943412	.	C	T	100	*	TYPE=SUBSTITUTE
CM032587.1	6943414	.	C	T	100	*	TYPE=SUBSTITUTE
CM032587.1	6943415	.	G	A	100	*	TYPE=SUBSTITUTE
CM032587.1	6943416	.	C	T	100	*	TYPE=SUBSTITUTE
CM032587.1	6943418	.	A	C	100	*	TYPE=SUBSTITUTE
CM032587.1	6943419	.	C	T	100	*	TYPE=SUBSTITUTE
CM032587.1	6943430	.	T	C	100	*	TYPE=SUBSTITUTE
CM032587.1	6943437	.	C	T	100	*	TYPE=SUBSTITUTE
CM032587.1	6943439	.	A	G	100	*	TYPE=SUBSTITUTE
CM032587.1	6943446	.	A	C	100	*	TYPE=SUBSTITUTE
CM032587.1	6943447	.	A	G	100	*	TYPE=SUBSTITUTE
CM032587.1	6943452	.	G	A	100	*	TYPE=SUBSTITUTE
CM032587.1	6943462	.	C	A	100	*	TYPE=SUBSTITUTE
CM032587.1	6943469	.	G	C	100	*	TYPE=SUBSTITUTE
CM032587.1	6943473	.	A	T	100	*	TYPE=SUBSTITUTE
CM032587.1	6943475	.	C	G	100	*	TYPE=SUBSTITUTE
CM032587.1	6943480	.	A	G	100	*	TYPE=SUBSTITUTE
CM032587.1	6943483	.	A	T	100	*	TYPE=SUBSTITUTE
CM032587.1	6943484	.	C	T	100	*	TYPE=SUBSTITUTE
CM032587.1	6943485	.	C	G	100	*	TYPE=SUBSTITUTE
CM032587.1	6943486	.	T	G	100	*	TYPE=SUBSTITUTE
CM032587.1	6943487	.	T	A	100	*	TYPE=SUBSTITUTE
CM032587.1	6943489	.	A	G	100	*	TYPE=SUBSTITUTE
CM032587.1	6943490	.	A	C	100	*	TYPE=SUBSTITUTE
CM032587.1	6943501	.	T	C	100	*	TYPE=SUBSTITUTE
CM032587.1	6943503	.	G	A	100	*	TYPE=SUBSTITUTE
CM032587.1	6943515	.	C	g	100	*	TYPE=SUBSTITUTE
CM032587.1	6943516	.	C	g	100	*	TYPE=SUBSTITUTE
CM032587.1	6943523	.	G	a	100	*	TYPE=SUBSTITUTE
CM032587.1	6943528	.	A	g	100	*	TYPE=SUBSTITUTE
CM032587.1	6943529	.	G	a	100	*	TYPE=SUBSTITUTE
CM032587.1	6943532	.	G	c	100	*	TYPE=SUBSTITUTE
CM032587.1	6943533	.	C	A	100	*	TYPE=SUBSTITUTE
CM032587.1	6943536	.	A	G	100	*	TYPE=SUBSTITUTE
CM032587.1	6943552	.	T	C	100	*	TYPE=SUBSTITUTE
CM032587.1	6943554	.	T	A	100	*	TYPE=SUBSTITUTE

CM032587.1	6943556	.	G	A	100	*	TYPE=SUBSTITUTE
CM032587.1	6943558	.	G	A	100	*	TYPE=SUBSTITUTE
CM032587.1	6943563	.	T	A	100	*	TYPE=SUBSTITUTE
CM032587.1	6943567	.	A	AT	100	*	TYPE=INSERT
CM032587.1	6943571	.	T	A	100	*	TYPE=SUBSTITUTE
CM032587.1	6943575	.	C	CTTCAAA	100	*	TYPE=INSERT
CM032587.1	6943578	.	T	TCAA	100	*	TYPE=INSERT
CM032587.1	6943580	.	G	C	100	*	TYPE=SUBSTITUTE
CM032587.1	6943582	.	A	T	100	*	TYPE=SUBSTITUTE
CM032587.1	6943583	.	A	T	100	*	TYPE=SUBSTITUTE
CM032587.1	6943586	.	G	A	100	*	TYPE=SUBSTITUTE
CM032587.1	6943590	.	T	A	100	*	TYPE=SUBSTITUTE
CM032587.1	6943591	.	A	AT	100	*	TYPE=INSERT
CM032587.1	6943593	.	T	C	100	*	TYPE=SUBSTITUTE
CM032587.1	6943595	.	G	A	100	*	TYPE=SUBSTITUTE
CM032587.1	6943596	.	G	C	100	*	TYPE=SUBSTITUTE
CM032587.1	6943598	.	A	G	100	*	TYPE=SUBSTITUTE
CM032587.1	6943599	.	T	C	100	*	TYPE=SUBSTITUTE
CM032587.1	6943600	.	C	T	100	*	TYPE=SUBSTITUTE
CM032587.1	6943603	.	A	AGT	100	*	TYPE=INSERT
CM032587.1	6943604	.	C	G	100	*	TYPE=SUBSTITUTE
CM032587.1	6943605	.	A	T	100	*	TYPE=SUBSTITUTE
CM032587.1	6943610	.	C	T	100	*	TYPE=SUBSTITUTE
CM032587.1	6943611	.	C	CGT	100	*	TYPE=INSERT
CM032587.1	6943612	.	T	G	100	*	TYPE=SUBSTITUTE
CM032587.1	6943616	.	G	GCCCGCCA	100	*	TYPE=INSERT
CM032587.1	6943618	.	T	C	100	*	TYPE=SUBSTITUTE
CM032587.1	6943619	.	G	A	100	*	TYPE=SUBSTITUTE
CM032587.1	6943620	.	T	C	100	*	TYPE=SUBSTITUTE
CM032587.1	6943623	.	A	T	100	*	TYPE=SUBSTITUTE
CM032587.1	6943626	.	A	C	100	*	TYPE=SUBSTITUTE
CM032587.1	6943635	.	G	A	100	*	TYPE=SUBSTITUTE
CM032587.1	6943636	.	G	T	100	*	TYPE=SUBSTITUTE
CM032587.1	6943641	.	C	T	100	*	TYPE=SUBSTITUTE
CM032587.1	6943644	.	C	T	100	*	TYPE=SUBSTITUTE
CM032587.1	6943649	.	A	G	100	*	TYPE=SUBSTITUTE
CM032587.1	6943653	.	A	C	100	*	TYPE=SUBSTITUTE
CM032587.1	6943660	.	A	G	100	*	TYPE=SUBSTITUTE
CM032587.1	6943665	.	G	A	100	*	TYPE=SUBSTITUTE
CM032587.1	6943666	.	G	A	100	*	TYPE=SUBSTITUTE
CM032587.1	6943670	.	G	A	100	*	TYPE=SUBSTITUTE
CM032587.1	6943680	.	T	C	100	*	TYPE=SUBSTITUTE
CM032587.1	6943681	.	T	C	100	*	TYPE=SUBSTITUTE
CM032587.1	6943686	.	A	C	100	*	TYPE=SUBSTITUTE
CM032587.1	6943688	.	T	C	100	*	TYPE=SUBSTITUTE
CM032587.1	6943690	.	T	C	100	*	TYPE=SUBSTITUTE
CM032587.1	6943695	.	T	C	100	*	TYPE=SUBSTITUTE
CM032587.1	6943698	.	T	A	100	*	TYPE=SUBSTITUTE
CM032587.1	6943700	.	A	G	100	*	TYPE=SUBSTITUTE
CM032587.1	6943702	.	A	G	100	*	TYPE=SUBSTITUTE
CM032587.1	6943705	.	G	C	100	*	TYPE=SUBSTITUTE
CM032587.1	6943706	.	C	T	100	*	TYPE=SUBSTITUTE
CM032587.1	6943716	.	A	G	100	*	TYPE=SUBSTITUTE
CM032587.1	6943719	.	C	T	100	*	TYPE=SUBSTITUTE
CM032587.1	6943721	.	G	A	100	*	TYPE=SUBSTITUTE
CM032587.1	6943726	.	T	C	100	*	TYPE=SUBSTITUTE
CM032587.1	6943731	.	T	C	100	*	TYPE=SUBSTITUTE
CM032587.1	6943736	.	G	A	100	*	TYPE=SUBSTITUTE
CM032587.1	6943739	.	G	A	100	*	TYPE=SUBSTITUTE
CM032587.1	6943743	.	T	C	100	*	TYPE=SUBSTITUTE
CM032587.1	6943746	.	A	G	100	*	TYPE=SUBSTITUTE
CM032587.1	6943747	.	G	T	100	*	TYPE=SUBSTITUTE
CM032587.1	6943749	.	T	C	100	*	TYPE=SUBSTITUTE
CM032587.1	6943755	.	A	G	100	*	TYPE=SUBSTITUTE

CM032587.1	6943762	.	G	A	100	*	TYPE=SUBSTITUTE
CM032587.1	6943764	.	T	C	100	*	TYPE=SUBSTITUTE
CM032587.1	6943767	.	G	C	100	*	TYPE=SUBSTITUTE
CM032587.1	6943771	.	A	C	100	*	TYPE=SUBSTITUTE
CM032587.1	6943773	.	G	A	100	*	TYPE=SUBSTITUTE
CM032587.1	6943779	.	C	T	100	*	TYPE=SUBSTITUTE
CM032587.1	6943781	.	T	C	100	*	TYPE=SUBSTITUTE
CM032587.1	6943782	.	A	G	100	*	TYPE=SUBSTITUTE
CM032587.1	6943783	.	G	C	100	*	TYPE=SUBSTITUTE
CM032587.1	6943784	.	G	A	100	*	TYPE=SUBSTITUTE
CM032587.1	6943785	.	C	T	100	*	TYPE=SUBSTITUTE
CM032587.1	6943789	.	A	G	100	*	TYPE=SUBSTITUTE
CM032587.1	6943790	.	T	C	100	*	TYPE=SUBSTITUTE
CM032587.1	6943791	.	G	T	100	*	TYPE=SUBSTITUTE
CM032587.1	6943792	.	G	A	100	*	TYPE=SUBSTITUTE
CM032587.1	6943805	.	T	C	100	*	TYPE=SUBSTITUTE
CM032587.1	6943813	.	G	A	100	*	TYPE=SUBSTITUTE
CM032587.1	6943816	.	A	G	100	*	TYPE=SUBSTITUTE
CM032587.1	6943817	.	A	G	100	*	TYPE=SUBSTITUTE
CM032587.1	6943820	.	T	A	100	*	TYPE=SUBSTITUTE
CM032587.1	6943824	.	T	A	100	*	TYPE=SUBSTITUTE
CM032587.1	6943828	.	C	T	100	*	TYPE=SUBSTITUTE
CM032587.1	6943830	.	C	G	100	*	TYPE=SUBSTITUTE
CM032587.1	6943835	.	G	A	100	*	TYPE=SUBSTITUTE
CM032587.1	6943837	.	G	A	100	*	TYPE=SUBSTITUTE
CM032587.1	6943839	.	A	G	100	*	TYPE=SUBSTITUTE
CM032587.1	6943841	.	T	G	100	*	TYPE=SUBSTITUTE
CM032587.1	6943848	.	C	G	100	*	TYPE=SUBSTITUTE
CM032587.1	6943851	.	C	T	100	*	TYPE=SUBSTITUTE
CM032587.1	6943857	.	T	C	100	*	TYPE=SUBSTITUTE
CM032587.1	6943868	.	A	T	100	*	TYPE=SUBSTITUTE
CM032587.1	6943870	.	T	A	100	*	TYPE=SUBSTITUTE
CM032587.1	6943871	.	C	A	100	*	TYPE=SUBSTITUTE
CM032587.1	6943873	.	A	G	100	*	TYPE=SUBSTITUTE
CM032587.1	6943882	.	A	G	100	*	TYPE=SUBSTITUTE
CM032587.1	6943883	.	G	A	100	*	TYPE=SUBSTITUTE
CM032587.1	6943891	.	A	T	100	*	TYPE=SUBSTITUTE
CM032587.1	6943892	.	A	G	100	*	TYPE=SUBSTITUTE
CM032587.1	6943893	.	C	T	100	*	TYPE=SUBSTITUTE
CM032587.1	6943896	.	A	G	100	*	TYPE=SUBSTITUTE
CM032587.1	6943903	.	C	A	100	*	TYPE=SUBSTITUTE
CM032587.1	6943909	.	C	A	100	*	TYPE=SUBSTITUTE
CM032587.1	6943910	.	A	G	100	*	TYPE=SUBSTITUTE
CM032587.1	6943916	.	G	A	100	*	TYPE=SUBSTITUTE
CM032587.1	6943923	.	C	T	100	*	TYPE=SUBSTITUTE
CM032587.1	6943934	.	C	A	100	*	TYPE=SUBSTITUTE
CM032587.1	6943938	.	G	A	100	*	TYPE=SUBSTITUTE
CM032587.1	6943940	.	C	T	100	*	TYPE=SUBSTITUTE
CM032587.1	6943951	.	C	T	100	*	TYPE=SUBSTITUTE
CM032587.1	6943956	.	G	A	100	*	TYPE=SUBSTITUTE
CM032587.1	6943959	.	C	T	100	*	TYPE=SUBSTITUTE
CM032587.1	6943962	.	A	G	100	*	TYPE=SUBSTITUTE
CM032587.1	6943977	.	G	A	100	*	TYPE=SUBSTITUTE
CM032587.1	6943982	.	T	C	100	*	TYPE=SUBSTITUTE
CM032587.1	6943985	.	A	G	100	*	TYPE=SUBSTITUTE
CM032587.1	6943989	.	A	C	100	*	TYPE=SUBSTITUTE
CM032587.1	6943992	.	C	T	100	*	TYPE=SUBSTITUTE
CM032587.1	6943993	.	G	A	100	*	TYPE=SUBSTITUTE
CM032587.1	6943999	.	T	C	100	*	TYPE=SUBSTITUTE
CM032587.1	6944005	.	A	G	100	*	TYPE=SUBSTITUTE
CM032587.1	6944006	.	G	A	100	*	TYPE=SUBSTITUTE
CM032587.1	6944011	.	C	T	100	*	TYPE=SUBSTITUTE
CM032587.1	6944013	.	A	G	100	*	TYPE=SUBSTITUTE
CM032587.1	6944015	.	A	G	100	*	TYPE=SUBSTITUTE

CM032587.1	6944016	.	G	A	100	*	TYPE=SUBSTITUTE
CM032587.1	6944017	.	C	G	100	*	TYPE=SUBSTITUTE
CM032587.1	6944018	.	G	A	100	*	TYPE=SUBSTITUTE
CM032587.1	6944040	.	A	G	100	*	TYPE=SUBSTITUTE
CM032587.1	6944042	.	G	C	100	*	TYPE=SUBSTITUTE
CM032587.1	6944046	.	G	C	100	*	TYPE=SUBSTITUTE
CM032587.1	6944058	.	G	T	100	*	TYPE=SUBSTITUTE
CM032587.1	6944063	.	T	C	100	*	TYPE=SUBSTITUTE
CM032587.1	6944066	.	TC	T	100	*	TYPE=DELETE
CM032587.1	6944068	.	A	T	100	*	TYPE=SUBSTITUTE
CM032587.1	6944073	.	CCA	C	100	*	TYPE=DELETE
CM032587.1	6944077	.	G	C	100	*	TYPE=SUBSTITUTE
CM032587.1	6944079	.	T	C	100	*	TYPE=SUBSTITUTE
CM032587.1	6944084	.	C	A	100	*	TYPE=SUBSTITUTE
CM032587.1	6944085	.	A	T	100	*	TYPE=SUBSTITUTE
CM032587.1	6944087	.	G	A	100	*	TYPE=SUBSTITUTE
CM032587.1	6944090	.	A	T	100	*	TYPE=SUBSTITUTE
CM032587.1	6944092	.	T	C	100	*	TYPE=SUBSTITUTE
CM032587.1	6944093	.	C	G	100	*	TYPE=SUBSTITUTE
CM032587.1	6944096	.	A	ATG	100	*	TYPE=INSERT
CM032587.1	6944098	.	T	G	100	*	TYPE=SUBSTITUTE
CM032587.1	6944110	.	G	A	100	*	TYPE=SUBSTITUTE
CM032587.1	6944111	.	A	C	100	*	TYPE=SUBSTITUTE
CM032587.1	6944114	.	C	T	100	*	TYPE=SUBSTITUTE
CM032587.1	6944118	.	T	C	100	*	TYPE=SUBSTITUTE
CM032587.1	6944120	.	A	G	100	*	TYPE=SUBSTITUTE
CM032587.1	6944125	.	A	T	100	*	TYPE=SUBSTITUTE
CM032587.1	6944138	.	G	A	100	*	TYPE=SUBSTITUTE
CM032587.1	6944140	.	G	T	100	*	TYPE=SUBSTITUTE
CM032587.1	6944153	.	G	C	100	*	TYPE=SUBSTITUTE
CM032587.1	6944158	.	A	C	100	*	TYPE=SUBSTITUTE
CM032587.1	6944166	.	G	C	100	*	TYPE=SUBSTITUTE
CM032587.1	6944177	.	C	T	100	*	TYPE=SUBSTITUTE
CM032587.1	6944181	.	T	C	100	*	TYPE=SUBSTITUTE
CM032587.1	6944197	.	T	C	100	*	TYPE=SUBSTITUTE
CM032587.1	6944198	.	T	G	100	*	TYPE=SUBSTITUTE
CM032587.1	6944204	.	A	G	100	*	TYPE=SUBSTITUTE
CM032587.1	6944208	.	G	A	100	*	TYPE=SUBSTITUTE
CM032587.1	6944210	.	T	C	100	*	TYPE=SUBSTITUTE
CM032587.1	6944218	.	G	A	100	*	TYPE=SUBSTITUTE
CM032587.1	6944221	.	G	A	100	*	TYPE=SUBSTITUTE
CM032587.1	6944225	.	T	C	100	*	TYPE=SUBSTITUTE
CM032587.1	6944229	.	G	A	100	*	TYPE=SUBSTITUTE
CM032587.1	6944230	.	A	G	100	*	TYPE=SUBSTITUTE
CM032587.1	6944240	.	G	A	100	*	TYPE=SUBSTITUTE
CM032587.1	6944251	.	C	G	100	*	TYPE=SUBSTITUTE
CM032587.1	6944253	.	A	G	100	*	TYPE=SUBSTITUTE
CM032587.1	6944254	.	A	G	100	*	TYPE=SUBSTITUTE
CM032587.1	6944262	.	A	G	100	*	TYPE=SUBSTITUTE
CM032587.1	6944263	.	A	G	100	*	TYPE=SUBSTITUTE
CM032587.1	6944264	.	T	C	100	*	TYPE=SUBSTITUTE
CM032587.1	6944267	.	C	T	100	*	TYPE=SUBSTITUTE
CM032587.1	6944268	.	A	G	100	*	TYPE=SUBSTITUTE
CM032587.1	6944273	.	C	A	100	*	TYPE=SUBSTITUTE
CM032587.1	6944276	.	A	T	100	*	TYPE=SUBSTITUTE
CM032587.1	6944282	.	T	C	100	*	TYPE=SUBSTITUTE
CM032587.1	6944284	.	A	T	100	*	TYPE=SUBSTITUTE
CM032587.1	6944285	.	G	A	100	*	TYPE=SUBSTITUTE
CM032587.1	6944287	.	G	C	100	*	TYPE=SUBSTITUTE
CM032587.1	6944288	.	T	C	100	*	TYPE=SUBSTITUTE
CM032587.1	6944289	.	C	A	100	*	TYPE=SUBSTITUTE
CM032587.1	6944296	.	G	T	100	*	TYPE=SUBSTITUTE
CM032587.1	6944300	.	G	A	100	*	TYPE=SUBSTITUTE
CM032587.1	6944303	.	C	T	100	*	TYPE=SUBSTITUTE

CM032587.1	6944306	.	G	T	100	*	TYPE=SUBSTITUTE
CM032587.1	6944322	.	A	G	100	*	TYPE=SUBSTITUTE
CM032587.1	6944324	.	A	G	100	*	TYPE=SUBSTITUTE
CM032587.1	6944335	.	A	C	100	*	TYPE=SUBSTITUTE
CM032587.1	6944348	.	C	G	100	*	TYPE=SUBSTITUTE
CM032587.1	6944349	.	A	G	100	*	TYPE=SUBSTITUTE
CM032587.1	6944350	.	C	A	100	*	TYPE=SUBSTITUTE
CM032587.1	6944364	.	A	G	100	*	TYPE=SUBSTITUTE
CM032587.1	6944366	.	G	T	100	*	TYPE=SUBSTITUTE
CM032587.1	6944379	.	G	A	100	*	TYPE=SUBSTITUTE
CM032587.1	6944380	.	C	G	100	*	TYPE=SUBSTITUTE
CM032587.1	6944381	.	T	G	100	*	TYPE=SUBSTITUTE
CM032587.1	6944382	.	C	G	100	*	TYPE=SUBSTITUTE
CM032587.1	6944384	.	G	A	100	*	TYPE=SUBSTITUTE
CM032587.1	6944385	.	G	A	100	*	TYPE=SUBSTITUTE
CM032587.1	6944386	.	A	G	100	*	TYPE=SUBSTITUTE
CM032587.1	6944388	.	A	G	100	*	TYPE=SUBSTITUTE
CM032587.1	6944389	.	A	G	100	*	TYPE=SUBSTITUTE
CM032587.1	6944390	.	T	C	100	*	TYPE=SUBSTITUTE
CM032587.1	6944391	.	G	A	100	*	TYPE=SUBSTITUTE
CM032587.1	6944392	.	A	T	100	*	TYPE=SUBSTITUTE
CM032587.1	6944396	.	A	T	100	*	TYPE=SUBSTITUTE
CM032587.1	6944397	.	T	C	100	*	TYPE=SUBSTITUTE
CM032587.1	6944400	.	A	G	100	*	TYPE=SUBSTITUTE
CM032587.1	6944401	.	G	A	100	*	TYPE=SUBSTITUTE
CM032587.1	6944404	.	G	A	100	*	TYPE=SUBSTITUTE
CM032587.1	6944405	.	A	T	100	*	TYPE=SUBSTITUTE
CM032587.1	6944406	.	G	A	100	*	TYPE=SUBSTITUTE
CM032587.1	6944408	.	G	C	100	*	TYPE=SUBSTITUTE
CM032587.1	6944409	.	T	C	100	*	TYPE=SUBSTITUTE
CM032587.1	6944410	.	G	A	100	*	TYPE=SUBSTITUTE
CM032587.1	6944418	.	C	T	100	*	TYPE=SUBSTITUTE
CM032587.1	6944420	.	G	A	100	*	TYPE=SUBSTITUTE
CM032587.1	6944423	.	T	C	100	*	TYPE=SUBSTITUTE
CM032587.1	6944426	.	T	C	100	*	TYPE=SUBSTITUTE
CM032587.1	6944429	.	G	A	100	*	TYPE=SUBSTITUTE
CM032587.1	6944433	.	G	A	100	*	TYPE=SUBSTITUTE
CM032587.1	6944449	.	C	A	100	*	TYPE=SUBSTITUTE
CM032587.1	6944450	.	G	T	100	*	TYPE=SUBSTITUTE
CM032587.1	6944451	.	A	G	100	*	TYPE=SUBSTITUTE
CM032587.1	6944453	.	C	T	100	*	TYPE=SUBSTITUTE
CM032587.1	6944459	.	C	G	100	*	TYPE=SUBSTITUTE
CM032587.1	6944462	.	G	A	100	*	TYPE=SUBSTITUTE
CM032587.1	6944463	.	C	A	100	*	TYPE=SUBSTITUTE
CM032587.1	6944466	.	C	T	100	*	TYPE=SUBSTITUTE
CM032587.1	6944470	.	C	T	100	*	TYPE=SUBSTITUTE
CM032587.1	6944473	.	A	C	100	*	TYPE=SUBSTITUTE
CM032587.1	6944474	.	A	T	100	*	TYPE=SUBSTITUTE
CM032587.1	6944477	.	T	C	100	*	TYPE=SUBSTITUTE
CM032587.1	6944489	.	T	A	100	*	TYPE=SUBSTITUTE
CM032587.1	6944495	.	A	C	100	*	TYPE=SUBSTITUTE
CM032587.1	6944513	.	C	A	100	*	TYPE=SUBSTITUTE
CM032587.1	6944519	.	C	T	100	*	TYPE=SUBSTITUTE
CM032587.1	6944522	.	C	T	100	*	TYPE=SUBSTITUTE
CM032587.1	6944525	.	T	G	100	*	TYPE=SUBSTITUTE
CM032587.1	6944536	.	A	G	100	*	TYPE=SUBSTITUTE
CM032587.1	6944537	.	G	C	100	*	TYPE=SUBSTITUTE
CM032587.1	6944543	.	T	G	100	*	TYPE=SUBSTITUTE
CM032587.1	6944546	.	G	A	100	*	TYPE=SUBSTITUTE
CM032587.1	6944558	.	T	G	100	*	TYPE=SUBSTITUTE
CM032587.1	6944559	.	G	A	100	*	TYPE=SUBSTITUTE
CM032587.1	6944560	.	C	T	100	*	TYPE=SUBSTITUTE
CM032587.1	6944564	.	A	G	100	*	TYPE=SUBSTITUTE
CM032587.1	6944566	.	C	A	100	*	TYPE=SUBSTITUTE

CM032587.1	6944573	.	A	C	100	*	TYPE=SUBSTITUTE
CM032587.1	6944576	.	T	C	100	*	TYPE=SUBSTITUTE
CM032587.1	6944585	.	C	T	100	*	TYPE=SUBSTITUTE
CM032587.1	6944609	.	C	T	100	*	TYPE=SUBSTITUTE
CM032587.1	6944616	.	C	G	100	*	TYPE=SUBSTITUTE
CM032587.1	6944631	.	A	C	100	*	TYPE=SUBSTITUTE
CM032587.1	6944632	.	G	A	100	*	TYPE=SUBSTITUTE
CM032587.1	6944633	.	A	G	100	*	TYPE=SUBSTITUTE
CM032587.1	6944637	.	C	T	100	*	TYPE=SUBSTITUTE
CM032587.1	6944639	.	C	G	100	*	TYPE=SUBSTITUTE
CM032587.1	6944645	.	T	C	100	*	TYPE=SUBSTITUTE
CM032587.1	6944646	.	G	A	100	*	TYPE=SUBSTITUTE
CM032587.1	6944649	.	G	A	100	*	TYPE=SUBSTITUTE
CM032587.1	6944651	.	C	T	100	*	TYPE=SUBSTITUTE
CM032587.1	6944652	.	C	T	100	*	TYPE=SUBSTITUTE
CM032587.1	6944654	.	A	G	100	*	TYPE=SUBSTITUTE
CM032587.1	6944655	.	C	T	100	*	TYPE=SUBSTITUTE
CM032587.1	6944660	.	A	G	100	*	TYPE=SUBSTITUTE
CM032587.1	6944681	.	A	G	100	*	TYPE=SUBSTITUTE
CM032587.1	6944684	.	C	T	100	*	TYPE=SUBSTITUTE
CM032587.1	6944705	.	G	T	100	*	TYPE=SUBSTITUTE
CM032587.1	6944709	.	G	A	100	*	TYPE=SUBSTITUTE
CM032587.1	6944714	.	A	G	100	*	TYPE=SUBSTITUTE
CM032587.1	6944719	.	C	T	100	*	TYPE=SUBSTITUTE
CM032587.1	6944720	.	C	G	100	*	TYPE=SUBSTITUTE
CM032587.1	6944725	.	A	G	100	*	TYPE=SUBSTITUTE
CM032587.1	6944726	.	G	A	100	*	TYPE=SUBSTITUTE
CM032587.1	6944732	.	A	G	100	*	TYPE=SUBSTITUTE
CM032587.1	6944735	.	A	G	100	*	TYPE=SUBSTITUTE
CM032587.1	6944760	.	T	C	100	*	TYPE=SUBSTITUTE
CM032587.1	6944762	.	T	C	100	*	TYPE=SUBSTITUTE
CM032587.1	6944765	.	A	C	100	*	TYPE=SUBSTITUTE
CM032587.1	6944768	.	C	T	100	*	TYPE=SUBSTITUTE
CM032587.1	6944771	.	A	G	100	*	TYPE=SUBSTITUTE
CM032587.1	6944777	.	T	C	100	*	TYPE=SUBSTITUTE
CM032587.1	6944785	.	A	G	100	*	TYPE=SUBSTITUTE
CM032587.1	6944786	.	G	A	100	*	TYPE=SUBSTITUTE
CM032587.1	6944799	.	A	G	100	*	TYPE=SUBSTITUTE
CM032587.1	6944813	.	G	C	100	*	TYPE=SUBSTITUTE
CM032587.1	6944816	.	G	A	100	*	TYPE=SUBSTITUTE
CM032587.1	6944831	.	T	C	100	*	TYPE=SUBSTITUTE
CM032587.1	6944834	.	A	G	100	*	TYPE=SUBSTITUTE
CM032587.1	6944837	.	C	A	100	*	TYPE=SUBSTITUTE
CM032587.1	6944846	.	A	G	100	*	TYPE=SUBSTITUTE
CM032587.1	6944853	.	G	A	100	*	TYPE=SUBSTITUTE
CM032587.1	6944854	.	G	A	100	*	TYPE=SUBSTITUTE
CM032587.1	6944863	.	C	T	100	*	TYPE=SUBSTITUTE
CM032587.1	6944865	.	C	T	100	*	TYPE=SUBSTITUTE
CM032587.1	6944871	.	A	T	100	*	TYPE=SUBSTITUTE
CM032587.1	6944874	.	G	C	100	*	TYPE=SUBSTITUTE
CM032587.1	6944875	.	A	G	100	*	TYPE=SUBSTITUTE
CM032587.1	6944876	.	T	C	100	*	TYPE=SUBSTITUTE
CM032587.1	6944877	.	A	G	100	*	TYPE=SUBSTITUTE
CM032587.1	6944878	.	C	A	100	*	TYPE=SUBSTITUTE
CM032587.1	6944887	.	C	A	100	*	TYPE=SUBSTITUTE
CM032587.1	6944888	.	G	T	100	*	TYPE=SUBSTITUTE
CM032587.1	6944900	.	C	T	100	*	TYPE=SUBSTITUTE
CM032587.1	6944903	.	C	T	100	*	TYPE=SUBSTITUTE
CM032587.1	6944908	.	T	C	100	*	TYPE=SUBSTITUTE
CM032587.1	6944911	.	T	C	100	*	TYPE=SUBSTITUTE
CM032587.1	6944915	.	T	C	100	*	TYPE=SUBSTITUTE
CM032587.1	6944916	.	A	G	100	*	TYPE=SUBSTITUTE
CM032587.1	6944918	.	G	A	100	*	TYPE=SUBSTITUTE
CM032587.1	6944920	.	G	A	100	*	TYPE=SUBSTITUTE

CM032587.1	6944922	.	A	G	100	*	TYPE=SUBSTITUTE
CM032587.1	6944924	.	G	A	100	*	TYPE=SUBSTITUTE
CM032587.1	6944929	.	G	A	100	*	TYPE=SUBSTITUTE
CM032587.1	6944940	.	A	T	100	*	TYPE=SUBSTITUTE
CM032587.1	6944942	.	T	C	100	*	TYPE=SUBSTITUTE
CM032587.1	6944951	.	T	C	100	*	TYPE=SUBSTITUTE
CM032587.1	6944957	.	T	C	100	*	TYPE=SUBSTITUTE
CM032587.1	6944966	.	C	T	100	*	TYPE=SUBSTITUTE
CM032587.1	6944972	.	T	C	100	*	TYPE=SUBSTITUTE
CM032587.1	6944973	.	G	C	100	*	TYPE=SUBSTITUTE
CM032587.1	6944974	.	G	A	100	*	TYPE=SUBSTITUTE
CM032587.1	6944975	.	C	G	100	*	TYPE=SUBSTITUTE
CM032587.1	6944976	.	C	G	100	*	TYPE=SUBSTITUTE
CM032587.1	6944981	.	G	A	100	*	TYPE=SUBSTITUTE
CM032587.1	6944984	.	C	T	100	*	TYPE=SUBSTITUTE
CM032587.1	6944989	.	G	A	100	*	TYPE=SUBSTITUTE
CM032587.1	6944993	.	T	C	100	*	TYPE=SUBSTITUTE
CM032587.1	6945001	.	A	G	100	*	TYPE=SUBSTITUTE
CM032587.1	6945002	.	A	T	100	*	TYPE=SUBSTITUTE
CM032587.1	6945008	.	T	C	100	*	TYPE=SUBSTITUTE
CM032587.1	6945014	.	C	G	100	*	TYPE=SUBSTITUTE
CM032587.1	6945015	.	G	A	100	*	TYPE=SUBSTITUTE
CM032587.1	6945017	.	C	T	100	*	TYPE=SUBSTITUTE
CM032587.1	6945029	.	T	C	100	*	TYPE=SUBSTITUTE
CM032587.1	6945032	.	T	G	100	*	TYPE=SUBSTITUTE
CM032587.1	6945050	.	T	C	100	*	TYPE=SUBSTITUTE
CM032587.1	6945056	.	A	G	100	*	TYPE=SUBSTITUTE
CM032587.1	6945071	.	T	C	100	*	TYPE=SUBSTITUTE
CM032587.1	6945074	.	A	G	100	*	TYPE=SUBSTITUTE
CM032587.1	6945077	.	C	T	100	*	TYPE=SUBSTITUTE
CM032587.1	6945083	.	C	CA	100	*	TYPE=INSERT
CM032587.1	6945086	.	A	G	100	*	TYPE=SUBSTITUTE
CM032587.1	6945090	.	G	A	100	*	TYPE=SUBSTITUTE
CM032587.1	6945091	.	TA	T	100	*	TYPE=DELETE
CM032587.1	6945095	.	C	T	100	*	TYPE=SUBSTITUTE
CM032587.1	6945099	.	T	C	100	*	TYPE=SUBSTITUTE
CM032587.1	6945107	.	C	T	100	*	TYPE=SUBSTITUTE
CM032587.1	6945116	.	T	A	100	*	TYPE=SUBSTITUTE
CM032587.1	6945131	.	T	A	100	*	TYPE=SUBSTITUTE
CM032587.1	6945132	.	A	C	100	*	TYPE=SUBSTITUTE
CM032587.1	6945136	.	T	C	100	*	TYPE=SUBSTITUTE
CM032587.1	6945137	.	G	C	100	*	TYPE=SUBSTITUTE
CM032587.1	6945140	.	T	C	100	*	TYPE=SUBSTITUTE
CM032587.1	6945141	.	G	A	100	*	TYPE=SUBSTITUTE
CM032587.1	6945146	.	G	T	100	*	TYPE=SUBSTITUTE
CM032587.1	6945148	.	A	G	100	*	TYPE=SUBSTITUTE
CM032587.1	6945149	.	G	A	100	*	TYPE=SUBSTITUTE
CM032587.1	6945150	.	A	C	100	*	TYPE=SUBSTITUTE
CM032587.1	6945161	.	C	A	100	*	TYPE=SUBSTITUTE
CM032587.1	6945162	.	C	A	100	*	TYPE=SUBSTITUTE
CM032587.1	6945167	.	G	A	100	*	TYPE=SUBSTITUTE
CM032587.1	6945170	.	T	C	100	*	TYPE=SUBSTITUTE
CM032587.1	6945176	.	C	T	100	*	TYPE=SUBSTITUTE
CM032587.1	6945186	.	G	a	100	*	TYPE=SUBSTITUTE
CM032587.1	6945187	.	C	a	100	*	TYPE=SUBSTITUTE
CM032587.1	6945190	.	C	t	100	*	TYPE=SUBSTITUTE
CM032587.1	6945200	.	G	A	100	*	TYPE=SUBSTITUTE
CM032587.1	6945204	.	G	A	100	*	TYPE=SUBSTITUTE
CM032587.1	6945205	.	C	T	100	*	TYPE=SUBSTITUTE
CM032587.1	6945207	.	T	C	100	*	TYPE=SUBSTITUTE
CM032587.1	6945216	.	A	G	100	*	TYPE=SUBSTITUTE
CM032587.1	6945248	.	T	C	100	*	TYPE=SUBSTITUTE
CM032587.1	6945251	.	C	T	100	*	TYPE=SUBSTITUTE
CM032587.1	6945261	.	A	T	100	*	TYPE=SUBSTITUTE

CM032587.1	6945263	.	T	C	100	*	TYPE=SUBSTITUTE
CM032587.1	6945275	.	T	C	100	*	TYPE=SUBSTITUTE
CM032587.1	6945276	.	T	A	100	*	TYPE=SUBSTITUTE
CM032587.1	6945279	.	T	C	100	*	TYPE=SUBSTITUTE
CM032587.1	6945286	.	C	G	100	*	TYPE=SUBSTITUTE
CM032587.1	6945290	.	A	G	100	*	TYPE=SUBSTITUTE
CM032587.1	6945296	.	G	A	100	*	TYPE=SUBSTITUTE
CM032587.1	6945307	.	G	C	100	*	TYPE=SUBSTITUTE
CM032587.1	6945308	.	C	T	100	*	TYPE=SUBSTITUTE
CM032587.1	6945314	.	A	G	100	*	TYPE=SUBSTITUTE
CM032587.1	6945317	.	A	C	100	*	TYPE=SUBSTITUTE
CM032587.1	6945320	.	A	G	100	*	TYPE=SUBSTITUTE
CM032587.1	6945344	.	A	G	100	*	TYPE=SUBSTITUTE
CM032587.1	6945351	.	G	A	100	*	TYPE=SUBSTITUTE
CM032587.1	6945367	.	A	G	100	*	TYPE=SUBSTITUTE
CM032587.1	6945374	.	C	T	100	*	TYPE=SUBSTITUTE
CM032587.1	6945380	.	C	T	100	*	TYPE=SUBSTITUTE
CM032587.1	6945401	.	T	C	100	*	TYPE=SUBSTITUTE
CM032587.1	6945416	.	A	G	100	*	TYPE=SUBSTITUTE
CM032587.1	6945419	.	C	T	100	*	TYPE=SUBSTITUTE
CM032587.1	6945424	.	G	T	100	*	TYPE=SUBSTITUTE
CM032587.1	6945425	.	A	G	100	*	TYPE=SUBSTITUTE
CM032587.1	6945428	.	C	T	100	*	TYPE=SUBSTITUTE
CM032587.1	6945431	.	G	A	100	*	TYPE=SUBSTITUTE
CM032587.1	6945447	.	A	C	100	*	TYPE=SUBSTITUTE
CM032587.1	6945450	.	T	G	100	*	TYPE=SUBSTITUTE
CM032587.1	6945452	.	C	A	100	*	TYPE=SUBSTITUTE
CM032587.1	6945455	.	C	T	100	*	TYPE=SUBSTITUTE
CM032587.1	6945457	.	A	C	100	*	TYPE=SUBSTITUTE
CM032587.1	6945462	.	T	A	100	*	TYPE=SUBSTITUTE
CM032587.1	6945463	.	T	C	100	*	TYPE=SUBSTITUTE
CM032587.1	6945464	.	A	C	100	*	TYPE=SUBSTITUTE
CM032587.1	6945468	.	T	C	100	*	TYPE=SUBSTITUTE
CM032587.1	6945472	.	C	T	100	*	TYPE=SUBSTITUTE
CM032587.1	6945473	.	T	G	100	*	TYPE=SUBSTITUTE
CM032587.1	6945474	.	C	G	100	*	TYPE=SUBSTITUTE
CM032587.1	6945475	.	T	C	100	*	TYPE=SUBSTITUTE
CM032587.1	6945482	.	C	T	100	*	TYPE=SUBSTITUTE
CM032587.1	6945491	.	A	G	100	*	TYPE=SUBSTITUTE
CM032587.1	6945494	.	T	A	100	*	TYPE=SUBSTITUTE
CM032587.1	6945495	.	T	A	100	*	TYPE=SUBSTITUTE
CM032587.1	6945497	.	T	C	100	*	TYPE=SUBSTITUTE
CM032587.1	6945498	.	C	T	100	*	TYPE=SUBSTITUTE
CM032587.1	6945500	.	C	T	100	*	TYPE=SUBSTITUTE
CM032587.1	6945504	.	A	G	100	*	TYPE=SUBSTITUTE
CM032587.1	6945506	.	T	C	100	*	TYPE=SUBSTITUTE
CM032587.1	6945507	.	T	C	100	*	TYPE=SUBSTITUTE
CM032587.1	6945510	.	G	A	100	*	TYPE=SUBSTITUTE
CM032587.1	6945512	.	T	G	100	*	TYPE=SUBSTITUTE
CM032587.1	6945515	.	T	C	100	*	TYPE=SUBSTITUTE
CM032587.1	6945516	.	G	A	100	*	TYPE=SUBSTITUTE
CM032587.1	6945518	.	C	T	100	*	TYPE=SUBSTITUTE
CM032587.1	6945519	.	C	G	100	*	TYPE=SUBSTITUTE
CM032587.1	6945528	.	A	G	100	*	TYPE=SUBSTITUTE
CM032587.1	6945530	.	T	C	100	*	TYPE=SUBSTITUTE
CM032587.1	6945533	.	C	A	100	*	TYPE=SUBSTITUTE
CM032587.1	6945539	.	A	G	100	*	TYPE=SUBSTITUTE
CM032587.1	6945541	.	A	C	100	*	TYPE=SUBSTITUTE
CM032587.1	6945543	.	G	A	100	*	TYPE=SUBSTITUTE
CM032587.1	6945548	.	T	C	100	*	TYPE=SUBSTITUTE
CM032587.1	6945554	.	C	A	100	*	TYPE=SUBSTITUTE
CM032587.1	6945556	.	C	T	100	*	TYPE=SUBSTITUTE
CM032587.1	6945569	.	A	G	100	*	TYPE=SUBSTITUTE
CM032587.1	6945570	.	C	T	100	*	TYPE=SUBSTITUTE

CM032587.1	6945573	.	G	A	100	*	TYPE=SUBSTITUTE
CM032587.1	6945581	.	C	T	100	*	TYPE=SUBSTITUTE
CM032587.1	6945584	.	T	A	100	*	TYPE=SUBSTITUTE
CM032587.1	6945587	.	T	A	100	*	TYPE=SUBSTITUTE
CM032587.1	6945596	.	A	G	100	*	TYPE=SUBSTITUTE
CM032587.1	6945599	.	A	G	100	*	TYPE=SUBSTITUTE
CM032587.1	6945604	.	A	T	100	*	TYPE=SUBSTITUTE
CM032587.1	6945605	.	C	G	100	*	TYPE=SUBSTITUTE
CM032587.1	6945612	.	T	C	100	*	TYPE=SUBSTITUTE
CM032587.1	6945613	.	C	A	100	*	TYPE=SUBSTITUTE
CM032587.1	6945615	.	A	G	100	*	TYPE=SUBSTITUTE
CM032587.1	6945617	.	A	T	100	*	TYPE=SUBSTITUTE
CM032587.1	6945618	.	T	G	100	*	TYPE=SUBSTITUTE
CM032587.1	6945619	.	A	G	100	*	TYPE=SUBSTITUTE
CM032587.1	6945626	.	G	A	100	*	TYPE=SUBSTITUTE
CM032587.1	6945627	.	C	A	100	*	TYPE=SUBSTITUTE
CM032587.1	6945629	.	G	A	100	*	TYPE=SUBSTITUTE
CM032587.1	6945632	.	T	A	100	*	TYPE=SUBSTITUTE
CM032587.1	6945633	.	G	C	100	*	TYPE=SUBSTITUTE
CM032587.1	6945638	.	G	A	100	*	TYPE=SUBSTITUTE
CM032587.1	6945641	.	A	T	100	*	TYPE=SUBSTITUTE
CM032587.1	6945642	.	T	G	100	*	TYPE=SUBSTITUTE
CM032587.1	6945644	.	T	C	100	*	TYPE=SUBSTITUTE
CM032587.1	6945647	.	T	C	100	*	TYPE=SUBSTITUTE
CM032587.1	6945653	.	C	T	100	*	TYPE=SUBSTITUTE
CM032587.1	6945665	.	A	G	100	*	TYPE=SUBSTITUTE
CM032587.1	6945668	.	A	G	100	*	TYPE=SUBSTITUTE
CM032587.1	6945686	.	C	T	100	*	TYPE=SUBSTITUTE
CM032587.1	6945694	.	G	T	100	*	TYPE=SUBSTITUTE
CM032587.1	6945701	.	C	T	100	*	TYPE=SUBSTITUTE
CM032587.1	6945706	.	A	C	100	*	TYPE=SUBSTITUTE
CM032587.1	6945716	.	A	G	100	*	TYPE=SUBSTITUTE
CM032587.1	6945725	.	T	C	100	*	TYPE=SUBSTITUTE
CM032587.1	6945727	.	T	A	100	*	TYPE=SUBSTITUTE
CM032587.1	6945742	.	C	G	100	*	TYPE=SUBSTITUTE
CM032587.1	6945743	.	C	G	100	*	TYPE=SUBSTITUTE
CM032587.1	6945744	.	T	C	100	*	TYPE=SUBSTITUTE
CM032587.1	6945755	.	T	C	100	*	TYPE=SUBSTITUTE
CM032587.1	6945762	.	T	C	100	*	TYPE=SUBSTITUTE
CM032587.1	6945768	.	G	A	100	*	TYPE=SUBSTITUTE
CM032587.1	6945773	.	C	A	100	*	TYPE=SUBSTITUTE
CM032587.1	6945774	.	C	T	100	*	TYPE=SUBSTITUTE
CM032587.1	6945775	.	TCAC	T	100	*	TYPE=DELETE
CM032587.1	6945787	.	C	A	100	*	TYPE=SUBSTITUTE
CM032587.1	6945788	.	G	T	100	*	TYPE=SUBSTITUTE
CM032587.1	6945792	.	A	T	100	*	TYPE=SUBSTITUTE
CM032587.1	6945795	.	G	C	100	*	TYPE=SUBSTITUTE
CM032587.1	6945798	.	G	A	100	*	TYPE=SUBSTITUTE
CM032587.1	6945799	.	G	A	100	*	TYPE=SUBSTITUTE
CM032587.1	6945800	.	C	A	100	*	TYPE=SUBSTITUTE
CM032587.1	6945804	.	T	C	100	*	TYPE=SUBSTITUTE
CM032587.1	6945807	.	G	A	100	*	TYPE=SUBSTITUTE
CM032587.1	6945808	.	T	C	100	*	TYPE=SUBSTITUTE
CM032587.1	6945809	.	T	G	100	*	TYPE=SUBSTITUTE
CM032587.1	6945811	.	A	G	100	*	TYPE=SUBSTITUTE
CM032587.1	6945816	.	C	A	100	*	TYPE=SUBSTITUTE
CM032587.1	6945817	.	A	C	100	*	TYPE=SUBSTITUTE
CM032587.1	6945818	.	T	G	100	*	TYPE=SUBSTITUTE
CM032587.1	6945828	.	C	A	100	*	TYPE=SUBSTITUTE
CM032587.1	6945829	.	G	A	100	*	TYPE=SUBSTITUTE
CM032587.1	6945830	.	A	C	100	*	TYPE=SUBSTITUTE
CM032587.1	6945831	.	T	C	100	*	TYPE=SUBSTITUTE
CM032587.1	6945835	.	T	G	100	*	TYPE=SUBSTITUTE
CM032587.1	6945837	.	A	C	100	*	TYPE=SUBSTITUTE

CM032587.1	6945838	.	G	A	100	*	TYPE=SUBSTITUTE
CM032587.1	6945840	.	A	T	100	*	TYPE=SUBSTITUTE
CM032587.1	6945842	.	G	C	100	*	TYPE=SUBSTITUTE
CM032587.1	6945845	.	G	C	100	*	TYPE=SUBSTITUTE
CM032587.1	6945851	.	T	C	100	*	TYPE=SUBSTITUTE
CM032587.1	6945852	.	A	G	100	*	TYPE=SUBSTITUTE
CM032587.1	6945857	.	T	G	100	*	TYPE=SUBSTITUTE
CM032587.1	6945861	.	A	G	100	*	TYPE=SUBSTITUTE
CM032587.1	6945869	.	G	A	100	*	TYPE=SUBSTITUTE
CM032587.1	6945874	.	G	A	100	*	TYPE=SUBSTITUTE
CM032587.1	6945875	.	A	C	100	*	TYPE=SUBSTITUTE
CM032587.1	6945878	.	T	G	100	*	TYPE=SUBSTITUTE
CM032587.1	6945880	.	T	C	100	*	TYPE=SUBSTITUTE
CM032587.1	6945881	.	T	C	100	*	TYPE=SUBSTITUTE
CM032587.1	6945884	.	C	G	100	*	TYPE=SUBSTITUTE
CM032587.1	6945890	.	G	T	100	*	TYPE=SUBSTITUTE
CM032587.1	6945902	.	T	C	100	*	TYPE=SUBSTITUTE
CM032587.1	6945905	.	G	A	100	*	TYPE=SUBSTITUTE
CM032587.1	6945909	.	T	C	100	*	TYPE=SUBSTITUTE
CM032587.1	6945911	.	T	G	100	*	TYPE=SUBSTITUTE
CM032587.1	6945915	.	T	C	100	*	TYPE=SUBSTITUTE
CM032587.1	6945919	.	T	A	100	*	TYPE=SUBSTITUTE
CM032587.1	6945922	.	G	C	100	*	TYPE=SUBSTITUTE
CM032587.1	6945931	.	C	G	100	*	TYPE=SUBSTITUTE
CM032587.1	6945932	.	T	C	100	*	TYPE=SUBSTITUTE
CM032587.1	6945937	.	G	A	100	*	TYPE=SUBSTITUTE
CM032587.1	6945939	.	A	ATAT	100	*	TYPE=INSERT
CM032587.1	6945940	.	G	A	100	*	TYPE=SUBSTITUTE
CM032587.1	6945941	.	G	T	100	*	TYPE=SUBSTITUTE
CM032587.1	6945943	.	C	T	100	*	TYPE=SUBSTITUTE
CM032587.1	6945945	.	C	A	100	*	TYPE=SUBSTITUTE
CM032587.1	6945946	.	G	A	100	*	TYPE=SUBSTITUTE
CM032587.1	6945949	.	A	G	100	*	TYPE=SUBSTITUTE
CM032587.1	6945954	.	T	G	100	*	TYPE=SUBSTITUTE
CM032587.1	6945955	.	C	T	100	*	TYPE=SUBSTITUTE
CM032587.1	6945959	.	C	T	100	*	TYPE=SUBSTITUTE
CM032587.1	6945961	.	A	C	100	*	TYPE=SUBSTITUTE
CM032587.1	6945965	.	C	T	100	*	TYPE=SUBSTITUTE
CM032587.1	6945974	.	C	T	100	*	TYPE=SUBSTITUTE
CM032587.1	6945975	.	C	T	100	*	TYPE=SUBSTITUTE
CM032587.1	6945976	.	G	A	100	*	TYPE=SUBSTITUTE
CM032587.1	6945981	.	T	A	100	*	TYPE=SUBSTITUTE
CM032587.1	6945982	.	T	A	100	*	TYPE=SUBSTITUTE
CM032587.1	6945983	.	A	AAGACT	100	*	TYPE=INSERT
CM032587.1	6945984	.	T	G	100	*	TYPE=SUBSTITUTE
CM032587.1	6945985	.	T	C	100	*	TYPE=SUBSTITUTE
CM032587.1	6945988	.	T	G	100	*	TYPE=SUBSTITUTE
CM032587.1	6945992	.	T	A	100	*	TYPE=SUBSTITUTE
CM032587.1	6945993	.	C	A	100	*	TYPE=SUBSTITUTE
CM032587.1	6945994	.	A	AT	100	*	TYPE=INSERT
CM032587.1	6945995	.	C	T	100	*	TYPE=SUBSTITUTE
CM032587.1	6945997	.	G	A	100	*	TYPE=SUBSTITUTE
CM032587.1	6945998	.	G	A	100	*	TYPE=SUBSTITUTE
CM032587.1	6946002	.	A	C	100	*	TYPE=SUBSTITUTE
CM032587.1	6946003	.	A	G	100	*	TYPE=SUBSTITUTE
CM032587.1	6946005	.	A	G	100	*	TYPE=SUBSTITUTE
CM032587.1	6946008	.	T	G	100	*	TYPE=SUBSTITUTE
CM032587.1	6946009	.	T	A	100	*	TYPE=SUBSTITUTE
CM032587.1	6946010	.	G	T	100	*	TYPE=SUBSTITUTE
CM032587.1	6946012	.	A	T	100	*	TYPE=SUBSTITUTE
CM032587.1	6946014	.	C	CT	100	*	TYPE=INSERT
CM032587.1	6946015	.	A	T	100	*	TYPE=SUBSTITUTE
CM032587.1	6946018	.	C	G	100	*	TYPE=SUBSTITUTE
CM032587.1	6946019	.	T	A	100	*	TYPE=SUBSTITUTE

CM032587.1	6946020	.	T	A	100	*	TYPE=SUBSTITUTE
CM032587.1	6946024	.	C	G	100	*	TYPE=SUBSTITUTE
CM032587.1	6946026	.	GA	G	100	*	TYPE=DELETE
CM032587.1	6946030	.	A	AGT	100	*	TYPE=INSERT
CM032587.1	6946032	.	T	G	100	*	TYPE=SUBSTITUTE
CM032587.1	6946035	.	T	C	100	*	TYPE=SUBSTITUTE
CM032587.1	6946036	.	G	A	100	*	TYPE=SUBSTITUTE
CM032587.1	6946038	.	T	C	100	*	TYPE=SUBSTITUTE
CM032587.1	6946039	.	G	GA	100	*	TYPE=INSERT
CM032587.1	6946040	.	T	C	100	*	TYPE=SUBSTITUTE
CM032587.1	6946044	.	A	AC	100	*	TYPE=INSERT
CM032587.1	6946045	.	T	A	100	*	TYPE=SUBSTITUTE
CM032587.1	6946047	.	G	T	100	*	TYPE=SUBSTITUTE
CM032587.1	6946049	.	A	ATT	100	*	TYPE=INSERT
CM032587.1	6946052	.	C	T	100	*	TYPE=SUBSTITUTE
CM032587.1	6946053	.	A	G	100	*	TYPE=SUBSTITUTE
CM032587.1	6946054	.	C	A	100	*	TYPE=SUBSTITUTE
CM032587.1	7676010	.	C	t	100	*	TYPE=SUBSTITUTE
CM032587.1	7676011	.	G	a	100	*	TYPE=SUBSTITUTE
CM032587.1	7676013	.	C	t	100	*	TYPE=SUBSTITUTE
CM032587.1	7676016	.	C	t	100	*	TYPE=SUBSTITUTE
CM032587.1	7676023	.	C	t	100	*	TYPE=SUBSTITUTE
CM032587.1	7676071	.	A	G	100	*	TYPE=SUBSTITUTE
CM032587.1	7676089	.	C	t	100	*	TYPE=SUBSTITUTE
CM032587.1	7676098	.	C	t	100	*	TYPE=SUBSTITUTE
CM032587.1	7676099	.	C	t	100	*	TYPE=SUBSTITUTE
CM032587.1	7676104	.	C	t	100	*	TYPE=SUBSTITUTE
CM032587.1	7676105	.	C	t	100	*	TYPE=SUBSTITUTE
CM032587.1	7676108	.	C	t	100	*	TYPE=SUBSTITUTE
CM032587.1	7676128	.	C	T	100	*	TYPE=SUBSTITUTE
CM032587.1	7676130	.	C	T	100	*	TYPE=SUBSTITUTE
CM032587.1	7676138	.	A	g	100	*	TYPE=SUBSTITUTE
CM032587.1	7676139	.	C	t	100	*	TYPE=SUBSTITUTE
CM032587.1	7676144	.	G	a	100	*	TYPE=SUBSTITUTE
CM032587.1	7676145	.	G	a	100	*	TYPE=SUBSTITUTE
CM032587.1	7676146	.	G	a	100	*	TYPE=SUBSTITUTE
CM032587.1	7676175	.	C	t	100	*	TYPE=SUBSTITUTE
CM032587.1	7676202	.	C	t	100	*	TYPE=SUBSTITUTE
CM032587.1	7676205	.	C	t	100	*	TYPE=SUBSTITUTE
CM032587.1	7676206	.	G	a	100	*	TYPE=SUBSTITUTE
CM032587.1	7676209	.	G	a	100	*	TYPE=SUBSTITUTE
CM032587.1	7676220	.	C	t	100	*	TYPE=SUBSTITUTE
CM032587.1	7676223	.	G	a	100	*	TYPE=SUBSTITUTE
CM032587.1	7676236	.	G	a	100	*	TYPE=SUBSTITUTE
CM032587.1	7676241	.	C	t	100	*	TYPE=SUBSTITUTE
CM032587.1	7676244	.	G	a	100	*	TYPE=SUBSTITUTE
CM032587.1	7676247	.	C	t	100	*	TYPE=SUBSTITUTE
CM032587.1	7676250	.	T	c	100	*	TYPE=SUBSTITUTE
CM032587.1	7676254	.	G	a	100	*	TYPE=SUBSTITUTE
CM032587.1	7676261	.	C	t	100	*	TYPE=SUBSTITUTE
CM032587.1	7676264	.	C	t	100	*	TYPE=SUBSTITUTE
CM032587.1	7676265	.	C	t	100	*	TYPE=SUBSTITUTE
CM032587.1	7676270	.	G	a	100	*	TYPE=SUBSTITUTE
CM032587.1	7676280	.	G	A	100	*	TYPE=SUBSTITUTE
CM032587.1	7676283	.	C	T	100	*	TYPE=SUBSTITUTE
CM032587.1	7676296	.	G	A	100	*	TYPE=SUBSTITUTE
CM032587.1	7676297	.	G	A	100	*	TYPE=SUBSTITUTE
CM032587.1	7676301	.	C	T	100	*	TYPE=SUBSTITUTE
CM032587.1	7676302	.	G	A	100	*	TYPE=SUBSTITUTE
CM032587.1	7676303	.	T	C	100	*	TYPE=SUBSTITUTE
CM032587.1	7676316	.	G	a	100	*	TYPE=SUBSTITUTE
CM032587.1	7676331	.	G	a	100	*	TYPE=SUBSTITUTE
CM032587.1	7676343	.	C	t	100	*	TYPE=SUBSTITUTE
CM032587.1	7676344	.	T	a	100	*	TYPE=SUBSTITUTE

CM032587.1	7676353	.	G	a	100	*	TYPE=SUBSTITUTE
CM032587.1	7676356	.	T	c	100	*	TYPE=SUBSTITUTE
CM032587.1	7676362	.	C	t	100	*	TYPE=SUBSTITUTE
CM032587.1	7676366	.	G	a	100	*	TYPE=SUBSTITUTE
CM032587.1	7676397	.	C	t	100	*	TYPE=SUBSTITUTE
CM032587.1	7676398	.	G	a	100	*	TYPE=SUBSTITUTE
CM032587.1	7676400	.	C	t	100	*	TYPE=SUBSTITUTE
CM032587.1	7676403	.	T	c	100	*	TYPE=SUBSTITUTE
CM032587.1	7676420	.	C	t	100	*	TYPE=SUBSTITUTE
CM032587.1	7676422	.	G	a	100	*	TYPE=SUBSTITUTE
CM032587.1	7676431	.	TTC	T	100	*	TYPE=DELETE
CM032587.1	7676436	.	C	t	100	*	TYPE=SUBSTITUTE
CM032587.1	7676439	.	C	t	100	*	TYPE=SUBSTITUTE
CM032587.1	7676450	.	A	g	100	*	TYPE=SUBSTITUTE
CM032587.1	7676466	.	C	t	100	*	TYPE=SUBSTITUTE
CM032587.1	7676467	.	C	t	100	*	TYPE=SUBSTITUTE
CM032587.1	7676468	.	G	a	100	*	TYPE=SUBSTITUTE
CM032587.1	7676473	.	C	t	100	*	TYPE=SUBSTITUTE
CM032587.1	7676474	.	C	t	100	*	TYPE=SUBSTITUTE
CM032587.1	7676479	.	G	a	100	*	TYPE=SUBSTITUTE
CM032587.1	7676484	.	C	t	100	*	TYPE=SUBSTITUTE
CM032587.1	7676490	.	G	A	100	*	TYPE=SUBSTITUTE
CM032587.1	7676491	.	C	T	100	*	TYPE=SUBSTITUTE
CM032587.1	7676493	.	A	G	100	*	TYPE=SUBSTITUTE
CM032587.1	7676503	.	C	t	100	*	TYPE=SUBSTITUTE
CM032587.1	7676504	.	G	a	100	*	TYPE=SUBSTITUTE
CM032587.1	7676508	.	C	t	100	*	TYPE=SUBSTITUTE
CM032587.1	7676520	.	G	a	100	*	TYPE=SUBSTITUTE
CM032587.1	7676527	.	C	t	100	*	TYPE=SUBSTITUTE
CM032587.1	7676535	.	G	a	100	*	TYPE=SUBSTITUTE
CM032587.1	7676537	.	G	a	100	*	TYPE=SUBSTITUTE
CM032587.1	7676545	.	G	a	100	*	TYPE=SUBSTITUTE
CM032587.1	7676554	.	G	a	100	*	TYPE=SUBSTITUTE
CM032587.1	7676556	.	C	t	100	*	TYPE=SUBSTITUTE
CM032587.1	7676572	.	G	a	100	*	TYPE=SUBSTITUTE
CM032587.1	7676574	.	G	a	100	*	TYPE=SUBSTITUTE
CM032587.1	7676575	.	G	a	100	*	TYPE=SUBSTITUTE
CM032587.1	7676583	.	G	a	100	*	TYPE=SUBSTITUTE
CM032587.1	7676585	.	G	a	100	*	TYPE=SUBSTITUTE
CM032587.1	7676586	.	C	t	100	*	TYPE=SUBSTITUTE
CM032587.1	7676589	.	C	t	100	*	TYPE=SUBSTITUTE
CM032587.1	7676590	.	G	a	100	*	TYPE=SUBSTITUTE
CM032587.1	7676596	.	T	c	100	*	TYPE=SUBSTITUTE
CM032587.1	7676616	.	C	t	100	*	TYPE=SUBSTITUTE
CM032587.1	7676626	.	G	a	100	*	TYPE=SUBSTITUTE
CM032587.1	7676646	.	G	a	100	*	TYPE=SUBSTITUTE
CM032587.1	7676652	.	G	a	100	*	TYPE=SUBSTITUTE
CM032587.1	7676663	.	A	g	100	*	TYPE=SUBSTITUTE
CM032587.1	7676677	.	C	T	100	*	TYPE=SUBSTITUTE
CM032587.1	7676678	.	C	T	100	*	TYPE=SUBSTITUTE
CM032587.1	7676684	.	G	A	100	*	TYPE=SUBSTITUTE
CM032587.1	7676688	.	T	c	100	*	TYPE=SUBSTITUTE
CM032587.1	7676697	.	C	t	100	*	TYPE=SUBSTITUTE
CM032587.1	7676698	.	G	a	100	*	TYPE=SUBSTITUTE
CM032587.1	7676699	.	C	t	100	*	TYPE=SUBSTITUTE
CM032587.1	7676710	.	C	t	100	*	TYPE=SUBSTITUTE
CM032587.1	7676712	.	G	a	100	*	TYPE=SUBSTITUTE
CM032587.1	7676713	.	G	a	100	*	TYPE=SUBSTITUTE
CM032587.1	7676714	.	C	t	100	*	TYPE=SUBSTITUTE
CM032587.1	7676733	.	C	t	100	*	TYPE=SUBSTITUTE
CM032587.1	7676737	.	G	a	100	*	TYPE=SUBSTITUTE
CM032587.1	7676740	.	A	g	100	*	TYPE=SUBSTITUTE
CM032587.1	7676743	.	C	t	100	*	TYPE=SUBSTITUTE
CM032587.1	7676745	.	G	a	100	*	TYPE=SUBSTITUTE

CM032587.1	7676758	.	C	t	100	*	TYPE=SUBSTITUTE
CM032587.1	7676761	.	C	t	100	*	TYPE=SUBSTITUTE
CM032587.1	7676764	.	G	a	100	*	TYPE=SUBSTITUTE
CM032587.1	7676787	.	A	G	100	*	TYPE=SUBSTITUTE
CM032587.1	7676798	.	G	A	100	*	TYPE=SUBSTITUTE
CM032587.1	7676802	.	C	T	100	*	TYPE=SUBSTITUTE
CM032587.1	7676812	.	C	T	100	*	TYPE=SUBSTITUTE
CM032587.1	7676813	.	G	A	100	*	TYPE=SUBSTITUTE
CM032587.1	7676832	.	C	t	100	*	TYPE=SUBSTITUTE
CM032587.1	7676833	.	C	t	100	*	TYPE=SUBSTITUTE
CM032587.1	7676843	.	G	a	100	*	TYPE=SUBSTITUTE
CM032587.1	7676844	.	C	t	100	*	TYPE=SUBSTITUTE
CM032587.1	7676846	.	C	t	100	*	TYPE=SUBSTITUTE
CM032587.1	7676847	.	G	a	100	*	TYPE=SUBSTITUTE
CM032587.1	7676868	.	C	t	100	*	TYPE=SUBSTITUTE
CM032587.1	7676876	.	T	c	100	*	TYPE=SUBSTITUTE
CM032587.1	7676879	.	C	t	100	*	TYPE=SUBSTITUTE
CM032587.1	7676895	.	C	t	100	*	TYPE=SUBSTITUTE
CM032587.1	7676903	.	C	t	100	*	TYPE=SUBSTITUTE
CM032587.1	7676928	.	C	t	100	*	TYPE=SUBSTITUTE
CM032587.1	7676933	.	G	a	100	*	TYPE=SUBSTITUTE
CM032587.1	7676934	.	G	a	100	*	TYPE=SUBSTITUTE
CM032587.1	7676937	.	C	t	100	*	TYPE=SUBSTITUTE
CM032587.1	7676940	.	G	a	100	*	TYPE=SUBSTITUTE
CM032587.1	7676941	.	C	t	100	*	TYPE=SUBSTITUTE
CM032587.1	7676943	.	G	a	100	*	TYPE=SUBSTITUTE
CM032587.1	7676945	.	T	c	100	*	TYPE=SUBSTITUTE
CM032587.1	7676950	.	G	a	100	*	TYPE=SUBSTITUTE
CM032587.1	7676957	.	G	a	100	*	TYPE=SUBSTITUTE
CM032587.1	7676968	.	G	a	100	*	TYPE=SUBSTITUTE
CM032587.1	7676985	.	C	t	100	*	TYPE=SUBSTITUTE
CM032587.1	7676993	.	G	a	100	*	TYPE=SUBSTITUTE
CM032587.1	7676995	.	G	T	100	*	TYPE=SUBSTITUTE
CM032587.1	7677002	.	T	C	100	*	TYPE=SUBSTITUTE
CM032587.1	7677003	.	T	C	100	*	TYPE=SUBSTITUTE
CM032587.1	7677011	.	C	T	100	*	TYPE=SUBSTITUTE
CM032587.1	7677012	.	G	A	100	*	TYPE=SUBSTITUTE
CM032587.1	7677024	.	C	t	100	*	TYPE=SUBSTITUTE
CM032587.1	7677039	.	C	t	100	*	TYPE=SUBSTITUTE
CM032587.1	7677040	.	G	a	100	*	TYPE=SUBSTITUTE
CM032587.1	7677043	.	T	c	100	*	TYPE=SUBSTITUTE
CM032587.1	7677048	.	C	t	100	*	TYPE=SUBSTITUTE
CM032587.1	7677055	.	T	c	100	*	TYPE=SUBSTITUTE
CM032587.1	7677057	.	A	g	100	*	TYPE=SUBSTITUTE
CM032587.1	7677060	.	C	t	100	*	TYPE=SUBSTITUTE
CM032587.1	7677072	.	C	t	100	*	TYPE=SUBSTITUTE
CM032587.1	7677073	.	C	t	100	*	TYPE=SUBSTITUTE
CM032587.1	7677079	.	G	a	100	*	TYPE=SUBSTITUTE
CM032587.1	7677080	.	C	t	100	*	TYPE=SUBSTITUTE
CM032587.1	7677091	.	T	c	100	*	TYPE=SUBSTITUTE
CM032587.1	7677107	.	G	a	100	*	TYPE=SUBSTITUTE
CM032587.1	7677125	.	G	a	100	*	TYPE=SUBSTITUTE
CM032587.1	7677127	.	C	t	100	*	TYPE=SUBSTITUTE
CM032587.1	7677136	.	G	a	100	*	TYPE=SUBSTITUTE
CM032587.1	7677148	.	G	a	100	*	TYPE=SUBSTITUTE
CM032587.1	7677156	.	C	t	100	*	TYPE=SUBSTITUTE
CM032587.1	7677172	.	C	t	100	*	TYPE=SUBSTITUTE
CM032587.1	7677174	.	C	t	100	*	TYPE=SUBSTITUTE
CM032587.1	7677176	.	C	t	100	*	TYPE=SUBSTITUTE
CM032587.1	7677200	.	A	G	100	*	TYPE=SUBSTITUTE
CM032587.1	7677214	.	A	g	100	*	TYPE=SUBSTITUTE
CM032587.1	7677217	.	G	a	100	*	TYPE=SUBSTITUTE
CM032587.1	7677231	.	G	a	100	*	TYPE=SUBSTITUTE
CM032587.1	7677233	.	C	t	100	*	TYPE=SUBSTITUTE

CM032587.1	7677234	.	G	a	100	*	TYPE=SUBSTITUTE
CM032587.1	7677237	.	G	a	100	*	TYPE=SUBSTITUTE
CM032587.1	7677238	.	G	a	100	*	TYPE=SUBSTITUTE
CM032587.1	7677241	.	G	a	100	*	TYPE=SUBSTITUTE
CM032587.1	7677266	.	T	c	100	*	TYPE=SUBSTITUTE
CM032587.1	7677274	.	G	a	100	*	TYPE=SUBSTITUTE
CM032587.1	7677284	.	C	t	100	*	TYPE=SUBSTITUTE
CM032587.1	7677301	.	C	t	100	*	TYPE=SUBSTITUTE
CM032587.1	7677303	.	C	t	100	*	TYPE=SUBSTITUTE
CM032587.1	7677305	.	C	t	100	*	TYPE=SUBSTITUTE
CM032587.1	7677306	.	G	a	100	*	TYPE=SUBSTITUTE
CM032587.1	7677322	.	G	a	100	*	TYPE=SUBSTITUTE
CM032587.1	7677323	.	C	t	100	*	TYPE=SUBSTITUTE
CM032587.1	7677329	.	G	a	100	*	TYPE=SUBSTITUTE
CM032587.1	7677335	.	C	t	100	*	TYPE=SUBSTITUTE
CM032587.1	7677339	.	G	a	100	*	TYPE=SUBSTITUTE
CM032587.1	7677355	.	C	t	100	*	TYPE=SUBSTITUTE
CM032587.1	7677356	.	G	A	100	*	TYPE=SUBSTITUTE
CM032587.1	7677367	.	A	G	100	*	TYPE=SUBSTITUTE
CM032587.1	7677386	.	G	a	100	*	TYPE=SUBSTITUTE
CM032587.1	7677413	.	T	c	100	*	TYPE=SUBSTITUTE
CM032587.1	7677417	.	C	t	100	*	TYPE=SUBSTITUTE
CM032587.1	7677418	.	C	t	100	*	TYPE=SUBSTITUTE
CM032587.1	7677442	.	G	a	100	*	TYPE=SUBSTITUTE
CM032587.1	7677453	.	C	t	100	*	TYPE=SUBSTITUTE
CM032587.1	7677454	.	G	a	100	*	TYPE=SUBSTITUTE
CM032587.1	7677459	.	C	t	100	*	TYPE=SUBSTITUTE
CM032587.1	7677468	.	G	a	100	*	TYPE=SUBSTITUTE
CM032587.1	7677470	.	C	t	100	*	TYPE=SUBSTITUTE
CM032587.1	7677473	.	C	t	100	*	TYPE=SUBSTITUTE
CM032587.1	7677476	.	C	t	100	*	TYPE=SUBSTITUTE
CM032587.1	7677480	.	C	t	100	*	TYPE=SUBSTITUTE
CM032587.1	7677481	.	G	a	100	*	TYPE=SUBSTITUTE
CM032587.1	7677491	.	C	t	100	*	TYPE=SUBSTITUTE
CM032587.1	7677495	.	C	t	100	*	TYPE=SUBSTITUTE
CM032587.1	7677496	.	C	t	100	*	TYPE=SUBSTITUTE
CM032587.1	7677504	.	C	t	100	*	TYPE=SUBSTITUTE
CM032587.1	7677512	.	G	a	100	*	TYPE=SUBSTITUTE
CM032587.1	7677515	.	C	t	100	*	TYPE=SUBSTITUTE
CM032587.1	7677516	.	G	a	100	*	TYPE=SUBSTITUTE
CM032587.1	7677520	.	A	g	100	*	TYPE=SUBSTITUTE
CM032587.1	7677527	.	C	t	100	*	TYPE=SUBSTITUTE
CM032587.1	7677535	.	A	g	100	*	TYPE=SUBSTITUTE
CM032587.1	7677543	.	G	a	100	*	TYPE=SUBSTITUTE
CM032588.1	6589	.	G	A	100	*	TYPE=SUBSTITUTE
CM032588.1	6592	.	C	T	100	*	TYPE=SUBSTITUTE
CM032588.1	6604	.	C	T	100	*	TYPE=SUBSTITUTE
CM032588.1	6605	.	G	A	100	*	TYPE=SUBSTITUTE
CM032588.1	6611	.	G	A	100	*	TYPE=SUBSTITUTE
CM032588.1	6624	.	G	A	100	*	TYPE=SUBSTITUTE
CM032588.1	6628	.	G	A	100	*	TYPE=SUBSTITUTE
CM032588.1	6633	.	T	G	100	*	TYPE=SUBSTITUTE
CM032588.1	6646	.	G	T	100	*	TYPE=SUBSTITUTE
CM032588.1	6659	.	T	G	100	*	TYPE=SUBSTITUTE
CM032588.1	6687	.	A	G	100	*	TYPE=SUBSTITUTE
CM032588.1	6693	.	C	A	100	*	TYPE=SUBSTITUTE
CM032588.1	6695	.	C	T	100	*	TYPE=SUBSTITUTE
CM032588.1	6700	.	A	T	100	*	TYPE=SUBSTITUTE
CM032588.1	6707	.	A	T	100	*	TYPE=SUBSTITUTE
CM032588.1	6741	.	C	G	100	*	TYPE=SUBSTITUTE
CM032588.1	6744	.	C	T	100	*	TYPE=SUBSTITUTE
CM032588.1	6748	.	G	A	100	*	TYPE=SUBSTITUTE
CM032588.1	6794	.	A	C	100	*	TYPE=SUBSTITUTE
CM032588.1	6800	.	C	T	100	*	TYPE=SUBSTITUTE

CM032588.1	6805	.	T	A	100	*	TYPE=SUBSTITUTE
CM032588.1	6846	.	GAG	G	100	*	TYPE=DELETE
CM032588.1	6858	.	T	G	100	*	TYPE=SUBSTITUTE
CM032588.1	6860	.	AC	A	100	*	TYPE=DELETE
CM032588.1	6880	.	G	A	100	*	TYPE=SUBSTITUTE
CM032588.1	6888	.	G	A	100	*	TYPE=SUBSTITUTE
CM032588.1	6902	.	A	G	100	*	TYPE=SUBSTITUTE
CM032588.1	6904	.	C	T	100	*	TYPE=SUBSTITUTE
CM032588.1	6908	.	A	C	100	*	TYPE=SUBSTITUTE
CM032588.1	6936	.	T	C	100	*	TYPE=SUBSTITUTE
CM032588.1	6939	.	C	T	100	*	TYPE=SUBSTITUTE
CM032588.1	6960	.	T	C	100	*	TYPE=SUBSTITUTE
CM032588.1	6990	.	A	G	100	*	TYPE=SUBSTITUTE
CM032588.1	7179	.	C	T	100	*	TYPE=SUBSTITUTE
CM032588.1	7191	.	G	A	100	*	TYPE=SUBSTITUTE
CM032588.1	7302	.	A	G	100	*	TYPE=SUBSTITUTE
CM032588.1	7316	.	G	T	100	*	TYPE=SUBSTITUTE
CM032588.1	7323	.	A	G	100	*	TYPE=SUBSTITUTE
CM032588.1	7347	.	G	A	100	*	TYPE=SUBSTITUTE
CM032588.1	7413	.	G	A	100	*	TYPE=SUBSTITUTE
CM032588.1	7419	.	A	G	100	*	TYPE=SUBSTITUTE
CM032588.1	7422	.	T	C	100	*	TYPE=SUBSTITUTE
CM032588.1	7778	.	C	T	100	*	TYPE=SUBSTITUTE
CM032588.1	7822	.	CT	C	100	*	TYPE=DELETE
CM032588.1	7894	.	G	A	100	*	TYPE=SUBSTITUTE
CM032588.1	8001	.	CCT	C	100	*	TYPE=DELETE
CM032588.1	8246	.	A	G	100	*	TYPE=SUBSTITUTE
CM032588.1	8392	.	T	G	100	*	TYPE=SUBSTITUTE
CM032588.1	8553	.	G	A	100	*	TYPE=SUBSTITUTE
CM032588.1	8739	.	C	A	100	*	TYPE=SUBSTITUTE
CM032588.1	9393	.	A	T	100	*	TYPE=SUBSTITUTE
CM032588.1	9498	.	C	T	100	*	TYPE=SUBSTITUTE
CM032588.1	9500	.	G	C	100	*	TYPE=SUBSTITUTE
CM032588.1	10005	.	A	G	100	*	TYPE=SUBSTITUTE
CM032588.1	10599	.	G	A	100	*	TYPE=SUBSTITUTE
CM032588.1	10689	.	T	C	100	*	TYPE=SUBSTITUTE
CM032588.1	10748	.	TAT	T	100	*	TYPE=DELETE
CM032588.1	10762	.	C	T	100	*	TYPE=SUBSTITUTE
CM032588.1	10777	.	T	G	100	*	TYPE=SUBSTITUTE
CM032588.1	10792	.	A	T	100	*	TYPE=SUBSTITUTE
CM032588.1	10798	.	A	T	100	*	TYPE=SUBSTITUTE
CM032588.1	10808	.	T	C	100	*	TYPE=SUBSTITUTE
CM032588.1	10834	.	A	C	100	*	TYPE=SUBSTITUTE
CM032588.1	11594	.	A	G	100	*	TYPE=SUBSTITUTE
CM032588.1	12236	.	G	A	100	*	TYPE=SUBSTITUTE
CM032588.1	12524	.	C	t	100	*	TYPE=SUBSTITUTE
CM032588.1	12986	.	TT	T	100	*	TYPE=DELETE
CM032588.1	13022	.	T	TN	100	*	TYPE=INSERT
CM032588.1	13469	.	C	T	100	*	TYPE=SUBSTITUTE
CM032588.1	13641	.	T	G	100	*	TYPE=SUBSTITUTE
CM032588.1	13644	.	T	C	100	*	TYPE=SUBSTITUTE
CM032588.1	13694	.	C	T	100	*	TYPE=SUBSTITUTE
CM032588.1	13715	.	A	G	100	*	TYPE=SUBSTITUTE
CM032588.1	13718	.	A	G	100	*	TYPE=SUBSTITUTE
CM032588.1	13733	.	C	T	100	*	TYPE=SUBSTITUTE
CM032588.1	13754	.	C	T	100	*	TYPE=SUBSTITUTE
CM032588.1	13790	.	A	G	100	*	TYPE=SUBSTITUTE
CM032588.1	13811	.	A	G	100	*	TYPE=SUBSTITUTE
CM032588.1	13826	.	T	C	100	*	TYPE=SUBSTITUTE
CM032588.1	13828	.	G	A	100	*	TYPE=SUBSTITUTE
CM032588.1	13859	.	C	T	100	*	TYPE=SUBSTITUTE
CM032588.1	13862	.	T	G	100	*	TYPE=SUBSTITUTE
CM032588.1	13892	.	A	G	100	*	TYPE=SUBSTITUTE
CM032588.1	13893	.	G	A	100	*	TYPE=SUBSTITUTE

CM032588.1	13949	.	C	T	100	*	TYPE=SUBSTITUTE
CM032588.1	14024	.	T	A	100	*	TYPE=SUBSTITUTE
CM032588.1	14033	.	C	T	100	*	TYPE=SUBSTITUTE
CM032588.1	14048	.	A	G	100	*	TYPE=SUBSTITUTE
CM032588.1	14055	.	T	A	100	*	TYPE=SUBSTITUTE
CM032588.1	14060	.	C	T	100	*	TYPE=SUBSTITUTE
CM032588.1	14068	.	T	C	100	*	TYPE=SUBSTITUTE
CM032588.1	14108	.	G	A	100	*	TYPE=SUBSTITUTE
CM032588.1	14123	.	A	G	100	*	TYPE=SUBSTITUTE
CM032588.1	14156	.	C	A	100	*	TYPE=SUBSTITUTE
CM032588.1	14216	.	C	T	100	*	TYPE=SUBSTITUTE
CM032588.1	14237	.	C	T	100	*	TYPE=SUBSTITUTE
CM032588.1	14288	.	A	G	100	*	TYPE=SUBSTITUTE
CM032588.1	14293	.	G	T	100	*	TYPE=SUBSTITUTE
CM032588.1	14333	.	T	A	100	*	TYPE=SUBSTITUTE
CM032588.1	14370	.	C	t	100	*	TYPE=SUBSTITUTE
CM032588.1	14374	.	A	g	100	*	TYPE=SUBSTITUTE
CM032588.1	14378	.	G	a	100	*	TYPE=SUBSTITUTE
CM032588.1	14399	.	A	G	100	*	TYPE=SUBSTITUTE
CM032588.1	14429	.	AAA	A	100	*	TYPE=DELETE
CM032588.1	14432	.	A	t	100	*	TYPE=SUBSTITUTE
CM032588.1	14435	.	A	g	100	*	TYPE=SUBSTITUTE
CM032588.1	14444	.	T	C	100	*	TYPE=SUBSTITUTE
CM032588.1	14445	.	G	A	100	*	TYPE=SUBSTITUTE
CM032588.1	14466	.	G	A	100	*	TYPE=SUBSTITUTE
CM032588.1	14472	.	C	A	100	*	TYPE=SUBSTITUTE
CM032588.1	14483	.	C	T	100	*	TYPE=SUBSTITUTE
CM032588.1	14486	.	A	G	100	*	TYPE=SUBSTITUTE
CM032588.1	14495	.	T	C	100	*	TYPE=SUBSTITUTE
CM032588.1	14496	.	G	A	100	*	TYPE=SUBSTITUTE
CM032588.1	14513	.	T	C	100	*	TYPE=SUBSTITUTE
CM032588.1	14516	.	C	T	100	*	TYPE=SUBSTITUTE
CM032588.1	14521	.	T	C	100	*	TYPE=SUBSTITUTE
CM032588.1	14537	.	C	T	100	*	TYPE=SUBSTITUTE
CM032588.1	14545	.	CTA	C	100	*	TYPE=DELETE
CM032588.1	14562	.	T	C	100	*	TYPE=SUBSTITUTE
CM032588.1	14566	.	T	C	100	*	TYPE=SUBSTITUTE
CM032588.1	14572	.	C	T	100	*	TYPE=SUBSTITUTE
CM032588.1	14576	.	A	G	100	*	TYPE=SUBSTITUTE
CM032588.1	14588	.	C	T	100	*	TYPE=SUBSTITUTE
CM032588.1	14604	.	A	G	100	*	TYPE=SUBSTITUTE
CM032588.1	14629	.	C	G	100	*	TYPE=SUBSTITUTE
CM032588.1	14630	.	G	T	100	*	TYPE=SUBSTITUTE
CM032588.1	14646	.	C	T	100	*	TYPE=SUBSTITUTE
CM032588.1	14652	.	T	C	100	*	TYPE=SUBSTITUTE
CM032588.1	14655	.	T	G	100	*	TYPE=SUBSTITUTE
CM032588.1	14659	.	C	T	100	*	TYPE=SUBSTITUTE
CM032588.1	14665	.	C	G	100	*	TYPE=SUBSTITUTE
CM032588.1	14666	.	C	T	100	*	TYPE=SUBSTITUTE
CM032588.1	14667	.	A	G	100	*	TYPE=SUBSTITUTE
CM032588.1	14668	.	A	T	100	*	TYPE=SUBSTITUTE
CM032588.1	14669	.	A	T	100	*	TYPE=SUBSTITUTE
CM032588.1	14674	.	T	C	100	*	TYPE=SUBSTITUTE
CM032588.1	14675	.	A	C	100	*	TYPE=SUBSTITUTE
CM032588.1	14679	.	A	T	100	*	TYPE=SUBSTITUTE
CM032588.1	14686	.	C	T	100	*	TYPE=SUBSTITUTE
CM032588.1	14688	.	T	C	100	*	TYPE=SUBSTITUTE
CM032588.1	14710	.	A	C	100	*	TYPE=SUBSTITUTE
CM032588.1	14723	.	T	C	100	*	TYPE=SUBSTITUTE
CM032588.1	14725	.	T	C	100	*	TYPE=SUBSTITUTE
CM032588.1	14750	.	A	C	100	*	TYPE=SUBSTITUTE
CM032588.1	14754	.	G	A	100	*	TYPE=SUBSTITUTE
CM032588.1	14762	.	T	C	100	*	TYPE=SUBSTITUTE
CM032588.1	14778	.	G	a	100	*	TYPE=SUBSTITUTE

CM032588.1	14779	.	T	g	100	*	TYPE=SUBSTITUTE
CM032588.1	14780	.	GTT	G	100	*	TYPE=DELETE
CM032588.1	14784	.	T	C	100	*	TYPE=SUBSTITUTE
CM032588.1	14793	.	A	AG	100	*	TYPE=INSERT
CM032588.1	14800	.	T	C	100	*	TYPE=SUBSTITUTE
CM032588.1	14828	.	G	A	100	*	TYPE=SUBSTITUTE
CM032588.1	14833	.	C	G	100	*	TYPE=SUBSTITUTE
CM032588.1	14836	.	T	C	100	*	TYPE=SUBSTITUTE
CM032588.1	14845	.	T	C	100	*	TYPE=SUBSTITUTE
CM032588.1	14858	.	CT	C	100	*	TYPE=DELETE
CM032588.1	14874	.	GG	G	100	*	TYPE=DELETE
CM032588.1	14890	.	G	T	100	*	TYPE=SUBSTITUTE
CM032588.1	14900	.	G	A	100	*	TYPE=SUBSTITUTE
CM032588.1	14904	.	T	C	100	*	TYPE=SUBSTITUTE
CM032588.1	14908	.	A	G	100	*	TYPE=SUBSTITUTE
CM032588.1	14910	.	C	A	100	*	TYPE=SUBSTITUTE
CM032588.1	14911	.	A	G	100	*	TYPE=SUBSTITUTE
CM032588.1	14916	.	A	T	100	*	TYPE=SUBSTITUTE
CM032588.1	14922	.	C	T	100	*	TYPE=SUBSTITUTE
CM032588.1	14927	.	A	AAACAATATT	100	*	TYPE=INSERT
CM032588.1	14928	.	G	A	100	*	TYPE=SUBSTITUTE
CM032588.1	14930	.	A	G	100	*	TYPE=SUBSTITUTE
CM032588.1	14934	.	A	G	100	*	TYPE=SUBSTITUTE
CM032588.1	14938	.	C	G	100	*	TYPE=SUBSTITUTE
CM032588.1	14939	.	C	A	100	*	TYPE=SUBSTITUTE
CM032588.1	14942	.	G	T	100	*	TYPE=SUBSTITUTE
CM032588.1	14943	.	G	C	100	*	TYPE=SUBSTITUTE
CM032588.1	14946	.	G	T	100	*	TYPE=SUBSTITUTE
CM032588.1	14947	.	T	G	100	*	TYPE=SUBSTITUTE
CM032588.1	14955	.	C	T	100	*	TYPE=SUBSTITUTE
CM032588.1	14961	.	A	T	100	*	TYPE=SUBSTITUTE
CM032588.1	14979	.	T	A	100	*	TYPE=SUBSTITUTE
CM032588.1	14982	.	T	G	100	*	TYPE=SUBSTITUTE
CM032588.1	15064	.	C	T	100	*	TYPE=SUBSTITUTE
CM032588.1	15074	.	G	A	100	*	TYPE=SUBSTITUTE
CM032588.1	15090	.	C	T	100	*	TYPE=SUBSTITUTE
CM032588.1	15110	.	C	T	100	*	TYPE=SUBSTITUTE
CM032588.1	15113	.	T	C	100	*	TYPE=SUBSTITUTE
CM032588.1	15123	.	T	C	100	*	TYPE=SUBSTITUTE
CM032588.1	15125	.	C	G	100	*	TYPE=SUBSTITUTE
CM032588.1	15129	.	G	A	100	*	TYPE=SUBSTITUTE
CM032588.1	15154	.	G	C	100	*	TYPE=SUBSTITUTE
CM032588.1	15177	.	C	A	100	*	TYPE=SUBSTITUTE
CM032588.1	15201	.	G	A	100	*	TYPE=SUBSTITUTE
CM032588.1	15224	.	A	T	100	*	TYPE=SUBSTITUTE
CM032588.1	15225	.	G	A	100	*	TYPE=SUBSTITUTE
CM032588.1	15230	.	G	A	100	*	TYPE=SUBSTITUTE
CM032588.1	15232	.	T	A	100	*	TYPE=SUBSTITUTE
CM032588.1	15243	.	C	A	100	*	TYPE=SUBSTITUTE
CM032588.1	15257	.	C	G	100	*	TYPE=SUBSTITUTE
CM032588.1	15259	.	T	TTTA	100	*	TYPE=INSERT
CM032588.1	15271	.	G	A	100	*	TYPE=SUBSTITUTE
CM032588.1	15278	.	C	G	100	*	TYPE=SUBSTITUTE
CM032588.1	15281	.	T	A	100	*	TYPE=SUBSTITUTE
CM032588.1	15300	.	A	G	100	*	TYPE=SUBSTITUTE
CM032588.1	15321	.	T	C	100	*	TYPE=SUBSTITUTE
CM032588.1	15330	.	C	G	100	*	TYPE=SUBSTITUTE
CM032588.1	15332	.	C	G	100	*	TYPE=SUBSTITUTE
CM032588.1	15341	.	G	A	100	*	TYPE=SUBSTITUTE
CM032588.1	15376	.	G	A	100	*	TYPE=SUBSTITUTE
CM032588.1	15380	.	G	A	100	*	TYPE=SUBSTITUTE
CM032588.1	15396	.	C	T	100	*	TYPE=SUBSTITUTE
CM032588.1	15417	.	A	C	100	*	TYPE=SUBSTITUTE
CM032588.1	15438	.	C	T	100	*	TYPE=SUBSTITUTE

CM032588.1	15481	.	C	G	100	*	TYPE=SUBSTITUTE
CM032588.1	15511	.	G	T	100	*	TYPE=SUBSTITUTE
CM032588.1	15540	.	T	C	100	*	TYPE=SUBSTITUTE
CM032588.1	15559	.	T	A	100	*	TYPE=SUBSTITUTE
CM032588.1	15587	.	T	C	100	*	TYPE=SUBSTITUTE
CM032588.1	15594	.	G	t	100	*	TYPE=SUBSTITUTE
CM032588.1	15608	.	C	A	100	*	TYPE=SUBSTITUTE
CM032588.1	15611	.	C	T	100	*	TYPE=SUBSTITUTE
CM032588.1	15626	.	T	C	100	*	TYPE=SUBSTITUTE
CM032588.1	15628	.	C	CT	100	*	TYPE=INSERT
CM032588.1	15646	.	T	G	100	*	TYPE=SUBSTITUTE
CM032588.1	15673	.	G	A	100	*	TYPE=SUBSTITUTE
CM032588.1	15687	.	T	C	100	*	TYPE=SUBSTITUTE
CM032588.1	16021	.	T	A	100	*	TYPE=SUBSTITUTE
CM032588.1	16027	.	C	T	100	*	TYPE=SUBSTITUTE
CM032588.1	16038	.	T	C	100	*	TYPE=SUBSTITUTE
CM032588.1	16048	.	C	T	100	*	TYPE=SUBSTITUTE
CM032588.1	16052	.	A	G	100	*	TYPE=SUBSTITUTE
CM032588.1	16105	.	G	T	100	*	TYPE=SUBSTITUTE
CM032588.1	16116	.	C	T	100	*	TYPE=SUBSTITUTE
CM032588.1	16122	.	A	G	100	*	TYPE=SUBSTITUTE
CM032588.1	16130	.	G	T	100	*	TYPE=SUBSTITUTE
CM032588.1	16132	.	C	T	100	*	TYPE=SUBSTITUTE
CM032588.1	16150	.	ATT	A	100	*	TYPE=DELETE
CM032588.1	16192	.	T	G	100	*	TYPE=SUBSTITUTE
CM032588.1	16209	.	T	C	100	*	TYPE=SUBSTITUTE
CM032588.1	16221	.	T	C	100	*	TYPE=SUBSTITUTE
CM032588.1	16233	.	G	A	100	*	TYPE=SUBSTITUTE
CM032588.1	16260	.	G	A	100	*	TYPE=SUBSTITUTE
CM032588.1	16299	.	C	A	100	*	TYPE=SUBSTITUTE
CM032588.1	16314	.	C	T	100	*	TYPE=SUBSTITUTE
CM032588.1	16385	.	A	G	100	*	TYPE=SUBSTITUTE
CM032588.1	16432	.	A	G	100	*	TYPE=SUBSTITUTE
CM032588.1	16477	.	A	G	100	*	TYPE=SUBSTITUTE
CM032588.1	16485	.	C	T	100	*	TYPE=SUBSTITUTE
CM032588.1	16500	.	A	G	100	*	TYPE=SUBSTITUTE
CM032588.1	16557	.	A	G	100	*	TYPE=SUBSTITUTE
CM032588.1	16563	.	T	C	100	*	TYPE=SUBSTITUTE
CM032588.1	16569	.	A	G	100	*	TYPE=SUBSTITUTE
CM032588.1	16575	.	C	T	100	*	TYPE=SUBSTITUTE
CM032588.1	16614	.	A	G	100	*	TYPE=SUBSTITUTE
CM032588.1	16665	.	C	A	100	*	TYPE=SUBSTITUTE
CM032588.1	16710	.	T	C	100	*	TYPE=SUBSTITUTE
CM032588.1	16734	.	A	G	100	*	TYPE=SUBSTITUTE
CM032588.1	16758	.	C	T	100	*	TYPE=SUBSTITUTE
CM032588.1	16791	.	C	T	100	*	TYPE=SUBSTITUTE
CM032588.1	16818	.	C	G	100	*	TYPE=SUBSTITUTE
CM032588.1	16936	.	C	T	100	*	TYPE=SUBSTITUTE
CM032588.1	16973	.	T	C	100	*	TYPE=SUBSTITUTE
CM032588.1	16984	.	G	A	100	*	TYPE=SUBSTITUTE
CM032588.1	16998	.	T	C	100	*	TYPE=SUBSTITUTE
CM032588.1	16999	.	C	T	100	*	TYPE=SUBSTITUTE
CM032588.1	17005	.	G	A	100	*	TYPE=SUBSTITUTE
CM032588.1	17010	.	G	T	100	*	TYPE=SUBSTITUTE
CM032588.1	17026	.	C	T	100	*	TYPE=SUBSTITUTE
CM032588.1	17039	.	C	G	100	*	TYPE=SUBSTITUTE
CM032588.1	17041	.	T	G	100	*	TYPE=SUBSTITUTE
CM032588.1	17044	.	T	C	100	*	TYPE=SUBSTITUTE
CM032588.1	17093	.	AAA	A	100	*	TYPE=DELETE
CM032588.1	17097	.	T	C	100	*	TYPE=SUBSTITUTE
CM032588.1	17129	.	G	C	100	*	TYPE=SUBSTITUTE
CM032588.1	17219	.	T	G	100	*	TYPE=SUBSTITUTE
CM032588.1	17220	.	C	T	100	*	TYPE=SUBSTITUTE
CM032588.1	17229	.	T	A	100	*	TYPE=SUBSTITUTE

CM032588.1	17239	.	A	G	100	*	TYPE=SUBSTITUTE
CM032588.1	17242	.	T	G	100	*	TYPE=SUBSTITUTE
CM032588.1	17298	.	G	A	100	*	TYPE=SUBSTITUTE
CM032588.1	17316	.	TT	T	100	*	TYPE=DELETE
CM032588.1	17332	.	T	G	100	*	TYPE=SUBSTITUTE
CM032588.1	17349	.	T	C	100	*	TYPE=SUBSTITUTE
CM032588.1	17368	.	T	G	100	*	TYPE=SUBSTITUTE
CM032588.1	17388	.	G	A	100	*	TYPE=SUBSTITUTE
CM032588.1	17406	.	T	C	100	*	TYPE=SUBSTITUTE
CM032588.1	17424	.	A	G	100	*	TYPE=SUBSTITUTE
CM032588.1	17458	.	GT	G	100	*	TYPE=DELETE
CM032588.1	17491	.	G	C	100	*	TYPE=SUBSTITUTE
CM032588.1	17499	.	T	G	100	*	TYPE=SUBSTITUTE
CM032588.1	17500	.	C	T	100	*	TYPE=SUBSTITUTE
CM032588.1	17512	.	G	A	100	*	TYPE=SUBSTITUTE
CM032588.1	17539	.	A	G	100	*	TYPE=SUBSTITUTE
CM032588.1	17545	.	C	T	100	*	TYPE=SUBSTITUTE
CM032588.1	17626	.	A	G	100	*	TYPE=SUBSTITUTE
CM032588.1	17638	.	G	A	100	*	TYPE=SUBSTITUTE
CM032588.1	17677	.	T	C	100	*	TYPE=SUBSTITUTE
CM032588.1	17680	.	T	C	100	*	TYPE=SUBSTITUTE
CM032588.1	17716	.	C	T	100	*	TYPE=SUBSTITUTE
CM032588.1	17726	.	C	T	100	*	TYPE=SUBSTITUTE
CM032588.1	17737	.	C	T	100	*	TYPE=SUBSTITUTE
CM032588.1	17764	.	G	A	100	*	TYPE=SUBSTITUTE
CM032588.1	17776	.	A	G	100	*	TYPE=SUBSTITUTE
CM032588.1	17809	.	A	G	100	*	TYPE=SUBSTITUTE
CM032588.1	17814	.	G	A	100	*	TYPE=SUBSTITUTE
CM032588.1	17827	.	G	A	100	*	TYPE=SUBSTITUTE
CM032588.1	17836	.	A	G	100	*	TYPE=SUBSTITUTE
CM032588.1	17905	.	C	A	100	*	TYPE=SUBSTITUTE
CM032588.1	17934	.	T	C	100	*	TYPE=SUBSTITUTE
CM032588.1	17968	.	G	A	100	*	TYPE=SUBSTITUTE
CM032588.1	17971	.	G	C	100	*	TYPE=SUBSTITUTE
CM032588.1	17977	.	G	T	100	*	TYPE=SUBSTITUTE
CM032588.1	17999	.	T	C	100	*	TYPE=SUBSTITUTE
CM032588.1	18001	.	A	G	100	*	TYPE=SUBSTITUTE
CM032588.1	18004	.	A	G	100	*	TYPE=SUBSTITUTE
CM032588.1	18006	.	A	C	100	*	TYPE=SUBSTITUTE
CM032588.1	18018	.	C	T	100	*	TYPE=SUBSTITUTE
CM032588.1	18028	.	A	G	100	*	TYPE=SUBSTITUTE
CM032588.1	18051	.	C	T	100	*	TYPE=SUBSTITUTE
CM032588.1	18067	.	G	A	100	*	TYPE=SUBSTITUTE
CM032588.1	18073	.	G	A	100	*	TYPE=SUBSTITUTE
CM032588.1	18079	.	A	G	100	*	TYPE=SUBSTITUTE
CM032588.1	18082	.	C	T	100	*	TYPE=SUBSTITUTE
CM032588.1	18096	.	ACT	A	100	*	TYPE=DELETE
CM032588.1	18125	.	A	G	100	*	TYPE=SUBSTITUTE
CM032588.1	18130	.	A	C	100	*	TYPE=SUBSTITUTE
CM032588.1	18140	.	T	C	100	*	TYPE=SUBSTITUTE
CM032588.1	18150	.	A	G	100	*	TYPE=SUBSTITUTE
CM032588.1	18165	.	A	T	100	*	TYPE=SUBSTITUTE
CM032588.1	18198	.	A	G	100	*	TYPE=SUBSTITUTE
CM032588.1	18203	.	C	T	100	*	TYPE=SUBSTITUTE
CM032588.1	18208	.	C	A	100	*	TYPE=SUBSTITUTE
CM032588.1	18212	.	A	G	100	*	TYPE=SUBSTITUTE
CM032588.1	18214	.	T	G	100	*	TYPE=SUBSTITUTE
CM032588.1	18216	.	T	C	100	*	TYPE=SUBSTITUTE
CM032588.1	18220	.	C	A	100	*	TYPE=SUBSTITUTE
CM032588.1	18224	.	A	G	100	*	TYPE=SUBSTITUTE
CM032588.1	18229	.	A	G	100	*	TYPE=SUBSTITUTE
CM032588.1	18237	.	T	C	100	*	TYPE=SUBSTITUTE
CM032588.1	18246	.	G	T	100	*	TYPE=SUBSTITUTE
CM032588.1	18251	.	G	A	100	*	TYPE=SUBSTITUTE

CM032588.1	18274	.	C	A	100	*	TYPE=SUBSTITUTE
CM032588.1	18308	.	G	A	100	*	TYPE=SUBSTITUTE
CM032588.1	18312	.	A	T	100	*	TYPE=SUBSTITUTE
CM032588.1	18318	.	C	T	100	*	TYPE=SUBSTITUTE
CM032588.1	18322	.	T	C	100	*	TYPE=SUBSTITUTE
CM032588.1	18325	.	C	G	100	*	TYPE=SUBSTITUTE
CM032588.1	18331	.	C	T	100	*	TYPE=SUBSTITUTE
CM032588.1	18336	.	C	T	100	*	TYPE=SUBSTITUTE
CM032588.1	18338	.	T	C	100	*	TYPE=SUBSTITUTE
CM032588.1	18343	.	C	A	100	*	TYPE=SUBSTITUTE
CM032588.1	18368	.	C	T	100	*	TYPE=SUBSTITUTE
CM032588.1	18378	.	A	G	100	*	TYPE=SUBSTITUTE
CM032588.1	18402	.	G	A	100	*	TYPE=SUBSTITUTE
CM032588.1	18424	.	T	C	100	*	TYPE=SUBSTITUTE
CM032588.1	18482	.	TT	T	100	*	TYPE=DELETE
CM032588.1	18524	.	C	CTT	100	*	TYPE=INSERT
CM032588.1	18532	.	C	G	100	*	TYPE=SUBSTITUTE
CM032588.1	18537	.	G	A	100	*	TYPE=SUBSTITUTE
CM032588.1	18564	.	C	G	100	*	TYPE=SUBSTITUTE
CM032588.1	18570	.	A	G	100	*	TYPE=SUBSTITUTE
CM032588.1	18571	.	T	C	100	*	TYPE=SUBSTITUTE
CM032588.1	18577	.	G	A	100	*	TYPE=SUBSTITUTE
CM032588.1	18581	.	T	C	100	*	TYPE=SUBSTITUTE
CM032588.1	18594	.	T	C	100	*	TYPE=SUBSTITUTE
CM032588.1	18601	.	A	T	100	*	TYPE=SUBSTITUTE
CM032588.1	18622	.	A	T	100	*	TYPE=SUBSTITUTE
CM032588.1	18628	.	G	T	100	*	TYPE=SUBSTITUTE
CM032588.1	18633	.	C	G	100	*	TYPE=SUBSTITUTE
CM032588.1	18641	.	G	A	100	*	TYPE=SUBSTITUTE
CM032588.1	18648	.	A	C	100	*	TYPE=SUBSTITUTE
CM032588.1	18669	.	G	A	100	*	TYPE=SUBSTITUTE
CM032588.1	18679	.	A	G	100	*	TYPE=SUBSTITUTE
CM032588.1	18681	.	T	C	100	*	TYPE=SUBSTITUTE
CM032588.1	18688	.	C	T	100	*	TYPE=SUBSTITUTE
CM032588.1	18690	.	T	TC	100	*	TYPE=INSERT
CM032588.1	18693	.	G	A	100	*	TYPE=SUBSTITUTE
CM032588.1	18694	.	A	C	100	*	TYPE=SUBSTITUTE
CM032588.1	18698	.	C	T	100	*	TYPE=SUBSTITUTE
CM032588.1	18699	.	C	T	100	*	TYPE=SUBSTITUTE
CM032588.1	18700	.	A	G	100	*	TYPE=SUBSTITUTE
CM032588.1	18707	.	T	C	100	*	TYPE=SUBSTITUTE
CM032588.1	18718	.	T	C	100	*	TYPE=SUBSTITUTE
CM032588.1	18722	.	A	G	100	*	TYPE=SUBSTITUTE
CM032588.1	18723	.	C	A	100	*	TYPE=SUBSTITUTE
CM032588.1	18724	.	A	G	100	*	TYPE=SUBSTITUTE
CM032588.1	18728	.	G	A	100	*	TYPE=SUBSTITUTE
CM032588.1	18732	.	T	C	100	*	TYPE=SUBSTITUTE
CM032588.1	18736	.	G	C	100	*	TYPE=SUBSTITUTE
CM032588.1	18741	.	G	A	100	*	TYPE=SUBSTITUTE
CM032588.1	18758	.	T	G	100	*	TYPE=SUBSTITUTE
CM032588.1	18791	.	A	C	100	*	TYPE=SUBSTITUTE
CM032588.1	18794	.	T	C	100	*	TYPE=SUBSTITUTE
CM032588.1	18802	.	C	T	100	*	TYPE=SUBSTITUTE
CM032588.1	18803	.	A	G	100	*	TYPE=SUBSTITUTE
CM032588.1	18824	.	T	C	100	*	TYPE=SUBSTITUTE
CM032588.1	18851	.	A	C	100	*	TYPE=SUBSTITUTE
CM032588.1	18884	.	T	C	100	*	TYPE=SUBSTITUTE
CM032588.1	18889	.	G	A	100	*	TYPE=SUBSTITUTE
CM032588.1	18900	.	A	G	100	*	TYPE=SUBSTITUTE
CM032588.1	18927	.	C	A	100	*	TYPE=SUBSTITUTE
CM032588.1	18935	.	G	A	100	*	TYPE=SUBSTITUTE
CM032588.1	18941	.	T	C	100	*	TYPE=SUBSTITUTE
CM032588.1	18942	.	G	A	100	*	TYPE=SUBSTITUTE
CM032588.1	18950	.	C	T	100	*	TYPE=SUBSTITUTE

CM032588.1	18974	.	G	T	100	*	TYPE=SUBSTITUTE
CM032588.1	18978	.	A	C	100	*	TYPE=SUBSTITUTE
CM032588.1	18989	.	G	T	100	*	TYPE=SUBSTITUTE
CM032588.1	19010	.	T	C	100	*	TYPE=SUBSTITUTE
CM032588.1	19076	.	C	T	100	*	TYPE=SUBSTITUTE
CM032588.1	19094	.	T	G	100	*	TYPE=SUBSTITUTE
CM032588.1	19187	.	C	T	100	*	TYPE=SUBSTITUTE
CM032588.1	19241	.	T	C	100	*	TYPE=SUBSTITUTE
CM032588.1	19268	.	G	A	100	*	TYPE=SUBSTITUTE
CM032588.1	19271	.	T	C	100	*	TYPE=SUBSTITUTE
CM032588.1	19310	.	A	G	100	*	TYPE=SUBSTITUTE
CM032588.1	19316	.	G	A	100	*	TYPE=SUBSTITUTE
CM032588.1	19333	.	G	A	100	*	TYPE=SUBSTITUTE
CM032588.1	19334	.	G	A	100	*	TYPE=SUBSTITUTE
CM032588.1	19337	.	T	C	100	*	TYPE=SUBSTITUTE
CM032588.1	19370	.	C	A	100	*	TYPE=SUBSTITUTE
CM032588.1	19373	.	C	T	100	*	TYPE=SUBSTITUTE
CM032588.1	19379	.	A	G	100	*	TYPE=SUBSTITUTE
CM032588.1	19388	.	G	A	100	*	TYPE=SUBSTITUTE
CM032588.1	19460	.	G	T	100	*	TYPE=SUBSTITUTE
CM032588.1	19490	.	T	C	100	*	TYPE=SUBSTITUTE
CM032588.1	19508	.	C	T	100	*	TYPE=SUBSTITUTE
CM032588.1	19550	.	G	A	100	*	TYPE=SUBSTITUTE
CM032588.1	19580	.	A	T	100	*	TYPE=SUBSTITUTE
CM032588.1	19596	.	C	T	100	*	TYPE=SUBSTITUTE
CM032588.1	19604	.	G	T	100	*	TYPE=SUBSTITUTE
CM032588.1	19637	.	A	G	100	*	TYPE=SUBSTITUTE
CM032588.1	19665	.	G	A	100	*	TYPE=SUBSTITUTE
CM032588.1	19706	.	T	G	100	*	TYPE=SUBSTITUTE
CM032588.1	19712	.	G	C	100	*	TYPE=SUBSTITUTE
CM032588.1	19723	.	T	G	100	*	TYPE=SUBSTITUTE
CM032588.1	19729	.	G	A	100	*	TYPE=SUBSTITUTE
CM032588.1	19730	.	G	A	100	*	TYPE=SUBSTITUTE
CM032588.1	19731	.	T	TTACA	100	*	TYPE=INSERT
CM032588.1	19738	.	G	A	100	*	TYPE=SUBSTITUTE
CM032588.1	19742	.	G	A	100	*	TYPE=SUBSTITUTE
CM032588.1	19746	.	A	G	100	*	TYPE=SUBSTITUTE
CM032588.1	19752	.	G	A	100	*	TYPE=SUBSTITUTE
CM032588.1	19754	.	G	A	100	*	TYPE=SUBSTITUTE
CM032588.1	19765	.	C	T	100	*	TYPE=SUBSTITUTE
CM032588.1	19769	.	T	C	100	*	TYPE=SUBSTITUTE
CM032588.1	19770	.	T	C	100	*	TYPE=SUBSTITUTE
CM032588.1	19772	.	T	C	100	*	TYPE=SUBSTITUTE
CM032588.1	19775	.	G	T	100	*	TYPE=SUBSTITUTE
CM032588.1	19786	.	G	A	100	*	TYPE=SUBSTITUTE
CM032588.1	19787	.	T	C	100	*	TYPE=SUBSTITUTE
CM032588.1	19811	.	T	C	100	*	TYPE=SUBSTITUTE
CM032588.1	19814	.	A	G	100	*	TYPE=SUBSTITUTE
CM032588.1	19856	.	G	A	100	*	TYPE=SUBSTITUTE
CM032588.1	19877	.	T	C	100	*	TYPE=SUBSTITUTE
CM032588.1	19883	.	A	G	100	*	TYPE=SUBSTITUTE
CM032588.1	19899	.	C	T	100	*	TYPE=SUBSTITUTE
CM032588.1	19915	.	C	T	100	*	TYPE=SUBSTITUTE
CM032588.1	19916	.	A	C	100	*	TYPE=SUBSTITUTE
CM032588.1	19921	.	G	T	100	*	TYPE=SUBSTITUTE
CM032588.1	19925	.	G	C	100	*	TYPE=SUBSTITUTE
CM032588.1	19942	.	A	T	100	*	TYPE=SUBSTITUTE
CM032588.1	19973	.	C	A	100	*	TYPE=SUBSTITUTE
CM032588.1	20029	.	A	G	100	*	TYPE=SUBSTITUTE
CM032588.1	20033	.	C	G	100	*	TYPE=SUBSTITUTE
CM032588.1	20110	.	G	C	100	*	TYPE=SUBSTITUTE
CM032588.1	20138	.	A	G	100	*	TYPE=SUBSTITUTE
CM032588.1	20142	.	C	CCA	100	*	TYPE=INSERT
CM032588.1	20148	.	T	C	100	*	TYPE=SUBSTITUTE

CM032588.1	20158	.	C	A	100	*	TYPE=SUBSTITUTE
CM032588.1	20185	.	A	G	100	*	TYPE=SUBSTITUTE
CM032588.1	20188	.	T	C	100	*	TYPE=SUBSTITUTE
CM032588.1	20337	.	A	C	100	*	TYPE=SUBSTITUTE
CM032588.1	20344	.	A	G	100	*	TYPE=SUBSTITUTE
CM032588.1	20368	.	A	G	100	*	TYPE=SUBSTITUTE
CM032588.1	20373	.	G	A	100	*	TYPE=SUBSTITUTE
CM032588.1	20422	.	G	A	100	*	TYPE=SUBSTITUTE
CM032588.1	20442	.	T	C	100	*	TYPE=SUBSTITUTE
CM032588.1	20459	.	C	T	100	*	TYPE=SUBSTITUTE
CM032588.1	20467	.	C	G	100	*	TYPE=SUBSTITUTE
CM032588.1	20511	.	A	G	100	*	TYPE=SUBSTITUTE
CM032588.1	20518	.	G	A	100	*	TYPE=SUBSTITUTE
CM032588.1	20525	.	A	G	100	*	TYPE=SUBSTITUTE
CM032588.1	20532	.	C	G	100	*	TYPE=SUBSTITUTE
CM032588.1	20538	.	C	T	100	*	TYPE=SUBSTITUTE
CM032588.1	20584	.	A	G	100	*	TYPE=SUBSTITUTE
CM032588.1	20608	.	T	C	100	*	TYPE=SUBSTITUTE
CM032588.1	20636	.	A	G	100	*	TYPE=SUBSTITUTE
CM032588.1	20688	.	T	C	100	*	TYPE=SUBSTITUTE
CM032588.1	20709	.	T	C	100	*	TYPE=SUBSTITUTE
CM032588.1	20712	.	C	T	100	*	TYPE=SUBSTITUTE
CM032588.1	20717	.	A	G	100	*	TYPE=SUBSTITUTE
CM032588.1	20730	.	A	G	100	*	TYPE=SUBSTITUTE
CM032588.1	20734	.	A	T	100	*	TYPE=SUBSTITUTE
CM032588.1	20735	.	A	C	100	*	TYPE=SUBSTITUTE
CM032588.1	20766	.	C	T	100	*	TYPE=SUBSTITUTE
CM032588.1	20828	.	T	C	100	*	TYPE=SUBSTITUTE
CM032588.1	20847	.	G	A	100	*	TYPE=SUBSTITUTE
CM032588.1	20868	.	G	A	100	*	TYPE=SUBSTITUTE
CM032588.1	20888	.	G	A	100	*	TYPE=SUBSTITUTE
CM032588.1	20896	.	C	T	100	*	TYPE=SUBSTITUTE
CM032588.1	20910	.	C	T	100	*	TYPE=SUBSTITUTE
CM032588.1	20956	.	T	C	100	*	TYPE=SUBSTITUTE
CM032588.1	21020	.	A	T	100	*	TYPE=SUBSTITUTE
CM032588.1	21027	.	A	C	100	*	TYPE=SUBSTITUTE
CM032588.1	21055	.	A	G	100	*	TYPE=SUBSTITUTE
CM032588.1	21057	.	A	G	100	*	TYPE=SUBSTITUTE
CM032588.1	21113	.	G	A	100	*	TYPE=SUBSTITUTE
CM032588.1	21118	.	G	A	100	*	TYPE=SUBSTITUTE
CM032588.1	21168	.	TT	T	100	*	TYPE=DELETE
CM032588.1	21202	.	A	G	100	*	TYPE=SUBSTITUTE
CM032588.1	21224	.	G	C	100	*	TYPE=SUBSTITUTE
CM032588.1	21230	.	C	T	100	*	TYPE=SUBSTITUTE
CM032588.1	21241	.	C	T	100	*	TYPE=SUBSTITUTE
CM032588.1	21258	.	C	T	100	*	TYPE=SUBSTITUTE
CM032588.1	21270	.	C	T	100	*	TYPE=SUBSTITUTE
CM032588.1	21273	.	T	C	100	*	TYPE=SUBSTITUTE
CM032588.1	21277	.	T	C	100	*	TYPE=SUBSTITUTE
CM032588.1	21287	.	A	T	100	*	TYPE=SUBSTITUTE
CM032588.1	21311	.	G	A	100	*	TYPE=SUBSTITUTE
CM032588.1	21330	.	C	T	100	*	TYPE=SUBSTITUTE
CM032588.1	21332	.	T	C	100	*	TYPE=SUBSTITUTE
CM032588.1	21367	.	G	A	100	*	TYPE=SUBSTITUTE
CM032588.1	21377	.	G	A	100	*	TYPE=SUBSTITUTE
CM032588.1	21443	.	T	C	100	*	TYPE=SUBSTITUTE
CM032588.1	21465	.	A	C	100	*	TYPE=SUBSTITUTE
CM032588.1	21470	.	A	G	100	*	TYPE=SUBSTITUTE
CM032588.1	21631	.	A	G	100	*	TYPE=SUBSTITUTE
CM032588.1	21642	.	A	C	100	*	TYPE=SUBSTITUTE
CM032588.1	21682	.	G	C	100	*	TYPE=SUBSTITUTE
CM032588.1	21693	.	G	A	100	*	TYPE=SUBSTITUTE
CM032588.1	21724	.	G	A	100	*	TYPE=SUBSTITUTE
CM032588.1	21771	.	G	C	100	*	TYPE=SUBSTITUTE

CM032588.1	21779	.	C	G	100	*	TYPE=SUBSTITUTE
CM032588.1	21789	.	A	G	100	*	TYPE=SUBSTITUTE
CM032588.1	21795	.	A	G	100	*	TYPE=SUBSTITUTE
CM032588.1	21800	.	G	A	100	*	TYPE=SUBSTITUTE
CM032588.1	21809	.	G	C	100	*	TYPE=SUBSTITUTE
CM032588.1	21816	.	C	T	100	*	TYPE=SUBSTITUTE
CM032588.1	21822	.	C	A	100	*	TYPE=SUBSTITUTE
CM032588.1	21848	.	A	G	100	*	TYPE=SUBSTITUTE
CM032588.1	21869	.	C	T	100	*	TYPE=SUBSTITUTE
CM032588.1	21876	.	C	T	100	*	TYPE=SUBSTITUTE
CM032588.1	21894	.	A	G	100	*	TYPE=SUBSTITUTE
CM032588.1	21899	.	T	G	100	*	TYPE=SUBSTITUTE
CM032588.1	21923	.	T	C	100	*	TYPE=SUBSTITUTE
CM032588.1	21933	.	G	A	100	*	TYPE=SUBSTITUTE
CM032588.1	21941	.	G	A	100	*	TYPE=SUBSTITUTE
CM032588.1	21946	.	G	A	100	*	TYPE=SUBSTITUTE
CM032588.1	21967	.	T	A	100	*	TYPE=SUBSTITUTE
CM032588.1	21971	.	A	G	100	*	TYPE=SUBSTITUTE
CM032588.1	21978	.	T	C	100	*	TYPE=SUBSTITUTE
CM032588.1	21991	.	G	A	100	*	TYPE=SUBSTITUTE
CM032588.1	22055	.	T	A	100	*	TYPE=SUBSTITUTE
CM032588.1	22059	.	C	A	100	*	TYPE=SUBSTITUTE
CM032588.1	22062	.	A	C	100	*	TYPE=SUBSTITUTE
CM032588.1	22093	.	A	C	100	*	TYPE=SUBSTITUTE
CM032588.1	22104	.	A	C	100	*	TYPE=SUBSTITUTE
CM032588.1	22105	.	T	C	100	*	TYPE=SUBSTITUTE
CM032588.1	22108	.	C	G	100	*	TYPE=SUBSTITUTE
CM032588.1	22110	.	C	T	100	*	TYPE=SUBSTITUTE
CM032588.1	22117	.	T	C	100	*	TYPE=SUBSTITUTE
CM032588.1	22125	.	A	C	100	*	TYPE=SUBSTITUTE
CM032588.1	22137	.	T	G	100	*	TYPE=SUBSTITUTE
CM032588.1	22139	.	C	T	100	*	TYPE=SUBSTITUTE
CM032588.1	22140	.	A	G	100	*	TYPE=SUBSTITUTE
CM032588.1	22152	.	T	C	100	*	TYPE=SUBSTITUTE
CM032588.1	22158	.	T	C	100	*	TYPE=SUBSTITUTE
CM032588.1	22164	.	C	G	100	*	TYPE=SUBSTITUTE
CM032588.1	22173	.	C	T	100	*	TYPE=SUBSTITUTE
CM032588.1	22174	.	C	A	100	*	TYPE=SUBSTITUTE
CM032588.1	22215	.	C	T	100	*	TYPE=SUBSTITUTE
CM032588.1	22256	.	C	T	100	*	TYPE=SUBSTITUTE
CM032588.1	22262	.	A	G	100	*	TYPE=SUBSTITUTE
CM032588.1	22265	.	G	A	100	*	TYPE=SUBSTITUTE
CM032588.1	22304	.	A	G	100	*	TYPE=SUBSTITUTE
CM032588.1	22335	.	CT	C	100	*	TYPE=DELETE
CM032588.1	22345	.	G	A	100	*	TYPE=SUBSTITUTE
CM032588.1	22370	.	G	T	100	*	TYPE=SUBSTITUTE
CM032588.1	22376	.	A	G	100	*	TYPE=SUBSTITUTE
CM032588.1	22379	.	T	C	100	*	TYPE=SUBSTITUTE
CM032588.1	22443	.	C	T	100	*	TYPE=SUBSTITUTE
CM032588.1	22481	.	CTG	C	100	*	TYPE=DELETE
CM032588.1	22486	.	A	G	100	*	TYPE=SUBSTITUTE
CM032588.1	22488	.	C	G	100	*	TYPE=SUBSTITUTE
CM032588.1	22507	.	T	G	100	*	TYPE=SUBSTITUTE
CM032588.1	22508	.	A	G	100	*	TYPE=SUBSTITUTE
CM032588.1	22510	.	G	C	100	*	TYPE=SUBSTITUTE
CM032588.1	22512	.	A	G	100	*	TYPE=SUBSTITUTE
CM032588.1	22514	.	A	C	100	*	TYPE=SUBSTITUTE
CM032588.1	22525	.	C	T	100	*	TYPE=SUBSTITUTE
CM032588.1	22544	.	G	C	100	*	TYPE=SUBSTITUTE
CM032588.1	22562	.	T	C	100	*	TYPE=SUBSTITUTE
CM032588.1	22601	.	A	G	100	*	TYPE=SUBSTITUTE
CM032588.1	22604	.	G	A	100	*	TYPE=SUBSTITUTE
CM032588.1	22624	.	C	T	100	*	TYPE=SUBSTITUTE
CM032588.1	22651	.	C	T	100	*	TYPE=SUBSTITUTE

CM032588.1	22652	.	T	G	100	*	TYPE=SUBSTITUTE
CM032588.1	22661	.	A	G	100	*	TYPE=SUBSTITUTE
CM032588.1	22672	.	C	A	100	*	TYPE=SUBSTITUTE
CM032588.1	22682	.	T	C	100	*	TYPE=SUBSTITUTE
CM032588.1	22685	.	A	G	100	*	TYPE=SUBSTITUTE
CM032588.1	22703	.	T	C	100	*	TYPE=SUBSTITUTE
CM032588.1	22720	.	G	T	100	*	TYPE=SUBSTITUTE
CM032588.1	22726	.	C	T	100	*	TYPE=SUBSTITUTE
CM032588.1	22736	.	A	G	100	*	TYPE=SUBSTITUTE
CM032588.1	22765	.	A	C	100	*	TYPE=SUBSTITUTE
CM032588.1	22769	.	A	G	100	*	TYPE=SUBSTITUTE
CM032588.1	22771	.	A	C	100	*	TYPE=SUBSTITUTE
CM032588.1	22782	.	T	C	100	*	TYPE=SUBSTITUTE
CM032588.1	22800	.	A	G	100	*	TYPE=SUBSTITUTE
CM032588.1	22819	.	C	T	100	*	TYPE=SUBSTITUTE
CM032588.1	22860	.	GG	G	100	*	TYPE=DELETE
CM032588.1	23050	.	C	G	100	*	TYPE=SUBSTITUTE
CM032588.1	23058	.	C	A	100	*	TYPE=SUBSTITUTE
CM032588.1	23065	.	T	C	100	*	TYPE=SUBSTITUTE
CM032588.1	23171	.	T	C	100	*	TYPE=SUBSTITUTE
CM032588.1	23186	.	C	T	100	*	TYPE=SUBSTITUTE
CM032588.1	23197	.	G	C	100	*	TYPE=SUBSTITUTE
CM032588.1	23198	.	T	A	100	*	TYPE=SUBSTITUTE
CM032588.1	23202	.	C	T	100	*	TYPE=SUBSTITUTE
CM032588.1	23241	.	T	C	100	*	TYPE=SUBSTITUTE
CM032588.1	23252	.	T	C	100	*	TYPE=SUBSTITUTE
CM032588.1	23267	.	T	G	100	*	TYPE=SUBSTITUTE
CM032588.1	23270	.	G	A	100	*	TYPE=SUBSTITUTE
CM032588.1	23400	.	A	C	100	*	TYPE=SUBSTITUTE
CM032588.1	23411	.	G	C	100	*	TYPE=SUBSTITUTE
CM032588.1	23417	.	T	A	100	*	TYPE=SUBSTITUTE
CM032588.1	23461	.	A	G	100	*	TYPE=SUBSTITUTE
CM032588.1	23465	.	G	A	100	*	TYPE=SUBSTITUTE
CM032588.1	23488	.	A	G	100	*	TYPE=SUBSTITUTE
CM032588.1	23491	.	G	A	100	*	TYPE=SUBSTITUTE
CM032588.1	23496	.	T	TTGATCTAAACCTCATACAA	100	*	TYPE=INSERT
CM032588.1	23500	.	G	T	100	*	TYPE=SUBSTITUTE
CM032588.1	23632	.	C	G	100	*	TYPE=SUBSTITUTE
CM032588.1	23721	.	G	A	100	*	TYPE=SUBSTITUTE
CM032588.1	23728	.	G	C	100	*	TYPE=SUBSTITUTE
CM032588.1	23756	.	GG	G	100	*	TYPE=DELETE
CM032588.1	23766	.	C	G	100	*	TYPE=SUBSTITUTE
CM032588.1	23786	.	T	C	100	*	TYPE=SUBSTITUTE
CM032588.1	23837	.	G	GTATAG	100	*	TYPE=INSERT
CM032588.1	23864	.	T	C	100	*	TYPE=SUBSTITUTE
CM032588.1	23880	.	T	C	100	*	TYPE=SUBSTITUTE
CM032588.1	23898	.	T	C	100	*	TYPE=SUBSTITUTE
CM032588.1	23933	.	C	T	100	*	TYPE=SUBSTITUTE
CM032588.1	23951	.	G	A	100	*	TYPE=SUBSTITUTE
CM032588.1	23961	.	T	C	100	*	TYPE=SUBSTITUTE
CM032588.1	23962	.	A	G	100	*	TYPE=SUBSTITUTE
CM032588.1	23978	.	G	A	100	*	TYPE=SUBSTITUTE
CM032588.1	23987	.	T	C	100	*	TYPE=SUBSTITUTE
CM032588.1	24007	.	C	T	100	*	TYPE=SUBSTITUTE
CM032588.1	24016	.	C	T	100	*	TYPE=SUBSTITUTE
CM032588.1	24019	.	T	C	100	*	TYPE=SUBSTITUTE
CM032588.1	24077	.	AA	A	100	*	TYPE=DELETE
CM032588.1	24099	.	C	A	100	*	TYPE=SUBSTITUTE
CM032588.1	24128	.	G	C	100	*	TYPE=SUBSTITUTE
CM032588.1	24140	.	C	G	100	*	TYPE=SUBSTITUTE
CM032588.1	24148	.	T	C	100	*	TYPE=SUBSTITUTE
CM032588.1	24150	.	A	G	100	*	TYPE=SUBSTITUTE
CM032588.1	24177	.	C	G	100	*	TYPE=SUBSTITUTE
CM032588.1	24222	.	A	G	100	*	TYPE=SUBSTITUTE

CM032588.1	24268	.	T	A	100	*	TYPE=SUBSTITUTE
CM032588.1	24269	.	T	A	100	*	TYPE=SUBSTITUTE
CM032588.1	24284	.	A	G	100	*	TYPE=SUBSTITUTE
CM032588.1	24325	.	T	C	100	*	TYPE=SUBSTITUTE
CM032588.1	24366	.	T	C	100	*	TYPE=SUBSTITUTE
CM032588.1	24413	.	C	G	100	*	TYPE=SUBSTITUTE
CM032588.1	24417	.	C	T	100	*	TYPE=SUBSTITUTE
CM032588.1	24421	.	A	G	100	*	TYPE=SUBSTITUTE
CM032588.1	24422	.	T	G	100	*	TYPE=SUBSTITUTE
CM032588.1	24423	.	A	G	100	*	TYPE=SUBSTITUTE
CM032588.1	24425	.	A	T	100	*	TYPE=SUBSTITUTE
CM032588.1	24434	.	A	G	100	*	TYPE=SUBSTITUTE
CM032588.1	24435	.	C	T	100	*	TYPE=SUBSTITUTE
CM032588.1	24441	.	T	C	100	*	TYPE=SUBSTITUTE
CM032588.1	24447	.	C	T	100	*	TYPE=SUBSTITUTE
CM032588.1	24472	.	A	G	100	*	TYPE=SUBSTITUTE
CM032588.1	24480	.	G	T	100	*	TYPE=SUBSTITUTE
CM032588.1	24490	.	T	G	100	*	TYPE=SUBSTITUTE
CM032588.1	24549	.	A	G	100	*	TYPE=SUBSTITUTE
CM032588.1	24565	.	A	G	100	*	TYPE=SUBSTITUTE
CM032588.1	24609	.	A	T	100	*	TYPE=SUBSTITUTE
CM032588.1	24611	.	A	G	100	*	TYPE=SUBSTITUTE
CM032588.1	24617	.	A	G	100	*	TYPE=SUBSTITUTE
CM032588.1	24633	.	C	T	100	*	TYPE=SUBSTITUTE
CM032588.1	24665	.	A	G	100	*	TYPE=SUBSTITUTE
CM032588.1	24674	.	G	A	100	*	TYPE=SUBSTITUTE
CM032588.1	24678	.	G	A	100	*	TYPE=SUBSTITUTE
CM032588.1	24684	.	A	T	100	*	TYPE=SUBSTITUTE
CM032588.1	24711	.	T	C	100	*	TYPE=SUBSTITUTE
CM032588.1	24712	.	G	C	100	*	TYPE=SUBSTITUTE
CM032588.1	24714	.	G	A	100	*	TYPE=SUBSTITUTE
CM032588.1	24745	.	A	C	100	*	TYPE=SUBSTITUTE
CM032588.1	24747	.	T	G	100	*	TYPE=SUBSTITUTE
CM032588.1	24755	.	G	A	100	*	TYPE=SUBSTITUTE
CM032588.1	24765	.	A	C	100	*	TYPE=SUBSTITUTE
CM032588.1	24792	.	G	A	100	*	TYPE=SUBSTITUTE
CM032588.1	24797	.	AG	A	100	*	TYPE=DELETE
CM032588.1	24811	.	G	A	100	*	TYPE=SUBSTITUTE
CM032588.1	24818	.	C	T	100	*	TYPE=SUBSTITUTE
CM032588.1	24819	.	G	A	100	*	TYPE=SUBSTITUTE
CM032588.1	24834	.	G	A	100	*	TYPE=SUBSTITUTE
CM032588.1	24835	.	G	A	100	*	TYPE=SUBSTITUTE
CM032588.1	24837	.	C	A	100	*	TYPE=SUBSTITUTE
CM032588.1	24847	.	G	A	100	*	TYPE=SUBSTITUTE
CM032588.1	24848	.	G	A	100	*	TYPE=SUBSTITUTE
CM032588.1	24853	.	C	T	100	*	TYPE=SUBSTITUTE
CM032588.1	24859	.	G	T	100	*	TYPE=SUBSTITUTE
CM032588.1	24860	.	A	G	100	*	TYPE=SUBSTITUTE
CM032588.1	24921	.	C	A	100	*	TYPE=SUBSTITUTE
CM032588.1	24943	.	C	T	100	*	TYPE=SUBSTITUTE
CM032588.1	24949	.	G	A	100	*	TYPE=SUBSTITUTE
CM032588.1	24955	.	A	G	100	*	TYPE=SUBSTITUTE
CM032588.1	24976	.	T	C	100	*	TYPE=SUBSTITUTE
CM032588.1	24990	.	G	C	100	*	TYPE=SUBSTITUTE
CM032588.1	25004	.	T	A	100	*	TYPE=SUBSTITUTE
CM032588.1	25015	.	C	T	100	*	TYPE=SUBSTITUTE
CM032588.1	25035	.	C	T	100	*	TYPE=SUBSTITUTE
CM032588.1	25056	.	C	T	100	*	TYPE=SUBSTITUTE
CM032588.1	25063	.	A	G	100	*	TYPE=SUBSTITUTE
CM032588.1	25068	.	G	C	100	*	TYPE=SUBSTITUTE
CM032588.1	25096	.	G	A	100	*	TYPE=SUBSTITUTE
CM032588.1	25108	.	C	A	100	*	TYPE=SUBSTITUTE
CM032588.1	25114	.	A	C	100	*	TYPE=SUBSTITUTE
CM032588.1	25120	.	G	T	100	*	TYPE=SUBSTITUTE

CM032588.1	25162	.	G	C	100	*	TYPE=SUBSTITUTE
CM032588.1	25167	.	C	T	100	*	TYPE=SUBSTITUTE
CM032588.1	25168	.	T	C	100	*	TYPE=SUBSTITUTE
CM032588.1	25172	.	C	A	100	*	TYPE=SUBSTITUTE
CM032588.1	25183	.	T	C	100	*	TYPE=SUBSTITUTE
CM032588.1	25201	.	T	C	100	*	TYPE=SUBSTITUTE
CM032588.1	25207	.	G	t	100	*	TYPE=SUBSTITUTE
CM032588.1	25245	.	C	G	100	*	TYPE=SUBSTITUTE
CM032588.1	25256	.	C	T	100	*	TYPE=SUBSTITUTE
CM032588.1	25287	.	A	G	100	*	TYPE=SUBSTITUTE
CM032588.1	25289	.	A	T	100	*	TYPE=SUBSTITUTE
CM032588.1	25305	.	A	G	100	*	TYPE=SUBSTITUTE
CM032588.1	25328	.	G	A	100	*	TYPE=SUBSTITUTE
CM032588.1	25338	.	T	C	100	*	TYPE=SUBSTITUTE
CM032588.1	25380	.	G	A	100	*	TYPE=SUBSTITUTE
CM032588.1	25385	.	C	G	100	*	TYPE=SUBSTITUTE
CM032588.1	25434	.	C	T	100	*	TYPE=SUBSTITUTE
CM032588.1	25535	.	T	C	100	*	TYPE=SUBSTITUTE
CM032588.1	25542	.	G	T	100	*	TYPE=SUBSTITUTE
CM032588.1	25587	.	T	C	100	*	TYPE=SUBSTITUTE
CM032588.1	25672	.	G	A	100	*	TYPE=SUBSTITUTE
CM032588.1	25677	.	G	C	100	*	TYPE=SUBSTITUTE
CM032588.1	25692	.	T	C	100	*	TYPE=SUBSTITUTE
CM032588.1	25695	.	C	T	100	*	TYPE=SUBSTITUTE
CM032588.1	25707	.	C	T	100	*	TYPE=SUBSTITUTE
CM032588.1	25731	.	C	G	100	*	TYPE=SUBSTITUTE
CM032588.1	25748	.	C	G	100	*	TYPE=SUBSTITUTE
CM032588.1	25752	.	T	C	100	*	TYPE=SUBSTITUTE
CM032588.1	25756	.	C	T	100	*	TYPE=SUBSTITUTE
CM032588.1	25783	.	A	G	100	*	TYPE=SUBSTITUTE
CM032588.1	25789	.	T	A	100	*	TYPE=SUBSTITUTE
CM032588.1	25804	.	C	G	100	*	TYPE=SUBSTITUTE
CM032588.1	25814	.	G	T	100	*	TYPE=SUBSTITUTE
CM032588.1	25819	.	T	C	100	*	TYPE=SUBSTITUTE
CM032588.1	25885	.	A	G	100	*	TYPE=SUBSTITUTE
CM032588.1	25893	.	T	C	100	*	TYPE=SUBSTITUTE
CM032588.1	25931	.	A	G	100	*	TYPE=SUBSTITUTE
CM032588.1	25953	.	C	A	100	*	TYPE=SUBSTITUTE
CM032588.1	25960	.	C	G	100	*	TYPE=SUBSTITUTE
CM032588.1	26018	.	A	G	100	*	TYPE=SUBSTITUTE
CM032588.1	26020	.	G	A	100	*	TYPE=SUBSTITUTE
CM032588.1	26037	.	A	G	100	*	TYPE=SUBSTITUTE
CM032588.1	26041	.	G	A	100	*	TYPE=SUBSTITUTE
CM032588.1	26121	.	G	C	100	*	TYPE=SUBSTITUTE
CM032588.1	26131	.	G	T	100	*	TYPE=SUBSTITUTE
CM032588.1	26146	.	G	T	100	*	TYPE=SUBSTITUTE
CM032588.1	26156	.	T	A	100	*	TYPE=SUBSTITUTE
CM032588.1	26182	.	C	T	100	*	TYPE=SUBSTITUTE
CM032588.1	26193	.	T	C	100	*	TYPE=SUBSTITUTE
CM032588.1	26214	.	T	C	100	*	TYPE=SUBSTITUTE
CM032588.1	26217	.	T	C	100	*	TYPE=SUBSTITUTE
CM032588.1	26317	.	G	A	100	*	TYPE=SUBSTITUTE
CM032588.1	26553	.	G	A	100	*	TYPE=SUBSTITUTE
CM032588.1	26606	.	G	A	100	*	TYPE=SUBSTITUTE
CM032588.1	26646	.	C	T	100	*	TYPE=SUBSTITUTE
CM032588.1	26657	.	T	C	100	*	TYPE=SUBSTITUTE
CM032588.1	26668	.	C	t	100	*	TYPE=SUBSTITUTE
CM032588.1	26678	.	C	t	100	*	TYPE=SUBSTITUTE
CM032588.1	26690	.	G	T	100	*	TYPE=SUBSTITUTE
CM032588.1	26773	.	T	C	100	*	TYPE=SUBSTITUTE
CM032588.1	26774	.	T	C	100	*	TYPE=SUBSTITUTE
CM032588.1	26778	.	C	G	100	*	TYPE=SUBSTITUTE
CM032588.1	26782	.	C	T	100	*	TYPE=SUBSTITUTE
CM032588.1	26798	.	G	A	100	*	TYPE=SUBSTITUTE

CM032588.1	26816	.	G	A	100	*	TYPE=SUBSTITUTE
CM032588.1	26838	.	A	C	100	*	TYPE=SUBSTITUTE
CM032588.1	26944	.	G	A	100	*	TYPE=SUBSTITUTE
CM032588.1	26970	.	T	C	100	*	TYPE=SUBSTITUTE
CM032588.1	27035	.	C	T	100	*	TYPE=SUBSTITUTE
CM032588.1	27068	.	A	G	100	*	TYPE=SUBSTITUTE
CM032588.1	27069	.	C	T	100	*	TYPE=SUBSTITUTE
CM032588.1	27080	.	A	C	100	*	TYPE=SUBSTITUTE
CM032588.1	27147	.	C	T	100	*	TYPE=SUBSTITUTE
CM032588.1	27363	.	C	A	100	*	TYPE=SUBSTITUTE
CM032588.1	27379	.	A	G	100	*	TYPE=SUBSTITUTE
CM032588.1	27560	.	C	T	100	*	TYPE=SUBSTITUTE
CM032588.1	27582	.	G	C	100	*	TYPE=SUBSTITUTE
CM032588.1	27588	.	T	C	100	*	TYPE=SUBSTITUTE
CM032588.1	27609	.	C	T	100	*	TYPE=SUBSTITUTE
CM032588.1	27610	.	G	T	100	*	TYPE=SUBSTITUTE
CM032588.1	27611	.	T	A	100	*	TYPE=SUBSTITUTE
CM032588.1	27760	.	T	C	100	*	TYPE=SUBSTITUTE
CM032588.1	27806	.	T	C	100	*	TYPE=SUBSTITUTE
CM032588.1	27830	.	A	C	100	*	TYPE=SUBSTITUTE
CM032588.1	27869	.	A	T	100	*	TYPE=SUBSTITUTE
CM032588.1	27929	.	G	T	100	*	TYPE=SUBSTITUTE
CM032588.1	27963	.	T	TA	100	*	TYPE=INSERT
CM032588.1	27965	.	T	A	100	*	TYPE=SUBSTITUTE
CM032588.1	27994	.	T	C	100	*	TYPE=SUBSTITUTE
CM032588.1	28024	.	CT	C	100	*	TYPE=DELETE
CM032588.1	28042	.	T	C	100	*	TYPE=SUBSTITUTE
CM032588.1	28045	.	T	C	100	*	TYPE=SUBSTITUTE
CM032588.1	28054	.	G	T	100	*	TYPE=SUBSTITUTE
CM032588.1	28068	.	G	C	100	*	TYPE=SUBSTITUTE
CM032588.1	28115	.	G	C	100	*	TYPE=SUBSTITUTE
CM032588.1	28120	.	GT	G	100	*	TYPE=DELETE
CM032588.1	28126	.	A	T	100	*	TYPE=SUBSTITUTE
CM032588.1	28130	.	A	T	100	*	TYPE=SUBSTITUTE
CM032588.1	28131	.	T	A	100	*	TYPE=SUBSTITUTE
CM032588.1	28148	.	G	T	100	*	TYPE=SUBSTITUTE
CM032588.1	28150	.	C	T	100	*	TYPE=SUBSTITUTE
CM032588.1	28205	.	T	C	100	*	TYPE=SUBSTITUTE
CM032588.1	28218	.	G	T	100	*	TYPE=SUBSTITUTE
CM032588.1	28220	.	C	G	100	*	TYPE=SUBSTITUTE
CM032588.1	28225	.	G	T	100	*	TYPE=SUBSTITUTE
CM032588.1	28226	.	C	A	100	*	TYPE=SUBSTITUTE
CM032588.1	28237	.	T	A	100	*	TYPE=SUBSTITUTE
CM032588.1	28257	.	G	C	100	*	TYPE=SUBSTITUTE
CM032588.1	28263	.	C	T	100	*	TYPE=SUBSTITUTE
CM032588.1	28272	.	T	G	100	*	TYPE=SUBSTITUTE
CM032588.1	28279	.	C	A	100	*	TYPE=SUBSTITUTE
CM032588.1	28289	.	A	T	100	*	TYPE=SUBSTITUTE
CM032588.1	28291	.	G	A	100	*	TYPE=SUBSTITUTE
CM032588.1	28296	.	C	T	100	*	TYPE=SUBSTITUTE
CM032588.1	28305	.	T	C	100	*	TYPE=SUBSTITUTE
CM032588.1	28307	.	A	G	100	*	TYPE=SUBSTITUTE
CM032588.1	28314	.	C	A	100	*	TYPE=SUBSTITUTE
CM032588.1	28330	.	A	G	100	*	TYPE=SUBSTITUTE
CM032588.1	28352	.	C	t	100	*	TYPE=SUBSTITUTE
CM032588.1	28355	.	T	a	100	*	TYPE=SUBSTITUTE
CM032588.1	28356	.	A	t	100	*	TYPE=SUBSTITUTE
CM032588.1	28361	.	G	c	100	*	TYPE=SUBSTITUTE
CM032588.1	28362	.	A	G	100	*	TYPE=SUBSTITUTE
CM032588.1	28377	.	G	A	100	*	TYPE=SUBSTITUTE
CM032588.1	28385	.	C	T	100	*	TYPE=SUBSTITUTE
CM032588.1	28388	.	C	T	100	*	TYPE=SUBSTITUTE
CM032588.1	28396	.	A	T	100	*	TYPE=SUBSTITUTE
CM032588.1	28397	.	T	C	100	*	TYPE=SUBSTITUTE

CM032588.1	28398	.	A	G	100	*	TYPE=SUBSTITUTE
CM032588.1	28400	.	C	T	100	*	TYPE=SUBSTITUTE
CM032588.1	28402	.	A	G	100	*	TYPE=SUBSTITUTE
CM032588.1	28403	.	A	G	100	*	TYPE=SUBSTITUTE
CM032588.1	28404	.	A	T	100	*	TYPE=SUBSTITUTE
CM032588.1	28405	.	T	A	100	*	TYPE=SUBSTITUTE
CM032588.1	28410	.	G	A	100	*	TYPE=SUBSTITUTE
CM032588.1	28415	.	A	G	100	*	TYPE=SUBSTITUTE
CM032588.1	28427	.	A	G	100	*	TYPE=SUBSTITUTE
CM032588.1	28439	.	T	C	100	*	TYPE=SUBSTITUTE
CM032588.1	28459	.	G	A	100	*	TYPE=SUBSTITUTE
CM032588.1	28462	.	T	C	100	*	TYPE=SUBSTITUTE
CM032588.1	28480	.	G	A	100	*	TYPE=SUBSTITUTE
CM032588.1	28484	.	C	A	100	*	TYPE=SUBSTITUTE
CM032588.1	28492	.	T	C	100	*	TYPE=SUBSTITUTE
CM032588.1	28493	.	T	C	100	*	TYPE=SUBSTITUTE
CM032588.1	28510	.	A	C	100	*	TYPE=SUBSTITUTE
CM032588.1	28530	.	G	T	100	*	TYPE=SUBSTITUTE
CM032588.1	28533	.	G	A	100	*	TYPE=SUBSTITUTE
CM032588.1	28537	.	T	C	100	*	TYPE=SUBSTITUTE
CM032588.1	28546	.	C	A	100	*	TYPE=SUBSTITUTE
CM032588.1	28559	.	G	A	100	*	TYPE=SUBSTITUTE
CM032588.1	28572	.	ACAAGAT	A	100	*	TYPE=DELETE
CM032588.1	28582	.	T	c	100	*	TYPE=SUBSTITUTE
CM032588.1	28583	.	C	t	100	*	TYPE=SUBSTITUTE
CM032588.1	28591	.	A	G	100	*	TYPE=SUBSTITUTE
CM032588.1	28610	.	G	C	100	*	TYPE=SUBSTITUTE
CM032588.1	28617	.	T	G	100	*	TYPE=SUBSTITUTE
CM032588.1	28618	.	C	G	100	*	TYPE=SUBSTITUTE
CM032588.1	28634	.	T	G	100	*	TYPE=SUBSTITUTE
CM032588.1	28663	.	C	T	100	*	TYPE=SUBSTITUTE
CM032588.1	28673	.	G	T	100	*	TYPE=SUBSTITUTE
CM032588.1	28688	.	C	T	100	*	TYPE=SUBSTITUTE
CM032588.1	28689	.	A	T	100	*	TYPE=SUBSTITUTE
CM032588.1	28702	.	G	A	100	*	TYPE=SUBSTITUTE
CM032588.1	28722	.	T	C	100	*	TYPE=SUBSTITUTE
CM032588.1	28727	.	A	G	100	*	TYPE=SUBSTITUTE
CM032588.1	28729	.	C	G	100	*	TYPE=SUBSTITUTE
CM032588.1	28732	.	C	T	100	*	TYPE=SUBSTITUTE
CM032588.1	28767	.	A	C	100	*	TYPE=SUBSTITUTE
CM032588.1	28772	.	C	T	100	*	TYPE=SUBSTITUTE
CM032588.1	28773	.	A	G	100	*	TYPE=SUBSTITUTE
CM032588.1	28777	.	T	G	100	*	TYPE=SUBSTITUTE
CM032588.1	28780	.	A	G	100	*	TYPE=SUBSTITUTE
CM032588.1	28793	.	T	C	100	*	TYPE=SUBSTITUTE
CM032588.1	28795	.	G	A	100	*	TYPE=SUBSTITUTE
CM032588.1	28801	.	G	A	100	*	TYPE=SUBSTITUTE
CM032588.1	28806	.	T	G	100	*	TYPE=SUBSTITUTE
CM032588.1	28807	.	G	C	100	*	TYPE=SUBSTITUTE
CM032588.1	28815	.	G	A	100	*	TYPE=SUBSTITUTE
CM032588.1	28835	.	C	T	100	*	TYPE=SUBSTITUTE
CM032588.1	28836	.	G	A	100	*	TYPE=SUBSTITUTE
CM032588.1	28853	.	G	A	100	*	TYPE=SUBSTITUTE
CM032588.1	28867	.	G	A	100	*	TYPE=SUBSTITUTE
CM032588.1	28871	.	G	C	100	*	TYPE=SUBSTITUTE
CM032588.1	28877	.	G	A	100	*	TYPE=SUBSTITUTE
CM032588.1	28886	.	C	T	100	*	TYPE=SUBSTITUTE
CM032588.1	28889	.	C	T	100	*	TYPE=SUBSTITUTE
CM032588.1	28891	.	A	G	100	*	TYPE=SUBSTITUTE
CM032588.1	28895	.	T	C	100	*	TYPE=SUBSTITUTE
CM032588.1	28896	.	A	G	100	*	TYPE=SUBSTITUTE
CM032588.1	28898	.	C	T	100	*	TYPE=SUBSTITUTE
CM032588.1	28919	.	A	T	100	*	TYPE=SUBSTITUTE
CM032588.1	28928	.	A	C	100	*	TYPE=SUBSTITUTE

CM032588.1 28943 .   G   T   100   *   TYPE=SUBSTITUTE
CM032588.1 28946 .   G   T   100   *   TYPE=SUBSTITUTE
CM032588.1 28981 .   A   G   100   *   TYPE=SUBSTITUTE
CM032588.1 28995 .   T   C   100   *   TYPE=SUBSTITUTE
CM032588.1 28996 .   T   C   100   *   TYPE=SUBSTITUTE
CM032588.1 28997 .   AGTGGGAG   A   100   *   TYPE=DELETE
CM032588.1 29014 .   C   G   100   *   TYPE=SUBSTITUTE
CM032588.1 29030 .   GTAGATTGCTATCTAGATGCTCAT   G   100   *
      TYPE=DELETE
CM032588.1 29061 .   A   G   100   *   TYPE=SUBSTITUTE
CM032588.1 29074 .   G   A   100   *   TYPE=SUBSTITUTE
CM032588.1 29076 .   GCT   G   100   *   TYPE=DELETE
CM032588.1 29082 .   A   T   100   *   TYPE=SUBSTITUTE
CM032588.1 29088 .   A   G   100   *   TYPE=SUBSTITUTE
CM032588.1 29089 .   TC   T   100   *   TYPE=DELETE
CM032588.1 29099 .   G   C   100   *   TYPE=SUBSTITUTE
CM032588.1 29119 .   A   T   100   *   TYPE=SUBSTITUTE
CM032588.1 29121 .   T   A   100   *   TYPE=SUBSTITUTE
CM032588.1 29126 .   C   T   100   *   TYPE=SUBSTITUTE
CM032588.1 29132 .   A   G   100   *   TYPE=SUBSTITUTE
CM032588.1 29143 .   C   T   100   *   TYPE=SUBSTITUTE
CM032588.1 29152 .   GT   G   100   *   TYPE=DELETE
CM032588.1 29175 .   T   C   100   *   TYPE=SUBSTITUTE
CM032588.1 29180 .   A   T   100   *   TYPE=SUBSTITUTE
CM032588.1 29184 .   T   A   100   *   TYPE=SUBSTITUTE
CM032588.1 29185 .   G   A   100   *   TYPE=SUBSTITUTE
CM032588.1 29187 .   C   G   100   *   TYPE=SUBSTITUTE
CM032588.1 29190 .   C   T   100   *   TYPE=SUBSTITUTE
CM032588.1 29195 .   A   G   100   *   TYPE=SUBSTITUTE
CM032588.1 29205 .   A   G   100   *   TYPE=SUBSTITUTE
CM032588.1 29218 .   T   C   100   *   TYPE=SUBSTITUTE
CM032588.1 29239 .   T   G   100   *   TYPE=SUBSTITUTE
CM032588.1 29248 .   T   C   100   *   TYPE=SUBSTITUTE
CM032588.1 29258 .   T   C   100   *   TYPE=SUBSTITUTE
CM032588.1 29278 .   T   C   100   *   TYPE=SUBSTITUTE
CM032588.1 29290 .   C   T   100   *   TYPE=SUBSTITUTE
CM032588.1 29305 .   G   A   100   *   TYPE=SUBSTITUTE
CM032588.1 29306 .   T   C   100   *   TYPE=SUBSTITUTE
CM032588.1 29308 .   C   G   100   *   TYPE=SUBSTITUTE
CM032588.1 29312 .   C   A   100   *   TYPE=SUBSTITUTE
CM032588.1 29331 .   T   G   100   *   TYPE=SUBSTITUTE
CM032588.1 29334 .   CT   C   100   *   TYPE=DELETE
CM032588.1 29352 .   T   C   100   *   TYPE=SUBSTITUTE
CM032588.1 29358 .   A   G   100   *   TYPE=SUBSTITUTE
CM032588.1 29361 .   C   G   100   *   TYPE=SUBSTITUTE
CM032588.1 29415 .   T   G   100   *   TYPE=SUBSTITUTE
CM032588.1 29417 .   T   C   100   *   TYPE=SUBSTITUTE
CM032588.1 29427 .   A   G   100   *   TYPE=SUBSTITUTE
CM032588.1 29436 .   A   G   100   *   TYPE=SUBSTITUTE
CM032588.1 29445 .   C   A   100   *   TYPE=SUBSTITUTE
CM032588.1 29460 .   A   G   100   *   TYPE=SUBSTITUTE
CM032588.1 29550 .   G   A   100   *   TYPE=SUBSTITUTE
CM032588.1 29591 .   T   C   100   *   TYPE=SUBSTITUTE
CM032588.1 29600 .   T   G   100   *   TYPE=SUBSTITUTE
CM032588.1 29601 .   T   C   100   *   TYPE=SUBSTITUTE
CM032588.1 29622 .   G   T   100   *   TYPE=SUBSTITUTE
CM032588.1 29633 .   A   G   100   *   TYPE=SUBSTITUTE
CM032588.1 29662 .   T   A   100   *   TYPE=SUBSTITUTE
CM032588.1 29680 .   C   T   100   *   TYPE=SUBSTITUTE
CM032588.1 29681 .   G   A   100   *   TYPE=SUBSTITUTE
CM032588.1 29693 .   A   G   100   *   TYPE=SUBSTITUTE
CM032588.1 29696 .   A   G   100   *   TYPE=SUBSTITUTE
CM032588.1 29705 .   T   C   100   *   TYPE=SUBSTITUTE
CM032588.1 29728 .   T   C   100   *   TYPE=SUBSTITUTE

CM032588.1	29739	.	A	G	100	*	TYPE=SUBSTITUTE
CM032588.1	29746	.	G	A	100	*	TYPE=SUBSTITUTE
CM032588.1	29749	.	T	G	100	*	TYPE=SUBSTITUTE
CM032588.1	29750	.	C	A	100	*	TYPE=SUBSTITUTE
CM032588.1	29754	.	A	C	100	*	TYPE=SUBSTITUTE
CM032588.1	29760	.	G	A	100	*	TYPE=SUBSTITUTE
CM032588.1	29772	.	G	T	100	*	TYPE=SUBSTITUTE
CM032588.1	29823	.	G	A	100	*	TYPE=SUBSTITUTE
CM032588.1	29838	.	C	G	100	*	TYPE=SUBSTITUTE
CM032588.1	29907	.	C	a	100	*	TYPE=SUBSTITUTE
CM032588.1	29934	.	T	C	100	*	TYPE=SUBSTITUTE
CM032588.1	30000	.	C	G	100	*	TYPE=SUBSTITUTE
CM032588.1	30024	.	G	T	100	*	TYPE=SUBSTITUTE
CM032588.1	30025	.	A	G	100	*	TYPE=SUBSTITUTE
CM032588.1	30042	.	G	T	100	*	TYPE=SUBSTITUTE
CM032588.1	30141	.	G	A	100	*	TYPE=SUBSTITUTE
CM032588.1	30161	.	C	T	100	*	TYPE=SUBSTITUTE
CM032588.1	30204	.	T	G	100	*	TYPE=SUBSTITUTE
CM032588.1	30238	.	T	C	100	*	TYPE=SUBSTITUTE
CM032588.1	30242	.	A	G	100	*	TYPE=SUBSTITUTE
CM032588.1	30254	.	T	G	100	*	TYPE=SUBSTITUTE
CM032588.1	30255	.	C	G	100	*	TYPE=SUBSTITUTE
CM032588.1	30261	.	C	T	100	*	TYPE=SUBSTITUTE
CM032588.1	30263	.	T	A	100	*	TYPE=SUBSTITUTE
CM032588.1	30268	.	G	A	100	*	TYPE=SUBSTITUTE
CM032588.1	30271	.	T	C	100	*	TYPE=SUBSTITUTE
CM032588.1	30276	.	A	G	100	*	TYPE=SUBSTITUTE
CM032588.1	30294	.	A	G	100	*	TYPE=SUBSTITUTE
CM032588.1	30306	.	C	T	100	*	TYPE=SUBSTITUTE
CM032588.1	30340	.	T	C	100	*	TYPE=SUBSTITUTE
CM032588.1	30343	.	T	G	100	*	TYPE=SUBSTITUTE
CM032588.1	30379	.	G	T	100	*	TYPE=SUBSTITUTE
CM032588.1	30466	.	G	A	100	*	TYPE=SUBSTITUTE
CM032588.1	30517	.	T	g	100	*	TYPE=SUBSTITUTE
CM032588.1	30522	.	G	t	100	*	TYPE=SUBSTITUTE
CM032588.1	30541	.	T	G	100	*	TYPE=SUBSTITUTE
CM032588.1	30556	.	A	G	100	*	TYPE=SUBSTITUTE
CM032588.1	30557	.	T	A	100	*	TYPE=SUBSTITUTE
CM032588.1	30558	.	T	C	100	*	TYPE=SUBSTITUTE
CM032588.1	30573	.	C	T	100	*	TYPE=SUBSTITUTE
CM032588.1	30577	.	A	G	100	*	TYPE=SUBSTITUTE
CM032588.1	30579	.	T	A	100	*	TYPE=SUBSTITUTE
CM032588.1	30582	.	T	C	100	*	TYPE=SUBSTITUTE
CM032588.1	30583	.	T	C	100	*	TYPE=SUBSTITUTE
CM032588.1	30588	.	G	C	100	*	TYPE=SUBSTITUTE
CM032588.1	30589	.	G	T	100	*	TYPE=SUBSTITUTE
CM032588.1	30603	.	A	T	100	*	TYPE=SUBSTITUTE
CM032588.1	30614	.	C	T	100	*	TYPE=SUBSTITUTE
CM032588.1	30618	.	T	G	100	*	TYPE=SUBSTITUTE
CM032588.1	30626	.	C	g	100	*	TYPE=SUBSTITUTE
CM032588.1	30635	.	C	t	100	*	TYPE=SUBSTITUTE
CM032588.1	30648	.	A	C	100	*	TYPE=SUBSTITUTE
CM032588.1	30658	.	A	G	100	*	TYPE=SUBSTITUTE
CM032588.1	30659	.	A	G	100	*	TYPE=SUBSTITUTE
CM032588.1	30660	.	A	G	100	*	TYPE=SUBSTITUTE
CM032588.1	30683	.	G	A	100	*	TYPE=SUBSTITUTE
CM032588.1	30689	.	T	C	100	*	TYPE=SUBSTITUTE
CM032588.1	30696	.	C	T	100	*	TYPE=SUBSTITUTE
CM032588.1	30703	.	G	A	100	*	TYPE=SUBSTITUTE
CM032588.1	30707	.	G	T	100	*	TYPE=SUBSTITUTE
CM032588.1	30709	.	C	T	100	*	TYPE=SUBSTITUTE
CM032588.1	30721	.	T	A	100	*	TYPE=SUBSTITUTE
CM032588.1	30729	.	G	A	100	*	TYPE=SUBSTITUTE
CM032588.1	30734	.	A	G	100	*	TYPE=SUBSTITUTE

CM032588.1	30749	.	G	A	100	*	TYPE=SUBSTITUTE
CM032588.1	30751	.	C	T	100	*	TYPE=SUBSTITUTE
CM032588.1	30752	.	C	T	100	*	TYPE=SUBSTITUTE
CM032588.1	30756	.	A	G	100	*	TYPE=SUBSTITUTE
CM032588.1	30777	.	T	C	100	*	TYPE=SUBSTITUTE
CM032588.1	30779	.	C	G	100	*	TYPE=SUBSTITUTE
CM032588.1	30792	.	T	C	100	*	TYPE=SUBSTITUTE
CM032588.1	30795	.	A	C	100	*	TYPE=SUBSTITUTE
CM032588.1	30797	.	G	C	100	*	TYPE=SUBSTITUTE
CM032588.1	30803	.	A	T	100	*	TYPE=SUBSTITUTE
CM032588.1	30820	.	C	A	100	*	TYPE=SUBSTITUTE
CM032588.1	30835	.	C	a	100	*	TYPE=SUBSTITUTE
CM032588.1	30836	.	a	ag	100	*	TYPE=INSERT
CM032588.1	30843	.	C	t	100	*	TYPE=SUBSTITUTE
CM032588.1	30848	.	G	C	100	*	TYPE=SUBSTITUTE
CM032588.1	30850	.	C	T	100	*	TYPE=SUBSTITUTE
CM032588.1	30851	.	G	A	100	*	TYPE=SUBSTITUTE
CM032588.1	30855	.	A	T	100	*	TYPE=SUBSTITUTE
CM032588.1	30858	.	A	T	100	*	TYPE=SUBSTITUTE
CM032588.1	30862	.	T	C	100	*	TYPE=SUBSTITUTE
CM032588.1	30878	.	A	G	100	*	TYPE=SUBSTITUTE
CM032588.1	30883	.	C	G	100	*	TYPE=SUBSTITUTE
CM032588.1	30890	.	A	G	100	*	TYPE=SUBSTITUTE
CM032588.1	30908	.	A	T	100	*	TYPE=SUBSTITUTE
CM032588.1	30940	.	A	G	100	*	TYPE=SUBSTITUTE
CM032588.1	30944	.	G	T	100	*	TYPE=SUBSTITUTE
CM032588.1	30951	.	A	C	100	*	TYPE=SUBSTITUTE
CM032588.1	30964	.	G	T	100	*	TYPE=SUBSTITUTE
CM032588.1	30967	.	T	C	100	*	TYPE=SUBSTITUTE
CM032588.1	30984	.	G	A	100	*	TYPE=SUBSTITUTE
CM032588.1	30986	.	T	C	100	*	TYPE=SUBSTITUTE
CM032588.1	30994	.	G	t	100	*	TYPE=SUBSTITUTE
CM032588.1	30995	.	A	g	100	*	TYPE=SUBSTITUTE
CM032588.1	31012	.	C	T	100	*	TYPE=SUBSTITUTE
CM032588.1	31027	.	C	G	100	*	TYPE=SUBSTITUTE
CM032588.1	31031	.	C	G	100	*	TYPE=SUBSTITUTE
CM032588.1	31035	.	C	G	100	*	TYPE=SUBSTITUTE
CM032588.1	31076	.	A	G	100	*	TYPE=SUBSTITUTE
CM032588.1	31080	.	G	A	100	*	TYPE=SUBSTITUTE
CM032588.1	31091	.	C	G	100	*	TYPE=SUBSTITUTE
CM032588.1	31101	.	C	T	100	*	TYPE=SUBSTITUTE
CM032588.1	31118	.	T	G	100	*	TYPE=SUBSTITUTE
CM032588.1	31127	.	C	T	100	*	TYPE=SUBSTITUTE
CM032588.1	31130	.	G	T	100	*	TYPE=SUBSTITUTE
CM032588.1	31137	.	T	C	100	*	TYPE=SUBSTITUTE
CM032588.1	31142	.	T	C	100	*	TYPE=SUBSTITUTE
CM032588.1	31150	.	A	G	100	*	TYPE=SUBSTITUTE
CM032588.1	31155	.	C	T	100	*	TYPE=SUBSTITUTE
CM032588.1	31158	.	T	G	100	*	TYPE=SUBSTITUTE
CM032588.1	31161	.	G	A	100	*	TYPE=SUBSTITUTE
CM032588.1	31167	.	T	C	100	*	TYPE=SUBSTITUTE
CM032588.1	31175	.	C	T	100	*	TYPE=SUBSTITUTE
CM032588.1	31186	.	A	T	100	*	TYPE=SUBSTITUTE
CM032588.1	31190	.	AATTCCATATAAGTCGCAAGTATGGT	A	100	*	TYPE=DELETE
CM032588.1	31222	.	A	G	100	*	TYPE=SUBSTITUTE
CM032588.1	31223	.	C	A	100	*	TYPE=SUBSTITUTE
CM032588.1	31224	.	A	C	100	*	TYPE=SUBSTITUTE
CM032588.1	31231	.	C	G	100	*	TYPE=SUBSTITUTE
CM032588.1	31255	.	t	tgtag	100	*	TYPE=INSERT
CM032588.1	31259	.	T	g	100	*	TYPE=SUBSTITUTE
CM032588.1	31267	.	G	A	100	*	TYPE=SUBSTITUTE
CM032588.1	31268	.	A	T	100	*	TYPE=SUBSTITUTE
CM032588.1	31275	.	C	A	100	*	TYPE=SUBSTITUTE

CM032588.1	31291	.	G	GTGTA	100	*	TYPE=INSERT
CM032588.1	31293	.	G	C	100	*	TYPE=SUBSTITUTE
CM032588.1	31300	.	A	T	100	*	TYPE=SUBSTITUTE
CM032588.1	31304	.	A	G	100	*	TYPE=SUBSTITUTE
CM032588.1	31337	.	T	C	100	*	TYPE=SUBSTITUTE
CM032588.1	31367	.	G	A	100	*	TYPE=SUBSTITUTE
CM032588.1	31373	.	T	C	100	*	TYPE=SUBSTITUTE
CM032588.1	31375	.	G	A	100	*	TYPE=SUBSTITUTE
CM032588.1	31413	.	T	C	100	*	TYPE=SUBSTITUTE
CM032588.1	31418	.	C	T	100	*	TYPE=SUBSTITUTE
CM032588.1	31452	.	G	A	100	*	TYPE=SUBSTITUTE
CM032588.1	31479	.	C	T	100	*	TYPE=SUBSTITUTE
CM032588.1	31536	.	T	G	100	*	TYPE=SUBSTITUTE
CM032588.1	31564	.	G	gtttg	100	*	TYPE=INSERT
CM032588.1	31652	.	T	C	100	*	TYPE=SUBSTITUTE
CM032588.1	31684	.	G	A	100	*	TYPE=SUBSTITUTE
CM032588.1	31686	.	C	T	100	*	TYPE=SUBSTITUTE
CM032588.1	31694	.	C	G	100	*	TYPE=SUBSTITUTE
CM032588.1	31696	.	A	G	100	*	TYPE=SUBSTITUTE
CM032588.1	31702	.	G	A	100	*	TYPE=SUBSTITUTE
CM032588.1	31704	.	A	T	100	*	TYPE=SUBSTITUTE
CM032588.1	31712	.	G	A	100	*	TYPE=SUBSTITUTE
CM032588.1	31718	.	G	A	100	*	TYPE=SUBSTITUTE
CM032588.1	31730	.	G	A	100	*	TYPE=SUBSTITUTE
CM032588.1	31735	.	G	A	100	*	TYPE=SUBSTITUTE
CM032588.1	31739	.	A	G	100	*	TYPE=SUBSTITUTE
CM032588.1	31744	.	T	C	100	*	TYPE=SUBSTITUTE
CM032588.1	31761	.	T	C	100	*	TYPE=SUBSTITUTE
CM032588.1	31797	.	A	C	100	*	TYPE=SUBSTITUTE
CM032588.1	31800	.	T	C	100	*	TYPE=SUBSTITUTE
CM032588.1	31805	.	G	A	100	*	TYPE=SUBSTITUTE
CM032588.1	31810	.	A	G	100	*	TYPE=SUBSTITUTE
CM032588.1	31825	.	A	T	100	*	TYPE=SUBSTITUTE
CM032588.1	31834	.	A	C	100	*	TYPE=SUBSTITUTE
CM032588.1	31846	.	G	A	100	*	TYPE=SUBSTITUTE
CM032588.1	31850	.	A	T	100	*	TYPE=SUBSTITUTE
CM032588.1	31860	.	C	A	100	*	TYPE=SUBSTITUTE
CM032588.1	31880	.	A	G	100	*	TYPE=SUBSTITUTE
CM032588.1	31893	.	C	T	100	*	TYPE=SUBSTITUTE
CM032588.1	31900	.	G	A	100	*	TYPE=SUBSTITUTE
CM032588.1	31901	.	T	C	100	*	TYPE=SUBSTITUTE
CM032588.1	31906	.	C	G	100	*	TYPE=SUBSTITUTE
CM032588.1	31930	.	A	G	100	*	TYPE=SUBSTITUTE
CM032588.1	31935	.	G	C	100	*	TYPE=SUBSTITUTE
CM032588.1	31939	.	G	A	100	*	TYPE=SUBSTITUTE
CM032588.1	31945	.	CG	C	100	*	TYPE=DELETE
CM032588.1	31951	.	CAG	C	100	*	TYPE=DELETE
CM032588.1	31975	.	T	A	100	*	TYPE=SUBSTITUTE
CM032588.1	31980	.	C	T	100	*	TYPE=SUBSTITUTE
CM032588.1	31994	.	G	A	100	*	TYPE=SUBSTITUTE
CM032588.1	32027	.	T	C	100	*	TYPE=SUBSTITUTE
CM032588.1	32032	.	G	A	100	*	TYPE=SUBSTITUTE
CM032588.1	32041	.	C	T	100	*	TYPE=SUBSTITUTE
CM032588.1	32068	.	G	A	100	*	TYPE=SUBSTITUTE
CM032588.1	32069	.	G	C	100	*	TYPE=SUBSTITUTE
CM032588.1	32072	.	G	A	100	*	TYPE=SUBSTITUTE
CM032588.1	32080	.	G	A	100	*	TYPE=SUBSTITUTE
CM032588.1	32104	.	T	C	100	*	TYPE=SUBSTITUTE
CM032588.1	32107	.	G	T	100	*	TYPE=SUBSTITUTE
CM032588.1	32114	.	C	G	100	*	TYPE=SUBSTITUTE
CM032588.1	32140	.	T	G	100	*	TYPE=SUBSTITUTE
CM032588.1	32176	.	C	T	100	*	TYPE=SUBSTITUTE
CM032588.1	32188	.	T	C	100	*	TYPE=SUBSTITUTE
CM032588.1	32207	.	C	A	100	*	TYPE=SUBSTITUTE

CM032588.1	32209	.	A	G	100	*	TYPE=SUBSTITUTE
CM032588.1	32230	.	T	C	100	*	TYPE=SUBSTITUTE
CM032588.1	32242	.	C	G	100	*	TYPE=SUBSTITUTE
CM032588.1	32272	.	T	C	100	*	TYPE=SUBSTITUTE
CM032588.1	32276	.	C	A	100	*	TYPE=SUBSTITUTE
CM032588.1	32287	.	A	G	100	*	TYPE=SUBSTITUTE
CM032588.1	32317	.	A	C	100	*	TYPE=SUBSTITUTE
CM032588.1	32337	.	T	A	100	*	TYPE=SUBSTITUTE
CM032588.1	32380	.	A	G	100	*	TYPE=SUBSTITUTE
CM032588.1	32388	.	C	A	100	*	TYPE=SUBSTITUTE
CM032588.1	32394	.	A	G	100	*	TYPE=SUBSTITUTE
CM032588.1	32396	.	T	C	100	*	TYPE=SUBSTITUTE
CM032588.1	32407	.	T	G	100	*	TYPE=SUBSTITUTE
CM032588.1	32416	.	G	A	100	*	TYPE=SUBSTITUTE
CM032588.1	32463	.	T	C	100	*	TYPE=SUBSTITUTE
CM032588.1	32464	.	G	T	100	*	TYPE=SUBSTITUTE
CM032588.1	32467	.	CTACTCCG	C	100	*	TYPE=DELETE
CM032588.1	32486	.	A	G	100	*	TYPE=SUBSTITUTE
CM032588.1	32488	.	A	G	100	*	TYPE=SUBSTITUTE
CM032588.1	32490	.	A	G	100	*	TYPE=SUBSTITUTE
CM032588.1	32492	.	A	G	100	*	TYPE=SUBSTITUTE
CM032588.1	32493	.	A	T	100	*	TYPE=SUBSTITUTE
CM032588.1	32525	.	C	T	100	*	TYPE=SUBSTITUTE
CM032588.1	32529	.	G	C	100	*	TYPE=SUBSTITUTE
CM032588.1	32534	.	T	C	100	*	TYPE=SUBSTITUTE
CM032588.1	32548	.	C	G	100	*	TYPE=SUBSTITUTE
CM032588.1	32564	.	C	g	100	*	TYPE=SUBSTITUTE
CM032588.1	32567	.	A	g	100	*	TYPE=SUBSTITUTE
CM032588.1	32586	.	C	T	100	*	TYPE=SUBSTITUTE
CM032588.1	32612	.	G	A	100	*	TYPE=SUBSTITUTE
CM032588.1	32636	.	T	C	100	*	TYPE=SUBSTITUTE
CM032588.1	32642	.	A	G	100	*	TYPE=SUBSTITUTE
CM032588.1	32726	.	T	C	100	*	TYPE=SUBSTITUTE
CM032588.1	32754	.	A	G	100	*	TYPE=SUBSTITUTE
CM032588.1	32767	.	A	C	100	*	TYPE=SUBSTITUTE
CM032588.1	32776	.	G	A	100	*	TYPE=SUBSTITUTE
CM032588.1	32777	.	A	G	100	*	TYPE=SUBSTITUTE
CM032588.1	32804	.	A	G	100	*	TYPE=SUBSTITUTE
CM032588.1	32811	.	C	T	100	*	TYPE=SUBSTITUTE
CM032588.1	32848	.	T	G	100	*	TYPE=SUBSTITUTE
CM032588.1	32859	.	G	A	100	*	TYPE=SUBSTITUTE
CM032588.1	32866	.	A	G	100	*	TYPE=SUBSTITUTE
CM032588.1	32872	.	A	G	100	*	TYPE=SUBSTITUTE
CM032588.1	32874	.	G	A	100	*	TYPE=SUBSTITUTE
CM032588.1	32875	.	T	G	100	*	TYPE=SUBSTITUTE
CM032588.1	32877	.	T	A	100	*	TYPE=SUBSTITUTE
CM032588.1	32900	.	C	G	100	*	TYPE=SUBSTITUTE
CM032588.1	32901	.	C	A	100	*	TYPE=SUBSTITUTE
CM032588.1	32906	.	A	G	100	*	TYPE=SUBSTITUTE
CM032588.1	32926	.	T	C	100	*	TYPE=SUBSTITUTE
CM032588.1	32939	.	C	T	100	*	TYPE=SUBSTITUTE
CM032588.1	32968	.	C	T	100	*	TYPE=SUBSTITUTE
CM032588.1	32974	.	C	T	100	*	TYPE=SUBSTITUTE
CM032588.1	32978	.	C	T	100	*	TYPE=SUBSTITUTE
CM032588.1	32980	.	G	A	100	*	TYPE=SUBSTITUTE
CM032588.1	32981	.	C	T	100	*	TYPE=SUBSTITUTE
CM032588.1	33019	.	A	G	100	*	TYPE=SUBSTITUTE
CM032588.1	33094	.	T	G	100	*	TYPE=SUBSTITUTE
CM032588.1	33110	.	T	C	100	*	TYPE=SUBSTITUTE
CM032588.1	33111	.	A	T	100	*	TYPE=SUBSTITUTE
CM032588.1	33115	.	A	G	100	*	TYPE=SUBSTITUTE
CM032588.1	33121	.	C	T	100	*	TYPE=SUBSTITUTE
CM032588.1	33131	.	A	G	100	*	TYPE=SUBSTITUTE
CM032588.1	33134	.	A	C	100	*	TYPE=SUBSTITUTE

CM032588.1	33160	.	A	G	100	*	TYPE=SUBSTITUTE
CM032588.1	33163	.	G	A	100	*	TYPE=SUBSTITUTE
CM032588.1	33165	.	G	A	100	*	TYPE=SUBSTITUTE
CM032588.1	33186	.	C	A	100	*	TYPE=SUBSTITUTE
CM032588.1	33203	.	A	T	100	*	TYPE=SUBSTITUTE
CM032588.1	33228	.	G	A	100	*	TYPE=SUBSTITUTE
CM032588.1	33230	.	C	T	100	*	TYPE=SUBSTITUTE
CM032588.1	33234	.	G	A	100	*	TYPE=SUBSTITUTE
CM032588.1	33797	.	G	T	100	*	TYPE=SUBSTITUTE
CM032588.1	33805	.	A	T	100	*	TYPE=SUBSTITUTE
CM032588.1	33810	.	T	C	100	*	TYPE=SUBSTITUTE
CM032588.1	33840	.	C	A	100	*	TYPE=SUBSTITUTE
CM032588.1	33861	.	C	T	100	*	TYPE=SUBSTITUTE
CM032588.1	33869	.	A	T	100	*	TYPE=SUBSTITUTE
CM032588.1	33878	.	C	T	100	*	TYPE=SUBSTITUTE
CM032588.1	33909	.	A	G	100	*	TYPE=SUBSTITUTE
CM032588.1	33911	.	T	C	100	*	TYPE=SUBSTITUTE
CM032588.1	33930	.	G	A	100	*	TYPE=SUBSTITUTE
CM032588.1	33978	.	C	A	100	*	TYPE=SUBSTITUTE
CM032588.1	34206	.	C	A	100	*	TYPE=SUBSTITUTE
CM032588.1	34289	.	G	A	100	*	TYPE=SUBSTITUTE
CM032588.1	35085	.	G	A	100	*	TYPE=SUBSTITUTE
CM032588.1	35290	.	T	C	100	*	TYPE=SUBSTITUTE
CM032588.1	36547	.	T	C	100	*	TYPE=SUBSTITUTE
CM032588.1	37168	.	G	T	100	*	TYPE=SUBSTITUTE
CM032588.1	37200	.	A	G	100	*	TYPE=SUBSTITUTE
CM032588.1	37257	.	GG	G	100	*	TYPE=DELETE
CM032588.1	37296	.	A	AA	100	*	TYPE=INSERT
CM032588.1	37297	.	G	A	100	*	TYPE=SUBSTITUTE
CM032588.1	37303	.	T	C	100	*	TYPE=SUBSTITUTE
CM032588.1	37311	.	T	C	100	*	TYPE=SUBSTITUTE
CM032588.1	37324	.	C	T	100	*	TYPE=SUBSTITUTE
CM032588.1	37354	.	T	G	100	*	TYPE=SUBSTITUTE
CM032588.1	37361	.	C	T	100	*	TYPE=SUBSTITUTE
CM032588.1	37373	.	A	G	100	*	TYPE=SUBSTITUTE
CM032588.1	37378	.	T	C	100	*	TYPE=SUBSTITUTE
CM032588.1	37389	.	A	G	100	*	TYPE=SUBSTITUTE
CM032588.1	37404	.	C	A	100	*	TYPE=SUBSTITUTE
CM032588.1	37412	.	T	A	100	*	TYPE=SUBSTITUTE
CM032588.1	37417	.	A	G	100	*	TYPE=SUBSTITUTE
CM032588.1	37449	.	C	G	100	*	TYPE=SUBSTITUTE
CM032588.1	38135	.	A	G	100	*	TYPE=SUBSTITUTE
CM032588.1	38137	.	C	T	100	*	TYPE=SUBSTITUTE
CM032588.1	38141	.	C	T	100	*	TYPE=SUBSTITUTE
CM032588.1	38146	.	G	A	100	*	TYPE=SUBSTITUTE
CM032588.1	38150	.	T	C	100	*	TYPE=SUBSTITUTE
CM032588.1	38157	.	G	T	100	*	TYPE=SUBSTITUTE
CM032588.1	38174	.	C	T	100	*	TYPE=SUBSTITUTE
CM032588.1	38211	.	C	G	100	*	TYPE=SUBSTITUTE
CM032588.1	38217	.	T	C	100	*	TYPE=SUBSTITUTE
CM032588.1	38242	.	T	C	100	*	TYPE=SUBSTITUTE
CM032588.1	38244	.	C	G	100	*	TYPE=SUBSTITUTE
CM032588.1	38262	.	G	C	100	*	TYPE=SUBSTITUTE
CM032588.1	38300	.	T	C	100	*	TYPE=SUBSTITUTE
CM032588.1	38379	.	G	A	100	*	TYPE=SUBSTITUTE
CM032588.1	38451	.	C	T	100	*	TYPE=SUBSTITUTE
CM032588.1	38660	.	A	G	100	*	TYPE=SUBSTITUTE
CM032588.1	38670	.	C	T	100	*	TYPE=SUBSTITUTE
CM032588.1	38707	.	G	A	100	*	TYPE=SUBSTITUTE
CM032588.1	38709	.	A	T	100	*	TYPE=SUBSTITUTE
CM032588.1	38720	.	C	T	100	*	TYPE=SUBSTITUTE
CM032588.1	38748	.	G	A	100	*	TYPE=SUBSTITUTE
CM032588.1	38938	.	G	A	100	*	TYPE=SUBSTITUTE
CM032588.1	39011	.	C	T	100	*	TYPE=SUBSTITUTE

CM032588.1	39109	.	G	T	100	*	TYPE=SUBSTITUTE
CM032588.1	39168	.	A	T	100	*	TYPE=SUBSTITUTE
CM032588.1	40267	.	C	T	100	*	TYPE=SUBSTITUTE
CM032588.1	40288	.	T	C	100	*	TYPE=SUBSTITUTE
CM032588.1	40384	.	T	C	100	*	TYPE=SUBSTITUTE
CM032588.1	40436	.	T	A	100	*	TYPE=SUBSTITUTE
CM032588.1	40478	.	C	T	100	*	TYPE=SUBSTITUTE
CM032588.1	40511	.	TT	T	100	*	TYPE=DELETE
CM032588.1	40608	.	C	A	100	*	TYPE=SUBSTITUTE
CM032588.1	40732	.	G	T	100	*	TYPE=SUBSTITUTE
CM032588.1	40785	.	A	G	100	*	TYPE=SUBSTITUTE
CM032588.1	40824	.	T	C	100	*	TYPE=SUBSTITUTE
CM032588.1	41056	.	T	C	100	*	TYPE=SUBSTITUTE
CM032588.1	41130	.	A	AA	100	*	TYPE=INSERT
CM032588.1	41254	.	T	A	100	*	TYPE=SUBSTITUTE
CM032588.1	41320	.	C	T	100	*	TYPE=SUBSTITUTE
CM032588.1	41645	.	A	G	100	*	TYPE=SUBSTITUTE
CM032588.1	41648	.	G	A	100	*	TYPE=SUBSTITUTE
CM032588.1	41929	.	A	G	100	*	TYPE=SUBSTITUTE
CM032588.1	42058	.	T	C	100	*	TYPE=SUBSTITUTE
CM032588.1	42063	.	T	C	100	*	TYPE=SUBSTITUTE
CM032588.1	42389	.	T	C	100	*	TYPE=SUBSTITUTE
CM032588.1	42630	.	C	G	100	*	TYPE=SUBSTITUTE
CM032588.1	42648	.	T	C	100	*	TYPE=SUBSTITUTE
CM032588.1	42732	.	T	A	100	*	TYPE=SUBSTITUTE
CM032588.1	42799	.	A	G	100	*	TYPE=SUBSTITUTE
CM032588.1	42805	.	A	G	100	*	TYPE=SUBSTITUTE
CM032588.1	42915	.	AA	A	100	*	TYPE=DELETE
CM032588.1	42917	.	A	g	100	*	TYPE=SUBSTITUTE
CM032588.1	42975	.	C	T	100	*	TYPE=SUBSTITUTE
CM032588.1	43039	.	C	T	100	*	TYPE=SUBSTITUTE
CM032588.1	43045	.	T	C	100	*	TYPE=SUBSTITUTE
CM032588.1	43067	.	T	C	100	*	TYPE=SUBSTITUTE
CM032588.1	43127	.	T	C	100	*	TYPE=SUBSTITUTE
CM032588.1	43345	.	A	G	100	*	TYPE=SUBSTITUTE
CM032588.1	43449	.	A	T	100	*	TYPE=SUBSTITUTE
CM032588.1	43503	.	G	T	100	*	TYPE=SUBSTITUTE
CM032588.1	43610	.	C	T	100	*	TYPE=SUBSTITUTE
CM032588.1	43626	.	T	C	100	*	TYPE=SUBSTITUTE
CM032588.1	43825	.	C	T	100	*	TYPE=SUBSTITUTE
CM032588.1	44186	.	A	G	100	*	TYPE=SUBSTITUTE
CM032588.1	44241	.	T	TT	100	*	TYPE=INSERT
CM032588.1	44296	.	T	C	100	*	TYPE=SUBSTITUTE
CM032588.1	44683	.	C	G	100	*	TYPE=SUBSTITUTE
CM032588.1	45158	.	C	T	100	*	TYPE=SUBSTITUTE
CM032588.1	45227	.	C	T	100	*	TYPE=SUBSTITUTE
CM032588.1	45400	.	C	T	100	*	TYPE=SUBSTITUTE
CM032588.1	45649	.	C	T	100	*	TYPE=SUBSTITUTE
CM032588.1	45734	.	T	C	100	*	TYPE=SUBSTITUTE
CM032588.1	45750	.	T	G	100	*	TYPE=SUBSTITUTE
CM032588.1	45753	.	TA	T	100	*	TYPE=DELETE
CM032588.1	45927	.	G	C	100	*	TYPE=SUBSTITUTE
CM032588.1	45953	.	G	A	100	*	TYPE=SUBSTITUTE
CM032588.1	45999	.	G	A	100	*	TYPE=SUBSTITUTE
CM032588.1	46011	.	A	C	100	*	TYPE=SUBSTITUTE
CM032588.1	46033	.	G	A	100	*	TYPE=SUBSTITUTE
CM032588.1	46207	.	G	A	100	*	TYPE=SUBSTITUTE
CM032588.1	46223	.	C	T	100	*	TYPE=SUBSTITUTE
CM032588.1	46383	.	T	TAAT	100	*	TYPE=INSERT
CM032588.1	46514	.	C	G	100	*	TYPE=SUBSTITUTE
CM032588.1	46520	.	C	T	100	*	TYPE=SUBSTITUTE
CM032588.1	46633	.	A	T	100	*	TYPE=SUBSTITUTE
CM032588.1	46762	.	T	C	100	*	TYPE=SUBSTITUTE
CM032588.1	46836	.	A	T	100	*	TYPE=SUBSTITUTE

CM032588.1	46840	.	G	A	100	*	TYPE=SUBSTITUTE
CM032588.1	47045	.	T	C	100	*	TYPE=SUBSTITUTE
CM032588.1	47129	.	AAA	A	100	*	TYPE=DELETE
CM032588.1	47160	.	T	C	100	*	TYPE=SUBSTITUTE
CM032588.1	47250	.	A	G	100	*	TYPE=SUBSTITUTE
CM032588.1	47437	.	G	A	100	*	TYPE=SUBSTITUTE
CM032588.1	47681	.	GG	G	100	*	TYPE=DELETE
CM032588.1	47699	.	G	T	100	*	TYPE=SUBSTITUTE
CM032588.1	47825	.	A	C	100	*	TYPE=SUBSTITUTE
CM032588.1	47871	.	A	G	100	*	TYPE=SUBSTITUTE
CM032588.1	47952	.	CC	C	100	*	TYPE=DELETE
CM032588.1	48610	.	T	C	100	*	TYPE=SUBSTITUTE
CM032588.1	49309	.	A	G	100	*	TYPE=SUBSTITUTE
CM032588.1	49667	.	A	G	100	*	TYPE=SUBSTITUTE
CM032588.1	49682	.	A	G	100	*	TYPE=SUBSTITUTE
CM032588.1	49689	.	A	G	100	*	TYPE=SUBSTITUTE
CM032588.1	49721	.	A	g	100	*	TYPE=SUBSTITUTE
CM032588.1	49744	.	T	C	100	*	TYPE=SUBSTITUTE
CM032588.1	49745	.	C	T	100	*	TYPE=SUBSTITUTE
CM032588.1	49770	.	A	G	100	*	TYPE=SUBSTITUTE
CM032588.1	50424	.	CC	C	100	*	TYPE=DELETE
CM032588.1	50646	.	CAG	C	100	*	TYPE=DELETE
CM032588.1	51059	.	C	T	100	*	TYPE=SUBSTITUTE
CM032588.1	51141	.	T	C	100	*	TYPE=SUBSTITUTE
CM032588.1	51173	.	T	C	100	*	TYPE=SUBSTITUTE
CM032588.1	51261	.	A	G	100	*	TYPE=SUBSTITUTE
CM032588.1	51330	.	A	G	100	*	TYPE=SUBSTITUTE
CM032588.1	51414	.	G	A	100	*	TYPE=SUBSTITUTE
CM032588.1	51727	.	T	C	100	*	TYPE=SUBSTITUTE
CM032588.1	52448	.	A	C	100	*	TYPE=SUBSTITUTE
CM032588.1	52467	.	A	G	100	*	TYPE=SUBSTITUTE
CM032588.1	52488	.	C	T	100	*	TYPE=SUBSTITUTE
CM032588.1	52600	.	T	C	100	*	TYPE=SUBSTITUTE
CM032588.1	52614	.	C	A	100	*	TYPE=SUBSTITUTE
CM032588.1	52686	.	A	G	100	*	TYPE=SUBSTITUTE
CM032588.1	52811	.	A	T	100	*	TYPE=SUBSTITUTE
CM032588.1	52890	.	T	C	100	*	TYPE=SUBSTITUTE
CM032588.1	53228	.	C	T	100	*	TYPE=SUBSTITUTE
CM032588.1	53340	.	T	C	100	*	TYPE=SUBSTITUTE
CM032588.1	53368	.	G	A	100	*	TYPE=SUBSTITUTE
CM032588.1	53411	.	T	C	100	*	TYPE=SUBSTITUTE
CM032588.1	53489	.	T	C	100	*	TYPE=SUBSTITUTE
CM032588.1	53549	.	C	T	100	*	TYPE=SUBSTITUTE
CM032588.1	53576	.	G	A	100	*	TYPE=SUBSTITUTE
CM032588.1	53582	.	G	A	100	*	TYPE=SUBSTITUTE
CM032588.1	53615	.	C	T	100	*	TYPE=SUBSTITUTE
CM032588.1	53715	.	A	G	100	*	TYPE=SUBSTITUTE
CM032588.1	53854	.	T	C	100	*	TYPE=SUBSTITUTE
CM032588.1	53891	.	C	T	100	*	TYPE=SUBSTITUTE
CM032588.1	53897	.	A	C	100	*	TYPE=SUBSTITUTE
CM032588.1	53963	.	G	A	100	*	TYPE=SUBSTITUTE
CM032588.1	54050	.	C	T	100	*	TYPE=SUBSTITUTE
CM032588.1	54051	.	A	G	100	*	TYPE=SUBSTITUTE
CM032588.1	54057	.	G	A	100	*	TYPE=SUBSTITUTE
CM032588.1	54062	.	T	G	100	*	TYPE=SUBSTITUTE
CM032588.1	54074	.	G	A	100	*	TYPE=SUBSTITUTE
CM032588.1	54143	.	G	A	100	*	TYPE=SUBSTITUTE
CM032588.1	54171	.	G	A	100	*	TYPE=SUBSTITUTE
CM032588.1	54178	.	G	C	100	*	TYPE=SUBSTITUTE
CM032588.1	54240	.	G	A	100	*	TYPE=SUBSTITUTE
CM032588.1	54287	.	C	T	100	*	TYPE=SUBSTITUTE
CM032588.1	54299	.	G	A	100	*	TYPE=SUBSTITUTE
CM032588.1	54326	.	T	C	100	*	TYPE=SUBSTITUTE
CM032588.1	54353	.	T	C	100	*	TYPE=SUBSTITUTE

CM032588.1	54356	.	C	A	100	*	TYPE=SUBSTITUTE
CM032588.1	54361	.	T	C	100	*	TYPE=SUBSTITUTE
CM032588.1	54378	.	T	C	100	*	TYPE=SUBSTITUTE
CM032588.1	54404	.	G	C	100	*	TYPE=SUBSTITUTE
CM032588.1	54416	.	G	A	100	*	TYPE=SUBSTITUTE
CM032588.1	54428	.	A	G	100	*	TYPE=SUBSTITUTE
CM032588.1	54469	.	G	T	100	*	TYPE=SUBSTITUTE
CM032588.1	54494	.	G	A	100	*	TYPE=SUBSTITUTE
CM032588.1	54504	.	G	T	100	*	TYPE=SUBSTITUTE
CM032588.1	54507	.	A	G	100	*	TYPE=SUBSTITUTE
CM032588.1	54510	.	G	A	100	*	TYPE=SUBSTITUTE
CM032588.1	54514	.	G	C	100	*	TYPE=SUBSTITUTE
CM032588.1	54527	.	C	T	100	*	TYPE=SUBSTITUTE
CM032588.1	54545	.	G	A	100	*	TYPE=SUBSTITUTE
CM032588.1	54575	.	G	A	100	*	TYPE=SUBSTITUTE
CM032588.1	54585	.	A	G	100	*	TYPE=SUBSTITUTE
CM032588.1	54596	.	G	A	100	*	TYPE=SUBSTITUTE
CM032588.1	54602	.	T	C	100	*	TYPE=SUBSTITUTE
CM032588.1	54608	.	G	C	100	*	TYPE=SUBSTITUTE
CM032588.1	54615	.	A	G	100	*	TYPE=SUBSTITUTE
CM032588.1	54616	.	C	T	100	*	TYPE=SUBSTITUTE
CM032588.1	54635	.	C	G	100	*	TYPE=SUBSTITUTE
CM032588.1	54638	.	T	C	100	*	TYPE=SUBSTITUTE
CM032588.1	54659	.	C	T	100	*	TYPE=SUBSTITUTE
CM032588.1	54677	.	T	A	100	*	TYPE=SUBSTITUTE
CM032588.1	54719	.	A	C	100	*	TYPE=SUBSTITUTE
CM032588.1	54725	.	C	A	100	*	TYPE=SUBSTITUTE
CM032588.1	54736	.	T	C	100	*	TYPE=SUBSTITUTE
CM032588.1	54782	.	A	G	100	*	TYPE=SUBSTITUTE
CM032588.1	54785	.	G	C	100	*	TYPE=SUBSTITUTE
CM032588.1	54817	.	G	A	100	*	TYPE=SUBSTITUTE
CM032588.1	54820	.	G	A	100	*	TYPE=SUBSTITUTE
CM032588.1	54840	.	A	G	100	*	TYPE=SUBSTITUTE
CM032588.1	54874	.	T	C	100	*	TYPE=SUBSTITUTE
CM032588.1	54875	.	C	T	100	*	TYPE=SUBSTITUTE
CM032588.1	54907	.	C	A	100	*	TYPE=SUBSTITUTE
CM032588.1	54977	.	A	G	100	*	TYPE=SUBSTITUTE
CM032588.1	55037	.	A	G	100	*	TYPE=SUBSTITUTE
CM032588.1	55038	.	G	A	100	*	TYPE=SUBSTITUTE
CM032588.1	55067	.	T	C	100	*	TYPE=SUBSTITUTE
CM032588.1	55076	.	A	G	100	*	TYPE=SUBSTITUTE
CM032588.1	55139	.	C	T	100	*	TYPE=SUBSTITUTE
CM032588.1	55175	.	A	G	100	*	TYPE=SUBSTITUTE
CM032588.1	55184	.	G	A	100	*	TYPE=SUBSTITUTE
CM032588.1	55188	.	A	T	100	*	TYPE=SUBSTITUTE
CM032588.1	55241	.	G	a	100	*	TYPE=SUBSTITUTE
CM032588.1	55256	.	G	A	100	*	TYPE=SUBSTITUTE
CM032588.1	55274	.	G	C	100	*	TYPE=SUBSTITUTE
CM032588.1	55289	.	A	G	100	*	TYPE=SUBSTITUTE
CM032588.1	55382	.	T	C	100	*	TYPE=SUBSTITUTE
CM032588.1	55418	.	T	C	100	*	TYPE=SUBSTITUTE
CM032588.1	55435	.	G	A	100	*	TYPE=SUBSTITUTE
CM032588.1	55457	.	A	G	100	*	TYPE=SUBSTITUTE
CM032588.1	55469	.	G	A	100	*	TYPE=SUBSTITUTE
CM032588.1	55502	.	T	G	100	*	TYPE=SUBSTITUTE
CM032588.1	55524	.	T	C	100	*	TYPE=SUBSTITUTE
CM032588.1	55528	.	C	G	100	*	TYPE=SUBSTITUTE
CM032588.1	55544	.	G	A	100	*	TYPE=SUBSTITUTE
CM032588.1	55558	.	G	C	100	*	TYPE=SUBSTITUTE
CM032588.1	55582	.	T	C	100	*	TYPE=SUBSTITUTE
CM032588.1	55586	.	C	T	100	*	TYPE=SUBSTITUTE
CM032588.1	55613	.	A	C	100	*	TYPE=SUBSTITUTE
CM032588.1	55685	.	C	T	100	*	TYPE=SUBSTITUTE
CM032588.1	55805	.	C	T	100	*	TYPE=SUBSTITUTE

CM032588.1	55814	.	T	C	100	*	TYPE=SUBSTITUTE
CM032588.1	55841	.	C	T	100	*	TYPE=SUBSTITUTE
CM032588.1	55849	.	G	T	100	*	TYPE=SUBSTITUTE
CM032588.1	55870	.	A	G	100	*	TYPE=SUBSTITUTE
CM032588.1	55880	.	C	T	100	*	TYPE=SUBSTITUTE
CM032588.1	55910	.	C	A	100	*	TYPE=SUBSTITUTE
CM032588.1	55969	.	C	T	100	*	TYPE=SUBSTITUTE
CM032588.1	56000	.	A	G	100	*	TYPE=SUBSTITUTE
CM032588.1	56006	.	A	G	100	*	TYPE=SUBSTITUTE
CM032588.1	56042	.	A	G	100	*	TYPE=SUBSTITUTE
CM032588.1	56105	.	C	T	100	*	TYPE=SUBSTITUTE
CM032588.1	56168	.	G	T	100	*	TYPE=SUBSTITUTE
CM032588.1	56170	.	G	T	100	*	TYPE=SUBSTITUTE
CM032588.1	56298	.	T	C	100	*	TYPE=SUBSTITUTE
CM032588.1	56306	.	G	A	100	*	TYPE=SUBSTITUTE
CM032588.1	56307	.	C	T	100	*	TYPE=SUBSTITUTE
CM032588.1	56322	.	A	G	100	*	TYPE=SUBSTITUTE
CM032588.1	56328	.	G	T	100	*	TYPE=SUBSTITUTE
CM032588.1	56352	.	C	T	100	*	TYPE=SUBSTITUTE
CM032588.1	56372	.	T	C	100	*	TYPE=SUBSTITUTE
CM032588.1	56414	.	G	A	100	*	TYPE=SUBSTITUTE
CM032588.1	56471	.	C	G	100	*	TYPE=SUBSTITUTE
CM032588.1	56477	.	C	A	100	*	TYPE=SUBSTITUTE
CM032588.1	56495	.	T	G	100	*	TYPE=SUBSTITUTE
CM032588.1	56504	.	C	G	100	*	TYPE=SUBSTITUTE
CM032588.1	56558	.	A	G	100	*	TYPE=SUBSTITUTE
CM032588.1	56666	.	G	T	100	*	TYPE=SUBSTITUTE
CM032588.1	56687	.	A	G	100	*	TYPE=SUBSTITUTE
CM032588.1	56689	.	C	T	100	*	TYPE=SUBSTITUTE
CM032588.1	56692	.	C	T	100	*	TYPE=SUBSTITUTE
CM032588.1	56747	.	G	A	100	*	TYPE=SUBSTITUTE
CM032588.1	56769	.	T	C	100	*	TYPE=SUBSTITUTE
CM032588.1	56772	.	A	G	100	*	TYPE=SUBSTITUTE
CM032588.1	56785	.	T	A	100	*	TYPE=SUBSTITUTE
CM032588.1	56819	.	T	G	100	*	TYPE=SUBSTITUTE
CM032588.1	56821	.	C	T	100	*	TYPE=SUBSTITUTE
CM032588.1	56834	.	C	T	100	*	TYPE=SUBSTITUTE
CM032588.1	56843	.	T	C	100	*	TYPE=SUBSTITUTE
CM032588.1	56852	.	A	G	100	*	TYPE=SUBSTITUTE
CM032588.1	56905	.	TGT	T	100	*	TYPE=DELETE
CM032588.1	56965	.	G	A	100	*	TYPE=SUBSTITUTE
CM032588.1	56967	.	T	C	100	*	TYPE=SUBSTITUTE
CM032588.1	56974	.	A	T	100	*	TYPE=SUBSTITUTE
CM032588.1	57032	.	C	T	100	*	TYPE=SUBSTITUTE
CM032588.1	57036	.	T	G	100	*	TYPE=SUBSTITUTE
CM032588.1	57051	.	C	T	100	*	TYPE=SUBSTITUTE
CM032588.1	57060	.	C	T	100	*	TYPE=SUBSTITUTE
CM032588.1	57064	.	C	T	100	*	TYPE=SUBSTITUTE
CM032588.1	57163	.	G	a	100	*	TYPE=SUBSTITUTE
CM032588.1	57175	.	A	g	100	*	TYPE=SUBSTITUTE
CM032588.1	57203	.	A	G	100	*	TYPE=SUBSTITUTE
CM032588.1	57208	.	C	T	100	*	TYPE=SUBSTITUTE
CM032588.1	57238	.	A	G	100	*	TYPE=SUBSTITUTE
CM032588.1	57258	.	T	C	100	*	TYPE=SUBSTITUTE
CM032588.1	57263	.	G	A	100	*	TYPE=SUBSTITUTE
CM032588.1	57264	.	C	G	100	*	TYPE=SUBSTITUTE
CM032588.1	57272	.	T	C	100	*	TYPE=SUBSTITUTE
CM032588.1	57289	.	A	G	100	*	TYPE=SUBSTITUTE
CM032588.1	57443	.	T	C	100	*	TYPE=SUBSTITUTE
CM032588.1	57465	.	T	C	100	*	TYPE=SUBSTITUTE
CM032588.1	57478	.	G	A	100	*	TYPE=SUBSTITUTE
CM032588.1	57480	.	C	G	100	*	TYPE=SUBSTITUTE
CM032588.1	57493	.	A	C	100	*	TYPE=SUBSTITUTE
CM032588.1	57504	.	A	C	100	*	TYPE=SUBSTITUTE

CM032588.1	57512	.	A	G	100	*	TYPE=SUBSTITUTE
CM032588.1	57533	.	G	A	100	*	TYPE=SUBSTITUTE
CM032588.1	57534	.	C	T	100	*	TYPE=SUBSTITUTE
CM032588.1	57549	.	G	A	100	*	TYPE=SUBSTITUTE
CM032588.1	57555	.	C	T	100	*	TYPE=SUBSTITUTE
CM032588.1	57566	.	C	T	100	*	TYPE=SUBSTITUTE
CM032588.1	57570	.	A	G	100	*	TYPE=SUBSTITUTE
CM032588.1	57585	.	A	AC	100	*	TYPE=INSERT
CM032588.1	57589	.	G	A	100	*	TYPE=SUBSTITUTE
CM032588.1	57590	.	A	AT	100	*	TYPE=INSERT
CM032588.1	57591	.	G	A	100	*	TYPE=SUBSTITUTE
CM032588.1	57596	.	A	C	100	*	TYPE=SUBSTITUTE
CM032588.1	57622	.	G	A	100	*	TYPE=SUBSTITUTE
CM032588.1	57629	.	A	C	100	*	TYPE=SUBSTITUTE
CM032588.1	57690	.	T	C	100	*	TYPE=SUBSTITUTE
CM032588.1	57697	.	G	A	100	*	TYPE=SUBSTITUTE
CM032588.1	57699	.	G	A	100	*	TYPE=SUBSTITUTE
CM032588.1	57700	.	T	G	100	*	TYPE=SUBSTITUTE
CM032588.1	57701	.	A	G	100	*	TYPE=SUBSTITUTE
CM032588.1	57717	.	A	C	100	*	TYPE=SUBSTITUTE
CM032588.1	57721	.	G	A	100	*	TYPE=SUBSTITUTE
CM032588.1	57722	.	G	A	100	*	TYPE=SUBSTITUTE
CM032588.1	57730	.	A	G	100	*	TYPE=SUBSTITUTE
CM032588.1	57754	.	G	T	100	*	TYPE=SUBSTITUTE
CM032588.1	57763	.	T	A	100	*	TYPE=SUBSTITUTE
CM032588.1	57764	.	C	G	100	*	TYPE=SUBSTITUTE
CM032588.1	57781	.	G	A	100	*	TYPE=SUBSTITUTE
CM032588.1	57788	.	T	C	100	*	TYPE=SUBSTITUTE
CM032588.1	57814	.	A	t	100	*	TYPE=SUBSTITUTE
CM032588.1	57821	.	T	C	100	*	TYPE=SUBSTITUTE
CM032588.1	57822	.	G	C	100	*	TYPE=SUBSTITUTE
CM032588.1	57833	.	C	T	100	*	TYPE=SUBSTITUTE
CM032588.1	57846	.	G	T	100	*	TYPE=SUBSTITUTE
CM032588.1	57850	.	A	G	100	*	TYPE=SUBSTITUTE
CM032588.1	57855	.	G	A	100	*	TYPE=SUBSTITUTE
CM032588.1	57870	.	G	C	100	*	TYPE=SUBSTITUTE
CM032588.1	57878	.	T	C	100	*	TYPE=SUBSTITUTE
CM032588.1	57880	.	G	A	100	*	TYPE=SUBSTITUTE
CM032588.1	57889	.	G	A	100	*	TYPE=SUBSTITUTE
CM032588.1	57895	.	T	C	100	*	TYPE=SUBSTITUTE
CM032588.1	57910	.	C	T	100	*	TYPE=SUBSTITUTE
CM032588.1	57917	.	C	G	100	*	TYPE=SUBSTITUTE
CM032588.1	57941	.	A	AGCGTTGGCTGTGAATT	100	*	TYPE=INSERT
CM032588.1	57942	.	C	G	100	*	TYPE=SUBSTITUTE
CM032588.1	57955	.	T	C	100	*	TYPE=SUBSTITUTE
CM032588.1	57968	.	T	C	100	*	TYPE=SUBSTITUTE
CM032588.1	57987	.	G	C	100	*	TYPE=SUBSTITUTE
CM032588.1	58014	.	A	G	100	*	TYPE=SUBSTITUTE
CM032588.1	58030	.	A	G	100	*	TYPE=SUBSTITUTE
CM032588.1	58038	.	G	A	100	*	TYPE=SUBSTITUTE
CM032588.1	58041	.	C	G	100	*	TYPE=SUBSTITUTE
CM032588.1	58052	.	A	C	100	*	TYPE=SUBSTITUTE
CM032588.1	58053	.	G	A	100	*	TYPE=SUBSTITUTE
CM032588.1	58054	.	C	T	100	*	TYPE=SUBSTITUTE
CM032588.1	58060	.	T	C	100	*	TYPE=SUBSTITUTE
CM032588.1	58064	.	G	C	100	*	TYPE=SUBSTITUTE
CM032588.1	58078	.	G	T	100	*	TYPE=SUBSTITUTE
CM032588.1	58086	.	C	A	100	*	TYPE=SUBSTITUTE
CM032588.1	58096	.	C	G	100	*	TYPE=SUBSTITUTE
CM032588.1	58097	.	T	C	100	*	TYPE=SUBSTITUTE
CM032588.1	58102	.	C	G	100	*	TYPE=SUBSTITUTE
CM032588.1	58114	.	T	C	100	*	TYPE=SUBSTITUTE
CM032588.1	58123	.	C	T	100	*	TYPE=SUBSTITUTE
CM032588.1	58138	.	G	T	100	*	TYPE=SUBSTITUTE

CM032588.1	58146	.	A	G	100	*	TYPE=SUBSTITUTE
CM032588.1	58167	.	C	A	100	*	TYPE=SUBSTITUTE
CM032588.1	58221	.	C	T	100	*	TYPE=SUBSTITUTE
CM032588.1	58228	.	C	G	100	*	TYPE=SUBSTITUTE
CM032588.1	58232	.	A	G	100	*	TYPE=SUBSTITUTE
CM032588.1	58259	.	C	T	100	*	TYPE=SUBSTITUTE
CM032588.1	58264	.	G	T	100	*	TYPE=SUBSTITUTE
CM032588.1	58265	.	AT	A	100	*	TYPE=DELETE
CM032588.1	58283	.	C	A	100	*	TYPE=SUBSTITUTE
CM032588.1	58284	.	C	A	100	*	TYPE=SUBSTITUTE
CM032588.1	58285	.	T	A	100	*	TYPE=SUBSTITUTE
CM032588.1	58286	.	T	A	100	*	TYPE=SUBSTITUTE
CM032588.1	58287	.	TTC	T	100	*	TYPE=DELETE
CM032588.1	58294	.	C	T	100	*	TYPE=SUBSTITUTE
CM032588.1	58295	.	A	G	100	*	TYPE=SUBSTITUTE
CM032588.1	58307	.	C	T	100	*	TYPE=SUBSTITUTE
CM032588.1	58328	.	G	A	100	*	TYPE=SUBSTITUTE
CM032588.1	58332	.	G	T	100	*	TYPE=SUBSTITUTE
CM032588.1	58346	.	C	T	100	*	TYPE=SUBSTITUTE
CM032588.1	58356	.	A	AAATATGCTAAGTACAACAGTC	100	*	TYPE=INSERT
CM032588.1	58380	.	T	G	100	*	TYPE=SUBSTITUTE
CM032588.1	58383	.	T	A	100	*	TYPE=SUBSTITUTE
CM032588.1	58384	.	C	A	100	*	TYPE=SUBSTITUTE
CM032588.1	58385	.	C	T	100	*	TYPE=SUBSTITUTE
CM032588.1	58406	.	G	GG	100	*	TYPE=INSERT
CM032588.1	58415	.	A	G	100	*	TYPE=SUBSTITUTE
CM032588.1	58442	.	G	C	100	*	TYPE=SUBSTITUTE
CM032588.1	58457	.	G	A	100	*	TYPE=SUBSTITUTE
CM032588.1	58476	.	C	G	100	*	TYPE=SUBSTITUTE
CM032588.1	58512	.	G	C	100	*	TYPE=SUBSTITUTE
CM032588.1	58534	.	A	T	100	*	TYPE=SUBSTITUTE
CM032588.1	58606	.	A	G	100	*	TYPE=SUBSTITUTE
CM032588.1	58632	.	C	CCTGC	100	*	TYPE=INSERT
CM032588.1	58633	.	G	A	100	*	TYPE=SUBSTITUTE
CM032588.1	58636	.	G	T	100	*	TYPE=SUBSTITUTE
CM032588.1	58668	.	T	C	100	*	TYPE=SUBSTITUTE
CM032588.1	58675	.	C	T	100	*	TYPE=SUBSTITUTE
CM032588.1	58676	.	G	A	100	*	TYPE=SUBSTITUTE
CM032588.1	58697	.	TATAGTAGTCTGTGCCCGTGCTGAAGGTAATACGCACAC	T	100	*	TYPE=DELETE
CM032588.1	58755	.	C	T	100	*	TYPE=SUBSTITUTE
CM032588.1	58756	.	A	G	100	*	TYPE=SUBSTITUTE
CM032588.1	58775	.	C	T	100	*	TYPE=SUBSTITUTE
CM032588.1	58778	.	T	C	100	*	TYPE=SUBSTITUTE
CM032588.1	58780	.	G	A	100	*	TYPE=SUBSTITUTE
CM032588.1	58789	.	A	G	100	*	TYPE=SUBSTITUTE
CM032588.1	58790	.	T	G	100	*	TYPE=SUBSTITUTE
CM032588.1	58795	.	AT	A	100	*	TYPE=DELETE
CM032588.1	58798	.	T	G	100	*	TYPE=SUBSTITUTE
CM032588.1	58804	.	T	C	100	*	TYPE=SUBSTITUTE
CM032588.1	58806	.	T	C	100	*	TYPE=SUBSTITUTE
CM032588.1	58807	.	G	T	100	*	TYPE=SUBSTITUTE
CM032588.1	58811	.	C	T	100	*	TYPE=SUBSTITUTE
CM032588.1	58813	.	G	A	100	*	TYPE=SUBSTITUTE
CM032588.1	58820	.	A	C	100	*	TYPE=SUBSTITUTE
CM032588.1	58822	.	T	A	100	*	TYPE=SUBSTITUTE
CM032588.1	58827	.	C	T	100	*	TYPE=SUBSTITUTE
CM032588.1	58842	.	A	G	100	*	TYPE=SUBSTITUTE
CM032588.1	58864	.	A	G	100	*	TYPE=SUBSTITUTE
CM032588.1	58897	.	A	G	100	*	TYPE=SUBSTITUTE
CM032588.1	58903	.	A	G	100	*	TYPE=SUBSTITUTE
CM032588.1	58927	.	C	T	100	*	TYPE=SUBSTITUTE
CM032588.1	58933	.	A	G	100	*	TYPE=SUBSTITUTE
CM032588.1	58947	.	T	C	100	*	TYPE=SUBSTITUTE

CM032588.1	58951	.	T	C	100	*	TYPE=SUBSTITUTE
CM032588.1	58979	.	A	C	100	*	TYPE=SUBSTITUTE
CM032588.1	58981	.	A	C	100	*	TYPE=SUBSTITUTE
CM032588.1	58984	.	C	T	100	*	TYPE=SUBSTITUTE
CM032588.1	58986	.	A	C	100	*	TYPE=SUBSTITUTE
CM032588.1	58988	.	A	G	100	*	TYPE=SUBSTITUTE
CM032588.1	59013	.	T	A	100	*	TYPE=SUBSTITUTE
CM032588.1	59014	.	T	C	100	*	TYPE=SUBSTITUTE
CM032588.1	59020	.	A	G	100	*	TYPE=SUBSTITUTE
CM032588.1	59021	.	A	G	100	*	TYPE=SUBSTITUTE
CM032588.1	59026	.	G	C	100	*	TYPE=SUBSTITUTE
CM032588.1	59037	.	A	G	100	*	TYPE=SUBSTITUTE
CM032588.1	59056	.	T	G	100	*	TYPE=SUBSTITUTE
CM032588.1	59060	.	T	C	100	*	TYPE=SUBSTITUTE
CM032588.1	59062	.	G	C	100	*	TYPE=SUBSTITUTE
CM032588.1	59068	.	G	A	100	*	TYPE=SUBSTITUTE
CM032588.1	59090	.	T	G	100	*	TYPE=SUBSTITUTE
CM032588.1	59111	.	TTT	T	100	*	TYPE=DELETE
CM032588.1	59157	.	A	G	100	*	TYPE=SUBSTITUTE
CM032588.1	59167	.	T	G	100	*	TYPE=SUBSTITUTE
CM032588.1	59183	.	G	A	100	*	TYPE=SUBSTITUTE
CM032588.1	59187	.	A	G	100	*	TYPE=SUBSTITUTE
CM032588.1	59190	.	G	A	100	*	TYPE=SUBSTITUTE
CM032588.1	59191	.	C	T	100	*	TYPE=SUBSTITUTE
CM032588.1	59193	.	T	C	100	*	TYPE=SUBSTITUTE
CM032588.1	59220	.	C	T	100	*	TYPE=SUBSTITUTE
CM032588.1	59221	.	G	GTAATA	100	*	TYPE=INSERT
CM032588.1	59252	.	C	G	100	*	TYPE=SUBSTITUTE
CM032588.1	59254	.	T	G	100	*	TYPE=SUBSTITUTE
CM032588.1	59273	.	C	T	100	*	TYPE=SUBSTITUTE
CM032588.1	59296	.	A	t	100	*	TYPE=SUBSTITUTE
CM032588.1	59308	.	G	A	100	*	TYPE=SUBSTITUTE
CM032588.1	59311	.	T	G	100	*	TYPE=SUBSTITUTE
CM032588.1	59359	.	T	C	100	*	TYPE=SUBSTITUTE
CM032588.1	59364	.	T	A	100	*	TYPE=SUBSTITUTE
CM032588.1	59368	.	T	C	100	*	TYPE=SUBSTITUTE
CM032588.1	59378	.	A	G	100	*	TYPE=SUBSTITUTE
CM032588.1	59379	.	A	G	100	*	TYPE=SUBSTITUTE
CM032588.1	59389	.	g	gtaa	100	*	TYPE=INSERT
CM032588.1	59407	.	A	G	100	*	TYPE=SUBSTITUTE
CM032588.1	59414	.	A	G	100	*	TYPE=SUBSTITUTE
CM032588.1	59489	.	T	C	100	*	TYPE=SUBSTITUTE
CM032588.1	59494	.	C	T	100	*	TYPE=SUBSTITUTE
CM032588.1	59540	.	G	C	100	*	TYPE=SUBSTITUTE
CM032588.1	59555	.	A	G	100	*	TYPE=SUBSTITUTE
CM032588.1	59563	.	G	A	100	*	TYPE=SUBSTITUTE
CM032588.1	59600	.	T	C	100	*	TYPE=SUBSTITUTE
CM032588.1	59627	.	C	A	100	*	TYPE=SUBSTITUTE
CM032588.1	59630	.	C	A	100	*	TYPE=SUBSTITUTE
CM032588.1	59649	.	A	C	100	*	TYPE=SUBSTITUTE
CM032588.1	59654	.	A	G	100	*	TYPE=SUBSTITUTE
CM032588.1	59659	.	G	A	100	*	TYPE=SUBSTITUTE
CM032588.1	59661	.	C	T	100	*	TYPE=SUBSTITUTE
CM032588.1	59677	.	G	A	100	*	TYPE=SUBSTITUTE
CM032588.1	59685	.	A	T	100	*	TYPE=SUBSTITUTE
CM032588.1	59692	.	C	T	100	*	TYPE=SUBSTITUTE
CM032588.1	59710	.	GAG	G	100	*	TYPE=DELETE
CM032588.1	59722	.	T	C	100	*	TYPE=SUBSTITUTE
CM032588.1	59729	.	A	G	100	*	TYPE=SUBSTITUTE
CM032588.1	59735	.	C	T	100	*	TYPE=SUBSTITUTE
CM032588.1	59755	.	C	T	100	*	TYPE=SUBSTITUTE
CM032588.1	59763	.	G	A	100	*	TYPE=SUBSTITUTE
CM032588.1	59776	.	C	T	100	*	TYPE=SUBSTITUTE
CM032588.1	59777	.	C	A	100	*	TYPE=SUBSTITUTE

CM032588.1	59789	.	C	G	100	*	TYPE=SUBSTITUTE
CM032588.1	59795	.	G	T	100	*	TYPE=SUBSTITUTE
CM032588.1	59796	.	C	T	100	*	TYPE=SUBSTITUTE
CM032588.1	59832	.	T	C	100	*	TYPE=SUBSTITUTE
CM032588.1	59900	.	GG	G	100	*	TYPE=DELETE
CM032588.1	60040	.	G	A	100	*	TYPE=SUBSTITUTE
CM032588.1	60089	.	T	C	100	*	TYPE=SUBSTITUTE
CM032588.1	60357	.	T	A	100	*	TYPE=SUBSTITUTE
CM032588.1	60373	.	A	C	100	*	TYPE=SUBSTITUTE
CM032588.1	61031	.	T	C	100	*	TYPE=SUBSTITUTE
CM032588.1	61235	.	T	C	100	*	TYPE=SUBSTITUTE
CM032588.1	61883	.	C	T	100	*	TYPE=SUBSTITUTE
CM032588.1	62167	.	C	G	100	*	TYPE=SUBSTITUTE
CM032588.1	62393	.	C	T	100	*	TYPE=SUBSTITUTE
CM032588.1	62399	.	G	A	100	*	TYPE=SUBSTITUTE
CM032588.1	62403	.	T	C	100	*	TYPE=SUBSTITUTE
CM032588.1	62455	.	C	A	100	*	TYPE=SUBSTITUTE
CM032588.1	62489	.	G	A	100	*	TYPE=SUBSTITUTE
CM032588.1	62493	.	A	G	100	*	TYPE=SUBSTITUTE
CM032588.1	62541	.	A	G	100	*	TYPE=SUBSTITUTE
CM032588.1	62569	.	A	C	100	*	TYPE=SUBSTITUTE
CM032588.1	62579	.	T	A	100	*	TYPE=SUBSTITUTE
CM032588.1	62580	.	T	C	100	*	TYPE=SUBSTITUTE
CM032588.1	62620	.	G	A	100	*	TYPE=SUBSTITUTE
CM032588.1	62642	.	G	A	100	*	TYPE=SUBSTITUTE
CM032588.1	62696	.	T	TGT	100	*	TYPE=INSERT
CM032588.1	62719	.	TT	T	100	*	TYPE=DELETE
CM032588.1	62735	.	T	C	100	*	TYPE=SUBSTITUTE
CM032588.1	62765	.	C	T	100	*	TYPE=SUBSTITUTE
CM032588.1	62766	.	C	T	100	*	TYPE=SUBSTITUTE
CM032588.1	62772	.	C	T	100	*	TYPE=SUBSTITUTE
CM032588.1	62781	.	T	c	100	*	TYPE=SUBSTITUTE
CM032588.1	62796	.	A	g	100	*	TYPE=SUBSTITUTE
CM032588.1	62817	.	C	t	100	*	TYPE=SUBSTITUTE
CM032588.1	62825	.	T	c	100	*	TYPE=SUBSTITUTE
CM032588.1	62837	.	G	C	100	*	TYPE=SUBSTITUTE
CM032588.1	62849	.	A	C	100	*	TYPE=SUBSTITUTE
CM032588.1	62983	.	G	A	100	*	TYPE=SUBSTITUTE
CM032588.1	62998	.	C	A	100	*	TYPE=SUBSTITUTE
CM032588.1	63070	.	A	G	100	*	TYPE=SUBSTITUTE
CM032588.1	63082	.	C	T	100	*	TYPE=SUBSTITUTE
CM032588.1	63091	.	T	C	100	*	TYPE=SUBSTITUTE
CM032588.1	63169	.	T	G	100	*	TYPE=SUBSTITUTE
CM032588.1	63217	.	T	C	100	*	TYPE=SUBSTITUTE
CM032588.1	63256	.	G	T	100	*	TYPE=SUBSTITUTE
CM032588.1	63268	.	T	C	100	*	TYPE=SUBSTITUTE
CM032588.1	63307	.	T	A	100	*	TYPE=SUBSTITUTE
CM032588.1	63320	.	C	a	100	*	TYPE=SUBSTITUTE
CM032588.1	63329	.	C	A	100	*	TYPE=SUBSTITUTE
CM032588.1	63345	.	A	G	100	*	TYPE=SUBSTITUTE
CM032588.1	63357	.	A	G	100	*	TYPE=SUBSTITUTE
CM032588.1	63388	.	TAG	T	100	*	TYPE=DELETE
CM032588.1	63438	.	C	T	100	*	TYPE=SUBSTITUTE
CM032588.1	63477	.	T	C	100	*	TYPE=SUBSTITUTE
CM032588.1	63690	.	TCT	T	100	*	TYPE=DELETE
CM032588.1	64124	.	T	C	100	*	TYPE=SUBSTITUTE
CM032588.1	64160	.	G	T	100	*	TYPE=SUBSTITUTE
CM032588.1	64578	.	A	G	100	*	TYPE=SUBSTITUTE
CM032588.1	65102	.	A	G	100	*	TYPE=SUBSTITUTE
CM032588.1	65126	.	A	G	100	*	TYPE=SUBSTITUTE
CM032588.1	65202	.	G	A	100	*	TYPE=SUBSTITUTE
CM032588.1	65534	.	A	G	100	*	TYPE=SUBSTITUTE
CM032588.1	65569	.	T	C	100	*	TYPE=SUBSTITUTE
CM032588.1	65720	.	T	A	100	*	TYPE=SUBSTITUTE

CM032588.1	65767	.	A	C	100	*	TYPE=SUBSTITUTE
CM032588.1	65815	.	G	C	100	*	TYPE=SUBSTITUTE
CM032588.1	65899	.	A	G	100	*	TYPE=SUBSTITUTE
CM032588.1	65951	.	G	A	100	*	TYPE=SUBSTITUTE
CM032588.1	65984	.	G	A	100	*	TYPE=SUBSTITUTE
CM032588.1	66164	.	G	A	100	*	TYPE=SUBSTITUTE
CM032588.1	66241	.	G	A	100	*	TYPE=SUBSTITUTE
CM032588.1	66274	.	T	A	100	*	TYPE=SUBSTITUTE
CM032588.1	66289	.	G	T	100	*	TYPE=SUBSTITUTE
CM032588.1	66306	.	T	C	100	*	TYPE=SUBSTITUTE
CM032588.1	66317	.	A	G	100	*	TYPE=SUBSTITUTE
CM032588.1	66504	.	C	T	100	*	TYPE=SUBSTITUTE
CM032588.1	66695	.	C	G	100	*	TYPE=SUBSTITUTE
CM032588.1	66810	.	CC	C	100	*	TYPE=DELETE
CM032588.1	66870	.	T	G	100	*	TYPE=SUBSTITUTE
CM032588.1	66871	.	A	T	100	*	TYPE=SUBSTITUTE
CM032588.1	66894	.	C	T	100	*	TYPE=SUBSTITUTE
CM032588.1	66910	.	G	T	100	*	TYPE=SUBSTITUTE
CM032588.1	66956	.	A	G	100	*	TYPE=SUBSTITUTE
CM032588.1	66958	.	C	T	100	*	TYPE=SUBSTITUTE
CM032588.1	66972	.	C	T	100	*	TYPE=SUBSTITUTE
CM032588.1	66983	.	C	T	100	*	TYPE=SUBSTITUTE
CM032588.1	67009	.	T	C	100	*	TYPE=SUBSTITUTE
CM032588.1	67027	.	C	G	100	*	TYPE=SUBSTITUTE
CM032588.1	67053	.	T	C	100	*	TYPE=SUBSTITUTE
CM032588.1	67060	.	C	T	100	*	TYPE=SUBSTITUTE
CM032588.1	67071	.	A	G	100	*	TYPE=SUBSTITUTE
CM032588.1	67081	.	A	G	100	*	TYPE=SUBSTITUTE
CM032588.1	67102	.	T	C	100	*	TYPE=SUBSTITUTE
CM032588.1	67137	.	G	T	100	*	TYPE=SUBSTITUTE
CM032588.1	67138	.	G	A	100	*	TYPE=SUBSTITUTE
CM032588.1	67154	.	C	A	100	*	TYPE=SUBSTITUTE
CM032588.1	67175	.	C	T	100	*	TYPE=SUBSTITUTE
CM032588.1	67223	.	G	A	100	*	TYPE=SUBSTITUTE
CM032588.1	67281	.	A	t	100	*	TYPE=SUBSTITUTE
CM032588.1	67346	.	G	A	100	*	TYPE=SUBSTITUTE
CM032588.1	67423	.	A	G	100	*	TYPE=SUBSTITUTE
CM032588.1	67424	.	G	A	100	*	TYPE=SUBSTITUTE
CM032588.1	67467	.	T	C	100	*	TYPE=SUBSTITUTE
CM032588.1	67487	.	C	G	100	*	TYPE=SUBSTITUTE
CM032588.1	67491	.	C	T	100	*	TYPE=SUBSTITUTE
CM032588.1	67500	.	T	C	100	*	TYPE=SUBSTITUTE
CM032588.1	67508	.	C	T	100	*	TYPE=SUBSTITUTE
CM032588.1	67575	.	G	A	100	*	TYPE=SUBSTITUTE
CM032588.1	67598	.	C	G	100	*	TYPE=SUBSTITUTE
CM032588.1	67628	.	C	T	100	*	TYPE=SUBSTITUTE
CM032588.1	67635	.	T	C	100	*	TYPE=SUBSTITUTE
CM032588.1	67648	.	T	G	100	*	TYPE=SUBSTITUTE
CM032588.1	67660	.	G	A	100	*	TYPE=SUBSTITUTE
CM032588.1	67661	.	C	T	100	*	TYPE=SUBSTITUTE
CM032588.1	67662	.	C	G	100	*	TYPE=SUBSTITUTE
CM032588.1	67671	.	G	A	100	*	TYPE=SUBSTITUTE
CM032588.1	67745	.	T	C	100	*	TYPE=SUBSTITUTE
CM032588.1	67780	.	G	A	100	*	TYPE=SUBSTITUTE
CM032588.1	67920	.	G	A	100	*	TYPE=SUBSTITUTE
CM032588.1	67939	.	A	G	100	*	TYPE=SUBSTITUTE
CM032588.1	67952	.	G	C	100	*	TYPE=SUBSTITUTE
CM032588.1	67958	.	G	A	100	*	TYPE=SUBSTITUTE
CM032588.1	67966	.	T	C	100	*	TYPE=SUBSTITUTE
CM032588.1	67981	.	C	G	100	*	TYPE=SUBSTITUTE
CM032588.1	67987	.	C	G	100	*	TYPE=SUBSTITUTE
CM032588.1	68043	.	C	T	100	*	TYPE=SUBSTITUTE
CM032588.1	68055	.	T	C	100	*	TYPE=SUBSTITUTE
CM032588.1	68067	.	T	c	100	*	TYPE=SUBSTITUTE

CM032588.1	68074	.	T	c	100	*	TYPE=SUBSTITUTE
CM032588.1	68094	.	A	G	100	*	TYPE=SUBSTITUTE
CM032588.1	68100	.	T	C	100	*	TYPE=SUBSTITUTE
CM032588.1	68110	.	T	C	100	*	TYPE=SUBSTITUTE
CM032588.1	68170	.	T	c	100	*	TYPE=SUBSTITUTE
CM032588.1	68225	.	G	A	100	*	TYPE=SUBSTITUTE
CM032588.1	68227	.	C	G	100	*	TYPE=SUBSTITUTE
CM032588.1	68278	.	T	G	100	*	TYPE=SUBSTITUTE
CM032588.1	68310	.	G	A	100	*	TYPE=SUBSTITUTE
CM032588.1	68319	.	C	T	100	*	TYPE=SUBSTITUTE
CM032588.1	68332	.	A	G	100	*	TYPE=SUBSTITUTE
CM032588.1	68340	.	G	C	100	*	TYPE=SUBSTITUTE
CM032588.1	68341	.	T	A	100	*	TYPE=SUBSTITUTE
CM032588.1	68353	.	T	C	100	*	TYPE=SUBSTITUTE
CM032588.1	68373	.	T	C	100	*	TYPE=SUBSTITUTE
CM032588.1	68384	.	T	A	100	*	TYPE=SUBSTITUTE
CM032588.1	68385	.	C	T	100	*	TYPE=SUBSTITUTE
CM032588.1	68410	.	T	A	100	*	TYPE=SUBSTITUTE
CM032588.1	68422	.	C	T	100	*	TYPE=SUBSTITUTE
CM032588.1	68432	.	G	T	100	*	TYPE=SUBSTITUTE
CM032588.1	68450	.	C	T	100	*	TYPE=SUBSTITUTE
CM032588.1	68469	.	A	G	100	*	TYPE=SUBSTITUTE
CM032588.1	68540	.	T	c	100	*	TYPE=SUBSTITUTE
CM032588.1	68575	.	C	T	100	*	TYPE=SUBSTITUTE
CM032588.1	68596	.	G	T	100	*	TYPE=SUBSTITUTE
CM032588.1	68613	.	C	T	100	*	TYPE=SUBSTITUTE
CM032588.1	68642	.	A	G	100	*	TYPE=SUBSTITUTE
CM032588.1	68643	.	A	G	100	*	TYPE=SUBSTITUTE
CM032588.1	68656	.	C	A	100	*	TYPE=SUBSTITUTE
CM032588.1	68657	.	A	C	100	*	TYPE=SUBSTITUTE
CM032588.1	68665	.	G	A	100	*	TYPE=SUBSTITUTE
CM032588.1	68692	.	A	G	100	*	TYPE=SUBSTITUTE
CM032588.1	68694	.	T	C	100	*	TYPE=SUBSTITUTE
CM032588.1	68704	.	G	T	100	*	TYPE=SUBSTITUTE
CM032588.1	68710	.	A	T	100	*	TYPE=SUBSTITUTE
CM032588.1	68716	.	T	C	100	*	TYPE=SUBSTITUTE
CM032588.1	68791	.	C	T	100	*	TYPE=SUBSTITUTE
CM032588.1	68827	.	C	T	100	*	TYPE=SUBSTITUTE
CM032588.1	68878	.	G	A	100	*	TYPE=SUBSTITUTE
CM032588.1	68881	.	A	G	100	*	TYPE=SUBSTITUTE
CM032588.1	68914	.	A	G	100	*	TYPE=SUBSTITUTE
CM032588.1	68932	.	A	G	100	*	TYPE=SUBSTITUTE
CM032588.1	68965	.	G	A	100	*	TYPE=SUBSTITUTE
CM032588.1	68995	.	G	C	100	*	TYPE=SUBSTITUTE
CM032588.1	69005	.	T	C	100	*	TYPE=SUBSTITUTE
CM032588.1	69007	.	G	T	100	*	TYPE=SUBSTITUTE
CM032588.1	69016	.	G	A	100	*	TYPE=SUBSTITUTE
CM032588.1	69027	.	C	T	100	*	TYPE=SUBSTITUTE
CM032588.1	69028	.	G	A	100	*	TYPE=SUBSTITUTE
CM032588.1	69052	.	A	g	100	*	TYPE=SUBSTITUTE
CM032588.1	69109	.	G	T	100	*	TYPE=SUBSTITUTE
CM032588.1	69169	.	G	A	100	*	TYPE=SUBSTITUTE
CM032588.1	69212	.	G	c	100	*	TYPE=SUBSTITUTE
CM032588.1	69294	.	C	G	100	*	TYPE=SUBSTITUTE
CM032588.1	69325	.	G	T	100	*	TYPE=SUBSTITUTE
CM032588.1	69338	.	G	A	100	*	TYPE=SUBSTITUTE
CM032588.1	69411	.	T	C	100	*	TYPE=SUBSTITUTE
CM032588.1	69433	.	A	G	100	*	TYPE=SUBSTITUTE
CM032588.1	69460	.	C	T	100	*	TYPE=SUBSTITUTE
CM032588.1	69465	.	T	A	100	*	TYPE=SUBSTITUTE
CM032588.1	69469	.	T	C	100	*	TYPE=SUBSTITUTE
CM032588.1	69471	.	A	G	100	*	TYPE=SUBSTITUTE
CM032588.1	69472	.	T	C	100	*	TYPE=SUBSTITUTE
CM032588.1	69475	.	T	C	100	*	TYPE=SUBSTITUTE

CM032588.1	69491	.	C	A	100	*	TYPE=SUBSTITUTE
CM032588.1	69492	.	T	A	100	*	TYPE=SUBSTITUTE
CM032588.1	69494	.	C	T	100	*	TYPE=SUBSTITUTE
CM032588.1	69495	.	C	A	100	*	TYPE=SUBSTITUTE
CM032588.1	69496	.	C	T	100	*	TYPE=SUBSTITUTE
CM032588.1	69498	.	T	C	100	*	TYPE=SUBSTITUTE
CM032588.1	69501	.	G	C	100	*	TYPE=SUBSTITUTE
CM032588.1	69502	.	A	T	100	*	TYPE=SUBSTITUTE
CM032588.1	69503	.	A	G	100	*	TYPE=SUBSTITUTE
CM032588.1	69519	.	C	T	100	*	TYPE=SUBSTITUTE
CM032588.1	69520	.	T	C	100	*	TYPE=SUBSTITUTE
CM032588.1	69531	.	C	G	100	*	TYPE=SUBSTITUTE
CM032588.1	69532	.	G	A	100	*	TYPE=SUBSTITUTE
CM032588.1	69535	.	T	C	100	*	TYPE=SUBSTITUTE
CM032588.1	69544	.	C	T	100	*	TYPE=SUBSTITUTE
CM032588.1	69553	.	T	C	100	*	TYPE=SUBSTITUTE
CM032588.1	69592	.	T	C	100	*	TYPE=SUBSTITUTE
CM032588.1	69613	.	C	t	100	*	TYPE=SUBSTITUTE
CM032588.1	69620	.	C	T	100	*	TYPE=SUBSTITUTE
CM032588.1	69622	.	T	C	100	*	TYPE=SUBSTITUTE
CM032588.1	69634	.	C	G	100	*	TYPE=SUBSTITUTE
CM032588.1	69701	.	C	G	100	*	TYPE=SUBSTITUTE
CM032588.1	69730	.	T	C	100	*	TYPE=SUBSTITUTE
CM032588.1	69736	.	C	A	100	*	TYPE=SUBSTITUTE
CM032588.1	69748	.	G	A	100	*	TYPE=SUBSTITUTE
CM032588.1	69753	.	G	A	100	*	TYPE=SUBSTITUTE
CM032588.1	69763	.	A	G	100	*	TYPE=SUBSTITUTE
CM032588.1	69769	.	C	T	100	*	TYPE=SUBSTITUTE
CM032588.1	69806	.	C	G	100	*	TYPE=SUBSTITUTE
CM032588.1	69808	.	A	G	100	*	TYPE=SUBSTITUTE
CM032588.1	69843	.	C	A	100	*	TYPE=SUBSTITUTE
CM032588.1	69856	.	A	G	100	*	TYPE=SUBSTITUTE
CM032588.1	69919	.	A	C	100	*	TYPE=SUBSTITUTE
CM032588.1	69946	.	T	C	100	*	TYPE=SUBSTITUTE
CM032588.1	69952	.	A	C	100	*	TYPE=SUBSTITUTE
CM032588.1	70027	.	T	C	100	*	TYPE=SUBSTITUTE
CM032588.1	70031	.	G	A	100	*	TYPE=SUBSTITUTE
CM032588.1	70042	.	G	A	100	*	TYPE=SUBSTITUTE
CM032588.1	70075	.	C	T	100	*	TYPE=SUBSTITUTE
CM032588.1	70078	.	G	A	100	*	TYPE=SUBSTITUTE
CM032588.1	70119	.	A	G	100	*	TYPE=SUBSTITUTE
CM032588.1	70120	.	G	C	100	*	TYPE=SUBSTITUTE
CM032588.1	70130	.	C	T	100	*	TYPE=SUBSTITUTE
CM032588.1	70174	.	C	T	100	*	TYPE=SUBSTITUTE
CM032588.1	70183	.	T	C	100	*	TYPE=SUBSTITUTE
CM032588.1	70190	.	A	G	100	*	TYPE=SUBSTITUTE
CM032588.1	70199	.	G	A	100	*	TYPE=SUBSTITUTE
CM032588.1	70204	.	T	C	100	*	TYPE=SUBSTITUTE
CM032588.1	70205	.	A	G	100	*	TYPE=SUBSTITUTE
CM032588.1	70237	.	C	T	100	*	TYPE=SUBSTITUTE
CM032588.1	70258	.	G	A	100	*	TYPE=SUBSTITUTE
CM032588.1	70264	.	T	G	100	*	TYPE=SUBSTITUTE
CM032588.1	70265	.	G	A	100	*	TYPE=SUBSTITUTE
CM032588.1	70308	.	A	G	100	*	TYPE=SUBSTITUTE
CM032588.1	70326	.	A	G	100	*	TYPE=SUBSTITUTE
CM032588.1	70336	.	T	C	100	*	TYPE=SUBSTITUTE
CM032588.1	70337	.	C	T	100	*	TYPE=SUBSTITUTE
CM032588.1	70339	.	C	T	100	*	TYPE=SUBSTITUTE
CM032588.1	70345	.	C	T	100	*	TYPE=SUBSTITUTE
CM032588.1	70350	.	T	C	100	*	TYPE=SUBSTITUTE
CM032588.1	70390	.	C	T	100	*	TYPE=SUBSTITUTE
CM032588.1	70409	.	A	G	100	*	TYPE=SUBSTITUTE
CM032588.1	70411	.	C	G	100	*	TYPE=SUBSTITUTE
CM032588.1	70417	.	T	C	100	*	TYPE=SUBSTITUTE

CM032588.1	70457	.	A	G	100	*	TYPE=SUBSTITUTE
CM032588.1	70459	.	T	C	100	*	TYPE=SUBSTITUTE
CM032588.1	70468	.	T	G	100	*	TYPE=SUBSTITUTE
CM032588.1	70471	.	T	C	100	*	TYPE=SUBSTITUTE
CM032588.1	70483	.	C	T	100	*	TYPE=SUBSTITUTE
CM032588.1	70493	.	A	G	100	*	TYPE=SUBSTITUTE
CM032588.1	70507	.	T	C	100	*	TYPE=SUBSTITUTE
CM032588.1	70528	.	G	A	100	*	TYPE=SUBSTITUTE
CM032588.1	70550	.	T	C	100	*	TYPE=SUBSTITUTE
CM032588.1	70556	.	C	A	100	*	TYPE=SUBSTITUTE
CM032588.1	70582	.	T	C	100	*	TYPE=SUBSTITUTE
CM032588.1	70700	.	C	CC	100	*	TYPE=INSERT
CM032588.1	70701	.	T	A	100	*	TYPE=SUBSTITUTE
CM032588.1	70705	.	A	G	100	*	TYPE=SUBSTITUTE
CM032588.1	70717	.	A	G	100	*	TYPE=SUBSTITUTE
CM032588.1	70721	.	T	C	100	*	TYPE=SUBSTITUTE
CM032588.1	70722	.	C	CTAA	100	*	TYPE=INSERT
CM032588.1	70742	.	C	T	100	*	TYPE=SUBSTITUTE
CM032588.1	70759	.	AA	A	100	*	TYPE=DELETE
CM032588.1	70761	.	T	C	100	*	TYPE=SUBSTITUTE
CM032588.1	70777	.	G	a	100	*	TYPE=SUBSTITUTE
CM032588.1	70788	.	G	a	100	*	TYPE=SUBSTITUTE
CM032588.1	70793	.	G	T	100	*	TYPE=SUBSTITUTE
CM032588.1	70801	.	T	C	100	*	TYPE=SUBSTITUTE
CM032588.1	70810	.	C	T	100	*	TYPE=SUBSTITUTE
CM032588.1	70811	.	A	G	100	*	TYPE=SUBSTITUTE
CM032588.1	70825	.	G	T	100	*	TYPE=SUBSTITUTE
CM032588.1	70837	.	A	G	100	*	TYPE=SUBSTITUTE
CM032588.1	70839	.	C	T	100	*	TYPE=SUBSTITUTE
CM032588.1	70840	.	C	T	100	*	TYPE=SUBSTITUTE
CM032588.1	70844	.	C	A	100	*	TYPE=SUBSTITUTE
CM032588.1	70849	.	G	A	100	*	TYPE=SUBSTITUTE
CM032588.1	70850	.	T	C	100	*	TYPE=SUBSTITUTE
CM032588.1	70852	.	T	A	100	*	TYPE=SUBSTITUTE
CM032588.1	70860	.	T	C	100	*	TYPE=SUBSTITUTE
CM032588.1	70864	.	C	G	100	*	TYPE=SUBSTITUTE
CM032588.1	70866	.	A	C	100	*	TYPE=SUBSTITUTE
CM032588.1	70867	.	G	A	100	*	TYPE=SUBSTITUTE
CM032588.1	70871	.	G	A	100	*	TYPE=SUBSTITUTE
CM032588.1	70876	.	G	A	100	*	TYPE=SUBSTITUTE
CM032588.1	70887	.	G	T	100	*	TYPE=SUBSTITUTE
CM032588.1	70892	.	A	C	100	*	TYPE=SUBSTITUTE
CM032588.1	70899	.	A	G	100	*	TYPE=SUBSTITUTE
CM032588.1	70902	.	A	G	100	*	TYPE=SUBSTITUTE
CM032588.1	70903	.	C	G	100	*	TYPE=SUBSTITUTE
CM032588.1	70910	.	T	A	100	*	TYPE=SUBSTITUTE
CM032588.1	70913	.	T	G	100	*	TYPE=SUBSTITUTE
CM032588.1	70924	.	C	T	100	*	TYPE=SUBSTITUTE
CM032588.1	70925	.	G	C	100	*	TYPE=SUBSTITUTE
CM032588.1	70927	.	C	A	100	*	TYPE=SUBSTITUTE
CM032588.1	70935	.	T	A	100	*	TYPE=SUBSTITUTE
CM032588.1	70936	.	T	C	100	*	TYPE=SUBSTITUTE
CM032588.1	70947	.	A	G	100	*	TYPE=SUBSTITUTE
CM032588.1	70950	.	G	A	100	*	TYPE=SUBSTITUTE
CM032588.1	70955	.	G	A	100	*	TYPE=SUBSTITUTE
CM032588.1	70958	.	G	C	100	*	TYPE=SUBSTITUTE
CM032588.1	70959	.	G	A	100	*	TYPE=SUBSTITUTE
CM032588.1	70962	.	A	G	100	*	TYPE=SUBSTITUTE
CM032588.1	70972	.	T	C	100	*	TYPE=SUBSTITUTE
CM032588.1	70982	.	C	T	100	*	TYPE=SUBSTITUTE
CM032588.1	70997	.	T	C	100	*	TYPE=SUBSTITUTE
CM032588.1	70998	.	C	T	100	*	TYPE=SUBSTITUTE
CM032588.1	71006	.	A	G	100	*	TYPE=SUBSTITUTE
CM032588.1	71012	.	C	T	100	*	TYPE=SUBSTITUTE

CM032588.1	71014	.	C	T	100	*	TYPE=SUBSTITUTE
CM032588.1	71017	.	C	T	100	*	TYPE=SUBSTITUTE
CM032588.1	71022	.	A	C	100	*	TYPE=SUBSTITUTE
CM032588.1	71044	.	C	A	100	*	TYPE=SUBSTITUTE
CM032588.1	71050	.	A	G	100	*	TYPE=SUBSTITUTE
CM032588.1	71056	.	G	T	100	*	TYPE=SUBSTITUTE
CM032588.1	71062	.	G	A	100	*	TYPE=SUBSTITUTE
CM032588.1	71064	.	G	T	100	*	TYPE=SUBSTITUTE
CM032588.1	71065	.	A	G	100	*	TYPE=SUBSTITUTE
CM032588.1	71068	.	G	T	100	*	TYPE=SUBSTITUTE
CM032588.1	71072	.	G	A	100	*	TYPE=SUBSTITUTE
CM032588.1	71074	.	T	C	100	*	TYPE=SUBSTITUTE
CM032588.1	71080	.	A	G	100	*	TYPE=SUBSTITUTE
CM032588.1	71083	.	T	C	100	*	TYPE=SUBSTITUTE
CM032588.1	71087	.	A	G	100	*	TYPE=SUBSTITUTE
CM032588.1	71102	.	G	T	100	*	TYPE=SUBSTITUTE
CM032588.1	71107	.	C	T	100	*	TYPE=SUBSTITUTE
CM032588.1	71126	.	G	T	100	*	TYPE=SUBSTITUTE
CM032588.1	71133	.	A	G	100	*	TYPE=SUBSTITUTE
CM032588.1	71138	.	T	C	100	*	TYPE=SUBSTITUTE
CM032588.1	71141	.	A	G	100	*	TYPE=SUBSTITUTE
CM032588.1	71150	.	G	A	100	*	TYPE=SUBSTITUTE
CM032588.1	71157	.	T	A	100	*	TYPE=SUBSTITUTE
CM032588.1	71162	.	T	G	100	*	TYPE=SUBSTITUTE
CM032588.1	71168	.	T	A	100	*	TYPE=SUBSTITUTE
CM032588.1	71173	.	A	G	100	*	TYPE=SUBSTITUTE
CM032588.1	71179	.	G	C	100	*	TYPE=SUBSTITUTE
CM032588.1	71182	.	TC	T	100	*	TYPE=DELETE
CM032588.1	71192	.	G	A	100	*	TYPE=SUBSTITUTE
CM032588.1	71194	.	A	T	100	*	TYPE=SUBSTITUTE
CM032588.1	71195	.	C	G	100	*	TYPE=SUBSTITUTE
CM032588.1	71209	.	A	G	100	*	TYPE=SUBSTITUTE
CM032588.1	71211	.	A	G	100	*	TYPE=SUBSTITUTE
CM032588.1	71214	.	C	T	100	*	TYPE=SUBSTITUTE
CM032588.1	71224	.	T	C	100	*	TYPE=SUBSTITUTE
CM032588.1	71240	.	C	A	100	*	TYPE=SUBSTITUTE
CM032588.1	71247	.	C	T	100	*	TYPE=SUBSTITUTE
CM032588.1	71253	.	G	C	100	*	TYPE=SUBSTITUTE
CM032588.1	71254	.	G	T	100	*	TYPE=SUBSTITUTE
CM032588.1	71270	.	G	A	100	*	TYPE=SUBSTITUTE
CM032588.1	71281	.	C	CTCTA	100	*	TYPE=INSERT
CM032588.1	71285	.	T	A	100	*	TYPE=SUBSTITUTE
CM032588.1	71305	.	T	C	100	*	TYPE=SUBSTITUTE
CM032588.1	71306	.	C	T	100	*	TYPE=SUBSTITUTE
CM032588.1	71310	.	G	A	100	*	TYPE=SUBSTITUTE
CM032588.1	71312	.	T	C	100	*	TYPE=SUBSTITUTE
CM032588.1	71318	.	T	C	100	*	TYPE=SUBSTITUTE
CM032588.1	71322	.	CA	C	100	*	TYPE=DELETE
CM032588.1	71327	.	T	C	100	*	TYPE=SUBSTITUTE
CM032588.1	71330	.	G	T	100	*	TYPE=SUBSTITUTE
CM032588.1	71338	.	A	T	100	*	TYPE=SUBSTITUTE
CM032588.1	71351	.	C	T	100	*	TYPE=SUBSTITUTE
CM032588.1	71352	.	T	C	100	*	TYPE=SUBSTITUTE
CM032588.1	71356	.	C	A	100	*	TYPE=SUBSTITUTE
CM032588.1	71357	.	A	G	100	*	TYPE=SUBSTITUTE
CM032588.1	71372	.	T	C	100	*	TYPE=SUBSTITUTE
CM032588.1	71375	.	C	T	100	*	TYPE=SUBSTITUTE
CM032588.1	71380	.	A	C	100	*	TYPE=SUBSTITUTE
CM032588.1	71411	.	C	T	100	*	TYPE=SUBSTITUTE
CM032588.1	71415	.	G	A	100	*	TYPE=SUBSTITUTE
CM032588.1	71417	.	A	G	100	*	TYPE=SUBSTITUTE
CM032588.1	71429	.	C	T	100	*	TYPE=SUBSTITUTE
CM032588.1	71433	.	G	T	100	*	TYPE=SUBSTITUTE
CM032588.1	71436	.	A	T	100	*	TYPE=SUBSTITUTE

CM032588.1	71443	.	G	A	100	*	TYPE=SUBSTITUTE
CM032588.1	71448	.	T	C	100	*	TYPE=SUBSTITUTE
CM032588.1	71454	.	C	T	100	*	TYPE=SUBSTITUTE
CM032588.1	71458	.	G	T	100	*	TYPE=SUBSTITUTE
CM032588.1	71462	.	C	T	100	*	TYPE=SUBSTITUTE
CM032588.1	71478	.	A	g	100	*	TYPE=SUBSTITUTE
CM032588.1	71480	.	g	ga	100	*	TYPE=INSERT
CM032588.1	71482	.	C	g	100	*	TYPE=SUBSTITUTE
CM032588.1	71493	.	T	G	100	*	TYPE=SUBSTITUTE
CM032588.1	71497	.	A	G	100	*	TYPE=SUBSTITUTE
CM032588.1	71515	.	A	G	100	*	TYPE=SUBSTITUTE
CM032588.1	71525	.	T	C	100	*	TYPE=SUBSTITUTE
CM032588.1	71529	.	A	G	100	*	TYPE=SUBSTITUTE
CM032588.1	71536	.	T	C	100	*	TYPE=SUBSTITUTE
CM032588.1	71539	.	G	C	100	*	TYPE=SUBSTITUTE
CM032588.1	71546	.	C	T	100	*	TYPE=SUBSTITUTE
CM032588.1	71592	.	T	G	100	*	TYPE=SUBSTITUTE
CM032588.1	71611	.	A	T	100	*	TYPE=SUBSTITUTE
CM032588.1	71615	.	G	A	100	*	TYPE=SUBSTITUTE
CM032588.1	71652	.	T	C	100	*	TYPE=SUBSTITUTE
CM032588.1	71665	.	T	C	100	*	TYPE=SUBSTITUTE
CM032588.1	71670	.	A	G	100	*	TYPE=SUBSTITUTE
CM032588.1	71673	.	G	A	100	*	TYPE=SUBSTITUTE
CM032588.1	71705	.	A	G	100	*	TYPE=SUBSTITUTE
CM032588.1	71767	.	T	C	100	*	TYPE=SUBSTITUTE
CM032588.1	71772	.	A	T	100	*	TYPE=SUBSTITUTE
CM032588.1	71785	.	T	C	100	*	TYPE=SUBSTITUTE
CM032588.1	71793	.	C	G	100	*	TYPE=SUBSTITUTE
CM032588.1	71823	.	C	A	100	*	TYPE=SUBSTITUTE
CM032588.1	71852	.	G	A	100	*	TYPE=SUBSTITUTE
CM032588.1	71874	.	A	G	100	*	TYPE=SUBSTITUTE
CM032588.1	71888	.	T	a	100	*	TYPE=SUBSTITUTE
CM032588.1	71922	.	T	C	100	*	TYPE=SUBSTITUTE
CM032588.1	71946	.	C	T	100	*	TYPE=SUBSTITUTE
CM032588.1	71959	.	G	C	100	*	TYPE=SUBSTITUTE
CM032588.1	72000	.	T	C	100	*	TYPE=SUBSTITUTE
CM032588.1	72059	.	A	G	100	*	TYPE=SUBSTITUTE
CM032588.1	72066	.	A	G	100	*	TYPE=SUBSTITUTE
CM032588.1	72075	.	C	G	100	*	TYPE=SUBSTITUTE
CM032588.1	72102	.	G	A	100	*	TYPE=SUBSTITUTE
CM032588.1	72106	.	G	C	100	*	TYPE=SUBSTITUTE
CM032588.1	72128	.	G	C	100	*	TYPE=SUBSTITUTE
CM032588.1	72136	.	C	G	100	*	TYPE=SUBSTITUTE
CM032588.1	72150	.	G	T	100	*	TYPE=SUBSTITUTE
CM032588.1	72168	.	G	C	100	*	TYPE=SUBSTITUTE
CM032588.1	72197	.	A	G	100	*	TYPE=SUBSTITUTE
CM032588.1	72216	.	G	A	100	*	TYPE=SUBSTITUTE
CM032588.1	72219	.	G	A	100	*	TYPE=SUBSTITUTE
CM032588.1	72252	.	C	T	100	*	TYPE=SUBSTITUTE
CM032588.1	72266	.	A	G	100	*	TYPE=SUBSTITUTE
CM032588.1	72267	.	T	C	100	*	TYPE=SUBSTITUTE
CM032588.1	72340	.	T	A	100	*	TYPE=SUBSTITUTE
CM032588.1	72363	.	C	A	100	*	TYPE=SUBSTITUTE
CM032588.1	72372	.	G	A	100	*	TYPE=SUBSTITUTE
CM032588.1	72390	.	A	G	100	*	TYPE=SUBSTITUTE
CM032588.1	72396	.	C	T	100	*	TYPE=SUBSTITUTE
CM032588.1	72404	.	C	A	100	*	TYPE=SUBSTITUTE
CM032588.1	72413	.	C	G	100	*	TYPE=SUBSTITUTE
CM032588.1	72417	.	C	T	100	*	TYPE=SUBSTITUTE
CM032588.1	72432	.	C	A	100	*	TYPE=SUBSTITUTE
CM032588.1	72455	.	C	A	100	*	TYPE=SUBSTITUTE
CM032588.1	72460	.	T	G	100	*	TYPE=SUBSTITUTE
CM032588.1	72462	.	G	A	100	*	TYPE=SUBSTITUTE
CM032588.1	72490	.	C	T	100	*	TYPE=SUBSTITUTE

CM032588.1	72493	.	C	G	100	*	TYPE=SUBSTITUTE
CM032588.1	72502	.	G	C	100	*	TYPE=SUBSTITUTE
CM032588.1	72530	.	A	G	100	*	TYPE=SUBSTITUTE
CM032588.1	72555	.	A	G	100	*	TYPE=SUBSTITUTE
CM032588.1	72573	.	A	C	100	*	TYPE=SUBSTITUTE
CM032588.1	72578	.	T	A	100	*	TYPE=SUBSTITUTE
CM032588.1	72615	.	G	C	100	*	TYPE=SUBSTITUTE
CM032588.1	72624	.	A	G	100	*	TYPE=SUBSTITUTE
CM032588.1	72629	.	T	C	100	*	TYPE=SUBSTITUTE
CM032588.1	72635	.	A	G	100	*	TYPE=SUBSTITUTE
CM032588.1	72676	.	T	A	100	*	TYPE=SUBSTITUTE
CM032588.1	72678	.	T	G	100	*	TYPE=SUBSTITUTE
CM032588.1	72680	.	C	T	100	*	TYPE=SUBSTITUTE
CM032588.1	72684	.	A	G	100	*	TYPE=SUBSTITUTE
CM032588.1	72699	.	A	C	100	*	TYPE=SUBSTITUTE
CM032588.1	72708	.	G	A	100	*	TYPE=SUBSTITUTE
CM032588.1	72721	.	G	T	100	*	TYPE=SUBSTITUTE
CM032588.1	72722	.	T	C	100	*	TYPE=SUBSTITUTE
CM032588.1	72726	.	C	T	100	*	TYPE=SUBSTITUTE
CM032588.1	72735	.	G	A	100	*	TYPE=SUBSTITUTE
CM032588.1	72737	.	C	T	100	*	TYPE=SUBSTITUTE
CM032588.1	72743	.	T	A	100	*	TYPE=SUBSTITUTE
CM032588.1	72795	.	C	T	100	*	TYPE=SUBSTITUTE
CM032588.1	72810	.	C	G	100	*	TYPE=SUBSTITUTE
CM032588.1	72825	.	C	T	100	*	TYPE=SUBSTITUTE
CM032588.1	72843	.	A	G	100	*	TYPE=SUBSTITUTE
CM032588.1	72856	.	C	G	100	*	TYPE=SUBSTITUTE
CM032588.1	72885	.	A	G	100	*	TYPE=SUBSTITUTE
CM032588.1	72891	.	G	A	100	*	TYPE=SUBSTITUTE
CM032588.1	72916	.	G	T	100	*	TYPE=SUBSTITUTE
CM032588.1	72930	.	A	G	100	*	TYPE=SUBSTITUTE
CM032588.1	72942	.	T	C	100	*	TYPE=SUBSTITUTE
CM032588.1	72950	.	G	C	100	*	TYPE=SUBSTITUTE
CM032588.1	72983	.	T	A	100	*	TYPE=SUBSTITUTE
CM032588.1	72999	.	A	G	100	*	TYPE=SUBSTITUTE
CM032588.1	73027	.	A	AAAT	100	*	TYPE=INSERT
CM032588.1	73029	.	A	AG	100	*	TYPE=INSERT
CM032588.1	73033	.	G	A	100	*	TYPE=SUBSTITUTE
CM032588.1	73035	.	C	T	100	*	TYPE=SUBSTITUTE
CM032588.1	73045	.	T	C	100	*	TYPE=SUBSTITUTE
CM032588.1	73056	.	A	G	100	*	TYPE=SUBSTITUTE
CM032588.1	73063	.	G	T	100	*	TYPE=SUBSTITUTE
CM032588.1	73065	.	T	A	100	*	TYPE=SUBSTITUTE
CM032588.1	73068	.	C	T	100	*	TYPE=SUBSTITUTE
CM032588.1	73071	.	C	T	100	*	TYPE=SUBSTITUTE
CM032588.1	73078	.	A	G	100	*	TYPE=SUBSTITUTE
CM032588.1	73080	.	A	G	100	*	TYPE=SUBSTITUTE
CM032588.1	73086	.	C	T	100	*	TYPE=SUBSTITUTE
CM032588.1	73089	.	C	T	100	*	TYPE=SUBSTITUTE
CM032588.1	73096	.	C	G	100	*	TYPE=SUBSTITUTE
CM032588.1	73099	.	A	G	100	*	TYPE=SUBSTITUTE
CM032588.1	73108	.	C	T	100	*	TYPE=SUBSTITUTE
CM032588.1	73110	.	A	G	100	*	TYPE=SUBSTITUTE
CM032588.1	73117	.	G	A	100	*	TYPE=SUBSTITUTE
CM032588.1	73121	.	A	C	100	*	TYPE=SUBSTITUTE
CM032588.1	73129	.	A	G	100	*	TYPE=SUBSTITUTE
CM032588.1	73137	.	G	GTAT	100	*	TYPE=INSERT
CM032588.1	73153	.	T	A	100	*	TYPE=SUBSTITUTE
CM032588.1	73160	.	T	C	100	*	TYPE=SUBSTITUTE
CM032588.1	73166	.	CATGCTCCA	C	100	*	TYPE=DELETE
CM032588.1	73176	.	G	T	100	*	TYPE=SUBSTITUTE
CM032588.1	73193	.	T	G	100	*	TYPE=SUBSTITUTE
CM032588.1	73245	.	C	T	100	*	TYPE=SUBSTITUTE
CM032588.1	73246	.	T	C	100	*	TYPE=SUBSTITUTE

CM032588.1	73275	.	C	G	100	*	TYPE=SUBSTITUTE
CM032588.1	73280	.	C	T	100	*	TYPE=SUBSTITUTE
CM032588.1	73301	.	TGCCC	T	100	*	TYPE=DELETE
CM032588.1	73306	.	G	A	100	*	TYPE=SUBSTITUTE
CM032588.1	73326	.	C	T	100	*	TYPE=SUBSTITUTE
CM032588.1	73375	.	C	T	100	*	TYPE=SUBSTITUTE
CM032588.1	73381	.	G	A	100	*	TYPE=SUBSTITUTE
CM032588.1	73386	.	G	A	100	*	TYPE=SUBSTITUTE
CM032588.1	73406	.	G	a	100	*	TYPE=SUBSTITUTE
CM032588.1	73408	.	A	c	100	*	TYPE=SUBSTITUTE
CM032588.1	73417	.	A	g	100	*	TYPE=SUBSTITUTE
CM032588.1	73434	.	A	G	100	*	TYPE=SUBSTITUTE
CM032588.1	73440	.	G	A	100	*	TYPE=SUBSTITUTE
CM032588.1	73449	.	A	G	100	*	TYPE=SUBSTITUTE
CM032588.1	73474	.	G	C	100	*	TYPE=SUBSTITUTE
CM032588.1	73481	.	G	A	100	*	TYPE=SUBSTITUTE
CM032588.1	73506	.	G	C	100	*	TYPE=SUBSTITUTE
CM032588.1	73533	.	A	g	100	*	TYPE=SUBSTITUTE
CM032588.1	73551	.	T	C	100	*	TYPE=SUBSTITUTE
CM032588.1	73557	.	T	TC	100	*	TYPE=INSERT
CM032588.1	73567	.	C	T	100	*	TYPE=SUBSTITUTE
CM032588.1	73570	.	C	T	100	*	TYPE=SUBSTITUTE
CM032588.1	73591	.	T	A	100	*	TYPE=SUBSTITUTE
CM032588.1	73619	.	G	T	100	*	TYPE=SUBSTITUTE
CM032588.1	73620	.	C	G	100	*	TYPE=SUBSTITUTE
CM032588.1	73622	.	A	T	100	*	TYPE=SUBSTITUTE
CM032588.1	73627	.	T	G	100	*	TYPE=SUBSTITUTE
CM032588.1	73664	.	T	C	100	*	TYPE=SUBSTITUTE
CM032588.1	73697	.	A	G	100	*	TYPE=SUBSTITUTE
CM032588.1	73718	.	C	T	100	*	TYPE=SUBSTITUTE
CM032588.1	73730	.	C	T	100	*	TYPE=SUBSTITUTE
CM032588.1	73769	.	G	A	100	*	TYPE=SUBSTITUTE
CM032588.1	73784	.	T	C	100	*	TYPE=SUBSTITUTE
CM032588.1	73889	.	A	G	100	*	TYPE=SUBSTITUTE
CM032588.1	73898	.	G	T	100	*	TYPE=SUBSTITUTE
CM032588.1	73919	.	T	C	100	*	TYPE=SUBSTITUTE
CM032588.1	73964	.	C	T	100	*	TYPE=SUBSTITUTE
CM032588.1	74048	.	T	C	100	*	TYPE=SUBSTITUTE
CM032588.1	74120	.	A	G	100	*	TYPE=SUBSTITUTE
CM032588.1	74147	.	C	T	100	*	TYPE=SUBSTITUTE
CM032588.1	74300	.	C	T	100	*	TYPE=SUBSTITUTE
CM032588.1	74307	.	GA	G	100	*	TYPE=DELETE
CM032588.1	74349	.	T	C	100	*	TYPE=SUBSTITUTE
CM032588.1	74475	.	A	G	100	*	TYPE=SUBSTITUTE
CM032588.1	74511	.	A	G	100	*	TYPE=SUBSTITUTE
CM032588.1	74567	.	G	A	100	*	TYPE=SUBSTITUTE
CM032588.1	74574	.	A	G	100	*	TYPE=SUBSTITUTE
CM032588.1	74616	.	A	G	100	*	TYPE=SUBSTITUTE
CM032588.1	74643	.	A	AA	100	*	TYPE=INSERT
CM032588.1	74656	.	G	a	100	*	TYPE=SUBSTITUTE
CM032588.1	74685	.	AA	A	100	*	TYPE=DELETE
CM032588.1	74726	.	AA	A	100	*	TYPE=DELETE
CM032588.1	74782	.	A	T	100	*	TYPE=SUBSTITUTE
CM032588.1	74814	.	G	A	100	*	TYPE=SUBSTITUTE
CM032588.1	74852	.	C	T	100	*	TYPE=SUBSTITUTE
CM032588.1	74862	.	T	C	100	*	TYPE=SUBSTITUTE
CM032588.1	74872	.	G	A	100	*	TYPE=SUBSTITUTE
CM032588.1	74885	.	C	G	100	*	TYPE=SUBSTITUTE
CM032588.1	74981	.	T	C	100	*	TYPE=SUBSTITUTE
CM032588.1	74983	.	C	T	100	*	TYPE=SUBSTITUTE
CM032588.1	74985	.	A	G	100	*	TYPE=SUBSTITUTE
CM032588.1	75013	.	C	T	100	*	TYPE=SUBSTITUTE
CM032588.1	75044	.	T	C	100	*	TYPE=SUBSTITUTE
CM032588.1	75045	.	G	A	100	*	TYPE=SUBSTITUTE

CM032588.1	75077	.	A	T	100	*	TYPE=SUBSTITUTE
CM032588.1	75086	.	A	G	100	*	TYPE=SUBSTITUTE
CM032588.1	75147	.	A	G	100	*	TYPE=SUBSTITUTE
CM032588.1	75156	.	A	G	100	*	TYPE=SUBSTITUTE
CM032588.1	75168	.	C	T	100	*	TYPE=SUBSTITUTE
CM032588.1	75178	.	T	A	100	*	TYPE=SUBSTITUTE
CM032588.1	75195	.	T	C	100	*	TYPE=SUBSTITUTE
CM032588.1	75216	.	T	C	100	*	TYPE=SUBSTITUTE
CM032588.1	75219	.	C	A	100	*	TYPE=SUBSTITUTE
CM032588.1	75221	.	C	A	100	*	TYPE=SUBSTITUTE
CM032588.1	75242	.	T	TA	100	*	TYPE=INSERT
CM032588.1	75246	.	G	T	100	*	TYPE=SUBSTITUTE
CM032588.1	75265	.	C	G	100	*	TYPE=SUBSTITUTE
CM032588.1	75283	.	C	t	100	*	TYPE=SUBSTITUTE
CM032588.1	75304	.	C	A	100	*	TYPE=SUBSTITUTE
CM032588.1	75307	.	G	C	100	*	TYPE=SUBSTITUTE
CM032588.1	75320	.	G	A	100	*	TYPE=SUBSTITUTE
CM032588.1	75327	.	G	GTA	100	*	TYPE=INSERT
CM032588.1	75355	.	G	A	100	*	TYPE=SUBSTITUTE
CM032588.1	75356	.	G	T	100	*	TYPE=SUBSTITUTE
CM032588.1	75366	.	T	C	100	*	TYPE=SUBSTITUTE
CM032588.1	75405	.	C	CTACAG	100	*	TYPE=INSERT
CM032588.1	75433	.	G	T	100	*	TYPE=SUBSTITUTE
CM032588.1	75472	.	A	G	100	*	TYPE=SUBSTITUTE
CM032588.1	75533	.	A	G	100	*	TYPE=SUBSTITUTE
CM032588.1	75547	.	A	G	100	*	TYPE=SUBSTITUTE
CM032588.1	75560	.	A	G	100	*	TYPE=SUBSTITUTE
CM032588.1	75569	.	G	C	100	*	TYPE=SUBSTITUTE
CM032588.1	75583	.	T	G	100	*	TYPE=SUBSTITUTE
CM032588.1	75611	.	T	C	100	*	TYPE=SUBSTITUTE
CM032588.1	75621	.	C	G	100	*	TYPE=SUBSTITUTE
CM032588.1	75645	.	T	A	100	*	TYPE=SUBSTITUTE
CM032588.1	75653	.	A	G	100	*	TYPE=SUBSTITUTE
CM032588.1	75656	.	C	T	100	*	TYPE=SUBSTITUTE
CM032588.1	75660	.	C	G	100	*	TYPE=SUBSTITUTE
CM032588.1	75670	.	C	T	100	*	TYPE=SUBSTITUTE
CM032588.1	75673	.	G	C	100	*	TYPE=SUBSTITUTE
CM032588.1	75683	.	A	G	100	*	TYPE=SUBSTITUTE
CM032588.1	75706	.	G	A	100	*	TYPE=SUBSTITUTE
CM032588.1	75707	.	CG	C	100	*	TYPE=DELETE
CM032588.1	75710	.	T	A	100	*	TYPE=SUBSTITUTE
CM032588.1	75723	.	A	G	100	*	TYPE=SUBSTITUTE
CM032588.1	75732	.	C	T	100	*	TYPE=SUBSTITUTE
CM032588.1	75736	.	T	C	100	*	TYPE=SUBSTITUTE
CM032588.1	75737	.	C	A	100	*	TYPE=SUBSTITUTE
CM032588.1	75742	.	G	T	100	*	TYPE=SUBSTITUTE
CM032588.1	75743	.	A	C	100	*	TYPE=SUBSTITUTE
CM032588.1	75795	.	A	G	100	*	TYPE=SUBSTITUTE
CM032588.1	75825	.	C	G	100	*	TYPE=SUBSTITUTE
CM032588.1	75842	.	C	A	100	*	TYPE=SUBSTITUTE
CM032588.1	75850	.	G	A	100	*	TYPE=SUBSTITUTE
CM032588.1	75861	.	A	G	100	*	TYPE=SUBSTITUTE
CM032588.1	75890	.	T	G	100	*	TYPE=SUBSTITUTE
CM032588.1	75891	.	G	T	100	*	TYPE=SUBSTITUTE
CM032588.1	75907	.	G	A	100	*	TYPE=SUBSTITUTE
CM032588.1	75909	.	C	G	100	*	TYPE=SUBSTITUTE
CM032588.1	75930	.	T	A	100	*	TYPE=SUBSTITUTE
CM032588.1	75933	.	C	T	100	*	TYPE=SUBSTITUTE
CM032588.1	75940	.	A	C	100	*	TYPE=SUBSTITUTE
CM032588.1	75943	.	C	T	100	*	TYPE=SUBSTITUTE
CM032588.1	75955	.	G	A	100	*	TYPE=SUBSTITUTE
CM032588.1	75957	.	T	C	100	*	TYPE=SUBSTITUTE
CM032588.1	75962	.	C	T	100	*	TYPE=SUBSTITUTE
CM032588.1	75982	.	A	ACAA	100	*	TYPE=INSERT

CM032588.1	75984	.	T	TAATAAC	100	*	TYPE=INSERT
CM032588.1	76002	.	C	T	100	*	TYPE=SUBSTITUTE
CM032588.1	76021	.	G	A	100	*	TYPE=SUBSTITUTE
CM032588.1	76052	.	T	C	100	*	TYPE=SUBSTITUTE
CM032588.1	76070	.	C	A	100	*	TYPE=SUBSTITUTE
CM032588.1	76077	.	A	G	100	*	TYPE=SUBSTITUTE
CM032588.1	76081	.	A	G	100	*	TYPE=SUBSTITUTE
CM032588.1	76099	.	T	C	100	*	TYPE=SUBSTITUTE
CM032588.1	76105	.	T	C	100	*	TYPE=SUBSTITUTE
CM032588.1	76109	.	G	A	100	*	TYPE=SUBSTITUTE
CM032588.1	76110	.	A	G	100	*	TYPE=SUBSTITUTE
CM032588.1	76111	.	A	G	100	*	TYPE=SUBSTITUTE
CM032588.1	76130	.	T	C	100	*	TYPE=SUBSTITUTE
CM032588.1	76153	.	C	T	100	*	TYPE=SUBSTITUTE
CM032588.1	76212	.	G	T	100	*	TYPE=SUBSTITUTE
CM032588.1	76251	.	T	C	100	*	TYPE=SUBSTITUTE
CM032588.1	76281	.	A	G	100	*	TYPE=SUBSTITUTE
CM032588.1	76341	.	T	C	100	*	TYPE=SUBSTITUTE
CM032588.1	76356	.	A	G	100	*	TYPE=SUBSTITUTE
CM032588.1	76475	.	CC	C	100	*	TYPE=DELETE
CM032588.1	76933	.	C	T	100	*	TYPE=SUBSTITUTE
CM032588.1	76987	.	C	A	100	*	TYPE=SUBSTITUTE
CM032588.1	76999	.	A	G	100	*	TYPE=SUBSTITUTE
CM032588.1	77015	.	G	A	100	*	TYPE=SUBSTITUTE
CM032588.1	77083	.	A	G	100	*	TYPE=SUBSTITUTE
CM032588.1	77092	.	G	A	100	*	TYPE=SUBSTITUTE
CM032588.1	77098	.	G	A	100	*	TYPE=SUBSTITUTE
CM032588.1	77106	.	C	T	100	*	TYPE=SUBSTITUTE
CM032588.1	77169	.	A	G	100	*	TYPE=SUBSTITUTE
CM032588.1	77201	.	G	C	100	*	TYPE=SUBSTITUTE
CM032588.1	77207	.	C	T	100	*	TYPE=SUBSTITUTE
CM032588.1	77209	.	A	G	100	*	TYPE=SUBSTITUTE
CM032588.1	77215	.	C	T	100	*	TYPE=SUBSTITUTE
CM032588.1	77238	.	G	A	100	*	TYPE=SUBSTITUTE
CM032588.1	77261	.	T	A	100	*	TYPE=SUBSTITUTE
CM032588.1	77264	.	T	C	100	*	TYPE=SUBSTITUTE
CM032588.1	77266	.	G	A	100	*	TYPE=SUBSTITUTE
CM032588.1	77267	.	A	C	100	*	TYPE=SUBSTITUTE
CM032588.1	77270	.	C	A	100	*	TYPE=SUBSTITUTE
CM032588.1	77271	.	G	C	100	*	TYPE=SUBSTITUTE
CM032588.1	77275	.	T	TGAA	100	*	TYPE=INSERT
CM032588.1	77296	.	GCATGGGTCGTGATCCGT	G	100	*	TYPE=DELETE
CM032588.1	77315	.	T	A	100	*	TYPE=SUBSTITUTE
CM032588.1	77323	.	T	A	100	*	TYPE=SUBSTITUTE
CM032588.1	77331	.	G	a	100	*	TYPE=SUBSTITUTE
CM032588.1	77340	.	C	t	100	*	TYPE=SUBSTITUTE
CM032588.1	77342	.	TC	T	100	*	TYPE=DELETE
CM032588.1	77346	.	A	AG	100	*	TYPE=INSERT
CM032588.1	77350	.	T	A	100	*	TYPE=SUBSTITUTE
CM032588.1	77353	.	C	A	100	*	TYPE=SUBSTITUTE
CM032588.1	77355	.	A	T	100	*	TYPE=SUBSTITUTE
CM032588.1	77366	.	T	C	100	*	TYPE=SUBSTITUTE
CM032588.1	77369	.	G	A	100	*	TYPE=SUBSTITUTE
CM032588.1	77381	.	T	G	100	*	TYPE=SUBSTITUTE
CM032588.1	77388	.	T	C	100	*	TYPE=SUBSTITUTE
CM032588.1	77389	.	G	A	100	*	TYPE=SUBSTITUTE
CM032588.1	77395	.	A	G	100	*	TYPE=SUBSTITUTE
CM032588.1	77402	.	T	G	100	*	TYPE=SUBSTITUTE
CM032588.1	77403	.	G	C	100	*	TYPE=SUBSTITUTE
CM032588.1	77427	.	A	G	100	*	TYPE=SUBSTITUTE
CM032588.1	77431	.	C	A	100	*	TYPE=SUBSTITUTE
CM032588.1	77443	.	A	C	100	*	TYPE=SUBSTITUTE
CM032588.1	77445	.	A	G	100	*	TYPE=SUBSTITUTE
CM032588.1	77464	.	A	G	100	*	TYPE=SUBSTITUTE

CM032588.1	77483	.	A	G	100	*	TYPE=SUBSTITUTE
CM032588.1	77495	.	G	A	100	*	TYPE=SUBSTITUTE
CM032588.1	77500	.	T	G	100	*	TYPE=SUBSTITUTE
CM032588.1	77505	.	G	A	100	*	TYPE=SUBSTITUTE
CM032588.1	77514	.	G	A	100	*	TYPE=SUBSTITUTE
CM032588.1	77518	.	G	A	100	*	TYPE=SUBSTITUTE
CM032588.1	77519	.	T	C	100	*	TYPE=SUBSTITUTE
CM032588.1	77521	.	G	T	100	*	TYPE=SUBSTITUTE
CM032588.1	77529	.	A	G	100	*	TYPE=SUBSTITUTE
CM032588.1	77532	.	GATGCCTAGGTTGGGAACCGCT	G	100	*	TYPE=DELETE
CM032588.1	77558	.	A	T	100	*	TYPE=SUBSTITUTE
CM032588.1	77559	.	T	C	100	*	TYPE=SUBSTITUTE
CM032588.1	77580	.	A	G	100	*	TYPE=SUBSTITUTE
CM032588.1	77596	.	C	T	100	*	TYPE=SUBSTITUTE
CM032588.1	77607	.	G	A	100	*	TYPE=SUBSTITUTE
CM032588.1	77613	.	C	A	100	*	TYPE=SUBSTITUTE
CM032588.1	77617	.	C	T	100	*	TYPE=SUBSTITUTE
CM032588.1	77618	.	C	A	100	*	TYPE=SUBSTITUTE
CM032588.1	77626	.	C	CT	100	*	TYPE=INSERT
CM032588.1	77632	.	C	T	100	*	TYPE=SUBSTITUTE
CM032588.1	77645	.	A	G	100	*	TYPE=SUBSTITUTE
CM032588.1	77652	.	A	G	100	*	TYPE=SUBSTITUTE
CM032588.1	77659	.	T	C	100	*	TYPE=SUBSTITUTE
CM032588.1	77670	.	C	T	100	*	TYPE=SUBSTITUTE
CM032588.1	77694	.	T	C	100	*	TYPE=SUBSTITUTE
CM032588.1	77753	.	G	T	100	*	TYPE=SUBSTITUTE
CM032588.1	77756	.	G	T	100	*	TYPE=SUBSTITUTE
CM032588.1	77774	.	A	C	100	*	TYPE=SUBSTITUTE
CM032588.1	77784	.	G	T	100	*	TYPE=SUBSTITUTE
CM032588.1	77797	.	G	C	100	*	TYPE=SUBSTITUTE
CM032588.1	77799	.	G	C	100	*	TYPE=SUBSTITUTE
CM032588.1	77870	.	A	T	100	*	TYPE=SUBSTITUTE
CM032588.1	77909	.	G	T	100	*	TYPE=SUBSTITUTE
CM032588.1	77922	.	A	T	100	*	TYPE=SUBSTITUTE
CM032588.1	77949	.	G	GCG	100	*	TYPE=INSERT
CM032588.1	77988	.	A	C	100	*	TYPE=SUBSTITUTE
CM032588.1	78004	.	A	G	100	*	TYPE=SUBSTITUTE
CM032588.1	78009	.	C	T	100	*	TYPE=SUBSTITUTE
CM032588.1	78011	.	AC	A	100	*	TYPE=DELETE
CM032588.1	78013	.	A	G	100	*	TYPE=SUBSTITUTE
CM032588.1	78015	.	A	C	100	*	TYPE=SUBSTITUTE
CM032588.1	78022	.	G	A	100	*	TYPE=SUBSTITUTE
CM032588.1	78027	.	C	T	100	*	TYPE=SUBSTITUTE
CM032588.1	78035	.	A	G	100	*	TYPE=SUBSTITUTE
CM032588.1	78037	.	C	T	100	*	TYPE=SUBSTITUTE
CM032588.1	78057	.	G	A	100	*	TYPE=SUBSTITUTE
CM032588.1	78067	.	C	T	100	*	TYPE=SUBSTITUTE
CM032588.1	78069	.	C	T	100	*	TYPE=SUBSTITUTE
CM032588.1	78096	.	C	T	100	*	TYPE=SUBSTITUTE
CM032588.1	78100	.	C	A	100	*	TYPE=SUBSTITUTE
CM032588.1	78108	.	A	G	100	*	TYPE=SUBSTITUTE
CM032588.1	78123	.	A	AGATGGTA	100	*	TYPE=INSERT
CM032588.1	78124	.	A	G	100	*	TYPE=SUBSTITUTE
CM032588.1	78128	.	T	C	100	*	TYPE=SUBSTITUTE
CM032588.1	78157	.	C	T	100	*	TYPE=SUBSTITUTE
CM032588.1	78180	.	C	G	100	*	TYPE=SUBSTITUTE
CM032588.1	78183	.	C	G	100	*	TYPE=SUBSTITUTE
CM032588.1	78206	.	A	G	100	*	TYPE=SUBSTITUTE
CM032588.1	78214	.	C	T	100	*	TYPE=SUBSTITUTE
CM032588.1	78223	.	T	C	100	*	TYPE=SUBSTITUTE
CM032588.1	78238	.	G	T	100	*	TYPE=SUBSTITUTE
CM032588.1	78257	.	C	T	100	*	TYPE=SUBSTITUTE
CM032588.1	78273	.	T	G	100	*	TYPE=SUBSTITUTE
CM032588.1	78274	.	C	t	100	*	TYPE=SUBSTITUTE

CM032588.1	78303	.	C	T	100	*	TYPE=SUBSTITUTE
CM032588.1	78304	.	T	C	100	*	TYPE=SUBSTITUTE
CM032588.1	78320	.	G	A	100	*	TYPE=SUBSTITUTE
CM032588.1	78328	.	C	T	100	*	TYPE=SUBSTITUTE
CM032588.1	78333	.	T	C	100	*	TYPE=SUBSTITUTE
CM032588.1	78340	.	A	C	100	*	TYPE=SUBSTITUTE
CM032588.1	78342	.	C	CTGTGT	100	*	TYPE=INSERT
CM032588.1	78355	.	T	C	100	*	TYPE=SUBSTITUTE
CM032588.1	78361	.	A	G	100	*	TYPE=SUBSTITUTE
CM032588.1	78373	.	G	C	100	*	TYPE=SUBSTITUTE
CM032588.1	78386	.	T	C	100	*	TYPE=SUBSTITUTE
CM032588.1	78413	.	CC	C	100	*	TYPE=DELETE
CM032588.1	78431	.	C	T	100	*	TYPE=SUBSTITUTE
CM032588.1	78457	.	A	G	100	*	TYPE=SUBSTITUTE
CM032588.1	78459	.	A	T	100	*	TYPE=SUBSTITUTE
CM032588.1	78471	.	A	G	100	*	TYPE=SUBSTITUTE
CM032588.1	78481	.	T	C	100	*	TYPE=SUBSTITUTE
CM032588.1	78525	.	G	A	100	*	TYPE=SUBSTITUTE
CM032588.1	78618	.	T	C	100	*	TYPE=SUBSTITUTE
CM032588.1	78699	.	T	A	100	*	TYPE=SUBSTITUTE
CM032588.1	78700	.	C	A	100	*	TYPE=SUBSTITUTE
CM032588.1	78707	.	G	C	100	*	TYPE=SUBSTITUTE
CM032588.1	78709	.	C	T	100	*	TYPE=SUBSTITUTE
CM032588.1	78713	.	G	A	100	*	TYPE=SUBSTITUTE
CM032588.1	78722	.	A	C	100	*	TYPE=SUBSTITUTE
CM032588.1	78725	.	G	A	100	*	TYPE=SUBSTITUTE
CM032588.1	78742	.	T	G	100	*	TYPE=SUBSTITUTE
CM032588.1	78745	.	A	G	100	*	TYPE=SUBSTITUTE
CM032588.1	78749	.	C	T	100	*	TYPE=SUBSTITUTE
CM032588.1	78831	.	T	C	100	*	TYPE=SUBSTITUTE
CM032588.1	78839	.	G	A	100	*	TYPE=SUBSTITUTE
CM032588.1	78902	.	C	A	100	*	TYPE=SUBSTITUTE
CM032588.1	78974	.	G	A	100	*	TYPE=SUBSTITUTE
CM032588.1	79001	.	C	T	100	*	TYPE=SUBSTITUTE
CM032588.1	79026	.	A	G	100	*	TYPE=SUBSTITUTE
CM032588.1	79030	.	G	T	100	*	TYPE=SUBSTITUTE
CM032588.1	79032	.	G	C	100	*	TYPE=SUBSTITUTE
CM032588.1	79036	.	C	T	100	*	TYPE=SUBSTITUTE
CM032588.1	79043	.	G	A	100	*	TYPE=SUBSTITUTE
CM032588.1	79049	.	A	AT	100	*	TYPE=INSERT
CM032588.1	79093	.	T	C	100	*	TYPE=SUBSTITUTE
CM032588.1	79100	.	T	A	100	*	TYPE=SUBSTITUTE
CM032588.1	79102	.	T	C	100	*	TYPE=SUBSTITUTE
CM032588.1	79123	.	C	G	100	*	TYPE=SUBSTITUTE
CM032588.1	79126	.	G	A	100	*	TYPE=SUBSTITUTE
CM032588.1	79186	.	G	a	100	*	TYPE=SUBSTITUTE
CM032588.1	79213	.	C	T	100	*	TYPE=SUBSTITUTE
CM032588.1	79228	.	C	t	100	*	TYPE=SUBSTITUTE
CM032588.1	79231	.	A	c	100	*	TYPE=SUBSTITUTE
CM032588.1	79264	.	C	T	100	*	TYPE=SUBSTITUTE
CM032588.1	79267	.	C	T	100	*	TYPE=SUBSTITUTE
CM032588.1	79337	.	C	T	100	*	TYPE=SUBSTITUTE
CM032588.1	79351	.	T	C	100	*	TYPE=SUBSTITUTE
CM032588.1	79357	.	A	G	100	*	TYPE=SUBSTITUTE
CM032588.1	79369	.	C	T	100	*	TYPE=SUBSTITUTE
CM032588.1	79381	.	A	C	100	*	TYPE=SUBSTITUTE
CM032588.1	79393	.	G	A	100	*	TYPE=SUBSTITUTE
CM032588.1	79399	.	G	C	100	*	TYPE=SUBSTITUTE
CM032588.1	79418	.	C	T	100	*	TYPE=SUBSTITUTE
CM032588.1	79422	.	A	G	100	*	TYPE=SUBSTITUTE
CM032588.1	79468	.	G	A	100	*	TYPE=SUBSTITUTE
CM032588.1	79504	.	C	T	100	*	TYPE=SUBSTITUTE
CM032588.1	79507	.	G	T	100	*	TYPE=SUBSTITUTE
CM032588.1	79516	.	C	T	100	*	TYPE=SUBSTITUTE

CM032588.1	79537	.	C	T	100	*	TYPE=SUBSTITUTE
CM032588.1	79582	.	G	A	100	*	TYPE=SUBSTITUTE
CM032588.1	79639	.	T	C	100	*	TYPE=SUBSTITUTE
CM032588.1	79642	.	C	T	100	*	TYPE=SUBSTITUTE
CM032588.1	79724	.	T	A	100	*	TYPE=SUBSTITUTE
CM032588.1	79735	.	C	T	100	*	TYPE=SUBSTITUTE
CM032588.1	79738	.	G	A	100	*	TYPE=SUBSTITUTE
CM032588.1	79766	.	T	C	100	*	TYPE=SUBSTITUTE
CM032588.1	79811	.	G	A	100	*	TYPE=SUBSTITUTE
CM032588.1	79820	.	T	C	100	*	TYPE=SUBSTITUTE
CM032588.1	79823	.	A	G	100	*	TYPE=SUBSTITUTE
CM032588.1	79875	.	C	G	100	*	TYPE=SUBSTITUTE
CM032588.1	79908	.	G	a	100	*	TYPE=SUBSTITUTE
CM032588.1	79955	.	T	C	100	*	TYPE=SUBSTITUTE
CM032588.1	80063	.	A	G	100	*	TYPE=SUBSTITUTE
CM032588.1	80138	.	A	T	100	*	TYPE=SUBSTITUTE
CM032588.1	80165	.	T	C	100	*	TYPE=SUBSTITUTE
CM032588.1	80219	.	C	T	100	*	TYPE=SUBSTITUTE
CM032588.1	80232	.	G	A	100	*	TYPE=SUBSTITUTE
CM032588.1	80305	.	C	G	100	*	TYPE=SUBSTITUTE
CM032588.1	80384	.	G	A	100	*	TYPE=SUBSTITUTE
CM032588.1	80406	.	C	T	100	*	TYPE=SUBSTITUTE
CM032588.1	80441	.	C	A	100	*	TYPE=SUBSTITUTE
CM032588.1	80456	.	T	C	100	*	TYPE=SUBSTITUTE
CM032588.1	80458	.	G	A	100	*	TYPE=SUBSTITUTE
CM032588.1	80460	.	C	G	100	*	TYPE=SUBSTITUTE
CM032588.1	80483	.	T	C	100	*	TYPE=SUBSTITUTE
CM032588.1	80510	.	A	G	100	*	TYPE=SUBSTITUTE
CM032588.1	80516	.	A	G	100	*	TYPE=SUBSTITUTE
CM032588.1	80528	.	C	A	100	*	TYPE=SUBSTITUTE
CM032588.1	80555	.	C	T	100	*	TYPE=SUBSTITUTE
CM032588.1	80558	.	A	G	100	*	TYPE=SUBSTITUTE
CM032588.1	80561	.	G	T	100	*	TYPE=SUBSTITUTE
CM032588.1	80615	.	A	G	100	*	TYPE=SUBSTITUTE
CM032588.1	80623	.	G	T	100	*	TYPE=SUBSTITUTE
CM032588.1	80627	.	C	T	100	*	TYPE=SUBSTITUTE
CM032588.1	80628	.	T	C	100	*	TYPE=SUBSTITUTE
CM032588.1	80630	.	G	A	100	*	TYPE=SUBSTITUTE
CM032588.1	80642	.	C	T	100	*	TYPE=SUBSTITUTE
CM032588.1	80645	.	T	C	100	*	TYPE=SUBSTITUTE
CM032588.1	80648	.	T	C	100	*	TYPE=SUBSTITUTE
CM032588.1	80657	.	T	TA	100	*	TYPE=INSERT
CM032588.1	80665	.	G	A	100	*	TYPE=SUBSTITUTE
CM032588.1	80667	.	G	C	100	*	TYPE=SUBSTITUTE
CM032588.1	80676	.	T	C	100	*	TYPE=SUBSTITUTE
CM032588.1	80687	.	AT	A	100	*	TYPE=DELETE
CM032588.1	80689	.	C	G	100	*	TYPE=SUBSTITUTE
CM032588.1	80699	.	GA	G	100	*	TYPE=DELETE
CM032588.1	80708	.	G	A	100	*	TYPE=SUBSTITUTE
CM032588.1	80711	.	T	G	100	*	TYPE=SUBSTITUTE
CM032588.1	80712	.	T	C	100	*	TYPE=SUBSTITUTE
CM032588.1	80726	.	T	A	100	*	TYPE=SUBSTITUTE
CM032588.1	80727	.	T	C	100	*	TYPE=SUBSTITUTE
CM032588.1	80731	.	T	C	100	*	TYPE=SUBSTITUTE
CM032588.1	80732	.	A	G	100	*	TYPE=SUBSTITUTE
CM032588.1	80738	.	G	A	100	*	TYPE=SUBSTITUTE
CM032588.1	80741	.	T	C	100	*	TYPE=SUBSTITUTE
CM032588.1	80747	.	T	A	100	*	TYPE=SUBSTITUTE
CM032588.1	80757	.	C	T	100	*	TYPE=SUBSTITUTE
CM032588.1	80762	.	A	T	100	*	TYPE=SUBSTITUTE
CM032588.1	80768	.	A	C	100	*	TYPE=SUBSTITUTE
CM032588.1	80777	.	T	TG	100	*	TYPE=INSERT
CM032588.1	80779	.	G	C	100	*	TYPE=SUBSTITUTE
CM032588.1	80786	.	T	C	100	*	TYPE=SUBSTITUTE

CM032588.1	80791	.	C	T	100	*	TYPE=SUBSTITUTE
CM032588.1	80970	.	C	T	100	*	TYPE=SUBSTITUTE
CM032588.1	81009	.	T	C	100	*	TYPE=SUBSTITUTE
CM032588.1	81038	.	G	A	100	*	TYPE=SUBSTITUTE
CM032588.1	81051	.	A	G	100	*	TYPE=SUBSTITUTE
CM032588.1	81075	.	A	G	100	*	TYPE=SUBSTITUTE
CM032588.1	81144	.	A	T	100	*	TYPE=SUBSTITUTE
CM032588.1	81150	.	A	G	100	*	TYPE=SUBSTITUTE
CM032588.1	81162	.	C	G	100	*	TYPE=SUBSTITUTE
CM032588.1	81183	.	G	A	100	*	TYPE=SUBSTITUTE
CM032588.1	81198	.	C	A	100	*	TYPE=SUBSTITUTE
CM032588.1	81204	.	A	G	100	*	TYPE=SUBSTITUTE
CM032588.1	81216	.	A	T	100	*	TYPE=SUBSTITUTE
CM032588.1	81231	.	A	G	100	*	TYPE=SUBSTITUTE
CM032588.1	81284	.	A	G	100	*	TYPE=SUBSTITUTE
CM032588.1	81369	.	G	A	100	*	TYPE=SUBSTITUTE
CM032588.1	81483	.	C	G	100	*	TYPE=SUBSTITUTE
CM032588.1	81519	.	A	G	100	*	TYPE=SUBSTITUTE
CM032588.1	81525	.	G	A	100	*	TYPE=SUBSTITUTE
CM032588.1	81528	.	T	C	100	*	TYPE=SUBSTITUTE
CM032588.1	81531	.	G	A	100	*	TYPE=SUBSTITUTE
CM032588.1	81540	.	C	T	100	*	TYPE=SUBSTITUTE
CM032588.1	81582	.	A	G	100	*	TYPE=SUBSTITUTE
CM032588.1	81588	.	A	G	100	*	TYPE=SUBSTITUTE
CM032588.1	81591	.	A	G	100	*	TYPE=SUBSTITUTE
CM032588.1	81603	.	A	G	100	*	TYPE=SUBSTITUTE
CM032588.1	81624	.	G	A	100	*	TYPE=SUBSTITUTE
CM032588.1	81636	.	C	G	100	*	TYPE=SUBSTITUTE
CM032588.1	81660	.	C	A	100	*	TYPE=SUBSTITUTE
CM032588.1	81667	.	A	G	100	*	TYPE=SUBSTITUTE
CM032588.1	81670	.	G	A	100	*	TYPE=SUBSTITUTE
CM032588.1	81679	.	A	G	100	*	TYPE=SUBSTITUTE
CM032588.1	81681	.	T	C	100	*	TYPE=SUBSTITUTE
CM032588.1	81750	.	G	A	100	*	TYPE=SUBSTITUTE
CM032588.1	81774	.	A	G	100	*	TYPE=SUBSTITUTE
CM032588.1	81866	.	G	T	100	*	TYPE=SUBSTITUTE
CM032588.1	81911	.	G	C	100	*	TYPE=SUBSTITUTE
CM032588.1	81918	.	G	C	100	*	TYPE=SUBSTITUTE
CM032588.1	81985	.	A	C	100	*	TYPE=SUBSTITUTE
CM032588.1	82000	.	T	A	100	*	TYPE=SUBSTITUTE
CM032588.1	82005	.	A	G	100	*	TYPE=SUBSTITUTE
CM032588.1	82023	.	C	G	100	*	TYPE=SUBSTITUTE
CM032588.1	82126	.	G	A	100	*	TYPE=SUBSTITUTE
CM032588.1	82172	.	T	G	100	*	TYPE=SUBSTITUTE
CM032588.1	82182	.	A	T	100	*	TYPE=SUBSTITUTE
CM032588.1	82203	.	A	G	100	*	TYPE=SUBSTITUTE
CM032588.1	82204	.	C	A	100	*	TYPE=SUBSTITUTE
CM032588.1	82208	.	C	G	100	*	TYPE=SUBSTITUTE
CM032588.1	82217	.	A	G	100	*	TYPE=SUBSTITUTE
CM032588.1	82226	.	A	G	100	*	TYPE=SUBSTITUTE
CM032588.1	82234	.	T	C	100	*	TYPE=SUBSTITUTE
CM032588.1	82242	.	T	C	100	*	TYPE=SUBSTITUTE
CM032588.1	82244	.	A	T	100	*	TYPE=SUBSTITUTE
CM032588.1	82247	.	A	C	100	*	TYPE=SUBSTITUTE
CM032588.1	82319	.	C	CA	100	*	TYPE=INSERT
CM032588.1	82326	.	T	G	100	*	TYPE=SUBSTITUTE
CM032588.1	82330	.	G	T	100	*	TYPE=SUBSTITUTE
CM032588.1	82334	.	C	G	100	*	TYPE=SUBSTITUTE
CM032588.1	82337	.	C	T	100	*	TYPE=SUBSTITUTE
CM032588.1	82341	.	T	A	100	*	TYPE=SUBSTITUTE
CM032588.1	82348	.	T	G	100	*	TYPE=SUBSTITUTE
CM032588.1	82354	.	T	G	100	*	TYPE=SUBSTITUTE
CM032588.1	82365	.	G	T	100	*	TYPE=SUBSTITUTE
CM032588.1	82373	.	C	T	100	*	TYPE=SUBSTITUTE

CM032588.1	82375	.	C	A	100	*	TYPE=SUBSTITUTE
CM032588.1	82381	.	G	A	100	*	TYPE=SUBSTITUTE
CM032588.1	82411	.	A	AA	100	*	TYPE=INSERT
CM032588.1	82433	.	A	c	100	*	TYPE=SUBSTITUTE
CM032588.1	82459	.	T	C	100	*	TYPE=SUBSTITUTE
CM032588.1	82478	.	T	C	100	*	TYPE=SUBSTITUTE
CM032588.1	82494	.	C	T	100	*	TYPE=SUBSTITUTE
CM032588.1	82496	.	C	T	100	*	TYPE=SUBSTITUTE
CM032588.1	82498	.	G	C	100	*	TYPE=SUBSTITUTE
CM032588.1	82514	.	T	A	100	*	TYPE=SUBSTITUTE
CM032588.1	82536	.	G	C	100	*	TYPE=SUBSTITUTE
CM032588.1	82566	.	T	G	100	*	TYPE=SUBSTITUTE
CM032588.1	82567	.	G	A	100	*	TYPE=SUBSTITUTE
CM032588.1	82635	.	C	A	100	*	TYPE=SUBSTITUTE
CM032588.1	82647	.	A	G	100	*	TYPE=SUBSTITUTE
CM032588.1	82676	.	G	a	100	*	TYPE=SUBSTITUTE
CM032588.1	82708	.	T	C	100	*	TYPE=SUBSTITUTE
CM032588.1	82760	.	C	G	100	*	TYPE=SUBSTITUTE
CM032588.1	82982	.	A	C	100	*	TYPE=SUBSTITUTE
CM032588.1	82985	.	C	T	100	*	TYPE=SUBSTITUTE
CM032588.1	83016	.	A	G	100	*	TYPE=SUBSTITUTE
CM032588.1	83018	.	T	A	100	*	TYPE=SUBSTITUTE
CM032588.1	83019	.	CT	C	100	*	TYPE=DELETE
CM032588.1	83067	.	C	T	100	*	TYPE=SUBSTITUTE
CM032588.1	83092	.	A	G	100	*	TYPE=SUBSTITUTE
CM032588.1	83094	.	A	G	100	*	TYPE=SUBSTITUTE
CM032588.1	83095	.	A	G	100	*	TYPE=SUBSTITUTE
CM032588.1	83096	.	T	C	100	*	TYPE=SUBSTITUTE
CM032588.1	83101	.	C	T	100	*	TYPE=SUBSTITUTE
CM032588.1	83111	.	C	T	100	*	TYPE=SUBSTITUTE
CM032588.1	83114	.	A	C	100	*	TYPE=SUBSTITUTE
CM032588.1	83115	.	G	A	100	*	TYPE=SUBSTITUTE
CM032588.1	83120	.	G	T	100	*	TYPE=SUBSTITUTE
CM032588.1	83211	.	A	G	100	*	TYPE=SUBSTITUTE
CM032588.1	83223	.	C	T	100	*	TYPE=SUBSTITUTE
CM032588.1	83230	.	C	G	100	*	TYPE=SUBSTITUTE
CM032588.1	83249	.	C	A	100	*	TYPE=SUBSTITUTE
CM032588.1	83252	.	C	A	100	*	TYPE=SUBSTITUTE
CM032588.1	83261	.	T	A	100	*	TYPE=SUBSTITUTE
CM032588.1	83282	.	C	T	100	*	TYPE=SUBSTITUTE
CM032588.1	83285	.	A	G	100	*	TYPE=SUBSTITUTE
CM032588.1	83312	.	A	c	100	*	TYPE=SUBSTITUTE
CM032588.1	83321	.	G	A	100	*	TYPE=SUBSTITUTE
CM032588.1	83322	.	G	A	100	*	TYPE=SUBSTITUTE
CM032588.1	83324	.	G	A	100	*	TYPE=SUBSTITUTE
CM032588.1	83327	.	C	T	100	*	TYPE=SUBSTITUTE
CM032588.1	83339	.	T	C	100	*	TYPE=SUBSTITUTE
CM032588.1	83342	.	G	A	100	*	TYPE=SUBSTITUTE
CM032588.1	83343	.	C	T	100	*	TYPE=SUBSTITUTE
CM032588.1	83351	.	C	T	100	*	TYPE=SUBSTITUTE
CM032588.1	83357	.	A	G	100	*	TYPE=SUBSTITUTE
CM032588.1	83366	.	T	C	100	*	TYPE=SUBSTITUTE
CM032588.1	83393	.	T	C	100	*	TYPE=SUBSTITUTE
CM032588.1	83498	.	C	T	100	*	TYPE=SUBSTITUTE
CM032588.1	83535	.	T	C	100	*	TYPE=SUBSTITUTE
CM032588.1	83542	.	T	C	100	*	TYPE=SUBSTITUTE
CM032588.1	83543	.	G	A	100	*	TYPE=SUBSTITUTE
CM032588.1	83544	.	T	C	100	*	TYPE=SUBSTITUTE
CM032588.1	83600	.	G	A	100	*	TYPE=SUBSTITUTE
CM032588.1	83630	.	G	A	100	*	TYPE=SUBSTITUTE
CM032588.1	83648	.	C	T	100	*	TYPE=SUBSTITUTE
CM032588.1	83666	.	T	C	100	*	TYPE=SUBSTITUTE
CM032588.1	83673	.	T	C	100	*	TYPE=SUBSTITUTE
CM032588.1	83680	.	G	A	100	*	TYPE=SUBSTITUTE

CM032588.1	83730	.	C	G	100	*	TYPE=SUBSTITUTE
CM032588.1	83750	.	A	C	100	*	TYPE=SUBSTITUTE
CM032588.1	83753	.	G	C	100	*	TYPE=SUBSTITUTE
CM032588.1	83758	.	G	A	100	*	TYPE=SUBSTITUTE
CM032588.1	83771	.	C	T	100	*	TYPE=SUBSTITUTE
CM032588.1	83786	.	C	T	100	*	TYPE=SUBSTITUTE
CM032588.1	83804	.	C	A	100	*	TYPE=SUBSTITUTE
CM032588.1	83806	.	C	T	100	*	TYPE=SUBSTITUTE
CM032588.1	83811	.	G	a	100	*	TYPE=SUBSTITUTE
CM032588.1	83822	.	t	tN	100	*	TYPE=INSERT
CM032588.1	83838	.	C	T	100	*	TYPE=SUBSTITUTE
CM032588.1	83845	.	A	G	100	*	TYPE=SUBSTITUTE
CM032588.1	83847	.	C	G	100	*	TYPE=SUBSTITUTE
CM032588.1	83848	.	C	T	100	*	TYPE=SUBSTITUTE
CM032588.1	83850	.	G	T	100	*	TYPE=SUBSTITUTE
CM032588.1	83854	.	C	T	100	*	TYPE=SUBSTITUTE
CM032588.1	83864	.	G	A	100	*	TYPE=SUBSTITUTE
CM032588.1	83879	.	C	T	100	*	TYPE=SUBSTITUTE
CM032588.1	83884	.	GA	G	100	*	TYPE=DELETE
CM032588.1	83894	.	C	T	100	*	TYPE=SUBSTITUTE
CM032588.1	83942	.	C	T	100	*	TYPE=SUBSTITUTE
CM032588.1	83996	.	C	T	100	*	TYPE=SUBSTITUTE
CM032588.1	84063	.	T	C	100	*	TYPE=SUBSTITUTE
CM032588.1	84065	.	A	G	100	*	TYPE=SUBSTITUTE
CM032588.1	84073	.	A	G	100	*	TYPE=SUBSTITUTE
CM032588.1	84098	.	C	T	100	*	TYPE=SUBSTITUTE
CM032588.1	84125	.	A	G	100	*	TYPE=SUBSTITUTE
CM032588.1	84197	.	C	T	100	*	TYPE=SUBSTITUTE
CM032588.1	84203	.	A	G	100	*	TYPE=SUBSTITUTE
CM032588.1	84211	.	C	T	100	*	TYPE=SUBSTITUTE
CM032588.1	84394	.	T	C	100	*	TYPE=SUBSTITUTE
CM032588.1	84397	.	C	T	100	*	TYPE=SUBSTITUTE
CM032588.1	84398	.	T	G	100	*	TYPE=SUBSTITUTE
CM032588.1	84401	.	T	TA	100	*	TYPE=INSERT
CM032588.1	84416	.	G	A	100	*	TYPE=SUBSTITUTE
CM032588.1	84428	.	C	T	100	*	TYPE=SUBSTITUTE
CM032588.1	84430	.	G	A	100	*	TYPE=SUBSTITUTE
CM032588.1	84445	.	G	T	100	*	TYPE=SUBSTITUTE
CM032588.1	84576	.	T	C	100	*	TYPE=SUBSTITUTE
CM032588.1	84597	.	T	C	100	*	TYPE=SUBSTITUTE
CM032588.1	84654	.	G	A	100	*	TYPE=SUBSTITUTE
CM032588.1	84675	.	A	G	100	*	TYPE=SUBSTITUTE
CM032588.1	84762	.	G	A	100	*	TYPE=SUBSTITUTE
CM032588.1	84789	.	G	A	100	*	TYPE=SUBSTITUTE
CM032588.1	84803	.	T	C	100	*	TYPE=SUBSTITUTE
CM032588.1	84873	.	G	C	100	*	TYPE=SUBSTITUTE
CM032588.1	84888	.	A	G	100	*	TYPE=SUBSTITUTE
CM032588.1	84909	.	G	A	100	*	TYPE=SUBSTITUTE
CM032588.1	84917	.	A	t	100	*	TYPE=SUBSTITUTE
CM032588.1	84932	.	C	t	100	*	TYPE=SUBSTITUTE
CM032588.1	84944	.	C	t	100	*	TYPE=SUBSTITUTE
CM032588.1	84960	.	G	A	100	*	TYPE=SUBSTITUTE
CM032588.1	84961	.	G	A	100	*	TYPE=SUBSTITUTE
CM032588.1	84963	.	T	C	100	*	TYPE=SUBSTITUTE
CM032588.1	84972	.	A	C	100	*	TYPE=SUBSTITUTE
CM032588.1	85002	.	G	A	100	*	TYPE=SUBSTITUTE
CM032588.1	85116	.	A	G	100	*	TYPE=SUBSTITUTE
CM032588.1	85126	.	G	A	100	*	TYPE=SUBSTITUTE
CM032588.1	85130	.	C	CA	100	*	TYPE=INSERT
CM032588.1	85143	.	G	A	100	*	TYPE=SUBSTITUTE
CM032588.1	85144	.	C	T	100	*	TYPE=SUBSTITUTE
CM032588.1	85145	.	T	G	100	*	TYPE=SUBSTITUTE
CM032588.1	85155	.	A	G	100	*	TYPE=SUBSTITUTE
CM032588.1	85175	.	A	G	100	*	TYPE=SUBSTITUTE

CM032588.1	85176	.	C	T	100	*	TYPE=SUBSTITUTE
CM032588.1	85234	.	C	t	100	*	TYPE=SUBSTITUTE
CM032588.1	85243	.	T	c	100	*	TYPE=SUBSTITUTE
CM032588.1	85252	.	C	T	100	*	TYPE=SUBSTITUTE
CM032588.1	85294	.	A	G	100	*	TYPE=SUBSTITUTE
CM032588.1	85385	.	G	C	100	*	TYPE=SUBSTITUTE
CM032588.1	85429	.	A	G	100	*	TYPE=SUBSTITUTE
CM032588.1	85471	.	C	A	100	*	TYPE=SUBSTITUTE
CM032588.1	85537	.	G	C	100	*	TYPE=SUBSTITUTE
CM032588.1	85564	.	C	A	100	*	TYPE=SUBSTITUTE
CM032588.1	85582	.	T	C	100	*	TYPE=SUBSTITUTE
CM032588.1	85585	.	G	A	100	*	TYPE=SUBSTITUTE
CM032588.1	85636	.	C	G	100	*	TYPE=SUBSTITUTE
CM032588.1	85659	.	TC	T	100	*	TYPE=DELETE
CM032588.1	85678	.	A	C	100	*	TYPE=SUBSTITUTE
CM032588.1	85689	.	G	T	100	*	TYPE=SUBSTITUTE
CM032588.1	85695	.	G	C	100	*	TYPE=SUBSTITUTE
CM032588.1	85697	.	C	G	100	*	TYPE=SUBSTITUTE
CM032588.1	85705	.	G	C	100	*	TYPE=SUBSTITUTE
CM032588.1	85707	.	T	G	100	*	TYPE=SUBSTITUTE
CM032588.1	85713	.	T	C	100	*	TYPE=SUBSTITUTE
CM032588.1	85718	.	G	A	100	*	TYPE=SUBSTITUTE
CM032588.1	85739	.	G	A	100	*	TYPE=SUBSTITUTE
CM032588.1	85751	.	A	G	100	*	TYPE=SUBSTITUTE
CM032588.1	85766	.	T	C	100	*	TYPE=SUBSTITUTE
CM032588.1	85772	.	C	T	100	*	TYPE=SUBSTITUTE
CM032588.1	85799	.	A	G	100	*	TYPE=SUBSTITUTE
CM032588.1	85802	.	C	T	100	*	TYPE=SUBSTITUTE
CM032588.1	85862	.	G	C	100	*	TYPE=SUBSTITUTE
CM032588.1	85905	.	A	G	100	*	TYPE=SUBSTITUTE
CM032588.1	85939	.	A	G	100	*	TYPE=SUBSTITUTE
CM032588.1	85946	.	T	C	100	*	TYPE=SUBSTITUTE
CM032588.1	86011	.	G	T	100	*	TYPE=SUBSTITUTE
CM032588.1	86019	.	C	T	100	*	TYPE=SUBSTITUTE
CM032588.1	86022	.	C	T	100	*	TYPE=SUBSTITUTE
CM032588.1	86035	.	G	C	100	*	TYPE=SUBSTITUTE
CM032588.1	86055	.	T	C	100	*	TYPE=SUBSTITUTE
CM032588.1	86097	.	C	T	100	*	TYPE=SUBSTITUTE
CM032588.1	86175	.	G	A	100	*	TYPE=SUBSTITUTE
CM032588.1	86187	.	G	A	100	*	TYPE=SUBSTITUTE
CM032588.1	86206	.	A	G	100	*	TYPE=SUBSTITUTE
CM032588.1	86219	.	C	T	100	*	TYPE=SUBSTITUTE
CM032588.1	86252	.	C	T	100	*	TYPE=SUBSTITUTE
CM032588.1	86266	.	C	A	100	*	TYPE=SUBSTITUTE
CM032588.1	86267	.	G	T	100	*	TYPE=SUBSTITUTE
CM032588.1	86272	.	G	T	100	*	TYPE=SUBSTITUTE
CM032588.1	86273	.	A	G	100	*	TYPE=SUBSTITUTE
CM032588.1	86274	.	A	G	100	*	TYPE=SUBSTITUTE
CM032588.1	86276	.	A	G	100	*	TYPE=SUBSTITUTE
CM032588.1	86277	.	T	C	100	*	TYPE=SUBSTITUTE
CM032588.1	86279	.	C	T	100	*	TYPE=SUBSTITUTE
CM032588.1	86282	.	T	G	100	*	TYPE=SUBSTITUTE
CM032588.1	86291	.	T	G	100	*	TYPE=SUBSTITUTE
CM032588.1	86297	.	C	G	100	*	TYPE=SUBSTITUTE
CM032588.1	86298	.	A	G	100	*	TYPE=SUBSTITUTE
CM032588.1	86375	.	A	G	100	*	TYPE=SUBSTITUTE
CM032588.1	86385	.	C	T	100	*	TYPE=SUBSTITUTE
CM032588.1	86396	.	G	C	100	*	TYPE=SUBSTITUTE
CM032588.1	86419	.	G	A	100	*	TYPE=SUBSTITUTE
CM032588.1	86429	.	T	G	100	*	TYPE=SUBSTITUTE
CM032588.1	86435	.	A	G	100	*	TYPE=SUBSTITUTE
CM032588.1	86445	.	A	C	100	*	TYPE=SUBSTITUTE
CM032588.1	86449	.	C	G	100	*	TYPE=SUBSTITUTE
CM032588.1	86451	.	A	C	100	*	TYPE=SUBSTITUTE

CM032588.1	86480	.	G	C	100	*	TYPE=SUBSTITUTE
CM032588.1	86507	.	T	A	100	*	TYPE=SUBSTITUTE
CM032588.1	86514	.	A	G	100	*	TYPE=SUBSTITUTE
CM032588.1	86519	.	A	G	100	*	TYPE=SUBSTITUTE
CM032588.1	86522	.	A	c	100	*	TYPE=SUBSTITUTE
CM032588.1	86526	.	T	c	100	*	TYPE=SUBSTITUTE
CM032588.1	86582	.	G	C	100	*	TYPE=SUBSTITUTE
CM032588.1	86586	.	G	A	100	*	TYPE=SUBSTITUTE
CM032588.1	86591	.	C	T	100	*	TYPE=SUBSTITUTE
CM032588.1	86598	.	A	G	100	*	TYPE=SUBSTITUTE
CM032588.1	86618	.	A	G	100	*	TYPE=SUBSTITUTE
CM032588.1	86656	.	C	T	100	*	TYPE=SUBSTITUTE
CM032588.1	86712	.	A	AG	100	*	TYPE=INSERT
CM032588.1	86713	.	T	C	100	*	TYPE=SUBSTITUTE
CM032588.1	86731	.	CC	C	100	*	TYPE=DELETE
CM032588.1	86734	.	G	A	100	*	TYPE=SUBSTITUTE
CM032588.1	86770	.	C	T	100	*	TYPE=SUBSTITUTE
CM032588.1	86791	.	G	A	100	*	TYPE=SUBSTITUTE
CM032588.1	86795	.	A	G	100	*	TYPE=SUBSTITUTE
CM032588.1	86815	.	A	G	100	*	TYPE=SUBSTITUTE
CM032588.1	86832	.	T	C	100	*	TYPE=SUBSTITUTE
CM032588.1	86835	.	C	A	100	*	TYPE=SUBSTITUTE
CM032588.1	86838	.	T	C	100	*	TYPE=SUBSTITUTE
CM032588.1	86847	.	T	A	100	*	TYPE=SUBSTITUTE
CM032588.1	86850	.	G	T	100	*	TYPE=SUBSTITUTE
CM032588.1	86856	.	A	G	100	*	TYPE=SUBSTITUTE
CM032588.1	86863	.	A	G	100	*	TYPE=SUBSTITUTE
CM032588.1	86878	.	A	C	100	*	TYPE=SUBSTITUTE
CM032588.1	86885	.	C	T	100	*	TYPE=SUBSTITUTE
CM032588.1	86909	.	C	T	100	*	TYPE=SUBSTITUTE
CM032588.1	86958	.	C	T	100	*	TYPE=SUBSTITUTE
CM032588.1	86961	.	G	A	100	*	TYPE=SUBSTITUTE
CM032588.1	86978	.	A	G	100	*	TYPE=SUBSTITUTE
CM032588.1	86996	.	T	C	100	*	TYPE=SUBSTITUTE
CM032588.1	87007	.	G	A	100	*	TYPE=SUBSTITUTE
CM032588.1	87009	.	G	A	100	*	TYPE=SUBSTITUTE
CM032588.1	87058	.	G	T	100	*	TYPE=SUBSTITUTE
CM032588.1	87073	.	G	A	100	*	TYPE=SUBSTITUTE
CM032588.1	87074	.	C	T	100	*	TYPE=SUBSTITUTE
CM032588.1	87095	.	C	T	100	*	TYPE=SUBSTITUTE
CM032588.1	87102	.	T	G	100	*	TYPE=SUBSTITUTE
CM032588.1	87111	.	C	T	100	*	TYPE=SUBSTITUTE
CM032588.1	87144	.	C	T	100	*	TYPE=SUBSTITUTE
CM032588.1	87146	.	C	T	100	*	TYPE=SUBSTITUTE
CM032588.1	87151	.	T	G	100	*	TYPE=SUBSTITUTE
CM032588.1	87191	.	G	A	100	*	TYPE=SUBSTITUTE
CM032588.1	87192	.	A	T	100	*	TYPE=SUBSTITUTE
CM032588.1	87194	.	A	C	100	*	TYPE=SUBSTITUTE
CM032588.1	87313	.	C	G	100	*	TYPE=SUBSTITUTE
CM032588.1	87331	.	CAC	C	100	*	TYPE=DELETE
CM032588.1	87377	.	G	A	100	*	TYPE=SUBSTITUTE
CM032588.1	87395	.	G	A	100	*	TYPE=SUBSTITUTE
CM032588.1	87424	.	G	A	100	*	TYPE=SUBSTITUTE
CM032588.1	87435	.	A	G	100	*	TYPE=SUBSTITUTE
CM032588.1	87465	.	C	T	100	*	TYPE=SUBSTITUTE
CM032588.1	87475	.	ACCTCTT	A	100	*	TYPE=DELETE
CM032588.1	87515	.	C	T	100	*	TYPE=SUBSTITUTE
CM032588.1	87552	.	T	C	100	*	TYPE=SUBSTITUTE
CM032588.1	87624	.	A	G	100	*	TYPE=SUBSTITUTE
CM032588.1	87648	.	C	T	100	*	TYPE=SUBSTITUTE
CM032588.1	87651	.	T	C	100	*	TYPE=SUBSTITUTE
CM032588.1	87675	.	A	C	100	*	TYPE=SUBSTITUTE
CM032588.1	87678	.	T	C	100	*	TYPE=SUBSTITUTE
CM032588.1	87705	.	T	A	100	*	TYPE=SUBSTITUTE

CM032588.1	87707	.	C	T	100	*	TYPE=SUBSTITUTE
CM032588.1	87710	.	C	T	100	*	TYPE=SUBSTITUTE
CM032588.1	87714	.	C	T	100	*	TYPE=SUBSTITUTE
CM032588.1	87724	.	G	A	100	*	TYPE=SUBSTITUTE
CM032588.1	87729	.	C	T	100	*	TYPE=SUBSTITUTE
CM032588.1	87731	.	G	T	100	*	TYPE=SUBSTITUTE
CM032588.1	87744	.	TTG	T	100	*	TYPE=DELETE
CM032588.1	87770	.	T	C	100	*	TYPE=SUBSTITUTE
CM032588.1	87800	.	G	A	100	*	TYPE=SUBSTITUTE
CM032588.1	87821	.	G	A	100	*	TYPE=SUBSTITUTE
CM032588.1	87872	.	C	A	100	*	TYPE=SUBSTITUTE
CM032588.1	87923	.	C	T	100	*	TYPE=SUBSTITUTE
CM032588.1	87944	.	A	T	100	*	TYPE=SUBSTITUTE
CM032588.1	87959	.	C	T	100	*	TYPE=SUBSTITUTE
CM032588.1	87968	.	C	G	100	*	TYPE=SUBSTITUTE
CM032588.1	87977	.	G	A	100	*	TYPE=SUBSTITUTE
CM032588.1	88004	.	T	C	100	*	TYPE=SUBSTITUTE
CM032588.1	88028	.	G	T	100	*	TYPE=SUBSTITUTE
CM032588.1	88037	.	A	G	100	*	TYPE=SUBSTITUTE
CM032588.1	88070	.	T	C	100	*	TYPE=SUBSTITUTE
CM032588.1	88089	.	G	A	100	*	TYPE=SUBSTITUTE
CM032588.1	88096	.	C	G	100	*	TYPE=SUBSTITUTE
CM032588.1	88157	.	G	A	100	*	TYPE=SUBSTITUTE
CM032588.1	88166	.	C	T	100	*	TYPE=SUBSTITUTE
CM032588.1	88214	.	C	T	100	*	TYPE=SUBSTITUTE
CM032588.1	88215	.	T	C	100	*	TYPE=SUBSTITUTE
CM032588.1	88219	.	G	A	100	*	TYPE=SUBSTITUTE
CM032588.1	88223	.	G	C	100	*	TYPE=SUBSTITUTE
CM032588.1	88230	.	A	G	100	*	TYPE=SUBSTITUTE
CM032588.1	88231	.	C	T	100	*	TYPE=SUBSTITUTE
CM032588.1	88233	.	T	G	100	*	TYPE=SUBSTITUTE
CM032588.1	88243	.	A	C	100	*	TYPE=SUBSTITUTE
CM032588.1	88316	.	T	C	100	*	TYPE=SUBSTITUTE
CM032588.1	88366	.	A	G	100	*	TYPE=SUBSTITUTE
CM032588.1	88370	.	T	C	100	*	TYPE=SUBSTITUTE
CM032588.1	88373	.	C	T	100	*	TYPE=SUBSTITUTE
CM032588.1	88397	.	T	C	100	*	TYPE=SUBSTITUTE
CM032588.1	88400	.	T	C	100	*	TYPE=SUBSTITUTE
CM032588.1	88412	.	G	A	100	*	TYPE=SUBSTITUTE
CM032588.1	88427	.	T	G	100	*	TYPE=SUBSTITUTE
CM032588.1	88436	.	G	A	100	*	TYPE=SUBSTITUTE
CM032588.1	88439	.	A	G	100	*	TYPE=SUBSTITUTE
CM032588.1	88442	.	C	T	100	*	TYPE=SUBSTITUTE
CM032588.1	88447	.	G	C	100	*	TYPE=SUBSTITUTE
CM032588.1	88475	.	A	G	100	*	TYPE=SUBSTITUTE
CM032588.1	88487	.	C	A	100	*	TYPE=SUBSTITUTE
CM032588.1	88490	.	C	T	100	*	TYPE=SUBSTITUTE
CM032588.1	88511	.	T	C	100	*	TYPE=SUBSTITUTE
CM032588.1	88523	.	C	G	100	*	TYPE=SUBSTITUTE
CM032588.1	88541	.	C	T	100	*	TYPE=SUBSTITUTE
CM032588.1	88546	.	G	A	100	*	TYPE=SUBSTITUTE
CM032588.1	88547	.	C	T	100	*	TYPE=SUBSTITUTE
CM032588.1	88612	.	G	A	100	*	TYPE=SUBSTITUTE
CM032588.1	88628	.	A	G	100	*	TYPE=SUBSTITUTE
CM032588.1	88650	.	A	G	100	*	TYPE=SUBSTITUTE
CM032588.1	88670	.	T	C	100	*	TYPE=SUBSTITUTE
CM032588.1	88802	.	A	T	100	*	TYPE=SUBSTITUTE
CM032588.1	88815	.	T	C	100	*	TYPE=SUBSTITUTE
CM032588.1	88856	.	T	C	100	*	TYPE=SUBSTITUTE
CM032588.1	88868	.	A	G	100	*	TYPE=SUBSTITUTE
CM032588.1	88898	.	T	C	100	*	TYPE=SUBSTITUTE
CM032588.1	88916	.	G	A	100	*	TYPE=SUBSTITUTE
CM032588.1	89012	.	T	C	100	*	TYPE=SUBSTITUTE
CM032588.1	89078	.	A	G	100	*	TYPE=SUBSTITUTE

CM032588.1	89184	.	C	T	100	*	TYPE=SUBSTITUTE
CM032588.1	89187	.	C	T	100	*	TYPE=SUBSTITUTE
CM032588.1	89189	.	T	C	100	*	TYPE=SUBSTITUTE
CM032588.1	89195	.	C	A	100	*	TYPE=SUBSTITUTE
CM032588.1	89196	.	G	A	100	*	TYPE=SUBSTITUTE
CM032588.1	89213	.	A	G	100	*	TYPE=SUBSTITUTE
CM032588.1	89243	.	C	T	100	*	TYPE=SUBSTITUTE
CM032588.1	89256	.	T	c	100	*	TYPE=SUBSTITUTE
CM032588.1	89282	.	A	T	100	*	TYPE=SUBSTITUTE
CM032588.1	89285	.	T	C	100	*	TYPE=SUBSTITUTE
CM032588.1	89290	.	T	G	100	*	TYPE=SUBSTITUTE
CM032588.1	90088	.	G	A	100	*	TYPE=SUBSTITUTE
CM032588.1	90092	.	G	A	100	*	TYPE=SUBSTITUTE
CM032588.1	90126	.	C	T	100	*	TYPE=SUBSTITUTE
CM032588.1	90162	.	A	G	100	*	TYPE=SUBSTITUTE
CM032588.1	90174	.	A	G	100	*	TYPE=SUBSTITUTE
CM032588.1	90240	.	G	A	100	*	TYPE=SUBSTITUTE
CM032588.1	90417	.	A	G	100	*	TYPE=SUBSTITUTE
CM032588.1	90575	.	C	G	100	*	TYPE=SUBSTITUTE
CM032588.1	90704	.	A	g	100	*	TYPE=SUBSTITUTE
CM032588.1	90707	.	T	G	100	*	TYPE=SUBSTITUTE
CM032588.1	90713	.	A	G	100	*	TYPE=SUBSTITUTE
CM032588.1	90725	.	G	C	100	*	TYPE=SUBSTITUTE
CM032588.1	90740	.	C	T	100	*	TYPE=SUBSTITUTE
CM032588.1	90770	.	T	C	100	*	TYPE=SUBSTITUTE
CM032588.1	90791	.	G	A	100	*	TYPE=SUBSTITUTE
CM032588.1	90803	.	G	A	100	*	TYPE=SUBSTITUTE
CM032588.1	90842	.	A	G	100	*	TYPE=SUBSTITUTE
CM032588.1	90859	.	G	C	100	*	TYPE=SUBSTITUTE
CM032588.1	90864	.	G	A	100	*	TYPE=SUBSTITUTE
CM032588.1	90870	.	C	T	100	*	TYPE=SUBSTITUTE
CM032588.1	90879	.	A	T	100	*	TYPE=SUBSTITUTE
CM032588.1	90893	.	T	C	100	*	TYPE=SUBSTITUTE
CM032588.1	90897	.	G	A	100	*	TYPE=SUBSTITUTE
CM032588.1	90898	.	T	G	100	*	TYPE=SUBSTITUTE
CM032588.1	90910	.	T	G	100	*	TYPE=SUBSTITUTE
CM032588.1	90924	.	A	G	100	*	TYPE=SUBSTITUTE
CM032588.1	90930	.	T	G	100	*	TYPE=SUBSTITUTE
CM032588.1	90969	.	G	T	100	*	TYPE=SUBSTITUTE
CM032588.1	90975	.	G	A	100	*	TYPE=SUBSTITUTE
CM032588.1	90987	.	A	T	100	*	TYPE=SUBSTITUTE
CM032588.1	90990	.	C	T	100	*	TYPE=SUBSTITUTE
CM032588.1	91089	.	A	G	100	*	TYPE=SUBSTITUTE
CM032588.1	91095	.	G	A	100	*	TYPE=SUBSTITUTE
CM032588.1	91124	.	AA	A	100	*	TYPE=DELETE
CM032588.1	91135	.	T	C	100	*	TYPE=SUBSTITUTE
CM032588.1	91147	.	G	A	100	*	TYPE=SUBSTITUTE
CM032588.1	91186	.	A	G	100	*	TYPE=SUBSTITUTE
CM032588.1	91203	.	A	T	100	*	TYPE=SUBSTITUTE
CM032588.1	91209	.	T	C	100	*	TYPE=SUBSTITUTE
CM032588.1	91242	.	G	C	100	*	TYPE=SUBSTITUTE
CM032588.1	91264	.	A	T	100	*	TYPE=SUBSTITUTE
CM032588.1	91266	.	A	g	100	*	TYPE=SUBSTITUTE
CM032588.1	91268	.	C	t	100	*	TYPE=SUBSTITUTE
CM032588.1	91285	.	T	A	100	*	TYPE=SUBSTITUTE
CM032588.1	91297	.	C	T	100	*	TYPE=SUBSTITUTE
CM032588.1	91307	.	G	A	100	*	TYPE=SUBSTITUTE
CM032588.1	91350	.	T	C	100	*	TYPE=SUBSTITUTE
CM032588.1	91382	.	C	T	100	*	TYPE=SUBSTITUTE
CM032588.1	91390	.	A	G	100	*	TYPE=SUBSTITUTE
CM032588.1	91451	.	A	G	100	*	TYPE=SUBSTITUTE
CM032588.1	91484	.	T	G	100	*	TYPE=SUBSTITUTE
CM032588.1	91657	.	A	G	100	*	TYPE=SUBSTITUTE
CM032588.1	91687	.	TT	T	100	*	TYPE=DELETE

CM032588.1	91776	.	A	G	100	*	TYPE=SUBSTITUTE
CM032588.1	91833	.	T	G	100	*	TYPE=SUBSTITUTE
CM032588.1	92060	.	T	C	100	*	TYPE=SUBSTITUTE
CM032588.1	92062	.	A	G	100	*	TYPE=SUBSTITUTE
CM032588.1	92146	.	C	T	100	*	TYPE=SUBSTITUTE
CM032588.1	92244	.	G	A	100	*	TYPE=SUBSTITUTE
CM032588.1	92255	.	G	A	100	*	TYPE=SUBSTITUTE
CM032588.1	92322	.	T	g	100	*	TYPE=SUBSTITUTE
CM032588.1	92411	.	T	C	100	*	TYPE=SUBSTITUTE
CM032588.1	92514	.	G	A	100	*	TYPE=SUBSTITUTE
CM032588.1	92601	.	A	G	100	*	TYPE=SUBSTITUTE
CM032588.1	92679	.	T	C	100	*	TYPE=SUBSTITUTE
CM032588.1	92881	.	T	G	100	*	TYPE=SUBSTITUTE
CM032588.1	92888	.	CAT	C	100	*	TYPE=DELETE
CM032588.1	93070	.	C	CC	100	*	TYPE=INSERT
CM032588.1	93214	.	T	TATGCACC	100	*	TYPE=INSERT
CM032588.1	93297	.	T	G	100	*	TYPE=SUBSTITUTE
CM032588.1	93674	.	AG	A	100	*	TYPE=DELETE
CM032588.1	93850	.	A	G	100	*	TYPE=SUBSTITUTE
CM032588.1	93867	.	T	C	100	*	TYPE=SUBSTITUTE
CM032588.1	93879	.	GGC	G	100	*	TYPE=DELETE
CM032588.1	94058	.	AT	A	100	*	TYPE=DELETE
CM032588.1	94082	.	G	A	100	*	TYPE=SUBSTITUTE
CM032588.1	94547	.	AAT	A	100	*	TYPE=DELETE
CM032588.1	94602	.	A	G	100	*	TYPE=SUBSTITUTE
CM032588.1	94855	.	C	T	100	*	TYPE=SUBSTITUTE
CM032588.1	94865	.	A	G	100	*	TYPE=SUBSTITUTE
CM032588.1	94890	.	T	C	100	*	TYPE=SUBSTITUTE
CM032588.1	94911	.	G	A	100	*	TYPE=SUBSTITUTE
CM032588.1	94997	.	CC	C	100	*	TYPE=DELETE
CM032588.1	95165	.	T	C	100	*	TYPE=SUBSTITUTE
CM032588.1	95179	.	G	C	100	*	TYPE=SUBSTITUTE
CM032588.1	95267	.	T	G	100	*	TYPE=SUBSTITUTE
CM032588.1	95836	.	T	C	100	*	TYPE=SUBSTITUTE
CM032588.1	95881	.	C	T	100	*	TYPE=SUBSTITUTE
CM032588.1	95889	.	G	A	100	*	TYPE=SUBSTITUTE
CM032588.1	95908	.	C	G	100	*	TYPE=SUBSTITUTE
CM032588.1	95923	.	A	G	100	*	TYPE=SUBSTITUTE
CM032588.1	95950	.	C	T	100	*	TYPE=SUBSTITUTE
CM032588.1	96340	.	A	G	100	*	TYPE=SUBSTITUTE
CM032588.1	96377	.	G	A	100	*	TYPE=SUBSTITUTE
CM032588.1	96536	.	T	A	100	*	TYPE=SUBSTITUTE
CM032588.1	96537	.	C	T	100	*	TYPE=SUBSTITUTE
CM032588.1	96635	.	CT	C	100	*	TYPE=DELETE
CM032588.1	96877	.	A	G	100	*	TYPE=SUBSTITUTE
CM032588.1	97384	.	A	G	100	*	TYPE=SUBSTITUTE
CM032588.1	97604	.	A	G	100	*	TYPE=SUBSTITUTE
CM032588.1	97608	.	G	C	100	*	TYPE=SUBSTITUTE
CM032588.1	97609	.	A	G	100	*	TYPE=SUBSTITUTE
CM032588.1	97622	.	C	T	100	*	TYPE=SUBSTITUTE
CM032588.1	97637	.	G	T	100	*	TYPE=SUBSTITUTE
CM032588.1	97654	.	G	A	100	*	TYPE=SUBSTITUTE
CM032588.1	97656	.	T	C	100	*	TYPE=SUBSTITUTE
CM032588.1	97657	.	G	A	100	*	TYPE=SUBSTITUTE
CM032588.1	97670	.	C	T	100	*	TYPE=SUBSTITUTE
CM032588.1	97687	.	G	A	100	*	TYPE=SUBSTITUTE
CM032588.1	97690	.	A	G	100	*	TYPE=SUBSTITUTE
CM032588.1	97711	.	T	C	100	*	TYPE=SUBSTITUTE
CM032588.1	97732	.	A	G	100	*	TYPE=SUBSTITUTE
CM032588.1	97758	.	C	T	100	*	TYPE=SUBSTITUTE
CM032588.1	97762	.	A	G	100	*	TYPE=SUBSTITUTE
CM032588.1	97771	.	G	A	100	*	TYPE=SUBSTITUTE
CM032588.1	97783	.	G	A	100	*	TYPE=SUBSTITUTE
CM032588.1	97899	.	G	A	100	*	TYPE=SUBSTITUTE

CM032588.1	97923	.	C	T	100	*	TYPE=SUBSTITUTE
CM032588.1	97927	.	N	NNNTAA	100	*	TYPE=INSERT
CM032588.1	97950	.	G	C	100	*	TYPE=SUBSTITUTE
CM032588.1	97978	.	G	A	100	*	TYPE=SUBSTITUTE
CM032588.1	98139	.	T	G	100	*	TYPE=SUBSTITUTE
CM032588.1	98183	.	A	G	100	*	TYPE=SUBSTITUTE
CM032588.1	98202	.	C	T	100	*	TYPE=SUBSTITUTE
CM032588.1	98212	.	A	T	100	*	TYPE=SUBSTITUTE
CM032588.1	98240	.	G	A	100	*	TYPE=SUBSTITUTE
CM032588.1	98262	.	C	T	100	*	TYPE=SUBSTITUTE
CM032588.1	98279	.	A	G	100	*	TYPE=SUBSTITUTE
CM032588.1	98283	.	G	C	100	*	TYPE=SUBSTITUTE
CM032588.1	98554	.	C	A	100	*	TYPE=SUBSTITUTE
CM032588.1	98562	.	AAC	A	100	*	TYPE=DELETE
CM032588.1	98573	.	C	T	100	*	TYPE=SUBSTITUTE
CM032588.1	98671	.	A	G	100	*	TYPE=SUBSTITUTE
CM032588.1	98962	.	C	T	100	*	TYPE=SUBSTITUTE
CM032588.1	99008	.	T	C	100	*	TYPE=SUBSTITUTE
CM032588.1	99014	.	T	C	100	*	TYPE=SUBSTITUTE
CM032588.1	99015	.	G	A	100	*	TYPE=SUBSTITUTE
CM032588.1	99022	.	G	A	100	*	TYPE=SUBSTITUTE
CM032588.1	99027	.	C	T	100	*	TYPE=SUBSTITUTE
CM032588.1	99028	.	A	G	100	*	TYPE=SUBSTITUTE
CM032588.1	99030	.	G	A	100	*	TYPE=SUBSTITUTE
CM032588.1	99260	.	C	T	100	*	TYPE=SUBSTITUTE
CM032588.1	99439	.	TT	T	100	*	TYPE=DELETE
CM032588.1	99464	.	C	t	100	*	TYPE=SUBSTITUTE
CM032588.1	99500	.	T	C	100	*	TYPE=SUBSTITUTE
CM032588.1	99554	.	T	G	100	*	TYPE=SUBSTITUTE
CM032588.1	99560	.	A	G	100	*	TYPE=SUBSTITUTE
CM032588.1	99563	.	T	C	100	*	TYPE=SUBSTITUTE
CM032588.1	99588	.	T	C	100	*	TYPE=SUBSTITUTE
CM032588.1	99755	.	A	G	100	*	TYPE=SUBSTITUTE
CM032588.1	99856	.	G	C	100	*	TYPE=SUBSTITUTE
CM032588.1	99905	.	C	T	100	*	TYPE=SUBSTITUTE
CM032588.1	99957	.	C	T	100	*	TYPE=SUBSTITUTE
CM032588.1	100068	.	T	C	100	*	TYPE=SUBSTITUTE
CM032588.1	100193	.	G	C	100	*	TYPE=SUBSTITUTE
CM032588.1	100313	.	G	A	100	*	TYPE=SUBSTITUTE
CM032588.1	100358	.	C	T	100	*	TYPE=SUBSTITUTE
CM032588.1	100488	.	C	T	100	*	TYPE=SUBSTITUTE
CM032588.1	100550	.	G	A	100	*	TYPE=SUBSTITUTE
CM032588.1	100630	.	G	A	100	*	TYPE=SUBSTITUTE
CM032588.1	100634	.	G	A	100	*	TYPE=SUBSTITUTE
CM032588.1	100643	.	G	t	100	*	TYPE=SUBSTITUTE
CM032588.1	100645	.	C	a	100	*	TYPE=SUBSTITUTE
CM032588.1	100664	.	A	G	100	*	TYPE=SUBSTITUTE
CM032588.1	100679	.	C	G	100	*	TYPE=SUBSTITUTE
CM032588.1	100681	.	A	G	100	*	TYPE=SUBSTITUTE
CM032588.1	100682	.	A	G	100	*	TYPE=SUBSTITUTE
CM032588.1	100686	.	C	G	100	*	TYPE=SUBSTITUTE
CM032588.1	100688	.	G	A	100	*	TYPE=SUBSTITUTE
CM032588.1	100689	.	G	A	100	*	TYPE=SUBSTITUTE
CM032588.1	100695	.	T	C	100	*	TYPE=SUBSTITUTE
CM032588.1	100735	.	CA	C	100	*	TYPE=DELETE
CM032588.1	100752	.	A	T	100	*	TYPE=SUBSTITUTE
CM032588.1	100758	.	G	C	100	*	TYPE=SUBSTITUTE
CM032588.1	100762	.	T	C	100	*	TYPE=SUBSTITUTE
CM032588.1	100768	.	T	G	100	*	TYPE=SUBSTITUTE
CM032588.1	100778	.	T	G	100	*	TYPE=SUBSTITUTE
CM032588.1	100781	.	C	T	100	*	TYPE=SUBSTITUTE
CM032588.1	100803	.	T	C	100	*	TYPE=SUBSTITUTE
CM032588.1	100805	.	A	T	100	*	TYPE=SUBSTITUTE
CM032588.1	100809	.	G	A	100	*	TYPE=SUBSTITUTE

CM032588.1	100822	.	T	A	100	*	TYPE=SUBSTITUTE
CM032588.1	100824	.	A	G	100	*	TYPE=SUBSTITUTE
CM032588.1	100829	.	A	G	100	*	TYPE=SUBSTITUTE
CM032588.1	100830	.	C	T	100	*	TYPE=SUBSTITUTE
CM032588.1	100831	.	A	G	100	*	TYPE=SUBSTITUTE
CM032588.1	100834	.	A	G	100	*	TYPE=SUBSTITUTE
CM032588.1	100846	.	G	T	100	*	TYPE=SUBSTITUTE
CM032588.1	100849	.	T	A	100	*	TYPE=SUBSTITUTE
CM032588.1	100850	.	C	T	100	*	TYPE=SUBSTITUTE
CM032588.1	100856	.	G	A	100	*	TYPE=SUBSTITUTE
CM032588.1	100859	.	A	G	100	*	TYPE=SUBSTITUTE
CM032588.1	100875	.	G	A	100	*	TYPE=SUBSTITUTE
CM032588.1	100882	.	A	T	100	*	TYPE=SUBSTITUTE
CM032588.1	100893	.	T	G	100	*	TYPE=SUBSTITUTE
CM032588.1	100909	.	A	G	100	*	TYPE=SUBSTITUTE
CM032588.1	100910	.	C	T	100	*	TYPE=SUBSTITUTE
CM032588.1	100965	.	G	A	100	*	TYPE=SUBSTITUTE
CM032588.1	100987	.	A	G	100	*	TYPE=SUBSTITUTE
CM032588.1	100989	.	C	T	100	*	TYPE=SUBSTITUTE
CM032588.1	101016	.	A	G	100	*	TYPE=SUBSTITUTE
CM032588.1	101062	.	T	G	100	*	TYPE=SUBSTITUTE
CM032588.1	101064	.	T	G	100	*	TYPE=SUBSTITUTE
CM032588.1	101087	.	T	C	100	*	TYPE=SUBSTITUTE
CM032588.1	101091	.	G	A	100	*	TYPE=SUBSTITUTE
CM032588.1	101092	.	G	C	100	*	TYPE=SUBSTITUTE
CM032588.1	101122	.	C	T	100	*	TYPE=SUBSTITUTE
CM032588.1	101186	.	A	C	100	*	TYPE=SUBSTITUTE
CM032588.1	101205	.	A	G	100	*	TYPE=SUBSTITUTE
CM032588.1	101206	.	A	C	100	*	TYPE=SUBSTITUTE
CM032588.1	101209	.	C	T	100	*	TYPE=SUBSTITUTE
CM032588.1	101213	.	T	C	100	*	TYPE=SUBSTITUTE
CM032588.1	101226	.	T	A	100	*	TYPE=SUBSTITUTE
CM032588.1	101232	.	C	T	100	*	TYPE=SUBSTITUTE
CM032588.1	101235	.	T	C	100	*	TYPE=SUBSTITUTE
CM032588.1	101237	.	G	A	100	*	TYPE=SUBSTITUTE
CM032588.1	101247	.	G	A	100	*	TYPE=SUBSTITUTE
CM032588.1	101280	.	A	G	100	*	TYPE=SUBSTITUTE
CM032588.1	101281	.	T	G	100	*	TYPE=SUBSTITUTE
CM032588.1	101288	.	G	A	100	*	TYPE=SUBSTITUTE
CM032588.1	101407	.	C	G	100	*	TYPE=SUBSTITUTE
CM032588.1	101424	.	C	T	100	*	TYPE=SUBSTITUTE
CM032588.1	101426	.	C	A	100	*	TYPE=SUBSTITUTE
CM032588.1	101533	.	A	G	100	*	TYPE=SUBSTITUTE
CM032588.1	101631	.	G	A	100	*	TYPE=SUBSTITUTE
CM032588.1	101695	.	C	G	100	*	TYPE=SUBSTITUTE
CM032588.1	101733	.	G	A	100	*	TYPE=SUBSTITUTE
CM032588.1	101738	.	A	G	100	*	TYPE=SUBSTITUTE
CM032588.1	101842	.	A	G	100	*	TYPE=SUBSTITUTE
CM032588.1	101890	.	C	T	100	*	TYPE=SUBSTITUTE
CM032588.1	101906	.	C	A	100	*	TYPE=SUBSTITUTE
CM032588.1	101943	.	G	A	100	*	TYPE=SUBSTITUTE
CM032588.1	102060	.	C	T	100	*	TYPE=SUBSTITUTE
CM032588.1	102128	.	G	T	100	*	TYPE=SUBSTITUTE
CM032588.1	102131	.	G	A	100	*	TYPE=SUBSTITUTE
CM032588.1	102178	.	C	T	100	*	TYPE=SUBSTITUTE
CM032588.1	102209	.	A	G	100	*	TYPE=SUBSTITUTE
CM032588.1	102212	.	G	T	100	*	TYPE=SUBSTITUTE
CM032588.1	102317	.	C	T	100	*	TYPE=SUBSTITUTE
CM032588.1	102372	.	A	C	100	*	TYPE=SUBSTITUTE
CM032588.1	102386	.	T	G	100	*	TYPE=SUBSTITUTE
CM032588.1	102453	.	T	C	100	*	TYPE=SUBSTITUTE
CM032588.1	102455	.	A	G	100	*	TYPE=SUBSTITUTE
CM032588.1	102500	.	A	G	100	*	TYPE=SUBSTITUTE
CM032588.1	102515	.	T	C	100	*	TYPE=SUBSTITUTE

CM032588.1	102552	.	A	G	100	*	TYPE=SUBSTITUTE
CM032588.1	102554	.	G	C	100	*	TYPE=SUBSTITUTE
CM032588.1	102686	.	A	G	100	*	TYPE=SUBSTITUTE
CM032588.1	102794	.	G	A	100	*	TYPE=SUBSTITUTE
CM032588.1	102803	.	C	T	100	*	TYPE=SUBSTITUTE
CM032588.1	102842	.	C	T	100	*	TYPE=SUBSTITUTE
CM032588.1	102851	.	A	C	100	*	TYPE=SUBSTITUTE
CM032588.1	102866	.	G	A	100	*	TYPE=SUBSTITUTE
CM032588.1	102884	.	C	A	100	*	TYPE=SUBSTITUTE
CM032588.1	102902	.	G	A	100	*	TYPE=SUBSTITUTE
CM032588.1	102908	.	T	C	100	*	TYPE=SUBSTITUTE
CM032588.1	102909	.	A	T	100	*	TYPE=SUBSTITUTE
CM032588.1	102950	.	C	T	100	*	TYPE=SUBSTITUTE
CM032588.1	102963	.	T	C	100	*	TYPE=SUBSTITUTE
CM032588.1	102967	.	C	T	100	*	TYPE=SUBSTITUTE
CM032588.1	102979	.	A	G	100	*	TYPE=SUBSTITUTE
CM032588.1	102992	.	T	C	100	*	TYPE=SUBSTITUTE
CM032588.1	103007	.	A	G	100	*	TYPE=SUBSTITUTE
CM032588.1	103040	.	G	A	100	*	TYPE=SUBSTITUTE
CM032588.1	103061	.	A	C	100	*	TYPE=SUBSTITUTE
CM032588.1	103064	.	G	A	100	*	TYPE=SUBSTITUTE
CM032588.1	103112	.	T	C	100	*	TYPE=SUBSTITUTE
CM032588.1	103166	.	G	A	100	*	TYPE=SUBSTITUTE
CM032588.1	103187	.	A	G	100	*	TYPE=SUBSTITUTE
CM032588.1	103331	.	C	G	100	*	TYPE=SUBSTITUTE
CM032588.1	103424	.	C	A	100	*	TYPE=SUBSTITUTE
CM032588.1	103561	.	C	T	100	*	TYPE=SUBSTITUTE
CM032588.1	103686	.	A	T	100	*	TYPE=SUBSTITUTE
CM032588.1	103725	.	A	T	100	*	TYPE=SUBSTITUTE
CM032588.1	103769	.	C	T	100	*	TYPE=SUBSTITUTE
CM032588.1	103974	.	G	T	100	*	TYPE=SUBSTITUTE
CM032588.1	103989	.	A	G	100	*	TYPE=SUBSTITUTE
CM032588.1	104019	.	A	C	100	*	TYPE=SUBSTITUTE
CM032588.1	104028	.	G	C	100	*	TYPE=SUBSTITUTE
CM032588.1	104118	.	C	T	100	*	TYPE=SUBSTITUTE
CM032588.1	104138	.	C	T	100	*	TYPE=SUBSTITUTE
CM032588.1	104196	.	G	A	100	*	TYPE=SUBSTITUTE
CM032588.1	104304	.	G	A	100	*	TYPE=SUBSTITUTE
CM032588.1	104334	.	A	G	100	*	TYPE=SUBSTITUTE
CM032588.1	104350	.	C	T	100	*	TYPE=SUBSTITUTE
CM032588.1	104352	.	G	A	100	*	TYPE=SUBSTITUTE
CM032588.1	104388	.	G	C	100	*	TYPE=SUBSTITUTE
CM032588.1	104408	.	G	A	100	*	TYPE=SUBSTITUTE
CM032588.1	104442	.	T	C	100	*	TYPE=SUBSTITUTE
CM032588.1	104469	.	A	C	100	*	TYPE=SUBSTITUTE
CM032588.1	104502	.	C	G	100	*	TYPE=SUBSTITUTE
CM032588.1	104535	.	G	A	100	*	TYPE=SUBSTITUTE
CM032588.1	104565	.	A	G	100	*	TYPE=SUBSTITUTE
CM032588.1	104612	.	T	C	100	*	TYPE=SUBSTITUTE
CM032588.1	104626	.	C	T	100	*	TYPE=SUBSTITUTE
CM032588.1	104629	.	C	T	100	*	TYPE=SUBSTITUTE
CM032588.1	104640	.	G	C	100	*	TYPE=SUBSTITUTE
CM032588.1	104652	.	C	A	100	*	TYPE=SUBSTITUTE
CM032588.1	104709	.	C	T	100	*	TYPE=SUBSTITUTE
CM032588.1	104754	.	A	g	100	*	TYPE=SUBSTITUTE
CM032588.1	105022	.	A	G	100	*	TYPE=SUBSTITUTE
CM032588.1	105033	.	C	G	100	*	TYPE=SUBSTITUTE
CM032588.1	105049	.	T	G	100	*	TYPE=SUBSTITUTE
CM032588.1	105050	.	A	G	100	*	TYPE=SUBSTITUTE
CM032588.1	105057	.	T	c	100	*	TYPE=SUBSTITUTE
CM032588.1	105063	.	C	t	100	*	TYPE=SUBSTITUTE
CM032588.1	105065	.	T	c	100	*	TYPE=SUBSTITUTE
CM032588.1	105091	.	C	CC	100	*	TYPE=INSERT
CM032588.1	105103	.	C	T	100	*	TYPE=SUBSTITUTE

CM032588.1	105130	.	t	tta	100	*	TYPE=INSERT
CM032588.1	105137	.	A	t	100	*	TYPE=SUBSTITUTE
CM032588.1	105167	.	G	A	100	*	TYPE=SUBSTITUTE
CM032588.1	105199	.	CTC	C	100	*	TYPE=DELETE
CM032588.1	105284	.	C	A	100	*	TYPE=SUBSTITUTE
CM032588.1	105299	.	C	T	100	*	TYPE=SUBSTITUTE
CM032588.1	105308	.	G	A	100	*	TYPE=SUBSTITUTE
CM032588.1	105342	.	C	T	100	*	TYPE=SUBSTITUTE
CM032588.1	105352	.	C	T	100	*	TYPE=SUBSTITUTE
CM032588.1	105443	.	AC	A	100	*	TYPE=DELETE
CM032588.1	105460	.	T	C	100	*	TYPE=SUBSTITUTE
CM032588.1	105484	.	C	T	100	*	TYPE=SUBSTITUTE
CM032588.1	105487	.	T	C	100	*	TYPE=SUBSTITUTE
CM032588.1	105505	.	A	G	100	*	TYPE=SUBSTITUTE
CM032588.1	105624	.	A	G	100	*	TYPE=SUBSTITUTE
CM032588.1	105660	.	T	C	100	*	TYPE=SUBSTITUTE
CM032588.1	106032	.	G	C	100	*	TYPE=SUBSTITUTE
CM032588.1	106033	.	G	A	100	*	TYPE=SUBSTITUTE
CM032588.1	106214	.	C	G	100	*	TYPE=SUBSTITUTE
CM032588.1	106250	.	G	A	100	*	TYPE=SUBSTITUTE
CM032588.1	106265	.	G	A	100	*	TYPE=SUBSTITUTE
CM032588.1	106270	.	CGG	C	100	*	TYPE=DELETE
CM032588.1	106308	.	G	A	100	*	TYPE=SUBSTITUTE
CM032588.1	106309	.	T	C	100	*	TYPE=SUBSTITUTE
CM032588.1	106338	.	T	C	100	*	TYPE=SUBSTITUTE
CM032588.1	106347	.	A	G	100	*	TYPE=SUBSTITUTE
CM032588.1	106364	.	C	T	100	*	TYPE=SUBSTITUTE
CM032588.1	106366	.	T	C	100	*	TYPE=SUBSTITUTE
CM032588.1	106383	.	T	C	100	*	TYPE=SUBSTITUTE
CM032588.1	106514	.	T	C	100	*	TYPE=SUBSTITUTE
CM032588.1	106523	.	G	A	100	*	TYPE=SUBSTITUTE
CM032588.1	106528	.	A	G	100	*	TYPE=SUBSTITUTE
CM032588.1	106609	.	G	A	100	*	TYPE=SUBSTITUTE
CM032588.1	106626	.	C	T	100	*	TYPE=SUBSTITUTE
CM032588.1	106635	.	A	T	100	*	TYPE=SUBSTITUTE
CM032588.1	106664	.	A	G	100	*	TYPE=SUBSTITUTE
CM032588.1	106665	.	G	A	100	*	TYPE=SUBSTITUTE
CM032588.1	106675	.	C	A	100	*	TYPE=SUBSTITUTE
CM032588.1	106741	.	T	C	100	*	TYPE=SUBSTITUTE
CM032588.1	106758	.	T	G	100	*	TYPE=SUBSTITUTE
CM032588.1	106764	.	C	T	100	*	TYPE=SUBSTITUTE
CM032588.1	106789	.	G	A	100	*	TYPE=SUBSTITUTE
CM032588.1	106794	.	T	C	100	*	TYPE=SUBSTITUTE
CM032588.1	106799	.	T	G	100	*	TYPE=SUBSTITUTE
CM032588.1	106805	.	C	T	100	*	TYPE=SUBSTITUTE
CM032588.1	106821	.	G	A	100	*	TYPE=SUBSTITUTE
CM032588.1	107022	.	C	T	100	*	TYPE=SUBSTITUTE
CM032588.1	107119	.	T	C	100	*	TYPE=SUBSTITUTE
CM032588.1	107205	.	A	C	100	*	TYPE=SUBSTITUTE
CM032588.1	107215	.	G	t	100	*	TYPE=SUBSTITUTE
CM032588.1	107253	.	G	T	100	*	TYPE=SUBSTITUTE
CM032588.1	107283	.	C	A	100	*	TYPE=SUBSTITUTE
CM032588.1	107300	.	A	G	100	*	TYPE=SUBSTITUTE
CM032588.1	107307	.	T	C	100	*	TYPE=SUBSTITUTE
CM032588.1	107309	.	T	C	100	*	TYPE=SUBSTITUTE
CM032588.1	107311	.	T	TTCACA	100	*	TYPE=INSERT
CM032588.1	107361	.	G	A	100	*	TYPE=SUBSTITUTE
CM032588.1	107363	.	G	A	100	*	TYPE=SUBSTITUTE
CM032588.1	107369	.	A	G	100	*	TYPE=SUBSTITUTE
CM032588.1	107392	.	A	G	100	*	TYPE=SUBSTITUTE
CM032588.1	107419	.	A	C	100	*	TYPE=SUBSTITUTE
CM032588.1	107423	.	T	C	100	*	TYPE=SUBSTITUTE
CM032588.1	107528	.	G	A	100	*	TYPE=SUBSTITUTE
CM032588.1	107568	.	G	A	100	*	TYPE=SUBSTITUTE

CM032588.1	107628	.	C	G	100	*	TYPE=SUBSTITUTE
CM032588.1	107668	.	G	A	100	*	TYPE=SUBSTITUTE
CM032588.1	107717	.	T	C	100	*	TYPE=SUBSTITUTE
CM032588.1	107740	.	T	C	100	*	TYPE=SUBSTITUTE
CM032588.1	107751	.	T	C	100	*	TYPE=SUBSTITUTE
CM032588.1	107769	.	C	T	100	*	TYPE=SUBSTITUTE
CM032588.1	107779	.	T	C	100	*	TYPE=SUBSTITUTE
CM032588.1	107802	.	A	G	100	*	TYPE=SUBSTITUTE
CM032588.1	107815	.	T	G	100	*	TYPE=SUBSTITUTE
CM032588.1	107824	.	C	G	100	*	TYPE=SUBSTITUTE
CM032588.1	107853	.	A	C	100	*	TYPE=SUBSTITUTE
CM032588.1	107866	.	G	A	100	*	TYPE=SUBSTITUTE
CM032588.1	107899	.	A	g	100	*	TYPE=SUBSTITUTE
CM032588.1	107906	.	C	t	100	*	TYPE=SUBSTITUTE
CM032588.1	107912	.	T	g	100	*	TYPE=SUBSTITUTE
CM032588.1	107952	.	A	G	100	*	TYPE=SUBSTITUTE
CM032588.1	108004	.	A	C	100	*	TYPE=SUBSTITUTE
CM032588.1	108005	.	T	C	100	*	TYPE=SUBSTITUTE
CM032588.1	108050	.	A	G	100	*	TYPE=SUBSTITUTE
CM032588.1	108112	.	C	A	100	*	TYPE=SUBSTITUTE
CM032588.1	108128	.	G	C	100	*	TYPE=SUBSTITUTE
CM032588.1	108139	.	T	C	100	*	TYPE=SUBSTITUTE
CM032588.1	108146	.	T	G	100	*	TYPE=SUBSTITUTE
CM032588.1	108176	.	T	C	100	*	TYPE=SUBSTITUTE
CM032588.1	108200	.	A	T	100	*	TYPE=SUBSTITUTE
CM032588.1	108209	.	G	A	100	*	TYPE=SUBSTITUTE
CM032588.1	108236	.	T	A	100	*	TYPE=SUBSTITUTE
CM032588.1	108248	.	C	T	100	*	TYPE=SUBSTITUTE
CM032588.1	108308	.	T	C	100	*	TYPE=SUBSTITUTE
CM032588.1	108317	.	C	T	100	*	TYPE=SUBSTITUTE
CM032588.1	108419	.	G	C	100	*	TYPE=SUBSTITUTE
CM032588.1	108427	.	C	A	100	*	TYPE=SUBSTITUTE
CM032588.1	108431	.	G	A	100	*	TYPE=SUBSTITUTE
CM032588.1	108441	.	C	A	100	*	TYPE=SUBSTITUTE
CM032588.1	108472	.	G	A	100	*	TYPE=SUBSTITUTE
CM032588.1	108483	.	C	T	100	*	TYPE=SUBSTITUTE
CM032588.1	108539	.	A	G	100	*	TYPE=SUBSTITUTE
CM032588.1	108546	.	T	G	100	*	TYPE=SUBSTITUTE
CM032588.1	108771	.	C	t	100	*	TYPE=SUBSTITUTE
CM032588.1	108783	.	G	A	100	*	TYPE=SUBSTITUTE
CM032588.1	108818	.	G	A	100	*	TYPE=SUBSTITUTE
CM032588.1	108826	.	C	T	100	*	TYPE=SUBSTITUTE
CM032588.1	108894	.	T	C	100	*	TYPE=SUBSTITUTE
CM032588.1	108899	.	G	A	100	*	TYPE=SUBSTITUTE
CM032588.1	108911	.	C	T	100	*	TYPE=SUBSTITUTE
CM032588.1	108938	.	G	A	100	*	TYPE=SUBSTITUTE
CM032588.1	108951	.	T	C	100	*	TYPE=SUBSTITUTE
CM032588.1	109092	.	A	G	100	*	TYPE=SUBSTITUTE
CM032588.1	109101	.	G	A	100	*	TYPE=SUBSTITUTE
CM032588.1	109114	.	T	G	100	*	TYPE=SUBSTITUTE
CM032588.1	109118	.	C	T	100	*	TYPE=SUBSTITUTE
CM032588.1	109173	.	C	T	100	*	TYPE=SUBSTITUTE
CM032588.1	109177	.	G	A	100	*	TYPE=SUBSTITUTE
CM032588.1	109203	.	C	G	100	*	TYPE=SUBSTITUTE
CM032588.1	109204	.	G	T	100	*	TYPE=SUBSTITUTE
CM032588.1	109216	.	C	T	100	*	TYPE=SUBSTITUTE
CM032588.1	109221	.	A	T	100	*	TYPE=SUBSTITUTE
CM032588.1	109223	.	A	G	100	*	TYPE=SUBSTITUTE
CM032588.1	109230	.	C	T	100	*	TYPE=SUBSTITUTE
CM032588.1	109244	.	C	A	100	*	TYPE=SUBSTITUTE
CM032588.1	109250	.	G	A	100	*	TYPE=SUBSTITUTE
CM032588.1	109258	.	A	G	100	*	TYPE=SUBSTITUTE
CM032588.1	109261	.	C	T	100	*	TYPE=SUBSTITUTE
CM032588.1	109269	.	T	C	100	*	TYPE=SUBSTITUTE

CM032588.1	109280	.	A	T	100	*	TYPE=SUBSTITUTE
CM032588.1	109291	.	A	G	100	*	TYPE=SUBSTITUTE
CM032588.1	109299	.	C	T	100	*	TYPE=SUBSTITUTE
CM032588.1	109317	.	G	a	100	*	TYPE=SUBSTITUTE
CM032588.1	109327	.	A	C	100	*	TYPE=SUBSTITUTE
CM032588.1	109329	.	C	T	100	*	TYPE=SUBSTITUTE
CM032588.1	109360	.	G	A	100	*	TYPE=SUBSTITUTE
CM032588.1	109379	.	A	T	100	*	TYPE=SUBSTITUTE
CM032588.1	109385	.	G	A	100	*	TYPE=SUBSTITUTE
CM032588.1	109496	.	A	T	100	*	TYPE=SUBSTITUTE
CM032588.1	109503	.	C	T	100	*	TYPE=SUBSTITUTE
CM032588.1	109542	.	A	g	100	*	TYPE=SUBSTITUTE
CM032588.1	109555	.	C	G	100	*	TYPE=SUBSTITUTE
CM032588.1	109642	.	T	A	100	*	TYPE=SUBSTITUTE
CM032588.1	109661	.	A	C	100	*	TYPE=SUBSTITUTE
CM032588.1	109662	.	C	A	100	*	TYPE=SUBSTITUTE
CM032588.1	109678	.	TA	T	100	*	TYPE=DELETE
CM032588.1	109681	.	ATT	A	100	*	TYPE=DELETE
CM032588.1	109687	.	G	A	100	*	TYPE=SUBSTITUTE
CM032588.1	109689	.	AT	A	100	*	TYPE=DELETE
CM032588.1	109695	.	G	A	100	*	TYPE=SUBSTITUTE
CM032588.1	109707	.	C	T	100	*	TYPE=SUBSTITUTE
CM032588.1	109716	.	GA	G	100	*	TYPE=DELETE
CM032588.1	109797	.	G	A	100	*	TYPE=SUBSTITUTE
CM032588.1	109818	.	T	C	100	*	TYPE=SUBSTITUTE
CM032588.1	109839	.	A	G	100	*	TYPE=SUBSTITUTE
CM032588.1	109950	.	A	G	100	*	TYPE=SUBSTITUTE
CM032588.1	110019	.	A	G	100	*	TYPE=SUBSTITUTE
CM032588.1	110052	.	C	T	100	*	TYPE=SUBSTITUTE
CM032588.1	110076	.	A	G	100	*	TYPE=SUBSTITUTE
CM032588.1	110077	.	T	C	100	*	TYPE=SUBSTITUTE
CM032588.1	110111	.	G	GN	100	*	TYPE=INSERT
CM032588.1	110122	.	T	C	100	*	TYPE=SUBSTITUTE
CM032588.1	110153	.	A	G	100	*	TYPE=SUBSTITUTE
CM032588.1	110231	.	C	G	100	*	TYPE=SUBSTITUTE
CM032588.1	110237	.	G	C	100	*	TYPE=SUBSTITUTE
CM032588.1	110276	.	G	A	100	*	TYPE=SUBSTITUTE
CM032588.1	110282	.	G	A	100	*	TYPE=SUBSTITUTE
CM032588.1	110283	.	CTA	C	100	*	TYPE=DELETE
CM032588.1	110316	.	G	C	100	*	TYPE=SUBSTITUTE
CM032588.1	110359	.	C	T	100	*	TYPE=SUBSTITUTE
CM032588.1	110365	.	G	A	100	*	TYPE=SUBSTITUTE
CM032588.1	110419	.	A	G	100	*	TYPE=SUBSTITUTE
CM032588.1	110425	.	T	C	100	*	TYPE=SUBSTITUTE
CM032588.1	110440	.	G	A	100	*	TYPE=SUBSTITUTE
CM032588.1	110524	.	A	G	100	*	TYPE=SUBSTITUTE
CM032588.1	110609	.	T	C	100	*	TYPE=SUBSTITUTE
CM032588.1	110615	.	G	A	100	*	TYPE=SUBSTITUTE
CM032588.1	110616	.	T	G	100	*	TYPE=SUBSTITUTE
CM032588.1	110648	.	A	G	100	*	TYPE=SUBSTITUTE
CM032588.1	110650	.	T	G	100	*	TYPE=SUBSTITUTE
CM032588.1	110663	.	C	G	100	*	TYPE=SUBSTITUTE
CM032588.1	110671	.	A	G	100	*	TYPE=SUBSTITUTE
CM032588.1	110673	.	A	G	100	*	TYPE=SUBSTITUTE
CM032588.1	110683	.	C	G	100	*	TYPE=SUBSTITUTE
CM032588.1	110684	.	A	G	100	*	TYPE=SUBSTITUTE
CM032588.1	110691	.	A	G	100	*	TYPE=SUBSTITUTE
CM032588.1	110693	.	A	G	100	*	TYPE=SUBSTITUTE
CM032588.1	110694	.	A	G	100	*	TYPE=SUBSTITUTE
CM032588.1	110716	.	G	a	100	*	TYPE=SUBSTITUTE
CM032588.1	110719	.	TG	T	100	*	TYPE=DELETE
CM032588.1	110774	.	G	T	100	*	TYPE=SUBSTITUTE
CM032588.1	110800	.	G	A	100	*	TYPE=SUBSTITUTE
CM032588.1	110807	.	G	A	100	*	TYPE=SUBSTITUTE

CM032588.1	110933	.	T	C	100	*	TYPE=SUBSTITUTE
CM032588.1	110940	.	C	T	100	*	TYPE=SUBSTITUTE
CM032588.1	111025	.	A	G	100	*	TYPE=SUBSTITUTE
CM032588.1	111059	.	C	T	100	*	TYPE=SUBSTITUTE
CM032588.1	111106	.	G	A	100	*	TYPE=SUBSTITUTE
CM032588.1	111111	.	C	T	100	*	TYPE=SUBSTITUTE
CM032588.1	111115	.	G	C	100	*	TYPE=SUBSTITUTE
CM032588.1	111137	.	C	T	100	*	TYPE=SUBSTITUTE
CM032588.1	111138	.	G	C	100	*	TYPE=SUBSTITUTE
CM032588.1	111141	.	A	AGCCC	100	*	TYPE=INSERT
CM032588.1	111142	.	A	G	100	*	TYPE=SUBSTITUTE
CM032588.1	111151	.	A	G	100	*	TYPE=SUBSTITUTE
CM032588.1	111173	.	A	G	100	*	TYPE=SUBSTITUTE
CM032588.1	111190	.	C	T	100	*	TYPE=SUBSTITUTE
CM032588.1	111191	.	T	A	100	*	TYPE=SUBSTITUTE
CM032588.1	111192	.	C	A	100	*	TYPE=SUBSTITUTE
CM032588.1	111196	.	C	G	100	*	TYPE=SUBSTITUTE
CM032588.1	111201	.	G	C	100	*	TYPE=SUBSTITUTE
CM032588.1	111202	.	A	G	100	*	TYPE=SUBSTITUTE
CM032588.1	111203	.	G	T	100	*	TYPE=SUBSTITUTE
CM032588.1	111205	.	T	C	100	*	TYPE=SUBSTITUTE
CM032588.1	111210	.	A	C	100	*	TYPE=SUBSTITUTE
CM032588.1	111216	.	C	A	100	*	TYPE=SUBSTITUTE
CM032588.1	111235	.	A	C	100	*	TYPE=SUBSTITUTE
CM032588.1	111238	.	C	T	100	*	TYPE=SUBSTITUTE
CM032588.1	111240	.	A	G	100	*	TYPE=SUBSTITUTE
CM032588.1	111243	.	C	G	100	*	TYPE=SUBSTITUTE
CM032588.1	111246	.	C	A	100	*	TYPE=SUBSTITUTE
CM032588.1	111252	.	A	C	100	*	TYPE=SUBSTITUTE
CM032588.1	111256	.	C	A	100	*	TYPE=SUBSTITUTE
CM032588.1	111259	.	C	T	100	*	TYPE=SUBSTITUTE
CM032588.1	111260	.	C	T	100	*	TYPE=SUBSTITUTE
CM032588.1	111268	.	C	T	100	*	TYPE=SUBSTITUTE
CM032588.1	111269	.	C	T	100	*	TYPE=SUBSTITUTE
CM032588.1	111275	.	G	A	100	*	TYPE=SUBSTITUTE
CM032588.1	111276	.	C	T	100	*	TYPE=SUBSTITUTE
CM032588.1	111291	.	G	c	100	*	TYPE=SUBSTITUTE
CM032588.1	111292	.	G	t	100	*	TYPE=SUBSTITUTE
CM032588.1	111293	.	G	t	100	*	TYPE=SUBSTITUTE
CM032588.1	111295	.	T	TTTGCG	100	*	TYPE=INSERT
CM032588.1	111304	.	A	G	100	*	TYPE=SUBSTITUTE
CM032588.1	111309	.	A	C	100	*	TYPE=SUBSTITUTE
CM032588.1	111310	.	G	T	100	*	TYPE=SUBSTITUTE
CM032588.1	111314	.	T	G	100	*	TYPE=SUBSTITUTE
CM032588.1	111318	.	T	C	100	*	TYPE=SUBSTITUTE
CM032588.1	111321	.	A	G	100	*	TYPE=SUBSTITUTE
CM032588.1	111336	.	T	C	100	*	TYPE=SUBSTITUTE
CM032588.1	111345	.	A	G	100	*	TYPE=SUBSTITUTE
CM032588.1	111349	.	C	T	100	*	TYPE=SUBSTITUTE
CM032588.1	111350	.	C	G	100	*	TYPE=SUBSTITUTE
CM032588.1	111351	.	G	C	100	*	TYPE=SUBSTITUTE
CM032588.1	111356	.	T	C	100	*	TYPE=SUBSTITUTE
CM032588.1	111361	.	TTGGTGTTCC	T	100	*	TYPE=DELETE
CM032588.1	111373	.	G	A	100	*	TYPE=SUBSTITUTE
CM032588.1	111378	.	C	T	100	*	TYPE=SUBSTITUTE
CM032588.1	111392	.	C	T	100	*	TYPE=SUBSTITUTE
CM032588.1	111396	.	C	T	100	*	TYPE=SUBSTITUTE
CM032588.1	111418	.	T	C	100	*	TYPE=SUBSTITUTE
CM032588.1	111430	.	C	T	100	*	TYPE=SUBSTITUTE
CM032588.1	111438	.	T	G	100	*	TYPE=SUBSTITUTE
CM032588.1	111445	.	A	C	100	*	TYPE=SUBSTITUTE
CM032588.1	111457	.	G	A	100	*	TYPE=SUBSTITUTE
CM032588.1	111466	.	C	A	100	*	TYPE=SUBSTITUTE
CM032588.1	111469	.	T	C	100	*	TYPE=SUBSTITUTE

CM032588.1	111472	.	A	G	100	*	TYPE=SUBSTITUTE
CM032588.1	111481	.	A	AT	100	*	TYPE=INSERT
CM032588.1	111489	.	A	G	100	*	TYPE=SUBSTITUTE
CM032588.1	111491	.	G	A	100	*	TYPE=SUBSTITUTE
CM032588.1	111493	.	A	G	100	*	TYPE=SUBSTITUTE
CM032588.1	111495	.	A	T	100	*	TYPE=SUBSTITUTE
CM032588.1	111499	.	A	G	100	*	TYPE=SUBSTITUTE
CM032588.1	111504	.	G	A	100	*	TYPE=SUBSTITUTE
CM032588.1	111523	.	C	CC	100	*	TYPE=INSERT
CM032588.1	111553	.	A	G	100	*	TYPE=SUBSTITUTE
CM032588.1	111565	.	A	C	100	*	TYPE=SUBSTITUTE
CM032588.1	111570	.	A	G	100	*	TYPE=SUBSTITUTE
CM032588.1	111585	.	C	G	100	*	TYPE=SUBSTITUTE
CM032588.1	111589	.	C	T	100	*	TYPE=SUBSTITUTE
CM032588.1	111603	.	A	G	100	*	TYPE=SUBSTITUTE
CM032588.1	111605	.	A	T	100	*	TYPE=SUBSTITUTE
CM032588.1	111610	.	G	C	100	*	TYPE=SUBSTITUTE
CM032588.1	111629	.	G	A	100	*	TYPE=SUBSTITUTE
CM032588.1	111646	.	T	a	100	*	TYPE=SUBSTITUTE
CM032588.1	111657	.	T	G	100	*	TYPE=SUBSTITUTE
CM032588.1	111662	.	G	a	100	*	TYPE=SUBSTITUTE
CM032588.1	111666	.	G	a	100	*	TYPE=SUBSTITUTE
CM032588.1	111668	.	a	aag	100	*	TYPE=INSERT
CM032588.1	111671	.	T	a	100	*	TYPE=SUBSTITUTE
CM032588.1	111681	.	G	T	100	*	TYPE=SUBSTITUTE
CM032588.1	111689	.	G	A	100	*	TYPE=SUBSTITUTE
CM032588.1	111707	.	C	G	100	*	TYPE=SUBSTITUTE
CM032588.1	111708	.	G	A	100	*	TYPE=SUBSTITUTE
CM032588.1	111711	.	G	A	100	*	TYPE=SUBSTITUTE
CM032588.1	111713	.	C	A	100	*	TYPE=SUBSTITUTE
CM032588.1	111715	.	A	G	100	*	TYPE=SUBSTITUTE
CM032588.1	111730	.	G	A	100	*	TYPE=SUBSTITUTE
CM032588.1	111733	.	A	G	100	*	TYPE=SUBSTITUTE
CM032588.1	111735	.	C	T	100	*	TYPE=SUBSTITUTE
CM032588.1	111736	.	A	G	100	*	TYPE=SUBSTITUTE
CM032588.1	111747	.	T	C	100	*	TYPE=SUBSTITUTE
CM032588.1	111749	.	G	A	100	*	TYPE=SUBSTITUTE
CM032588.1	111754	.	T	A	100	*	TYPE=SUBSTITUTE
CM032588.1	111759	.	T	C	100	*	TYPE=SUBSTITUTE
CM032588.1	111761	.	T	A	100	*	TYPE=SUBSTITUTE
CM032588.1	111762	.	T	C	100	*	TYPE=SUBSTITUTE
CM032588.1	111763	.	A	G	100	*	TYPE=SUBSTITUTE
CM032588.1	111765	.	G	T	100	*	TYPE=SUBSTITUTE
CM032588.1	111777	.	C	CTCCTG	100	*	TYPE=INSERT
CM032588.1	111778	.	C	T	100	*	TYPE=SUBSTITUTE
CM032588.1	111780	.	G	A	100	*	TYPE=SUBSTITUTE
CM032588.1	111787	.	T	C	100	*	TYPE=SUBSTITUTE
CM032588.1	111791	.	T	G	100	*	TYPE=SUBSTITUTE
CM032588.1	111803	.	G	A	100	*	TYPE=SUBSTITUTE
CM032588.1	111809	.	A	G	100	*	TYPE=SUBSTITUTE
CM032588.1	111831	.	A	G	100	*	TYPE=SUBSTITUTE
CM032588.1	111832	.	CT	C	100	*	TYPE=DELETE
CM032588.1	111845	.	T	C	100	*	TYPE=SUBSTITUTE
CM032588.1	111854	.	C	A	100	*	TYPE=SUBSTITUTE
CM032588.1	111860	.	C	T	100	*	TYPE=SUBSTITUTE
CM032588.1	111861	.	C	T	100	*	TYPE=SUBSTITUTE
CM032588.1	111862	.	C	T	100	*	TYPE=SUBSTITUTE
CM032588.1	111870	.	A	AG	100	*	TYPE=INSERT
CM032588.1	111877	.	A	G	100	*	TYPE=SUBSTITUTE
CM032588.1	111879	.	G	C	100	*	TYPE=SUBSTITUTE
CM032588.1	111882	.	C	A	100	*	TYPE=SUBSTITUTE
CM032588.1	111890	.	A	ATAC	100	*	TYPE=INSERT
CM032588.1	111894	.	C	T	100	*	TYPE=SUBSTITUTE
CM032588.1	111897	.	A	G	100	*	TYPE=SUBSTITUTE

CM032588.1	111901	.	C	A	100	*	TYPE=SUBSTITUTE
CM032588.1	111913	.	T	TGTTCAAA	100	*	TYPE=INSERT
CM032588.1	111914	.	A	C	100	*	TYPE=SUBSTITUTE
CM032588.1	111919	.	G	A	100	*	TYPE=SUBSTITUTE
CM032588.1	111931	.	C	A	100	*	TYPE=SUBSTITUTE
CM032588.1	111935	.	T	C	100	*	TYPE=SUBSTITUTE
CM032588.1	111953	.	A	G	100	*	TYPE=SUBSTITUTE
CM032588.1	111982	.	A	T	100	*	TYPE=SUBSTITUTE
CM032588.1	111983	.	C	T	100	*	TYPE=SUBSTITUTE
CM032588.1	111987	.	A	G	100	*	TYPE=SUBSTITUTE
CM032588.1	111990	.	G	T	100	*	TYPE=SUBSTITUTE
CM032588.1	111999	.	T	G	100	*	TYPE=SUBSTITUTE
CM032588.1	112013	.	CAG	C	100	*	TYPE=DELETE
CM032588.1	112018	.	C	G	100	*	TYPE=SUBSTITUTE
CM032588.1	112019	.	C	T	100	*	TYPE=SUBSTITUTE
CM032588.1	112020	.	C	T	100	*	TYPE=SUBSTITUTE
CM032588.1	112021	.	A	T	100	*	TYPE=SUBSTITUTE
CM032588.1	112024	.	T	G	100	*	TYPE=SUBSTITUTE
CM032588.1	112025	.	C	A	100	*	TYPE=SUBSTITUTE
CM032588.1	112028	.	A	G	100	*	TYPE=SUBSTITUTE
CM032588.1	112029	.	G	T	100	*	TYPE=SUBSTITUTE
CM032588.1	112031	.	G	A	100	*	TYPE=SUBSTITUTE
CM032588.1	112033	.	T	C	100	*	TYPE=SUBSTITUTE
CM032588.1	112036	.	T	A	100	*	TYPE=SUBSTITUTE
CM032588.1	112044	.	A	G	100	*	TYPE=SUBSTITUTE
CM032588.1	112046	.	A	G	100	*	TYPE=SUBSTITUTE
CM032588.1	112054	.	A	G	100	*	TYPE=SUBSTITUTE
CM032588.1	112063	.	G	A	100	*	TYPE=SUBSTITUTE
CM032588.1	112074	.	GAGAGTT	G	100	*	TYPE=DELETE
CM032588.1	112085	.	G	A	100	*	TYPE=SUBSTITUTE
CM032588.1	112091	.	A	C	100	*	TYPE=SUBSTITUTE
CM032588.1	112098	.	A	AG	100	*	TYPE=INSERT
CM032588.1	112099	.	T	G	100	*	TYPE=SUBSTITUTE
CM032588.1	112100	.	T	C	100	*	TYPE=SUBSTITUTE
CM032588.1	112131	.	TACT	T	100	*	TYPE=DELETE
CM032588.1	112161	.	C	CAAC	100	*	TYPE=INSERT
CM032588.1	112162	.	C	A	100	*	TYPE=SUBSTITUTE
CM032588.1	112169	.	A	T	100	*	TYPE=SUBSTITUTE
CM032588.1	112174	.	A	C	100	*	TYPE=SUBSTITUTE
CM032588.1	112178	.	T	C	100	*	TYPE=SUBSTITUTE
CM032588.1	112179	.	G	C	100	*	TYPE=SUBSTITUTE
CM032588.1	112182	.	C	T	100	*	TYPE=SUBSTITUTE
CM032588.1	112192	.	G	A	100	*	TYPE=SUBSTITUTE
CM032588.1	112193	.	A	G	100	*	TYPE=SUBSTITUTE
CM032588.1	112200	.	G	A	100	*	TYPE=SUBSTITUTE
CM032588.1	112204	.	T	C	100	*	TYPE=SUBSTITUTE
CM032588.1	112208	.	A	C	100	*	TYPE=SUBSTITUTE
CM032588.1	112209	.	T	C	100	*	TYPE=SUBSTITUTE
CM032588.1	112220	.	T	A	100	*	TYPE=SUBSTITUTE
CM032588.1	112225	.	T	C	100	*	TYPE=SUBSTITUTE
CM032588.1	112226	.	G	T	100	*	TYPE=SUBSTITUTE
CM032588.1	112227	.	A	G	100	*	TYPE=SUBSTITUTE
CM032588.1	112230	.	C	T	100	*	TYPE=SUBSTITUTE
CM032588.1	112234	.	C	T	100	*	TYPE=SUBSTITUTE
CM032588.1	112240	.	G	T	100	*	TYPE=SUBSTITUTE
CM032588.1	112245	.	A	C	100	*	TYPE=SUBSTITUTE
CM032588.1	112252	.	A	C	100	*	TYPE=SUBSTITUTE
CM032588.1	112254	.	C	A	100	*	TYPE=SUBSTITUTE
CM032588.1	112263	.	C	T	100	*	TYPE=SUBSTITUTE
CM032588.1	112264	.	T	G	100	*	TYPE=SUBSTITUTE
CM032588.1	112267	.	A	G	100	*	TYPE=SUBSTITUTE
CM032588.1	112301	.	T	G	100	*	TYPE=SUBSTITUTE
CM032588.1	112311	.	C	A	100	*	TYPE=SUBSTITUTE
CM032588.1	112337	.	C	T	100	*	TYPE=SUBSTITUTE

CM032588.1	112342	.	G	A	100	*	TYPE=SUBSTITUTE
CM032588.1	112349	.	C	T	100	*	TYPE=SUBSTITUTE
CM032588.1	112397	.	A	G	100	*	TYPE=SUBSTITUTE
CM032588.1	112416	.	A	T	100	*	TYPE=SUBSTITUTE
CM032588.1	112503	.	A	G	100	*	TYPE=SUBSTITUTE
CM032588.1	112514	.	A	G	100	*	TYPE=SUBSTITUTE
CM032588.1	112562	.	G	A	100	*	TYPE=SUBSTITUTE
CM032588.1	112592	.	T	C	100	*	TYPE=SUBSTITUTE
CM032588.1	112676	.	T	C	100	*	TYPE=SUBSTITUTE
CM032588.1	112679	.	C	G	100	*	TYPE=SUBSTITUTE
CM032588.1	112682	.	A	G	100	*	TYPE=SUBSTITUTE
CM032588.1	112700	.	A	G	100	*	TYPE=SUBSTITUTE
CM032588.1	112703	.	T	C	100	*	TYPE=SUBSTITUTE
CM032588.1	112706	.	C	T	100	*	TYPE=SUBSTITUTE
CM032588.1	112734	.	T	C	100	*	TYPE=SUBSTITUTE
CM032588.1	112745	.	A	C	100	*	TYPE=SUBSTITUTE
CM032588.1	112766	.	T	C	100	*	TYPE=SUBSTITUTE
CM032588.1	112772	.	G	A	100	*	TYPE=SUBSTITUTE
CM032588.1	112784	.	C	A	100	*	TYPE=SUBSTITUTE
CM032588.1	112802	.	C	T	100	*	TYPE=SUBSTITUTE
CM032588.1	112805	.	G	A	100	*	TYPE=SUBSTITUTE
CM032588.1	112815	.	T	G	100	*	TYPE=SUBSTITUTE
CM032588.1	112817	.	C	T	100	*	TYPE=SUBSTITUTE
CM032588.1	112819	.	T	C	100	*	TYPE=SUBSTITUTE
CM032588.1	112821	.	T	C	100	*	TYPE=SUBSTITUTE
CM032588.1	112822	.	A	G	100	*	TYPE=SUBSTITUTE
CM032588.1	112824	.	C	T	100	*	TYPE=SUBSTITUTE
CM032588.1	112826	.	C	G	100	*	TYPE=SUBSTITUTE
CM032588.1	112839	.	T	C	100	*	TYPE=SUBSTITUTE
CM032588.1	112853	.	T	C	100	*	TYPE=SUBSTITUTE
CM032588.1	112857	.	T	C	100	*	TYPE=SUBSTITUTE
CM032588.1	112860	.	C	T	100	*	TYPE=SUBSTITUTE
CM032588.1	112865	.	GTA	G	100	*	TYPE=DELETE
CM032588.1	112888	.	C	T	100	*	TYPE=SUBSTITUTE
CM032588.1	112912	.	T	C	100	*	TYPE=SUBSTITUTE
CM032588.1	112934	.	C	T	100	*	TYPE=SUBSTITUTE
CM032588.1	112936	.	G	A	100	*	TYPE=SUBSTITUTE
CM032588.1	112957	.	C	T	100	*	TYPE=SUBSTITUTE
CM032588.1	112960	.	T	C	100	*	TYPE=SUBSTITUTE
CM032588.1	112999	.	G	C	100	*	TYPE=SUBSTITUTE
CM032588.1	113032	.	T	C	100	*	TYPE=SUBSTITUTE
CM032588.1	113047	.	G	T	100	*	TYPE=SUBSTITUTE
CM032588.1	113053	.	T	C	100	*	TYPE=SUBSTITUTE
CM032588.1	113059	.	T	C	100	*	TYPE=SUBSTITUTE
CM032588.1	113089	.	C	T	100	*	TYPE=SUBSTITUTE
CM032588.1	113095	.	G	A	100	*	TYPE=SUBSTITUTE
CM032588.1	113275	.	T	C	100	*	TYPE=SUBSTITUTE
CM032588.1	113281	.	G	A	100	*	TYPE=SUBSTITUTE
CM032588.1	113287	.	C	T	100	*	TYPE=SUBSTITUTE
CM032588.1	113293	.	G	A	100	*	TYPE=SUBSTITUTE
CM032588.1	113295	.	C	A	100	*	TYPE=SUBSTITUTE
CM032588.1	113296	.	A	G	100	*	TYPE=SUBSTITUTE
CM032588.1	113305	.	A	G	100	*	TYPE=SUBSTITUTE
CM032588.1	113323	.	C	T	100	*	TYPE=SUBSTITUTE
CM032588.1	113332	.	T	G	100	*	TYPE=SUBSTITUTE
CM032588.1	113339	.	T	C	100	*	TYPE=SUBSTITUTE
CM032588.1	113341	.	G	A	100	*	TYPE=SUBSTITUTE
CM032588.1	113352	.	G	C	100	*	TYPE=SUBSTITUTE
CM032588.1	113356	.	T	C	100	*	TYPE=SUBSTITUTE
CM032588.1	113410	.	G	A	100	*	TYPE=SUBSTITUTE
CM032588.1	113427	.	C	A	100	*	TYPE=SUBSTITUTE
CM032588.1	113452	.	T	A	100	*	TYPE=SUBSTITUTE
CM032588.1	113455	.	C	T	100	*	TYPE=SUBSTITUTE
CM032588.1	113487	.	G	A	100	*	TYPE=SUBSTITUTE

CM032588.1	113533	.	C	T	100	*	TYPE=SUBSTITUTE
CM032588.1	113581	.	A	G	100	*	TYPE=SUBSTITUTE
CM032588.1	113590	.	T	C	100	*	TYPE=SUBSTITUTE
CM032588.1	113593	.	C	T	100	*	TYPE=SUBSTITUTE
CM032588.1	113599	.	C	T	100	*	TYPE=SUBSTITUTE
CM032588.1	113626	.	T	C	100	*	TYPE=SUBSTITUTE
CM032588.1	113629	.	T	C	100	*	TYPE=SUBSTITUTE
CM032588.1	113631	.	A	G	100	*	TYPE=SUBSTITUTE
CM032588.1	113662	.	C	T	100	*	TYPE=SUBSTITUTE
CM032588.1	113695	.	A	G	100	*	TYPE=SUBSTITUTE
CM032588.1	113698	.	G	C	100	*	TYPE=SUBSTITUTE
CM032588.1	113700	.	A	G	100	*	TYPE=SUBSTITUTE
CM032588.1	113767	.	A	G	100	*	TYPE=SUBSTITUTE
CM032588.1	113785	.	C	G	100	*	TYPE=SUBSTITUTE
CM032588.1	113824	.	A	G	100	*	TYPE=SUBSTITUTE
CM032588.1	113830	.	T	C	100	*	TYPE=SUBSTITUTE
CM032588.1	113854	.	G	C	100	*	TYPE=SUBSTITUTE
CM032588.1	113860	.	T	C	100	*	TYPE=SUBSTITUTE
CM032588.1	113887	.	G	A	100	*	TYPE=SUBSTITUTE
CM032588.1	113893	.	G	A	100	*	TYPE=SUBSTITUTE
CM032588.1	113928	.	C	T	100	*	TYPE=SUBSTITUTE
CM032588.1	113967	.	C	T	100	*	TYPE=SUBSTITUTE
CM032588.1	113978	.	C	T	100	*	TYPE=SUBSTITUTE
CM032588.1	113987	.	A	G	100	*	TYPE=SUBSTITUTE
CM032588.1	113992	.	A	G	100	*	TYPE=SUBSTITUTE
CM032588.1	114005	.	T	C	100	*	TYPE=SUBSTITUTE
CM032588.1	114015	.	C	g	100	*	TYPE=SUBSTITUTE
CM032588.1	114023	.	c	ca	100	*	TYPE=INSERT
CM032588.1	114032	.	G	A	100	*	TYPE=SUBSTITUTE
CM032588.1	114036	.	G	T	100	*	TYPE=SUBSTITUTE
CM032588.1	114051	.	C	T	100	*	TYPE=SUBSTITUTE
CM032588.1	114064	.	T	C	100	*	TYPE=SUBSTITUTE
CM032588.1	114067	.	T	C	100	*	TYPE=SUBSTITUTE
CM032588.1	114080	.	C	G	100	*	TYPE=SUBSTITUTE
CM032588.1	114090	.	T	C	100	*	TYPE=SUBSTITUTE
CM032588.1	114098	.	G	A	100	*	TYPE=SUBSTITUTE
CM032588.1	114105	.	A	G	100	*	TYPE=SUBSTITUTE
CM032588.1	114113	.	G	t	100	*	TYPE=SUBSTITUTE
CM032588.1	114122	.	C	t	100	*	TYPE=SUBSTITUTE
CM032588.1	114131	.	T	C	100	*	TYPE=SUBSTITUTE
CM032588.1	114135	.	T	C	100	*	TYPE=SUBSTITUTE
CM032588.1	114142	.	C	T	100	*	TYPE=SUBSTITUTE
CM032588.1	114159	.	A	T	100	*	TYPE=SUBSTITUTE
CM032588.1	114187	.	A	G	100	*	TYPE=SUBSTITUTE
CM032588.1	114190	.	T	A	100	*	TYPE=SUBSTITUTE
CM032588.1	114191	.	T	A	100	*	TYPE=SUBSTITUTE
CM032588.1	114202	.	T	C	100	*	TYPE=SUBSTITUTE
CM032588.1	114218	.	T	G	100	*	TYPE=SUBSTITUTE
CM032588.1	114219	.	T	C	100	*	TYPE=SUBSTITUTE
CM032588.1	114227	.	A	G	100	*	TYPE=SUBSTITUTE
CM032588.1	114233	.	A	G	100	*	TYPE=SUBSTITUTE
CM032588.1	114236	.	T	C	100	*	TYPE=SUBSTITUTE
CM032588.1	114281	.	A	G	100	*	TYPE=SUBSTITUTE
CM032588.1	114297	.	A	G	100	*	TYPE=SUBSTITUTE
CM032588.1	114305	.	TTAGGCC	T	100	*	TYPE=DELETE
CM032588.1	114313	.	A	G	100	*	TYPE=SUBSTITUTE
CM032588.1	114332	.	C	T	100	*	TYPE=SUBSTITUTE
CM032588.1	114342	.	A	C	100	*	TYPE=SUBSTITUTE
CM032588.1	114349	.	C	T	100	*	TYPE=SUBSTITUTE
CM032588.1	114355	.	A	G	100	*	TYPE=SUBSTITUTE
CM032588.1	114368	.	G	A	100	*	TYPE=SUBSTITUTE
CM032588.1	114383	.	a	atct	100	*	TYPE=INSERT
CM032588.1	114386	.	C	t	100	*	TYPE=SUBSTITUTE
CM032588.1	114390	.	C	a	100	*	TYPE=SUBSTITUTE

CM032588.1	114405	.	C	T	100	*	TYPE=SUBSTITUTE
CM032588.1	114417	.	C	T	100	*	TYPE=SUBSTITUTE
CM032588.1	114425	.	A	C	100	*	TYPE=SUBSTITUTE
CM032588.1	114429	.	T	A	100	*	TYPE=SUBSTITUTE
CM032588.1	114440	.	C	G	100	*	TYPE=SUBSTITUTE
CM032588.1	114445	.	A	G	100	*	TYPE=SUBSTITUTE
CM032588.1	114450	.	C	A	100	*	TYPE=SUBSTITUTE
CM032588.1	114451	.	A	G	100	*	TYPE=SUBSTITUTE
CM032588.1	114455	.	A	G	100	*	TYPE=SUBSTITUTE
CM032588.1	114458	.	TC	T	100	*	TYPE=DELETE
CM032588.1	114461	.	A	C	100	*	TYPE=SUBSTITUTE
CM032588.1	114465	.	A	T	100	*	TYPE=SUBSTITUTE
CM032588.1	114467	.	G	A	100	*	TYPE=SUBSTITUTE
CM032588.1	114476	.	GGC	G	100	*	TYPE=DELETE
CM032588.1	114483	.	A	G	100	*	TYPE=SUBSTITUTE
CM032588.1	114484	.	T	TA	100	*	TYPE=INSERT
CM032588.1	114489	.	A	T	100	*	TYPE=SUBSTITUTE
CM032588.1	114498	.	C	t	100	*	TYPE=SUBSTITUTE
CM032588.1	114510	.	G	a	100	*	TYPE=SUBSTITUTE
CM032588.1	114519	.	G	A	100	*	TYPE=SUBSTITUTE
CM032588.1	114523	.	G	A	100	*	TYPE=SUBSTITUTE
CM032588.1	114527	.	A	G	100	*	TYPE=SUBSTITUTE
CM032588.1	114539	.	G	A	100	*	TYPE=SUBSTITUTE
CM032588.1	114558	.	C	A	100	*	TYPE=SUBSTITUTE
CM032588.1	114581	.	C	G	100	*	TYPE=SUBSTITUTE
CM032588.1	114591	.	T	C	100	*	TYPE=SUBSTITUTE
CM032588.1	114592	.	G	A	100	*	TYPE=SUBSTITUTE
CM032588.1	114601	.	T	A	100	*	TYPE=SUBSTITUTE
CM032588.1	114618	.	A	G	100	*	TYPE=SUBSTITUTE
CM032588.1	114629	.	T	C	100	*	TYPE=SUBSTITUTE
CM032588.1	114656	.	A	G	100	*	TYPE=SUBSTITUTE
CM032588.1	114663	.	C	T	100	*	TYPE=SUBSTITUTE
CM032588.1	114665	.	G	A	100	*	TYPE=SUBSTITUTE
CM032588.1	114705	.	A	G	100	*	TYPE=SUBSTITUTE
CM032588.1	114708	.	G	T	100	*	TYPE=SUBSTITUTE
CM032588.1	114710	.	G	A	100	*	TYPE=SUBSTITUTE
CM032588.1	114733	.	C	G	100	*	TYPE=SUBSTITUTE
CM032588.1	114802	.	A	C	100	*	TYPE=SUBSTITUTE
CM032588.1	114825	.	T	c	100	*	TYPE=SUBSTITUTE
CM032588.1	114830	.	T	c	100	*	TYPE=SUBSTITUTE
CM032588.1	114836	.	T	c	100	*	TYPE=SUBSTITUTE
CM032588.1	114842	.	c	ct	100	*	TYPE=INSERT
CM032588.1	114843	.	A	c	100	*	TYPE=SUBSTITUTE
CM032588.1	114850	.	A	G	100	*	TYPE=SUBSTITUTE
CM032588.1	114853	.	G	A	100	*	TYPE=SUBSTITUTE
CM032588.1	114855	.	G	A	100	*	TYPE=SUBSTITUTE
CM032588.1	114863	.	T	C	100	*	TYPE=SUBSTITUTE
CM032588.1	114868	.	T	A	100	*	TYPE=SUBSTITUTE
CM032588.1	114869	.	C	T	100	*	TYPE=SUBSTITUTE
CM032588.1	114911	.	A	C	100	*	TYPE=SUBSTITUTE
CM032588.1	114914	.	C	T	100	*	TYPE=SUBSTITUTE
CM032588.1	114935	.	A	C	100	*	TYPE=SUBSTITUTE
CM032588.1	114953	.	G	A	100	*	TYPE=SUBSTITUTE
CM032588.1	114973	.	C	G	100	*	TYPE=SUBSTITUTE
CM032588.1	114985	.	C	G	100	*	TYPE=SUBSTITUTE
CM032588.1	114991	.	T	C	100	*	TYPE=SUBSTITUTE
CM032588.1	114995	.	A	C	100	*	TYPE=SUBSTITUTE
CM032588.1	115008	.	G	A	100	*	TYPE=SUBSTITUTE
CM032588.1	115026	.	C	T	100	*	TYPE=SUBSTITUTE
CM032588.1	115046	.	G	A	100	*	TYPE=SUBSTITUTE
CM032588.1	115071	.	T	A	100	*	TYPE=SUBSTITUTE
CM032588.1	115078	.	A	AG	100	*	TYPE=INSERT
CM032588.1	115107	.	G	C	100	*	TYPE=SUBSTITUTE
CM032588.1	115111	.	C	A	100	*	TYPE=SUBSTITUTE

CM032588.1	115177	.	C	t	100	*	TYPE=SUBSTITUTE
CM032588.1	115180	.	G	a	100	*	TYPE=SUBSTITUTE
CM032588.1	115192	.	A	c	100	*	TYPE=SUBSTITUTE
CM032588.1	115214	.	C	T	100	*	TYPE=SUBSTITUTE
CM032588.1	115222	.	C	G	100	*	TYPE=SUBSTITUTE
CM032588.1	115225	.	C	T	100	*	TYPE=SUBSTITUTE
CM032588.1	115258	.	G	T	100	*	TYPE=SUBSTITUTE
CM032588.1	115260	.	C	T	100	*	TYPE=SUBSTITUTE
CM032588.1	115267	.	C	T	100	*	TYPE=SUBSTITUTE
CM032588.1	115270	.	T	C	100	*	TYPE=SUBSTITUTE
CM032588.1	115276	.	C	T	100	*	TYPE=SUBSTITUTE
CM032588.1	115281	.	G	A	100	*	TYPE=SUBSTITUTE
CM032588.1	115294	.	T	C	100	*	TYPE=SUBSTITUTE
CM032588.1	115378	.	A	G	100	*	TYPE=SUBSTITUTE
CM032588.1	115398	.	G	GT	100	*	TYPE=INSERT
CM032588.1	115401	.	A	C	100	*	TYPE=SUBSTITUTE
CM032588.1	115449	.	C	T	100	*	TYPE=SUBSTITUTE
CM032588.1	115470	.	G	A	100	*	TYPE=SUBSTITUTE
CM032588.1	115506	.	C	T	100	*	TYPE=SUBSTITUTE
CM032588.1	115527	.	T	C	100	*	TYPE=SUBSTITUTE
CM032588.1	115535	.	A	T	100	*	TYPE=SUBSTITUTE
CM032588.1	115578	.	C	G	100	*	TYPE=SUBSTITUTE
CM032588.1	115605	.	C	T	100	*	TYPE=SUBSTITUTE
CM032588.1	115613	.	G	A	100	*	TYPE=SUBSTITUTE
CM032588.1	115638	.	G	C	100	*	TYPE=SUBSTITUTE
CM032588.1	115662	.	A	T	100	*	TYPE=SUBSTITUTE
CM032588.1	115667	.	G	C	100	*	TYPE=SUBSTITUTE
CM032588.1	115680	.	C	T	100	*	TYPE=SUBSTITUTE
CM032588.1	115682	.	A	G	100	*	TYPE=SUBSTITUTE
CM032588.1	115693	.	C	T	100	*	TYPE=SUBSTITUTE
CM032588.1	115695	.	C	A	100	*	TYPE=SUBSTITUTE
CM032588.1	115737	.	C	T	100	*	TYPE=SUBSTITUTE
CM032588.1	115761	.	A	G	100	*	TYPE=SUBSTITUTE
CM032588.1	115771	.	G	A	100	*	TYPE=SUBSTITUTE
CM032588.1	115776	.	G	A	100	*	TYPE=SUBSTITUTE
CM032588.1	115779	.	A	C	100	*	TYPE=SUBSTITUTE
CM032588.1	115794	.	T	C	100	*	TYPE=SUBSTITUTE
CM032588.1	115827	.	T	C	100	*	TYPE=SUBSTITUTE
CM032588.1	115890	.	G	T	100	*	TYPE=SUBSTITUTE
CM032588.1	115978	.	T	G	100	*	TYPE=SUBSTITUTE
CM032588.1	115995	.	C	T	100	*	TYPE=SUBSTITUTE
CM032588.1	116004	.	A	G	100	*	TYPE=SUBSTITUTE
CM032588.1	116029	.	G	A	100	*	TYPE=SUBSTITUTE
CM032588.1	116070	.	C	T	100	*	TYPE=SUBSTITUTE
CM032588.1	116091	.	G	T	100	*	TYPE=SUBSTITUTE
CM032588.1	116130	.	G	A	100	*	TYPE=SUBSTITUTE
CM032588.1	116146	.	G	A	100	*	TYPE=SUBSTITUTE
CM032588.1	116163	.	C	T	100	*	TYPE=SUBSTITUTE
CM032588.1	116187	.	A	G	100	*	TYPE=SUBSTITUTE
CM032588.1	116234	.	G	T	100	*	TYPE=SUBSTITUTE
CM032588.1	116235	.	C	T	100	*	TYPE=SUBSTITUTE
CM032588.1	116248	.	A	G	100	*	TYPE=SUBSTITUTE
CM032588.1	116253	.	G	A	100	*	TYPE=SUBSTITUTE
CM032588.1	116262	.	C	T	100	*	TYPE=SUBSTITUTE
CM032588.1	116275	.	T	TC	100	*	TYPE=INSERT
CM032588.1	116279	.	T	TGAGA	100	*	TYPE=INSERT
CM032588.1	116281	.	T	G	100	*	TYPE=SUBSTITUTE
CM032588.1	116285	.	A	T	100	*	TYPE=SUBSTITUTE
CM032588.1	116289	.	A	G	100	*	TYPE=SUBSTITUTE
CM032588.1	116295	.	G	A	100	*	TYPE=SUBSTITUTE
CM032588.1	116297	.	G	GC	100	*	TYPE=INSERT
CM032588.1	116306	.	G	A	100	*	TYPE=SUBSTITUTE
CM032588.1	116323	.	A	G	100	*	TYPE=SUBSTITUTE
CM032588.1	116337	.	T	A	100	*	TYPE=SUBSTITUTE

CM032588.1	116340	.	T	C	100	*	TYPE=SUBSTITUTE
CM032588.1	116341	.	T	G	100	*	TYPE=SUBSTITUTE
CM032588.1	116356	.	T	G	100	*	TYPE=SUBSTITUTE
CM032588.1	116361	.	A	G	100	*	TYPE=SUBSTITUTE
CM032588.1	116377	.	A	G	100	*	TYPE=SUBSTITUTE
CM032588.1	116378	.	C	T	100	*	TYPE=SUBSTITUTE
CM032588.1	116379	.	C	A	100	*	TYPE=SUBSTITUTE
CM032588.1	116393	.	T	C	100	*	TYPE=SUBSTITUTE
CM032588.1	116412	.	T	C	100	*	TYPE=SUBSTITUTE
CM032588.1	116416	.	AG	A	100	*	TYPE=DELETE
CM032588.1	116422	.	C	T	100	*	TYPE=SUBSTITUTE
CM032588.1	116426	.	G	C	100	*	TYPE=SUBSTITUTE
CM032588.1	116428	.	T	C	100	*	TYPE=SUBSTITUTE
CM032588.1	116447	.	A	C	100	*	TYPE=SUBSTITUTE
CM032588.1	116480	.	T	G	100	*	TYPE=SUBSTITUTE
CM032588.1	116496	.	T	C	100	*	TYPE=SUBSTITUTE
CM032588.1	116502	.	G	A	100	*	TYPE=SUBSTITUTE
CM032588.1	116510	.	C	T	100	*	TYPE=SUBSTITUTE
CM032588.1	116519	.	G	T	100	*	TYPE=SUBSTITUTE
CM032588.1	116522	.	C	T	100	*	TYPE=SUBSTITUTE
CM032588.1	116529	.	A	T	100	*	TYPE=SUBSTITUTE
CM032588.1	116530	.	T	A	100	*	TYPE=SUBSTITUTE
CM032588.1	116532	.	A	T	100	*	TYPE=SUBSTITUTE
CM032588.1	116541	.	G	A	100	*	TYPE=SUBSTITUTE
CM032588.1	116542	.	T	C	100	*	TYPE=SUBSTITUTE
CM032588.1	116551	.	G	A	100	*	TYPE=SUBSTITUTE
CM032588.1	116554	.	A	T	100	*	TYPE=SUBSTITUTE
CM032588.1	116609	.	T	G	100	*	TYPE=SUBSTITUTE
CM032588.1	116611	.	T	C	100	*	TYPE=SUBSTITUTE
CM032588.1	116612	.	G	A	100	*	TYPE=SUBSTITUTE
CM032588.1	116644	.	A	G	100	*	TYPE=SUBSTITUTE
CM032588.1	116666	.	G	A	100	*	TYPE=SUBSTITUTE
CM032588.1	116670	.	A	C	100	*	TYPE=SUBSTITUTE
CM032588.1	116672	.	G	A	100	*	TYPE=SUBSTITUTE
CM032588.1	116680	.	G	C	100	*	TYPE=SUBSTITUTE
CM032588.1	116687	.	G	A	100	*	TYPE=SUBSTITUTE
CM032588.1	116703	.	C	G	100	*	TYPE=SUBSTITUTE
CM032588.1	116746	.	A	G	100	*	TYPE=SUBSTITUTE
CM032588.1	116787	.	C	t	100	*	TYPE=SUBSTITUTE
CM032588.1	116788	.	A	t	100	*	TYPE=SUBSTITUTE
CM032588.1	116799	.	T	A	100	*	TYPE=SUBSTITUTE
CM032588.1	116816	.	A	C	100	*	TYPE=SUBSTITUTE
CM032588.1	116857	.	C	T	100	*	TYPE=SUBSTITUTE
CM032588.1	116860	.	C	G	100	*	TYPE=SUBSTITUTE
CM032588.1	116874	.	A	T	100	*	TYPE=SUBSTITUTE
CM032588.1	116879	.	A	G	100	*	TYPE=SUBSTITUTE
CM032588.1	116885	.	A	G	100	*	TYPE=SUBSTITUTE
CM032588.1	116893	.	T	C	100	*	TYPE=SUBSTITUTE
CM032588.1	116903	.	ACGT	A	100	*	TYPE=DELETE
CM032588.1	116921	.	A	G	100	*	TYPE=SUBSTITUTE
CM032588.1	116937	.	G	A	100	*	TYPE=SUBSTITUTE
CM032588.1	116941	.	G	C	100	*	TYPE=SUBSTITUTE
CM032588.1	116962	.	G	A	100	*	TYPE=SUBSTITUTE
CM032588.1	116978	.	T	C	100	*	TYPE=SUBSTITUTE
CM032588.1	116985	.	CG	C	100	*	TYPE=DELETE
CM032588.1	116988	.	G	C	100	*	TYPE=SUBSTITUTE
CM032588.1	116995	.	G	C	100	*	TYPE=SUBSTITUTE
CM032588.1	117005	.	G	A	100	*	TYPE=SUBSTITUTE
CM032588.1	117008	.	T	TA	100	*	TYPE=INSERT
CM032588.1	117010	.	G	A	100	*	TYPE=SUBSTITUTE
CM032588.1	117026	.	C	T	100	*	TYPE=SUBSTITUTE
CM032588.1	117030	.	C	T	100	*	TYPE=SUBSTITUTE
CM032588.1	117031	.	G	C	100	*	TYPE=SUBSTITUTE
CM032588.1	117056	.	C	A	100	*	TYPE=SUBSTITUTE

CM032588.1	117063	.	T	C	100	*	TYPE=SUBSTITUTE
CM032588.1	117120	.	G	A	100	*	TYPE=SUBSTITUTE
CM032588.1	117122	.	C	T	100	*	TYPE=SUBSTITUTE
CM032588.1	117130	.	T	C	100	*	TYPE=SUBSTITUTE
CM032588.1	117131	.	GA	G	100	*	TYPE=DELETE
CM032588.1	117139	.	T	C	100	*	TYPE=SUBSTITUTE
CM032588.1	117143	.	A	G	100	*	TYPE=SUBSTITUTE
CM032588.1	117144	.	C	T	100	*	TYPE=SUBSTITUTE
CM032588.1	117146	.	AG	A	100	*	TYPE=DELETE
CM032588.1	117164	.	T	G	100	*	TYPE=SUBSTITUTE
CM032588.1	117166	.	TAG	T	100	*	TYPE=DELETE
CM032588.1	117171	.	G	A	100	*	TYPE=SUBSTITUTE
CM032588.1	117183	.	C	T	100	*	TYPE=SUBSTITUTE
CM032588.1	117198	.	C	T	100	*	TYPE=SUBSTITUTE
CM032588.1	117201	.	A	G	100	*	TYPE=SUBSTITUTE
CM032588.1	117204	.	C	T	100	*	TYPE=SUBSTITUTE
CM032588.1	117254	.	A	G	100	*	TYPE=SUBSTITUTE
CM032588.1	117258	.	G	T	100	*	TYPE=SUBSTITUTE
CM032588.1	117294	.	C	T	100	*	TYPE=SUBSTITUTE
CM032588.1	117327	.	C	T	100	*	TYPE=SUBSTITUTE
CM032588.1	117366	.	C	T	100	*	TYPE=SUBSTITUTE
CM032588.1	117369	.	G	C	100	*	TYPE=SUBSTITUTE
CM032588.1	117408	.	T	C	100	*	TYPE=SUBSTITUTE
CM032588.1	117477	.	A	G	100	*	TYPE=SUBSTITUTE
CM032588.1	117498	.	G	T	100	*	TYPE=SUBSTITUTE
CM032588.1	117511	.	A	T	100	*	TYPE=SUBSTITUTE
CM032588.1	117519	.	C	T	100	*	TYPE=SUBSTITUTE
CM032588.1	117534	.	A	G	100	*	TYPE=SUBSTITUTE
CM032588.1	117537	.	T	C	100	*	TYPE=SUBSTITUTE
CM032588.1	117572	.	T	C	100	*	TYPE=SUBSTITUTE
CM032588.1	117585	.	G	A	100	*	TYPE=SUBSTITUTE
CM032588.1	117586	.	T	A	100	*	TYPE=SUBSTITUTE
CM032588.1	117588	.	C	T	100	*	TYPE=SUBSTITUTE
CM032588.1	117639	.	C	A	100	*	TYPE=SUBSTITUTE
CM032588.1	117640	.	C	T	100	*	TYPE=SUBSTITUTE
CM032588.1	117657	.	G	A	100	*	TYPE=SUBSTITUTE
CM032588.1	117659	.	A	C	100	*	TYPE=SUBSTITUTE
CM032588.1	117662	.	T	G	100	*	TYPE=SUBSTITUTE
CM032588.1	117679	.	C	T	100	*	TYPE=SUBSTITUTE
CM032588.1	117687	.	C	G	100	*	TYPE=SUBSTITUTE
CM032588.1	117702	.	G	A	100	*	TYPE=SUBSTITUTE
CM032588.1	117705	.	A	G	100	*	TYPE=SUBSTITUTE
CM032588.1	117717	.	C	T	100	*	TYPE=SUBSTITUTE
CM032588.1	117726	.	T	A	100	*	TYPE=SUBSTITUTE
CM032588.1	117728	.	G	C	100	*	TYPE=SUBSTITUTE
CM032588.1	117738	.	C	T	100	*	TYPE=SUBSTITUTE
CM032588.1	117810	.	T	A	100	*	TYPE=SUBSTITUTE
CM032588.1	117822	.	G	A	100	*	TYPE=SUBSTITUTE
CM032588.1	117834	.	C	T	100	*	TYPE=SUBSTITUTE
CM032588.1	117838	.	T	C	100	*	TYPE=SUBSTITUTE
CM032588.1	117844	.	C	T	100	*	TYPE=SUBSTITUTE
CM032588.1	117849	.	T	G	100	*	TYPE=SUBSTITUTE
CM032588.1	117850	.	G	A	100	*	TYPE=SUBSTITUTE
CM032588.1	117855	.	G	A	100	*	TYPE=SUBSTITUTE
CM032588.1	117872	.	C	t	100	*	TYPE=SUBSTITUTE
CM032588.1	117873	.	C	t	100	*	TYPE=SUBSTITUTE
CM032588.1	117878	.	CT	C	100	*	TYPE=DELETE
CM032588.1	117949	.	T	G	100	*	TYPE=SUBSTITUTE
CM032588.1	117961	.	C	T	100	*	TYPE=SUBSTITUTE
CM032588.1	117963	.	C	T	100	*	TYPE=SUBSTITUTE
CM032588.1	117965	.	G	GTTGGTAT	100	*	TYPE=INSERT
CM032588.1	117968	.	C	CGA	100	*	TYPE=INSERT
CM032588.1	117976	.	A	C	100	*	TYPE=SUBSTITUTE
CM032588.1	117988	.	G	A	100	*	TYPE=SUBSTITUTE

CM032588.1	117989	.	A	G	100	*	TYPE=SUBSTITUTE
CM032588.1	117993	.	G	A	100	*	TYPE=SUBSTITUTE
CM032588.1	118001	.	T	A	100	*	TYPE=SUBSTITUTE
CM032588.1	118003	.	A	T	100	*	TYPE=SUBSTITUTE
CM032588.1	118024	.	T	C	100	*	TYPE=SUBSTITUTE
CM032588.1	118025	.	T	C	100	*	TYPE=SUBSTITUTE
CM032588.1	118030	.	G	C	100	*	TYPE=SUBSTITUTE
CM032588.1	118034	.	G	A	100	*	TYPE=SUBSTITUTE
CM032588.1	118066	.	C	A	100	*	TYPE=SUBSTITUTE
CM032588.1	118070	.	T	G	100	*	TYPE=SUBSTITUTE
CM032588.1	118082	.	A	G	100	*	TYPE=SUBSTITUTE
CM032588.1	118108	.	G	A	100	*	TYPE=SUBSTITUTE
CM032588.1	118125	.	A	C	100	*	TYPE=SUBSTITUTE
CM032588.1	118139	.	A	G	100	*	TYPE=SUBSTITUTE
CM032588.1	118140	.	G	A	100	*	TYPE=SUBSTITUTE
CM032588.1	118143	.	A	G	100	*	TYPE=SUBSTITUTE
CM032588.1	118147	.	C	T	100	*	TYPE=SUBSTITUTE
CM032588.1	118152	.	G	A	100	*	TYPE=SUBSTITUTE
CM032588.1	118153	.	T	A	100	*	TYPE=SUBSTITUTE
CM032588.1	118158	.	A	G	100	*	TYPE=SUBSTITUTE
CM032588.1	118161	.	A	G	100	*	TYPE=SUBSTITUTE
CM032588.1	118171	.	G	C	100	*	TYPE=SUBSTITUTE
CM032588.1	118175	.	T	C	100	*	TYPE=SUBSTITUTE
CM032588.1	118185	.	A	G	100	*	TYPE=SUBSTITUTE
CM032588.1	118190	.	A	T	100	*	TYPE=SUBSTITUTE
CM032588.1	118191	.	T	C	100	*	TYPE=SUBSTITUTE
CM032588.1	118195	.	T	G	100	*	TYPE=SUBSTITUTE
CM032588.1	118197	.	G	GT	100	*	TYPE=INSERT
CM032588.1	118206	.	G	A	100	*	TYPE=SUBSTITUTE
CM032588.1	118214	.	T	C	100	*	TYPE=SUBSTITUTE
CM032588.1	118220	.	AATTG	A	100	*	TYPE=DELETE
CM032588.1	118228	.	G	A	100	*	TYPE=SUBSTITUTE
CM032588.1	118231	.	T	G	100	*	TYPE=SUBSTITUTE
CM032588.1	118238	.	A	G	100	*	TYPE=SUBSTITUTE
CM032588.1	118252	.	C	G	100	*	TYPE=SUBSTITUTE
CM032588.1	118265	.	A	G	100	*	TYPE=SUBSTITUTE
CM032588.1	118270	.	CAAAA	C	100	*	TYPE=DELETE
CM032588.1	118327	.	T	C	100	*	TYPE=SUBSTITUTE
CM032588.1	118328	.	G	A	100	*	TYPE=SUBSTITUTE
CM032588.1	118335	.	T	A	100	*	TYPE=SUBSTITUTE
CM032588.1	118338	.	T	C	100	*	TYPE=SUBSTITUTE
CM032588.1	118344	.	G	A	100	*	TYPE=SUBSTITUTE
CM032588.1	118345	.	C	A	100	*	TYPE=SUBSTITUTE
CM032588.1	118347	.	G	A	100	*	TYPE=SUBSTITUTE
CM032588.1	118352	.	G	A	100	*	TYPE=SUBSTITUTE
CM032588.1	118358	.	G	A	100	*	TYPE=SUBSTITUTE
CM032588.1	118360	.	G	T	100	*	TYPE=SUBSTITUTE
CM032588.1	118367	.	G	t	100	*	TYPE=SUBSTITUTE
CM032588.1	118373	.	T	c	100	*	TYPE=SUBSTITUTE
CM032588.1	118383	.	C	a	100	*	TYPE=SUBSTITUTE
CM032588.1	118404	.	A	G	100	*	TYPE=SUBSTITUTE
CM032588.1	118414	.	T	G	100	*	TYPE=SUBSTITUTE
CM032588.1	118418	.	T	C	100	*	TYPE=SUBSTITUTE
CM032588.1	118431	.	T	C	100	*	TYPE=SUBSTITUTE
CM032588.1	118432	.	A	G	100	*	TYPE=SUBSTITUTE
CM032588.1	118437	.	G	C	100	*	TYPE=SUBSTITUTE
CM032588.1	118456	.	A	T	100	*	TYPE=SUBSTITUTE
CM032588.1	118464	.	A	T	100	*	TYPE=SUBSTITUTE
CM032588.1	118481	.	G	T	100	*	TYPE=SUBSTITUTE
CM032588.1	118482	.	T	C	100	*	TYPE=SUBSTITUTE
CM032588.1	118492	.	C	G	100	*	TYPE=SUBSTITUTE
CM032588.1	118498	.	TG	T	100	*	TYPE=DELETE
CM032588.1	118515	.	A	G	100	*	TYPE=SUBSTITUTE
CM032588.1	118518	.	A	C	100	*	TYPE=SUBSTITUTE

CM032588.1	118520	.	C	A	100	*	TYPE=SUBSTITUTE
CM032588.1	118522	.	C	T	100	*	TYPE=SUBSTITUTE
CM032588.1	118531	.	A	C	100	*	TYPE=SUBSTITUTE
CM032588.1	118561	.	A	G	100	*	TYPE=SUBSTITUTE
CM032588.1	118562	.	G	A	100	*	TYPE=SUBSTITUTE
CM032588.1	118565	.	A	T	100	*	TYPE=SUBSTITUTE
CM032588.1	118588	.	G	A	100	*	TYPE=SUBSTITUTE
CM032588.1	118591	.	T	G	100	*	TYPE=SUBSTITUTE
CM032588.1	118606	.	A	G	100	*	TYPE=SUBSTITUTE
CM032588.1	118612	.	C	G	100	*	TYPE=SUBSTITUTE
CM032588.1	118626	.	G	C	100	*	TYPE=SUBSTITUTE
CM032588.1	118627	.	AG	A	100	*	TYPE=DELETE
CM032588.1	118654	.					

TTATAGTCAATATTCCCAAAGGCTGGGGCGTAGCCTTCCTCTGACCTAGCTATTGCAGCTTTACTGACCCTCTTGATAAT
AAATATAACCTC T 100 * TYPE=DELETE

CM032588.1	118746	.	T	A	100	*	TYPE=SUBSTITUTE
CM032588.1	118797	.	G	A	100	*	TYPE=SUBSTITUTE
CM032588.1	118859	.	A	C	100	*	TYPE=SUBSTITUTE
CM032588.1	118861	.	A	G	100	*	TYPE=SUBSTITUTE
CM032588.1	118946	.	T	C	100	*	TYPE=SUBSTITUTE
CM032588.1	119014	.	T	C	100	*	TYPE=SUBSTITUTE
CM032588.1	119084	.	G	C	100	*	TYPE=SUBSTITUTE
CM032588.1	119189	.	T	C	100	*	TYPE=SUBSTITUTE
CM032588.1	119195	.	T	C	100	*	TYPE=SUBSTITUTE
CM032588.1	119213	.	C	T	100	*	TYPE=SUBSTITUTE
CM032588.1	119225	.	A	G	100	*	TYPE=SUBSTITUTE
CM032588.1	119270	.	C	T	100	*	TYPE=SUBSTITUTE
CM032588.1	119281	.	T	C	100	*	TYPE=SUBSTITUTE
CM032588.1	119293	.	G	T	100	*	TYPE=SUBSTITUTE
CM032588.1	119311	.	T	C	100	*	TYPE=SUBSTITUTE
CM032588.1	119312	.	A	G	100	*	TYPE=SUBSTITUTE
CM032588.1	119336	.	A	G	100	*	TYPE=SUBSTITUTE
CM032588.1	119348	.	T	C	100	*	TYPE=SUBSTITUTE
CM032588.1	119357	.	A	G	100	*	TYPE=SUBSTITUTE
CM032588.1	119401	.	ACT	A	100	*	TYPE=DELETE
CM032588.1	119411	.	A	G	100	*	TYPE=SUBSTITUTE
CM032588.1	119412	.	T	C	100	*	TYPE=SUBSTITUTE
CM032588.1	119420	.	G	T	100	*	TYPE=SUBSTITUTE
CM032588.1	119425	.	G	A	100	*	TYPE=SUBSTITUTE
CM032588.1	119438	.	A	G	100	*	TYPE=SUBSTITUTE
CM032588.1	119439	.	T	A	100	*	TYPE=SUBSTITUTE
CM032588.1	119449	.	C	G	100	*	TYPE=SUBSTITUTE
CM032588.1	119476	.	G	C	100	*	TYPE=SUBSTITUTE
CM032588.1	119539	.	A	G	100	*	TYPE=SUBSTITUTE
CM032588.1	119590	.	C	T	100	*	TYPE=SUBSTITUTE
CM032588.1	119595	.	G	A	100	*	TYPE=SUBSTITUTE
CM032588.1	119611	.	T	C	100	*	TYPE=SUBSTITUTE
CM032588.1	119638	.	G	A	100	*	TYPE=SUBSTITUTE
CM032588.1	119698	.	A	T	100	*	TYPE=SUBSTITUTE
CM032588.1	119743	.	T	C	100	*	TYPE=SUBSTITUTE
CM032588.1	119749	.	A	G	100	*	TYPE=SUBSTITUTE
CM032588.1	119770	.	C	T	100	*	TYPE=SUBSTITUTE
CM032588.1	119773	.	A	C	100	*	TYPE=SUBSTITUTE
CM032588.1	119821	.	G	a	100	*	TYPE=SUBSTITUTE
CM032588.1	119847	.	TG	T	100	*	TYPE=DELETE
CM032588.1	119849	.	C	T	100	*	TYPE=SUBSTITUTE
CM032588.1	119855	.	C	T	100	*	TYPE=SUBSTITUTE
CM032588.1	119864	.	A	C	100	*	TYPE=SUBSTITUTE
CM032588.1	119871	.	G	A	100	*	TYPE=SUBSTITUTE
CM032588.1	119877	.	A	G	100	*	TYPE=SUBSTITUTE
CM032588.1	119878	.	T	C	100	*	TYPE=SUBSTITUTE
CM032588.1	119887	.	G	T	100	*	TYPE=SUBSTITUTE
CM032588.1	119892	.	C	A	100	*	TYPE=SUBSTITUTE

CM032588.1	119895	.	G	A	100	*	TYPE=SUBSTITUTE
CM032588.1	119911	.	A	G	100	*	TYPE=SUBSTITUTE
CM032588.1	119924	.	G	T	100	*	TYPE=SUBSTITUTE
CM032588.1	119972	.	C	T	100	*	TYPE=SUBSTITUTE
CM032588.1	119989	.	T	C	100	*	TYPE=SUBSTITUTE
CM032588.1	119995	.	T	C	100	*	TYPE=SUBSTITUTE
CM032588.1	120023	.	C	T	100	*	TYPE=SUBSTITUTE
CM032588.1	120038	.	T	C	100	*	TYPE=SUBSTITUTE
CM032588.1	120094	.	A	T	100	*	TYPE=SUBSTITUTE
CM032588.1	120098	.	T	C	100	*	TYPE=SUBSTITUTE
CM032588.1	120100	.	A	G	100	*	TYPE=SUBSTITUTE
CM032588.1	120109	.	C	T	100	*	TYPE=SUBSTITUTE
CM032588.1	120114	.	C	T	100	*	TYPE=SUBSTITUTE
CM032588.1	120117	.	A	T	100	*	TYPE=SUBSTITUTE
CM032588.1	120122	.	G	C	100	*	TYPE=SUBSTITUTE
CM032588.1	120130	.	A	G	100	*	TYPE=SUBSTITUTE
CM032588.1	120131	.	C	T	100	*	TYPE=SUBSTITUTE
CM032588.1	120132	.	C	A	100	*	TYPE=SUBSTITUTE
CM032588.1	120138	.	T	C	100	*	TYPE=SUBSTITUTE
CM032588.1	120157	.	T	G	100	*	TYPE=SUBSTITUTE
CM032588.1	120161	.	G	A	100	*	TYPE=SUBSTITUTE
CM032588.1	120164	.	T	C	100	*	TYPE=SUBSTITUTE
CM032588.1	120171	.	T	C	100	*	TYPE=SUBSTITUTE
CM032588.1	120203	.	A	G	100	*	TYPE=SUBSTITUTE
CM032588.1	120238	.	A	G	100	*	TYPE=SUBSTITUTE
CM032588.1	120246	.	T	C	100	*	TYPE=SUBSTITUTE
CM032588.1	120293	.	T	C	100	*	TYPE=SUBSTITUTE
CM032588.1	120299	.	G	A	100	*	TYPE=SUBSTITUTE
CM032588.1	120305	.	T	G	100	*	TYPE=SUBSTITUTE
CM032588.1	120311	.	T	C	100	*	TYPE=SUBSTITUTE
CM032588.1	120330	.	G	A	100	*	TYPE=SUBSTITUTE
CM032588.1	120385	.	C	G	100	*	TYPE=SUBSTITUTE
CM032588.1	120418	.	T	C	100	*	TYPE=SUBSTITUTE
CM032588.1	120419	.	GA	G	100	*	TYPE=DELETE
CM032588.1	120421	.	C	G	100	*	TYPE=SUBSTITUTE
CM032588.1	120459	.	A	C	100	*	TYPE=SUBSTITUTE
CM032588.1	120463	.	C	T	100	*	TYPE=SUBSTITUTE
CM032588.1	120468	.	A	G	100	*	TYPE=SUBSTITUTE
CM032588.1	120469	.	G	C	100	*	TYPE=SUBSTITUTE
CM032588.1	120512	.	A	T	100	*	TYPE=SUBSTITUTE
CM032588.1	120563	.	G	C	100	*	TYPE=SUBSTITUTE
CM032588.1	120597	.	T	C	100	*	TYPE=SUBSTITUTE
CM032588.1	120600	.	A	G	100	*	TYPE=SUBSTITUTE
CM032588.1	120603	.	G	A	100	*	TYPE=SUBSTITUTE
CM032588.1	120620	.	A	G	100	*	TYPE=SUBSTITUTE
CM032588.1	120638	.	G	A	100	*	TYPE=SUBSTITUTE
CM032588.1	120669	.	T	G	100	*	TYPE=SUBSTITUTE
CM032588.1	120675	.	C	G	100	*	TYPE=SUBSTITUTE
CM032588.1	120719	.	C	A	100	*	TYPE=SUBSTITUTE
CM032588.1	120733	.	T	C	100	*	TYPE=SUBSTITUTE
CM032588.1	120740	.	A	G	100	*	TYPE=SUBSTITUTE
CM032588.1	120753	.	A	T	100	*	TYPE=SUBSTITUTE
CM032588.1	120812	.	C	T	100	*	TYPE=SUBSTITUTE
CM032588.1	120843	.	A	T	100	*	TYPE=SUBSTITUTE
CM032588.1	120861	.	G	A	100	*	TYPE=SUBSTITUTE
CM032588.1	120879	.	T	C	100	*	TYPE=SUBSTITUTE
CM032588.1	120896	.	G	A	100	*	TYPE=SUBSTITUTE
CM032588.1	120918	.	G	A	100	*	TYPE=SUBSTITUTE
CM032588.1	120921	.	T	C	100	*	TYPE=SUBSTITUTE
CM032588.1	120925	.	T	G	100	*	TYPE=SUBSTITUTE
CM032588.1	120930	.	G	C	100	*	TYPE=SUBSTITUTE
CM032588.1	120933	.	G	C	100	*	TYPE=SUBSTITUTE
CM032588.1	120940	.	T	C	100	*	TYPE=SUBSTITUTE
CM032588.1	120941	.	C	G	100	*	TYPE=SUBSTITUTE

CM032588.1	120943	.	G	A	100	*	TYPE=SUBSTITUTE
CM032588.1	120946	.	AT	A	100	*	TYPE=DELETE
CM032588.1	120957	.	A	T	100	*	TYPE=SUBSTITUTE
CM032588.1	120959	.	A	G	100	*	TYPE=SUBSTITUTE
CM032588.1	120961	.	C	T	100	*	TYPE=SUBSTITUTE
CM032588.1	120962	.	G	T	100	*	TYPE=SUBSTITUTE
CM032588.1	120969	.	G	A	100	*	TYPE=SUBSTITUTE
CM032588.1	120972	.	G	A	100	*	TYPE=SUBSTITUTE
CM032588.1	120973	.	T	A	100	*	TYPE=SUBSTITUTE
CM032588.1	120978	.	T	G	100	*	TYPE=SUBSTITUTE
CM032588.1	120983	.	C	G	100	*	TYPE=SUBSTITUTE
CM032588.1	121000	.	T	C	100	*	TYPE=SUBSTITUTE
CM032588.1	121003	.	A	C	100	*	TYPE=SUBSTITUTE
CM032588.1	121005	.	A	T	100	*	TYPE=SUBSTITUTE
CM032588.1	121006	.	A	G	100	*	TYPE=SUBSTITUTE
CM032588.1	121011	.	T	G	100	*	TYPE=SUBSTITUTE
CM032588.1	121014	.	C	T	100	*	TYPE=SUBSTITUTE
CM032588.1	121020	.	C	T	100	*	TYPE=SUBSTITUTE
CM032588.1	121022	.	G	A	100	*	TYPE=SUBSTITUTE
CM032588.1	121030	.	T	G	100	*	TYPE=SUBSTITUTE
CM032588.1	121033	.	G	A	100	*	TYPE=SUBSTITUTE
CM032588.1	121035	.	A	G	100	*	TYPE=SUBSTITUTE
CM032588.1	121038	.	A	G	100	*	TYPE=SUBSTITUTE
CM032588.1	121040	.	C	A	100	*	TYPE=SUBSTITUTE
CM032588.1	121041	.	A	G	100	*	TYPE=SUBSTITUTE
CM032588.1	121061	.	A	G	100	*	TYPE=SUBSTITUTE
CM032588.1	121066	.	G	A	100	*	TYPE=SUBSTITUTE
CM032588.1	121069	.	TCA	T	100	*	TYPE=DELETE
CM032588.1	121082	.	A	G	100	*	TYPE=SUBSTITUTE
CM032588.1	121089	.	A	C	100	*	TYPE=SUBSTITUTE
CM032588.1	121093	.	G	A	100	*	TYPE=SUBSTITUTE
CM032588.1	121095	.	T	G	100	*	TYPE=SUBSTITUTE
CM032588.1	121100	.	G	A	100	*	TYPE=SUBSTITUTE
CM032588.1	121103	.	C	T	100	*	TYPE=SUBSTITUTE
CM032588.1	121113	.	T	C	100	*	TYPE=SUBSTITUTE
CM032588.1	121117	.	C	T	100	*	TYPE=SUBSTITUTE
CM032588.1	121121	.	G	A	100	*	TYPE=SUBSTITUTE
CM032588.1	121128	.	C	A	100	*	TYPE=SUBSTITUTE
CM032588.1	121130	.	CG	C	100	*	TYPE=DELETE
CM032588.1	121132	.	A	C	100	*	TYPE=SUBSTITUTE
CM032588.1	121136	.	A	G	100	*	TYPE=SUBSTITUTE
CM032588.1	121138	.	A	G	100	*	TYPE=SUBSTITUTE
CM032588.1	121140	.	G	T	100	*	TYPE=SUBSTITUTE
CM032588.1	121141	.	T	G	100	*	TYPE=SUBSTITUTE
CM032588.1	121148	.	A	G	100	*	TYPE=SUBSTITUTE
CM032588.1	121151	.	G	A	100	*	TYPE=SUBSTITUTE
CM032588.1	121153	.	T	G	100	*	TYPE=SUBSTITUTE
CM032588.1	121154	.	G	A	100	*	TYPE=SUBSTITUTE
CM032588.1	121156	.	GA	G	100	*	TYPE=DELETE
CM032588.1	121161	.	T	C	100	*	TYPE=SUBSTITUTE
CM032588.1	121162	.	C	CT	100	*	TYPE=INSERT
CM032588.1	121163	.	A	T	100	*	TYPE=SUBSTITUTE
CM032588.1	121166	.	C	T	100	*	TYPE=SUBSTITUTE
CM032588.1	121167	.	C	G	100	*	TYPE=SUBSTITUTE
CM032588.1	121170	.	A	G	100	*	TYPE=SUBSTITUTE
CM032588.1	121173	.	A	G	100	*	TYPE=SUBSTITUTE
CM032588.1	121178	.	A	G	100	*	TYPE=SUBSTITUTE
CM032588.1	121179	.	CTCTA	C	100	*	TYPE=DELETE
CM032588.1	121185	.	G	A	100	*	TYPE=SUBSTITUTE
CM032588.1	121191	.	A	C	100	*	TYPE=SUBSTITUTE
CM032588.1	121209	.	C	T	100	*	TYPE=SUBSTITUTE
CM032588.1	121215	.	C	T	100	*	TYPE=SUBSTITUTE
CM032588.1	121218	.	A	T	100	*	TYPE=SUBSTITUTE
CM032588.1	121219	.	T	C	100	*	TYPE=SUBSTITUTE

```
CM032588.1  121220      .    T    C    100  *    TYPE=SUBSTITUTE
CM032588.1  121252      .    C    G    100  *    TYPE=SUBSTITUTE
CM032588.1  121256      .    A    G    100  *    TYPE=SUBSTITUTE
CM032588.1  121257      .    C    T    100  *    TYPE=SUBSTITUTE
CM032588.1  121270      .    C

CGTCCTTTAGTCAAGTCAAAGTGGTCTGTATCTGAAGTATCACGCTGCTTTTTCTTGTCGCAAAACATTCTAGCTGTCAT
GTACGCCGAATATAACTTG     100  *    TYPE=INSERT
CM032588.1  121279      .    C    G    100  *    TYPE=SUBSTITUTE
CM032588.1  121303      .    G    A    100  *    TYPE=SUBSTITUTE
CM032588.1  121310      .    T    TATGGAC   100  *    TYPE=INSERT
CM032588.1  121313      .    T    G    100  *    TYPE=SUBSTITUTE
CM032588.1  121318      .    G    A    100  *    TYPE=SUBSTITUTE
CM032588.1  121319      .    C    T    100  *    TYPE=SUBSTITUTE
CM032588.1  121327      .    G    T    100  *    TYPE=SUBSTITUTE
CM032588.1  121330      .    A    G    100  *    TYPE=SUBSTITUTE
CM032588.1  121334      .    C    T    100  *    TYPE=SUBSTITUTE
CM032588.1  121338      .    A    G    100  *    TYPE=SUBSTITUTE
CM032588.1  121343      .    C    T    100  *    TYPE=SUBSTITUTE
CM032588.1  121345      .    T    C    100  *    TYPE=SUBSTITUTE
CM032588.1  121347      .    T    C    100  *    TYPE=SUBSTITUTE
CM032588.1  121349      .    A    G    100  *    TYPE=SUBSTITUTE
CM032588.1  121350      .    G    A    100  *    TYPE=SUBSTITUTE
CM032588.1  121352      .    T    C    100  *    TYPE=SUBSTITUTE
CM032588.1  121356      .    A    C    100  *    TYPE=SUBSTITUTE
CM032588.1  121358      .    C    A    100  *    TYPE=SUBSTITUTE
CM032588.1  121362      .    A    AC   100  *    TYPE=INSERT
CM032588.1  121373      .    C    G    100  *    TYPE=SUBSTITUTE
CM032588.1  121377      .    G    A    100  *    TYPE=SUBSTITUTE
CM032588.1  121461      .    A    G    100  *    TYPE=SUBSTITUTE
CM032588.1  121487      .    A    G    100  *    TYPE=SUBSTITUTE
CM032588.1  121543      .    T    C    100  *    TYPE=SUBSTITUTE
CM032588.1  121564      .    A    G    100  *    TYPE=SUBSTITUTE
CM032588.1  121573      .    G    A    100  *    TYPE=SUBSTITUTE
CM032588.1  121600      .    C    T    100  *    TYPE=SUBSTITUTE
CM032588.1  121632      .    G    T    100  *    TYPE=SUBSTITUTE
CM032588.1  121640      .    T    A    100  *    TYPE=SUBSTITUTE
CM032588.1  121659      .    A    T    100  *    TYPE=SUBSTITUTE
CM032588.1  121660      .    T    A    100  *    TYPE=SUBSTITUTE
CM032588.1  121661      .    G    GCA  100  *    TYPE=INSERT
CM032588.1  121677      .    T    C    100  *    TYPE=SUBSTITUTE
CM032588.1  121687      .    G    A    100  *    TYPE=SUBSTITUTE
CM032588.1  121692      .    T    C    100  *    TYPE=SUBSTITUTE
CM032588.1  121697      .    A    G    100  *    TYPE=SUBSTITUTE
CM032588.1  121787      .    G    A    100  *    TYPE=SUBSTITUTE
CM032588.1  121799      .    T    C    100  *    TYPE=SUBSTITUTE
CM032588.1  121892      .    T    C    100  *    TYPE=SUBSTITUTE
CM032588.1  121906      .    C    T    100  *    TYPE=SUBSTITUTE
CM032588.1  121937      .    A    G    100  *    TYPE=SUBSTITUTE
CM032588.1  121972      .    C    T    100  *    TYPE=SUBSTITUTE
CM032588.1  121981      .    A    G    100  *    TYPE=SUBSTITUTE
CM032588.1  122003      .    A    G    100  *    TYPE=SUBSTITUTE
CM032588.1  122094      .    A    G    100  *    TYPE=SUBSTITUTE
CM032588.1  122099      .    C    T    100  *    TYPE=SUBSTITUTE
CM032588.1  122105      .    G    A    100  *    TYPE=SUBSTITUTE
CM032588.1  122162      .    T    C    100  *    TYPE=SUBSTITUTE
CM032588.1  122243      .    C    T    100  *    TYPE=SUBSTITUTE
CM032588.1  122255      .    T    C    100  *    TYPE=SUBSTITUTE
CM032588.1  122308      .    T    C    100  *    TYPE=SUBSTITUTE
CM032588.1  122309      .    G    A    100  *    TYPE=SUBSTITUTE
CM032588.1  122312      .    T    C    100  *    TYPE=SUBSTITUTE
CM032588.1  122333      .    A    G    100  *    TYPE=SUBSTITUTE
CM032588.1  122360      .    A    G    100  *    TYPE=SUBSTITUTE
CM032588.1  122426      .    G    A    100  *    TYPE=SUBSTITUTE
```

CM032588.1	122429	.	C	T	100	*	TYPE=SUBSTITUTE
CM032588.1	122437	.	T	C	100	*	TYPE=SUBSTITUTE
CM032588.1	122447	.	G	A	100	*	TYPE=SUBSTITUTE
CM032588.1	122461	.	T	TN	100	*	TYPE=INSERT
CM032588.1	122469	.	AC	A	100	*	TYPE=DELETE
CM032588.1	122501	.	C	T	100	*	TYPE=SUBSTITUTE
CM032588.1	122517	.	G	A	100	*	TYPE=SUBSTITUTE
CM032588.1	122555	.	C	G	100	*	TYPE=SUBSTITUTE
CM032588.1	122582	.	A	G	100	*	TYPE=SUBSTITUTE
CM032588.1	122589	.	GC	G	100	*	TYPE=DELETE
CM032588.1	122605	.	T	A	100	*	TYPE=SUBSTITUTE
CM032588.1	122630	.	A	G	100	*	TYPE=SUBSTITUTE
CM032588.1	122648	.	C	T	100	*	TYPE=SUBSTITUTE
CM032588.1	122754	.	G	A	100	*	TYPE=SUBSTITUTE
CM032588.1	122757	.	A	T	100	*	TYPE=SUBSTITUTE
CM032588.1	122790	.	C	T	100	*	TYPE=SUBSTITUTE
CM032588.1	122823	.	T	C	100	*	TYPE=SUBSTITUTE
CM032588.1	122856	.	A	G	100	*	TYPE=SUBSTITUTE
CM032588.1	122919	.	T	A	100	*	TYPE=SUBSTITUTE
CM032588.1	122928	.	G	A	100	*	TYPE=SUBSTITUTE
CM032588.1	122945	.	A	G	100	*	TYPE=SUBSTITUTE
CM032588.1	122972	.	A	G	100	*	TYPE=SUBSTITUTE
CM032588.1	123012	.	C	T	100	*	TYPE=SUBSTITUTE
CM032588.1	123034	.	G	A	100	*	TYPE=SUBSTITUTE
CM032588.1	123057	.	A	C	100	*	TYPE=SUBSTITUTE
CM032588.1	123153	.	G	A	100	*	TYPE=SUBSTITUTE
CM032588.1	123389	.	C	T	100	*	TYPE=SUBSTITUTE
CM032588.1	123406	.	A	G	100	*	TYPE=SUBSTITUTE
CM032588.1	123547	.	C	G	100	*	TYPE=SUBSTITUTE
CM032588.1	123552	.	G	A	100	*	TYPE=SUBSTITUTE
CM032588.1	123570	.	A	G	100	*	TYPE=SUBSTITUTE
CM032588.1	123575	.	A	G	100	*	TYPE=SUBSTITUTE
CM032588.1	123607	.	C	G	100	*	TYPE=SUBSTITUTE
CM032588.1	123624	.	A	G	100	*	TYPE=SUBSTITUTE
CM032588.1	123628	.	A	G	100	*	TYPE=SUBSTITUTE
CM032588.1	123631	.	G	C	100	*	TYPE=SUBSTITUTE
CM032588.1	123660	.	T	G	100	*	TYPE=SUBSTITUTE
CM032588.1	123662	.	C	CAGGTGATGGCATA	100	*	TYPE=INSERT
CM032588.1	123665	.	T	C	100	*	TYPE=SUBSTITUTE
CM032588.1	123673	.	C	G	100	*	TYPE=SUBSTITUTE
CM032588.1	123732	.	A	T	100	*	TYPE=SUBSTITUTE
CM032588.1	123733	.	A	T	100	*	TYPE=SUBSTITUTE
CM032588.1	123768	.	CG	C	100	*	TYPE=DELETE
CM032588.1	123788	.	G	A	100	*	TYPE=SUBSTITUTE
CM032588.1	123794	.	AT	A	100	*	TYPE=DELETE
CM032588.1	123825	.	C	A	100	*	TYPE=SUBSTITUTE
CM032588.1	124140	.	A	G	100	*	TYPE=SUBSTITUTE
CM032588.1	124199	.	G	A	100	*	TYPE=SUBSTITUTE
CM032588.1	124245	.	T	A	100	*	TYPE=SUBSTITUTE
CM032588.1	124257	.	T	G	100	*	TYPE=SUBSTITUTE
CM032588.1	124322	.	T	A	100	*	TYPE=SUBSTITUTE
CM032588.1	124339	.	C	A	100	*	TYPE=SUBSTITUTE
CM032588.1	124344	.	T	A	100	*	TYPE=SUBSTITUTE
CM032588.1	124345	.	C	T	100	*	TYPE=SUBSTITUTE
CM032588.1	124387	.	C	G	100	*	TYPE=SUBSTITUTE
CM032588.1	125023	.	C	T	100	*	TYPE=SUBSTITUTE
CM032588.1	125078	.	A	G	100	*	TYPE=SUBSTITUTE
CM032588.1	125081	.	T	A	100	*	TYPE=SUBSTITUTE
CM032588.1	125210	.	C	T	100	*	TYPE=SUBSTITUTE
CM032588.1	125306	.	G	A	100	*	TYPE=SUBSTITUTE
CM032588.1	125404	.	T	C	100	*	TYPE=SUBSTITUTE
CM032588.1	125634	.	C	T	100	*	TYPE=SUBSTITUTE
CM032588.1	126053	.	T	C	100	*	TYPE=SUBSTITUTE
CM032588.1	126152	.	C	t	100	*	TYPE=SUBSTITUTE

CM032588.1	126211	.	A	T	100	*	TYPE=SUBSTITUTE
CM032588.1	126284	.	T	c	100	*	TYPE=SUBSTITUTE
CM032588.1	126292	.	G	a	100	*	TYPE=SUBSTITUTE
CM032588.1	126398	.	T	G	100	*	TYPE=SUBSTITUTE
CM032588.1	126414	.	T	C	100	*	TYPE=SUBSTITUTE
CM032588.1	126430	.	G	A	100	*	TYPE=SUBSTITUTE
CM032588.1	126432	.	A	T	100	*	TYPE=SUBSTITUTE
CM032588.1	126450	.	T	C	100	*	TYPE=SUBSTITUTE
CM032588.1	126452	.	A	T	100	*	TYPE=SUBSTITUTE
CM032588.1	126480	.	T	C	100	*	TYPE=SUBSTITUTE
CM032588.1	126497	.	C	T	100	*	TYPE=SUBSTITUTE
CM032588.1	126498	.	C	G	100	*	TYPE=SUBSTITUTE
CM032588.1	126640	.	G	A	100	*	TYPE=SUBSTITUTE
CM032588.1	126654	.	T	C	100	*	TYPE=SUBSTITUTE
CM032588.1	126756	.	T	C	100	*	TYPE=SUBSTITUTE
CM032588.1	126781	.	A	C	100	*	TYPE=SUBSTITUTE
CM032588.1	126869	.	A	T	100	*	TYPE=SUBSTITUTE
CM032588.1	126895	.	G	C	100	*	TYPE=SUBSTITUTE
CM032588.1	126896	.	C	T	100	*	TYPE=SUBSTITUTE
CM032588.1	126902	.	T	G	100	*	TYPE=SUBSTITUTE
CM032588.1	126903	.	C	T	100	*	TYPE=SUBSTITUTE
CM032588.1	126989	.	C	T	100	*	TYPE=SUBSTITUTE
CM032588.1	126998	.	T	C	100	*	TYPE=SUBSTITUTE
CM032588.1	127007	.	G	A	100	*	TYPE=SUBSTITUTE
CM032588.1	127091	.	G	T	100	*	TYPE=SUBSTITUTE
CM032588.1	127136	.	C	T	100	*	TYPE=SUBSTITUTE
CM032588.1	127265	.	A	T	100	*	TYPE=SUBSTITUTE
CM032588.1	127272	.	G	T	100	*	TYPE=SUBSTITUTE
CM032588.1	127292	.	C	G	100	*	TYPE=SUBSTITUTE
CM032588.1	127301	.	C	T	100	*	TYPE=SUBSTITUTE
CM032588.1	127334	.	T	C	100	*	TYPE=SUBSTITUTE
CM032588.1	127335	.	T	C	100	*	TYPE=SUBSTITUTE
CM032588.1	127343	.	T	C	100	*	TYPE=SUBSTITUTE
CM032588.1	127349	.	A	G	100	*	TYPE=SUBSTITUTE
CM032588.1	127352	.	G	A	100	*	TYPE=SUBSTITUTE
CM032588.1	127406	.	A	G	100	*	TYPE=SUBSTITUTE
CM032588.1	127451	.	C	T	100	*	TYPE=SUBSTITUTE
CM032588.1	127482	.	A	G	100	*	TYPE=SUBSTITUTE
CM032588.1	127487	.	T	C	100	*	TYPE=SUBSTITUTE
CM032588.1	127488	.	G	C	100	*	TYPE=SUBSTITUTE
CM032588.1	127493	.	T	C	100	*	TYPE=SUBSTITUTE
CM032588.1	127514	.	T	c	100	*	TYPE=SUBSTITUTE
CM032588.1	127549	.	C	T	100	*	TYPE=SUBSTITUTE
CM032588.1	127553	.	A	G	100	*	TYPE=SUBSTITUTE
CM032588.1	127562	.	T	C	100	*	TYPE=SUBSTITUTE
CM032588.1	127567	.	G	A	100	*	TYPE=SUBSTITUTE
CM032588.1	127570	.	G	T	100	*	TYPE=SUBSTITUTE
CM032588.1	127571	.	A	G	100	*	TYPE=SUBSTITUTE
CM032588.1	127580	.	T	A	100	*	TYPE=SUBSTITUTE
CM032588.1	127584	.	A	G	100	*	TYPE=SUBSTITUTE
CM032588.1	127616	.	T	C	100	*	TYPE=SUBSTITUTE
CM032588.1	127640	.	A	G	100	*	TYPE=SUBSTITUTE
CM032588.1	127658	.	T	C	100	*	TYPE=SUBSTITUTE
CM032588.1	127673	.	A	G	100	*	TYPE=SUBSTITUTE
CM032588.1	127678	.	T	C	100	*	TYPE=SUBSTITUTE
CM032588.1	127733	.	G	A	100	*	TYPE=SUBSTITUTE
CM032588.1	127762	.	T	G	100	*	TYPE=SUBSTITUTE
CM032588.1	127787	.	A	G	100	*	TYPE=SUBSTITUTE
CM032588.1	127847	.	T	G	100	*	TYPE=SUBSTITUTE
CM032588.1	127880	.	C	T	100	*	TYPE=SUBSTITUTE
CM032588.1	127909	.	T	C	100	*	TYPE=SUBSTITUTE
CM032588.1	127920	.	T	G	100	*	TYPE=SUBSTITUTE
CM032588.1	127926	.	G	T	100	*	TYPE=SUBSTITUTE
CM032588.1	127928	.	T	C	100	*	TYPE=SUBSTITUTE

CM032588.1	127934	.	T	C	100	*	TYPE=SUBSTITUTE
CM032588.1	127960	.	A	T	100	*	TYPE=SUBSTITUTE
CM032588.1	127992	.	C	T	100	*	TYPE=SUBSTITUTE
CM032588.1	127996	.	TGCTC	T	100	*	TYPE=DELETE
CM032588.1	128006	.	A	C	100	*	TYPE=SUBSTITUTE
CM032588.1	128012	.	C	T	100	*	TYPE=SUBSTITUTE
CM032588.1	128014	.	C	T	100	*	TYPE=SUBSTITUTE
CM032588.1	128015	.	A	G	100	*	TYPE=SUBSTITUTE
CM032588.1	128017	.	C	A	100	*	TYPE=SUBSTITUTE
CM032588.1	128051	.	C	t	100	*	TYPE=SUBSTITUTE
CM032588.1	128052	.	T	c	100	*	TYPE=SUBSTITUTE
CM032588.1	128060	.	C	T	100	*	TYPE=SUBSTITUTE
CM032588.1	128084	.	G	A	100	*	TYPE=SUBSTITUTE
CM032588.1	128141	.	C	T	100	*	TYPE=SUBSTITUTE
CM032588.1	128146	.	C	T	100	*	TYPE=SUBSTITUTE
CM032588.1	128172	.	A	G	100	*	TYPE=SUBSTITUTE
CM032588.1	128181	.	T	C	100	*	TYPE=SUBSTITUTE
CM032588.1	128184	.	T	G	100	*	TYPE=SUBSTITUTE
CM032588.1	128221	.	T	C	100	*	TYPE=SUBSTITUTE
CM032588.1	128239	.	A	G	100	*	TYPE=SUBSTITUTE
CM032588.1	128256	.	T	C	100	*	TYPE=SUBSTITUTE
CM032588.1	128263	.	C	A	100	*	TYPE=SUBSTITUTE
CM032588.1	128264	.	GC	G	100	*	TYPE=DELETE
CM032588.1	128271	.	G	A	100	*	TYPE=SUBSTITUTE
CM032588.1	128275	.	C	T	100	*	TYPE=SUBSTITUTE
CM032588.1	128284	.	C	G	100	*	TYPE=SUBSTITUTE
CM032588.1	128314	.	C	A	100	*	TYPE=SUBSTITUTE
CM032588.1	128316	.	A	G	100	*	TYPE=SUBSTITUTE
CM032588.1	128343	.	G	C	100	*	TYPE=SUBSTITUTE
CM032588.1	128385	.	TA	T	100	*	TYPE=DELETE
CM032588.1	128388	.	G	A	100	*	TYPE=SUBSTITUTE
CM032588.1	128391	.	A	T	100	*	TYPE=SUBSTITUTE
CM032588.1	128418	.	T	C	100	*	TYPE=SUBSTITUTE
CM032588.1	128421	.	G	C	100	*	TYPE=SUBSTITUTE
CM032588.1	128491	.	G	C	100	*	TYPE=SUBSTITUTE
CM032588.1	128525	.	T	C	100	*	TYPE=SUBSTITUTE
CM032588.1	128562	.	T	C	100	*	TYPE=SUBSTITUTE
CM032588.1	128566	.	T	C	100	*	TYPE=SUBSTITUTE
CM032588.1	128576	.	T	A	100	*	TYPE=SUBSTITUTE
CM032588.1	128584	.	C	T	100	*	TYPE=SUBSTITUTE
CM032588.1	128611	.	A	G	100	*	TYPE=SUBSTITUTE
CM032588.1	128624	.	C	A	100	*	TYPE=SUBSTITUTE
CM032588.1	128628	.	TAC	T	100	*	TYPE=DELETE
CM032588.1	128661	.	A	g	100	*	TYPE=SUBSTITUTE
CM032588.1	128664	.	C	a	100	*	TYPE=SUBSTITUTE
CM032588.1	128670	.	G	a	100	*	TYPE=SUBSTITUTE
CM032588.1	128671	.	G	t	100	*	TYPE=SUBSTITUTE
CM032588.1	128672	.	T	a	100	*	TYPE=SUBSTITUTE
CM032588.1	128674	.	AG	A	100	*	TYPE=DELETE
CM032588.1	128683	.	TAAAAAAAAA	T	100	*	TYPE=DELETE
CM032588.1	128695	.	A	g	100	*	TYPE=SUBSTITUTE
CM032588.1	128706	.	A	c	100	*	TYPE=SUBSTITUTE
CM032588.1	128788	.	A	G	100	*	TYPE=SUBSTITUTE
CM032588.1	128803	.	T	C	100	*	TYPE=SUBSTITUTE
CM032588.1	128820	.	A	G	100	*	TYPE=SUBSTITUTE
CM032588.1	128837	.	T	C	100	*	TYPE=SUBSTITUTE
CM032588.1	128839	.	C	A	100	*	TYPE=SUBSTITUTE
CM032588.1	128863	.	T	C	100	*	TYPE=SUBSTITUTE
CM032588.1	128870	.	A	G	100	*	TYPE=SUBSTITUTE
CM032588.1	128874	.	C	T	100	*	TYPE=SUBSTITUTE
CM032588.1	128876	.	TCCC	T	100	*	TYPE=DELETE
CM032588.1	129000	.	A	G	100	*	TYPE=SUBSTITUTE
CM032588.1	129013	.	T	A	100	*	TYPE=SUBSTITUTE
CM032588.1	129014	.	C	T	100	*	TYPE=SUBSTITUTE

CM032588.1	129018	.	A	T	100	*	TYPE=SUBSTITUTE
CM032588.1	129107	.	T	C	100	*	TYPE=SUBSTITUTE
CM032588.1	129178	.	C	A	100	*	TYPE=SUBSTITUTE
CM032588.1	129268	.	C	A	100	*	TYPE=SUBSTITUTE
CM032588.1	129290	.	T	G	100	*	TYPE=SUBSTITUTE
CM032588.1	129309	.	A	G	100	*	TYPE=SUBSTITUTE
CM032588.1	129314	.	T	C	100	*	TYPE=SUBSTITUTE
CM032588.1	129326	.	C	G	100	*	TYPE=SUBSTITUTE
CM032588.1	129344	.	G	C	100	*	TYPE=SUBSTITUTE
CM032588.1	129347	.	T	A	100	*	TYPE=SUBSTITUTE
CM032588.1	129388	.	T	G	100	*	TYPE=SUBSTITUTE
CM032588.1	129450	.	C	T	100	*	TYPE=SUBSTITUTE
CM032588.1	129606	.	A	G	100	*	TYPE=SUBSTITUTE
CM032588.1	129694	.	A	G	100	*	TYPE=SUBSTITUTE
CM032588.1	129727	.	G	A	100	*	TYPE=SUBSTITUTE
CM032588.1	129735	.	A	C	100	*	TYPE=SUBSTITUTE
CM032588.1	129806	.	C	G	100	*	TYPE=SUBSTITUTE
CM032588.1	129935	.	C	T	100	*	TYPE=SUBSTITUTE
CM032588.1	130044	.	A	G	100	*	TYPE=SUBSTITUTE
CM032588.1	130084	.	G	A	100	*	TYPE=SUBSTITUTE
CM032588.1	130134	.	T	A	100	*	TYPE=SUBSTITUTE
CM032588.1	130214	.	G	A	100	*	TYPE=SUBSTITUTE
CM032588.1	130232	.	C	T	100	*	TYPE=SUBSTITUTE
CM032588.1	130319	.	G	T	100	*	TYPE=SUBSTITUTE
CM032588.1	130346	.	C	T	100	*	TYPE=SUBSTITUTE
CM032588.1	130378	.	A	G	100	*	TYPE=SUBSTITUTE
CM032588.1	130394	.	T	C	100	*	TYPE=SUBSTITUTE
CM032588.1	130433	.	T	C	100	*	TYPE=SUBSTITUTE
CM032588.1	130441	.	T	C	100	*	TYPE=SUBSTITUTE
CM032588.1	130475	.	C	G	100	*	TYPE=SUBSTITUTE
CM032588.1	130525	.	A	T	100	*	TYPE=SUBSTITUTE
CM032588.1	130556	.	T	C	100	*	TYPE=SUBSTITUTE
CM032588.1	130590	.	A	G	100	*	TYPE=SUBSTITUTE
CM032588.1	130602	.	C	t	100	*	TYPE=SUBSTITUTE
CM032588.1	130691	.	A	G	100	*	TYPE=SUBSTITUTE
CM032588.1	130704	.	C	A	100	*	TYPE=SUBSTITUTE
CM032588.1	130725	.	A	G	100	*	TYPE=SUBSTITUTE
CM032588.1	130762	.	C	T	100	*	TYPE=SUBSTITUTE
CM032588.1	130769	.	C	T	100	*	TYPE=SUBSTITUTE
CM032588.1	130811	.	C	T	100	*	TYPE=SUBSTITUTE
CM032588.1	130817	.	C	T	100	*	TYPE=SUBSTITUTE
CM032588.1	130826	.	C	T	100	*	TYPE=SUBSTITUTE
CM032588.1	130835	.	A	T	100	*	TYPE=SUBSTITUTE
CM032588.1	130952	.	C	T	100	*	TYPE=SUBSTITUTE
CM032588.1	131032	.	A	G	100	*	TYPE=SUBSTITUTE
CM032588.1	131048	.	T	G	100	*	TYPE=SUBSTITUTE
CM032588.1	131172	.	C	T	100	*	TYPE=SUBSTITUTE
CM032588.1	131175	.	G	C	100	*	TYPE=SUBSTITUTE
CM032588.1	131202	.	C	A	100	*	TYPE=SUBSTITUTE
CM032588.1	131216	.	A	G	100	*	TYPE=SUBSTITUTE
CM032588.1	131228	.	G	A	100	*	TYPE=SUBSTITUTE
CM032588.1	131258	.	T	C	100	*	TYPE=SUBSTITUTE
CM032588.1	131317	.	G	C	100	*	TYPE=SUBSTITUTE
CM032588.1	131327	.	C	T	100	*	TYPE=SUBSTITUTE
CM032588.1	131337	.	A	G	100	*	TYPE=SUBSTITUTE
CM032588.1	131365	.	A	T	100	*	TYPE=SUBSTITUTE
CM032588.1	131392	.	G	A	100	*	TYPE=SUBSTITUTE
CM032588.1	131405	.	A	G	100	*	TYPE=SUBSTITUTE
CM032588.1	131454	.	A	G	100	*	TYPE=SUBSTITUTE
CM032588.1	131463	.	G	A	100	*	TYPE=SUBSTITUTE
CM032588.1	131468	.	C	T	100	*	TYPE=SUBSTITUTE
CM032588.1	131602	.	G	A	100	*	TYPE=SUBSTITUTE
CM032588.1	131805	.	C	T	100	*	TYPE=SUBSTITUTE
CM032588.1	131881	.	C	A	100	*	TYPE=SUBSTITUTE

CM032588.1	131941	.	A	T	100	*	TYPE=SUBSTITUTE
CM032588.1	131971	.	T	C	100	*	TYPE=SUBSTITUTE
CM032588.1	131976	.	C	T	100	*	TYPE=SUBSTITUTE
CM032588.1	132031	.	T	C	100	*	TYPE=SUBSTITUTE
CM032588.1	132041	.	A	G	100	*	TYPE=SUBSTITUTE
CM032588.1	132078	.	A	aa	100	*	TYPE=INSERT
CM032588.1	132097	.	TTTT	T	100	*	TYPE=DELETE
CM032588.1	132101	.	T	A	100	*	TYPE=SUBSTITUTE
CM032588.1	132106	.	C	T	100	*	TYPE=SUBSTITUTE
CM032588.1	132107	.	A	T	100	*	TYPE=SUBSTITUTE
CM032588.1	132115	.	T	G	100	*	TYPE=SUBSTITUTE
CM032588.1	132145	.	G	A	100	*	TYPE=SUBSTITUTE
CM032588.1	132157	.	G	A	100	*	TYPE=SUBSTITUTE
CM032588.1	132161	.	G	C	100	*	TYPE=SUBSTITUTE
CM032588.1	132169	.	C	T	100	*	TYPE=SUBSTITUTE
CM032588.1	132174	.	C	T	100	*	TYPE=SUBSTITUTE
CM032588.1	132177	.	T	C	100	*	TYPE=SUBSTITUTE
CM032588.1	132191	.	T	G	100	*	TYPE=SUBSTITUTE
CM032588.1	132194	.	G	A	100	*	TYPE=SUBSTITUTE
CM032588.1	132218	.	T	G	100	*	TYPE=SUBSTITUTE
CM032588.1	132219	.	TC	T	100	*	TYPE=DELETE
CM032588.1	132223	.	G	C	100	*	TYPE=SUBSTITUTE
CM032588.1	132233	.	A	C	100	*	TYPE=SUBSTITUTE
CM032588.1	132234	.	G	C	100	*	TYPE=SUBSTITUTE
CM032588.1	132235	.	AT	A	100	*	TYPE=DELETE
CM032588.1	132241	.	T	C	100	*	TYPE=SUBSTITUTE
CM032588.1	132244	.	T	C	100	*	TYPE=SUBSTITUTE
CM032588.1	132260	.	C	T	100	*	TYPE=SUBSTITUTE
CM032588.1	132261	.	T	G	100	*	TYPE=SUBSTITUTE
CM032588.1	132267	.	C	T	100	*	TYPE=SUBSTITUTE
CM032588.1	132296	.	C	T	100	*	TYPE=SUBSTITUTE
CM032588.1	132303	.	G	T	100	*	TYPE=SUBSTITUTE
CM032588.1	132314	.	T	C	100	*	TYPE=SUBSTITUTE
CM032588.1	132316	.	A	T	100	*	TYPE=SUBSTITUTE
CM032588.1	132317	.	G	A	100	*	TYPE=SUBSTITUTE
CM032588.1	132335	.	T	C	100	*	TYPE=SUBSTITUTE
CM032588.1	132452	.	A	G	100	*	TYPE=SUBSTITUTE
CM032588.1	132472	.	T	C	100	*	TYPE=SUBSTITUTE
CM032588.1	132594	.	G	A	100	*	TYPE=SUBSTITUTE
CM032588.1	132618	.	T	G	100	*	TYPE=SUBSTITUTE
CM032588.1	132742	.	T	C	100	*	TYPE=SUBSTITUTE
CM032588.1	132806	.	G	C	100	*	TYPE=SUBSTITUTE
CM032588.1	132856	.	G	T	100	*	TYPE=SUBSTITUTE
CM032588.1	132950	.	G	C	100	*	TYPE=SUBSTITUTE
CM032588.1	133056	.	A	C	100	*	TYPE=SUBSTITUTE
CM032588.1	133085	.	AG	A	100	*	TYPE=DELETE
CM032588.1	133094	.	A	G	100	*	TYPE=SUBSTITUTE
CM032588.1	133095	.	G	A	100	*	TYPE=SUBSTITUTE
CM032588.1	133097	.	A	G	100	*	TYPE=SUBSTITUTE
CM032588.1	133105	.	G	A	100	*	TYPE=SUBSTITUTE
CM032588.1	133122	.	G	A	100	*	TYPE=SUBSTITUTE
CM032588.1	133129	.	A	G	100	*	TYPE=SUBSTITUTE
CM032588.1	133141	.	AC	A	100	*	TYPE=DELETE
CM032588.1	133147	.	CGCAGAGGATCCA	C	100	*	TYPE=DELETE
CM032588.1	133160	.	T	A	100	*	TYPE=SUBSTITUTE
CM032588.1	133166	.	T	C	100	*	TYPE=SUBSTITUTE
CM032588.1	133169	.	G	C	100	*	TYPE=SUBSTITUTE
CM032588.1	133170	.	G	C	100	*	TYPE=SUBSTITUTE
CM032588.1	133175	.	G	C	100	*	TYPE=SUBSTITUTE
CM032588.1	133182	.	A	G	100	*	TYPE=SUBSTITUTE
CM032588.1	133189	.	C	T	100	*	TYPE=SUBSTITUTE
CM032588.1	133192	.	A	C	100	*	TYPE=SUBSTITUTE
CM032588.1	133193	.	G	A	100	*	TYPE=SUBSTITUTE
CM032588.1	133194	.	C	T	100	*	TYPE=SUBSTITUTE

CM032588.1	133196	.	A	G	100	*	TYPE=SUBSTITUTE
CM032588.1	133197	.	C	T	100	*	TYPE=SUBSTITUTE
CM032588.1	133202	.	T	G	100	*	TYPE=SUBSTITUTE
CM032588.1	133204	.	C	T	100	*	TYPE=SUBSTITUTE
CM032588.1	133207	.	CA	C	100	*	TYPE=DELETE
CM032588.1	133221	.	C	T	100	*	TYPE=SUBSTITUTE
CM032588.1	133224	.	T	C	100	*	TYPE=SUBSTITUTE
CM032588.1	133226	.	A	G	100	*	TYPE=SUBSTITUTE
CM032588.1	133237	.	T	G	100	*	TYPE=SUBSTITUTE
CM032588.1	133250	.	C	T	100	*	TYPE=SUBSTITUTE
CM032588.1	133283	.	G	A	100	*	TYPE=SUBSTITUTE
CM032588.1	133292	.	C	T	100	*	TYPE=SUBSTITUTE
CM032588.1	133296	.	G	A	100	*	TYPE=SUBSTITUTE
CM032588.1	133305	.	T	A	100	*	TYPE=SUBSTITUTE
CM032588.1	133317	.	C	A	100	*	TYPE=SUBSTITUTE
CM032588.1	133337	.	C	T	100	*	TYPE=SUBSTITUTE
CM032588.1	133361	.	C	t	100	*	TYPE=SUBSTITUTE
CM032588.1	133392	.	G	A	100	*	TYPE=SUBSTITUTE
CM032588.1	133394	.	A	G	100	*	TYPE=SUBSTITUTE
CM032588.1	133397	.	G	A	100	*	TYPE=SUBSTITUTE
CM032588.1	133407	.	T	A	100	*	TYPE=SUBSTITUTE
CM032588.1	133443	.	A	G	100	*	TYPE=SUBSTITUTE
CM032588.1	133460	.	C	T	100	*	TYPE=SUBSTITUTE
CM032588.1	133469	.	G	A	100	*	TYPE=SUBSTITUTE
CM032588.1	133499	.	C	T	100	*	TYPE=SUBSTITUTE
CM032588.1	133504	.	C	T	100	*	TYPE=SUBSTITUTE
CM032588.1	133514	.	G	T	100	*	TYPE=SUBSTITUTE
CM032588.1	133523	.	C	T	100	*	TYPE=SUBSTITUTE
CM032588.1	133542	.	G	A	100	*	TYPE=SUBSTITUTE
CM032588.1	133580	.	G	A	100	*	TYPE=SUBSTITUTE
CM032588.1	133589	.	C	T	100	*	TYPE=SUBSTITUTE
CM032588.1	133759	.	T	C	100	*	TYPE=SUBSTITUTE
CM032588.1	133801	.	G	C	100	*	TYPE=SUBSTITUTE
CM032588.1	133810	.	A	G	100	*	TYPE=SUBSTITUTE
CM032588.1	133816	.	A	C	100	*	TYPE=SUBSTITUTE
CM032588.1	133868	.	A	G	100	*	TYPE=SUBSTITUTE
CM032588.1	133891	.	T	C	100	*	TYPE=SUBSTITUTE
CM032588.1	133896	.	T	G	100	*	TYPE=SUBSTITUTE
CM032588.1	133958	.	T	a	100	*	TYPE=SUBSTITUTE
CM032588.1	133970	.	G	A	100	*	TYPE=SUBSTITUTE
CM032588.1	133983	.	G	C	100	*	TYPE=SUBSTITUTE
CM032588.1	134038	.	A	G	100	*	TYPE=SUBSTITUTE
CM032588.1	134049	.	T	C	100	*	TYPE=SUBSTITUTE
CM032588.1	134168	.	T	A	100	*	TYPE=SUBSTITUTE
CM032588.1	134171	.	A	G	100	*	TYPE=SUBSTITUTE
CM032588.1	134174	.	T	C	100	*	TYPE=SUBSTITUTE
CM032588.1	134344	.	G	A	100	*	TYPE=SUBSTITUTE
CM032588.1	134353	.	T	C	100	*	TYPE=SUBSTITUTE
CM032588.1	134770	.	T	C	100	*	TYPE=SUBSTITUTE
CM032588.1	134885	.	G	C	100	*	TYPE=SUBSTITUTE
CM032588.1	134960	.	T	c	100	*	TYPE=SUBSTITUTE
CM032588.1	135666	.	G	A	100	*	TYPE=SUBSTITUTE
CM032588.1	135824	.	G	A	100	*	TYPE=SUBSTITUTE
CM032588.1	135892	.	G	A	100	*	TYPE=SUBSTITUTE
CM032588.1	136643	.	G	T	100	*	TYPE=SUBSTITUTE
CM032588.1	136644	.	A	C	100	*	TYPE=SUBSTITUTE
CM032588.1	136686	.	G	t	100	*	TYPE=SUBSTITUTE
CM032588.1	136692	.	G	c	100	*	TYPE=SUBSTITUTE
CM032588.1	136695	.	T	c	100	*	TYPE=SUBSTITUTE
CM032588.1	136709	.	G	A	100	*	TYPE=SUBSTITUTE
CM032588.1	136716	.	C	T	100	*	TYPE=SUBSTITUTE
CM032588.1	136722	.	G	T	100	*	TYPE=SUBSTITUTE
CM032588.1	136755	.	T	A	100	*	TYPE=SUBSTITUTE
CM032588.1	136791	.	C	T	100	*	TYPE=SUBSTITUTE

CM032588.1	136794	.	T	C	100	*	TYPE=SUBSTITUTE
CM032588.1	136839	.	C	T	100	*	TYPE=SUBSTITUTE
CM032588.1	136851	.	C	G	100	*	TYPE=SUBSTITUTE
CM032588.1	136860	.	T	G	100	*	TYPE=SUBSTITUTE
CM032588.1	136881	.	T	C	100	*	TYPE=SUBSTITUTE
CM032588.1	136913	.	T	A	100	*	TYPE=SUBSTITUTE
CM032588.1	136953	.	G	T	100	*	TYPE=SUBSTITUTE
CM032588.1	136981	.	T	A	100	*	TYPE=SUBSTITUTE
CM032588.1	137000	.	C	G	100	*	TYPE=SUBSTITUTE
CM032588.1	137014	.	G	A	100	*	TYPE=SUBSTITUTE
CM032588.1	137016	.	A	G	100	*	TYPE=SUBSTITUTE
CM032588.1	137025	.	G	T	100	*	TYPE=SUBSTITUTE
CM032588.1	137034	.	A	G	100	*	TYPE=SUBSTITUTE
CM032588.1	137073	.	C	T	100	*	TYPE=SUBSTITUTE
CM032588.1	137135	.	T	C	100	*	TYPE=SUBSTITUTE
CM032588.1	137190	.	A	T	100	*	TYPE=SUBSTITUTE
CM032588.1	137304	.	G	A	100	*	TYPE=SUBSTITUTE
CM032588.1	137313	.	A	G	100	*	TYPE=SUBSTITUTE
CM032588.1	137328	.	T	C	100	*	TYPE=SUBSTITUTE
CM032588.1	137334	.	G	A	100	*	TYPE=SUBSTITUTE
CM032588.1	137358	.	G	A	100	*	TYPE=SUBSTITUTE
CM032588.1	137368	.	A	T	100	*	TYPE=SUBSTITUTE
CM032588.1	137375	.	A	T	100	*	TYPE=SUBSTITUTE
CM032588.1	137395	.	T	C	100	*	TYPE=SUBSTITUTE
CM032588.1	137419	.	T	G	100	*	TYPE=SUBSTITUTE
CM032588.1	137427	.	TTGG	T	100	*	TYPE=DELETE
CM032588.1	137433	.	T	A	100	*	TYPE=SUBSTITUTE
CM032588.1	137440	.	G	A	100	*	TYPE=SUBSTITUTE
CM032588.1	137443	.	A	G	100	*	TYPE=SUBSTITUTE
CM032588.1	137467	.	G	A	100	*	TYPE=SUBSTITUTE
CM032588.1	137482	.	T	C	100	*	TYPE=SUBSTITUTE
CM032588.1	137488	.	A	G	100	*	TYPE=SUBSTITUTE
CM032588.1	137491	.	C	T	100	*	TYPE=SUBSTITUTE
CM032588.1	137493	.	A	G	100	*	TYPE=SUBSTITUTE
CM032588.1	137533	.	G	A	100	*	TYPE=SUBSTITUTE
CM032588.1	137537	.	T	C	100	*	TYPE=SUBSTITUTE
CM032588.1	137538	.	T	C	100	*	TYPE=SUBSTITUTE
CM032588.1	137550	.	T	C	100	*	TYPE=SUBSTITUTE
CM032588.1	137554	.	G	T	100	*	TYPE=SUBSTITUTE
CM032588.1	137569	.	A	G	100	*	TYPE=SUBSTITUTE
CM032588.1	137593	.	T	C	100	*	TYPE=SUBSTITUTE
CM032588.1	137611	.	A	T	100	*	TYPE=SUBSTITUTE
CM032588.1	137623	.	A	G	100	*	TYPE=SUBSTITUTE
CM032588.1	137626	.	A	G	100	*	TYPE=SUBSTITUTE
CM032588.1	137644	.	A	G	100	*	TYPE=SUBSTITUTE
CM032588.1	137670	.	C	T	100	*	TYPE=SUBSTITUTE
CM032588.1	137683	.	A	G	100	*	TYPE=SUBSTITUTE
CM032588.1	137706	.	C	T	100	*	TYPE=SUBSTITUTE
CM032588.1	137713	.	G	A	100	*	TYPE=SUBSTITUTE
CM032588.1	137719	.	A	G	100	*	TYPE=SUBSTITUTE
CM032588.1	137722	.	T	C	100	*	TYPE=SUBSTITUTE
CM032588.1	137727	.	C	T	100	*	TYPE=SUBSTITUTE
CM032588.1	137730	.	C	T	100	*	TYPE=SUBSTITUTE
CM032588.1	137733	.	C	T	100	*	TYPE=SUBSTITUTE
CM032588.1	137737	.	C	T	100	*	TYPE=SUBSTITUTE
CM032588.1	137741	.	T	A	100	*	TYPE=SUBSTITUTE
CM032588.1	137742	.	T	A	100	*	TYPE=SUBSTITUTE
CM032588.1	137747	.	C	T	100	*	TYPE=SUBSTITUTE
CM032588.1	137748	.	G	T	100	*	TYPE=SUBSTITUTE
CM032588.1	137758	.	T	C	100	*	TYPE=SUBSTITUTE
CM032588.1	137779	.	A	C	100	*	TYPE=SUBSTITUTE
CM032588.1	137785	.	C	T	100	*	TYPE=SUBSTITUTE
CM032588.1	137825	.	G	A	100	*	TYPE=SUBSTITUTE
CM032588.1	137991	.	T	G	100	*	TYPE=SUBSTITUTE

CM032588.1	138028	.	T	C	100	*	TYPE=SUBSTITUTE
CM032588.1	138051	.	C	T	100	*	TYPE=SUBSTITUTE
CM032588.1	138083	.	A	G	100	*	TYPE=SUBSTITUTE
CM032588.1	138088	.	T	C	100	*	TYPE=SUBSTITUTE
CM032588.1	138090	.	A	G	100	*	TYPE=SUBSTITUTE
CM032588.1	138124	.	A	G	100	*	TYPE=SUBSTITUTE
CM032588.1	138133	.	C	T	100	*	TYPE=SUBSTITUTE
CM032588.1	138135	.	T	A	100	*	TYPE=SUBSTITUTE
CM032588.1	138149	.	T	C	100	*	TYPE=SUBSTITUTE
CM032588.1	138154	.	A	G	100	*	TYPE=SUBSTITUTE
CM032588.1	138178	.	A	G	100	*	TYPE=SUBSTITUTE
CM032588.1	138205	.	A	G	100	*	TYPE=SUBSTITUTE
CM032588.1	138219	.	T	C	100	*	TYPE=SUBSTITUTE
CM032588.1	138235	.	T	C	100	*	TYPE=SUBSTITUTE
CM032588.1	138250	.	G	A	100	*	TYPE=SUBSTITUTE
CM032588.1	138253	.	G	A	100	*	TYPE=SUBSTITUTE
CM032588.1	138259	.	T	C	100	*	TYPE=SUBSTITUTE
CM032588.1	138268	.	G	A	100	*	TYPE=SUBSTITUTE
CM032588.1	138276	.	G	T	100	*	TYPE=SUBSTITUTE
CM032588.1	138278	.	G	T	100	*	TYPE=SUBSTITUTE
CM032588.1	138279	.	T	C	100	*	TYPE=SUBSTITUTE
CM032588.1	138287	.	C	T	100	*	TYPE=SUBSTITUTE
CM032588.1	138289	.	T	C	100	*	TYPE=SUBSTITUTE
CM032588.1	138290	.	A	G	100	*	TYPE=SUBSTITUTE
CM032588.1	138294	.	T	C	100	*	TYPE=SUBSTITUTE
CM032588.1	138299	.	A	G	100	*	TYPE=SUBSTITUTE
CM032588.1	138303	.	T	G	100	*	TYPE=SUBSTITUTE
CM032588.1	138328	.	A	C	100	*	TYPE=SUBSTITUTE
CM032588.1	138343	.	A	G	100	*	TYPE=SUBSTITUTE
CM032588.1	138345	.	A	G	100	*	TYPE=SUBSTITUTE
CM032588.1	138348	.	A	G	100	*	TYPE=SUBSTITUTE
CM032588.1	138358	.	T	A	100	*	TYPE=SUBSTITUTE
CM032588.1	138362	.	G	A	100	*	TYPE=SUBSTITUTE
CM032588.1	138363	.	A	G	100	*	TYPE=SUBSTITUTE
CM032588.1	138371	.	G	A	100	*	TYPE=SUBSTITUTE
CM032588.1	138378	.	A	G	100	*	TYPE=SUBSTITUTE
CM032588.1	138385	.	GA	G	100	*	TYPE=DELETE
CM032588.1	138390	.	G	C	100	*	TYPE=SUBSTITUTE
CM032588.1	138391	.	C	T	100	*	TYPE=SUBSTITUTE
CM032588.1	138395	.	T	A	100	*	TYPE=SUBSTITUTE
CM032588.1	138397	.	A	T	100	*	TYPE=SUBSTITUTE
CM032588.1	138406	.	G	C	100	*	TYPE=SUBSTITUTE
CM032588.1	138407	.	G	A	100	*	TYPE=SUBSTITUTE
CM032588.1	138420	.	A	G	100	*	TYPE=SUBSTITUTE
CM032588.1	138424	.	A	T	100	*	TYPE=SUBSTITUTE
CM032588.1	138441	.	C	T	100	*	TYPE=SUBSTITUTE
CM032588.1	138472	.	G	A	100	*	TYPE=SUBSTITUTE
CM032588.1	138479	.	T	C	100	*	TYPE=SUBSTITUTE
CM032588.1	138485	.	T	G	100	*	TYPE=SUBSTITUTE
CM032588.1	138486	.	A	T	100	*	TYPE=SUBSTITUTE
CM032588.1	138491	.	G	C	100	*	TYPE=SUBSTITUTE
CM032588.1	138500	.	A	G	100	*	TYPE=SUBSTITUTE
CM032588.1	138505	.	A	G	100	*	TYPE=SUBSTITUTE
CM032588.1	138538	.	T	C	100	*	TYPE=SUBSTITUTE
CM032588.1	138541	.	G	A	100	*	TYPE=SUBSTITUTE
CM032588.1	138545	.	A	G	100	*	TYPE=SUBSTITUTE
CM032588.1	138581	.	G	A	100	*	TYPE=SUBSTITUTE
CM032588.1	138583	.	T	C	100	*	TYPE=SUBSTITUTE
CM032588.1	138585	.	A	G	100	*	TYPE=SUBSTITUTE
CM032588.1	138606	.	G	C	100	*	TYPE=SUBSTITUTE
CM032588.1	138610	.	C	T	100	*	TYPE=SUBSTITUTE
CM032588.1	138619	.	A	G	100	*	TYPE=SUBSTITUTE
CM032588.1	138650	.	T	TCA	100	*	TYPE=INSERT
CM032588.1	138651	.	T	C	100	*	TYPE=SUBSTITUTE

CM032588.1	138709	.	A	G	100	*	TYPE=SUBSTITUTE
CM032588.1	138723	.	G	a	100	*	TYPE=SUBSTITUTE
CM032588.1	138733	.	G	a	100	*	TYPE=SUBSTITUTE
CM032588.1	138748	.	T	C	100	*	TYPE=SUBSTITUTE
CM032588.1	138819	.	A	G	100	*	TYPE=SUBSTITUTE
CM032588.1	138834	.	T	A	100	*	TYPE=SUBSTITUTE
CM032588.1	138835	.	C	T	100	*	TYPE=SUBSTITUTE
CM032588.1	138839	.	A	G	100	*	TYPE=SUBSTITUTE
CM032588.1	138996	.	G	GT	100	*	TYPE=INSERT
CM032588.1	138999	.	C	T	100	*	TYPE=SUBSTITUTE
CM032588.1	139039	.	G	A	100	*	TYPE=SUBSTITUTE
CM032588.1	139042	.	G	A	100	*	TYPE=SUBSTITUTE
CM032588.1	139057	.	T	A	100	*	TYPE=SUBSTITUTE
CM032588.1	139064	.	A	G	100	*	TYPE=SUBSTITUTE
CM032588.1	139065	.	T	A	100	*	TYPE=SUBSTITUTE
CM032588.1	139076	.	C	T	100	*	TYPE=SUBSTITUTE
CM032588.1	139078	.	C	T	100	*	TYPE=SUBSTITUTE
CM032588.1	139085	.	G	A	100	*	TYPE=SUBSTITUTE
CM032588.1	139087	.	A	G	100	*	TYPE=SUBSTITUTE
CM032588.1	139089	.	A	T	100	*	TYPE=SUBSTITUTE
CM032588.1	139090	.	C	T	100	*	TYPE=SUBSTITUTE
CM032588.1	139091	.	A	G	100	*	TYPE=SUBSTITUTE
CM032588.1	139104	.	C	G	100	*	TYPE=SUBSTITUTE
CM032588.1	139117	.	T	G	100	*	TYPE=SUBSTITUTE
CM032588.1	139119	.	C	T	100	*	TYPE=SUBSTITUTE
CM032588.1	139124	.	T	A	100	*	TYPE=SUBSTITUTE
CM032588.1	139129	.	T	C	100	*	TYPE=SUBSTITUTE
CM032588.1	139130	.	T	A	100	*	TYPE=SUBSTITUTE
CM032588.1	139131	.	T	A	100	*	TYPE=SUBSTITUTE
CM032588.1	139132	.	G	T	100	*	TYPE=SUBSTITUTE
CM032588.1	139134	.	T	G	100	*	TYPE=SUBSTITUTE
CM032588.1	139135	.	G	A	100	*	TYPE=SUBSTITUTE
CM032588.1	139143	.	A	G	100	*	TYPE=SUBSTITUTE
CM032588.1	139155	.	C	G	100	*	TYPE=SUBSTITUTE
CM032588.1	139156	.	C	G	100	*	TYPE=SUBSTITUTE
CM032588.1	139182	.	G	GA	100	*	TYPE=INSERT
CM032588.1	139195	.	C	T	100	*	TYPE=SUBSTITUTE
CM032588.1	139263	.	G	A	100	*	TYPE=SUBSTITUTE
CM032588.1	139286	.	C	T	100	*	TYPE=SUBSTITUTE
CM032588.1	139301	.	C	G	100	*	TYPE=SUBSTITUTE
CM032588.1	139359	.	G	C	100	*	TYPE=SUBSTITUTE
CM032588.1	139415	.	G	C	100	*	TYPE=SUBSTITUTE
CM032588.1	139428	.	C	T	100	*	TYPE=SUBSTITUTE
CM032588.1	139444	.	G	T	100	*	TYPE=SUBSTITUTE
CM032588.1	139483	.	A	T	100	*	TYPE=SUBSTITUTE
CM032588.1	139484	.	T	G	100	*	TYPE=SUBSTITUTE
CM032588.1	139497	.	C	T	100	*	TYPE=SUBSTITUTE
CM032588.1	139507	.	A	C	100	*	TYPE=SUBSTITUTE
CM032588.1	139508	.	G	A	100	*	TYPE=SUBSTITUTE
CM032588.1	139509	.	T	C	100	*	TYPE=SUBSTITUTE
CM032588.1	139700	.	C	T	100	*	TYPE=SUBSTITUTE
CM032588.1	139733	.	C	T	100	*	TYPE=SUBSTITUTE
CM032588.1	139780	.	T	A	100	*	TYPE=SUBSTITUTE
CM032588.1	139783	.	T	C	100	*	TYPE=SUBSTITUTE
CM032588.1	139811	.	T	C	100	*	TYPE=SUBSTITUTE
CM032588.1	139831	.	T	C	100	*	TYPE=SUBSTITUTE
CM032588.1	139833	.	A	C	100	*	TYPE=SUBSTITUTE
CM032588.1	139838	.	T	G	100	*	TYPE=SUBSTITUTE
CM032588.1	139839	.	C	T	100	*	TYPE=SUBSTITUTE
CM032588.1	139846	.	T	G	100	*	TYPE=SUBSTITUTE
CM032588.1	139886	.	C	T	100	*	TYPE=SUBSTITUTE
CM032588.1	139894	.	A	T	100	*	TYPE=SUBSTITUTE
CM032588.1	139913	.	G	T	100	*	TYPE=SUBSTITUTE
CM032588.1	139920	.	G	A	100	*	TYPE=SUBSTITUTE

CM032588.1	139930	.	A	G	100	*	TYPE=SUBSTITUTE
CM032588.1	139947	.	C	T	100	*	TYPE=SUBSTITUTE
CM032588.1	140032	.	A	G	100	*	TYPE=SUBSTITUTE
CM032588.1	140051	.	C	t	100	*	TYPE=SUBSTITUTE
CM032588.1	140112	.	G	A	100	*	TYPE=SUBSTITUTE
CM032588.1	140115	.	T	A	100	*	TYPE=SUBSTITUTE
CM032588.1	140126	.	T	C	100	*	TYPE=SUBSTITUTE
CM032588.1	140144	.	G	A	100	*	TYPE=SUBSTITUTE
CM032588.1	140147	.	C	T	100	*	TYPE=SUBSTITUTE
CM032588.1	140151	.	C	T	100	*	TYPE=SUBSTITUTE
CM032588.1	140179	.	A	T	100	*	TYPE=SUBSTITUTE
CM032588.1	140226	.	T	C	100	*	TYPE=SUBSTITUTE
CM032588.1	140248	.	A	G	100	*	TYPE=SUBSTITUTE
CM032588.1	140274	.	A	G	100	*	TYPE=SUBSTITUTE
CM032588.1	140277	.	G	A	100	*	TYPE=SUBSTITUTE
CM032588.1	140289	.	T	C	100	*	TYPE=SUBSTITUTE
CM032588.1	140316	.	C	A	100	*	TYPE=SUBSTITUTE
CM032588.1	140329	.	G	T	100	*	TYPE=SUBSTITUTE
CM032588.1	140330	.	A	C	100	*	TYPE=SUBSTITUTE
CM032588.1	140376	.	T	C	100	*	TYPE=SUBSTITUTE
CM032588.1	140385	.	C	T	100	*	TYPE=SUBSTITUTE
CM032588.1	140403	.	C	T	100	*	TYPE=SUBSTITUTE
CM032588.1	140437	.	A	G	100	*	TYPE=SUBSTITUTE
CM032588.1	140441	.	G	A	100	*	TYPE=SUBSTITUTE
CM032588.1	140445	.	C	t	100	*	TYPE=SUBSTITUTE
CM032588.1	140448	.	A	c	100	*	TYPE=SUBSTITUTE
CM032588.1	140452	.	T	g	100	*	TYPE=SUBSTITUTE
CM032588.1	140455	.	C	t	100	*	TYPE=SUBSTITUTE
CM032588.1	140457	.	A	g	100	*	TYPE=SUBSTITUTE
CM032588.1	140459	.	T	c	100	*	TYPE=SUBSTITUTE
CM032588.1	140460	.	A	t	100	*	TYPE=SUBSTITUTE
CM032588.1	140471	.	C	G	100	*	TYPE=SUBSTITUTE
CM032588.1	140473	.	A	G	100	*	TYPE=SUBSTITUTE
CM032588.1	140474	.	G	T	100	*	TYPE=SUBSTITUTE
CM032588.1	140481	.	A	C	100	*	TYPE=SUBSTITUTE
CM032588.1	140484	.	G	A	100	*	TYPE=SUBSTITUTE
CM032588.1	140489	.	G	A	100	*	TYPE=SUBSTITUTE
CM032588.1	140498	.	C	T	100	*	TYPE=SUBSTITUTE
CM032588.1	140501	.	T	C	100	*	TYPE=SUBSTITUTE
CM032588.1	140586	.	G	T	100	*	TYPE=SUBSTITUTE
CM032588.1	140589	.	A	G	100	*	TYPE=SUBSTITUTE
CM032588.1	140605	.	A	t	100	*	TYPE=SUBSTITUTE
CM032588.1	140612	.	A	t	100	*	TYPE=SUBSTITUTE
CM032588.1	140615	.	G	a	100	*	TYPE=SUBSTITUTE
CM032588.1	140651	.	C	T	100	*	TYPE=SUBSTITUTE
CM032588.1	140654	.	C	T	100	*	TYPE=SUBSTITUTE
CM032588.1	140676	.	G	A	100	*	TYPE=SUBSTITUTE
CM032588.1	140766	.	TT	T	100	*	TYPE=DELETE
CM032588.1	140867	.	C	T	100	*	TYPE=SUBSTITUTE
CM032588.1	141245	.	GG	G	100	*	TYPE=DELETE
CM032588.1	141331	.	C	T	100	*	TYPE=SUBSTITUTE
CM032588.1	141366	.	A	G	100	*	TYPE=SUBSTITUTE
CM032588.1	141385	.	C	T	100	*	TYPE=SUBSTITUTE
CM032588.1	141595	.	T	C	100	*	TYPE=SUBSTITUTE
CM032588.1	141598	.	T	G	100	*	TYPE=SUBSTITUTE
CM032588.1	141604	.	A	G	100	*	TYPE=SUBSTITUTE
CM032588.1	141634	.	G	A	100	*	TYPE=SUBSTITUTE
CM032588.1	141649	.	T	C	100	*	TYPE=SUBSTITUTE
CM032588.1	141655	.	T	C	100	*	TYPE=SUBSTITUTE
CM032588.1	141656	.	C	A	100	*	TYPE=SUBSTITUTE
CM032588.1	141658	.	A	G	100	*	TYPE=SUBSTITUTE
CM032588.1	141661	.	T	A	100	*	TYPE=SUBSTITUTE
CM032588.1	141694	.	G	A	100	*	TYPE=SUBSTITUTE
CM032588.1	141700	.	C	T	100	*	TYPE=SUBSTITUTE

CM032588.1	141703	.	T	C	100	*	TYPE=SUBSTITUTE
CM032588.1	141724	.	C	T	100	*	TYPE=SUBSTITUTE
CM032588.1	141742	.	A	G	100	*	TYPE=SUBSTITUTE
CM032588.1	141757	.	G	A	100	*	TYPE=SUBSTITUTE
CM032588.1	141766	.	C	T	100	*	TYPE=SUBSTITUTE
CM032588.1	141769	.	A	G	100	*	TYPE=SUBSTITUTE
CM032588.1	141781	.	C	T	100	*	TYPE=SUBSTITUTE
CM032588.1	141790	.	C	T	100	*	TYPE=SUBSTITUTE
CM032588.1	141793	.	C	T	100	*	TYPE=SUBSTITUTE
CM032588.1	141796	.	T	C	100	*	TYPE=SUBSTITUTE
CM032588.1	141799	.	C	T	100	*	TYPE=SUBSTITUTE
CM032588.1	141837	.	C	T	100	*	TYPE=SUBSTITUTE
CM032588.1	141841	.	G	C	100	*	TYPE=SUBSTITUTE
CM032588.1	141849	.	G	C	100	*	TYPE=SUBSTITUTE
CM032588.1	141853	.	A	G	100	*	TYPE=SUBSTITUTE
CM032588.1	141857	.	A	G	100	*	TYPE=SUBSTITUTE
CM032588.1	141877	.	TAG	T	100	*	TYPE=DELETE
CM032588.1	141918	.	G	A	100	*	TYPE=SUBSTITUTE
CM032588.1	141924	.	C	T	100	*	TYPE=SUBSTITUTE
CM032588.1	141939	.	A	G	100	*	TYPE=SUBSTITUTE
CM032588.1	141972	.	T	C	100	*	TYPE=SUBSTITUTE
CM032588.1	141996	.	T	C	100	*	TYPE=SUBSTITUTE
CM032588.1	142008	.	C	T	100	*	TYPE=SUBSTITUTE
CM032588.1	142038	.	A	T	100	*	TYPE=SUBSTITUTE
CM032588.1	142068	.	T	C	100	*	TYPE=SUBSTITUTE
CM032588.1	142080	.	C	G	100	*	TYPE=SUBSTITUTE
CM032588.1	142090	.	T	C	100	*	TYPE=SUBSTITUTE
CM032588.1	142095	.	C	T	100	*	TYPE=SUBSTITUTE
CM032588.1	142110	.	T	C	100	*	TYPE=SUBSTITUTE
CM032588.1	142113	.	G	A	100	*	TYPE=SUBSTITUTE
CM032588.1	142239	.	A	C	100	*	TYPE=SUBSTITUTE
CM032588.1	142248	.	C	T	100	*	TYPE=SUBSTITUTE
CM032588.1	142257	.	T	A	100	*	TYPE=SUBSTITUTE
CM032588.1	142263	.	C	T	100	*	TYPE=SUBSTITUTE
CM032588.1	142269	.	G	T	100	*	TYPE=SUBSTITUTE
CM032588.1	142300	.	C	T	100	*	TYPE=SUBSTITUTE
CM032588.1	142320	.	G	A	100	*	TYPE=SUBSTITUTE
CM032588.1	142335	.	T	C	100	*	TYPE=SUBSTITUTE
CM032588.1	142353	.	G	T	100	*	TYPE=SUBSTITUTE
CM032588.1	142365	.	C	T	100	*	TYPE=SUBSTITUTE
CM032588.1	142393	.	C	A	100	*	TYPE=SUBSTITUTE
CM032588.1	142395	.	G	A	100	*	TYPE=SUBSTITUTE
CM032588.1	142398	.	T	C	100	*	TYPE=SUBSTITUTE
CM032588.1	142399	.	A	AT	100	*	TYPE=INSERT
CM032588.1	142400	.	A	C	100	*	TYPE=SUBSTITUTE
CM032588.1	142417	.	C	A	100	*	TYPE=SUBSTITUTE
CM032588.1	142418	.	T	C	100	*	TYPE=SUBSTITUTE
CM032588.1	142426	.	T	g	100	*	TYPE=SUBSTITUTE
CM032588.1	142435	.	T	c	100	*	TYPE=SUBSTITUTE
CM032588.1	142449	.	A	C	100	*	TYPE=SUBSTITUTE
CM032588.1	142462	.	G	A	100	*	TYPE=SUBSTITUTE
CM032588.1	142468	.	C	A	100	*	TYPE=SUBSTITUTE
CM032588.1	142474	.	T	C	100	*	TYPE=SUBSTITUTE
CM032588.1	142475	.	G	T	100	*	TYPE=SUBSTITUTE
CM032588.1	142482	.	A	G	100	*	TYPE=SUBSTITUTE
CM032588.1	142485	.	T	C	100	*	TYPE=SUBSTITUTE
CM032588.1	142498	.	C	A	100	*	TYPE=SUBSTITUTE
CM032588.1	142500	.	C	T	100	*	TYPE=SUBSTITUTE
CM032588.1	142501	.	T	G	100	*	TYPE=SUBSTITUTE
CM032588.1	142514	.	AAT	A	100	*	TYPE=DELETE
CM032588.1	142531	.	C	T	100	*	TYPE=SUBSTITUTE
CM032588.1	142561	.	G	A	100	*	TYPE=SUBSTITUTE
CM032588.1	142621	.	G	A	100	*	TYPE=SUBSTITUTE
CM032588.1	142696	.	A	G	100	*	TYPE=SUBSTITUTE

CM032588.1	142718	.	C	G	100	*	TYPE=SUBSTITUTE
CM032588.1	142723	.	A	G	100	*	TYPE=SUBSTITUTE
CM032588.1	142736	.	G	A	100	*	TYPE=SUBSTITUTE
CM032588.1	142747	.	A	C	100	*	TYPE=SUBSTITUTE
CM032588.1	142753	.	G	A	100	*	TYPE=SUBSTITUTE
CM032588.1	142768	.	T	C	100	*	TYPE=SUBSTITUTE
CM032588.1	142817	.	G	A	100	*	TYPE=SUBSTITUTE
CM032588.1	142850	.	G	A	100	*	TYPE=SUBSTITUTE
CM032588.1	142852	.	A	C	100	*	TYPE=SUBSTITUTE
CM032588.1	142915	.	A	G	100	*	TYPE=SUBSTITUTE
CM032588.1	142932	.	A	G	100	*	TYPE=SUBSTITUTE
CM032588.1	142962	.	T	C	100	*	TYPE=SUBSTITUTE
CM032588.1	142964	.	A	G	100	*	TYPE=SUBSTITUTE
CM032588.1	142967	.	G	A	100	*	TYPE=SUBSTITUTE
CM032588.1	142975	.	T	TGTACAG	100	*	TYPE=INSERT
CM032588.1	142992	.	a	at	100	*	TYPE=INSERT
CM032588.1	143002	.	G	A	100	*	TYPE=SUBSTITUTE
CM032588.1	143004	.	C	T	100	*	TYPE=SUBSTITUTE
CM032588.1	143007	.	T	A	100	*	TYPE=SUBSTITUTE
CM032588.1	143008	.	A	T	100	*	TYPE=SUBSTITUTE
CM032588.1	143011	.	TGTACGGA	T	100	*	TYPE=DELETE
CM032588.1	143026	.	AT	A	100	*	TYPE=DELETE
CM032588.1	143038	.	TG	T	100	*	TYPE=DELETE
CM032588.1	143041	.	T	A	100	*	TYPE=SUBSTITUTE
CM032588.1	143043	.	T	C	100	*	TYPE=SUBSTITUTE
CM032588.1	143048	.	TTC	T	100	*	TYPE=DELETE
CM032588.1	143051	.	A	C	100	*	TYPE=SUBSTITUTE
CM032588.1	143054	.	TCCC	T	100	*	TYPE=DELETE
CM032588.1	143062	.	C	G	100	*	TYPE=SUBSTITUTE
CM032588.1	143066	.	T	A	100	*	TYPE=SUBSTITUTE
CM032588.1	143090	.	A	G	100	*	TYPE=SUBSTITUTE
CM032588.1	143095	.	A	G	100	*	TYPE=SUBSTITUTE
CM032588.1	143099	.	A	T	100	*	TYPE=SUBSTITUTE
CM032588.1	143101	.	C	T	100	*	TYPE=SUBSTITUTE
CM032588.1	143103	.	A	G	100	*	TYPE=SUBSTITUTE
CM032588.1	143120	.	C	T	100	*	TYPE=SUBSTITUTE
CM032588.1	143121	.	C	G	100	*	TYPE=SUBSTITUTE
CM032588.1	143122	.	A	G	100	*	TYPE=SUBSTITUTE
CM032588.1	143132	.	A	ACCT	100	*	TYPE=INSERT
CM032588.1	143139	.	G	A	100	*	TYPE=SUBSTITUTE
CM032588.1	143154	.	G	A	100	*	TYPE=SUBSTITUTE
CM032588.1	143156	.	A	T	100	*	TYPE=SUBSTITUTE
CM032588.1	143267	.	T	C	100	*	TYPE=SUBSTITUTE
CM032588.1	143304	.	G	C	100	*	TYPE=SUBSTITUTE
CM032588.1	143327	.	T	C	100	*	TYPE=SUBSTITUTE
CM032588.1	143350	.	A	G	100	*	TYPE=SUBSTITUTE
CM032588.1	143352	.	T	TGG	100	*	TYPE=INSERT
CM032588.1	143374	.	GTAATG	G	100	*	TYPE=DELETE
CM032588.1	143387	.	T	C	100	*	TYPE=SUBSTITUTE
CM032588.1	143401	.	CG	C	100	*	TYPE=DELETE
CM032588.1	143443	.	T	C	100	*	TYPE=SUBSTITUTE
CM032588.1	143480	.	T	G	100	*	TYPE=SUBSTITUTE
CM032588.1	143515	.	G	A	100	*	TYPE=SUBSTITUTE
CM032588.1	143532	.	G	A	100	*	TYPE=SUBSTITUTE
CM032588.1	143612	.	TT	T	100	*	TYPE=DELETE
CM032588.1	143615	.	G	T	100	*	TYPE=SUBSTITUTE
CM032588.1	143619	.	C	T	100	*	TYPE=SUBSTITUTE
CM032588.1	143659	.	G	T	100	*	TYPE=SUBSTITUTE
CM032588.1	143670	.	C	T	100	*	TYPE=SUBSTITUTE
CM032588.1	143679	.	T	C	100	*	TYPE=SUBSTITUTE
CM032588.1	143686	.	CATT	C	100	*	TYPE=DELETE
CM032588.1	143693	.	C	G	100	*	TYPE=SUBSTITUTE
CM032588.1	143701	.	A	T	100	*	TYPE=SUBSTITUTE
CM032588.1	143708	.	T	C	100	*	TYPE=SUBSTITUTE

CM032588.1	143710	.	C	T	100	*	TYPE=SUBSTITUTE
CM032588.1	143718	.	C	G	100	*	TYPE=SUBSTITUTE
CM032588.1	143719	.	C	A	100	*	TYPE=SUBSTITUTE
CM032588.1	143728	.	G	C	100	*	TYPE=SUBSTITUTE
CM032588.1	143746	.	G	A	100	*	TYPE=SUBSTITUTE
CM032588.1	143750	.	G	A	100	*	TYPE=SUBSTITUTE
CM032588.1	143764	.	T	C	100	*	TYPE=SUBSTITUTE
CM032588.1	143774	.	A	T	100	*	TYPE=SUBSTITUTE
CM032588.1	143788	.	G	A	100	*	TYPE=SUBSTITUTE
CM032588.1	143794	.	A	T	100	*	TYPE=SUBSTITUTE
CM032588.1	143797	.	C	T	100	*	TYPE=SUBSTITUTE
CM032588.1	143836	.	G	A	100	*	TYPE=SUBSTITUTE
CM032588.1	143899	.	A	G	100	*	TYPE=SUBSTITUTE
CM032588.1	143902	.	T	G	100	*	TYPE=SUBSTITUTE
CM032588.1	143911	.	T	C	100	*	TYPE=SUBSTITUTE
CM032588.1	143929	.	C	T	100	*	TYPE=SUBSTITUTE
CM032588.1	143935	.	G	C	100	*	TYPE=SUBSTITUTE
CM032588.1	143938	.	A	G	100	*	TYPE=SUBSTITUTE
CM032588.1	143953	.	C	G	100	*	TYPE=SUBSTITUTE
CM032588.1	143961	.	G	A	100	*	TYPE=SUBSTITUTE
CM032588.1	143962	.	C	T	100	*	TYPE=SUBSTITUTE
CM032588.1	143972	.	A	G	100	*	TYPE=SUBSTITUTE
CM032588.1	143975	.	T	G	100	*	TYPE=SUBSTITUTE
CM032588.1	143978	.	G	C	100	*	TYPE=SUBSTITUTE
CM032588.1	143980	.	T	G	100	*	TYPE=SUBSTITUTE
CM032588.1	143987	.	T	C	100	*	TYPE=SUBSTITUTE
CM032588.1	143989	.	A	AGG	100	*	TYPE=INSERT
CM032588.1	144000	.	T	G	100	*	TYPE=SUBSTITUTE
CM032588.1	144022	.	C	A	100	*	TYPE=SUBSTITUTE
CM032588.1	144034	.	G	A	100	*	TYPE=SUBSTITUTE
CM032588.1	144052	.	A	G	100	*	TYPE=SUBSTITUTE
CM032588.1	144055	.	C	T	100	*	TYPE=SUBSTITUTE
CM032588.1	144064	.	T	A	100	*	TYPE=SUBSTITUTE
CM032588.1	144070	.	A	G	100	*	TYPE=SUBSTITUTE
CM032588.1	144073	.	G	A	100	*	TYPE=SUBSTITUTE
CM032588.1	144076	.	A	G	100	*	TYPE=SUBSTITUTE
CM032588.1	144087	.	A	G	100	*	TYPE=SUBSTITUTE
CM032588.1	144106	.	A	G	100	*	TYPE=SUBSTITUTE
CM032588.1	144121	.	T	C	100	*	TYPE=SUBSTITUTE
CM032588.1	144146	.	G	A	100	*	TYPE=SUBSTITUTE
CM032588.1	144181	.	C	T	100	*	TYPE=SUBSTITUTE
CM032588.1	144212	.	G	T	100	*	TYPE=SUBSTITUTE
CM032588.1	144236	.	c	cagcaac	100	*	TYPE=INSERT
CM032588.1	144250	.	C	T	100	*	TYPE=SUBSTITUTE
CM032588.1	144265	.	T	A	100	*	TYPE=SUBSTITUTE
CM032588.1	144268	.	G	A	100	*	TYPE=SUBSTITUTE
CM032588.1	144280	.	C	T	100	*	TYPE=SUBSTITUTE
CM032588.1	144301	.	C	T	100	*	TYPE=SUBSTITUTE
CM032588.1	144307	.	C	A	100	*	TYPE=SUBSTITUTE
CM032588.1	144313	.	A	G	100	*	TYPE=SUBSTITUTE
CM032588.1	144316	.	C	A	100	*	TYPE=SUBSTITUTE
CM032588.1	144340	.	C	T	100	*	TYPE=SUBSTITUTE
CM032588.1	144343	.	C	T	100	*	TYPE=SUBSTITUTE
CM032588.1	144364	.	C	t	100	*	TYPE=SUBSTITUTE
CM032588.1	144367	.	C	t	100	*	TYPE=SUBSTITUTE
CM032588.1	144382	.	T	C	100	*	TYPE=SUBSTITUTE
CM032588.1	144415	.	T	c	100	*	TYPE=SUBSTITUTE
CM032588.1	144425	.	A	G	100	*	TYPE=SUBSTITUTE
CM032588.1	144446	.	G	C	100	*	TYPE=SUBSTITUTE
CM032588.1	144499	.	C	T	100	*	TYPE=SUBSTITUTE
CM032588.1	144509	.	A	C	100	*	TYPE=SUBSTITUTE
CM032588.1	144526	.	T	C	100	*	TYPE=SUBSTITUTE
CM032588.1	144534	.	A	G	100	*	TYPE=SUBSTITUTE
CM032588.1	144536	.	A	G	100	*	TYPE=SUBSTITUTE

CM032588.1	144541	.	T	A	100	*	TYPE=SUBSTITUTE
CM032588.1	144545	.	C	G	100	*	TYPE=SUBSTITUTE
CM032588.1	144546	.	C	G	100	*	TYPE=SUBSTITUTE
CM032588.1	144574	.	C	T	100	*	TYPE=SUBSTITUTE
CM032588.1	144598	.	T	A	100	*	TYPE=SUBSTITUTE
CM032588.1	144643	.	G	C	100	*	TYPE=SUBSTITUTE
CM032588.1	144688	.	T	C	100	*	TYPE=SUBSTITUTE
CM032588.1	144692	.	T	C	100	*	TYPE=SUBSTITUTE
CM032588.1	144718	.	T	C	100	*	TYPE=SUBSTITUTE
CM032588.1	144733	.	T	C	100	*	TYPE=SUBSTITUTE
CM032588.1	144748	.	A	T	100	*	TYPE=SUBSTITUTE
CM032588.1	144751	.	A	G	100	*	TYPE=SUBSTITUTE
CM032588.1	144757	.	A	T	100	*	TYPE=SUBSTITUTE
CM032588.1	144805	.	C	A	100	*	TYPE=SUBSTITUTE
CM032588.1	144835	.	C	A	100	*	TYPE=SUBSTITUTE
CM032588.1	144841	.	T	C	100	*	TYPE=SUBSTITUTE
CM032588.1	144850	.	T	C	100	*	TYPE=SUBSTITUTE
CM032588.1	144883	.	A	C	100	*	TYPE=SUBSTITUTE
CM032588.1	144892	.	C	A	100	*	TYPE=SUBSTITUTE
CM032588.1	144913	.	G	A	100	*	TYPE=SUBSTITUTE
CM032588.1	144931	.	T	C	100	*	TYPE=SUBSTITUTE
CM032588.1	144946	.	C	A	100	*	TYPE=SUBSTITUTE
CM032588.1	144973	.	A	T	100	*	TYPE=SUBSTITUTE
CM032588.1	145015	.	G	A	100	*	TYPE=SUBSTITUTE
CM032588.1	145018	.	G	A	100	*	TYPE=SUBSTITUTE
CM032588.1	145111	.	T	C	100	*	TYPE=SUBSTITUTE
CM032588.1	145210	.	T	A	100	*	TYPE=SUBSTITUTE
CM032588.1	145222	.	G	A	100	*	TYPE=SUBSTITUTE
CM032588.1	145285	.	A	G	100	*	TYPE=SUBSTITUTE
CM032588.1	145294	.	C	T	100	*	TYPE=SUBSTITUTE
CM032588.1	145318	.	T	c	100	*	TYPE=SUBSTITUTE
CM032588.1	145361	.	C	A	100	*	TYPE=SUBSTITUTE
CM032588.1	145378	.	A	G	100	*	TYPE=SUBSTITUTE
CM032588.1	145417	.	A	G	100	*	TYPE=SUBSTITUTE
CM032588.1	145420	.	C	T	100	*	TYPE=SUBSTITUTE
CM032588.1	145424	.	C	T	100	*	TYPE=SUBSTITUTE
CM032588.1	145429	.	A	G	100	*	TYPE=SUBSTITUTE
CM032588.1	145450	.	C	A	100	*	TYPE=SUBSTITUTE
CM032588.1	145483	.	G	A	100	*	TYPE=SUBSTITUTE
CM032588.1	145499	.	A	C	100	*	TYPE=SUBSTITUTE
CM032588.1	145500	.	C	T	100	*	TYPE=SUBSTITUTE
CM032588.1	145508	.	C	G	100	*	TYPE=SUBSTITUTE
CM032588.1	145510	.	A	C	100	*	TYPE=SUBSTITUTE
CM032588.1	145511	.	C	T	100	*	TYPE=SUBSTITUTE
CM032588.1	145518	.	AATT	A	100	*	TYPE=DELETE
CM032588.1	145522	.	A	T	100	*	TYPE=SUBSTITUTE
CM032588.1	145525	.	T	C	100	*	TYPE=SUBSTITUTE
CM032588.1	145527	.	G	C	100	*	TYPE=SUBSTITUTE
CM032588.1	145531	.	G	T	100	*	TYPE=SUBSTITUTE
CM032588.1	145540	.	A	T	100	*	TYPE=SUBSTITUTE
CM032588.1	145547	.	G	A	100	*	TYPE=SUBSTITUTE
CM032588.1	145568	.	T	c	100	*	TYPE=SUBSTITUTE
CM032588.1	145571	.	A	g	100	*	TYPE=SUBSTITUTE
CM032588.1	145589	.	A	G	100	*	TYPE=SUBSTITUTE
CM032588.1	145628	.	T	C	100	*	TYPE=SUBSTITUTE
CM032588.1	145664	.	T	C	100	*	TYPE=SUBSTITUTE
CM032588.1	145685	.	C	T	100	*	TYPE=SUBSTITUTE
CM032588.1	145709	.	C	T	100	*	TYPE=SUBSTITUTE
CM032588.1	145727	.	C	T	100	*	TYPE=SUBSTITUTE
CM032588.1	145733	.	A	G	100	*	TYPE=SUBSTITUTE
CM032588.1	145745	.	G	A	100	*	TYPE=SUBSTITUTE
CM032588.1	145772	.	T	C	100	*	TYPE=SUBSTITUTE
CM032588.1	145801	.	GA	G	100	*	TYPE=DELETE
CM032588.1	145814	.	T	C	100	*	TYPE=SUBSTITUTE

CM032588.1	145838	.	A	G	100	*	TYPE=SUBSTITUTE
CM032588.1	145845	.	A	G	100	*	TYPE=SUBSTITUTE
CM032588.1	145958	.	G	T	100	*	TYPE=SUBSTITUTE
CM032588.1	146015	.	C	A	100	*	TYPE=SUBSTITUTE
CM032588.1	146047	.	G	A	100	*	TYPE=SUBSTITUTE
CM032588.1	146051	.	A	G	100	*	TYPE=SUBSTITUTE
CM032588.1	146057	.	A	G	100	*	TYPE=SUBSTITUTE
CM032588.1	146072	.	C	T	100	*	TYPE=SUBSTITUTE
CM032588.1	146087	.	G	A	100	*	TYPE=SUBSTITUTE
CM032588.1	146091	.	A	G	100	*	TYPE=SUBSTITUTE
CM032588.1	146093	.	T	C	100	*	TYPE=SUBSTITUTE
CM032588.1	146104	.	C	T	100	*	TYPE=SUBSTITUTE
CM032588.1	146115	.	G	A	100	*	TYPE=SUBSTITUTE
CM032588.1	146117	.	A	T	100	*	TYPE=SUBSTITUTE
CM032588.1	146120	.	A	T	100	*	TYPE=SUBSTITUTE
CM032588.1	146122	.	C	T	100	*	TYPE=SUBSTITUTE
CM032588.1	146133	.	A	T	100	*	TYPE=SUBSTITUTE
CM032588.1	146142	.	C	G	100	*	TYPE=SUBSTITUTE
CM032588.1	146145	.	A	T	100	*	TYPE=SUBSTITUTE
CM032588.1	146152	.	ACATG	A	100	*	TYPE=DELETE
CM032588.1	146160	.	T	G	100	*	TYPE=SUBSTITUTE
CM032588.1	146171	.	C	A	100	*	TYPE=SUBSTITUTE
CM032588.1	146190	.	C	CG	100	*	TYPE=INSERT
CM032588.1	146191	.	A	G	100	*	TYPE=SUBSTITUTE
CM032588.1	146192	.	A	G	100	*	TYPE=SUBSTITUTE
CM032588.1	146194	.	C	T	100	*	TYPE=SUBSTITUTE
CM032588.1	146195	.	C	A	100	*	TYPE=SUBSTITUTE
CM032588.1	146203	.	T	C	100	*	TYPE=SUBSTITUTE
CM032588.1	146206	.	A	G	100	*	TYPE=SUBSTITUTE
CM032588.1	146210	.	T	C	100	*	TYPE=SUBSTITUTE
CM032588.1	146211	.	G	A	100	*	TYPE=SUBSTITUTE
CM032588.1	146245	.	C	CCTAC	100	*	TYPE=INSERT
CM032588.1	146252	.	T	C	100	*	TYPE=SUBSTITUTE
CM032588.1	146269	.	A	T	100	*	TYPE=SUBSTITUTE
CM032588.1	146270	.	G	C	100	*	TYPE=SUBSTITUTE
CM032588.1	146271	.	T	C	100	*	TYPE=SUBSTITUTE
CM032588.1	146272	.	A	T	100	*	TYPE=SUBSTITUTE
CM032588.1	146282	.	A	AT	100	*	TYPE=INSERT
CM032588.1	146326	.	TT	T	100	*	TYPE=DELETE
CM032588.1	146378	.	T	C	100	*	TYPE=SUBSTITUTE
CM032588.1	146381	.	A	G	100	*	TYPE=SUBSTITUTE
CM032588.1	146382	.	G	C	100	*	TYPE=SUBSTITUTE
CM032588.1	146385	.	G	A	100	*	TYPE=SUBSTITUTE
CM032588.1	146471	.	CC	C	100	*	TYPE=DELETE
CM032588.1	146566	.	A	G	100	*	TYPE=SUBSTITUTE
CM032588.1	146572	.	T	G	100	*	TYPE=SUBSTITUTE
CM032588.1	146577	.	C	a	100	*	TYPE=SUBSTITUTE
CM032588.1	146578	.	A	g	100	*	TYPE=SUBSTITUTE
CM032588.1	146616	.	C	A	100	*	TYPE=SUBSTITUTE
CM032588.1	146618	.	C	T	100	*	TYPE=SUBSTITUTE
CM032588.1	146619	.	T	C	100	*	TYPE=SUBSTITUTE
CM032588.1	146620	.	G	A	100	*	TYPE=SUBSTITUTE
CM032588.1	146657	.	A	C	100	*	TYPE=SUBSTITUTE
CM032588.1	146666	.	T	G	100	*	TYPE=SUBSTITUTE
CM032588.1	146783	.	A	T	100	*	TYPE=SUBSTITUTE
CM032588.1	146788	.	C	G	100	*	TYPE=SUBSTITUTE
CM032588.1	146789	.	A	G	100	*	TYPE=SUBSTITUTE
CM032588.1	146876	.	G	A	100	*	TYPE=SUBSTITUTE
CM032588.1	146906	.	TT	T	100	*	TYPE=DELETE
CM032588.1	147068	.	C	T	100	*	TYPE=SUBSTITUTE
CM032588.1	147200	.	C	T	100	*	TYPE=SUBSTITUTE
CM032588.1	147221	.	G	A	100	*	TYPE=SUBSTITUTE
CM032588.1	147266	.	G	C	100	*	TYPE=SUBSTITUTE
CM032588.1	147347	.	C	T	100	*	TYPE=SUBSTITUTE

CM032588.1	147365	.	A	G	100	*	TYPE=SUBSTITUTE
CM032588.1	147389	.	C	T	100	*	TYPE=SUBSTITUTE
CM032588.1	147428	.	C	T	100	*	TYPE=SUBSTITUTE
CM032588.1	147431	.	T	C	100	*	TYPE=SUBSTITUTE
CM032588.1	147440	.	T	A	100	*	TYPE=SUBSTITUTE
CM032588.1	147488	.	T	G	100	*	TYPE=SUBSTITUTE
CM032588.1	147491	.	A	G	100	*	TYPE=SUBSTITUTE
CM032588.1	147551	.	T	C	100	*	TYPE=SUBSTITUTE
CM032588.1	147593	.	A	C	100	*	TYPE=SUBSTITUTE
CM032588.1	149485	.	C	t	100	*	TYPE=SUBSTITUTE
CM032588.1	149524	.	C	t	100	*	TYPE=SUBSTITUTE
CM032588.1	149527	.	A	c	100	*	TYPE=SUBSTITUTE
CM032588.1	149533	.	G	A	100	*	TYPE=SUBSTITUTE
CM032588.1	149542	.	G	A	100	*	TYPE=SUBSTITUTE
CM032588.1	149710	.	T	C	100	*	TYPE=SUBSTITUTE
CM032588.1	149818	.	C	T	100	*	TYPE=SUBSTITUTE
CM032588.1	149839	.	G	A	100	*	TYPE=SUBSTITUTE
CM032588.1	149857	.	G	T	100	*	TYPE=SUBSTITUTE
CM032588.1	149859	.	TTA	T	100	*	TYPE=DELETE
CM032588.1	149867	.	A	T	100	*	TYPE=SUBSTITUTE
CM032588.1	149872	.	T	A	100	*	TYPE=SUBSTITUTE
CM032588.1	149875	.	G	A	100	*	TYPE=SUBSTITUTE
CM032588.1	149887	.	C	A	100	*	TYPE=SUBSTITUTE
CM032588.1	149899	.	t	tc	100	*	TYPE=INSERT
CM032588.1	149904	.	A	g	100	*	TYPE=SUBSTITUTE
CM032588.1	149909	.	G	t	100	*	TYPE=SUBSTITUTE
CM032588.1	149927	.	CTATAGC	C	100	*	TYPE=DELETE
CM032588.1	149941	.	A	t	100	*	TYPE=SUBSTITUTE
CM032588.1	149951	.	A	g	100	*	TYPE=SUBSTITUTE
CM032588.1	149964	.	C	T	100	*	TYPE=SUBSTITUTE
CM032588.1	149970	.	A	AACT	100	*	TYPE=INSERT
CM032588.1	149971	.	G	A	100	*	TYPE=SUBSTITUTE
CM032588.1	149986	.	C	T	100	*	TYPE=SUBSTITUTE
CM032588.1	150013	.	T	C	100	*	TYPE=SUBSTITUTE
CM032588.1	150027	.	T	C	100	*	TYPE=SUBSTITUTE
CM032588.1	150044	.	T	C	100	*	TYPE=SUBSTITUTE
CM032588.1	150047	.	G	C	100	*	TYPE=SUBSTITUTE
CM032588.1	150099	.	G	A	100	*	TYPE=SUBSTITUTE
CM032588.1	150104	.	A	AGCG	100	*	TYPE=INSERT
CM032588.1	150122	.	A	G	100	*	TYPE=SUBSTITUTE
CM032588.1	150142	.	ATGCGCCGAGAGGA	A	100	*	TYPE=DELETE
CM032588.1	150163	.	TG	T	100	*	TYPE=DELETE
CM032588.1	150174	.	A	C	100	*	TYPE=SUBSTITUTE
CM032588.1	150185	.	G	A	100	*	TYPE=SUBSTITUTE
CM032588.1	150188	.	C	T	100	*	TYPE=SUBSTITUTE
CM032588.1	150191	.	A	G	100	*	TYPE=SUBSTITUTE
CM032588.1	150198	.	G	T	100	*	TYPE=SUBSTITUTE
CM032588.1	150203	.	T	C	100	*	TYPE=SUBSTITUTE
CM032588.1	150210	.	T	C	100	*	TYPE=SUBSTITUTE
CM032588.1	150216	.	t	tta	100	*	TYPE=INSERT
CM032588.1	150223	.	T	a	100	*	TYPE=SUBSTITUTE
CM032588.1	150226	.	T	TCG	100	*	TYPE=INSERT
CM032588.1	150245	.	T	C	100	*	TYPE=SUBSTITUTE
CM032588.1	150255	.	G	A	100	*	TYPE=SUBSTITUTE
CM032588.1	150257	.	C	A	100	*	TYPE=SUBSTITUTE
CM032588.1	150259	.	A	G	100	*	TYPE=SUBSTITUTE
CM032588.1	150260	.	G	A	100	*	TYPE=SUBSTITUTE
CM032588.1	150287	.	G	C	100	*	TYPE=SUBSTITUTE
CM032588.1	150290	.	T	C	100	*	TYPE=SUBSTITUTE
CM032588.1	150291	.	A	G	100	*	TYPE=SUBSTITUTE
CM032588.1	150296	.	G	T	100	*	TYPE=SUBSTITUTE
CM032588.1	150301	.	A	G	100	*	TYPE=SUBSTITUTE
CM032588.1	150339	.	A	G	100	*	TYPE=SUBSTITUTE
CM032588.1	150390	.	G	A	100	*	TYPE=SUBSTITUTE

CM032588.1	150453	.	C	T	100	*	TYPE=SUBSTITUTE
CM032588.1	150454	.	T	C	100	*	TYPE=SUBSTITUTE
CM032588.1	150456	.	C	T	100	*	TYPE=SUBSTITUTE
CM032588.1	150458	.	G	A	100	*	TYPE=SUBSTITUTE
CM032588.1	150460	.	G	T	100	*	TYPE=SUBSTITUTE
CM032588.1	150463	.	G	A	100	*	TYPE=SUBSTITUTE
CM032588.1	150473	.	TATGG	T	100	*	TYPE=DELETE
CM032588.1	150482	.	G	C	100	*	TYPE=SUBSTITUTE
CM032588.1	150517	.	A	G	100	*	TYPE=SUBSTITUTE
CM032588.1	150519	.	A	C	100	*	TYPE=SUBSTITUTE
CM032588.1	150522	.	T	C	100	*	TYPE=SUBSTITUTE
CM032588.1	150524	.	A	T	100	*	TYPE=SUBSTITUTE
CM032588.1	150590	.	T	C	100	*	TYPE=SUBSTITUTE
CM032588.1	150593	.	G	A	100	*	TYPE=SUBSTITUTE
CM032588.1	150634	.	A	g	100	*	TYPE=SUBSTITUTE
CM032588.1	150645	.	G	A	100	*	TYPE=SUBSTITUTE
CM032588.1	150652	.	A	G	100	*	TYPE=SUBSTITUTE
CM032588.1	150661	.	A	G	100	*	TYPE=SUBSTITUTE
CM032588.1	150665	.	T	C	100	*	TYPE=SUBSTITUTE
CM032588.1	150685	.	C	t	100	*	TYPE=SUBSTITUTE
CM032588.1	150692	.	T	c	100	*	TYPE=SUBSTITUTE
CM032588.1	150696	.	T	G	100	*	TYPE=SUBSTITUTE
CM032588.1	150699	.	T	C	100	*	TYPE=SUBSTITUTE
CM032588.1	150713	.	A	C	100	*	TYPE=SUBSTITUTE
CM032588.1	150781	.	C	A	100	*	TYPE=SUBSTITUTE
CM032588.1	150830	.	T	C	100	*	TYPE=SUBSTITUTE
CM032588.1	150866	.	A	G	100	*	TYPE=SUBSTITUTE
CM032588.1	150872	.	C	T	100	*	TYPE=SUBSTITUTE
CM032588.1	150878	.	A	G	100	*	TYPE=SUBSTITUTE
CM032588.1	150907	.	C	T	100	*	TYPE=SUBSTITUTE
CM032588.1	150957	.	A	G	100	*	TYPE=SUBSTITUTE
CM032588.1	150962	.	T	C	100	*	TYPE=SUBSTITUTE
CM032588.1	151000	.	T	C	100	*	TYPE=SUBSTITUTE
CM032588.1	151001	.	T	C	100	*	TYPE=SUBSTITUTE
CM032588.1	151049	.	A	G	100	*	TYPE=SUBSTITUTE
CM032588.1	151102	.	C	A	100	*	TYPE=SUBSTITUTE
CM032588.1	151136	.	T	G	100	*	TYPE=SUBSTITUTE
CM032588.1	151151	.	A	C	100	*	TYPE=SUBSTITUTE
CM032588.1	151152	.	C	T	100	*	TYPE=SUBSTITUTE
CM032588.1	151172	.	T	C	100	*	TYPE=SUBSTITUTE
CM032588.1	151175	.	A	G	100	*	TYPE=SUBSTITUTE
CM032588.1	151181	.	C	T	100	*	TYPE=SUBSTITUTE
CM032588.1	151202	.	A	G	100	*	TYPE=SUBSTITUTE
CM032588.1	151236	.	T	C	100	*	TYPE=SUBSTITUTE
CM032588.1	151238	.	G	A	100	*	TYPE=SUBSTITUTE
CM032588.1	151263	.	A	c	100	*	TYPE=SUBSTITUTE
CM032588.1	151278	.	A	AGCAGCG	100	*	TYPE=INSERT
CM032588.1	151283	.	C	T	100	*	TYPE=SUBSTITUTE
CM032588.1	151316	.	G	A	100	*	TYPE=SUBSTITUTE
CM032588.1	151327	.	G	A	100	*	TYPE=SUBSTITUTE
CM032588.1	151331	.	T	C	100	*	TYPE=SUBSTITUTE
CM032588.1	151336	.	A	G	100	*	TYPE=SUBSTITUTE
CM032588.1	151355	.	A	G	100	*	TYPE=SUBSTITUTE
CM032588.1	151364	.	A	g	100	*	TYPE=SUBSTITUTE
CM032588.1	151369	.	C	t	100	*	TYPE=SUBSTITUTE
CM032588.1	151370	.	A	g	100	*	TYPE=SUBSTITUTE
CM032588.1	151380	.	G	t	100	*	TYPE=SUBSTITUTE
CM032588.1	151385	.	G	A	100	*	TYPE=SUBSTITUTE
CM032588.1	151386	.	G	A	100	*	TYPE=SUBSTITUTE
CM032588.1	151387	.	C	T	100	*	TYPE=SUBSTITUTE
CM032588.1	151420	.	A	G	100	*	TYPE=SUBSTITUTE
CM032588.1	151425	.	T	A	100	*	TYPE=SUBSTITUTE
CM032588.1	151451	.	T	C	100	*	TYPE=SUBSTITUTE
CM032588.1	151456	.	G	T	100	*	TYPE=SUBSTITUTE

CM032588.1	151481	.	A	C	100	*	TYPE=SUBSTITUTE
CM032588.1	151488	.	T	C	100	*	TYPE=SUBSTITUTE
CM032588.1	151490	.	C	T	100	*	TYPE=SUBSTITUTE
CM032588.1	151496	.	T	G	100	*	TYPE=SUBSTITUTE
CM032588.1	151500	.	A	C	100	*	TYPE=SUBSTITUTE
CM032588.1	151513	.	C	T	100	*	TYPE=SUBSTITUTE
CM032588.1	151517	.	T	A	100	*	TYPE=SUBSTITUTE
CM032588.1	151522	.	TC	T	100	*	TYPE=DELETE
CM032588.1	151524	.	C	T	100	*	TYPE=SUBSTITUTE
CM032588.1	151526	.	C	T	100	*	TYPE=SUBSTITUTE
CM032588.1	151636	.	G	A	100	*	TYPE=SUBSTITUTE
CM032588.1	151653	.	G	A	100	*	TYPE=SUBSTITUTE
CM032588.1	151684	.	G	A	100	*	TYPE=SUBSTITUTE
CM032588.1	151703	.	C	T	100	*	TYPE=SUBSTITUTE
CM032588.1	151715	.	ACCTCAAGG	A	100	*	TYPE=DELETE
CM032588.1	151788	.	C	T	100	*	TYPE=SUBSTITUTE
CM032588.1	151843	.	C	G	100	*	TYPE=SUBSTITUTE
CM032588.1	151853	.	G	A	100	*	TYPE=SUBSTITUTE
CM032588.1	151859	.	A	G	100	*	TYPE=SUBSTITUTE
CM032588.1	151860	.	G	A	100	*	TYPE=SUBSTITUTE
CM032588.1	151952	.	G	C	100	*	TYPE=SUBSTITUTE
CM032588.1	151953	.	A	G	100	*	TYPE=SUBSTITUTE
CM032588.1	151955	.	G	A	100	*	TYPE=SUBSTITUTE
CM032588.1	152023	.	T	C	100	*	TYPE=SUBSTITUTE
CM032588.1	152141	.	G	A	100	*	TYPE=SUBSTITUTE
CM032588.1	152153	.	G	T	100	*	TYPE=SUBSTITUTE
CM032588.1	152450	.	CAG	C	100	*	TYPE=DELETE
CM032588.1	152683	.	A	G	100	*	TYPE=SUBSTITUTE
CM032588.1	152725	.	C	t	100	*	TYPE=SUBSTITUTE
CM032588.1	152728	.	T	a	100	*	TYPE=SUBSTITUTE
CM032588.1	152729	.	T	C	100	*	TYPE=SUBSTITUTE
CM032588.1	152824	.	G	A	100	*	TYPE=SUBSTITUTE
CM032588.1	152862	.	A	C	100	*	TYPE=SUBSTITUTE
CM032588.1	152950	.	C	T	100	*	TYPE=SUBSTITUTE
CM032588.1	152964	.	A	C	100	*	TYPE=SUBSTITUTE
CM032588.1	153043	.	T	C	100	*	TYPE=SUBSTITUTE
CM032588.1	153073	.	G	A	100	*	TYPE=SUBSTITUTE
CM032588.1	153124	.	G	A	100	*	TYPE=SUBSTITUTE
CM032588.1	153148	.	T	C	100	*	TYPE=SUBSTITUTE
CM032588.1	153168	.	CC	C	100	*	TYPE=DELETE
CM032588.1	153177	.	T	A	100	*	TYPE=SUBSTITUTE
CM032588.1	153203	.	G	A	100	*	TYPE=SUBSTITUTE
CM032588.1	153212	.	AG	A	100	*	TYPE=DELETE
CM032588.1	153219	.	C	T	100	*	TYPE=SUBSTITUTE
CM032588.1	153222	.	C	T	100	*	TYPE=SUBSTITUTE
CM032588.1	153228	.	G	C	100	*	TYPE=SUBSTITUTE
CM032588.1	153261	.	A	G	100	*	TYPE=SUBSTITUTE
CM032588.1	153345	.	C	A	100	*	TYPE=SUBSTITUTE
CM032588.1	153393	.	A	G	100	*	TYPE=SUBSTITUTE
CM032588.1	153408	.	G	A	100	*	TYPE=SUBSTITUTE
CM032588.1	153579	.	G	C	100	*	TYPE=SUBSTITUTE
CM032588.1	153726	.	G	A	100	*	TYPE=SUBSTITUTE
CM032588.1	153987	.	GT	G	100	*	TYPE=DELETE
CM032588.1	154416	.	A	G	100	*	TYPE=SUBSTITUTE
CM032588.1	154917	.	C	T	100	*	TYPE=SUBSTITUTE
CM032588.1	155015	.	T	C	100	*	TYPE=SUBSTITUTE
CM032588.1	156905	.	A	G	100	*	TYPE=SUBSTITUTE
CM032588.1	158587	.	ACA	A	100	*	TYPE=DELETE
CM032588.1	158651	.	C	A	100	*	TYPE=SUBSTITUTE
CM032588.1	159351	.	G	A	100	*	TYPE=SUBSTITUTE
CM032588.1	160293	.	G	A	100	*	TYPE=SUBSTITUTE
CM032588.1	160325	.	C	G	100	*	TYPE=SUBSTITUTE
CM032588.1	160446	.	C	A	100	*	TYPE=SUBSTITUTE
CM032588.1	161026	.	AGA	A	100	*	TYPE=DELETE

CM032588.1	161385	.	T	C	100	*	TYPE=SUBSTITUTE
CM032588.1	161417	.	C	T	100	*	TYPE=SUBSTITUTE
CM032588.1	161447	.	A	G	100	*	TYPE=SUBSTITUTE
CM032588.1	161468	.	G	A	100	*	TYPE=SUBSTITUTE
CM032588.1	161498	.	C	T	100	*	TYPE=SUBSTITUTE
CM032588.1	161500	.	G	C	100	*	TYPE=SUBSTITUTE
CM032588.1	161504	.	C	T	100	*	TYPE=SUBSTITUTE
CM032588.1	161537	.	C	T	100	*	TYPE=SUBSTITUTE
CM032588.1	161540	.	C	T	100	*	TYPE=SUBSTITUTE
CM032588.1	161570	.	G	A	100	*	TYPE=SUBSTITUTE
CM032588.1	161576	.	T	C	100	*	TYPE=SUBSTITUTE
CM032588.1	161585	.	C	T	100	*	TYPE=SUBSTITUTE
CM032588.1	161594	.	G	A	100	*	TYPE=SUBSTITUTE
CM032588.1	161597	.	C	T	100	*	TYPE=SUBSTITUTE
CM032588.1	161614	.	G	A	100	*	TYPE=SUBSTITUTE
CM032588.1	161640	.	T	C	100	*	TYPE=SUBSTITUTE
CM032588.1	161642	.	G	T	100	*	TYPE=SUBSTITUTE
CM032588.1	161669	.	C	T	100	*	TYPE=SUBSTITUTE
CM032588.1	161695	.	G	A	100	*	TYPE=SUBSTITUTE
CM032588.1	161710	.	G	T	100	*	TYPE=SUBSTITUTE
CM032588.1	161723	.	C	T	100	*	TYPE=SUBSTITUTE
CM032588.1	161727	.	A	G	100	*	TYPE=SUBSTITUTE
CM032588.1	161741	.	G	A	100	*	TYPE=SUBSTITUTE
CM032588.1	161792	.	A	C	100	*	TYPE=SUBSTITUTE
CM032588.1	161798	.	C	T	100	*	TYPE=SUBSTITUTE
CM032588.1	161801	.	G	A	100	*	TYPE=SUBSTITUTE
CM032588.1	161825	.	T	G	100	*	TYPE=SUBSTITUTE
CM032588.1	161828	.	G	A	100	*	TYPE=SUBSTITUTE
CM032588.1	161834	.	A	G	100	*	TYPE=SUBSTITUTE
CM032588.1	161840	.	A	G	100	*	TYPE=SUBSTITUTE
CM032588.1	161846	.	T	C	100	*	TYPE=SUBSTITUTE
CM032588.1	161849	.	C	T	100	*	TYPE=SUBSTITUTE
CM032588.1	161865	.	T	G	100	*	TYPE=SUBSTITUTE
CM032588.1	161876	.	G	A	100	*	TYPE=SUBSTITUTE
CM032588.1	161879	.	G	A	100	*	TYPE=SUBSTITUTE
CM032588.1	161891	.	C	A	100	*	TYPE=SUBSTITUTE
CM032588.1	161893	.	T	G	100	*	TYPE=SUBSTITUTE
CM032588.1	161895	.	A	G	100	*	TYPE=SUBSTITUTE
CM032588.1	161903	.	AG	A	100	*	TYPE=DELETE
CM032588.1	161905	.	C	T	100	*	TYPE=SUBSTITUTE
CM032588.1	161907	.	T	C	100	*	TYPE=SUBSTITUTE
CM032588.1	161910	.	T	C	100	*	TYPE=SUBSTITUTE
CM032588.1	161923	.	A	G	100	*	TYPE=SUBSTITUTE
CM032588.1	161948	.	G	C	100	*	TYPE=SUBSTITUTE
CM032588.1	161970	.	T	C	100	*	TYPE=SUBSTITUTE
CM032588.1	161989	.	T	C	100	*	TYPE=SUBSTITUTE
CM032588.1	162069	.	C	T	100	*	TYPE=SUBSTITUTE
CM032588.1	162081	.	T	C	100	*	TYPE=SUBSTITUTE
CM032588.1	162096	.	A	C	100	*	TYPE=SUBSTITUTE
CM032588.1	162105	.	G	A	100	*	TYPE=SUBSTITUTE
CM032588.1	162122	.	G	A	100	*	TYPE=SUBSTITUTE
CM032588.1	162132	.	A	G	100	*	TYPE=SUBSTITUTE
CM032588.1	162144	.	T	C	100	*	TYPE=SUBSTITUTE
CM032588.1	162174	.	G	A	100	*	TYPE=SUBSTITUTE
CM032588.1	162183	.	C	T	100	*	TYPE=SUBSTITUTE
CM032588.1	162195	.	T	C	100	*	TYPE=SUBSTITUTE
CM032588.1	162213	.	C	T	100	*	TYPE=SUBSTITUTE
CM032588.1	162222	.	C	T	100	*	TYPE=SUBSTITUTE
CM032588.1	162234	.	A	G	100	*	TYPE=SUBSTITUTE
CM032588.1	162237	.	A	G	100	*	TYPE=SUBSTITUTE
CM032588.1	162243	.	T	C	100	*	TYPE=SUBSTITUTE
CM032588.1	162249	.	T	C	100	*	TYPE=SUBSTITUTE
CM032588.1	162255	.	C	T	100	*	TYPE=SUBSTITUTE
CM032588.1	162285	.	T	C	100	*	TYPE=SUBSTITUTE

CM032588.1	162306	.	T	C	100	*	TYPE=SUBSTITUTE
CM032588.1	162325	.	A	G	100	*	TYPE=SUBSTITUTE
CM032588.1	162348	.	A	G	100	*	TYPE=SUBSTITUTE
CM032588.1	162365	.	G	C	100	*	TYPE=SUBSTITUTE
CM032588.1	162369	.	C	T	100	*	TYPE=SUBSTITUTE
CM032588.1	162375	.	T	C	100	*	TYPE=SUBSTITUTE
CM032588.1	162402	.	C	G	100	*	TYPE=SUBSTITUTE
CM032588.1	162423	.	C	T	100	*	TYPE=SUBSTITUTE
CM032588.1	162427	.	A	C	100	*	TYPE=SUBSTITUTE
CM032588.1	162432	.	A	G	100	*	TYPE=SUBSTITUTE
CM032588.1	162444	.	AT	A	100	*	TYPE=DELETE
CM032588.1	162452	.	G	A	100	*	TYPE=SUBSTITUTE
CM032588.1	162459	.	C	T	100	*	TYPE=SUBSTITUTE
CM032588.1	162460	.	A	G	100	*	TYPE=SUBSTITUTE
CM032588.1	162466	.	T	A	100	*	TYPE=SUBSTITUTE
CM032588.1	162468	.	G	T	100	*	TYPE=SUBSTITUTE
CM032588.1	162475	.	C	T	100	*	TYPE=SUBSTITUTE
CM032588.1	162480	.	T	C	100	*	TYPE=SUBSTITUTE
CM032588.1	162488	.	GA	G	100	*	TYPE=DELETE
CM032588.1	162494	.	T	G	100	*	TYPE=SUBSTITUTE
CM032588.1	162512	.	T	G	100	*	TYPE=SUBSTITUTE
CM032588.1	162551	.	C	T	100	*	TYPE=SUBSTITUTE
CM032588.1	162572	.	A	G	100	*	TYPE=SUBSTITUTE
CM032588.1	162576	.	T	C	100	*	TYPE=SUBSTITUTE
CM032588.1	162618	.	T	G	100	*	TYPE=SUBSTITUTE
CM032588.1	162636	.	T	C	100	*	TYPE=SUBSTITUTE
CM032588.1	162641	.	C	A	100	*	TYPE=SUBSTITUTE
CM032588.1	162642	.	G	A	100	*	TYPE=SUBSTITUTE
CM032588.1	162665	.	C	T	100	*	TYPE=SUBSTITUTE
CM032588.1	162670	.	A	G	100	*	TYPE=SUBSTITUTE
CM032588.1	162678	.	G	A	100	*	TYPE=SUBSTITUTE
CM032588.1	162689	.	T	G	100	*	TYPE=SUBSTITUTE
CM032588.1	162712	.	C	CGAC	100	*	TYPE=INSERT
CM032588.1	162716	.	A	T	100	*	TYPE=SUBSTITUTE
CM032588.1	162725	.	T	C	100	*	TYPE=SUBSTITUTE
CM032588.1	162728	.	A	G	100	*	TYPE=SUBSTITUTE
CM032588.1	162733	.	A	G	100	*	TYPE=SUBSTITUTE
CM032588.1	162739	.	C	T	100	*	TYPE=SUBSTITUTE
CM032588.1	162769	.	T	TCCAAcgtaaa	100	*	TYPE=INSERT
CM032588.1	162794	.	GACAA	G	100	*	TYPE=DELETE
CM032588.1	162801	.	C	T	100	*	TYPE=SUBSTITUTE
CM032588.1	162803	.	G	C	100	*	TYPE=SUBSTITUTE
CM032588.1	162805	.	G	A	100	*	TYPE=SUBSTITUTE
CM032588.1	162808	.	T	A	100	*	TYPE=SUBSTITUTE
CM032588.1	162809	.	C	T	100	*	TYPE=SUBSTITUTE
CM032588.1	162821	.	A	G	100	*	TYPE=SUBSTITUTE
CM032588.1	162832	.	G	A	100	*	TYPE=SUBSTITUTE
CM032588.1	162845	.	A	G	100	*	TYPE=SUBSTITUTE
CM032588.1	162851	.	T	C	100	*	TYPE=SUBSTITUTE
CM032588.1	162854	.	A	G	100	*	TYPE=SUBSTITUTE
CM032588.1	162869	.	C	T	100	*	TYPE=SUBSTITUTE
CM032588.1	162875	.	G	A	100	*	TYPE=SUBSTITUTE
CM032588.1	162878	.	G	C	100	*	TYPE=SUBSTITUTE
CM032588.1	162881	.	G	A	100	*	TYPE=SUBSTITUTE
CM032588.1	162908	.	A	G	100	*	TYPE=SUBSTITUTE
CM032588.1	163040	.	A	G	100	*	TYPE=SUBSTITUTE
CM032588.1	163049	.	G	A	100	*	TYPE=SUBSTITUTE
CM032588.1	163088	.	T	C	100	*	TYPE=SUBSTITUTE
CM032588.1	163097	.	G	A	100	*	TYPE=SUBSTITUTE
CM032588.1	163150	.	G	A	100	*	TYPE=SUBSTITUTE
CM032588.1	163236	.	T	C	100	*	TYPE=SUBSTITUTE
CM032588.1	163262	.	A	G	100	*	TYPE=SUBSTITUTE
CM032588.1	163325	.	T	C	100	*	TYPE=SUBSTITUTE
CM032588.1	163463	.	C	T	100	*	TYPE=SUBSTITUTE

CM032588.1	163604	.	G	A	100	*	TYPE=SUBSTITUTE
CM032588.1	163808	.	G	A	100	*	TYPE=SUBSTITUTE
CM032588.1	163809	.	T	C	100	*	TYPE=SUBSTITUTE
CM032588.1	163811	.	A	G	100	*	TYPE=SUBSTITUTE
CM032588.1	163838	.	A	G	100	*	TYPE=SUBSTITUTE
CM032588.1	163841	.	T	G	100	*	TYPE=SUBSTITUTE
CM032588.1	163844	.	C	T	100	*	TYPE=SUBSTITUTE
CM032588.1	163919	.	A	G	100	*	TYPE=SUBSTITUTE
CM032588.1	163931	.	A	G	100	*	TYPE=SUBSTITUTE
CM032588.1	163955	.	T	G	100	*	TYPE=SUBSTITUTE
CM032588.1	163985	.	G	A	100	*	TYPE=SUBSTITUTE
CM032588.1	163997	.	G	A	100	*	TYPE=SUBSTITUTE
CM032588.1	164042	.	A	G	100	*	TYPE=SUBSTITUTE
CM032588.1	164045	.	A	G	100	*	TYPE=SUBSTITUTE
CM032588.1	164072	.	G	T	100	*	TYPE=SUBSTITUTE
CM032588.1	164075	.	A	T	100	*	TYPE=SUBSTITUTE
CM032588.1	164078	.	T	C	100	*	TYPE=SUBSTITUTE
CM032588.1	164116	.	A	G	100	*	TYPE=SUBSTITUTE
CM032588.1	164150	.	C	T	100	*	TYPE=SUBSTITUTE
CM032588.1	164255	.	T	C	100	*	TYPE=SUBSTITUTE
CM032588.1	164270	.	A	G	100	*	TYPE=SUBSTITUTE
CM032588.1	164285	.	A	G	100	*	TYPE=SUBSTITUTE
CM032588.1	164291	.	G	A	100	*	TYPE=SUBSTITUTE
CM032588.1	164297	.	T	C	100	*	TYPE=SUBSTITUTE
CM032588.1	164363	.	T	A	100	*	TYPE=SUBSTITUTE
CM032588.1	164365	.	C	T	100	*	TYPE=SUBSTITUTE
CM032588.1	164385	.	A	G	100	*	TYPE=SUBSTITUTE
CM032588.1	164401	.	G	a	100	*	TYPE=SUBSTITUTE
CM032588.1	164414	.	TC	T	100	*	TYPE=DELETE
CM032588.1	164419	.	T	c	100	*	TYPE=SUBSTITUTE
CM032588.1	164426	.	G	a	100	*	TYPE=SUBSTITUTE
CM032588.1	164440	.	C	T	100	*	TYPE=SUBSTITUTE
CM032588.1	164493	.	A	G	100	*	TYPE=SUBSTITUTE
CM032588.1	164507	.	T	G	100	*	TYPE=SUBSTITUTE
CM032588.1	164523	.	G	A	100	*	TYPE=SUBSTITUTE
CM032588.1	164524	.	T	C	100	*	TYPE=SUBSTITUTE
CM032588.1	164526	.	G	A	100	*	TYPE=SUBSTITUTE
CM032588.1	164558	.	CTC	C	100	*	TYPE=DELETE
CM032588.1	164582	.	T	c	100	*	TYPE=SUBSTITUTE
CM032588.1	164594	.	T	c	100	*	TYPE=SUBSTITUTE
CM032588.1	164606	.	G	C	100	*	TYPE=SUBSTITUTE
CM032588.1	164612	.	A	G	100	*	TYPE=SUBSTITUTE
CM032588.1	164617	.	CA	C	100	*	TYPE=DELETE
CM032588.1	164626	.	C	G	100	*	TYPE=SUBSTITUTE
CM032588.1	164801	.	T	g	100	*	TYPE=SUBSTITUTE
CM032588.1	164806	.	G	A	100	*	TYPE=SUBSTITUTE
CM032588.1	164817	.	A	G	100	*	TYPE=SUBSTITUTE
CM032588.1	164820	.	T	A	100	*	TYPE=SUBSTITUTE
CM032588.1	164866	.	A	T	100	*	TYPE=SUBSTITUTE
CM032588.1	164884	.	C	T	100	*	TYPE=SUBSTITUTE
CM032588.1	164895	.	C	T	100	*	TYPE=SUBSTITUTE
CM032588.1	164899	.	T	C	100	*	TYPE=SUBSTITUTE
CM032588.1	164902	.	A	C	100	*	TYPE=SUBSTITUTE
CM032588.1	164919	.	A	C	100	*	TYPE=SUBSTITUTE
CM032588.1	164934	.	T	G	100	*	TYPE=SUBSTITUTE
CM032588.1	164937	.	C	T	100	*	TYPE=SUBSTITUTE
CM032588.1	164940	.	T	C	100	*	TYPE=SUBSTITUTE
CM032588.1	164944	.	T	C	100	*	TYPE=SUBSTITUTE
CM032588.1	164945	.	G	A	100	*	TYPE=SUBSTITUTE
CM032588.1	164949	.	T	A	100	*	TYPE=SUBSTITUTE
CM032588.1	164950	.	T	C	100	*	TYPE=SUBSTITUTE
CM032588.1	164962	.	G	a	100	*	TYPE=SUBSTITUTE
CM032588.1	164975	.	C	t	100	*	TYPE=SUBSTITUTE
CM032588.1	165019	.	C	T	100	*	TYPE=SUBSTITUTE

CM032588.1	165041	.	T	A	100	*	TYPE=SUBSTITUTE
CM032588.1	165044	.	A	G	100	*	TYPE=SUBSTITUTE
CM032588.1	165045	.	C	A	100	*	TYPE=SUBSTITUTE
CM032588.1	165066	.	C	G	100	*	TYPE=SUBSTITUTE
CM032588.1	165148	.	AA	A	100	*	TYPE=DELETE
CM032588.1	165256	.	G	GGG	100	*	TYPE=INSERT
CM032588.1	165323	.	C	T	100	*	TYPE=SUBSTITUTE
CM032588.1	165345	.	C	T	100	*	TYPE=SUBSTITUTE
CM032588.1	165362	.	T	C	100	*	TYPE=SUBSTITUTE
CM032588.1	165370	.	T	C	100	*	TYPE=SUBSTITUTE
CM032588.1	165380	.	G	A	100	*	TYPE=SUBSTITUTE
CM032588.1	165411	.	C	T	100	*	TYPE=SUBSTITUTE
CM032588.1	165491	.	G	C	100	*	TYPE=SUBSTITUTE
CM032588.1	165504	.	C	T	100	*	TYPE=SUBSTITUTE
CM032588.1	165511	.	AG	A	100	*	TYPE=DELETE
CM032588.1	165526	.	A	G	100	*	TYPE=SUBSTITUTE
CM032588.1	165533	.	C	A	100	*	TYPE=SUBSTITUTE
CM032588.1	165551	.	G	A	100	*	TYPE=SUBSTITUTE
CM032588.1	165561	.	G	A	100	*	TYPE=SUBSTITUTE
CM032588.1	165569	.	C	T	100	*	TYPE=SUBSTITUTE
CM032588.1	165571	.	A	C	100	*	TYPE=SUBSTITUTE
CM032588.1	165573	.	A	G	100	*	TYPE=SUBSTITUTE
CM032588.1	165582	.	C	T	100	*	TYPE=SUBSTITUTE
CM032588.1	165588	.	C	T	100	*	TYPE=SUBSTITUTE
CM032588.1	165600	.	G	C	100	*	TYPE=SUBSTITUTE
CM032588.1	165614	.	C	A	100	*	TYPE=SUBSTITUTE
CM032588.1	165625	.	C	T	100	*	TYPE=SUBSTITUTE
CM032588.1	165627	.	T	C	100	*	TYPE=SUBSTITUTE
CM032588.1	165649	.	C	G	100	*	TYPE=SUBSTITUTE
CM032588.1	165662	.	G	t	100	*	TYPE=SUBSTITUTE
CM032588.1	165664	.	A	g	100	*	TYPE=SUBSTITUTE
CM032588.1	165669	.	G	a	100	*	TYPE=SUBSTITUTE
CM032588.1	165708	.	T	A	100	*	TYPE=SUBSTITUTE
CM032588.1	165715	.	T	C	100	*	TYPE=SUBSTITUTE
CM032588.1	165735	.	G	T	100	*	TYPE=SUBSTITUTE
CM032588.1	165737	.	g	gatgtat	100	*	TYPE=INSERT
CM032588.1	165739	.	A	t	100	*	TYPE=SUBSTITUTE
CM032588.1	165749	.	C	A	100	*	TYPE=SUBSTITUTE
CM032588.1	165753	.	G	T	100	*	TYPE=SUBSTITUTE
CM032588.1	165754	.	G	C	100	*	TYPE=SUBSTITUTE
CM032588.1	165768	.	CTT	C	100	*	TYPE=DELETE
CM032588.1	165775	.	A	T	100	*	TYPE=SUBSTITUTE
CM032588.1	165779	.	G	A	100	*	TYPE=SUBSTITUTE
CM032588.1	165780	.	T	C	100	*	TYPE=SUBSTITUTE
CM032588.1	165782	.	T	C	100	*	TYPE=SUBSTITUTE
CM032588.1	165786	.	A	G	100	*	TYPE=SUBSTITUTE
CM032588.1	165787	.	G	C	100	*	TYPE=SUBSTITUTE
CM032588.1	165793	.	G	A	100	*	TYPE=SUBSTITUTE
CM032588.1	165800	.	C	G	100	*	TYPE=SUBSTITUTE
CM032588.1	165808	.	A	G	100	*	TYPE=SUBSTITUTE
CM032588.1	165813	.	T	C	100	*	TYPE=SUBSTITUTE
CM032588.1	165815	.	T	C	100	*	TYPE=SUBSTITUTE
CM032588.1	165816	.	A	G	100	*	TYPE=SUBSTITUTE
CM032588.1	165821	.	G	A	100	*	TYPE=SUBSTITUTE
CM032588.1	165824	.	T	C	100	*	TYPE=SUBSTITUTE
CM032588.1	165841	.	GC	G	100	*	TYPE=DELETE
CM032588.1	165844	.	A	T	100	*	TYPE=SUBSTITUTE
CM032588.1	165849	.	A	G	100	*	TYPE=SUBSTITUTE
CM032588.1	165856	.	C	T	100	*	TYPE=SUBSTITUTE
CM032588.1	165857	.	A	G	100	*	TYPE=SUBSTITUTE
CM032588.1	165860	.	G	A	100	*	TYPE=SUBSTITUTE
CM032588.1	165862	.	C	A	100	*	TYPE=SUBSTITUTE
CM032588.1	165870	.	G	T	100	*	TYPE=SUBSTITUTE
CM032588.1	165873	.	C	T	100	*	TYPE=SUBSTITUTE

CM032588.1	165877	.	C	CCAGCTG	100	*	TYPE=INSERT
CM032588.1	165883	.	C	G	100	*	TYPE=SUBSTITUTE
CM032588.1	165889	.	A	T	100	*	TYPE=SUBSTITUTE
CM032588.1	165894	.	A	G	100	*	TYPE=SUBSTITUTE
CM032588.1	165897	.	C	T	100	*	TYPE=SUBSTITUTE
CM032588.1	165907	.	G	A	100	*	TYPE=SUBSTITUTE
CM032588.1	165915	.	A	G	100	*	TYPE=SUBSTITUTE
CM032588.1	165917	.	T	C	100	*	TYPE=SUBSTITUTE
CM032588.1	165923	.	C	T	100	*	TYPE=SUBSTITUTE
CM032588.1	165924	.	C	T	100	*	TYPE=SUBSTITUTE
CM032588.1	165927	.	T	G	100	*	TYPE=SUBSTITUTE
CM032588.1	165934	.	T	A	100	*	TYPE=SUBSTITUTE
CM032588.1	165941	.	A	T	100	*	TYPE=SUBSTITUTE
CM032588.1	165949	.	A	G	100	*	TYPE=SUBSTITUTE
CM032588.1	165950	.	T	A	100	*	TYPE=SUBSTITUTE
CM032588.1	165959	.	C	A	100	*	TYPE=SUBSTITUTE
CM032588.1	165965	.	T	A	100	*	TYPE=SUBSTITUTE
CM032588.1	165970	.	G	A	100	*	TYPE=SUBSTITUTE
CM032588.1	165971	.	C	G	100	*	TYPE=SUBSTITUTE
CM032588.1	165990	.	T	C	100	*	TYPE=SUBSTITUTE
CM032588.1	165995	.	G	A	100	*	TYPE=SUBSTITUTE
CM032588.1	165997	.	G	A	100	*	TYPE=SUBSTITUTE
CM032588.1	166003	.	C	T	100	*	TYPE=SUBSTITUTE
CM032588.1	166006	.	G	A	100	*	TYPE=SUBSTITUTE
CM032588.1	166022	.	A	C	100	*	TYPE=SUBSTITUTE
CM032588.1	166037	.	G	A	100	*	TYPE=SUBSTITUTE
CM032588.1	166040	.	T	C	100	*	TYPE=SUBSTITUTE
CM032588.1	166041	.	A	G	100	*	TYPE=SUBSTITUTE
CM032588.1	166042	.	A	G	100	*	TYPE=SUBSTITUTE
CM032588.1	166045	.	T	C	100	*	TYPE=SUBSTITUTE
CM032588.1	166049	.	A	G	100	*	TYPE=SUBSTITUTE
CM032588.1	166052	.	G	A	100	*	TYPE=SUBSTITUTE
CM032588.1	166056	.	C	T	100	*	TYPE=SUBSTITUTE
CM032588.1	166060	.	C	CTG	100	*	TYPE=INSERT
CM032588.1	166063	.	T	G	100	*	TYPE=SUBSTITUTE
CM032588.1	166072	.	A	G	100	*	TYPE=SUBSTITUTE
CM032588.1	166082	.	G	A	100	*	TYPE=SUBSTITUTE
CM032588.1	166086	.	A	G	100	*	TYPE=SUBSTITUTE
CM032588.1	166088	.	T	C	100	*	TYPE=SUBSTITUTE
CM032588.1	166092	.	G	C	100	*	TYPE=SUBSTITUTE
CM032588.1	166094	.	CAT	C	100	*	TYPE=DELETE
CM032588.1	166105	.	T	C	100	*	TYPE=SUBSTITUTE
CM032588.1	166106	.	C	G	100	*	TYPE=SUBSTITUTE
CM032588.1	166107	.	A	G	100	*	TYPE=SUBSTITUTE
CM032588.1	166108	.	G	A	100	*	TYPE=SUBSTITUTE
CM032588.1	166127	.	G	T	100	*	TYPE=SUBSTITUTE
CM032588.1	166128	.	T	C	100	*	TYPE=SUBSTITUTE
CM032588.1	166136	.	T	A	100	*	TYPE=SUBSTITUTE
CM032588.1	166137	.	C	T	100	*	TYPE=SUBSTITUTE
CM032588.1	166139	.	C	G	100	*	TYPE=SUBSTITUTE
CM032588.1	166160	.	A	T	100	*	TYPE=SUBSTITUTE
CM032588.1	166164	.	C	T	100	*	TYPE=SUBSTITUTE
CM032588.1	166170	.	C	T	100	*	TYPE=SUBSTITUTE
CM032588.1	166178	.	C	CGGCCAGGTACCTCAGAAGTGACTTA	100	*	TYPE=INSERT
CM032588.1	166182	.	T	TGAG	100	*	TYPE=INSERT
CM032588.1	166184	.	A	AGAATTAGAG	100	*	TYPE=INSERT
CM032588.1	166187	.	C	CT	100	*	TYPE=INSERT
CM032588.1	166190	.	T	A	100	*	TYPE=SUBSTITUTE
CM032588.1	166192	.	C	T	100	*	TYPE=SUBSTITUTE
CM032588.1	166194	.	T	G	100	*	TYPE=SUBSTITUTE
CM032588.1	166195	.	T	C	100	*	TYPE=SUBSTITUTE
CM032588.1	166198	.	A	AT	100	*	TYPE=INSERT
CM032588.1	166201	.	A	AGGacttg	100	*	TYPE=INSERT

CM032588.1	166205	.	a	aagtcaag	100	*	TYPE=INSERT
CM032588.1	166207	.	A	t	100	*	TYPE=SUBSTITUTE
CM032588.1	166208	.	A	c	100	*	TYPE=SUBSTITUTE
CM032588.1	166210	.	A	AGCCATACATTCtgc	100	*	TYPE=INSERT
CM032588.1	166214	.	G	a	100	*	TYPE=SUBSTITUTE
CM032588.1	166221	.	G	a	100	*	TYPE=SUBSTITUTE
CM032588.1	166224	.	G	a	100	*	TYPE=SUBSTITUTE
CM032588.1	166227	.	A	g	100	*	TYPE=SUBSTITUTE
CM032588.1	166229	.	a	at	100	*	TYPE=INSERT
CM032588.1	166233	.	A	g	100	*	TYPE=SUBSTITUTE
CM032588.1	166235	.	T	a	100	*	TYPE=SUBSTITUTE
CM032588.1	166243	.	GA	G	100	*	TYPE=DELETE
CM032588.1	166245	.	T	C	100	*	TYPE=SUBSTITUTE
CM032588.1	166252	.	G	A	100	*	TYPE=SUBSTITUTE
CM032588.1	166257	.	C	T	100	*	TYPE=SUBSTITUTE
CM032588.1	166263	.	C	T	100	*	TYPE=SUBSTITUTE
CM032588.1	166282	.	T	G	100	*	TYPE=SUBSTITUTE
CM032588.1	166285	.	G	A	100	*	TYPE=SUBSTITUTE
CM032588.1	166290	.	T	G	100	*	TYPE=SUBSTITUTE
CM032588.1	166292	.	T	C	100	*	TYPE=SUBSTITUTE
CM032588.1	166293	.	A	C	100	*	TYPE=SUBSTITUTE
CM032588.1	166294	.	T	A	100	*	TYPE=SUBSTITUTE
CM032588.1	166299	.	A	G	100	*	TYPE=SUBSTITUTE
CM032588.1	166303	.	T	C	100	*	TYPE=SUBSTITUTE
CM032588.1	166311	.	A	G	100	*	TYPE=SUBSTITUTE
CM032588.1	166313	.	C	CGTATCGTCGT	100	*	TYPE=INSERT
CM032588.1	166314	.	A	T	100	*	TYPE=SUBSTITUTE
CM032588.1	166317	.	C	T	100	*	TYPE=SUBSTITUTE
CM032588.1	166329	.	T	C	100	*	TYPE=SUBSTITUTE
CM032588.1	166341	.	A	G	100	*	TYPE=SUBSTITUTE
CM032588.1	166345	.	A	T	100	*	TYPE=SUBSTITUTE
CM032588.1	166352	.	T	A	100	*	TYPE=SUBSTITUTE
CM032588.1	166354	.	T	G	100	*	TYPE=SUBSTITUTE
CM032588.1	166356	.	C	T	100	*	TYPE=SUBSTITUTE
CM032588.1	166359	.	C	T	100	*	TYPE=SUBSTITUTE
CM032588.1	166361	.	T	C	100	*	TYPE=SUBSTITUTE
CM032588.1	166362	.	A	G	100	*	TYPE=SUBSTITUTE
CM032588.1	166363	.	G	C	100	*	TYPE=SUBSTITUTE
CM032588.1	166364	.	C	A	100	*	TYPE=SUBSTITUTE
CM032588.1	166372	.	T	C	100	*	TYPE=SUBSTITUTE
CM032588.1	166379	.	G	A	100	*	TYPE=SUBSTITUTE
CM032588.1	166382	.	T	G	100	*	TYPE=SUBSTITUTE
CM032588.1	166385	.	A	T	100	*	TYPE=SUBSTITUTE
CM032588.1	166386	.	T	C	100	*	TYPE=SUBSTITUTE
CM032588.1	166388	.	C	G	100	*	TYPE=SUBSTITUTE
CM032588.1	166391	.	T	TC	100	*	TYPE=INSERT
CM032588.1	166393	.	A	G	100	*	TYPE=SUBSTITUTE
CM032588.1	166398	.	A	C	100	*	TYPE=SUBSTITUTE
CM032588.1	166400	.	T	C	100	*	TYPE=SUBSTITUTE
CM032588.1	166408	.	G	C	100	*	TYPE=SUBSTITUTE
CM032588.1	166418	.	G	A	100	*	TYPE=SUBSTITUTE
CM032588.1	166423	.	G	T	100	*	TYPE=SUBSTITUTE
CM032588.1	166425	.	T	C	100	*	TYPE=SUBSTITUTE
CM032588.1	166427	.	G	A	100	*	TYPE=SUBSTITUTE
CM032588.1	166445	.	A	G	100	*	TYPE=SUBSTITUTE
CM032588.1	166455	.	G	A	100	*	TYPE=SUBSTITUTE
CM032588.1	166463	.	A	C	100	*	TYPE=SUBSTITUTE
CM032588.1	166488	.	C	T	100	*	TYPE=SUBSTITUTE
CM032588.1	166492	.	G	A	100	*	TYPE=SUBSTITUTE
CM032588.1	166498	.	A	G	100	*	TYPE=SUBSTITUTE
CM032588.1	166503	.	T	C	100	*	TYPE=SUBSTITUTE
CM032588.1	166507	.	G	A	100	*	TYPE=SUBSTITUTE
CM032588.1	166511	.	G	C	100	*	TYPE=SUBSTITUTE
CM032588.1	166512	.	A	C	100	*	TYPE=SUBSTITUTE

CM032588.1	166525	.	A	G	100	*	TYPE=SUBSTITUTE
CM032588.1	166529	.	G	A	100	*	TYPE=SUBSTITUTE
CM032588.1	166531	.	C	T	100	*	TYPE=SUBSTITUTE
CM032588.1	166535	.	C	T	100	*	TYPE=SUBSTITUTE
CM032588.1	166545	.	C	T	100	*	TYPE=SUBSTITUTE
CM032588.1	166553	.	G	C	100	*	TYPE=SUBSTITUTE
CM032588.1	166554	.	TGCACAA	T	100	*	TYPE=DELETE
CM032588.1	166561	.	A	C	100	*	TYPE=SUBSTITUTE
CM032588.1	166564	.	A	G	100	*	TYPE=SUBSTITUTE
CM032588.1	166565	.	T	G	100	*	TYPE=SUBSTITUTE
CM032588.1	166568	.	C	A	100	*	TYPE=SUBSTITUTE
CM032588.1	166569	.	G	GAA	100	*	TYPE=INSERT
CM032588.1	166570	.	T	A	100	*	TYPE=SUBSTITUTE
CM032588.1	166575	.	A	G	100	*	TYPE=SUBSTITUTE
CM032588.1	166576	.	G	A	100	*	TYPE=SUBSTITUTE
CM032588.1	166577	.	A	T	100	*	TYPE=SUBSTITUTE
CM032588.1	166579	.	A	C	100	*	TYPE=SUBSTITUTE
CM032588.1	166587	.	A	G	100	*	TYPE=SUBSTITUTE
CM032588.1	166591	.	C	A	100	*	TYPE=SUBSTITUTE
CM032588.1	166594	.	A	C	100	*	TYPE=SUBSTITUTE
CM032588.1	166596	.	G	A	100	*	TYPE=SUBSTITUTE
CM032588.1	166607	.	T	C	100	*	TYPE=SUBSTITUTE
CM032588.1	166610	.	A	G	100	*	TYPE=SUBSTITUTE
CM032588.1	166623	.	T	A	100	*	TYPE=SUBSTITUTE
CM032588.1	166631	.	G	A	100	*	TYPE=SUBSTITUTE
CM032588.1	166644	.	C	T	100	*	TYPE=SUBSTITUTE
CM032588.1	166647	.	T	C	100	*	TYPE=SUBSTITUTE
CM032588.1	166648	.	A	G	100	*	TYPE=SUBSTITUTE
CM032588.1	166649	.	A	G	100	*	TYPE=SUBSTITUTE
CM032588.1	166659	.	G	a	100	*	TYPE=SUBSTITUTE
CM032588.1	166690	.	T	G	100	*	TYPE=SUBSTITUTE
CM032588.1	166691	.	G	T	100	*	TYPE=SUBSTITUTE
CM032588.1	166707	.	C	T	100	*	TYPE=SUBSTITUTE
CM032588.1	166709	.	C	T	100	*	TYPE=SUBSTITUTE
CM032588.1	166719	.	T	C	100	*	TYPE=SUBSTITUTE
CM032588.1	166727	.	C	A	100	*	TYPE=SUBSTITUTE
CM032588.1	166734	.	T	G	100	*	TYPE=SUBSTITUTE
CM032588.1	166736	.	A	G	100	*	TYPE=SUBSTITUTE
CM032588.1	166739	.	A	G	100	*	TYPE=SUBSTITUTE
CM032588.1	166743	.	A	G	100	*	TYPE=SUBSTITUTE
CM032588.1	166745	.	G	A	100	*	TYPE=SUBSTITUTE
CM032588.1	166757	.	T	C	100	*	TYPE=SUBSTITUTE
CM032588.1	166767	.	A	T	100	*	TYPE=SUBSTITUTE
CM032588.1	166784	.	T	C	100	*	TYPE=SUBSTITUTE
CM032588.1	166789	.	A	G	100	*	TYPE=SUBSTITUTE
CM032588.1	166790	.	T	C	100	*	TYPE=SUBSTITUTE
CM032588.1	166801	.	G	A	100	*	TYPE=SUBSTITUTE
CM032588.1	166805	.	T	C	100	*	TYPE=SUBSTITUTE
CM032588.1	166808	.	C	T	100	*	TYPE=SUBSTITUTE
CM032588.1	166812	.	C	T	100	*	TYPE=SUBSTITUTE
CM032588.1	166820	.	G	A	100	*	TYPE=SUBSTITUTE
CM032588.1	166821	.	A	G	100	*	TYPE=SUBSTITUTE
CM032588.1	166825	.	C	T	100	*	TYPE=SUBSTITUTE
CM032588.1	166836	.	T	C	100	*	TYPE=SUBSTITUTE
CM032588.1	166844	.	T	C	100	*	TYPE=SUBSTITUTE
CM032588.1	166880	.	G	GT	100	*	TYPE=INSERT
CM032588.1	167024	.	G	C	100	*	TYPE=SUBSTITUTE
CM032588.1	167026	.	A	G	100	*	TYPE=SUBSTITUTE
CM032588.1	167043	.	C	G	100	*	TYPE=SUBSTITUTE
CM032588.1	167070	.	AA	A	100	*	TYPE=DELETE
CM032588.1	167093	.	C	T	100	*	TYPE=SUBSTITUTE
CM032588.1	167128	.	C	G	100	*	TYPE=SUBSTITUTE
CM032588.1	167142	.	C	T	100	*	TYPE=SUBSTITUTE
CM032588.1	167143	.	T	C	100	*	TYPE=SUBSTITUTE

CM032588.1	167144	.	C	T	100	*	TYPE=SUBSTITUTE
CM032588.1	167192	.	G	T	100	*	TYPE=SUBSTITUTE
CM032588.1	167204	.	T	C	100	*	TYPE=SUBSTITUTE
CM032588.1	167253	.	G	T	100	*	TYPE=SUBSTITUTE
CM032588.1	167290	.	G	A	100	*	TYPE=SUBSTITUTE
CM032588.1	167306	.	C	T	100	*	TYPE=SUBSTITUTE
CM032588.1	167318	.	A	G	100	*	TYPE=SUBSTITUTE
CM032588.1	167354	.	T	C	100	*	TYPE=SUBSTITUTE
CM032588.1	167363	.	C	T	100	*	TYPE=SUBSTITUTE
CM032588.1	167372	.	C	T	100	*	TYPE=SUBSTITUTE
CM032588.1	167381	.	A	G	100	*	TYPE=SUBSTITUTE
CM032588.1	167453	.	TTTT	T	100	*	TYPE=DELETE
CM032588.1	167457	.	T	G	100	*	TYPE=SUBSTITUTE
CM032588.1	167463	.	T	C	100	*	TYPE=SUBSTITUTE
CM032588.1	167467	.	A	G	100	*	TYPE=SUBSTITUTE
CM032588.1	167487	.	G	A	100	*	TYPE=SUBSTITUTE
CM032588.1	167519	.	A	G	100	*	TYPE=SUBSTITUTE
CM032588.1	167522	.	C	T	100	*	TYPE=SUBSTITUTE
CM032588.1	167532	.	C	A	100	*	TYPE=SUBSTITUTE
CM032588.1	167537	.	CA	C	100	*	TYPE=DELETE
CM032588.1	167556	.	A	G	100	*	TYPE=SUBSTITUTE
CM032588.1	167583	.	C	T	100	*	TYPE=SUBSTITUTE
CM032588.1	167626	.	A	G	100	*	TYPE=SUBSTITUTE
CM032588.1	167646	.	C	A	100	*	TYPE=SUBSTITUTE
CM032588.1	167658	.	C	A	100	*	TYPE=SUBSTITUTE
CM032588.1	167661	.	C	T	100	*	TYPE=SUBSTITUTE
CM032588.1	167698	.	A	C	100	*	TYPE=SUBSTITUTE
CM032588.1	167701	.	G	A	100	*	TYPE=SUBSTITUTE
CM032588.1	167727	.	A	G	100	*	TYPE=SUBSTITUTE
CM032588.1	167733	.	C	T	100	*	TYPE=SUBSTITUTE
CM032588.1	167739	.	G	A	100	*	TYPE=SUBSTITUTE
CM032588.1	167763	.	T	C	100	*	TYPE=SUBSTITUTE
CM032588.1	167796	.	A	G	100	*	TYPE=SUBSTITUTE
CM032588.1	167802	.	G	A	100	*	TYPE=SUBSTITUTE
CM032588.1	167804	.	C	T	100	*	TYPE=SUBSTITUTE
CM032588.1	167902	.	G	A	100	*	TYPE=SUBSTITUTE
CM032588.1	167922	.	C	t	100	*	TYPE=SUBSTITUTE
CM032588.1	167928	.	G	a	100	*	TYPE=SUBSTITUTE
CM032588.1	167934	.	G	T	100	*	TYPE=SUBSTITUTE
CM032588.1	167950	.	G	A	100	*	TYPE=SUBSTITUTE
CM032588.1	167952	.	G	A	100	*	TYPE=SUBSTITUTE
CM032588.1	167956	.	G	C	100	*	TYPE=SUBSTITUTE
CM032588.1	167966	.	A	G	100	*	TYPE=SUBSTITUTE
CM032588.1	167972	.	A	C	100	*	TYPE=SUBSTITUTE
CM032588.1	168001	.	C	T	100	*	TYPE=SUBSTITUTE
CM032588.1	168009	.	C	T	100	*	TYPE=SUBSTITUTE
CM032588.1	168013	.	A	G	100	*	TYPE=SUBSTITUTE
CM032588.1	168019	.	G	A	100	*	TYPE=SUBSTITUTE
CM032588.1	168024	.	C	T	100	*	TYPE=SUBSTITUTE
CM032588.1	168030	.	T	C	100	*	TYPE=SUBSTITUTE
CM032588.1	168034	.	T	C	100	*	TYPE=SUBSTITUTE
CM032588.1	168064	.	T	A	100	*	TYPE=SUBSTITUTE
CM032588.1	168068	.	GA	G	100	*	TYPE=DELETE
CM032588.1	168098	.	G	A	100	*	TYPE=SUBSTITUTE
CM032588.1	168117	.	A	T	100	*	TYPE=SUBSTITUTE
CM032588.1	168121	.	A	G	100	*	TYPE=SUBSTITUTE
CM032588.1	168123	.	T	C	100	*	TYPE=SUBSTITUTE
CM032588.1	168124	.	T	C	100	*	TYPE=SUBSTITUTE
CM032588.1	168125	.	G	A	100	*	TYPE=SUBSTITUTE
CM032588.1	168129	.	T	C	100	*	TYPE=SUBSTITUTE
CM032588.1	168157	.	C	A	100	*	TYPE=SUBSTITUTE
CM032588.1	168160	.	T	A	100	*	TYPE=SUBSTITUTE
CM032588.1	168171	.	A	G	100	*	TYPE=SUBSTITUTE
CM032588.1	168191	.	G	C	100	*	TYPE=SUBSTITUTE

CM032588.1	168193	.	T	C	100	*	TYPE=SUBSTITUTE
CM032588.1	168197	.	C	T	100	*	TYPE=SUBSTITUTE
CM032588.1	168202	.	C	T	100	*	TYPE=SUBSTITUTE
CM032588.1	168211	.	T	G	100	*	TYPE=SUBSTITUTE
CM032588.1	168224	.	A	T	100	*	TYPE=SUBSTITUTE
CM032588.1	168236	.	T	C	100	*	TYPE=SUBSTITUTE
CM032588.1	168255	.	A	G	100	*	TYPE=SUBSTITUTE
CM032588.1	168329	.	T	G	100	*	TYPE=SUBSTITUTE
CM032588.1	168356	.	C	G	100	*	TYPE=SUBSTITUTE
CM032588.1	168359	.	C	A	100	*	TYPE=SUBSTITUTE
CM032588.1	168365	.	T	C	100	*	TYPE=SUBSTITUTE
CM032588.1	168368	.	G	C	100	*	TYPE=SUBSTITUTE
CM032588.1	168369	.	G	C	100	*	TYPE=SUBSTITUTE
CM032588.1	168416	.	A	G	100	*	TYPE=SUBSTITUTE
CM032588.1	168419	.	G	A	100	*	TYPE=SUBSTITUTE
CM032588.1	168428	.	T	C	100	*	TYPE=SUBSTITUTE
CM032588.1	168491	.	C	T	100	*	TYPE=SUBSTITUTE
CM032588.1	168497	.	G	A	100	*	TYPE=SUBSTITUTE
CM032588.1	168498	.	C	T	100	*	TYPE=SUBSTITUTE
CM032588.1	168510	.	T	C	100	*	TYPE=SUBSTITUTE
CM032588.1	168519	.	G	A	100	*	TYPE=SUBSTITUTE
CM032588.1	168536	.	T	C	100	*	TYPE=SUBSTITUTE
CM032588.1	168551	.	C	T	100	*	TYPE=SUBSTITUTE
CM032588.1	168557	.	A	G	100	*	TYPE=SUBSTITUTE
CM032588.1	168581	.	G	A	100	*	TYPE=SUBSTITUTE
CM032588.1	168602	.	G	A	100	*	TYPE=SUBSTITUTE
CM032588.1	168605	.	T	A	100	*	TYPE=SUBSTITUTE
CM032588.1	168623	.	C	A	100	*	TYPE=SUBSTITUTE
CM032588.1	168656	.	C	T	100	*	TYPE=SUBSTITUTE
CM032588.1	168721	.	T	C	100	*	TYPE=SUBSTITUTE
CM032588.1	168815	.	T	C	100	*	TYPE=SUBSTITUTE
CM032588.1	168832	.	G	A	100	*	TYPE=SUBSTITUTE
CM032588.1	168878	.	C	G	100	*	TYPE=SUBSTITUTE
CM032588.1	169127	.	C	T	100	*	TYPE=SUBSTITUTE
CM032588.1	169181	.	A	G	100	*	TYPE=SUBSTITUTE
CM032588.1	169211	.	T	C	100	*	TYPE=SUBSTITUTE
CM032588.1	169228	.	T	C	100	*	TYPE=SUBSTITUTE
CM032588.1	169235	.	T	C	100	*	TYPE=SUBSTITUTE
CM032588.1	169285	.	T	A	100	*	TYPE=SUBSTITUTE
CM032588.1	169372	.	T	C	100	*	TYPE=SUBSTITUTE
CM032588.1	169373	.	G	A	100	*	TYPE=SUBSTITUTE
CM032588.1	169379	.	A	G	100	*	TYPE=SUBSTITUTE
CM032588.1	169382	.	G	A	100	*	TYPE=SUBSTITUTE
CM032588.1	169385	.	G	A	100	*	TYPE=SUBSTITUTE
CM032588.1	169391	.	T	C	100	*	TYPE=SUBSTITUTE
CM032588.1	169402	.	GTA	G	100	*	TYPE=DELETE
CM032588.1	169406	.	G	T	100	*	TYPE=SUBSTITUTE
CM032588.1	169409	.	T	C	100	*	TYPE=SUBSTITUTE
CM032588.1	169415	.	AGC	A	100	*	TYPE=DELETE
CM032588.1	169419	.	C	G	100	*	TYPE=SUBSTITUTE
CM032588.1	169421	.	AG	A	100	*	TYPE=DELETE
CM032588.1	169431	.	C	A	100	*	TYPE=SUBSTITUTE
CM032588.1	169436	.	C	A	100	*	TYPE=SUBSTITUTE
CM032588.1	169439	.	T	C	100	*	TYPE=SUBSTITUTE
CM032588.1	169448	.	T	C	100	*	TYPE=SUBSTITUTE
CM032588.1	169449	.	GTGT	G	100	*	TYPE=DELETE
CM032588.1	169455	.	G	T	100	*	TYPE=SUBSTITUTE
CM032588.1	169456	.	TACAG	T	100	*	TYPE=DELETE
CM032588.1	169464	.	G	A	100	*	TYPE=SUBSTITUTE
CM032588.1	169474	.	C	T	100	*	TYPE=SUBSTITUTE
CM032588.1	169483	.	G	A	100	*	TYPE=SUBSTITUTE
CM032588.1	169528	.	A	G	100	*	TYPE=SUBSTITUTE
CM032588.1	169543	.	C	T	100	*	TYPE=SUBSTITUTE
CM032588.1	169564	.	C	T	100	*	TYPE=SUBSTITUTE

CM032588.1	169565	.	G	A	100	*	TYPE=SUBSTITUTE
CM032588.1	169594	.	T	C	100	*	TYPE=SUBSTITUTE
CM032588.1	169615	.	G	A	100	*	TYPE=SUBSTITUTE
CM032588.1	169672	.	C	A	100	*	TYPE=SUBSTITUTE
CM032588.1	169693	.	T	G	100	*	TYPE=SUBSTITUTE
CM032588.1	169696	.	G	A	100	*	TYPE=SUBSTITUTE
CM032588.1	169717	.	A	G	100	*	TYPE=SUBSTITUTE
CM032588.1	169721	.	G	A	100	*	TYPE=SUBSTITUTE
CM032588.1	169753	.	G	A	100	*	TYPE=SUBSTITUTE
CM032588.1	169769	.	G	A	100	*	TYPE=SUBSTITUTE
CM032588.1	169783	.	C	g	100	*	TYPE=SUBSTITUTE
CM032588.1	169795	.	T	C	100	*	TYPE=SUBSTITUTE
CM032588.1	169808	.	G	C	100	*	TYPE=SUBSTITUTE
CM032588.1	169813	.	G	C	100	*	TYPE=SUBSTITUTE
CM032588.1	169828	.	G	T	100	*	TYPE=SUBSTITUTE
CM032588.1	169882	.	CTT	C	100	*	TYPE=DELETE
CM032588.1	169885	.	T	A	100	*	TYPE=SUBSTITUTE
CM032588.1	169888	.	C	G	100	*	TYPE=SUBSTITUTE
CM032588.1	169890	.	A	G	100	*	TYPE=SUBSTITUTE
CM032588.1	169924	.	G	A	100	*	TYPE=SUBSTITUTE
CM032588.1	169953	.	T	C	100	*	TYPE=SUBSTITUTE
CM032588.1	169985	.	G	C	100	*	TYPE=SUBSTITUTE
CM032588.1	169997	.	G	A	100	*	TYPE=SUBSTITUTE
CM032588.1	170005	.	T	TCAta	100	*	TYPE=INSERT
CM032588.1	170013	.	t	tttttta	100	*	TYPE=INSERT
CM032588.1	170016	.	A	t	100	*	TYPE=SUBSTITUTE
CM032588.1	170018	.	T	a	100	*	TYPE=SUBSTITUTE
CM032588.1	170019	.	G	a	100	*	TYPE=SUBSTITUTE
CM032588.1	170027	.	C	t	100	*	TYPE=SUBSTITUTE
CM032588.1	170033	.	C	T	100	*	TYPE=SUBSTITUTE
CM032588.1	170034	.	A	G	100	*	TYPE=SUBSTITUTE
CM032588.1	170037	.	C	T	100	*	TYPE=SUBSTITUTE
CM032588.1	170038	.	A	G	100	*	TYPE=SUBSTITUTE
CM032588.1	170040	.	A	C	100	*	TYPE=SUBSTITUTE
CM032588.1	170051	.	TA	T	100	*	TYPE=DELETE
CM032588.1	170055	.	A	G	100	*	TYPE=SUBSTITUTE
CM032588.1	170058	.	AG	A	100	*	TYPE=DELETE
CM032588.1	170060	.	A	T	100	*	TYPE=SUBSTITUTE
CM032588.1	170062	.	C	T	100	*	TYPE=SUBSTITUTE
CM032588.1	170066	.	A	AT	100	*	TYPE=INSERT
CM032588.1	170068	.	T	A	100	*	TYPE=SUBSTITUTE
CM032588.1	170071	.	G	A	100	*	TYPE=SUBSTITUTE
CM032588.1	170072	.	C	G	100	*	TYPE=SUBSTITUTE
CM032588.1	170076	.	C	T	100	*	TYPE=SUBSTITUTE
CM032588.1	170080	.	C	T	100	*	TYPE=SUBSTITUTE
CM032588.1	170081	.	A	T	100	*	TYPE=SUBSTITUTE
CM032588.1	170084	.	C	T	100	*	TYPE=SUBSTITUTE
CM032588.1	170085	.	A	T	100	*	TYPE=SUBSTITUTE
CM032588.1	170086	.	C	T	100	*	TYPE=SUBSTITUTE
CM032588.1	170091	.	T	G	100	*	TYPE=SUBSTITUTE
CM032588.1	170094	.	G	A	100	*	TYPE=SUBSTITUTE
CM032588.1	170107	.	T	G	100	*	TYPE=SUBSTITUTE
CM032588.1	170114	.	G	C	100	*	TYPE=SUBSTITUTE
CM032588.1	170132	.	T	C	100	*	TYPE=SUBSTITUTE
CM032588.1	170138	.	A	G	100	*	TYPE=SUBSTITUTE
CM032588.1	170173	.	G	A	100	*	TYPE=SUBSTITUTE
CM032588.1	170201	.	C	T	100	*	TYPE=SUBSTITUTE
CM032588.1	170228	.	T	G	100	*	TYPE=SUBSTITUTE
CM032588.1	170233	.	C	T	100	*	TYPE=SUBSTITUTE
CM032588.1	170241	.	CATAG	C	100	*	TYPE=DELETE
CM032588.1	170255	.	TG	T	100	*	TYPE=DELETE
CM032588.1	170264	.	G	A	100	*	TYPE=SUBSTITUTE
CM032588.1	170267	.	T	C	100	*	TYPE=SUBSTITUTE
CM032588.1	170274	.	A	G	100	*	TYPE=SUBSTITUTE

CM032588.1	170281	.	T	C	100	*	TYPE=SUBSTITUTE
CM032588.1	170292	.	T	C	100	*	TYPE=SUBSTITUTE
CM032588.1	170329	.	T	C	100	*	TYPE=SUBSTITUTE
CM032588.1	170345	.	T	C	100	*	TYPE=SUBSTITUTE
CM032588.1	170362	.	A	T	100	*	TYPE=SUBSTITUTE
CM032588.1	170374	.	C	T	100	*	TYPE=SUBSTITUTE
CM032588.1	170382	.	T	C	100	*	TYPE=SUBSTITUTE
CM032588.1	170383	.	A	G	100	*	TYPE=SUBSTITUTE
CM032588.1	170385	.	T	C	100	*	TYPE=SUBSTITUTE
CM032588.1	170388	.	G	A	100	*	TYPE=SUBSTITUTE
CM032588.1	170393	.	C	T	100	*	TYPE=SUBSTITUTE
CM032588.1	170397	.	G	A	100	*	TYPE=SUBSTITUTE
CM032588.1	170399	.	C	T	100	*	TYPE=SUBSTITUTE
CM032588.1	170404	.	C	T	100	*	TYPE=SUBSTITUTE
CM032588.1	170411	.	G	A	100	*	TYPE=SUBSTITUTE
CM032588.1	170432	.	G	A	100	*	TYPE=SUBSTITUTE
CM032588.1	170437	.	A	G	100	*	TYPE=SUBSTITUTE
CM032588.1	170468	.	T	C	100	*	TYPE=SUBSTITUTE
CM032588.1	170480	.	G	A	100	*	TYPE=SUBSTITUTE
CM032588.1	170484	.	C	T	100	*	TYPE=SUBSTITUTE
CM032588.1	170488	.	T	C	100	*	TYPE=SUBSTITUTE
CM032588.1	170496	.	CA	C	100	*	TYPE=DELETE
CM032588.1	170502	.	C	T	100	*	TYPE=SUBSTITUTE
CM032588.1	170510	.	A	G	100	*	TYPE=SUBSTITUTE
CM032588.1	170519	.	G	A	100	*	TYPE=SUBSTITUTE
CM032588.1	170521	.	G	C	100	*	TYPE=SUBSTITUTE
CM032588.1	170529	.	C	t	100	*	TYPE=SUBSTITUTE
CM032588.1	170530	.	A	g	100	*	TYPE=SUBSTITUTE
CM032588.1	170533	.	G	t	100	*	TYPE=SUBSTITUTE
CM032588.1	170536	.	C	t	100	*	TYPE=SUBSTITUTE
CM032588.1	170538	.	C	t	100	*	TYPE=SUBSTITUTE
CM032588.1	170539	.	A	t	100	*	TYPE=SUBSTITUTE
CM032588.1	170543	.	T	c	100	*	TYPE=SUBSTITUTE
CM032588.1	170549	.	C	T	100	*	TYPE=SUBSTITUTE
CM032588.1	170551	.	A	C	100	*	TYPE=SUBSTITUTE
CM032588.1	170552	.	T	C	100	*	TYPE=SUBSTITUTE
CM032588.1	170555	.	A	C	100	*	TYPE=SUBSTITUTE
CM032588.1	170556	.	T	C	100	*	TYPE=SUBSTITUTE
CM032588.1	170558	.	G	A	100	*	TYPE=SUBSTITUTE
CM032588.1	170565	.	C	T	100	*	TYPE=SUBSTITUTE
CM032588.1	170576	.	G	A	100	*	TYPE=SUBSTITUTE
CM032588.1	170581	.	G	A	100	*	TYPE=SUBSTITUTE
CM032588.1	170584	.	T	C	100	*	TYPE=SUBSTITUTE
CM032588.1	170600	.	T	C	100	*	TYPE=SUBSTITUTE
CM032588.1	170608	.	G	A	100	*	TYPE=SUBSTITUTE
CM032588.1	170609	.	A	T	100	*	TYPE=SUBSTITUTE
CM032588.1	170611	.	T	C	100	*	TYPE=SUBSTITUTE
CM032588.1	170614	.	A	G	100	*	TYPE=SUBSTITUTE
CM032588.1	170619	.	C	T	100	*	TYPE=SUBSTITUTE
CM032588.1	170624	.	G	A	100	*	TYPE=SUBSTITUTE
CM032588.1	170630	.	T	C	100	*	TYPE=SUBSTITUTE
CM032588.1	170634	.	T	C	100	*	TYPE=SUBSTITUTE
CM032588.1	170637	.	A	G	100	*	TYPE=SUBSTITUTE
CM032588.1	170645	.	C	G	100	*	TYPE=SUBSTITUTE
CM032588.1	170648	.	A	G	100	*	TYPE=SUBSTITUTE
CM032588.1	170660	.	C	A	100	*	TYPE=SUBSTITUTE
CM032588.1	170662	.	G	T	100	*	TYPE=SUBSTITUTE
CM032588.1	170664	.	G	A	100	*	TYPE=SUBSTITUTE
CM032588.1	170667	.	G	A	100	*	TYPE=SUBSTITUTE
CM032588.1	170678	.	TG	T	100	*	TYPE=DELETE
CM032588.1	170680	.	T	C	100	*	TYPE=SUBSTITUTE
CM032588.1	170684	.	C	T	100	*	TYPE=SUBSTITUTE
CM032588.1	170685	.	G	A	100	*	TYPE=SUBSTITUTE
CM032588.1	170688	.	G	C	100	*	TYPE=SUBSTITUTE

CM032588.1	170689	.	G	T	100	*	TYPE=SUBSTITUTE
CM032588.1	170690	.	G	A	100	*	TYPE=SUBSTITUTE
CM032588.1	170691	.	A	G	100	*	TYPE=SUBSTITUTE
CM032588.1	170693	.	A	G	100	*	TYPE=SUBSTITUTE
CM032588.1	170697	.	C	G	100	*	TYPE=SUBSTITUTE
CM032588.1	170698	.	A	C	100	*	TYPE=SUBSTITUTE
CM032588.1	170699	.	A	C	100	*	TYPE=SUBSTITUTE
CM032588.1	170700	.	T	C	100	*	TYPE=SUBSTITUTE
CM032588.1	170701	.	A	G	100	*	TYPE=SUBSTITUTE
CM032588.1	170705	.	T	C	100	*	TYPE=SUBSTITUTE
CM032588.1	170709	.	T	C	100	*	TYPE=SUBSTITUTE
CM032588.1	170710	.	G	T	100	*	TYPE=SUBSTITUTE
CM032588.1	170711	.	G	A	100	*	TYPE=SUBSTITUTE
CM032588.1	170714	.	T	G	100	*	TYPE=SUBSTITUTE
CM032588.1	170719	.	G	A	100	*	TYPE=SUBSTITUTE
CM032588.1	170725	.	G	A	100	*	TYPE=SUBSTITUTE
CM032588.1	170726	.	T	A	100	*	TYPE=SUBSTITUTE
CM032588.1	170739	.	G	A	100	*	TYPE=SUBSTITUTE
CM032588.1	170740	.	C	T	100	*	TYPE=SUBSTITUTE
CM032588.1	170742	.	T	C	100	*	TYPE=SUBSTITUTE
CM032588.1	170745	.	G	C	100	*	TYPE=SUBSTITUTE
CM032588.1	170750	.	A	C	100	*	TYPE=SUBSTITUTE
CM032588.1	170751	.	T	C	100	*	TYPE=SUBSTITUTE
CM032588.1	170761	.	A	G	100	*	TYPE=SUBSTITUTE
CM032588.1	170766	.	T	C	100	*	TYPE=SUBSTITUTE
CM032588.1	170773	.	T	C	100	*	TYPE=SUBSTITUTE
CM032588.1	170793	.	C	t	100	*	TYPE=SUBSTITUTE
CM032588.1	170826	.	A	G	100	*	TYPE=SUBSTITUTE
CM032588.1	170831	.	A	G	100	*	TYPE=SUBSTITUTE
CM032588.1	170834	.	A	G	100	*	TYPE=SUBSTITUTE
CM032588.1	170837	.	G	A	100	*	TYPE=SUBSTITUTE
CM032588.1	170839	.	T	C	100	*	TYPE=SUBSTITUTE
CM032588.1	170849	.	A	G	100	*	TYPE=SUBSTITUTE
CM032588.1	170851	.	T	C	100	*	TYPE=SUBSTITUTE
CM032588.1	170855	.	A	G	100	*	TYPE=SUBSTITUTE
CM032588.1	170863	.	T	G	100	*	TYPE=SUBSTITUTE
CM032588.1	170865	.	C	T	100	*	TYPE=SUBSTITUTE
CM032588.1	170872	.	G	A	100	*	TYPE=SUBSTITUTE
CM032588.1	170873	.	C	T	100	*	TYPE=SUBSTITUTE
CM032588.1	170878	.	G	T	100	*	TYPE=SUBSTITUTE
CM032588.1	170880	.	G	A	100	*	TYPE=SUBSTITUTE
CM032588.1	170888	.	T	C	100	*	TYPE=SUBSTITUTE
CM032588.1	170902	.	C	T	100	*	TYPE=SUBSTITUTE
CM032588.1	170904	.	T	C	100	*	TYPE=SUBSTITUTE
CM032588.1	170910	.	G	C	100	*	TYPE=SUBSTITUTE
CM032588.1	170931	.	A	ATA	100	*	TYPE=INSERT
CM032588.1	170932	.	A	G	100	*	TYPE=SUBSTITUTE
CM032588.1	170936	.	C	T	100	*	TYPE=SUBSTITUTE
CM032588.1	170945	.	G	A	100	*	TYPE=SUBSTITUTE
CM032588.1	170948	.	T	A	100	*	TYPE=SUBSTITUTE
CM032588.1	170952	.	T	C	100	*	TYPE=SUBSTITUTE
CM032588.1	170957	.	G	A	100	*	TYPE=SUBSTITUTE
CM032588.1	170959	.	C	G	100	*	TYPE=SUBSTITUTE
CM032588.1	170960	.	T	C	100	*	TYPE=SUBSTITUTE
CM032588.1	170963	.	A	G	100	*	TYPE=SUBSTITUTE
CM032588.1	170971	.	A	G	100	*	TYPE=SUBSTITUTE
CM032588.1	170978	.	G	C	100	*	TYPE=SUBSTITUTE
CM032588.1	170981	.	A	T	100	*	TYPE=SUBSTITUTE
CM032588.1	170985	.	G	T	100	*	TYPE=SUBSTITUTE
CM032588.1	170999	.	C	T	100	*	TYPE=SUBSTITUTE
CM032588.1	171018	.	G	A	100	*	TYPE=SUBSTITUTE
CM032588.1	171019	.	A	G	100	*	TYPE=SUBSTITUTE
CM032588.1	171033	.	T	C	100	*	TYPE=SUBSTITUTE
CM032588.1	171036	.	T	C	100	*	TYPE=SUBSTITUTE

CM032588.1	171043	.	T	C	100	*	TYPE=SUBSTITUTE
CM032588.1	171054	.	T	C	100	*	TYPE=SUBSTITUTE
CM032588.1	171078	.	T	C	100	*	TYPE=SUBSTITUTE
CM032588.1	171079	.	C	T	100	*	TYPE=SUBSTITUTE
CM032588.1	171081	.	A	G	100	*	TYPE=SUBSTITUTE
CM032588.1	171145	.	G	T	100	*	TYPE=SUBSTITUTE
CM032588.1	171147	.	C	T	100	*	TYPE=SUBSTITUTE
CM032588.1	171148	.	G	A	100	*	TYPE=SUBSTITUTE
CM032588.1	171154	.	C	G	100	*	TYPE=SUBSTITUTE
CM032588.1	171166	.	T	C	100	*	TYPE=SUBSTITUTE
CM032588.1	171175	.	A	G	100	*	TYPE=SUBSTITUTE
CM032588.1	171241	.	G	A	100	*	TYPE=SUBSTITUTE
CM032588.1	171250	.	C	T	100	*	TYPE=SUBSTITUTE
CM032588.1	171280	.	T	C	100	*	TYPE=SUBSTITUTE
CM032588.1	171298	.	T	A	100	*	TYPE=SUBSTITUTE
CM032588.1	171328	.	T	C	100	*	TYPE=SUBSTITUTE
CM032588.1	171336	.	G	A	100	*	TYPE=SUBSTITUTE
CM032588.1	171347	.	T	G	100	*	TYPE=SUBSTITUTE
CM032588.1	171349	.	C	G	100	*	TYPE=SUBSTITUTE
CM032588.1	171362	.	T	A	100	*	TYPE=SUBSTITUTE
CM032588.1	171364	.	C	T	100	*	TYPE=SUBSTITUTE
CM032588.1	171367	.	T	C	100	*	TYPE=SUBSTITUTE
CM032588.1	171381	.	C	T	100	*	TYPE=SUBSTITUTE
CM032588.1	171390	.	C	T	100	*	TYPE=SUBSTITUTE
CM032588.1	171409	.	G	T	100	*	TYPE=SUBSTITUTE
CM032588.1	171436	.	G	A	100	*	TYPE=SUBSTITUTE
CM032588.1	171448	.	C	T	100	*	TYPE=SUBSTITUTE
CM032588.1	171451	.	G	A	100	*	TYPE=SUBSTITUTE
CM032588.1	171459	.	A	G	100	*	TYPE=SUBSTITUTE
CM032588.1	171478	.	C	A	100	*	TYPE=SUBSTITUTE
CM032588.1	171484	.	A	G	100	*	TYPE=SUBSTITUTE
CM032588.1	171487	.	G	A	100	*	TYPE=SUBSTITUTE
CM032588.1	171505	.	A	G	100	*	TYPE=SUBSTITUTE
CM032588.1	171508	.	G	A	100	*	TYPE=SUBSTITUTE
CM032588.1	171514	.	T	C	100	*	TYPE=SUBSTITUTE
CM032588.1	171541	.	T	C	100	*	TYPE=SUBSTITUTE
CM032588.1	171567	.	T	G	100	*	TYPE=SUBSTITUTE
CM032588.1	171571	.	C	A	100	*	TYPE=SUBSTITUTE
CM032588.1	171577	.	C	T	100	*	TYPE=SUBSTITUTE
CM032588.1	171589	.	A	G	100	*	TYPE=SUBSTITUTE
CM032588.1	171591	.	T	A	100	*	TYPE=SUBSTITUTE
CM032588.1	171605	.	T	A	100	*	TYPE=SUBSTITUTE
CM032588.1	171628	.	A	T	100	*	TYPE=SUBSTITUTE
CM032588.1	171637	.	C	T	100	*	TYPE=SUBSTITUTE
CM032588.1	171646	.	A	C	100	*	TYPE=SUBSTITUTE
CM032588.1	171652	.	T	A	100	*	TYPE=SUBSTITUTE
CM032588.1	171691	.	C	T	100	*	TYPE=SUBSTITUTE
CM032588.1	171730	.	T	C	100	*	TYPE=SUBSTITUTE
CM032588.1	171745	.	A	G	100	*	TYPE=SUBSTITUTE
CM032588.1	171766	.	C	T	100	*	TYPE=SUBSTITUTE
CM032588.1	171840	.	T	C	100	*	TYPE=SUBSTITUTE
CM032588.1	171843	.	A	G	100	*	TYPE=SUBSTITUTE
CM032588.1	171845	.	A	G	100	*	TYPE=SUBSTITUTE
CM032588.1	171853	.	G	T	100	*	TYPE=SUBSTITUTE
CM032588.1	171854	.	G	A	100	*	TYPE=SUBSTITUTE
CM032588.1	171862	.	A	G	100	*	TYPE=SUBSTITUTE
CM032588.1	171865	.	G	T	100	*	TYPE=SUBSTITUTE
CM032588.1	171886	.	G	A	100	*	TYPE=SUBSTITUTE
CM032588.1	171910	.	A	G	100	*	TYPE=SUBSTITUTE
CM032588.1	171943	.	G	A	100	*	TYPE=SUBSTITUTE
CM032588.1	171949	.	G	A	100	*	TYPE=SUBSTITUTE
CM032588.1	171952	.	G	A	100	*	TYPE=SUBSTITUTE
CM032588.1	171955	.	T	C	100	*	TYPE=SUBSTITUTE
CM032588.1	171961	.	T	C	100	*	TYPE=SUBSTITUTE

CM032588.1	171990	.	C	T	100	*	TYPE=SUBSTITUTE
CM032588.1	172490	.	GACCCCCC	G	100	*	TYPE=DELETE
CM032588.1	172498	.	C	G	100	*	TYPE=SUBSTITUTE
CM032588.1	172610	.	C	T	100	*	TYPE=SUBSTITUTE
CM032588.1	172712	.	A	T	100	*	TYPE=SUBSTITUTE
CM032588.1	172858	.	AA	A	100	*	TYPE=DELETE
CM032588.1	172955	.	A	C	100	*	TYPE=SUBSTITUTE
CM032588.1	173501	.	AA	A	100	*	TYPE=DELETE
CM032588.1	173566	.	C	A	100	*	TYPE=SUBSTITUTE
CM032588.1	173586	.	C	G	100	*	TYPE=SUBSTITUTE
CM032588.1	173601	.	T	c	100	*	TYPE=SUBSTITUTE
CM032588.1	173607	.	G	t	100	*	TYPE=SUBSTITUTE
CM032588.1	173629	.	C	T	100	*	TYPE=SUBSTITUTE
CM032588.1	173637	.	G	C	100	*	TYPE=SUBSTITUTE
CM032588.1	173640	.	C	T	100	*	TYPE=SUBSTITUTE
CM032588.1	173661	.	T	C	100	*	TYPE=SUBSTITUTE
CM032588.1	173667	.	T	C	100	*	TYPE=SUBSTITUTE
CM032588.1	173673	.	A	G	100	*	TYPE=SUBSTITUTE
CM032588.1	173706	.	T	C	100	*	TYPE=SUBSTITUTE
CM032588.1	173709	.	C	T	100	*	TYPE=SUBSTITUTE
CM032588.1	173745	.	G	A	100	*	TYPE=SUBSTITUTE
CM032588.1	173771	.	A	G	100	*	TYPE=SUBSTITUTE
CM032588.1	173772	.	C	T	100	*	TYPE=SUBSTITUTE
CM032588.1	173777	.	C	T	100	*	TYPE=SUBSTITUTE
CM032588.1	173780	.	T	C	100	*	TYPE=SUBSTITUTE
CM032588.1	173801	.	A	G	100	*	TYPE=SUBSTITUTE
CM032588.1	173814	.	G	t	100	*	TYPE=SUBSTITUTE
CM032588.1	173827	.	G	a	100	*	TYPE=SUBSTITUTE
CM032588.1	173841	.	C	T	100	*	TYPE=SUBSTITUTE
CM032588.1	173877	.	T	c	100	*	TYPE=SUBSTITUTE
CM032588.1	173889	.	G	A	100	*	TYPE=SUBSTITUTE
CM032588.1	173925	.	G	A	100	*	TYPE=SUBSTITUTE
CM032588.1	173932	.	G	C	100	*	TYPE=SUBSTITUTE
CM032588.1	173933	.	G	A	100	*	TYPE=SUBSTITUTE
CM032588.1	173937	.	A	G	100	*	TYPE=SUBSTITUTE
CM032588.1	173944	.	T	A	100	*	TYPE=SUBSTITUTE
CM032588.1	173958	.	T	C	100	*	TYPE=SUBSTITUTE
CM032588.1	173979	.	A	G	100	*	TYPE=SUBSTITUTE
CM032588.1	173994	.	G	T	100	*	TYPE=SUBSTITUTE
CM032588.1	173997	.	G	C	100	*	TYPE=SUBSTITUTE
CM032588.1	174114	.	G	C	100	*	TYPE=SUBSTITUTE
CM032588.1	174128	.	A	G	100	*	TYPE=SUBSTITUTE
CM032588.1	174141	.	G	A	100	*	TYPE=SUBSTITUTE
CM032588.1	174189	.	A	T	100	*	TYPE=SUBSTITUTE
CM032588.1	174252	.	A	G	100	*	TYPE=SUBSTITUTE
CM032588.1	174322	.	A	G	100	*	TYPE=SUBSTITUTE
CM032588.1	174332	.	G	A	100	*	TYPE=SUBSTITUTE
CM032588.1	174944	.	G	C	100	*	TYPE=SUBSTITUTE
CM032588.1	175284	.	T	C	100	*	TYPE=SUBSTITUTE
CM032588.1	175290	.	G	T	100	*	TYPE=SUBSTITUTE
CM032588.1	175316	.	G	A	100	*	TYPE=SUBSTITUTE
CM032588.1	175327	.	A	C	100	*	TYPE=SUBSTITUTE
CM032588.1	175331	.	G	T	100	*	TYPE=SUBSTITUTE
CM032588.1	175337	.	C	T	100	*	TYPE=SUBSTITUTE
CM032588.1	175339	.	A	G	100	*	TYPE=SUBSTITUTE
CM032588.1	175345	.	T	C	100	*	TYPE=SUBSTITUTE
CM032588.1	175349	.	C	T	100	*	TYPE=SUBSTITUTE
CM032588.1	175353	.	G	A	100	*	TYPE=SUBSTITUTE
CM032588.1	175356	.	T	A	100	*	TYPE=SUBSTITUTE
CM032588.1	175357	.	GA	G	100	*	TYPE=DELETE
CM032588.1	175359	.	A	T	100	*	TYPE=SUBSTITUTE
CM032588.1	175379	.	A	AA	100	*	TYPE=INSERT
CM032588.1	175382	.	G	C	100	*	TYPE=SUBSTITUTE
CM032588.1	175384	.	G	C	100	*	TYPE=SUBSTITUTE

CM032588.1	175386	.	A	G	100	*	TYPE=SUBSTITUTE
CM032588.1	175387	.	G	A	100	*	TYPE=SUBSTITUTE
CM032588.1	175390	.	T	A	100	*	TYPE=SUBSTITUTE
CM032588.1	175423	.	A	G	100	*	TYPE=SUBSTITUTE
CM032588.1	175464	.	T	C	100	*	TYPE=SUBSTITUTE
CM032588.1	175465	.	G	A	100	*	TYPE=SUBSTITUTE
CM032588.1	175471	.	G	C	100	*	TYPE=SUBSTITUTE
CM032588.1	175477	.	T	C	100	*	TYPE=SUBSTITUTE
CM032588.1	175486	.	A	C	100	*	TYPE=SUBSTITUTE
CM032588.1	175507	.	G	C	100	*	TYPE=SUBSTITUTE
CM032588.1	175522	.	T	C	100	*	TYPE=SUBSTITUTE
CM032588.1	175531	.	A	G	100	*	TYPE=SUBSTITUTE
CM032588.1	175588	.	G	A	100	*	TYPE=SUBSTITUTE
CM032588.1	175600	.	C	T	100	*	TYPE=SUBSTITUTE
CM032588.1	175612	.	A	G	100	*	TYPE=SUBSTITUTE
CM032588.1	175624	.	G	A	100	*	TYPE=SUBSTITUTE
CM032588.1	175645	.	G	A	100	*	TYPE=SUBSTITUTE
CM032588.1	176004	.	T	A	100	*	TYPE=SUBSTITUTE
CM032588.1	176089	.	C	T	100	*	TYPE=SUBSTITUTE
CM032588.1	176532	.	T	C	100	*	TYPE=SUBSTITUTE
CM032588.1	176604	.	G	A	100	*	TYPE=SUBSTITUTE
CM032588.1	176790	.	G	A	100	*	TYPE=SUBSTITUTE
CM032588.1	177663	.	G	A	100	*	TYPE=SUBSTITUTE
CM032588.1	177809	.	T	C	100	*	TYPE=SUBSTITUTE
CM032588.1	177845	.	C	A	100	*	TYPE=SUBSTITUTE
CM032588.1	177848	.	G	T	100	*	TYPE=SUBSTITUTE
CM032588.1	177851	.	C	t	100	*	TYPE=SUBSTITUTE
CM032588.1	177854	.	T	c	100	*	TYPE=SUBSTITUTE
CM032588.1	177876	.	C	T	100	*	TYPE=SUBSTITUTE
CM032588.1	177879	.	T	G	100	*	TYPE=SUBSTITUTE
CM032588.1	177884	.	T	C	100	*	TYPE=SUBSTITUTE
CM032588.1	177886	.	A	C	100	*	TYPE=SUBSTITUTE
CM032588.1	177888	.	A	ACTG	100	*	TYPE=INSERT
CM032588.1	177892	.	T	TG	100	*	TYPE=INSERT
CM032588.1	177894	.	A	T	100	*	TYPE=SUBSTITUTE
CM032588.1	177896	.	T	C	100	*	TYPE=SUBSTITUTE
CM032588.1	177899	.	T	G	100	*	TYPE=SUBSTITUTE
CM032588.1	177901	.	C	T	100	*	TYPE=SUBSTITUTE
CM032588.1	177902	.	TGAAG	T	100	*	TYPE=DELETE
CM032588.1	177907	.	A	T	100	*	TYPE=SUBSTITUTE
CM032588.1	177916	.	C	T	100	*	TYPE=SUBSTITUTE
CM032588.1	177919	.	A	C	100	*	TYPE=SUBSTITUTE
CM032588.1	177920	.	A	G	100	*	TYPE=SUBSTITUTE
CM032588.1	177922	.	A	G	100	*	TYPE=SUBSTITUTE
CM032588.1	177934	.	T	G	100	*	TYPE=SUBSTITUTE
CM032588.1	177938	.	C	T	100	*	TYPE=SUBSTITUTE
CM032588.1	177948	.	G	A	100	*	TYPE=SUBSTITUTE
CM032588.1	177950	.	C	T	100	*	TYPE=SUBSTITUTE
CM032588.1	177957	.	G	C	100	*	TYPE=SUBSTITUTE
CM032588.1	177958	.	A	G	100	*	TYPE=SUBSTITUTE
CM032588.1	177960	.	C	T	100	*	TYPE=SUBSTITUTE
CM032588.1	177973	.	T	C	100	*	TYPE=SUBSTITUTE
CM032588.1	177978	.	TG	T	100	*	TYPE=DELETE
CM032588.1	177984	.	T	C	100	*	TYPE=SUBSTITUTE
CM032588.1	177996	.	T	C	100	*	TYPE=SUBSTITUTE
CM032588.1	178005	.	C	T	100	*	TYPE=SUBSTITUTE
CM032588.1	178010	.	G	A	100	*	TYPE=SUBSTITUTE
CM032588.1	178022	.	T	C	100	*	TYPE=SUBSTITUTE
CM032588.1	178026	.	T	A	100	*	TYPE=SUBSTITUTE
CM032588.1	178034	.	G	A	100	*	TYPE=SUBSTITUTE
CM032588.1	178042	.	G	A	100	*	TYPE=SUBSTITUTE
CM032588.1	178054	.	C	A	100	*	TYPE=SUBSTITUTE
CM032588.1	178065	.	G	T	100	*	TYPE=SUBSTITUTE
CM032588.1	178070	.	A	T	100	*	TYPE=SUBSTITUTE

CM032588.1	178072	.	A	T	100	*	TYPE=SUBSTITUTE
CM032588.1	178088	.	C	T	100	*	TYPE=SUBSTITUTE
CM032588.1	178101	.	C	T	100	*	TYPE=SUBSTITUTE
CM032588.1	178102	.	C	G	100	*	TYPE=SUBSTITUTE
CM032588.1	178106	.	AAC	A	100	*	TYPE=DELETE
CM032588.1	178109	.	A	G	100	*	TYPE=SUBSTITUTE
CM032588.1	178112	.	G	T	100	*	TYPE=SUBSTITUTE
CM032588.1	178116	.	C	T	100	*	TYPE=SUBSTITUTE
CM032588.1	178119	.	G	A	100	*	TYPE=SUBSTITUTE
CM032588.1	178121	.	T	C	100	*	TYPE=SUBSTITUTE
CM032588.1	178161	.	G	A	100	*	TYPE=SUBSTITUTE
CM032588.1	178194	.	A	G	100	*	TYPE=SUBSTITUTE
CM032588.1	178235	.	T	TTGT	100	*	TYPE=INSERT
CM032588.1	178236	.	G	A	100	*	TYPE=SUBSTITUTE
CM032588.1	178244	.	A	G	100	*	TYPE=SUBSTITUTE
CM032588.1	178245	.	T	G	100	*	TYPE=SUBSTITUTE
CM032588.1	178253	.	A	T	100	*	TYPE=SUBSTITUTE
CM032588.1	178298	.	A	G	100	*	TYPE=SUBSTITUTE
CM032588.1	178377	.	C	T	100	*	TYPE=SUBSTITUTE
CM032588.1	178383	.	C	T	100	*	TYPE=SUBSTITUTE
CM032588.1	178392	.	C	G	100	*	TYPE=SUBSTITUTE
CM032588.1	178404	.	G	T	100	*	TYPE=SUBSTITUTE
CM032588.1	178416	.	C	T	100	*	TYPE=SUBSTITUTE
CM032588.1	178434	.	G	a	100	*	TYPE=SUBSTITUTE
CM032588.1	178437	.	T	c	100	*	TYPE=SUBSTITUTE
CM032588.1	178476	.	C	T	100	*	TYPE=SUBSTITUTE
CM032588.1	178488	.	A	AN	100	*	TYPE=INSERT
CM032588.1	178506	.	A	G	100	*	TYPE=SUBSTITUTE
CM032588.1	178563	.	G	A	100	*	TYPE=SUBSTITUTE
CM032588.1	178599	.	G	A	100	*	TYPE=SUBSTITUTE
CM032588.1	178614	.	C	G	100	*	TYPE=SUBSTITUTE
CM032588.1	178629	.	T	C	100	*	TYPE=SUBSTITUTE
CM032588.1	178652	.	A	G	100	*	TYPE=SUBSTITUTE
CM032588.1	178662	.	C	A	100	*	TYPE=SUBSTITUTE
CM032588.1	178674	.	C	T	100	*	TYPE=SUBSTITUTE
CM032588.1	178683	.	C	A	100	*	TYPE=SUBSTITUTE
CM032588.1	178698	.	T	G	100	*	TYPE=SUBSTITUTE
CM032588.1	178700	.	A	G	100	*	TYPE=SUBSTITUTE
CM032588.1	178704	.	T	C	100	*	TYPE=SUBSTITUTE
CM032588.1	178749	.	C	G	100	*	TYPE=SUBSTITUTE
CM032588.1	178752	.	A	G	100	*	TYPE=SUBSTITUTE
CM032588.1	178770	.	T	A	100	*	TYPE=SUBSTITUTE
CM032588.1	178836	.	A	G	100	*	TYPE=SUBSTITUTE
CM032588.1	178839	.	A	G	100	*	TYPE=SUBSTITUTE
CM032588.1	178857	.	G	A	100	*	TYPE=SUBSTITUTE
CM032588.1	178896	.	G	A	100	*	TYPE=SUBSTITUTE
CM032588.1	178948	.	G	A	100	*	TYPE=SUBSTITUTE
CM032588.1	178962	.	C	T	100	*	TYPE=SUBSTITUTE
CM032588.1	179043	.	T	G	100	*	TYPE=SUBSTITUTE
CM032588.1	179049	.	C	T	100	*	TYPE=SUBSTITUTE
CM032588.1	179236	.	A	T	100	*	TYPE=SUBSTITUTE
CM032588.1	179310	.	T	G	100	*	TYPE=SUBSTITUTE
CM032588.1	179538	.	C	T	100	*	TYPE=SUBSTITUTE
CM032588.1	179586	.	C	T	100	*	TYPE=SUBSTITUTE
CM032588.1	179592	.	C	t	100	*	TYPE=SUBSTITUTE
CM032588.1	179596	.	G	t	100	*	TYPE=SUBSTITUTE
CM032588.1	179597	.	T	g	100	*	TYPE=SUBSTITUTE
CM032588.1	179609	.	A	G	100	*	TYPE=SUBSTITUTE
CM032588.1	179621	.	A	G	100	*	TYPE=SUBSTITUTE
CM032588.1	179625	.	C	T	100	*	TYPE=SUBSTITUTE
CM032588.1	179630	.	C	T	100	*	TYPE=SUBSTITUTE
CM032588.1	179652	.	T	c	100	*	TYPE=SUBSTITUTE
CM032588.1	179670	.	G	c	100	*	TYPE=SUBSTITUTE
CM032588.1	179697	.	C	G	100	*	TYPE=SUBSTITUTE

CM032588.1	179730	.	G	A	100	*	TYPE=SUBSTITUTE
CM032588.1	179778	.	T	C	100	*	TYPE=SUBSTITUTE
CM032588.1	179817	.	A	G	100	*	TYPE=SUBSTITUTE
CM032588.1	179819	.	T	C	100	*	TYPE=SUBSTITUTE
CM032588.1	179826	.	C	T	100	*	TYPE=SUBSTITUTE
CM032588.1	179859	.	G	A	100	*	TYPE=SUBSTITUTE
CM032588.1	179862	.	g	gaac	100	*	TYPE=INSERT
CM032588.1	179866	.	G	t	100	*	TYPE=SUBSTITUTE
CM032588.1	179874	.	A	c	100	*	TYPE=SUBSTITUTE
CM032588.1	179876	.	A	t	100	*	TYPE=SUBSTITUTE
CM032588.1	179878	.	T	c	100	*	TYPE=SUBSTITUTE
CM032588.1	179880	.	T	C	100	*	TYPE=SUBSTITUTE
CM032588.1	179891	.	G	A	100	*	TYPE=SUBSTITUTE
CM032588.1	179894	.	C	T	100	*	TYPE=SUBSTITUTE
CM032588.1	179899	.	A	G	100	*	TYPE=SUBSTITUTE
CM032588.1	179910	.	A	G	100	*	TYPE=SUBSTITUTE
CM032588.1	179925	.	CATTGG	C	100	*	TYPE=DELETE
CM032588.1	179932	.	A	C	100	*	TYPE=SUBSTITUTE
CM032588.1	179933	.	CATT	C	100	*	TYPE=DELETE
CM032588.1	179940	.	T	A	100	*	TYPE=SUBSTITUTE
CM032588.1	179942	.	G	A	100	*	TYPE=SUBSTITUTE
CM032588.1	179948	.	G	C	100	*	TYPE=SUBSTITUTE
CM032588.1	179976	.	A	G	100	*	TYPE=SUBSTITUTE
CM032588.1	179986	.	C	T	100	*	TYPE=SUBSTITUTE
CM032588.1	179991	.	T	C	100	*	TYPE=SUBSTITUTE
CM032588.1	180076	.	A	G	100	*	TYPE=SUBSTITUTE
CM032588.1	180077	.	C	T	100	*	TYPE=SUBSTITUTE
CM032588.1	180080	.	T	C	100	*	TYPE=SUBSTITUTE
CM032588.1	180081	.	G	A	100	*	TYPE=SUBSTITUTE
CM032588.1	180106	.	G	A	100	*	TYPE=SUBSTITUTE
CM032588.1	180121	.	TAC	T	100	*	TYPE=DELETE
CM032588.1	180124	.	A	G	100	*	TYPE=SUBSTITUTE
CM032588.1	180136	.	G	A	100	*	TYPE=SUBSTITUTE
CM032588.1	180139	.	A	G	100	*	TYPE=SUBSTITUTE
CM032588.1	180148	.	A	G	100	*	TYPE=SUBSTITUTE
CM032588.1	180159	.	T	C	100	*	TYPE=SUBSTITUTE
CM032588.1	180162	.	T	C	100	*	TYPE=SUBSTITUTE
CM032588.1	180165	.	T	TTCGGGC	100	*	TYPE=INSERT
CM032588.1	180171	.	A	AGATT	100	*	TYPE=INSERT
CM032588.1	180180	.	C	G	100	*	TYPE=SUBSTITUTE
CM032588.1	180185	.	G	A	100	*	TYPE=SUBSTITUTE
CM032588.1	180203	.	C	T	100	*	TYPE=SUBSTITUTE
CM032588.1	180232	.	A	T	100	*	TYPE=SUBSTITUTE
CM032588.1	180265	.	G	A	100	*	TYPE=SUBSTITUTE
CM032588.1	180281	.	A	G	100	*	TYPE=SUBSTITUTE
CM032588.1	180291	.	C	T	100	*	TYPE=SUBSTITUTE
CM032588.1	180295	.	A	G	100	*	TYPE=SUBSTITUTE
CM032588.1	180317	.	T	A	100	*	TYPE=SUBSTITUTE
CM032588.1	180325	.	T	G	100	*	TYPE=SUBSTITUTE
CM032588.1	180326	.	G	A	100	*	TYPE=SUBSTITUTE
CM032588.1	180329	.	A	C	100	*	TYPE=SUBSTITUTE
CM032588.1	180368	.	C	A	100	*	TYPE=SUBSTITUTE
CM032588.1	180387	.	C	A	100	*	TYPE=SUBSTITUTE
CM032588.1	180428	.	C	A	100	*	TYPE=SUBSTITUTE
CM032588.1	180431	.	G	C	100	*	TYPE=SUBSTITUTE
CM032588.1	180446	.	A	G	100	*	TYPE=SUBSTITUTE
CM032588.1	180470	.	C	t	100	*	TYPE=SUBSTITUTE
CM032588.1	180527	.	G	C	100	*	TYPE=SUBSTITUTE
CM032588.1	180551	.	T	C	100	*	TYPE=SUBSTITUTE
CM032588.1	180569	.	A	g	100	*	TYPE=SUBSTITUTE
CM032588.1	180620	.	T	C	100	*	TYPE=SUBSTITUTE
CM032588.1	180662	.	A	g	100	*	TYPE=SUBSTITUTE
CM032588.1	180773	.	A	C	100	*	TYPE=SUBSTITUTE
CM032588.1	180818	.	T	C	100	*	TYPE=SUBSTITUTE

CM032588.1	180821	.	T	G	100	*	TYPE=SUBSTITUTE
CM032588.1	180836	.	T	c	100	*	TYPE=SUBSTITUTE
CM032588.1	180845	.	C	T	100	*	TYPE=SUBSTITUTE
CM032588.1	180857	.	T	C	100	*	TYPE=SUBSTITUTE
CM032588.1	180893	.	C	T	100	*	TYPE=SUBSTITUTE
CM032588.1	180899	.	G	C	100	*	TYPE=SUBSTITUTE
CM032588.1	180914	.	G	A	100	*	TYPE=SUBSTITUTE
CM032588.1	180932	.	T	C	100	*	TYPE=SUBSTITUTE
CM032588.1	180944	.	C	T	100	*	TYPE=SUBSTITUTE
CM032588.1	180950	.	T	C	100	*	TYPE=SUBSTITUTE
CM032588.1	180989	.	G	A	100	*	TYPE=SUBSTITUTE
CM032588.1	180992	.	G	A	100	*	TYPE=SUBSTITUTE
CM032588.1	180998	.	T	A	100	*	TYPE=SUBSTITUTE
CM032588.1	181007	.	A	C	100	*	TYPE=SUBSTITUTE
CM032588.1	181022	.	T	C	100	*	TYPE=SUBSTITUTE
CM032588.1	181028	.	C	T	100	*	TYPE=SUBSTITUTE
CM032588.1	181109	.	C	T	100	*	TYPE=SUBSTITUTE
CM032588.1	181118	.	T	C	100	*	TYPE=SUBSTITUTE
CM032588.1	181133	.	G	A	100	*	TYPE=SUBSTITUTE
CM032588.1	181176	.	G	A	100	*	TYPE=SUBSTITUTE
CM032588.1	181178	.	C	T	100	*	TYPE=SUBSTITUTE
CM032588.1	181217	.	T	C	100	*	TYPE=SUBSTITUTE
CM032588.1	181271	.	C	T	100	*	TYPE=SUBSTITUTE
CM032588.1	181298	.	C	T	100	*	TYPE=SUBSTITUTE
CM032588.1	181301	.	G	C	100	*	TYPE=SUBSTITUTE
CM032588.1	181310	.	T	C	100	*	TYPE=SUBSTITUTE
CM032588.1	181337	.	G	T	100	*	TYPE=SUBSTITUTE
CM032588.1	181340	.	A	G	100	*	TYPE=SUBSTITUTE
CM032588.1	181349	.	G	A	100	*	TYPE=SUBSTITUTE
CM032588.1	181352	.	A	G	100	*	TYPE=SUBSTITUTE
CM032588.1	181391	.	T	C	100	*	TYPE=SUBSTITUTE
CM032588.1	181406	.	T	A	100	*	TYPE=SUBSTITUTE
CM032588.1	181412	.	C	T	100	*	TYPE=SUBSTITUTE
CM032588.1	181427	.	G	A	100	*	TYPE=SUBSTITUTE
CM032588.1	181430	.	C	T	100	*	TYPE=SUBSTITUTE
CM032588.1	181433	.	T	C	100	*	TYPE=SUBSTITUTE
CM032588.1	181448	.	C	G	100	*	TYPE=SUBSTITUTE
CM032588.1	181460	.	A	G	100	*	TYPE=SUBSTITUTE
CM032588.1	181466	.	C	T	100	*	TYPE=SUBSTITUTE
CM032588.1	181484	.	A	G	100	*	TYPE=SUBSTITUTE
CM032588.1	181500	.	T	C	100	*	TYPE=SUBSTITUTE
CM032588.1	181508	.	A	G	100	*	TYPE=SUBSTITUTE
CM032588.1	181535	.	G	T	100	*	TYPE=SUBSTITUTE
CM032588.1	181541	.	G	A	100	*	TYPE=SUBSTITUTE
CM032588.1	181544	.	C	G	100	*	TYPE=SUBSTITUTE
CM032588.1	181545	.	T	c	100	*	TYPE=SUBSTITUTE
CM032588.1	290969	.	G	C	100	*	TYPE=SUBSTITUTE
CM032588.1	291022	.	T	C	100	*	TYPE=SUBSTITUTE
CM032588.1	291024	.	A	C	100	*	TYPE=SUBSTITUTE
CM032588.1	291029	.	T	C	100	*	TYPE=SUBSTITUTE
CM032588.1	291033	.	T	TA	100	*	TYPE=INSERT
CM032588.1	291035	.	G	C	100	*	TYPE=SUBSTITUTE
CM032588.1	291049	.	T	C	100	*	TYPE=SUBSTITUTE
CM032588.1	291070	.	T	C	100	*	TYPE=SUBSTITUTE
CM032588.1	291088	.	TT	T	100	*	TYPE=DELETE
CM032588.1	291093	.	C	T	100	*	TYPE=SUBSTITUTE
CM032588.1	291122	.	C	A	100	*	TYPE=SUBSTITUTE
CM032588.1	291132	.	T	C	100	*	TYPE=SUBSTITUTE
CM032588.1	291159	.	A	G	100	*	TYPE=SUBSTITUTE
CM032588.1	291173	.	G	C	100	*	TYPE=SUBSTITUTE
CM032588.1	291180	.	C	T	100	*	TYPE=SUBSTITUTE
CM032588.1	291204	.	C	T	100	*	TYPE=SUBSTITUTE
CM032588.1	291208	.	T	C	100	*	TYPE=SUBSTITUTE
CM032588.1	291212	.	C	T	100	*	TYPE=SUBSTITUTE

CM032588.1	291240	.	G	A	100	*	TYPE=SUBSTITUTE
CM032588.1	291266	.	A	G	100	*	TYPE=SUBSTITUTE
CM032588.1	291267	.	G	A	100	*	TYPE=SUBSTITUTE
CM032588.1	291318	.	G	A	100	*	TYPE=SUBSTITUTE
CM032588.1	291320	.	A	G	100	*	TYPE=SUBSTITUTE
CM032588.1	291342	.	A	G	100	*	TYPE=SUBSTITUTE
CM032588.1	291344	.	C	A	100	*	TYPE=SUBSTITUTE
CM032588.1	291346	.	A	T	100	*	TYPE=SUBSTITUTE
CM032588.1	291347	.	A	G	100	*	TYPE=SUBSTITUTE
CM032588.1	291348	.	A	G	100	*	TYPE=SUBSTITUTE
CM032588.1	291350	.	A	G	100	*	TYPE=SUBSTITUTE
CM032588.1	291354	.	C	G	100	*	TYPE=SUBSTITUTE
CM032588.1	291355	.	A	C	100	*	TYPE=SUBSTITUTE
CM032588.1	291361	.	A	G	100	*	TYPE=SUBSTITUTE
CM032588.1	291370	.	C	T	100	*	TYPE=SUBSTITUTE
CM032588.1	291376	.	A	C	100	*	TYPE=SUBSTITUTE
CM032588.1	291377	.	A	T	100	*	TYPE=SUBSTITUTE
CM032588.1	291385	.	C	T	100	*	TYPE=SUBSTITUTE
CM032588.1	291386	.	T	g	100	*	TYPE=SUBSTITUTE
CM032588.1	291428	.	T	C	100	*	TYPE=SUBSTITUTE
CM032588.1	291434	.	T	C	100	*	TYPE=SUBSTITUTE
CM032588.1	291477	.	G	A	100	*	TYPE=SUBSTITUTE
CM032588.1	291485	.	C	T	100	*	TYPE=SUBSTITUTE
CM032588.1	291539	.	C	T	100	*	TYPE=SUBSTITUTE
CM032588.1	291540	.	A	AC	100	*	TYPE=INSERT
CM032588.1	291554	.	C	T	100	*	TYPE=SUBSTITUTE
CM032588.1	291555	.	C	T	100	*	TYPE=SUBSTITUTE
CM032588.1	291563	.	A	G	100	*	TYPE=SUBSTITUTE
CM032588.1	291573	.	G	T	100	*	TYPE=SUBSTITUTE
CM032588.1	291575	.	T	C	100	*	TYPE=SUBSTITUTE
CM032588.1	291577	.	G	T	100	*	TYPE=SUBSTITUTE
CM032588.1	291578	.	C	T	100	*	TYPE=SUBSTITUTE
CM032588.1	291584	.	G	A	100	*	TYPE=SUBSTITUTE
CM032588.1	291592	.	C	T	100	*	TYPE=SUBSTITUTE
CM032588.1	291595	.	C	T	100	*	TYPE=SUBSTITUTE
CM032588.1	291601	.	C	t	100	*	TYPE=SUBSTITUTE
CM032588.1	291602	.	A	g	100	*	TYPE=SUBSTITUTE
CM032588.1	291603	.	G	t	100	*	TYPE=SUBSTITUTE
CM032588.1	291604	.	T	c	100	*	TYPE=SUBSTITUTE
CM032588.1	291609	.	A	g	100	*	TYPE=SUBSTITUTE
CM032588.1	291611	.	A	g	100	*	TYPE=SUBSTITUTE
CM032588.1	291616	.	A	g	100	*	TYPE=SUBSTITUTE
CM032588.1	291621	.	A	G	100	*	TYPE=SUBSTITUTE
CM032588.1	291623	.	G	A	100	*	TYPE=SUBSTITUTE
CM032588.1	291664	.	T	C	100	*	TYPE=SUBSTITUTE
CM032588.1	291670	.	A	G	100	*	TYPE=SUBSTITUTE
CM032588.1	291687	.	A	G	100	*	TYPE=SUBSTITUTE
CM032588.1	291689	.	G	A	100	*	TYPE=SUBSTITUTE
CM032588.1	291775	.	A	G	100	*	TYPE=SUBSTITUTE
CM032588.1	291781	.	C	T	100	*	TYPE=SUBSTITUTE
CM032588.1	291814	.	C	T	100	*	TYPE=SUBSTITUTE
CM032588.1	291826	.	A	C	100	*	TYPE=SUBSTITUTE
CM032588.1	291853	.	C	A	100	*	TYPE=SUBSTITUTE
CM032588.1	291885	.	T	A	100	*	TYPE=SUBSTITUTE
CM032588.1	291893	.	G	A	100	*	TYPE=SUBSTITUTE
CM032588.1	291913	.	A	G	100	*	TYPE=SUBSTITUTE
CM032588.1	291914	.	C	T	100	*	TYPE=SUBSTITUTE
CM032588.1	291954	.	T	A	100	*	TYPE=SUBSTITUTE
CM032588.1	291965	.	C	A	100	*	TYPE=SUBSTITUTE
CM032588.1	291975	.	C	g	100	*	TYPE=SUBSTITUTE
CM032588.1	291986	.	G	a	100	*	TYPE=SUBSTITUTE
CM032588.1	291989	.	A	G	100	*	TYPE=SUBSTITUTE
CM032588.1	292011	.	C	T	100	*	TYPE=SUBSTITUTE
CM032588.1	292033	.	A	G	100	*	TYPE=SUBSTITUTE

CM032588.1	292054	.	T	C	100	*	TYPE=SUBSTITUTE
CM032588.1	292063	.	G	C	100	*	TYPE=SUBSTITUTE
CM032588.1	292094	.	G	A	100	*	TYPE=SUBSTITUTE
CM032588.1	292095	.	T	C	100	*	TYPE=SUBSTITUTE
CM032588.1	292111	.	A	G	100	*	TYPE=SUBSTITUTE
CM032588.1	292116	.	A	G	100	*	TYPE=SUBSTITUTE
CM032588.1	292124	.	G	A	100	*	TYPE=SUBSTITUTE
CM032588.1	292127	.	A	G	100	*	TYPE=SUBSTITUTE
CM032588.1	292133	.	A	C	100	*	TYPE=SUBSTITUTE
CM032588.1	292139	.	A	G	100	*	TYPE=SUBSTITUTE
CM032588.1	292193	.	C	G	100	*	TYPE=SUBSTITUTE
CM032588.1	292198	.	A	C	100	*	TYPE=SUBSTITUTE
CM032588.1	292205	.	A	G	100	*	TYPE=SUBSTITUTE
CM032588.1	292216	.	C	A	100	*	TYPE=SUBSTITUTE
CM032588.1	292219	.	C	T	100	*	TYPE=SUBSTITUTE
CM032588.1	292228	.	C	T	100	*	TYPE=SUBSTITUTE
CM032588.1	292231	.	C	T	100	*	TYPE=SUBSTITUTE
CM032588.1	292274	.	G	C	100	*	TYPE=SUBSTITUTE
CM032588.1	292275	.	C	T	100	*	TYPE=SUBSTITUTE
CM032588.1	292291	.	G	A	100	*	TYPE=SUBSTITUTE
CM032588.1	292300	.	C	T	100	*	TYPE=SUBSTITUTE
CM032588.1	292318	.	G	C	100	*	TYPE=SUBSTITUTE
CM032588.1	292366	.	C	T	100	*	TYPE=SUBSTITUTE
CM032588.1	292398	.	C	T	100	*	TYPE=SUBSTITUTE
CM032588.1	292405	.	A	G	100	*	TYPE=SUBSTITUTE
CM032588.1	292415	.	C	A	100	*	TYPE=SUBSTITUTE
CM032588.1	292417	.	T	C	100	*	TYPE=SUBSTITUTE
CM032588.1	292423	.	A	G	100	*	TYPE=SUBSTITUTE
CM032588.1	292438	.	G	C	100	*	TYPE=SUBSTITUTE
CM032588.1	292444	.	C	T	100	*	TYPE=SUBSTITUTE
CM032588.1	292445	.	C	T	100	*	TYPE=SUBSTITUTE
CM032588.1	292489	.	A	G	100	*	TYPE=SUBSTITUTE
CM032588.1	292495	.	T	A	100	*	TYPE=SUBSTITUTE
CM032588.1	292499	.	T	C	100	*	TYPE=SUBSTITUTE
CM032588.1	292500	.	G	A	100	*	TYPE=SUBSTITUTE
CM032588.1	292513	.	T	C	100	*	TYPE=SUBSTITUTE
CM032588.1	292516	.	T	C	100	*	TYPE=SUBSTITUTE
CM032588.1	292522	.	A	G	100	*	TYPE=SUBSTITUTE
CM032588.1	292537	.	G	A	100	*	TYPE=SUBSTITUTE
CM032588.1	292547	.	A	G	100	*	TYPE=SUBSTITUTE
CM032588.1	292548	.	A	G	100	*	TYPE=SUBSTITUTE
CM032588.1	292549	.	C	A	100	*	TYPE=SUBSTITUTE
CM032588.1	292571	.	G	A	100	*	TYPE=SUBSTITUTE
CM032588.1	292574	.	T	A	100	*	TYPE=SUBSTITUTE
CM032588.1	292628	.	T	C	100	*	TYPE=SUBSTITUTE
CM032588.1	292642	.	T	C	100	*	TYPE=SUBSTITUTE
CM032588.1	292728	.	A	C	100	*	TYPE=SUBSTITUTE
CM032588.1	292729	.	A	T	100	*	TYPE=SUBSTITUTE
CM032588.1	292780	.	C	T	100	*	TYPE=SUBSTITUTE
CM032588.1	292786	.	G	C	100	*	TYPE=SUBSTITUTE
CM032588.1	292809	.	A	G	100	*	TYPE=SUBSTITUTE
CM032588.1	292820	.	A	T	100	*	TYPE=SUBSTITUTE
CM032588.1	292844	.	T	C	100	*	TYPE=SUBSTITUTE
CM032588.1	292874	.	C	T	100	*	TYPE=SUBSTITUTE
CM032588.1	292911	.	G	A	100	*	TYPE=SUBSTITUTE
CM032588.1	292944	.	A	T	100	*	TYPE=SUBSTITUTE
CM032588.1	292974	.	T	A	100	*	TYPE=SUBSTITUTE
CM032588.1	292983	.	C	T	100	*	TYPE=SUBSTITUTE
CM032588.1	293004	.	T	C	100	*	TYPE=SUBSTITUTE
CM032588.1	293014	.	G	C	100	*	TYPE=SUBSTITUTE
CM032588.1	293039	.	C	A	100	*	TYPE=SUBSTITUTE
CM032588.1	293066	.	T	G	100	*	TYPE=SUBSTITUTE
CM032588.1	293092	.	T	A	100	*	TYPE=SUBSTITUTE
CM032588.1	293134	.	A	G	100	*	TYPE=SUBSTITUTE

CM032588.1	293197	.	C	T	100	*	TYPE=SUBSTITUTE
CM032588.1	293228	.	T	A	100	*	TYPE=SUBSTITUTE
CM032588.1	293298	.	A	G	100	*	TYPE=SUBSTITUTE
CM032588.1	293301	.	C	T	100	*	TYPE=SUBSTITUTE
CM032588.1	293317	.	A	G	100	*	TYPE=SUBSTITUTE
CM032588.1	293326	.	C	T	100	*	TYPE=SUBSTITUTE
CM032588.1	293335	.	G	C	100	*	TYPE=SUBSTITUTE
CM032588.1	293352	.	A	C	100	*	TYPE=SUBSTITUTE
CM032588.1	293384	.	T	G	100	*	TYPE=SUBSTITUTE
CM032588.1	293395	.	A	G	100	*	TYPE=SUBSTITUTE
CM032588.1	293396	.	C	A	100	*	TYPE=SUBSTITUTE
CM032588.1	293409	.	A	G	100	*	TYPE=SUBSTITUTE
CM032588.1	293482	.	C	T	100	*	TYPE=SUBSTITUTE
CM032588.1	293486	.	A	G	100	*	TYPE=SUBSTITUTE
CM032588.1	293567	.	G	GG	100	*	TYPE=INSERT
CM032588.1	293576	.	C	T	100	*	TYPE=SUBSTITUTE
CM032588.1	293631	.	G	A	100	*	TYPE=SUBSTITUTE
CM032588.1	293717	.	T	C	100	*	TYPE=SUBSTITUTE
CM032588.1	293801	.	T	C	100	*	TYPE=SUBSTITUTE
CM032588.1	293829	.	G	A	100	*	TYPE=SUBSTITUTE
CM032588.1	293858	.	A	G	100	*	TYPE=SUBSTITUTE
CM032588.1	293926	.	A	G	100	*	TYPE=SUBSTITUTE
CM032588.1	293942	.	A	ac	100	*	TYPE=INSERT
CM032588.1	293990	.	C	T	100	*	TYPE=SUBSTITUTE
CM032588.1	293992	.	A	G	100	*	TYPE=SUBSTITUTE
CM032588.1	294074	.	C	t	100	*	TYPE=SUBSTITUTE
CM032588.1	294095	.	T	C	100	*	TYPE=SUBSTITUTE
CM032588.1	294104	.	C	G	100	*	TYPE=SUBSTITUTE
CM032588.1	294121	.	A	G	100	*	TYPE=SUBSTITUTE
CM032588.1	294126	.	T	C	100	*	TYPE=SUBSTITUTE
CM032588.1	294127	.	G	A	100	*	TYPE=SUBSTITUTE
CM032588.1	294131	.	T	A	100	*	TYPE=SUBSTITUTE
CM032588.1	294136	.	A	G	100	*	TYPE=SUBSTITUTE
CM032588.1	294137	.	T	C	100	*	TYPE=SUBSTITUTE
CM032588.1	294138	.	T	C	100	*	TYPE=SUBSTITUTE
CM032588.1	294147	.	T	G	100	*	TYPE=SUBSTITUTE
CM032588.1	294150	.	C	G	100	*	TYPE=SUBSTITUTE
CM032588.1	294162	.	T	C	100	*	TYPE=SUBSTITUTE
CM032588.1	294163	.	T	C	100	*	TYPE=SUBSTITUTE
CM032588.1	294164	.	T	C	100	*	TYPE=SUBSTITUTE
CM032588.1	294167	.	AG	A	100	*	TYPE=DELETE
CM032588.1	294183	.	C	T	100	*	TYPE=SUBSTITUTE
CM032588.1	294195	.	C	T	100	*	TYPE=SUBSTITUTE
CM032588.1	294207	.	T	C	100	*	TYPE=SUBSTITUTE
CM032588.1	294237	.	C	T	100	*	TYPE=SUBSTITUTE
CM032588.1	294253	.	T	C	100	*	TYPE=SUBSTITUTE
CM032588.1	294262	.	T	C	100	*	TYPE=SUBSTITUTE
CM032588.1	294263	.	G	A	100	*	TYPE=SUBSTITUTE
CM032588.1	294271	.	A	G	100	*	TYPE=SUBSTITUTE
CM032588.1	294273	.	G	A	100	*	TYPE=SUBSTITUTE
CM032588.1	294280	.	G	C	100	*	TYPE=SUBSTITUTE
CM032588.1	294283	.	A	G	100	*	TYPE=SUBSTITUTE
CM032588.1	294340	.	T	C	100	*	TYPE=SUBSTITUTE
CM032588.1	294360	.	C	T	100	*	TYPE=SUBSTITUTE
CM032588.1	294378	.	A	G	100	*	TYPE=SUBSTITUTE
CM032588.1	294393	.	T	C	100	*	TYPE=SUBSTITUTE
CM032588.1	294399	.	G	T	100	*	TYPE=SUBSTITUTE
CM032588.1	294408	.	T	C	100	*	TYPE=SUBSTITUTE
CM032588.1	294456	.	A	T	100	*	TYPE=SUBSTITUTE
CM032588.1	294466	.	A	T	100	*	TYPE=SUBSTITUTE
CM032588.1	294489	.	C	T	100	*	TYPE=SUBSTITUTE
CM032588.1	294555	.	T	C	100	*	TYPE=SUBSTITUTE
CM032588.1	294564	.	T	C	100	*	TYPE=SUBSTITUTE
CM032588.1	294636	.	C	T	100	*	TYPE=SUBSTITUTE

CM032588.1	294648	.	T	C	100	*	TYPE=SUBSTITUTE
CM032588.1	294791	.	G	A	100	*	TYPE=SUBSTITUTE
CM032588.1	294807	.	C	T	100	*	TYPE=SUBSTITUTE
CM032588.1	294819	.	A	T	100	*	TYPE=SUBSTITUTE
CM032588.1	294823	.	A	G	100	*	TYPE=SUBSTITUTE
CM032588.1	294852	.	T	C	100	*	TYPE=SUBSTITUTE
CM032588.1	294866	.	G	A	100	*	TYPE=SUBSTITUTE
CM032588.1	294869	.	A	C	100	*	TYPE=SUBSTITUTE
CM032588.1	294877	.	A	G	100	*	TYPE=SUBSTITUTE
CM032588.1	294892	.	T	A	100	*	TYPE=SUBSTITUTE
CM032588.1	294909	.	C	A	100	*	TYPE=SUBSTITUTE
CM032588.1	294924	.	T	C	100	*	TYPE=SUBSTITUTE
CM032588.1	294934	.	A	T	100	*	TYPE=SUBSTITUTE
CM032588.1	294951	.	G	A	100	*	TYPE=SUBSTITUTE
CM032588.1	294957	.	A	C	100	*	TYPE=SUBSTITUTE
CM032588.1	294961	.	C	T	100	*	TYPE=SUBSTITUTE
CM032588.1	294979	.	A	G	100	*	TYPE=SUBSTITUTE
CM032588.1	295019	.	C	T	100	*	TYPE=SUBSTITUTE
CM032588.1	295027	.	C	A	100	*	TYPE=SUBSTITUTE
CM032588.1	295039	.	A	G	100	*	TYPE=SUBSTITUTE
CM032588.1	295086	.	C	G	100	*	TYPE=SUBSTITUTE
CM032588.1	295090	.	T	C	100	*	TYPE=SUBSTITUTE
CM032588.1	295094	.	C	T	100	*	TYPE=SUBSTITUTE
CM032588.1	295095	.	C	T	100	*	TYPE=SUBSTITUTE
CM032588.1	295096	.	G	A	100	*	TYPE=SUBSTITUTE
CM032588.1	295105	.	A	G	100	*	TYPE=SUBSTITUTE
CM032588.1	295123	.	G	A	100	*	TYPE=SUBSTITUTE
CM032588.1	295135	.	T	A	100	*	TYPE=SUBSTITUTE
CM032588.1	295143	.	C	A	100	*	TYPE=SUBSTITUTE
CM032588.1	295147	.	T	C	100	*	TYPE=SUBSTITUTE
CM032588.1	295185	.	A	G	100	*	TYPE=SUBSTITUTE
CM032588.1	295186	.	T	C	100	*	TYPE=SUBSTITUTE
CM032588.1	295189	.	T	C	100	*	TYPE=SUBSTITUTE
CM032588.1	295225	.	T	C	100	*	TYPE=SUBSTITUTE
CM032588.1	295237	.	T	C	100	*	TYPE=SUBSTITUTE
CM032588.1	295238	.	G	A	100	*	TYPE=SUBSTITUTE
CM032588.1	295243	.	T	C	100	*	TYPE=SUBSTITUTE
CM032588.1	295248	.	C	T	100	*	TYPE=SUBSTITUTE
CM032588.1	295269	.	T	A	100	*	TYPE=SUBSTITUTE
CM032588.1	295273	.	T	C	100	*	TYPE=SUBSTITUTE
CM032588.1	295275	.	A	T	100	*	TYPE=SUBSTITUTE
CM032588.1	295276	.	A	G	100	*	TYPE=SUBSTITUTE
CM032588.1	295279	.	T	A	100	*	TYPE=SUBSTITUTE
CM032588.1	295285	.	A	G	100	*	TYPE=SUBSTITUTE
CM032588.1	295288	.	C	T	100	*	TYPE=SUBSTITUTE
CM032588.1	295312	.	A	G	100	*	TYPE=SUBSTITUTE
CM032588.1	295351	.	C	T	100	*	TYPE=SUBSTITUTE
CM032588.1	295354	.	C	A	100	*	TYPE=SUBSTITUTE
CM032588.1	295366	.	C	T	100	*	TYPE=SUBSTITUTE
CM032588.1	295369	.	T	G	100	*	TYPE=SUBSTITUTE
CM032588.1	295377	.	C	T	100	*	TYPE=SUBSTITUTE
CM032588.1	295378	.	T	C	100	*	TYPE=SUBSTITUTE
CM032588.1	295411	.	G	A	100	*	TYPE=SUBSTITUTE
CM032588.1	295426	.	G	A	100	*	TYPE=SUBSTITUTE
CM032588.1	295429	.	A	G	100	*	TYPE=SUBSTITUTE
CM032588.1	295441	.	G	A	100	*	TYPE=SUBSTITUTE
CM032588.1	295444	.	C	T	100	*	TYPE=SUBSTITUTE
CM032588.1	295462	.	A	G	100	*	TYPE=SUBSTITUTE
CM032588.1	295465	.	A	G	100	*	TYPE=SUBSTITUTE
CM032588.1	295477	.	T	C	100	*	TYPE=SUBSTITUTE
CM032588.1	295486	.	G	A	100	*	TYPE=SUBSTITUTE
CM032588.1	295498	.	A	C	100	*	TYPE=SUBSTITUTE
CM032588.1	295510	.	T	A	100	*	TYPE=SUBSTITUTE
CM032588.1	295513	.	T	A	100	*	TYPE=SUBSTITUTE

CM032588.1	295566	.	A	G	100	*	TYPE=SUBSTITUTE
CM032588.1	295581	.	T	C	100	*	TYPE=SUBSTITUTE
CM032588.1	295588	.	A	G	100	*	TYPE=SUBSTITUTE
CM032588.1	295594	.	A	C	100	*	TYPE=SUBSTITUTE
CM032588.1	295635	.	T	C	100	*	TYPE=SUBSTITUTE
CM032588.1	295663	.	T	C	100	*	TYPE=SUBSTITUTE
CM032588.1	295666	.	G	C	100	*	TYPE=SUBSTITUTE
CM032588.1	295668	.	T	C	100	*	TYPE=SUBSTITUTE
CM032588.1	295670	.	G	A	100	*	TYPE=SUBSTITUTE
CM032588.1	295672	.	G	C	100	*	TYPE=SUBSTITUTE
CM032588.1	295674	.	C	t	100	*	TYPE=SUBSTITUTE
CM032588.1	295735	.	G	T	100	*	TYPE=SUBSTITUTE
CM032588.1	295738	.	A	G	100	*	TYPE=SUBSTITUTE
CM032588.1	295744	.	C	G	100	*	TYPE=SUBSTITUTE
CM032588.1	295774	.	T	C	100	*	TYPE=SUBSTITUTE
CM032588.1	295783	.	T	A	100	*	TYPE=SUBSTITUTE
CM032588.1	295816	.	G	A	100	*	TYPE=SUBSTITUTE
CM032588.1	295825	.	G	A	100	*	TYPE=SUBSTITUTE
CM032588.1	295834	.	A	G	100	*	TYPE=SUBSTITUTE
CM032588.1	295837	.	C	T	100	*	TYPE=SUBSTITUTE
CM032588.1	295872	.	T	C	100	*	TYPE=SUBSTITUTE
CM032588.1	295902	.	C	T	100	*	TYPE=SUBSTITUTE
CM032588.1	295903	.	A	G	100	*	TYPE=SUBSTITUTE
CM032588.1	295915	.	A	G	100	*	TYPE=SUBSTITUTE
CM032588.1	295921	.	A	G	100	*	TYPE=SUBSTITUTE
CM032588.1	295931	.	G	A	100	*	TYPE=SUBSTITUTE
CM032588.1	295942	.	A	G	100	*	TYPE=SUBSTITUTE
CM032588.1	295945	.	A	G	100	*	TYPE=SUBSTITUTE
CM032588.1	295948	.	G	A	100	*	TYPE=SUBSTITUTE
CM032588.1	295960	.	T	C	100	*	TYPE=SUBSTITUTE
CM032588.1	295963	.	G	A	100	*	TYPE=SUBSTITUTE
CM032588.1	295969	.	G	A	100	*	TYPE=SUBSTITUTE
CM032588.1	295972	.	A	G	100	*	TYPE=SUBSTITUTE
CM032588.1	295975	.	T	C	100	*	TYPE=SUBSTITUTE
CM032588.1	296020	.	A	G	100	*	TYPE=SUBSTITUTE
CM032588.1	296028	.	G	A	100	*	TYPE=SUBSTITUTE
CM032588.1	296053	.	C	T	100	*	TYPE=SUBSTITUTE
CM032588.1	296108	.	G	A	100	*	TYPE=SUBSTITUTE
CM032588.1	296109	.	G	A	100	*	TYPE=SUBSTITUTE
CM032588.1	296116	.	A	G	100	*	TYPE=SUBSTITUTE
CM032588.1	296178	.	T	C	100	*	TYPE=SUBSTITUTE
CM032588.1	296269	.	A	G	100	*	TYPE=SUBSTITUTE
CM032588.1	296284	.	C	T	100	*	TYPE=SUBSTITUTE
CM032588.1	296317	.	A	G	100	*	TYPE=SUBSTITUTE
CM032588.1	296362	.	C	T	100	*	TYPE=SUBSTITUTE
CM032588.1	296364	.	C	T	100	*	TYPE=SUBSTITUTE
CM032588.1	296365	.	T	C	100	*	TYPE=SUBSTITUTE
CM032588.1	296371	.	T	C	100	*	TYPE=SUBSTITUTE
CM032588.1	296386	.	C	T	100	*	TYPE=SUBSTITUTE
CM032588.1	296389	.	A	G	100	*	TYPE=SUBSTITUTE
CM032588.1	296404	.	T	G	100	*	TYPE=SUBSTITUTE
CM032588.1	296416	.	G	A	100	*	TYPE=SUBSTITUTE
CM032588.1	296434	.	T	C	100	*	TYPE=SUBSTITUTE
CM032588.1	296440	.	G	T	100	*	TYPE=SUBSTITUTE
CM032588.1	296446	.	G	C	100	*	TYPE=SUBSTITUTE
CM032588.1	296455	.	A	G	100	*	TYPE=SUBSTITUTE
CM032588.1	296456	.	G	A	100	*	TYPE=SUBSTITUTE
CM032588.1	296458	.	C	T	100	*	TYPE=SUBSTITUTE
CM032588.1	296479	.	A	G	100	*	TYPE=SUBSTITUTE
CM032588.1	296487	.	A	G	100	*	TYPE=SUBSTITUTE
CM032588.1	296497	.	G	T	100	*	TYPE=SUBSTITUTE
CM032588.1	296498	.	T	C	100	*	TYPE=SUBSTITUTE
CM032588.1	296536	.	C	T	100	*	TYPE=SUBSTITUTE
CM032588.1	296542	.	C	A	100	*	TYPE=SUBSTITUTE

CM032588.1	296551	.	C	A	100	*	TYPE=SUBSTITUTE
CM032588.1	296566	.	G	A	100	*	TYPE=SUBSTITUTE
CM032588.1	296578	.	A	C	100	*	TYPE=SUBSTITUTE
CM032588.1	296627	.	A	T	100	*	TYPE=SUBSTITUTE
CM032588.1	296630	.	G	A	100	*	TYPE=SUBSTITUTE
CM032588.1	296654	.	A	G	100	*	TYPE=SUBSTITUTE
CM032588.1	296663	.	G	C	100	*	TYPE=SUBSTITUTE
CM032588.1	296666	.	A	C	100	*	TYPE=SUBSTITUTE
CM032588.1	296688	.	T	G	100	*	TYPE=SUBSTITUTE
CM032588.1	296699	.	A	C	100	*	TYPE=SUBSTITUTE
CM032588.1	296700	.	A	G	100	*	TYPE=SUBSTITUTE
CM032588.1	296711	.	G	A	100	*	TYPE=SUBSTITUTE
CM032588.1	296722	.	C	T	100	*	TYPE=SUBSTITUTE
CM032588.1	296755	.	G	A	100	*	TYPE=SUBSTITUTE
CM032588.1	296765	.	T	C	100	*	TYPE=SUBSTITUTE
CM032588.1	296788	.	G	C	100	*	TYPE=SUBSTITUTE
CM032588.1	296827	.	AT	A	100	*	TYPE=DELETE
CM032588.1	296830	.	A	C	100	*	TYPE=SUBSTITUTE
CM032588.1	296847	.	G	C	100	*	TYPE=SUBSTITUTE
CM032588.1	296857	.	G	A	100	*	TYPE=SUBSTITUTE
CM032588.1	296858	.	C	T	100	*	TYPE=SUBSTITUTE
CM032588.1	296893	.	C	A	100	*	TYPE=SUBSTITUTE
CM032588.1	296919	.	A	G	100	*	TYPE=SUBSTITUTE
CM032588.1	296929	.	C	T	100	*	TYPE=SUBSTITUTE
CM032588.1	296930	.	G	C	100	*	TYPE=SUBSTITUTE
CM032588.1	296934	.	A	C	100	*	TYPE=SUBSTITUTE
CM032588.1	296939	.	A	G	100	*	TYPE=SUBSTITUTE
CM032588.1	296942	.	C	A	100	*	TYPE=SUBSTITUTE
CM032588.1	296973	.	C	G	100	*	TYPE=SUBSTITUTE
CM032588.1	296978	.	T	C	100	*	TYPE=SUBSTITUTE
CM032588.1	297007	.	GA	G	100	*	TYPE=DELETE
CM032588.1	297011	.	T	G	100	*	TYPE=SUBSTITUTE
CM032588.1	297013	.	C	G	100	*	TYPE=SUBSTITUTE
CM032588.1	297025	.	A	G	100	*	TYPE=SUBSTITUTE
CM032588.1	297030	.	T	C	100	*	TYPE=SUBSTITUTE
CM032588.1	297034	.	C	T	100	*	TYPE=SUBSTITUTE
CM032588.1	297051	.	T	G	100	*	TYPE=SUBSTITUTE
CM032588.1	297072	.	C	T	100	*	TYPE=SUBSTITUTE
CM032588.1	297073	.	G	A	100	*	TYPE=SUBSTITUTE
CM032588.1	297131	.	C	T	100	*	TYPE=SUBSTITUTE
CM032588.1	297134	.	C	T	100	*	TYPE=SUBSTITUTE
CM032588.1	297148	.	T	C	100	*	TYPE=SUBSTITUTE
CM032588.1	297150	.	C	T	100	*	TYPE=SUBSTITUTE
CM032588.1	297157	.	T	C	100	*	TYPE=SUBSTITUTE
CM032588.1	297195	.	T	C	100	*	TYPE=SUBSTITUTE
CM032588.1	297199	.	A	G	100	*	TYPE=SUBSTITUTE
CM032588.1	297200	.	G	T	100	*	TYPE=SUBSTITUTE
CM032588.1	297208	.	TGCATGAGGCCG	T	100	*	TYPE=DELETE
CM032588.1	297286	.	G	A	100	*	TYPE=SUBSTITUTE
CM032588.1	297299	.	G	A	100	*	TYPE=SUBSTITUTE
CM032588.1	297302	.	T	C	100	*	TYPE=SUBSTITUTE
CM032588.1	297308	.	C	T	100	*	TYPE=SUBSTITUTE
CM032588.1	297311	.	G	A	100	*	TYPE=SUBSTITUTE
CM032588.1	297344	.	G	A	100	*	TYPE=SUBSTITUTE
CM032588.1	297350	.	C	T	100	*	TYPE=SUBSTITUTE
CM032588.1	297360	.	CT	C	100	*	TYPE=DELETE
CM032588.1	297377	.	T	TTGCC	100	*	TYPE=INSERT
CM032588.1	297384	.	T	C	100	*	TYPE=SUBSTITUTE
CM032588.1	297389	.	G	T	100	*	TYPE=SUBSTITUTE
CM032588.1	297391	.	A	G	100	*	TYPE=SUBSTITUTE
CM032588.1	297396	.	G	A	100	*	TYPE=SUBSTITUTE
CM032588.1	297397	.	A	T	100	*	TYPE=SUBSTITUTE
CM032588.1	297400	.	T	C	100	*	TYPE=SUBSTITUTE
CM032588.1	297401	.	T	A	100	*	TYPE=SUBSTITUTE

CM032588.1	297402	.	G	A	100	*	TYPE=SUBSTITUTE
CM032588.1	297413	.	A	G	100	*	TYPE=SUBSTITUTE
CM032588.1	297425	.	A	G	100	*	TYPE=SUBSTITUTE
CM032588.1	297440	.	G	A	100	*	TYPE=SUBSTITUTE
CM032588.1	297446	.	A	G	100	*	TYPE=SUBSTITUTE
CM032588.1	297449	.	C	T	100	*	TYPE=SUBSTITUTE
CM032588.1	297452	.	T	C	100	*	TYPE=SUBSTITUTE
CM032588.1	297473	.	T	A	100	*	TYPE=SUBSTITUTE
CM032588.1	297479	.	A	G	100	*	TYPE=SUBSTITUTE
CM032588.1	297491	.	G	A	100	*	TYPE=SUBSTITUTE
CM032588.1	297497	.	G	A	100	*	TYPE=SUBSTITUTE
CM032588.1	297542	.	C	T	100	*	TYPE=SUBSTITUTE
CM032588.1	297563	.	G	A	100	*	TYPE=SUBSTITUTE
CM032588.1	297569	.	T	A	100	*	TYPE=SUBSTITUTE
CM032588.1	297578	.	A	C	100	*	TYPE=SUBSTITUTE
CM032588.1	297587	.	G	A	100	*	TYPE=SUBSTITUTE
CM032588.1	297599	.	T	C	100	*	TYPE=SUBSTITUTE
CM032588.1	297605	.	G	C	100	*	TYPE=SUBSTITUTE
CM032588.1	297607	.	G	A	100	*	TYPE=SUBSTITUTE
CM032588.1	297629	.	G	A	100	*	TYPE=SUBSTITUTE
CM032588.1	297660	.	CA	C	100	*	TYPE=DELETE
CM032588.1	297664	.	C	G	100	*	TYPE=SUBSTITUTE
CM032588.1	297687	.	T	C	100	*	TYPE=SUBSTITUTE
CM032588.1	297691	.	C	A	100	*	TYPE=SUBSTITUTE
CM032588.1	297693	.	A	G	100	*	TYPE=SUBSTITUTE
CM032588.1	297694	.	C	T	100	*	TYPE=SUBSTITUTE
CM032588.1	297695	.	T	C	100	*	TYPE=SUBSTITUTE
CM032588.1	297719	.	A	G	100	*	TYPE=SUBSTITUTE
CM032588.1	297744	.	A	G	100	*	TYPE=SUBSTITUTE
CM032588.1	297762	.	G	T	100	*	TYPE=SUBSTITUTE
CM032588.1	297765	.	G	A	100	*	TYPE=SUBSTITUTE
CM032588.1	297777	.	G	T	100	*	TYPE=SUBSTITUTE
CM032588.1	297783	.	C	T	100	*	TYPE=SUBSTITUTE
CM032588.1	297786	.	C	T	100	*	TYPE=SUBSTITUTE
CM032588.1	297828	.	A	g	100	*	TYPE=SUBSTITUTE
CM032588.1	297841	.	G	T	100	*	TYPE=SUBSTITUTE
CM032588.1	297856	.	G	A	100	*	TYPE=SUBSTITUTE
CM032588.1	297857	.	A	T	100	*	TYPE=SUBSTITUTE
CM032588.1	297859	.	A	G	100	*	TYPE=SUBSTITUTE
CM032588.1	297862	.	C	T	100	*	TYPE=SUBSTITUTE
CM032588.1	297865	.	G	A	100	*	TYPE=SUBSTITUTE
CM032588.1	297870	.	G	C	100	*	TYPE=SUBSTITUTE
CM032588.1	297871	.	A	G	100	*	TYPE=SUBSTITUTE
CM032588.1	297873	.	C	T	100	*	TYPE=SUBSTITUTE
CM032588.1	297875	.	A	T	100	*	TYPE=SUBSTITUTE
CM032588.1	297876	.	A	G	100	*	TYPE=SUBSTITUTE
CM032588.1	297879	.	G	A	100	*	TYPE=SUBSTITUTE
CM032588.1	297880	.	G	T	100	*	TYPE=SUBSTITUTE
CM032588.1	297882	.	A	G	100	*	TYPE=SUBSTITUTE
CM032588.1	297886	.	T	C	100	*	TYPE=SUBSTITUTE
CM032588.1	297887	.	T	A	100	*	TYPE=SUBSTITUTE
CM032588.1	297890	.	T	C	100	*	TYPE=SUBSTITUTE
CM032588.1	297903	.	A	G	100	*	TYPE=SUBSTITUTE
CM032588.1	297947	.	A	G	100	*	TYPE=SUBSTITUTE
CM032588.1	297963	.	T	A	100	*	TYPE=SUBSTITUTE
CM032588.1	297972	.	A	G	100	*	TYPE=SUBSTITUTE
CM032588.1	298002	.	C	G	100	*	TYPE=SUBSTITUTE
CM032588.1	298011	.	C	T	100	*	TYPE=SUBSTITUTE
CM032588.1	298016	.	G	A	100	*	TYPE=SUBSTITUTE
CM032588.1	298026	.	G	A	100	*	TYPE=SUBSTITUTE
CM032588.1	298029	.	T	C	100	*	TYPE=SUBSTITUTE
CM032588.1	298031	.	G	A	100	*	TYPE=SUBSTITUTE
CM032588.1	298035	.	T	C	100	*	TYPE=SUBSTITUTE
CM032588.1	298037	.	G	A	100	*	TYPE=SUBSTITUTE

CM032588.1	298038	.	C	T	100	*	TYPE=SUBSTITUTE
CM032588.1	298050	.	A	G	100	*	TYPE=SUBSTITUTE
CM032588.1	298051	.	T	C	100	*	TYPE=SUBSTITUTE
CM032588.1	298056	.	T	C	100	*	TYPE=SUBSTITUTE
CM032588.1	298064	.	A	C	100	*	TYPE=SUBSTITUTE
CM032588.1	298071	.	C	T	100	*	TYPE=SUBSTITUTE
CM032588.1	298083	.	C	T	100	*	TYPE=SUBSTITUTE
CM032588.1	298085	.	A	G	100	*	TYPE=SUBSTITUTE
CM032588.1	298089	.	A	G	100	*	TYPE=SUBSTITUTE
CM032588.1	298095	.	T	C	100	*	TYPE=SUBSTITUTE
CM032588.1	298098	.	T	C	100	*	TYPE=SUBSTITUTE
CM032588.1	298101	.	G	C	100	*	TYPE=SUBSTITUTE
CM032588.1	298170	.	T	C	100	*	TYPE=SUBSTITUTE
CM032588.1	298176	.	C	T	100	*	TYPE=SUBSTITUTE
CM032588.1	298197	.	G	A	100	*	TYPE=SUBSTITUTE
CM032588.1	298203	.	A	G	100	*	TYPE=SUBSTITUTE
CM032588.1	298232	.	G	C	100	*	TYPE=SUBSTITUTE
CM032588.1	298239	.	A	T	100	*	TYPE=SUBSTITUTE
CM032588.1	298254	.	A	G	100	*	TYPE=SUBSTITUTE
CM032588.1	298269	.	T	C	100	*	TYPE=SUBSTITUTE
CM032588.1	298275	.	T	C	100	*	TYPE=SUBSTITUTE
CM032588.1	298284	.	C	T	100	*	TYPE=SUBSTITUTE
CM032588.1	298301	.	A	G	100	*	TYPE=SUBSTITUTE
CM032588.1	298311	.	G	A	100	*	TYPE=SUBSTITUTE
CM032588.1	298317	.	G	A	100	*	TYPE=SUBSTITUTE
CM032588.1	298344	.	G	A	100	*	TYPE=SUBSTITUTE
CM032588.1	298367	.	G	C	100	*	TYPE=SUBSTITUTE
CM032588.1	298386	.	T	G	100	*	TYPE=SUBSTITUTE
CM032588.1	298407	.	A	T	100	*	TYPE=SUBSTITUTE
CM032588.1	298416	.	C	T	100	*	TYPE=SUBSTITUTE
CM032588.1	298431	.	G	C	100	*	TYPE=SUBSTITUTE
CM032588.1	298446	.	G	C	100	*	TYPE=SUBSTITUTE
CM032588.1	298464	.	A	G	100	*	TYPE=SUBSTITUTE
CM032588.1	298479	.	G	A	100	*	TYPE=SUBSTITUTE
CM032588.1	298482	.	T	C	100	*	TYPE=SUBSTITUTE
CM032588.1	298502	.	A	C	100	*	TYPE=SUBSTITUTE
CM032588.1	298505	.	C	T	100	*	TYPE=SUBSTITUTE
CM032588.1	298509	.	T	G	100	*	TYPE=SUBSTITUTE
CM032588.1	298525	.	C	T	100	*	TYPE=SUBSTITUTE
CM032588.1	298555	.	G	A	100	*	TYPE=SUBSTITUTE
CM032588.1	298558	.	G	A	100	*	TYPE=SUBSTITUTE
CM032588.1	298560	.	T	C	100	*	TYPE=SUBSTITUTE
CM032588.1	298572	.	GTT	G	100	*	TYPE=DELETE
CM032588.1	298610	.	C	T	100	*	TYPE=SUBSTITUTE
CM032588.1	298614	.	T	C	100	*	TYPE=SUBSTITUTE
CM032588.1	298652	.	A	T	100	*	TYPE=SUBSTITUTE
CM032588.1	298653	.	G	T	100	*	TYPE=SUBSTITUTE
CM032588.1	298654	.	A	T	100	*	TYPE=SUBSTITUTE
CM032588.1	298663	.	G	A	100	*	TYPE=SUBSTITUTE
CM032588.1	298666	.	G	T	100	*	TYPE=SUBSTITUTE
CM032588.1	298667	.	A	C	100	*	TYPE=SUBSTITUTE
CM032588.1	298668	.	G	A	100	*	TYPE=SUBSTITUTE
CM032588.1	298672	.	T	G	100	*	TYPE=SUBSTITUTE
CM032588.1	298673	.	G	A	100	*	TYPE=SUBSTITUTE
CM032588.1	298677	.	T	C	100	*	TYPE=SUBSTITUTE
CM032588.1	298680	.	C	A	100	*	TYPE=SUBSTITUTE
CM032588.1	298681	.	C	T	100	*	TYPE=SUBSTITUTE
CM032588.1	298684	.	A	G	100	*	TYPE=SUBSTITUTE
CM032588.1	298687	.	G	T	100	*	TYPE=SUBSTITUTE
CM032588.1	298693	.	T	C	100	*	TYPE=SUBSTITUTE
CM032588.1	298733	.	T	C	100	*	TYPE=SUBSTITUTE
CM032588.1	298763	.	G	A	100	*	TYPE=SUBSTITUTE
CM032588.1	298769	.	G	A	100	*	TYPE=SUBSTITUTE
CM032588.1	298784	.	C	T	100	*	TYPE=SUBSTITUTE

CM032588.1	298790	.	G	C	100	*	TYPE=SUBSTITUTE
CM032588.1	298793	.	A	G	100	*	TYPE=SUBSTITUTE
CM032588.1	298796	.	T	C	100	*	TYPE=SUBSTITUTE
CM032588.1	298805	.	A	G	100	*	TYPE=SUBSTITUTE
CM032588.1	298848	.	AA	A	100	*	TYPE=DELETE
CM032588.1	298857	.	T	TC	100	*	TYPE=INSERT
CM032588.1	298862	.	T	G	100	*	TYPE=SUBSTITUTE
CM032588.1	298863	.	C	T	100	*	TYPE=SUBSTITUTE
CM032588.1	298865	.	A	C	100	*	TYPE=SUBSTITUTE
CM032588.1	298878	.	T	C	100	*	TYPE=SUBSTITUTE
CM032588.1	298879	.	T	A	100	*	TYPE=SUBSTITUTE
CM032588.1	298880	.	G	A	100	*	TYPE=SUBSTITUTE
CM032588.1	298884	.	C	G	100	*	TYPE=SUBSTITUTE
CM032588.1	298885	.	A	G	100	*	TYPE=SUBSTITUTE
CM032588.1	298910	.	A	G	100	*	TYPE=SUBSTITUTE
CM032588.1	298928	.	T	C	100	*	TYPE=SUBSTITUTE
CM032588.1	298943	.	G	a	100	*	TYPE=SUBSTITUTE
CM032588.1	298949	.	A	g	100	*	TYPE=SUBSTITUTE
CM032588.1	298952	.	G	a	100	*	TYPE=SUBSTITUTE
CM032588.1	298955	.	C	g	100	*	TYPE=SUBSTITUTE
CM032588.1	298961	.	G	A	100	*	TYPE=SUBSTITUTE
CM032588.1	299003	.	T	G	100	*	TYPE=SUBSTITUTE
CM032588.1	299012	.	G	A	100	*	TYPE=SUBSTITUTE
CM032588.1	299021	.	C	T	100	*	TYPE=SUBSTITUTE
CM032588.1	299030	.	T	G	100	*	TYPE=SUBSTITUTE
CM032588.1	299033	.	G	A	100	*	TYPE=SUBSTITUTE
CM032588.1	299043	.	CT	C	100	*	TYPE=DELETE
CM032588.1	299046	.	T	g	100	*	TYPE=SUBSTITUTE
CM032588.1	299048	.	TC	T	100	*	TYPE=DELETE
CM032588.1	299053	.	A	g	100	*	TYPE=SUBSTITUTE
CM032588.1	299060	.	C	T	100	*	TYPE=SUBSTITUTE
CM032588.1	299063	.	T	C	100	*	TYPE=SUBSTITUTE
CM032588.1	299067	.	A	T	100	*	TYPE=SUBSTITUTE
CM032588.1	299074	.	A	G	100	*	TYPE=SUBSTITUTE
CM032588.1	299089	.	A	T	100	*	TYPE=SUBSTITUTE
CM032588.1	299107	.	A	G	100	*	TYPE=SUBSTITUTE
CM032588.1	299116	.	C	A	100	*	TYPE=SUBSTITUTE
CM032588.1	299118	.	C	T	100	*	TYPE=SUBSTITUTE
CM032588.1	299122	.	C	T	100	*	TYPE=SUBSTITUTE
CM032588.1	299149	.	A	C	100	*	TYPE=SUBSTITUTE
CM032588.1	299157	.	T	C	100	*	TYPE=SUBSTITUTE
CM032588.1	299182	.	A	G	100	*	TYPE=SUBSTITUTE
CM032588.1	299185	.	A	G	100	*	TYPE=SUBSTITUTE
CM032588.1	299188	.	G	A	100	*	TYPE=SUBSTITUTE
CM032588.1	299202	.	T	G	100	*	TYPE=SUBSTITUTE
CM032588.1	299221	.	G	A	100	*	TYPE=SUBSTITUTE
CM032588.1	299245	.	C	T	100	*	TYPE=SUBSTITUTE
CM032588.1	299278	.	G	T	100	*	TYPE=SUBSTITUTE
CM032588.1	299289	.	C	G	100	*	TYPE=SUBSTITUTE
CM032588.1	299305	.	T	C	100	*	TYPE=SUBSTITUTE
CM032588.1	299306	.	A	G	100	*	TYPE=SUBSTITUTE
CM032588.1	299310	.	G	A	100	*	TYPE=SUBSTITUTE
CM032588.1	299311	.	A	G	100	*	TYPE=SUBSTITUTE
CM032588.1	299321	.	G	A	100	*	TYPE=SUBSTITUTE
CM032588.1	299322	.	G	A	100	*	TYPE=SUBSTITUTE
CM032588.1	299323	.	A	AT	100	*	TYPE=INSERT
CM032588.1	299327	.	T	C	100	*	TYPE=SUBSTITUTE
CM032588.1	299330	.	A	G	100	*	TYPE=SUBSTITUTE
CM032588.1	299333	.	T	C	100	*	TYPE=SUBSTITUTE
CM032588.1	299354	.	A	G	100	*	TYPE=SUBSTITUTE
CM032588.1	299359	.	G	A	100	*	TYPE=SUBSTITUTE
CM032588.1	299367	.	G	A	100	*	TYPE=SUBSTITUTE
CM032588.1	299377	.	C	T	100	*	TYPE=SUBSTITUTE
CM032588.1	299379	.	A	G	100	*	TYPE=SUBSTITUTE

CM032588.1	299381	.	G	A	100	*	TYPE=SUBSTITUTE
CM032588.1	299382	.	C	A	100	*	TYPE=SUBSTITUTE
CM032588.1	299392	.	A	G	100	*	TYPE=SUBSTITUTE
CM032588.1	299393	.	A	G	100	*	TYPE=SUBSTITUTE
CM032588.1	299398	.	T	C	100	*	TYPE=SUBSTITUTE
CM032588.1	299420	.	A	C	100	*	TYPE=SUBSTITUTE
CM032588.1	299422	.	C	T	100	*	TYPE=SUBSTITUTE
CM032588.1	299423	.	T	C	100	*	TYPE=SUBSTITUTE
CM032588.1	299432	.	G	A	100	*	TYPE=SUBSTITUTE
CM032588.1	299454	.	C	T	100	*	TYPE=SUBSTITUTE
CM032588.1	299457	.	G	A	100	*	TYPE=SUBSTITUTE
CM032588.1	299469	.	T	a	100	*	TYPE=SUBSTITUTE
CM032588.1	299470	.	C	g	100	*	TYPE=SUBSTITUTE
CM032588.1	299488	.	A	g	100	*	TYPE=SUBSTITUTE
CM032588.1	299490	.	G	a	100	*	TYPE=SUBSTITUTE
CM032588.1	299492	.	G	c	100	*	TYPE=SUBSTITUTE
CM032588.1	299497	.	G	a	100	*	TYPE=SUBSTITUTE
CM032588.1	299504	.	T	G	100	*	TYPE=SUBSTITUTE
CM032588.1	299510	.	G	A	100	*	TYPE=SUBSTITUTE
CM032588.1	299511	.	A	C	100	*	TYPE=SUBSTITUTE
CM032588.1	299533	.	C	T	100	*	TYPE=SUBSTITUTE
CM032588.1	299564	.	C	T	100	*	TYPE=SUBSTITUTE
CM032588.1	299567	.	T	TG	100	*	TYPE=INSERT
CM032588.1	299569	.	A	C	100	*	TYPE=SUBSTITUTE
CM032588.1	299576	.	G	A	100	*	TYPE=SUBSTITUTE
CM032588.1	299578	.	A	G	100	*	TYPE=SUBSTITUTE
CM032588.1	299583	.	G	A	100	*	TYPE=SUBSTITUTE
CM032588.1	299586	.	T	C	100	*	TYPE=SUBSTITUTE
CM032588.1	299600	.	T	G	100	*	TYPE=SUBSTITUTE
CM032588.1	299647	.	G	a	100	*	TYPE=SUBSTITUTE
CM032588.1	299651	.	C	a	100	*	TYPE=SUBSTITUTE
CM032588.1	299652	.	T	C	100	*	TYPE=SUBSTITUTE
CM032588.1	299688	.	A	C	100	*	TYPE=SUBSTITUTE
CM032588.1	299695	.	A	G	100	*	TYPE=SUBSTITUTE
CM032588.1	299752	.	A	G	100	*	TYPE=SUBSTITUTE
CM032588.1	299792	.	C	T	100	*	TYPE=SUBSTITUTE
CM032588.1	299811	.	G	A	100	*	TYPE=SUBSTITUTE
CM032588.1	299829	.	G	T	100	*	TYPE=SUBSTITUTE
CM032588.1	299849	.	C	A	100	*	TYPE=SUBSTITUTE
CM032588.1	299850	.	T	C	100	*	TYPE=SUBSTITUTE
CM032588.1	299885	.	C	T	100	*	TYPE=SUBSTITUTE
CM032588.1	299898	.	C	T	100	*	TYPE=SUBSTITUTE
CM032588.1	299908	.	A	C	100	*	TYPE=SUBSTITUTE
CM032588.1	299912	.	C	T	100	*	TYPE=SUBSTITUTE
CM032588.1	299928	.	G	T	100	*	TYPE=SUBSTITUTE
CM032588.1	299931	.	C	G	100	*	TYPE=SUBSTITUTE
CM032588.1	299934	.	A	C	100	*	TYPE=SUBSTITUTE
CM032588.1	299936	.	C	T	100	*	TYPE=SUBSTITUTE
CM032588.1	299939	.	C	T	100	*	TYPE=SUBSTITUTE
CM032588.1	299953	.	A	G	100	*	TYPE=SUBSTITUTE
CM032588.1	299958	.	A	G	100	*	TYPE=SUBSTITUTE
CM032588.1	299968	.	C	G	100	*	TYPE=SUBSTITUTE
CM032588.1	300006	.	G	A	100	*	TYPE=SUBSTITUTE
CM032588.1	300009	.	A	G	100	*	TYPE=SUBSTITUTE
CM032588.1	300016	.	A	G	100	*	TYPE=SUBSTITUTE
CM032588.1	300029	.	C	T	100	*	TYPE=SUBSTITUTE
CM032588.1	300050	.	A	G	100	*	TYPE=SUBSTITUTE
CM032588.1	300056	.	A	G	100	*	TYPE=SUBSTITUTE
CM032588.1	300057	.	G	T	100	*	TYPE=SUBSTITUTE
CM032588.1	300068	.	T	C	100	*	TYPE=SUBSTITUTE
CM032588.1	300072	.	A	T	100	*	TYPE=SUBSTITUTE
CM032588.1	300076	.	T	C	100	*	TYPE=SUBSTITUTE
CM032588.1	300077	.	G	A	100	*	TYPE=SUBSTITUTE
CM032588.1	300078	.	G	a	100	*	TYPE=SUBSTITUTE

CM032588.1	300081	.	T	g	100	*	TYPE=SUBSTITUTE
CM032588.1	300084	.	G	c	100	*	TYPE=SUBSTITUTE
CM032588.1	300085	.	C	t	100	*	TYPE=SUBSTITUTE
CM032588.1	300107	.	C	A	100	*	TYPE=SUBSTITUTE
CM032588.1	300114	.	G	GA	100	*	TYPE=INSERT
CM032588.1	300118	.	G	A	100	*	TYPE=SUBSTITUTE
CM032588.1	300119	.	C	T	100	*	TYPE=SUBSTITUTE
CM032588.1	300120	.	C	T	100	*	TYPE=SUBSTITUTE
CM032588.1	300125	.	G	T	100	*	TYPE=SUBSTITUTE
CM032588.1	300128	.	A	ATATG	100	*	TYPE=INSERT
CM032588.1	300140	.	G	A	100	*	TYPE=SUBSTITUTE
CM032588.1	300144	.	G	T	100	*	TYPE=SUBSTITUTE
CM032588.1	300157	.	G	A	100	*	TYPE=SUBSTITUTE
CM032588.1	300174	.	C	T	100	*	TYPE=SUBSTITUTE
CM032588.1	300177	.	T	C	100	*	TYPE=SUBSTITUTE
CM032588.1	300181	.	G	C	100	*	TYPE=SUBSTITUTE
CM032588.1	300184	.	C	T	100	*	TYPE=SUBSTITUTE
CM032588.1	300202	.	G	t	100	*	TYPE=SUBSTITUTE
CM032588.1	300209	.	G	t	100	*	TYPE=SUBSTITUTE
CM032588.1	300210	.	C	t	100	*	TYPE=SUBSTITUTE
CM032588.1	300223	.	G	T	100	*	TYPE=SUBSTITUTE
CM032588.1	300226	.	T	A	100	*	TYPE=SUBSTITUTE
CM032588.1	300227	.	G	A	100	*	TYPE=SUBSTITUTE
CM032588.1	300244	.	T	C	100	*	TYPE=SUBSTITUTE
CM032588.1	300246	.	A	G	100	*	TYPE=SUBSTITUTE
CM032588.1	300256	.	T	G	100	*	TYPE=SUBSTITUTE
CM032588.1	300265	.	A	G	100	*	TYPE=SUBSTITUTE
CM032588.1	300271	.	G	A	100	*	TYPE=SUBSTITUTE
CM032588.1	300277	.	A	AG	100	*	TYPE=INSERT
CM032588.1	300278	.	A	T	100	*	TYPE=SUBSTITUTE
CM032588.1	300286	.	T	G	100	*	TYPE=SUBSTITUTE
CM032588.1	300289	.	C	T	100	*	TYPE=SUBSTITUTE
CM032588.1	300294	.	G	T	100	*	TYPE=SUBSTITUTE
CM032588.1	300300	.	A	C	100	*	TYPE=SUBSTITUTE
CM032588.1	300304	.	G	C	100	*	TYPE=SUBSTITUTE
CM032588.1	300307	.	G	A	100	*	TYPE=SUBSTITUTE
CM032588.1	300308	.	G	A	100	*	TYPE=SUBSTITUTE
CM032588.1	300315	.	A	G	100	*	TYPE=SUBSTITUTE
CM032588.1	300318	.	A	T	100	*	TYPE=SUBSTITUTE
CM032588.1	300330	.	T	C	100	*	TYPE=SUBSTITUTE
CM032588.1	300332	.	G	C	100	*	TYPE=SUBSTITUTE
CM032588.1	300337	.	G	GA	100	*	TYPE=INSERT
CM032588.1	300349	.	A	G	100	*	TYPE=SUBSTITUTE
CM032588.1	300354	.	A	T	100	*	TYPE=SUBSTITUTE
CM032588.1	300356	.	G	A	100	*	TYPE=SUBSTITUTE
CM032588.1	300362	.	G	T	100	*	TYPE=SUBSTITUTE
CM032588.1	300372	.	C	A	100	*	TYPE=SUBSTITUTE
CM032588.1	300373	.	G	A	100	*	TYPE=SUBSTITUTE
CM032588.1	300374	.	G	C	100	*	TYPE=SUBSTITUTE
CM032588.1	300375	.	T	G	100	*	TYPE=SUBSTITUTE
CM032588.1	300377	.	A	T	100	*	TYPE=SUBSTITUTE
CM032588.1	300378	.	T	G	100	*	TYPE=SUBSTITUTE
CM032588.1	300379	.	G	C	100	*	TYPE=SUBSTITUTE
CM032588.1	300382	.	A	G	100	*	TYPE=SUBSTITUTE
CM032588.1	300384	.	C	A	100	*	TYPE=SUBSTITUTE
CM032588.1	300386	.	C	T	100	*	TYPE=SUBSTITUTE
CM032588.1	300406	.	A	G	100	*	TYPE=SUBSTITUTE
CM032588.1	300426	.	G	A	100	*	TYPE=SUBSTITUTE
CM032588.1	300429	.	A	T	100	*	TYPE=SUBSTITUTE
CM032588.1	300430	.	A	T	100	*	TYPE=SUBSTITUTE
CM032588.1	300436	.	A	G	100	*	TYPE=SUBSTITUTE
CM032588.1	300448	.	G	A	100	*	TYPE=SUBSTITUTE
CM032588.1	300505	.	A	G	100	*	TYPE=SUBSTITUTE
CM032588.1	300523	.	G	A	100	*	TYPE=SUBSTITUTE

CM032588.1	300547	.	A	G	100	*	TYPE=SUBSTITUTE
CM032588.1	300559	.	A	G	100	*	TYPE=SUBSTITUTE
CM032588.1	300571	.	A	G	100	*	TYPE=SUBSTITUTE
CM032588.1	300574	.	C	T	100	*	TYPE=SUBSTITUTE
CM032588.1	300589	.	G	A	100	*	TYPE=SUBSTITUTE
CM032588.1	300613	.	G	A	100	*	TYPE=SUBSTITUTE
CM032588.1	300631	.	T	C	100	*	TYPE=SUBSTITUTE
CM032588.1	300652	.	T	C	100	*	TYPE=SUBSTITUTE
CM032588.1	300700	.	C	T	100	*	TYPE=SUBSTITUTE
CM032588.1	300715	.	A	C	100	*	TYPE=SUBSTITUTE
CM032588.1	300763	.	A	G	100	*	TYPE=SUBSTITUTE
CM032588.1	300802	.	C	T	100	*	TYPE=SUBSTITUTE
CM032588.1	300820	.	C	G	100	*	TYPE=SUBSTITUTE
CM032588.1	300844	.	A	C	100	*	TYPE=SUBSTITUTE
CM032588.1	300853	.	G	A	100	*	TYPE=SUBSTITUTE
CM032588.1	300868	.	C	G	100	*	TYPE=SUBSTITUTE
CM032588.1	300877	.	T	C	100	*	TYPE=SUBSTITUTE
CM032588.1	300880	.	A	C	100	*	TYPE=SUBSTITUTE
CM032588.1	300905	.	C	T	100	*	TYPE=SUBSTITUTE
CM032588.1	300928	.	A	G	100	*	TYPE=SUBSTITUTE
CM032588.1	300943	.	A	G	100	*	TYPE=SUBSTITUTE
CM032588.1	300997	.	A	G	100	*	TYPE=SUBSTITUTE
CM032588.1	301000	.	A	G	100	*	TYPE=SUBSTITUTE
CM032588.1	301003	.	G	A	100	*	TYPE=SUBSTITUTE
CM032588.1	301010	.	CG	C	100	*	TYPE=DELETE
CM032588.1	301018	.	G	A	100	*	TYPE=SUBSTITUTE
CM032588.1	301069	.	G	C	100	*	TYPE=SUBSTITUTE
CM032588.1	301078	.	G	A	100	*	TYPE=SUBSTITUTE
CM032588.1	301087	.	C	T	100	*	TYPE=SUBSTITUTE
CM032588.1	301089	.	A	G	100	*	TYPE=SUBSTITUTE
CM032588.1	301099	.	G	C	100	*	TYPE=SUBSTITUTE
CM032588.1	301110	.	TG	T	100	*	TYPE=DELETE
CM032588.1	301117	.	A	T	100	*	TYPE=SUBSTITUTE
CM032588.1	301123	.	G	T	100	*	TYPE=SUBSTITUTE
CM032588.1	301129	.	T	G	100	*	TYPE=SUBSTITUTE
CM032588.1	301134	.	G	A	100	*	TYPE=SUBSTITUTE
CM032588.1	301141	.	A	G	100	*	TYPE=SUBSTITUTE
CM032588.1	301142	.	A	T	100	*	TYPE=SUBSTITUTE
CM032588.1	301143	.	A	C	100	*	TYPE=SUBSTITUTE
CM032588.1	301145	.	G	A	100	*	TYPE=SUBSTITUTE
CM032588.1	301152	.	C	T	100	*	TYPE=SUBSTITUTE
CM032588.1	301172	.	T	C	100	*	TYPE=SUBSTITUTE
CM032588.1	301184	.	T	C	100	*	TYPE=SUBSTITUTE
CM032588.1	301185	.	TAC	T	100	*	TYPE=DELETE
CM032588.1	301226	.	T	C	100	*	TYPE=SUBSTITUTE
CM032588.1	301249	.	T	C	100	*	TYPE=SUBSTITUTE
CM032588.1	301253	.	C	T	100	*	TYPE=SUBSTITUTE
CM032588.1	301271	.	T	C	100	*	TYPE=SUBSTITUTE
CM032588.1	301318	.	A	G	100	*	TYPE=SUBSTITUTE
CM032588.1	301323	.	T	G	100	*	TYPE=SUBSTITUTE
CM032588.1	301331	.	T	C	100	*	TYPE=SUBSTITUTE
CM032588.1	301332	.	G	A	100	*	TYPE=SUBSTITUTE
CM032588.1	301352	.	G	C	100	*	TYPE=SUBSTITUTE
CM032588.1	301355	.	C	A	100	*	TYPE=SUBSTITUTE
CM032588.1	301360	.	G	A	100	*	TYPE=SUBSTITUTE
CM032588.1	301369	.	C	T	100	*	TYPE=SUBSTITUTE
CM032588.1	301370	.	A	G	100	*	TYPE=SUBSTITUTE
CM032588.1	301371	.	G	A	100	*	TYPE=SUBSTITUTE
CM032588.1	301432	.	T	A	100	*	TYPE=SUBSTITUTE
CM032588.1	301437	.	C	G	100	*	TYPE=SUBSTITUTE
CM032588.1	301472	.	G	T	100	*	TYPE=SUBSTITUTE
CM032588.1	301494	.	T	C	100	*	TYPE=SUBSTITUTE
CM032588.1	301499	.	T	G	100	*	TYPE=SUBSTITUTE
CM032588.1	301501	.	T	A	100	*	TYPE=SUBSTITUTE

CM032588.1	301530	.	C	A	100	*	TYPE=SUBSTITUTE
CM032588.1	301546	.	C	T	100	*	TYPE=SUBSTITUTE
CM032588.1	301551	.	T	C	100	*	TYPE=SUBSTITUTE
CM032588.1	301559	.	A	C	100	*	TYPE=SUBSTITUTE
CM032588.1	301565	.	GC	G	100	*	TYPE=DELETE
CM032588.1	301595	.	A	T	100	*	TYPE=SUBSTITUTE
CM032588.1	301597	.	G	A	100	*	TYPE=SUBSTITUTE
CM032588.1	301640	.	G	A	100	*	TYPE=SUBSTITUTE
CM032588.1	301669	.	G	A	100	*	TYPE=SUBSTITUTE
CM032588.1	301674	.	C	T	100	*	TYPE=SUBSTITUTE
CM032588.1	301675	.	C	T	100	*	TYPE=SUBSTITUTE
CM032588.1	301676	.	A	G	100	*	TYPE=SUBSTITUTE
CM032588.1	301715	.	A	G	100	*	TYPE=SUBSTITUTE
CM032588.1	301724	.	C	G	100	*	TYPE=SUBSTITUTE
CM032588.1	301748	.	C	T	100	*	TYPE=SUBSTITUTE
CM032588.1	301757	.	T	C	100	*	TYPE=SUBSTITUTE
CM032588.1	301788	.	T	C	100	*	TYPE=SUBSTITUTE
CM032588.1	301791	.	T	C	100	*	TYPE=SUBSTITUTE
CM032588.1	301820	.	AA	A	100	*	TYPE=DELETE
CM032588.1	301827	.	C	t	100	*	TYPE=SUBSTITUTE
CM032588.1	301838	.	T	c	100	*	TYPE=SUBSTITUTE
CM032588.1	301853	.	TG	T	100	*	TYPE=DELETE
CM032588.1	301909	.	A	G	100	*	TYPE=SUBSTITUTE
CM032588.1	301914	.	G	T	100	*	TYPE=SUBSTITUTE
CM032588.1	301917	.	A	T	100	*	TYPE=SUBSTITUTE
CM032588.1	301922	.	A	G	100	*	TYPE=SUBSTITUTE
CM032588.1	301924	.	T	G	100	*	TYPE=SUBSTITUTE
CM032588.1	301931	.	A	T	100	*	TYPE=SUBSTITUTE
CM032588.1	301939	.	G	A	100	*	TYPE=SUBSTITUTE
CM032588.1	301951	.	T	A	100	*	TYPE=SUBSTITUTE
CM032588.1	301957	.	T	C	100	*	TYPE=SUBSTITUTE
CM032588.1	301963	.	T	G	100	*	TYPE=SUBSTITUTE
CM032588.1	301972	.	T	TC	100	*	TYPE=INSERT
CM032588.1	301985	.	G	A	100	*	TYPE=SUBSTITUTE
CM032588.1	301988	.	A	C	100	*	TYPE=SUBSTITUTE
CM032588.1	301989	.	T	C	100	*	TYPE=SUBSTITUTE
CM032588.1	302028	.	C	T	100	*	TYPE=SUBSTITUTE
CM032588.1	302030	.	C	A	100	*	TYPE=SUBSTITUTE
CM032588.1	302034	.	T	A	100	*	TYPE=SUBSTITUTE
CM032588.1	302042	.	T	A	100	*	TYPE=SUBSTITUTE
CM032588.1	302044	.	C	T	100	*	TYPE=SUBSTITUTE
CM032588.1	302052	.	A	C	100	*	TYPE=SUBSTITUTE
CM032588.1	302053	.	G	A	100	*	TYPE=SUBSTITUTE
CM032588.1	302056	.	A	C	100	*	TYPE=SUBSTITUTE
CM032588.1	302057	.	T	C	100	*	TYPE=SUBSTITUTE
CM032588.1	302068	.	C	T	100	*	TYPE=SUBSTITUTE
CM032588.1	302069	.	C	T	100	*	TYPE=SUBSTITUTE
CM032588.1	302070	.	G	A	100	*	TYPE=SUBSTITUTE
CM032588.1	302071	.	A	AG	100	*	TYPE=INSERT
CM032588.1	302076	.	T	C	100	*	TYPE=SUBSTITUTE
CM032588.1	302077	.	T	C	100	*	TYPE=SUBSTITUTE
CM032588.1	302081	.	C	A	100	*	TYPE=SUBSTITUTE
CM032588.1	302092	.	A	C	100	*	TYPE=SUBSTITUTE
CM032588.1	302105	.	C	T	100	*	TYPE=SUBSTITUTE
CM032588.1	302126	.	C	T	100	*	TYPE=SUBSTITUTE
CM032588.1	302141	.	G	A	100	*	TYPE=SUBSTITUTE
CM032588.1	302159	.	G	T	100	*	TYPE=SUBSTITUTE
CM032588.1	302183	.	T	C	100	*	TYPE=SUBSTITUTE
CM032588.1	302198	.	G	A	100	*	TYPE=SUBSTITUTE
CM032588.1	302234	.	C	T	100	*	TYPE=SUBSTITUTE
CM032588.1	302276	.	G	A	100	*	TYPE=SUBSTITUTE
CM032588.1	302291	.	A	C	100	*	TYPE=SUBSTITUTE
CM032588.1	302294	.	T	C	100	*	TYPE=SUBSTITUTE
CM032588.1	302309	.	C	T	100	*	TYPE=SUBSTITUTE

CM032588.1	302327	.	C	T	100	*	TYPE=SUBSTITUTE
CM032588.1	302330	.	C	T	100	*	TYPE=SUBSTITUTE
CM032588.1	302331	.	T	C	100	*	TYPE=SUBSTITUTE
CM032588.1	302345	.	G	A	100	*	TYPE=SUBSTITUTE
CM032588.1	302363	.	G	A	100	*	TYPE=SUBSTITUTE
CM032588.1	302366	.	T	C	100	*	TYPE=SUBSTITUTE
CM032588.1	302384	.	A	T	100	*	TYPE=SUBSTITUTE
CM032588.1	302408	.	A	G	100	*	TYPE=SUBSTITUTE
CM032588.1	302477	.	T	C	100	*	TYPE=SUBSTITUTE
CM032588.1	302480	.	G	A	100	*	TYPE=SUBSTITUTE
CM032588.1	302486	.	C	T	100	*	TYPE=SUBSTITUTE
CM032588.1	302489	.	C	T	100	*	TYPE=SUBSTITUTE
CM032588.1	302516	.	T	C	100	*	TYPE=SUBSTITUTE
CM032588.1	302531	.	T	C	100	*	TYPE=SUBSTITUTE
CM032588.1	302558	.	A	G	100	*	TYPE=SUBSTITUTE
CM032588.1	302576	.	T	C	100	*	TYPE=SUBSTITUTE
CM032588.1	302579	.	T	C	100	*	TYPE=SUBSTITUTE
CM032588.1	302594	.	C	G	100	*	TYPE=SUBSTITUTE
CM032588.1	302612	.	G	A	100	*	TYPE=SUBSTITUTE
CM032588.1	302615	.	A	G	100	*	TYPE=SUBSTITUTE
CM032588.1	302625	.	T	C	100	*	TYPE=SUBSTITUTE
CM032588.1	302681	.	A	G	100	*	TYPE=SUBSTITUTE
CM032588.1	302708	.	A	G	100	*	TYPE=SUBSTITUTE
CM032588.1	302714	.	G	t	100	*	TYPE=SUBSTITUTE
CM032588.1	302720	.	T	g	100	*	TYPE=SUBSTITUTE
CM032588.1	302729	.	T	g	100	*	TYPE=SUBSTITUTE
CM032588.1	302744	.	T	C	100	*	TYPE=SUBSTITUTE
CM032588.1	302746	.	A	C	100	*	TYPE=SUBSTITUTE
CM032588.1	302751	.	G	A	100	*	TYPE=SUBSTITUTE
CM032588.1	302753	.	T	C	100	*	TYPE=SUBSTITUTE
CM032588.1	302756	.	G	T	100	*	TYPE=SUBSTITUTE
CM032588.1	302760	.	T	A	100	*	TYPE=SUBSTITUTE
CM032588.1	302761	.	C	G	100	*	TYPE=SUBSTITUTE
CM032588.1	302762	.	G	C	100	*	TYPE=SUBSTITUTE
CM032588.1	302774	.	C	T	100	*	TYPE=SUBSTITUTE
CM032588.1	302828	.	C	A	100	*	TYPE=SUBSTITUTE
CM032588.1	302876	.	T	C	100	*	TYPE=SUBSTITUTE
CM032588.1	302879	.	T	C	100	*	TYPE=SUBSTITUTE
CM032588.1	302882	.	A	G	100	*	TYPE=SUBSTITUTE
CM032588.1	302888	.	G	A	100	*	TYPE=SUBSTITUTE
CM032588.1	302904	.	G	C	100	*	TYPE=SUBSTITUTE
CM032588.1	302927	.	T	C	100	*	TYPE=SUBSTITUTE
CM032588.1	302948	.	T	C	100	*	TYPE=SUBSTITUTE
CM032588.1	302978	.	T	C	100	*	TYPE=SUBSTITUTE
CM032588.1	303029	.	C	T	100	*	TYPE=SUBSTITUTE
CM032588.1	303038	.	A	G	100	*	TYPE=SUBSTITUTE
CM032588.1	303081	.	G	A	100	*	TYPE=SUBSTITUTE
CM032588.1	303095	.	T	C	100	*	TYPE=SUBSTITUTE
CM032588.1	303096	.	G	A	100	*	TYPE=SUBSTITUTE
CM032588.1	303101	.	C	G	100	*	TYPE=SUBSTITUTE
CM032588.1	303120	.	A	G	100	*	TYPE=SUBSTITUTE
CM032588.1	303197	.	A	G	100	*	TYPE=SUBSTITUTE
CM032588.1	303221	.	C	G	100	*	TYPE=SUBSTITUTE
CM032588.1	303225	.	C	T	100	*	TYPE=SUBSTITUTE
CM032588.1	303260	.	C	A	100	*	TYPE=SUBSTITUTE
CM032588.1	303290	.	C	T	100	*	TYPE=SUBSTITUTE
CM032588.1	303368	.	C	T	100	*	TYPE=SUBSTITUTE
CM032588.1	303377	.	T	C	100	*	TYPE=SUBSTITUTE
CM032588.1	303389	.	A	G	100	*	TYPE=SUBSTITUTE
CM032588.1	303398	.	A	G	100	*	TYPE=SUBSTITUTE
CM032588.1	303401	.	G	A	100	*	TYPE=SUBSTITUTE
CM032588.1	303425	.	A	G	100	*	TYPE=SUBSTITUTE
CM032588.1	303437	.	T	C	100	*	TYPE=SUBSTITUTE
CM032588.1	303440	.	T	C	100	*	TYPE=SUBSTITUTE

CM032588.1	303473	.	T	C	100	*	TYPE=SUBSTITUTE
CM032588.1	303488	.	T	G	100	*	TYPE=SUBSTITUTE
CM032588.1	303512	.	T	A	100	*	TYPE=SUBSTITUTE
CM032588.1	303530	.	T	C	100	*	TYPE=SUBSTITUTE
CM032588.1	303548	.	G	A	100	*	TYPE=SUBSTITUTE
CM032588.1	303560	.	G	A	100	*	TYPE=SUBSTITUTE
CM032588.1	303578	.	C	T	100	*	TYPE=SUBSTITUTE
CM032588.1	303632	.	A	G	100	*	TYPE=SUBSTITUTE
CM032588.1	303638	.	G	T	100	*	TYPE=SUBSTITUTE
CM032588.1	303653	.	C	G	100	*	TYPE=SUBSTITUTE
CM032588.1	303667	.	A	C	100	*	TYPE=SUBSTITUTE
CM032588.1	303683	.	C	A	100	*	TYPE=SUBSTITUTE
CM032588.1	303698	.	T	A	100	*	TYPE=SUBSTITUTE
CM032588.1	303731	.	A	G	100	*	TYPE=SUBSTITUTE
CM032588.1	303737	.	T	C	100	*	TYPE=SUBSTITUTE
CM032588.1	303743	.	C	T	100	*	TYPE=SUBSTITUTE
CM032588.1	303787	.	A	C	100	*	TYPE=SUBSTITUTE
CM032588.1	303789	.	A	C	100	*	TYPE=SUBSTITUTE
CM032588.1	303792	.	A	G	100	*	TYPE=SUBSTITUTE
CM032588.1	303816	.	G	A	100	*	TYPE=SUBSTITUTE
CM032588.1	303819	.	T	A	100	*	TYPE=SUBSTITUTE
CM032588.1	303825	.	G	A	100	*	TYPE=SUBSTITUTE
CM032588.1	303875	.	C	A	100	*	TYPE=SUBSTITUTE
CM032588.1	303876	.	G	GT	100	*	TYPE=INSERT
CM032588.1	303879	.	A	C	100	*	TYPE=SUBSTITUTE
CM032588.1	303893	.	G	A	100	*	TYPE=SUBSTITUTE
CM032588.1	303894	.	G	T	100	*	TYPE=SUBSTITUTE
CM032588.1	303896	.	C	A	100	*	TYPE=SUBSTITUTE
CM032588.1	303919	.	G	A	100	*	TYPE=SUBSTITUTE
CM032588.1	303938	.	A	G	100	*	TYPE=SUBSTITUTE
CM032588.1	303940	.	T	C	100	*	TYPE=SUBSTITUTE
CM032588.1	303999	.	G	A	100	*	TYPE=SUBSTITUTE
CM032588.1	304008	.	A	G	100	*	TYPE=SUBSTITUTE
CM032588.1	304031	.	C	CT	100	*	TYPE=INSERT
CM032588.1	304033	.	G	T	100	*	TYPE=SUBSTITUTE
CM032588.1	304034	.	A	G	100	*	TYPE=SUBSTITUTE
CM032588.1	304043	.	G	A	100	*	TYPE=SUBSTITUTE
CM032588.1	304047	.	TTATGATGATAGGTAAATTAACCTTAATACGACCCTA	T	100	*	TYPE=DELETE
CM032588.1	304096	.	T	C	100	*	TYPE=SUBSTITUTE
CM032588.1	304166	.	C	T	100	*	TYPE=SUBSTITUTE
CM032588.1	304167	.	A	T	100	*	TYPE=SUBSTITUTE
CM032588.1	304194	.	A	AC	100	*	TYPE=INSERT
CM032588.1	304195	.	G	A	100	*	TYPE=SUBSTITUTE
CM032588.1	304202	.	A	T	100	*	TYPE=SUBSTITUTE
CM032588.1	304203	.	G	C	100	*	TYPE=SUBSTITUTE
CM032588.1	304208	.	AT	A	100	*	TYPE=DELETE
CM032588.1	304214	.	G	A	100	*	TYPE=SUBSTITUTE
CM032588.1	304219	.	C	T	100	*	TYPE=SUBSTITUTE
CM032588.1	304221	.	T	C	100	*	TYPE=SUBSTITUTE
CM032588.1	304225	.	C	T	100	*	TYPE=SUBSTITUTE
CM032588.1	304234	.	T	C	100	*	TYPE=SUBSTITUTE
CM032588.1	304243	.	T	A	100	*	TYPE=SUBSTITUTE
CM032588.1	304263	.	G	A	100	*	TYPE=SUBSTITUTE
CM032588.1	304265	.	T	C	100	*	TYPE=SUBSTITUTE
CM032588.1	304301	.	G	A	100	*	TYPE=SUBSTITUTE
CM032588.1	304326	.	G	A	100	*	TYPE=SUBSTITUTE
CM032588.1	304333	.	C	T	100	*	TYPE=SUBSTITUTE
CM032588.1	304357	.	C	T	100	*	TYPE=SUBSTITUTE
CM032588.1	304358	.	A	G	100	*	TYPE=SUBSTITUTE
CM032588.1	304361	.	G	C	100	*	TYPE=SUBSTITUTE
CM032588.1	304366	.	G	C	100	*	TYPE=SUBSTITUTE
CM032588.1	304370	.	G	T	100	*	TYPE=SUBSTITUTE
CM032588.1	304378	.	T	A	100	*	TYPE=SUBSTITUTE

CM032588.1	304388	.	A	G	100	*	TYPE=SUBSTITUTE
CM032588.1	304400	.	C	T	100	*	TYPE=SUBSTITUTE
CM032588.1	304408	.	T	A	100	*	TYPE=SUBSTITUTE
CM032588.1	304410	.	C	A	100	*	TYPE=SUBSTITUTE
CM032588.1	304451	.	C	A	100	*	TYPE=SUBSTITUTE
CM032588.1	304588	.	C	T	100	*	TYPE=SUBSTITUTE
CM032588.1	304594	.	T	C	100	*	TYPE=SUBSTITUTE
CM032588.1	304597	.	A	G	100	*	TYPE=SUBSTITUTE
CM032588.1	304654	.	C	T	100	*	TYPE=SUBSTITUTE
CM032588.1	304686	.	T	TN	100	*	TYPE=INSERT
CM032588.1	304702	.	A	G	100	*	TYPE=SUBSTITUTE
CM032588.1	304711	.	G	A	100	*	TYPE=SUBSTITUTE
CM032588.1	304717	.	A	G	100	*	TYPE=SUBSTITUTE
CM032588.1	304720	.	C	A	100	*	TYPE=SUBSTITUTE
CM032588.1	304733	.	T	C	100	*	TYPE=SUBSTITUTE
CM032588.1	304758	.	T	C	100	*	TYPE=SUBSTITUTE
CM032588.1	304794	.	G	C	100	*	TYPE=SUBSTITUTE
CM032588.1	304815	.	T	A	100	*	TYPE=SUBSTITUTE
CM032588.1	304828	.	G	A	100	*	TYPE=SUBSTITUTE
CM032588.1	304835	.	GT	G	100	*	TYPE=DELETE
CM032588.1	304843	.	C	T	100	*	TYPE=SUBSTITUTE
CM032588.1	304844	.	T	G	100	*	TYPE=SUBSTITUTE
CM032588.1	304851	.	A	G	100	*	TYPE=SUBSTITUTE
CM032588.1	304852	.	C	A	100	*	TYPE=SUBSTITUTE
CM032588.1	304853	.	G	A	100	*	TYPE=SUBSTITUTE
CM032588.1	304854	.	C	A	100	*	TYPE=SUBSTITUTE
CM032588.1	304855	.	G	T	100	*	TYPE=SUBSTITUTE
CM032588.1	304856	.	T	G	100	*	TYPE=SUBSTITUTE
CM032588.1	304857	.	A	C	100	*	TYPE=SUBSTITUTE
CM032588.1	304864	.	T	C	100	*	TYPE=SUBSTITUTE
CM032588.1	304870	.	A	G	100	*	TYPE=SUBSTITUTE
CM032588.1	304873	.	A	G	100	*	TYPE=SUBSTITUTE
CM032588.1	304876	.	A	T	100	*	TYPE=SUBSTITUTE
CM032588.1	304954	.	T	G	100	*	TYPE=SUBSTITUTE
CM032588.1	304980	.	T	G	100	*	TYPE=SUBSTITUTE
CM032588.1	304982	.	G	C	100	*	TYPE=SUBSTITUTE
CM032588.1	304984	.	T	C	100	*	TYPE=SUBSTITUTE
CM032588.1	304992	.	A	T	100	*	TYPE=SUBSTITUTE
CM032588.1	304996	.	ATGTTTGGCG	A	100	*	TYPE=DELETE
CM032588.1	305007	.	G	A	100	*	TYPE=SUBSTITUTE
CM032588.1	305023	.	A	G	100	*	TYPE=SUBSTITUTE
CM032588.1	305026	.	T	C	100	*	TYPE=SUBSTITUTE
CM032588.1	305027	.	C	A	100	*	TYPE=SUBSTITUTE
CM032588.1	305028	.	A	T	100	*	TYPE=SUBSTITUTE
CM032588.1	305030	.	C	T	100	*	TYPE=SUBSTITUTE
CM032588.1	305083	.	T	C	100	*	TYPE=SUBSTITUTE
CM032588.1	305098	.	A	G	100	*	TYPE=SUBSTITUTE
CM032588.1	305104	.	T	C	100	*	TYPE=SUBSTITUTE
CM032588.1	305131	.	G	A	100	*	TYPE=SUBSTITUTE
CM032588.1	305137	.	T	A	100	*	TYPE=SUBSTITUTE
CM032588.1	305154	.	TTA	T	100	*	TYPE=DELETE
CM032588.1	305159	.	G	A	100	*	TYPE=SUBSTITUTE
CM032588.1	305178	.	A	C	100	*	TYPE=SUBSTITUTE
CM032588.1	305179	.	G	C	100	*	TYPE=SUBSTITUTE
CM032588.1	305180	.	C	T	100	*	TYPE=SUBSTITUTE
CM032588.1	305182	.	G	A	100	*	TYPE=SUBSTITUTE
CM032588.1	305186	.	G	A	100	*	TYPE=SUBSTITUTE
CM032588.1	305191	.	C	T	100	*	TYPE=SUBSTITUTE
CM032588.1	305194	.	G	A	100	*	TYPE=SUBSTITUTE
CM032588.1	305195	.	G	A	100	*	TYPE=SUBSTITUTE
CM032588.1	305198	.	C	A	100	*	TYPE=SUBSTITUTE
CM032588.1	305199	.	G	T	100	*	TYPE=SUBSTITUTE
CM032588.1	305214	.	G	A	100	*	TYPE=SUBSTITUTE
CM032588.1	305226	.	A	G	100	*	TYPE=SUBSTITUTE

CM032588.1	305229	.	A	G	100	*	TYPE=SUBSTITUTE
CM032588.1	305244	.	A	T	100	*	TYPE=SUBSTITUTE
CM032588.1	305262	.	G	A	100	*	TYPE=SUBSTITUTE
CM032588.1	305268	.	T	C	100	*	TYPE=SUBSTITUTE
CM032588.1	305277	.	G	A	100	*	TYPE=SUBSTITUTE
CM032588.1	305286	.	A	G	100	*	TYPE=SUBSTITUTE
CM032588.1	305298	.	C	A	100	*	TYPE=SUBSTITUTE
CM032588.1	305364	.	G	C	100	*	TYPE=SUBSTITUTE
CM032588.1	305382	.	A	G	100	*	TYPE=SUBSTITUTE
CM032588.1	305396	.	A	G	100	*	TYPE=SUBSTITUTE
CM032588.1	305397	.	C	T	100	*	TYPE=SUBSTITUTE
CM032588.1	305408	.	G	A	100	*	TYPE=SUBSTITUTE
CM032588.1	305439	.	A	G	100	*	TYPE=SUBSTITUTE
CM032588.1	305466	.	T	G	100	*	TYPE=SUBSTITUTE
CM032588.1	305472	.	G	A	100	*	TYPE=SUBSTITUTE
CM032588.1	305481	.	G	A	100	*	TYPE=SUBSTITUTE
CM032588.1	305490	.	G	A	100	*	TYPE=SUBSTITUTE
CM032588.1	305496	.	C	T	100	*	TYPE=SUBSTITUTE
CM032588.1	305499	.	G	A	100	*	TYPE=SUBSTITUTE
CM032588.1	305508	.	A	G	100	*	TYPE=SUBSTITUTE
CM032588.1	305514	.	T	C	100	*	TYPE=SUBSTITUTE
CM032588.1	305520	.	C	A	100	*	TYPE=SUBSTITUTE
CM032588.1	305522	.	A	G	100	*	TYPE=SUBSTITUTE
CM032588.1	305523	.	C	T	100	*	TYPE=SUBSTITUTE
CM032588.1	305526	.	A	G	100	*	TYPE=SUBSTITUTE
CM032588.1	305532	.	T	C	100	*	TYPE=SUBSTITUTE
CM032588.1	305559	.	C	T	100	*	TYPE=SUBSTITUTE
CM032588.1	305581	.	T	C	100	*	TYPE=SUBSTITUTE
CM032588.1	305803	.	A	C	100	*	TYPE=SUBSTITUTE
CM032588.1	305858	.	C	T	100	*	TYPE=SUBSTITUTE
CM032588.1	305864	.	T	C	100	*	TYPE=SUBSTITUTE
CM032588.1	305933	.	T	g	100	*	TYPE=SUBSTITUTE
CM032588.1	305948	.	A	t	100	*	TYPE=SUBSTITUTE
CM032588.1	305949	.	G	c	100	*	TYPE=SUBSTITUTE
CM032588.1	305950	.	A	T	100	*	TYPE=SUBSTITUTE
CM032588.1	305957	.	A	T	100	*	TYPE=SUBSTITUTE
CM032588.1	305984	.	G	A	100	*	TYPE=SUBSTITUTE
CM032588.1	306017	.	A	G	100	*	TYPE=SUBSTITUTE
CM032588.1	306022	.	C	a	100	*	TYPE=SUBSTITUTE
CM032588.1	306036	.	A	C	100	*	TYPE=SUBSTITUTE
CM032588.1	306056	.	G	GA	100	*	TYPE=INSERT
CM032588.1	306060	.	C	T	100	*	TYPE=SUBSTITUTE
CM032588.1	306068	.	G	C	100	*	TYPE=SUBSTITUTE
CM032588.1	306072	.	C	G	100	*	TYPE=SUBSTITUTE
CM032588.1	306092	.	G	a	100	*	TYPE=SUBSTITUTE
CM032588.1	306099	.	G	a	100	*	TYPE=SUBSTITUTE
CM032588.1	306100	.	a	attag	100	*	TYPE=INSERT
CM032588.1	306112	.	C	CAATGA	100	*	TYPE=INSERT
CM032588.1	306114	.	G	A	100	*	TYPE=SUBSTITUTE
CM032588.1	306116	.	G	T	100	*	TYPE=SUBSTITUTE
CM032588.1	306119	.	T	A	100	*	TYPE=SUBSTITUTE
CM032588.1	306132	.	G	T	100	*	TYPE=SUBSTITUTE
CM032588.1	306135	.	G	A	100	*	TYPE=SUBSTITUTE
CM032588.1	306147	.	G	A	100	*	TYPE=SUBSTITUTE
CM032588.1	306148	.	C	T	100	*	TYPE=SUBSTITUTE
CM032588.1	306156	.	C	CT	100	*	TYPE=INSERT
CM032588.1	306169	.	T	A	100	*	TYPE=SUBSTITUTE
CM032588.1	306176	.	A	G	100	*	TYPE=SUBSTITUTE
CM032588.1	306177	.	C	A	100	*	TYPE=SUBSTITUTE
CM032588.1	306181	.	A	T	100	*	TYPE=SUBSTITUTE
CM032588.1	306182	.	A	G	100	*	TYPE=SUBSTITUTE
CM032588.1	306196	.	T	C	100	*	TYPE=SUBSTITUTE
CM032588.1	306203	.	A	G	100	*	TYPE=SUBSTITUTE
CM032588.1	306227	.	T	C	100	*	TYPE=SUBSTITUTE

CM032588.1	306229	.	T	C	100	*	TYPE=SUBSTITUTE
CM032588.1	306230	.	G	A	100	*	TYPE=SUBSTITUTE
CM032588.1	306266	.	G	C	100	*	TYPE=SUBSTITUTE
CM032588.1	306267	.	C	T	100	*	TYPE=SUBSTITUTE
CM032588.1	306286	.	A	C	100	*	TYPE=SUBSTITUTE
CM032588.1	306287	.	C	T	100	*	TYPE=SUBSTITUTE
CM032588.1	306291	.	C	T	100	*	TYPE=SUBSTITUTE
CM032588.1	306297	.	T	C	100	*	TYPE=SUBSTITUTE
CM032588.1	306311	.	A	T	100	*	TYPE=SUBSTITUTE
CM032588.1	306323	.	T	C	100	*	TYPE=SUBSTITUTE
CM032588.1	306326	.	T	C	100	*	TYPE=SUBSTITUTE
CM032588.1	306334	.	T	C	100	*	TYPE=SUBSTITUTE
CM032588.1	306336	.	T	A	100	*	TYPE=SUBSTITUTE
CM032588.1	306347	.	C	A	100	*	TYPE=SUBSTITUTE
CM032588.1	306353	.	C	T	100	*	TYPE=SUBSTITUTE
CM032588.1	306356	.	T	c	100	*	TYPE=SUBSTITUTE
CM032588.1	306359	.	C	g	100	*	TYPE=SUBSTITUTE
CM032588.1	306361	.	C	a	100	*	TYPE=SUBSTITUTE
CM032588.1	306364	.	G	a	100	*	TYPE=SUBSTITUTE
CM032588.1	306370	.	C	t	100	*	TYPE=SUBSTITUTE
CM032588.1	306373	.	T	c	100	*	TYPE=SUBSTITUTE
CM032588.1	306379	.	G	A	100	*	TYPE=SUBSTITUTE
CM032588.1	306406	.	T	C	100	*	TYPE=SUBSTITUTE
CM032588.1	306409	.	C	G	100	*	TYPE=SUBSTITUTE
CM032588.1	306412	.	G	A	100	*	TYPE=SUBSTITUTE
CM032588.1	306413	.	T	A	100	*	TYPE=SUBSTITUTE
CM032588.1	306419	.	C	A	100	*	TYPE=SUBSTITUTE
CM032588.1	306421	.	G	T	100	*	TYPE=SUBSTITUTE
CM032588.1	306427	.	C	T	100	*	TYPE=SUBSTITUTE
CM032588.1	306432	.	G	T	100	*	TYPE=SUBSTITUTE
CM032588.1	306440	.	C	T	100	*	TYPE=SUBSTITUTE
CM032588.1	306454	.	T	C	100	*	TYPE=SUBSTITUTE
CM032588.1	306478	.	A	T	100	*	TYPE=SUBSTITUTE
CM032588.1	306510	.	A	C	100	*	TYPE=SUBSTITUTE
CM032588.1	306576	.	C	T	100	*	TYPE=SUBSTITUTE
CM032588.1	306588	.	A	T	100	*	TYPE=SUBSTITUTE
CM032588.1	306597	.	A	G	100	*	TYPE=SUBSTITUTE
CM032588.1	306618	.	G	A	100	*	TYPE=SUBSTITUTE
CM032588.1	306620	.	T	C	100	*	TYPE=SUBSTITUTE
CM032588.1	306621	.	G	A	100	*	TYPE=SUBSTITUTE
CM032588.1	306633	.	A	G	100	*	TYPE=SUBSTITUTE
CM032588.1	306696	.	C	G	100	*	TYPE=SUBSTITUTE
CM032588.1	306734	.	G	a	100	*	TYPE=SUBSTITUTE
CM032588.1	306735	.	C	T	100	*	TYPE=SUBSTITUTE
CM032588.1	306736	.	A	G	100	*	TYPE=SUBSTITUTE
CM032588.1	306753	.	T	C	100	*	TYPE=SUBSTITUTE
CM032588.1	306768	.	G	T	100	*	TYPE=SUBSTITUTE
CM032588.1	306804	.	G	t	100	*	TYPE=SUBSTITUTE
CM032588.1	306853	.	T	C	100	*	TYPE=SUBSTITUTE
CM032588.1	306867	.	T	C	100	*	TYPE=SUBSTITUTE
CM032588.1	306873	.	T	C	100	*	TYPE=SUBSTITUTE
CM032588.1	306906	.	A	G	100	*	TYPE=SUBSTITUTE
CM032588.1	306927	.	A	G	100	*	TYPE=SUBSTITUTE
CM032588.1	306975	.	T	A	100	*	TYPE=SUBSTITUTE
CM032588.1	306993	.	T	C	100	*	TYPE=SUBSTITUTE
CM032588.1	307011	.	C	T	100	*	TYPE=SUBSTITUTE
CM032588.1	307103	.	T	G	100	*	TYPE=SUBSTITUTE
CM032588.1	307107	.	T	A	100	*	TYPE=SUBSTITUTE
CM032588.1	307114	.	G	A	100	*	TYPE=SUBSTITUTE
CM032588.1	307119	.	C	T	100	*	TYPE=SUBSTITUTE
CM032588.1	307225	.	TTC	T	100	*	TYPE=DELETE
CM032588.1	307235	.	C	G	100	*	TYPE=SUBSTITUTE
CM032588.1	307241	.	C	T	100	*	TYPE=SUBSTITUTE
CM032588.1	307251	.	T	C	100	*	TYPE=SUBSTITUTE

CM032588.1	307252	.	C	T	100	*	TYPE=SUBSTITUTE
CM032588.1	307256	.	C	T	100	*	TYPE=SUBSTITUTE
CM032588.1	307259	.	T	C	100	*	TYPE=SUBSTITUTE
CM032588.1	307267	.	G	A	100	*	TYPE=SUBSTITUTE
CM032588.1	307275	.	C	T	100	*	TYPE=SUBSTITUTE
CM032588.1	307284	.	G	A	100	*	TYPE=SUBSTITUTE
CM032588.1	307292	.	T	C	100	*	TYPE=SUBSTITUTE
CM032588.1	307295	.	G	A	100	*	TYPE=SUBSTITUTE
CM032588.1	307310	.	A	G	100	*	TYPE=SUBSTITUTE
CM032588.1	307410	.	C	G	100	*	TYPE=SUBSTITUTE
CM032588.1	307418	.	C	T	100	*	TYPE=SUBSTITUTE
CM032588.1	307437	.	A	C	100	*	TYPE=SUBSTITUTE
CM032588.1	307442	.	T	C	100	*	TYPE=SUBSTITUTE
CM032588.1	307457	.	G	A	100	*	TYPE=SUBSTITUTE
CM032588.1	307619	.	C	T	100	*	TYPE=SUBSTITUTE
CM032588.1	307658	.	C	G	100	*	TYPE=SUBSTITUTE
CM032588.1	307673	.	G	C	100	*	TYPE=SUBSTITUTE
CM032588.1	307679	.	A	G	100	*	TYPE=SUBSTITUTE
CM032588.1	307712	.	T	A	100	*	TYPE=SUBSTITUTE
CM032588.1	307727	.	C	A	100	*	TYPE=SUBSTITUTE
CM032588.1	307799	.	T	C	100	*	TYPE=SUBSTITUTE
CM032588.1	307814	.	C	T	100	*	TYPE=SUBSTITUTE
CM032588.1	307832	.	A	G	100	*	TYPE=SUBSTITUTE
CM032588.1	307858	.	C	T	100	*	TYPE=SUBSTITUTE
CM032588.1	307898	.	C	A	100	*	TYPE=SUBSTITUTE
CM032588.1	307903	.	C	T	100	*	TYPE=SUBSTITUTE
CM032588.1	307913	.	C	G	100	*	TYPE=SUBSTITUTE
CM032588.1	307916	.	A	G	100	*	TYPE=SUBSTITUTE
CM032588.1	307953	.	T	A	100	*	TYPE=SUBSTITUTE
CM032588.1	307990	.	C	T	100	*	TYPE=SUBSTITUTE
CM032588.1	308070	.	A	G	100	*	TYPE=SUBSTITUTE
CM032588.1	308080	.	A	G	100	*	TYPE=SUBSTITUTE
CM032588.1	308089	.	T	C	100	*	TYPE=SUBSTITUTE
CM032588.1	308091	.	C	T	100	*	TYPE=SUBSTITUTE
CM032588.1	308155	.	T	C	100	*	TYPE=SUBSTITUTE
CM032588.1	308160	.	G	A	100	*	TYPE=SUBSTITUTE
CM032588.1	308165	.	C	T	100	*	TYPE=SUBSTITUTE
CM032588.1	308168	.	C	G	100	*	TYPE=SUBSTITUTE
CM032588.1	308173	.	A	G	100	*	TYPE=SUBSTITUTE
CM032588.1	308174	.	A	T	100	*	TYPE=SUBSTITUTE
CM032588.1	308181	.	A	G	100	*	TYPE=SUBSTITUTE
CM032588.1	308183	.	C	T	100	*	TYPE=SUBSTITUTE
CM032588.1	308184	.	T	G	100	*	TYPE=SUBSTITUTE
CM032588.1	308191	.	A	G	100	*	TYPE=SUBSTITUTE
CM032588.1	308193	.	T	C	100	*	TYPE=SUBSTITUTE
CM032588.1	308195	.	G	A	100	*	TYPE=SUBSTITUTE
CM032588.1	308202	.	T	A	100	*	TYPE=SUBSTITUTE
CM032588.1	308207	.	C	T	100	*	TYPE=SUBSTITUTE
CM032588.1	308218	.	T	A	100	*	TYPE=SUBSTITUTE
CM032588.1	308228	.	T	C	100	*	TYPE=SUBSTITUTE
CM032588.1	308234	.	G	A	100	*	TYPE=SUBSTITUTE
CM032588.1	308242	.	T	C	100	*	TYPE=SUBSTITUTE
CM032588.1	308243	.	G	A	100	*	TYPE=SUBSTITUTE
CM032588.1	308244	.	C	T	100	*	TYPE=SUBSTITUTE
CM032588.1	308246	.	T	C	100	*	TYPE=SUBSTITUTE
CM032588.1	308253	.	A	T	100	*	TYPE=SUBSTITUTE
CM032588.1	308257	.	T	C	100	*	TYPE=SUBSTITUTE
CM032588.1	308268	.	G	T	100	*	TYPE=SUBSTITUTE
CM032588.1	308282	.	C	T	100	*	TYPE=SUBSTITUTE
CM032588.1	308292	.	T	C	100	*	TYPE=SUBSTITUTE
CM032588.1	308295	.	A	G	100	*	TYPE=SUBSTITUTE
CM032588.1	308298	.	C	T	100	*	TYPE=SUBSTITUTE
CM032588.1	308303	.	A	T	100	*	TYPE=SUBSTITUTE
CM032588.1	308318	.	C	G	100	*	TYPE=SUBSTITUTE

CM032588.1	308353	.	C	G	100	*	TYPE=SUBSTITUTE
CM032588.1	308355	.	C	T	100	*	TYPE=SUBSTITUTE
CM032588.1	308418	.	T	C	100	*	TYPE=SUBSTITUTE
CM032588.1	308420	.	A	G	100	*	TYPE=SUBSTITUTE
CM032588.1	308422	.	A	C	100	*	TYPE=SUBSTITUTE
CM032588.1	308427	.	G	C	100	*	TYPE=SUBSTITUTE
CM032588.1	308430	.	T	C	100	*	TYPE=SUBSTITUTE
CM032588.1	308431	.	A	G	100	*	TYPE=SUBSTITUTE
CM032588.1	308434	.	G	A	100	*	TYPE=SUBSTITUTE
CM032588.1	308438	.	C	T	100	*	TYPE=SUBSTITUTE
CM032588.1	308439	.	A	G	100	*	TYPE=SUBSTITUTE
CM032588.1	308441	.	T	G	100	*	TYPE=SUBSTITUTE
CM032588.1	308442	.	G	A	100	*	TYPE=SUBSTITUTE
CM032588.1	308449	.	T	G	100	*	TYPE=SUBSTITUTE
CM032588.1	308450	.	G	C	100	*	TYPE=SUBSTITUTE
CM032588.1	308451	.	G	C	100	*	TYPE=SUBSTITUTE
CM032588.1	308452	.	G	T	100	*	TYPE=SUBSTITUTE
CM032588.1	308454	.	T	G	100	*	TYPE=SUBSTITUTE
CM032588.1	308455	.	A	T	100	*	TYPE=SUBSTITUTE
CM032588.1	308456	.	C	T	100	*	TYPE=SUBSTITUTE
CM032588.1	308457	.	C	A	100	*	TYPE=SUBSTITUTE
CM032588.1	308459	.	TG	T	100	*	TYPE=DELETE
CM032588.1	308463	.	T	C	100	*	TYPE=SUBSTITUTE
CM032588.1	308465	.	G	A	100	*	TYPE=SUBSTITUTE
CM032588.1	308468	.	C	T	100	*	TYPE=SUBSTITUTE
CM032588.1	308470	.	G	A	100	*	TYPE=SUBSTITUTE
CM032588.1	308476	.	T	C	100	*	TYPE=SUBSTITUTE
CM032588.1	308477	.	G	T	100	*	TYPE=SUBSTITUTE
CM032588.1	308482	.	A	T	100	*	TYPE=SUBSTITUTE
CM032588.1	308484	.	T	C	100	*	TYPE=SUBSTITUTE
CM032588.1	308487	.	A	G	100	*	TYPE=SUBSTITUTE
CM032588.1	308490	.	G	T	100	*	TYPE=SUBSTITUTE
CM032588.1	308491	.	C	CT	100	*	TYPE=INSERT
CM032588.1	308492	.	C	T	100	*	TYPE=SUBSTITUTE
CM032588.1	308494	.	C	T	100	*	TYPE=SUBSTITUTE
CM032588.1	308495	.	C	T	100	*	TYPE=SUBSTITUTE
CM032588.1	308500	.	A	G	100	*	TYPE=SUBSTITUTE
CM032588.1	308501	.	C	A	100	*	TYPE=SUBSTITUTE
CM032588.1	308504	.	T	C	100	*	TYPE=SUBSTITUTE
CM032588.1	308507	.	G	A	100	*	TYPE=SUBSTITUTE
CM032588.1	308508	.	G	A	100	*	TYPE=SUBSTITUTE
CM032588.1	308509	.	A	G	100	*	TYPE=SUBSTITUTE
CM032588.1	308512	.	G	A	100	*	TYPE=SUBSTITUTE
CM032588.1	308516	.	T	C	100	*	TYPE=SUBSTITUTE
CM032588.1	308517	.	G	A	100	*	TYPE=SUBSTITUTE
CM032588.1	308525	.	A	C	100	*	TYPE=SUBSTITUTE
CM032588.1	308541	.	G	A	100	*	TYPE=SUBSTITUTE
CM032588.1	308544	.	AC	A	100	*	TYPE=DELETE
CM032588.1	308546	.	G	A	100	*	TYPE=SUBSTITUTE
CM032588.1	308547	.	G	T	100	*	TYPE=SUBSTITUTE
CM032588.1	308548	.	C	A	100	*	TYPE=SUBSTITUTE
CM032588.1	308550	.	C	G	100	*	TYPE=SUBSTITUTE
CM032588.1	308562	.	A	G	100	*	TYPE=SUBSTITUTE
CM032588.1	308564	.	G	A	100	*	TYPE=SUBSTITUTE
CM032588.1	308569	.	A	ATG	100	*	TYPE=INSERT
CM032588.1	308576	.	G	A	100	*	TYPE=SUBSTITUTE
CM032588.1	308582	.	A	C	100	*	TYPE=SUBSTITUTE
CM032588.1	308586	.	G	T	100	*	TYPE=SUBSTITUTE
CM032588.1	308598	.	A	C	100	*	TYPE=SUBSTITUTE
CM032588.1	308602	.	G	A	100	*	TYPE=SUBSTITUTE
CM032588.1	308625	.	T	C	100	*	TYPE=SUBSTITUTE
CM032588.1	308629	.	A	G	100	*	TYPE=SUBSTITUTE
CM032588.1	308642	.	T	A	100	*	TYPE=SUBSTITUTE
CM032588.1	308645	.	C	T	100	*	TYPE=SUBSTITUTE

CM032588.1	308651	.	A	C	100	*	TYPE=SUBSTITUTE
CM032588.1	308684	.	T	C	100	*	TYPE=SUBSTITUTE
CM032588.1	308690	.	A	G	100	*	TYPE=SUBSTITUTE
CM032588.1	308729	.	C	T	100	*	TYPE=SUBSTITUTE
CM032588.1	308735	.	A	G	100	*	TYPE=SUBSTITUTE
CM032588.1	308747	.	T	C	100	*	TYPE=SUBSTITUTE
CM032588.1	308748	.	C	T	100	*	TYPE=SUBSTITUTE
CM032588.1	308759	.	A	G	100	*	TYPE=SUBSTITUTE
CM032588.1	308792	.	A	G	100	*	TYPE=SUBSTITUTE
CM032588.1	308836	.	G	T	100	*	TYPE=SUBSTITUTE
CM032588.1	308867	.	T	A	100	*	TYPE=SUBSTITUTE
CM032588.1	308873	.	A	G	100	*	TYPE=SUBSTITUTE
CM032588.1	308893	.	C	T	100	*	TYPE=SUBSTITUTE
CM032588.1	308903	.	C	T	100	*	TYPE=SUBSTITUTE
CM032588.1	308912	.	T	C	100	*	TYPE=SUBSTITUTE
CM032588.1	308913	.	C	T	100	*	TYPE=SUBSTITUTE
CM032588.1	308933	.	A	G	100	*	TYPE=SUBSTITUTE
CM032588.1	308934	.	C	T	100	*	TYPE=SUBSTITUTE
CM032588.1	308975	.	C	T	100	*	TYPE=SUBSTITUTE
CM032588.1	308978	.	G	A	100	*	TYPE=SUBSTITUTE
CM032588.1	308981	.	C	T	100	*	TYPE=SUBSTITUTE
CM032588.1	308984	.	T	G	100	*	TYPE=SUBSTITUTE
CM032588.1	308990	.	C	T	100	*	TYPE=SUBSTITUTE
CM032588.1	309047	.	T	C	100	*	TYPE=SUBSTITUTE
CM032588.1	309074	.	A	G	100	*	TYPE=SUBSTITUTE
CM032588.1	309077	.	G	A	100	*	TYPE=SUBSTITUTE
CM032588.1	309110	.	T	C	100	*	TYPE=SUBSTITUTE
CM032588.1	309116	.	C	T	100	*	TYPE=SUBSTITUTE
CM032588.1	309137	.	C	T	100	*	TYPE=SUBSTITUTE
CM032588.1	309143	.	A	G	100	*	TYPE=SUBSTITUTE
CM032588.1	309146	.	T	C	100	*	TYPE=SUBSTITUTE
CM032588.1	309149	.	G	A	100	*	TYPE=SUBSTITUTE
CM032588.1	309155	.	G	T	100	*	TYPE=SUBSTITUTE
CM032588.1	309156	.	G	T	100	*	TYPE=SUBSTITUTE
CM032588.1	309157	.	A	C	100	*	TYPE=SUBSTITUTE
CM032588.1	309172	.	G	A	100	*	TYPE=SUBSTITUTE
CM032588.1	309182	.	A	G	100	*	TYPE=SUBSTITUTE
CM032588.1	309194	.	A	G	100	*	TYPE=SUBSTITUTE
CM032588.1	309197	.	C	T	100	*	TYPE=SUBSTITUTE
CM032588.1	309200	.	T	C	100	*	TYPE=SUBSTITUTE
CM032588.1	309212	.	A	G	100	*	TYPE=SUBSTITUTE
CM032588.1	309215	.	G	C	100	*	TYPE=SUBSTITUTE
CM032588.1	309224	.	A	G	100	*	TYPE=SUBSTITUTE
CM032588.1	309237	.	A	G	100	*	TYPE=SUBSTITUTE
CM032588.1	309241	.	A	T	100	*	TYPE=SUBSTITUTE
CM032588.1	309263	.	G	C	100	*	TYPE=SUBSTITUTE
CM032588.1	309275	.	G	C	100	*	TYPE=SUBSTITUTE
CM032588.1	309278	.	T	C	100	*	TYPE=SUBSTITUTE
CM032588.1	309294	.	G	c	100	*	TYPE=SUBSTITUTE
CM032588.1	309311	.	G	C	100	*	TYPE=SUBSTITUTE
CM032588.1	309314	.	T	C	100	*	TYPE=SUBSTITUTE
CM032588.1	309317	.	G	A	100	*	TYPE=SUBSTITUTE
CM032588.1	309325	.	G	A	100	*	TYPE=SUBSTITUTE
CM032588.1	309338	.	G	A	100	*	TYPE=SUBSTITUTE
CM032588.1	309341	.	G	A	100	*	TYPE=SUBSTITUTE
CM032588.1	309351	.	A	C	100	*	TYPE=SUBSTITUTE
CM032588.1	309356	.	G	A	100	*	TYPE=SUBSTITUTE
CM032588.1	309371	.	A	G	100	*	TYPE=SUBSTITUTE
CM032588.1	309374	.	G	C	100	*	TYPE=SUBSTITUTE
CM032588.1	309395	.	G	A	100	*	TYPE=SUBSTITUTE
CM032588.1	309410	.	C	T	100	*	TYPE=SUBSTITUTE
CM032588.1	309411	.	A	T	100	*	TYPE=SUBSTITUTE
CM032588.1	309413	.	C	A	100	*	TYPE=SUBSTITUTE
CM032588.1	309424	.	T	C	100	*	TYPE=SUBSTITUTE

CM032588.1	309431	.	G	A	100	*	TYPE=SUBSTITUTE
CM032588.1	309437	.	G	C	100	*	TYPE=SUBSTITUTE
CM032588.1	309449	.	A	G	100	*	TYPE=SUBSTITUTE
CM032588.1	309453	.	A	T	100	*	TYPE=SUBSTITUTE
CM032588.1	309456	.	T	C	100	*	TYPE=SUBSTITUTE
CM032588.1	309458	.	T	A	100	*	TYPE=SUBSTITUTE
CM032588.1	309463	.	G	C	100	*	TYPE=SUBSTITUTE
CM032588.1	309464	.	G	A	100	*	TYPE=SUBSTITUTE
CM032588.1	309467	.	T	g	100	*	TYPE=SUBSTITUTE
CM032588.1	309473	.	G	a	100	*	TYPE=SUBSTITUTE
CM032588.1	309476	.	T	g	100	*	TYPE=SUBSTITUTE
CM032588.1	309477	.	T	g	100	*	TYPE=SUBSTITUTE
CM032588.1	309506	.	A	G	100	*	TYPE=SUBSTITUTE
CM032588.1	309525	.	G	C	100	*	TYPE=SUBSTITUTE
CM032588.1	309555	.	C	T	100	*	TYPE=SUBSTITUTE
CM032588.1	309556	.	C	T	100	*	TYPE=SUBSTITUTE
CM032588.1	309566	.	A	G	100	*	TYPE=SUBSTITUTE
CM032588.1	309584	.	G	A	100	*	TYPE=SUBSTITUTE
CM032588.1	309589	.	C	A	100	*	TYPE=SUBSTITUTE
CM032588.1	309596	.	A	G	100	*	TYPE=SUBSTITUTE
CM032588.1	309604	.	C	A	100	*	TYPE=SUBSTITUTE
CM032588.1	309613	.	A	G	100	*	TYPE=SUBSTITUTE
CM032588.1	309622	.	G	T	100	*	TYPE=SUBSTITUTE
CM032588.1	309629	.	C	T	100	*	TYPE=SUBSTITUTE
CM032588.1	309635	.	A	G	100	*	TYPE=SUBSTITUTE
CM032588.1	309638	.	G	A	100	*	TYPE=SUBSTITUTE
CM032588.1	309645	.	A	G	100	*	TYPE=SUBSTITUTE
CM032588.1	309648	.	A	C	100	*	TYPE=SUBSTITUTE
CM032588.1	309652	.	G	T	100	*	TYPE=SUBSTITUTE
CM032588.1	309653	.	G	A	100	*	TYPE=SUBSTITUTE
CM032588.1	309655	.	A	G	100	*	TYPE=SUBSTITUTE
CM032588.1	309656	.	G	C	100	*	TYPE=SUBSTITUTE
CM032588.1	309663	.	G	A	100	*	TYPE=SUBSTITUTE
CM032588.1	309669	.	G	T	100	*	TYPE=SUBSTITUTE
CM032588.1	309681	.	T	G	100	*	TYPE=SUBSTITUTE
CM032588.1	309687	.	C	T	100	*	TYPE=SUBSTITUTE
CM032588.1	309688	.	C	G	100	*	TYPE=SUBSTITUTE
CM032588.1	309689	.	C	T	100	*	TYPE=SUBSTITUTE
CM032588.1	309691	.	A	G	100	*	TYPE=SUBSTITUTE
CM032588.1	309692	.	T	C	100	*	TYPE=SUBSTITUTE
CM032588.1	309697	.	T	G	100	*	TYPE=SUBSTITUTE
CM032588.1	309706	.	T	C	100	*	TYPE=SUBSTITUTE
CM032588.1	309711	.	A	G	100	*	TYPE=SUBSTITUTE
CM032588.1	309739	.	T	C	100	*	TYPE=SUBSTITUTE
CM032588.1	309757	.	ACCTA	A	100	*	TYPE=DELETE
CM032588.1	309771	.	A	G	100	*	TYPE=SUBSTITUTE
CM032588.1	309780	.	GA	G	100	*	TYPE=DELETE
CM032588.1	309783	.	A	G	100	*	TYPE=SUBSTITUTE
CM032588.1	309785	.	T	G	100	*	TYPE=SUBSTITUTE
CM032588.1	309786	.	T	A	100	*	TYPE=SUBSTITUTE
CM032588.1	309787	.	A	C	100	*	TYPE=SUBSTITUTE
CM032588.1	309790	.	C	T	100	*	TYPE=SUBSTITUTE
CM032588.1	309791	.	C	A	100	*	TYPE=SUBSTITUTE
CM032588.1	309793	.	T	A	100	*	TYPE=SUBSTITUTE
CM032588.1	309802	.	A	G	100	*	TYPE=SUBSTITUTE
CM032588.1	309818	.	A	T	100	*	TYPE=SUBSTITUTE
CM032588.1	309819	.	G	T	100	*	TYPE=SUBSTITUTE
CM032588.1	309824	.	C	CT	100	*	TYPE=INSERT
CM032588.1	309845	.	A	C	100	*	TYPE=SUBSTITUTE
CM032588.1	309860	.	G	A	100	*	TYPE=SUBSTITUTE
CM032588.1	309861	.	C	T	100	*	TYPE=SUBSTITUTE
CM032588.1	309871	.	T	C	100	*	TYPE=SUBSTITUTE
CM032588.1	309876	.	G	A	100	*	TYPE=SUBSTITUTE
CM032588.1	309885	.	G	A	100	*	TYPE=SUBSTITUTE

CM032588.1 309889 . C G 100 * TYPE=SUBSTITUTE
CM032588.1 309899 . C T 100 * TYPE=SUBSTITUTE
CM032588.1 309901 . A G 100 * TYPE=SUBSTITUTE
CM032588.1 309913 . T A 100 * TYPE=SUBSTITUTE
CM032588.1 309920 . C CA 100 * TYPE=INSERT
CM032588.1 309934 . C T 100 * TYPE=SUBSTITUTE
CM032588.1 309937 . C G 100 * TYPE=SUBSTITUTE
CM032588.1 309942 . C T 100 * TYPE=SUBSTITUTE
CM032588.1 309946 . C G 100 * TYPE=SUBSTITUTE
CM032588.1 309948 . A G 100 * TYPE=SUBSTITUTE
CM032588.1 309954 . T A 100 * TYPE=SUBSTITUTE
CM032588.1 309960 . T A 100 * TYPE=SUBSTITUTE
CM032588.1 309977 . A G 100 * TYPE=SUBSTITUTE
CM032588.1 309984 . T C 100 * TYPE=SUBSTITUTE
CM032588.1 309995 . G A 100 * TYPE=SUBSTITUTE
CM032588.1 309997 . G A 100 * TYPE=SUBSTITUTE
CM032588.1 309998 . A C 100 * TYPE=SUBSTITUTE
CM032588.1 310000 . G C 100 * TYPE=SUBSTITUTE
CM032588.1 310011 . G

GCTGTATACCTACGCAATGGTCCATATTCACTCCATTTTTAGAACATTCGGTTATATCTGCTATACTATGTCTATACTAT
ATAAGCTATATGCGATTAAGT 100 * TYPE=INSERT
CM032588.1 310013 . T A 100 * TYPE=SUBSTITUTE
CM032588.1 310018 . G A 100 * TYPE=SUBSTITUTE
CM032588.1 310023 . G GCAAGTTTA 100 * TYPE=INSERT
CM032588.1 310026 . G GTA 100 * TYPE=INSERT
CM032588.1 310028 . T TGAAAGA 100 * TYPE=INSERT
CM032588.1 310030 . C T 100 * TYPE=SUBSTITUTE
CM032588.1 310032 . G A 100 * TYPE=SUBSTITUTE
CM032588.1 310035 . T C 100 * TYPE=SUBSTITUTE
CM032588.1 310040 . G C 100 * TYPE=SUBSTITUTE
CM032588.1 310042 . T C 100 * TYPE=SUBSTITUTE
CM032588.1 310046 . C T 100 * TYPE=SUBSTITUTE
CM032588.1 310050 . A T 100 * TYPE=SUBSTITUTE
CM032588.1 310060 . T A 100 * TYPE=SUBSTITUTE
CM032588.1 310061 . T C 100 * TYPE=SUBSTITUTE
CM032588.1 310067 . T C 100 * TYPE=SUBSTITUTE
CM032588.1 310079 . G C 100 * TYPE=SUBSTITUTE
CM032588.1 310090 . AAAT A 100 * TYPE=DELETE
CM032588.1 310095 . A G 100 * TYPE=SUBSTITUTE
CM032588.1 310106 . T C 100 * TYPE=SUBSTITUTE
CM032588.1 310107 . C T 100 * TYPE=SUBSTITUTE
CM032588.1 310108 . G A 100 * TYPE=SUBSTITUTE
CM032588.1 310113 . C A 100 * TYPE=SUBSTITUTE
CM032588.1 310119 . G T 100 * TYPE=SUBSTITUTE
CM032588.1 310120 . T C 100 * TYPE=SUBSTITUTE
CM032588.1 310123 . A G 100 * TYPE=SUBSTITUTE
CM032588.1 310129 . C A 100 * TYPE=SUBSTITUTE
CM032588.1 310132 . G A 100 * TYPE=SUBSTITUTE
CM032588.1 310137 . T C 100 * TYPE=SUBSTITUTE
CM032588.1 310142 . A G 100 * TYPE=SUBSTITUTE
CM032588.1 310143 . G A 100 * TYPE=SUBSTITUTE
CM032588.1 310153 . TGGCAAAGTACTTGAC T 100 * TYPE=DELETE
CM032588.1 310169 . G T 100 * TYPE=SUBSTITUTE
CM032588.1 310173 . G A 100 * TYPE=SUBSTITUTE
CM032588.1 310174 . C A 100 * TYPE=SUBSTITUTE
CM032588.1 310188 . G T 100 * TYPE=SUBSTITUTE
CM032588.1 310189 . G A 100 * TYPE=SUBSTITUTE
CM032588.1 310198 . C A 100 * TYPE=SUBSTITUTE
CM032588.1 310205 . C T 100 * TYPE=SUBSTITUTE
CM032588.1 310234 . A C 100 * TYPE=SUBSTITUTE
CM032588.1 310249 . C T 100 * TYPE=SUBSTITUTE
CM032588.1 310250 . T C 100 * TYPE=SUBSTITUTE
CM032588.1 310251 . A G 100 * TYPE=SUBSTITUTE

CM032588.1	310258	.	A	G	100	*	TYPE=SUBSTITUTE
CM032588.1	310276	.	A	G	100	*	TYPE=SUBSTITUTE
CM032588.1	310285	.	C	T	100	*	TYPE=SUBSTITUTE
CM032588.1	310309	.	A	G	100	*	TYPE=SUBSTITUTE
CM032588.1	310311	.	A	G	100	*	TYPE=SUBSTITUTE
CM032588.1	310313	.	C	G	100	*	TYPE=SUBSTITUTE
CM032588.1	310321	.	A	C	100	*	TYPE=SUBSTITUTE
CM032588.1	310328	.	A	G	100	*	TYPE=SUBSTITUTE
CM032588.1	310334	.	A	C	100	*	TYPE=SUBSTITUTE
CM032588.1	310337	.	C	T	100	*	TYPE=SUBSTITUTE
CM032588.1	310338	.	C	T	100	*	TYPE=SUBSTITUTE
CM032588.1	310340	.	G	C	100	*	TYPE=SUBSTITUTE
CM032588.1	310347	.	A	T	100	*	TYPE=SUBSTITUTE
CM032588.1	310350	.	T	C	100	*	TYPE=SUBSTITUTE
CM032588.1	310360	.	C	G	100	*	TYPE=SUBSTITUTE
CM032588.1	310362	.	C	G	100	*	TYPE=SUBSTITUTE
CM032588.1	310363	.	G	A	100	*	TYPE=SUBSTITUTE
CM032588.1	310368	.	C	T	100	*	TYPE=SUBSTITUTE
CM032588.1	310370	.	T	C	100	*	TYPE=SUBSTITUTE
CM032588.1	310385	.	C	T	100	*	TYPE=SUBSTITUTE
CM032588.1	310397	.	ACTTACTGGC	A	100	*	TYPE=DELETE
CM032588.1	310409	.	A	AG	100	*	TYPE=INSERT
CM032588.1	310411	.	A	G	100	*	TYPE=SUBSTITUTE
CM032588.1	310421	.	C	T	100	*	TYPE=SUBSTITUTE
CM032588.1	310425	.	C	T	100	*	TYPE=SUBSTITUTE
CM032588.1	310434	.	A	G	100	*	TYPE=SUBSTITUTE
CM032588.1	310436	.	C	A	100	*	TYPE=SUBSTITUTE
CM032588.1	310437	.	C	T	100	*	TYPE=SUBSTITUTE
CM032588.1	310445	.	G	A	100	*	TYPE=SUBSTITUTE
CM032588.1	310451	.	T	C	100	*	TYPE=SUBSTITUTE
CM032588.1	310452	.	C	A	100	*	TYPE=SUBSTITUTE
CM032588.1	310456	.	C	T	100	*	TYPE=SUBSTITUTE
CM032588.1	310458	.	C	CATATTAATT	100	*	TYPE=INSERT
CM032588.1	310461	.	A	C	100	*	TYPE=SUBSTITUTE
CM032588.1	310470	.	C	T	100	*	TYPE=SUBSTITUTE
CM032588.1	310489	.	G	C	100	*	TYPE=SUBSTITUTE
CM032588.1	310491	.	A	G	100	*	TYPE=SUBSTITUTE
CM032588.1	310492	.	A	G	100	*	TYPE=SUBSTITUTE
CM032588.1	310494	.	G	A	100	*	TYPE=SUBSTITUTE
CM032588.1	310497	.	T	C	100	*	TYPE=SUBSTITUTE
CM032588.1	310557	.	C	t	100	*	TYPE=SUBSTITUTE
CM032588.1	310566	.	C	T	100	*	TYPE=SUBSTITUTE
CM032588.1	310599	.	A	G	100	*	TYPE=SUBSTITUTE
CM032588.1	310608	.	C	T	100	*	TYPE=SUBSTITUTE
CM032588.1	310611	.	T	C	100	*	TYPE=SUBSTITUTE
CM032588.1	310612	.	T	C	100	*	TYPE=SUBSTITUTE
CM032588.1	310647	.	T	C	100	*	TYPE=SUBSTITUTE
CM032588.1	310653	.	T	C	100	*	TYPE=SUBSTITUTE
CM032588.1	310713	.	T	C	100	*	TYPE=SUBSTITUTE
CM032588.1	310714	.	C	G	100	*	TYPE=SUBSTITUTE
CM032588.1	310720	.	C	T	100	*	TYPE=SUBSTITUTE
CM032588.1	310732	.	C	T	100	*	TYPE=SUBSTITUTE
CM032588.1	310755	.	C	T	100	*	TYPE=SUBSTITUTE
CM032588.1	310761	.	G	A	100	*	TYPE=SUBSTITUTE
CM032588.1	310764	.	T	C	100	*	TYPE=SUBSTITUTE
CM032588.1	310765	.	T	C	100	*	TYPE=SUBSTITUTE
CM032588.1	310766	.	C	T	100	*	TYPE=SUBSTITUTE
CM032588.1	310767	.	C	G	100	*	TYPE=SUBSTITUTE
CM032588.1	310769	.	C	G	100	*	TYPE=SUBSTITUTE
CM032588.1	310782	.	A	G	100	*	TYPE=SUBSTITUTE
CM032588.1	310794	.	C	T	100	*	TYPE=SUBSTITUTE
CM032588.1	310805	.	C	A	100	*	TYPE=SUBSTITUTE
CM032588.1	310806	.	C	T	100	*	TYPE=SUBSTITUTE
CM032588.1	310808	.	A	C	100	*	TYPE=SUBSTITUTE

CM032588.1	310811	.	T	A	100	*	TYPE=SUBSTITUTE
CM032588.1	310822	.	A	G	100	*	TYPE=SUBSTITUTE
CM032588.1	310824	.	T	C	100	*	TYPE=SUBSTITUTE
CM032588.1	310830	.	G	A	100	*	TYPE=SUBSTITUTE
CM032588.1	310842	.	T	C	100	*	TYPE=SUBSTITUTE
CM032588.1	310860	.	A	C	100	*	TYPE=SUBSTITUTE
CM032588.1	310871	.	C	T	100	*	TYPE=SUBSTITUTE
CM032588.1	310872	.	G	A	100	*	TYPE=SUBSTITUTE
CM032588.1	310877	.	T	C	100	*	TYPE=SUBSTITUTE
CM032588.1	310887	.	G	A	100	*	TYPE=SUBSTITUTE
CM032588.1	310890	.	C	A	100	*	TYPE=SUBSTITUTE
CM032588.1	310893	.	T	C	100	*	TYPE=SUBSTITUTE
CM032588.1	310902	.	C	T	100	*	TYPE=SUBSTITUTE
CM032588.1	310909	.	A	C	100	*	TYPE=SUBSTITUTE
CM032588.1	310910	.	C	T	100	*	TYPE=SUBSTITUTE
CM032588.1	310921	.	G	A	100	*	TYPE=SUBSTITUTE
CM032588.1	310925	.	G	A	100	*	TYPE=SUBSTITUTE
CM032588.1	310926	.	T	C	100	*	TYPE=SUBSTITUTE
CM032588.1	310927	.	T	C	100	*	TYPE=SUBSTITUTE
CM032588.1	310930	.	A	C	100	*	TYPE=SUBSTITUTE
CM032588.1	310939	.	G	T	100	*	TYPE=SUBSTITUTE
CM032588.1	310944	.	A	G	100	*	TYPE=SUBSTITUTE
CM032588.1	310947	.	A	T	100	*	TYPE=SUBSTITUTE
CM032588.1	310953	.	C	T	100	*	TYPE=SUBSTITUTE
CM032588.1	310956	.	G	A	100	*	TYPE=SUBSTITUTE
CM032588.1	310961	.	T	A	100	*	TYPE=SUBSTITUTE
CM032588.1	310965	.	A	G	100	*	TYPE=SUBSTITUTE
CM032588.1	310977	.	C	G	100	*	TYPE=SUBSTITUTE
CM032588.1	310983	.	G	A	100	*	TYPE=SUBSTITUTE
CM032588.1	310996	.	G	C	100	*	TYPE=SUBSTITUTE
CM032588.1	311005	.	A	C	100	*	TYPE=SUBSTITUTE
CM032588.1	311007	.	G	A	100	*	TYPE=SUBSTITUTE
CM032588.1	311009	.	A	G	100	*	TYPE=SUBSTITUTE
CM032588.1	311011	.	A	G	100	*	TYPE=SUBSTITUTE
CM032588.1	311012	.	G	C	100	*	TYPE=SUBSTITUTE
CM032588.1	311031	.	C	G	100	*	TYPE=SUBSTITUTE
CM032588.1	311056	.	G	C	100	*	TYPE=SUBSTITUTE
CM032588.1	311059	.	T	C	100	*	TYPE=SUBSTITUTE
CM032588.1	311070	.	T	G	100	*	TYPE=SUBSTITUTE
CM032588.1	311080	.	C	A	100	*	TYPE=SUBSTITUTE
CM032588.1	311085	.	T	C	100	*	TYPE=SUBSTITUTE
CM032588.1	311088	.	C	T	100	*	TYPE=SUBSTITUTE
CM032588.1	311136	.	A	C	100	*	TYPE=SUBSTITUTE
CM032588.1	311148	.	T	G	100	*	TYPE=SUBSTITUTE
CM032588.1	311161	.	A	C	100	*	TYPE=SUBSTITUTE
CM032588.1	311163	.	T	A	100	*	TYPE=SUBSTITUTE
CM032588.1	311172	.	C	T	100	*	TYPE=SUBSTITUTE
CM032588.1	311196	.	C	A	100	*	TYPE=SUBSTITUTE
CM032588.1	311244	.	G	A	100	*	TYPE=SUBSTITUTE
CM032588.1	311259	.	G	C	100	*	TYPE=SUBSTITUTE
CM032588.1	311280	.	T	C	100	*	TYPE=SUBSTITUTE
CM032588.1	311292	.	T	C	100	*	TYPE=SUBSTITUTE
CM032588.1	311295	.	G	A	100	*	TYPE=SUBSTITUTE
CM032588.1	311322	.	A	G	100	*	TYPE=SUBSTITUTE
CM032588.1	311325	.	T	C	100	*	TYPE=SUBSTITUTE
CM032588.1	311328	.	T	C	100	*	TYPE=SUBSTITUTE
CM032588.1	311370	.	C	G	100	*	TYPE=SUBSTITUTE
CM032588.1	311373	.	A	G	100	*	TYPE=SUBSTITUTE
CM032588.1	311380	.	C	T	100	*	TYPE=SUBSTITUTE
CM032588.1	311382	.	T	G	100	*	TYPE=SUBSTITUTE
CM032588.1	311391	.	C	T	100	*	TYPE=SUBSTITUTE
CM032588.1	311396	.	C	G	100	*	TYPE=SUBSTITUTE
CM032588.1	311408	.	T	C	100	*	TYPE=SUBSTITUTE
CM032588.1	311415	.	G	A	100	*	TYPE=SUBSTITUTE

CM032588.1	311449	.	C	G	100	*	TYPE=SUBSTITUTE
CM032588.1	311455	.	G	A	100	*	TYPE=SUBSTITUTE
CM032588.1	311456	.	T	C	100	*	TYPE=SUBSTITUTE
CM032588.1	311458	.	G	A	100	*	TYPE=SUBSTITUTE
CM032588.1	311471	.	T	A	100	*	TYPE=SUBSTITUTE
CM032588.1	311483	.	C	G	100	*	TYPE=SUBSTITUTE
CM032588.1	311493	.	C	T	100	*	TYPE=SUBSTITUTE
CM032588.1	311495	.	A	T	100	*	TYPE=SUBSTITUTE
CM032588.1	311496	.	C	CT	100	*	TYPE=INSERT
CM032588.1	311512	.	A	G	100	*	TYPE=SUBSTITUTE
CM032588.1	311532	.	A	G	100	*	TYPE=SUBSTITUTE
CM032588.1	311534	.	A	G	100	*	TYPE=SUBSTITUTE
CM032588.1	311539	.	G	T	100	*	TYPE=SUBSTITUTE
CM032588.1	311540	.	C	T	100	*	TYPE=SUBSTITUTE
CM032588.1	311554	.	A	G	100	*	TYPE=SUBSTITUTE
CM032588.1	311555	.	G	C	100	*	TYPE=SUBSTITUTE
CM032588.1	311556	.	G	C	100	*	TYPE=SUBSTITUTE
CM032588.1	311560	.	C	G	100	*	TYPE=SUBSTITUTE
CM032588.1	311562	.	T	C	100	*	TYPE=SUBSTITUTE
CM032588.1	311569	.	A	G	100	*	TYPE=SUBSTITUTE
CM032588.1	311570	.	T	A	100	*	TYPE=SUBSTITUTE
CM032588.1	311571	.	C	T	100	*	TYPE=SUBSTITUTE
CM032588.1	311573	.	T	C	100	*	TYPE=SUBSTITUTE
CM032588.1	311576	.	T	c	100	*	TYPE=SUBSTITUTE
CM032588.1	311579	.	G	a	100	*	TYPE=SUBSTITUTE
CM032588.1	311580	.	C	a	100	*	TYPE=SUBSTITUTE
CM032588.1	311581	.	G	a	100	*	TYPE=SUBSTITUTE
CM032588.1	311582	.	T	a	100	*	TYPE=SUBSTITUTE
CM032588.1	311584	.	C	g	100	*	TYPE=SUBSTITUTE
CM032588.1	311585	.	T	a	100	*	TYPE=SUBSTITUTE
CM032588.1	311586	.	C	a	100	*	TYPE=SUBSTITUTE
CM032588.1	311587	.	A	g	100	*	TYPE=SUBSTITUTE
CM032588.1	311588	.	C	a	100	*	TYPE=SUBSTITUTE
CM032588.1	311589	.	A	g	100	*	TYPE=SUBSTITUTE
CM032588.1	311592	.	G	a	100	*	TYPE=SUBSTITUTE
CM032588.1	311596	.	C	a	100	*	TYPE=SUBSTITUTE
CM032588.1	311598	.	G	a	100	*	TYPE=SUBSTITUTE
CM032588.1	311600	.	A	g	100	*	TYPE=SUBSTITUTE
CM032588.1	311601	.	G	a	100	*	TYPE=SUBSTITUTE
CM032588.1	311614	.	G	A	100	*	TYPE=SUBSTITUTE
CM032588.1	311616	.	G	T	100	*	TYPE=SUBSTITUTE
CM032588.1	311619	.	C	T	100	*	TYPE=SUBSTITUTE
CM032588.1	311623	.	A	G	100	*	TYPE=SUBSTITUTE
CM032588.1	311625	.	T	A	100	*	TYPE=SUBSTITUTE
CM032588.1	311637	.	A	G	100	*	TYPE=SUBSTITUTE
CM032588.1	311643	.	C	T	100	*	TYPE=SUBSTITUTE
CM032588.1	311655	.	T	C	100	*	TYPE=SUBSTITUTE
CM032588.1	311658	.	C	A	100	*	TYPE=SUBSTITUTE
CM032588.1	311666	.	C	T	100	*	TYPE=SUBSTITUTE
CM032588.1	311688	.	T	C	100	*	TYPE=SUBSTITUTE
CM032588.1	311689	.	C	T	100	*	TYPE=SUBSTITUTE
CM032588.1	311694	.	A	G	100	*	TYPE=SUBSTITUTE
CM032588.1	311708	.	A	G	100	*	TYPE=SUBSTITUTE
CM032588.1	311712	.	A	T	100	*	TYPE=SUBSTITUTE
CM032588.1	311726	.	A	G	100	*	TYPE=SUBSTITUTE
CM032588.1	311739	.	G	C	100	*	TYPE=SUBSTITUTE
CM032588.1	311746	.	G	A	100	*	TYPE=SUBSTITUTE
CM032588.1	311751	.	C	T	100	*	TYPE=SUBSTITUTE
CM032588.1	311793	.	T	C	100	*	TYPE=SUBSTITUTE
CM032588.1	311809	.	A	G	100	*	TYPE=SUBSTITUTE
CM032588.1	311823	.	A	G	100	*	TYPE=SUBSTITUTE
CM032588.1	311828	.	C	T	100	*	TYPE=SUBSTITUTE
CM032588.1	311829	.	G	T	100	*	TYPE=SUBSTITUTE
CM032588.1	311835	.	C	t	100	*	TYPE=SUBSTITUTE

CM032588.1	311838	.	C	t	100	*	TYPE=SUBSTITUTE
CM032588.1	311856	.	C	T	100	*	TYPE=SUBSTITUTE
CM032588.1	311871	.	G	T	100	*	TYPE=SUBSTITUTE
CM032588.1	311884	.	T	C	100	*	TYPE=SUBSTITUTE
CM032588.1	311892	.	T	C	100	*	TYPE=SUBSTITUTE
CM032588.1	311895	.	A	C	100	*	TYPE=SUBSTITUTE
CM032588.1	311912	.	A	C	100	*	TYPE=SUBSTITUTE
CM032588.1	311929	.	G	T	100	*	TYPE=SUBSTITUTE
CM032588.1	311932	.	C	A	100	*	TYPE=SUBSTITUTE
CM032588.1	311935	.	G	C	100	*	TYPE=SUBSTITUTE
CM032588.1	311936	.	T	A	100	*	TYPE=SUBSTITUTE
CM032588.1	311943	.	G	A	100	*	TYPE=SUBSTITUTE
CM032588.1	311953	.	G	A	100	*	TYPE=SUBSTITUTE
CM032588.1	311955	.	G	A	100	*	TYPE=SUBSTITUTE
CM032588.1	311965	.	C	A	100	*	TYPE=SUBSTITUTE
CM032588.1	311973	.	T	C	100	*	TYPE=SUBSTITUTE
CM032588.1	311976	.	A	G	100	*	TYPE=SUBSTITUTE
CM032588.1	311984	.	C	T	100	*	TYPE=SUBSTITUTE
CM032588.1	312007	.	G	A	100	*	TYPE=SUBSTITUTE
CM032588.1	312017	.	C	T	100	*	TYPE=SUBSTITUTE
CM032588.1	312019	.	T	A	100	*	TYPE=SUBSTITUTE
CM032588.1	312025	.	G	A	100	*	TYPE=SUBSTITUTE
CM032588.1	312026	.	G	A	100	*	TYPE=SUBSTITUTE
CM032588.1	312027	.	C	G	100	*	TYPE=SUBSTITUTE
CM032588.1	312028	.	G	A	100	*	TYPE=SUBSTITUTE
CM032588.1	312029	.	T	C	100	*	TYPE=SUBSTITUTE
CM032588.1	312035	.	G	C	100	*	TYPE=SUBSTITUTE
CM032588.1	312039	.	A	G	100	*	TYPE=SUBSTITUTE
CM032588.1	312042	.	C	G	100	*	TYPE=SUBSTITUTE
CM032588.1	312045	.	T	C	100	*	TYPE=SUBSTITUTE
CM032588.1	312048	.	T	C	100	*	TYPE=SUBSTITUTE
CM032588.1	312060	.	C	T	100	*	TYPE=SUBSTITUTE
CM032588.1	312066	.	T	C	100	*	TYPE=SUBSTITUTE
CM032588.1	312072	.	C	T	100	*	TYPE=SUBSTITUTE
CM032588.1	312078	.	G	A	100	*	TYPE=SUBSTITUTE
CM032588.1	312080	.	G	A	100	*	TYPE=SUBSTITUTE
CM032588.1	312099	.	C	T	100	*	TYPE=SUBSTITUTE
CM032588.1	312103	.	C	T	100	*	TYPE=SUBSTITUTE
CM032588.1	312108	.	G	C	100	*	TYPE=SUBSTITUTE
CM032588.1	312109	.	G	A	100	*	TYPE=SUBSTITUTE
CM032588.1	312114	.	C	T	100	*	TYPE=SUBSTITUTE
CM032588.1	312115	.	T	C	100	*	TYPE=SUBSTITUTE
CM032588.1	312117	.	C	T	100	*	TYPE=SUBSTITUTE
CM032588.1	312123	.	T	G	100	*	TYPE=SUBSTITUTE
CM032588.1	312131	.	A	G	100	*	TYPE=SUBSTITUTE
CM032588.1	312132	.	G	A	100	*	TYPE=SUBSTITUTE
CM032588.1	312138	.	T	G	100	*	TYPE=SUBSTITUTE
CM032588.1	312139	.	A	G	100	*	TYPE=SUBSTITUTE
CM032588.1	312141	.	T	C	100	*	TYPE=SUBSTITUTE
CM032588.1	312143	.	G	A	100	*	TYPE=SUBSTITUTE
CM032588.1	312144	.	T	A	100	*	TYPE=SUBSTITUTE
CM032588.1	312147	.	A	G	100	*	TYPE=SUBSTITUTE
CM032588.1	312150	.	C	T	100	*	TYPE=SUBSTITUTE
CM032588.1	312156	.	C	A	100	*	TYPE=SUBSTITUTE
CM032588.1	312162	.	T	C	100	*	TYPE=SUBSTITUTE
CM032588.1	312177	.	C	T	100	*	TYPE=SUBSTITUTE
CM032588.1	312193	.	A	C	100	*	TYPE=SUBSTITUTE
CM032588.1	312201	.	C	T	100	*	TYPE=SUBSTITUTE
CM032588.1	312219	.	G	A	100	*	TYPE=SUBSTITUTE
CM032588.1	312220	.	T	C	100	*	TYPE=SUBSTITUTE
CM032588.1	312228	.	T	C	100	*	TYPE=SUBSTITUTE
CM032588.1	312231	.	T	G	100	*	TYPE=SUBSTITUTE
CM032588.1	312234	.	T	C	100	*	TYPE=SUBSTITUTE
CM032588.1	312249	.	A	g	100	*	TYPE=SUBSTITUTE

CM032588.1	312270	.	C	T	100	*	TYPE=SUBSTITUTE
CM032588.1	312280	.	G	a	100	*	TYPE=SUBSTITUTE
CM032588.1	312283	.	G	c	100	*	TYPE=SUBSTITUTE
CM032588.1	312284	.	C	t	100	*	TYPE=SUBSTITUTE
CM032588.1	312286	.	G	a	100	*	TYPE=SUBSTITUTE
CM032588.1	312288	.	T	c	100	*	TYPE=SUBSTITUTE
CM032588.1	312291	.	T	c	100	*	TYPE=SUBSTITUTE
CM032588.1	312292	.	C	a	100	*	TYPE=SUBSTITUTE
CM032588.1	312294	.	T	G	100	*	TYPE=SUBSTITUTE
CM032588.1	312295	.	T	C	100	*	TYPE=SUBSTITUTE
CM032588.1	312297	.	G	A	100	*	TYPE=SUBSTITUTE
CM032588.1	312299	.	A	G	100	*	TYPE=SUBSTITUTE
CM032588.1	312300	.	C	A	100	*	TYPE=SUBSTITUTE
CM032588.1	312305	.	A	G	100	*	TYPE=SUBSTITUTE
CM032588.1	312312	.	T	C	100	*	TYPE=SUBSTITUTE
CM032588.1	312315	.	C	G	100	*	TYPE=SUBSTITUTE
CM032588.1	312317	.	G	A	100	*	TYPE=SUBSTITUTE
CM032588.1	312318	.	G	A	100	*	TYPE=SUBSTITUTE
CM032588.1	312322	.	A	G	100	*	TYPE=SUBSTITUTE
CM032588.1	312324	.	C	A	100	*	TYPE=SUBSTITUTE
CM032588.1	312327	.	C	G	100	*	TYPE=SUBSTITUTE
CM032588.1	312328	.	C	T	100	*	TYPE=SUBSTITUTE
CM032588.1	312335	.	C	G	100	*	TYPE=SUBSTITUTE
CM032588.1	312337	.	A	T	100	*	TYPE=SUBSTITUTE
CM032588.1	312338	.	A	C	100	*	TYPE=SUBSTITUTE
CM032588.1	312342	.	G	T	100	*	TYPE=SUBSTITUTE
CM032588.1	312345	.	A	T	100	*	TYPE=SUBSTITUTE
CM032588.1	312366	.	T	C	100	*	TYPE=SUBSTITUTE
CM032588.1	312370	.	A	C	100	*	TYPE=SUBSTITUTE
CM032588.1	312376	.	A	G	100	*	TYPE=SUBSTITUTE
CM032588.1	312378	.	A	G	100	*	TYPE=SUBSTITUTE
CM032588.1	312387	.	C	T	100	*	TYPE=SUBSTITUTE
CM032588.1	312389	.	A	G	100	*	TYPE=SUBSTITUTE
CM032588.1	312397	.	C	T	100	*	TYPE=SUBSTITUTE
CM032588.1	312399	.	G	A	100	*	TYPE=SUBSTITUTE
CM032588.1	312414	.	T	C	100	*	TYPE=SUBSTITUTE
CM032588.1	312420	.	C	T	100	*	TYPE=SUBSTITUTE
CM032588.1	312423	.	C	T	100	*	TYPE=SUBSTITUTE
CM032588.1	312432	.	T	G	100	*	TYPE=SUBSTITUTE
CM032588.1	312442	.	T	C	100	*	TYPE=SUBSTITUTE
CM032588.1	312445	.	T	C	100	*	TYPE=SUBSTITUTE
CM032588.1	312462	.	A	G	100	*	TYPE=SUBSTITUTE
CM032588.1	312467	.	A	G	100	*	TYPE=SUBSTITUTE
CM032588.1	312501	.	A	C	100	*	TYPE=SUBSTITUTE
CM032588.1	312507	.	A	T	100	*	TYPE=SUBSTITUTE
CM032588.1	312512	.	G	A	100	*	TYPE=SUBSTITUTE
CM032588.1	312519	.	G	A	100	*	TYPE=SUBSTITUTE
CM032588.1	312525	.	A	G	100	*	TYPE=SUBSTITUTE
CM032588.1	312528	.	C	T	100	*	TYPE=SUBSTITUTE
CM032588.1	312530	.	G	A	100	*	TYPE=SUBSTITUTE
CM032588.1	312534	.	A	G	100	*	TYPE=SUBSTITUTE
CM032588.1	312535	.	C	G	100	*	TYPE=SUBSTITUTE
CM032588.1	312536	.	C	T	100	*	TYPE=SUBSTITUTE
CM032588.1	312538	.	C	G	100	*	TYPE=SUBSTITUTE
CM032588.1	312540	.	A	G	100	*	TYPE=SUBSTITUTE
CM032588.1	312582	.	G	A	100	*	TYPE=SUBSTITUTE
CM032588.1	312586	.	G	A	100	*	TYPE=SUBSTITUTE
CM032588.1	312588	.	C	G	100	*	TYPE=SUBSTITUTE
CM032588.1	312591	.	T	C	100	*	TYPE=SUBSTITUTE
CM032588.1	312606	.	A	G	100	*	TYPE=SUBSTITUTE
CM032588.1	312630	.	A	T	100	*	TYPE=SUBSTITUTE
CM032588.1	312633	.	C	T	100	*	TYPE=SUBSTITUTE
CM032588.1	312666	.	T	C	100	*	TYPE=SUBSTITUTE
CM032588.1	312677	.	T	C	100	*	TYPE=SUBSTITUTE

CM032588.1	312735	.	A	G	100	*	TYPE=SUBSTITUTE
CM032588.1	312783	.	T	C	100	*	TYPE=SUBSTITUTE
CM032588.1	312787	.	G	A	100	*	TYPE=SUBSTITUTE
CM032588.1	312845	.	T	C	100	*	TYPE=SUBSTITUTE
CM032588.1	312918	.	A	G	100	*	TYPE=SUBSTITUTE
CM032588.1	312925	.	T	C	100	*	TYPE=SUBSTITUTE
CM032588.1	312937	.	G	C	100	*	TYPE=SUBSTITUTE
CM032588.1	312948	.	T	A	100	*	TYPE=SUBSTITUTE
CM032588.1	312963	.	T	G	100	*	TYPE=SUBSTITUTE
CM032588.1	312980	.	A	T	100	*	TYPE=SUBSTITUTE
CM032588.1	312996	.	GGGAGAAAATGGA	G	100	*	TYPE=DELETE
CM032588.1	313016	.	C	T	100	*	TYPE=SUBSTITUTE
CM032588.1	313023	.	G	A	100	*	TYPE=SUBSTITUTE
CM032588.1	313030	.	T	C	100	*	TYPE=SUBSTITUTE
CM032588.1	313039	.	T	C	100	*	TYPE=SUBSTITUTE
CM032588.1	313059	.	A	G	100	*	TYPE=SUBSTITUTE
CM032588.1	313062	.	G	A	100	*	TYPE=SUBSTITUTE
CM032588.1	313233	.	C	T	100	*	TYPE=SUBSTITUTE
CM032588.1	313260	.	G	A	100	*	TYPE=SUBSTITUTE
CM032588.1	313285	.	A	G	100	*	TYPE=SUBSTITUTE
CM032588.1	313301	.	C	T	100	*	TYPE=SUBSTITUTE
CM032588.1	313349	.	A	T	100	*	TYPE=SUBSTITUTE
CM032588.1	313377	.	A	G	100	*	TYPE=SUBSTITUTE
CM032588.1	313391	.	A	G	100	*	TYPE=SUBSTITUTE
CM032588.1	313451	.	C	T	100	*	TYPE=SUBSTITUTE
CM032588.1	313461	.	A	G	100	*	TYPE=SUBSTITUTE
CM032588.1	313466	.	T	C	100	*	TYPE=SUBSTITUTE
CM032588.1	313474	.	G	A	100	*	TYPE=SUBSTITUTE
CM032588.1	313476	.	G	A	100	*	TYPE=SUBSTITUTE
CM032588.1	313531	.	A	G	100	*	TYPE=SUBSTITUTE
CM032588.1	313557	.	A	G	100	*	TYPE=SUBSTITUTE
CM032588.1	313575	.	G	A	100	*	TYPE=SUBSTITUTE
CM032588.1	313590	.	G	A	100	*	TYPE=SUBSTITUTE
CM032588.1	313605	.	G	A	100	*	TYPE=SUBSTITUTE
CM032588.1	313607	.	C	T	100	*	TYPE=SUBSTITUTE
CM032588.1	313614	.	A	G	100	*	TYPE=SUBSTITUTE
CM032588.1	313615	.	C	T	100	*	TYPE=SUBSTITUTE
CM032588.1	313616	.	G	A	100	*	TYPE=SUBSTITUTE
CM032588.1	313617	.	C	T	100	*	TYPE=SUBSTITUTE
CM032588.1	313620	.	G	A	100	*	TYPE=SUBSTITUTE
CM032588.1	313640	.	T	G	100	*	TYPE=SUBSTITUTE
CM032588.1	313648	.	G	A	100	*	TYPE=SUBSTITUTE
CM032588.1	313651	.	G	A	100	*	TYPE=SUBSTITUTE
CM032588.1	313654	.	A	G	100	*	TYPE=SUBSTITUTE
CM032588.1	313659	.	G	A	100	*	TYPE=SUBSTITUTE
CM032588.1	313664	.	C	G	100	*	TYPE=SUBSTITUTE
CM032588.1	313665	.	A	G	100	*	TYPE=SUBSTITUTE
CM032588.1	313671	.	T	C	100	*	TYPE=SUBSTITUTE
CM032588.1	313676	.	G	T	100	*	TYPE=SUBSTITUTE
CM032588.1	313677	.	G	T	100	*	TYPE=SUBSTITUTE
CM032588.1	313806	.	G	A	100	*	TYPE=SUBSTITUTE
CM032588.1	313972	.	A	C	100	*	TYPE=SUBSTITUTE
CM032588.1	314005	.	G	A	100	*	TYPE=SUBSTITUTE
CM032588.1	314054	.	G	C	100	*	TYPE=SUBSTITUTE
CM032588.1	314055	.	G	T	100	*	TYPE=SUBSTITUTE
CM032588.1	314187	.	G	T	100	*	TYPE=SUBSTITUTE
CM032588.1	314452	.	C	G	100	*	TYPE=SUBSTITUTE
CM032588.1	314485	.	G	A	100	*	TYPE=SUBSTITUTE
CM032588.1	314486	.	C	G	100	*	TYPE=SUBSTITUTE
CM032588.1	314518	.	G	A	100	*	TYPE=SUBSTITUTE
CM032588.1	314526	.	A	C	100	*	TYPE=SUBSTITUTE
CM032588.1	314544	.	A	T	100	*	TYPE=SUBSTITUTE
CM032588.1	314556	.	T	c	100	*	TYPE=SUBSTITUTE
CM032588.1	314565	.	G	a	100	*	TYPE=SUBSTITUTE

CM032588.1	314579	.	G	A	100	*	TYPE=SUBSTITUTE
CM032588.1	314593	.	T	G	100	*	TYPE=SUBSTITUTE
CM032588.1	314605	.	G	A	100	*	TYPE=SUBSTITUTE
CM032588.1	314623	.	C	a	100	*	TYPE=SUBSTITUTE
CM032588.1	314647	.	T	C	100	*	TYPE=SUBSTITUTE
CM032588.1	314681	.	T	C	100	*	TYPE=SUBSTITUTE
CM032588.1	314683	.	C	G	100	*	TYPE=SUBSTITUTE
CM032588.1	314687	.	C	T	100	*	TYPE=SUBSTITUTE
CM032588.1	314699	.	CA	C	100	*	TYPE=DELETE
CM032588.1	314712	.	a	at	100	*	TYPE=INSERT
CM032588.1	314746	.	T	C	100	*	TYPE=SUBSTITUTE
CM032588.1	314756	.	T	A	100	*	TYPE=SUBSTITUTE
CM032588.1	314812	.	T	TT	100	*	TYPE=INSERT
CM032588.1	314827	.	G	A	100	*	TYPE=SUBSTITUTE
CM032588.1	314847	.	A	T	100	*	TYPE=SUBSTITUTE
CM032588.1	314848	.	C	T	100	*	TYPE=SUBSTITUTE
CM032588.1	314859	.	G	T	100	*	TYPE=SUBSTITUTE
CM032588.1	314861	.	G	C	100	*	TYPE=SUBSTITUTE
CM032588.1	314870	.	C	T	100	*	TYPE=SUBSTITUTE
CM032588.1	314873	.	C	T	100	*	TYPE=SUBSTITUTE
CM032588.1	314876	.	C	A	100	*	TYPE=SUBSTITUTE
CM032588.1	314891	.	G	A	100	*	TYPE=SUBSTITUTE
CM032588.1	314900	.	A	C	100	*	TYPE=SUBSTITUTE
CM032588.1	314912	.	T	C	100	*	TYPE=SUBSTITUTE
CM032588.1	314930	.	A	G	100	*	TYPE=SUBSTITUTE
CM032588.1	314975	.	T	c	100	*	TYPE=SUBSTITUTE
CM032588.1	315017	.	G	a	100	*	TYPE=SUBSTITUTE
CM032588.1	315035	.	C	t	100	*	TYPE=SUBSTITUTE
CM032588.1	315041	.	C	a	100	*	TYPE=SUBSTITUTE
CM032588.1	315044	.	A	g	100	*	TYPE=SUBSTITUTE
CM032588.1	315053	.	C	T	100	*	TYPE=SUBSTITUTE
CM032588.1	315059	.	T	C	100	*	TYPE=SUBSTITUTE
CM032588.1	315080	.	C	T	100	*	TYPE=SUBSTITUTE
CM032588.1	315098	.	G	a	100	*	TYPE=SUBSTITUTE
CM032588.1	315125	.	G	a	100	*	TYPE=SUBSTITUTE
CM032588.1	315128	.	A	t	100	*	TYPE=SUBSTITUTE
CM032588.1	315137	.	G	a	100	*	TYPE=SUBSTITUTE
CM032588.1	315140	.	A	g	100	*	TYPE=SUBSTITUTE
CM032588.1	315164	.	T	a	100	*	TYPE=SUBSTITUTE
CM032588.1	315170	.	C	a	100	*	TYPE=SUBSTITUTE
CM032588.1	315176	.	C	T	100	*	TYPE=SUBSTITUTE
CM032588.1	315213	.	A	G	100	*	TYPE=SUBSTITUTE
CM032588.1	315257	.	G	A	100	*	TYPE=SUBSTITUTE
CM032588.1	315278	.	C	G	100	*	TYPE=SUBSTITUTE
CM032588.1	315287	.	CAATGCCTCCGAA	C	100	*	TYPE=DELETE
CM032588.1	315302	.	A	T	100	*	TYPE=SUBSTITUTE
CM032588.1	315398	.	T	C	100	*	TYPE=SUBSTITUTE
CM032588.1	315435	.	A	G	100	*	TYPE=SUBSTITUTE
CM032588.1	315455	.	G	C	100	*	TYPE=SUBSTITUTE
CM032588.1	315458	.	C	T	100	*	TYPE=SUBSTITUTE
CM032588.1	315468	.	C	t	100	*	TYPE=SUBSTITUTE
CM032588.1	315473	.	A	g	100	*	TYPE=SUBSTITUTE
CM032588.1	315485	.	A	g	100	*	TYPE=SUBSTITUTE
CM032588.1	315515	.	T	C	100	*	TYPE=SUBSTITUTE
CM032588.1	315530	.	T	C	100	*	TYPE=SUBSTITUTE
CM032588.1	315557	.	T	C	100	*	TYPE=SUBSTITUTE
CM032588.1	315581	.	G	T	100	*	TYPE=SUBSTITUTE
CM032588.1	315602	.	T	C	100	*	TYPE=SUBSTITUTE
CM032588.1	315614	.	C	G	100	*	TYPE=SUBSTITUTE
CM032588.1	315620	.	T	C	100	*	TYPE=SUBSTITUTE
CM032588.1	315634	.	A	t	100	*	TYPE=SUBSTITUTE
CM032588.1	315635	.	G	c	100	*	TYPE=SUBSTITUTE
CM032588.1	315641	.	T	c	100	*	TYPE=SUBSTITUTE
CM032588.1	315648	.	T	g	100	*	TYPE=SUBSTITUTE

CM032588.1	315677	.	C	T	100	*	TYPE=SUBSTITUTE
CM032588.1	315686	.	G	C	100	*	TYPE=SUBSTITUTE
CM032588.1	315689	.	T	C	100	*	TYPE=SUBSTITUTE
CM032588.1	315717	.	G	C	100	*	TYPE=SUBSTITUTE
CM032588.1	315719	.	G	A	100	*	TYPE=SUBSTITUTE
CM032588.1	315728	.	C	T	100	*	TYPE=SUBSTITUTE
CM032588.1	315731	.	C	T	100	*	TYPE=SUBSTITUTE
CM032588.1	315824	.	T	C	100	*	TYPE=SUBSTITUTE
CM032588.1	315827	.	T	C	100	*	TYPE=SUBSTITUTE
CM032588.1	315830	.	A	T	100	*	TYPE=SUBSTITUTE
CM032588.1	315842	.	C	T	100	*	TYPE=SUBSTITUTE
CM032588.1	315899	.	T	c	100	*	TYPE=SUBSTITUTE
CM032588.1	315932	.	T	C	100	*	TYPE=SUBSTITUTE
CM032588.1	315989	.	T	C	100	*	TYPE=SUBSTITUTE
CM032588.1	316055	.	T	C	100	*	TYPE=SUBSTITUTE
CM032588.1	316085	.	A	G	100	*	TYPE=SUBSTITUTE
CM032588.1	316097	.	C	G	100	*	TYPE=SUBSTITUTE
CM032588.1	316115	.	C	T	100	*	TYPE=SUBSTITUTE
CM032588.1	316124	.	C	G	100	*	TYPE=SUBSTITUTE
CM032588.1	316136	.	G	A	100	*	TYPE=SUBSTITUTE
CM032588.1	316169	.	G	C	100	*	TYPE=SUBSTITUTE
CM032588.1	316170	.	T	C	100	*	TYPE=SUBSTITUTE
CM032588.1	316286	.	T	C	100	*	TYPE=SUBSTITUTE
CM032588.1	316289	.	T	C	100	*	TYPE=SUBSTITUTE
CM032588.1	316292	.	T	C	100	*	TYPE=SUBSTITUTE
CM032588.1	316295	.	C	T	100	*	TYPE=SUBSTITUTE
CM032588.1	316319	.	A	G	100	*	TYPE=SUBSTITUTE
CM032588.1	316334	.	G	A	100	*	TYPE=SUBSTITUTE
CM032588.1	316360	.	A	C	100	*	TYPE=SUBSTITUTE
CM032588.1	316364	.	G	A	100	*	TYPE=SUBSTITUTE
CM032588.1	316382	.	C	T	100	*	TYPE=SUBSTITUTE
CM032588.1	316436	.	T	C	100	*	TYPE=SUBSTITUTE
CM032588.1	316448	.	GCT	G	100	*	TYPE=DELETE
CM032588.1	316458	.	T	A	100	*	TYPE=SUBSTITUTE
CM032588.1	316473	.	G	A	100	*	TYPE=SUBSTITUTE
CM032588.1	316483	.	G	A	100	*	TYPE=SUBSTITUTE
CM032588.1	316488	.	G	T	100	*	TYPE=SUBSTITUTE
CM032588.1	316490	.	T	G	100	*	TYPE=SUBSTITUTE
CM032588.1	316494	.	C	T	100	*	TYPE=SUBSTITUTE
CM032588.1	316534	.	C	T	100	*	TYPE=SUBSTITUTE
CM032588.1	316546	.	A	G	100	*	TYPE=SUBSTITUTE
CM032588.1	316579	.	T	c	100	*	TYPE=SUBSTITUTE
CM032588.1	316594	.	G	A	100	*	TYPE=SUBSTITUTE
CM032588.1	316630	.	T	C	100	*	TYPE=SUBSTITUTE
CM032588.1	316641	.	T	A	100	*	TYPE=SUBSTITUTE
CM032588.1	316645	.	C	G	100	*	TYPE=SUBSTITUTE
CM032588.1	316647	.	C	T	100	*	TYPE=SUBSTITUTE
CM032588.1	316652	.	G	C	100	*	TYPE=SUBSTITUTE
CM032588.1	316669	.	C	G	100	*	TYPE=SUBSTITUTE
CM032588.1	316740	.	A	c	100	*	TYPE=SUBSTITUTE
CM032588.1	316852	.	G	t	100	*	TYPE=SUBSTITUTE
CM032588.1	316853	.	G	a	100	*	TYPE=SUBSTITUTE
CM032588.1	316860	.	C	CG	100	*	TYPE=INSERT
CM032588.1	316896	.	T	tttt	100	*	TYPE=INSERT
CM032588.1	317004	.	T	C	100	*	TYPE=SUBSTITUTE
CM032588.1	317029	.	G	a	100	*	TYPE=SUBSTITUTE
CM032588.1	317117	.	T	C	100	*	TYPE=SUBSTITUTE
CM032588.1	317121	.	C	T	100	*	TYPE=SUBSTITUTE
CM032588.1	317128	.	G	T	100	*	TYPE=SUBSTITUTE
CM032588.1	317138	.	T	C	100	*	TYPE=SUBSTITUTE
CM032588.1	317139	.	G	C	100	*	TYPE=SUBSTITUTE
CM032588.1	317141	.	C	G	100	*	TYPE=SUBSTITUTE
CM032588.1	317163	.	A	T	100	*	TYPE=SUBSTITUTE
CM032588.1	317167	.	C	A	100	*	TYPE=SUBSTITUTE

CM032588.1	317208	.	G	A	100	*	TYPE=SUBSTITUTE
CM032588.1	317213	.	T	A	100	*	TYPE=SUBSTITUTE
CM032588.1	317217	.	G	A	100	*	TYPE=SUBSTITUTE
CM032588.1	317235	.	A	G	100	*	TYPE=SUBSTITUTE
CM032588.1	317244	.	G	A	100	*	TYPE=SUBSTITUTE
CM032588.1	317253	.	G	A	100	*	TYPE=SUBSTITUTE
CM032588.1	317254	.	G	A	100	*	TYPE=SUBSTITUTE
CM032588.1	317255	.	T	C	100	*	TYPE=SUBSTITUTE
CM032588.1	317262	.	A	G	100	*	TYPE=SUBSTITUTE
CM032588.1	317264	.	T	C	100	*	TYPE=SUBSTITUTE
CM032588.1	317307	.	A	G	100	*	TYPE=SUBSTITUTE
CM032588.1	317315	.	A	G	100	*	TYPE=SUBSTITUTE
CM032588.1	317325	.	A	G	100	*	TYPE=SUBSTITUTE
CM032588.1	317328	.	T	G	100	*	TYPE=SUBSTITUTE
CM032588.1	317331	.	C	T	100	*	TYPE=SUBSTITUTE
CM032588.1	317334	.	G	A	100	*	TYPE=SUBSTITUTE
CM032588.1	317336	.	A	T	100	*	TYPE=SUBSTITUTE
CM032588.1	317340	.	G	A	100	*	TYPE=SUBSTITUTE
CM032588.1	317349	.	A	C	100	*	TYPE=SUBSTITUTE
CM032588.1	317397	.	T	C	100	*	TYPE=SUBSTITUTE
CM032588.1	317403	.	A	G	100	*	TYPE=SUBSTITUTE
CM032588.1	317409	.	T	C	100	*	TYPE=SUBSTITUTE
CM032588.1	317412	.	C	T	100	*	TYPE=SUBSTITUTE
CM032588.1	317430	.	A	G	100	*	TYPE=SUBSTITUTE
CM032588.1	317448	.	A	G	100	*	TYPE=SUBSTITUTE
CM032588.1	317498	.	G	A	100	*	TYPE=SUBSTITUTE
CM032588.1	317502	.	C	T	100	*	TYPE=SUBSTITUTE
CM032588.1	317529	.	A	G	100	*	TYPE=SUBSTITUTE
CM032588.1	317538	.	C	T	100	*	TYPE=SUBSTITUTE
CM032588.1	317541	.	A	G	100	*	TYPE=SUBSTITUTE
CM032588.1	317559	.	G	C	100	*	TYPE=SUBSTITUTE
CM032588.1	317565	.	A	G	100	*	TYPE=SUBSTITUTE
CM032588.1	317571	.	C	A	100	*	TYPE=SUBSTITUTE
CM032588.1	317577	.	A	G	100	*	TYPE=SUBSTITUTE
CM032588.1	317583	.	G	A	100	*	TYPE=SUBSTITUTE
CM032588.1	317592	.	A	G	100	*	TYPE=SUBSTITUTE
CM032588.1	317615	.	A	G	100	*	TYPE=SUBSTITUTE
CM032588.1	317616	.	A	G	100	*	TYPE=SUBSTITUTE
CM032588.1	317620	.	G	A	100	*	TYPE=SUBSTITUTE
CM032588.1	317627	.	G	A	100	*	TYPE=SUBSTITUTE
CM032588.1	317637	.	T	C	100	*	TYPE=SUBSTITUTE
CM032588.1	317655	.	G	a	100	*	TYPE=SUBSTITUTE
CM032588.1	317658	.	C	t	100	*	TYPE=SUBSTITUTE
CM032588.1	317661	.	C	t	100	*	TYPE=SUBSTITUTE
CM032588.1	317670	.	A	G	100	*	TYPE=SUBSTITUTE
CM032588.1	317675	.	C	G	100	*	TYPE=SUBSTITUTE
CM032588.1	317676	.	T	G	100	*	TYPE=SUBSTITUTE
CM032588.1	317680	.	G	t	100	*	TYPE=SUBSTITUTE
CM032588.1	317683	.	T	c	100	*	TYPE=SUBSTITUTE
CM032588.1	317684	.	G	t	100	*	TYPE=SUBSTITUTE
CM032588.1	317724	.	A	G	100	*	TYPE=SUBSTITUTE
CM032588.1	317739	.	A	G	100	*	TYPE=SUBSTITUTE
CM032588.1	317748	.	A	G	100	*	TYPE=SUBSTITUTE
CM032588.1	317754	.	T	C	100	*	TYPE=SUBSTITUTE
CM032588.1	317760	.	T	G	100	*	TYPE=SUBSTITUTE
CM032588.1	317778	.	G	A	100	*	TYPE=SUBSTITUTE
CM032588.1	317796	.	T	A	100	*	TYPE=SUBSTITUTE
CM032588.1	317823	.	G	C	100	*	TYPE=SUBSTITUTE
CM032588.1	317826	.	T	C	100	*	TYPE=SUBSTITUTE
CM032588.1	317832	.	T	A	100	*	TYPE=SUBSTITUTE
CM032588.1	317838	.	T	G	100	*	TYPE=SUBSTITUTE
CM032588.1	317853	.	A	G	100	*	TYPE=SUBSTITUTE
CM032588.1	317901	.	G	A	100	*	TYPE=SUBSTITUTE
CM032588.1	317922	.	G	A	100	*	TYPE=SUBSTITUTE

CM032588.1	317925	.	T	C	100	*	TYPE=SUBSTITUTE
CM032588.1	317939	.	C	T	100	*	TYPE=SUBSTITUTE
CM032588.1	317942	.	T	C	100	*	TYPE=SUBSTITUTE
CM032588.1	317943	.	T	C	100	*	TYPE=SUBSTITUTE
CM032588.1	317944	.	C	T	100	*	TYPE=SUBSTITUTE
CM032588.1	317949	.	A	G	100	*	TYPE=SUBSTITUTE
CM032588.1	317955	.	C	A	100	*	TYPE=SUBSTITUTE
CM032588.1	317976	.	G	A	100	*	TYPE=SUBSTITUTE
CM032588.1	317982	.	A	G	100	*	TYPE=SUBSTITUTE
CM032588.1	317985	.	G	A	100	*	TYPE=SUBSTITUTE
CM032588.1	317986	.	C	T	100	*	TYPE=SUBSTITUTE
CM032588.1	317988	.	G	A	100	*	TYPE=SUBSTITUTE
CM032588.1	318015	.	A	G	100	*	TYPE=SUBSTITUTE
CM032588.1	318042	.	T	G	100	*	TYPE=SUBSTITUTE
CM032588.1	318045	.	T	C	100	*	TYPE=SUBSTITUTE
CM032588.1	318047	.	A	G	100	*	TYPE=SUBSTITUTE
CM032588.1	318048	.	T	C	100	*	TYPE=SUBSTITUTE
CM032588.1	318084	.	G	A	100	*	TYPE=SUBSTITUTE
CM032588.1	318096	.	T	G	100	*	TYPE=SUBSTITUTE
CM032588.1	318101	.	C	A	100	*	TYPE=SUBSTITUTE
CM032588.1	318105	.	A	G	100	*	TYPE=SUBSTITUTE
CM032588.1	318114	.	G	A	100	*	TYPE=SUBSTITUTE
CM032588.1	318129	.	A	G	100	*	TYPE=SUBSTITUTE
CM032588.1	318132	.	G	A	100	*	TYPE=SUBSTITUTE
CM032588.1	318138	.	A	C	100	*	TYPE=SUBSTITUTE
CM032588.1	318168	.	G	A	100	*	TYPE=SUBSTITUTE
CM032588.1	318174	.	T	C	100	*	TYPE=SUBSTITUTE
CM032588.1	318177	.	A	C	100	*	TYPE=SUBSTITUTE
CM032588.1	318201	.	A	G	100	*	TYPE=SUBSTITUTE
CM032588.1	318213	.	A	C	100	*	TYPE=SUBSTITUTE
CM032588.1	318216	.	G	A	100	*	TYPE=SUBSTITUTE
CM032588.1	318228	.	G	A	100	*	TYPE=SUBSTITUTE
CM032588.1	318245	.	G	A	100	*	TYPE=SUBSTITUTE
CM032588.1	318276	.	A	G	100	*	TYPE=SUBSTITUTE
CM032588.1	318279	.	C	A	100	*	TYPE=SUBSTITUTE
CM032588.1	318318	.	T	G	100	*	TYPE=SUBSTITUTE
CM032588.1	318320	.	T	C	100	*	TYPE=SUBSTITUTE
CM032588.1	318336	.	G	A	100	*	TYPE=SUBSTITUTE
CM032588.1	318394	.	A	G	100	*	TYPE=SUBSTITUTE
CM032588.1	318396	.	G	C	100	*	TYPE=SUBSTITUTE
CM032588.1	318453	.	A	G	100	*	TYPE=SUBSTITUTE
CM032588.1	318474	.	A	G	100	*	TYPE=SUBSTITUTE
CM032588.1	318504	.	A	G	100	*	TYPE=SUBSTITUTE
CM032588.1	318594	.	A	G	100	*	TYPE=SUBSTITUTE
CM032588.1	318659	.	G	A	100	*	TYPE=SUBSTITUTE
CM032588.1	318693	.	G	A	100	*	TYPE=SUBSTITUTE
CM032588.1	318696	.	G	T	100	*	TYPE=SUBSTITUTE
CM032588.1	318697	.	A	T	100	*	TYPE=SUBSTITUTE
CM032588.1	318699	.	G	A	100	*	TYPE=SUBSTITUTE
CM032588.1	318701	.	A	T	100	*	TYPE=SUBSTITUTE
CM032588.1	318702	.	T	G	100	*	TYPE=SUBSTITUTE
CM032588.1	318703	.	T	C	100	*	TYPE=SUBSTITUTE
CM032588.1	318705	.	G	A	100	*	TYPE=SUBSTITUTE
CM032588.1	318710	.	G	C	100	*	TYPE=SUBSTITUTE
CM032588.1	318711	.	C	T	100	*	TYPE=SUBSTITUTE
CM032588.1	318716	.	G	A	100	*	TYPE=SUBSTITUTE
CM032588.1	318722	.	G	A	100	*	TYPE=SUBSTITUTE
CM032588.1	318726	.	C	T	100	*	TYPE=SUBSTITUTE
CM032588.1	318729	.	T	G	100	*	TYPE=SUBSTITUTE
CM032588.1	318759	.	T	G	100	*	TYPE=SUBSTITUTE
CM032588.1	318760	.	A	G	100	*	TYPE=SUBSTITUTE
CM032588.1	318770	.	G	A	100	*	TYPE=SUBSTITUTE
CM032588.1	318778	.	G	T	100	*	TYPE=SUBSTITUTE
CM032588.1	318779	.	T	A	100	*	TYPE=SUBSTITUTE

CM032588.1	318782	.	T	G	100	*	TYPE=SUBSTITUTE
CM032588.1	318812	.	TAAGA	T	100	*	TYPE=DELETE
CM032588.1	318819	.	T	G	100	*	TYPE=SUBSTITUTE
CM032588.1	318824	.	A	G	100	*	TYPE=SUBSTITUTE
CM032588.1	318825	.	G	A	100	*	TYPE=SUBSTITUTE
CM032588.1	318856	.	C	T	100	*	TYPE=SUBSTITUTE
CM032588.1	318864	.	A	G	100	*	TYPE=SUBSTITUTE
CM032588.1	318888	.	C	T	100	*	TYPE=SUBSTITUTE
CM032588.1	318895	.	T	C	100	*	TYPE=SUBSTITUTE
CM032588.1	318899	.	T	G	100	*	TYPE=SUBSTITUTE
CM032588.1	318900	.	C	T	100	*	TYPE=SUBSTITUTE
CM032588.1	318909	.	C	G	100	*	TYPE=SUBSTITUTE
CM032588.1	318910	.	C	T	100	*	TYPE=SUBSTITUTE
CM032588.1	318940	.	A	G	100	*	TYPE=SUBSTITUTE
CM032588.1	318942	.	C	T	100	*	TYPE=SUBSTITUTE
CM032588.1	318944	.	G	C	100	*	TYPE=SUBSTITUTE
CM032588.1	318946	.	G	A	100	*	TYPE=SUBSTITUTE
CM032588.1	318947	.	T	C	100	*	TYPE=SUBSTITUTE
CM032588.1	318951	.	C	T	100	*	TYPE=SUBSTITUTE
CM032588.1	318955	.	G	T	100	*	TYPE=SUBSTITUTE
CM032588.1	318963	.	G	GAC	100	*	TYPE=INSERT
CM032588.1	318965	.	A	G	100	*	TYPE=SUBSTITUTE
CM032588.1	318973	.	A	G	100	*	TYPE=SUBSTITUTE
CM032588.1	318984	.	A	G	100	*	TYPE=SUBSTITUTE
CM032588.1	318985	.	AAG	A	100	*	TYPE=DELETE
CM032588.1	318988	.	A	T	100	*	TYPE=SUBSTITUTE
CM032588.1	318992	.	A	G	100	*	TYPE=SUBSTITUTE
CM032588.1	318993	.	G	T	100	*	TYPE=SUBSTITUTE
CM032588.1	318996	.	T	C	100	*	TYPE=SUBSTITUTE
CM032588.1	319002	.	T	C	100	*	TYPE=SUBSTITUTE
CM032588.1	319003	.	G	A	100	*	TYPE=SUBSTITUTE
CM032588.1	319007	.	A	C	100	*	TYPE=SUBSTITUTE
CM032588.1	319010	.	G	T	100	*	TYPE=SUBSTITUTE
CM032588.1	319012	.	T	C	100	*	TYPE=SUBSTITUTE
CM032588.1	319015	.	C	T	100	*	TYPE=SUBSTITUTE
CM032588.1	319016	.	T	A	100	*	TYPE=SUBSTITUTE
CM032588.1	319019	.	G	T	100	*	TYPE=SUBSTITUTE
CM032588.1	319021	.	A	G	100	*	TYPE=SUBSTITUTE
CM032588.1	319023	.	C	T	100	*	TYPE=SUBSTITUTE
CM032588.1	319024	.	ACT	A	100	*	TYPE=DELETE
CM032588.1	319027	.	C	T	100	*	TYPE=SUBSTITUTE
CM032588.1	319029	.	T	G	100	*	TYPE=SUBSTITUTE
CM032588.1	319034	.	A	G	100	*	TYPE=SUBSTITUTE
CM032588.1	319038	.	G	A	100	*	TYPE=SUBSTITUTE
CM032588.1	319039	.	A	T	100	*	TYPE=SUBSTITUTE
CM032588.1	319040	.	G	A	100	*	TYPE=SUBSTITUTE
CM032588.1	319043	.	T	TG	100	*	TYPE=INSERT
CM032588.1	319047	.	G	A	100	*	TYPE=SUBSTITUTE
CM032588.1	319049	.	G	t	100	*	TYPE=SUBSTITUTE
CM032588.1	319053	.	G	a	100	*	TYPE=SUBSTITUTE
CM032588.1	319056	.	GATAT	G	100	*	TYPE=DELETE
CM032588.1	319062	.	A	g	100	*	TYPE=SUBSTITUTE
CM032588.1	319064	.	A	g	100	*	TYPE=SUBSTITUTE
CM032588.1	319070	.	ATG	A	100	*	TYPE=DELETE
CM032588.1	319075	.	A	g	100	*	TYPE=SUBSTITUTE
CM032588.1	319081	.	G	a	100	*	TYPE=SUBSTITUTE
CM032588.1	319083	.	G	a	100	*	TYPE=SUBSTITUTE
CM032588.1	319089	.	A	G	100	*	TYPE=SUBSTITUTE
CM032588.1	319090	.	GCTAC	G	100	*	TYPE=DELETE
CM032588.1	319096	.	T	A	100	*	TYPE=SUBSTITUTE
CM032588.1	319100	.	GAT	G	100	*	TYPE=DELETE
CM032588.1	319107	.	A	G	100	*	TYPE=SUBSTITUTE
CM032588.1	319109	.	T	C	100	*	TYPE=SUBSTITUTE
CM032588.1	319110	.	T	C	100	*	TYPE=SUBSTITUTE

CM032588.1	319130	.	T	G	100	*	TYPE=SUBSTITUTE
CM032588.1	319132	.	G	A	100	*	TYPE=SUBSTITUTE
CM032588.1	319133	.	C	G	100	*	TYPE=SUBSTITUTE
CM032588.1	319135	.	A	G	100	*	TYPE=SUBSTITUTE
CM032588.1	319138	.	G	C	100	*	TYPE=SUBSTITUTE
CM032588.1	319140	.	CAAAG	C	100	*	TYPE=DELETE
CM032588.1	319145	.	C	G	100	*	TYPE=SUBSTITUTE
CM032588.1	319147	.	T	G	100	*	TYPE=SUBSTITUTE
CM032588.1	319148	.	C	T	100	*	TYPE=SUBSTITUTE
CM032588.1	319151	.	T	C	100	*	TYPE=SUBSTITUTE
CM032588.1	319170	.	CAAGAGTGCAG	C	100	*	TYPE=DELETE
CM032588.1	319181	.	A	T	100	*	TYPE=SUBSTITUTE
CM032588.1	319184	.	A	G	100	*	TYPE=SUBSTITUTE
CM032588.1	319190	.	A	G	100	*	TYPE=SUBSTITUTE
CM032588.1	319191	.	TTA	T	100	*	TYPE=DELETE
CM032588.1	319200	.	TA	T	100	*	TYPE=DELETE
CM032588.1	319204	.	CAT	C	100	*	TYPE=DELETE
CM032588.1	319209	.	TCTGATGC	T	100	*	TYPE=DELETE
CM032588.1	319217	.	G	A	100	*	TYPE=SUBSTITUTE
CM032588.1	319218	.	A	G	100	*	TYPE=SUBSTITUTE
CM032588.1	319219	.	C	T	100	*	TYPE=SUBSTITUTE
CM032588.1	319235	.	G	A	100	*	TYPE=SUBSTITUTE
CM032588.1	319239	.	CT	C	100	*	TYPE=DELETE
CM032588.1	319244	.	A	C	100	*	TYPE=SUBSTITUTE
CM032588.1	319246	.	G	GC	100	*	TYPE=INSERT
CM032588.1	319247	.	T	A	100	*	TYPE=SUBSTITUTE
CM032588.1	319250	.	C	T	100	*	TYPE=SUBSTITUTE
CM032588.1	319251	.	T	C	100	*	TYPE=SUBSTITUTE
CM032588.1	319252	.	T	G	100	*	TYPE=SUBSTITUTE
CM032588.1	319257	.	A	G	100	*	TYPE=SUBSTITUTE
CM032588.1	319261	.	C	T	100	*	TYPE=SUBSTITUTE
CM032588.1	319270	.	A	G	100	*	TYPE=SUBSTITUTE
CM032588.1	319274	.	C	T	100	*	TYPE=SUBSTITUTE
CM032588.1	319280	.	T	C	100	*	TYPE=SUBSTITUTE
CM032588.1	319282	.	G	T	100	*	TYPE=SUBSTITUTE
CM032588.1	319284	.	G	GAGAAGTTTCATGA	100	*	TYPE=INSERT
CM032588.1	319292	.	C	T	100	*	TYPE=SUBSTITUTE
CM032588.1	319297	.	A	G	100	*	TYPE=SUBSTITUTE
CM032588.1	319299	.	G	C	100	*	TYPE=SUBSTITUTE
CM032588.1	319309	.	A	G	100	*	TYPE=SUBSTITUTE
CM032588.1	319310	.	G	A	100	*	TYPE=SUBSTITUTE
CM032588.1	319319	.	A	AC	100	*	TYPE=INSERT
CM032588.1	319323	.	T	A	100	*	TYPE=SUBSTITUTE
CM032588.1	319324	.	C	T	100	*	TYPE=SUBSTITUTE
CM032588.1	319334	.	G	A	100	*	TYPE=SUBSTITUTE
CM032588.1	319335	.	A	G	100	*	TYPE=SUBSTITUTE
CM032588.1	319339	.	G	T	100	*	TYPE=SUBSTITUTE
CM032588.1	319357	.	C	A	100	*	TYPE=SUBSTITUTE
CM032588.1	319359	.	A	T	100	*	TYPE=SUBSTITUTE
CM032588.1	319360	.	C	G	100	*	TYPE=SUBSTITUTE
CM032588.1	319361	.	A	G	100	*	TYPE=SUBSTITUTE
CM032588.1	319362	.	C	A	100	*	TYPE=SUBSTITUTE
CM032588.1	319364	.	T	A	100	*	TYPE=SUBSTITUTE
CM032588.1	319369	.	T	C	100	*	TYPE=SUBSTITUTE
CM032588.1	319374	.	G	A	100	*	TYPE=SUBSTITUTE
CM032588.1	319376	.	A	T	100	*	TYPE=SUBSTITUTE
CM032588.1	319377	.	G	A	100	*	TYPE=SUBSTITUTE
CM032588.1	319382	.	T	C	100	*	TYPE=SUBSTITUTE
CM032588.1	319386	.	T	C	100	*	TYPE=SUBSTITUTE
CM032588.1	319389	.	C	CT	100	*	TYPE=INSERT
CM032588.1	319390	.	C	T	100	*	TYPE=SUBSTITUTE
CM032588.1	319392	.	A	T	100	*	TYPE=SUBSTITUTE
CM032588.1	319395	.	G	A	100	*	TYPE=SUBSTITUTE
CM032588.1	319401	.	T	G	100	*	TYPE=SUBSTITUTE

CM032588.1	319406	.	AG	A	100	*	TYPE=DELETE
CM032588.1	319413	.	G	A	100	*	TYPE=SUBSTITUTE
CM032588.1	319418	.	G	A	100	*	TYPE=SUBSTITUTE
CM032588.1	319421	.	G	T	100	*	TYPE=SUBSTITUTE
CM032588.1	319426	.	C	G	100	*	TYPE=SUBSTITUTE
CM032588.1	319427	.	C	G	100	*	TYPE=SUBSTITUTE
CM032588.1	319432	.	AGAG	A	100	*	TYPE=DELETE
CM032588.1	319439	.	GTTCCATTTCCATATTATATATAATATAGTAC	G	100	*	TYPE=DELETE
CM032588.1	319471	.	T	C	100	*	TYPE=SUBSTITUTE
CM032588.1	319487	.	T	C	100	*	TYPE=SUBSTITUTE
CM032588.1	319496	.	C	T	100	*	TYPE=SUBSTITUTE
CM032588.1	319500	.	C	T	100	*	TYPE=SUBSTITUTE
CM032588.1	319524	.	C	T	100	*	TYPE=SUBSTITUTE
CM032588.1	319558	.	G	GG	100	*	TYPE=INSERT
CM032588.1	319664	.	C	G	100	*	TYPE=SUBSTITUTE
CM032588.1	319665	.	A	ATGTT	100	*	TYPE=INSERT
CM032588.1	319691	.	C	A	100	*	TYPE=SUBSTITUTE
CM032588.1	319692	.	T	C	100	*	TYPE=SUBSTITUTE
CM032588.1	319694	.	T	C	100	*	TYPE=SUBSTITUTE
CM032588.1	319736	.	T	C	100	*	TYPE=SUBSTITUTE
CM032588.1	319743	.	C	T	100	*	TYPE=SUBSTITUTE
CM032588.1	319744	.	C	T	100	*	TYPE=SUBSTITUTE
CM032588.1	319745	.	T	G	100	*	TYPE=SUBSTITUTE
CM032588.1	319759	.	C	T	100	*	TYPE=SUBSTITUTE
CM032588.1	319765	.	A	T	100	*	TYPE=SUBSTITUTE
CM032588.1	319767	.	G	C	100	*	TYPE=SUBSTITUTE
CM032588.1	319770	.	C	T	100	*	TYPE=SUBSTITUTE
CM032588.1	319778	.	G	A	100	*	TYPE=SUBSTITUTE
CM032588.1	319784	.	A	T	100	*	TYPE=SUBSTITUTE
CM032588.1	319785	.	A	T	100	*	TYPE=SUBSTITUTE
CM032588.1	319788	.	A	AT	100	*	TYPE=INSERT
CM032588.1	319804	.	C	T	100	*	TYPE=SUBSTITUTE
CM032588.1	319806	.	A	T	100	*	TYPE=SUBSTITUTE
CM032588.1	319810	.	T	TC	100	*	TYPE=INSERT
CM032588.1	319811	.	T	C	100	*	TYPE=SUBSTITUTE
CM032588.1	319814	.	C	A	100	*	TYPE=SUBSTITUTE
CM032588.1	319815	.	A	T	100	*	TYPE=SUBSTITUTE
CM032588.1	319818	.	A	G	100	*	TYPE=SUBSTITUTE
CM032588.1	319828	.	G	A	100	*	TYPE=SUBSTITUTE
CM032588.1	319830	.	C	T	100	*	TYPE=SUBSTITUTE
CM032588.1	319833	.	G	C	100	*	TYPE=SUBSTITUTE
CM032588.1	319860	.	G	GGATTTAACC	100	*	TYPE=INSERT
CM032588.1	319864	.	C	CT	100	*	TYPE=INSERT
CM032588.1	319871	.	G	A	100	*	TYPE=SUBSTITUTE
CM032588.1	319874	.	C	G	100	*	TYPE=SUBSTITUTE
CM032588.1	319877	.	A	C	100	*	TYPE=SUBSTITUTE
CM032588.1	319881	.	G	C	100	*	TYPE=SUBSTITUTE
CM032588.1	319890	.	C	A	100	*	TYPE=SUBSTITUTE
CM032588.1	319896	.	C	T	100	*	TYPE=SUBSTITUTE
CM032588.1	319904	.	C	A	100	*	TYPE=SUBSTITUTE
CM032588.1	319941	.	A	T	100	*	TYPE=SUBSTITUTE
CM032588.1	319953	.	T	c	100	*	TYPE=SUBSTITUTE
CM032588.1	319965	.	A	G	100	*	TYPE=SUBSTITUTE
CM032588.1	319972	.	T	C	100	*	TYPE=SUBSTITUTE
CM032588.1	319982	.	C	CTAG	100	*	TYPE=INSERT
CM032588.1	319983	.	A	C	100	*	TYPE=SUBSTITUTE
CM032588.1	319987	.	G	A	100	*	TYPE=SUBSTITUTE
CM032588.1	319993	.	A	T	100	*	TYPE=SUBSTITUTE
CM032588.1	319998	.	A	G	100	*	TYPE=SUBSTITUTE
CM032588.1	320004	.	G	A	100	*	TYPE=SUBSTITUTE
CM032588.1	320011	.	G	A	100	*	TYPE=SUBSTITUTE
CM032588.1	320012	.	G	C	100	*	TYPE=SUBSTITUTE
CM032588.1	320013	.	C	T	100	*	TYPE=SUBSTITUTE

CM032588.1	320019	.	C	T	100	*	TYPE=SUBSTITUTE
CM032588.1	320034	.	T	C	100	*	TYPE=SUBSTITUTE
CM032588.1	320040	.	C	T	100	*	TYPE=SUBSTITUTE
CM032588.1	320057	.	T	C	100	*	TYPE=SUBSTITUTE
CM032588.1	320064	.	G	C	100	*	TYPE=SUBSTITUTE
CM032588.1	320070	.	A	T	100	*	TYPE=SUBSTITUTE
CM032588.1	320076	.	A	G	100	*	TYPE=SUBSTITUTE
CM032588.1	320085	.	G	A	100	*	TYPE=SUBSTITUTE
CM032588.1	320133	.	C	T	100	*	TYPE=SUBSTITUTE
CM032588.1	320136	.	A	T	100	*	TYPE=SUBSTITUTE
CM032588.1	320155	.	C	T	100	*	TYPE=SUBSTITUTE
CM032588.1	320166	.	C	A	100	*	TYPE=SUBSTITUTE
CM032588.1	320196	.	C	T	100	*	TYPE=SUBSTITUTE
CM032588.1	320199	.	T	A	100	*	TYPE=SUBSTITUTE
CM032588.1	320202	.	G	C	100	*	TYPE=SUBSTITUTE
CM032588.1	320226	.	A	C	100	*	TYPE=SUBSTITUTE
CM032588.1	320231	.	T	A	100	*	TYPE=SUBSTITUTE
CM032588.1	320260	.	A	G	100	*	TYPE=SUBSTITUTE
CM032588.1	320265	.	T	C	100	*	TYPE=SUBSTITUTE
CM032588.1	320287	.	T	C	100	*	TYPE=SUBSTITUTE
CM032588.1	320294	.	T	C	100	*	TYPE=SUBSTITUTE
CM032588.1	320305	.	A	G	100	*	TYPE=SUBSTITUTE
CM032588.1	320317	.	A	G	100	*	TYPE=SUBSTITUTE
CM032588.1	320325	.	T	C	100	*	TYPE=SUBSTITUTE
CM032588.1	320327	.	A	G	100	*	TYPE=SUBSTITUTE
CM032588.1	320332	.	T	C	100	*	TYPE=SUBSTITUTE
CM032588.1	320334	.	T	C	100	*	TYPE=SUBSTITUTE
CM032588.1	320337	.	A	C	100	*	TYPE=SUBSTITUTE
CM032588.1	320339	.	A	G	100	*	TYPE=SUBSTITUTE
CM032588.1	320343	.	A	T	100	*	TYPE=SUBSTITUTE
CM032588.1	320345	.	A	G	100	*	TYPE=SUBSTITUTE
CM032588.1	320348	.	A	G	100	*	TYPE=SUBSTITUTE
CM032588.1	320379	.	G	T	100	*	TYPE=SUBSTITUTE
CM032588.1	320380	.	A	T	100	*	TYPE=SUBSTITUTE
CM032588.1	320384	.	T	C	100	*	TYPE=SUBSTITUTE
CM032588.1	320395	.	C	G	100	*	TYPE=SUBSTITUTE
CM032588.1	320399	.	A	T	100	*	TYPE=SUBSTITUTE
CM032588.1	320405	.	C	T	100	*	TYPE=SUBSTITUTE
CM032588.1	320423	.	G	A	100	*	TYPE=SUBSTITUTE
CM032588.1	320429	.	GCA	G	100	*	TYPE=DELETE
CM032588.1	320432	.	G	T	100	*	TYPE=SUBSTITUTE
CM032588.1	320441	.	T	C	100	*	TYPE=SUBSTITUTE
CM032588.1	320444	.	G	A	100	*	TYPE=SUBSTITUTE
CM032588.1	320453	.	C	T	100	*	TYPE=SUBSTITUTE
CM032588.1	320459	.	C	T	100	*	TYPE=SUBSTITUTE
CM032588.1	320460	.	T	C	100	*	TYPE=SUBSTITUTE
CM032588.1	320465	.	G	A	100	*	TYPE=SUBSTITUTE
CM032588.1	320471	.	C	A	100	*	TYPE=SUBSTITUTE
CM032588.1	320474	.	G	T	100	*	TYPE=SUBSTITUTE
CM032588.1	320489	.	G	A	100	*	TYPE=SUBSTITUTE
CM032588.1	320490	.	T	C	100	*	TYPE=SUBSTITUTE
CM032588.1	320510	.	G	A	100	*	TYPE=SUBSTITUTE
CM032588.1	320512	.	A	T	100	*	TYPE=SUBSTITUTE
CM032588.1	320524	.	C	G	100	*	TYPE=SUBSTITUTE
CM032588.1	320532	.	G	C	100	*	TYPE=SUBSTITUTE
CM032588.1	320552	.	A	G	100	*	TYPE=SUBSTITUTE
CM032588.1	320560	.	A	AN	100	*	TYPE=INSERT
CM032588.1	320574	.	T	C	100	*	TYPE=SUBSTITUTE
CM032588.1	320578	.	T	C	100	*	TYPE=SUBSTITUTE
CM032588.1	320585	.	T	A	100	*	TYPE=SUBSTITUTE
CM032588.1	320586	.	A	G	100	*	TYPE=SUBSTITUTE
CM032588.1	320591	.	G	C	100	*	TYPE=SUBSTITUTE
CM032588.1	320596	.	C	T	100	*	TYPE=SUBSTITUTE
CM032588.1	320597	.	C	T	100	*	TYPE=SUBSTITUTE

CM032588.1	320598	.	C	T	100	*	TYPE=SUBSTITUTE
CM032588.1	320599	.	A	G	100	*	TYPE=SUBSTITUTE
CM032588.1	320601	.	G	T	100	*	TYPE=SUBSTITUTE
CM032588.1	320603	.	G	A	100	*	TYPE=SUBSTITUTE
CM032588.1	320611	.	T	G	100	*	TYPE=SUBSTITUTE
CM032588.1	320612	.	A	G	100	*	TYPE=SUBSTITUTE
CM032588.1	320613	.	C	T	100	*	TYPE=SUBSTITUTE
CM032588.1	320628	.	G	A	100	*	TYPE=SUBSTITUTE
CM032588.1	320650	.	C	G	100	*	TYPE=SUBSTITUTE
CM032588.1	320675	.	A	G	100	*	TYPE=SUBSTITUTE
CM032588.1	320681	.	G	T	100	*	TYPE=SUBSTITUTE
CM032588.1	320694	.	A	G	100	*	TYPE=SUBSTITUTE
CM032588.1	320695	.	C	T	100	*	TYPE=SUBSTITUTE
CM032588.1	320704	.	T	C	100	*	TYPE=SUBSTITUTE
CM032588.1	320719	.	A	T	100	*	TYPE=SUBSTITUTE
CM032588.1	320722	.	C	T	100	*	TYPE=SUBSTITUTE
CM032588.1	320755	.	T	C	100	*	TYPE=SUBSTITUTE
CM032588.1	320756	.	A	T	100	*	TYPE=SUBSTITUTE
CM032588.1	320765	.	CCATGGCTCTATTCA	C	100	*	TYPE=DELETE
CM032588.1	320786	.	G	A	100	*	TYPE=SUBSTITUTE
CM032588.1	320790	.	TC	T	100	*	TYPE=DELETE
CM032588.1	320792	.	A	G	100	*	TYPE=SUBSTITUTE
CM032588.1	320808	.	C	G	100	*	TYPE=SUBSTITUTE
CM032588.1	320812	.	C	T	100	*	TYPE=SUBSTITUTE
CM032588.1	320824	.	C	T	100	*	TYPE=SUBSTITUTE
CM032588.1	320827	.	G	A	100	*	TYPE=SUBSTITUTE
CM032588.1	320830	.	A	G	100	*	TYPE=SUBSTITUTE
CM032588.1	320833	.	G	A	100	*	TYPE=SUBSTITUTE
CM032588.1	320836	.	G	C	100	*	TYPE=SUBSTITUTE
CM032588.1	320857	.	C	T	100	*	TYPE=SUBSTITUTE
CM032588.1	320893	.	A	G	100	*	TYPE=SUBSTITUTE
CM032588.1	320911	.	T	A	100	*	TYPE=SUBSTITUTE
CM032588.1	320929	.	AAGTCACTTGTCAA	A	100	*	TYPE=DELETE
CM032588.1	320944	.	C	G	100	*	TYPE=SUBSTITUTE
CM032588.1	320951	.	T	C	100	*	TYPE=SUBSTITUTE
CM032588.1	320952	.	C	T	100	*	TYPE=SUBSTITUTE
CM032588.1	320955	.	G	A	100	*	TYPE=SUBSTITUTE
CM032588.1	320962	.	C	T	100	*	TYPE=SUBSTITUTE
CM032588.1	320964	.	T	c	100	*	TYPE=SUBSTITUTE
CM032588.1	320966	.	C	a	100	*	TYPE=SUBSTITUTE
CM032588.1	320967	.	A	g	100	*	TYPE=SUBSTITUTE
CM032588.1	320973	.	g	gaaga	100	*	TYPE=INSERT
CM032588.1	320975	.	T	c	100	*	TYPE=SUBSTITUTE
CM032588.1	320980	.	A	G	100	*	TYPE=SUBSTITUTE
CM032588.1	320983	.	A	G	100	*	TYPE=SUBSTITUTE
CM032588.1	320989	.	C	A	100	*	TYPE=SUBSTITUTE
CM032588.1	320990	.	G	A	100	*	TYPE=SUBSTITUTE
CM032588.1	320992	.	TAG	T	100	*	TYPE=DELETE
CM032588.1	321048	.	T	C	100	*	TYPE=SUBSTITUTE
CM032588.1	321070	.	G	A	100	*	TYPE=SUBSTITUTE
CM032588.1	321552	.	T	C	100	*	TYPE=SUBSTITUTE
CM032588.1	321600	.	CC	C	100	*	TYPE=DELETE
CM032588.1	321640	.	C	G	100	*	TYPE=SUBSTITUTE
CM032588.1	321651	.	A	G	100	*	TYPE=SUBSTITUTE
CM032588.1	321807	.	A	G	100	*	TYPE=SUBSTITUTE
CM032588.1	321876	.	G	T	100	*	TYPE=SUBSTITUTE
CM032588.1	321957	.	G	C	100	*	TYPE=SUBSTITUTE
CM032588.1	322418	.	TTA	T	100	*	TYPE=DELETE
CM032588.1	322706	.	C	A	100	*	TYPE=SUBSTITUTE
CM032588.1	322762	.	C	T	100	*	TYPE=SUBSTITUTE
CM032588.1	322908	.	A	C	100	*	TYPE=SUBSTITUTE
CM032588.1	322970	.	T	C	100	*	TYPE=SUBSTITUTE
CM032588.1	322972	.	A	G	100	*	TYPE=SUBSTITUTE
CM032588.1	322978	.	C	T	100	*	TYPE=SUBSTITUTE

CM032588.1	322996	.	A	G	100	*	TYPE=SUBSTITUTE
CM032588.1	322998	.	A	G	100	*	TYPE=SUBSTITUTE
CM032588.1	323017	.	C	G	100	*	TYPE=SUBSTITUTE
CM032588.1	323026	.	T	G	100	*	TYPE=SUBSTITUTE
CM032588.1	323029	.	C	T	100	*	TYPE=SUBSTITUTE
CM032588.1	323044	.	A	T	100	*	TYPE=SUBSTITUTE
CM032588.1	323056	.	G	A	100	*	TYPE=SUBSTITUTE
CM032588.1	323080	.	A	G	100	*	TYPE=SUBSTITUTE
CM032588.1	323095	.	G	A	100	*	TYPE=SUBSTITUTE
CM032588.1	323113	.	G	A	100	*	TYPE=SUBSTITUTE
CM032588.1	323179	.	C	T	100	*	TYPE=SUBSTITUTE
CM032588.1	323230	.	A	g	100	*	TYPE=SUBSTITUTE
CM032588.1	323246	.	A	g	100	*	TYPE=SUBSTITUTE
CM032588.1	323247	.	C	t	100	*	TYPE=SUBSTITUTE
CM032588.1	323281	.	A	T	100	*	TYPE=SUBSTITUTE
CM032588.1	323282	.	G	A	100	*	TYPE=SUBSTITUTE
CM032588.1	323283	.	A	C	100	*	TYPE=SUBSTITUTE
CM032588.1	323296	.	C	T	100	*	TYPE=SUBSTITUTE
CM032588.1	323312	.	G	C	100	*	TYPE=SUBSTITUTE
CM032588.1	323329	.	A	G	100	*	TYPE=SUBSTITUTE
CM032588.1	323367	.	T	TAAATGTACCTAGT	100	*	TYPE=INSERT
CM032588.1	323368	.	T	A	100	*	TYPE=SUBSTITUTE
CM032588.1	323382	.	A	g	100	*	TYPE=SUBSTITUTE
CM032588.1	323385	.	C	t	100	*	TYPE=SUBSTITUTE
CM032588.1	323397	.	T	C	100	*	TYPE=SUBSTITUTE
CM032588.1	323405	.	T	A	100	*	TYPE=SUBSTITUTE
CM032588.1	323410	.	G	T	100	*	TYPE=SUBSTITUTE
CM032588.1	323412	.	A	G	100	*	TYPE=SUBSTITUTE
CM032588.1	323413	.	T	C	100	*	TYPE=SUBSTITUTE
CM032588.1	323417	.	T	C	100	*	TYPE=SUBSTITUTE
CM032588.1	323418	.	A	G	100	*	TYPE=SUBSTITUTE
CM032588.1	323426	.	A	G	100	*	TYPE=SUBSTITUTE
CM032588.1	323429	.	A	T	100	*	TYPE=SUBSTITUTE
CM032588.1	323450	.	T	C	100	*	TYPE=SUBSTITUTE
CM032588.1	323452	.	C	A	100	*	TYPE=SUBSTITUTE
CM032588.1	323453	.	A	G	100	*	TYPE=SUBSTITUTE
CM032588.1	323484	.	C	T	100	*	TYPE=SUBSTITUTE
CM032588.1	323486	.	C	T	100	*	TYPE=SUBSTITUTE
CM032588.1	323496	.	T	C	100	*	TYPE=SUBSTITUTE
CM032588.1	323497	.	C	T	100	*	TYPE=SUBSTITUTE
CM032588.1	323498	.	G	C	100	*	TYPE=SUBSTITUTE
CM032588.1	323511	.	T	C	100	*	TYPE=SUBSTITUTE
CM032588.1	323530	.	G	T	100	*	TYPE=SUBSTITUTE
CM032588.1	323540	.	T	C	100	*	TYPE=SUBSTITUTE
CM032588.1	323543	.	T	G	100	*	TYPE=SUBSTITUTE
CM032588.1	323572	.	A	T	100	*	TYPE=SUBSTITUTE
CM032588.1	323577	.	A	G	100	*	TYPE=SUBSTITUTE
CM032588.1	323579	.	T	A	100	*	TYPE=SUBSTITUTE
CM032588.1	323581	.	C	T	100	*	TYPE=SUBSTITUTE
CM032588.1	323603	.	G	A	100	*	TYPE=SUBSTITUTE
CM032588.1	323615	.	C	A	100	*	TYPE=SUBSTITUTE
CM032588.1	323619	.	C	A	100	*	TYPE=SUBSTITUTE
CM032588.1	323622	.	C	A	100	*	TYPE=SUBSTITUTE
CM032588.1	323623	.	T	A	100	*	TYPE=SUBSTITUTE
CM032588.1	323632	.	G	A	100	*	TYPE=SUBSTITUTE
CM032588.1	323633	.	T	G	100	*	TYPE=SUBSTITUTE
CM032588.1	323654	.	G	A	100	*	TYPE=SUBSTITUTE
CM032588.1	323659	.	C	A	100	*	TYPE=SUBSTITUTE
CM032588.1	323664	.	C	T	100	*	TYPE=SUBSTITUTE
CM032588.1	323685	.	C	T	100	*	TYPE=SUBSTITUTE
CM032588.1	323703	.	T	C	100	*	TYPE=SUBSTITUTE
CM032588.1	323713	.	C	T	100	*	TYPE=SUBSTITUTE
CM032588.1	323745	.	C	T	100	*	TYPE=SUBSTITUTE
CM032588.1	323792	.	C	T	100	*	TYPE=SUBSTITUTE

CM032588.1	323794	.	G	T	100	*	TYPE=SUBSTITUTE
CM032588.1	323857	.	A	G	100	*	TYPE=SUBSTITUTE
CM032588.1	323859	.	A	G	100	*	TYPE=SUBSTITUTE
CM032588.1	323869	.	A	G	100	*	TYPE=SUBSTITUTE
CM032588.1	323882	.	C	A	100	*	TYPE=SUBSTITUTE
CM032588.1	323901	.	G	T	100	*	TYPE=SUBSTITUTE
CM032588.1	323917	.	G	C	100	*	TYPE=SUBSTITUTE
CM032588.1	323923	.	C	T	100	*	TYPE=SUBSTITUTE
CM032588.1	323935	.	A	G	100	*	TYPE=SUBSTITUTE
CM032588.1	323945	.	G	A	100	*	TYPE=SUBSTITUTE
CM032588.1	323950	.	C	T	100	*	TYPE=SUBSTITUTE
CM032588.1	323991	.	C	T	100	*	TYPE=SUBSTITUTE
CM032588.1	324022	.	C	T	100	*	TYPE=SUBSTITUTE
CM032588.1	324063	.	A	g	100	*	TYPE=SUBSTITUTE
CM032588.1	324070	.	C	A	100	*	TYPE=SUBSTITUTE
CM032588.1	324081	.	C	T	100	*	TYPE=SUBSTITUTE
CM032588.1	324119	.	G	A	100	*	TYPE=SUBSTITUTE
CM032588.1	324124	.	T	g	100	*	TYPE=SUBSTITUTE
CM032588.1	324126	.	G	a	100	*	TYPE=SUBSTITUTE
CM032588.1	324129	.	T	c	100	*	TYPE=SUBSTITUTE
CM032588.1	324138	.	C	t	100	*	TYPE=SUBSTITUTE
CM032588.1	324143	.	A	G	100	*	TYPE=SUBSTITUTE
CM032588.1	324144	.	G	A	100	*	TYPE=SUBSTITUTE
CM032588.1	324148	.	G	A	100	*	TYPE=SUBSTITUTE
CM032588.1	324170	.	G	A	100	*	TYPE=SUBSTITUTE
CM032588.1	324175	.	A	G	100	*	TYPE=SUBSTITUTE
CM032588.1	324192	.	G	T	100	*	TYPE=SUBSTITUTE
CM032588.1	324193	.	A	C	100	*	TYPE=SUBSTITUTE
CM032588.1	324226	.	G	A	100	*	TYPE=SUBSTITUTE
CM032588.1	324229	.	G	A	100	*	TYPE=SUBSTITUTE
CM032588.1	324253	.	G	A	100	*	TYPE=SUBSTITUTE
CM032588.1	324275	.	GT	G	100	*	TYPE=DELETE
CM032588.1	324277	.	A	G	100	*	TYPE=SUBSTITUTE
CM032588.1	324293	.	C	T	100	*	TYPE=SUBSTITUTE
CM032588.1	324294	.	A	T	100	*	TYPE=SUBSTITUTE
CM032588.1	324301	.	C	G	100	*	TYPE=SUBSTITUTE
CM032588.1	324302	.	T	C	100	*	TYPE=SUBSTITUTE
CM032588.1	324311	.	A	G	100	*	TYPE=SUBSTITUTE
CM032588.1	324312	.	G	A	100	*	TYPE=SUBSTITUTE
CM032588.1	324313	.	C	T	100	*	TYPE=SUBSTITUTE
CM032588.1	324314	.	G	C	100	*	TYPE=SUBSTITUTE
CM032588.1	324315	.	G	A	100	*	TYPE=SUBSTITUTE
CM032588.1	324335	.	A	C	100	*	TYPE=SUBSTITUTE
CM032588.1	324350	.	T	C	100	*	TYPE=SUBSTITUTE
CM032588.1	324368	.	G	A	100	*	TYPE=SUBSTITUTE
CM032588.1	324380	.	A	G	100	*	TYPE=SUBSTITUTE
CM032588.1	324389	.	G	A	100	*	TYPE=SUBSTITUTE
CM032588.1	324422	.	G	A	100	*	TYPE=SUBSTITUTE
CM032588.1	324424	.	C	T	100	*	TYPE=SUBSTITUTE
CM032588.1	324431	.	T	C	100	*	TYPE=SUBSTITUTE
CM032588.1	324437	.	T	C	100	*	TYPE=SUBSTITUTE
CM032588.1	324440	.	G	A	100	*	TYPE=SUBSTITUTE
CM032588.1	324444	.	C	T	100	*	TYPE=SUBSTITUTE
CM032588.1	324455	.	T	C	100	*	TYPE=SUBSTITUTE
CM032588.1	324467	.	T	C	100	*	TYPE=SUBSTITUTE
CM032588.1	324493	.	T	C	100	*	TYPE=SUBSTITUTE
CM032588.1	324503	.	A	G	100	*	TYPE=SUBSTITUTE
CM032588.1	324543	.	A	G	100	*	TYPE=SUBSTITUTE
CM032588.1	324547	.	A	G	100	*	TYPE=SUBSTITUTE
CM032588.1	324554	.	A	G	100	*	TYPE=SUBSTITUTE
CM032588.1	324563	.	G	A	100	*	TYPE=SUBSTITUTE
CM032588.1	324578	.	A	G	100	*	TYPE=SUBSTITUTE
CM032588.1	324583	.	G	T	100	*	TYPE=SUBSTITUTE
CM032588.1	324599	.	A	G	100	*	TYPE=SUBSTITUTE

CM032588.1	324602	.	T	G	100	*	TYPE=SUBSTITUTE
CM032588.1	324603	.	C	G	100	*	TYPE=SUBSTITUTE
CM032588.1	324608	.	A	G	100	*	TYPE=SUBSTITUTE
CM032588.1	324610	.	C	T	100	*	TYPE=SUBSTITUTE
CM032588.1	324627	.	A	G	100	*	TYPE=SUBSTITUTE
CM032588.1	324698	.	C	T	100	*	TYPE=SUBSTITUTE
CM032588.1	324707	.	T	C	100	*	TYPE=SUBSTITUTE
CM032588.1	324710	.	G	A	100	*	TYPE=SUBSTITUTE
CM032588.1	324737	.	A	C	100	*	TYPE=SUBSTITUTE
CM032588.1	324751	.	G	A	100	*	TYPE=SUBSTITUTE
CM032588.1	324761	.	T	G	100	*	TYPE=SUBSTITUTE
CM032588.1	324764	.	A	G	100	*	TYPE=SUBSTITUTE
CM032588.1	324778	.	T	C	100	*	TYPE=SUBSTITUTE
CM032588.1	324784	.	A	G	100	*	TYPE=SUBSTITUTE
CM032588.1	324797	.	G	C	100	*	TYPE=SUBSTITUTE
CM032588.1	324799	.	T	C	100	*	TYPE=SUBSTITUTE
CM032588.1	324803	.	A	G	100	*	TYPE=SUBSTITUTE
CM032588.1	324806	.	G	A	100	*	TYPE=SUBSTITUTE
CM032588.1	324810	.	G	A	100	*	TYPE=SUBSTITUTE
CM032588.1	324811	.	A	T	100	*	TYPE=SUBSTITUTE
CM032588.1	324813	.	C	T	100	*	TYPE=SUBSTITUTE
CM032588.1	324815	.	A	G	100	*	TYPE=SUBSTITUTE
CM032588.1	324829	.	C	T	100	*	TYPE=SUBSTITUTE
CM032588.1	324835	.	T	TA	100	*	TYPE=INSERT
CM032588.1	324836	.	T	A	100	*	TYPE=SUBSTITUTE
CM032588.1	324847	.	C	T	100	*	TYPE=SUBSTITUTE
CM032588.1	324848	.	G	A	100	*	TYPE=SUBSTITUTE
CM032588.1	324852	.	T	C	100	*	TYPE=SUBSTITUTE
CM032588.1	324856	.	G	T	100	*	TYPE=SUBSTITUTE
CM032588.1	324859	.	T	TA	100	*	TYPE=INSERT
CM032588.1	324864	.	G	C	100	*	TYPE=SUBSTITUTE
CM032588.1	324865	.	T	C	100	*	TYPE=SUBSTITUTE
CM032588.1	324867	.	A	C	100	*	TYPE=SUBSTITUTE
CM032588.1	324882	.	A	G	100	*	TYPE=SUBSTITUTE
CM032588.1	324893	.	T	c	100	*	TYPE=SUBSTITUTE
CM032588.1	324900	.	C	a	100	*	TYPE=SUBSTITUTE
CM032588.1	324905	.	A	g	100	*	TYPE=SUBSTITUTE
CM032588.1	324906	.	C	a	100	*	TYPE=SUBSTITUTE
CM032588.1	324915	.	G	A	100	*	TYPE=SUBSTITUTE
CM032588.1	324934	.	C	T	100	*	TYPE=SUBSTITUTE
CM032588.1	324949	.	T	G	100	*	TYPE=SUBSTITUTE
CM032588.1	324953	.	T	C	100	*	TYPE=SUBSTITUTE
CM032588.1	324956	.	G	A	100	*	TYPE=SUBSTITUTE
CM032588.1	324961	.	T	C	100	*	TYPE=SUBSTITUTE
CM032588.1	324977	.	t	ta	100	*	TYPE=INSERT
CM032588.1	324981	.	A	g	100	*	TYPE=SUBSTITUTE
CM032588.1	324992	.	G	A	100	*	TYPE=SUBSTITUTE
CM032588.1	324993	.	C	G	100	*	TYPE=SUBSTITUTE
CM032588.1	325007	.	G	C	100	*	TYPE=SUBSTITUTE
CM032588.1	325011	.	T	C	100	*	TYPE=SUBSTITUTE
CM032588.1	325016	.	T	C	100	*	TYPE=SUBSTITUTE
CM032588.1	325018	.	A	G	100	*	TYPE=SUBSTITUTE
CM032588.1	325032	.	T	A	100	*	TYPE=SUBSTITUTE
CM032588.1	325036	.	A	C	100	*	TYPE=SUBSTITUTE
CM032588.1	325041	.	C	A	100	*	TYPE=SUBSTITUTE
CM032588.1	325048	.	C	G	100	*	TYPE=SUBSTITUTE
CM032588.1	325051	.	T	A	100	*	TYPE=SUBSTITUTE
CM032588.1	325053	.	C	G	100	*	TYPE=SUBSTITUTE
CM032588.1	325056	.	T	C	100	*	TYPE=SUBSTITUTE
CM032588.1	325073	.	T	C	100	*	TYPE=SUBSTITUTE
CM032588.1	325087	.	G	A	100	*	TYPE=SUBSTITUTE
CM032588.1	325101	.	T	C	100	*	TYPE=SUBSTITUTE
CM032588.1	325114	.	C	G	100	*	TYPE=SUBSTITUTE
CM032588.1	325130	.	A	T	100	*	TYPE=SUBSTITUTE

CM032588.1	325133	.	CCATGTA	C	100	*	TYPE=DELETE
CM032588.1	325143	.	G	T	100	*	TYPE=SUBSTITUTE
CM032588.1	325156	.	G	A	100	*	TYPE=SUBSTITUTE
CM032588.1	325157	.	A	G	100	*	TYPE=SUBSTITUTE
CM032588.1	325185	.	C	T	100	*	TYPE=SUBSTITUTE
CM032588.1	325197	.	T	A	100	*	TYPE=SUBSTITUTE
CM032588.1	325224	.	C	T	100	*	TYPE=SUBSTITUTE
CM032588.1	325228	.	G	A	100	*	TYPE=SUBSTITUTE
CM032588.1	325229	.	G	T	100	*	TYPE=SUBSTITUTE
CM032588.1	325233	.	T	A	100	*	TYPE=SUBSTITUTE
CM032588.1	325238	.	G	A	100	*	TYPE=SUBSTITUTE
CM032588.1	325260	.	C	T	100	*	TYPE=SUBSTITUTE
CM032588.1	325283	.	t	ttt	100	*	TYPE=INSERT
CM032588.1	325286	.	A	t	100	*	TYPE=SUBSTITUTE
CM032588.1	325287	.	T	a	100	*	TYPE=SUBSTITUTE
CM032588.1	325305	.	C	A	100	*	TYPE=SUBSTITUTE
CM032588.1	325313	.	T	G	100	*	TYPE=SUBSTITUTE
CM032588.1	325327	.	T	C	100	*	TYPE=SUBSTITUTE
CM032588.1	325338	.	C	T	100	*	TYPE=SUBSTITUTE
CM032588.1	325341	.	A	G	100	*	TYPE=SUBSTITUTE
CM032588.1	325348	.	T	C	100	*	TYPE=SUBSTITUTE
CM032588.1	325349	.	A	G	100	*	TYPE=SUBSTITUTE
CM032588.1	325376	.	AA	A	100	*	TYPE=DELETE
CM032588.1	325396	.	A	C	100	*	TYPE=SUBSTITUTE
CM032588.1	325397	.	T	G	100	*	TYPE=SUBSTITUTE
CM032588.1	325438	.	T	C	100	*	TYPE=SUBSTITUTE
CM032588.1	325441	.	C	G	100	*	TYPE=SUBSTITUTE
CM032588.1	325451	.	C	T	100	*	TYPE=SUBSTITUTE
CM032588.1	325456	.	C	T	100	*	TYPE=SUBSTITUTE
CM032588.1	325459	.	A	G	100	*	TYPE=SUBSTITUTE
CM032588.1	325466	.	C	A	100	*	TYPE=SUBSTITUTE
CM032588.1	325476	.	C	A	100	*	TYPE=SUBSTITUTE
CM032588.1	325480	.	T	A	100	*	TYPE=SUBSTITUTE
CM032588.1	325488	.	A	G	100	*	TYPE=SUBSTITUTE
CM032588.1	325492	.	G	GA	100	*	TYPE=INSERT
CM032588.1	325502	.	A	T	100	*	TYPE=SUBSTITUTE
CM032588.1	325509	.	A	G	100	*	TYPE=SUBSTITUTE
CM032588.1	325512	.	A	T	100	*	TYPE=SUBSTITUTE
CM032588.1	325529	.	T	C	100	*	TYPE=SUBSTITUTE
CM032588.1	325540	.	A	T	100	*	TYPE=SUBSTITUTE
CM032588.1	325550	.	G	T	100	*	TYPE=SUBSTITUTE
CM032588.1	325559	.	T	A	100	*	TYPE=SUBSTITUTE
CM032588.1	325560	.	A	G	100	*	TYPE=SUBSTITUTE
CM032588.1	325568	.	A	T	100	*	TYPE=SUBSTITUTE
CM032588.1	325572	.	G	A	100	*	TYPE=SUBSTITUTE
CM032588.1	325577	.	A	G	100	*	TYPE=SUBSTITUTE
CM032588.1	325584	.	G	C	100	*	TYPE=SUBSTITUTE
CM032588.1	325593	.	C	A	100	*	TYPE=SUBSTITUTE
CM032588.1	325606	.	T	A	100	*	TYPE=SUBSTITUTE
CM032588.1	325608	.	T	G	100	*	TYPE=SUBSTITUTE
CM032588.1	325610	.	G	A	100	*	TYPE=SUBSTITUTE
CM032588.1	325618	.	T	C	100	*	TYPE=SUBSTITUTE
CM032588.1	325627	.	A	G	100	*	TYPE=SUBSTITUTE
CM032588.1	325634	.	A	G	100	*	TYPE=SUBSTITUTE
CM032588.1	325638	.	T	C	100	*	TYPE=SUBSTITUTE
CM032588.1	325674	.	T	C	100	*	TYPE=SUBSTITUTE
CM032588.1	325688	.	T	C	100	*	TYPE=SUBSTITUTE
CM032588.1	325702	.	T	A	100	*	TYPE=SUBSTITUTE
CM032588.1	325704	.	G	A	100	*	TYPE=SUBSTITUTE
CM032588.1	325709	.	G	A	100	*	TYPE=SUBSTITUTE
CM032588.1	325718	.	C	A	100	*	TYPE=SUBSTITUTE
CM032588.1	325737	.	T	C	100	*	TYPE=SUBSTITUTE
CM032588.1	325743	.	G	C	100	*	TYPE=SUBSTITUTE
CM032588.1	325744	.	G	C	100	*	TYPE=SUBSTITUTE

CM032588.1	325746	.	G	A	100	*	TYPE=SUBSTITUTE
CM032588.1	325762	.	C	T	100	*	TYPE=SUBSTITUTE
CM032588.1	325768	.	C	T	100	*	TYPE=SUBSTITUTE
CM032588.1	325824	.	T	C	100	*	TYPE=SUBSTITUTE
CM032588.1	325844	.	A	T	100	*	TYPE=SUBSTITUTE
CM032588.1	325902	.	A	C	100	*	TYPE=SUBSTITUTE
CM032588.1	325919	.	T	C	100	*	TYPE=SUBSTITUTE
CM032588.1	325920	.	G	A	100	*	TYPE=SUBSTITUTE
CM032588.1	325932	.	A	G	100	*	TYPE=SUBSTITUTE
CM032588.1	325940	.	G	A	100	*	TYPE=SUBSTITUTE
CM032588.1	325990	.	T	A	100	*	TYPE=SUBSTITUTE
CM032588.1	326014	.	C	G	100	*	TYPE=SUBSTITUTE
CM032588.1	326044	.	A	C	100	*	TYPE=SUBSTITUTE
CM032588.1	326047	.	T	C	100	*	TYPE=SUBSTITUTE
CM032588.1	326048	.	C	G	100	*	TYPE=SUBSTITUTE
CM032588.1	326055	.	T	C	100	*	TYPE=SUBSTITUTE
CM032588.1	326071	.	C	A	100	*	TYPE=SUBSTITUTE
CM032588.1	326080	.	T	C	100	*	TYPE=SUBSTITUTE
CM032588.1	326081	.	T	A	100	*	TYPE=SUBSTITUTE
CM032588.1	326084	.	G	A	100	*	TYPE=SUBSTITUTE
CM032588.1	326088	.	C	T	100	*	TYPE=SUBSTITUTE
CM032588.1	326093	.	G	T	100	*	TYPE=SUBSTITUTE
CM032588.1	326099	.	A	G	100	*	TYPE=SUBSTITUTE
CM032588.1	326112	.	C	T	100	*	TYPE=SUBSTITUTE
CM032588.1	326120	.	C	T	100	*	TYPE=SUBSTITUTE
CM032588.1	326134	.	C	T	100	*	TYPE=SUBSTITUTE
CM032588.1	326151	.	G	C	100	*	TYPE=SUBSTITUTE
CM032588.1	326189	.	T	C	100	*	TYPE=SUBSTITUTE
CM032588.1	326192	.	T	C	100	*	TYPE=SUBSTITUTE
CM032588.1	326222	.	G	A	100	*	TYPE=SUBSTITUTE
CM032588.1	326257	.	G	T	100	*	TYPE=SUBSTITUTE
CM032588.1	326264	.	C	T	100	*	TYPE=SUBSTITUTE
CM032588.1	326276	.	T	C	100	*	TYPE=SUBSTITUTE
CM032588.1	326296	.	G	A	100	*	TYPE=SUBSTITUTE
CM032588.1	326303	.	G	T	100	*	TYPE=SUBSTITUTE
CM032588.1	326315	.	A	G	100	*	TYPE=SUBSTITUTE
CM032588.1	326329	.	A	G	100	*	TYPE=SUBSTITUTE
CM032588.1	326330	.	G	A	100	*	TYPE=SUBSTITUTE
CM032588.1	326351	.	G	A	100	*	TYPE=SUBSTITUTE
CM032588.1	326369	.	T	G	100	*	TYPE=SUBSTITUTE
CM032588.1	326407	.	A	G	100	*	TYPE=SUBSTITUTE
CM032588.1	326410	.	T	C	100	*	TYPE=SUBSTITUTE
CM032588.1	326471	.	T	C	100	*	TYPE=SUBSTITUTE
CM032588.1	326492	.	G	A	100	*	TYPE=SUBSTITUTE
CM032588.1	326513	.	C	T	100	*	TYPE=SUBSTITUTE
CM032588.1	326522	.	T	G	100	*	TYPE=SUBSTITUTE
CM032588.1	326528	.	C	A	100	*	TYPE=SUBSTITUTE
CM032588.1	326537	.	C	G	100	*	TYPE=SUBSTITUTE
CM032588.1	326540	.	G	T	100	*	TYPE=SUBSTITUTE
CM032588.1	326543	.	A	C	100	*	TYPE=SUBSTITUTE
CM032588.1	326555	.	C	T	100	*	TYPE=SUBSTITUTE
CM032588.1	326567	.	C	T	100	*	TYPE=SUBSTITUTE
CM032588.1	326585	.	G	A	100	*	TYPE=SUBSTITUTE
CM032588.1	326611	.	A	G	100	*	TYPE=SUBSTITUTE
CM032588.1	326651	.	G	A	100	*	TYPE=SUBSTITUTE
CM032588.1	326666	.	T	G	100	*	TYPE=SUBSTITUTE
CM032588.1	326690	.	T	C	100	*	TYPE=SUBSTITUTE
CM032588.1	326705	.	A	G	100	*	TYPE=SUBSTITUTE
CM032588.1	326750	.	T	C	100	*	TYPE=SUBSTITUTE
CM032588.1	326768	.	A	G	100	*	TYPE=SUBSTITUTE
CM032588.1	326771	.	G	T	100	*	TYPE=SUBSTITUTE
CM032588.1	326779	.	G	A	100	*	TYPE=SUBSTITUTE
CM032588.1	326795	.	C	T	100	*	TYPE=SUBSTITUTE
CM032588.1	326804	.	A	G	100	*	TYPE=SUBSTITUTE

CM032588.1	326816	.	G	A	100	*	TYPE=SUBSTITUTE
CM032588.1	326915	.	T	C	100	*	TYPE=SUBSTITUTE
CM032588.1	326945	.	T	A	100	*	TYPE=SUBSTITUTE
CM032588.1	326948	.	A	G	100	*	TYPE=SUBSTITUTE
CM032588.1	326954	.	T	C	100	*	TYPE=SUBSTITUTE
CM032588.1	326972	.	T	C	100	*	TYPE=SUBSTITUTE
CM032588.1	327014	.	G	A	100	*	TYPE=SUBSTITUTE
CM032588.1	327017	.	A	G	100	*	TYPE=SUBSTITUTE
CM032588.1	327020	.	C	G	100	*	TYPE=SUBSTITUTE
CM032588.1	327033	.	C	T	100	*	TYPE=SUBSTITUTE
CM032588.1	327034	.	A	T	100	*	TYPE=SUBSTITUTE
CM032588.1	327037	.	C	T	100	*	TYPE=SUBSTITUTE
CM032588.1	327046	.	G	A	100	*	TYPE=SUBSTITUTE
CM032588.1	327065	.	G	T	100	*	TYPE=SUBSTITUTE
CM032588.1	327092	.	C	T	100	*	TYPE=SUBSTITUTE
CM032588.1	327112	.	T	G	100	*	TYPE=SUBSTITUTE
CM032588.1	327113	.	A	G	100	*	TYPE=SUBSTITUTE
CM032588.1	327140	.	T	G	100	*	TYPE=SUBSTITUTE
CM032588.1	327149	.	A	G	100	*	TYPE=SUBSTITUTE
CM032588.1	327155	.	G	A	100	*	TYPE=SUBSTITUTE
CM032588.1	327191	.	A	T	100	*	TYPE=SUBSTITUTE
CM032588.1	327194	.	T	A	100	*	TYPE=SUBSTITUTE
CM032588.1	327206	.	C	t	100	*	TYPE=SUBSTITUTE
CM032588.1	327215	.	A	g	100	*	TYPE=SUBSTITUTE
CM032588.1	327223	.	C	A	100	*	TYPE=SUBSTITUTE
CM032588.1	327224	.	C	A	100	*	TYPE=SUBSTITUTE
CM032588.1	327233	.	A	G	100	*	TYPE=SUBSTITUTE
CM032588.1	327245	.	A	T	100	*	TYPE=SUBSTITUTE
CM032588.1	327254	.	T	G	100	*	TYPE=SUBSTITUTE
CM032588.1	327280	.	A	G	100	*	TYPE=SUBSTITUTE
CM032588.1	327281	.	T	C	100	*	TYPE=SUBSTITUTE
CM032588.1	327284	.	C	T	100	*	TYPE=SUBSTITUTE
CM032588.1	327287	.	G	A	100	*	TYPE=SUBSTITUTE
CM032588.1	327302	.	G	A	100	*	TYPE=SUBSTITUTE
CM032588.1	327323	.	A	C	100	*	TYPE=SUBSTITUTE
CM032588.1	327326	.	G	T	100	*	TYPE=SUBSTITUTE
CM032588.1	327328	.	T	C	100	*	TYPE=SUBSTITUTE
CM032588.1	327329	.	A	G	100	*	TYPE=SUBSTITUTE
CM032588.1	327373	.	G	A	100	*	TYPE=SUBSTITUTE
CM032588.1	327374	.	T	A	100	*	TYPE=SUBSTITUTE
CM032588.1	327395	.	G	A	100	*	TYPE=SUBSTITUTE
CM032588.1	327404	.	T	A	100	*	TYPE=SUBSTITUTE
CM032588.1	327406	.	A	G	100	*	TYPE=SUBSTITUTE
CM032588.1	327413	.	T	C	100	*	TYPE=SUBSTITUTE
CM032588.1	327415	.	T	A	100	*	TYPE=SUBSTITUTE
CM032588.1	327430	.	C	T	100	*	TYPE=SUBSTITUTE
CM032588.1	327431	.	G	A	100	*	TYPE=SUBSTITUTE
CM032588.1	327443	.	G	A	100	*	TYPE=SUBSTITUTE
CM032588.1	327455	.	C	A	100	*	TYPE=SUBSTITUTE
CM032588.1	327464	.	G	A	100	*	TYPE=SUBSTITUTE
CM032588.1	327467	.	T	G	100	*	TYPE=SUBSTITUTE
CM032588.1	327482	.	G	A	100	*	TYPE=SUBSTITUTE
CM032588.1	327487	.	G	A	100	*	TYPE=SUBSTITUTE
CM032588.1	327488	.	G	A	100	*	TYPE=SUBSTITUTE
CM032588.1	327491	.	T	C	100	*	TYPE=SUBSTITUTE
CM032588.1	327512	.	C	T	100	*	TYPE=SUBSTITUTE
CM032588.1	327515	.	T	C	100	*	TYPE=SUBSTITUTE
CM032588.1	327518	.	G	A	100	*	TYPE=SUBSTITUTE
CM032588.1	327557	.	T	G	100	*	TYPE=SUBSTITUTE
CM032588.1	327560	.	G	A	100	*	TYPE=SUBSTITUTE
CM032588.1	327587	.	G	A	100	*	TYPE=SUBSTITUTE
CM032588.1	327590	.	C	T	100	*	TYPE=SUBSTITUTE
CM032588.1	327596	.	G	A	100	*	TYPE=SUBSTITUTE
CM032588.1	327613	.	A	G	100	*	TYPE=SUBSTITUTE

CM032588.1	327617	.	C	A	100	*	TYPE=SUBSTITUTE
CM032588.1	327626	.	T	C	100	*	TYPE=SUBSTITUTE
CM032588.1	327629	.	T	C	100	*	TYPE=SUBSTITUTE
CM032588.1	327635	.	T	C	100	*	TYPE=SUBSTITUTE
CM032588.1	327641	.	G	A	100	*	TYPE=SUBSTITUTE
CM032588.1	327647	.	C	T	100	*	TYPE=SUBSTITUTE
CM032588.1	327671	.	G	A	100	*	TYPE=SUBSTITUTE
CM032588.1	327701	.	A	G	100	*	TYPE=SUBSTITUTE
CM032588.1	327715	.	G	A	100	*	TYPE=SUBSTITUTE
CM032588.1	327740	.	T	C	100	*	TYPE=SUBSTITUTE
CM032588.1	327743	.	C	T	100	*	TYPE=SUBSTITUTE
CM032588.1	327779	.	G	A	100	*	TYPE=SUBSTITUTE
CM032588.1	327794	.	T	C	100	*	TYPE=SUBSTITUTE
CM032588.1	327869	.	T	C	100	*	TYPE=SUBSTITUTE
CM032588.1	327872	.	C	T	100	*	TYPE=SUBSTITUTE
CM032588.1	327888	.	C	T	100	*	TYPE=SUBSTITUTE
CM032588.1	327892	.	A	G	100	*	TYPE=SUBSTITUTE
CM032588.1	327897	.	A	G	100	*	TYPE=SUBSTITUTE
CM032588.1	327905	.	C	A	100	*	TYPE=SUBSTITUTE
CM032588.1	327919	.	C	T	100	*	TYPE=SUBSTITUTE
CM032588.1	327923	.	T	C	100	*	TYPE=SUBSTITUTE
CM032588.1	327928	.	G	T	100	*	TYPE=SUBSTITUTE
CM032588.1	327939	.	C	T	100	*	TYPE=SUBSTITUTE
CM032588.1	327946	.	A	T	100	*	TYPE=SUBSTITUTE
CM032588.1	327955	.	G	A	100	*	TYPE=SUBSTITUTE
CM032588.1	327973	.	G	A	100	*	TYPE=SUBSTITUTE
CM032588.1	327983	.	C	G	100	*	TYPE=SUBSTITUTE
CM032588.1	328001	.	A	G	100	*	TYPE=SUBSTITUTE
CM032588.1	328021	.	C	T	100	*	TYPE=SUBSTITUTE
CM032588.1	328097	.	G	A	100	*	TYPE=SUBSTITUTE
CM032588.1	328100	.	T	C	100	*	TYPE=SUBSTITUTE
CM032588.1	328109	.	A	G	100	*	TYPE=SUBSTITUTE
CM032588.1	328112	.	G	A	100	*	TYPE=SUBSTITUTE
CM032588.1	328186	.	T	C	100	*	TYPE=SUBSTITUTE
CM032588.1	328188	.	G	A	100	*	TYPE=SUBSTITUTE
CM032588.1	328220	.	A	c	100	*	TYPE=SUBSTITUTE
CM032588.1	328226	.	T	c	100	*	TYPE=SUBSTITUTE
CM032588.1	328235	.	A	G	100	*	TYPE=SUBSTITUTE
CM032588.1	328236	.	C	T	100	*	TYPE=SUBSTITUTE
CM032588.1	328238	.	C	T	100	*	TYPE=SUBSTITUTE
CM032588.1	328244	.	C	T	100	*	TYPE=SUBSTITUTE
CM032588.1	328259	.	G	A	100	*	TYPE=SUBSTITUTE
CM032588.1	328265	.	T	C	100	*	TYPE=SUBSTITUTE
CM032588.1	328280	.	C	T	100	*	TYPE=SUBSTITUTE
CM032588.1	328292	.	G	T	100	*	TYPE=SUBSTITUTE
CM032588.1	328293	.	G	A	100	*	TYPE=SUBSTITUTE
CM032588.1	328295	.	T	C	100	*	TYPE=SUBSTITUTE
CM032588.1	328302	.	T	G	100	*	TYPE=SUBSTITUTE
CM032588.1	328303	.	T	C	100	*	TYPE=SUBSTITUTE
CM032588.1	328314	.	C	G	100	*	TYPE=SUBSTITUTE
CM032588.1	328315	.	C	T	100	*	TYPE=SUBSTITUTE
CM032588.1	328318	.	C	T	100	*	TYPE=SUBSTITUTE
CM032588.1	328327	.	T	C	100	*	TYPE=SUBSTITUTE
CM032588.1	328329	.	A	G	100	*	TYPE=SUBSTITUTE
CM032588.1	328331	.	G	A	100	*	TYPE=SUBSTITUTE
CM032588.1	328343	.	G	A	100	*	TYPE=SUBSTITUTE
CM032588.1	328361	.	T	C	100	*	TYPE=SUBSTITUTE
CM032588.1	328362	.	G	A	100	*	TYPE=SUBSTITUTE
CM032588.1	328363	.	C	T	100	*	TYPE=SUBSTITUTE
CM032588.1	328370	.	G	T	100	*	TYPE=SUBSTITUTE
CM032588.1	328371	.	G	T	100	*	TYPE=SUBSTITUTE
CM032588.1	328373	.	A	G	100	*	TYPE=SUBSTITUTE
CM032588.1	328376	.	G	A	100	*	TYPE=SUBSTITUTE
CM032588.1	328382	.	G	T	100	*	TYPE=SUBSTITUTE

CM032588.1	328394	.	G	A	100	*	TYPE=SUBSTITUTE
CM032588.1	328412	.	G	A	100	*	TYPE=SUBSTITUTE
CM032588.1	328415	.	T	G	100	*	TYPE=SUBSTITUTE
CM032588.1	328417	.	C	T	100	*	TYPE=SUBSTITUTE
CM032588.1	328460	.	G	a	100	*	TYPE=SUBSTITUTE
CM032588.1	328464	.	A	g	100	*	TYPE=SUBSTITUTE
CM032588.1	328465	.	C	g	100	*	TYPE=SUBSTITUTE
CM032588.1	328466	.	G	a	100	*	TYPE=SUBSTITUTE
CM032588.1	328485	.	G	A	100	*	TYPE=SUBSTITUTE
CM032588.1	328499	.	T	G	100	*	TYPE=SUBSTITUTE
CM032588.1	328514	.	G	A	100	*	TYPE=SUBSTITUTE
CM032588.1	328520	.	A	G	100	*	TYPE=SUBSTITUTE
CM032588.1	328532	.	T	C	100	*	TYPE=SUBSTITUTE
CM032588.1	328541	.	G	A	100	*	TYPE=SUBSTITUTE
CM032588.1	328559	.	G	A	100	*	TYPE=SUBSTITUTE
CM032588.1	328566	.	A	C	100	*	TYPE=SUBSTITUTE
CM032588.1	328584	.	T	C	100	*	TYPE=SUBSTITUTE
CM032588.1	328589	.	A	G	100	*	TYPE=SUBSTITUTE
CM032588.1	328593	.	A	AAG	100	*	TYPE=INSERT
CM032588.1	328595	.	T	C	100	*	TYPE=SUBSTITUTE
CM032588.1	328614	.	G	A	100	*	TYPE=SUBSTITUTE
CM032588.1	328616	.	A	G	100	*	TYPE=SUBSTITUTE
CM032588.1	328654	.	A	G	100	*	TYPE=SUBSTITUTE
CM032588.1	328681	.	TC	T	100	*	TYPE=DELETE
CM032588.1	328686	.	G	A	100	*	TYPE=SUBSTITUTE
CM032588.1	328703	.	G	C	100	*	TYPE=SUBSTITUTE
CM032588.1	328705	.	T	C	100	*	TYPE=SUBSTITUTE
CM032588.1	328710	.	T	G	100	*	TYPE=SUBSTITUTE
CM032588.1	328714	.	T	C	100	*	TYPE=SUBSTITUTE
CM032588.1	328715	.	A	G	100	*	TYPE=SUBSTITUTE
CM032588.1	328737	.	A	G	100	*	TYPE=SUBSTITUTE
CM032588.1	328863	.	ACA	A	100	*	TYPE=DELETE
CM032588.1	328919	.	G	A	100	*	TYPE=SUBSTITUTE
CM032588.1	328922	.	T	C	100	*	TYPE=SUBSTITUTE
CM032588.1	328967	.	G	A	100	*	TYPE=SUBSTITUTE
CM032588.1	328979	.	C	T	100	*	TYPE=SUBSTITUTE
CM032588.1	329010	.	T	C	100	*	TYPE=SUBSTITUTE
CM032588.1	329019	.	T	C	100	*	TYPE=SUBSTITUTE
CM032588.1	329040	.	TCACTAGAG	T	100	*	TYPE=DELETE
CM032588.1	329052	.	T	G	100	*	TYPE=SUBSTITUTE
CM032588.1	329053	.	A	G	100	*	TYPE=SUBSTITUTE
CM032588.1	329086	.	T	G	100	*	TYPE=SUBSTITUTE
CM032588.1	329089	.	A	G	100	*	TYPE=SUBSTITUTE
CM032588.1	329093	.	A	G	100	*	TYPE=SUBSTITUTE
CM032588.1	329112	.	A	G	100	*	TYPE=SUBSTITUTE
CM032588.1	329113	.	G	A	100	*	TYPE=SUBSTITUTE
CM032588.1	329125	.	A	G	100	*	TYPE=SUBSTITUTE
CM032588.1	329184	.	T	C	100	*	TYPE=SUBSTITUTE
CM032588.1	329190	.	C	G	100	*	TYPE=SUBSTITUTE
CM032588.1	329195	.	T	G	100	*	TYPE=SUBSTITUTE
CM032588.1	329198	.	A	C	100	*	TYPE=SUBSTITUTE
CM032588.1	329202	.	C	A	100	*	TYPE=SUBSTITUTE
CM032588.1	329203	.	A	G	100	*	TYPE=SUBSTITUTE
CM032588.1	329207	.	C	A	100	*	TYPE=SUBSTITUTE
CM032588.1	329211	.	T	G	100	*	TYPE=SUBSTITUTE
CM032588.1	329232	.	G	A	100	*	TYPE=SUBSTITUTE
CM032588.1	329233	.	A	G	100	*	TYPE=SUBSTITUTE
CM032588.1	329237	.	C	A	100	*	TYPE=SUBSTITUTE
CM032588.1	329245	.	C	T	100	*	TYPE=SUBSTITUTE
CM032588.1	329254	.	T	C	100	*	TYPE=SUBSTITUTE
CM032588.1	329255	.	G	A	100	*	TYPE=SUBSTITUTE
CM032588.1	329277	.	G	T	100	*	TYPE=SUBSTITUTE
CM032588.1	329280	.	G	A	100	*	TYPE=SUBSTITUTE
CM032588.1	329295	.	C	G	100	*	TYPE=SUBSTITUTE

CM032588.1	329297	.	A	C	100	*	TYPE=SUBSTITUTE
CM032588.1	329316	.	A	AGAAAAAGATGATTTCATGTTT	100	*	TYPE=INSERT
CM032588.1	329317	.	A	G	100	*	TYPE=SUBSTITUTE
CM032588.1	329318	.	C	A	100	*	TYPE=SUBSTITUTE
CM032588.1	329321	.	A	G	100	*	TYPE=SUBSTITUTE
CM032588.1	329330	.	T	C	100	*	TYPE=SUBSTITUTE
CM032588.1	329331	.	G	A	100	*	TYPE=SUBSTITUTE
CM032588.1	329342	.	C	T	100	*	TYPE=SUBSTITUTE
CM032588.1	329351	.	G	A	100	*	TYPE=SUBSTITUTE
CM032588.1	329352	.	T	C	100	*	TYPE=SUBSTITUTE
CM032588.1	329354	.	G	A	100	*	TYPE=SUBSTITUTE
CM032588.1	329359	.	C	A	100	*	TYPE=SUBSTITUTE
CM032588.1	329362	.	C	G	100	*	TYPE=SUBSTITUTE
CM032588.1	329367	.	A	T	100	*	TYPE=SUBSTITUTE
CM032588.1	329386	.	T	C	100	*	TYPE=SUBSTITUTE
CM032588.1	329417	.	A	G	100	*	TYPE=SUBSTITUTE
CM032588.1	329426	.	T	C	100	*	TYPE=SUBSTITUTE
CM032588.1	329483	.	A	g	100	*	TYPE=SUBSTITUTE
CM032588.1	329498	.	G	t	100	*	TYPE=SUBSTITUTE
CM032588.1	329501	.	C	T	100	*	TYPE=SUBSTITUTE
CM032588.1	329507	.	C	T	100	*	TYPE=SUBSTITUTE
CM032588.1	329510	.	T	C	100	*	TYPE=SUBSTITUTE
CM032588.1	329522	.	T	G	100	*	TYPE=SUBSTITUTE
CM032588.1	329531	.	C	T	100	*	TYPE=SUBSTITUTE
CM032588.1	329534	.	C	T	100	*	TYPE=SUBSTITUTE
CM032588.1	329555	.	T	C	100	*	TYPE=SUBSTITUTE
CM032588.1	329564	.	G	A	100	*	TYPE=SUBSTITUTE
CM032588.1	329627	.	A	c	100	*	TYPE=SUBSTITUTE
CM032588.1	329638	.	A	C	100	*	TYPE=SUBSTITUTE
CM032588.1	329660	.	T	C	100	*	TYPE=SUBSTITUTE
CM032588.1	329663	.	C	A	100	*	TYPE=SUBSTITUTE
CM032588.1	329714	.	G	a	100	*	TYPE=SUBSTITUTE
CM032588.1	329723	.	G	A	100	*	TYPE=SUBSTITUTE
CM032588.1	329744	.	T	C	100	*	TYPE=SUBSTITUTE
CM032588.1	329774	.	A	G	100	*	TYPE=SUBSTITUTE
CM032588.1	329783	.	C	T	100	*	TYPE=SUBSTITUTE
CM032588.1	329808	.	G	T	100	*	TYPE=SUBSTITUTE
CM032588.1	329809	.	T	C	100	*	TYPE=SUBSTITUTE
CM032588.1	329852	.	T	C	100	*	TYPE=SUBSTITUTE
CM032588.1	329885	.	C	t	100	*	TYPE=SUBSTITUTE
CM032588.1	329897	.	A	g	100	*	TYPE=SUBSTITUTE
CM032588.1	329924	.	G	A	100	*	TYPE=SUBSTITUTE
CM032588.1	329933	.	A	G	100	*	TYPE=SUBSTITUTE
CM032588.1	329963	.	G	A	100	*	TYPE=SUBSTITUTE
CM032588.1	329987	.	G	A	100	*	TYPE=SUBSTITUTE
CM032588.1	330029	.	G	A	100	*	TYPE=SUBSTITUTE
CM032588.1	330035	.	A	C	100	*	TYPE=SUBSTITUTE
CM032588.1	330038	.	A	G	100	*	TYPE=SUBSTITUTE
CM032588.1	330062	.	T	A	100	*	TYPE=SUBSTITUTE
CM032588.1	330080	.	C	T	100	*	TYPE=SUBSTITUTE
CM032588.1	330104	.	A	G	100	*	TYPE=SUBSTITUTE
CM032588.1	330110	.	A	G	100	*	TYPE=SUBSTITUTE
CM032588.1	330113	.	T	C	100	*	TYPE=SUBSTITUTE
CM032588.1	330116	.	A	G	100	*	TYPE=SUBSTITUTE
CM032588.1	330146	.	A	g	100	*	TYPE=SUBSTITUTE
CM032588.1	330160	.	G	A	100	*	TYPE=SUBSTITUTE
CM032588.1	330179	.	C	T	100	*	TYPE=SUBSTITUTE
CM032588.1	330200	.	A	G	100	*	TYPE=SUBSTITUTE
CM032588.1	330212	.	A	G	100	*	TYPE=SUBSTITUTE
CM032588.1	330224	.	G	A	100	*	TYPE=SUBSTITUTE
CM032588.1	330242	.	C	T	100	*	TYPE=SUBSTITUTE
CM032588.1	330254	.	G	T	100	*	TYPE=SUBSTITUTE
CM032588.1	330278	.	A	G	100	*	TYPE=SUBSTITUTE

CM032588.1	330311	.	G	A	100	*	TYPE=SUBSTITUTE
CM032588.1	330314	.	G	A	100	*	TYPE=SUBSTITUTE
CM032588.1	330317	.	G	A	100	*	TYPE=SUBSTITUTE
CM032588.1	330320	.	A	C	100	*	TYPE=SUBSTITUTE
CM032588.1	330323	.	C	T	100	*	TYPE=SUBSTITUTE
CM032588.1	330350	.	C	T	100	*	TYPE=SUBSTITUTE
CM032588.1	330359	.	A	G	100	*	TYPE=SUBSTITUTE
CM032588.1	330362	.	A	G	100	*	TYPE=SUBSTITUTE
CM032588.1	330365	.	T	C	100	*	TYPE=SUBSTITUTE
CM032588.1	330389	.	A	T	100	*	TYPE=SUBSTITUTE
CM032588.1	330410	.	A	G	100	*	TYPE=SUBSTITUTE
CM032588.1	330452	.	G	a	100	*	TYPE=SUBSTITUTE
CM032588.1	330455	.	G	a	100	*	TYPE=SUBSTITUTE
CM032588.1	330490	.	G	C	100	*	TYPE=SUBSTITUTE
CM032588.1	330530	.	G	A	100	*	TYPE=SUBSTITUTE
CM032588.1	330545	.	C	A	100	*	TYPE=SUBSTITUTE
CM032588.1	330548	.	C	T	100	*	TYPE=SUBSTITUTE
CM032588.1	330551	.	C	A	100	*	TYPE=SUBSTITUTE
CM032588.1	330554	.	A	G	100	*	TYPE=SUBSTITUTE
CM032588.1	330563	.	A	g	100	*	TYPE=SUBSTITUTE
CM032588.1	330569	.	A	g	100	*	TYPE=SUBSTITUTE
CM032588.1	330572	.	A	g	100	*	TYPE=SUBSTITUTE
CM032588.1	330622	.	G	A	100	*	TYPE=SUBSTITUTE
CM032588.1	330638	.	A	G	100	*	TYPE=SUBSTITUTE
CM032588.1	330650	.	A	G	100	*	TYPE=SUBSTITUTE
CM032588.1	330653	.	T	C	100	*	TYPE=SUBSTITUTE
CM032588.1	330667	.	T	C	100	*	TYPE=SUBSTITUTE
CM032588.1	330677	.	A	G	100	*	TYPE=SUBSTITUTE
CM032588.1	330689	.	A	G	100	*	TYPE=SUBSTITUTE
CM032588.1	330692	.	T	G	100	*	TYPE=SUBSTITUTE
CM032588.1	330701	.	T	C	100	*	TYPE=SUBSTITUTE
CM032588.1	330704	.	T	C	100	*	TYPE=SUBSTITUTE
CM032588.1	330728	.	G	A	100	*	TYPE=SUBSTITUTE
CM032588.1	330746	.	T	C	100	*	TYPE=SUBSTITUTE
CM032588.1	330806	.	C	T	100	*	TYPE=SUBSTITUTE
CM032588.1	330808	.	A	G	100	*	TYPE=SUBSTITUTE
CM032588.1	330860	.	A	G	100	*	TYPE=SUBSTITUTE
CM032588.1	330893	.	G	A	100	*	TYPE=SUBSTITUTE
CM032588.1	330896	.	G	A	100	*	TYPE=SUBSTITUTE
CM032588.1	330914	.	G	A	100	*	TYPE=SUBSTITUTE
CM032588.1	330923	.	G	A	100	*	TYPE=SUBSTITUTE
CM032588.1	330929	.	A	G	100	*	TYPE=SUBSTITUTE
CM032588.1	330953	.	G	A	100	*	TYPE=SUBSTITUTE
CM032588.1	330983	.	T	G	100	*	TYPE=SUBSTITUTE
CM032588.1	331001	.	C	T	100	*	TYPE=SUBSTITUTE
CM032588.1	331022	.	A	G	100	*	TYPE=SUBSTITUTE
CM032588.1	331043	.	A	g	100	*	TYPE=SUBSTITUTE
CM032588.1	331046	.	A	G	100	*	TYPE=SUBSTITUTE
CM032588.1	331079	.	C	T	100	*	TYPE=SUBSTITUTE
CM032588.1	331086	.	A	G	100	*	TYPE=SUBSTITUTE
CM032588.1	331091	.	G	C	100	*	TYPE=SUBSTITUTE
CM032588.1	331094	.	G	A	100	*	TYPE=SUBSTITUTE
CM032588.1	331106	.	C	T	100	*	TYPE=SUBSTITUTE
CM032588.1	331130	.	A	C	100	*	TYPE=SUBSTITUTE
CM032588.1	331142	.	A	G	100	*	TYPE=SUBSTITUTE
CM032588.1	331148	.	G	A	100	*	TYPE=SUBSTITUTE
CM032588.1	331157	.	G	A	100	*	TYPE=SUBSTITUTE
CM032588.1	331160	.	G	A	100	*	TYPE=SUBSTITUTE
CM032588.1	331163	.	G	A	100	*	TYPE=SUBSTITUTE
CM032588.1	331183	.	G	a	100	*	TYPE=SUBSTITUTE
CM032588.1	331207	.	C	T	100	*	TYPE=SUBSTITUTE
CM032588.1	331214	.	T	C	100	*	TYPE=SUBSTITUTE
CM032588.1	331244	.	G	a	100	*	TYPE=SUBSTITUTE
CM032588.1	331301	.	G	A	100	*	TYPE=SUBSTITUTE

CM032588.1	331313	.	A	G	100	*	TYPE=SUBSTITUTE
CM032588.1	331319	.	T	G	100	*	TYPE=SUBSTITUTE
CM032588.1	331358	.	A	G	100	*	TYPE=SUBSTITUTE
CM032588.1	331364	.	A	C	100	*	TYPE=SUBSTITUTE
CM032588.1	331376	.	G	T	100	*	TYPE=SUBSTITUTE
CM032588.1	331379	.	C	T	100	*	TYPE=SUBSTITUTE
CM032588.1	331384	.	G	T	100	*	TYPE=SUBSTITUTE
CM032588.1	331388	.	C	T	100	*	TYPE=SUBSTITUTE
CM032588.1	331391	.	A	G	100	*	TYPE=SUBSTITUTE
CM032588.1	331412	.	T	C	100	*	TYPE=SUBSTITUTE
CM032588.1	331430	.	T	A	100	*	TYPE=SUBSTITUTE
CM032588.1	331431	.	G	A	100	*	TYPE=SUBSTITUTE
CM032588.1	331453	.	A	G	100	*	TYPE=SUBSTITUTE
CM032588.1	331475	.	G	A	100	*	TYPE=SUBSTITUTE
CM032588.1	331477	.	T	C	100	*	TYPE=SUBSTITUTE
CM032588.1	331478	.	C	G	100	*	TYPE=SUBSTITUTE
CM032588.1	331487	.	A	G	100	*	TYPE=SUBSTITUTE
CM032588.1	331496	.	T	C	100	*	TYPE=SUBSTITUTE
CM032588.1	331499	.	C	T	100	*	TYPE=SUBSTITUTE
CM032588.1	331508	.	G	A	100	*	TYPE=SUBSTITUTE
CM032588.1	331523	.	C	T	100	*	TYPE=SUBSTITUTE
CM032588.1	331537	.	T	C	100	*	TYPE=SUBSTITUTE
CM032588.1	331539	.	A	G	100	*	TYPE=SUBSTITUTE
CM032588.1	331556	.	G	A	100	*	TYPE=SUBSTITUTE
CM032588.1	331592	.	G	C	100	*	TYPE=SUBSTITUTE
CM032588.1	331595	.	T	A	100	*	TYPE=SUBSTITUTE
CM032588.1	331598	.	T	G	100	*	TYPE=SUBSTITUTE
CM032588.1	331601	.	A	C	100	*	TYPE=SUBSTITUTE
CM032588.1	331607	.	T	C	100	*	TYPE=SUBSTITUTE
CM032588.1	331613	.	G	A	100	*	TYPE=SUBSTITUTE
CM032588.1	331628	.	G	A	100	*	TYPE=SUBSTITUTE
CM032588.1	331631	.	T	C	100	*	TYPE=SUBSTITUTE
CM032588.1	331634	.	G	A	100	*	TYPE=SUBSTITUTE
CM032588.1	331664	.	G	A	100	*	TYPE=SUBSTITUTE
CM032588.1	331673	.	T	C	100	*	TYPE=SUBSTITUTE
CM032588.1	331678	.	C	G	100	*	TYPE=SUBSTITUTE
CM032588.1	331679	.	G	A	100	*	TYPE=SUBSTITUTE
CM032588.1	331706	.	T	C	100	*	TYPE=SUBSTITUTE
CM032588.1	331708	.	A	G	100	*	TYPE=SUBSTITUTE
CM032588.1	331721	.	C	T	100	*	TYPE=SUBSTITUTE
CM032588.1	331724	.	G	A	100	*	TYPE=SUBSTITUTE
CM032588.1	331733	.	C	G	100	*	TYPE=SUBSTITUTE
CM032588.1	331736	.	A	G	100	*	TYPE=SUBSTITUTE
CM032588.1	331742	.	C	T	100	*	TYPE=SUBSTITUTE
CM032588.1	331745	.	C	T	100	*	TYPE=SUBSTITUTE
CM032588.1	331748	.	C	T	100	*	TYPE=SUBSTITUTE
CM032588.1	331763	.	T	C	100	*	TYPE=SUBSTITUTE
CM032588.1	331769	.	T	C	100	*	TYPE=SUBSTITUTE
CM032588.1	331793	.	T	A	100	*	TYPE=SUBSTITUTE
CM032588.1	331802	.	A	T	100	*	TYPE=SUBSTITUTE
CM032588.1	331808	.	A	G	100	*	TYPE=SUBSTITUTE
CM032588.1	331832	.	A	G	100	*	TYPE=SUBSTITUTE
CM032588.1	331835	.	G	C	100	*	TYPE=SUBSTITUTE
CM032588.1	331838	.	A	G	100	*	TYPE=SUBSTITUTE
CM032588.1	331844	.	G	A	100	*	TYPE=SUBSTITUTE
CM032588.1	331853	.	A	G	100	*	TYPE=SUBSTITUTE
CM032588.1	331859	.	C	T	100	*	TYPE=SUBSTITUTE
CM032588.1	331866	.	G	A	100	*	TYPE=SUBSTITUTE
CM032588.1	331868	.	T	C	100	*	TYPE=SUBSTITUTE
CM032588.1	331886	.	G	A	100	*	TYPE=SUBSTITUTE
CM032588.1	331907	.	C	T	100	*	TYPE=SUBSTITUTE
CM032588.1	331916	.	A	G	100	*	TYPE=SUBSTITUTE
CM032588.1	331928	.	C	G	100	*	TYPE=SUBSTITUTE
CM032588.1	331931	.	T	C	100	*	TYPE=SUBSTITUTE

CM032588.1	331934	.	C	T	100	*	TYPE=SUBSTITUTE
CM032588.1	331940	.	C	A	100	*	TYPE=SUBSTITUTE
CM032588.1	331943	.	A	G	100	*	TYPE=SUBSTITUTE
CM032588.1	331949	.	T	A	100	*	TYPE=SUBSTITUTE
CM032588.1	331982	.	A	G	100	*	TYPE=SUBSTITUTE
CM032588.1	332015	.	C	G	100	*	TYPE=SUBSTITUTE
CM032588.1	332018	.	T	C	100	*	TYPE=SUBSTITUTE
CM032588.1	332042	.	T	C	100	*	TYPE=SUBSTITUTE
CM032588.1	332048	.	G	A	100	*	TYPE=SUBSTITUTE
CM032588.1	332071	.	T	C	100	*	TYPE=SUBSTITUTE
CM032588.1	332084	.	C	A	100	*	TYPE=SUBSTITUTE
CM032588.1	332093	.	A	G	100	*	TYPE=SUBSTITUTE
CM032588.1	332106	.	A	T	100	*	TYPE=SUBSTITUTE
CM032588.1	332174	.	G	A	100	*	TYPE=SUBSTITUTE
CM032588.1	332182	.	A	G	100	*	TYPE=SUBSTITUTE
CM032588.1	332186	.	C	G	100	*	TYPE=SUBSTITUTE
CM032588.1	332216	.	A	T	100	*	TYPE=SUBSTITUTE
CM032588.1	332249	.	C	A	100	*	TYPE=SUBSTITUTE
CM032588.1	332270	.	T	C	100	*	TYPE=SUBSTITUTE
CM032588.1	332273	.	C	G	100	*	TYPE=SUBSTITUTE
CM032588.1	332282	.	T	G	100	*	TYPE=SUBSTITUTE
CM032588.1	332342	.	G	A	100	*	TYPE=SUBSTITUTE
CM032588.1	332384	.	C	T	100	*	TYPE=SUBSTITUTE
CM032588.1	332387	.	T	A	100	*	TYPE=SUBSTITUTE
CM032588.1	332399	.	T	G	100	*	TYPE=SUBSTITUTE
CM032588.1	332426	.	G	A	100	*	TYPE=SUBSTITUTE
CM032588.1	332432	.	A	G	100	*	TYPE=SUBSTITUTE
CM032588.1	332435	.	C	T	100	*	TYPE=SUBSTITUTE
CM032588.1	332441	.	A	G	100	*	TYPE=SUBSTITUTE
CM032588.1	332465	.	C	T	100	*	TYPE=SUBSTITUTE
CM032588.1	332483	.	G	A	100	*	TYPE=SUBSTITUTE
CM032588.1	332498	.	G	A	100	*	TYPE=SUBSTITUTE
CM032588.1	332510	.	T	C	100	*	TYPE=SUBSTITUTE
CM032588.1	332513	.	A	G	100	*	TYPE=SUBSTITUTE
CM032588.1	332561	.	T	C	100	*	TYPE=SUBSTITUTE
CM032588.1	332564	.	G	T	100	*	TYPE=SUBSTITUTE
CM032588.1	332588	.	T	C	100	*	TYPE=SUBSTITUTE
CM032588.1	332600	.	C	T	100	*	TYPE=SUBSTITUTE
CM032588.1	332603	.	T	G	100	*	TYPE=SUBSTITUTE
CM032588.1	332609	.	G	A	100	*	TYPE=SUBSTITUTE
CM032588.1	332630	.	C	T	100	*	TYPE=SUBSTITUTE
CM032588.1	332640	.	G	T	100	*	TYPE=SUBSTITUTE
CM032588.1	332653	.	G	A	100	*	TYPE=SUBSTITUTE
CM032588.1	332663	.	G	C	100	*	TYPE=SUBSTITUTE
CM032588.1	332678	.	C	G	100	*	TYPE=SUBSTITUTE
CM032588.1	332732	.	T	G	100	*	TYPE=SUBSTITUTE
CM032588.1	332753	.	A	G	100	*	TYPE=SUBSTITUTE
CM032588.1	332776	.	C	A	100	*	TYPE=SUBSTITUTE
CM032588.1	332783	.	A	T	100	*	TYPE=SUBSTITUTE
CM032588.1	332792	.	C	T	100	*	TYPE=SUBSTITUTE
CM032588.1	332795	.	G	C	100	*	TYPE=SUBSTITUTE
CM032588.1	332804	.	A	G	100	*	TYPE=SUBSTITUTE
CM032588.1	332848	.	C	G	100	*	TYPE=SUBSTITUTE
CM032588.1	332858	.	C	A	100	*	TYPE=SUBSTITUTE
CM032588.1	332884	.	A	AAAC	100	*	TYPE=INSERT
CM032588.1	332899	.	A	ATAG	100	*	TYPE=INSERT
CM032588.1	332900	.	C	A	100	*	TYPE=SUBSTITUTE
CM032588.1	332905	.	T	C	100	*	TYPE=SUBSTITUTE
CM032588.1	332908	.	T	C	100	*	TYPE=SUBSTITUTE
CM032588.1	332912	.	G	C	100	*	TYPE=SUBSTITUTE
CM032588.1	332933	.	TC	T	100	*	TYPE=DELETE
CM032588.1	332937	.	A	G	100	*	TYPE=SUBSTITUTE
CM032588.1	332955	.	C	G	100	*	TYPE=SUBSTITUTE
CM032588.1	332970	.	T	C	100	*	TYPE=SUBSTITUTE

CM032588.1	332982	.	G	T	100	*	TYPE=SUBSTITUTE
CM032588.1	332991	.	G	A	100	*	TYPE=SUBSTITUTE
CM032588.1	332994	.	T	C	100	*	TYPE=SUBSTITUTE
CM032588.1	333018	.	G	T	100	*	TYPE=SUBSTITUTE
CM032588.1	333024	.	A	G	100	*	TYPE=SUBSTITUTE
CM032588.1	333039	.	T	C	100	*	TYPE=SUBSTITUTE
CM032588.1	333051	.	A	G	100	*	TYPE=SUBSTITUTE
CM032588.1	333090	.	G	A	100	*	TYPE=SUBSTITUTE
CM032588.1	333096	.	C	G	100	*	TYPE=SUBSTITUTE
CM032588.1	333114	.	G	C	100	*	TYPE=SUBSTITUTE
CM032588.1	333159	.	G	T	100	*	TYPE=SUBSTITUTE
CM032588.1	333169	.	A	G	100	*	TYPE=SUBSTITUTE
CM032588.1	333212	.	A	G	100	*	TYPE=SUBSTITUTE
CM032588.1	333236	.	C	T	100	*	TYPE=SUBSTITUTE
CM032588.1	333260	.	C	T	100	*	TYPE=SUBSTITUTE
CM032588.1	333261	.	CTAA	C	100	*	TYPE=DELETE
CM032588.1	333279	.	C	G	100	*	TYPE=SUBSTITUTE
CM032588.1	333287	.	T	A	100	*	TYPE=SUBSTITUTE
CM032588.1	333342	.	C	T	100	*	TYPE=SUBSTITUTE
CM032588.1	333401	.	C	G	100	*	TYPE=SUBSTITUTE
CM032588.1	333404	.	G	C	100	*	TYPE=SUBSTITUTE
CM032588.1	333415	.	T	G	100	*	TYPE=SUBSTITUTE
CM032588.1	333445	.	G	A	100	*	TYPE=SUBSTITUTE
CM032588.1	333449	.	T	A	100	*	TYPE=SUBSTITUTE
CM032588.1	333454	.	A	G	100	*	TYPE=SUBSTITUTE
CM032588.1	333518	.	T	C	100	*	TYPE=SUBSTITUTE
CM032588.1	333532	.	T	C	100	*	TYPE=SUBSTITUTE
CM032588.1	333563	.	C	G	100	*	TYPE=SUBSTITUTE
CM032588.1	333578	.	A	G	100	*	TYPE=SUBSTITUTE
CM032588.1	333582	.	C	T	100	*	TYPE=SUBSTITUTE
CM032588.1	333585	.	C	T	100	*	TYPE=SUBSTITUTE
CM032588.1	333596	.	C	T	100	*	TYPE=SUBSTITUTE
CM032588.1	333600	.	G	A	100	*	TYPE=SUBSTITUTE
CM032588.1	333602	.	G	A	100	*	TYPE=SUBSTITUTE
CM032588.1	333606	.	T	C	100	*	TYPE=SUBSTITUTE
CM032588.1	333607	.	G	A	100	*	TYPE=SUBSTITUTE
CM032588.1	333623	.	G	A	100	*	TYPE=SUBSTITUTE
CM032588.1	333627	.	TC	T	100	*	TYPE=DELETE
CM032588.1	333631	.	G	GA	100	*	TYPE=INSERT
CM032588.1	333649	.	G	A	100	*	TYPE=SUBSTITUTE
CM032588.1	333650	.	TTC	T	100	*	TYPE=DELETE
CM032588.1	333654	.	C	T	100	*	TYPE=SUBSTITUTE
CM032588.1	333655	.	T	G	100	*	TYPE=SUBSTITUTE
CM032588.1	333656	.	T	G	100	*	TYPE=SUBSTITUTE
CM032588.1	333660	.	T	G	100	*	TYPE=SUBSTITUTE
CM032588.1	333667	.	G	C	100	*	TYPE=SUBSTITUTE
CM032588.1	333673	.	G	A	100	*	TYPE=SUBSTITUTE
CM032588.1	333674	.	C	G	100	*	TYPE=SUBSTITUTE
CM032588.1	333677	.	A	C	100	*	TYPE=SUBSTITUTE
CM032588.1	333687	.	C	T	100	*	TYPE=SUBSTITUTE
CM032588.1	333689	.	A	G	100	*	TYPE=SUBSTITUTE
CM032588.1	333690	.	G	A	100	*	TYPE=SUBSTITUTE
CM032588.1	333692	.	A	G	100	*	TYPE=SUBSTITUTE
CM032588.1	333693	.	C	T	100	*	TYPE=SUBSTITUTE
CM032588.1	333700	.	AC	A	100	*	TYPE=DELETE
CM032588.1	333702	.	A	T	100	*	TYPE=SUBSTITUTE
CM032588.1	333706	.	G	A	100	*	TYPE=SUBSTITUTE
CM032588.1	333709	.	A	G	100	*	TYPE=SUBSTITUTE
CM032588.1	333710	.	A	C	100	*	TYPE=SUBSTITUTE
CM032588.1	333712	.	G	T	100	*	TYPE=SUBSTITUTE
CM032588.1	333731	.	G	T	100	*	TYPE=SUBSTITUTE
CM032588.1	333744	.	A	G	100	*	TYPE=SUBSTITUTE
CM032588.1	333747	.	A	G	100	*	TYPE=SUBSTITUTE
CM032588.1	333750	.	T	C	100	*	TYPE=SUBSTITUTE

CM032588.1	333751	.	C	A	100	*	TYPE=SUBSTITUTE
CM032588.1	333756	.	A	T	100	*	TYPE=SUBSTITUTE
CM032588.1	333758	.	C	G	100	*	TYPE=SUBSTITUTE
CM032588.1	333759	.	A	C	100	*	TYPE=SUBSTITUTE
CM032588.1	333771	.	G	A	100	*	TYPE=SUBSTITUTE
CM032588.1	333772	.	A	G	100	*	TYPE=SUBSTITUTE
CM032588.1	333773	.	T	C	100	*	TYPE=SUBSTITUTE
CM032588.1	333775	.	C	G	100	*	TYPE=SUBSTITUTE
CM032588.1	333779	.	T	A	100	*	TYPE=SUBSTITUTE
CM032588.1	333781	.	A	G	100	*	TYPE=SUBSTITUTE
CM032588.1	333782	.	G	A	100	*	TYPE=SUBSTITUTE
CM032588.1	333783	.	G	A	100	*	TYPE=SUBSTITUTE
CM032588.1	333788	.	A	G	100	*	TYPE=SUBSTITUTE
CM032588.1	333795	.	T	A	100	*	TYPE=SUBSTITUTE
CM032588.1	333805	.	G	A	100	*	TYPE=SUBSTITUTE
CM032588.1	333809	.	A	G	100	*	TYPE=SUBSTITUTE
CM032588.1	333811	.	T	C	100	*	TYPE=SUBSTITUTE
CM032588.1	333812	.	C	T	100	*	TYPE=SUBSTITUTE
CM032588.1	333816	.	A	C	100	*	TYPE=SUBSTITUTE
CM032588.1	333817	.	T	G	100	*	TYPE=SUBSTITUTE
CM032588.1	333818	.	T	C	100	*	TYPE=SUBSTITUTE
CM032588.1	333820	.	C	T	100	*	TYPE=SUBSTITUTE
CM032588.1	333821	.	C	A	100	*	TYPE=SUBSTITUTE
CM032588.1	333827	.	AT	A	100	*	TYPE=DELETE
CM032588.1	333831	.	A	C	100	*	TYPE=SUBSTITUTE
CM032588.1	333840	.	T	A	100	*	TYPE=SUBSTITUTE
CM032588.1	333847	.	T	C	100	*	TYPE=SUBSTITUTE
CM032588.1	333848	.	A	G	100	*	TYPE=SUBSTITUTE
CM032588.1	333849	.	G	A	100	*	TYPE=SUBSTITUTE
CM032588.1	333853	.	T	C	100	*	TYPE=SUBSTITUTE
CM032588.1	333857	.	C	T	100	*	TYPE=SUBSTITUTE
CM032588.1	333861	.	A	G	100	*	TYPE=SUBSTITUTE
CM032588.1	333867	.	C	T	100	*	TYPE=SUBSTITUTE
CM032588.1	333872	.	A	G	100	*	TYPE=SUBSTITUTE
CM032588.1	333873	.	T	C	100	*	TYPE=SUBSTITUTE
CM032588.1	333885	.	T	C	100	*	TYPE=SUBSTITUTE
CM032588.1	333893	.	G	A	100	*	TYPE=SUBSTITUTE
CM032588.1	333909	.	G	A	100	*	TYPE=SUBSTITUTE
CM032588.1	333925	.	T	G	100	*	TYPE=SUBSTITUTE
CM032588.1	333935	.	A	G	100	*	TYPE=SUBSTITUTE
CM032588.1	333940	.	T	G	100	*	TYPE=SUBSTITUTE
CM032588.1	333941	.	T	TAGG	100	*	TYPE=INSERT
CM032588.1	333942	.	G	T	100	*	TYPE=SUBSTITUTE
CM032588.1	333948	.	C	T	100	*	TYPE=SUBSTITUTE
CM032588.1	333952	.	AG	A	100	*	TYPE=DELETE
CM032588.1	333955	.	C	T	100	*	TYPE=SUBSTITUTE
CM032588.1	333968	.	G	A	100	*	TYPE=SUBSTITUTE
CM032588.1	333978	.	A	G	100	*	TYPE=SUBSTITUTE
CM032588.1	333990	.	T	C	100	*	TYPE=SUBSTITUTE
CM032588.1	333993	.	T	A	100	*	TYPE=SUBSTITUTE
CM032588.1	334001	.	A	T	100	*	TYPE=SUBSTITUTE
CM032588.1	334003	.	G	A	100	*	TYPE=SUBSTITUTE
CM032588.1	334009	.	T	C	100	*	TYPE=SUBSTITUTE
CM032588.1	334017	.	TA	T	100	*	TYPE=DELETE
CM032588.1	334021	.	C	T	100	*	TYPE=SUBSTITUTE
CM032588.1	334026	.	G	C	100	*	TYPE=SUBSTITUTE
CM032588.1	334036	.	C	T	100	*	TYPE=SUBSTITUTE
CM032588.1	334038	.	C	T	100	*	TYPE=SUBSTITUTE
CM032588.1	334058	.	CATGTAC	C	100	*	TYPE=DELETE
CM032588.1	334066	.	A	G	100	*	TYPE=SUBSTITUTE
CM032588.1	334070	.	CA	C	100	*	TYPE=DELETE
CM032588.1	334074	.	G	T	100	*	TYPE=SUBSTITUTE
CM032588.1	334075	.	C	T	100	*	TYPE=SUBSTITUTE
CM032588.1	334078	.	C	A	100	*	TYPE=SUBSTITUTE

CM032588.1	334079	.	G	A	100	*	TYPE=SUBSTITUTE
CM032588.1	334085	.	A	G	100	*	TYPE=SUBSTITUTE
CM032588.1	334088	.	T	G	100	*	TYPE=SUBSTITUTE
CM032588.1	334090	.	G	C	100	*	TYPE=SUBSTITUTE
CM032588.1	334091	.	A	C	100	*	TYPE=SUBSTITUTE
CM032588.1	334101	.	T	C	100	*	TYPE=SUBSTITUTE
CM032588.1	334107	.	T	C	100	*	TYPE=SUBSTITUTE
CM032588.1	334146	.	A	G	100	*	TYPE=SUBSTITUTE
CM032588.1	334161	.	T	C	100	*	TYPE=SUBSTITUTE
CM032588.1	334188	.	T	C	100	*	TYPE=SUBSTITUTE
CM032588.1	334200	.	C	T	100	*	TYPE=SUBSTITUTE
CM032588.1	334205	.	A	G	100	*	TYPE=SUBSTITUTE
CM032588.1	334211	.	G	a	100	*	TYPE=SUBSTITUTE
CM032588.1	334224	.	C	t	100	*	TYPE=SUBSTITUTE
CM032588.1	334239	.	A	C	100	*	TYPE=SUBSTITUTE
CM032588.1	334260	.	A	G	100	*	TYPE=SUBSTITUTE
CM032588.1	334263	.	A	G	100	*	TYPE=SUBSTITUTE
CM032588.1	334269	.	A	G	100	*	TYPE=SUBSTITUTE
CM032588.1	334291	.	AA	A	100	*	TYPE=DELETE
CM032588.1	334293	.	A	G	100	*	TYPE=SUBSTITUTE
CM032588.1	334310	.	A	C	100	*	TYPE=SUBSTITUTE
CM032588.1	334322	.	G	T	100	*	TYPE=SUBSTITUTE
CM032588.1	334323	.	A	T	100	*	TYPE=SUBSTITUTE
CM032588.1	334328	.	A	G	100	*	TYPE=SUBSTITUTE
CM032588.1	334329	.	T	A	100	*	TYPE=SUBSTITUTE
CM032588.1	334337	.	A	G	100	*	TYPE=SUBSTITUTE
CM032588.1	334351	.	A	G	100	*	TYPE=SUBSTITUTE
CM032588.1	334360	.	T	C	100	*	TYPE=SUBSTITUTE
CM032588.1	334375	.	C	A	100	*	TYPE=SUBSTITUTE
CM032588.1	334399	.	A	G	100	*	TYPE=SUBSTITUTE
CM032588.1	334435	.	T	A	100	*	TYPE=SUBSTITUTE
CM032588.1	334441	.	C	G	100	*	TYPE=SUBSTITUTE
CM032588.1	334468	.	G	A	100	*	TYPE=SUBSTITUTE
CM032588.1	334483	.	G	A	100	*	TYPE=SUBSTITUTE
CM032588.1	334498	.	A	G	100	*	TYPE=SUBSTITUTE
CM032588.1	334501	.	A	C	100	*	TYPE=SUBSTITUTE
CM032588.1	334507	.	A	G	100	*	TYPE=SUBSTITUTE
CM032588.1	334519	.	G	T	100	*	TYPE=SUBSTITUTE
CM032588.1	334546	.	A	C	100	*	TYPE=SUBSTITUTE
CM032588.1	334588	.	A	C	100	*	TYPE=SUBSTITUTE
CM032588.1	334627	.	G	A	100	*	TYPE=SUBSTITUTE
CM032588.1	334693	.	G	A	100	*	TYPE=SUBSTITUTE
CM032588.1	334702	.	A	G	100	*	TYPE=SUBSTITUTE
CM032588.1	334708	.	A	G	100	*	TYPE=SUBSTITUTE
CM032588.1	334725	.	C	G	100	*	TYPE=SUBSTITUTE
CM032588.1	334759	.	A	G	100	*	TYPE=SUBSTITUTE
CM032588.1	334765	.	A	G	100	*	TYPE=SUBSTITUTE
CM032588.1	334774	.	A	G	100	*	TYPE=SUBSTITUTE
CM032588.1	334795	.	G	A	100	*	TYPE=SUBSTITUTE
CM032588.1	334798	.	A	G	100	*	TYPE=SUBSTITUTE
CM032588.1	334804	.	G	C	100	*	TYPE=SUBSTITUTE
CM032588.1	334810	.	G	A	100	*	TYPE=SUBSTITUTE
CM032588.1	334828	.	C	T	100	*	TYPE=SUBSTITUTE
CM032588.1	334830	.	A	G	100	*	TYPE=SUBSTITUTE
CM032588.1	334831	.	C	T	100	*	TYPE=SUBSTITUTE
CM032588.1	334840	.	T	c	100	*	TYPE=SUBSTITUTE
CM032588.1	334849	.	A	g	100	*	TYPE=SUBSTITUTE
CM032588.1	334852	.	T	c	100	*	TYPE=SUBSTITUTE
CM032588.1	334864	.	A	G	100	*	TYPE=SUBSTITUTE
CM032588.1	334875	.	G	A	100	*	TYPE=SUBSTITUTE
CM032588.1	334885	.	A	C	100	*	TYPE=SUBSTITUTE
CM032588.1	334899	.	G	A	100	*	TYPE=SUBSTITUTE
CM032588.1	334905	.	C	T	100	*	TYPE=SUBSTITUTE
CM032588.1	334909	.	G	A	100	*	TYPE=SUBSTITUTE

CM032588.1	334930	.	T	C	100	*	TYPE=SUBSTITUTE
CM032588.1	334942	.	G	A	100	*	TYPE=SUBSTITUTE
CM032588.1	334957	.	T	C	100	*	TYPE=SUBSTITUTE
CM032588.1	334969	.	C	G	100	*	TYPE=SUBSTITUTE
CM032588.1	334972	.	T	G	100	*	TYPE=SUBSTITUTE
CM032588.1	334975	.	A	T	100	*	TYPE=SUBSTITUTE
CM032588.1	334994	.	A	G	100	*	TYPE=SUBSTITUTE
CM032588.1	334995	.	G	T	100	*	TYPE=SUBSTITUTE
CM032588.1	334996	.	G	C	100	*	TYPE=SUBSTITUTE
CM032588.1	334999	.	C	T	100	*	TYPE=SUBSTITUTE
CM032588.1	335008	.	G	c	100	*	TYPE=SUBSTITUTE
CM032588.1	335027	.	G	T	100	*	TYPE=SUBSTITUTE
CM032588.1	335050	.	T	C	100	*	TYPE=SUBSTITUTE
CM032588.1	335053	.	G	A	100	*	TYPE=SUBSTITUTE
CM032588.1	335056	.	A	G	100	*	TYPE=SUBSTITUTE
CM032588.1	335058	.	C	T	100	*	TYPE=SUBSTITUTE
CM032588.1	335074	.	A	T	100	*	TYPE=SUBSTITUTE
CM032588.1	335103	.	G	T	100	*	TYPE=SUBSTITUTE
CM032588.1	335109	.	T	C	100	*	TYPE=SUBSTITUTE
CM032588.1	335110	.	G	A	100	*	TYPE=SUBSTITUTE
CM032588.1	335179	.	G	A	100	*	TYPE=SUBSTITUTE
CM032588.1	335182	.	A	G	100	*	TYPE=SUBSTITUTE
CM032588.1	335188	.	A	G	100	*	TYPE=SUBSTITUTE
CM032588.1	335260	.	A	G	100	*	TYPE=SUBSTITUTE
CM032588.1	335268	.	A	G	100	*	TYPE=SUBSTITUTE
CM032588.1	335278	.	A	G	100	*	TYPE=SUBSTITUTE
CM032588.1	335281	.	G	A	100	*	TYPE=SUBSTITUTE
CM032588.1	335284	.	G	A	100	*	TYPE=SUBSTITUTE
CM032588.1	335302	.	G	A	100	*	TYPE=SUBSTITUTE
CM032588.1	335311	.	G	A	100	*	TYPE=SUBSTITUTE
CM032588.1	335335	.	C	T	100	*	TYPE=SUBSTITUTE
CM032588.1	335338	.	A	G	100	*	TYPE=SUBSTITUTE
CM032588.1	335395	.	C	T	100	*	TYPE=SUBSTITUTE
CM032588.1	335398	.	G	T	100	*	TYPE=SUBSTITUTE
CM032588.1	335431	.	G	C	100	*	TYPE=SUBSTITUTE
CM032588.1	335449	.	A	G	100	*	TYPE=SUBSTITUTE
CM032588.1	335575	.	A	G	100	*	TYPE=SUBSTITUTE
CM032588.1	335608	.	T	C	100	*	TYPE=SUBSTITUTE
CM032588.1	335617	.	G	A	100	*	TYPE=SUBSTITUTE
CM032588.1	335626	.	A	G	100	*	TYPE=SUBSTITUTE
CM032588.1	335629	.	A	G	100	*	TYPE=SUBSTITUTE
CM032588.1	335665	.	T	C	100	*	TYPE=SUBSTITUTE
CM032588.1	335701	.	A	G	100	*	TYPE=SUBSTITUTE
CM032588.1	335713	.	G	A	100	*	TYPE=SUBSTITUTE
CM032588.1	335716	.	C	T	100	*	TYPE=SUBSTITUTE
CM032588.1	335752	.	G	T	100	*	TYPE=SUBSTITUTE
CM032588.1	335781	.	T	C	100	*	TYPE=SUBSTITUTE
CM032588.1	335794	.	C	G	100	*	TYPE=SUBSTITUTE
CM032588.1	335797	.	G	A	100	*	TYPE=SUBSTITUTE
CM032588.1	335827	.	T	G	100	*	TYPE=SUBSTITUTE
CM032588.1	335830	.	A	G	100	*	TYPE=SUBSTITUTE
CM032588.1	335831	.	G	A	100	*	TYPE=SUBSTITUTE
CM032588.1	335836	.	C	T	100	*	TYPE=SUBSTITUTE
CM032588.1	335841	.	A	G	100	*	TYPE=SUBSTITUTE
CM032588.1	335842	.	C	T	100	*	TYPE=SUBSTITUTE
CM032588.1	335852	.	G	A	100	*	TYPE=SUBSTITUTE
CM032588.1	335859	.	T	C	100	*	TYPE=SUBSTITUTE
CM032588.1	335870	.	T	A	100	*	TYPE=SUBSTITUTE
CM032588.1	335899	.	A	G	100	*	TYPE=SUBSTITUTE
CM032588.1	335915	.	C	T	100	*	TYPE=SUBSTITUTE
CM032588.1	335922	.	A	G	100	*	TYPE=SUBSTITUTE
CM032588.1	335927	.	C	G	100	*	TYPE=SUBSTITUTE
CM032588.1	335942	.	G	A	100	*	TYPE=SUBSTITUTE
CM032588.1	335944	.	G	C	100	*	TYPE=SUBSTITUTE

CM032588.1	335960	.	G	A	100	*	TYPE=SUBSTITUTE
CM032588.1	335964	.	C	t	100	*	TYPE=SUBSTITUTE
CM032588.1	335975	.	C	t	100	*	TYPE=SUBSTITUTE
CM032588.1	335978	.	T	c	100	*	TYPE=SUBSTITUTE
CM032588.1	335980	.	G	a	100	*	TYPE=SUBSTITUTE
CM032588.1	335988	.	G	A	100	*	TYPE=SUBSTITUTE
CM032588.1	335989	.	C	A	100	*	TYPE=SUBSTITUTE
CM032588.1	336003	.	C	T	100	*	TYPE=SUBSTITUTE
CM032588.1	336011	.	C	A	100	*	TYPE=SUBSTITUTE
CM032588.1	336032	.	T	C	100	*	TYPE=SUBSTITUTE
CM032588.1	336042	.	G	T	100	*	TYPE=SUBSTITUTE
CM032588.1	336047	.	A	C	100	*	TYPE=SUBSTITUTE
CM032588.1	336050	.	G	A	100	*	TYPE=SUBSTITUTE
CM032588.1	336051	.	G	A	100	*	TYPE=SUBSTITUTE
CM032588.1	336055	.	G	A	100	*	TYPE=SUBSTITUTE
CM032588.1	336056	.	G	C	100	*	TYPE=SUBSTITUTE
CM032588.1	336063	.	T	G	100	*	TYPE=SUBSTITUTE
CM032588.1	336100	.	A	G	100	*	TYPE=SUBSTITUTE
CM032588.1	336133	.	T	C	100	*	TYPE=SUBSTITUTE
CM032588.1	336163	.	A	G	100	*	TYPE=SUBSTITUTE
CM032588.1	336167	.	C	CT	100	*	TYPE=INSERT
CM032588.1	336189	.	G	T	100	*	TYPE=SUBSTITUTE
CM032588.1	336204	.	G	A	100	*	TYPE=SUBSTITUTE
CM032588.1	336206	.	T	C	100	*	TYPE=SUBSTITUTE
CM032588.1	336263	.	CC	C	100	*	TYPE=DELETE
CM032588.1	336274	.	C	A	100	*	TYPE=SUBSTITUTE
CM032588.1	336289	.	G	T	100	*	TYPE=SUBSTITUTE
CM032588.1	336301	.	G	C	100	*	TYPE=SUBSTITUTE
CM032588.1	336306	.	A	G	100	*	TYPE=SUBSTITUTE
CM032588.1	336321	.	G	A	100	*	TYPE=SUBSTITUTE
CM032588.1	336322	.	G	A	100	*	TYPE=SUBSTITUTE
CM032588.1	336323	.	C	A	100	*	TYPE=SUBSTITUTE
CM032588.1	336335	.	CG	C	100	*	TYPE=DELETE
CM032588.1	336340	.	C	T	100	*	TYPE=SUBSTITUTE
CM032588.1	336345	.	G	A	100	*	TYPE=SUBSTITUTE
CM032588.1	336356	.	C	G	100	*	TYPE=SUBSTITUTE
CM032588.1	336357	.	A	G	100	*	TYPE=SUBSTITUTE
CM032588.1	336358	.	G	T	100	*	TYPE=SUBSTITUTE
CM032588.1	336361	.	T	C	100	*	TYPE=SUBSTITUTE
CM032588.1	336364	.	G	T	100	*	TYPE=SUBSTITUTE
CM032588.1	336368	.	G	A	100	*	TYPE=SUBSTITUTE
CM032588.1	336370	.	C	G	100	*	TYPE=SUBSTITUTE
CM032588.1	336371	.	C	T	100	*	TYPE=SUBSTITUTE
CM032588.1	336375	.	C	T	100	*	TYPE=SUBSTITUTE
CM032588.1	336376	.	T	C	100	*	TYPE=SUBSTITUTE
CM032588.1	336379	.	T	C	100	*	TYPE=SUBSTITUTE
CM032588.1	336382	.	C	T	100	*	TYPE=SUBSTITUTE
CM032588.1	336392	.	G	C	100	*	TYPE=SUBSTITUTE
CM032588.1	336397	.	G	A	100	*	TYPE=SUBSTITUTE
CM032588.1	336401	.	G	C	100	*	TYPE=SUBSTITUTE
CM032588.1	336405	.	A	G	100	*	TYPE=SUBSTITUTE
CM032588.1	336407	.	C	G	100	*	TYPE=SUBSTITUTE
CM032588.1	336408	.	G	A	100	*	TYPE=SUBSTITUTE
CM032588.1	336411	.	G	A	100	*	TYPE=SUBSTITUTE
CM032588.1	336417	.	T	C	100	*	TYPE=SUBSTITUTE
CM032588.1	336418	.	G	C	100	*	TYPE=SUBSTITUTE
CM032588.1	336419	.	C	T	100	*	TYPE=SUBSTITUTE
CM032588.1	336420	.	C	T	100	*	TYPE=SUBSTITUTE
CM032588.1	336423	.	C	G	100	*	TYPE=SUBSTITUTE
CM032588.1	336424	.	A	C	100	*	TYPE=SUBSTITUTE
CM032588.1	336427	.	G	A	100	*	TYPE=SUBSTITUTE
CM032588.1	336429	.	T	G	100	*	TYPE=SUBSTITUTE
CM032588.1	336433	.	C	T	100	*	TYPE=SUBSTITUTE
CM032588.1	336439	.	C	G	100	*	TYPE=SUBSTITUTE

CM032588.1	336447	.	C	A	100	*	TYPE=SUBSTITUTE
CM032588.1	336450	.	T	G	100	*	TYPE=SUBSTITUTE
CM032588.1	336455	.	A	G	100	*	TYPE=SUBSTITUTE
CM032588.1	336461	.	G	T	100	*	TYPE=SUBSTITUTE
CM032588.1	336463	.	G	A	100	*	TYPE=SUBSTITUTE
CM032588.1	336469	.	A	G	100	*	TYPE=SUBSTITUTE
CM032588.1	336473	.	G	A	100	*	TYPE=SUBSTITUTE
CM032588.1	336475	.	G	C	100	*	TYPE=SUBSTITUTE
CM032588.1	336477	.	T	C	100	*	TYPE=SUBSTITUTE
CM032588.1	336480	.	A	C	100	*	TYPE=SUBSTITUTE
CM032588.1	336481	.	C	T	100	*	TYPE=SUBSTITUTE
CM032588.1	336482	.	A	G	100	*	TYPE=SUBSTITUTE
CM032588.1	336483	.	G	T	100	*	TYPE=SUBSTITUTE
CM032588.1	336484	.	G	T	100	*	TYPE=SUBSTITUTE
CM032588.1	336486	.	C	T	100	*	TYPE=SUBSTITUTE
CM032588.1	336492	.	A	G	100	*	TYPE=SUBSTITUTE
CM032588.1	336496	.	A	t	100	*	TYPE=SUBSTITUTE
CM032588.1	336497	.	g	gaaata	100	*	TYPE=INSERT
CM032588.1	336498	.	G	a	100	*	TYPE=SUBSTITUTE
CM032588.1	336499	.	C	a	100	*	TYPE=SUBSTITUTE
CM032588.1	336504	.	T	c	100	*	TYPE=SUBSTITUTE
CM032588.1	336506	.	T	G	100	*	TYPE=SUBSTITUTE
CM032588.1	336511	.	G	A	100	*	TYPE=SUBSTITUTE
CM032588.1	336512	.	G	T	100	*	TYPE=SUBSTITUTE
CM032588.1	336513	.	C	CACACAAATT	100	*	TYPE=INSERT
CM032588.1	336516	.	A	T	100	*	TYPE=SUBSTITUTE
CM032588.1	336519	.	G	GAA	100	*	TYPE=INSERT
CM032588.1	336521	.	A	ATGAT	100	*	TYPE=INSERT
CM032588.1	336524	.	T	A	100	*	TYPE=SUBSTITUTE
CM032588.1	336526	.	G	A	100	*	TYPE=SUBSTITUTE
CM032588.1	336528	.	A	T	100	*	TYPE=SUBSTITUTE
CM032588.1	336529	.	G	GCCTA	100	*	TYPE=INSERT
CM032588.1	336532	.	T	C	100	*	TYPE=SUBSTITUTE
CM032588.1	336533	.	C	T	100	*	TYPE=SUBSTITUTE
CM032588.1	336535	.	T	TA	100	*	TYPE=INSERT
CM032588.1	336539	.	T	C	100	*	TYPE=SUBSTITUTE
CM032588.1	336541	.	A	T	100	*	TYPE=SUBSTITUTE
CM032588.1	336545	.	A	C	100	*	TYPE=SUBSTITUTE
CM032588.1	336548	.	T	C	100	*	TYPE=SUBSTITUTE
CM032588.1	336558	.	A	T	100	*	TYPE=SUBSTITUTE
CM032588.1	336573	.	C	t	100	*	TYPE=SUBSTITUTE
CM032588.1	336575	.	C	t	100	*	TYPE=SUBSTITUTE
CM032588.1	336576	.	A	g	100	*	TYPE=SUBSTITUTE
CM032588.1	336579	.	G	t	100	*	TYPE=SUBSTITUTE
CM032588.1	336583	.	G	t	100	*	TYPE=SUBSTITUTE
CM032588.1	336592	.	T	G	100	*	TYPE=SUBSTITUTE
CM032588.1	336593	.	T	A	100	*	TYPE=SUBSTITUTE
CM032588.1	336604	.	A	G	100	*	TYPE=SUBSTITUTE
CM032588.1	336608	.	T	G	100	*	TYPE=SUBSTITUTE
CM032588.1	336634	.	T	C	100	*	TYPE=SUBSTITUTE
CM032588.1	336635	.	T	G	100	*	TYPE=SUBSTITUTE
CM032588.1	336640	.	G	A	100	*	TYPE=SUBSTITUTE
CM032588.1	336650	.	C	T	100	*	TYPE=SUBSTITUTE
CM032588.1	336651	.	T	C	100	*	TYPE=SUBSTITUTE
CM032588.1	336665	.	A	C	100	*	TYPE=SUBSTITUTE
CM032588.1	336666	.	T	C	100	*	TYPE=SUBSTITUTE
CM032588.1	336677	.	C	G	100	*	TYPE=SUBSTITUTE
CM032588.1	336681	.	A	G	100	*	TYPE=SUBSTITUTE
CM032588.1	336687	.	G	A	100	*	TYPE=SUBSTITUTE
CM032588.1	336690	.	A	C	100	*	TYPE=SUBSTITUTE
CM032588.1	336704	.	T	G	100	*	TYPE=SUBSTITUTE
CM032588.1	336714	.	G	A	100	*	TYPE=SUBSTITUTE
CM032588.1	336720	.	C	T	100	*	TYPE=SUBSTITUTE
CM032588.1	336729	.	G	A	100	*	TYPE=SUBSTITUTE

CM032588.1	336749	.	T	G	100	*	TYPE=SUBSTITUTE
CM032588.1	336765	.	C	T	100	*	TYPE=SUBSTITUTE
CM032588.1	336768	.	A	G	100	*	TYPE=SUBSTITUTE
CM032588.1	336783	.	C	T	100	*	TYPE=SUBSTITUTE
CM032588.1	336786	.	T	C	100	*	TYPE=SUBSTITUTE
CM032588.1	336807	.	G	A	100	*	TYPE=SUBSTITUTE
CM032588.1	336810	.	C	G	100	*	TYPE=SUBSTITUTE
CM032588.1	336831	.	C	T	100	*	TYPE=SUBSTITUTE
CM032588.1	336849	.	C	T	100	*	TYPE=SUBSTITUTE
CM032588.1	336879	.	G	A	100	*	TYPE=SUBSTITUTE
CM032588.1	336983	.	G	A	100	*	TYPE=SUBSTITUTE
CM032588.1	336984	.	T	C	100	*	TYPE=SUBSTITUTE
CM032588.1	337038	.	G	A	100	*	TYPE=SUBSTITUTE
CM032588.1	337056	.	C	T	100	*	TYPE=SUBSTITUTE
CM032588.1	337071	.	G	A	100	*	TYPE=SUBSTITUTE
CM032588.1	337074	.	T	C	100	*	TYPE=SUBSTITUTE
CM032588.1	337080	.	A	G	100	*	TYPE=SUBSTITUTE
CM032588.1	337131	.	A	G	100	*	TYPE=SUBSTITUTE
CM032588.1	337164	.	C	G	100	*	TYPE=SUBSTITUTE
CM032588.1	337166	.	A	G	100	*	TYPE=SUBSTITUTE
CM032588.1	337167	.	A	G	100	*	TYPE=SUBSTITUTE
CM032588.1	337185	.	A	G	100	*	TYPE=SUBSTITUTE
CM032588.1	337203	.	G	A	100	*	TYPE=SUBSTITUTE
CM032588.1	337218	.	G	A	100	*	TYPE=SUBSTITUTE
CM032588.1	337224	.	G	A	100	*	TYPE=SUBSTITUTE
CM032588.1	337230	.	G	A	100	*	TYPE=SUBSTITUTE
CM032588.1	337236	.	G	A	100	*	TYPE=SUBSTITUTE
CM032588.1	337245	.	G	A	100	*	TYPE=SUBSTITUTE
CM032588.1	337248	.	G	A	100	*	TYPE=SUBSTITUTE
CM032588.1	337263	.	A	G	100	*	TYPE=SUBSTITUTE
CM032588.1	337266	.	G	A	100	*	TYPE=SUBSTITUTE
CM032588.1	337272	.	G	A	100	*	TYPE=SUBSTITUTE
CM032588.1	337287	.	G	A	100	*	TYPE=SUBSTITUTE
CM032588.1	337313	.	CCT	C	100	*	TYPE=DELETE
CM032588.1	337321	.	T	C	100	*	TYPE=SUBSTITUTE
CM032588.1	337331	.	A	G	100	*	TYPE=SUBSTITUTE
CM032588.1	337336	.	T	A	100	*	TYPE=SUBSTITUTE
CM032588.1	337339	.	C	G	100	*	TYPE=SUBSTITUTE
CM032588.1	337347	.	T	G	100	*	TYPE=SUBSTITUTE
CM032588.1	337354	.	A	G	100	*	TYPE=SUBSTITUTE
CM032588.1	337357	.	G	T	100	*	TYPE=SUBSTITUTE
CM032588.1	337367	.	G	T	100	*	TYPE=SUBSTITUTE
CM032588.1	337379	.	T	C	100	*	TYPE=SUBSTITUTE
CM032588.1	337388	.	G	A	100	*	TYPE=SUBSTITUTE
CM032588.1	337480	.	A	G	100	*	TYPE=SUBSTITUTE
CM032588.1	337553	.	C	T	100	*	TYPE=SUBSTITUTE
CM032588.1	337556	.	C	T	100	*	TYPE=SUBSTITUTE
CM032588.1	337578	.	A	G	100	*	TYPE=SUBSTITUTE
CM032588.1	337590	.	C	T	100	*	TYPE=SUBSTITUTE
CM032588.1	337592	.	A	T	100	*	TYPE=SUBSTITUTE
CM032588.1	337593	.	A	C	100	*	TYPE=SUBSTITUTE
CM032588.1	337632	.	A	G	100	*	TYPE=SUBSTITUTE
CM032588.1	337737	.	A	G	100	*	TYPE=SUBSTITUTE
CM032588.1	337751	.	A	T	100	*	TYPE=SUBSTITUTE
CM032588.1	337769	.	T	G	100	*	TYPE=SUBSTITUTE
CM032588.1	337773	.	C	T	100	*	TYPE=SUBSTITUTE
CM032588.1	337804	.	G	A	100	*	TYPE=SUBSTITUTE
CM032588.1	337818	.	T	C	100	*	TYPE=SUBSTITUTE
CM032588.1	337829	.	G	A	100	*	TYPE=SUBSTITUTE
CM032588.1	337832	.	C	A	100	*	TYPE=SUBSTITUTE
CM032588.1	337858	.	TTG	T	100	*	TYPE=DELETE
CM032588.1	337861	.	T	A	100	*	TYPE=SUBSTITUTE
CM032588.1	337871	.	TCAAGATG	T	100	*	TYPE=DELETE
CM032588.1	337879	.	A	G	100	*	TYPE=SUBSTITUTE

CM032588.1	337883	.	T	A	100	*	TYPE=SUBSTITUTE
CM032588.1	337885	.	TTG	T	100	*	TYPE=DELETE
CM032588.1	337888	.	C	A	100	*	TYPE=SUBSTITUTE
CM032588.1	337890	.	T	G	100	*	TYPE=SUBSTITUTE
CM032588.1	337891	.	A	G	100	*	TYPE=SUBSTITUTE
CM032588.1	337892	.	C	A	100	*	TYPE=SUBSTITUTE
CM032588.1	337894	.	C	A	100	*	TYPE=SUBSTITUTE
CM032588.1	337912	.	A	G	100	*	TYPE=SUBSTITUTE
CM032588.1	337918	.	A	G	100	*	TYPE=SUBSTITUTE
CM032588.1	337936	.	A	G	100	*	TYPE=SUBSTITUTE
CM032588.1	337950	.	A	T	100	*	TYPE=SUBSTITUTE
CM032588.1	337955	.	C	G	100	*	TYPE=SUBSTITUTE
CM032588.1	337956	.	T	C	100	*	TYPE=SUBSTITUTE
CM032588.1	337958	.	T	A	100	*	TYPE=SUBSTITUTE
CM032588.1	337970	.	C	A	100	*	TYPE=SUBSTITUTE
CM032588.1	337972	.	A	G	100	*	TYPE=SUBSTITUTE
CM032588.1	337977	.	C	T	100	*	TYPE=SUBSTITUTE
CM032588.1	337979	.	C	T	100	*	TYPE=SUBSTITUTE
CM032588.1	337980	.	T	A	100	*	TYPE=SUBSTITUTE
CM032588.1	337987	.	C	T	100	*	TYPE=SUBSTITUTE
CM032588.1	337992	.	T	C	100	*	TYPE=SUBSTITUTE
CM032588.1	337995	.	C	A	100	*	TYPE=SUBSTITUTE
CM032588.1	338000	.	C	T	100	*	TYPE=SUBSTITUTE
CM032588.1	338003	.	G	A	100	*	TYPE=SUBSTITUTE
CM032588.1	338004	.	T	G	100	*	TYPE=SUBSTITUTE
CM032588.1	338021	.	T	C	100	*	TYPE=SUBSTITUTE
CM032588.1	338034	.	A	T	100	*	TYPE=SUBSTITUTE
CM032588.1	338045	.	G	A	100	*	TYPE=SUBSTITUTE
CM032588.1	338051	.	C	T	100	*	TYPE=SUBSTITUTE
CM032588.1	338069	.	C	T	100	*	TYPE=SUBSTITUTE
CM032588.1	338072	.	C	T	100	*	TYPE=SUBSTITUTE
CM032588.1	338075	.	A	C	100	*	TYPE=SUBSTITUTE
CM032588.1	338076	.	C	T	100	*	TYPE=SUBSTITUTE
CM032588.1	338080	.	T	A	100	*	TYPE=SUBSTITUTE
CM032588.1	338123	.	T	C	100	*	TYPE=SUBSTITUTE
CM032588.1	338127	.	C	T	100	*	TYPE=SUBSTITUTE
CM032588.1	338135	.	C	T	100	*	TYPE=SUBSTITUTE
CM032588.1	338141	.	C	T	100	*	TYPE=SUBSTITUTE
CM032588.1	338144	.	C	T	100	*	TYPE=SUBSTITUTE
CM032588.1	338153	.	T	C	100	*	TYPE=SUBSTITUTE
CM032588.1	338154	.	A	G	100	*	TYPE=SUBSTITUTE
CM032588.1	338156	.	C	T	100	*	TYPE=SUBSTITUTE
CM032588.1	338168	.	T	C	100	*	TYPE=SUBSTITUTE
CM032588.1	338174	.	A	G	100	*	TYPE=SUBSTITUTE
CM032588.1	338186	.	G	A	100	*	TYPE=SUBSTITUTE
CM032588.1	338189	.	C	G	100	*	TYPE=SUBSTITUTE
CM032588.1	338194	.	A	C	100	*	TYPE=SUBSTITUTE
CM032588.1	338222	.	A	G	100	*	TYPE=SUBSTITUTE
CM032588.1	338249	.	C	T	100	*	TYPE=SUBSTITUTE
CM032588.1	338258	.	T	C	100	*	TYPE=SUBSTITUTE
CM032588.1	338267	.	T	C	100	*	TYPE=SUBSTITUTE
CM032588.1	338273	.	G	C	100	*	TYPE=SUBSTITUTE
CM032588.1	338279	.	C	T	100	*	TYPE=SUBSTITUTE
CM032588.1	338291	.	G	A	100	*	TYPE=SUBSTITUTE
CM032588.1	338303	.	T	A	100	*	TYPE=SUBSTITUTE
CM032588.1	338316	.	C	T	100	*	TYPE=SUBSTITUTE
CM032588.1	338330	.	G	A	100	*	TYPE=SUBSTITUTE
CM032588.1	338342	.	G	A	100	*	TYPE=SUBSTITUTE
CM032588.1	338349	.	T	C	100	*	TYPE=SUBSTITUTE
CM032588.1	338354	.	T	A	100	*	TYPE=SUBSTITUTE
CM032588.1	338360	.	T	C	100	*	TYPE=SUBSTITUTE
CM032588.1	338363	.	T	C	100	*	TYPE=SUBSTITUTE
CM032588.1	338374	.	A	G	100	*	TYPE=SUBSTITUTE
CM032588.1	338376	.	A	AC	100	*	TYPE=INSERT

CM032588.1	338380	.	T	A	100	*	TYPE=SUBSTITUTE
CM032588.1	338381	.	A	G	100	*	TYPE=SUBSTITUTE
CM032588.1	338398	.	G	A	100	*	TYPE=SUBSTITUTE
CM032588.1	338404	.	AG	A	100	*	TYPE=DELETE
CM032588.1	338417	.	G	T	100	*	TYPE=SUBSTITUTE
CM032588.1	338446	.	C	A	100	*	TYPE=SUBSTITUTE
CM032588.1	338474	.	C	T	100	*	TYPE=SUBSTITUTE
CM032588.1	338507	.	A	C	100	*	TYPE=SUBSTITUTE
CM032588.1	338513	.	G	A	100	*	TYPE=SUBSTITUTE
CM032588.1	338525	.	C	T	100	*	TYPE=SUBSTITUTE
CM032588.1	338570	.	A	T	100	*	TYPE=SUBSTITUTE
CM032588.1	338576	.	A	T	100	*	TYPE=SUBSTITUTE
CM032588.1	338585	.	C	T	100	*	TYPE=SUBSTITUTE
CM032588.1	338609	.	A	G	100	*	TYPE=SUBSTITUTE
CM032588.1	338612	.	T	C	100	*	TYPE=SUBSTITUTE
CM032588.1	338615	.	C	T	100	*	TYPE=SUBSTITUTE
CM032588.1	338621	.	C	T	100	*	TYPE=SUBSTITUTE
CM032588.1	338648	.	C	T	100	*	TYPE=SUBSTITUTE
CM032588.1	338663	.	A	G	100	*	TYPE=SUBSTITUTE
CM032588.1	338669	.	C	G	100	*	TYPE=SUBSTITUTE
CM032588.1	338687	.	T	C	100	*	TYPE=SUBSTITUTE
CM032588.1	338699	.	T	C	100	*	TYPE=SUBSTITUTE
CM032588.1	338711	.	C	T	100	*	TYPE=SUBSTITUTE
CM032588.1	338765	.	C	T	100	*	TYPE=SUBSTITUTE
CM032588.1	338769	.	C	T	100	*	TYPE=SUBSTITUTE
CM032588.1	338791	.	T	C	100	*	TYPE=SUBSTITUTE
CM032588.1	338827	.	G	C	100	*	TYPE=SUBSTITUTE
CM032588.1	338837	.	T	A	100	*	TYPE=SUBSTITUTE
CM032588.1	338839	.	G	A	100	*	TYPE=SUBSTITUTE
CM032588.1	338845	.	A	G	100	*	TYPE=SUBSTITUTE
CM032588.1	338853	.	A	G	100	*	TYPE=SUBSTITUTE
CM032588.1	338855	.	AG	A	100	*	TYPE=DELETE
CM032588.1	338876	.	G	A	100	*	TYPE=SUBSTITUTE
CM032588.1	338907	.	A	G	100	*	TYPE=SUBSTITUTE
CM032588.1	338913	.	T	A	100	*	TYPE=SUBSTITUTE
CM032588.1	338916	.	G	T	100	*	TYPE=SUBSTITUTE
CM032588.1	338955	.	C	G	100	*	TYPE=SUBSTITUTE
CM032588.1	338973	.	A	G	100	*	TYPE=SUBSTITUTE
CM032588.1	338998	.	A	C	100	*	TYPE=SUBSTITUTE
CM032588.1	339027	.	C	T	100	*	TYPE=SUBSTITUTE
CM032588.1	339054	.	G	A	100	*	TYPE=SUBSTITUTE
CM032588.1	339057	.	A	G	100	*	TYPE=SUBSTITUTE
CM032588.1	339060	.	C	T	100	*	TYPE=SUBSTITUTE
CM032588.1	339063	.	G	A	100	*	TYPE=SUBSTITUTE
CM032588.1	339069	.	A	T	100	*	TYPE=SUBSTITUTE
CM032588.1	339081	.	A	C	100	*	TYPE=SUBSTITUTE
CM032588.1	339090	.	T	C	100	*	TYPE=SUBSTITUTE
CM032588.1	339099	.	G	C	100	*	TYPE=SUBSTITUTE
CM032588.1	339108	.	A	G	100	*	TYPE=SUBSTITUTE
CM032588.1	339111	.	C	T	100	*	TYPE=SUBSTITUTE
CM032588.1	339157	.	A	G	100	*	TYPE=SUBSTITUTE
CM032588.1	339166	.	C	T	100	*	TYPE=SUBSTITUTE
CM032588.1	339183	.	A	g	100	*	TYPE=SUBSTITUTE
CM032588.1	339216	.	T	C	100	*	TYPE=SUBSTITUTE
CM032588.1	339230	.	A	G	100	*	TYPE=SUBSTITUTE
CM032588.1	339231	.	A	C	100	*	TYPE=SUBSTITUTE
CM032588.1	339232	.	C	A	100	*	TYPE=SUBSTITUTE
CM032588.1	339235	.	C	T	100	*	TYPE=SUBSTITUTE
CM032588.1	339241	.	A	G	100	*	TYPE=SUBSTITUTE
CM032588.1	339248	.	G	A	100	*	TYPE=SUBSTITUTE
CM032588.1	339249	.	C	T	100	*	TYPE=SUBSTITUTE
CM032588.1	339254	.	T	A	100	*	TYPE=SUBSTITUTE
CM032588.1	339258	.	G	T	100	*	TYPE=SUBSTITUTE
CM032588.1	339262	.	C	A	100	*	TYPE=SUBSTITUTE

CM032588.1	339274	.	T	C	100	*	TYPE=SUBSTITUTE
CM032588.1	339276	.	G	A	100	*	TYPE=SUBSTITUTE
CM032588.1	339279	.	T	C	100	*	TYPE=SUBSTITUTE
CM032588.1	339285	.	AG	A	100	*	TYPE=DELETE
CM032588.1	339292	.	G	A	100	*	TYPE=SUBSTITUTE
CM032588.1	339307	.	A	T	100	*	TYPE=SUBSTITUTE
CM032588.1	339319	.	C	T	100	*	TYPE=SUBSTITUTE
CM032588.1	339340	.	C	T	100	*	TYPE=SUBSTITUTE
CM032588.1	339385	.	G	A	100	*	TYPE=SUBSTITUTE
CM032588.1	339388	.	C	T	100	*	TYPE=SUBSTITUTE
CM032588.1	339428	.	C	T	100	*	TYPE=SUBSTITUTE
CM032588.1	339433	.	C	T	100	*	TYPE=SUBSTITUTE
CM032588.1	339447	.	G	A	100	*	TYPE=SUBSTITUTE
CM032588.1	339450	.	C	T	100	*	TYPE=SUBSTITUTE
CM032588.1	339451	.	G	T	100	*	TYPE=SUBSTITUTE
CM032588.1	339454	.	T	C	100	*	TYPE=SUBSTITUTE
CM032588.1	339466	.	A	G	100	*	TYPE=SUBSTITUTE
CM032588.1	339468	.	CG	C	100	*	TYPE=DELETE
CM032588.1	339473	.	A	T	100	*	TYPE=SUBSTITUTE
CM032588.1	339491	.	C	T	100	*	TYPE=SUBSTITUTE
CM032588.1	339551	.	T	C	100	*	TYPE=SUBSTITUTE
CM032588.1	339557	.	C	T	100	*	TYPE=SUBSTITUTE
CM032588.1	339572	.	A	G	100	*	TYPE=SUBSTITUTE
CM032588.1	339581	.	T	A	100	*	TYPE=SUBSTITUTE
CM032588.1	339582	.	T	A	100	*	TYPE=SUBSTITUTE
CM032588.1	339590	.	C	T	100	*	TYPE=SUBSTITUTE
CM032588.1	339598	.	G	T	100	*	TYPE=SUBSTITUTE
CM032588.1	339617	.	CCAAAAAAAAAAAAAAAAAAAA	C	100	*	TYPE=DELETE
CM032588.1	339648	.	A	g	100	*	TYPE=SUBSTITUTE
CM032588.1	339656	.	A	G	100	*	TYPE=SUBSTITUTE
CM032588.1	339659	.	A	G	100	*	TYPE=SUBSTITUTE
CM032588.1	339669	.	T	G	100	*	TYPE=SUBSTITUTE
CM032588.1	339685	.	G	T	100	*	TYPE=SUBSTITUTE
CM032588.1	339689	.	A	G	100	*	TYPE=SUBSTITUTE
CM032588.1	339697	.	C	T	100	*	TYPE=SUBSTITUTE
CM032588.1	339703	.	C	A	100	*	TYPE=SUBSTITUTE
CM032588.1	339704	.	T	A	100	*	TYPE=SUBSTITUTE
CM032588.1	339705	.	T	G	100	*	TYPE=SUBSTITUTE
CM032588.1	339711	.	G	C	100	*	TYPE=SUBSTITUTE
CM032588.1	339712	.	C	T	100	*	TYPE=SUBSTITUTE
CM032588.1	339716	.	A	C	100	*	TYPE=SUBSTITUTE
CM032588.1	339741	.	A	AG	100	*	TYPE=INSERT
CM032588.1	339746	.	C	T	100	*	TYPE=SUBSTITUTE
CM032588.1	339753	.	T	C	100	*	TYPE=SUBSTITUTE
CM032588.1	339754	.	A	G	100	*	TYPE=SUBSTITUTE
CM032588.1	339756	.	A	G	100	*	TYPE=SUBSTITUTE
CM032588.1	339785	.	C	G	100	*	TYPE=SUBSTITUTE
CM032588.1	339795	.	A	G	100	*	TYPE=SUBSTITUTE
CM032588.1	339829	.	T	C	100	*	TYPE=SUBSTITUTE
CM032588.1	339832	.	C	T	100	*	TYPE=SUBSTITUTE
CM032588.1	339836	.	T	G	100	*	TYPE=SUBSTITUTE
CM032588.1	339837	.	C	G	100	*	TYPE=SUBSTITUTE
CM032588.1	339842	.	T	A	100	*	TYPE=SUBSTITUTE
CM032588.1	339844	.	G	A	100	*	TYPE=SUBSTITUTE
CM032588.1	339858	.	C	T	100	*	TYPE=SUBSTITUTE
CM032588.1	339863	.	C	A	100	*	TYPE=SUBSTITUTE
CM032588.1	339865	.	C	T	100	*	TYPE=SUBSTITUTE
CM032588.1	339869	.	C	T	100	*	TYPE=SUBSTITUTE
CM032588.1	339893	.	C	T	100	*	TYPE=SUBSTITUTE
CM032588.1	339904	.	T	C	100	*	TYPE=SUBSTITUTE
CM032588.1	339911	.	C	T	100	*	TYPE=SUBSTITUTE
CM032588.1	339914	.	A	T	100	*	TYPE=SUBSTITUTE
CM032588.1	339917	.	C	A	100	*	TYPE=SUBSTITUTE

CM032588.1	339918	.	A	G	100	*	TYPE=SUBSTITUTE
CM032588.1	339923	.	G	A	100	*	TYPE=SUBSTITUTE
CM032588.1	339933	.	A	G	100	*	TYPE=SUBSTITUTE
CM032588.1	339941	.	TTGA	T	100	*	TYPE=DELETE
CM032588.1	339948	.	C	A	100	*	TYPE=SUBSTITUTE
CM032588.1	339960	.	A	G	100	*	TYPE=SUBSTITUTE
CM032588.1	340004	.	C	T	100	*	TYPE=SUBSTITUTE
CM032588.1	340025	.	G	T	100	*	TYPE=SUBSTITUTE
CM032588.1	340031	.	C	A	100	*	TYPE=SUBSTITUTE
CM032588.1	340036	.	T	C	100	*	TYPE=SUBSTITUTE
CM032588.1	340044	.	TG	T	100	*	TYPE=DELETE
CM032588.1	340047	.	T	A	100	*	TYPE=SUBSTITUTE
CM032588.1	340062	.	G	A	100	*	TYPE=SUBSTITUTE
CM032588.1	340070	.	G	T	100	*	TYPE=SUBSTITUTE
CM032588.1	340073	.	C	T	100	*	TYPE=SUBSTITUTE
CM032588.1	340074	.	G	C	100	*	TYPE=SUBSTITUTE
CM032588.1	340083	.	C	G	100	*	TYPE=SUBSTITUTE
CM032588.1	340086	.	T	C	100	*	TYPE=SUBSTITUTE
CM032588.1	340095	.	A	G	100	*	TYPE=SUBSTITUTE
CM032588.1	340098	.	T	C	100	*	TYPE=SUBSTITUTE
CM032588.1	340105	.	T	C	100	*	TYPE=SUBSTITUTE
CM032588.1	340107	.	A	G	100	*	TYPE=SUBSTITUTE
CM032588.1	340111	.	T	C	100	*	TYPE=SUBSTITUTE
CM032588.1	340129	.	T	G	100	*	TYPE=SUBSTITUTE
CM032588.1	340145	.	A	G	100	*	TYPE=SUBSTITUTE
CM032588.1	340167	.	A	G	100	*	TYPE=SUBSTITUTE
CM032588.1	340184	.	A	G	100	*	TYPE=SUBSTITUTE
CM032588.1	340187	.	A	G	100	*	TYPE=SUBSTITUTE
CM032588.1	340192	.	G	GA	100	*	TYPE=INSERT
CM032588.1	340198	.	C	T	100	*	TYPE=SUBSTITUTE
CM032588.1	340200	.	T	C	100	*	TYPE=SUBSTITUTE
CM032588.1	340205	.	A	G	100	*	TYPE=SUBSTITUTE
CM032588.1	340264	.	A	AA	100	*	TYPE=INSERT
CM032588.1	340273	.	G	A	100	*	TYPE=SUBSTITUTE
CM032588.1	340386	.	C	T	100	*	TYPE=SUBSTITUTE
CM032588.1	340393	.	C	T	100	*	TYPE=SUBSTITUTE
CM032588.1	340396	.	C	T	100	*	TYPE=SUBSTITUTE
CM032588.1	340451	.	CC	C	100	*	TYPE=DELETE
CM032588.1	340457	.	C	A	100	*	TYPE=SUBSTITUTE
CM032588.1	340458	.	C	A	100	*	TYPE=SUBSTITUTE
CM032588.1	340465	.	A	T	100	*	TYPE=SUBSTITUTE
CM032588.1	340470	.	T	C	100	*	TYPE=SUBSTITUTE
CM032588.1	340475	.	T	C	100	*	TYPE=SUBSTITUTE
CM032588.1	340484	.	G	C	100	*	TYPE=SUBSTITUTE
CM032588.1	340493	.	AG	A	100	*	TYPE=DELETE
CM032588.1	340515	.	G	A	100	*	TYPE=SUBSTITUTE
CM032588.1	340517	.	A	G	100	*	TYPE=SUBSTITUTE
CM032588.1	340525	.	T	A	100	*	TYPE=SUBSTITUTE
CM032588.1	340534	.	T	C	100	*	TYPE=SUBSTITUTE
CM032588.1	340537	.	A	G	100	*	TYPE=SUBSTITUTE
CM032588.1	340539	.	C	T	100	*	TYPE=SUBSTITUTE
CM032588.1	340542	.	T	C	100	*	TYPE=SUBSTITUTE
CM032588.1	340547	.	A	g	100	*	TYPE=SUBSTITUTE
CM032588.1	340551	.	T	c	100	*	TYPE=SUBSTITUTE
CM032588.1	340554	.	c	ca	100	*	TYPE=INSERT
CM032588.1	340557	.	G	a	100	*	TYPE=SUBSTITUTE
CM032588.1	340560	.	G	a	100	*	TYPE=SUBSTITUTE
CM032588.1	340569	.	G	A	100	*	TYPE=SUBSTITUTE
CM032588.1	340576	.	A	T	100	*	TYPE=SUBSTITUTE
CM032588.1	340578	.	T	TTTGTCAA	100	*	TYPE=INSERT
CM032588.1	340581	.	T	C	100	*	TYPE=SUBSTITUTE
CM032588.1	340584	.	G	T	100	*	TYPE=SUBSTITUTE
CM032588.1	340585	.	A	C	100	*	TYPE=SUBSTITUTE
CM032588.1	340586	.	A	C	100	*	TYPE=SUBSTITUTE

CM032588.1	340587	.	A	C	100	*	TYPE=SUBSTITUTE
CM032588.1	340588	.	A	C	100	*	TYPE=SUBSTITUTE
CM032588.1	340589	.	A	C	100	*	TYPE=SUBSTITUTE
CM032588.1	340590	.	A	C	100	*	TYPE=SUBSTITUTE
CM032588.1	340592	.	A	C	100	*	TYPE=SUBSTITUTE
CM032588.1	340593	.	A	C	100	*	TYPE=SUBSTITUTE
CM032588.1	340596	.	A	G	100	*	TYPE=SUBSTITUTE
CM032588.1	340597	.	T	G	100	*	TYPE=SUBSTITUTE
CM032588.1	340600	.	G	A	100	*	TYPE=SUBSTITUTE
CM032588.1	340602	.	A	G	100	*	TYPE=SUBSTITUTE
CM032588.1	340607	.	A	C	100	*	TYPE=SUBSTITUTE
CM032588.1	340609	.	A	G	100	*	TYPE=SUBSTITUTE
CM032588.1	340633	.	G	A	100	*	TYPE=SUBSTITUTE
CM032588.1	340646	.	T	A	100	*	TYPE=SUBSTITUTE
CM032588.1	340661	.	G	A	100	*	TYPE=SUBSTITUTE
CM032588.1	340664	.	G	A	100	*	TYPE=SUBSTITUTE
CM032588.1	340669	.	A	G	100	*	TYPE=SUBSTITUTE
CM032588.1	340673	.	A	T	100	*	TYPE=SUBSTITUTE
CM032588.1	340694	.	C	T	100	*	TYPE=SUBSTITUTE
CM032588.1	340697	.	A	C	100	*	TYPE=SUBSTITUTE
CM032588.1	340703	.	C	T	100	*	TYPE=SUBSTITUTE
CM032588.1	340705	.	T	C	100	*	TYPE=SUBSTITUTE
CM032588.1	340706	.	C	T	100	*	TYPE=SUBSTITUTE
CM032588.1	340707	.	A	G	100	*	TYPE=SUBSTITUTE
CM032588.1	340710	.	T	C	100	*	TYPE=SUBSTITUTE
CM032588.1	340718	.	G	C	100	*	TYPE=SUBSTITUTE
CM032588.1	340724	.	A	G	100	*	TYPE=SUBSTITUTE
CM032588.1	340725	.	G	A	100	*	TYPE=SUBSTITUTE
CM032588.1	340731	.	T	C	100	*	TYPE=SUBSTITUTE
CM032588.1	340748	.	G	T	100	*	TYPE=SUBSTITUTE
CM032588.1	340749	.	a	aaagctcttc	100	*	TYPE=INSERT
CM032588.1	340757	.	T	C	100	*	TYPE=SUBSTITUTE
CM032588.1	340761	.	A	G	100	*	TYPE=SUBSTITUTE
CM032588.1	340762	.	G	T	100	*	TYPE=SUBSTITUTE
CM032588.1	340777	.	T	C	100	*	TYPE=SUBSTITUTE
CM032588.1	340813	.	A	G	100	*	TYPE=SUBSTITUTE
CM032588.1	340841	.	C	T	100	*	TYPE=SUBSTITUTE
CM032588.1	340842	.	G	A	100	*	TYPE=SUBSTITUTE
CM032588.1	340849	.	G	T	100	*	TYPE=SUBSTITUTE
CM032588.1	340858	.	A	T	100	*	TYPE=SUBSTITUTE
CM032588.1	340864	.	G	T	100	*	TYPE=SUBSTITUTE
CM032588.1	340895	.	A	T	100	*	TYPE=SUBSTITUTE
CM032588.1	340899	.	C	T	100	*	TYPE=SUBSTITUTE
CM032588.1	340934	.	T	A	100	*	TYPE=SUBSTITUTE
CM032588.1	340955	.	G	A	100	*	TYPE=SUBSTITUTE
CM032588.1	340997	.	C	T	100	*	TYPE=SUBSTITUTE
CM032588.1	341001	.	C	A	100	*	TYPE=SUBSTITUTE
CM032588.1	341006	.	T	C	100	*	TYPE=SUBSTITUTE
CM032588.1	341020	.	C	T	100	*	TYPE=SUBSTITUTE
CM032588.1	341025	.	T	C	100	*	TYPE=SUBSTITUTE
CM032588.1	341054	.	C	T	100	*	TYPE=SUBSTITUTE
CM032588.1	341062	.	C	CTT	100	*	TYPE=INSERT
CM032588.1	341066	.	C	A	100	*	TYPE=SUBSTITUTE
CM032588.1	341068	.	T	G	100	*	TYPE=SUBSTITUTE
CM032588.1	341087	.	T	G	100	*	TYPE=SUBSTITUTE
CM032588.1	341104	.	T	G	100	*	TYPE=SUBSTITUTE
CM032588.1	341146	.	G	A	100	*	TYPE=SUBSTITUTE
CM032588.1	341153	.	T	G	100	*	TYPE=SUBSTITUTE
CM032588.1	341167	.	C	A	100	*	TYPE=SUBSTITUTE
CM032588.1	341176	.	A	G	100	*	TYPE=SUBSTITUTE
CM032588.1	341185	.	C	G	100	*	TYPE=SUBSTITUTE
CM032588.1	341209	.	T	C	100	*	TYPE=SUBSTITUTE
CM032588.1	341227	.	C	T	100	*	TYPE=SUBSTITUTE
CM032588.1	341239	.	C	T	100	*	TYPE=SUBSTITUTE

CM032588.1	341254	.	T	C	100	*	TYPE=SUBSTITUTE
CM032588.1	341307	.	A	T	100	*	TYPE=SUBSTITUTE
CM032588.1	341308	.	C	T	100	*	TYPE=SUBSTITUTE
CM032588.1	341318	.	G	A	100	*	TYPE=SUBSTITUTE
CM032588.1	341322	.	C	T	100	*	TYPE=SUBSTITUTE
CM032588.1	341331	.	G	GA	100	*	TYPE=INSERT
CM032588.1	341336	.	A	G	100	*	TYPE=SUBSTITUTE
CM032588.1	341339	.	T	C	100	*	TYPE=SUBSTITUTE
CM032588.1	341348	.	G	C	100	*	TYPE=SUBSTITUTE
CM032588.1	341352	.	AG	A	100	*	TYPE=DELETE
CM032588.1	341359	.	A	G	100	*	TYPE=SUBSTITUTE
CM032588.1	341383	.	T	G	100	*	TYPE=SUBSTITUTE
CM032588.1	341452	.	T	C	100	*	TYPE=SUBSTITUTE
CM032588.1	341497	.	C	G	100	*	TYPE=SUBSTITUTE
CM032588.1	341527	.	C	T	100	*	TYPE=SUBSTITUTE
CM032588.1	341528	.	A	T	100	*	TYPE=SUBSTITUTE
CM032588.1	341542	.	C	T	100	*	TYPE=SUBSTITUTE
CM032588.1	341561	.	C	T	100	*	TYPE=SUBSTITUTE
CM032588.1	341565	.	TA	T	100	*	TYPE=DELETE
CM032588.1	341568	.	T	C	100	*	TYPE=SUBSTITUTE
CM032588.1	341574	.	A	G	100	*	TYPE=SUBSTITUTE
CM032588.1	341582	.	G	A	100	*	TYPE=SUBSTITUTE
CM032588.1	341664	.	G	A	100	*	TYPE=SUBSTITUTE
CM032588.1	341666	.	C	T	100	*	TYPE=SUBSTITUTE
CM032588.1	341672	.	A	G	100	*	TYPE=SUBSTITUTE
CM032588.1	341693	.	G	A	100	*	TYPE=SUBSTITUTE
CM032588.1	341705	.	C	T	100	*	TYPE=SUBSTITUTE
CM032588.1	341735	.	T	C	100	*	TYPE=SUBSTITUTE
CM032588.1	341747	.	G	A	100	*	TYPE=SUBSTITUTE
CM032588.1	341783	.	A	G	100	*	TYPE=SUBSTITUTE
CM032588.1	341797	.	C	T	100	*	TYPE=SUBSTITUTE
CM032588.1	341799	.	A	G	100	*	TYPE=SUBSTITUTE
CM032588.1	341804	.	G	A	100	*	TYPE=SUBSTITUTE
CM032588.1	341808	.	T	C	100	*	TYPE=SUBSTITUTE
CM032588.1	341810	.	T	TG	100	*	TYPE=INSERT
CM032588.1	341811	.	A	T	100	*	TYPE=SUBSTITUTE
CM032588.1	341812	.	A	C	100	*	TYPE=SUBSTITUTE
CM032588.1	341833	.	CA	C	100	*	TYPE=DELETE
CM032588.1	341866	.	A	G	100	*	TYPE=SUBSTITUTE
CM032588.1	341933	.	A	G	100	*	TYPE=SUBSTITUTE
CM032588.1	341984	.	C	T	100	*	TYPE=SUBSTITUTE
CM032588.1	341985	.	A	T	100	*	TYPE=SUBSTITUTE
CM032588.1	341999	.	C	T	100	*	TYPE=SUBSTITUTE
CM032588.1	342005	.	G	A	100	*	TYPE=SUBSTITUTE
CM032588.1	342083	.	T	C	100	*	TYPE=SUBSTITUTE
CM032588.1	342086	.	C	A	100	*	TYPE=SUBSTITUTE
CM032588.1	342089	.	C	T	100	*	TYPE=SUBSTITUTE
CM032588.1	342092	.	G	C	100	*	TYPE=SUBSTITUTE
CM032588.1	342156	.	G	A	100	*	TYPE=SUBSTITUTE
CM032588.1	342157	.	G	T	100	*	TYPE=SUBSTITUTE
CM032588.1	342165	.	T	TG	100	*	TYPE=INSERT
CM032588.1	342174	.	C	G	100	*	TYPE=SUBSTITUTE
CM032588.1	342175	.	G	T	100	*	TYPE=SUBSTITUTE
CM032588.1	342178	.	T	C	100	*	TYPE=SUBSTITUTE
CM032588.1	342182	.	A	G	100	*	TYPE=SUBSTITUTE
CM032588.1	342197	.	A	G	100	*	TYPE=SUBSTITUTE
CM032588.1	342201	.	A	T	100	*	TYPE=SUBSTITUTE
CM032588.1	342207	.	G	A	100	*	TYPE=SUBSTITUTE
CM032588.1	342213	.	G	GC	100	*	TYPE=INSERT
CM032588.1	342217	.	G	A	100	*	TYPE=SUBSTITUTE
CM032588.1	342223	.	T	C	100	*	TYPE=SUBSTITUTE
CM032588.1	342225	.	A	T	100	*	TYPE=SUBSTITUTE
CM032588.1	342226	.	T	C	100	*	TYPE=SUBSTITUTE
CM032588.1	342242	.	A	C	100	*	TYPE=SUBSTITUTE

CM032588.1	342248	.	T	TC	100	*	TYPE=INSERT
CM032588.1	342251	.	G	A	100	*	TYPE=SUBSTITUTE
CM032588.1	342256	.	G	T	100	*	TYPE=SUBSTITUTE
CM032588.1	342261	.	C	T	100	*	TYPE=SUBSTITUTE
CM032588.1	342271	.	G	T	100	*	TYPE=SUBSTITUTE
CM032588.1	342277	.	A	C	100	*	TYPE=SUBSTITUTE
CM032588.1	342278	.	T	C	100	*	TYPE=SUBSTITUTE
CM032588.1	342284	.	T	G	100	*	TYPE=SUBSTITUTE
CM032588.1	342286	.	T	G	100	*	TYPE=SUBSTITUTE
CM032588.1	342287	.	T	C	100	*	TYPE=SUBSTITUTE
CM032588.1	342290	.	G	T	100	*	TYPE=SUBSTITUTE
CM032588.1	342302	.	T	C	100	*	TYPE=SUBSTITUTE
CM032588.1	342315	.	A	T	100	*	TYPE=SUBSTITUTE
CM032588.1	342320	.	C	A	100	*	TYPE=SUBSTITUTE
CM032588.1	342325	.	T	C	100	*	TYPE=SUBSTITUTE
CM032588.1	342327	.	A	C	100	*	TYPE=SUBSTITUTE
CM032588.1	342329	.	A	G	100	*	TYPE=SUBSTITUTE
CM032588.1	342337	.	T	G	100	*	TYPE=SUBSTITUTE
CM032588.1	342338	.	A	G	100	*	TYPE=SUBSTITUTE
CM032588.1	342343	.	C	A	100	*	TYPE=SUBSTITUTE
CM032588.1	342347	.	A	T	100	*	TYPE=SUBSTITUTE
CM032588.1	342348	.	T	G	100	*	TYPE=SUBSTITUTE
CM032588.1	342349	.	A	C	100	*	TYPE=SUBSTITUTE
CM032588.1	342350	.	A	G	100	*	TYPE=SUBSTITUTE
CM032588.1	342352	.	T	G	100	*	TYPE=SUBSTITUTE
CM032588.1	342356	.	C	CGGA	100	*	TYPE=INSERT
CM032588.1	342360	.	C	T	100	*	TYPE=SUBSTITUTE
CM032588.1	342361	.	T	TGTCCGGGAG	100	*	TYPE=INSERT
CM032588.1	342364	.	T	TG	100	*	TYPE=INSERT
CM032588.1	342366	.	T	A	100	*	TYPE=SUBSTITUTE
CM032588.1	342376	.	T	C	100	*	TYPE=SUBSTITUTE
CM032588.1	342381	.	C	T	100	*	TYPE=SUBSTITUTE
CM032588.1	342382	.	A	G	100	*	TYPE=SUBSTITUTE
CM032588.1	342384	.	GA	G	100	*	TYPE=DELETE
CM032588.1	342386	.	G	T	100	*	TYPE=SUBSTITUTE
CM032588.1	342389	.	C	T	100	*	TYPE=SUBSTITUTE
CM032588.1	342396	.	A	G	100	*	TYPE=SUBSTITUTE
CM032588.1	342397	.	A	G	100	*	TYPE=SUBSTITUTE
CM032588.1	342402	.	T	TGATCTTTAG	100	*	TYPE=INSERT
CM032588.1	342407	.	A	T	100	*	TYPE=SUBSTITUTE
CM032588.1	342410	.	A	G	100	*	TYPE=SUBSTITUTE
CM032588.1	342413	.	T	G	100	*	TYPE=SUBSTITUTE
CM032588.1	342414	.	G	A	100	*	TYPE=SUBSTITUTE
CM032588.1	342417	.	C	T	100	*	TYPE=SUBSTITUTE
CM032588.1	342423	.	A	T	100	*	TYPE=SUBSTITUTE
CM032588.1	342424	.	C	G	100	*	TYPE=SUBSTITUTE
CM032588.1	342428	.	T	C	100	*	TYPE=SUBSTITUTE
CM032588.1	342436	.	T	C	100	*	TYPE=SUBSTITUTE
CM032588.1	342439	.	T	G	100	*	TYPE=SUBSTITUTE
CM032588.1	342441	.	G	A	100	*	TYPE=SUBSTITUTE
CM032588.1	342444	.	C	T	100	*	TYPE=SUBSTITUTE
CM032588.1	342448	.	TCA	T	100	*	TYPE=DELETE
CM032588.1	342451	.	A	T	100	*	TYPE=SUBSTITUTE
CM032588.1	342453	.	A	C	100	*	TYPE=SUBSTITUTE
CM032588.1	342455	.	C	G	100	*	TYPE=SUBSTITUTE
CM032588.1	342460	.	T	G	100	*	TYPE=SUBSTITUTE
CM032588.1	342463	.	C	A	100	*	TYPE=SUBSTITUTE
CM032588.1	342471	.	C	T	100	*	TYPE=SUBSTITUTE
CM032588.1	342472	.	A	G	100	*	TYPE=SUBSTITUTE
CM032588.1	342473	.	C	A	100	*	TYPE=SUBSTITUTE
CM032588.1	342474	.	C	A	100	*	TYPE=SUBSTITUTE
CM032588.1	342486	.	A	C	100	*	TYPE=SUBSTITUTE
CM032588.1	342488	.	C	T	100	*	TYPE=SUBSTITUTE
CM032588.1	342489	.	T	C	100	*	TYPE=SUBSTITUTE

CM032588.1	342491	.	T	C	100	*	TYPE=SUBSTITUTE
CM032588.1	342494	.	T	C	100	*	TYPE=SUBSTITUTE
CM032588.1	342496	.	G	T	100	*	TYPE=SUBSTITUTE
CM032588.1	342511	.	T	C	100	*	TYPE=SUBSTITUTE
CM032588.1	342512	.	G	c	100	*	TYPE=SUBSTITUTE
CM032588.1	342514	.	G	t	100	*	TYPE=SUBSTITUTE
CM032588.1	342515	.	A	t	100	*	TYPE=SUBSTITUTE
CM032588.1	342520	.	G	a	100	*	TYPE=SUBSTITUTE
CM032588.1	342522	.	G	t	100	*	TYPE=SUBSTITUTE
CM032588.1	342528	.	G	t	100	*	TYPE=SUBSTITUTE
CM032588.1	342530	.	G	a	100	*	TYPE=SUBSTITUTE
CM032588.1	342531	.	A	g	100	*	TYPE=SUBSTITUTE
CM032588.1	342539	.	T	C	100	*	TYPE=SUBSTITUTE
CM032588.1	342541	.	A	G	100	*	TYPE=SUBSTITUTE
CM032588.1	342543	.	T	C	100	*	TYPE=SUBSTITUTE
CM032588.1	342544	.	T	C	100	*	TYPE=SUBSTITUTE
CM032588.1	342545	.	T	A	100	*	TYPE=SUBSTITUTE
CM032588.1	342547	.	A	T	100	*	TYPE=SUBSTITUTE
CM032588.1	342555	.	G	A	100	*	TYPE=SUBSTITUTE
CM032588.1	342557	.	T	G	100	*	TYPE=SUBSTITUTE
CM032588.1	342560	.	C	G	100	*	TYPE=SUBSTITUTE
CM032588.1	342564	.	T	A	100	*	TYPE=SUBSTITUTE
CM032588.1	342569	.	G	T	100	*	TYPE=SUBSTITUTE
CM032588.1	342572	.	C	T	100	*	TYPE=SUBSTITUTE
CM032588.1	342573	.	C	T	100	*	TYPE=SUBSTITUTE
CM032588.1	342577	.	A	T	100	*	TYPE=SUBSTITUTE
CM032588.1	342588	.	G	A	100	*	TYPE=SUBSTITUTE
CM032588.1	342589	.	A	G	100	*	TYPE=SUBSTITUTE
CM032588.1	342595	.	G	T	100	*	TYPE=SUBSTITUTE
CM032588.1	342598	.	C	A	100	*	TYPE=SUBSTITUTE
CM032588.1	342600	.	T	C	100	*	TYPE=SUBSTITUTE
CM032588.1	342608	.	G	T	100	*	TYPE=SUBSTITUTE
CM032588.1	342611	.	A	G	100	*	TYPE=SUBSTITUTE
CM032588.1	342613	.	G	A	100	*	TYPE=SUBSTITUTE
CM032588.1	342614	.	T	C	100	*	TYPE=SUBSTITUTE
CM032588.1	342634	.	G	T	100	*	TYPE=SUBSTITUTE
CM032588.1	342637	.	C	T	100	*	TYPE=SUBSTITUTE
CM032588.1	342640	.	C	T	100	*	TYPE=SUBSTITUTE
CM032588.1	342641	.	A	G	100	*	TYPE=SUBSTITUTE
CM032588.1	342648	.	A	G	100	*	TYPE=SUBSTITUTE
CM032588.1	342650	.	A	G	100	*	TYPE=SUBSTITUTE
CM032588.1	342653	.	T	C	100	*	TYPE=SUBSTITUTE
CM032588.1	342659	.	A	G	100	*	TYPE=SUBSTITUTE
CM032588.1	342660	.	C	T	100	*	TYPE=SUBSTITUTE
CM032588.1	342667	.	T	C	100	*	TYPE=SUBSTITUTE
CM032588.1	342672	.	T	A	100	*	TYPE=SUBSTITUTE
CM032588.1	342675	.	C	T	100	*	TYPE=SUBSTITUTE
CM032588.1	342677	.	T	A	100	*	TYPE=SUBSTITUTE
CM032588.1	342681	.	C	T	100	*	TYPE=SUBSTITUTE
CM032588.1	342689	.	C	T	100	*	TYPE=SUBSTITUTE
CM032588.1	342695	.	G	A	100	*	TYPE=SUBSTITUTE
CM032588.1	342696	.	G	A	100	*	TYPE=SUBSTITUTE
CM032588.1	342697	.	G	C	100	*	TYPE=SUBSTITUTE
CM032588.1	342702	.	A	AAC	100	*	TYPE=INSERT
CM032588.1	342708	.	CAA	C	100	*	TYPE=DELETE
CM032588.1	342711	.	C	T	100	*	TYPE=SUBSTITUTE
CM032588.1	342715	.	G	A	100	*	TYPE=SUBSTITUTE
CM032588.1	342716	.	G	C	100	*	TYPE=SUBSTITUTE
CM032588.1	342717	.	C	G	100	*	TYPE=SUBSTITUTE
CM032588.1	342719	.	T	G	100	*	TYPE=SUBSTITUTE
CM032588.1	342720	.	C	G	100	*	TYPE=SUBSTITUTE
CM032588.1	342721	.	C	T	100	*	TYPE=SUBSTITUTE
CM032588.1	342722	.	C	T	100	*	TYPE=SUBSTITUTE
CM032588.1	342729	.	A	T	100	*	TYPE=SUBSTITUTE

CM032588.1	342732	.	T	C	100	*	TYPE=SUBSTITUTE
CM032588.1	342734	.	C	A	100	*	TYPE=SUBSTITUTE
CM032588.1	342736	.	T	C	100	*	TYPE=SUBSTITUTE
CM032588.1	342747	.	G	T	100	*	TYPE=SUBSTITUTE
CM032588.1	342756	.	G	A	100	*	TYPE=SUBSTITUTE
CM032588.1	342760	.	C	T	100	*	TYPE=SUBSTITUTE
CM032588.1	342771	.	T	G	100	*	TYPE=SUBSTITUTE
CM032588.1	342798	.	A	C	100	*	TYPE=SUBSTITUTE
CM032588.1	342804	.	A	C	100	*	TYPE=SUBSTITUTE
CM032588.1	342815	.	T	C	100	*	TYPE=SUBSTITUTE
CM032588.1	342816	.	A	G	100	*	TYPE=SUBSTITUTE
CM032588.1	342822	.	T	C	100	*	TYPE=SUBSTITUTE
CM032588.1	342826	.	T	C	100	*	TYPE=SUBSTITUTE
CM032588.1	342827	.	C	T	100	*	TYPE=SUBSTITUTE
CM032588.1	342828	.	C	A	100	*	TYPE=SUBSTITUTE
CM032588.1	342844	.	A	ATA	100	*	TYPE=INSERT
CM032588.1	342845	.	A	C	100	*	TYPE=SUBSTITUTE
CM032588.1	342847	.	G	A	100	*	TYPE=SUBSTITUTE
CM032588.1	342851	.	T	C	100	*	TYPE=SUBSTITUTE
CM032588.1	342866	.	T	C	100	*	TYPE=SUBSTITUTE
CM032588.1	342936	.	C	A	100	*	TYPE=SUBSTITUTE
CM032588.1	342948	.	A	G	100	*	TYPE=SUBSTITUTE
CM032588.1	342971	.	T	TT	100	*	TYPE=INSERT
CM032588.1	342993	.	A	C	100	*	TYPE=SUBSTITUTE
CM032588.1	343003	.	GC	G	100	*	TYPE=DELETE
CM032588.1	343011	.	A	G	100	*	TYPE=SUBSTITUTE
CM032588.1	343035	.	T	C	100	*	TYPE=SUBSTITUTE
CM032588.1	343058	.	TC	T	100	*	TYPE=DELETE
CM032588.1	343064	.	T	TC	100	*	TYPE=INSERT
CM032588.1	343067	.	T	G	100	*	TYPE=SUBSTITUTE
CM032588.1	343071	.	C	T	100	*	TYPE=SUBSTITUTE
CM032588.1	343072	.	A	T	100	*	TYPE=SUBSTITUTE
CM032588.1	343073	.	C	T	100	*	TYPE=SUBSTITUTE
CM032588.1	343074	.	C	T	100	*	TYPE=SUBSTITUTE
CM032588.1	343079	.	A	T	100	*	TYPE=SUBSTITUTE
CM032588.1	343089	.	G	A	100	*	TYPE=SUBSTITUTE
CM032588.1	343093	.	G	A	100	*	TYPE=SUBSTITUTE
CM032588.1	343100	.	C	A	100	*	TYPE=SUBSTITUTE
CM032588.1	343104	.	A	C	100	*	TYPE=SUBSTITUTE
CM032588.1	343105	.	T	A	100	*	TYPE=SUBSTITUTE
CM032588.1	343124	.	T	A	100	*	TYPE=SUBSTITUTE
CM032588.1	343126	.	A	C	100	*	TYPE=SUBSTITUTE
CM032588.1	343135	.	G	C	100	*	TYPE=SUBSTITUTE
CM032588.1	343149	.	TATCCTCATCACCCCTAA	T	100	*	TYPE=DELETE
CM032588.1	343203	.	A	T	100	*	TYPE=SUBSTITUTE
CM032588.1	343278	.	A	G	100	*	TYPE=SUBSTITUTE
CM032588.1	343320	.	C	G	100	*	TYPE=SUBSTITUTE
CM032588.1	343355	.	A	AA	100	*	TYPE=INSERT
CM032588.1	343379	.	C	G	100	*	TYPE=SUBSTITUTE
CM032588.1	343401	.	A	g	100	*	TYPE=SUBSTITUTE
CM032588.1	343407	.	T	A	100	*	TYPE=SUBSTITUTE
CM032588.1	343417	.	T	C	100	*	TYPE=SUBSTITUTE
CM032588.1	343443	.	G	GCCA	100	*	TYPE=INSERT
CM032588.1	343466	.	G	A	100	*	TYPE=SUBSTITUTE
CM032588.1	343490	.	T	A	100	*	TYPE=SUBSTITUTE
CM032588.1	343507	.	T	C	100	*	TYPE=SUBSTITUTE
CM032588.1	343539	.	GGG	G	100	*	TYPE=DELETE
CM032588.1	343544	.	GT	G	100	*	TYPE=DELETE
CM032588.1	343551	.	C	G	100	*	TYPE=SUBSTITUTE
CM032588.1	343575	.	T	TCGTAC	100	*	TYPE=INSERT
CM032588.1	343576	.	T	A	100	*	TYPE=SUBSTITUTE
CM032588.1	343640	.	T	TT	100	*	TYPE=INSERT
CM032588.1	343677	.	A	AA	100	*	TYPE=INSERT

CM032588.1	343689	.	CA	C	100	*	TYPE=DELETE
CM032588.1	343725	.	G	A	100	*	TYPE=SUBSTITUTE
CM032588.1	343729	.	C	A	100	*	TYPE=SUBSTITUTE
CM032588.1	343730	.	CG	C	100	*	TYPE=DELETE
CM032588.1	343786	.	TG	T	100	*	TYPE=DELETE
CM032588.1	343879	.	TT	T	100	*	TYPE=DELETE
CM032588.1	343896	.	A	ACTA	100	*	TYPE=INSERT
CM032588.1	343899	.	T	A	100	*	TYPE=SUBSTITUTE
CM032588.1	343991	.	T	A	100	*	TYPE=SUBSTITUTE
CM032588.1	344086	.	C	T	100	*	TYPE=SUBSTITUTE
CM032588.1	344110	.	C	T	100	*	TYPE=SUBSTITUTE
CM032588.1	344122	.	G	A	100	*	TYPE=SUBSTITUTE
CM032588.1	344146	.	C	T	100	*	TYPE=SUBSTITUTE
CM032588.1	344150	.	T	A	100	*	TYPE=SUBSTITUTE
CM032588.1	344159	.	T	A	100	*	TYPE=SUBSTITUTE
CM032588.1	344173	.	C	A	100	*	TYPE=SUBSTITUTE
CM032588.1	344189	.	T	C	100	*	TYPE=SUBSTITUTE
CM032588.1	344195	.	G	C	100	*	TYPE=SUBSTITUTE
CM032588.1	344219	.	A	G	100	*	TYPE=SUBSTITUTE
CM032588.1	344224	.	T	C	100	*	TYPE=SUBSTITUTE
CM032588.1	344255	.	T	C	100	*	TYPE=SUBSTITUTE
CM032588.1	344264	.	G	A	100	*	TYPE=SUBSTITUTE
CM032588.1	344266	.	T	C	100	*	TYPE=SUBSTITUTE
CM032588.1	344267	.	A	G	100	*	TYPE=SUBSTITUTE
CM032588.1	344297	.	G	a	100	*	TYPE=SUBSTITUTE
CM032588.1	344309	.	A	G	100	*	TYPE=SUBSTITUTE
CM032588.1	344314	.	TAG	T	100	*	TYPE=DELETE
CM032588.1	344320	.	C	T	100	*	TYPE=SUBSTITUTE
CM032588.1	344348	.	CAAAAGTTCC	C	100	*	TYPE=DELETE
CM032588.1	344358	.	G	C	100	*	TYPE=SUBSTITUTE
CM032588.1	344362	.	T	G	100	*	TYPE=SUBSTITUTE
CM032588.1	344364	.	G	C	100	*	TYPE=SUBSTITUTE
CM032588.1	344398	.	G	T	100	*	TYPE=SUBSTITUTE
CM032588.1	344407	.	A	G	100	*	TYPE=SUBSTITUTE
CM032588.1	344419	.	T	C	100	*	TYPE=SUBSTITUTE
CM032588.1	344489	.	C	T	100	*	TYPE=SUBSTITUTE
CM032588.1	344497	.	G	A	100	*	TYPE=SUBSTITUTE
[truncated: 630,293 more chars]
